# 14-3-3 signal adaptor and scaffold proteins mediate GPCR trafficking

**DOI:** 10.1038/s41598-019-47478-w

**Published:** 2019-08-01

**Authors:** Luwa Yuan, Shahar Barbash, Sathapana Kongsamut, Alex Eishingdrelo, Thomas P. Sakmar, Haifeng Eishingdrelo

**Affiliations:** 1 BioInvenu Corp., 50 Williams Parkway, Unit A2, East Hanover, NJ 07936 USA; 2 Laboratory of Chemical Biology & Signal Transduction, The Rockefeller University, 1230 York Avenue, New York, New York, 10065 USA; 3 Department of Neurobiology, Care Sciences and Society, Division for Neurogeriatrics, Center for Alzheimer Research, Karolinska Institutet, 141 57 Huddinge, Sweden

**Keywords:** Protein translocation, Assay systems

## Abstract

Receptor trafficking is pivotal for the temporal and spatial control of GPCR signaling and is regulated by multiple cellular proteins. We provide evidence that GPCRs interact with 14-3-3 signal adaptor/scaffold proteins and that this interaction regulates receptor trafficking in two ways. We found GPCR/14-3-3 interaction signals can be agonist-induced or agonist-inhibited. Some GPCRs associate with 14-3-3 proteins at the cell membrane and agonist treatments result in disrupted GPCR/14-3-3 interaction signals. The diminished GPCR/14-3-3 interaction signals are temporally correlated with increased GPCR/β-arrestin interaction signals in response to agonist treatment. Other GPCRs showed agonist-induced GPCR/14-3-3 interaction signal increases that occur later than agonist-induced GPCR/β-arrestin interaction signals, indicating that GPCR/14-3-3 interaction occurred after receptor endocytosis. These two types of GPCR/14-3-3 interaction patterns correlate with different receptor trafficking patterns. In addition, the bioinformatic analysis predicts that approximately 90% of GPCRs contain at least one putative 14-3-3 binding motif, suggesting GPCR/14-3-3 association could be a general phenomenon. Based on these results and collective evidence, we propose a working model whereby 14-3-3 serves as a sorting factor to regulate receptor trafficking.

## Introduction

Agonist-induced internalization of GPCRs has been recognized as being coincident with the desensitization of G protein-mediated signaling^1^. The phenomenon was mechanistically illustrated by the discovery of β-arrestins that are recruited to agonist-bound GPCRs, terminating G-protein signaling and promoting GPCR endocytosis^2^. β-arrestins shuttle receptors from the cell membrane to endosomes through clathrin-coated pits and also initiate G-protein-independent signaling through β-arrestin-mediated kinase cascades. Internalized GPCRs travel through a series of endosomal compartments, and may recycle back to the cell membrane for another round of activation (resensitization) or move toward lysosomes for degradation.

Receptor trafficking and recycling are crucial for the temporal and spatial control of GPCR signaling. GPCRs trafficking within a series of endosomal compartments undergo dynamic post-translational modifications and encounter various cytoplasmic proteins. The fate of GPCRs after endocytosis is likely determined by multiple post-endocytotic regulatory processes, which include phosphorylation status of GPCRs on specific sites, ubiquitination of GPCRs, and interaction with sorting and scaffold proteins^3^. It has been postulated that the cytoplasmic domains of GPCRs regulate trafficking through interaction with sorting adaptors or scaffolding proteins in addition to β-arrestins. However, precisely what sorting determinants guide receptor recycling pathways remains an elusive and fundamental biological question.

In addition to heterotrimeric G proteins and β-arrestins, many other cellular effectors are recruited to GPCRs to regulate signaling. Signal adaptor proteins such as 14-3-3 can interact with GPCRs in response to agonist treatment^4^. The 14-3-3 protein family consists of seven isoforms. They are ~30-kDa acidic proteins, ubiquitously and abundantly expressed in cells, and are present in the cytoplasm, intracellular organelles and associated with the plasma membrane. The highest expression of 14-3-3 proteins is found in the brain, where they make up approximately 1% of total soluble protein. Some of the 14-3-3 isoforms are particularly enriched in synapses, presumably to regulate neurotransmission and plasticity^5^. 14-3-3 proteins form homo- or heterodimers and function as scaffold proteins to change client protein conformation, facilitate or inhibit client protein interactions, mask or protect client protein phosphorylation status, and transport client proteins among different compartments. Previously, we have shown that 14-3-3 proteins can interact with GPCRs by utilizing a cell-based protein-protein interaction LinkLight assay technology^4^. We can pharmacologically characterize GPCR/14-3-3 interaction signals. The interaction is phosphorylation-dependent and can be mediated by GPCR agonists. The GPCR/14-3-3 association may be β-arrestin-independent, and importantly, the onset of interaction signals of GPCR/14-3-3 is relatively slower than GPCR/β-arrestin interaction signals, suggesting GPCR/14-3-3 association occurs after receptor internalization. However, the physiological role of 14-3-3 in GPCR signaling is not clear.

Promotion of cell surface transport of membrane proteins is one of the emerging roles of 14-3-3 proteins. The C-terminal tail of the human ĸ-opioid receptor (KOR) interacts with 14-3-3ζ and transports de novo synthesized receptor along the endoplasmic reticulum (ER) to the Golgi and to the cell membrane^6^. In the early endosome, transmembrane proteins internalized by endocytosis can be sorted to be delivered to a variety of destinations, including lysosomes, recycling endosomes, and Golgi. A variety of scaffold molecules and factors have been implicated in the process^7–10^. Trafficking from endosomes to the Golgi is also an important trafficking pathway and is often referred to as retrograde transport or retrograde recycling. Although 14-3-3 proteins are known to transport proteins between different cellular compartments, very little is known in the literature to link 14-3-3 to a physiological role in receptor recycling.

Here, we report that GPCR/14-3-3 interaction signals correlate with receptor recycling and cell-surface expression patterns, and suggest a possible physiological role of 14-3-3 in GPCR signaling. We investigated over two dozen GPCR/14-3-3 interaction signals in response to ligand treatment by using LinkLight assay technology^11^. GPCR agonist treatment enhances some GPCR/14-3-3 interaction signals while others are diminished upon agonist treatment. In general, agonist-induced GPCR/14-3-3 interaction signals occur in a timeframe relatively after GPCR/β-arrestin interaction signals, whereas agonist-inhibited GPCR/14-3-3 interaction signals occur in the same timeframe as agonist-induced GPCR/β-arrestin interaction signals. We also identified some GPCRs with known trafficking patterns that correlate with GPCR/14-3-3 interaction signals. Agonist-induced GPCR/14-3-3 interaction signals correlate temporally with known GPCR recycling back to the cell membrane, whereas agonist-inhibited GPCR/14-3-3 interaction signals correlate temporally with known GPCR depletion from the cell membrane. Based on the collective evidence of GPCR/14-3-3 interaction signals, and known 14-3-3 protein function in protein transport, we propose a working model for a role of 14-3-3 in GPCR trafficking.

## Results

### GPCR agonists can selectively promote or disrupt GPCR/14-3-3 association depending on the state of pre-coupling

**Figure 1:**
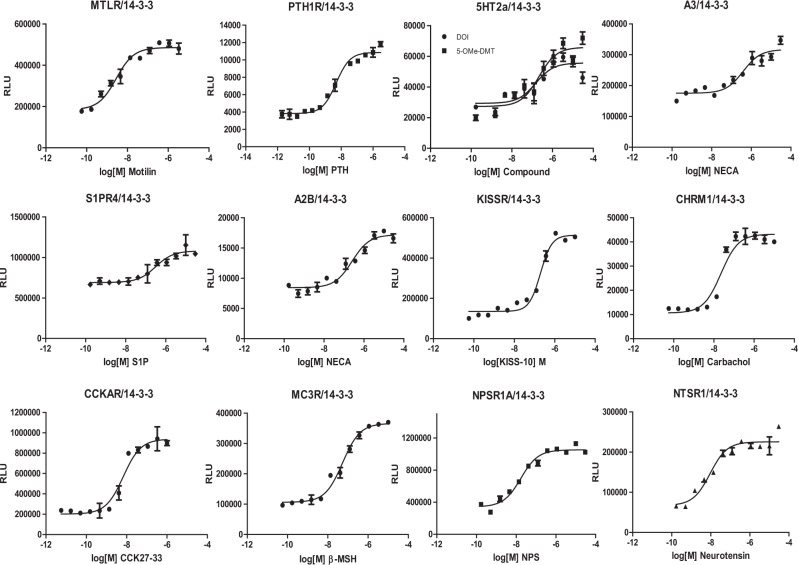
Agonist-induced GPCR/14-3-3 interaction signals. Dose-response curves of GPCR/14-3-3 interaction signals for motilin receptor (MTLR), parathyroid hormone 1 receptor (PTH1R), 5-hydroxytryptamine receptor 2A (5HT2A), adenosine A3 (A3), sphingosine-1-phosphate receptor 4 (S1PR4), adenosine A2B (A2B), Kisspeptin receptor (KISS1R), muscarinic 1 (CHRM1), cholecystokinin A receptor (CCKAR), melanocortin receptor 3 (MC3R), neurotensin receptor 1 (NTSR1), and neuropeptide S receptor 1 (NPSR1).

We applied the cell-based protein-protein interaction LinkLight assay technology to investigate GPCR/14-3-3 interactions by generating over two dozen GPCR/14-3-3 stable assay cell lines. HEK293 cells stably expressing 14-3-3ε-pLuc (14-3-3ε-permuted luciferase) were transfected with GPCR-TEV (tobacco etch virus) fusion gene plasmids and were selected with hygromycin B. Monoclonal stable lines were evaluated for agonist responses. Many GPCRs respond to agonist stimulation with increased GPCR/14-3-3 interaction signals. The interaction signals can be pharmacologically characterized, and EC_50_ values for agonist-induced 14-3-3 interaction are comparable to those obtained with other effector assays. Motilin receptor (MTLR), parathyroid hormone 1 receptor (PTH1R), 5-hydroxytryptamine receptor 2 A (5HT2A), adenosine A3 (A3), sphingosine-1-phosphate receptor 4 (S1PR4), adenosine A2B (A2B), Kisspeptin receptor (KISS1R), muscarinic 1 (CHRM1), neurotensin receptor 1 (NTSR1), neuropeptide S receptor 1(NPSR1), cholecystokinin A receptor (CCKAR), and melanocortin receptor 3 (MC3R) all showed agonist-induced GPCR/14-3-3 interaction signals (Fig. 1). Previously, we also showed that beta-2 adrenergic receptor (ADRB2), beta-3 adrenergic receptor (ADRB3), alpha-1A adrenergic receptor (α1A), dopamine receptor D2 (DRD2), cholinergic receptor, and muscarinic cholinergic receptor 1, 3, and 5 (CHRM1, CHRM3, and CHRM5) interacted with 14-3-3 in response to agonist treatment^3^. The results demonstrate that activation of those GPCRs triggers signaling pathways that facilitate the recruitment of 14-3-3 proteins to the receptor complexes.

**Figure 2:**
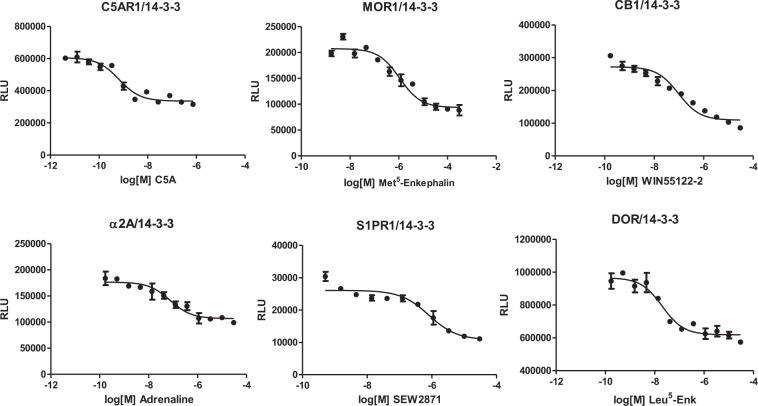
Agonist-disrupted GPCR/14-3-3 interaction signals. Dose-response curves of GPCR/14-3-3 interaction signals for sphingosine-1-phosphate receptor 1 (S1PR1), complement C5a receptor 1 (C5AR1), cannabinoid receptor 1 (CB1), galanin receptor 1 (GALR1), adrenergic receptor α2A (α2A), and δ-opioid receptor (DOR1).

Surprisingly, we also observed that agonist treatment of certain GPCRs inhibited GPCR/14-3-3 interaction signals. In the same GPCR/14-3-3 assay, complement C5a receptor 1 (C5AR1), μ-opioid receptor (MOR1), δ-opioid receptor (DOR1), cannabinoid receptor 1 (CB1), adrenergic receptor α2A (α2A), dopamine receptor D4 (DRD4), sphingosine-1-phosphate receptor 1 (S1PR1), and galanin receptor 1 (GALR1) all showed diminished interaction signals in response to agonist treatment. Agonist-inhibited GPCR/14-3-3 interaction signals were pharmacologically characterized (Fig. 2). The results suggest that GPCRs and 14-3-3 can exist in at least two association states: with certain GPCRs, agonist-induced interaction with 14-3-3 is clearly observed; with certain other GPCRs, pre-formed complexes with 14-3-3 proteins are dissociated upon agonist treatment. We went on to attempt to reconcile these apparently paradoxical observations.

### Agonist-induced GPCR/14-3-3 interaction signals appear at a relative later timeframe than GPCR/β-arrestin interaction signals

Comparing and contrasting different timeframes of signaling cascades and cellular trafficking can help to reconcile their degree of association. G-protein signaling is initiated in a matter of a few seconds after agonist ligand binding, whereas the GPCR/β-arrestin association can persist on a time scale of minutes to hours. The LinkLight assay is an end-point readout assay and cannot assess protein-protein interaction instantaneously. However, the technology has the advantage in that it does not require strict spatial and orientation requirements and enable to the use of full-length proteins, which retain all potential regulatory elements, to obtain physiologically relevant protein-protein interaction signals. Once a permuted luciferase and a TEV are brought into close proximity during the protein interactions, TEV protease cleaves permuted luciferase, and the cleaved permuted luciferase fragment is spontaneously refolded back to form an active luciferase, even the two interaction partners are separated. The luciferase signal intensity is relative to the amounts of active luciferases generated. Thus, we are able to determine the relative timeframes to achieve maximum interaction signals between GPCR/β-arrestin and GPCR/14-3-3 LinkLight assays. The only difference between the two assays is the signal adaptor proteins: β-arrestin vs. 14-3-3. All the other conditions are the same, and we can presume that the times for the TEV protease to cleave the permuted luciferase, and cleaved luciferase to fold active luciferase to be the same. Thus, the timeframes needed to reach maximum signals for GPCR/14-3-3 interaction and for GPCR/β-arrestin interaction could indicate the time difference between GPCR/β-arrestin and GPCR/14-3-3 interactions. Previously, we have compared the timeframes of α1AR/14-3-3 and α1AR/β-arrestin interaction signals and showed that α1AR/14-3-3 interaction signals occurred over a longer timeframe than α1AR/β-arrestin interaction signals^4^.

**Figure 3:**
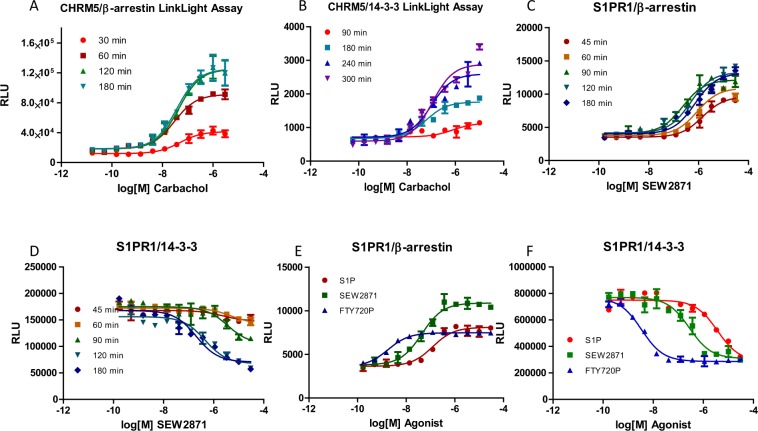
Comparison of timeframes between agonist-mediated GPCR/β-arrestin and GPCR/14-3-3 interaction signals. (**A**,**B**) Dose-response curves of agonist-induced CHRM5/β-arrestin and CHRM5/14-3-3 interaction signals at different times. (**C**,**D**) Dose-response curves of agonist-induced S1PR1/β-arrestin and agonist-disrupted S1PR1/14-3-3 interaction signals at different times. (**E**,**F**) Comparison of dose-response curves for both S1PR1/β-arrestin and S1PR1/14-3-3 interaction signals with multiple S1PR1 agonists after 3 hours incubation of ligands.

We investigated muscarinic cholinergic receptor 5 (CHRM5) interaction signals with β-arrestin and 14-3-3 in the LinkLight assays. Our data show that the CHRM5/14-3-3 interaction signals had a significantly longer timeframe than the CHRM5/β-arrestin interaction signals. The CHRM5/β-arrestin interaction signals appeared 30 min after the onset of carbachol treatment and reached a plateau after between 2 and 3 hours, whereas CHRM5/14-3-3 interaction signals began to appear 1 hour after initiation of agonist treatment, and reached a plateau at between 4 to 5 hours (Fig. 3A,B). Since GPCR/β-arrestin signals correlate with receptor internalization, our data suggest that the agonist-induced GPCR/14-3-3 interaction happens after the receptor is internalized. We hypothesize that ligand-bound GPCRs enter endosomes, undergo post-translational modifications and recruit 14-3-3 scaffolding proteins.

### Agonist-disrupted GPCR/14-3-3 interaction signals display a similar timeframe as agonist-induced GPCR/β-arrestin interaction signals

Next, we investigated the timeframe of agonist-disrupted GPCR/14-3-3 interaction signals. We compared the timeframes of S1PR1/β-arrestin and S1PR1/14-3-3 LinkLight assay signals needed to reach maximum interaction signals. The S1PR1-specific agonist SEW2871 induced S1PR1/β-arrestin signals were observed at 45 min after agonist treatment and the signals reached the maximum plateau at around 2 hours. Similarly, S1PR1/14-3-3 interaction signals started to appear after 45 min of agonist treatment and signals reached a plateau at around 2 to 3 hours after agonist treatment. The time to reach the maximum signal plateau for S1PR1/14-3-3 interaction signals appears overlapping or slightly delayed as compared with the S1PR1/β-arrestin interaction signals (Fig. 3C,D). The constant S1PR1/14-3-3 interaction signals in the absence of agonist treatment suggest that S1PR1 likely forms a complex with 14-3-3 at the cell membrane. Agonist-inhibited S1PR1/14-3-3 interaction signals occur coincidently with agonist-induced S1PR1/β-arrestin interaction signals, suggesting that receptor recruitment of β-arrestin might happen concurrently with the release of 14-3-3. The concurrent phenomenon suggests that S1PR1 can associate with 14-3-3 proteins at the cell membrane without agonist binding, while subsequent agonist binding results in the release of 14-3-3 proteins and recruitment of β-arrestins.

It is interesting to note the timing differences between agonist-induced and agonist-disrupted GPCR/14-3-3 interaction signals. The timeframe to reach maximum responses for agonist-inhibited GPCR/14-3-3 interaction signals is much faster than the timeframe for agonist-induced GPCR/14-3-3 interaction signals. It takes 2-3 hours to reach a maximum signal plateau for agonist-inhibited S1PR1/14-3-3 interaction signals, whereas it takes 4-5 hours to reach a maximum signal plateau for agonist-stimulated CHRM5/14-3-3 interaction signals. Our data suggest that some GPCRs recruit 14-3-3 proteins after receptor internalization into endosomes, while other GPCRs initially release 14-3-3 proteins at the cell surface in response to agonist treatment. It is important to note that we only add a TEV protease sequence at the C-terminus, and we did not fuse any piece of another GPCR sequence to enhance β-arrestin or 14-3-3 interaction signals. We think a chimeric GPCR with a piece of another GPCR sequence may change intracellular protein interaction and trafficking patterns and give misleading information. We also do not use the truncated protein domains in the assays. We believe using truncated protein domains in order to achieve optimum protein-protein interaction signals as used in other techniques could result in a loss of regulatory elements, not reflecting the real relevant physiological situation.

We also compared agonists in the S1PR1/β-arrestin and S1PR1/14-3-3 LinkLight assays to see if the agonist rank order of potency remains the same (Fig. 3E,F). The rank order (EC_50_) of S1PR1 agonists in the S1PR1/β-arrestin LinkLight assay (EC_50_ values FTY720P (1.8 nM) > SEW2871 (44 nM) > S1P (129 nM)) was the same as the rank order of S1PR1 agonists in the S1PR1/14-3-3 LinkLight assay (IC_50_ values FTY720P (3.4 nM) > SEW2871 (278 nM) > S1P (3.5 uM)). These data suggest that agonist-induced S1PR1/β-arrestin and agonist-disrupted S1PR1/14-3-3 pathways are closely related events.

### Chemical chaperone-mediated cell-membrane trafficking is regulated through GPCR/14-3-3 interaction

**Figure 4:**
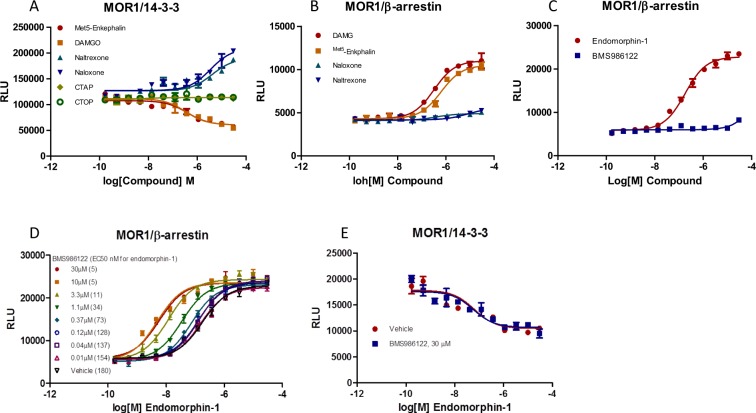
Comparison of multiple mu-opioid agonists and antagonists for both MOR1/14-3-3 and MOR1/β-arrestin interaction signals, and selective effect of the positive allosteric modulator (PAM) BMS986122 on MOR1/β-arrestin interaction. (**A**) Dose-response curves of MOR1/14-3-3 interaction signals in response to multiple agonists and antagonists. (**B**) Dose-response curves of MOR1/β-arrestin interaction signals in response to multiple agonists and antagonists. (**C**) Dose-response curves of MOR1/β-arrestin interaction signals in response to MOR1 agonist endomorphin-1 or PAM BMS986122 alone. (**D**) Dose-response curves of MOR1/β-arrestin interaction signals in the presence of 20 nM endomorphin-1 and with various concentration of BMS986122. (**E**) Dose-response curves of MOR1/14-3-3 interaction signals in response to various concentration of endomorphin-1, in the presence and absence of 30 μM BMS986122.

To explain the two patterns of GPCR/14-3-3 interaction signals in response to agonist treatment, we investigated the role of 14-3-3 protein to transport GPCRs to the cell membrane. Some GPCR antagonists are known to promote receptor cell surface expression and increase receptor cell membrane density. For example, the opioid overdose reversal drug naloxone, a MOR1 antagonist, is known to promote MOR1 trafficking to the cell membrane and increase cell membrane MOR1 density^12^ and is called a chemical chaperone. However, the mechanism of action is not clear and it has been speculated that chemical chaperone binding results in receptor conformation change and transport to the cell surface. We investigated the effect of naloxone on MOR1/14-3-3 interaction. MOR1 peptide agonists DAMGO and Met^5^-Enkephalin showed dose-dependent inhibition MOR1/14-3-3 interaction signals, suggesting the existence of a preassembled MOR1/14-3-3 complex presumably at the cell membrane. However, small molecule antagonist naloxone exhibited a dose-dependent increase in MOR1/14-3-3 interaction signals. Another MOR1 antagonist naltrexone also showed a dose-dependent increase in MOR1/14-3-3 interaction signals (Fig. 4A). The data suggest naloxone and naltrexone promote MOR1/14-3-3 association and MOR1/14-3-3 association leads to trafficking to the cell surface. To examine if naloxone and naltrexone have activity on MOR1/β-arrestin interaction signals, we performed the MOR1/β-arrestin LinkLight assays. MOR1 agonists DAMGO and Met5-Enkephalin showed a dose-dependent increase of MOR1/β-arrestin interaction signals, while naloxone and naltrexone exhibited no activity (Fig. 4B).

Chemical chaperone-mediated cell-membrane trafficking is thought to occur by ligands binding to intracellular pools of receptors^12^. To test this idea, we used the MOR1 peptide antagonists, CTAP and CTOP, which do not enter cells. We found that neither CTAP nor CTOP had an effect on the MOR1/14-3-3 LinkLight interaction signal (Fig. 4A). Our MOR1/14-3-3 interaction signal data provide a mechanistic explanation to previous observations that antagonists naloxone and naltrexone can promote MOR cell surface expression. Thus, MOR1 cell surface trafficking mediated by naloxone and naltrexone is mediated by MOR1/14-3-3 association. Ligands can differentially modulate GPCR/β-arrestin and GPCR/14-3-3 interaction signals.

Previously, we used adrenergic receptor β3 which does not recruit β-arrestin in response to agonist treatment as an example to show that GPCR/14-3-3 pathway can be independent of the GPCR/β-arrestin pathway^4^. The independent pathways suggest the possibility that a ligand could selectively modulate one pathway over another pathway. We were interested to investigate whether ligands can differentially modulate receptor GPCR/β-arrestin and GPCR/14-3-3 pathways. We tested a MOR1 positive allosteric modulator BMS986122 in MOR1/β-arrestin and MOR1/14-3-3 LinkLight assays. We first reproduced the previously reported result showing the positive allosteric activity of BMS986122^13^ using the MOR1/β-arrestin LinkLight assay. BMS986122 alone did not produce MOR1/β-arrestin interaction signals alone (Fig. 4C), but enhanced agonist endomorphin-1-induced signals. Without BMS986122, the endomorphin-1 EC_50_ was 180 nM, but in the presence of 10 or 30 μM of BMS986122, the EC_50_ was shifted leftward 36-fold to 5 nM (Fig. 4D). In contrast, BMS986122 has no activity in the MOR1/14-3-3 LinkLight assay, and there was no IC_50_ change by combining BMS986122 and endomorphin-1 in the assay, indicating that BMS986122 has no allosteric modulation activity on the MOR1/14-3-3 pathway (Fig. 4E). These results suggest that certain ligands can selectively modulate MOR1/β-arrestin and MOR1/14-3-3 pathways.

### The bioinformatic analysis predicts that a large number of GPCRs have at least one putative 14-3-3 binding motif

How many GPCRs possess the ability to interact with 14-3-3 proteins? We performed bioinformatic analysis of 399 human GPCRs for potential 14-3-3 binding motifs. We utilized the 14-3-3pred tool^14^ to search for putative motifs. We wanted to search for cytoplasmic regions of GPCRs. For this purpose, we utilized the GPCRdb numbering scheme^15^. The GPCRdb numbering system places every amino acid in relation to a set reference point for all Class-A GPCRs. Out of 399 human GPCRs of all classes, 358 (~90%) had at least one detected 14-3-3 motif by the 14-3-3Pred method (supplementary Table S1). The Supplementary Table S1 includes the full results with gene names, UniProt IDs, sequences, the position of the detected motif, and the specifically detected motif sequences, and the full detection results for all algorithm score levels. Note that this tool is threshold-sensitive. The Supplementary Table 2 shows the number of detected hits and GPCRs for different algorithm score thresholds. However, not every putative 14-3-3 motif identified by bioinformatic analysis may actually bind 14-3-3 proteins in a given cell environment. Many factors such as post-translational modifications, the existence of other competing proteins, and pH environment can influence the binding. In addition, 14-3-3-binding sites in many proteins do not comply with the optimal motifs; other structural features may contribute to the binding.

### Mutagenesis of a phosphorylation site of 14-3-3 binding motifs abolishes dopamine-induced DRD5/14-3-3 interaction

**Figure 5:**
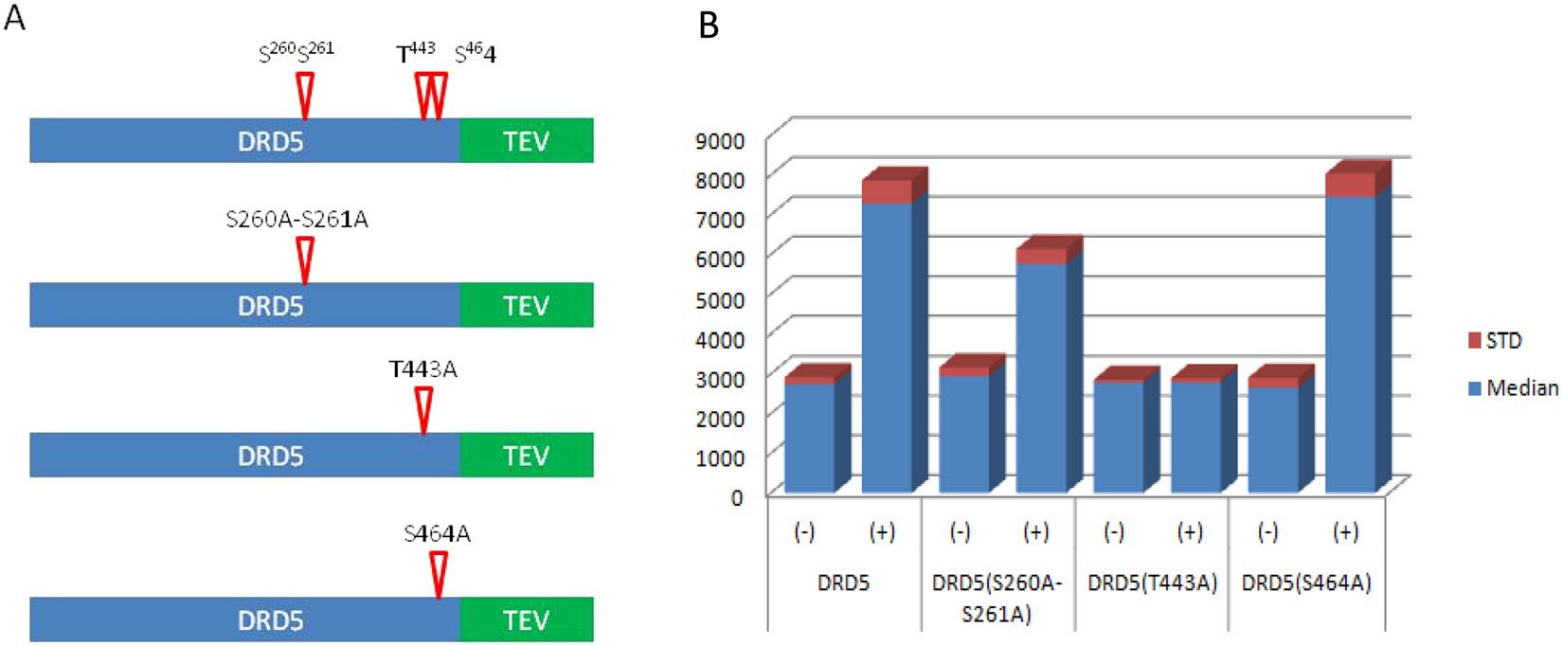
Mutagenesis study of putative 14-3-3 binding motifs in DRD5. (**A**) Three putative 14-3-3 binding motifs with four potential serine and threonine phosphorylation sites were identified by 14-3-3pred analysis. The diagrams of wild type and three DRD5 mutants with point mutations from serine or threonine to alanine are indicated. (**B**) DRD5-TEV wild type and DRD5 mutants were transiently expressed in the 14-3-3-pLuc HEK293 host cells and examed for with (+) or without (−) 10 μM dopamine-induced DRD5/14-3-3 interaction signals.

Bioinformatic analysis using 14-3-3pred tool indicates multiple putative 14-3-3 motifs can be present for a GPCR. To examine whether a particular 14-3-3 motif or multiple 14-3-3 motifs participate in recruiting 14-3-3 proteins, we used dopamine receptor 5 (DRD5) as an example. Based on the 14-3-3pred tool^14^ analysis of DRD5 (dopamine receptor 5), three putative 14-3-3 binding motifs: VQIRRI^260^S^261^SLER, MFQIYQ^443^TSPDG, and DCEGEI^464^SLDKI and 4 potential phosphorylation sites, two on the intracellular loop 3 ^260^S^261^S, and two on the C-terminal tail ^443^T and ^464^S were identified. To examine which of the 14-3-3 binding motifs contribute to binding, we generated three DRD5-TEV expression constructs by mutating putative phosphorylation residues serine and threonine to alanine. DRD5(S260A-S261A)-TEV, DRD5(T443A)-TEV, and DRD5(S464A)-TEV expression plasmids were constructed by the point mutation using PCR primers. DRD5(S260A-S261A)-TEV plasmid has two adjacent serine replaced by alanine at residues 260 and 261 located in the intracellular loop 3. DRD5(T443A)-TEV, and DRD5(S464A)-TEV expression plasmids have a threonine at residue 443 replaced by alanine and serine at residue 464 replaced with alanine located in the C-terminal tail, respectively (Fig. 5A). The DRD5-TEV point mutation plasmids and wild type DRD5-TEV expression plasmids were transfected into the host 14-3-3γ-pLuc HEK293 cells. We then looked for the transient expression of the wild type and mutant DRD5-TEV plasmids, and assessed dopamine stimulated signals. The positive control of wild type DRD5-TEV expression plasmid showed dopamine-induced signals as did DRD5(S260A-S261A)-TEV and DRD5(S464A)-TEV mutants in the transfected cells. However, the DRD5(T443A)-TEV expression plasmid transfected cells lost dopamine responsiveness (Fig. 5B). The results indicate the motif MFQIYQ^443^TSPDG in the C-terminal tail of DRD5 is the 14-3-3 binding site. The phosphorylation of the threonine443 residue is required for 14-3-3 binding.

### Intracellular kinases can interact with 14-3-3 proteins in response to GPCR activation

**Figure 6:**
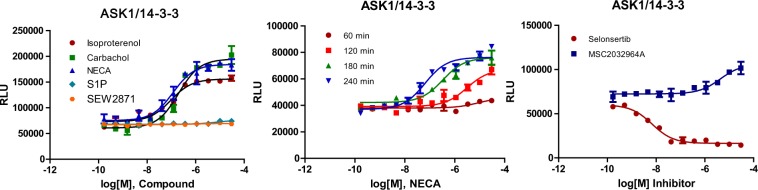
Dose-response curves of ASK1/14-3-3 interaction signals. (**A**) Dose-response curves of ASK1/14-3-3 interaction signals in response to GPCR agonist stimulation. (**B**) Timeframe of ASK1/14-3-3 interaction signals in response to adenosine receptor agonist NECA treatment. (**C**) Dose-response curves of ASK1/14-3-3 interaction signals in response to ASK1 inhibitors selonsertib and MSC2032964A.

14-3-3 proteins are known to interact with a large number of very diverse proteins. Previously, we have shown that activation of GPCRs can promote the interaction of 14-3-3 with the kinase Raf-1^16^, which is a component of the Raf/MEK/ERK signaling pathway. The data suggests 14-3-3 proteins likely participate in the ERK signaling cascade. In addition to Raf-1 kinase, a long list of other kinases such as ASK1, PI3K, PI4K, RSK, PKC, CDK11, MEK, and ChK1, are known to interact with 14-3-3 proteins^17,18^. The Raf/MEK/ERK signaling pathway plays a central role in the survival and mitogenic signaling, while ASK/JNK and p38 MAP kinase cascades are preferentially activated by environmental stresses and are actively involved in various stress responses including cell death, survival, and differentiation. Studies have shown that the activation of GPCRs can lead to ASK1 activation^19^. Thus, we want to look if 14-3-3 proteins are involved in the ASK1 signaling pathway in response to GPCR agonists. We have established an ASK1/14-3-3 LinkLight assay cell line and looked for ASK1/14-3-3 interaction signals in response to agonist treatment. It is known that HEK293 cells express several GPCRs endogenously, such as adrenergic receptors, adenosine receptors, muscarinic acetylcholine receptors, and S1P receptors. The ASK1/14-3-3 cells treated with GPCR agonists adrenaline, NECA, and carbachol showed dose-dependent ASK1/14-3-3 interaction signals. However, not every GPCR agonist was observed to promote ASK1/14-3-3 interaction. S1PR1 agonists S1P and SEW2871 did not promote ASK1/14-3-3 interaction (Fig. 6A). We also looked at the relative timeframe of agonist-induced ASK1/14-3-3 interaction signals. The ASK1/14-3-3 interaction signals appeared to plateau around 4 hours after agonist treatment, suggesting the ASK1/14-3-3 interaction signaling is generated after receptor internalization and long after G protein signaling has ended (Fig. 6B). GPCR-mediated kinase/14-3-3 interactions suggest that 14-3-3 proteins have multiple roles in GPCR trafficking and signaling. Since ASK1 is a drug target for multiple diseases, we tested two ASK1 inhibitors in the ASK1/14-3-3 LinkLight assay. To our surprise, selonsertib showed a dose-dependent inhibition of ASK1/14-3-3 interaction signals, whereas MSC2032964A did not inhibit ASK1/14-3-3 interaction signals, but slightly promoted ASK1/14-3-3 interaction signals (Fig. 6C). The results suggest that the two ASK1 inhibitors likely have different mechanisms of action.

## Discussion

Our GPCR/14-3-3 interaction results indicate there are two types of GPCR/14-3-3 complexes: an agonist-induced GPCR/14-3-3 complex and an apparent pre-formed GPCR/14-3-3 complex that is disrupted by agonist treatment. We have studied over two dozen GPCRs and their 14-3-3 interaction patterns are summarized in Supplementary Table 3. Since β-arrestins have been correlated with GPCR endocytosis, agonist-induced GPCR/14-3-3 interaction signals lag behind GPCR/β-arrestin interaction signals, suggesting that the recruitment of 14-3-3 proteins occurs after receptor internalization. In other cases, agonist-disrupted GPCR/14-3-3 interaction signals happen coincidently with GPCR/β-arrestin interaction signals, suggesting that some GPCRs associate with 14-3-3 even without agonist stimulation and that subsequent agonist stimulation disrupts the preassembled GPCR/14-3-3 complexes. Thus, some GPCRs can form GPCR/14-3-3 complexes at the cell membrane in an agonist-independent fashion, while other GPCRs form GPCR/14-3-3 complexes in the endosomes in an agonist-dependent fashion. We speculate that the two types of GPCR/14-3-3 association occur at different cellular locations.

GPCR trafficking and recycling involve the sorting of endocytosed receptors into carrier vesicles and their return to the plasma membrane via a process regulated by the small GTPase family of Rab proteins^20,21^. It is already known that 14-3-3 proteins interact with intracellular trafficking proteins. The Rab family of small GTPase proteins regulates many steps of membrane trafficking, including vesicle formation, vesicle movement, and membrane fusion. The Rabs function as molecular switches that alternate between the activated GTP-bound form and the inactive GDP-bound form. Phosphorylation of a Rab-GTPase activating protein (Rab-GAP) AS160 at Ser341 and Thr642 promotes the recruitment of 14-3-3. The binding leads to inhibition of the Rab-GAP-activity of AS160, shifting the equilibrium of Rabs to the active GTP-bound form, and enabling them to promote cell-surface expression of GLUT4^22^. Co-localization of 14-3-3 proteins with the Rab11-positive recycling endosomes has also been observed^23^. 14-3-3 proteins can recruit microtubule motor proteins dynein/dynactin to the client protein complex N-cadherin/β-catenin and thereby mediate the ER export of N-cadherin^24^. In addition, 14-3-3 proteins can compete with coat protein complex I (COPI) to bind client proteins for forwarding transport of proteins to the cell membrane. The C-terminal tail of human KOR contains an endoplasmic reticulum retention motif RXR adjacent to a 14-3-3 motif. Association of 14-3-3 with KOR reduces COPI binding to RXR, thus 14-3-3 binding can overcome ER retention by COPI^6^. The protein Numb participates in clathrin-dependent endocytosis by directly interacting with the clathrin-associated adaptor complex AP-2. Phosphorylation of Numb on Ser283 impairs its binding to the AP-2 complex and simultaneously recruits 14–3–3 proteins *in vitro*. Taken together, 14-3-3 plays a major role in the regulated transport of membrane proteins, including GPCRs, to the cell surface.

**Figure 7:**
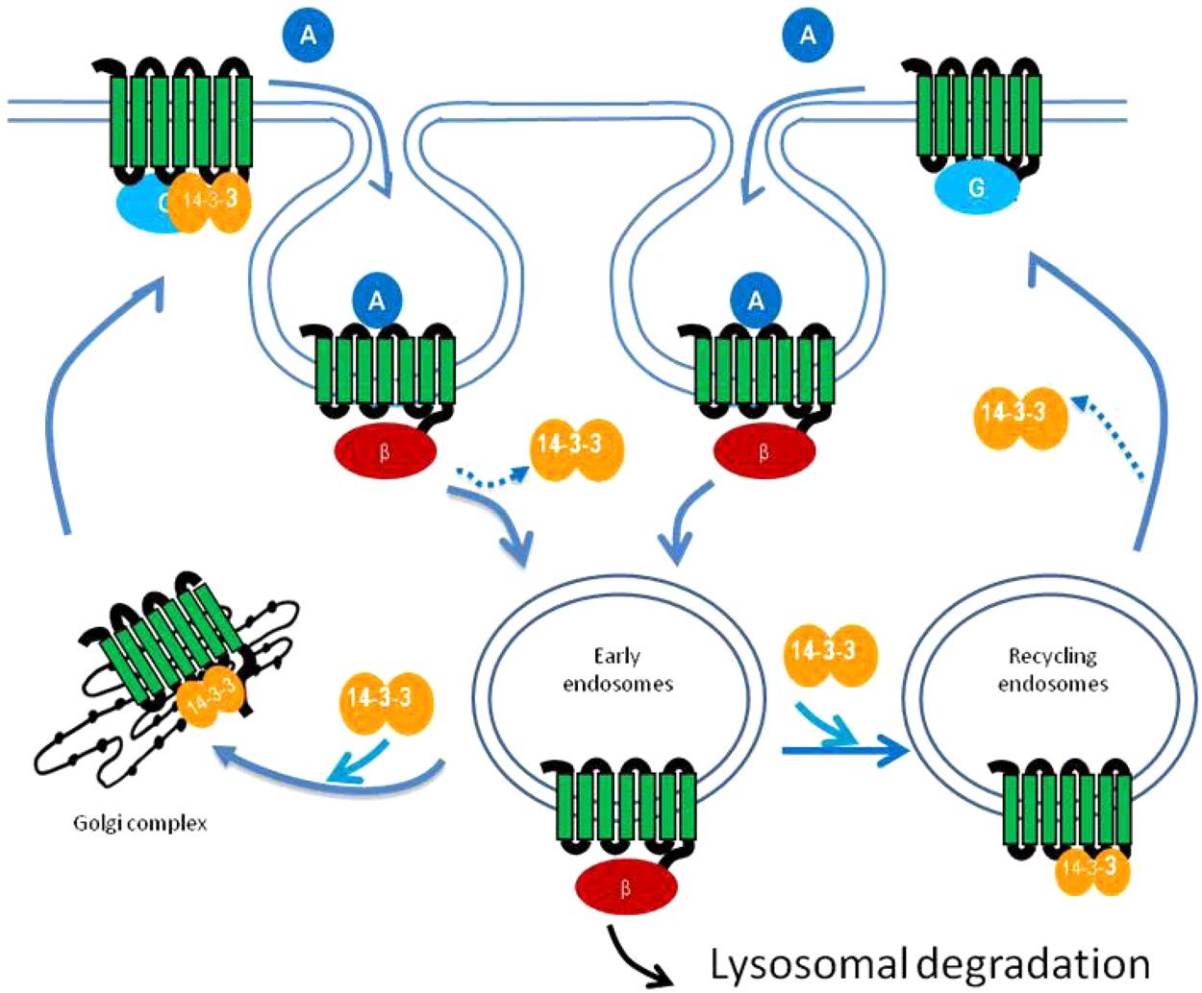
A working model of 14-3-3 roles in GPCR trafficking. Some GPCRs can associate with 14-3-3 proteins at the cell membrane. Agonist binding promotes 14-3-3 protein dissociation and recruits β-arrestins. Some GPCRs that have no 14-3-3 protein bound at the cell membrane can recruit 14-3-3 proteins after endocytosis in response to agonist treatment. 14-3-3 scaffold proteins serve as a sorting factor to direct GPCRs to recycling endosomes and trafficking to the cell membrane. However, the model does not rule out the possibility in that some GPCRs can associate with 14-3-3 proteins at the cell membrane, agonist stimulation promotes 14-3-3 dissociation and receptor endocytosis, the internalized receptors can recruit 14-3-3 proteins again to direct receptor trafficking. Abbreviations: A stands for an agonist, G stands for G-proteins, β stands for β-arrestins, and 14-3-3 stands for 14-3-3 protein.

Our findings and literature evidence lead us to propose a working model of GPCR trafficking (Fig. 7). Agonist binding leads to G protein signaling and receptor phosphorylation. Phosphorylated receptors recruit β-arrestins and undergo clathrin-mediated internalization. GPCRs preassembled with 14-3-3 proteins at the cell membrane release 14-3-3 proteins in response to agonist binding. Presumably, post-translational modifications and competing cellular proteins could play roles that lead to 14-3-3 dissociation from the preassembled GPCR/14-3-3 complexes. Internalized receptors could move to lysosomes for degradation or be retrogradely transported to the Golgi compartment, where receptors could bind 14-3-3 proteins and be transported back to the cell membrane. This trafficking pathway could also apply to receptors preassembled with 14-3-3 on the cell membrane. On the other hand, internalized GPCRs without 14-3-3 association at the cell membrane may recruit 14-3-3 proteins in endosomes. Likely, post-translational modifications, highly dynamic membrane compartments and the slightly acidic pH environment in the recycling endosomes enable GPCR/14-3-3 association there. 14-3-3 proteins serve as a sorting factor in the endosomes to direct internalized receptors to recycle back to the cell membrane. This quick and short trafficking pathway could account for rapid recycling some of the GPCRs. Potentially, a post-translational modification may lead to 14-3-3 dissociation from GPCRs during cell membrane anchoring. The two different cellular locations of 14-3-3/GPCR associations suggest at least two different recycling pathways. Our GPCR trafficking model supports the observation^25^ that GPCRs are capable of following more than one endosomal trafficking itinerary back to the cell surface. Thus, in the proposed GPCR trafficking model, ligand binding activates G protein-coupled receptor kinases and perhaps other kinases, kinases tag GPCRs with phosphates to give a signal for transportation, then 14-3-3 proteins come in to bind these tagged proteins and give an instruction for where to go, and together with trafficking proteins such as Rabs, and dynein/dynactin transport client proteins to their destinations.

The formation of GPCR/14-3-3 complexes at different locations correlate with receptor recycling and trafficking patterns. The preassembled GPCR/14-3-3 complexes at the cell membrane likely originate from the ER/Golgi complexes forward-trafficking to the cell membrane. 14-3-3 protein promoting de novo synthesized GPCRs forward-trafficking to the cell membrane has been shown for GPR15 and KOR^6,26^. The existence of the GPR15/14-3-3 complex at the cell membrane was demonstrated by the co-immunoprecipitation of 14-3-3 in the surface pool of GPR15. Co-localization of KOR with 14-3-3 at the plasma membrane and intracellular compartments have also been observed. Our naloxone-induced MOR1/14-3-3 interaction signals provide a mechanism to explain the previous observation that naloxone serves essentially as a chemical chaperone promoting MOR cell surface expression. The antagonist naloxone binds intracellular MOR1 and promotes MOR1/14-3-3 association and cell surface expression; in contrast, peptide antagonists CTAP and CTOP do not promote MOR1/14-3-3 interaction, presumably because they do not have access to the interior of the cell. GPCRs associated with 14-3-3 at the cell membrane likely undergo rapid endocytosis and slow recycling processes, moving to the lysosome for degradation or having a long recycling time through retrograde transport to the Golgi pathway. S1PR1 was shown to be quickly depleted from the cell membrane once activated^27^. The diminished S1PR1/14-3-3 interaction signals occurred in the same timeframe as the increased S1PR1/β-arrestin interaction signals in response to agonist stimulation. Similarly, GalR1 and cannabinoid receptors showed agonist-disrupted GPCR/14-3-3 interaction signals, these GPCRs are also known to be rapidly internalized following agonist binding and receptor activation^28^. Post-endocytosis, the internalized galanin receptor, and cannabinoid receptors undergo rapid degradation with sustained agonist treatment, and resensitization depends on the delivery of newly synthesized receptors^29^. Indeed, these receptors are associated with 14-3-3 at the cell membrane as we observed.

On the other hand, the agonist-promoted GPCR/14-3-3 association in endosomes is coincidently linked to the recycling of internalized receptors. We have shown agonist-induced GPCR/14-3-3 interaction signals for CCKAR^30^, KISS1R^31^, CHRM3, CHRM5^32^, 5HT2a^33^, and PTHR^34^. These receptors are known to recycle back to the plasma membrane. For example, while the ligand cholecystokinin (CCK) sorted to lysosomes, no CCKAR degradation was observed during recycling^30^. Similarly, the bulk of internalized KISS1R was recycled back to the membrane with little degradation^31^. The phenomenon of these receptors recycling back to the cell membrane linked to their interaction with 14-3-3 proteins in the endosomes suggests the 14-3-3 proteins serve as a sorting factor in the endosomes to direct internalized receptor to recycling endosomes. However, we cannot rule out the possibility in that some receptors can associate with 14-3-3 at the cell membrane, dissociate 14-3-3 proteins upon agonist treatment, and recruit 14-3-3 proteins again in the endosomes, the LinkLight assay will not be able to assess the GPCR/14-3-3 interaction signals in this situation. Time-sensitive, location-sensitive detection and signal sensitivity as determined by the population of GPCR/14-3-3 association status remain big challenges; 14-3-3 is such a ubiquitously expressed protein that confocal imaging experiments to demonstrate co-localization and movement of GPCRs coupled to 14-3-3 are drowned by the large signal from the total 14-3-3 pool.

Post-translational modifications may play roles for these two types of GPCR/14-3-3 binding modes. Phosphorylation of serine and threonine sites of 14-3-3 binding motifs can enhance binding affinity^35^. However, phosphorylation-independent binding may also be present. Phosphorylation of a mode III of 14-3-3 binding motif at the C-terminal tail of GPR15 is required for 14-3-3 binding and cell surface expression^26^. But, phosphorylation of the putative 14-3-3 motif on the KOR is not required for KOR/14-3-3 association^6^. The non-phosphorylated C-terminal tail of KOR can bind 14-3-3 proteins, suggesting some diversity in 14-3-3 mediated forward trafficking of membrane proteins. In addition, phosphorylation of different residues adjacent to the putative 14-3-3 binding motifs could inhibit the recruitment of 14-3-3 proteins. For example, phosphorylation of a second serine residue in the C-terminus of TASK-1 inhibits 14-3-3 binding and blocks the transport of TASK-1 to the cell membrane^36^. Potentially, other scaffolding proteins could block, compete or enhance 14-3-3 protein access to client proteins.

The cytoplasmic regions of GPCRs harbor multiple binding motifs that facilitate recruitment, release, and exchange of different cellular effectors. β-arrestins, 14-3-3 proteins and others are such cellular effectors to interact with GPCRs and modulate signaling and trafficking. PSD95/Disc large/Zona occludens (PDZ) domain-containing proteins are known to interact with some GPCRs^37^. It is estimated that 20% of GPCRs have PDZ-binding motifs^38^. The ADRB2 contains a “recycling sequence” for a PDZ ligand which partitions into actin stabilized, retromer tubules via interaction with Sorting Nexin 27^39^. PSD-95 has been suggested to form a complex with GPR30 and to promote GPR30 membrane localization. The PDZ domain-containing protein NHERF1 is reported to facilitate the endocytosis of a number of GPCRs. NHERF1 enhances chemokine receptor 5 (CCR5) endocytosis and β-arrestin1 recruitment. CHRM1 and CHRM3 have no PDZ-binding motif and their recycling is not through PDZdomain-containing proteins. In addition, the somatostatin receptor subtype 5 (SSTR5) associates with a PDZ protein and the association inhibited SSTR5 recycling^40^. PDZdomain-containing proteins appear to play a complex role in regulating the endocytosis and recycling of a variety of different GPCRs. Recycling is a multi-level complex network of pathways. It is very likely that multiple scaffolding proteins are required for regulating recycling and trafficking between intracellular membrane compartments, following endocytosis, and as well as transport back to the cell surface. Given the vast number and diversity of GPCRs, sorting of distinct GPCRs in the endosomal system is regulated by combination, integration, and competition of scaffolding proteins and post-translational modifications and depends on the cellular context and the pathways available in a particular cell type. Based on the fact that the C-terminal and intracellular loops contain elements regulating receptor trafficking and recycling, an assay development practice of fusing a GPCR intracellular sequence to another GPCR to boost GPCR/β-arrestin or GPCR/14-3-3 interaction signals could alter GPCR recycling/trafficking pattern and generate artificial results.

To explore the question of how universal GPCR/14-3-3 interaction is, we applied the 1433pred tool^14^ in our bioinformatic analyses. The analyses predict a large number of GPCRs containing putative 14-3-3 binding motifs (Supplemental Table S1). First, 358 (~90%) out of 399 GPCRs analyzed contain at least one putative 14-3-3 binding motif in the cytoplasmic regions, suggesting a common phenomenon of GPCR/14-3-3 association. Many GPCRs have multiple 14-3-3 binding motifs (Supplemental Table S2). It is likely some of them bind to 14-3-3 proteins. Second, GPCRs with 14-3-3 binding motifs are not limited to neurotransmitter receptors or neuronal GPCRs, but rather include almost all classes neuronal or non-neuronal GPCRs. Third, a large number of orphan GPCRs, including the elusive cadherin EGF LAG seven-pass G-type receptor family, the adhesion GPCR family, the leucine-rich repeat-containing GPCR family, and frizzled GPCR family, all have a large number of putative 14-3-3 binding motifs. For many of these receptors, signaling pathways have not been identified or characterized. It is also interesting to note that 14-3-3 binding sites share substantial homology with many kinase recognition sites. However, what factors determine GPCR/14-3-3 association at the cell membrane or in the endosomes remain to be elucidated. There are also phosphorylation-independent and non-canonical 14-3-3 binding possibilities. Their roles in GPCR/14-3-3 interactions have yet to be defined. In addition, seven members of the 14-3-3 protein family may have preferences for particular GPCRs and may play different physiological roles. Based on 14-3-3pred tool analysis of DRD5, there are three putative 14-3-3 binding motifs with four potential phosphorylation serine and threonine sites identified. Mutagenesis study showed only the 443 residue threonine in the C-terminal tail is involved in the binding of 14-3-3, potentially through phosphorylation. Clearly, the bioinformatic analysis is a very useful tool to identify putative 14-3-3 motifs, but actual binding motifs will have to be confirmed by experiments.

It has become increasingly evident that some GPCRs have sustained the ability to signal in the endosomal compartments. GPCRs can activate G-protein-dependent as well as G-protein-independent signaling pathways in the endosomes. G-protein-independent signals are mainly through GPCR/scaffold complexes that regulate MAPK signaling. Previously, we have shown that β-arrestin can associate with ERK2^16^ in response to GPCR agonist stimulation, suggesting a non-canonical MAPK pathway mediated by β-arrestins. Additionally, we have shown that 14-3-3 can associate with Raf1 and ASK1 in response to GPCR agonist stimulation. The two kinase signaling cascades seem to be apparently connected through 14-3-3 proteins. It is likely these connections facilitate the formation of large molecular complexes that coordinate responses of multiple signaling pathways to incoming stimuli, allowing signal transduction between different cellular compartments. Thus, in endosomal signaling pathways, multiple cellular components and signaling cascades are involved. GPCRs in endosomes could generate 14-3-3-mediated signals in various subcellular compartments in addition to well-known G-proteins and β-arrestin-mediated signals. Given the fact that a very diverse and large number of proteins such as receptors, channels, intracellular kinases, nuclear transcription factors interact with 14-3-3 proteins, modulating GPCR/14-3-3 association could send signals through 14-3-3 interaction networks and modulate cellular functions. Our previous results that GPCR activation can promote Raf-1/14-3-3 association^4^ and current results that GPCR activation can also promote ASK1/14-3-3 association suggest multiple kinase cascades could be modulated through 14-3-3 signal adaptor proteins. The kinase/14-3-3 association could play roles in the endosomal signaling.

Importantly, our data with BMS986122 indicate that it is possible to identify compounds that can differentially modulate GPCR/14-3-3 and GPCR/β-arrestin signaling pathways. These observations have fundamental implications for GPCR pharmacology and suggest new mechanisms that could be exploited in GPCR-directed pharmacotherapy. For example, MOR1 trafficking has been linked to opioid tolerance after acute exposure to agonist, but it is also involved in the resensitization process. Naloxone is a life-saving medication to treat opioid overdose. Naltrexone has been used to treat both alcohol and opioid drug dependence. Our finding that both naloxone and naltrexone promote MOR1/14-3-3 interaction signals indicates GPCR recycling and trafficking processes could be modulated through GPCR/14-3-3 interaction. Thus, understanding the diversity of signaling at opioid receptors and how receptor trafficking and recycling leads to modulation of pain and reward can lead us to identify novel opioid receptor drug candidates that may have a more targeted pharmacological response or overcome undesirable side effects.

Identifying the molecular machinery that regulates trafficking of distinct GPCRs will enable the development of new strategies to manipulate receptor signaling and recycling, to discover more efficacious and safer drugs. This work opens new possibilities for screening drug leads that regulate GPCR trafficking and endosomal signaling pathways.

## Materials and Methods

Compounds and chemicals were purchased from Sigma-Aldrich (St. Louis, MO, USA), AdooQ (Irvine, CA, USA), AOBIOUS (Gloucester, MA, USA) and Tocris Biosciences (Bristol, UK).

### Cell lines and cell culture

HEK293 cells were routinely maintained and passaged in standard DMEM with 10% FBS and Pen/Strep (Gibco catalog # 15070). Cells were cultured in a 37 °C incubator with 5% CO2. Cell culture medium was replaced every three to four days, and cells were passaged at 90% confluence. Stable GPCR/14-3-3 and GPCR/β-arrestin LinkLight cells are maintained with HEK293 culture media with 400 μg/ml G418 and 100 μg/ml Hygromycin B.

### Plasmid construction and generation of stable cell lines

Full-length cDNAs of human GPCRs without stop codons were subcloned in frame with the TEV protease vector as previously described^11^. The β-arrestin-2-permuted luciferase (β-arr-2-pLuc) expression plasmid was also previously described^4^. 14-3-3ɛ and 14-3-3β full-length cDNAs without stop codons were used to replace β-arrestin-2 in the β-arr-2-pLuc for construction of the 14-3-3ɛ-pLuc and 14-3-3β-pLuc expression plasmids. Transfections of HEK293 cells were performed with PEI transfection reagent (catalog # 23966-1, Polysciences). Monoclonal cell lines stably expressing both GPCR-TEV and the 14-3-3ɛ-pLuc fusion protein were selected using 400 μg/ml G418 (Invitrogen/Life Technologies, catalog # 10131-027) and 100 μg/ml Hygromycin B (Life Technologies, catalog # 10687-010). Multiple stable clones were selected for evaluation.

### Luciferase assay

The LinkLight cells stably expressing both GPCR-TEV and 14-3-3ɛ -pLuc were seeded into a poly-D-lysine coated 384-well white plate (Becton Dickinson, 356660) at 20,000 cells per well with 30 μl of the regular medium. Cells were cultured overnight. The following day, the culture medium was replaced with 15 μl/well of serum-free DMEM. Then, the stimulant or agonist was added at 5 μl/well and incubated for 240 min or for other times as indicated. An equal volume of luciferase detection reagents such as Bright-Glo (Promega), One-Glo (Promega), BriteLite (Perkin Elmer) or NeoLite (Perkin Elmer) was added for a 1 to 3-minute incubation. The luminescence signals were recorded using a luminescence plate reader (EnSpire or EnVision). Optimum luminescent signals were observed between 2 to 10 minutes after adding luciferase detection reagent.

### Measurement of the effects of ligands on GPCR/14-3-3 signaling

The GPCR/14-3-3 LinkLight cells were seeded in poly-D-lysine coated white 384-well plates. After overnight culture, the culture medium was replaced with 15 μl of serum-free DMEM, and a serial 1:3 dilution of GPCR ligand (5 µl/well) was added to the cells. After agonist incubation in a 37 °C, 5% CO2 incubator (240 min for agonist-induced GPCR/14-3-3 interaction signals and 180 min for agonist-inhibited GPCR/14-3-3 interaction signals, unless otherwise specified), a luciferase detection reagent (10 µl/well) was added to the cells, and luminescence signals were recorded.

### Measurement of agonist-induced GPCR/β-arrestin-2 signaling

The GPCR/β-arrestin-2 LinkLight cells were seeded in a poly-D-lysine coated white 384-well plates. After overnight culture, the culture medium was replaced with 15 μl of serum-free DMEM, and a serial 1:3 dilution of GPCR ligand (5 µl/well) was added to the cells. After agonist incubation in a 37 °C, 5% CO2 incubator (120 min unless otherwise specified), a luciferase detection reagent (10 µl/well) was added to the cells, and luminescence signals were recorded.

Construction of DRD5-TEV point mutation expression plasmids and analysis their interaction signals with 14-3-3 proteins in response to dopamine by transient expression.

Site-directed mutagenesis and overlap PCR primers containing nucleotide point mutations were used to generate PCR fragments converting S260, S261 to A260, A261 (S260A-S261A), T443 to A443 (T443A), and S464 to A464 (T464A) respectively. The mutated PCR fragments were then cloned into a vector inframe with TEV sequence and DNA sequence determination was performed to ensure the correct generation of all mutants. The wild type DRD5-TEV and mutant DRD5-TEV expression plasmids were transfected into 14-3-3-pLuc reporter host cells (600,000 cells/well) in 6-well plates (3 μg plasmid/well) by PEI transfection reagent (catalog # 23966-1, Polysciences). After overnight culture, the transfected cells were split into a white 384-well poly-D-Lysine-coated plate, 24 wells per transfected plasmid, with 20,000 cell/35 μl/well and continuing cultures. After overnight culture, 12 wells were added 5 μl/well of 80 μM dopamine (final concentration: 10 μM) and 12 wells 5 μl/well of DMEM media as background controls. After incubation of four hours in a 37 °C, 5% CO2 incubator, a luciferase assay was performed. The transient expression experiments were repeated three times and results were repeatable.

### Measurement of GPCR agonist-induced ASK11/14-3-3 interaction

The monoclonal ASK1/14-3-3 LinkLight cells were generated by stable expression of ASK1-TEV plasmid in the 14-3-3-pLuc HEK293 host cells with hygromycin B (100 μg/ml) selection. The ASK1/14-3-3 LinkLight cells were seeded in a poly-D-lysine coated white 384-well plates with 20,000 cells in 35 μl/well. After overnight culture, the culture medium was replaced with 25 μl of serum-free DMEM/well, and a serial 1:3 dilution of adrenaline, carbachol, and NECA (5 µl/well and 6 fold of the final concentration) was added to the cells. After designated times of ligand incubation in a 37 °C, 5% CO2 incubator, a luciferase detection reagent (20 µl/well) was added to the cells, and luminescence signals were recorded.

### Measurement of ASK1 inhibitor responses

For ASK1 inhibitor assays, ASK1/14-3-3 LinkLight cells were seeded in a 384-well plate. After overnight cultures, 5 μl of inhibitors were added to each well started with 30 μM (final concentration) then a 1:3 serial dilutions. After incubation of three hours in a 37 °C, 5% CO2 incubator, a luciferase detection reagent (20 µl/well) was added to the cells, and luminescence signals were recorded.

### Bioinformatic analysis

To search for 14-3-3 motif across amino acid sequences of GPCRs, we worked with UniProt protein identifiers and sequences. We marked every hit along with GPCR sequences in cytosolic regions only. We also marked the position of the hits in relation to the GPCRdb numbering scheme. GPCRdb Numbering is based on the Ballesteros-Weinstein Numbering scheme^15^. We detected 14-3-3 motifs with the 14-3-3 motif prediction tool^14^ that scores the hits based on resemblance to known 14-3-3 motifs. We used three methods: the artificial neural network (ANN), position-specific scoring matrix (PSSM), and support vector machines (SVM) to show how several score thresholds affect the results. The consensus score is the average of the three aforementioned methods.

Dose-response curve data analysis: Concentration-response curves were analyzed using Prism software (GraphPad Software, Inc. San Diego, CA). All values are expressed as the mean ± SD (n = 3).

## Supplementary information

Supplementary table 1

Supplementary table 2

Supplementary table 3

## Data Availability

The “target-open” LinkLight assay system in which researchers can generate their interested target assays, as well as aforementioned assays, can be purchased from BioInvenu with Material Transfer Agreements.
