# Supplementary material for: 14-3-3 signal adaptor and scaffold proteins mediate GPCR trafficking
Source: Sci Rep. 2019 Aug 1;9:11156. doi: 10.1038/s41598-019-47478-w (PMC6673703; doi:10.1038/s41598-019-47478-w)
Supplement: Supplementary file 1 — Supplementary table 1 [file 41598_2019_47478_MOESM1_ESM.pdf]

**14-3-3 signal adaptor and scaffold proteins mediate GPCR trafficking**

Luwa Yuan[1], Shahar Barbash[2], Sathapana Kongsamut[1], Alex Eishingdrelo[1], Thomas P. Sakmar[2,3], Haifeng Eishingdrelo[1*]

| GPCR | Site | Peptide | ANN | PSSM | SVM | Consensus |
|---|---|---|---|---|---|---|
| sp\|P28222\|5HT1B_HUMAN 5-hydroxytryptamine receptor 1B OS=Homo sapiens GN=HTR1B PE=1 SV=1 | 389 | LIRFKCtS--- | 0.405 | 0.385 | -0.515 | 0.092 |
| sp\|P28222\|5HT1B_HUMAN 5-hydroxytryptamine receptor 1B OS=Homo sapiens GN=HTR1B PE=1 SV=1 | 390 | IRFKCTs---- | 0.611 | 0.427 | -0.025 | 0.338 |
| sp\|P28221\|5HT1D_HUMAN 5-hydroxytryptamine receptor 1D OS=Homo sapiens GN=HTR1D PE=1 SV=1 | 377 | VPFRKAs---- | 0.533 | 0.902 | 0.004 | 0.48 |
| sp\|P28566\|5HT1E_HUMAN 5-hydroxytryptamine receptor 1E OS=Homo sapiens GN=HTR1E PE=1 SV=1 | 365 | IRCREHt---- | 0.817 | 1.3 | 0.592 | 0.903 |
| sp\|P28223\|5HT2A_HUMAN 5-hydroxytryptamine receptor 2A OS=Homo sapiens GN=HTR2A PE=1 SV=2 | 386 | YTLFNKtYRSA | 0.213 | -0.142 | -0.549 | -0.159 |
| sp\|P28223\|5HT2A_HUMAN 5-hydroxytryptamine receptor 2A OS=Homo sapiens GN=HTR2A PE=1 SV=2 | 389 | FNKTYRsAFSR | 0.156 | -0.014 | -0.961 | -0.273 |
| sp\|P28223\|5HT2A_HUMAN 5-hydroxytryptamine receptor 2A OS=Homo sapiens GN=HTR2A PE=1 SV=2 | 392 | TYRSAFsRYIQ | 0.425 | 0.16 | -0.483 | 0.034 |
| sp\|P28223\|5HT2A_HUMAN 5-hydroxytryptamine receptor 2A OS=Homo sapiens GN=HTR2A PE=1 SV=2 | 413 | QLILVNtIPAL | 0.726 | 0.681 | 0.336 | 0.581 |
| sp\|P28223\|5HT2A_HUMAN 5-hydroxytryptamine receptor 2A OS=Homo sapiens GN=HTR2A PE=1 SV=2 | 421 | PALAYKsSQLQ | 0.267 | -0.076 | -0.596 | -0.135 |
| sp\|P28223\|5HT2A_HUMAN 5-hydroxytryptamine receptor 2A OS=Homo sapiens GN=HTR2A PE=1 SV=2 | 422 | ALAYKSsQLQM | 0.101 | -0.187 | -1.105 | -0.397 |
| sp\|P28223\|5HT2A_HUMAN 5-hydroxytryptamine receptor 2A OS=Homo sapiens GN=HTR2A PE=1 SV=2 | 432 | MGQKKNsKQDA | 0.602 | 0.189 | 0.012 | 0.268 |
| sp\|P28223\|5HT2A_HUMAN 5-hydroxytryptamine receptor 2A OS=Homo sapiens GN=HTR2A PE=1 SV=2 | 438 | SKQDAKtTDND | 0.589 | 0.279 | -0.135 | 0.244 |
| sp\|P28223\|5HT2A_HUMAN 5-hydroxytryptamine receptor 2A OS=Homo sapiens GN=HTR2A PE=1 SV=2 | 439 | KQDAKTtDNDC | 0.151 | -0.154 | -0.993 | -0.332 |
| sp\|P28223\|5HT2A_HUMAN 5-hydroxytryptamine receptor 2A OS=Homo sapiens GN=HTR2A PE=1 SV=2 | 444 | TTDNDCsMVAL | 0.182 | 0.065 | -0.919 | -0.224 |
| sp\|P28223\|5HT2A_HUMAN 5-hydroxytryptamine receptor 2A OS=Homo sapiens GN=HTR2A PE=1 SV=2 | 453 | ALGKQHsEEAS | 0.401 | 0.29 | -0.413 | 0.093 |
| sp\|P28223\|5HT2A_HUMAN 5-hydroxytryptamine receptor 2A OS=Homo sapiens GN=HTR2A PE=1 SV=2 | 457 | QHSEEAsKDNS | 0.121 | -0.081 | -1.468 | -0.476 |
| sp\|P28223\|5HT2A_HUMAN 5-hydroxytryptamine receptor 2A OS=Homo sapiens GN=HTR2A PE=1 SV=2 | 461 | EASKDNsDGVN | 0.413 | 0.074 | -0.63 | -0.048 |
| sp\|P28223\|5HT2A_HUMAN 5-hydroxytryptamine receptor 2A OS=Homo sapiens GN=HTR2A PE=1 SV=2 | 469 | GVNEKVsCV-- | 0.15 | 0.134 | -1.123 | -0.28 |
| sp\|P41595\|5HT2B_HUMAN 5-hydroxytryptamine receptor 2B OS=Homo sapiens GN=HTR2B PE=1 SV=1 | 386 | YTLFNKtFRDA | 0.636 | 0.093 | 0.237 | 0.322 |
| sp\|P41595\|5HT2B_HUMAN 5-hydroxytryptamine receptor 2B OS=Homo sapiens GN=HTR2B PE=1 SV=1 | 396 | AFGRYItCNYR | 0.712 | 0.905 | 0.486 | 0.701 |
| sp\|P41595\|5HT2B_HUMAN 5-hydroxytryptamine receptor 2B OS=Homo sapiens GN=HTR2B PE=1 SV=1 | 402 | TCNYRAtKSVK | 0.319 | 0.099 | -0.669 | -0.084 |
| sp\|P41595\|5HT2B_HUMAN 5-hydroxytryptamine receptor 2B OS=Homo sapiens GN=HTR2B PE=1 SV=1 | 404 | NYRATKsVKTL | 0.484 | 0.336 | 0.054 | 0.291 |
| sp\|P41595\|5HT2B_HUMAN 5-hydroxytryptamine receptor 2B OS=Homo sapiens GN=HTR2B PE=1 SV=1 | 407 | ATKSVKtLRKR | 0.495 | -0.039 | -0.241 | 0.072 |
| sp\|P41595\|5HT2B_HUMAN 5-hydroxytryptamine receptor 2B OS=Homo sapiens GN=HTR2B PE=1 SV=1 | 412 | KTLRKRsSKIY | 0.459 | 0.688 | -0.3 | 0.282 |
| sp\|P41595\|5HT2B_HUMAN 5-hydroxytryptamine receptor 2B OS=Homo sapiens GN=HTR2B PE=1 SV=1 | 413 | TLRKRSsKIYF | 0.552 | 0.356 | 0.046 | 0.318 |
| sp\|P41595\|5HT2B_HUMAN 5-hydroxytryptamine receptor 2B OS=Homo sapiens GN=HTR2B PE=1 SV=1 | 425 | NPMAENsKFFK | 0.155 | -0.176 | -1.063 | -0.361 |
| sp\|P41595\|5HT2B_HUMAN 5-hydroxytryptamine receptor 2B OS=Homo sapiens GN=HTR2B PE=1 SV=1 | 444 | NPAMYQsPMRL | 0.105 | -0.41 | -1.209 | -0.505 |
| sp\|P41595\|5HT2B_HUMAN 5-hydroxytryptamine receptor 2B OS=Homo sapiens GN=HTR2B PE=1 SV=1 | 450 | SPMRLRsSTIQ | 0.269 | 0.611 | -0.775 | 0.035 |
| sp\|P41595\|5HT2B_HUMAN 5-hydroxytryptamine receptor 2B OS=Homo sapiens GN=HTR2B PE=1 SV=1 | 451 | PMRLRSsTIQS | 0.293 | 0.354 | -0.438 | 0.07 |
| sp\|P41595\|5HT2B_HUMAN 5-hydroxytryptamine receptor 2B OS=Homo sapiens GN=HTR2B PE=1 SV=1 | 452 | MRLRSStIQSS | 0.633 | 1.199 | 0.648 | 0.827 |
| sp\|P41595\|5HT2B_HUMAN 5-hydroxytryptamine receptor 2B OS=Homo sapiens GN=HTR2B PE=1 SV=1 | 455 | RSSTIQsSSII | 0.174 | -0.145 | -0.817 | -0.263 |
| sp\|P41595\|5HT2B_HUMAN 5-hydroxytryptamine receptor 2B OS=Homo sapiens GN=HTR2B PE=1 SV=1 | 456 | SSTIQSsSIIL | 0.044 | -0.225 | -1.506 | -0.562 |
| sp\|P41595\|5HT2B_HUMAN 5-hydroxytryptamine receptor 2B OS=Homo sapiens GN=HTR2B PE=1 SV=1 | 457 | STIQSSsIILL | 0.15 | 0.075 | -0.665 | -0.147 |
| sp\|P41595\|5HT2B_HUMAN 5-hydroxytryptamine receptor 2B OS=Homo sapiens GN=HTR2B PE=1 SV=1 | 463 | SIILLDtLLLT | 0.103 | -0.092 | -1.338 | -0.442 |
| sp\|P41595\|5HT2B_HUMAN 5-hydroxytryptamine receptor 2B OS=Homo sapiens GN=HTR2B PE=1 SV=1 | 467 | LDTLLLtENEG | 0.103 | -0.091 | -0.962 | -0.317 |
| sp\|P41595\|5HT2B_HUMAN 5-hydroxytryptamine receptor 2B OS=Homo sapiens GN=HTR2B PE=1 SV=1 | 474 | ENEGDKtEEQV | 0.163 | -0.087 | -0.714 | -0.213 |
| sp\|P41595\|5HT2B_HUMAN 5-hydroxytryptamine receptor 2B OS=Homo sapiens GN=HTR2B PE=1 SV=1 | 479 | KTEEQVsYV-- | 0.111 | -0.057 | -1.302 | -0.416 |
| sp\|P28335\|5HT2C_HUMAN 5-hydroxytryptamine receptor 2C OS=Homo sapiens GN=HTR2C PE=1 SV=1 | 380 | IYRRAFsNYLR | 0.902 | 1.249 | 1.094 | 1.082 |
| sp\|P28335\|5HT2C_HUMAN 5-hydroxytryptamine receptor 2C OS=Homo sapiens GN=HTR2C PE=1 SV=1 | 404 | IPRVAAtALSG | 0.31 | 0.219 | -0.707 | -0.059 |
| sp\|P28335\|5HT2C_HUMAN 5-hydroxytryptamine receptor 2C OS=Homo sapiens GN=HTR2C PE=1 SV=1 | 407 | VAATALsGREL | 0.16 | -0.165 | -0.839 | -0.281 |
| sp\|P28335\|5HT2C_HUMAN 5-hydroxytryptamine receptor 2C OS=Homo sapiens GN=HTR2C PE=1 SV=1 | 419 | VNIYRHtNEPV | 0.245 | 0.076 | -0.903 | -0.194 |
| sp\|P28335\|5HT2C_HUMAN 5-hydroxytryptamine receptor 2C OS=Homo sapiens GN=HTR2C PE=1 SV=1 | 428 | PVIEKAsDNEP | 0.136 | -0.005 | -1.05 | -0.306 |
| sp\|P28335\|5HT2C_HUMAN 5-hydroxytryptamine receptor 2C OS=Homo sapiens GN=HTR2C PE=1 SV=1 | 448 | ELPVNPsSVVS | 0.071 | -0.144 | -1.262 | -0.445 |
| sp\|P28335\|5HT2C_HUMAN 5-hydroxytryptamine receptor 2C OS=Homo sapiens GN=HTR2C PE=1 SV=1 | 449 | LPVNPSsVVSE | 0.03 | -0.321 | -1.461 | -0.584 |

| | | | | | | |
|---|---|---|---|---|---|---|
| sp\|P28335\|5HT2C_HUMAN 5-hydroxytryptamine receptor 2C OS=Homo sapiens GN=HTR2C PE=1 SV=1 | 452 | NPSSVVsERIS | 0.273 | -0.173 | -0.872 | -0.257 |
| sp\|P28335\|5HT2C_HUMAN 5-hydroxytryptamine receptor 2C OS=Homo sapiens GN=HTR2C PE=1 SV=1 | 456 | VVSERIsSV-- | 0.139 | 0.115 | -0.974 | -0.24 |
| sp\|P28335\|5HT2C_HUMAN 5-hydroxytryptamine receptor 2C OS=Homo sapiens GN=HTR2C PE=1 SV=1 | 457 | VSERISsV--- | 0.458 | 0.827 | 0.191 | 0.492 |
| sp\|Q13639\|5HT4R_HUMAN 5-hydroxytryptamine receptor 4 OS=Homo sapiens GN=HTR4 PE=1 SV=2 | 318 | YAFLNKsFRRA | 0.385 | -0.08 | -0.349 | -0.015 |
| sp\|Q13639\|5HT4R_HUMAN 5-hydroxytryptamine receptor 4 OS=Homo sapiens GN=HTR4 PE=1 SV=2 | 338 | ERYRRPsILGQ | 0.501 | 0.974 | 0.278 | 0.584 |
| sp\|Q13639\|5HT4R_HUMAN 5-hydroxytryptamine receptor 4 OS=Homo sapiens GN=HTR4 PE=1 SV=2 | 343 | PSILGQtVPCS | 0.342 | 0.431 | -0.376 | 0.132 |
| sp\|Q13639\|5HT4R_HUMAN 5-hydroxytryptamine receptor 4 OS=Homo sapiens GN=HTR4 PE=1 SV=2 | 347 | GQTVPCsTTTI | 0.253 | -0.098 | -0.684 | -0.176 |
| sp\|Q13639\|5HT4R_HUMAN 5-hydroxytryptamine receptor 4 OS=Homo sapiens GN=HTR4 PE=1 SV=2 | 348 | QTVPCStTTIN | 0.18 | -0.193 | -1.118 | -0.377 |
| sp\|Q13639\|5HT4R_HUMAN 5-hydroxytryptamine receptor 4 OS=Homo sapiens GN=HTR4 PE=1 SV=2 | 349 | TVPCSTtTING | 0.205 | 0.118 | -0.69 | -0.122 |
| sp\|Q13639\|5HT4R_HUMAN 5-hydroxytryptamine receptor 4 OS=Homo sapiens GN=HTR4 PE=1 SV=2 | 350 | VPCSTTtINGS | 0.081 | -0.22 | -1.076 | -0.405 |
| sp\|Q13639\|5HT4R_HUMAN 5-hydroxytryptamine receptor 4 OS=Homo sapiens GN=HTR4 PE=1 SV=2 | 354 | TTTINGsTHVL | 0.154 | -0.078 | -0.997 | -0.307 |
| sp\|Q13639\|5HT4R_HUMAN 5-hydroxytryptamine receptor 4 OS=Homo sapiens GN=HTR4 PE=1 SV=2 | 355 | TTINGStHVLR | 0.067 | -0.178 | -1.236 | -0.449 |
| sp\|Q13639\|5HT4R_HUMAN 5-hydroxytryptamine receptor 4 OS=Homo sapiens GN=HTR4 PE=1 SV=2 | 370 | CGGQWEsQCHP | 0.069 | -0.317 | -1.301 | -0.516 |
| sp\|Q13639\|5HT4R_HUMAN 5-hydroxytryptamine receptor 4 OS=Homo sapiens GN=HTR4 PE=1 SV=2 | 377 | QCHPPAtSPLV | 0.377 | 0.327 | -0.599 | 0.035 |
| sp\|Q13639\|5HT4R_HUMAN 5-hydroxytryptamine receptor 4 OS=Homo sapiens GN=HTR4 PE=1 SV=2 | 378 | CHPPATsPLVA | 0.061 | -0.367 | -1.485 | -0.597 |
| sp\|Q13639\|5HT4R_HUMAN 5-hydroxytryptamine receptor 4 OS=Homo sapiens GN=HTR4 PE=1 SV=2 | 386 | LVAAQPsDT-- | 0.145 | 0 | -1.023 | -0.293 |
| sp\|Q13639\|5HT4R_HUMAN 5-hydroxytryptamine receptor 4 OS=Homo sapiens GN=HTR4 PE=1 SV=2 | 388 | AAQPSDt---- | 0.278 | 0.156 | -0.734 | -0.1 |
| sp\|P47898\|5HT5A_HUMAN 5-hydroxytryptamine receptor 5A OS=Homo sapiens GN=HTR5A PE=2 SV=1 | 347 | FNKNYNsAFKN | 0.302 | 0.099 | -0.56 | -0.053 |
| sp\|P47898\|5HT5A_HUMAN 5-hydroxytryptamine receptor 5A OS=Homo sapiens GN=HTR5A PE=2 SV=1 | 354 | AFKNFFsRQH- | 0.187 | -0.098 | -0.679 | -0.197 |
| sp\|P50406\|5HT6R_HUMAN 5-hydroxytryptamine receptor 6 OS=Homo sapiens GN=HTR6 PE=1 SV=1 | 347 | PRERQAsLASP | 0.599 | 1.028 | 0.342 | 0.656 |
| sp\|P50406\|5HT6R_HUMAN 5-hydroxytryptamine receptor 6 OS=Homo sapiens GN=HTR6 PE=1 SV=1 | 350 | RQASLAsPSLR | 0.055 | -0.415 | -1.415 | -0.592 |
| sp\|P50406\|5HT6R_HUMAN 5-hydroxytryptamine receptor 6 OS=Homo sapiens GN=HTR6 PE=1 SV=1 | 352 | ASLASPsLRTS | 0.408 | -0.026 | -0.247 | 0.045 |
| sp\|P50406\|5HT6R_HUMAN 5-hydroxytryptamine receptor 6 OS=Homo sapiens GN=HTR6 PE=1 SV=1 | 355 | ASPSLRtSHSG | 0.034 | -0.383 | -2.004 | -0.784 |
| sp\|P50406\|5HT6R_HUMAN 5-hydroxytryptamine receptor 6 OS=Homo sapiens GN=HTR6 PE=1 SV=1 | 356 | SPSLRTsHSGP | 0.11 | -0.178 | -1.213 | -0.427 |
| sp\|P50406\|5HT6R_HUMAN 5-hydroxytryptamine receptor 6 OS=Homo sapiens GN=HTR6 PE=1 SV=1 | 358 | SLRTSHsGPRP | 0.86 | 0.889 | 0.566 | 0.772 |
| sp\|P50406\|5HT6R_HUMAN 5-hydroxytryptamine receptor 6 OS=Homo sapiens GN=HTR6 PE=1 SV=1 | 365 | GPRPGLsLQQV | 0.361 | 0.136 | -0.517 | -0.007 |
| sp\|P50406\|5HT6R_HUMAN 5-hydroxytryptamine receptor 6 OS=Homo sapiens GN=HTR6 PE=1 SV=1 | 378 | LPLPPDsDSDS | 0.087 | -0.302 | -1.309 | -0.508 |
| sp\|P50406\|5HT6R_HUMAN 5-hydroxytryptamine receptor 6 OS=Homo sapiens GN=HTR6 PE=1 SV=1 | 380 | LPPDSDsDSDA | 0.245 | 0.011 | -0.879 | -0.208 |
| sp\|P50406\|5HT6R_HUMAN 5-hydroxytryptamine receptor 6 OS=Homo sapiens GN=HTR6 PE=1 SV=1 | 382 | PDSDSDsDAGS | 0.191 | -0.082 | -1.001 | -0.297 |
| sp\|P50406\|5HT6R_HUMAN 5-hydroxytryptamine receptor 6 OS=Homo sapiens GN=HTR6 PE=1 SV=1 | 386 | SDSDAGsGGSS | 0.048 | -0.298 | -1.66 | -0.637 |
| sp\|P50406\|5HT6R_HUMAN 5-hydroxytryptamine receptor 6 OS=Homo sapiens GN=HTR6 PE=1 SV=1 | 389 | DAGSGGsSGLR | 0.068 | -0.296 | -1.538 | -0.589 |
| sp\|P50406\|5HT6R_HUMAN 5-hydroxytryptamine receptor 6 OS=Homo sapiens GN=HTR6 PE=1 SV=1 | 390 | AGSGGSsGLRL | 0.036 | -0.384 | -1.632 | -0.66 |
| sp\|P50406\|5HT6R_HUMAN 5-hydroxytryptamine receptor 6 OS=Homo sapiens GN=HTR6 PE=1 SV=1 | 395 | SSGLRLtAQLL | 0.124 | 0.036 | -1.127 | -0.322 |
| sp\|P50406\|5HT6R_HUMAN 5-hydroxytryptamine receptor 6 OS=Homo sapiens GN=HTR6 PE=1 SV=1 | 405 | LLPGEAtQDPP | 0.124 | -0.152 | -1.179 | -0.402 |
| sp\|P50406\|5HT6R_HUMAN 5-hydroxytryptamine receptor 6 OS=Homo sapiens GN=HTR6 PE=1 SV=1 | 412 | QDPPLPtRAAA | 0.037 | -0.472 | -1.643 | -0.693 |
| sp\|P50406\|5HT6R_HUMAN 5-hydroxytryptamine receptor 6 OS=Homo sapiens GN=HTR6 PE=1 SV=1 | 439 | HPLGIPtN--- | 0.11 | 0.023 | -0.894 | -0.254 |
| sp\|P34969\|5HT7R_HUMAN 5-hydroxytryptamine receptor 7 OS=Homo sapiens GN=HTR7 PE=1 SV=2 | 393 | FNRDLRtTYRS | 0.157 | 0.042 | -1.261 | -0.354 |
| sp\|P34969\|5HT7R_HUMAN 5-hydroxytryptamine receptor 7 OS=Homo sapiens GN=HTR7 PE=1 SV=2 | 394 | NRDLRTtYRSL | 0.202 | 0.199 | -0.681 | -0.093 |
| sp\|P34969\|5HT7R_HUMAN 5-hydroxytryptamine receptor 7 OS=Homo sapiens GN=HTR7 PE=1 SV=2 | 397 | LRTTYRsLLQC | 0.217 | 0.135 | -0.748 | -0.132 |
| sp\|P34969\|5HT7R_HUMAN 5-hydroxytryptamine receptor 7 OS=Homo sapiens GN=HTR7 PE=1 SV=2 | 411 | NINRKLsAAGM | 0.352 | 0.734 | -0.18 | 0.302 |
| sp\|P34969\|5HT7R_HUMAN 5-hydroxytryptamine receptor 7 OS=Homo sapiens GN=HTR7 PE=1 SV=2 | 436 | FVLRACtRRVL | 0.763 | 0.948 | 0.433 | 0.715 |
| sp\|P34969\|5HT7R_HUMAN 5-hydroxytryptamine receptor 7 OS=Homo sapiens GN=HTR7 PE=1 SV=2 | 450 | EKRPPVsVWVL | 0.634 | 0.288 | 0.134 | 0.352 |
| sp\|P34969\|5HT7R_HUMAN 5-hydroxytryptamine receptor 7 OS=Homo sapiens GN=HTR7 PE=1 SV=2 | 456 | SVWVLQsPDHH | 0.015 | -0.471 | -2.046 | -0.834 |
| sp\|P34969\|5HT7R_HUMAN 5-hydroxytryptamine receptor 7 OS=Homo sapiens GN=HTR7 PE=1 SV=2 | 469 | LADKMLtTVEK | 0.15 | -0.219 | -0.996 | -0.355 |
| sp\|P34969\|5HT7R_HUMAN 5-hydroxytryptamine receptor 7 OS=Homo sapiens GN=HTR7 PE=1 SV=2 | 470 | ADKMLTtVEKK | 0.082 | -0.291 | -1.224 | -0.478 |
| sp\|P11229\|ACM1_HUMAN Muscarinic acetylcholine receptor M1 OS=Homo sapiens GN=CHRM1 PE=1 SV=2 | 428 | NKAFRDtFRLL | 0.644 | 0.22 | 0.08 | 0.315 |
| sp\|P11229\|ACM1_HUMAN Muscarinic acetylcholine receptor M1 OS=Homo sapiens GN=CHRM1 PE=1 SV=2 | 451 | IPKRPGsVHRT | 0.52 | 0.581 | 0.037 | 0.379 |
| sp\|P11229\|ACM1_HUMAN Muscarinic acetylcholine receptor M1 OS=Homo sapiens GN=CHRM1 PE=1 SV=2 | 455 | PGSVHRtPSRQ | 0.036 | -0.534 | -1.956 | -0.818 |
| sp\|P11229\|ACM1_HUMAN Muscarinic acetylcholine receptor M1 OS=Homo sapiens GN=CHRM1 PE=1 SV=2 | 457 | SVHRTPsRQC- | 0.216 | 0.718 | -0.543 | 0.13 |

| | | | | | | |
|---|---|---|---|---|---|---|
| sp\|P08172\|ACM2_HUMAN Muscarinic acetylcholine receptor M2 OS=Homo sapiens GN=CHRM2 PE=1 SV=1 | 446 | YALCNAtFKKT | 0.253 | -0.075 | -0.58 | -0.134 |
| sp\|P08172\|ACM2_HUMAN Muscarinic acetylcholine receptor M2 OS=Homo sapiens GN=CHRM2 PE=1 SV=1 | 450 | NATFKKtFKHL | 0.221 | 0.001 | -0.328 | -0.035 |
| sp\|P08172\|ACM2_HUMAN Muscarinic acetylcholine receptor M2 OS=Homo sapiens GN=CHRM2 PE=1 SV=1 | 465 | YKNIGAtR--- | 0.189 | 0.079 | -0.938 | -0.223 |
| sp\|P20309\|ACM3_HUMAN Muscarinic acetylcholine receptor M3 OS=Homo sapiens GN=CHRM3 PE=1 SV=1 | 550 | YALCNKtFRTT | 0.381 | -0.082 | -0.204 | 0.032 |
| sp\|P20309\|ACM3_HUMAN Muscarinic acetylcholine receptor M3 OS=Homo sapiens GN=CHRM3 PE=1 SV=1 | 553 | CNKTFRtTFKM | 0.14 | -0.156 | -0.995 | -0.337 |
| sp\|P20309\|ACM3_HUMAN Muscarinic acetylcholine receptor M3 OS=Homo sapiens GN=CHRM3 PE=1 SV=1 | 554 | NKTFRTtFKML | 0.592 | 0.272 | 0.165 | 0.343 |
| sp\|P20309\|ACM3_HUMAN Muscarinic acetylcholine receptor M3 OS=Homo sapiens GN=CHRM3 PE=1 SV=1 | 578 | QYQQRQsVIFH | 0.235 | 0.042 | -0.59 | -0.104 |
| sp\|P08173\|ACM4_HUMAN Muscarinic acetylcholine receptor M4 OS=Homo sapiens GN=CHRM4 PE=1 SV=2 | 459 | YALCNAtFKKT | 0.253 | -0.075 | -0.58 | -0.134 |
| sp\|P08173\|ACM4_HUMAN Muscarinic acetylcholine receptor M4 OS=Homo sapiens GN=CHRM4 PE=1 SV=2 | 463 | NATFKKtFRHL | 0.254 | -0.037 | -0.303 | -0.029 |
| sp\|P08173\|ACM4_HUMAN Muscarinic acetylcholine receptor M4 OS=Homo sapiens GN=CHRM4 PE=1 SV=2 | 477 | QYRNIGtAR-- | 0.285 | 0.263 | -0.463 | 0.028 |
| sp\|P08912\|ACM5_HUMAN Muscarinic acetylcholine receptor M5 OS=Homo sapiens GN=CHRM5 PE=2 SV=2 | 501 | YALCNRtFRKT | 0.178 | -0.164 | -0.858 | -0.281 |
| sp\|P08912\|ACM5_HUMAN Muscarinic acetylcholine receptor M5 OS=Homo sapiens GN=CHRM5 PE=2 SV=2 | 505 | NRTFRKtFKML | 0.627 | 0.48 | 0.325 | 0.477 |
| sp\|P08912\|ACM5_HUMAN Muscarinic acetylcholine receptor M5 OS=Homo sapiens GN=CHRM5 PE=2 SV=2 | 529 | LYWQGNsKLP- | 0.135 | 0.04 | -0.878 | -0.234 |
| sp\|P35348\|ADA1A_HUMAN Alpha-1A adrenergic receptor OS=Homo sapiens GN=ADRA1A PE=1 SV=2 | 330 | IIYPCSsQEFK | 0.094 | -0.253 | -1.243 | -0.467 |
| sp\|P35348\|ADA1A_HUMAN Alpha-1A adrenergic receptor OS=Homo sapiens GN=ADRA1A PE=1 SV=2 | 351 | CLCRKQsSKHA | 0.58 | 0.803 | 0.444 | 0.609 |
| sp\|P35348\|ADA1A_HUMAN Alpha-1A adrenergic receptor OS=Homo sapiens GN=ADRA1A PE=1 SV=2 | 352 | LCRKQSsKHAL | 0.508 | 0.357 | -0.142 | 0.241 |
| sp\|P35348\|ADA1A_HUMAN Alpha-1A adrenergic receptor OS=Homo sapiens GN=ADRA1A PE=1 SV=2 | 359 | KHALGYtLHPP | 0.121 | -0.076 | -0.99 | -0.315 |
| sp\|P35348\|ADA1A_HUMAN Alpha-1A adrenergic receptor OS=Homo sapiens GN=ADRA1A PE=1 SV=2 | 364 | YTLHPPsQAVE | 0.083 | -0.344 | -1.357 | -0.539 |
| sp\|P35348\|ADA1A_HUMAN Alpha-1A adrenergic receptor OS=Homo sapiens GN=ADRA1A PE=1 SV=2 | 381 | VRIPVGsRETF | 0.072 | -0.115 | -1.398 | -0.48 |
| sp\|P35348\|ADA1A_HUMAN Alpha-1A adrenergic receptor OS=Homo sapiens GN=ADRA1A PE=1 SV=2 | 384 | PVGSREtFYRI | 0.322 | 0.081 | -0.588 | -0.062 |
| sp\|P35348\|ADA1A_HUMAN Alpha-1A adrenergic receptor OS=Homo sapiens GN=ADRA1A PE=1 SV=2 | 389 | ETFYRIsKTDG | 0.578 | 0.177 | -0.094 | 0.22 |
| sp\|P35348\|ADA1A_HUMAN Alpha-1A adrenergic receptor OS=Homo sapiens GN=ADRA1A PE=1 SV=2 | 391 | FYRISKtDGVC | 0.821 | 0.682 | 0.445 | 0.649 |
| sp\|P35348\|ADA1A_HUMAN Alpha-1A adrenergic receptor OS=Homo sapiens GN=ADRA1A PE=1 SV=2 | 401 | CEWKFFsSMPR | 0.26 | 0.088 | -0.683 | -0.112 |
| sp\|P35348\|ADA1A_HUMAN Alpha-1A adrenergic receptor OS=Homo sapiens GN=ADRA1A PE=1 SV=2 | 402 | EWKFFSsMPRG | 0.509 | 0.495 | -0.294 | 0.237 |
| sp\|P35348\|ADA1A_HUMAN Alpha-1A adrenergic receptor OS=Homo sapiens GN=ADRA1A PE=1 SV=2 | 407 | SSMPRGsARIT | 0.125 | -0.176 | -1.213 | -0.421 |
| sp\|P35348\|ADA1A_HUMAN Alpha-1A adrenergic receptor OS=Homo sapiens GN=ADRA1A PE=1 SV=2 | 411 | RGSARItVSKD | 0.308 | 0.024 | -0.52 | -0.063 |
| sp\|P35348\|ADA1A_HUMAN Alpha-1A adrenergic receptor OS=Homo sapiens GN=ADRA1A PE=1 SV=2 | 413 | SARITVsKDQS | 0.308 | 0.142 | -0.52 | -0.023 |
| sp\|P35348\|ADA1A_HUMAN Alpha-1A adrenergic receptor OS=Homo sapiens GN=ADRA1A PE=1 SV=2 | 417 | TVSKDQsSCTT | 0.099 | -0.276 | -1.152 | -0.443 |
| sp\|P35348\|ADA1A_HUMAN Alpha-1A adrenergic receptor OS=Homo sapiens GN=ADRA1A PE=1 SV=2 | 418 | VSKDQSsCTTA | 0.131 | -0.16 | -1.165 | -0.398 |
| sp\|P35348\|ADA1A_HUMAN Alpha-1A adrenergic receptor OS=Homo sapiens GN=ADRA1A PE=1 SV=2 | 420 | KDQSSCtTARV | 0.217 | -0.078 | -1.259 | -0.373 |
| sp\|P35348\|ADA1A_HUMAN Alpha-1A adrenergic receptor OS=Homo sapiens GN=ADRA1A PE=1 SV=2 | 421 | DQSSCTtARVR | 0.28 | -0.108 | -0.754 | -0.194 |
| sp\|P35348\|ADA1A_HUMAN Alpha-1A adrenergic receptor OS=Homo sapiens GN=ADRA1A PE=1 SV=2 | 426 | TTARVRsKSFL | 0.323 | 0.663 | -0.29 | 0.232 |
| sp\|P35348\|ADA1A_HUMAN Alpha-1A adrenergic receptor OS=Homo sapiens GN=ADRA1A PE=1 SV=2 | 428 | ARVRSKsFLQV | 0.88 | 1.339 | 1.046 | 1.088 |
| sp\|P35348\|ADA1A_HUMAN Alpha-1A adrenergic receptor OS=Homo sapiens GN=ADRA1A PE=1 SV=2 | 439 | CCCVGPsTPSL | 0.307 | 0.374 | -0.497 | 0.061 |
| sp\|P35348\|ADA1A_HUMAN Alpha-1A adrenergic receptor OS=Homo sapiens GN=ADRA1A PE=1 SV=2 | 440 | CCVGPStPSLD | 0.055 | -0.424 | -1.401 | -0.59 |
| sp\|P35348\|ADA1A_HUMAN Alpha-1A adrenergic receptor OS=Homo sapiens GN=ADRA1A PE=1 SV=2 | 442 | VGPSTPsLDKN | 0.127 | -0.115 | -0.879 | -0.289 |
| sp\|P35348\|ADA1A_HUMAN Alpha-1A adrenergic receptor OS=Homo sapiens GN=ADRA1A PE=1 SV=2 | 451 | KNHQVPtIKVH | 0.047 | -0.418 | -1.521 | -0.631 |
| sp\|P35348\|ADA1A_HUMAN Alpha-1A adrenergic receptor OS=Homo sapiens GN=ADRA1A PE=1 SV=2 | 456 | PTIKVHtISLS | 0.266 | 0.054 | -0.819 | -0.166 |
| sp\|P35348\|ADA1A_HUMAN Alpha-1A adrenergic receptor OS=Homo sapiens GN=ADRA1A PE=1 SV=2 | 458 | IKVHTIsLSEN | 0.427 | 0.115 | -0.228 | 0.105 |
| sp\|P35348\|ADA1A_HUMAN Alpha-1A adrenergic receptor OS=Homo sapiens GN=ADRA1A PE=1 SV=2 | 460 | VHTISLsENGE | 0.247 | 0.137 | -0.523 | -0.046 |
| sp\|P35368\|ADA1B_HUMAN Alpha-1B adrenergic receptor OS=Homo sapiens GN=ADRA1B PE=1 SV=3 | 352 | IIYPCSsKEFK | 0.116 | -0.204 | -0.917 | -0.335 |
| sp\|P35368\|ADA1B_HUMAN Alpha-1B adrenergic receptor OS=Homo sapiens GN=ADRA1B PE=1 SV=3 | 387 | LGGCAYtYRPW | 0.148 | -0.089 | -0.726 | -0.222 |
| sp\|P35368\|ADA1B_HUMAN Alpha-1B adrenergic receptor OS=Homo sapiens GN=ADRA1B PE=1 SV=3 | 392 | YTYRPWtRGGS | 0.5 | 0.596 | 0.097 | 0.398 |
| sp\|P35368\|ADA1B_HUMAN Alpha-1B adrenergic receptor OS=Homo sapiens GN=ADRA1B PE=1 SV=3 | 396 | PWTRGGsLERS | 0.503 | 0.749 | 0.051 | 0.434 |
| sp\|P35368\|ADA1B_HUMAN Alpha-1B adrenergic receptor OS=Homo sapiens GN=ADRA1B PE=1 SV=3 | 400 | GGSLERsQSRK | 0.086 | -0.346 | -1.848 | -0.703 |
| sp\|P35368\|ADA1B_HUMAN Alpha-1B adrenergic receptor OS=Homo sapiens GN=ADRA1B PE=1 SV=3 | 402 | SLERSQsRKDS | 0.784 | 1.03 | 0.723 | 0.846 |
| sp\|P35368\|ADA1B_HUMAN Alpha-1B adrenergic receptor OS=Homo sapiens GN=ADRA1B PE=1 SV=3 | 406 | SQSRKDsLDDS | 0.592 | 0.773 | 0.261 | 0.542 |
| sp\|P35368\|ADA1B_HUMAN Alpha-1B adrenergic receptor OS=Homo sapiens GN=ADRA1B PE=1 SV=3 | 410 | KDSLDDsGSCL | 0.04 | -0.347 | -1.743 | -0.683 |
| sp\|P35368\|ADA1B_HUMAN Alpha-1B adrenergic receptor OS=Homo sapiens GN=ADRA1B PE=1 SV=3 | 412 | SLDDSGsCLSG | 0.139 | 0.124 | -1.121 | -0.286 |

| | | | | | | |
|---|---|---|---|---|---|---|
| sp\|P35368\|ADA1B_HUMAN Alpha-1B adrenergic receptor OS=Homo sapiens GN=ADRA1B PE=1 SV=3 | 415 | DSGSCLsGSQR | 0.083 | -0.343 | -1.328 | -0.529 |
| sp\|P35368\|ADA1B_HUMAN Alpha-1B adrenergic receptor OS=Homo sapiens GN=ADRA1B PE=1 SV=3 | 417 | GSCLSGsQRTL | 0.289 | -0.008 | -0.848 | -0.189 |
| sp\|P35368\|ADA1B_HUMAN Alpha-1B adrenergic receptor OS=Homo sapiens GN=ADRA1B PE=1 SV=3 | 420 | LSGSQRtLPSA | 0.228 | 0.306 | -0.516 | 0.006 |
| sp\|P35368\|ADA1B_HUMAN Alpha-1B adrenergic receptor OS=Homo sapiens GN=ADRA1B PE=1 SV=3 | 423 | SQRTLPsASPS | 0.08 | -0.106 | -1.45 | -0.492 |
| sp\|P35368\|ADA1B_HUMAN Alpha-1B adrenergic receptor OS=Homo sapiens GN=ADRA1B PE=1 SV=3 | 425 | RTLPSAsPSPG | 0.169 | -0.225 | -0.94 | -0.332 |
| sp\|P35368\|ADA1B_HUMAN Alpha-1B adrenergic receptor OS=Homo sapiens GN=ADRA1B PE=1 SV=3 | 427 | LPSASPsPGYL | 0.159 | -0.228 | -0.827 | -0.299 |
| sp\|P35368\|ADA1B_HUMAN Alpha-1B adrenergic receptor OS=Homo sapiens GN=ADRA1B PE=1 SV=3 | 455 | APGALLsLPAP | 0.341 | 0.34 | -0.315 | 0.122 |
| sp\|P35368\|ADA1B_HUMAN Alpha-1B adrenergic receptor OS=Homo sapiens GN=ADRA1B PE=1 SV=3 | 470 | RRGRHDsGPLF | 0.858 | 1.497 | 0.911 | 1.089 |
| sp\|P35368\|ADA1B_HUMAN Alpha-1B adrenergic receptor OS=Homo sapiens GN=ADRA1B PE=1 SV=3 | 475 | DSGPLFtFKLL | 0.13 | -0.081 | -0.913 | -0.288 |
| sp\|P35368\|ADA1B_HUMAN Alpha-1B adrenergic receptor OS=Homo sapiens GN=ADRA1B PE=1 SV=3 | 480 | FTFKLLtEPES | 0.647 | 0.576 | -0.062 | 0.387 |
| sp\|P35368\|ADA1B_HUMAN Alpha-1B adrenergic receptor OS=Homo sapiens GN=ADRA1B PE=1 SV=3 | 484 | LLTEPEsPGTD | 0.115 | -0.337 | -0.987 | -0.403 |
| sp\|P35368\|ADA1B_HUMAN Alpha-1B adrenergic receptor OS=Homo sapiens GN=ADRA1B PE=1 SV=3 | 487 | EPESPGtDGGA | 0.11 | -0.315 | -1.068 | -0.424 |
| sp\|P35368\|ADA1B_HUMAN Alpha-1B adrenergic receptor OS=Homo sapiens GN=ADRA1B PE=1 SV=3 | 492 | GTDGGAsNGGC | 0.154 | -0.198 | -1.268 | -0.437 |
| sp\|P35368\|ADA1B_HUMAN Alpha-1B adrenergic receptor OS=Homo sapiens GN=ADRA1B PE=1 SV=3 | 511 | GQPGFKsNMPL | 0.15 | -0.086 | -0.918 | -0.285 |
| sp\|P25100\|ADA1D_HUMAN Alpha-1D adrenergic receptor OS=Homo sapiens GN=ADRA1D PE=1 SV=2 | 406 | LIYPCSsREFK | 0.071 | -0.287 | -1.282 | -0.499 |
| sp\|P25100\|ADA1D_HUMAN Alpha-1D adrenergic receptor OS=Homo sapiens GN=ADRA1D PE=1 SV=2 | 441 | GHHWRAsTSGL | 0.283 | 0.034 | -0.872 | -0.185 |
| sp\|P25100\|ADA1D_HUMAN Alpha-1D adrenergic receptor OS=Homo sapiens GN=ADRA1D PE=1 SV=2 | 442 | HHWRAStSGLR | 0.567 | 1.048 | 0.218 | 0.611 |
| sp\|P25100\|ADA1D_HUMAN Alpha-1D adrenergic receptor OS=Homo sapiens GN=ADRA1D PE=1 SV=2 | 443 | HWRASTsGLRQ | 0.404 | 0.293 | -0.284 | 0.138 |
| sp\|P25100\|ADA1D_HUMAN Alpha-1D adrenergic receptor OS=Homo sapiens GN=ADRA1D PE=1 SV=2 | 452 | RQDCAPsSGDA | 0.399 | 0.009 | -0.424 | -0.005 |
| sp\|P25100\|ADA1D_HUMAN Alpha-1D adrenergic receptor OS=Homo sapiens GN=ADRA1D PE=1 SV=2 | 453 | QDCAPSsGDAP | 0.058 | -0.334 | -1.183 | -0.486 |
| sp\|P25100\|ADA1D_HUMAN Alpha-1D adrenergic receptor OS=Homo sapiens GN=ADRA1D PE=1 SV=2 | 465 | GAPLALtALPD | 0.13 | -0.095 | -1.257 | -0.407 |
| sp\|P25100\|ADA1D_HUMAN Alpha-1D adrenergic receptor OS=Homo sapiens GN=ADRA1D PE=1 SV=2 | 477 | DPEPPGtPEMQ | 0.028 | -0.739 | -2.056 | -0.922 |
| sp\|P25100\|ADA1D_HUMAN Alpha-1D adrenergic receptor OS=Homo sapiens GN=ADRA1D PE=1 SV=2 | 486 | MQAPVAsRRKP | 0.122 | -0.233 | -1.012 | -0.374 |
| sp\|P25100\|ADA1D_HUMAN Alpha-1D adrenergic receptor OS=Homo sapiens GN=ADRA1D PE=1 SV=2 | 492 | SRRKPPsAFRE | 0.435 | 0.228 | -0.552 | 0.037 |
| sp\|P25100\|ADA1D_HUMAN Alpha-1D adrenergic receptor OS=Homo sapiens GN=ADRA1D PE=1 SV=2 | 507 | GPFRRPtTQLR | 0.793 | 0.975 | 0.536 | 0.768 |
| sp\|P25100\|ADA1D_HUMAN Alpha-1D adrenergic receptor OS=Homo sapiens GN=ADRA1D PE=1 SV=2 | 508 | PFRRPTtQLRA | 0.707 | 0.879 | 0.386 | 0.657 |
| sp\|P25100\|ADA1D_HUMAN Alpha-1D adrenergic receptor OS=Homo sapiens GN=ADRA1D PE=1 SV=2 | 515 | QLRAKVsSLSH | 0.18 | -0.022 | -0.995 | -0.279 |
| sp\|P25100\|ADA1D_HUMAN Alpha-1D adrenergic receptor OS=Homo sapiens GN=ADRA1D PE=1 SV=2 | 516 | LRAKVSsLSHK | 0.25 | 0.087 | -0.368 | -0.01 |
| sp\|P25100\|ADA1D_HUMAN Alpha-1D adrenergic receptor OS=Homo sapiens GN=ADRA1D PE=1 SV=2 | 518 | AKVSSLsHKIR | 0.392 | 0.114 | -0.326 | 0.06 |
| sp\|P25100\|ADA1D_HUMAN Alpha-1D adrenergic receptor OS=Homo sapiens GN=ADRA1D PE=1 SV=2 | 537 | AACAQRsEVEA | 0.141 | -0.103 | -1.27 | -0.411 |
| sp\|P25100\|ADA1D_HUMAN Alpha-1D adrenergic receptor OS=Homo sapiens GN=ADRA1D PE=1 SV=2 | 543 | SEVEAVsLGVP | 0.3 | 0.05 | -0.684 | -0.111 |
| sp\|P25100\|ADA1D_HUMAN Alpha-1D adrenergic receptor OS=Homo sapiens GN=ADRA1D PE=1 SV=2 | 555 | EVAEGAtCQAY | 0.149 | 0.098 | -0.749 | -0.167 |
| sp\|P25100\|ADA1D_HUMAN Alpha-1D adrenergic receptor OS=Homo sapiens GN=ADRA1D PE=1 SV=2 | 565 | YELADYsNLRE | 0.095 | -0.234 | -1.373 | -0.504 |
| sp\|P25100\|ADA1D_HUMAN Alpha-1D adrenergic receptor OS=Homo sapiens GN=ADRA1D PE=1 SV=2 | 570 | YSNLREtDI-- | 0.213 | 0.16 | -0.928 | -0.185 |
| sp\|P18089\|ADA2B_HUMAN Alpha-2B adrenergic receptor OS=Homo sapiens GN=ADRA2B PE=1 SV=4 | 427 | LNPVIYtIFNQ | 0.051 | -0.179 | -1.121 | -0.416 |
| sp\|P18089\|ADA2B_HUMAN Alpha-2B adrenergic receptor OS=Homo sapiens GN=ADRA2B PE=1 SV=4 | 446 | ILCRPWtQTAW | 0.792 | 0.841 | 0.507 | 0.713 |
| sp\|P18089\|ADA2B_HUMAN Alpha-2B adrenergic receptor OS=Homo sapiens GN=ADRA2B PE=1 SV=4 | 448 | CRPWTQtAW-- | 0.31 | 0.405 | -0.501 | 0.071 |
| sp\|P18825\|ADA2C_HUMAN Alpha-2C adrenergic receptor OS=Homo sapiens GN=ADRA2C PE=2 SV=2 | 447 | NQDFRRsFKHI | 0.301 | 0.134 | -0.291 | 0.048 |
| sp\|P08588\|ADRB1_HUMAN Beta-1 adrenergic receptor OS=Homo sapiens GN=ADRB1 PE=1 SV=2 | 380 | PIIYCRsPDFR | 0.146 | -0.178 | -0.974 | -0.335 |
| sp\|P08588\|ADRB1_HUMAN Beta-1 adrenergic receptor OS=Homo sapiens GN=ADRB1 PE=1 SV=2 | 404 | ARRRHAtHGDR | 0.955 | 1.503 | 1.475 | 1.311 |
| sp\|P08588\|ADRB1_HUMAN Beta-1 adrenergic receptor OS=Homo sapiens GN=ADRB1 PE=1 SV=2 | 412 | GDRPRAsGCLA | 0.388 | 0.141 | -0.439 | 0.03 |
| sp\|P08588\|ADRB1_HUMAN Beta-1 adrenergic receptor OS=Homo sapiens GN=ADRB1 PE=1 SV=2 | 423 | RPGPPPsPGAA | 0.07 | -0.455 | -1.166 | -0.517 |
| sp\|P08588\|ADRB1_HUMAN Beta-1 adrenergic receptor OS=Homo sapiens GN=ADRB1 PE=1 SV=2 | 428 | PSPGAAsDDDD | 0.53 | 0.138 | -0.223 | 0.148 |
| sp\|P08588\|ADRB1_HUMAN Beta-1 adrenergic receptor OS=Homo sapiens GN=ADRB1 PE=1 SV=2 | 439 | DDVVGAtPPAR | 0.193 | 0.083 | -0.774 | -0.166 |
| sp\|P08588\|ADRB1_HUMAN Beta-1 adrenergic receptor OS=Homo sapiens GN=ADRB1 PE=1 SV=2 | 459 | GGAAADsDSSL | 0.188 | -0.088 | -0.963 | -0.288 |
| sp\|P08588\|ADRB1_HUMAN Beta-1 adrenergic receptor OS=Homo sapiens GN=ADRB1 PE=1 SV=2 | 461 | AAADSDsSLDE | 0.25 | -0.022 | -0.897 | -0.223 |
| sp\|P08588\|ADRB1_HUMAN Beta-1 adrenergic receptor OS=Homo sapiens GN=ADRB1 PE=1 SV=2 | 462 | AADSDSsLDEP | 0.135 | -0.23 | -1.082 | -0.392 |
| sp\|P08588\|ADRB1_HUMAN Beta-1 adrenergic receptor OS=Homo sapiens GN=ADRB1 PE=1 SV=2 | 473 | CRPGFAsESKV | 0.167 | 0.163 | -1.064 | -0.245 |
| sp\|P08588\|ADRB1_HUMAN Beta-1 adrenergic receptor OS=Homo sapiens GN=ADRB1 PE=1 SV=2 | 475 | PGFASEsKV-- | 0.332 | 0.247 | -0.401 | 0.059 |

| | | | | | | |
|---|---|---|---|---|---|---|
| sp\|P07550\|ADRB2_HUMAN Beta-2 adrenergic receptor OS=Homo sapiens GN=ADRB2 PE=1 SV=3 | 329 | PLIYCRsPDFR | 0.201 | -0.157 | -0.83 | -0.262 |
| sp\|P07550\|ADRB2_HUMAN Beta-2 adrenergic receptor OS=Homo sapiens GN=ADRB2 PE=1 SV=3 | 345 | LLCLRRsSLKA | 0.215 | 0.078 | -0.948 | -0.218 |
| sp\|P07550\|ADRB2_HUMAN Beta-2 adrenergic receptor OS=Homo sapiens GN=ADRB2 PE=1 SV=3 | 346 | LCLRRSsLKAY | 0.877 | 1.153 | 1.087 | 1.039 |
| sp\|P07550\|ADRB2_HUMAN Beta-2 adrenergic receptor OS=Homo sapiens GN=ADRB2 PE=1 SV=3 | 355 | AYGNGYsSNGN | 0.101 | -0.081 | -0.827 | -0.269 |
| sp\|P07550\|ADRB2_HUMAN Beta-2 adrenergic receptor OS=Homo sapiens GN=ADRB2 PE=1 SV=3 | 356 | YGNGYSsNGNT | 0.105 | -0.126 | -1.175 | -0.399 |
| sp\|P07550\|ADRB2_HUMAN Beta-2 adrenergic receptor OS=Homo sapiens GN=ADRB2 PE=1 SV=3 | 360 | YSSNGNtGEQS | 0.069 | -0.261 | -1.148 | -0.447 |
| sp\|P07550\|ADRB2_HUMAN Beta-2 adrenergic receptor OS=Homo sapiens GN=ADRB2 PE=1 SV=3 | 364 | GNTGEQsGYHV | 0.101 | -0.207 | -1.155 | -0.42 |
| sp\|P07550\|ADRB2_HUMAN Beta-2 adrenergic receptor OS=Homo sapiens GN=ADRB2 PE=1 SV=3 | 384 | CEDLPGtEDFV | 0.103 | -0.179 | -1.355 | -0.477 |
| sp\|P07550\|ADRB2_HUMAN Beta-2 adrenergic receptor OS=Homo sapiens GN=ADRB2 PE=1 SV=3 | 393 | FVGHQGtVPSD | 0.211 | 0.401 | -0.566 | 0.015 |
| sp\|P07550\|ADRB2_HUMAN Beta-2 adrenergic receptor OS=Homo sapiens GN=ADRB2 PE=1 SV=3 | 396 | HQGTVPsDNID | 0.235 | -0.02 | -0.642 | -0.142 |
| sp\|P07550\|ADRB2_HUMAN Beta-2 adrenergic receptor OS=Homo sapiens GN=ADRB2 PE=1 SV=3 | 401 | PSDNIDsQGRN | 0.154 | -0.258 | -1.2 | -0.435 |
| sp\|P07550\|ADRB2_HUMAN Beta-2 adrenergic receptor OS=Homo sapiens GN=ADRB2 PE=1 SV=3 | 407 | SQGRNCsTNDS | 0.585 | 0.806 | 0.185 | 0.525 |
| sp\|P07550\|ADRB2_HUMAN Beta-2 adrenergic receptor OS=Homo sapiens GN=ADRB2 PE=1 SV=3 | 408 | QGRNCStNDSL | 0.201 | 0.119 | -0.555 | -0.078 |
| sp\|P07550\|ADRB2_HUMAN Beta-2 adrenergic receptor OS=Homo sapiens GN=ADRB2 PE=1 SV=3 | 411 | NCSTNDsLL-- | 0.305 | 0.078 | -0.792 | -0.136 |
| sp\|P13945\|ADRB3_HUMAN Beta-3 adrenergic receptor OS=Homo sapiens GN=ADRB3 PE=1 SV=3 | 349 | PLIYCRsPDFR | 0.201 | -0.157 | -0.83 | -0.262 |
| sp\|P13945\|ADRB3_HUMAN Beta-3 adrenergic receptor OS=Homo sapiens GN=ADRB3 PE=1 SV=3 | 354 | RSPDFRsAFRR | 0.141 | -0.16 | -1.505 | -0.508 |
| sp\|P13945\|ADRB3_HUMAN Beta-3 adrenergic receptor OS=Homo sapiens GN=ADRB3 PE=1 SV=3 | 382 | RPALFPsGVPA | 0.099 | -0.257 | -1.193 | -0.45 |
| sp\|P13945\|ADRB3_HUMAN Beta-3 adrenergic receptor OS=Homo sapiens GN=ADRB3 PE=1 SV=3 | 389 | GVPAARsSPAQ | 0.47 | 0.459 | -0.285 | 0.215 |
| sp\|P13945\|ADRB3_HUMAN Beta-3 adrenergic receptor OS=Homo sapiens GN=ADRB3 PE=1 SV=3 | 390 | VPAARSsPAQP | 0.054 | -0.503 | -1.445 | -0.631 |
| sp\|P13945\|ADRB3_HUMAN Beta-3 adrenergic receptor OS=Homo sapiens GN=ADRB3 PE=1 SV=3 | 404 | QRLDGAsWGVS | 0.211 | 0.279 | -0.724 | -0.078 |
| sp\|P13945\|ADRB3_HUMAN Beta-3 adrenergic receptor OS=Homo sapiens GN=ADRB3 PE=1 SV=3 | 408 | GASWGVs---- | 0.272 | -0.019 | -0.743 | -0.163 |
| sp\|P21728\|DRD1_HUMAN D(1A) dopamine receptor OS=Homo sapiens GN=DRD1 PE=1 SV=1 | 342 | DFRKAFsTLLG | 0.697 | 0.381 | 0.05 | 0.376 |
| sp\|P21728\|DRD1_HUMAN D(1A) dopamine receptor OS=Homo sapiens GN=DRD1 PE=1 SV=1 | 343 | FRKAFStLLGC | 0.281 | 0.133 | -0.656 | -0.081 |
| sp\|P21728\|DRD1_HUMAN D(1A) dopamine receptor OS=Homo sapiens GN=DRD1 PE=1 SV=1 | 354 | YRLCPAtNNAI | 0.481 | 0.259 | -0.057 | 0.228 |
| sp\|P21728\|DRD1_HUMAN D(1A) dopamine receptor OS=Homo sapiens GN=DRD1 PE=1 SV=1 | 360 | TNNAIEtVSIN | 0.082 | -0.284 | -1.335 | -0.512 |
| sp\|P21728\|DRD1_HUMAN D(1A) dopamine receptor OS=Homo sapiens GN=DRD1 PE=1 SV=1 | 362 | NAIETVsINNN | 0.142 | 0.005 | -1.008 | -0.287 |
| sp\|P21728\|DRD1_HUMAN D(1A) dopamine receptor OS=Homo sapiens GN=DRD1 PE=1 SV=1 | 372 | NGAAMFsSHHE | 0.152 | -0.217 | -0.73 | -0.265 |
| sp\|P21728\|DRD1_HUMAN D(1A) dopamine receptor OS=Homo sapiens GN=DRD1 PE=1 SV=1 | 373 | GAAMFSsHHEP | 0.183 | -0.191 | -1.054 | -0.354 |
| sp\|P21728\|DRD1_HUMAN D(1A) dopamine receptor OS=Homo sapiens GN=DRD1 PE=1 SV=1 | 380 | HHEPRGsISKE | 0.072 | -0.161 | -1.399 | -0.496 |
| sp\|P21728\|DRD1_HUMAN D(1A) dopamine receptor OS=Homo sapiens GN=DRD1 PE=1 SV=1 | 382 | EPRGSIsKECN | 0.467 | 0.423 | -0.055 | 0.278 |
| sp\|P21728\|DRD1_HUMAN D(1A) dopamine receptor OS=Homo sapiens GN=DRD1 PE=1 SV=1 | 397 | IPHAVGsSEDL | 0.121 | -0.191 | -1.212 | -0.427 |
| sp\|P21728\|DRD1_HUMAN D(1A) dopamine receptor OS=Homo sapiens GN=DRD1 PE=1 SV=1 | 398 | PHAVGSsEDLK | 0.079 | -0.106 | -1.226 | -0.418 |
| sp\|P21728\|DRD1_HUMAN D(1A) dopamine receptor OS=Homo sapiens GN=DRD1 PE=1 SV=1 | 417 | RPLEKLsPALS | 0.02 | -0.521 | -2.026 | -0.842 |
| sp\|P21728\|DRD1_HUMAN D(1A) dopamine receptor OS=Homo sapiens GN=DRD1 PE=1 SV=1 | 421 | KLSPALsVILD | 0.226 | 0.031 | -0.484 | -0.076 |
| sp\|P21728\|DRD1_HUMAN D(1A) dopamine receptor OS=Homo sapiens GN=DRD1 PE=1 SV=1 | 428 | VILDYDtDVSL | 0.16 | -0.027 | -1.143 | -0.337 |
| sp\|P21728\|DRD1_HUMAN D(1A) dopamine receptor OS=Homo sapiens GN=DRD1 PE=1 SV=1 | 431 | DYDTDVsLEKI | 0.515 | 0.032 | -0.375 | 0.057 |
| sp\|P21728\|DRD1_HUMAN D(1A) dopamine receptor OS=Homo sapiens GN=DRD1 PE=1 SV=1 | 439 | EKIQPItQNGQ | 0.251 | -0.146 | -0.59 | -0.162 |
| sp\|P21728\|DRD1_HUMAN D(1A) dopamine receptor OS=Homo sapiens GN=DRD1 PE=1 SV=1 | 446 | QNGQHPt---- | 0.136 | -0.032 | -0.893 | -0.263 |
| sp\|P35462\|DRD3_HUMAN D(3) dopamine receptor OS=Homo sapiens GN=DRD3 PE=1 SV=2 | 399 | AFLKILsC--- | 0.29 | 0.195 | -0.444 | 0.014 |
| sp\|P21918\|DRD5_HUMAN D(1B) dopamine receptor OS=Homo sapiens GN=DRD5 PE=1 SV=2 | 376 | AQLLGCsHFCS | 0.152 | -0.102 | -1.105 | -0.352 |
| sp\|P21918\|DRD5_HUMAN D(1B) dopamine receptor OS=Homo sapiens GN=DRD5 PE=1 SV=2 | 380 | GCSHFCsRTPV | 0.312 | -0.159 | -1.367 | -0.405 |
| sp\|P21918\|DRD5_HUMAN D(1B) dopamine receptor OS=Homo sapiens GN=DRD5 PE=1 SV=2 | 382 | SHFCSRtPVET | 0.094 | -0.216 | -1.595 | -0.572 |
| sp\|P21918\|DRD5_HUMAN D(1B) dopamine receptor OS=Homo sapiens GN=DRD5 PE=1 SV=2 | 386 | SRTPVEtVNIS | 0.163 | 0.042 | -0.968 | -0.254 |
| sp\|P21918\|DRD5_HUMAN D(1B) dopamine receptor OS=Homo sapiens GN=DRD5 PE=1 SV=2 | 390 | VETVNIsNELI | 0.166 | -0.099 | -0.905 | -0.279 |
| sp\|P21918\|DRD5_HUMAN D(1B) dopamine receptor OS=Homo sapiens GN=DRD5 PE=1 SV=2 | 395 | ISNELIsYNQD | 0.135 | -0.211 | -0.931 | -0.336 |
| sp\|P21918\|DRD5_HUMAN D(1B) dopamine receptor OS=Homo sapiens GN=DRD5 PE=1 SV=2 | 419 | MMPNAVtPGNR | 0.229 | -0.02 | -0.71 | -0.167 |
| sp\|P21918\|DRD5_HUMAN D(1B) dopamine receptor OS=Homo sapiens GN=DRD5 PE=1 SV=2 | 443 | MFQIYQtSPDG | 0.789 | 0.703 | 0.532 | 0.675 |
| sp\|P21918\|DRD5_HUMAN D(1B) dopamine receptor OS=Homo sapiens GN=DRD5 PE=1 SV=2 | 444 | FQIYQTsPDGD | 0.112 | -0.194 | -0.796 | -0.293 |
| sp\|P21918\|DRD5_HUMAN D(1B) dopamine receptor OS=Homo sapiens GN=DRD5 PE=1 SV=2 | 453 | GDPVAEsVWEL | 0.185 | -0.02 | -1.053 | -0.296 |

| | | | | | | |
|---|---|---|---|---|---|---|
| sp\|P21918\|DRD5_HUMAN D(1B) dopamine receptor OS=Homo sapiens GN=DRD5 PE=1 SV=2 | 464 | DCEGEIsLDKI | 0.569 | 0.107 | -0.23 | 0.149 |
| sp\|P21918\|DRD5_HUMAN D(1B) dopamine receptor OS=Homo sapiens GN=DRD5 PE=1 SV=2 | 469 | ISLDKItPFTP | 0.064 | -0.525 | -1.378 | -0.613 |
| sp\|P21918\|DRD5_HUMAN D(1B) dopamine receptor OS=Homo sapiens GN=DRD5 PE=1 SV=2 | 472 | DKITPFtPNGF | 0.149 | -0.337 | -0.887 | -0.358 |
| sp\|P35367\|HRH1_HUMAN Histamine H1 receptor OS=Homo sapiens GN=HRH1 PE=1 SV=1 | 478 | NENFKKtFKRI | 0.325 | 0.001 | -0.526 | -0.067 |
| sp\|P35367\|HRH1_HUMAN Histamine H1 receptor OS=Homo sapiens GN=HRH1 PE=1 SV=1 | 487 | RILHIRs---- | 0.2 | 0.181 | -0.713 | -0.111 |
| sp\|P25021\|HRH2_HUMAN Histamine H2 receptor OS=Homo sapiens GN=HRH2 PE=2 SV=1 | 297 | LNRDFRtGYQQ | 0.163 | -0.027 | -1.026 | -0.297 |
| sp\|P25021\|HRH2_HUMAN Histamine H2 receptor OS=Homo sapiens GN=HRH2 PE=2 SV=1 | 312 | RLANRNsHKTS | 0.524 | 0.237 | -0.088 | 0.224 |
| sp\|P25021\|HRH2_HUMAN Histamine H2 receptor OS=Homo sapiens GN=HRH2 PE=2 SV=1 | 315 | NRNSHKtSLRS | 0.184 | 0.061 | -1.061 | -0.272 |
| sp\|P25021\|HRH2_HUMAN Histamine H2 receptor OS=Homo sapiens GN=HRH2 PE=2 SV=1 | 316 | RNSHKTsLRSN | 0.136 | -0.177 | -0.814 | -0.285 |
| sp\|P25021\|HRH2_HUMAN Histamine H2 receptor OS=Homo sapiens GN=HRH2 PE=2 SV=1 | 319 | HKTSLRsNASQ | 0.061 | -0.207 | -1.55 | -0.565 |
| sp\|P25021\|HRH2_HUMAN Histamine H2 receptor OS=Homo sapiens GN=HRH2 PE=2 SV=1 | 322 | SLRSNAsQLSR | 0.244 | 0.104 | -0.906 | -0.186 |
| sp\|P25021\|HRH2_HUMAN Histamine H2 receptor OS=Homo sapiens GN=HRH2 PE=2 SV=1 | 325 | SNASQLsRTQS | 0.038 | -0.455 | -1.794 | -0.737 |
| sp\|P25021\|HRH2_HUMAN Histamine H2 receptor OS=Homo sapiens GN=HRH2 PE=2 SV=1 | 327 | ASQLSRtQSRE | 0.155 | -0.107 | -1.418 | -0.457 |
| sp\|P25021\|HRH2_HUMAN Histamine H2 receptor OS=Homo sapiens GN=HRH2 PE=2 SV=1 | 329 | QLSRTQsREPR | 0.473 | 0.792 | 0.179 | 0.481 |
| sp\|P25021\|HRH2_HUMAN Histamine H2 receptor OS=Homo sapiens GN=HRH2 PE=2 SV=1 | 346 | LKLQVWsGTEV | 0.446 | -0.05 | -0.508 | -0.037 |
| sp\|P25021\|HRH2_HUMAN Histamine H2 receptor OS=Homo sapiens GN=HRH2 PE=2 SV=1 | 348 | LQVWSGtEVTA | 0.233 | 0.077 | -0.344 | -0.011 |
| sp\|P25021\|HRH2_HUMAN Histamine H2 receptor OS=Homo sapiens GN=HRH2 PE=2 SV=1 | 351 | WSGTEVtAPQG | 0.588 | 0.313 | -0.187 | 0.238 |
| sp\|P25021\|HRH2_HUMAN Histamine H2 receptor OS=Homo sapiens GN=HRH2 PE=2 SV=1 | 357 | TAPQGAtDR-- | 0.134 | -0.098 | -1.148 | -0.371 |
| sp\|Q9Y5N1\|HRH3_HUMAN Histamine H3 receptor OS=Homo sapiens GN=HRH3 PE=1 SV=2 | 418 | YPLCHHsFRRA | 0.487 | 0.025 | -0.424 | 0.029 |
| sp\|Q9Y5N1\|HRH3_HUMAN Histamine H3 receptor OS=Homo sapiens GN=HRH3 PE=1 SV=2 | 424 | SFRRAFtKLLC | 0.75 | 1.086 | 0.519 | 0.785 |
| sp\|Q9Y5N1\|HRH3_HUMAN Histamine H3 receptor OS=Homo sapiens GN=HRH3 PE=1 SV=2 | 438 | LKIQPHsSLEH | 0.208 | -0.057 | -0.819 | -0.223 |
| sp\|Q9Y5N1\|HRH3_HUMAN Histamine H3 receptor OS=Homo sapiens GN=HRH3 PE=1 SV=2 | 439 | KIQPHSsLEHC | 0.153 | -0.055 | -0.897 | -0.266 |
| sp\|Q9H3N8\|HRH4_HUMAN Histamine H4 receptor OS=Homo sapiens GN=HRH4 PE=1 SV=2 | 382 | KKQPLPsQHSR | 0.129 | -0.173 | -1.245 | -0.43 |
| sp\|Q9H3N8\|HRH4_HUMAN Histamine H4 receptor OS=Homo sapiens GN=HRH4 PE=1 SV=2 | 385 | PLPSQHsRSVS | 0.106 | -0.065 | -1.345 | -0.435 |
| sp\|Q9H3N8\|HRH4_HUMAN Histamine H4 receptor OS=Homo sapiens GN=HRH4 PE=1 SV=2 | 387 | PSQHSRsVSS- | 0.141 | 0.097 | -0.893 | -0.218 |
| sp\|Q9H3N8\|HRH4_HUMAN Histamine H4 receptor OS=Homo sapiens GN=HRH4 PE=1 SV=2 | 389 | QHSRSVsS--- | 0.779 | 1.122 | 0.471 | 0.791 |
| sp\|Q9H3N8\|HRH4_HUMAN Histamine H4 receptor OS=Homo sapiens GN=HRH4 PE=1 SV=2 | 390 | HSRSVSs---- | 0.227 | 0.213 | -0.713 | -0.091 |
| sp\|Q96RJ0\|TAAR1_HUMAN Trace amine-associated receptor 1 OS=Homo sapiens GN=TAAR1 PE=2 SV=1 | 328 | KIFQKDsSRCK | 0.133 | -0.206 | -1.176 | -0.416 |
| sp\|Q96RJ0\|TAAR1_HUMAN Trace amine-associated receptor 1 OS=Homo sapiens GN=TAAR1 PE=2 SV=1 | 329 | IFQKDSsRCKL | 0.168 | -0.177 | -0.93 | -0.313 |
| sp\|Q96RJ0\|TAAR1_HUMAN Trace amine-associated receptor 1 OS=Homo sapiens GN=TAAR1 PE=2 SV=1 | 338 | KLFLELsS--- | 0.148 | 0.067 | -1.179 | -0.321 |
| sp\|Q96RJ0\|TAAR1_HUMAN Trace amine-associated receptor 1 OS=Homo sapiens GN=TAAR1 PE=2 SV=1 | 339 | LFLELSs---- | 0.066 | -0.024 | -1.173 | -0.377 |
| sp\|P30556\|AGTR1_HUMAN Type-1 angiotensin II receptor OS=Homo sapiens GN=AGTR1 PE=1 SV=1 | 326 | IPPKAKsHSNL | 0.423 | 0.085 | -0.408 | 0.033 |
| sp\|P30556\|AGTR1_HUMAN Type-1 angiotensin II receptor OS=Homo sapiens GN=AGTR1 PE=1 SV=1 | 328 | PKAKSHsNLST | 0.517 | 0.307 | -0.088 | 0.245 |
| sp\|P30556\|AGTR1_HUMAN Type-1 angiotensin II receptor OS=Homo sapiens GN=AGTR1 PE=1 SV=1 | 331 | KSHSNLsTKMS | 0.039 | -0.41 | -1.679 | -0.683 |
| sp\|P30556\|AGTR1_HUMAN Type-1 angiotensin II receptor OS=Homo sapiens GN=AGTR1 PE=1 SV=1 | 332 | SHSNLStKMST | 0.024 | -0.368 | -1.97 | -0.771 |
| sp\|P30556\|AGTR1_HUMAN Type-1 angiotensin II receptor OS=Homo sapiens GN=AGTR1 PE=1 SV=1 | 335 | NLSTKMsTLSY | 0.165 | 0.036 | -0.991 | -0.263 |
| sp\|P30556\|AGTR1_HUMAN Type-1 angiotensin II receptor OS=Homo sapiens GN=AGTR1 PE=1 SV=1 | 336 | LSTKMStLSYR | 0.226 | -0.16 | -0.563 | -0.166 |
| sp\|P30556\|AGTR1_HUMAN Type-1 angiotensin II receptor OS=Homo sapiens GN=AGTR1 PE=1 SV=1 | 338 | TKMSTLsYRPS | 0.058 | -0.216 | -1.236 | -0.465 |
| sp\|P30556\|AGTR1_HUMAN Type-1 angiotensin II receptor OS=Homo sapiens GN=AGTR1 PE=1 SV=1 | 342 | TLSYRPsDNVS | 0.453 | 0.194 | -0.273 | 0.125 |
| sp\|P30556\|AGTR1_HUMAN Type-1 angiotensin II receptor OS=Homo sapiens GN=AGTR1 PE=1 SV=1 | 346 | RPSDNVsSSTK | 0.189 | -0.23 | -1.07 | -0.37 |
| sp\|P30556\|AGTR1_HUMAN Type-1 angiotensin II receptor OS=Homo sapiens GN=AGTR1 PE=1 SV=1 | 347 | PSDNVSsSTKK | 0.07 | -0.325 | -1.384 | -0.546 |
| sp\|P30556\|AGTR1_HUMAN Type-1 angiotensin II receptor OS=Homo sapiens GN=AGTR1 PE=1 SV=1 | 348 | SDNVSSsTKKP | 0.032 | -0.218 | -1.677 | -0.621 |
| sp\|P30556\|AGTR1_HUMAN Type-1 angiotensin II receptor OS=Homo sapiens GN=AGTR1 PE=1 SV=1 | 349 | DNVSSStKKPA | 0.137 | -0.156 | -0.834 | -0.284 |
| sp\|P50052\|AGTR2_HUMAN Type-2 angiotensin II receptor OS=Homo sapiens GN=AGTR2 PE=1 SV=1 | 331 | FQQKLRsVFRV | 0.234 | 0.033 | -1.021 | -0.251 |
| sp\|P50052\|AGTR2_HUMAN Type-2 angiotensin II receptor OS=Homo sapiens GN=AGTR2 PE=1 SV=1 | 338 | VFRVPItWLQG | 0.231 | 0.124 | -0.483 | -0.043 |
| sp\|P50052\|AGTR2_HUMAN Type-2 angiotensin II receptor OS=Homo sapiens GN=AGTR2 PE=1 SV=1 | 346 | LQGKREsMSCR | 0.21 | 0.175 | -0.743 | -0.119 |
| sp\|P50052\|AGTR2_HUMAN Type-2 angiotensin II receptor OS=Homo sapiens GN=AGTR2 PE=1 SV=1 | 348 | GKRESMsCRKS | 0.745 | 0.634 | 0.266 | 0.548 |
| sp\|P50052\|AGTR2_HUMAN Type-2 angiotensin II receptor OS=Homo sapiens GN=AGTR2 PE=1 SV=1 | 352 | SMSCRKsSSLR | 0.2 | 0.109 | -0.824 | -0.172 |
| sp\|P50052\|AGTR2_HUMAN Type-2 angiotensin II receptor OS=Homo sapiens GN=AGTR2 PE=1 SV=1 | 353 | MSCRKSsSLRE | 0.303 | 0.568 | -0.564 | 0.102 |

| | | | | | | |
|---|---|---|---|---|---|---|
| sp\|P50052\|AGTR2_HUMAN Type-2 angiotensin II receptor OS=Homo sapiens GN=AGTR2 PE=1 SV=1 | 354 | SCRKSSsLREM | 0.803 | 0.458 | 0.373 | 0.545 |
| sp\|P50052\|AGTR2_HUMAN Type-2 angiotensin II receptor OS=Homo sapiens GN=AGTR2 PE=1 SV=1 | 360 | SLREMEtFVS- | 0.217 | 0.177 | -0.837 | -0.148 |
| sp\|P50052\|AGTR2_HUMAN Type-2 angiotensin II receptor OS=Homo sapiens GN=AGTR2 PE=1 SV=1 | 363 | EMETFVs---- | 0.209 | 0.142 | -0.856 | -0.168 |
| sp\|P35414\|APJ_HUMAN Apelin receptor OS=Homo sapiens GN=APLNR PE=1 SV=1 | 321 | RFRQACtSMLC | 0.633 | 0.289 | -0.259 | 0.221 |
| sp\|P35414\|APJ_HUMAN Apelin receptor OS=Homo sapiens GN=APLNR PE=1 SV=1 | 322 | FRQACTsMLCC | 0.298 | 0.373 | -0.756 | -0.028 |
| sp\|P35414\|APJ_HUMAN Apelin receptor OS=Homo sapiens GN=APLNR PE=1 SV=1 | 329 | MLCCGQsRCAG | 0.309 | -0.008 | -0.701 | -0.133 |
| sp\|P35414\|APJ_HUMAN Apelin receptor OS=Homo sapiens GN=APLNR PE=1 SV=1 | 334 | QSRCAGtSHSS | 0.201 | 0.026 | -0.993 | -0.255 |
| sp\|P35414\|APJ_HUMAN Apelin receptor OS=Homo sapiens GN=APLNR PE=1 SV=1 | 335 | SRCAGTsHSSS | 0.049 | -0.093 | -1.507 | -0.517 |
| sp\|P35414\|APJ_HUMAN Apelin receptor OS=Homo sapiens GN=APLNR PE=1 SV=1 | 337 | CAGTSHsSSGE | 0.247 | 0.072 | -0.637 | -0.106 |
| sp\|P35414\|APJ_HUMAN Apelin receptor OS=Homo sapiens GN=APLNR PE=1 SV=1 | 338 | AGTSHSsSGEK | 0.183 | -0.18 | -0.921 | -0.306 |
| sp\|P35414\|APJ_HUMAN Apelin receptor OS=Homo sapiens GN=APLNR PE=1 SV=1 | 339 | GTSHSSsGEKS | 0.163 | -0.039 | -0.947 | -0.274 |
| sp\|P35414\|APJ_HUMAN Apelin receptor OS=Homo sapiens GN=APLNR PE=1 SV=1 | 343 | SSSGEKsASYS | 0.071 | -0.259 | -1.543 | -0.577 |
| sp\|P35414\|APJ_HUMAN Apelin receptor OS=Homo sapiens GN=APLNR PE=1 SV=1 | 345 | SGEKSAsYSSG | 0.224 | 0.029 | -0.683 | -0.143 |
| sp\|P35414\|APJ_HUMAN Apelin receptor OS=Homo sapiens GN=APLNR PE=1 SV=1 | 347 | EKSASYsSGHS | 0.369 | 0.188 | -0.055 | 0.167 |
| sp\|P35414\|APJ_HUMAN Apelin receptor OS=Homo sapiens GN=APLNR PE=1 SV=1 | 348 | KSASYSsGHSQ | 0.051 | -0.378 | -1.519 | -0.615 |
| sp\|P35414\|APJ_HUMAN Apelin receptor OS=Homo sapiens GN=APLNR PE=1 SV=1 | 351 | SYSSGHsQGPG | 0.134 | -0.135 | -1.248 | -0.416 |
| sp\|P35414\|APJ_HUMAN Apelin receptor OS=Homo sapiens GN=APLNR PE=1 SV=1 | 369 | EQMHEKsIPYS | 0.322 | 0.364 | -0.166 | 0.173 |
| sp\|P35414\|APJ_HUMAN Apelin receptor OS=Homo sapiens GN=APLNR PE=1 SV=1 | 373 | EKSIPYsQETL | 0.151 | -0.043 | -0.778 | -0.223 |
| sp\|P35414\|APJ_HUMAN Apelin receptor OS=Homo sapiens GN=APLNR PE=1 SV=1 | 376 | IPYSQEtLVVD | 0.182 | -0.057 | -0.869 | -0.248 |
| sp\|P28336\|NMBR_HUMAN Neuromedin-B receptor OS=Homo sapiens GN=NMBR PE=1 SV=2 | 328 | FALYLLsESFR | 0.074 | -0.116 | -1.197 | -0.413 |
| sp\|P28336\|NMBR_HUMAN Neuromedin-B receptor OS=Homo sapiens GN=NMBR PE=1 SV=2 | 330 | LYLLSEsFRRH | 0.359 | -0.007 | -0.638 | -0.095 |
| sp\|P28336\|NMBR_HUMAN Neuromedin-B receptor OS=Homo sapiens GN=NMBR PE=1 SV=2 | 337 | FRRHFNsQLCC | 0.369 | 0.416 | -0.896 | -0.037 |
| sp\|P28336\|NMBR_HUMAN Neuromedin-B receptor OS=Homo sapiens GN=NMBR PE=1 SV=2 | 345 | LCCGRKsYQER | 0.52 | 0.223 | -0.079 | 0.221 |
| sp\|P28336\|NMBR_HUMAN Neuromedin-B receptor OS=Homo sapiens GN=NMBR PE=1 SV=2 | 351 | SYQERGtSYLL | 0.133 | 0.081 | -1.127 | -0.304 |
| sp\|P28336\|NMBR_HUMAN Neuromedin-B receptor OS=Homo sapiens GN=NMBR PE=1 SV=2 | 352 | YQERGTsYLLS | 0.228 | 0.662 | -0.166 | 0.241 |
| sp\|P28336\|NMBR_HUMAN Neuromedin-B receptor OS=Homo sapiens GN=NMBR PE=1 SV=2 | 356 | GTSYLLsSSAV | 0.097 | -0.137 | -1.384 | -0.475 |
| sp\|P28336\|NMBR_HUMAN Neuromedin-B receptor OS=Homo sapiens GN=NMBR PE=1 SV=2 | 357 | TSYLLSsSAVR | 0.042 | -0.314 | -1.658 | -0.643 |
| sp\|P28336\|NMBR_HUMAN Neuromedin-B receptor OS=Homo sapiens GN=NMBR PE=1 SV=2 | 358 | SYLLSSsAVRM | 0.209 | 0.048 | -0.952 | -0.232 |
| sp\|P28336\|NMBR_HUMAN Neuromedin-B receptor OS=Homo sapiens GN=NMBR PE=1 SV=2 | 363 | SSAVRMtSLKS | 0.09 | -0.023 | -1.245 | -0.393 |
| sp\|P28336\|NMBR_HUMAN Neuromedin-B receptor OS=Homo sapiens GN=NMBR PE=1 SV=2 | 364 | SAVRMTsLKSN | 0.462 | 0.725 | 0.067 | 0.418 |
| sp\|P28336\|NMBR_HUMAN Neuromedin-B receptor OS=Homo sapiens GN=NMBR PE=1 SV=2 | 367 | RMTSLKsNAKN | 0.101 | -0.032 | -1.213 | -0.381 |
| sp\|P28336\|NMBR_HUMAN Neuromedin-B receptor OS=Homo sapiens GN=NMBR PE=1 SV=2 | 374 | NAKNMVtNSVL | 0.192 | -0.134 | -0.784 | -0.242 |
| sp\|P28336\|NMBR_HUMAN Neuromedin-B receptor OS=Homo sapiens GN=NMBR PE=1 SV=2 | 376 | KNMVTNsVLLN | 0.071 | -0.04 | -1.302 | -0.424 |
| sp\|P28336\|NMBR_HUMAN Neuromedin-B receptor OS=Homo sapiens GN=NMBR PE=1 SV=2 | 383 | VLLNGHsMKQE | 0.203 | 0.1 | -0.571 | -0.089 |
| sp\|P30550\|GRPR_HUMAN Gastrin-releasing peptide receptor OS=Homo sapiens GN=GRPR PE=2 SV=1 | 326 | FALYLLsKSFR | 0.061 | -0.2 | -1.254 | -0.464 |
| sp\|P30550\|GRPR_HUMAN Gastrin-releasing peptide receptor OS=Homo sapiens GN=GRPR PE=2 SV=1 | 328 | LYLLSKsFRKQ | 0.536 | 0.226 | 0.098 | 0.287 |
| sp\|P30550\|GRPR_HUMAN Gastrin-releasing peptide receptor OS=Homo sapiens GN=GRPR PE=2 SV=1 | 335 | FRKQFNtQLLC | 0.305 | 0.202 | -0.921 | -0.138 |
| sp\|P30550\|GRPR_HUMAN Gastrin-releasing peptide receptor OS=Homo sapiens GN=GRPR PE=2 SV=1 | 348 | PGLIIRsHSTG | 0.126 | -0.11 | -0.909 | -0.298 |
| sp\|P30550\|GRPR_HUMAN Gastrin-releasing peptide receptor OS=Homo sapiens GN=GRPR PE=2 SV=1 | 350 | LIIRSHsTGRS | 0.843 | 1.22 | 0.849 | 0.971 |
| sp\|P30550\|GRPR_HUMAN Gastrin-releasing peptide receptor OS=Homo sapiens GN=GRPR PE=2 SV=1 | 351 | IIRSHStGRST | 0.189 | -0.002 | -0.72 | -0.178 |
| sp\|P30550\|GRPR_HUMAN Gastrin-releasing peptide receptor OS=Homo sapiens GN=GRPR PE=2 SV=1 | 354 | SHSTGRsTTCM | 0.051 | -0.248 | -1.945 | -0.714 |
| sp\|P30550\|GRPR_HUMAN Gastrin-releasing peptide receptor OS=Homo sapiens GN=GRPR PE=2 SV=1 | 355 | HSTGRStTCMT | 0.094 | -0.177 | -1.043 | -0.375 |
| sp\|P30550\|GRPR_HUMAN Gastrin-releasing peptide receptor OS=Homo sapiens GN=GRPR PE=2 SV=1 | 356 | STGRSTtCMTS | 0.624 | 0.99 | 0.214 | 0.609 |
| sp\|P30550\|GRPR_HUMAN Gastrin-releasing peptide receptor OS=Homo sapiens GN=GRPR PE=2 SV=1 | 359 | RSTTCMtSLKS | 0.193 | -0.031 | -0.981 | -0.273 |
| sp\|P30550\|GRPR_HUMAN Gastrin-releasing peptide receptor OS=Homo sapiens GN=GRPR PE=2 SV=1 | 360 | STTCMTsLKST | 0.152 | -0.195 | -0.863 | -0.302 |
| sp\|P30550\|GRPR_HUMAN Gastrin-releasing peptide receptor OS=Homo sapiens GN=GRPR PE=2 SV=1 | 363 | CMTSLKsTNPS | 0.14 | -0.003 | -0.689 | -0.184 |
| sp\|P30550\|GRPR_HUMAN Gastrin-releasing peptide receptor OS=Homo sapiens GN=GRPR PE=2 SV=1 | 364 | MTSLKStNPSV | 0.241 | 0.282 | -0.994 | -0.157 |
| sp\|P30550\|GRPR_HUMAN Gastrin-releasing peptide receptor OS=Homo sapiens GN=GRPR PE=2 SV=1 | 367 | LKSTNPsVATF | 0.184 | -0.173 | -0.721 | -0.237 |
| sp\|P30550\|GRPR_HUMAN Gastrin-releasing peptide receptor OS=Homo sapiens GN=GRPR PE=2 SV=1 | 370 | TNPSVAtFSLI | 0.151 | -0.158 | -0.934 | -0.314 |

| | | | | | | |
|---|---|---|---|---|---|---|
| sp\|P30550\|GRPR_HUMAN Gastrin-releasing peptide receptor OS=Homo sapiens GN=GRPR PE=2 SV=1 | 372 | PSVATFsLING | 0.246 | 0.107 | -0.463 | -0.037 |
| sp\|P32247\|BRS3_HUMAN Bombesin receptor subtype-3 OS=Homo sapiens GN=BRS3 PE=1 SV=1 | 334 | FALYWLsKSFQ | 0.077 | -0.166 | -1.145 | -0.411 |
| sp\|P32247\|BRS3_HUMAN Bombesin receptor subtype-3 OS=Homo sapiens GN=BRS3 PE=1 SV=1 | 336 | LYWLSKsFQKH | 0.435 | 0.343 | -0.067 | 0.237 |
| sp\|P32247\|BRS3_HUMAN Bombesin receptor subtype-3 OS=Homo sapiens GN=BRS3 PE=1 SV=1 | 360 | EPPVADtSLTT | 0.036 | -0.335 | -1.755 | -0.685 |
| sp\|P32247\|BRS3_HUMAN Bombesin receptor subtype-3 OS=Homo sapiens GN=BRS3 PE=1 SV=1 | 361 | PPVADTsLTTL | 0.343 | -0.028 | -0.494 | -0.06 |
| sp\|P32247\|BRS3_HUMAN Bombesin receptor subtype-3 OS=Homo sapiens GN=BRS3 PE=1 SV=1 | 363 | VADTSLtTLAV | 0.153 | 0.022 | -1.061 | -0.295 |
| sp\|P32247\|BRS3_HUMAN Bombesin receptor subtype-3 OS=Homo sapiens GN=BRS3 PE=1 SV=1 | 364 | ADTSLTtLAVM | 0.106 | -0.209 | -0.992 | -0.365 |
| sp\|P32247\|BRS3_HUMAN Bombesin receptor subtype-3 OS=Homo sapiens GN=BRS3 PE=1 SV=1 | 370 | TLAVMGtVPGT | 0.374 | 0.285 | -0.107 | 0.184 |
| sp\|P32247\|BRS3_HUMAN Bombesin receptor subtype-3 OS=Homo sapiens GN=BRS3 PE=1 SV=1 | 374 | MGTVPGtGSIQ | 0.055 | -0.325 | -1.362 | -0.544 |
| sp\|P32247\|BRS3_HUMAN Bombesin receptor subtype-3 OS=Homo sapiens GN=BRS3 PE=1 SV=1 | 376 | TVPGTGsIQMS | 0.03 | -0.299 | -1.66 | -0.643 |
| sp\|P32247\|BRS3_HUMAN Bombesin receptor subtype-3 OS=Homo sapiens GN=BRS3 PE=1 SV=1 | 380 | TGSIQMsEISV | 0.087 | 0.046 | -1.114 | -0.327 |
| sp\|P32247\|BRS3_HUMAN Bombesin receptor subtype-3 OS=Homo sapiens GN=BRS3 PE=1 SV=1 | 383 | IQMSEIsVTSF | 0.098 | -0.193 | -1.059 | -0.385 |
| sp\|P32247\|BRS3_HUMAN Bombesin receptor subtype-3 OS=Homo sapiens GN=BRS3 PE=1 SV=1 | 385 | MSEISVtSFTG | 0.526 | 0.167 | -0.073 | 0.207 |
| sp\|P32247\|BRS3_HUMAN Bombesin receptor subtype-3 OS=Homo sapiens GN=BRS3 PE=1 SV=1 | 386 | SEISVTsFTGC | 0.145 | -0.221 | -1.339 | -0.472 |
| sp\|P32247\|BRS3_HUMAN Bombesin receptor subtype-3 OS=Homo sapiens GN=BRS3 PE=1 SV=1 | 388 | ISVTSFtGCSV | 0.151 | -0.059 | -0.96 | -0.289 |
| sp\|P32247\|BRS3_HUMAN Bombesin receptor subtype-3 OS=Homo sapiens GN=BRS3 PE=1 SV=1 | 391 | TSFTGCsVKQA | 0.139 | -0.239 | -1.093 | -0.398 |
| sp\|P46663\|BKRB1_HUMAN B1 bradykinin receptor OS=Homo sapiens GN=BDKRB1 PE=1 SV=3 | 321 | VGRLFRtKVWE | 0.219 | 0.091 | -0.797 | -0.162 |
| sp\|P46663\|BKRB1_HUMAN B1 bradykinin receptor OS=Homo sapiens GN=BDKRB1 PE=1 SV=3 | 331 | ELYKQCtPKSL | 0.091 | -0.214 | -1.199 | -0.441 |
| sp\|P46663\|BKRB1_HUMAN B1 bradykinin receptor OS=Homo sapiens GN=BDKRB1 PE=1 SV=3 | 334 | KQCTPKsLAPI | 0.306 | -0.078 | -0.232 | -0.001 |
| sp\|P46663\|BKRB1_HUMAN B1 bradykinin receptor OS=Homo sapiens GN=BDKRB1 PE=1 SV=3 | 339 | KSLAPIsSSHR | 0.069 | -0.41 | -1.205 | -0.515 |
| sp\|P46663\|BKRB1_HUMAN B1 bradykinin receptor OS=Homo sapiens GN=BDKRB1 PE=1 SV=3 | 340 | SLAPISsSHRK | 0.111 | -0.267 | -1.148 | -0.435 |
| sp\|P46663\|BKRB1_HUMAN B1 bradykinin receptor OS=Homo sapiens GN=BDKRB1 PE=1 SV=3 | 341 | LAPISSsHRKE | 0.125 | -0.105 | -0.968 | -0.316 |
| sp\|P30411\|BKRB2_HUMAN B2 bradykinin receptor OS=Homo sapiens GN=BDKRB2 PE=1 SV=2 | 343 | KRFRKKsWEVY | 0.693 | 1.202 | 0.513 | 0.803 |
| sp\|P30411\|BKRB2_HUMAN B2 bradykinin receptor OS=Homo sapiens GN=BDKRB2 PE=1 SV=2 | 358 | QKGGCRsEPIQ | 0.645 | 0.535 | 0.057 | 0.412 |
| sp\|P30411\|BKRB2_HUMAN B2 bradykinin receptor OS=Homo sapiens GN=BDKRB2 PE=1 SV=2 | 366 | PIQMENsMGTL | 0.479 | 0.26 | -0.456 | 0.094 |
| sp\|P30411\|BKRB2_HUMAN B2 bradykinin receptor OS=Homo sapiens GN=BDKRB2 PE=1 SV=2 | 369 | MENSMGtLRTS | 0.195 | -0.205 | -1.01 | -0.34 |
| sp\|P30411\|BKRB2_HUMAN B2 bradykinin receptor OS=Homo sapiens GN=BDKRB2 PE=1 SV=2 | 372 | SMGTLRtSISV | 0.018 | -0.191 | -2.265 | -0.813 |
| sp\|P30411\|BKRB2_HUMAN B2 bradykinin receptor OS=Homo sapiens GN=BDKRB2 PE=1 SV=2 | 373 | MGTLRTsISVE | 0.232 | 0.075 | -0.566 | -0.086 |
| sp\|P30411\|BKRB2_HUMAN B2 bradykinin receptor OS=Homo sapiens GN=BDKRB2 PE=1 SV=2 | 375 | TLRTSIsVERQ | 0.635 | 0.36 | -0.059 | 0.312 |
| sp\|P30411\|BKRB2_HUMAN B2 bradykinin receptor OS=Homo sapiens GN=BDKRB2 PE=1 SV=2 | 389 | LQDWAGsRQ-- | 0.141 | -0.068 | -0.891 | -0.273 |
| sp\|P32238\|CCKAR_HUMAN Cholecystokinin receptor type A OS=Homo sapiens GN=CCKAR PE=1 SV=1 | 384 | RLGFMAtFPCC | 0.774 | 0.635 | 0.24 | 0.55 |
| sp\|P32238\|CCKAR_HUMAN Cholecystokinin receptor type A OS=Homo sapiens GN=CCKAR PE=1 SV=1 | 408 | EEEEGGtTGAS | 0.056 | -0.19 | -1.385 | -0.506 |
| sp\|P32238\|CCKAR_HUMAN Cholecystokinin receptor type A OS=Homo sapiens GN=CCKAR PE=1 SV=1 | 409 | EEEGGTtGASL | 0.031 | -0.28 | -1.432 | -0.56 |
| sp\|P32238\|CCKAR_HUMAN Cholecystokinin receptor type A OS=Homo sapiens GN=CCKAR PE=1 SV=1 | 412 | GGTTGAsLSRF | 0.201 | -0.124 | -0.83 | -0.251 |
| sp\|P32238\|CCKAR_HUMAN Cholecystokinin receptor type A OS=Homo sapiens GN=CCKAR PE=1 SV=1 | 414 | TTGASLsRFSY | 0.124 | -0.056 | -1.03 | -0.321 |
| sp\|P32238\|CCKAR_HUMAN Cholecystokinin receptor type A OS=Homo sapiens GN=CCKAR PE=1 SV=1 | 417 | ASLSRFsYSHM | 0.111 | -0.187 | -0.956 | -0.344 |
| sp\|P32238\|CCKAR_HUMAN Cholecystokinin receptor type A OS=Homo sapiens GN=CCKAR PE=1 SV=1 | 419 | LSRFSYsHMSA | 0.507 | 0.415 | 0.179 | 0.367 |
| sp\|P32238\|CCKAR_HUMAN Cholecystokinin receptor type A OS=Homo sapiens GN=CCKAR PE=1 SV=1 | 422 | FSYSHMsASVP | 0.143 | 0.047 | -1.063 | -0.291 |
| sp\|P32238\|CCKAR_HUMAN Cholecystokinin receptor type A OS=Homo sapiens GN=CCKAR PE=1 SV=1 | 424 | YSHMSAsVPPQ | 0.595 | 0.492 | 0.156 | 0.414 |
| sp\|P32239\|GASR_HUMAN Gastrin/cholecystokinin type B receptor OS=Homo sapiens GN=CCKBR PE=1 SV=1 | 404 | RQACLEtCARC | 0.099 | -0.245 | -1.588 | -0.578 |
| sp\|P32239\|GASR_HUMAN Gastrin/cholecystokinin type B receptor OS=Homo sapiens GN=CCKBR PE=1 SV=1 | 427 | PDEDPPtPSIA | 0.04 | -0.644 | -1.637 | -0.747 |
| sp\|P32239\|GASR_HUMAN Gastrin/cholecystokinin type B receptor OS=Homo sapiens GN=CCKBR PE=1 SV=1 | 429 | EDPPTPsIASL | 0.016 | -0.401 | -1.789 | -0.725 |
| sp\|P32239\|GASR_HUMAN Gastrin/cholecystokinin type B receptor OS=Homo sapiens GN=CCKBR PE=1 SV=1 | 432 | PTPSIAsLSRL | 0.234 | -0.118 | -0.81 | -0.231 |
| sp\|P32239\|GASR_HUMAN Gastrin/cholecystokinin type B receptor OS=Homo sapiens GN=CCKBR PE=1 SV=1 | 434 | PSIASLsRLSY | 0.117 | -0.035 | -1.077 | -0.332 |
| sp\|P32239\|GASR_HUMAN Gastrin/cholecystokinin type B receptor OS=Homo sapiens GN=CCKBR PE=1 SV=1 | 437 | ASLSRLsYTTI | 0.208 | -0.12 | -0.786 | -0.233 |
| sp\|P32239\|GASR_HUMAN Gastrin/cholecystokinin type B receptor OS=Homo sapiens GN=CCKBR PE=1 SV=1 | 439 | LSRLSYtTIST | 0.267 | 0.324 | -0.138 | 0.151 |
| sp\|P32239\|GASR_HUMAN Gastrin/cholecystokinin type B receptor OS=Homo sapiens GN=CCKBR PE=1 SV=1 | 440 | SRLSYTtISTL | 0.09 | -0.01 | -1.036 | -0.319 |
| sp\|P32239\|GASR_HUMAN Gastrin/cholecystokinin type B receptor OS=Homo sapiens GN=CCKBR PE=1 SV=1 | 442 | LSYTTIsTLGP | 0.056 | -0.264 | -1.334 | -0.514 |
| sp\|P32239\|GASR_HUMAN Gastrin/cholecystokinin type B receptor OS=Homo sapiens GN=CCKBR PE=1 SV=1 | 443 | SYTTIStLGPG | 0.254 | -0.036 | -0.497 | -0.093 |

| | | | | | | |
|---|---|---|---|---|---|---|
| sp\|Q16581\|C3AR_HUMAN C3a anaphylatoxin chemotactic receptor OS=Homo sapiens GN=C3AR1 PE=1 SV=2 | 449 | RKKARQsIQGI | 0.705 | 0.242 | 0.422 | 0.456 |
| sp\|Q16581\|C3AR_HUMAN C3a anaphylatoxin chemotactic receptor OS=Homo sapiens GN=C3AR1 PE=1 SV=2 | 459 | ILEAAFsEELT | 0.466 | 0.162 | -0.065 | 0.188 |
| sp\|Q16581\|C3AR_HUMAN C3a anaphylatoxin chemotactic receptor OS=Homo sapiens GN=C3AR1 PE=1 SV=2 | 463 | AFSEELtRSTH | 0.029 | -0.486 | -2.043 | -0.833 |
| sp\|Q16581\|C3AR_HUMAN C3a anaphylatoxin chemotactic receptor OS=Homo sapiens GN=C3AR1 PE=1 SV=2 | 465 | SEELTRsTHCP | 0.049 | -0.139 | -1.769 | -0.62 |
| sp\|Q16581\|C3AR_HUMAN C3a anaphylatoxin chemotactic receptor OS=Homo sapiens GN=C3AR1 PE=1 SV=2 | 466 | EELTRStHCPS | 0.101 | -0.186 | -1.267 | -0.451 |
| sp\|Q16581\|C3AR_HUMAN C3a anaphylatoxin chemotactic receptor OS=Homo sapiens GN=C3AR1 PE=1 SV=2 | 470 | RSTHCPsNNVI | 0.366 | -0.03 | -0.423 | -0.029 |
| sp\|Q16581\|C3AR_HUMAN C3a anaphylatoxin chemotactic receptor OS=Homo sapiens GN=C3AR1 PE=1 SV=2 | 475 | PSNNVIsERNS | 0.202 | -0.075 | -0.936 | -0.27 |
| sp\|Q16581\|C3AR_HUMAN C3a anaphylatoxin chemotactic receptor OS=Homo sapiens GN=C3AR1 PE=1 SV=2 | 479 | VISERNsTTV- | 0.344 | 0.179 | -0.744 | -0.074 |
| sp\|Q16581\|C3AR_HUMAN C3a anaphylatoxin chemotactic receptor OS=Homo sapiens GN=C3AR1 PE=1 SV=2 | 480 | ISERNStTV-- | 0.513 | 0.8 | 0.164 | 0.492 |
| sp\|Q16581\|C3AR_HUMAN C3a anaphylatoxin chemotactic receptor OS=Homo sapiens GN=C3AR1 PE=1 SV=2 | 481 | SERNSTtV--- | 0.434 | 0.51 | -0.147 | 0.266 |
| sp\|P21730\|C5AR1_HUMAN C5a anaphylatoxin chemotactic receptor 1 OS=Homo sapiens GN=C5AR1 PE=1 SV | 314 | QGRLRKsLPSL | 0.895 | 0.985 | 1.202 | 1.027 |
| sp\|P21730\|C5AR1_HUMAN C5a anaphylatoxin chemotactic receptor 1 OS=Homo sapiens GN=C5AR1 PE=1 S\ | 317 | LRKSLPsLLRN | 0.231 | 0.061 | -0.771 | -0.16 |
| sp\|P21730\|C5AR1_HUMAN C5a anaphylatoxin chemotactic receptor 1 OS=Homo sapiens GN=C5AR1 PE=1 S\ | 324 | LLRNVLtEESV | 0.239 | 0.266 | -0.619 | -0.038 |
| sp\|P21730\|C5AR1_HUMAN C5a anaphylatoxin chemotactic receptor 1 OS=Homo sapiens GN=C5AR1 PE=1 S\ | 327 | NVLTEEsVVRE | 0.112 | -0.241 | -1.46 | -0.53 |
| sp\|P21730\|C5AR1_HUMAN C5a anaphylatoxin chemotactic receptor 1 OS=Homo sapiens GN=C5AR1 PE=1 S\ | 332 | ESVVREsKSFT | 0.053 | -0.236 | -1.456 | -0.546 |
| sp\|P21730\|C5AR1_HUMAN C5a anaphylatoxin chemotactic receptor 1 OS=Homo sapiens GN=C5AR1 PE=1 SV | 334 | VVRESKsFTRS | 0.611 | 0.402 | 0.082 | 0.365 |
| sp\|P21730\|C5AR1_HUMAN C5a anaphylatoxin chemotactic receptor 1 OS=Homo sapiens GN=C5AR1 PE=1 S\ | 336 | RESKSFtRSTV | 0.37 | 0.097 | -0.714 | -0.082 |
| sp\|P21730\|C5AR1_HUMAN C5a anaphylatoxin chemotactic receptor 1 OS=Homo sapiens GN=C5AR1 PE=1 S\ | 338 | SKSFTRsTVDT | 0.152 | -0.016 | -1.041 | -0.302 |
| sp\|P21730\|C5AR1_HUMAN C5a anaphylatoxin chemotactic receptor 1 OS=Homo sapiens GN=C5AR1 PE=1 S\ | 339 | KSFTRStVDTM | 0.229 | -0.038 | -0.732 | -0.18 |
| sp\|P21730\|C5AR1_HUMAN C5a anaphylatoxin chemotactic receptor 1 OS=Homo sapiens GN=C5AR1 PE=1 S\ | 342 | TRSTVDtMAQK | 0.159 | -0.009 | -1.175 | -0.342 |
| sp\|P21730\|C5AR1_HUMAN C5a anaphylatoxin chemotactic receptor 1 OS=Homo sapiens GN=C5AR1 PE=1 SV | 347 | DTMAQKtQAV- | 0.175 | -0.068 | -1.227 | -0.373 |
| sp\|Q9P296\|C5AR2_HUMAN C5a anaphylatoxin chemotactic receptor 2 OS=Homo sapiens GN=C5AR2 PE=1 S\ | 302 | RAQLRRsLPAA | 0.857 | 0.797 | 0.643 | 0.766 |
| sp\|Q9P296\|C5AR2_HUMAN C5a anaphylatoxin chemotactic receptor 2 OS=Homo sapiens GN=C5AR2 PE=1 S\ | 314 | HWALREsQGQD | 0.424 | 0.162 | -0.407 | 0.06 |
| sp\|Q9P296\|C5AR2_HUMAN C5a anaphylatoxin chemotactic receptor 2 OS=Homo sapiens GN=C5AR2 PE=1 S\ | 320 | SQGQDEsVDSK | 0.063 | -0.261 | -1.324 | -0.507 |
| sp\|Q9P296\|C5AR2_HUMAN C5a anaphylatoxin chemotactic receptor 2 OS=Homo sapiens GN=C5AR2 PE=1 S\ | 323 | QDESVDsKKST | 0.046 | -0.441 | -1.578 | -0.658 |
| sp\|Q9P296\|C5AR2_HUMAN C5a anaphylatoxin chemotactic receptor 2 OS=Homo sapiens GN=C5AR2 PE=1 S\ | 326 | SVDSKKsTSHD | 0.049 | -0.292 | -1.341 | -0.528 |
| sp\|Q9P296\|C5AR2_HUMAN C5a anaphylatoxin chemotactic receptor 2 OS=Homo sapiens GN=C5AR2 PE=1 S\ | 327 | VDSKKStSHDL | 0.131 | -0.18 | -1.057 | -0.369 |
| sp\|Q9P296\|C5AR2_HUMAN C5a anaphylatoxin chemotactic receptor 2 OS=Homo sapiens GN=C5AR2 PE=1 S\ | 328 | DSKKSTsHDLV | 0.732 | 0.278 | 0.074 | 0.361 |
| sp\|Q9P296\|C5AR2_HUMAN C5a anaphylatoxin chemotactic receptor 2 OS=Homo sapiens GN=C5AR2 PE=1 S\ | 333 | TSHDLVsEMEV | 0.205 | -0.096 | -1.239 | -0.377 |
| sp\|P25101\|EDNRA_HUMAN Endothelin-1 receptor OS=Homo sapiens GN=EDNRA PE=1 SV=1 | 373 | IALYFVsKKFK | 0.223 | -0.22 | -0.66 | -0.219 |
| sp\|P25101\|EDNRA_HUMAN Endothelin-1 receptor OS=Homo sapiens GN=EDNRA PE=1 SV=1 | 382 | FKNCFQsCLCC | 0.263 | 0.117 | -1.082 | -0.234 |
| sp\|P25101\|EDNRA_HUMAN Endothelin-1 receptor OS=Homo sapiens GN=EDNRA PE=1 SV=1 | 391 | CCCCYQsKSLM | 0.333 | 0.013 | -0.625 | -0.093 |
| sp\|P25101\|EDNRA_HUMAN Endothelin-1 receptor OS=Homo sapiens GN=EDNRA PE=1 SV=1 | 393 | CCYQSKsLMTS | 0.736 | 0.387 | 0.546 | 0.556 |
| sp\|P25101\|EDNRA_HUMAN Endothelin-1 receptor OS=Homo sapiens GN=EDNRA PE=1 SV=1 | 396 | QSKSLMtSVPM | 0.108 | -0.165 | -1.03 | -0.362 |
| sp\|P25101\|EDNRA_HUMAN Endothelin-1 receptor OS=Homo sapiens GN=EDNRA PE=1 SV=1 | 397 | SKSLMTsVPMN | 0.558 | 0.369 | -0.142 | 0.262 |
| sp\|P25101\|EDNRA_HUMAN Endothelin-1 receptor OS=Homo sapiens GN=EDNRA PE=1 SV=1 | 403 | SVPMNGtSIQW | 0.039 | -0.332 | -1.507 | -0.6 |
| sp\|P25101\|EDNRA_HUMAN Endothelin-1 receptor OS=Homo sapiens GN=EDNRA PE=1 SV=1 | 404 | VPMNGTsIQWK | 0.061 | -0.212 | -0.97 | -0.374 |
| sp\|P25101\|EDNRA_HUMAN Endothelin-1 receptor OS=Homo sapiens GN=EDNRA PE=1 SV=1 | 417 | DQNNHNtDRSS | 0.163 | -0.084 | -1.111 | -0.344 |
| sp\|P25101\|EDNRA_HUMAN Endothelin-1 receptor OS=Homo sapiens GN=EDNRA PE=1 SV=1 | 420 | NHNTDRsSHKD | 0.096 | -0.161 | -1.678 | -0.581 |
| sp\|P25101\|EDNRA_HUMAN Endothelin-1 receptor OS=Homo sapiens GN=EDNRA PE=1 SV=1 | 421 | HNTDRSsHKDS | 0.247 | 0.063 | -0.544 | -0.078 |
| sp\|P25101\|EDNRA_HUMAN Endothelin-1 receptor OS=Homo sapiens GN=EDNRA PE=1 SV=1 | 425 | RSSHKDsMN-- | 0.136 | 0.017 | -1.111 | -0.319 |
| sp\|P24530\|EDNRB_HUMAN Endothelin receptor type B OS=Homo sapiens GN=EDNRB PE=1 SV=1 | 390 | IALYLVsKRFK | 0.204 | -0.284 | -0.729 | -0.27 |
| sp\|P24530\|EDNRB_HUMAN Endothelin receptor type B OS=Homo sapiens GN=EDNRB PE=1 SV=1 | 399 | FKNCFKsCLCC | 0.373 | 0.168 | -0.822 | -0.094 |
| sp\|P24530\|EDNRB_HUMAN Endothelin receptor type B OS=Homo sapiens GN=EDNRB PE=1 SV=1 | 407 | LCCWCQsFEEK | 0.546 | 0.07 | -0.208 | 0.136 |
| sp\|P24530\|EDNRB_HUMAN Endothelin receptor type B OS=Homo sapiens GN=EDNRB PE=1 SV=1 | 413 | SFEEKQsLEEK | 0.15 | -0.089 | -0.788 | -0.242 |
| sp\|P24530\|EDNRB_HUMAN Endothelin receptor type B OS=Homo sapiens GN=EDNRB PE=1 SV=1 | 419 | SLEEKQsCLKF | 0.065 | -0.08 | -1.418 | -0.478 |
| sp\|P24530\|EDNRB_HUMAN Endothelin receptor type B OS=Homo sapiens GN=EDNRB PE=1 SV=1 | 435 | GYDNFRsSNKY | 0.178 | -0.025 | -1.079 | -0.309 |
| sp\|P24530\|EDNRB_HUMAN Endothelin receptor type B OS=Homo sapiens GN=EDNRB PE=1 SV=1 | 436 | YDNFRSsNKYS | 0.079 | -0.156 | -1.427 | -0.501 |
| sp\|P24530\|EDNRB_HUMAN Endothelin receptor type B OS=Homo sapiens GN=EDNRB PE=1 SV=1 | 440 | RSSNKYsSS-- | 0.064 | -0.044 | -1.145 | -0.375 |

| | | | | | | |
|---|---|---|---|---|---|---|
| sp\|P24530\|EDNRB_HUMAN Endothelin receptor type B OS=Homo sapiens GN=EDNRB PE=1 SV=1 | 441 | SSNKYSsS--- | 0.123 | -0.063 | -1.289 | -0.41 |
| sp\|P24530\|EDNRB_HUMAN Endothelin receptor type B OS=Homo sapiens GN=EDNRB PE=1 SV=1 | 442 | SNKYSSs---- | 0.199 | 0.179 | -0.545 | -0.056 |
| sp\|P21462\|FPR1_HUMAN fMet-Leu-Phe receptor OS=Homo sapiens GN=FPR1 PE=1 SV=3 | 319 | IHALPAsLERA | 0.299 | -0.114 | -0.695 | -0.17 |
| sp\|P21462\|FPR1_HUMAN fMet-Leu-Phe receptor OS=Homo sapiens GN=FPR1 PE=1 SV=3 | 325 | SLERALtEDST | 0.572 | 0.917 | 0.341 | 0.61 |
| sp\|P21462\|FPR1_HUMAN fMet-Leu-Phe receptor OS=Homo sapiens GN=FPR1 PE=1 SV=3 | 328 | RALTEDsTQTS | 0.132 | -0.25 | -1.39 | -0.503 |
| sp\|P21462\|FPR1_HUMAN fMet-Leu-Phe receptor OS=Homo sapiens GN=FPR1 PE=1 SV=3 | 329 | ALTEDStQTSD | 0.097 | -0.191 | -1.359 | -0.484 |
| sp\|P21462\|FPR1_HUMAN fMet-Leu-Phe receptor OS=Homo sapiens GN=FPR1 PE=1 SV=3 | 331 | TEDSTQtSDTA | 0.081 | -0.192 | -1.228 | -0.446 |
| sp\|P21462\|FPR1_HUMAN fMet-Leu-Phe receptor OS=Homo sapiens GN=FPR1 PE=1 SV=3 | 332 | EDSTQTsDTAT | 0.1 | -0.169 | -1.177 | -0.415 |
| sp\|P21462\|FPR1_HUMAN fMet-Leu-Phe receptor OS=Homo sapiens GN=FPR1 PE=1 SV=3 | 334 | STQTSDtATNS | 0.293 | 0.093 | -1.155 | -0.256 |
| sp\|P21462\|FPR1_HUMAN fMet-Leu-Phe receptor OS=Homo sapiens GN=FPR1 PE=1 SV=3 | 336 | QTSDTAtNSTL | 0.1 | -0.126 | -1.371 | -0.466 |
| sp\|P21462\|FPR1_HUMAN fMet-Leu-Phe receptor OS=Homo sapiens GN=FPR1 PE=1 SV=3 | 338 | SDTATNsTLPS | 0.045 | -0.267 | -1.653 | -0.625 |
| sp\|P21462\|FPR1_HUMAN fMet-Leu-Phe receptor OS=Homo sapiens GN=FPR1 PE=1 SV=3 | 339 | DTATNStLPSA | 0.587 | 0.362 | 0.031 | 0.327 |
| sp\|P21462\|FPR1_HUMAN fMet-Leu-Phe receptor OS=Homo sapiens GN=FPR1 PE=1 SV=3 | 342 | TNSTLPsAEVE | 0.069 | -0.299 | -1.567 | -0.599 |
| sp\|P25090\|FPR2_HUMAN N-formyl peptide receptor 2 OS=Homo sapiens GN=FPR2 PE=2 SV=2 | 316 | RERLIHsLPTS | 0.873 | 0.907 | 0.782 | 0.854 |
| sp\|P25090\|FPR2_HUMAN N-formyl peptide receptor 2 OS=Homo sapiens GN=FPR2 PE=2 SV=2 | 319 | LIHSLPtSLER | 0.065 | -0.219 | -1.458 | -0.537 |
| sp\|P25090\|FPR2_HUMAN N-formyl peptide receptor 2 OS=Homo sapiens GN=FPR2 PE=2 SV=2 | 320 | IHSLPTsLERA | 0.218 | -0.179 | -0.852 | -0.271 |
| sp\|P25090\|FPR2_HUMAN N-formyl peptide receptor 2 OS=Homo sapiens GN=FPR2 PE=2 SV=2 | 326 | SLERALsEDSA | 0.635 | 0.945 | 0.45 | 0.677 |
| sp\|P25090\|FPR2_HUMAN N-formyl peptide receptor 2 OS=Homo sapiens GN=FPR2 PE=2 SV=2 | 329 | RALSEDsAPTN | 0.485 | 0.304 | -0.627 | 0.054 |
| sp\|P25090\|FPR2_HUMAN N-formyl peptide receptor 2 OS=Homo sapiens GN=FPR2 PE=2 SV=2 | 332 | SEDSAPtNDTA | 0.124 | -0.254 | -1.344 | -0.491 |
| sp\|P25090\|FPR2_HUMAN N-formyl peptide receptor 2 OS=Homo sapiens GN=FPR2 PE=2 SV=2 | 335 | SAPTNDtAANS | 0.058 | -0.315 | -1.957 | -0.738 |
| sp\|P25090\|FPR2_HUMAN N-formyl peptide receptor 2 OS=Homo sapiens GN=FPR2 PE=2 SV=2 | 339 | NDTAANsASPP | 0.128 | -0.17 | -1.17 | -0.404 |
| sp\|P25090\|FPR2_HUMAN N-formyl peptide receptor 2 OS=Homo sapiens GN=FPR2 PE=2 SV=2 | 341 | TAANSAsPPAE | 0.525 | 0.373 | 0.237 | 0.378 |
| sp\|P25090\|FPR2_HUMAN N-formyl peptide receptor 2 OS=Homo sapiens GN=FPR2 PE=2 SV=2 | 346 | ASPPAEtELQA | 0.116 | -0.225 | -1.171 | -0.427 |
| sp\|P25089\|FPR3_HUMAN N-formyl peptide receptor 3 OS=Homo sapiens GN=FPR3 PE=2 SV=2 | 316 | QERLIRsLPTS | 0.707 | 0.699 | 0.286 | 0.564 |
| sp\|P25089\|FPR3_HUMAN N-formyl peptide receptor 3 OS=Homo sapiens GN=FPR3 PE=2 SV=2 | 319 | LIRSLPtSLER | 0.194 | 0.073 | -0.849 | -0.194 |
| sp\|P25089\|FPR3_HUMAN N-formyl peptide receptor 3 OS=Homo sapiens GN=FPR3 PE=2 SV=2 | 320 | IRSLPTsLERA | 0.334 | 0.058 | -0.371 | 0.007 |
| sp\|P25089\|FPR3_HUMAN N-formyl peptide receptor 3 OS=Homo sapiens GN=FPR3 PE=2 SV=2 | 326 | SLERALtEVPD | 0.688 | 0.944 | 0.48 | 0.704 |
| sp\|P25089\|FPR3_HUMAN N-formyl peptide receptor 3 OS=Homo sapiens GN=FPR3 PE=2 SV=2 | 331 | LTEVPDsAQTS | 0.089 | -0.277 | -1.342 | -0.51 |
| sp\|P25089\|FPR3_HUMAN N-formyl peptide receptor 3 OS=Homo sapiens GN=FPR3 PE=2 SV=2 | 334 | VPDSAQtSNTD | 0.187 | -0.128 | -0.782 | -0.241 |
| sp\|P25089\|FPR3_HUMAN N-formyl peptide receptor 3 OS=Homo sapiens GN=FPR3 PE=2 SV=2 | 335 | PDSAQTsNTDT | 0.135 | -0.229 | -1.181 | -0.425 |
| sp\|P25089\|FPR3_HUMAN N-formyl peptide receptor 3 OS=Homo sapiens GN=FPR3 PE=2 SV=2 | 337 | SAQTSNtDTTS | 0.383 | 0.13 | -0.792 | -0.093 |
| sp\|P25089\|FPR3_HUMAN N-formyl peptide receptor 3 OS=Homo sapiens GN=FPR3 PE=2 SV=2 | 339 | QTSNTDtTSAS | 0.057 | -0.208 | -1.352 | -0.501 |
| sp\|P25089\|FPR3_HUMAN N-formyl peptide receptor 3 OS=Homo sapiens GN=FPR3 PE=2 SV=2 | 340 | TSNTDTtSASP | 0.037 | -0.418 | -1.831 | -0.737 |
| sp\|P25089\|FPR3_HUMAN N-formyl peptide receptor 3 OS=Homo sapiens GN=FPR3 PE=2 SV=2 | 341 | SNTDTTsASPP | 0.037 | -0.282 | -1.684 | -0.643 |
| sp\|P25089\|FPR3_HUMAN N-formyl peptide receptor 3 OS=Homo sapiens GN=FPR3 PE=2 SV=2 | 343 | TDTTSAsPPEE | 0.484 | 0.272 | -0.164 | 0.197 |
| sp\|P25089\|FPR3_HUMAN N-formyl peptide receptor 3 OS=Homo sapiens GN=FPR3 PE=2 SV=2 | 348 | ASPPEEtELQA | 0.072 | -0.342 | -1.54 | -0.603 |
| sp\|P47211\|GALR1_HUMAN Galanin receptor type 1 OS=Homo sapiens GN=GALR1 PE=1 SV=3 | 307 | IIYAFLsENFR | 0.173 | -0.025 | -0.707 | -0.186 |
| sp\|P47211\|GALR1_HUMAN Galanin receptor type 1 OS=Homo sapiens GN=GALR1 PE=1 SV=3 | 326 | CHIRKDsHLSD | 0.364 | 0.715 | -0.434 | 0.215 |
| sp\|P47211\|GALR1_HUMAN Galanin receptor type 1 OS=Homo sapiens GN=GALR1 PE=1 SV=3 | 329 | RKDSHLsDTKE | 0.277 | -0.046 | -0.988 | -0.252 |
| sp\|P47211\|GALR1_HUMAN Galanin receptor type 1 OS=Homo sapiens GN=GALR1 PE=1 SV=3 | 331 | DSHLSDtKESK | 0.142 | -0.128 | -1.166 | -0.384 |
| sp\|P47211\|GALR1_HUMAN Galanin receptor type 1 OS=Homo sapiens GN=GALR1 PE=1 SV=3 | 334 | LSDTKEsKSRI | 0.081 | -0.353 | -1.437 | -0.57 |
| sp\|P47211\|GALR1_HUMAN Galanin receptor type 1 OS=Homo sapiens GN=GALR1 PE=1 SV=3 | 336 | DTKESKsRIDT | 0.461 | 0.152 | -0.14 | 0.158 |
| sp\|P47211\|GALR1_HUMAN Galanin receptor type 1 OS=Homo sapiens GN=GALR1 PE=1 SV=3 | 340 | SKSRIDtPPST | 0.554 | 0.859 | 0.271 | 0.561 |
| sp\|P47211\|GALR1_HUMAN Galanin receptor type 1 OS=Homo sapiens GN=GALR1 PE=1 SV=3 | 343 | RIDTPPsTNCT | 0.142 | -0.111 | -0.932 | -0.3 |
| sp\|P47211\|GALR1_HUMAN Galanin receptor type 1 OS=Homo sapiens GN=GALR1 PE=1 SV=3 | 344 | IDTPPStNCTH | 0.026 | -0.621 | -1.847 | -0.814 |
| sp\|P47211\|GALR1_HUMAN Galanin receptor type 1 OS=Homo sapiens GN=GALR1 PE=1 SV=3 | 347 | PPSTNCtHV-- | 0.29 | 0.049 | -0.876 | -0.179 |
| sp\|O43603\|GALR2_HUMAN Galanin receptor type 2 OS=Homo sapiens GN=GALR2 PE=1 SV=1 | 296 | IVYALVsKHFR | 0.194 | -0.164 | -0.774 | -0.248 |
| sp\|O43603\|GALR2_HUMAN Galanin receptor type 2 OS=Homo sapiens GN=GALR2 PE=1 SV=1 | 305 | FRKGFRtICAG | 0.206 | 0.205 | -0.668 | -0.086 |
| sp\|O43603\|GALR2_HUMAN Galanin receptor type 2 OS=Homo sapiens GN=GALR2 PE=1 SV=1 | 319 | RAPGRAsGRVC | 0.188 | -0.078 | -1.029 | -0.306 |

| | | | | | | |
|---|---|---|---|---|---|---|
| sp\|O43603\|GALR2_HUMAN Galanin receptor type 2 OS=Homo sapiens GN=GALR2 PE=1 SV=1 | 329 | CAAARGtHSGS | 0.187 | -0.134 | -0.797 | -0.248 |
| sp\|O43603\|GALR2_HUMAN Galanin receptor type 2 OS=Homo sapiens GN=GALR2 PE=1 SV=1 | 331 | AARGTHsGSVL | 0.201 | 0.238 | -0.69 | -0.084 |
| sp\|O43603\|GALR2_HUMAN Galanin receptor type 2 OS=Homo sapiens GN=GALR2 PE=1 SV=1 | 333 | RGTHSGsVLER | 0.292 | 0.127 | -0.397 | 0.007 |
| sp\|O43603\|GALR2_HUMAN Galanin receptor type 2 OS=Homo sapiens GN=GALR2 PE=1 SV=1 | 339 | SVLEREsSDLL | 0.121 | 0.045 | -1.067 | -0.3 |
| sp\|O43603\|GALR2_HUMAN Galanin receptor type 2 OS=Homo sapiens GN=GALR2 PE=1 SV=1 | 340 | VLERESsDLLH | 0.439 | 0.681 | -0.364 | 0.252 |
| sp\|O43603\|GALR2_HUMAN Galanin receptor type 2 OS=Homo sapiens GN=GALR2 PE=1 SV=1 | 346 | SDLLHMsEAAG | 0.213 | 0.093 | -0.733 | -0.142 |
| sp\|O43603\|GALR2_HUMAN Galanin receptor type 2 OS=Homo sapiens GN=GALR2 PE=1 SV=1 | 359 | RPCPGAsQPCI | 0.533 | 0.436 | -0.31 | 0.22 |
| sp\|O43603\|GALR2_HUMAN Galanin receptor type 2 OS=Homo sapiens GN=GALR2 PE=1 SV=1 | 371 | EPCPGPsWQGP | 0.051 | -0.282 | -1.339 | -0.523 |
| sp\|O43603\|GALR2_HUMAN Galanin receptor type 2 OS=Homo sapiens GN=GALR2 PE=1 SV=1 | 380 | GPKAGDsILTV | 0.092 | -0.281 | -1.507 | -0.565 |
| sp\|O43603\|GALR2_HUMAN Galanin receptor type 2 OS=Homo sapiens GN=GALR2 PE=1 SV=1 | 383 | AGDSILtVDVA | 0.11 | -0.168 | -0.897 | -0.318 |
| sp\|O60755\|GALR3_HUMAN Galanin receptor type 3 OS=Homo sapiens GN=GALR3 PE=1 SV=1 | 295 | LVYALAsRHFR | 0.09 | -0.221 | -1.261 | -0.464 |
| sp\|O60755\|GALR3_HUMAN Galanin receptor type 3 OS=Homo sapiens GN=GALR3 PE=1 SV=1 | 327 | RRVRPAsSGPP | 0.588 | 0.947 | 0.269 | 0.601 |
| sp\|O60755\|GALR3_HUMAN Galanin receptor type 3 OS=Homo sapiens GN=GALR3 PE=1 SV=1 | 328 | RVRPASsGPPG | 0.633 | 0.605 | 0.223 | 0.487 |
| sp\|O60755\|GALR3_HUMAN Galanin receptor type 3 OS=Homo sapiens GN=GALR3 PE=1 SV=1 | 340 | PGDARPsGRLL | 0.258 | -0.062 | -0.558 | -0.121 |
| sp\|Q92847\|GHSR_HUMAN Growth hormone secretagogue receptor type 1 OS=Homo sapiens GN=GHSR PE=: | 327 | ILYNIMsKKYR | 0.317 | 0.127 | -0.04 | 0.135 |
| sp\|Q92847\|GHSR_HUMAN Growth hormone secretagogue receptor type 1 OS=Homo sapiens GN=GHSR PE=: | 344 | LGFEPFsQRKL | 0.076 | -0.211 | -1.167 | -0.434 |
| sp\|Q92847\|GHSR_HUMAN Growth hormone secretagogue receptor type 1 OS=Homo sapiens GN=GHSR PE=: | 349 | FSQRKLsTLKD | 0.338 | 0.77 | -0.194 | 0.305 |
| sp\|Q92847\|GHSR_HUMAN Growth hormone secretagogue receptor type 1 OS=Homo sapiens GN=GHSR PE=: | 350 | SQRKLStLKDE | 0.415 | 0.177 | -0.266 | 0.109 |
| sp\|Q92847\|GHSR_HUMAN Growth hormone secretagogue receptor type 1 OS=Homo sapiens GN=GHSR PE=: | 355 | STLKDEsSRAW | 0.18 | -0.166 | -1.073 | -0.353 |
| sp\|Q92847\|GHSR_HUMAN Growth hormone secretagogue receptor type 1 OS=Homo sapiens GN=GHSR PE=: | 356 | TLKDESsRAWT | 0.108 | -0.211 | -1.193 | -0.432 |
| sp\|Q92847\|GHSR_HUMAN Growth hormone secretagogue receptor type 1 OS=Homo sapiens GN=GHSR PE=: | 360 | ESSRAWtESSI | 0.544 | 0.799 | 0.286 | 0.543 |
| sp\|Q92847\|GHSR_HUMAN Growth hormone secretagogue receptor type 1 OS=Homo sapiens GN=GHSR PE=: | 362 | SRAWTEsSINT | 0.077 | 0.032 | -1.175 | -0.355 |
| sp\|Q92847\|GHSR_HUMAN Growth hormone secretagogue receptor type 1 OS=Homo sapiens GN=GHSR PE=: | 363 | RAWTESsINT- | 0.093 | -0.076 | -1.231 | -0.405 |
| sp\|Q92847\|GHSR_HUMAN Growth hormone secretagogue receptor type 1 OS=Homo sapiens GN=GHSR PE=: | 366 | TESSINt---- | 0.163 | 0.006 | -1.002 | -0.278 |
| sp\|P30968\|GNRHR_HUMAN Gonadotropin-releasing hormone receptor OS=Homo sapiens GN=GNRHR PE=1 | 327 | LIYGYFsL--- | 0.351 | 0.333 | -0.12 | 0.188 |
| sp\|Q969F8\|KISSR_HUMAN KiSS-1 receptor OS=Homo sapiens GN=KISS1R PE=1 SV=2 | 328 | LYAFLGsHFRQ | 0.152 | -0.203 | -1.124 | -0.392 |
| sp\|Q969F8\|KISSR_HUMAN KiSS-1 receptor OS=Homo sapiens GN=KISS1R PE=1 SV=2 | 354 | PRRPGPsDPAA | 0.791 | 0.86 | 0.577 | 0.743 |
| sp\|Q969F8\|KISSR_HUMAN KiSS-1 receptor OS=Homo sapiens GN=KISS1R PE=1 SV=2 | 368 | ELLRLGsHPAP | 0.827 | 1.314 | 0.967 | 1.036 |
| sp\|Q969F8\|KISSR_HUMAN KiSS-1 receptor OS=Homo sapiens GN=KISS1R PE=1 SV=2 | 380 | RAQKPGsSGLA | 0.239 | -0.091 | -0.745 | -0.199 |
| sp\|Q969F8\|KISSR_HUMAN KiSS-1 receptor OS=Homo sapiens GN=KISS1R PE=1 SV=2 | 381 | AQKPGSsGLAA | 0.051 | -0.283 | -1.122 | -0.451 |
| sp\|Q99705\|MCHR1_HUMAN Melanin-concentrating hormone receptor 1 OS=Homo sapiens GN=MCHR1 PE= | 386 | YIVLCEtFRKR | 0.306 | 0.025 | -0.609 | -0.093 |
| sp\|Q99705\|MCHR1_HUMAN Melanin-concentrating hormone receptor 1 OS=Homo sapiens GN=MCHR1 PE= | 394 | RKRLVLsVKPA | 0.408 | 0.196 | -0.328 | 0.092 |
| sp\|Q99705\|MCHR1_HUMAN Melanin-concentrating hormone receptor 1 OS=Homo sapiens GN=MCHR1 PE= | 407 | GQLRAVsNAQT | 0.723 | 0.779 | 0.493 | 0.665 |
| sp\|Q99705\|MCHR1_HUMAN Melanin-concentrating hormone receptor 1 OS=Homo sapiens GN=MCHR1 PE= | 411 | AVSNAQtADEE | 0.264 | 0.045 | -0.592 | -0.094 |
| sp\|Q99705\|MCHR1_HUMAN Melanin-concentrating hormone receptor 1 OS=Homo sapiens GN=MCHR1 PE= | 417 | TADEERtESKG | 0.04 | -0.241 | -1.863 | -0.688 |
| sp\|Q99705\|MCHR1_HUMAN Melanin-concentrating hormone receptor 1 OS=Homo sapiens GN=MCHR1 PE= | 419 | DEERTEsKGT- | 0.614 | 0.817 | 0.033 | 0.488 |
| sp\|Q99705\|MCHR1_HUMAN Melanin-concentrating hormone receptor 1 OS=Homo sapiens GN=MCHR1 PE= | 422 | RTESKGt---- | 0.114 | -0.091 | -1.299 | -0.425 |
| sp\|Q969V1\|MCHR2_HUMAN Melanin-concentrating hormone receptor 2 OS=Homo sapiens GN=MCHR2 PE= | 310 | FLYILLsGNFQ | 0.081 | -0.088 | -1.148 | -0.385 |
| sp\|Q969V1\|MCHR2_HUMAN Melanin-concentrating hormone receptor 2 OS=Homo sapiens GN=MCHR2 PE= | 325 | QIQRRAtEKEI | 0.91 | 1.285 | 1.209 | 1.135 |
| sp\|Q969V1\|MCHR2_HUMAN Melanin-concentrating hormone receptor 2 OS=Homo sapiens GN=MCHR2 PE= | 335 | INNMGNtLKSH | 0.098 | -0.296 | -1.162 | -0.453 |
| sp\|Q969V1\|MCHR2_HUMAN Melanin-concentrating hormone receptor 2 OS=Homo sapiens GN=MCHR2 PE= | 338 | MGNTLKsHF-- | 0.304 | 0.16 | -0.55 | -0.029 |
| sp\|Q01726\|MSHR_HUMAN Melanocyte-stimulating hormone receptor OS=Homo sapiens GN=MC1R PE=1 SV | 302 | LIYAFHsQELR | 0.194 | 0.068 | -0.878 | -0.205 |
| sp\|Q01726\|MSHR_HUMAN Melanocyte-stimulating hormone receptor OS=Homo sapiens GN=MC1R PE=1 SV | 308 | SQELRRtLKEV | 0.302 | 0.106 | -0.734 | -0.109 |
| sp\|Q01726\|MSHR_HUMAN Melanocyte-stimulating hormone receptor OS=Homo sapiens GN=MC1R PE=1 SV | 314 | TLKEVLtCSW- | 0.128 | 0.053 | -1 | -0.273 |
| sp\|Q01726\|MSHR_HUMAN Melanocyte-stimulating hormone receptor OS=Homo sapiens GN=MC1R PE=1 SV | 316 | KEVLTCsW--- | 0.139 | 0.183 | -1.245 | -0.308 |
| sp\|Q01718\|ACTHR_HUMAN Adrenocorticotropic hormone receptor OS=Homo sapiens GN=MC2R PE=1 SV=1 | 280 | FIYAFRsPELR | 0.072 | -0.227 | -1.383 | -0.513 |
| sp\|Q01718\|ACTHR_HUMAN Adrenocorticotropic hormone receptor OS=Homo sapiens GN=MC2R PE=1 SV=1 | 294 | KKMIFCsRYW- | 0.258 | 0.027 | -0.95 | -0.222 |
| sp\|P41968\|MC3R_HUMAN Melanocortin receptor 3 OS=Homo sapiens GN=MC3R PE=1 SV=3 | 303 | LIYAFRsLELR | 0.219 | 0.074 | -0.64 | -0.116 |
| sp\|P41968\|MC3R_HUMAN Melanocortin receptor 3 OS=Homo sapiens GN=MC3R PE=1 SV=3 | 309 | SLELRNtFREI | 0.736 | 0.288 | 0.099 | 0.374 |

| | | | | | | |
|---|---|---|---|---|---|---|
| sp\|P32245\|MC4R_HUMAN Melanocortin receptor 4 OS=Homo sapiens GN=MC4R PE=1 SV=2 | 306 | LIYALRsQELR | 0.079 | -0.107 | -1.387 | -0.472 |
| sp\|P32245\|MC4R_HUMAN Melanocortin receptor 4 OS=Homo sapiens GN=MC4R PE=1 SV=2 | 312 | SQELRKtFKEI | 0.523 | 0.221 | 0.113 | 0.286 |
| sp\|P32245\|MC4R_HUMAN Melanocortin receptor 4 OS=Homo sapiens GN=MC4R PE=1 SV=2 | 329 | GGLCDLsSRY- | 0.139 | -0.214 | -1.194 | -0.423 |
| sp\|P32245\|MC4R_HUMAN Melanocortin receptor 4 OS=Homo sapiens GN=MC4R PE=1 SV=2 | 330 | GLCDLSsRY-- | 0.173 | 0.056 | -1.198 | -0.323 |
| sp\|P33032\|MC5R_HUMAN Melanocortin receptor 5 OS=Homo sapiens GN=MC5R PE=1 SV=3 | 299 | LIYAFRsQEMR | 0.089 | -0.159 | -1.436 | -0.502 |
| sp\|P33032\|MC5R_HUMAN Melanocortin receptor 5 OS=Homo sapiens GN=MC5R PE=1 SV=3 | 305 | SQEMRKtFKEI | 0.548 | 0.178 | 0.196 | 0.307 |
| sp\|P33032\|MC5R_HUMAN Melanocortin receptor 5 OS=Homo sapiens GN=MC5R PE=1 SV=3 | 320 | GFRIACsFPRR | 0.942 | 0.902 | 0.916 | 0.92 |
| sp\|O43193\|MTLR_HUMAN Motilin receptor OS=Homo sapiens GN=MLNR PE=2 SV=1 | 359 | ILYNLIsKKYR | 0.201 | -0.061 | -0.533 | -0.131 |
| sp\|O43193\|MTLR_HUMAN Motilin receptor OS=Homo sapiens GN=MLNR PE=2 SV=1 | 375 | LLLARKsRPRG | 0.838 | 0.582 | 0.592 | 0.671 |
| sp\|O43193\|MTLR_HUMAN Motilin receptor OS=Homo sapiens GN=MLNR PE=2 SV=1 | 383 | PRGFHRsRDTA | 0.253 | 0.157 | -0.773 | -0.121 |
| sp\|O43193\|MTLR_HUMAN Motilin receptor OS=Homo sapiens GN=MLNR PE=2 SV=1 | 386 | FHRSRDtAGEV | 0.66 | 0.407 | -0.629 | 0.146 |
| sp\|O43193\|MTLR_HUMAN Motilin receptor OS=Homo sapiens GN=MLNR PE=2 SV=1 | 394 | GEVAGDtGGDT | 0.173 | -0.198 | -1.098 | -0.374 |
| sp\|O43193\|MTLR_HUMAN Motilin receptor OS=Homo sapiens GN=MLNR PE=2 SV=1 | 398 | GDTGGDtVGYT | 0.095 | -0.255 | -1.215 | -0.458 |
| sp\|O43193\|MTLR_HUMAN Motilin receptor OS=Homo sapiens GN=MLNR PE=2 SV=1 | 402 | GDTVGYtETSA | 0.07 | -0.104 | -1.069 | -0.368 |
| sp\|O43193\|MTLR_HUMAN Motilin receptor OS=Homo sapiens GN=MLNR PE=2 SV=1 | 404 | TVGYTEtSANV | 0.049 | -0.126 | -1.734 | -0.604 |
| sp\|O43193\|MTLR_HUMAN Motilin receptor OS=Homo sapiens GN=MLNR PE=2 SV=1 | 405 | VGYTETsANVK | 0.159 | -0.11 | -0.955 | -0.302 |
| sp\|O43193\|MTLR_HUMAN Motilin receptor OS=Homo sapiens GN=MLNR PE=2 SV=1 | 410 | TSANVKtMG-- | 0.264 | 0.205 | -0.381 | 0.029 |
| sp\|Q9HB89\|NMUR1_HUMAN Neuromedin-U receptor 1 OS=Homo sapiens GN=NMUR1 PE=2 SV=1 | 360 | VLYSLMsSRFR | 0.136 | -0.116 | -0.978 | -0.319 |
| sp\|Q9HB89\|NMUR1_HUMAN Neuromedin-U receptor 1 OS=Homo sapiens GN=NMUR1 PE=2 SV=1 | 361 | LYSLMSsRFRE | 0.109 | -0.345 | -1.307 | -0.514 |
| sp\|Q9HB89\|NMUR1_HUMAN Neuromedin-U receptor 1 OS=Homo sapiens GN=NMUR1 PE=2 SV=1 | 366 | SSRFREtFQEA | 0.634 | 0.309 | -0.135 | 0.269 |
| sp\|Q9HB89\|NMUR1_HUMAN Neuromedin-U receptor 1 OS=Homo sapiens GN=NMUR1 PE=2 SV=1 | 385 | RLRPRHsSHSL | 0.675 | 0.56 | 0.081 | 0.439 |
| sp\|Q9HB89\|NMUR1_HUMAN Neuromedin-U receptor 1 OS=Homo sapiens GN=NMUR1 PE=2 SV=1 | 386 | LRPRHSsHSLS | 0.403 | 0.919 | -0.092 | 0.41 |
| sp\|Q9HB89\|NMUR1_HUMAN Neuromedin-U receptor 1 OS=Homo sapiens GN=NMUR1 PE=2 SV=1 | 388 | PRHSSHsLSRM | 0.502 | 0.41 | -0.119 | 0.264 |
| sp\|Q9HB89\|NMUR1_HUMAN Neuromedin-U receptor 1 OS=Homo sapiens GN=NMUR1 PE=2 SV=1 | 390 | HSSHSLsRMTT | 0.14 | -0.076 | -0.773 | -0.236 |
| sp\|Q9HB89\|NMUR1_HUMAN Neuromedin-U receptor 1 OS=Homo sapiens GN=NMUR1 PE=2 SV=1 | 393 | HSLSRMtTGST | 0.245 | 0.123 | -0.448 | -0.027 |
| sp\|Q9HB89\|NMUR1_HUMAN Neuromedin-U receptor 1 OS=Homo sapiens GN=NMUR1 PE=2 SV=1 | 394 | SLSRMTtGSTL | 0.457 | 0.693 | 0.07 | 0.407 |
| sp\|Q9HB89\|NMUR1_HUMAN Neuromedin-U receptor 1 OS=Homo sapiens GN=NMUR1 PE=2 SV=1 | 396 | SRMTTGsTLCD | 0.069 | 0.12 | -1.375 | -0.395 |
| sp\|Q9HB89\|NMUR1_HUMAN Neuromedin-U receptor 1 OS=Homo sapiens GN=NMUR1 PE=2 SV=1 | 397 | RMTTGStLCDV | 0.169 | 0.023 | -0.983 | -0.264 |
| sp\|Q9HB89\|NMUR1_HUMAN Neuromedin-U receptor 1 OS=Homo sapiens GN=NMUR1 PE=2 SV=1 | 403 | TLCDVGsLGSW | 0.295 | 0.075 | -0.678 | -0.103 |
| sp\|Q9HB89\|NMUR1_HUMAN Neuromedin-U receptor 1 OS=Homo sapiens GN=NMUR1 PE=2 SV=1 | 406 | DVGSLGsWVHP | 0.028 | -0.3 | -1.861 | -0.711 |
| sp\|Q9HB89\|NMUR1_HUMAN Neuromedin-U receptor 1 OS=Homo sapiens GN=NMUR1 PE=2 SV=1 | 423 | PEAQQEtDPS- | 0.394 | 0.421 | -0.493 | 0.107 |
| sp\|Q9HB89\|NMUR1_HUMAN Neuromedin-U receptor 1 OS=Homo sapiens GN=NMUR1 PE=2 SV=1 | 426 | QQETDPs---- | 0.117 | -0.076 | -0.994 | -0.318 |
| sp\|Q9GZQ4\|NMUR2_HUMAN Neuromedin-U receptor 2 OS=Homo sapiens GN=NMUR2 PE=1 SV=2 | 331 | IIYNLLsRRFQ | 0.053 | -0.338 | -1.443 | -0.576 |
| sp\|Q9GZQ4\|NMUR2_HUMAN Neuromedin-U receptor 2 OS=Homo sapiens GN=NMUR2 PE=1 SV=2 | 343 | AFQNVIsSFHK | 0.17 | -0.144 | -0.7 | -0.225 |
| sp\|Q9GZQ4\|NMUR2_HUMAN Neuromedin-U receptor 2 OS=Homo sapiens GN=NMUR2 PE=1 SV=2 | 344 | FQNVISsFHKQ | 0.087 | -0.066 | -1.037 | -0.339 |
| sp\|Q9GZQ4\|NMUR2_HUMAN Neuromedin-U receptor 2 OS=Homo sapiens GN=NMUR2 PE=1 SV=2 | 351 | FHKQWHsQHDP | 0.495 | 0.216 | -0.529 | 0.061 |
| sp\|Q9GZQ4\|NMUR2_HUMAN Neuromedin-U receptor 2 OS=Homo sapiens GN=NMUR2 PE=1 SV=2 | 367 | QRNIFLtECHF | 0.062 | -0.002 | -1.291 | -0.41 |
| sp\|Q9GZQ4\|NMUR2_HUMAN Neuromedin-U receptor 2 OS=Homo sapiens GN=NMUR2 PE=1 SV=2 | 375 | CHFVELtEDIG | 0.173 | 0.011 | -1.091 | -0.302 |
| sp\|Q9GZQ4\|NMUR2_HUMAN Neuromedin-U receptor 2 OS=Homo sapiens GN=NMUR2 PE=1 SV=2 | 386 | PQFPCQsSMHN | 0.165 | -0.101 | -0.743 | -0.226 |
| sp\|Q9GZQ4\|NMUR2_HUMAN Neuromedin-U receptor 2 OS=Homo sapiens GN=NMUR2 PE=1 SV=2 | 387 | QFPCQSsMHNS | 0.08 | -0.039 | -1.519 | -0.493 |
| sp\|Q9GZQ4\|NMUR2_HUMAN Neuromedin-U receptor 2 OS=Homo sapiens GN=NMUR2 PE=1 SV=2 | 391 | QSSMHNsHLPA | 0.208 | -0.189 | -0.927 | -0.303 |
| sp\|Q9GZQ4\|NMUR2_HUMAN Neuromedin-U receptor 2 OS=Homo sapiens GN=NMUR2 PE=1 SV=2 | 398 | HLPAALsSEQM | 0.157 | -0.029 | -0.72 | -0.197 |
| sp\|Q9GZQ4\|NMUR2_HUMAN Neuromedin-U receptor 2 OS=Homo sapiens GN=NMUR2 PE=1 SV=2 | 399 | LPAALSsEQMS | 0.066 | -0.235 | -1.27 | -0.48 |
| sp\|Q9GZQ4\|NMUR2_HUMAN Neuromedin-U receptor 2 OS=Homo sapiens GN=NMUR2 PE=1 SV=2 | 403 | LSSEQMsRTNY | 0.146 | -0.063 | -1.292 | -0.403 |
| sp\|Q9GZQ4\|NMUR2_HUMAN Neuromedin-U receptor 2 OS=Homo sapiens GN=NMUR2 PE=1 SV=2 | 405 | SEQMSRtNYQS | 0.158 | 0.012 | -1.165 | -0.332 |
| sp\|Q9GZQ4\|NMUR2_HUMAN Neuromedin-U receptor 2 OS=Homo sapiens GN=NMUR2 PE=1 SV=2 | 409 | SRTNYQsFHFN | 0.223 | 0.2 | -0.563 | -0.047 |
| sp\|Q9GZQ4\|NMUR2_HUMAN Neuromedin-U receptor 2 OS=Homo sapiens GN=NMUR2 PE=1 SV=2 | 415 | SFHFNKt---- | 0.227 | 0.118 | -0.612 | -0.089 |
| sp\|Q9GZQ6\|NPFF1_HUMAN Neuropeptide FF receptor 1 OS=Homo sapiens GN=NPFFR1 PE=2 SV=1 | 353 | RLCPRPsGSHK | 0.222 | -0.078 | -0.702 | -0.186 |
| sp\|Q9GZQ6\|NPFF1_HUMAN Neuropeptide FF receptor 1 OS=Homo sapiens GN=NPFFR1 PE=2 SV=1 | 355 | CPRPSGsHKEA | 0.55 | 0.28 | 0.002 | 0.277 |

| | | | | | | |
|---|---|---|---|---|---|---|
| sp\|Q9GZQ6\|NPFF1_HUMAN Neuropeptide FF receptor 1 OS=Homo sapiens GN=NPFFR1 PE=2 SV=1 | 361 | SHKEAYsERPG | 0.178 | 0.027 | -0.884 | -0.226 |
| sp\|Q9GZQ6\|NPFF1_HUMAN Neuropeptide FF receptor 1 OS=Homo sapiens GN=NPFFR1 PE=2 SV=1 | 379 | FVVVRPsDSGL | 0.168 | 0.08 | -0.865 | -0.206 |
| sp\|Q9GZQ6\|NPFF1_HUMAN Neuropeptide FF receptor 1 OS=Homo sapiens GN=NPFFR1 PE=2 SV=1 | 381 | VVRPSDsGLPS | 0.189 | 0.097 | -0.729 | -0.148 |
| sp\|Q9GZQ6\|NPFF1_HUMAN Neuropeptide FF receptor 1 OS=Homo sapiens GN=NPFFR1 PE=2 SV=1 | 385 | SDSGLPsESGP | 0.028 | -0.433 | -1.798 | -0.734 |
| sp\|Q9GZQ6\|NPFF1_HUMAN Neuropeptide FF receptor 1 OS=Homo sapiens GN=NPFFR1 PE=2 SV=1 | 387 | SGLPSEsGPSS | 0.272 | 0.37 | -0.373 | 0.09 |
| sp\|Q9GZQ6\|NPFF1_HUMAN Neuropeptide FF receptor 1 OS=Homo sapiens GN=NPFFR1 PE=2 SV=1 | 390 | PSESGPsSGAP | 0.105 | -0.266 | -1.047 | -0.403 |
| sp\|Q9GZQ6\|NPFF1_HUMAN Neuropeptide FF receptor 1 OS=Homo sapiens GN=NPFFR1 PE=2 SV=1 | 391 | SESGPSsGAPR | 0.035 | -0.47 | -1.698 | -0.711 |
| sp\|Q9GZQ6\|NPFF1_HUMAN Neuropeptide FF receptor 1 OS=Homo sapiens GN=NPFFR1 PE=2 SV=1 | 419 | REGPGCsHLPL | 0.09 | -0.184 | -1.448 | -0.514 |
| sp\|Q9GZQ6\|NPFF1_HUMAN Neuropeptide FF receptor 1 OS=Homo sapiens GN=NPFFR1 PE=2 SV=1 | 424 | CSHLPLtIPAW | 0.251 | 0.308 | -0.272 | 0.096 |
| sp\|Q9Y5X5\|NPFF2_HUMAN Neuropeptide FF receptor 2 OS=Homo sapiens GN=NPFFR2 PE=1 SV=2 | 471 | YALKAKsHVLI | 0.617 | 0.206 | 0.225 | 0.349 |
| sp\|Q9Y5X5\|NPFF2_HUMAN Neuropeptide FF receptor 2 OS=Homo sapiens GN=NPFFR2 PE=1 SV=2 | 477 | SHVLINtSNQL | 0.147 | -0.053 | -1.137 | -0.348 |
| sp\|Q9Y5X5\|NPFF2_HUMAN Neuropeptide FF receptor 2 OS=Homo sapiens GN=NPFFR2 PE=1 SV=2 | 478 | HVLINTsNQLV | 0.166 | 0.03 | -0.857 | -0.22 |
| sp\|Q9Y5X5\|NPFF2_HUMAN Neuropeptide FF receptor 2 OS=Homo sapiens GN=NPFFR2 PE=1 SV=2 | 485 | NQLVQEsTFQN | 0.081 | -0.154 | -1.208 | -0.427 |
| sp\|Q9Y5X5\|NPFF2_HUMAN Neuropeptide FF receptor 2 OS=Homo sapiens GN=NPFFR2 PE=1 SV=2 | 486 | QLVQEStFQNP | 0.21 | 0.001 | -0.804 | -0.198 |
| sp\|Q9Y5X5\|NPFF2_HUMAN Neuropeptide FF receptor 2 OS=Homo sapiens GN=NPFFR2 PE=1 SV=2 | 494 | QNPHGEtLLYR | 0.065 | -0.302 | -1.354 | -0.53 |
| sp\|Q9Y5X5\|NPFF2_HUMAN Neuropeptide FF receptor 2 OS=Homo sapiens GN=NPFFR2 PE=1 SV=2 | 500 | TLLYRKsAEKP | 0.391 | 0.22 | -0.248 | 0.121 |
| sp\|Q9Y5X5\|NPFF2_HUMAN Neuropeptide FF receptor 2 OS=Homo sapiens GN=NPFFR2 PE=1 SV=2 | 516 | MEELKEtTNSS | 0.077 | -0.159 | -1.403 | -0.495 |
| sp\|Q9Y5X5\|NPFF2_HUMAN Neuropeptide FF receptor 2 OS=Homo sapiens GN=NPFFR2 PE=1 SV=2 | 517 | EELKETtNSSE | 0.074 | -0.252 | -1.515 | -0.564 |
| sp\|Q9Y5X5\|NPFF2_HUMAN Neuropeptide FF receptor 2 OS=Homo sapiens GN=NPFFR2 PE=1 SV=2 | 519 | LKETTNsSEI- | 0.278 | 0.053 | -0.747 | -0.139 |
| sp\|Q9Y5X5\|NPFF2_HUMAN Neuropeptide FF receptor 2 OS=Homo sapiens GN=NPFFR2 PE=1 SV=2 | 520 | KETTNSsEI-- | 0.12 | 0.025 | -0.974 | -0.276 |
| sp\|Q6W5P4\|NPSR1_HUMAN Neuropeptide S receptor OS=Homo sapiens GN=NPSR1 PE=1 SV=1 | 334 | LIYCVFsSSIS | 0.128 | -0.074 | -1.098 | -0.348 |
| sp\|Q6W5P4\|NPSR1_HUMAN Neuropeptide S receptor OS=Homo sapiens GN=NPSR1 PE=1 SV=1 | 335 | IYCVFSsSISF | 0.05 | -0.219 | -1.436 | -0.535 |
| sp\|Q6W5P4\|NPSR1_HUMAN Neuropeptide S receptor OS=Homo sapiens GN=NPSR1 PE=1 SV=1 | 336 | YCVFSSsISFP | 0.1 | -0.052 | -1.121 | -0.358 |
| sp\|Q6W5P4\|NPSR1_HUMAN Neuropeptide S receptor OS=Homo sapiens GN=NPSR1 PE=1 SV=1 | 338 | VFSSSIsFPCR | 0.823 | 0.714 | 0.791 | 0.776 |
| sp\|Q6W5P4\|NPSR1_HUMAN Neuropeptide S receptor OS=Homo sapiens GN=NPSR1 PE=1 SV=1 | 346 | PCREQRsQDSR | 0.165 | 0.18 | -1.18 | -0.278 |
| sp\|Q6W5P4\|NPSR1_HUMAN Neuropeptide S receptor OS=Homo sapiens GN=NPSR1 PE=1 SV=1 | 349 | EQRSQDsRMTF | 0.098 | -0.107 | -1.214 | -0.408 |
| sp\|Q6W5P4\|NPSR1_HUMAN Neuropeptide S receptor OS=Homo sapiens GN=NPSR1 PE=1 SV=1 | 352 | SQDSRMtFRER | 0.389 | 0.129 | -0.335 | 0.061 |
| sp\|Q6W5P4\|NPSR1_HUMAN Neuropeptide S receptor OS=Homo sapiens GN=NPSR1 PE=1 SV=1 | 357 | MTFRERtERHE | 0.605 | 0.787 | 0.006 | 0.466 |
| sp\|Q6W5P4\|NPSR1_HUMAN Neuropeptide S receptor OS=Homo sapiens GN=NPSR1 PE=1 SV=1 | 366 | HEMQILsKPEF | 0.407 | 0.491 | 0.145 | 0.348 |
| sp\|P48145\|NPBW1_HUMAN Neuropeptides B/W receptor type 1 OS=Homo sapiens GN=NPBWR1 PE=1 SV=2 | 313 | YAFLDAsFRRN | 0.279 | -0.103 | -0.863 | -0.229 |
| sp\|P48145\|NPBW1_HUMAN Neuropeptides B/W receptor type 1 OS=Homo sapiens GN=NPBWR1 PE=1 SV=2 | 323 | NLRQLItCRAA | 0.667 | 0.34 | 0.2 | 0.402 |
| sp\|P48146\|NPBW2_HUMAN Neuropeptides B/W receptor type 2 OS=Homo sapiens GN=NPBWR2 PE=1 SV=2 | 329 | FRKNFRsILRC | 0.104 | 0.039 | -1.284 | -0.38 |
| sp\|P25929\|NPY1R_HUMAN Neuropeptide Y receptor type 1 OS=Homo sapiens GN=NPY1R PE=1 SV=1 | 312 | HLTAMIsTCVN | 0.272 | -0.027 | -0.569 | -0.108 |
| sp\|P25929\|NPY1R_HUMAN Neuropeptide Y receptor type 1 OS=Homo sapiens GN=NPY1R PE=1 SV=1 | 313 | LTAMIStCVNP | 0.091 | -0.127 | -1.119 | -0.385 |
| sp\|P25929\|NPY1R_HUMAN Neuropeptide Y receptor type 1 OS=Homo sapiens GN=NPY1R PE=1 SV=1 | 342 | NFCDFRsRDDD | 0.361 | 0.019 | -0.731 | -0.117 |
| sp\|P25929\|NPY1R_HUMAN Neuropeptide Y receptor type 1 OS=Homo sapiens GN=NPY1R PE=1 SV=1 | 349 | RDDDYEtIAMS | 0.056 | -0.338 | -1.737 | -0.673 |
| sp\|P25929\|NPY1R_HUMAN Neuropeptide Y receptor type 1 OS=Homo sapiens GN=NPY1R PE=1 SV=1 | 353 | YETIAMsTMHT | 0.197 | 0.055 | -0.616 | -0.121 |
| sp\|P25929\|NPY1R_HUMAN Neuropeptide Y receptor type 1 OS=Homo sapiens GN=NPY1R PE=1 SV=1 | 354 | ETIAMStMHTD | 0.176 | -0.084 | -1.018 | -0.309 |
| sp\|P25929\|NPY1R_HUMAN Neuropeptide Y receptor type 1 OS=Homo sapiens GN=NPY1R PE=1 SV=1 | 357 | AMSTMHtDVSK | 0.135 | -0.01 | -1.165 | -0.347 |
| sp\|P25929\|NPY1R_HUMAN Neuropeptide Y receptor type 1 OS=Homo sapiens GN=NPY1R PE=1 SV=1 | 360 | TMHTDVsKTSL | 0.116 | -0.084 | -1.157 | -0.375 |
| sp\|P25929\|NPY1R_HUMAN Neuropeptide Y receptor type 1 OS=Homo sapiens GN=NPY1R PE=1 SV=1 | 362 | HTDVSKtSLKQ | 0.151 | 0.079 | -0.819 | -0.196 |
| sp\|P25929\|NPY1R_HUMAN Neuropeptide Y receptor type 1 OS=Homo sapiens GN=NPY1R PE=1 SV=1 | 363 | TDVSKTsLKQA | 0.1 | -0.248 | -0.875 | -0.341 |
| sp\|P25929\|NPY1R_HUMAN Neuropeptide Y receptor type 1 OS=Homo sapiens GN=NPY1R PE=1 SV=1 | 368 | TSLKQAsPVAF | 0.113 | -0.296 | -1.094 | -0.426 |
| sp\|P49146\|NPY2R_HUMAN Neuropeptide Y receptor type 2 OS=Homo sapiens GN=NPY2R PE=1 SV=1 | 317 | HIIAMCsTFAN | 0.366 | 0.139 | -0.437 | 0.023 |
| sp\|P49146\|NPY2R_HUMAN Neuropeptide Y receptor type 2 OS=Homo sapiens GN=NPY2R PE=1 SV=1 | 318 | IIAMCStFANP | 0.234 | -0.053 | -0.643 | -0.154 |
| sp\|P49146\|NPY2R_HUMAN Neuropeptide Y receptor type 2 OS=Homo sapiens GN=NPY2R PE=1 SV=1 | 330 | LYGWMNsNYRK | 0.222 | -0.208 | -0.997 | -0.328 |
| sp\|P49146\|NPY2R_HUMAN Neuropeptide Y receptor type 2 OS=Homo sapiens GN=NPY2R PE=1 SV=1 | 338 | YRKAFLsAFRC | 0.194 | 0.036 | -1.078 | -0.283 |
| sp\|P49146\|NPY2R_HUMAN Neuropeptide Y receptor type 2 OS=Homo sapiens GN=NPY2R PE=1 SV=1 | 351 | RLDAIHsEVSV | 0.309 | 0.201 | -0.639 | -0.043 |
| sp\|P49146\|NPY2R_HUMAN Neuropeptide Y receptor type 2 OS=Homo sapiens GN=NPY2R PE=1 SV=1 | 354 | AIHSEVsVTFK | 0.163 | -0.25 | -1.253 | -0.447 |

| | | | | | | |
|---|---|---|---|---|---|---|
| sp\|P49146\|NPY2R_HUMAN Neuropeptide Y receptor type 2 OS=Homo sapiens GN=NPY2R PE=1 SV=1 | 356 | HSEVSVtFKAK | 0.502 | 0.227 | 0.227 | 0.319 |
| sp\|P49146\|NPY2R_HUMAN Neuropeptide Y receptor type 2 OS=Homo sapiens GN=NPY2R PE=1 SV=1 | 369 | LEVRKNsGPND | 0.829 | 1.32 | 0.746 | 0.965 |
| sp\|P49146\|NPY2R_HUMAN Neuropeptide Y receptor type 2 OS=Homo sapiens GN=NPY2R PE=1 SV=1 | 374 | NSGPNDsFTEA | 0.229 | -0.199 | -0.785 | -0.252 |
| sp\|P49146\|NPY2R_HUMAN Neuropeptide Y receptor type 2 OS=Homo sapiens GN=NPY2R PE=1 SV=1 | 376 | GPNDSFtEATN | 0.381 | 0.237 | -0.529 | 0.03 |
| sp\|P49146\|NPY2R_HUMAN Neuropeptide Y receptor type 2 OS=Homo sapiens GN=NPY2R PE=1 SV=1 | 379 | DSFTEAtNV-- | 0.188 | -0.035 | -1.377 | -0.408 |
| sp\|P50391\|NPY4R_HUMAN Neuropeptide Y receptor type 4 OS=Homo sapiens GN=NPY4R PE=2 SV=1 | 314 | HLLAMAsTCVN | 0.291 | 0.002 | -0.601 | -0.103 |
| sp\|P50391\|NPY4R_HUMAN Neuropeptide Y receptor type 4 OS=Homo sapiens GN=NPY4R PE=2 SV=1 | 315 | LLAMAStCVNP | 0.207 | 0.07 | -0.736 | -0.153 |
| sp\|P50391\|NPY4R_HUMAN Neuropeptide Y receptor type 4 OS=Homo sapiens GN=NPY4R PE=2 SV=1 | 327 | IYGFLNtNFKK | 0.197 | -0.095 | -1.063 | -0.32 |
| sp\|P50391\|NPY4R_HUMAN Neuropeptide Y receptor type 4 OS=Homo sapiens GN=NPY4R PE=2 SV=1 | 339 | IKALVLtCQQS | 0.298 | 0.027 | -0.526 | -0.067 |
| sp\|P50391\|NPY4R_HUMAN Neuropeptide Y receptor type 4 OS=Homo sapiens GN=NPY4R PE=2 SV=1 | 343 | VLTCQQsAPLE | 0.627 | 0.546 | -0.023 | 0.383 |
| sp\|P50391\|NPY4R_HUMAN Neuropeptide Y receptor type 4 OS=Homo sapiens GN=NPY4R PE=2 SV=1 | 349 | SAPLEEsEHLP | 0.062 | -0.248 | -1.816 | -0.667 |
| sp\|P50391\|NPY4R_HUMAN Neuropeptide Y receptor type 4 OS=Homo sapiens GN=NPY4R PE=2 SV=1 | 355 | SEHLPLsTVHT | 0.024 | -0.424 | -1.925 | -0.775 |
| sp\|P50391\|NPY4R_HUMAN Neuropeptide Y receptor type 4 OS=Homo sapiens GN=NPY4R PE=2 SV=1 | 356 | EHLPLStVHTE | 0.053 | -0.266 | -1.569 | -0.594 |
| sp\|P50391\|NPY4R_HUMAN Neuropeptide Y receptor type 4 OS=Homo sapiens GN=NPY4R PE=2 SV=1 | 359 | PLSTVHtEVSK | 0.319 | 0.139 | -0.608 | -0.05 |
| sp\|P50391\|NPY4R_HUMAN Neuropeptide Y receptor type 4 OS=Homo sapiens GN=NPY4R PE=2 SV=1 | 362 | TVHTEVsKGSL | 0.133 | -0.121 | -1.043 | -0.344 |
| sp\|P50391\|NPY4R_HUMAN Neuropeptide Y receptor type 4 OS=Homo sapiens GN=NPY4R PE=2 SV=1 | 365 | TEVSKGsLRLS | 0.085 | -0.261 | -1.329 | -0.502 |
| sp\|P50391\|NPY4R_HUMAN Neuropeptide Y receptor type 4 OS=Homo sapiens GN=NPY4R PE=2 SV=1 | 369 | KGSLRLsGRSN | 0.093 | -0.155 | -1.135 | -0.399 |
| sp\|P50391\|NPY4R_HUMAN Neuropeptide Y receptor type 4 OS=Homo sapiens GN=NPY4R PE=2 SV=1 | 372 | LRLSGRsNPI- | 0.344 | 0.517 | -0.517 | 0.115 |
| sp\|Q15761\|NPY5R_HUMAN Neuropeptide Y receptor type 5 OS=Homo sapiens GN=NPY5R PE=2 SV=2 | 438 | IKADLVsLIHC | 0.409 | -0.03 | -0.249 | 0.043 |
| sp\|P41143\|OPRD_HUMAN Delta-type opioid receptor OS=Homo sapiens GN=OPRD1 PE=1 SV=4 | 343 | CGRPDPsSFSR | 0.201 | 0.007 | -0.566 | -0.119 |
| sp\|P41143\|OPRD_HUMAN Delta-type opioid receptor OS=Homo sapiens GN=OPRD1 PE=1 SV=4 | 344 | GRPDPSsFSRA | 0.123 | -0.062 | -1.145 | -0.361 |
| sp\|P41143\|OPRD_HUMAN Delta-type opioid receptor OS=Homo sapiens GN=OPRD1 PE=1 SV=4 | 346 | PDPSSFsRARE | 0.11 | -0.218 | -1.215 | -0.441 |
| sp\|P41143\|OPRD_HUMAN Delta-type opioid receptor OS=Homo sapiens GN=OPRD1 PE=1 SV=4 | 352 | SRAREAtARER | 0.744 | 1.013 | 0.286 | 0.681 |
| sp\|P41143\|OPRD_HUMAN Delta-type opioid receptor OS=Homo sapiens GN=OPRD1 PE=1 SV=4 | 358 | TARERVtACTP | 0.339 | 0.126 | -0.727 | -0.087 |
| sp\|P41143\|OPRD_HUMAN Delta-type opioid receptor OS=Homo sapiens GN=OPRD1 PE=1 SV=4 | 361 | ERVTACtPSDG | 0.189 | -0.045 | -1.025 | -0.294 |
| sp\|P41143\|OPRD_HUMAN Delta-type opioid receptor OS=Homo sapiens GN=OPRD1 PE=1 SV=4 | 363 | VTACTPsDGPG | 0.234 | -0.102 | -0.85 | -0.239 |
| sp\|P41145\|OPRK_HUMAN Kappa-type opioid receptor OS=Homo sapiens GN=OPRK1 PE=1 SV=2 | 356 | MRMERQsTSRV | 0.246 | 0.302 | -0.902 | -0.118 |
| sp\|P41145\|OPRK_HUMAN Kappa-type opioid receptor OS=Homo sapiens GN=OPRK1 PE=1 SV=2 | 357 | RMERQStSRVR | 0.375 | 0.828 | -0.152 | 0.35 |
| sp\|P41145\|OPRK_HUMAN Kappa-type opioid receptor OS=Homo sapiens GN=OPRK1 PE=1 SV=2 | 358 | MERQSTsRVRN | 0.633 | 0.361 | -0.111 | 0.294 |
| sp\|P41145\|OPRK_HUMAN Kappa-type opioid receptor OS=Homo sapiens GN=OPRK1 PE=1 SV=2 | 363 | TSRVRNtVQDP | 0.538 | 0.325 | -0.182 | 0.227 |
| sp\|P35372\|OPRM_HUMAN Mu-type opioid receptor OS=Homo sapiens GN=OPRM1 PE=1 SV=2 | 356 | REFCIPtSSNI | 0.192 | -0.076 | -1.056 | -0.313 |
| sp\|P35372\|OPRM_HUMAN Mu-type opioid receptor OS=Homo sapiens GN=OPRM1 PE=1 SV=2 | 357 | EFCIPTsSNIE | 0.16 | -0.137 | -0.739 | -0.239 |
| sp\|P35372\|OPRM_HUMAN Mu-type opioid receptor OS=Homo sapiens GN=OPRM1 PE=1 SV=2 | 358 | FCIPTSsNIEQ | 0.082 | -0.049 | -1.341 | -0.436 |
| sp\|P35372\|OPRM_HUMAN Mu-type opioid receptor OS=Homo sapiens GN=OPRM1 PE=1 SV=2 | 365 | NIEQQNsTRIR | 0.351 | 0.002 | -0.56 | -0.069 |
| sp\|P35372\|OPRM_HUMAN Mu-type opioid receptor OS=Homo sapiens GN=OPRM1 PE=1 SV=2 | 366 | IEQQNStRIRQ | 0.129 | -0.287 | -1.131 | -0.43 |
| sp\|P35372\|OPRM_HUMAN Mu-type opioid receptor OS=Homo sapiens GN=OPRM1 PE=1 SV=2 | 372 | TRIRQNtRDHP | 0.442 | 0.928 | -0.051 | 0.44 |
| sp\|P35372\|OPRM_HUMAN Mu-type opioid receptor OS=Homo sapiens GN=OPRM1 PE=1 SV=2 | 377 | NTRDHPsTANT | 0.325 | 0.108 | -0.858 | -0.142 |
| sp\|P35372\|OPRM_HUMAN Mu-type opioid receptor OS=Homo sapiens GN=OPRM1 PE=1 SV=2 | 378 | TRDHPStANTV | 0.113 | -0.01 | -1.14 | -0.346 |
| sp\|P35372\|OPRM_HUMAN Mu-type opioid receptor OS=Homo sapiens GN=OPRM1 PE=1 SV=2 | 381 | HPSTANtVDRT | 0.295 | -0.011 | -0.693 | -0.136 |
| sp\|P35372\|OPRM_HUMAN Mu-type opioid receptor OS=Homo sapiens GN=OPRM1 PE=1 SV=2 | 385 | ANTVDRtNHQL | 0.08 | -0.238 | -1.41 | -0.523 |
| sp\|P35372\|OPRM_HUMAN Mu-type opioid receptor OS=Homo sapiens GN=OPRM1 PE=1 SV=2 | 396 | ENLEAEtAPLP | 0.282 | 0.355 | -0.533 | 0.035 |
| sp\|P41146\|OPRX_HUMAN Nociceptin receptor OS=Homo sapiens GN=OPRL1 PE=1 SV=1 | 337 | RKFCCAsALRR | 0.614 | 0.105 | -0.566 | 0.051 |
| sp\|P41146\|OPRX_HUMAN Nociceptin receptor OS=Homo sapiens GN=OPRL1 PE=1 SV=1 | 346 | RRDVQVsDRVR | 0.29 | 0.18 | -0.787 | -0.106 |
| sp\|P41146\|OPRX_HUMAN Nociceptin receptor OS=Homo sapiens GN=OPRL1 PE=1 SV=1 | 351 | VSDRVRsIAKD | 0.315 | 0.587 | -0.376 | 0.175 |
| sp\|P41146\|OPRX_HUMAN Nociceptin receptor OS=Homo sapiens GN=OPRL1 PE=1 SV=1 | 362 | VALACKtSETV | 0.246 | -0.139 | -0.835 | -0.243 |
| sp\|P41146\|OPRX_HUMAN Nociceptin receptor OS=Homo sapiens GN=OPRL1 PE=1 SV=1 | 363 | ALACKTsETVP | 0.268 | -0.007 | -0.725 | -0.155 |
| sp\|P41146\|OPRX_HUMAN Nociceptin receptor OS=Homo sapiens GN=OPRL1 PE=1 SV=1 | 365 | ACKTSEtVPRP | 0.744 | 0.539 | -0.006 | 0.426 |
| sp\|O43613\|OX1R_HUMAN Orexin receptor type 1 OS=Homo sapiens GN=HCRTR1 PE=1 SV=2 | 362 | IIYNFLsGKFR | 0.071 | -0.183 | -1.04 | -0.384 |
| sp\|O43613\|OX1R_HUMAN Orexin receptor type 1 OS=Homo sapiens GN=HCRTR1 PE=1 SV=2 | 374 | QFKAAFsCCLP | 0.248 | 0.032 | -0.518 | -0.079 |

| | | | | | | |
|---|---|---|---|---|---|---|
| sp\|O43613\|OX1R_HUMAN Orexin receptor type 1 OS=Homo sapiens GN=HCRTR1 PE=1 SV=2 | 385 | GLGPCGsLKAP | 0.345 | 0.058 | -0.336 | 0.022 |
| sp\|O43613\|OX1R_HUMAN Orexin receptor type 1 OS=Homo sapiens GN=HCRTR1 PE=1 SV=2 | 390 | GSLKAPsPRSS | 0.143 | -0.412 | -1.129 | -0.466 |
| sp\|O43613\|OX1R_HUMAN Orexin receptor type 1 OS=Homo sapiens GN=HCRTR1 PE=1 SV=2 | 393 | KAPSPRsSASH | 0.014 | -0.605 | -2.397 | -0.996 |
| sp\|O43613\|OX1R_HUMAN Orexin receptor type 1 OS=Homo sapiens GN=HCRTR1 PE=1 SV=2 | 394 | APSPRSsASHK | 0.073 | -0.251 | -1.365 | -0.514 |
| sp\|O43613\|OX1R_HUMAN Orexin receptor type 1 OS=Homo sapiens GN=HCRTR1 PE=1 SV=2 | 396 | SPRSSAsHKSL | 0.351 | 0.292 | -0.426 | 0.072 |
| sp\|O43613\|OX1R_HUMAN Orexin receptor type 1 OS=Homo sapiens GN=HCRTR1 PE=1 SV=2 | 399 | SSASHKsLSLQ | 0.211 | -0.1 | -0.692 | -0.194 |
| sp\|O43613\|OX1R_HUMAN Orexin receptor type 1 OS=Homo sapiens GN=HCRTR1 PE=1 SV=2 | 401 | ASHKSLsLQSR | 0.343 | 0.131 | -0.269 | 0.068 |
| sp\|O43613\|OX1R_HUMAN Orexin receptor type 1 OS=Homo sapiens GN=HCRTR1 PE=1 SV=2 | 404 | KSLSLQsRCSI | 0.055 | -0.399 | -1.525 | -0.623 |
| sp\|O43613\|OX1R_HUMAN Orexin receptor type 1 OS=Homo sapiens GN=HCRTR1 PE=1 SV=2 | 407 | SLQSRCsISKI | 0.296 | 0.094 | -0.726 | -0.112 |
| sp\|O43613\|OX1R_HUMAN Orexin receptor type 1 OS=Homo sapiens GN=HCRTR1 PE=1 SV=2 | 409 | QSRCSIsKISE | 0.411 | 0.25 | -0.126 | 0.178 |
| sp\|O43613\|OX1R_HUMAN Orexin receptor type 1 OS=Homo sapiens GN=HCRTR1 PE=1 SV=2 | 412 | CSISKIsEHVV | 0.218 | -0.025 | -0.985 | -0.264 |
| sp\|O43613\|OX1R_HUMAN Orexin receptor type 1 OS=Homo sapiens GN=HCRTR1 PE=1 SV=2 | 418 | SEHVVLtSVTT | 0.025 | -0.361 | -2.063 | -0.8 |
| sp\|O43613\|OX1R_HUMAN Orexin receptor type 1 OS=Homo sapiens GN=HCRTR1 PE=1 SV=2 | 419 | EHVVLTsVTTV | 0.07 | -0.164 | -1.687 | -0.594 |
| sp\|O43613\|OX1R_HUMAN Orexin receptor type 1 OS=Homo sapiens GN=HCRTR1 PE=1 SV=2 | 421 | VVLTSVtTVLP | 0.262 | 0.07 | -0.532 | -0.067 |
| sp\|O43613\|OX1R_HUMAN Orexin receptor type 1 OS=Homo sapiens GN=HCRTR1 PE=1 SV=2 | 422 | VLTSVTtVLP- | 0.167 | -0.073 | -0.878 | -0.261 |
| sp\|O43614\|OX2R_HUMAN Orexin receptor type 2 OS=Homo sapiens GN=HCRTR2 PE=1 SV=2 | 368 | IIYNFLsGKFR | 0.071 | -0.183 | -1.04 | -0.384 |
| sp\|O43614\|OX2R_HUMAN Orexin receptor type 2 OS=Homo sapiens GN=HCRTR2 PE=1 SV=2 | 380 | EFKAAFsCCCL | 0.317 | 0.175 | -0.322 | 0.057 |
| sp\|O43614\|OX2R_HUMAN Orexin receptor type 2 OS=Homo sapiens GN=HCRTR2 PE=1 SV=2 | 395 | RQEDRLtRGRT | 0.224 | -0.073 | -0.858 | -0.236 |
| sp\|O43614\|OX2R_HUMAN Orexin receptor type 2 OS=Homo sapiens GN=HCRTR2 PE=1 SV=2 | 399 | RLTRGRtSTES | 0.593 | 0.847 | 0.027 | 0.489 |
| sp\|O43614\|OX2R_HUMAN Orexin receptor type 2 OS=Homo sapiens GN=HCRTR2 PE=1 SV=2 | 400 | LTRGRTsTESR | 0.333 | 0.266 | -0.342 | 0.086 |
| sp\|O43614\|OX2R_HUMAN Orexin receptor type 2 OS=Homo sapiens GN=HCRTR2 PE=1 SV=2 | 401 | TRGRTStESRK | 0.282 | 0.863 | -0.29 | 0.285 |
| sp\|O43614\|OX2R_HUMAN Orexin receptor type 2 OS=Homo sapiens GN=HCRTR2 PE=1 SV=2 | 403 | GRTSTEsRKSL | 0.089 | 0.046 | -1.196 | -0.354 |
| sp\|O43614\|OX2R_HUMAN Orexin receptor type 2 OS=Homo sapiens GN=HCRTR2 PE=1 SV=2 | 406 | STESRKsLTTQ | 0.509 | 0.032 | -0.339 | 0.067 |
| sp\|O43614\|OX2R_HUMAN Orexin receptor type 2 OS=Homo sapiens GN=HCRTR2 PE=1 SV=2 | 408 | ESRKSLtTQIS | 0.53 | 0.336 | -0.044 | 0.274 |
| sp\|O43614\|OX2R_HUMAN Orexin receptor type 2 OS=Homo sapiens GN=HCRTR2 PE=1 SV=2 | 409 | SRKSLTtQISN | 0.061 | -0.025 | -1.407 | -0.457 |
| sp\|O43614\|OX2R_HUMAN Orexin receptor type 2 OS=Homo sapiens GN=HCRTR2 PE=1 SV=2 | 412 | SLTTQIsNFDN | 0.262 | 0.023 | -0.87 | -0.195 |
| sp\|O43614\|OX2R_HUMAN Orexin receptor type 2 OS=Homo sapiens GN=HCRTR2 PE=1 SV=2 | 418 | SNFDNIsKLSE | 0.062 | -0.284 | -1.435 | -0.552 |
| sp\|O43614\|OX2R_HUMAN Orexin receptor type 2 OS=Homo sapiens GN=HCRTR2 PE=1 SV=2 | 421 | DNISKLsEQVV | 0.096 | -0.148 | -1.448 | -0.5 |
| sp\|O43614\|OX2R_HUMAN Orexin receptor type 2 OS=Homo sapiens GN=HCRTR2 PE=1 SV=2 | 427 | SEQVVLtSIST | 0.024 | -0.286 | -1.903 | -0.722 |
| sp\|O43614\|OX2R_HUMAN Orexin receptor type 2 OS=Homo sapiens GN=HCRTR2 PE=1 SV=2 | 428 | EQVVLTsISTL | 0.032 | -0.239 | -1.407 | -0.538 |
| sp\|O43614\|OX2R_HUMAN Orexin receptor type 2 OS=Homo sapiens GN=HCRTR2 PE=1 SV=2 | 430 | VVLTSIsTLPA | 0.19 | -0.005 | -0.645 | -0.153 |
| sp\|O43614\|OX2R_HUMAN Orexin receptor type 2 OS=Homo sapiens GN=HCRTR2 PE=1 SV=2 | 431 | VLTSIStLPAA | 0.722 | 0.551 | 0.722 | 0.665 |
| sp\|Q96P65\|QRFPR_HUMAN Pyroglutamylated RFamide peptide receptor OS=Homo sapiens GN=QRFPR PE=2 | 345 | FKKNVLsAVCY | 0.364 | 0.228 | -0.485 | 0.036 |
| sp\|Q96P65\|QRFPR_HUMAN Pyroglutamylated RFamide peptide receptor OS=Homo sapiens GN=QRFPR PE=2 | 355 | YCIVNKtFSPA | 0.225 | 0.011 | -0.527 | -0.097 |
| sp\|Q96P65\|QRFPR_HUMAN Pyroglutamylated RFamide peptide receptor OS=Homo sapiens GN=QRFPR PE=2 | 357 | IVNKTFsPAQR | 0.11 | -0.285 | -0.974 | -0.383 |
| sp\|Q96P65\|QRFPR_HUMAN Pyroglutamylated RFamide peptide receptor OS=Homo sapiens GN=QRFPR PE=2 | 365 | AQRHGNsGITM | 0.186 | 0.022 | -0.597 | -0.13 |
| sp\|Q96P65\|QRFPR_HUMAN Pyroglutamylated RFamide peptide receptor OS=Homo sapiens GN=QRFPR PE=2 | 368 | HGNSGItMMRK | 0.11 | -0.157 | -1.262 | -0.436 |
| sp\|Q96P65\|QRFPR_HUMAN Pyroglutamylated RFamide peptide receptor OS=Homo sapiens GN=QRFPR PE=2 | 377 | RKKAKFsLREN | 0.636 | 0.16 | 0.118 | 0.305 |
| sp\|Q96P65\|QRFPR_HUMAN Pyroglutamylated RFamide peptide receptor OS=Homo sapiens GN=QRFPR PE=2 | 386 | ENPVEEtKGEA | 0.083 | -0.277 | -1.316 | -0.503 |
| sp\|Q96P65\|QRFPR_HUMAN Pyroglutamylated RFamide peptide receptor OS=Homo sapiens GN=QRFPR PE=2 | 392 | TKGEAFsDGNI | 0.468 | 0.27 | -0.275 | 0.154 |
| sp\|Q96P65\|QRFPR_HUMAN Pyroglutamylated RFamide peptide receptor OS=Homo sapiens GN=QRFPR PE=2 | 404 | VKLCEQtEEKK | 0.286 | -0.039 | -0.73 | -0.161 |
| sp\|Q96P65\|QRFPR_HUMAN Pyroglutamylated RFamide peptide receptor OS=Homo sapiens GN=QRFPR PE=2 | 419 | HLALFRsELAE | 0.231 | 0.166 | -0.669 | -0.091 |
| sp\|Q96P65\|QRFPR_HUMAN Pyroglutamylated RFamide peptide receptor OS=Homo sapiens GN=QRFPR PE=2 | 425 | SELAENsPLDS | 0.067 | -0.433 | -1.795 | -0.72 |
| sp\|Q96P65\|QRFPR_HUMAN Pyroglutamylated RFamide peptide receptor OS=Homo sapiens GN=QRFPR PE=2 | 429 | ENSPLDsGH-- | 0.051 | -0.257 | -1.501 | -0.569 |
| sp\|P49683\|PRLHR_HUMAN Prolactin-releasing peptide receptor OS=Homo sapiens GN=PRLHR PE=1 SV=3 | 341 | YAWLHDsFREE | 0.263 | 0.006 | -0.808 | -0.18 |
| sp\|P49683\|PRLHR_HUMAN Prolactin-releasing peptide receptor OS=Homo sapiens GN=PRLHR PE=1 SV=3 | 365 | PHGQNMtVSVV | 0.201 | 0.075 | -0.996 | -0.24 |
| sp\|P49683\|PRLHR_HUMAN Prolactin-releasing peptide receptor OS=Homo sapiens GN=PRLHR PE=1 SV=3 | 367 | GQNMTVsVVI- | 0.248 | 0.009 | -0.729 | -0.157 |
| sp\|P25116\|PAR1_HUMAN Proteinase-activated receptor 1 OS=Homo sapiens GN=F2R PE=1 SV=2 | 375 | LIYYYAsSECQ | 0.177 | 0.064 | -0.806 | -0.188 |
| sp\|P25116\|PAR1_HUMAN Proteinase-activated receptor 1 OS=Homo sapiens GN=F2R PE=1 SV=2 | 376 | IYYYASsECQR | 0.281 | 0.01 | -0.399 | -0.036 |

| | | | | | | |
|---|---|---|---|---|---|---|
| sp\|P25116\|PAR1_HUMAN Proteinase-activated receptor 1 OS=Homo sapiens GN=F2R PE=1 SV=2 | 384 | CQRYVYsILCC | 0.242 | 0.242 | -0.41 | 0.025 |
| sp\|P25116\|PAR1_HUMAN Proteinase-activated receptor 1 OS=Homo sapiens GN=F2R PE=1 SV=2 | 391 | ILCCKEsSDPS | 0.226 | -0.127 | -0.998 | -0.3 |
| sp\|P25116\|PAR1_HUMAN Proteinase-activated receptor 1 OS=Homo sapiens GN=F2R PE=1 SV=2 | 392 | LCCKESsDPSS | 0.487 | 0.421 | -0.488 | 0.14 |
| sp\|P25116\|PAR1_HUMAN Proteinase-activated receptor 1 OS=Homo sapiens GN=F2R PE=1 SV=2 | 395 | KESSDPsSYNS | 0.065 | -0.307 | -1.743 | -0.662 |
| sp\|P25116\|PAR1_HUMAN Proteinase-activated receptor 1 OS=Homo sapiens GN=F2R PE=1 SV=2 | 396 | ESSDPSsYNSS | 0.029 | -0.384 | -1.563 | -0.639 |
| sp\|P25116\|PAR1_HUMAN Proteinase-activated receptor 1 OS=Homo sapiens GN=F2R PE=1 SV=2 | 399 | DPSSYNsSGQL | 0.296 | -0.086 | -0.848 | -0.213 |
| sp\|P25116\|PAR1_HUMAN Proteinase-activated receptor 1 OS=Homo sapiens GN=F2R PE=1 SV=2 | 400 | PSSYNSsGQLM | 0.127 | -0.179 | -0.718 | -0.257 |
| sp\|P25116\|PAR1_HUMAN Proteinase-activated receptor 1 OS=Homo sapiens GN=F2R PE=1 SV=2 | 406 | SGQLMAsKMDT | 0.237 | -0.072 | -0.734 | -0.19 |
| sp\|P25116\|PAR1_HUMAN Proteinase-activated receptor 1 OS=Homo sapiens GN=F2R PE=1 SV=2 | 410 | MASKMDtCSSN | 0.162 | -0.125 | -1.157 | -0.373 |
| sp\|P25116\|PAR1_HUMAN Proteinase-activated receptor 1 OS=Homo sapiens GN=F2R PE=1 SV=2 | 412 | SKMDTCsSNLN | 0.24 | 0.105 | -0.915 | -0.19 |
| sp\|P25116\|PAR1_HUMAN Proteinase-activated receptor 1 OS=Homo sapiens GN=F2R PE=1 SV=2 | 413 | KMDTCSsNLNN | 0.078 | -0.074 | -1.69 | -0.562 |
| sp\|P25116\|PAR1_HUMAN Proteinase-activated receptor 1 OS=Homo sapiens GN=F2R PE=1 SV=2 | 418 | SSNLNNsIYKK | 0.096 | -0.238 | -1.367 | -0.503 |
| sp\|P25116\|PAR1_HUMAN Proteinase-activated receptor 1 OS=Homo sapiens GN=F2R PE=1 SV=2 | 425 | IYKKLLt---- | 0.33 | 0.146 | -0.523 | -0.016 |
| sp\|P55085\|PAR2_HUMAN Proteinase-activated receptor 2 OS=Homo sapiens GN=F2RL1 PE=1 SV=1 | 348 | FVYYFVsHDFR | 0.307 | 0.055 | -0.454 | -0.031 |
| sp\|P55085\|PAR2_HUMAN Proteinase-activated receptor 2 OS=Homo sapiens GN=F2RL1 PE=1 SV=1 | 363 | NALLCRsVRTV | 0.209 | -0.139 | -1.116 | -0.349 |
| sp\|P55085\|PAR2_HUMAN Proteinase-activated receptor 2 OS=Homo sapiens GN=F2RL1 PE=1 SV=1 | 366 | LCRSVRtVKQM | 0.319 | 0.129 | -0.613 | -0.055 |
| sp\|P55085\|PAR2_HUMAN Proteinase-activated receptor 2 OS=Homo sapiens GN=F2RL1 PE=1 SV=1 | 373 | VKQMQVsLTSK | 0.469 | 0.002 | -0.333 | 0.046 |
| sp\|P55085\|PAR2_HUMAN Proteinase-activated receptor 2 OS=Homo sapiens GN=F2RL1 PE=1 SV=1 | 375 | QMQVSLtSKKH | 0.054 | 0.043 | -1.286 | -0.396 |
| sp\|P55085\|PAR2_HUMAN Proteinase-activated receptor 2 OS=Homo sapiens GN=F2RL1 PE=1 SV=1 | 376 | MQVSLTsKKHS | 0.064 | -0.193 | -1.057 | -0.395 |
| sp\|P55085\|PAR2_HUMAN Proteinase-activated receptor 2 OS=Homo sapiens GN=F2RL1 PE=1 SV=1 | 380 | LTSKKHsRKSS | 0.122 | -0.145 | -1.149 | -0.391 |
| sp\|P55085\|PAR2_HUMAN Proteinase-activated receptor 2 OS=Homo sapiens GN=F2RL1 PE=1 SV=1 | 383 | KKHSRKsSSYS | 0.204 | -0.041 | -0.908 | -0.248 |
| sp\|P55085\|PAR2_HUMAN Proteinase-activated receptor 2 OS=Homo sapiens GN=F2RL1 PE=1 SV=1 | 384 | KHSRKSsSYSS | 0.115 | 0.535 | -1.008 | -0.119 |
| sp\|P55085\|PAR2_HUMAN Proteinase-activated receptor 2 OS=Homo sapiens GN=F2RL1 PE=1 SV=1 | 385 | HSRKSSsYSSS | 0.223 | 0.234 | -0.492 | -0.012 |
| sp\|P55085\|PAR2_HUMAN Proteinase-activated receptor 2 OS=Homo sapiens GN=F2RL1 PE=1 SV=1 | 387 | RKSSSYsSSST | 0.189 | 0.092 | -0.572 | -0.097 |
| sp\|P55085\|PAR2_HUMAN Proteinase-activated receptor 2 OS=Homo sapiens GN=F2RL1 PE=1 SV=1 | 388 | KSSSYSsSSTT | 0.037 | -0.462 | -1.698 | -0.708 |
| sp\|P55085\|PAR2_HUMAN Proteinase-activated receptor 2 OS=Homo sapiens GN=F2RL1 PE=1 SV=1 | 389 | SSSYSSsSTTV | 0.152 | -0.084 | -1.093 | -0.342 |
| sp\|P55085\|PAR2_HUMAN Proteinase-activated receptor 2 OS=Homo sapiens GN=F2RL1 PE=1 SV=1 | 390 | SSYSSSsTTVK | 0.116 | -0.126 | -1.108 | -0.373 |
| sp\|P55085\|PAR2_HUMAN Proteinase-activated receptor 2 OS=Homo sapiens GN=F2RL1 PE=1 SV=1 | 391 | SYSSSStTVKT | 0.104 | -0.088 | -1.153 | -0.379 |
| sp\|P55085\|PAR2_HUMAN Proteinase-activated receptor 2 OS=Homo sapiens GN=F2RL1 PE=1 SV=1 | 392 | YSSSSTtVKTS | 0.185 | -0.018 | -0.562 | -0.132 |
| sp\|P55085\|PAR2_HUMAN Proteinase-activated receptor 2 OS=Homo sapiens GN=F2RL1 PE=1 SV=1 | 395 | SSTTVKtSY-- | 0.149 | -0.017 | -1.006 | -0.291 |
| sp\|P55085\|PAR2_HUMAN Proteinase-activated receptor 2 OS=Homo sapiens GN=F2RL1 PE=1 SV=1 | 396 | STTVKTsY--- | 0.058 | -0.089 | -1.406 | -0.479 |
| sp\|Q96RI0\|PAR4_HUMAN Proteinase-activated receptor 4 OS=Homo sapiens GN=F2RL3 PE=1 SV=3 | 344 | FIYYYVsAEFR | 0.316 | 0.143 | -0.495 | -0.012 |
| sp\|Q96RI0\|PAR4_HUMAN Proteinase-activated receptor 4 OS=Homo sapiens GN=F2RL3 PE=1 SV=3 | 359 | AGLFQRsPGDT | 0.108 | -0.328 | -1.266 | -0.495 |
| sp\|Q96RI0\|PAR4_HUMAN Proteinase-activated receptor 4 OS=Homo sapiens GN=F2RL3 PE=1 SV=3 | 363 | QRSPGDtVASK | 0.047 | -0.206 | -1.472 | -0.544 |
| sp\|Q96RI0\|PAR4_HUMAN Proteinase-activated receptor 4 OS=Homo sapiens GN=F2RL3 PE=1 SV=3 | 366 | PGDTVAsKASA | 0.137 | -0.193 | -1.055 | -0.37 |
| sp\|Q96RI0\|PAR4_HUMAN Proteinase-activated receptor 4 OS=Homo sapiens GN=F2RL3 PE=1 SV=3 | 369 | TVASKAsAEGG | 0.122 | -0.151 | -1.073 | -0.367 |
| sp\|Q96RI0\|PAR4_HUMAN Proteinase-activated receptor 4 OS=Homo sapiens GN=F2RL3 PE=1 SV=3 | 374 | ASAEGGsRGMG | 0.061 | -0.404 | -1.616 | -0.653 |
| sp\|Q96RI0\|PAR4_HUMAN Proteinase-activated receptor 4 OS=Homo sapiens GN=F2RL3 PE=1 SV=3 | 379 | GSRGMGtHSSL | 0.16 | -0.008 | -0.897 | -0.248 |
| sp\|Q96RI0\|PAR4_HUMAN Proteinase-activated receptor 4 OS=Homo sapiens GN=F2RL3 PE=1 SV=3 | 381 | RGMGTHsSLLQ | 0.11 | 0.081 | -1.028 | -0.279 |
| sp\|Q96RI0\|PAR4_HUMAN Proteinase-activated receptor 4 OS=Homo sapiens GN=F2RL3 PE=1 SV=3 | 382 | GMGTHSsLLQ- | 0.185 | 0.121 | -0.88 | -0.191 |
| sp\|Q9HBX9\|RXFP1_HUMAN Relaxin receptor 1 OS=Homo sapiens GN=RXFP1 PE=1 SV=2 | 685 | ILYTLTtRPFK | 0.42 | 0.274 | -0.31 | 0.128 |
| sp\|Q9HBX9\|RXFP1_HUMAN Relaxin receptor 1 OS=Homo sapiens GN=RXFP1 PE=1 SV=2 | 704 | NYRQRKsMDSK | 0.719 | 0.539 | 0.381 | 0.546 |
| sp\|Q9HBX9\|RXFP1_HUMAN Relaxin receptor 1 OS=Homo sapiens GN=RXFP1 PE=1 SV=2 | 707 | QRKSMDsKGQK | 0.313 | -0.022 | -0.468 | -0.059 |
| sp\|Q9HBX9\|RXFP1_HUMAN Relaxin receptor 1 OS=Homo sapiens GN=RXFP1 PE=1 SV=2 | 712 | DSKGQKtYAPS | 0.103 | -0.216 | -1.074 | -0.396 |
| sp\|Q9HBX9\|RXFP1_HUMAN Relaxin receptor 1 OS=Homo sapiens GN=RXFP1 PE=1 SV=2 | 716 | QKTYAPsFIWV | 0.464 | 0.174 | -0.02 | 0.206 |
| sp\|Q9HBX9\|RXFP1_HUMAN Relaxin receptor 1 OS=Homo sapiens GN=RXFP1 PE=1 SV=2 | 739 | MKPDLFtYPCE | 0.531 | 0.534 | -0.155 | 0.303 |
| sp\|Q9HBX9\|RXFP1_HUMAN Relaxin receptor 1 OS=Homo sapiens GN=RXFP1 PE=1 SV=2 | 745 | TYPCEMsLISQ | 0.199 | 0.007 | -0.834 | -0.209 |
| sp\|Q9HBX9\|RXFP1_HUMAN Relaxin receptor 1 OS=Homo sapiens GN=RXFP1 PE=1 SV=2 | 748 | CEMSLIsQSTR | 0.088 | -0.231 | -1.409 | -0.517 |
| sp\|Q9HBX9\|RXFP1_HUMAN Relaxin receptor 1 OS=Homo sapiens GN=RXFP1 PE=1 SV=2 | 750 | MSLISQsTRLN | 0.391 | 0.193 | -0.24 | 0.115 |

| | | | | | | |
|---|---|---|---|---|---|---|
| sp\|Q9HBX9\|RXFP1_HUMAN Relaxin receptor 1 OS=Homo sapiens GN=RXFP1 PE=1 SV=2 | 751 | SLISQStRLNS | 0.043 | -0.22 | -1.99 | -0.722 |
| sp\|Q9HBX9\|RXFP1_HUMAN Relaxin receptor 1 OS=Homo sapiens GN=RXFP1 PE=1 SV=2 | 755 | QSTRLNsYS-- | 0.357 | 0.792 | -0.223 | 0.309 |
| sp\|Q9HBX9\|RXFP1_HUMAN Relaxin receptor 1 OS=Homo sapiens GN=RXFP1 PE=1 SV=2 | 757 | TRLNSYs---- | 0.375 | 0.587 | 0.076 | 0.346 |
| sp\|Q8WXD0\|RXFP2_HUMAN Relaxin receptor 2 OS=Homo sapiens GN=RXFP2 PE=1 SV=1 | 695 | ILYTLTtNFFK | 0.148 | -0.17 | -1.02 | -0.347 |
| sp\|Q8WXD0\|RXFP2_HUMAN Relaxin receptor 2 OS=Homo sapiens GN=RXFP2 PE=1 SV=1 | 713 | HKHQRKsIFKI | 0.563 | 0.286 | 0.357 | 0.402 |
| sp\|Q8WXD0\|RXFP2_HUMAN Relaxin receptor 2 OS=Homo sapiens GN=RXFP2 PE=1 SV=1 | 721 | FKIKKKsLSTS | 0.542 | 0.199 | -0.025 | 0.239 |
| sp\|Q8WXD0\|RXFP2_HUMAN Relaxin receptor 2 OS=Homo sapiens GN=RXFP2 PE=1 SV=1 | 723 | IKKKSLsTSIV | 0.625 | 0.225 | -0.075 | 0.258 |
| sp\|Q8WXD0\|RXFP2_HUMAN Relaxin receptor 2 OS=Homo sapiens GN=RXFP2 PE=1 SV=1 | 724 | KKKSLStSIVW | 0.098 | -0.131 | -1.104 | -0.379 |
| sp\|Q8WXD0\|RXFP2_HUMAN Relaxin receptor 2 OS=Homo sapiens GN=RXFP2 PE=1 SV=1 | 725 | KKSLSTsIVWI | 0.462 | 0.23 | 0.041 | 0.244 |
| sp\|Q8WXD0\|RXFP2_HUMAN Relaxin receptor 2 OS=Homo sapiens GN=RXFP2 PE=1 SV=1 | 732 | IVWIEDsSSLK | 0.062 | -0.171 | -1.502 | -0.537 |
| sp\|Q8WXD0\|RXFP2_HUMAN Relaxin receptor 2 OS=Homo sapiens GN=RXFP2 PE=1 SV=1 | 733 | VWIEDSsSLKL | 0.037 | -0.204 | -1.751 | -0.639 |
| sp\|Q8WXD0\|RXFP2_HUMAN Relaxin receptor 2 OS=Homo sapiens GN=RXFP2 PE=1 SV=1 | 734 | WIEDSSsLKLG | 0.459 | 0.253 | 0.023 | 0.245 |
| sp\|Q8WXD0\|RXFP2_HUMAN Relaxin receptor 2 OS=Homo sapiens GN=RXFP2 PE=1 SV=1 | 744 | GVLNKItLGDS | 0.414 | 0.129 | -0.102 | 0.147 |
| sp\|Q8WXD0\|RXFP2_HUMAN Relaxin receptor 2 OS=Homo sapiens GN=RXFP2 PE=1 SV=1 | 748 | KITLGDsIMKP | 0.054 | -0.269 | -1.543 | -0.586 |
| sp\|Q8WXD0\|RXFP2_HUMAN Relaxin receptor 2 OS=Homo sapiens GN=RXFP2 PE=1 SV=1 | 754 | SIMKPVs---- | 0.315 | 0.143 | -0.554 | -0.032 |
| sp\|Q9NSD7\|RL3R1_HUMAN Relaxin-3 receptor 1 OS=Homo sapiens GN=RXFP3 PE=1 SV=1 | 402 | FRKALKsLLWR | 0.487 | 0.386 | 0.143 | 0.339 |
| sp\|Q9NSD7\|RL3R1_HUMAN Relaxin-3 receptor 1 OS=Homo sapiens GN=RXFP3 PE=1 SV=1 | 409 | LLWRIAsPSIT | 0.417 | 0.675 | 0.083 | 0.392 |
| sp\|Q9NSD7\|RL3R1_HUMAN Relaxin-3 receptor 1 OS=Homo sapiens GN=RXFP3 PE=1 SV=1 | 411 | WRIASPsITSM | 0.224 | 0.185 | -0.427 | -0.006 |
| sp\|Q9NSD7\|RL3R1_HUMAN Relaxin-3 receptor 1 OS=Homo sapiens GN=RXFP3 PE=1 SV=1 | 413 | IASPSItSMRP | 0.219 | -0.134 | -0.81 | -0.242 |
| sp\|Q9NSD7\|RL3R1_HUMAN Relaxin-3 receptor 1 OS=Homo sapiens GN=RXFP3 PE=1 SV=1 | 414 | ASPSITsMRPF | 0.065 | -0.289 | -1.268 | -0.497 |
| sp\|Q9NSD7\|RL3R1_HUMAN Relaxin-3 receptor 1 OS=Homo sapiens GN=RXFP3 PE=1 SV=1 | 419 | TSMRPFtATTK | 0.524 | 0.7 | 0.115 | 0.446 |
| sp\|Q9NSD7\|RL3R1_HUMAN Relaxin-3 receptor 1 OS=Homo sapiens GN=RXFP3 PE=1 SV=1 | 421 | MRPFTAtTKPE | 0.171 | 0.17 | -0.826 | -0.162 |
| sp\|Q9NSD7\|RL3R1_HUMAN Relaxin-3 receptor 1 OS=Homo sapiens GN=RXFP3 PE=1 SV=1 | 422 | RPFTATtKPEH | 0.638 | 0.43 | -0.107 | 0.32 |
| sp\|Q9NSD7\|RL3R1_HUMAN Relaxin-3 receptor 1 OS=Homo sapiens GN=RXFP3 PE=1 SV=1 | 456 | PGVVVYsGGRY | 0.221 | -0.029 | -0.765 | -0.191 |
| sp\|Q9NSD7\|RL3R1_HUMAN Relaxin-3 receptor 1 OS=Homo sapiens GN=RXFP3 PE=1 SV=1 | 465 | RYDLLPsSSAY | 0.154 | -0.088 | -1.205 | -0.38 |
| sp\|Q9NSD7\|RL3R1_HUMAN Relaxin-3 receptor 1 OS=Homo sapiens GN=RXFP3 PE=1 SV=1 | 466 | YDLLPSsSAY- | 0.035 | -0.385 | -1.67 | -0.673 |
| sp\|Q9NSD7\|RL3R1_HUMAN Relaxin-3 receptor 1 OS=Homo sapiens GN=RXFP3 PE=1 SV=1 | 467 | DLLPSSsAY-- | 0.377 | 0.323 | -0.44 | 0.087 |
| sp\|Q8TDU9\|RL3R2_HUMAN Relaxin-3 receptor 2 OS=Homo sapiens GN=RXFP4 PE=1 SV=1 | 323 | RQALAGtFRDL | 0.409 | 0.101 | -0.206 | 0.101 |
| sp\|Q8TDU9\|RL3R2_HUMAN Relaxin-3 receptor 2 OS=Homo sapiens GN=RXFP4 PE=1 SV=1 | 354 | GRRWVAsNPRE | 0.892 | 0.831 | 0.388 | 0.704 |
| sp\|Q8TDU9\|RL3R2_HUMAN Relaxin-3 receptor 2 OS=Homo sapiens GN=RXFP4 PE=1 SV=1 | 359 | ASNPREsRPST | 0.283 | 0.212 | -0.744 | -0.083 |
| sp\|Q8TDU9\|RL3R2_HUMAN Relaxin-3 receptor 2 OS=Homo sapiens GN=RXFP4 PE=1 SV=1 | 362 | PRESRPsTLLT | 0.224 | 0.145 | -0.694 | -0.108 |
| sp\|Q8TDU9\|RL3R2_HUMAN Relaxin-3 receptor 2 OS=Homo sapiens GN=RXFP4 PE=1 SV=1 | 363 | RESRPStLLTN | 0.515 | 0.737 | 0.057 | 0.436 |
| sp\|Q8TDU9\|RL3R2_HUMAN Relaxin-3 receptor 2 OS=Homo sapiens GN=RXFP4 PE=1 SV=1 | 366 | RPSTLLtNLDR | 0.088 | -0.184 | -1.476 | -0.524 |
| sp\|Q8TDU9\|RL3R2_HUMAN Relaxin-3 receptor 2 OS=Homo sapiens GN=RXFP4 PE=1 SV=1 | 372 | TNLDRGtPG-- | 0.112 | -0.187 | -1.185 | -0.42 |
| sp\|P30872\|SSR1_HUMAN Somatostatin receptor type 1 OS=Homo sapiens GN=SSTR1 PE=1 SV=1 | 316 | ILGYANsCANP | 0.362 | 0.196 | -0.539 | 0.006 |
| sp\|P30872\|SSR1_HUMAN Somatostatin receptor type 1 OS=Homo sapiens GN=SSTR1 PE=1 SV=1 | 327 | ILYGFLsDNFK | 0.183 | -0.076 | -0.797 | -0.23 |
| sp\|P30872\|SSR1_HUMAN Somatostatin receptor type 1 OS=Homo sapiens GN=SSTR1 PE=1 SV=1 | 333 | SDNFKRsFQRI | 0.107 | -0.177 | -1.318 | -0.463 |
| sp\|P30872\|SSR1_HUMAN Somatostatin receptor type 1 OS=Homo sapiens GN=SSTR1 PE=1 SV=1 | 341 | QRILCLsWMDN | 0.304 | 0.346 | -0.582 | 0.023 |
| sp\|P30872\|SSR1_HUMAN Somatostatin receptor type 1 OS=Homo sapiens GN=SSTR1 PE=1 SV=1 | 356 | PVDYYAtALKS | 0.136 | 0 | -1.103 | -0.322 |
| sp\|P30872\|SSR1_HUMAN Somatostatin receptor type 1 OS=Homo sapiens GN=SSTR1 PE=1 SV=1 | 360 | YATALKsRAYS | 0.089 | -0.291 | -1.236 | -0.479 |
| sp\|P30872\|SSR1_HUMAN Somatostatin receptor type 1 OS=Homo sapiens GN=SSTR1 PE=1 SV=1 | 364 | LKSRAYsVEDF | 0.798 | 1.085 | 1.061 | 0.981 |
| sp\|P30872\|SSR1_HUMAN Somatostatin receptor type 1 OS=Homo sapiens GN=SSTR1 PE=1 SV=1 | 375 | QPENLEsGGVF | 0.07 | -0.216 | -1.218 | -0.455 |
| sp\|P30872\|SSR1_HUMAN Somatostatin receptor type 1 OS=Homo sapiens GN=SSTR1 PE=1 SV=1 | 383 | GVFRNGtCTSR | 0.539 | 0.851 | 0.06 | 0.483 |
| sp\|P30872\|SSR1_HUMAN Somatostatin receptor type 1 OS=Homo sapiens GN=SSTR1 PE=1 SV=1 | 385 | FRNGTCtSRIT | 0.191 | 0.143 | -0.919 | -0.195 |
| sp\|P30872\|SSR1_HUMAN Somatostatin receptor type 1 OS=Homo sapiens GN=SSTR1 PE=1 SV=1 | 386 | RNGTCTsRITT | 0.104 | -0.262 | -1.059 | -0.406 |
| sp\|P30872\|SSR1_HUMAN Somatostatin receptor type 1 OS=Homo sapiens GN=SSTR1 PE=1 SV=1 | 389 | TCTSRItTL-- | 0.262 | 0.162 | -0.803 | -0.126 |
| sp\|P30872\|SSR1_HUMAN Somatostatin receptor type 1 OS=Homo sapiens GN=SSTR1 PE=1 SV=1 | 390 | CTSRITtL--- | 0.812 | 1.079 | 0.958 | 0.95 |
| sp\|P30874\|SSR2_HUMAN Somatostatin receptor type 2 OS=Homo sapiens GN=SSTR2 PE=1 SV=1 | 316 | ILYAFLsDNFK | 0.216 | -0.105 | -0.801 | -0.23 |
| sp\|P30874\|SSR2_HUMAN Somatostatin receptor type 2 OS=Homo sapiens GN=SSTR2 PE=1 SV=1 | 322 | SDNFKKsFQNV | 0.163 | -0.037 | -1.156 | -0.343 |

| | | | | | | |
|---|---|---|---|---|---|---|
| sp\|P30874\|SSR2_HUMAN Somatostatin receptor type 2 OS=Homo sapiens GN=SSTR2 PE=1 SV=1 | 333 | LCLVKVsGTDD | 0.338 | -0.048 | -0.573 | -0.094 |
| sp\|P30874\|SSR2_HUMAN Somatostatin receptor type 2 OS=Homo sapiens GN=SSTR2 PE=1 SV=1 | 335 | LVKVSGtDDGE | 0.268 | 0.104 | -0.443 | -0.024 |
| sp\|P30874\|SSR2_HUMAN Somatostatin receptor type 2 OS=Homo sapiens GN=SSTR2 PE=1 SV=1 | 341 | TDDGERsDSKQ | 0.029 | -0.362 | -2.073 | -0.802 |
| sp\|P30874\|SSR2_HUMAN Somatostatin receptor type 2 OS=Homo sapiens GN=SSTR2 PE=1 SV=1 | 343 | DGERSDsKQDK | 0.871 | 0.956 | 0.905 | 0.911 |
| sp\|P30874\|SSR2_HUMAN Somatostatin receptor type 2 OS=Homo sapiens GN=SSTR2 PE=1 SV=1 | 348 | DSKQDKsRLNE | 0.247 | -0.155 | -0.884 | -0.264 |
| sp\|P30874\|SSR2_HUMAN Somatostatin receptor type 2 OS=Homo sapiens GN=SSTR2 PE=1 SV=1 | 353 | KSRLNEtTETQ | 0.215 | -0.038 | -1 | -0.274 |
| sp\|P30874\|SSR2_HUMAN Somatostatin receptor type 2 OS=Homo sapiens GN=SSTR2 PE=1 SV=1 | 354 | SRLNETtETQR | 0.23 | 0.127 | -0.621 | -0.088 |
| sp\|P30874\|SSR2_HUMAN Somatostatin receptor type 2 OS=Homo sapiens GN=SSTR2 PE=1 SV=1 | 356 | LNETTEtQRTL | 0.075 | -0.287 | -1.36 | -0.524 |
| sp\|P30874\|SSR2_HUMAN Somatostatin receptor type 2 OS=Homo sapiens GN=SSTR2 PE=1 SV=1 | 359 | TTETQRtLLNG | 0.156 | -0.043 | -1.248 | -0.378 |
| sp\|P30874\|SSR2_HUMAN Somatostatin receptor type 2 OS=Homo sapiens GN=SSTR2 PE=1 SV=1 | 367 | LNGDLQtSI-- | 0.051 | -0.092 | -1.343 | -0.461 |
| sp\|P30874\|SSR2_HUMAN Somatostatin receptor type 2 OS=Homo sapiens GN=SSTR2 PE=1 SV=1 | 368 | NGDLQTsI--- | 0.104 | 0.007 | -1.093 | -0.327 |
| sp\|P32745\|SSR3_HUMAN Somatostatin receptor type 3 OS=Homo sapiens GN=SSTR3 PE=1 SV=1 | 317 | ILYGFLsYRFK | 0.099 | -0.249 | -1.068 | -0.406 |
| sp\|P32745\|SSR3_HUMAN Somatostatin receptor type 3 OS=Homo sapiens GN=SSTR3 PE=1 SV=1 | 332 | RVLLRPsRRVR | 0.266 | -0.009 | -0.705 | -0.149 |
| sp\|P32745\|SSR3_HUMAN Somatostatin receptor type 3 OS=Homo sapiens GN=SSTR3 PE=1 SV=1 | 337 | PSRRVRsQEPT | 0.583 | 0.86 | -0.032 | 0.47 |
| sp\|P32745\|SSR3_HUMAN Somatostatin receptor type 3 OS=Homo sapiens GN=SSTR3 PE=1 SV=1 | 341 | VRSQEPtVGPP | 0.175 | -0.034 | -0.807 | -0.222 |
| sp\|P32745\|SSR3_HUMAN Somatostatin receptor type 3 OS=Homo sapiens GN=SSTR3 PE=1 SV=1 | 348 | VGPPEKtEEED | 0.215 | -0.071 | -0.657 | -0.171 |
| sp\|P32745\|SSR3_HUMAN Somatostatin receptor type 3 OS=Homo sapiens GN=SSTR3 PE=1 SV=1 | 361 | EEDGEEsREGG | 0.058 | -0.34 | -1.659 | -0.647 |
| sp\|P32745\|SSR3_HUMAN Somatostatin receptor type 3 OS=Homo sapiens GN=SSTR3 PE=1 SV=1 | 375 | EMNGRVsQITQ | 0.168 | 0.065 | -0.926 | -0.231 |
| sp\|P32745\|SSR3_HUMAN Somatostatin receptor type 3 OS=Homo sapiens GN=SSTR3 PE=1 SV=1 | 378 | GRVSQItQPGT | 0.461 | 0.502 | -0.349 | 0.205 |
| sp\|P32745\|SSR3_HUMAN Somatostatin receptor type 3 OS=Homo sapiens GN=SSTR3 PE=1 SV=1 | 382 | QITQPGtSGQE | 0.16 | -0.181 | -0.731 | -0.251 |
| sp\|P32745\|SSR3_HUMAN Somatostatin receptor type 3 OS=Homo sapiens GN=SSTR3 PE=1 SV=1 | 383 | ITQPGTsGQER | 0.191 | -0.14 | -0.723 | -0.224 |
| sp\|P32745\|SSR3_HUMAN Somatostatin receptor type 3 OS=Homo sapiens GN=SSTR3 PE=1 SV=1 | 390 | GQERPPsRVAS | 0.449 | 0.664 | 0.143 | 0.419 |
| sp\|P32745\|SSR3_HUMAN Somatostatin receptor type 3 OS=Homo sapiens GN=SSTR3 PE=1 SV=1 | 394 | PPSRVAsKEQQ | 0.62 | 0.736 | 0.153 | 0.503 |
| sp\|P32745\|SSR3_HUMAN Somatostatin receptor type 3 OS=Homo sapiens GN=SSTR3 PE=1 SV=1 | 405 | LLPQEAsTGEK | 0.398 | -0.003 | -0.509 | -0.038 |
| sp\|P32745\|SSR3_HUMAN Somatostatin receptor type 3 OS=Homo sapiens GN=SSTR3 PE=1 SV=1 | 406 | LPQEAStGEKS | 0.059 | -0.167 | -1.43 | -0.513 |
| sp\|P32745\|SSR3_HUMAN Somatostatin receptor type 3 OS=Homo sapiens GN=SSTR3 PE=1 SV=1 | 410 | ASTGEKsSTMR | 0.185 | -0.216 | -0.925 | -0.319 |
| sp\|P32745\|SSR3_HUMAN Somatostatin receptor type 3 OS=Homo sapiens GN=SSTR3 PE=1 SV=1 | 411 | STGEKSsTMRI | 0.047 | -0.322 | -1.802 | -0.692 |
| sp\|P32745\|SSR3_HUMAN Somatostatin receptor type 3 OS=Homo sapiens GN=SSTR3 PE=1 SV=1 | 412 | TGEKSStMRIS | 0.356 | 0.123 | -0.39 | 0.03 |
| sp\|P32745\|SSR3_HUMAN Somatostatin receptor type 3 OS=Homo sapiens GN=SSTR3 PE=1 SV=1 | 416 | SSTMRIsYL-- | 0.149 | 0.008 | -0.985 | -0.276 |
| sp\|P31391\|SSR4_HUMAN Somatostatin receptor type 4 OS=Homo sapiens GN=SSTR4 PE=2 SV=2 | 315 | ILYGFLsDNFR | 0.201 | -0.017 | -0.696 | -0.171 |
| sp\|P31391\|SSR4_HUMAN Somatostatin receptor type 4 OS=Homo sapiens GN=SSTR4 PE=2 SV=2 | 349 | PLDYYAtALKS | 0.261 | 0.094 | -0.816 | -0.154 |
| sp\|P31391\|SSR4_HUMAN Somatostatin receptor type 4 OS=Homo sapiens GN=SSTR4 PE=2 SV=2 | 353 | YATALKsKGGA | 0.273 | -0.11 | -0.315 | -0.051 |
| sp\|P31391\|SSR4_HUMAN Somatostatin receptor type 4 OS=Homo sapiens GN=SSTR4 PE=2 SV=2 | 383 | RKRIPLtRTTT | 0.31 | 0.041 | -0.583 | -0.077 |
| sp\|P31391\|SSR4_HUMAN Somatostatin receptor type 4 OS=Homo sapiens GN=SSTR4 PE=2 SV=2 | 385 | RIPLTRtTTF- | 0.085 | -0.044 | -1.526 | -0.495 |
| sp\|P31391\|SSR4_HUMAN Somatostatin receptor type 4 OS=Homo sapiens GN=SSTR4 PE=2 SV=2 | 386 | IPLTRTtTF-- | 0.349 | 0.171 | -0.461 | 0.02 |
| sp\|P31391\|SSR4_HUMAN Somatostatin receptor type 4 OS=Homo sapiens GN=SSTR4 PE=2 SV=2 | 387 | PLTRTTtF--- | 0.811 | 1.233 | 0.943 | 0.996 |
| sp\|P35346\|SSR5_HUMAN Somatostatin receptor type 5 OS=Homo sapiens GN=SSTR5 PE=1 SV=3 | 308 | VLYGFLsDNFR | 0.147 | -0.05 | -0.881 | -0.261 |
| sp\|P35346\|SSR5_HUMAN Somatostatin receptor type 5 OS=Homo sapiens GN=SSTR5 PE=1 SV=3 | 314 | SDNFRQsFQKV | 0.214 | 0.051 | -0.984 | -0.24 |
| sp\|P35346\|SSR5_HUMAN Somatostatin receptor type 5 OS=Homo sapiens GN=SSTR5 PE=1 SV=3 | 325 | LCLRKGsGAKD | 0.335 | 0.635 | -0.233 | 0.246 |
| sp\|P35346\|SSR5_HUMAN Somatostatin receptor type 5 OS=Homo sapiens GN=SSTR5 PE=1 SV=3 | 333 | AKDADAtEPRP | 0.66 | 0.373 | -0.155 | 0.293 |
| sp\|P35346\|SSR5_HUMAN Somatostatin receptor type 5 OS=Homo sapiens GN=SSTR5 PE=1 SV=3 | 347 | RQQQEAtPPAH | 0.439 | 0.204 | -0.33 | 0.104 |
| sp\|P35346\|SSR5_HUMAN Somatostatin receptor type 5 OS=Homo sapiens GN=SSTR5 PE=1 SV=3 | 361 | ANGLMQtSKL- | 0.07 | -0.245 | -1.299 | -0.491 |
| sp\|P35346\|SSR5_HUMAN Somatostatin receptor type 5 OS=Homo sapiens GN=SSTR5 PE=1 SV=3 | 362 | NGLMQTsKL-- | 0.116 | -0.017 | -0.953 | -0.285 |
| sp\|P25103\|NK1R_HUMAN Substance-P receptor OS=Homo sapiens GN=TACR1 PE=1 SV=1 | 327 | RCCPFIsAGDY | 0.636 | 0.174 | -0.494 | 0.105 |
| sp\|P25103\|NK1R_HUMAN Substance-P receptor OS=Homo sapiens GN=TACR1 PE=1 SV=1 | 338 | EGLEMKsTRYL | 0.136 | -0.161 | -0.724 | -0.25 |
| sp\|P25103\|NK1R_HUMAN Substance-P receptor OS=Homo sapiens GN=TACR1 PE=1 SV=1 | 339 | GLEMKStRYLQ | 0.147 | -0.188 | -1.124 | -0.388 |
| sp\|P25103\|NK1R_HUMAN Substance-P receptor OS=Homo sapiens GN=TACR1 PE=1 SV=1 | 344 | STRYLQtQGSV | 0.193 | 0.081 | -1.183 | -0.303 |
| sp\|P25103\|NK1R_HUMAN Substance-P receptor OS=Homo sapiens GN=TACR1 PE=1 SV=1 | 347 | YLQTQGsVYKV | 0.131 | 0.007 | -1.251 | -0.371 |
| sp\|P25103\|NK1R_HUMAN Substance-P receptor OS=Homo sapiens GN=TACR1 PE=1 SV=1 | 352 | GSVYKVsRLET | 0.16 | -0.213 | -1.067 | -0.373 |

| | | | | | | |
|---|---|---|---|---|---|---|
| sp\|P25103\|NK1R_HUMAN Substance-P receptor OS=Homo sapiens GN=TACR1 PE=1 SV=1 | 356 | KVSRLEtTIST | 0.149 | 0.566 | -0.606 | 0.036 |
| sp\|P25103\|NK1R_HUMAN Substance-P receptor OS=Homo sapiens GN=TACR1 PE=1 SV=1 | 357 | VSRLETtISTV | 0.164 | -0.06 | -1.155 | -0.35 |
| sp\|P25103\|NK1R_HUMAN Substance-P receptor OS=Homo sapiens GN=TACR1 PE=1 SV=1 | 359 | RLETTIsTVVG | 0.284 | 0.109 | -0.637 | -0.081 |
| sp\|P25103\|NK1R_HUMAN Substance-P receptor OS=Homo sapiens GN=TACR1 PE=1 SV=1 | 360 | LETTIStVVGA | 0.1 | -0.205 | -0.913 | -0.339 |
| sp\|P25103\|NK1R_HUMAN Substance-P receptor OS=Homo sapiens GN=TACR1 PE=1 SV=1 | 376 | EDGPKAtPSSL | 0.012 | -0.604 | -2.111 | -0.901 |
| sp\|P25103\|NK1R_HUMAN Substance-P receptor OS=Homo sapiens GN=TACR1 PE=1 SV=1 | 378 | GPKATPsSLDL | 0.179 | -0.03 | -0.866 | -0.239 |
| sp\|P25103\|NK1R_HUMAN Substance-P receptor OS=Homo sapiens GN=TACR1 PE=1 SV=1 | 379 | PKATPSsLDLT | 0.357 | -0.005 | -0.165 | 0.062 |
| sp\|P25103\|NK1R_HUMAN Substance-P receptor OS=Homo sapiens GN=TACR1 PE=1 SV=1 | 383 | PSSLDLtSNCS | 0.078 | -0.182 | -1.27 | -0.458 |
| sp\|P25103\|NK1R_HUMAN Substance-P receptor OS=Homo sapiens GN=TACR1 PE=1 SV=1 | 384 | SSLDLTsNCSS | 0.028 | -0.412 | -2.052 | -0.812 |
| sp\|P25103\|NK1R_HUMAN Substance-P receptor OS=Homo sapiens GN=TACR1 PE=1 SV=1 | 387 | DLTSNCsSRSD | 0.327 | -0.105 | -0.75 | -0.176 |
| sp\|P25103\|NK1R_HUMAN Substance-P receptor OS=Homo sapiens GN=TACR1 PE=1 SV=1 | 388 | LTSNCSsRSDS | 0.1 | -0.262 | -1.179 | -0.447 |
| sp\|P25103\|NK1R_HUMAN Substance-P receptor OS=Homo sapiens GN=TACR1 PE=1 SV=1 | 390 | SNCSSRsDSKT | 0.081 | -0.136 | -1.437 | -0.497 |
| sp\|P25103\|NK1R_HUMAN Substance-P receptor OS=Homo sapiens GN=TACR1 PE=1 SV=1 | 392 | CSSRSDsKTMT | 0.705 | 0.792 | 0.492 | 0.663 |
| sp\|P25103\|NK1R_HUMAN Substance-P receptor OS=Homo sapiens GN=TACR1 PE=1 SV=1 | 394 | SRSDSKtMTES | 0.495 | 0.476 | -0.304 | 0.222 |
| sp\|P25103\|NK1R_HUMAN Substance-P receptor OS=Homo sapiens GN=TACR1 PE=1 SV=1 | 396 | SDSKTMtESFS | 0.07 | -0.086 | -1.315 | -0.444 |
| sp\|P25103\|NK1R_HUMAN Substance-P receptor OS=Homo sapiens GN=TACR1 PE=1 SV=1 | 398 | SKTMTEsFSFS | 0.093 | -0.162 | -1.23 | -0.433 |
| sp\|P25103\|NK1R_HUMAN Substance-P receptor OS=Homo sapiens GN=TACR1 PE=1 SV=1 | 400 | TMTESFsFSSN | 0.13 | 0.269 | -0.644 | -0.082 |
| sp\|P25103\|NK1R_HUMAN Substance-P receptor OS=Homo sapiens GN=TACR1 PE=1 SV=1 | 402 | TESFSFsSNVL | 0.333 | 0.228 | -0.323 | 0.079 |
| sp\|P25103\|NK1R_HUMAN Substance-P receptor OS=Homo sapiens GN=TACR1 PE=1 SV=1 | 403 | ESFSFSsNVLS | 0.047 | -0.3 | -1.732 | -0.662 |
| sp\|P25103\|NK1R_HUMAN Substance-P receptor OS=Homo sapiens GN=TACR1 PE=1 SV=1 | 407 | FSSNVLs---- | 0.113 | 0.03 | -0.983 | -0.28 |
| sp\|P21452\|NK2R_HUMAN Substance-K receptor OS=Homo sapiens GN=TACR2 PE=1 SV=3 | 316 | LNHRFRsGFRL | 0.305 | 0.596 | -0.4 | 0.167 |
| sp\|P21452\|NK2R_HUMAN Substance-K receptor OS=Homo sapiens GN=TACR2 PE=1 SV=3 | 329 | RCCPWVtPTKE | 0.24 | -0.311 | -1.122 | -0.398 |
| sp\|P21452\|NK2R_HUMAN Substance-K receptor OS=Homo sapiens GN=TACR2 PE=1 SV=3 | 331 | CPWVTPtKEDK | 0.109 | 0.009 | -0.881 | -0.254 |
| sp\|P21452\|NK2R_HUMAN Substance-K receptor OS=Homo sapiens GN=TACR2 PE=1 SV=3 | 339 | EDKLELtPTTS | 0.031 | -0.556 | -1.842 | -0.789 |
| sp\|P21452\|NK2R_HUMAN Substance-K receptor OS=Homo sapiens GN=TACR2 PE=1 SV=3 | 341 | KLELTPtTSLS | 0.096 | -0.114 | -1.219 | -0.412 |
| sp\|P21452\|NK2R_HUMAN Substance-K receptor OS=Homo sapiens GN=TACR2 PE=1 SV=3 | 342 | LELTPTtSLST | 0.038 | -0.346 | -1.538 | -0.615 |
| sp\|P21452\|NK2R_HUMAN Substance-K receptor OS=Homo sapiens GN=TACR2 PE=1 SV=3 | 343 | ELTPTTsLSTR | 0.222 | 0.05 | -0.348 | -0.025 |
| sp\|P21452\|NK2R_HUMAN Substance-K receptor OS=Homo sapiens GN=TACR2 PE=1 SV=3 | 345 | TPTTSLsTRVN | 0.19 | 0.023 | -0.767 | -0.185 |
| sp\|P21452\|NK2R_HUMAN Substance-K receptor OS=Homo sapiens GN=TACR2 PE=1 SV=3 | 346 | PTTSLStRVNR | 0.085 | -0.241 | -1.495 | -0.55 |
| sp\|P21452\|NK2R_HUMAN Substance-K receptor OS=Homo sapiens GN=TACR2 PE=1 SV=3 | 353 | RVNRCHtKETL | 0.754 | 1.033 | 0.555 | 0.781 |
| sp\|P21452\|NK2R_HUMAN Substance-K receptor OS=Homo sapiens GN=TACR2 PE=1 SV=3 | 356 | RCHTKEtLFMA | 0.221 | -0.087 | -0.989 | -0.285 |
| sp\|P21452\|NK2R_HUMAN Substance-K receptor OS=Homo sapiens GN=TACR2 PE=1 SV=3 | 363 | LFMAGDtAPSE | 0.254 | 0.291 | -0.561 | -0.005 |
| sp\|P21452\|NK2R_HUMAN Substance-K receptor OS=Homo sapiens GN=TACR2 PE=1 SV=3 | 366 | AGDTAPsEATS | 0.15 | -0.168 | -1.063 | -0.36 |
| sp\|P21452\|NK2R_HUMAN Substance-K receptor OS=Homo sapiens GN=TACR2 PE=1 SV=3 | 369 | TAPSEAtSGEA | 0.145 | -0.236 | -1.239 | -0.443 |
| sp\|P21452\|NK2R_HUMAN Substance-K receptor OS=Homo sapiens GN=TACR2 PE=1 SV=3 | 370 | APSEATsGEAG | 0.153 | -0.08 | -0.896 | -0.274 |
| sp\|P21452\|NK2R_HUMAN Substance-K receptor OS=Homo sapiens GN=TACR2 PE=1 SV=3 | 380 | GRPQDGsGLWF | 0.085 | -0.035 | -1.154 | -0.368 |
| sp\|P21452\|NK2R_HUMAN Substance-K receptor OS=Homo sapiens GN=TACR2 PE=1 SV=3 | 392 | YGLLAPtKTHV | 0.226 | -0.117 | -0.768 | -0.22 |
| sp\|P21452\|NK2R_HUMAN Substance-K receptor OS=Homo sapiens GN=TACR2 PE=1 SV=3 | 394 | LLAPTKtHVEI | 0.431 | 0.182 | 0.082 | 0.232 |
| sp\|P29371\|NK3R_HUMAN Neuromedin-K receptor OS=Homo sapiens GN=TACR3 PE=1 SV=1 | 380 | CPFIKVsSYDE | 0.242 | -0.062 | -0.772 | -0.197 |
| sp\|P29371\|NK3R_HUMAN Neuromedin-K receptor OS=Homo sapiens GN=TACR3 PE=1 SV=1 | 381 | PFIKVSsYDEL | 0.34 | 0.076 | -0.448 | -0.011 |
| sp\|P29371\|NK3R_HUMAN Neuromedin-K receptor OS=Homo sapiens GN=TACR3 PE=1 SV=1 | 389 | DELELKtTRFH | 0.067 | -0.356 | -1.621 | -0.637 |
| sp\|P29371\|NK3R_HUMAN Neuromedin-K receptor OS=Homo sapiens GN=TACR3 PE=1 SV=1 | 390 | ELELKTtRFHP | 0.082 | -0.221 | -1.048 | -0.396 |
| sp\|P29371\|NK3R_HUMAN Neuromedin-K receptor OS=Homo sapiens GN=TACR3 PE=1 SV=1 | 398 | FHPNRQsSMYT | 0.123 | 0.009 | -1.136 | -0.335 |
| sp\|P29371\|NK3R_HUMAN Neuromedin-K receptor OS=Homo sapiens GN=TACR3 PE=1 SV=1 | 399 | HPNRQSsMYTV | 0.299 | 0.851 | -0.535 | 0.205 |
| sp\|P29371\|NK3R_HUMAN Neuromedin-K receptor OS=Homo sapiens GN=TACR3 PE=1 SV=1 | 402 | RQSSMYtVTRM | 0.189 | -0.168 | -0.75 | -0.243 |
| sp\|P29371\|NK3R_HUMAN Neuromedin-K receptor OS=Homo sapiens GN=TACR3 PE=1 SV=1 | 404 | SSMYTVtRMES | 0.127 | -0.132 | -1.116 | -0.374 |
| sp\|P29371\|NK3R_HUMAN Neuromedin-K receptor OS=Homo sapiens GN=TACR3 PE=1 SV=1 | 408 | TVTRMEsMTVV | 0.547 | 0.84 | -0.166 | 0.407 |
| sp\|P29371\|NK3R_HUMAN Neuromedin-K receptor OS=Homo sapiens GN=TACR3 PE=1 SV=1 | 410 | TRMESMtVVFD | 0.3 | 0.497 | -0.205 | 0.197 |
| sp\|P29371\|NK3R_HUMAN Neuromedin-K receptor OS=Homo sapiens GN=TACR3 PE=1 SV=1 | 420 | DPNDADtTRSS | 0.113 | -0.257 | -1.606 | -0.583 |

| | | | | | | |
|---|---|---|---|---|---|---|
| sp\|P29371\|NK3R_HUMAN Neuromedin-K receptor OS=Homo sapiens GN=TACR3 PE=1 SV=1 | 421 | PNDADTtRSSR | 0.051 | -0.393 | -1.484 | -0.609 |
| sp\|P29371\|NK3R_HUMAN Neuromedin-K receptor OS=Homo sapiens GN=TACR3 PE=1 SV=1 | 423 | DADTTRsSRKK | 0.061 | -0.315 | -1.735 | -0.663 |
| sp\|P29371\|NK3R_HUMAN Neuromedin-K receptor OS=Homo sapiens GN=TACR3 PE=1 SV=1 | 424 | ADTTRSsRKKR | 0.097 | -0.225 | -1.253 | -0.46 |
| sp\|P29371\|NK3R_HUMAN Neuromedin-K receptor OS=Homo sapiens GN=TACR3 PE=1 SV=1 | 430 | SRKKRAtPRDP | 0.435 | 0.06 | -0.448 | 0.016 |
| sp\|P29371\|NK3R_HUMAN Neuromedin-K receptor OS=Homo sapiens GN=TACR3 PE=1 SV=1 | 435 | ATPRDPsFNGC | 0.526 | 0.607 | -0.033 | 0.367 |
| sp\|P29371\|NK3R_HUMAN Neuromedin-K receptor OS=Homo sapiens GN=TACR3 PE=1 SV=1 | 440 | PSFNGCsRRNS | 0.11 | -0.248 | -1.372 | -0.503 |
| sp\|P29371\|NK3R_HUMAN Neuromedin-K receptor OS=Homo sapiens GN=TACR3 PE=1 SV=1 | 444 | GCSRRNsKSAS | 0.838 | 1.084 | 0.61 | 0.844 |
| sp\|P29371\|NK3R_HUMAN Neuromedin-K receptor OS=Homo sapiens GN=TACR3 PE=1 SV=1 | 446 | SRRNSKsASAT | 0.545 | 0.653 | 0.266 | 0.488 |
| sp\|P29371\|NK3R_HUMAN Neuromedin-K receptor OS=Homo sapiens GN=TACR3 PE=1 SV=1 | 448 | RNSKSAsATSS | 0.423 | 0.154 | -0.486 | 0.03 |
| sp\|P29371\|NK3R_HUMAN Neuromedin-K receptor OS=Homo sapiens GN=TACR3 PE=1 SV=1 | 450 | SKSASAtSSFI | 0.304 | 0.055 | -0.546 | -0.062 |
| sp\|P29371\|NK3R_HUMAN Neuromedin-K receptor OS=Homo sapiens GN=TACR3 PE=1 SV=1 | 451 | KSASATsSFIS | 0.175 | -0.175 | -0.885 | -0.295 |
| sp\|P29371\|NK3R_HUMAN Neuromedin-K receptor OS=Homo sapiens GN=TACR3 PE=1 SV=1 | 452 | SASATSsFISS | 0.039 | -0.251 | -1.533 | -0.582 |
| sp\|P29371\|NK3R_HUMAN Neuromedin-K receptor OS=Homo sapiens GN=TACR3 PE=1 SV=1 | 455 | ATSSFIsSPYT | 0.37 | 0.162 | -0.694 | -0.054 |
| sp\|P29371\|NK3R_HUMAN Neuromedin-K receptor OS=Homo sapiens GN=TACR3 PE=1 SV=1 | 456 | TSSFISsPYTS | 0.039 | -0.541 | -1.582 | -0.695 |
| sp\|P29371\|NK3R_HUMAN Neuromedin-K receptor OS=Homo sapiens GN=TACR3 PE=1 SV=1 | 459 | FISSPYtSVDE | 0.183 | 0.012 | -0.678 | -0.161 |
| sp\|P29371\|NK3R_HUMAN Neuromedin-K receptor OS=Homo sapiens GN=TACR3 PE=1 SV=1 | 460 | ISSPYTsVDEY | 0.358 | -0.058 | -0.428 | -0.043 |
| sp\|P29371\|NK3R_HUMAN Neuromedin-K receptor OS=Homo sapiens GN=TACR3 PE=1 SV=1 | 465 | TSVDEYs---- | 0.133 | 0.052 | -1.074 | -0.296 |
| sp\|P34981\|TRFR_HUMAN Thyrotropin-releasing hormone receptor OS=Homo sapiens GN=TRHR PE=1 SV=1 | 324 | VIYNLMsQKFR | 0.085 | -0.062 | -1.095 | -0.357 |
| sp\|P34981\|TRFR_HUMAN Thyrotropin-releasing hormone receptor OS=Homo sapiens GN=TRHR PE=1 SV=1 | 342 | NCKQKPtEKPA | 0.233 | -0.082 | -0.581 | -0.143 |
| sp\|P34981\|TRFR_HUMAN Thyrotropin-releasing hormone receptor OS=Homo sapiens GN=TRHR PE=1 SV=1 | 349 | EKPANYsVALN | 0.2 | 0.01 | -0.559 | -0.116 |
| sp\|P34981\|TRFR_HUMAN Thyrotropin-releasing hormone receptor OS=Homo sapiens GN=TRHR PE=1 SV=1 | 355 | SVALNYsVIKE | 0.092 | -0.066 | -0.882 | -0.285 |
| sp\|P34981\|TRFR_HUMAN Thyrotropin-releasing hormone receptor OS=Homo sapiens GN=TRHR PE=1 SV=1 | 360 | YSVIKEsDHFS | 0.064 | -0.228 | -1.605 | -0.59 |
| sp\|P34981\|TRFR_HUMAN Thyrotropin-releasing hormone receptor OS=Homo sapiens GN=TRHR PE=1 SV=1 | 364 | KESDHFsTELD | 0.217 | -0.007 | -0.95 | -0.247 |
| sp\|P34981\|TRFR_HUMAN Thyrotropin-releasing hormone receptor OS=Homo sapiens GN=TRHR PE=1 SV=1 | 365 | ESDHFStELDD | 0.118 | -0.125 | -1.05 | -0.352 |
| sp\|P34981\|TRFR_HUMAN Thyrotropin-releasing hormone receptor OS=Homo sapiens GN=TRHR PE=1 SV=1 | 371 | TELDDItVTDT | 0.25 | -0.115 | -0.869 | -0.245 |
| sp\|P34981\|TRFR_HUMAN Thyrotropin-releasing hormone receptor OS=Homo sapiens GN=TRHR PE=1 SV=1 | 373 | LDDITVtDTYL | 0.145 | -0.091 | -1.091 | -0.346 |
| sp\|P34981\|TRFR_HUMAN Thyrotropin-releasing hormone receptor OS=Homo sapiens GN=TRHR PE=1 SV=1 | 375 | DITVTDtYLSA | 0.051 | -0.192 | -1.593 | -0.578 |
| sp\|P34981\|TRFR_HUMAN Thyrotropin-releasing hormone receptor OS=Homo sapiens GN=TRHR PE=1 SV=1 | 378 | VTDTYLsATKV | 0.129 | -0.184 | -1.594 | -0.55 |
| sp\|P34981\|TRFR_HUMAN Thyrotropin-releasing hormone receptor OS=Homo sapiens GN=TRHR PE=1 SV=1 | 380 | DTYLSAtKVSF | 0.231 | 0.102 | -0.735 | -0.134 |
| sp\|P34981\|TRFR_HUMAN Thyrotropin-releasing hormone receptor OS=Homo sapiens GN=TRHR PE=1 SV=1 | 383 | LSATKVsFDDT | 0.356 | -0.045 | -0.241 | 0.023 |
| sp\|P34981\|TRFR_HUMAN Thyrotropin-releasing hormone receptor OS=Homo sapiens GN=TRHR PE=1 SV=1 | 387 | KVSFDDtCLAS | 0.109 | -0.099 | -1.293 | -0.428 |
| sp\|P34981\|TRFR_HUMAN Thyrotropin-releasing hormone receptor OS=Homo sapiens GN=TRHR PE=1 SV=1 | 391 | DDTCLAsEVSF | 0.095 | -0.147 | -1.333 | -0.462 |
| sp\|P34981\|TRFR_HUMAN Thyrotropin-releasing hormone receptor OS=Homo sapiens GN=TRHR PE=1 SV=1 | 394 | CLASEVsFSQS | 0.399 | -0.031 | -0.382 | -0.005 |
| sp\|P34981\|TRFR_HUMAN Thyrotropin-releasing hormone receptor OS=Homo sapiens GN=TRHR PE=1 SV=1 | 396 | ASEVSFsQS-- | 0.154 | 0.097 | -0.974 | -0.241 |
| sp\|P34981\|TRFR_HUMAN Thyrotropin-releasing hormone receptor OS=Homo sapiens GN=TRHR PE=1 SV=1 | 398 | EVSFSQs---- | 0.281 | 0.322 | -0.346 | 0.086 |
| sp\|Q9UKP6\|UR2R_HUMAN Urotensin-2 receptor OS=Homo sapiens GN=UTS2R PE=1 SV=1 | 319 | FLYTLLtRNYR | 0.149 | -0.059 | -1.006 | -0.305 |
| sp\|Q9UKP6\|UR2R_HUMAN Urotensin-2 receptor OS=Homo sapiens GN=UTS2R PE=1 SV=1 | 335 | RVRGPGsGGGR | 0.238 | 0.052 | -0.43 | -0.047 |
| sp\|Q9UKP6\|UR2R_HUMAN Urotensin-2 receptor OS=Homo sapiens GN=UTS2R PE=1 SV=1 | 344 | GRGPVPsLQPR | 0.336 | 0.148 | -0.38 | 0.035 |
| sp\|Q9UKP6\|UR2R_HUMAN Urotensin-2 receptor OS=Homo sapiens GN=UTS2R PE=1 SV=1 | 355 | ARFQRCsGRSL | 0.383 | 0.228 | -0.479 | 0.044 |
| sp\|Q9UKP6\|UR2R_HUMAN Urotensin-2 receptor OS=Homo sapiens GN=UTS2R PE=1 SV=1 | 358 | QRCSGRsLSSC | 0.054 | -0.055 | -1.497 | -0.499 |
| sp\|Q9UKP6\|UR2R_HUMAN Urotensin-2 receptor OS=Homo sapiens GN=UTS2R PE=1 SV=1 | 360 | CSGRSLsSCSP | 0.254 | 0.722 | -0.166 | 0.27 |
| sp\|Q9UKP6\|UR2R_HUMAN Urotensin-2 receptor OS=Homo sapiens GN=UTS2R PE=1 SV=1 | 361 | SGRSLSsCSPQ | 0.064 | -0.135 | -1.535 | -0.535 |
| sp\|Q9UKP6\|UR2R_HUMAN Urotensin-2 receptor OS=Homo sapiens GN=UTS2R PE=1 SV=1 | 363 | RSLSSCsPQPT | 0.176 | -0.266 | -0.914 | -0.335 |
| sp\|Q9UKP6\|UR2R_HUMAN Urotensin-2 receptor OS=Homo sapiens GN=UTS2R PE=1 SV=1 | 367 | SCSPQPtDSLV | 0.076 | -0.221 | -1.841 | -0.662 |
| sp\|Q9UKP6\|UR2R_HUMAN Urotensin-2 receptor OS=Homo sapiens GN=UTS2R PE=1 SV=1 | 369 | SPQPTDsLVLA | 0.117 | -0.098 | -1.074 | -0.352 |
| sp\|P37288\|V1AR_HUMAN Vasopressin V1a receptor OS=Homo sapiens GN=AVPR1A PE=1 SV=1 | 352 | WIYMFFsGHLL | 0.194 | 0.011 | -0.63 | -0.142 |
| sp\|P37288\|V1AR_HUMAN Vasopressin V1a receptor OS=Homo sapiens GN=AVPR1A PE=1 SV=1 | 362 | LQDCVQsFPCC | 0.52 | 0.454 | -0.116 | 0.286 |
| sp\|P37288\|V1AR_HUMAN Vasopressin V1a receptor OS=Homo sapiens GN=AVPR1A PE=1 SV=1 | 378 | KFNKEDtDSMS | 0.154 | -0.243 | -1.489 | -0.526 |
| sp\|P37288\|V1AR_HUMAN Vasopressin V1a receptor OS=Homo sapiens GN=AVPR1A PE=1 SV=1 | 380 | NKEDTDsMSRR | 0.184 | -0.037 | -1.142 | -0.332 |

| | | | | | | |
|---|---|---|---|---|---|---|
| sp\|P37288\|V1AR_HUMAN Vasopressin V1a receptor OS=Homo sapiens GN=AVPR1A PE=1 SV=1 | 382 | EDTDSMsRRQT | 0.232 | 0.009 | -0.508 | -0.089 |
| sp\|P37288\|V1AR_HUMAN Vasopressin V1a receptor OS=Homo sapiens GN=AVPR1A PE=1 SV=1 | 386 | SMSRRQtFYSN | 0.559 | 1.081 | 0.274 | 0.638 |
| sp\|P37288\|V1AR_HUMAN Vasopressin V1a receptor OS=Homo sapiens GN=AVPR1A PE=1 SV=1 | 389 | RRQTFYsNNRS | 0.287 | 0.21 | -0.817 | -0.107 |
| sp\|P37288\|V1AR_HUMAN Vasopressin V1a receptor OS=Homo sapiens GN=AVPR1A PE=1 SV=1 | 393 | FYSNNRsPTNS | 0.094 | -0.256 | -1.352 | -0.505 |
| sp\|P37288\|V1AR_HUMAN Vasopressin V1a receptor OS=Homo sapiens GN=AVPR1A PE=1 SV=1 | 395 | SNNRSPtNSTG | 0.414 | 0.711 | -0.206 | 0.306 |
| sp\|P37288\|V1AR_HUMAN Vasopressin V1a receptor OS=Homo sapiens GN=AVPR1A PE=1 SV=1 | 397 | NRSPTNsTGMW | 0.207 | 0.171 | -0.774 | -0.132 |
| sp\|P37288\|V1AR_HUMAN Vasopressin V1a receptor OS=Homo sapiens GN=AVPR1A PE=1 SV=1 | 398 | RSPTNStGMWK | 0.083 | -0.292 | -1.22 | -0.476 |
| sp\|P37288\|V1AR_HUMAN Vasopressin V1a receptor OS=Homo sapiens GN=AVPR1A PE=1 SV=1 | 404 | TGMWKDsPKSS | 0.026 | -0.597 | -1.807 | -0.793 |
| sp\|P37288\|V1AR_HUMAN Vasopressin V1a receptor OS=Homo sapiens GN=AVPR1A PE=1 SV=1 | 407 | WKDSPKsSKSI | 0.167 | -0.153 | -0.52 | -0.169 |
| sp\|P37288\|V1AR_HUMAN Vasopressin V1a receptor OS=Homo sapiens GN=AVPR1A PE=1 SV=1 | 408 | KDSPKSsKSIK | 0.031 | -0.498 | -1.71 | -0.726 |
| sp\|P37288\|V1AR_HUMAN Vasopressin V1a receptor OS=Homo sapiens GN=AVPR1A PE=1 SV=1 | 410 | SPKSSKsIKFI | 0.252 | 0.043 | -0.313 | -0.006 |
| sp\|P37288\|V1AR_HUMAN Vasopressin V1a receptor OS=Homo sapiens GN=AVPR1A PE=1 SV=1 | 417 | IKFIPVsT--- | 0.434 | 0.127 | -0.319 | 0.081 |
| sp\|P37288\|V1AR_HUMAN Vasopressin V1a receptor OS=Homo sapiens GN=AVPR1A PE=1 SV=1 | 418 | KFIPVSt---- | 0.114 | -0.013 | -1.092 | -0.33 |
| sp\|P47901\|V1BR_HUMAN Vasopressin V1b receptor OS=Homo sapiens GN=AVPR1B PE=2 SV=1 | 343 | IYMGFNsHLLP | 0.209 | 0.005 | -0.88 | -0.222 |
| sp\|P47901\|V1BR_HUMAN Vasopressin V1b receptor OS=Homo sapiens GN=AVPR1B PE=2 SV=1 | 368 | RMRRRLsDGSL | 0.837 | 1.428 | 0.832 | 1.032 |
| sp\|P47901\|V1BR_HUMAN Vasopressin V1b receptor OS=Homo sapiens GN=AVPR1B PE=2 SV=1 | 371 | RRLSDGsLSSR | 0.132 | 0.043 | -0.908 | -0.244 |
| sp\|P47901\|V1BR_HUMAN Vasopressin V1b receptor OS=Homo sapiens GN=AVPR1B PE=2 SV=1 | 373 | LSDGSLsSRHT | 0.08 | -0.18 | -0.88 | -0.327 |
| sp\|P47901\|V1BR_HUMAN Vasopressin V1b receptor OS=Homo sapiens GN=AVPR1B PE=2 SV=1 | 374 | SDGSLSsRHTT | 0.018 | -0.536 | -2.169 | -0.896 |
| sp\|P47901\|V1BR_HUMAN Vasopressin V1b receptor OS=Homo sapiens GN=AVPR1B PE=2 SV=1 | 377 | SLSSRHtTLLT | 0.243 | 0.114 | -0.796 | -0.146 |
| sp\|P47901\|V1BR_HUMAN Vasopressin V1b receptor OS=Homo sapiens GN=AVPR1B PE=2 SV=1 | 378 | LSSRHTtLLTR | 0.707 | 0.848 | 0.602 | 0.719 |
| sp\|P47901\|V1BR_HUMAN Vasopressin V1b receptor OS=Homo sapiens GN=AVPR1B PE=2 SV=1 | 381 | RHTTLLtRSSC | 0.036 | -0.388 | -2.296 | -0.883 |
| sp\|P47901\|V1BR_HUMAN Vasopressin V1b receptor OS=Homo sapiens GN=AVPR1B PE=2 SV=1 | 383 | TTLLTRsSCPA | 0.048 | -0.297 | -1.623 | -0.624 |
| sp\|P47901\|V1BR_HUMAN Vasopressin V1b receptor OS=Homo sapiens GN=AVPR1B PE=2 SV=1 | 384 | TLLTRSsCPAT | 0.719 | 0.691 | 0.399 | 0.603 |
| sp\|P47901\|V1BR_HUMAN Vasopressin V1b receptor OS=Homo sapiens GN=AVPR1B PE=2 SV=1 | 388 | RSSCPAtLSLS | 0.251 | -0.118 | -0.776 | -0.214 |
| sp\|P47901\|V1BR_HUMAN Vasopressin V1b receptor OS=Homo sapiens GN=AVPR1B PE=2 SV=1 | 390 | SCPATLsLSLS | 0.062 | -0.133 | -1.444 | -0.505 |
| sp\|P47901\|V1BR_HUMAN Vasopressin V1b receptor OS=Homo sapiens GN=AVPR1B PE=2 SV=1 | 392 | PATLSLsLSLT | 0.259 | 0.122 | -0.373 | 0.003 |
| sp\|P47901\|V1BR_HUMAN Vasopressin V1b receptor OS=Homo sapiens GN=AVPR1B PE=2 SV=1 | 394 | TLSLSLsLTLS | 0.474 | 0.248 | -0.151 | 0.19 |
| sp\|P47901\|V1BR_HUMAN Vasopressin V1b receptor OS=Homo sapiens GN=AVPR1B PE=2 SV=1 | 396 | SLSLSLtLSGR | 0.325 | 0.145 | -0.305 | 0.055 |
| sp\|P47901\|V1BR_HUMAN Vasopressin V1b receptor OS=Homo sapiens GN=AVPR1B PE=2 SV=1 | 398 | SLSLTLsGRPR | 0.071 | -0.207 | -1.23 | -0.455 |
| sp\|P47901\|V1BR_HUMAN Vasopressin V1b receptor OS=Homo sapiens GN=AVPR1B PE=2 SV=1 | 406 | RPRPEEsPRDL | 0.228 | -0.179 | -1.026 | -0.326 |
| sp\|P47901\|V1BR_HUMAN Vasopressin V1b receptor OS=Homo sapiens GN=AVPR1B PE=2 SV=1 | 418 | LADGEGtAETI | 0.097 | -0.193 | -1.3 | -0.465 |
| sp\|P47901\|V1BR_HUMAN Vasopressin V1b receptor OS=Homo sapiens GN=AVPR1B PE=2 SV=1 | 421 | GEGTAEtIIF- | 0.101 | -0.13 | -1.34 | -0.456 |
| sp\|P30518\|V2R_HUMAN Vasopressin V2 receptor OS=Homo sapiens GN=AVPR2 PE=1 SV=1 | 329 | WIYASFsSSVS | 0.186 | 0.135 | -0.576 | -0.085 |
| sp\|P30518\|V2R_HUMAN Vasopressin V2 receptor OS=Homo sapiens GN=AVPR2 PE=1 SV=1 | 330 | IYASFSsSVSS | 0.061 | -0.262 | -1.439 | -0.547 |
| sp\|P30518\|V2R_HUMAN Vasopressin V2 receptor OS=Homo sapiens GN=AVPR2 PE=1 SV=1 | 331 | YASFSSsVSSE | 0.113 | -0.064 | -1.016 | -0.322 |
| sp\|P30518\|V2R_HUMAN Vasopressin V2 receptor OS=Homo sapiens GN=AVPR2 PE=1 SV=1 | 333 | SFSSSVsSELR | 0.295 | 0.046 | -0.503 | -0.054 |
| sp\|P30518\|V2R_HUMAN Vasopressin V2 receptor OS=Homo sapiens GN=AVPR2 PE=1 SV=1 | 334 | FSSSVSsELRS | 0.079 | -0.239 | -1.522 | -0.561 |
| sp\|P30518\|V2R_HUMAN Vasopressin V2 receptor OS=Homo sapiens GN=AVPR2 PE=1 SV=1 | 338 | VSSELRsLLCC | 0.053 | -0.214 | -1.679 | -0.613 |
| sp\|P30518\|V2R_HUMAN Vasopressin V2 receptor OS=Homo sapiens GN=AVPR2 PE=1 SV=1 | 347 | CCARGRtPPSL | 0.604 | 1.047 | 0.355 | 0.669 |
| sp\|P30518\|V2R_HUMAN Vasopressin V2 receptor OS=Homo sapiens GN=AVPR2 PE=1 SV=1 | 350 | RGRTPPsLGPQ | 0.472 | 0.11 | -0.123 | 0.153 |
| sp\|P30518\|V2R_HUMAN Vasopressin V2 receptor OS=Homo sapiens GN=AVPR2 PE=1 SV=1 | 357 | LGPQDEsCTTA | 0.148 | -0.194 | -1.001 | -0.349 |
| sp\|P30518\|V2R_HUMAN Vasopressin V2 receptor OS=Homo sapiens GN=AVPR2 PE=1 SV=1 | 359 | PQDESCtTASS | 0.11 | 0.001 | -1.088 | -0.326 |
| sp\|P30518\|V2R_HUMAN Vasopressin V2 receptor OS=Homo sapiens GN=AVPR2 PE=1 SV=1 | 360 | QDESCTtASSS | 0.051 | -0.294 | -1.5 | -0.581 |
| sp\|P30518\|V2R_HUMAN Vasopressin V2 receptor OS=Homo sapiens GN=AVPR2 PE=1 SV=1 | 362 | ESCTTAsSSLA | 0.082 | -0.173 | -1.297 | -0.463 |
| sp\|P30518\|V2R_HUMAN Vasopressin V2 receptor OS=Homo sapiens GN=AVPR2 PE=1 SV=1 | 363 | SCTTASsSLAK | 0.135 | -0.103 | -1.188 | -0.385 |
| sp\|P30518\|V2R_HUMAN Vasopressin V2 receptor OS=Homo sapiens GN=AVPR2 PE=1 SV=1 | 364 | CTTASSsLAKD | 0.466 | 0.145 | -0.026 | 0.195 |
| sp\|P30518\|V2R_HUMAN Vasopressin V2 receptor OS=Homo sapiens GN=AVPR2 PE=1 SV=1 | 369 | SSLAKDtSS-- | 0.045 | -0.305 | -1.814 | -0.691 |
| sp\|P30518\|V2R_HUMAN Vasopressin V2 receptor OS=Homo sapiens GN=AVPR2 PE=1 SV=1 | 370 | SLAKDTsS--- | 0.277 | 0.132 | -0.616 | -0.069 |
| sp\|P30518\|V2R_HUMAN Vasopressin V2 receptor OS=Homo sapiens GN=AVPR2 PE=1 SV=1 | 371 | LAKDTSs---- | 0.103 | 0.05 | -1.051 | -0.299 |

| | | | | | | |
|---|---|---|---|---|---|---|
| sp\|P30559\|OXYR_HUMAN Oxytocin receptor OS=Homo sapiens GN=OXTR PE=2 SV=2 | 333 | WIYMLFtGHLF | 0.11 | -0.082 | -0.85 | -0.274 |
| sp\|P30559\|OXYR_HUMAN Oxytocin receptor OS=Homo sapiens GN=OXTR PE=2 SV=2 | 348 | QRFLCCsASYL | 0.311 | 0.214 | -0.906 | -0.127 |
| sp\|P30559\|OXYR_HUMAN Oxytocin receptor OS=Homo sapiens GN=OXTR PE=2 SV=2 | 350 | FLCCSAsYLKG | 0.522 | 0.303 | -0.327 | 0.166 |
| sp\|P30559\|OXYR_HUMAN Oxytocin receptor OS=Homo sapiens GN=OXTR PE=2 SV=2 | 360 | GRRLGEtSASK | 0.142 | 0.168 | -1.104 | -0.265 |
| sp\|P30559\|OXYR_HUMAN Oxytocin receptor OS=Homo sapiens GN=OXTR PE=2 SV=2 | 361 | RRLGETsASKK | 0.14 | 0.083 | -1.048 | -0.275 |
| sp\|P30559\|OXYR_HUMAN Oxytocin receptor OS=Homo sapiens GN=OXTR PE=2 SV=2 | 363 | LGETSAsKKSN | 0.262 | 0.099 | -0.384 | -0.008 |
| sp\|P30559\|OXYR_HUMAN Oxytocin receptor OS=Homo sapiens GN=OXTR PE=2 SV=2 | 366 | TSASKKsNSSS | 0.037 | -0.361 | -1.553 | -0.626 |
| sp\|P30559\|OXYR_HUMAN Oxytocin receptor OS=Homo sapiens GN=OXTR PE=2 SV=2 | 368 | ASKKSNsSSFV | 0.39 | 0.055 | -0.719 | -0.091 |
| sp\|P30559\|OXYR_HUMAN Oxytocin receptor OS=Homo sapiens GN=OXTR PE=2 SV=2 | 369 | SKKSNSsSFVL | 0.182 | -0.068 | -0.778 | -0.221 |
| sp\|P30559\|OXYR_HUMAN Oxytocin receptor OS=Homo sapiens GN=OXTR PE=2 SV=2 | 370 | KKSNSSsFVLS | 0.323 | 0.186 | -0.247 | 0.087 |
| sp\|P30559\|OXYR_HUMAN Oxytocin receptor OS=Homo sapiens GN=OXTR PE=2 SV=2 | 374 | SSSFVLsHRSS | 0.064 | -0.339 | -1.587 | -0.621 |
| sp\|P30559\|OXYR_HUMAN Oxytocin receptor OS=Homo sapiens GN=OXTR PE=2 SV=2 | 377 | FVLSHRsSSQR | 0.102 | -0.094 | -1.162 | -0.385 |
| sp\|P30559\|OXYR_HUMAN Oxytocin receptor OS=Homo sapiens GN=OXTR PE=2 SV=2 | 378 | VLSHRSsSQRS | 0.207 | -0.074 | -0.904 | -0.257 |
| sp\|P30559\|OXYR_HUMAN Oxytocin receptor OS=Homo sapiens GN=OXTR PE=2 SV=2 | 379 | LSHRSSsQRSC | 0.272 | 0.646 | -0.418 | 0.167 |
| sp\|P30559\|OXYR_HUMAN Oxytocin receptor OS=Homo sapiens GN=OXTR PE=2 SV=2 | 382 | RSSSQRsCSQP | 0.051 | -0.252 | -1.707 | -0.636 |
| sp\|P30559\|OXYR_HUMAN Oxytocin receptor OS=Homo sapiens GN=OXTR PE=2 SV=2 | 384 | SSQRSCsQPST | 0.862 | 1.363 | 0.712 | 0.979 |
| sp\|P30559\|OXYR_HUMAN Oxytocin receptor OS=Homo sapiens GN=OXTR PE=2 SV=2 | 387 | RSCSQPsTA-- | 0.092 | -0.131 | -1.44 | -0.493 |
| sp\|P30559\|OXYR_HUMAN Oxytocin receptor OS=Homo sapiens GN=OXTR PE=2 SV=2 | 388 | SCSQPStA--- | 0.124 | -0.037 | -1.227 | -0.38 |
| sp\|Q99788\|CML1_HUMAN Chemokine-like receptor 1 OS=Homo sapiens GN=CMKLR1 PE=1 SV=2 | 332 | FKVALFsRLVN | 0.185 | 0.018 | -0.988 | -0.262 |
| sp\|Q99788\|CML1_HUMAN Chemokine-like receptor 1 OS=Homo sapiens GN=CMKLR1 PE=1 SV=2 | 339 | RLVNALsEDTG | 0.394 | 0.222 | -0.208 | 0.136 |
| sp\|Q99788\|CML1_HUMAN Chemokine-like receptor 1 OS=Homo sapiens GN=CMKLR1 PE=1 SV=2 | 342 | NALSEDtGHSS | 0.058 | -0.375 | -1.708 | -0.675 |
| sp\|Q99788\|CML1_HUMAN Chemokine-like receptor 1 OS=Homo sapiens GN=CMKLR1 PE=1 SV=2 | 345 | SEDTGHsSYPS | 0.049 | -0.231 | -1.763 | -0.648 |
| sp\|Q99788\|CML1_HUMAN Chemokine-like receptor 1 OS=Homo sapiens GN=CMKLR1 PE=1 SV=2 | 346 | EDTGHSsYPSH | 0.089 | 0.125 | -0.956 | -0.247 |
| sp\|Q99788\|CML1_HUMAN Chemokine-like receptor 1 OS=Homo sapiens GN=CMKLR1 PE=1 SV=2 | 349 | GHSSYPsHRSF | 0.112 | -0.266 | -1.394 | -0.516 |
| sp\|Q99788\|CML1_HUMAN Chemokine-like receptor 1 OS=Homo sapiens GN=CMKLR1 PE=1 SV=2 | 352 | SYPSHRsFTKM | 0.145 | -0.114 | -1.261 | -0.41 |
| sp\|Q99788\|CML1_HUMAN Chemokine-like receptor 1 OS=Homo sapiens GN=CMKLR1 PE=1 SV=2 | 354 | PSHRSFtKMSS | 0.565 | 0.953 | 0.396 | 0.638 |
| sp\|Q99788\|CML1_HUMAN Chemokine-like receptor 1 OS=Homo sapiens GN=CMKLR1 PE=1 SV=2 | 357 | RSFTKMsSMNE | 0.189 | -0.013 | -1.143 | -0.322 |
| sp\|Q99788\|CML1_HUMAN Chemokine-like receptor 1 OS=Homo sapiens GN=CMKLR1 PE=1 SV=2 | 358 | SFTKMSsMNER | 0.296 | 0.035 | -0.483 | -0.051 |
| sp\|Q99788\|CML1_HUMAN Chemokine-like receptor 1 OS=Homo sapiens GN=CMKLR1 PE=1 SV=2 | 363 | SSMNERtSMNE | 0.051 | -0.218 | -1.783 | -0.65 |
| sp\|Q99788\|CML1_HUMAN Chemokine-like receptor 1 OS=Homo sapiens GN=CMKLR1 PE=1 SV=2 | 364 | SMNERTsMNER | 0.189 | 0.287 | -0.834 | -0.119 |
| sp\|Q99788\|CML1_HUMAN Chemokine-like receptor 1 OS=Homo sapiens GN=CMKLR1 PE=1 SV=2 | 370 | SMNEREtGML- | 0.083 | 0.067 | -1.475 | -0.442 |
| sp\|P32246\|CCR1_HUMAN C-C chemokine receptor type 1 OS=Homo sapiens GN=CCR1 PE=1 SV=1 | 332 | KWLPFLsVDRL | 0.131 | -0.133 | -1.075 | -0.359 |
| sp\|P32246\|CCR1_HUMAN C-C chemokine receptor type 1 OS=Homo sapiens GN=CCR1 PE=1 SV=1 | 340 | DRLERVsSTSP | 0.254 | 0.146 | -0.856 | -0.152 |
| sp\|P32246\|CCR1_HUMAN C-C chemokine receptor type 1 OS=Homo sapiens GN=CCR1 PE=1 SV=1 | 341 | RLERVSsTSPS | 0.459 | 0.726 | -0.098 | 0.362 |
| sp\|P32246\|CCR1_HUMAN C-C chemokine receptor type 1 OS=Homo sapiens GN=CCR1 PE=1 SV=1 | 342 | LERVSStSPST | 0.426 | 0.664 | -0.035 | 0.352 |
| sp\|P32246\|CCR1_HUMAN C-C chemokine receptor type 1 OS=Homo sapiens GN=CCR1 PE=1 SV=1 | 343 | ERVSSTsPSTG | 0.104 | -0.043 | -0.927 | -0.289 |
| sp\|P32246\|CCR1_HUMAN C-C chemokine receptor type 1 OS=Homo sapiens GN=CCR1 PE=1 SV=1 | 345 | VSSTSPsTGEH | 0.255 | -0.099 | -0.776 | -0.207 |
| sp\|P32246\|CCR1_HUMAN C-C chemokine receptor type 1 OS=Homo sapiens GN=CCR1 PE=1 SV=1 | 346 | SSTSPStGEHE | 0.035 | -0.527 | -1.663 | -0.718 |
| sp\|P32246\|CCR1_HUMAN C-C chemokine receptor type 1 OS=Homo sapiens GN=CCR1 PE=1 SV=1 | 352 | TGEHELsAGF- | 0.112 | -0.17 | -1.239 | -0.432 |
| sp\|P41597\|CCR2_HUMAN C-C chemokine receptor type 2 OS=Homo sapiens GN=CCR2 PE=1 SV=1 | 314 | VGEKFRsLFHI | 0.41 | 0.022 | -0.159 | 0.091 |
| sp\|P41597\|CCR2_HUMAN C-C chemokine receptor type 2 OS=Homo sapiens GN=CCR2 PE=1 SV=1 | 346 | GKNVKVtTQGL | 0.325 | -0.009 | -0.581 | -0.088 |
| sp\|P41597\|CCR2_HUMAN C-C chemokine receptor type 2 OS=Homo sapiens GN=CCR2 PE=1 SV=1 | 347 | KNVKVTtQGLL | 0.259 | -0.052 | -0.792 | -0.195 |
| sp\|P41597\|CCR2_HUMAN C-C chemokine receptor type 2 OS=Homo sapiens GN=CCR2 PE=1 SV=1 | 359 | GRGKGKsIGRA | 0.393 | 0.169 | -0.294 | 0.089 |
| sp\|P41597\|CCR2_HUMAN C-C chemokine receptor type 2 OS=Homo sapiens GN=CCR2 PE=1 SV=1 | 367 | GRAPEAsLQDK | 0.63 | 0.296 | 0.055 | 0.327 |
| sp\|P51677\|CCR3_HUMAN C-C chemokine receptor type 3 OS=Homo sapiens GN=CCR3 PE=1 SV=1 | 333 | YIPFLPsEKLE | 0.12 | -0.061 | -1.034 | -0.325 |
| sp\|P51677\|CCR3_HUMAN C-C chemokine receptor type 3 OS=Homo sapiens GN=CCR3 PE=1 SV=1 | 339 | SEKLERtSSVS | 0.046 | -0.246 | -1.975 | -0.725 |
| sp\|P51677\|CCR3_HUMAN C-C chemokine receptor type 3 OS=Homo sapiens GN=CCR3 PE=1 SV=1 | 340 | EKLERTsSVSP | 0.103 | 0.004 | -0.937 | -0.277 |
| sp\|P51677\|CCR3_HUMAN C-C chemokine receptor type 3 OS=Homo sapiens GN=CCR3 PE=1 SV=1 | 341 | KLERTSsVSPS | 0.308 | 0.721 | -0.168 | 0.287 |
| sp\|P51677\|CCR3_HUMAN C-C chemokine receptor type 3 OS=Homo sapiens GN=CCR3 PE=1 SV=1 | 343 | ERTSSVsPSTA | 0.19 | -0.053 | -0.615 | -0.159 |

| | | | | | | |
|---|---|---|---|---|---|---|
| sp\|P51677\|CCR3_HUMAN C-C chemokine receptor type 3 OS=Homo sapiens GN=CCR3 PE=1 SV=1 | 345 | TSSVSPsTAEP | 0.117 | -0.151 | -1.056 | -0.363 |
| sp\|P51677\|CCR3_HUMAN C-C chemokine receptor type 3 OS=Homo sapiens GN=CCR3 PE=1 SV=1 | 346 | SSVSPStAEPE | 0.041 | -0.442 | -1.844 | -0.748 |
| sp\|P51677\|CCR3_HUMAN C-C chemokine receptor type 3 OS=Homo sapiens GN=CCR3 PE=1 SV=1 | 352 | TAEPELsIVF- | 0.038 | -0.366 | -1.671 | -0.666 |
| sp\|P51679\|CCR4_HUMAN C-C chemokine receptor type 4 OS=Homo sapiens GN=CCR4 PE=1 SV=1 | 321 | ILQLFKtCRGL | 0.548 | 0.175 | -0.077 | 0.215 |
| sp\|P51679\|CCR4_HUMAN C-C chemokine receptor type 4 OS=Homo sapiens GN=CCR4 PE=1 SV=1 | 339 | GLLQIYsADTP | 0.421 | 0.18 | -0.063 | 0.179 |
| sp\|P51679\|CCR4_HUMAN C-C chemokine receptor type 4 OS=Homo sapiens GN=CCR4 PE=1 SV=1 | 342 | QIYSADtPSSS | 0.034 | -0.479 | -1.856 | -0.767 |
| sp\|P51679\|CCR4_HUMAN C-C chemokine receptor type 4 OS=Homo sapiens GN=CCR4 PE=1 SV=1 | 344 | YSADTPsSSYT | 0.052 | -0.308 | -1.488 | -0.581 |
| sp\|P51679\|CCR4_HUMAN C-C chemokine receptor type 4 OS=Homo sapiens GN=CCR4 PE=1 SV=1 | 345 | SADTPSsSYTQ | 0.033 | -0.463 | -1.911 | -0.78 |
| sp\|P51679\|CCR4_HUMAN C-C chemokine receptor type 4 OS=Homo sapiens GN=CCR4 PE=1 SV=1 | 346 | ADTPSSsYTQS | 0.105 | -0.177 | -0.981 | -0.351 |
| sp\|P51679\|CCR4_HUMAN C-C chemokine receptor type 4 OS=Homo sapiens GN=CCR4 PE=1 SV=1 | 348 | TPSSSYtQSTM | 0.126 | -0.059 | -0.93 | -0.288 |
| sp\|P51679\|CCR4_HUMAN C-C chemokine receptor type 4 OS=Homo sapiens GN=CCR4 PE=1 SV=1 | 350 | SSSYTQsTMDH | 0.093 | -0.183 | -1.237 | -0.442 |
| sp\|P51679\|CCR4_HUMAN C-C chemokine receptor type 4 OS=Homo sapiens GN=CCR4 PE=1 SV=1 | 351 | SSYTQStMDHD | 0.058 | -0.158 | -1.426 | -0.509 |
| sp\|P51681\|CCR5_HUMAN C-C chemokine receptor type 5 OS=Homo sapiens GN=CCR5 PE=1 SV=1 | 325 | RFCKCCsIFQQ | 0.608 | 0.042 | -0.249 | 0.134 |
| sp\|P51681\|CCR5_HUMAN C-C chemokine receptor type 5 OS=Homo sapiens GN=CCR5 PE=1 SV=1 | 336 | EAPERAsSVYT | 0.075 | -0.123 | -1.365 | -0.471 |
| sp\|P51681\|CCR5_HUMAN C-C chemokine receptor type 5 OS=Homo sapiens GN=CCR5 PE=1 SV=1 | 337 | APERASsVYTR | 0.571 | 0.764 | 0.193 | 0.509 |
| sp\|P51681\|CCR5_HUMAN C-C chemokine receptor type 5 OS=Homo sapiens GN=CCR5 PE=1 SV=1 | 340 | RASSVYtRSTG | 0.106 | -0.232 | -1.25 | -0.459 |
| sp\|P51681\|CCR5_HUMAN C-C chemokine receptor type 5 OS=Homo sapiens GN=CCR5 PE=1 SV=1 | 342 | SSVYTRsTGEQ | 0.086 | -0.109 | -1.319 | -0.447 |
| sp\|P51681\|CCR5_HUMAN C-C chemokine receptor type 5 OS=Homo sapiens GN=CCR5 PE=1 SV=1 | 343 | SVYTRStGEQE | 0.083 | -0.162 | -1.262 | -0.447 |
| sp\|P51681\|CCR5_HUMAN C-C chemokine receptor type 5 OS=Homo sapiens GN=CCR5 PE=1 SV=1 | 349 | TGEQEIsVGL- | 0.298 | -0.029 | -0.515 | -0.082 |
| sp\|P51684\|CCR6_HUMAN C-C chemokine receptor type 6 OS=Homo sapiens GN=CCR6 PE=1 SV=2 | 343 | VRRKYKsSGFS | 0.626 | 0.447 | 0.082 | 0.385 |
| sp\|P51684\|CCR6_HUMAN C-C chemokine receptor type 6 OS=Homo sapiens GN=CCR6 PE=1 SV=2 | 344 | RRKYKSsGFSC | 0.083 | 0.015 | -1.061 | -0.321 |
| sp\|P51684\|CCR6_HUMAN C-C chemokine receptor type 6 OS=Homo sapiens GN=CCR6 PE=1 SV=2 | 347 | YKSSGFsCAGR | 0.169 | -0.077 | -0.698 | -0.202 |
| sp\|P51684\|CCR6_HUMAN C-C chemokine receptor type 6 OS=Homo sapiens GN=CCR6 PE=1 SV=2 | 353 | SCAGRYsENIS | 0.381 | 0.259 | -0.278 | 0.121 |
| sp\|P51684\|CCR6_HUMAN C-C chemokine receptor type 6 OS=Homo sapiens GN=CCR6 PE=1 SV=2 | 357 | RYSENIsRQTS | 0.179 | -0.133 | -0.955 | -0.303 |
| sp\|P51684\|CCR6_HUMAN C-C chemokine receptor type 6 OS=Homo sapiens GN=CCR6 PE=1 SV=2 | 360 | ENISRQtSETA | 0.119 | -0.106 | -0.932 | -0.306 |
| sp\|P51684\|CCR6_HUMAN C-C chemokine receptor type 6 OS=Homo sapiens GN=CCR6 PE=1 SV=2 | 361 | NISRQTsETAD | 0.76 | 1.043 | 0.703 | 0.835 |
| sp\|P51684\|CCR6_HUMAN C-C chemokine receptor type 6 OS=Homo sapiens GN=CCR6 PE=1 SV=2 | 363 | SRQTSEtADND | 0.466 | 0.483 | -0.47 | 0.16 |
| sp\|P51684\|CCR6_HUMAN C-C chemokine receptor type 6 OS=Homo sapiens GN=CCR6 PE=1 SV=2 | 370 | ADNDNAsSFTM | 0.136 | -0.21 | -1.158 | -0.411 |
| sp\|P51684\|CCR6_HUMAN C-C chemokine receptor type 6 OS=Homo sapiens GN=CCR6 PE=1 SV=2 | 371 | DNDNASsFTM- | 0.19 | -0.142 | -0.942 | -0.298 |
| sp\|P51684\|CCR6_HUMAN C-C chemokine receptor type 6 OS=Homo sapiens GN=CCR6 PE=1 SV=2 | 373 | DNASSFtM--- | 0.336 | 0.326 | -0.4 | 0.087 |
| sp\|P32248\|CCR7_HUMAN C-C chemokine receptor type 7 OS=Homo sapiens GN=CCR7 PE=2 SV=2 | 348 | KDLGCLsQEQL | 0.077 | -0.234 | -1.249 | -0.469 |
| sp\|P32248\|CCR7_HUMAN C-C chemokine receptor type 7 OS=Homo sapiens GN=CCR7 PE=2 SV=2 | 356 | EQLRQWsSCRH | 0.208 | 0.427 | -0.715 | -0.027 |
| sp\|P32248\|CCR7_HUMAN C-C chemokine receptor type 7 OS=Homo sapiens GN=CCR7 PE=2 SV=2 | 357 | QLRQWSsCRHI | 0.543 | 0.292 | 0.156 | 0.33 |
| sp\|P32248\|CCR7_HUMAN C-C chemokine receptor type 7 OS=Homo sapiens GN=CCR7 PE=2 SV=2 | 364 | CRHIRRsSMSV | 0.149 | 0.254 | -1.159 | -0.252 |
| sp\|P32248\|CCR7_HUMAN C-C chemokine receptor type 7 OS=Homo sapiens GN=CCR7 PE=2 SV=2 | 365 | RHIRRSsMSVE | 0.521 | 1.013 | -0.257 | 0.426 |
| sp\|P32248\|CCR7_HUMAN C-C chemokine receptor type 7 OS=Homo sapiens GN=CCR7 PE=2 SV=2 | 367 | IRRSSMsVEAE | 0.77 | 0.771 | 0.649 | 0.73 |
| sp\|P32248\|CCR7_HUMAN C-C chemokine receptor type 7 OS=Homo sapiens GN=CCR7 PE=2 SV=2 | 372 | MSVEAEtTTTF | 0.15 | -0.096 | -1.115 | -0.354 |
| sp\|P32248\|CCR7_HUMAN C-C chemokine receptor type 7 OS=Homo sapiens GN=CCR7 PE=2 SV=2 | 373 | SVEAETtTTFS | 0.069 | -0.317 | -1.56 | -0.603 |
| sp\|P32248\|CCR7_HUMAN C-C chemokine receptor type 7 OS=Homo sapiens GN=CCR7 PE=2 SV=2 | 374 | VEAETTtTFSP | 0.038 | -0.212 | -1.486 | -0.553 |
| sp\|P32248\|CCR7_HUMAN C-C chemokine receptor type 7 OS=Homo sapiens GN=CCR7 PE=2 SV=2 | 375 | EAETTTtFSP- | 0.088 | -0.123 | -1.071 | -0.369 |
| sp\|P32248\|CCR7_HUMAN C-C chemokine receptor type 7 OS=Homo sapiens GN=CCR7 PE=2 SV=2 | 377 | ETTTTFsP--- | 0.066 | -0.155 | -1.255 | -0.448 |
| sp\|P51685\|CCR8_HUMAN C-C chemokine receptor type 8 OS=Homo sapiens GN=CCR8 PE=1 SV=1 | 312 | KFKKHLsEIFQ | 0.251 | 0.013 | -0.56 | -0.099 |
| sp\|P51685\|CCR8_HUMAN C-C chemokine receptor type 8 OS=Homo sapiens GN=CCR8 PE=1 SV=1 | 318 | SEIFQKsCSQI | 0.165 | 0.029 | -0.959 | -0.255 |
| sp\|P51685\|CCR8_HUMAN C-C chemokine receptor type 8 OS=Homo sapiens GN=CCR8 PE=1 SV=1 | 320 | IFQKSCsQIFN | 0.574 | 0.229 | -0.191 | 0.204 |
| sp\|P51685\|CCR8_HUMAN C-C chemokine receptor type 8 OS=Homo sapiens GN=CCR8 PE=1 SV=1 | 334 | RQMPREsCEKS | 0.151 | 0.091 | -0.97 | -0.243 |
| sp\|P51685\|CCR8_HUMAN C-C chemokine receptor type 8 OS=Homo sapiens GN=CCR8 PE=1 SV=1 | 338 | RESCEKsSSCQ | 0.161 | -0.14 | -1.316 | -0.432 |
| sp\|P51685\|CCR8_HUMAN C-C chemokine receptor type 8 OS=Homo sapiens GN=CCR8 PE=1 SV=1 | 339 | ESCEKSsSCQQ | 0.026 | -0.462 | -1.905 | -0.78 |
| sp\|P51685\|CCR8_HUMAN C-C chemokine receptor type 8 OS=Homo sapiens GN=CCR8 PE=1 SV=1 | 340 | SCEKSSsCQQH | 0.344 | 0.095 | -0.653 | -0.071 |
| sp\|P51685\|CCR8_HUMAN C-C chemokine receptor type 8 OS=Homo sapiens GN=CCR8 PE=1 SV=1 | 345 | SSCQQHsSRSS | 0.072 | -0.202 | -1.46 | -0.53 |

| | | | | | | |
|---|---|---|---|---|---|---|
| sp\|P51685\|CCR8_HUMAN C-C chemokine receptor type 8 OS=Homo sapiens GN=CCR8 PE=1 SV=1 | 346 | SCQQHSsRSSS | 0.059 | -0.227 | -1.689 | -0.619 |
| sp\|P51685\|CCR8_HUMAN C-C chemokine receptor type 8 OS=Homo sapiens GN=CCR8 PE=1 SV=1 | 348 | QQHSSRsSSVD | 0.087 | -0.018 | -1.072 | -0.334 |
| sp\|P51685\|CCR8_HUMAN C-C chemokine receptor type 8 OS=Homo sapiens GN=CCR8 PE=1 SV=1 | 349 | QHSSRSsSVDY | 0.197 | -0.016 | -1.139 | -0.319 |
| sp\|P51685\|CCR8_HUMAN C-C chemokine receptor type 8 OS=Homo sapiens GN=CCR8 PE=1 SV=1 | 350 | HSSRSSsVDYI | 0.741 | 1.042 | 0.888 | 0.89 |
| sp\|P59551\|T2R60_HUMAN Taste receptor type 2 member 60 OS=Homo sapiens GN=TAS2R60 PE=2 SV=1 | 309 | LRAVLKsRRSS | 0.07 | -0.052 | -1.062 | -0.348 |
| sp\|P59551\|T2R60_HUMAN Taste receptor type 2 member 60 OS=Homo sapiens GN=TAS2R60 PE=2 SV=1 | 312 | VLKSRRsSRCG | 0.361 | 0.095 | -0.589 | -0.044 |
| sp\|P59551\|T2R60_HUMAN Taste receptor type 2 member 60 OS=Homo sapiens GN=TAS2R60 PE=2 SV=1 | 313 | LKSRRSsRCGT | 0.569 | 0.691 | 0.239 | 0.5 |
| sp\|P59551\|T2R60_HUMAN Taste receptor type 2 member 60 OS=Homo sapiens GN=TAS2R60 PE=2 SV=1 | 317 | RSSRCGtP--- | 0.429 | 0.598 | -0.256 | 0.257 |
| sp\|P51686\|CCR9_HUMAN C-C chemokine receptor type 9 OS=Homo sapiens GN=CCR9 PE=1 SV=2 | 331 | RRDLVKtLKNL | 0.54 | 0.414 | -0.118 | 0.279 |
| sp\|P51686\|CCR9_HUMAN C-C chemokine receptor type 9 OS=Homo sapiens GN=CCR9 PE=1 SV=2 | 339 | KNLGCIsQAQW | 0.125 | -0.258 | -1.063 | -0.399 |
| sp\|P51686\|CCR9_HUMAN C-C chemokine receptor type 9 OS=Homo sapiens GN=CCR9 PE=1 SV=2 | 345 | SQAQWVsFTRR | 0.433 | -0.06 | -0.287 | 0.029 |
| sp\|P51686\|CCR9_HUMAN C-C chemokine receptor type 9 OS=Homo sapiens GN=CCR9 PE=1 SV=2 | 347 | AQWVSFtRREG | 0.226 | 0.141 | -0.534 | -0.056 |
| sp\|P51686\|CCR9_HUMAN C-C chemokine receptor type 9 OS=Homo sapiens GN=CCR9 PE=1 SV=2 | 352 | FTRREGsLKLS | 0.794 | 1.096 | 0.571 | 0.82 |
| sp\|P51686\|CCR9_HUMAN C-C chemokine receptor type 9 OS=Homo sapiens GN=CCR9 PE=1 SV=2 | 356 | EGSLKLsSMLL | 0.051 | -0.179 | -1.326 | -0.485 |
| sp\|P51686\|CCR9_HUMAN C-C chemokine receptor type 9 OS=Homo sapiens GN=CCR9 PE=1 SV=2 | 357 | GSLKLSsMLLE | 0.134 | -0.064 | -1.205 | -0.378 |
| sp\|P51686\|CCR9_HUMAN C-C chemokine receptor type 9 OS=Homo sapiens GN=CCR9 PE=1 SV=2 | 362 | SSMLLEtTSGA | 0.043 | -0.369 | -1.766 | -0.697 |
| sp\|P51686\|CCR9_HUMAN C-C chemokine receptor type 9 OS=Homo sapiens GN=CCR9 PE=1 SV=2 | 363 | SMLLETtSGAL | 0.143 | 0.087 | -0.926 | -0.232 |
| sp\|P51686\|CCR9_HUMAN C-C chemokine receptor type 9 OS=Homo sapiens GN=CCR9 PE=1 SV=2 | 364 | MLLETTsGALS | 0.114 | 0.033 | -0.878 | -0.244 |
| sp\|P51686\|CCR9_HUMAN C-C chemokine receptor type 9 OS=Homo sapiens GN=CCR9 PE=1 SV=2 | 368 | TTSGALsL--- | 0.274 | 0.154 | -0.52 | -0.031 |
| sp\|P46092\|CCR10_HUMAN C-C chemokine receptor type 10 OS=Homo sapiens GN=CCR10 PE=1 SV=3 | 329 | RLLRGGsCPSG | 0.816 | 1.369 | 0.8 | 0.995 |
| sp\|P46092\|CCR10_HUMAN C-C chemokine receptor type 10 OS=Homo sapiens GN=CCR10 PE=1 SV=3 | 332 | RGGSCPsGPQP | 0.387 | 0.226 | -0.265 | 0.116 |
| sp\|P46092\|CCR10_HUMAN C-C chemokine receptor type 10 OS=Homo sapiens GN=CCR10 PE=1 SV=3 | 347 | PRRPRLsSCSA | 0.244 | 0.308 | -0.567 | -0.005 |
| sp\|P46092\|CCR10_HUMAN C-C chemokine receptor type 10 OS=Homo sapiens GN=CCR10 PE=1 SV=3 | 348 | RRPRLSsCSAP | 0.317 | 0.954 | -0.246 | 0.342 |
| sp\|P46092\|CCR10_HUMAN C-C chemokine receptor type 10 OS=Homo sapiens GN=CCR10 PE=1 SV=3 | 350 | PRLSSCsAPTE | 0.844 | 0.865 | 0.476 | 0.728 |
| sp\|P46092\|CCR10_HUMAN C-C chemokine receptor type 10 OS=Homo sapiens GN=CCR10 PE=1 SV=3 | 353 | SSCSAPtETHS | 0.131 | -0.221 | -1.181 | -0.424 |
| sp\|P46092\|CCR10_HUMAN C-C chemokine receptor type 10 OS=Homo sapiens GN=CCR10 PE=1 SV=3 | 355 | CSAPTEtHSLS | 0.08 | -0.201 | -1.183 | -0.435 |
| sp\|P46092\|CCR10_HUMAN C-C chemokine receptor type 10 OS=Homo sapiens GN=CCR10 PE=1 SV=3 | 357 | APTETHsLSWD | 0.197 | 0.109 | -0.609 | -0.101 |
| sp\|P46092\|CCR10_HUMAN C-C chemokine receptor type 10 OS=Homo sapiens GN=CCR10 PE=1 SV=3 | 359 | TETHSLsWDN- | 0.238 | 0.262 | -0.598 | -0.033 |
| sp\|P25024\|CXCR1_HUMAN C-X-C chemokine receptor type 1 OS=Homo sapiens GN=CXCR1 PE=1 SV=2 | 327 | AMHGLVsKEFL | 0.078 | -0.103 | -1.283 | -0.436 |
| sp\|P25024\|CXCR1_HUMAN C-X-C chemokine receptor type 1 OS=Homo sapiens GN=CXCR1 PE=1 SV=2 | 337 | LARHRVtSYTS | 0.475 | 0.212 | -0.276 | 0.137 |
| sp\|P25024\|CXCR1_HUMAN C-X-C chemokine receptor type 1 OS=Homo sapiens GN=CXCR1 PE=1 SV=2 | 338 | ARHRVTsYTSS | 0.372 | 0.848 | -0.265 | 0.318 |
| sp\|P25024\|CXCR1_HUMAN C-X-C chemokine receptor type 1 OS=Homo sapiens GN=CXCR1 PE=1 SV=2 | 340 | HRVTSYtSSSV | 0.16 | 0.375 | -0.721 | -0.062 |
| sp\|P25024\|CXCR1_HUMAN C-X-C chemokine receptor type 1 OS=Homo sapiens GN=CXCR1 PE=1 SV=2 | 341 | RVTSYTsSSVN | 0.122 | -0.081 | -1.096 | -0.352 |
| sp\|P25024\|CXCR1_HUMAN C-X-C chemokine receptor type 1 OS=Homo sapiens GN=CXCR1 PE=1 SV=2 | 342 | VTSYTSsSVNV | 0.077 | -0.131 | -1.569 | -0.541 |
| sp\|P25024\|CXCR1_HUMAN C-X-C chemokine receptor type 1 OS=Homo sapiens GN=CXCR1 PE=1 SV=2 | 343 | TSYTSSsVNVS | 0.164 | 0.042 | -0.689 | -0.161 |
| sp\|P25024\|CXCR1_HUMAN C-X-C chemokine receptor type 1 OS=Homo sapiens GN=CXCR1 PE=1 SV=2 | 347 | SSSVNVsSNL- | 0.143 | -0.175 | -1.02 | -0.351 |
| sp\|P25024\|CXCR1_HUMAN C-X-C chemokine receptor type 1 OS=Homo sapiens GN=CXCR1 PE=1 SV=2 | 348 | SSVNVSsNL-- | 0.037 | -0.188 | -1.792 | -0.648 |
| sp\|P25025\|CXCR2_HUMAN C-X-C chemokine receptor type 2 OS=Homo sapiens GN=CXCR2 PE=1 SV=2 | 336 | AIHGLIsKDSL | 0.101 | -0.063 | -1.04 | -0.334 |
| sp\|P25025\|CXCR2_HUMAN C-X-C chemokine receptor type 2 OS=Homo sapiens GN=CXCR2 PE=1 SV=2 | 339 | GLISKDsLPKD | 0.714 | 0.53 | 0.18 | 0.475 |
| sp\|P25025\|CXCR2_HUMAN C-X-C chemokine receptor type 2 OS=Homo sapiens GN=CXCR2 PE=1 SV=2 | 344 | DSLPKDsRPSF | 0.17 | 0.038 | -0.99 | -0.261 |
| sp\|P25025\|CXCR2_HUMAN C-X-C chemokine receptor type 2 OS=Homo sapiens GN=CXCR2 PE=1 SV=2 | 347 | PKDSRPsFVGS | 0.437 | 0.069 | -0.33 | 0.059 |
| sp\|P25025\|CXCR2_HUMAN C-X-C chemokine receptor type 2 OS=Homo sapiens GN=CXCR2 PE=1 SV=2 | 351 | RPSFVGsSSGH | 0.06 | -0.397 | -1.768 | -0.702 |
| sp\|P25025\|CXCR2_HUMAN C-X-C chemokine receptor type 2 OS=Homo sapiens GN=CXCR2 PE=1 SV=2 | 352 | PSFVGSsSGHT | 0.04 | -0.326 | -1.414 | -0.567 |
| sp\|P25025\|CXCR2_HUMAN C-X-C chemokine receptor type 2 OS=Homo sapiens GN=CXCR2 PE=1 SV=2 | 353 | SFVGSSsGHTS | 0.118 | -0.011 | -0.865 | -0.253 |
| sp\|P25025\|CXCR2_HUMAN C-X-C chemokine receptor type 2 OS=Homo sapiens GN=CXCR2 PE=1 SV=2 | 356 | GSSSGHtSTTL | 0.166 | -0.124 | -1.055 | -0.338 |
| sp\|P25025\|CXCR2_HUMAN C-X-C chemokine receptor type 2 OS=Homo sapiens GN=CXCR2 PE=1 SV=2 | 357 | SSSGHTsTTL- | 0.142 | -0.128 | -1.127 | -0.371 |
| sp\|P25025\|CXCR2_HUMAN C-X-C chemokine receptor type 2 OS=Homo sapiens GN=CXCR2 PE=1 SV=2 | 358 | SSGHTStTL-- | 0.033 | -0.166 | -1.81 | -0.648 |
| sp\|P25025\|CXCR2_HUMAN C-X-C chemokine receptor type 2 OS=Homo sapiens GN=CXCR2 PE=1 SV=2 | 359 | SGHTSTtL--- | 0.331 | 0.292 | -0.333 | 0.097 |
| sp\|P49682\|CXCR3_HUMAN C-X-C chemokine receptor type 3 OS=Homo sapiens GN=CXCR3 PE=1 SV=2 | 349 | GLQRQPsSSRR | 0.584 | 0.795 | -0.056 | 0.441 |

| | | | | | | |
|---|---|---|---|---|---|---|
| sp\|P49682\|CXCR3_HUMAN C-X-C chemokine receptor type 3 OS=Homo sapiens GN=CXCR3 PE=1 SV=2 | 350 | LQRQPSsSRRD | 0.211 | -0.109 | -0.557 | -0.152 |
| sp\|P49682\|CXCR3_HUMAN C-X-C chemokine receptor type 3 OS=Homo sapiens GN=CXCR3 PE=1 SV=2 | 351 | QRQPSSsRRDS | 0.289 | 0.212 | -0.467 | 0.011 |
| sp\|P49682\|CXCR3_HUMAN C-X-C chemokine receptor type 3 OS=Homo sapiens GN=CXCR3 PE=1 SV=2 | 355 | SSSRRDsSWSE | 0.454 | 0.685 | -0.341 | 0.266 |
| sp\|P49682\|CXCR3_HUMAN C-X-C chemokine receptor type 3 OS=Homo sapiens GN=CXCR3 PE=1 SV=2 | 356 | SSRRDSsWSET | 0.405 | 0.85 | -0.114 | 0.38 |
| sp\|P49682\|CXCR3_HUMAN C-X-C chemokine receptor type 3 OS=Homo sapiens GN=CXCR3 PE=1 SV=2 | 358 | RRDSSWsETSE | 0.374 | 0.298 | -0.471 | 0.067 |
| sp\|P49682\|CXCR3_HUMAN C-X-C chemokine receptor type 3 OS=Homo sapiens GN=CXCR3 PE=1 SV=2 | 360 | DSSWSEtSEAS | 0.29 | 0 | -0.674 | -0.128 |
| sp\|P49682\|CXCR3_HUMAN C-X-C chemokine receptor type 3 OS=Homo sapiens GN=CXCR3 PE=1 SV=2 | 361 | SSWSETsEASY | 0.058 | -0.134 | -1.535 | -0.537 |
| sp\|P49682\|CXCR3_HUMAN C-X-C chemokine receptor type 3 OS=Homo sapiens GN=CXCR3 PE=1 SV=2 | 364 | SETSEAsYSGL | 0.067 | -0.294 | -1.551 | -0.593 |
| sp\|P49682\|CXCR3_HUMAN C-X-C chemokine receptor type 3 OS=Homo sapiens GN=CXCR3 PE=1 SV=2 | 366 | TSEASYsGL-- | 0.195 | 0.169 | -0.494 | -0.043 |
| sp\|P61073\|CXCR4_HUMAN C-X-C chemokine receptor type 4 OS=Homo sapiens GN=CXCR4 PE=1 SV=1 | 311 | LGAKFKtSAQH | 0.197 | -0.204 | -0.709 | -0.239 |
| sp\|P61073\|CXCR4_HUMAN C-X-C chemokine receptor type 4 OS=Homo sapiens GN=CXCR4 PE=1 SV=1 | 312 | GAKFKTsAQHA | 0.232 | -0.061 | -0.677 | -0.169 |
| sp\|P61073\|CXCR4_HUMAN C-X-C chemokine receptor type 4 OS=Homo sapiens GN=CXCR4 PE=1 SV=1 | 318 | SAQHALtSVSR | 0.063 | -0.162 | -1.409 | -0.503 |
| sp\|P61073\|CXCR4_HUMAN C-X-C chemokine receptor type 4 OS=Homo sapiens GN=CXCR4 PE=1 SV=1 | 319 | AQHALTsVSRG | 0.062 | -0.33 | -1.507 | -0.592 |
| sp\|P61073\|CXCR4_HUMAN C-X-C chemokine receptor type 4 OS=Homo sapiens GN=CXCR4 PE=1 SV=1 | 321 | HALTSVsRGSS | 0.299 | 0.072 | -0.483 | -0.037 |
| sp\|P61073\|CXCR4_HUMAN C-X-C chemokine receptor type 4 OS=Homo sapiens GN=CXCR4 PE=1 SV=1 | 324 | TSVSRGsSLKI | 0.101 | -0.16 | -1.268 | -0.442 |
| sp\|P61073\|CXCR4_HUMAN C-X-C chemokine receptor type 4 OS=Homo sapiens GN=CXCR4 PE=1 SV=1 | 325 | SVSRGSsLKIL | 0.38 | 0.733 | 0.017 | 0.377 |
| sp\|P61073\|CXCR4_HUMAN C-X-C chemokine receptor type 4 OS=Homo sapiens GN=CXCR4 PE=1 SV=1 | 330 | SSLKILsKGKR | 0.172 | -0.123 | -0.684 | -0.212 |
| sp\|P61073\|CXCR4_HUMAN C-X-C chemokine receptor type 4 OS=Homo sapiens GN=CXCR4 PE=1 SV=1 | 338 | GKRGGHsSVST | 0.288 | 0.255 | -0.504 | 0.013 |
| sp\|P61073\|CXCR4_HUMAN C-X-C chemokine receptor type 4 OS=Homo sapiens GN=CXCR4 PE=1 SV=1 | 339 | KRGGHSsVSTE | 0.076 | -0.003 | -1.214 | -0.38 |
| sp\|P61073\|CXCR4_HUMAN C-X-C chemokine receptor type 4 OS=Homo sapiens GN=CXCR4 PE=1 SV=1 | 341 | GGHSSVsTESE | 0.229 | 0.002 | -0.742 | -0.17 |
| sp\|P61073\|CXCR4_HUMAN C-X-C chemokine receptor type 4 OS=Homo sapiens GN=CXCR4 PE=1 SV=1 | 342 | GHSSVStESES | 0.091 | -0.166 | -1.577 | -0.551 |
| sp\|P61073\|CXCR4_HUMAN C-X-C chemokine receptor type 4 OS=Homo sapiens GN=CXCR4 PE=1 SV=1 | 344 | SSVSTEsESSS | 0.025 | -0.302 | -2.011 | -0.763 |
| sp\|P61073\|CXCR4_HUMAN C-X-C chemokine receptor type 4 OS=Homo sapiens GN=CXCR4 PE=1 SV=1 | 346 | VSTESEsSSFH | 0.056 | -0.28 | -1.62 | -0.615 |
| sp\|P61073\|CXCR4_HUMAN C-X-C chemokine receptor type 4 OS=Homo sapiens GN=CXCR4 PE=1 SV=1 | 347 | STESESsSFHS | 0.044 | -0.422 | -1.785 | -0.721 |
| sp\|P61073\|CXCR4_HUMAN C-X-C chemokine receptor type 4 OS=Homo sapiens GN=CXCR4 PE=1 SV=1 | 348 | TESESSsFHSS | 0.11 | 0.025 | -1.034 | -0.3 |
| sp\|P61073\|CXCR4_HUMAN C-X-C chemokine receptor type 4 OS=Homo sapiens GN=CXCR4 PE=1 SV=1 | 351 | ESSSFHsS--- | 0.14 | 0.017 | -1.116 | -0.32 |
| sp\|P61073\|CXCR4_HUMAN C-X-C chemokine receptor type 4 OS=Homo sapiens GN=CXCR4 PE=1 SV=1 | 352 | SSSFHSs---- | 0.115 | -0.043 | -1.244 | -0.391 |
| sp\|P32302\|CXCR5_HUMAN C-X-C chemokine receptor type 5 OS=Homo sapiens GN=CXCR5 PE=1 SV=1 | 331 | AGVKFRsDLSR | 0.127 | -0.106 | -1.347 | -0.442 |
| sp\|P32302\|CXCR5_HUMAN C-X-C chemokine receptor type 5 OS=Homo sapiens GN=CXCR5 PE=1 SV=1 | 334 | KFRSDLsRLLT | 0.109 | -0.089 | -1.12 | -0.367 |
| sp\|P32302\|CXCR5_HUMAN C-X-C chemokine receptor type 5 OS=Homo sapiens GN=CXCR5 PE=1 SV=1 | 338 | DLSRLLtKLGC | 0.404 | 0.65 | -0.298 | 0.252 |
| sp\|P32302\|CXCR5_HUMAN C-X-C chemokine receptor type 5 OS=Homo sapiens GN=CXCR5 PE=1 SV=1 | 343 | LTKLGCtGPAS | 0.529 | 0.41 | -0.141 | 0.266 |
| sp\|P32302\|CXCR5_HUMAN C-X-C chemokine receptor type 5 OS=Homo sapiens GN=CXCR5 PE=1 SV=1 | 347 | GCTGPAsLCQL | 0.411 | 0.033 | -0.287 | 0.052 |
| sp\|P32302\|CXCR5_HUMAN C-X-C chemokine receptor type 5 OS=Homo sapiens GN=CXCR5 PE=1 SV=1 | 354 | LCQLFPsWRRS | 0.169 | -0.102 | -1.229 | -0.387 |
| sp\|P32302\|CXCR5_HUMAN C-X-C chemokine receptor type 5 OS=Homo sapiens GN=CXCR5 PE=1 SV=1 | 358 | FPSWRRsSLSE | 0.104 | -0.07 | -1.269 | -0.412 |
| sp\|P32302\|CXCR5_HUMAN C-X-C chemokine receptor type 5 OS=Homo sapiens GN=CXCR5 PE=1 SV=1 | 359 | PSWRRSsLSES | 0.677 | 1.032 | 0.496 | 0.735 |
| sp\|P32302\|CXCR5_HUMAN C-X-C chemokine receptor type 5 OS=Homo sapiens GN=CXCR5 PE=1 SV=1 | 361 | WRRSSLsESEN | 0.501 | 0.577 | -0.009 | 0.356 |
| sp\|P32302\|CXCR5_HUMAN C-X-C chemokine receptor type 5 OS=Homo sapiens GN=CXCR5 PE=1 SV=1 | 363 | RSSLSEsENAT | 0.418 | 0.211 | -0.197 | 0.144 |
| sp\|P32302\|CXCR5_HUMAN C-X-C chemokine receptor type 5 OS=Homo sapiens GN=CXCR5 PE=1 SV=1 | 367 | SESENAtSLTT | 0.052 | -0.291 | -1.661 | -0.633 |
| sp\|P32302\|CXCR5_HUMAN C-X-C chemokine receptor type 5 OS=Homo sapiens GN=CXCR5 PE=1 SV=1 | 368 | ESENATsLTTF | 0.299 | -0.025 | -0.244 | 0.01 |
| sp\|P32302\|CXCR5_HUMAN C-X-C chemokine receptor type 5 OS=Homo sapiens GN=CXCR5 PE=1 SV=1 | 370 | ENATSLtTF-- | 0.154 | 0.138 | -0.598 | -0.102 |
| sp\|P32302\|CXCR5_HUMAN C-X-C chemokine receptor type 5 OS=Homo sapiens GN=CXCR5 PE=1 SV=1 | 371 | NATSLTtF--- | 0.182 | 0.047 | -0.796 | -0.189 |
| sp\|O00574\|CXCR6_HUMAN C-X-C chemokine receptor type 6 OS=Homo sapiens GN=CXCR6 PE=2 SV=1 | 292 | VLYAFVsLKFR | 0.415 | -0.012 | -0.296 | 0.036 |
| sp\|O00574\|CXCR6_HUMAN C-X-C chemokine receptor type 6 OS=Homo sapiens GN=CXCR6 PE=2 SV=1 | 315 | LPYLGVsHQWK | 0.163 | -0.158 | -0.813 | -0.269 |
| sp\|O00574\|CXCR6_HUMAN C-X-C chemokine receptor type 6 OS=Homo sapiens GN=CXCR6 PE=2 SV=1 | 320 | VSHQWKsSEDN | 0.258 | -0.031 | -0.544 | -0.106 |
| sp\|O00574\|CXCR6_HUMAN C-X-C chemokine receptor type 6 OS=Homo sapiens GN=CXCR6 PE=2 SV=1 | 321 | SHQWKSsEDNS | 0.094 | -0.068 | -1.47 | -0.481 |
| sp\|O00574\|CXCR6_HUMAN C-X-C chemokine receptor type 6 OS=Homo sapiens GN=CXCR6 PE=2 SV=1 | 325 | KSSEDNsKTFS | 0.092 | -0.3 | -1.477 | -0.562 |
| sp\|O00574\|CXCR6_HUMAN C-X-C chemokine receptor type 6 OS=Homo sapiens GN=CXCR6 PE=2 SV=1 | 327 | SEDNSKtFSAS | 0.257 | 0.18 | -0.347 | 0.03 |
| sp\|O00574\|CXCR6_HUMAN C-X-C chemokine receptor type 6 OS=Homo sapiens GN=CXCR6 PE=2 SV=1 | 329 | DNSKTFsASHN | 0.144 | -0.098 | -1.052 | -0.335 |
| sp\|O00574\|CXCR6_HUMAN C-X-C chemokine receptor type 6 OS=Homo sapiens GN=CXCR6 PE=2 SV=1 | 331 | SKTFSAsHNVE | 0.637 | 0.306 | 0.084 | 0.342 |

| | | | | | | |
|---|---|---|---|---|---|---|
| sp\|O00574\|CXCR6_HUMAN C-X-C chemokine receptor type 6 OS=Homo sapiens GN=CXCR6 PE=2 SV=1 | 337 | SHNVEAtSMFQ | 0.042 | -0.319 | -2.244 | -0.84 |
| sp\|O00574\|CXCR6_HUMAN C-X-C chemokine receptor type 6 OS=Homo sapiens GN=CXCR6 PE=2 SV=1 | 338 | HNVEATsMFQL | 0.163 | 0.124 | -0.605 | -0.106 |
| sp\|P49238\|CX3C1_HUMAN CX3C chemokine receptor 1 OS=Homo sapiens GN=CX3CR1 PE=1 SV=1 | 319 | AVLCGRsVHVD | 0.146 | -0.069 | -1.1 | -0.341 |
| sp\|P49238\|CX3C1_HUMAN CX3C chemokine receptor 1 OS=Homo sapiens GN=CX3CR1 PE=1 SV=1 | 325 | SVHVDFsSSES | 0.028 | -0.268 | -1.854 | -0.698 |
| sp\|P49238\|CX3C1_HUMAN CX3C chemokine receptor 1 OS=Homo sapiens GN=CX3CR1 PE=1 SV=1 | 326 | VHVDFSsSESQ | 0.036 | -0.319 | -2.165 | -0.816 |
| sp\|P49238\|CX3C1_HUMAN CX3C chemokine receptor 1 OS=Homo sapiens GN=CX3CR1 PE=1 SV=1 | 327 | HVDFSSsESQR | 0.202 | 0.172 | -0.453 | -0.026 |
| sp\|P49238\|CX3C1_HUMAN CX3C chemokine receptor 1 OS=Homo sapiens GN=CX3CR1 PE=1 SV=1 | 329 | DFSSSEsQRSR | 0.217 | -0.093 | -0.956 | -0.277 |
| sp\|P49238\|CX3C1_HUMAN CX3C chemokine receptor 1 OS=Homo sapiens GN=CX3CR1 PE=1 SV=1 | 332 | SSESQRsRHGS | 0.048 | -0.401 | -1.866 | -0.74 |
| sp\|P49238\|CX3C1_HUMAN CX3C chemokine receptor 1 OS=Homo sapiens GN=CX3CR1 PE=1 SV=1 | 336 | QRSRHGsVLSS | 0.312 | 0.902 | -0.249 | 0.322 |
| sp\|P49238\|CX3C1_HUMAN CX3C chemokine receptor 1 OS=Homo sapiens GN=CX3CR1 PE=1 SV=1 | 339 | RHGSVLsSNFT | 0.05 | -0.279 | -1.734 | -0.654 |
| sp\|P49238\|CX3C1_HUMAN CX3C chemokine receptor 1 OS=Homo sapiens GN=CX3CR1 PE=1 SV=1 | 340 | HGSVLSsNFTY | 0.067 | -0.192 | -1.348 | -0.491 |
| sp\|P49238\|CX3C1_HUMAN CX3C chemokine receptor 1 OS=Homo sapiens GN=CX3CR1 PE=1 SV=1 | 343 | VLSSNFtYHTS | 0.195 | -0.104 | -0.766 | -0.225 |
| sp\|P49238\|CX3C1_HUMAN CX3C chemokine receptor 1 OS=Homo sapiens GN=CX3CR1 PE=1 SV=1 | 346 | SNFTYHtSDGD | 0.22 | -0.059 | -0.77 | -0.203 |
| sp\|P49238\|CX3C1_HUMAN CX3C chemokine receptor 1 OS=Homo sapiens GN=CX3CR1 PE=1 SV=1 | 347 | NFTYHTsDGDA | 0.6 | 0.218 | 0.177 | 0.332 |
| sp\|P46094\|XCR1_HUMAN Chemokine XC receptor 1 OS=Homo sapiens GN=XCR1 PE=1 SV=1 | 296 | VGVKFRtHLKH | 0.099 | -0.241 | -1.536 | -0.559 |
| sp\|P46094\|XCR1_HUMAN Chemokine XC receptor 1 OS=Homo sapiens GN=XCR1 PE=1 SV=1 | 314 | CRLQAPsPASI | 0.181 | -0.094 | -0.593 | -0.169 |
| sp\|P46094\|XCR1_HUMAN Chemokine XC receptor 1 OS=Homo sapiens GN=XCR1 PE=1 SV=1 | 317 | QAPSPAsIPHS | 0.165 | 0.096 | -0.661 | -0.133 |
| sp\|P46094\|XCR1_HUMAN Chemokine XC receptor 1 OS=Homo sapiens GN=XCR1 PE=1 SV=1 | 321 | PASIPHsPGAF | 0.134 | -0.225 | -0.849 | -0.313 |
| sp\|P46094\|XCR1_HUMAN Chemokine XC receptor 1 OS=Homo sapiens GN=XCR1 PE=1 SV=1 | 331 | FAYEGAsFY-- | 0.125 | 0.149 | -0.939 | -0.222 |
| sp\|Q16570\|ACKR1_HUMAN Atypical chemokine receptor 1 OS=Homo sapiens GN=ACKR1 PE=1 SV=3 | 312 | LFCHQAtRTLL | 0.214 | -0.053 | -0.866 | -0.235 |
| sp\|Q16570\|ACKR1_HUMAN Atypical chemokine receptor 1 OS=Homo sapiens GN=ACKR1 PE=1 SV=3 | 314 | CHQATRtLLPS | 0.125 | 0.011 | -1.145 | -0.336 |
| sp\|Q16570\|ACKR1_HUMAN Atypical chemokine receptor 1 OS=Homo sapiens GN=ACKR1 PE=1 SV=3 | 318 | TRTLLPsLPLP | 0.544 | 0.604 | 0.06 | 0.403 |
| sp\|Q16570\|ACKR1_HUMAN Atypical chemokine receptor 1 OS=Homo sapiens GN=ACKR1 PE=1 SV=3 | 326 | PLPEGWsSHLD | 0.153 | -0.081 | -1.016 | -0.315 |
| sp\|Q16570\|ACKR1_HUMAN Atypical chemokine receptor 1 OS=Homo sapiens GN=ACKR1 PE=1 SV=3 | 327 | LPEGWSsHLDT | 0.125 | -0.14 | -0.908 | -0.308 |
| sp\|Q16570\|ACKR1_HUMAN Atypical chemokine receptor 1 OS=Homo sapiens GN=ACKR1 PE=1 SV=3 | 331 | WSSHLDtLGSK | 0.139 | -0.264 | -0.926 | -0.35 |
| sp\|Q16570\|ACKR1_HUMAN Atypical chemokine receptor 1 OS=Homo sapiens GN=ACKR1 PE=1 SV=3 | 334 | HLDTLGsKS-- | 0.086 | -0.003 | -1.234 | -0.384 |
| sp\|Q16570\|ACKR1_HUMAN Atypical chemokine receptor 1 OS=Homo sapiens GN=ACKR1 PE=1 SV=3 | 336 | DTLGSKs---- | 0.533 | 0.347 | -0.081 | 0.266 |
| sp\|O00590\|ACKR2_HUMAN Atypical chemokine receptor 2 OS=Homo sapiens GN=ACKR2 PE=1 SV=2 | 316 | ILYAFSsHRFR | 0.195 | -0.186 | -0.815 | -0.269 |
| sp\|O00590\|ACKR2_HUMAN Atypical chemokine receptor 2 OS=Homo sapiens GN=ACKR2 PE=1 SV=2 | 339 | WHLAPGtAQAS | 0.101 | -0.183 | -1.183 | -0.422 |
| sp\|O00590\|ACKR2_HUMAN Atypical chemokine receptor 2 OS=Homo sapiens GN=ACKR2 PE=1 SV=2 | 343 | PGTAQAsLSSC | 0.122 | -0.123 | -1.019 | -0.34 |
| sp\|O00590\|ACKR2_HUMAN Atypical chemokine receptor 2 OS=Homo sapiens GN=ACKR2 PE=1 SV=2 | 345 | TAQASLsSCSE | 0.081 | -0.14 | -1.229 | -0.429 |
| sp\|O00590\|ACKR2_HUMAN Atypical chemokine receptor 2 OS=Homo sapiens GN=ACKR2 PE=1 SV=2 | 346 | AQASLSsCSES | 0.044 | -0.261 | -1.55 | -0.589 |
| sp\|O00590\|ACKR2_HUMAN Atypical chemokine receptor 2 OS=Homo sapiens GN=ACKR2 PE=1 SV=2 | 348 | ASLSSCsESSI | 0.276 | 0.066 | -0.616 | -0.091 |
| sp\|O00590\|ACKR2_HUMAN Atypical chemokine receptor 2 OS=Homo sapiens GN=ACKR2 PE=1 SV=2 | 350 | LSSCSEsSILT | 0.164 | -0.049 | -0.774 | -0.22 |
| sp\|O00590\|ACKR2_HUMAN Atypical chemokine receptor 2 OS=Homo sapiens GN=ACKR2 PE=1 SV=2 | 351 | SSCSESsILTA | 0.035 | -0.447 | -1.835 | -0.749 |
| sp\|O00590\|ACKR2_HUMAN Atypical chemokine receptor 2 OS=Homo sapiens GN=ACKR2 PE=1 SV=2 | 354 | SESSILtAQEE | 0.083 | -0.222 | -1.417 | -0.519 |
| sp\|O00590\|ACKR2_HUMAN Atypical chemokine receptor 2 OS=Homo sapiens GN=ACKR2 PE=1 SV=2 | 360 | TAQEEMtGMND | 0.169 | 0.028 | -1.075 | -0.293 |
| sp\|O00590\|ACKR2_HUMAN Atypical chemokine receptor 2 OS=Homo sapiens GN=ACKR2 PE=1 SV=2 | 370 | DLGERQsENYP | 0.301 | 0.177 | -0.595 | -0.039 |
| sp\|O00590\|ACKR2_HUMAN Atypical chemokine receptor 2 OS=Homo sapiens GN=ACKR2 PE=1 SV=2 | 383 | EDVGNKsA--- | 0.156 | 0.102 | -0.728 | -0.157 |
| sp\|P25106\|ACKR3_HUMAN Atypical chemokine receptor 3 OS=Homo sapiens GN=ACKR3 PE=1 SV=3 | 335 | AFIFKYsAKTG | 0.182 | 0.028 | -0.766 | -0.185 |
| sp\|P25106\|ACKR3_HUMAN Atypical chemokine receptor 3 OS=Homo sapiens GN=ACKR3 PE=1 SV=3 | 338 | FKYSAKtGLTK | 0.342 | 0.042 | -0.348 | 0.012 |
| sp\|P25106\|ACKR3_HUMAN Atypical chemokine receptor 3 OS=Homo sapiens GN=ACKR3 PE=1 SV=3 | 341 | SAKTGLtKLID | 0.073 | -0.258 | -1.356 | -0.514 |
| sp\|P25106\|ACKR3_HUMAN Atypical chemokine receptor 3 OS=Homo sapiens GN=ACKR3 PE=1 SV=3 | 347 | TKLIDAsRVSE | 0.136 | -0.154 | -1.113 | -0.377 |
| sp\|P25106\|ACKR3_HUMAN Atypical chemokine receptor 3 OS=Homo sapiens GN=ACKR3 PE=1 SV=3 | 350 | IDASRVsETEY | 0.607 | 0.089 | -0.179 | 0.172 |
| sp\|P25106\|ACKR3_HUMAN Atypical chemokine receptor 3 OS=Homo sapiens GN=ACKR3 PE=1 SV=3 | 352 | ASRVSEtEYSA | 0.3 | 0.273 | -0.49 | 0.028 |
| sp\|P25106\|ACKR3_HUMAN Atypical chemokine receptor 3 OS=Homo sapiens GN=ACKR3 PE=1 SV=3 | 355 | VSETEYsALEQ | 0.129 | -0.153 | -1.26 | -0.428 |
| sp\|P25106\|ACKR3_HUMAN Atypical chemokine receptor 3 OS=Homo sapiens GN=ACKR3 PE=1 SV=3 | 360 | YSALEQsTK-- | 0.119 | -0.031 | -1.004 | -0.305 |
| sp\|P25106\|ACKR3_HUMAN Atypical chemokine receptor 3 OS=Homo sapiens GN=ACKR3 PE=1 SV=3 | 361 | SALEQStK--- | 0.043 | -0.112 | -1.626 | -0.565 |
| sp\|Q9NPB9\|ACKR4_HUMAN Atypical chemokine receptor 4 OS=Homo sapiens GN=ACKR4 PE=1 SV=1 | 309 | YVFMGAsFKNY | 0.239 | 0.049 | -0.723 | -0.145 |

| | | | | | | |
|---|---|---|---|---|---|---|
| sp\|Q9NPB9\|ACKR4_HUMAN Atypical chemokine receptor 4 OS=Homo sapiens GN=ACKR4 PE=1 SV=1 | 323 | VAKKYGsWRRQ | 0.235 | -0.193 | -0.983 | -0.314 |
| sp\|Q9NPB9\|ACKR4_HUMAN Atypical chemokine receptor 4 OS=Homo sapiens GN=ACKR4 PE=1 SV=1 | 330 | WRRQRQsVEEF | 0.633 | 0.584 | 0.344 | 0.52 |
| sp\|Q9NPB9\|ACKR4_HUMAN Atypical chemokine receptor 4 OS=Homo sapiens GN=ACKR4 PE=1 SV=1 | 338 | EEFPFDsEGPT | 0.113 | -0.198 | -1.208 | -0.431 |
| sp\|Q9NPB9\|ACKR4_HUMAN Atypical chemokine receptor 4 OS=Homo sapiens GN=ACKR4 PE=1 SV=1 | 342 | FDSEGPtEPTS | 0.197 | 0.288 | -0.66 | -0.058 |
| sp\|Q9NPB9\|ACKR4_HUMAN Atypical chemokine receptor 4 OS=Homo sapiens GN=ACKR4 PE=1 SV=1 | 345 | EGPTEPtSTFS | 0.045 | -0.415 | -1.788 | -0.719 |
| sp\|Q9NPB9\|ACKR4_HUMAN Atypical chemokine receptor 4 OS=Homo sapiens GN=ACKR4 PE=1 SV=1 | 346 | GPTEPTsTFSI | 0.111 | -0.13 | -0.891 | -0.303 |
| sp\|Q9NPB9\|ACKR4_HUMAN Atypical chemokine receptor 4 OS=Homo sapiens GN=ACKR4 PE=1 SV=1 | 347 | PTEPTStFSI- | 0.123 | -0.109 | -1.043 | -0.343 |
| sp\|Q9NPB9\|ACKR4_HUMAN Atypical chemokine receptor 4 OS=Homo sapiens GN=ACKR4 PE=1 SV=1 | 349 | EPTSTFsI--- | 0.074 | 0.037 | -0.993 | -0.294 |
| sp\|O00421\|CCRL2_HUMAN C-C chemokine receptor-like 2 OS=Homo sapiens GN=CCRL2 PE=1 SV=2 | 306 | YAFLDGtFSKY | 0.112 | -0.161 | -1.297 | -0.449 |
| sp\|O00421\|CCRL2_HUMAN C-C chemokine receptor-like 2 OS=Homo sapiens GN=CCRL2 PE=1 SV=2 | 308 | FLDGTFsKYLC | 0.162 | 0.124 | -0.744 | -0.153 |
| sp\|O00421\|CCRL2_HUMAN C-C chemokine receptor-like 2 OS=Homo sapiens GN=CCRL2 PE=1 SV=2 | 319 | RCFHLRsNTPL | 0.157 | -0.088 | -1.398 | -0.443 |
| sp\|O00421\|CCRL2_HUMAN C-C chemokine receptor-like 2 OS=Homo sapiens GN=CCRL2 PE=1 SV=2 | 321 | FHLRSNtPLQP | 0.53 | 0.774 | -0.039 | 0.422 |
| sp\|O00421\|CCRL2_HUMAN C-C chemokine receptor-like 2 OS=Homo sapiens GN=CCRL2 PE=1 SV=2 | 329 | LQPRGQsAQGT | 0.298 | 0.661 | -0.005 | 0.318 |
| sp\|O00421\|CCRL2_HUMAN C-C chemokine receptor-like 2 OS=Homo sapiens GN=CCRL2 PE=1 SV=2 | 333 | GQSAQGtSREE | 0.096 | -0.289 | -1.332 | -0.508 |
| sp\|O00421\|CCRL2_HUMAN C-C chemokine receptor-like 2 OS=Homo sapiens GN=CCRL2 PE=1 SV=2 | 334 | QSAQGTsREEP | 0.074 | -0.321 | -1.153 | -0.467 |
| sp\|O00421\|CCRL2_HUMAN C-C chemokine receptor-like 2 OS=Homo sapiens GN=CCRL2 PE=1 SV=2 | 341 | REEPDHsTEV- | 0.191 | 0.03 | -0.995 | -0.258 |
| sp\|O00421\|CCRL2_HUMAN C-C chemokine receptor-like 2 OS=Homo sapiens GN=CCRL2 PE=1 SV=2 | 342 | EEPDHStEV-- | 0.091 | 0.062 | -1.342 | -0.396 |
| sp\|P23945\|FSHR_HUMAN Follicle-stimulating hormone receptor OS=Homo sapiens GN=FSHR PE=1 SV=3 | 630 | FLYAIFtKNFR | 0.301 | 0.109 | -0.139 | 0.09 |
| sp\|P23945\|FSHR_HUMAN Follicle-stimulating hormone receptor OS=Homo sapiens GN=FSHR PE=1 SV=3 | 642 | DFFILLsKCGC | 0.072 | -0.306 | -1.481 | -0.572 |
| sp\|P23945\|FSHR_HUMAN Follicle-stimulating hormone receptor OS=Homo sapiens GN=FSHR PE=1 SV=3 | 656 | QAQIYRtETSS | 0.09 | -0.047 | -1.307 | -0.421 |
| sp\|P23945\|FSHR_HUMAN Follicle-stimulating hormone receptor OS=Homo sapiens GN=FSHR PE=1 SV=3 | 658 | QIYRTEtSSTV | 0.276 | 0.749 | -0.512 | 0.171 |
| sp\|P23945\|FSHR_HUMAN Follicle-stimulating hormone receptor OS=Homo sapiens GN=FSHR PE=1 SV=3 | 659 | IYRTETsSTVH | 0.371 | 0.047 | -0.815 | -0.132 |
| sp\|P23945\|FSHR_HUMAN Follicle-stimulating hormone receptor OS=Homo sapiens GN=FSHR PE=1 SV=3 | 660 | YRTETSsTVHN | 0.057 | 0.081 | -1.148 | -0.337 |
| sp\|P23945\|FSHR_HUMAN Follicle-stimulating hormone receptor OS=Homo sapiens GN=FSHR PE=1 SV=3 | 661 | RTETSStVHNT | 0.335 | 0.123 | -0.645 | -0.062 |
| sp\|P23945\|FSHR_HUMAN Follicle-stimulating hormone receptor OS=Homo sapiens GN=FSHR PE=1 SV=3 | 665 | SSTVHNtHPRN | 0.562 | 0.381 | -0.402 | 0.18 |
| sp\|P23945\|FSHR_HUMAN Follicle-stimulating hormone receptor OS=Homo sapiens GN=FSHR PE=1 SV=3 | 673 | PRNGHCsSAPR | 0.235 | 0.117 | -0.885 | -0.178 |
| sp\|P23945\|FSHR_HUMAN Follicle-stimulating hormone receptor OS=Homo sapiens GN=FSHR PE=1 SV=3 | 674 | RNGHCSsAPRV | 0.432 | 0.297 | -0.643 | 0.029 |
| sp\|P23945\|FSHR_HUMAN Follicle-stimulating hormone receptor OS=Homo sapiens GN=FSHR PE=1 SV=3 | 679 | SSAPRVtNGST | 0.176 | -0.142 | -0.894 | -0.287 |
| sp\|P23945\|FSHR_HUMAN Follicle-stimulating hormone receptor OS=Homo sapiens GN=FSHR PE=1 SV=3 | 682 | PRVTNGsTYIL | 0.211 | 0.133 | -0.792 | -0.149 |
| sp\|P23945\|FSHR_HUMAN Follicle-stimulating hormone receptor OS=Homo sapiens GN=FSHR PE=1 SV=3 | 683 | RVTNGStYILV | 0.047 | -0.161 | -1.316 | -0.477 |
| sp\|P23945\|FSHR_HUMAN Follicle-stimulating hormone receptor OS=Homo sapiens GN=FSHR PE=1 SV=3 | 690 | YILVPLsHLAQ | 0.076 | -0.106 | -1.029 | -0.353 |
| sp\|Q9HBW0\|LPAR2_HUMAN Lysophosphatidic acid receptor 2 OS=Homo sapiens GN=LPAR2 PE=1 SV=2 | 349 | GHPLMDsTL-- | 0.088 | -0.127 | -1.754 | -0.598 |
| sp\|Q9HBW0\|LPAR2_HUMAN Lysophosphatidic acid receptor 2 OS=Homo sapiens GN=LPAR2 PE=1 SV=2 | 350 | HPLMDStL--- | 0.194 | 0.077 | -0.615 | -0.115 |
| sp\|P22888\|LSHR_HUMAN Lutropin-choriogonadotropic hormone receptor OS=Homo sapiens GN=LHCGR PE= | 627 | FLYAIFtKTFQ | 0.244 | 0.006 | -0.529 | -0.093 |
| sp\|P22888\|LSHR_HUMAN Lutropin-choriogonadotropic hormone receptor OS=Homo sapiens GN=LHCGR PE= | 629 | YAIFTKtFQRD | 0.373 | 0.109 | -0.296 | 0.062 |
| sp\|P22888\|LSHR_HUMAN Lutropin-choriogonadotropic hormone receptor OS=Homo sapiens GN=LHCGR PE= | 639 | DFFLLLsKFGC | 0.116 | -0.213 | -1.216 | -0.438 |
| sp\|P22888\|LSHR_HUMAN Lutropin-choriogonadotropic hormone receptor OS=Homo sapiens GN=LHCGR PE= | 657 | YRRKDFsAYTS | 0.536 | 0.504 | -0.11 | 0.31 |
| sp\|P22888\|LSHR_HUMAN Lutropin-choriogonadotropic hormone receptor OS=Homo sapiens GN=LHCGR PE= | 660 | KDFSAYtSNCK | 0.149 | -0.062 | -0.848 | -0.254 |
| sp\|P22888\|LSHR_HUMAN Lutropin-choriogonadotropic hormone receptor OS=Homo sapiens GN=LHCGR PE= | 661 | DFSAYTsNCKN | 0.166 | -0.215 | -1.183 | -0.411 |
| sp\|P22888\|LSHR_HUMAN Lutropin-choriogonadotropic hormone receptor OS=Homo sapiens GN=LHCGR PE= | 668 | NCKNGFtGSNK | 0.105 | -0.104 | -1.031 | -0.343 |
| sp\|P22888\|LSHR_HUMAN Lutropin-choriogonadotropic hormone receptor OS=Homo sapiens GN=LHCGR PE= | 670 | KNGFTGsNKPS | 0.029 | -0.354 | -1.811 | -0.712 |
| sp\|P22888\|LSHR_HUMAN Lutropin-choriogonadotropic hormone receptor OS=Homo sapiens GN=LHCGR PE= | 674 | TGSNKPsQSTL | 0.041 | -0.347 | -1.468 | -0.591 |
| sp\|P22888\|LSHR_HUMAN Lutropin-choriogonadotropic hormone receptor OS=Homo sapiens GN=LHCGR PE= | 676 | SNKPSQsTLKL | 0.127 | 0 | -0.789 | -0.221 |
| sp\|P22888\|LSHR_HUMAN Lutropin-choriogonadotropic hormone receptor OS=Homo sapiens GN=LHCGR PE= | 677 | NKPSQStLKLS | 0.127 | -0.092 | -0.955 | -0.307 |
| sp\|P22888\|LSHR_HUMAN Lutropin-choriogonadotropic hormone receptor OS=Homo sapiens GN=LHCGR PE= | 681 | QSTLKLsTLHC | 0.034 | -0.394 | -1.789 | -0.716 |
| sp\|P22888\|LSHR_HUMAN Lutropin-choriogonadotropic hormone receptor OS=Homo sapiens GN=LHCGR PE= | 682 | STLKLStLHCQ | 0.228 | -0.062 | -0.969 | -0.268 |
| sp\|P22888\|LSHR_HUMAN Lutropin-choriogonadotropic hormone receptor OS=Homo sapiens GN=LHCGR PE= | 688 | TLHCQGtALLD | 0.114 | -0.059 | -1.373 | -0.439 |
| sp\|P22888\|LSHR_HUMAN Lutropin-choriogonadotropic hormone receptor OS=Homo sapiens GN=LHCGR PE= | 694 | TALLDKtRYTE | 0.131 | -0.237 | -1.049 | -0.385 |
| sp\|P22888\|LSHR_HUMAN Lutropin-choriogonadotropic hormone receptor OS=Homo sapiens GN=LHCGR PE= | 697 | LDKTRYtEC-- | 0.251 | 0.223 | -0.476 | -0.001 |

| | | | | | | |
|---|---|---|---|---|---|---|
| sp\|P16473\|TSHR_HUMAN Thyrotropin receptor OS=Homo sapiens GN=TSHR PE=1 SV=2 | 682 | FLYAIFtKAFQ | 0.161 | -0.036 | -0.66 | -0.178 |
| sp\|P16473\|TSHR_HUMAN Thyrotropin receptor OS=Homo sapiens GN=TSHR PE=1 SV=2 | 694 | DVFILLsKFGI | 0.14 | -0.087 | -0.865 | -0.271 |
| sp\|P16473\|TSHR_HUMAN Thyrotropin receptor OS=Homo sapiens GN=TSHR PE=1 SV=2 | 716 | RVPPKNsTDIQ | 0.13 | -0.138 | -1.18 | -0.396 |
| sp\|P16473\|TSHR_HUMAN Thyrotropin receptor OS=Homo sapiens GN=TSHR PE=1 SV=2 | 717 | VPPKNStDIQV | 0.151 | -0.176 | -1.119 | -0.381 |
| sp\|P16473\|TSHR_HUMAN Thyrotropin receptor OS=Homo sapiens GN=TSHR PE=1 SV=2 | 725 | IQVQKVtHDMR | 0.334 | -0.1 | -0.347 | -0.038 |
| sp\|P16473\|TSHR_HUMAN Thyrotropin receptor OS=Homo sapiens GN=TSHR PE=1 SV=2 | 745 | YELIENsHLTP | 0.115 | -0.143 | -1.319 | -0.449 |
| sp\|P16473\|TSHR_HUMAN Thyrotropin receptor OS=Homo sapiens GN=TSHR PE=1 SV=2 | 748 | IENSHLtPKKQ | 0.04 | -0.526 | -1.857 | -0.781 |
| sp\|P16473\|TSHR_HUMAN Thyrotropin receptor OS=Homo sapiens GN=TSHR PE=1 SV=2 | 756 | KKQGQIsEEYM | 0.239 | 0.062 | -0.702 | -0.134 |
| sp\|P16473\|TSHR_HUMAN Thyrotropin receptor OS=Homo sapiens GN=TSHR PE=1 SV=2 | 762 | SEEYMQtVL-- | 0.106 | -0.029 | -1.098 | -0.34 |
| sp\|Q8TCW9\|PKR1_HUMAN Prokineticin receptor 1 OS=Homo sapiens GN=PROKR1 PE=1 SV=1 | 349 | VTVKNDtVKYF | 0.199 | -0.189 | -0.982 | -0.324 |
| sp\|Q8TCW9\|PKR1_HUMAN Prokineticin receptor 1 OS=Homo sapiens GN=PROKR1 PE=1 SV=1 | 364 | LLHWKAsYNGG | 0.227 | -0.086 | -0.538 | -0.132 |
| sp\|Q8TCW9\|PKR1_HUMAN Prokineticin receptor 1 OS=Homo sapiens GN=PROKR1 PE=1 SV=1 | 370 | SYNGGKsSADL | 0.124 | -0.068 | -0.969 | -0.304 |
| sp\|Q8TCW9\|PKR1_HUMAN Prokineticin receptor 1 OS=Homo sapiens GN=PROKR1 PE=1 SV=1 | 371 | YNGGKSsADLD | 0.08 | -0.143 | -1.1 | -0.388 |
| sp\|Q8TCW9\|PKR1_HUMAN Prokineticin receptor 1 OS=Homo sapiens GN=PROKR1 PE=1 SV=1 | 378 | ADLDLKtIGMP | 0.09 | -0.265 | -1.189 | -0.455 |
| sp\|Q8TCW9\|PKR1_HUMAN Prokineticin receptor 1 OS=Homo sapiens GN=PROKR1 PE=1 SV=1 | 384 | TIGMPAtEEVD | 0.209 | 0.018 | -0.667 | -0.147 |
| sp\|Q8NFJ6\|PKR2_HUMAN Prokineticin receptor 2 OS=Homo sapiens GN=PROKR2 PE=1 SV=1 | 340 | VTVKNNtMKYF | 0.285 | 0.035 | -0.794 | -0.158 |
| sp\|Q8NFJ6\|PKR2_HUMAN Prokineticin receptor 2 OS=Homo sapiens GN=PROKR2 PE=1 SV=1 | 355 | LLHWRPsQRGS | 0.282 | -0.104 | -0.691 | -0.171 |
| sp\|Q8NFJ6\|PKR2_HUMAN Prokineticin receptor 2 OS=Homo sapiens GN=PROKR2 PE=1 SV=1 | 359 | RPSQRGsKSSA | 0.123 | -0.131 | -1.008 | -0.339 |
| sp\|Q8NFJ6\|PKR2_HUMAN Prokineticin receptor 2 OS=Homo sapiens GN=PROKR2 PE=1 SV=1 | 361 | SQRGSKsSADL | 0.509 | 0.424 | 0.186 | 0.373 |
| sp\|Q8NFJ6\|PKR2_HUMAN Prokineticin receptor 2 OS=Homo sapiens GN=PROKR2 PE=1 SV=1 | 362 | QRGSKSsADLD | 0.115 | 0.086 | -1.022 | -0.274 |
| sp\|Q8NFJ6\|PKR2_HUMAN Prokineticin receptor 2 OS=Homo sapiens GN=PROKR2 PE=1 SV=1 | 369 | ADLDLRtNGVP | 0.058 | -0.253 | -1.768 | -0.654 |
| sp\|Q8NFJ6\|PKR2_HUMAN Prokineticin receptor 2 OS=Homo sapiens GN=PROKR2 PE=1 SV=1 | 374 | RTNGVPtTEEV | 0.179 | -0.114 | -1.323 | -0.419 |
| sp\|Q8NFJ6\|PKR2_HUMAN Prokineticin receptor 2 OS=Homo sapiens GN=PROKR2 PE=1 SV=1 | 375 | TNGVPTtEEVD | 0.067 | -0.166 | -1.057 | -0.385 |
| sp\|O14842\|FFAR1_HUMAN Free fatty acid receptor 1 OS=Homo sapiens GN=FFAR1 PE=1 SV=1 | 287 | RGPGLKtVCAA | 0.145 | -0.093 | -0.687 | -0.212 |
| sp\|O14842\|FFAR1_HUMAN Free fatty acid receptor 1 OS=Homo sapiens GN=FFAR1 PE=1 SV=1 | 293 | TVCAARtQGGK | 0.215 | -0.126 | -1.002 | -0.304 |
| sp\|O14842\|FFAR1_HUMAN Free fatty acid receptor 1 OS=Homo sapiens GN=FFAR1 PE=1 SV=1 | 298 | RTQGGKsQK-- | 0.186 | 0.084 | -0.907 | -0.212 |
| sp\|O15552\|FFAR2_HUMAN Free fatty acid receptor 2 OS=Homo sapiens GN=FFAR2 PE=1 SV=1 | 277 | LLFYFSsSVVR | 0.171 | 0.013 | -0.83 | -0.215 |
| sp\|O15552\|FFAR2_HUMAN Free fatty acid receptor 2 OS=Homo sapiens GN=FFAR2 PE=1 SV=1 | 278 | LFYFSSsVVRR | 0.257 | 0.106 | -0.415 | -0.017 |
| sp\|O15552\|FFAR2_HUMAN Free fatty acid receptor 2 OS=Homo sapiens GN=FFAR2 PE=1 SV=1 | 296 | VLRNQGsSLLG | 0.158 | 0.124 | -0.881 | -0.2 |
| sp\|O15552\|FFAR2_HUMAN Free fatty acid receptor 2 OS=Homo sapiens GN=FFAR2 PE=1 SV=1 | 297 | LRNQGSsLLGR | 0.152 | 0.065 | -0.623 | -0.135 |
| sp\|O15552\|FFAR2_HUMAN Free fatty acid receptor 2 OS=Homo sapiens GN=FFAR2 PE=1 SV=1 | 306 | GRRGKDtAEGT | 0.294 | 0.275 | -0.647 | -0.026 |
| sp\|O15552\|FFAR2_HUMAN Free fatty acid receptor 2 OS=Homo sapiens GN=FFAR2 PE=1 SV=1 | 310 | KDTAEGtNEDR | 0.092 | -0.294 | -1.433 | -0.545 |
| sp\|O15552\|FFAR2_HUMAN Free fatty acid receptor 2 OS=Homo sapiens GN=FFAR2 PE=1 SV=1 | 324 | QGEGMPsSDFT | 0.083 | -0.34 | -1.042 | -0.433 |
| sp\|O15552\|FFAR2_HUMAN Free fatty acid receptor 2 OS=Homo sapiens GN=FFAR2 PE=1 SV=1 | 325 | GEGMPSsDFTT | 0.096 | -0.27 | -1.261 | -0.478 |
| sp\|O15552\|FFAR2_HUMAN Free fatty acid receptor 2 OS=Homo sapiens GN=FFAR2 PE=1 SV=1 | 328 | MPSSDFtTE-- | 0.134 | 0.022 | -1.042 | -0.295 |
| sp\|O15552\|FFAR2_HUMAN Free fatty acid receptor 2 OS=Homo sapiens GN=FFAR2 PE=1 SV=1 | 329 | PSSDFTtE--- | 0.244 | 0.111 | -0.818 | -0.154 |
| sp\|O14843\|FFAR3_HUMAN Free fatty acid receptor 3 OS=Homo sapiens GN=FFAR3 PE=1 SV=1 | 280 | FVYYFSsSGFQ | 0.08 | -0.125 | -1.241 | -0.429 |
| sp\|O14843\|FFAR3_HUMAN Free fatty acid receptor 3 OS=Homo sapiens GN=FFAR3 PE=1 SV=1 | 281 | VYYFSSsGFQA | 0.268 | 0.058 | -0.329 | -0.001 |
| sp\|O14843\|FFAR3_HUMAN Free fatty acid receptor 3 OS=Homo sapiens GN=FFAR3 PE=1 SV=1 | 305 | GQWQQEsSMEL | 0.152 | 0.072 | -0.965 | -0.247 |
| sp\|O14843\|FFAR3_HUMAN Free fatty acid receptor 3 OS=Homo sapiens GN=FFAR3 PE=1 SV=1 | 306 | QWQQESsMELK | 0.122 | -0.005 | -1.09 | -0.324 |
| sp\|O14843\|FFAR3_HUMAN Free fatty acid receptor 3 OS=Homo sapiens GN=FFAR3 PE=1 SV=1 | 328 | RPAERKtSEHS | 0.157 | 0.019 | -0.739 | -0.188 |
| sp\|O14843\|FFAR3_HUMAN Free fatty acid receptor 3 OS=Homo sapiens GN=FFAR3 PE=1 SV=1 | 329 | PAERKTsEHSQ | 0.464 | 0.753 | 0.001 | 0.406 |
| sp\|O14843\|FFAR3_HUMAN Free fatty acid receptor 3 OS=Homo sapiens GN=FFAR3 PE=1 SV=1 | 332 | RKTSEHsQGCG | 0.506 | 0.172 | -0.545 | 0.044 |
| sp\|O14843\|FFAR3_HUMAN Free fatty acid receptor 3 OS=Homo sapiens GN=FFAR3 PE=1 SV=1 | 337 | HSQGCGtGGQV | 0.218 | -0.064 | -0.777 | -0.208 |
| sp\|O14843\|FFAR3_HUMAN Free fatty acid receptor 3 OS=Homo sapiens GN=FFAR3 PE=1 SV=1 | 346 | QVACAEs---- | 0.227 | 0.106 | -0.794 | -0.154 |
| sp\|Q5NUL3\|FFAR4_HUMAN Free fatty acid receptor 4 OS=Homo sapiens GN=FFAR4 PE=1 SV=2 | 363 | EKGAILtDTSV | 0.119 | -0.098 | -1.157 | -0.379 |
| sp\|Q5NUL3\|FFAR4_HUMAN Free fatty acid receptor 4 OS=Homo sapiens GN=FFAR4 PE=1 SV=2 | 365 | GAILTDtSVKR | 0.09 | -0.132 | -1.488 | -0.51 |
| sp\|Q5NUL3\|FFAR4_HUMAN Free fatty acid receptor 4 OS=Homo sapiens GN=FFAR4 PE=1 SV=2 | 366 | AILTDTsVKRN | 0.186 | -0.118 | -0.994 | -0.309 |
| sp\|Q5NUL3\|FFAR4_HUMAN Free fatty acid receptor 4 OS=Homo sapiens GN=FFAR4 PE=1 SV=2 | 373 | VKRNDLsIISG | 0.146 | 0.075 | -0.546 | -0.108 |

| | | | | | | |
|---|---|---|---|---|---|---|
| sp\|Q5NUL3\|FFAR4_HUMAN Free fatty acid receptor 4 OS=Homo sapiens GN=FFAR4 PE=1 SV=2 | 376 | NDLSIIsG--- | 0.105 | -0.093 | -0.913 | -0.3 |
| sp\|O15529\|GPR42_HUMAN G-protein coupled receptor 42 OS=Homo sapiens GN=GPR42 PE=1 SV=1 | 280 | FVYYFSsSGFQ | 0.08 | -0.125 | -1.241 | -0.429 |
| sp\|O15529\|GPR42_HUMAN G-protein coupled receptor 42 OS=Homo sapiens GN=GPR42 PE=1 SV=1 | 281 | VYYFSSsGFQA | 0.268 | 0.058 | -0.329 | -0.001 |
| sp\|O15529\|GPR42_HUMAN G-protein coupled receptor 42 OS=Homo sapiens GN=GPR42 PE=1 SV=1 | 305 | GQWQQEsSMEL | 0.152 | 0.072 | -0.965 | -0.247 |
| sp\|O15529\|GPR42_HUMAN G-protein coupled receptor 42 OS=Homo sapiens GN=GPR42 PE=1 SV=1 | 306 | QWQQESsMELK | 0.122 | -0.005 | -1.09 | -0.324 |
| sp\|O15529\|GPR42_HUMAN G-protein coupled receptor 42 OS=Homo sapiens GN=GPR42 PE=1 SV=1 | 328 | RPAERKtSEHS | 0.157 | 0.019 | -0.739 | -0.188 |
| sp\|O15529\|GPR42_HUMAN G-protein coupled receptor 42 OS=Homo sapiens GN=GPR42 PE=1 SV=1 | 329 | PAERKTsEHSQ | 0.464 | 0.753 | 0.001 | 0.406 |
| sp\|O15529\|GPR42_HUMAN G-protein coupled receptor 42 OS=Homo sapiens GN=GPR42 PE=1 SV=1 | 332 | RKTSEHsQGCG | 0.506 | 0.172 | -0.545 | 0.044 |
| sp\|O15529\|GPR42_HUMAN G-protein coupled receptor 42 OS=Homo sapiens GN=GPR42 PE=1 SV=1 | 337 | HSQGCGtGGQV | 0.218 | -0.064 | -0.777 | -0.208 |
| sp\|Q15722\|LT4R1_HUMAN Leukotriene B4 receptor 1 OS=Homo sapiens GN=LTB4R PE=1 SV=2 | 295 | GGGLLRsAGVG | 0.154 | 0.022 | -1.19 | -0.338 |
| sp\|Q15722\|LT4R1_HUMAN Leukotriene B4 receptor 1 OS=Homo sapiens GN=LTB4R PE=1 SV=2 | 308 | AKLLEGtGSEA | 0.137 | -0.241 | -1.215 | -0.44 |
| sp\|Q15722\|LT4R1_HUMAN Leukotriene B4 receptor 1 OS=Homo sapiens GN=LTB4R PE=1 SV=2 | 310 | LLEGTGsEASS | 0.063 | -0.071 | -1.144 | -0.384 |
| sp\|Q15722\|LT4R1_HUMAN Leukotriene B4 receptor 1 OS=Homo sapiens GN=LTB4R PE=1 SV=2 | 313 | GTGSEAsSTRR | 0.208 | -0.226 | -1.367 | -0.462 |
| sp\|Q15722\|LT4R1_HUMAN Leukotriene B4 receptor 1 OS=Homo sapiens GN=LTB4R PE=1 SV=2 | 314 | TGSEASsTRRG | 0.103 | -0.284 | -1.254 | -0.478 |
| sp\|Q15722\|LT4R1_HUMAN Leukotriene B4 receptor 1 OS=Homo sapiens GN=LTB4R PE=1 SV=2 | 315 | GSEASStRRGG | 0.286 | -0.138 | -0.578 | -0.143 |
| sp\|Q15722\|LT4R1_HUMAN Leukotriene B4 receptor 1 OS=Homo sapiens GN=LTB4R PE=1 SV=2 | 320 | STRRGGsLGQT | 0.742 | 0.935 | 0.512 | 0.73 |
| sp\|Q15722\|LT4R1_HUMAN Leukotriene B4 receptor 1 OS=Homo sapiens GN=LTB4R PE=1 SV=2 | 324 | GGSLGQtARSG | 0.108 | -0.162 | -1.17 | -0.408 |
| sp\|Q15722\|LT4R1_HUMAN Leukotriene B4 receptor 1 OS=Homo sapiens GN=LTB4R PE=1 SV=2 | 327 | LGQTARsGPAA | 0.572 | 0.525 | 0.202 | 0.433 |
| sp\|Q15722\|LT4R1_HUMAN Leukotriene B4 receptor 1 OS=Homo sapiens GN=LTB4R PE=1 SV=2 | 337 | ALEPGPsESLT | 0.1 | -0.198 | -1.068 | -0.389 |
| sp\|Q15722\|LT4R1_HUMAN Leukotriene B4 receptor 1 OS=Homo sapiens GN=LTB4R PE=1 SV=2 | 339 | EPGPSEsLTAS | 0.302 | 0.13 | -0.405 | 0.009 |
| sp\|Q15722\|LT4R1_HUMAN Leukotriene B4 receptor 1 OS=Homo sapiens GN=LTB4R PE=1 SV=2 | 341 | GPSESLtASSP | 0.06 | -0.052 | -1.444 | -0.479 |
| sp\|Q15722\|LT4R1_HUMAN Leukotriene B4 receptor 1 OS=Homo sapiens GN=LTB4R PE=1 SV=2 | 343 | SESLTAsSPLK | 0.3 | 0.339 | -0.698 | -0.02 |
| sp\|Q15722\|LT4R1_HUMAN Leukotriene B4 receptor 1 OS=Homo sapiens GN=LTB4R PE=1 SV=2 | 344 | ESLTASsPLKL | 0.04 | -0.439 | -1.603 | -0.667 |
| sp\|Q9NPC1\|LT4R2_HUMAN Leukotriene B4 receptor 2 OS=Homo sapiens GN=LTB4R2 PE=2 SV=1 | 304 | QAARAGtTALA | 0.451 | 0.687 | 0.048 | 0.395 |
| sp\|Q9NPC1\|LT4R2_HUMAN Leukotriene B4 receptor 2 OS=Homo sapiens GN=LTB4R2 PE=2 SV=1 | 305 | AARAGTtALAF | 0.189 | 0.085 | -0.82 | -0.182 |
| sp\|Q9NPC1\|LT4R2_HUMAN Leukotriene B4 receptor 2 OS=Homo sapiens GN=LTB4R2 PE=2 SV=1 | 311 | TALAFFsSSVN | 0.095 | -0.184 | -1.31 | -0.466 |
| sp\|Q9NPC1\|LT4R2_HUMAN Leukotriene B4 receptor 2 OS=Homo sapiens GN=LTB4R2 PE=2 SV=1 | 312 | ALAFFSsSVNP | 0.124 | -0.105 | -1.281 | -0.421 |
| sp\|Q9NPC1\|LT4R2_HUMAN Leukotriene B4 receptor 2 OS=Homo sapiens GN=LTB4R2 PE=2 SV=1 | 313 | LAFFSSsVNPV | 0.263 | 0.092 | -0.592 | -0.079 |
| sp\|Q9NPC1\|LT4R2_HUMAN Leukotriene B4 receptor 2 OS=Homo sapiens GN=LTB4R2 PE=2 SV=1 | 322 | PVLYVFtAGDL | 0.475 | 0.258 | -0.103 | 0.21 |
| sp\|Q9NPC1\|LT4R2_HUMAN Leukotriene B4 receptor 2 OS=Homo sapiens GN=LTB4R2 PE=2 SV=1 | 336 | AGPRFLtRLFE | 0.157 | 0.43 | -0.899 | -0.104 |
| sp\|Q9NPC1\|LT4R2_HUMAN Leukotriene B4 receptor 2 OS=Homo sapiens GN=LTB4R2 PE=2 SV=1 | 342 | TRLFEGsGEAR | 0.157 | 0.076 | -0.846 | -0.204 |
| sp\|Q9NPC1\|LT4R2_HUMAN Leukotriene B4 receptor 2 OS=Homo sapiens GN=LTB4R2 PE=2 SV=1 | 351 | ARGGGRsREGT | 0.043 | -0.153 | -1.536 | -0.549 |
| sp\|Q9NPC1\|LT4R2_HUMAN Leukotriene B4 receptor 2 OS=Homo sapiens GN=LTB4R2 PE=2 SV=1 | 355 | GRSREGtMELR | 0.609 | 1.078 | 0.049 | 0.579 |
| sp\|Q9NPC1\|LT4R2_HUMAN Leukotriene B4 receptor 2 OS=Homo sapiens GN=LTB4R2 PE=2 SV=1 | 360 | GTMELRtTPQL | 0.346 | 0.435 | -0.552 | 0.076 |
| sp\|Q9NPC1\|LT4R2_HUMAN Leukotriene B4 receptor 2 OS=Homo sapiens GN=LTB4R2 PE=2 SV=1 | 361 | TMELRTtPQLK | 0.131 | -0.136 | -0.958 | -0.321 |
| sp\|Q9Y271\|CLTR1_HUMAN Cysteinyl leukotriene receptor 1 OS=Homo sapiens GN=CYSLTR1 PE=1 SV=1 | 307 | NFRKRLsTFRK | 0.64 | 0.281 | -0.007 | 0.305 |
| sp\|Q9Y271\|CLTR1_HUMAN Cysteinyl leukotriene receptor 1 OS=Homo sapiens GN=CYSLTR1 PE=1 SV=1 | 308 | FRKRLStFRKH | 0.574 | 0.939 | 0.16 | 0.558 |
| sp\|Q9Y271\|CLTR1_HUMAN Cysteinyl leukotriene receptor 1 OS=Homo sapiens GN=CYSLTR1 PE=1 SV=1 | 313 | STFRKHsLSSV | 0.498 | 0.844 | -0.228 | 0.371 |
| sp\|Q9Y271\|CLTR1_HUMAN Cysteinyl leukotriene receptor 1 OS=Homo sapiens GN=CYSLTR1 PE=1 SV=1 | 315 | FRKHSLsSVTY | 0.412 | 0.43 | -0.19 | 0.217 |
| sp\|Q9Y271\|CLTR1_HUMAN Cysteinyl leukotriene receptor 1 OS=Homo sapiens GN=CYSLTR1 PE=1 SV=1 | 316 | RKHSLSsVTYV | 0.14 | -0.167 | -1.449 | -0.492 |
| sp\|Q9Y271\|CLTR1_HUMAN Cysteinyl leukotriene receptor 1 OS=Homo sapiens GN=CYSLTR1 PE=1 SV=1 | 318 | HSLSSVtYVPR | 0.276 | 0.034 | -0.325 | -0.005 |
| sp\|Q9Y271\|CLTR1_HUMAN Cysteinyl leukotriene receptor 1 OS=Homo sapiens GN=CYSLTR1 PE=1 SV=1 | 326 | VPRKKAsLPEK | 0.896 | 0.747 | 0.801 | 0.815 |
| sp\|Q9NS75\|CLTR2_HUMAN Cysteinyl leukotriene receptor 2 OS=Homo sapiens GN=CYSLTR2 PE=1 SV=1 | 318 | FKDRLKsALRK | 0.7 | 0.874 | 0.226 | 0.6 |
| sp\|Q9NS75\|CLTR2_HUMAN Cysteinyl leukotriene receptor 2 OS=Homo sapiens GN=CYSLTR2 PE=1 SV=1 | 330 | HPQKAKtKCVF | 0.39 | 0.142 | -0.193 | 0.113 |
| sp\|Q9NS75\|CLTR2_HUMAN Cysteinyl leukotriene receptor 2 OS=Homo sapiens GN=CYSLTR2 PE=1 SV=1 | 337 | KCVFPVsVWLR | 0.411 | 0.022 | -0.548 | -0.038 |
| sp\|Q9NS75\|CLTR2_HUMAN Cysteinyl leukotriene receptor 2 OS=Homo sapiens GN=CYSLTR2 PE=1 SV=1 | 344 | VWLRKEtRV-- | 0.348 | 0.782 | -0.332 | 0.266 |
| sp\|Q8TDS5\|OXER1_HUMAN Oxoeicosanoid receptor 1 OS=Homo sapiens GN=OXER1 PE=1 SV=1 | 359 | VLYCFSsPNFL | 0.074 | -0.372 | -1.338 | -0.545 |
| sp\|Q8TDS5\|OXER1_HUMAN Oxoeicosanoid receptor 1 OS=Homo sapiens GN=OXER1 PE=1 SV=1 | 366 | PNFLHQsRALL | 0.151 | -0.1 | -0.984 | -0.311 |
| sp\|Q8TDS5\|OXER1_HUMAN Oxoeicosanoid receptor 1 OS=Homo sapiens GN=OXER1 PE=1 SV=1 | 373 | RALLGLtRGRQ | 0.086 | -0.321 | -1.55 | -0.595 |

| | | | | | | |
|---|---|---|---|---|---|---|
| sp\|Q8TDS5\|OXER1_HUMAN Oxoeicosanoid receptor 1 OS=Homo sapiens GN=OXER1 PE=1 SV=1 | 381 | GRQGPVsDESS | 0.225 | 0.161 | -0.748 | -0.121 |
| sp\|Q8TDS5\|OXER1_HUMAN Oxoeicosanoid receptor 1 OS=Homo sapiens GN=OXER1 PE=1 SV=1 | 384 | GPVSDEsSYQP | 0.075 | -0.307 | -1.583 | -0.605 |
| sp\|Q8TDS5\|OXER1_HUMAN Oxoeicosanoid receptor 1 OS=Homo sapiens GN=OXER1 PE=1 SV=1 | 385 | PVSDESsYQPS | 0.059 | -0.262 | -1.494 | -0.566 |
| sp\|Q8TDS5\|OXER1_HUMAN Oxoeicosanoid receptor 1 OS=Homo sapiens GN=OXER1 PE=1 SV=1 | 389 | ESSYQPsRQWR | 0.089 | -0.232 | -1.055 | -0.399 |
| sp\|Q8TDS5\|OXER1_HUMAN Oxoeicosanoid receptor 1 OS=Homo sapiens GN=OXER1 PE=1 SV=1 | 398 | WRYREAsRKAE | 0.631 | 0.952 | 0.329 | 0.637 |
| sp\|Q8TDS5\|OXER1_HUMAN Oxoeicosanoid receptor 1 OS=Homo sapiens GN=OXER1 PE=1 SV=1 | 414 | KVQGEVsLEKE | 0.263 | -0.023 | -0.712 | -0.157 |
| sp\|Q8TDS5\|OXER1_HUMAN Oxoeicosanoid receptor 1 OS=Homo sapiens GN=OXER1 PE=1 SV=1 | 420 | SLEKEGsSQG- | 0.224 | -0.121 | -0.993 | -0.297 |
| sp\|Q8TDS5\|OXER1_HUMAN Oxoeicosanoid receptor 1 OS=Homo sapiens GN=OXER1 PE=1 SV=1 | 421 | LEKEGSsQG-- | 0.07 | -0.07 | -1.417 | -0.472 |
| sp\|Q99677\|LPAR4_HUMAN Lysophosphatidic acid receptor 4 OS=Homo sapiens GN=LPAR4 PE=1 SV=1 | 345 | PLTTKPsLPAI | 0.861 | 0.685 | 0.951 | 0.832 |
| sp\|Q99677\|LPAR4_HUMAN Lysophosphatidic acid receptor 4 OS=Homo sapiens GN=LPAR4 PE=1 SV=1 | 354 | AIQEEVsDQTT | 0.265 | -0.022 | -0.909 | -0.222 |
| sp\|Q99677\|LPAR4_HUMAN Lysophosphatidic acid receptor 4 OS=Homo sapiens GN=LPAR4 PE=1 SV=1 | 357 | EEVSDQtTNNG | 0.11 | -0.106 | -1.17 | -0.389 |
| sp\|Q99677\|LPAR4_HUMAN Lysophosphatidic acid receptor 4 OS=Homo sapiens GN=LPAR4 PE=1 SV=1 | 358 | EVSDQTtNNGG | 0.101 | -0.133 | -1.102 | -0.378 |
| sp\|Q99677\|LPAR4_HUMAN Lysophosphatidic acid receptor 4 OS=Homo sapiens GN=LPAR4 PE=1 SV=1 | 368 | GELMLEsTF-- | 0.151 | -0.039 | -1.218 | -0.369 |
| sp\|Q99677\|LPAR4_HUMAN Lysophosphatidic acid receptor 4 OS=Homo sapiens GN=LPAR4 PE=1 SV=1 | 369 | ELMLEStF--- | 0.206 | 0.202 | -0.75 | -0.114 |
| sp\|Q9H1C0\|LPAR5_HUMAN Lysophosphatidic acid receptor 5 OS=Homo sapiens GN=LPAR5 PE=2 SV=1 | 307 | AEGFRNtLRGL | 0.552 | 0.148 | -0.247 | 0.151 |
| sp\|Q9H1C0\|LPAR5_HUMAN Lysophosphatidic acid receptor 5 OS=Homo sapiens GN=LPAR5 PE=2 SV=1 | 313 | TLRGLGtPHRA | 0.156 | -0.223 | -1.159 | -0.409 |
| sp\|Q9H1C0\|LPAR5_HUMAN Lysophosphatidic acid receptor 5 OS=Homo sapiens GN=LPAR5 PE=2 SV=1 | 319 | TPHRARtSATN | 0.325 | 0.686 | -0.455 | 0.185 |
| sp\|Q9H1C0\|LPAR5_HUMAN Lysophosphatidic acid receptor 5 OS=Homo sapiens GN=LPAR5 PE=2 SV=1 | 320 | PHRARTsATNG | 0.676 | 0.419 | -0.392 | 0.234 |
| sp\|Q9H1C0\|LPAR5_HUMAN Lysophosphatidic acid receptor 5 OS=Homo sapiens GN=LPAR5 PE=2 SV=1 | 322 | RARTSAtNGTR | 0.738 | 0.432 | 0.074 | 0.415 |
| sp\|Q9H1C0\|LPAR5_HUMAN Lysophosphatidic acid receptor 5 OS=Homo sapiens GN=LPAR5 PE=2 SV=1 | 325 | TSATNGtRAAL | 0.105 | -0.265 | -1.082 | -0.414 |
| sp\|Q9H1C0\|LPAR5_HUMAN Lysophosphatidic acid receptor 5 OS=Homo sapiens GN=LPAR5 PE=2 SV=1 | 332 | RAALAQsERSA | 0.232 | -0.008 | -0.599 | -0.125 |
| sp\|Q9H1C0\|LPAR5_HUMAN Lysophosphatidic acid receptor 5 OS=Homo sapiens GN=LPAR5 PE=2 SV=1 | 335 | LAQSERsAVTT | 0.073 | -0.192 | -1.535 | -0.551 |
| sp\|Q9H1C0\|LPAR5_HUMAN Lysophosphatidic acid receptor 5 OS=Homo sapiens GN=LPAR5 PE=2 SV=1 | 338 | SERSAVtTDAT | 0.481 | 0.198 | -0.284 | 0.132 |
| sp\|Q9H1C0\|LPAR5_HUMAN Lysophosphatidic acid receptor 5 OS=Homo sapiens GN=LPAR5 PE=2 SV=1 | 339 | ERSAVTtDATR | 0.209 | 0.067 | -0.771 | -0.165 |
| sp\|Q9H1C0\|LPAR5_HUMAN Lysophosphatidic acid receptor 5 OS=Homo sapiens GN=LPAR5 PE=2 SV=1 | 342 | AVTTDAtRPDA | 0.586 | 0.365 | -0.085 | 0.289 |
| sp\|Q9H1C0\|LPAR5_HUMAN Lysophosphatidic acid receptor 5 OS=Homo sapiens GN=LPAR5 PE=2 SV=1 | 348 | TRPDAAsQGLL | 0.35 | 0.271 | -0.687 | -0.022 |
| sp\|Q9H1C0\|LPAR5_HUMAN Lysophosphatidic acid receptor 5 OS=Homo sapiens GN=LPAR5 PE=2 SV=1 | 355 | QGLLRPsDSHS | 0.135 | -0.102 | -0.979 | -0.315 |
| sp\|Q9H1C0\|LPAR5_HUMAN Lysophosphatidic acid receptor 5 OS=Homo sapiens GN=LPAR5 PE=2 SV=1 | 357 | LLRPSDsHSLS | 0.494 | 0.324 | -0.168 | 0.217 |
| sp\|Q9H1C0\|LPAR5_HUMAN Lysophosphatidic acid receptor 5 OS=Homo sapiens GN=LPAR5 PE=2 SV=1 | 359 | RPSDSHsLSSF | 0.333 | 0.257 | -0.408 | 0.061 |
| sp\|Q9H1C0\|LPAR5_HUMAN Lysophosphatidic acid receptor 5 OS=Homo sapiens GN=LPAR5 PE=2 SV=1 | 361 | SDSHSLsSFTQ | 0.071 | -0.174 | -1.215 | -0.439 |
| sp\|Q9H1C0\|LPAR5_HUMAN Lysophosphatidic acid receptor 5 OS=Homo sapiens GN=LPAR5 PE=2 SV=1 | 362 | DSHSLSsFTQC | 0.093 | -0.367 | -1.611 | -0.628 |
| sp\|Q9H1C0\|LPAR5_HUMAN Lysophosphatidic acid receptor 5 OS=Homo sapiens GN=LPAR5 PE=2 SV=1 | 364 | HSLSSFtQCPQ | 0.136 | -0.091 | -0.91 | -0.288 |
| sp\|Q9H1C0\|LPAR5_HUMAN Lysophosphatidic acid receptor 5 OS=Homo sapiens GN=LPAR5 PE=2 SV=1 | 370 | TQCPQDsAL-- | 0.062 | -0.09 | -1.587 | -0.538 |
| sp\|P43657\|LPAR6_HUMAN Lysophosphatidic acid receptor 6 OS=Homo sapiens GN=LPAR6 PE=1 SV=3 | 295 | IVYYFTsDTIQ | 0.267 | -0.057 | -0.782 | -0.191 |
| sp\|P43657\|LPAR6_HUMAN Lysophosphatidic acid receptor 6 OS=Homo sapiens GN=LPAR6 PE=1 SV=3 | 297 | YYFTSDtIQNS | 0.31 | 0.136 | -0.656 | -0.07 |
| sp\|P43657\|LPAR6_HUMAN Lysophosphatidic acid receptor 6 OS=Homo sapiens GN=LPAR6 PE=1 SV=3 | 301 | SDTIQNsIKMK | 0.044 | -0.308 | -1.593 | -0.619 |
| sp\|P43657\|LPAR6_HUMAN Lysophosphatidic acid receptor 6 OS=Homo sapiens GN=LPAR6 PE=1 SV=3 | 308 | IKMKNWsVRRS | 0.556 | -0.046 | -0.233 | 0.092 |
| sp\|P43657\|LPAR6_HUMAN Lysophosphatidic acid receptor 6 OS=Homo sapiens GN=LPAR6 PE=1 SV=3 | 312 | NWSVRRsDFRF | 0.187 | 0.032 | -1.056 | -0.279 |
| sp\|P43657\|LPAR6_HUMAN Lysophosphatidic acid receptor 6 OS=Homo sapiens GN=LPAR6 PE=1 SV=3 | 317 | RSDFRFsEVHG | 0.334 | 0.157 | -0.421 | 0.023 |
| sp\|P43657\|LPAR6_HUMAN Lysophosphatidic acid receptor 6 OS=Homo sapiens GN=LPAR6 PE=1 SV=3 | 332 | IQHNLQtLKSK | 0.078 | -0.162 | -0.783 | -0.289 |
| sp\|P43657\|LPAR6_HUMAN Lysophosphatidic acid receptor 6 OS=Homo sapiens GN=LPAR6 PE=1 SV=3 | 335 | NLQTLKsKIFD | 0.278 | 0.061 | -0.375 | -0.012 |
| sp\|P43657\|LPAR6_HUMAN Lysophosphatidic acid receptor 6 OS=Homo sapiens GN=LPAR6 PE=1 SV=3 | 342 | KIFDNEsAA-- | 0.18 | 0.096 | -1.162 | -0.295 |
| sp\|P21453\|S1PR1_HUMAN Sphingosine 1-phosphate receptor 1 OS=Homo sapiens GN=S1PR1 PE=1 SV=2 | 327 | AFIRIMsCCKC | 0.552 | 0.875 | 0.075 | 0.501 |
| sp\|P21453\|S1PR1_HUMAN Sphingosine 1-phosphate receptor 1 OS=Homo sapiens GN=S1PR1 PE=1 SV=2 | 333 | SCCKCPsGDSA | 0.292 | -0.099 | -0.823 | -0.21 |
| sp\|P21453\|S1PR1_HUMAN Sphingosine 1-phosphate receptor 1 OS=Homo sapiens GN=S1PR1 PE=1 SV=2 | 336 | KCPSGDsAGKF | 0.07 | -0.255 | -1.686 | -0.624 |
| sp\|P21453\|S1PR1_HUMAN Sphingosine 1-phosphate receptor 1 OS=Homo sapiens GN=S1PR1 PE=1 SV=2 | 351 | IAGMEFsRSKS | 0.057 | -0.361 | -1.608 | -0.637 |
| sp\|P21453\|S1PR1_HUMAN Sphingosine 1-phosphate receptor 1 OS=Homo sapiens GN=S1PR1 PE=1 SV=2 | 353 | GMEFSRsKSDN | 0.365 | 0.339 | -0.397 | 0.102 |
| sp\|P21453\|S1PR1_HUMAN Sphingosine 1-phosphate receptor 1 OS=Homo sapiens GN=S1PR1 PE=1 SV=2 | 355 | EFSRSKsDNSS | 0.77 | 1.102 | 0.98 | 0.951 |
| sp\|P21453\|S1PR1_HUMAN Sphingosine 1-phosphate receptor 1 OS=Homo sapiens GN=S1PR1 PE=1 SV=2 | 358 | RSKSDNsSHPQ | 0.192 | -0.193 | -1.086 | -0.362 |

| | | | | | | |
|---|---|---|---|---|---|---|
| sp\|P21453\|S1PR1_HUMAN Sphingosine 1-phosphate receptor 1 OS=Homo sapiens GN=S1PR1 PE=1 SV=2 | 359 | SKSDNSsHPQK | 0.579 | 0.327 | -0.082 | 0.275 |
| sp\|P21453\|S1PR1_HUMAN Sphingosine 1-phosphate receptor 1 OS=Homo sapiens GN=S1PR1 PE=1 SV=2 | 371 | EGDNPEtIMSS | 0.027 | -0.359 | -1.509 | -0.614 |
| sp\|P21453\|S1PR1_HUMAN Sphingosine 1-phosphate receptor 1 OS=Homo sapiens GN=S1PR1 PE=1 SV=2 | 374 | NPETIMsSGNV | 0.288 | 0.077 | -0.817 | -0.151 |
| sp\|P21453\|S1PR1_HUMAN Sphingosine 1-phosphate receptor 1 OS=Homo sapiens GN=S1PR1 PE=1 SV=2 | 375 | PETIMSsGNVN | 0.129 | -0.118 | -0.947 | -0.312 |
| sp\|P21453\|S1PR1_HUMAN Sphingosine 1-phosphate receptor 1 OS=Homo sapiens GN=S1PR1 PE=1 SV=2 | 380 | SSGNVNsSS-- | 0.06 | -0.12 | -1.555 | -0.538 |
| sp\|P21453\|S1PR1_HUMAN Sphingosine 1-phosphate receptor 1 OS=Homo sapiens GN=S1PR1 PE=1 SV=2 | 381 | SGNVNSsS--- | 0.048 | -0.122 | -1.522 | -0.532 |
| sp\|P21453\|S1PR1_HUMAN Sphingosine 1-phosphate receptor 1 OS=Homo sapiens GN=S1PR1 PE=1 SV=2 | 382 | GNVNSSs---- | 0.195 | 0.198 | -0.569 | -0.059 |
| sp\|O95136\|S1PR2_HUMAN Sphingosine 1-phosphate receptor 2 OS=Homo sapiens GN=S1PR2 PE=1 SV=2 | 292 | VIYTWRsRDLR | 0.174 | -0.013 | -0.925 | -0.255 |
| sp\|O95136\|S1PR2_HUMAN Sphingosine 1-phosphate receptor 2 OS=Homo sapiens GN=S1PR2 PE=1 SV=2 | 320 | GRRRGGtPGHH | 0.449 | 0.789 | -0.092 | 0.382 |
| sp\|O95136\|S1PR2_HUMAN Sphingosine 1-phosphate receptor 2 OS=Homo sapiens GN=S1PR2 PE=1 SV=2 | 330 | HLLPLRsSSSL | 0.046 | -0.108 | -1.375 | -0.479 |
| sp\|O95136\|S1PR2_HUMAN Sphingosine 1-phosphate receptor 2 OS=Homo sapiens GN=S1PR2 PE=1 SV=2 | 331 | LLPLRSsSSLE | 0.113 | -0.05 | -1.121 | -0.353 |
| sp\|O95136\|S1PR2_HUMAN Sphingosine 1-phosphate receptor 2 OS=Homo sapiens GN=S1PR2 PE=1 SV=2 | 332 | LPLRSSsSLER | 0.56 | 0.903 | 0.314 | 0.592 |
| sp\|O95136\|S1PR2_HUMAN Sphingosine 1-phosphate receptor 2 OS=Homo sapiens GN=S1PR2 PE=1 SV=2 | 333 | PLRSSSsLERG | 0.735 | 0.447 | 0.291 | 0.491 |
| sp\|O95136\|S1PR2_HUMAN Sphingosine 1-phosphate receptor 2 OS=Homo sapiens GN=S1PR2 PE=1 SV=2 | 342 | RGMHMPtSPTF | 0.378 | 0.291 | -0.179 | 0.163 |
| sp\|O95136\|S1PR2_HUMAN Sphingosine 1-phosphate receptor 2 OS=Homo sapiens GN=S1PR2 PE=1 SV=2 | 343 | GMHMPTsPTFL | 0.059 | -0.397 | -1.511 | -0.616 |
| sp\|O95136\|S1PR2_HUMAN Sphingosine 1-phosphate receptor 2 OS=Homo sapiens GN=S1PR2 PE=1 SV=2 | 345 | HMPTSPtFLEG | 0.267 | 0.276 | -0.465 | 0.026 |
| sp\|O95136\|S1PR2_HUMAN Sphingosine 1-phosphate receptor 2 OS=Homo sapiens GN=S1PR2 PE=1 SV=2 | 351 | TFLEGNtVV-- | 0.11 | 0.062 | -1.01 | -0.279 |
| sp\|Q99500\|S1PR3_HUMAN Sphingosine 1-phosphate receptor 3 OS=Homo sapiens GN=S1PR3 PE=1 SV=2 | 302 | VIYTLAsKEMR | 0.147 | -0.104 | -1.051 | -0.336 |
| sp\|Q99500\|S1PR3_HUMAN Sphingosine 1-phosphate receptor 3 OS=Homo sapiens GN=S1PR3 PE=1 SV=2 | 326 | GRGARAsPIQP | 0.179 | -0.018 | -0.803 | -0.214 |
| sp\|Q99500\|S1PR3_HUMAN Sphingosine 1-phosphate receptor 3 OS=Homo sapiens GN=S1PR3 PE=1 SV=2 | 335 | QPALDPsRSKS | 0.044 | -0.407 | -1.737 | -0.7 |
| sp\|Q99500\|S1PR3_HUMAN Sphingosine 1-phosphate receptor 3 OS=Homo sapiens GN=S1PR3 PE=1 SV=2 | 337 | ALDPSRsKSSS | 0.11 | -0.003 | -1.047 | -0.313 |
| sp\|Q99500\|S1PR3_HUMAN Sphingosine 1-phosphate receptor 3 OS=Homo sapiens GN=S1PR3 PE=1 SV=2 | 339 | DPSRSKsSSSN | 0.622 | 0.913 | 0.265 | 0.6 |
| sp\|Q99500\|S1PR3_HUMAN Sphingosine 1-phosphate receptor 3 OS=Homo sapiens GN=S1PR3 PE=1 SV=2 | 340 | PSRSKSsSSNN | 0.105 | -0.052 | -1.334 | -0.427 |
| sp\|Q99500\|S1PR3_HUMAN Sphingosine 1-phosphate receptor 3 OS=Homo sapiens GN=S1PR3 PE=1 SV=2 | 341 | SRSKSSsSNNS | 0.289 | 0.31 | -0.587 | 0.004 |
| sp\|Q99500\|S1PR3_HUMAN Sphingosine 1-phosphate receptor 3 OS=Homo sapiens GN=S1PR3 PE=1 SV=2 | 342 | RSKSSSsNNSS | 0.159 | -0.015 | -0.836 | -0.231 |
| sp\|Q99500\|S1PR3_HUMAN Sphingosine 1-phosphate receptor 3 OS=Homo sapiens GN=S1PR3 PE=1 SV=2 | 345 | SSSSNNsSHSP | 0.061 | -0.332 | -1.599 | -0.623 |
| sp\|Q99500\|S1PR3_HUMAN Sphingosine 1-phosphate receptor 3 OS=Homo sapiens GN=S1PR3 PE=1 SV=2 | 346 | SSSNNSsHSPK | 0.042 | -0.429 | -1.446 | -0.611 |
| sp\|Q99500\|S1PR3_HUMAN Sphingosine 1-phosphate receptor 3 OS=Homo sapiens GN=S1PR3 PE=1 SV=2 | 348 | SNNSSHsPKVK | 0.098 | -0.241 | -1.203 | -0.449 |
| sp\|Q99500\|S1PR3_HUMAN Sphingosine 1-phosphate receptor 3 OS=Homo sapiens GN=S1PR3 PE=1 SV=2 | 358 | KEDLPHtAPSS | 0.301 | 0.382 | -0.712 | -0.01 |
| sp\|Q99500\|S1PR3_HUMAN Sphingosine 1-phosphate receptor 3 OS=Homo sapiens GN=S1PR3 PE=1 SV=2 | 361 | LPHTAPsSCIM | 0.098 | -0.29 | -1.324 | -0.505 |
| sp\|Q99500\|S1PR3_HUMAN Sphingosine 1-phosphate receptor 3 OS=Homo sapiens GN=S1PR3 PE=1 SV=2 | 362 | PHTAPSsCIMD | 0.104 | -0.162 | -1.112 | -0.39 |
| sp\|O95977\|S1PR4_HUMAN Sphingosine 1-phosphate receptor 4 OS=Homo sapiens GN=S1PR4 PE=1 SV=1 | 311 | IIYSFRsREVC | 0.079 | -0.207 | -1.602 | -0.577 |
| sp\|O95977\|S1PR4_HUMAN Sphingosine 1-phosphate receptor 4 OS=Homo sapiens GN=S1PR4 PE=1 SV=1 | 320 | VCRAVLsFLCC | 0.275 | 0.16 | -0.886 | -0.15 |
| sp\|O95977\|S1PR4_HUMAN Sphingosine 1-phosphate receptor 4 OS=Homo sapiens GN=S1PR4 PE=1 SV=1 | 346 | RAVEAHsGAST | 0.092 | -0.072 | -1.207 | -0.396 |
| sp\|O95977\|S1PR4_HUMAN Sphingosine 1-phosphate receptor 4 OS=Homo sapiens GN=S1PR4 PE=1 SV=1 | 349 | EAHSGAsTTDS | 0.085 | -0.231 | -1.376 | -0.507 |
| sp\|O95977\|S1PR4_HUMAN Sphingosine 1-phosphate receptor 4 OS=Homo sapiens GN=S1PR4 PE=1 SV=1 | 350 | AHSGAStTDSS | 0.077 | -0.168 | -1.482 | -0.524 |
| sp\|O95977\|S1PR4_HUMAN Sphingosine 1-phosphate receptor 4 OS=Homo sapiens GN=S1PR4 PE=1 SV=1 | 351 | HSGASTtDSSL | 0.14 | 0.093 | -0.725 | -0.164 |
| sp\|O95977\|S1PR4_HUMAN Sphingosine 1-phosphate receptor 4 OS=Homo sapiens GN=S1PR4 PE=1 SV=1 | 353 | GASTTDsSLRP | 0.062 | -0.359 | -1.893 | -0.73 |
| sp\|O95977\|S1PR4_HUMAN Sphingosine 1-phosphate receptor 4 OS=Homo sapiens GN=S1PR4 PE=1 SV=1 | 354 | ASTTDSsLRPR | 0.169 | -0.268 | -0.828 | -0.309 |
| sp\|O95977\|S1PR4_HUMAN Sphingosine 1-phosphate receptor 4 OS=Homo sapiens GN=S1PR4 PE=1 SV=1 | 360 | SLRPRDsFRGS | 0.575 | 0.215 | -0.193 | 0.199 |
| sp\|O95977\|S1PR4_HUMAN Sphingosine 1-phosphate receptor 4 OS=Homo sapiens GN=S1PR4 PE=1 SV=1 | 364 | RDSFRGsRSLS | 0.097 | -0.182 | -1.42 | -0.502 |
| sp\|O95977\|S1PR4_HUMAN Sphingosine 1-phosphate receptor 4 OS=Homo sapiens GN=S1PR4 PE=1 SV=1 | 366 | SFRGSRsLSFR | 0.392 | 0.351 | -0.163 | 0.193 |
| sp\|O95977\|S1PR4_HUMAN Sphingosine 1-phosphate receptor 4 OS=Homo sapiens GN=S1PR4 PE=1 SV=1 | 368 | RGSRSLsFRMR | 0.785 | 0.975 | 0.84 | 0.867 |
| sp\|O95977\|S1PR4_HUMAN Sphingosine 1-phosphate receptor 4 OS=Homo sapiens GN=S1PR4 PE=1 SV=1 | 376 | RMREPLsSISS | 0.049 | 0.089 | -1.253 | -0.372 |
| sp\|O95977\|S1PR4_HUMAN Sphingosine 1-phosphate receptor 4 OS=Homo sapiens GN=S1PR4 PE=1 SV=1 | 377 | MREPLSsISSV | 0.048 | -0.069 | -1.526 | -0.516 |
| sp\|O95977\|S1PR4_HUMAN Sphingosine 1-phosphate receptor 4 OS=Homo sapiens GN=S1PR4 PE=1 SV=1 | 379 | EPLSSIsSVRS | 0.151 | -0.078 | -0.975 | -0.301 |
| sp\|O95977\|S1PR4_HUMAN Sphingosine 1-phosphate receptor 4 OS=Homo sapiens GN=S1PR4 PE=1 SV=1 | 380 | PLSSISsVRSI | 0.166 | -0.106 | -0.608 | -0.183 |
| sp\|O95977\|S1PR4_HUMAN Sphingosine 1-phosphate receptor 4 OS=Homo sapiens GN=S1PR4 PE=1 SV=1 | 383 | SISSVRsI--- | 0.085 | -0.025 | -1.406 | -0.449 |
| sp\|Q9H228\|S1PR5_HUMAN Sphingosine 1-phosphate receptor 5 OS=Homo sapiens GN=S1PR5 PE=2 SV=1 | 327 | VCCGRHsCGRD | 0.674 | 0.346 | -0.191 | 0.276 |

| | | | | | | |
|---|---|---|---|---|---|---|
| sp\|Q9H228\|S1PR5_HUMAN Sphingosine 1-phosphate receptor 5 OS=Homo sapiens GN=S1PR5 PE=2 SV=1 | 333 | SCGRDPsGSQQ | 0.25 | 0.516 | -0.63 | 0.045 |
| sp\|Q9H228\|S1PR5_HUMAN Sphingosine 1-phosphate receptor 5 OS=Homo sapiens GN=S1PR5 PE=2 SV=1 | 335 | GRDPSGsQQSA | 0.191 | 0.17 | -0.828 | -0.156 |
| sp\|Q9H228\|S1PR5_HUMAN Sphingosine 1-phosphate receptor 5 OS=Homo sapiens GN=S1PR5 PE=2 SV=1 | 338 | PSGSQQsASAA | 0.087 | -0.135 | -1.202 | -0.417 |
| sp\|Q9H228\|S1PR5_HUMAN Sphingosine 1-phosphate receptor 5 OS=Homo sapiens GN=S1PR5 PE=2 SV=1 | 340 | GSQQSAsAAEA | 0.595 | 0.244 | -0.11 | 0.243 |
| sp\|Q9H228\|S1PR5_HUMAN Sphingosine 1-phosphate receptor 5 OS=Homo sapiens GN=S1PR5 PE=2 SV=1 | 345 | ASAAEAsGGLR | 0.241 | -0.153 | -0.764 | -0.225 |
| sp\|Q9H228\|S1PR5_HUMAN Sphingosine 1-phosphate receptor 5 OS=Homo sapiens GN=S1PR5 PE=2 SV=1 | 359 | PPGLDGsFSGS | 0.062 | -0.259 | -1.42 | -0.539 |
| sp\|Q9H228\|S1PR5_HUMAN Sphingosine 1-phosphate receptor 5 OS=Homo sapiens GN=S1PR5 PE=2 SV=1 | 361 | GLDGSFsGSER | 0.419 | 0.245 | -0.15 | 0.171 |
| sp\|Q9H228\|S1PR5_HUMAN Sphingosine 1-phosphate receptor 5 OS=Homo sapiens GN=S1PR5 PE=2 SV=1 | 363 | DGSFSGsERSS | 0.207 | 0.011 | -0.775 | -0.186 |
| sp\|Q9H228\|S1PR5_HUMAN Sphingosine 1-phosphate receptor 5 OS=Homo sapiens GN=S1PR5 PE=2 SV=1 | 366 | FSGSERsSPQR | 0.304 | 0.287 | -0.642 | -0.017 |
| sp\|Q9H228\|S1PR5_HUMAN Sphingosine 1-phosphate receptor 5 OS=Homo sapiens GN=S1PR5 PE=2 SV=1 | 367 | SGSERSsPQRD | 0.063 | -0.415 | -1.511 | -0.621 |
| sp\|Q9H228\|S1PR5_HUMAN Sphingosine 1-phosphate receptor 5 OS=Homo sapiens GN=S1PR5 PE=2 SV=1 | 375 | QRDGLDtSGST | 0.062 | -0.088 | -1.398 | -0.475 |
| sp\|Q9H228\|S1PR5_HUMAN Sphingosine 1-phosphate receptor 5 OS=Homo sapiens GN=S1PR5 PE=2 SV=1 | 376 | RDGLDTsGSTG | 0.056 | -0.291 | -1.463 | -0.566 |
| sp\|Q9H228\|S1PR5_HUMAN Sphingosine 1-phosphate receptor 5 OS=Homo sapiens GN=S1PR5 PE=2 SV=1 | 378 | GLDTSGsTGSP | 0.25 | 0.107 | -0.706 | -0.116 |
| sp\|Q9H228\|S1PR5_HUMAN Sphingosine 1-phosphate receptor 5 OS=Homo sapiens GN=S1PR5 PE=2 SV=1 | 379 | LDTSGStGSPG | 0.021 | -0.499 | -1.742 | -0.74 |
| sp\|Q9H228\|S1PR5_HUMAN Sphingosine 1-phosphate receptor 5 OS=Homo sapiens GN=S1PR5 PE=2 SV=1 | 381 | TSGSTGsPGAP | 0.03 | -0.501 | -1.708 | -0.726 |
| sp\|Q9H228\|S1PR5_HUMAN Sphingosine 1-phosphate receptor 5 OS=Homo sapiens GN=S1PR5 PE=2 SV=1 | 386 | GSPGAPtAART | 0.13 | -0.251 | -1.295 | -0.472 |
| sp\|Q9H228\|S1PR5_HUMAN Sphingosine 1-phosphate receptor 5 OS=Homo sapiens GN=S1PR5 PE=2 SV=1 | 390 | APTAARtLVSE | 0.186 | -0.067 | -0.906 | -0.262 |
| sp\|Q9H228\|S1PR5_HUMAN Sphingosine 1-phosphate receptor 5 OS=Homo sapiens GN=S1PR5 PE=2 SV=1 | 393 | AARTLVsEPAA | 0.843 | 0.693 | 0.501 | 0.679 |
| sp\|P21554\|CNR1_HUMAN Cannabinoid receptor 1 OS=Homo sapiens GN=CNR1 PE=1 SV=1 | 401 | IIYALRsKDLR | 0.182 | -0.004 | -0.734 | -0.185 |
| sp\|P21554\|CNR1_HUMAN Cannabinoid receptor 1 OS=Homo sapiens GN=CNR1 PE=1 SV=1 | 410 | LRHAFRsMFPS | 0.092 | 0.102 | -1.284 | -0.363 |
| sp\|P21554\|CNR1_HUMAN Cannabinoid receptor 1 OS=Homo sapiens GN=CNR1 PE=1 SV=1 | 414 | FRSMFPsCEGT | 0.2 | 0.085 | -0.797 | -0.171 |
| sp\|P21554\|CNR1_HUMAN Cannabinoid receptor 1 OS=Homo sapiens GN=CNR1 PE=1 SV=1 | 418 | FPSCEGtAQPL | 0.143 | -0.119 | -1.29 | -0.422 |
| sp\|P21554\|CNR1_HUMAN Cannabinoid receptor 1 OS=Homo sapiens GN=CNR1 PE=1 SV=1 | 425 | AQPLDNsMGDS | 0.221 | 0.05 | -0.932 | -0.22 |
| sp\|P21554\|CNR1_HUMAN Cannabinoid receptor 1 OS=Homo sapiens GN=CNR1 PE=1 SV=1 | 429 | DNSMGDsDCLH | 0.058 | -0.524 | -1.912 | -0.793 |
| sp\|P21554\|CNR1_HUMAN Cannabinoid receptor 1 OS=Homo sapiens GN=CNR1 PE=1 SV=1 | 441 | HANNAAsVHRA | 0.287 | 0.002 | -0.642 | -0.118 |
| sp\|P21554\|CNR1_HUMAN Cannabinoid receptor 1 OS=Homo sapiens GN=CNR1 PE=1 SV=1 | 448 | VHRAAEsCIKS | 0.253 | 0.181 | -0.934 | -0.167 |
| sp\|P21554\|CNR1_HUMAN Cannabinoid receptor 1 OS=Homo sapiens GN=CNR1 PE=1 SV=1 | 452 | AESCIKsTVKI | 0.253 | -0.059 | -0.645 | -0.15 |
| sp\|P21554\|CNR1_HUMAN Cannabinoid receptor 1 OS=Homo sapiens GN=CNR1 PE=1 SV=1 | 453 | ESCIKStVKIA | 0.066 | -0.248 | -1.236 | -0.473 |
| sp\|P21554\|CNR1_HUMAN Cannabinoid receptor 1 OS=Homo sapiens GN=CNR1 PE=1 SV=1 | 460 | VKIAKVtMSVS | 0.197 | -0.004 | -0.967 | -0.258 |
| sp\|P21554\|CNR1_HUMAN Cannabinoid receptor 1 OS=Homo sapiens GN=CNR1 PE=1 SV=1 | 462 | IAKVTMsVSTD | 0.186 | 0.038 | -0.617 | -0.131 |
| sp\|P21554\|CNR1_HUMAN Cannabinoid receptor 1 OS=Homo sapiens GN=CNR1 PE=1 SV=1 | 464 | KVTMSVsTDTS | 0.42 | 0.099 | -0.225 | 0.098 |
| sp\|P21554\|CNR1_HUMAN Cannabinoid receptor 1 OS=Homo sapiens GN=CNR1 PE=1 SV=1 | 465 | VTMSVStDTSA | 0.1 | -0.243 | -1.428 | -0.524 |
| sp\|P21554\|CNR1_HUMAN Cannabinoid receptor 1 OS=Homo sapiens GN=CNR1 PE=1 SV=1 | 467 | MSVSTDtSAEA | 0.093 | -0.228 | -1.342 | -0.492 |
| sp\|P21554\|CNR1_HUMAN Cannabinoid receptor 1 OS=Homo sapiens GN=CNR1 PE=1 SV=1 | 468 | SVSTDTsAEAL | 0.122 | -0.041 | -1.091 | -0.337 |
| sp\|P34972\|CNR2_HUMAN Cannabinoid receptor 2 OS=Homo sapiens GN=CNR2 PE=1 SV=1 | 303 | VIYALRsGEIR | 0.09 | -0.155 | -1.257 | -0.441 |
| sp\|P34972\|CNR2_HUMAN Cannabinoid receptor 2 OS=Homo sapiens GN=CNR2 PE=1 SV=1 | 308 | RSGEIRsSAHH | 0.027 | -0.398 | -1.892 | -0.754 |
| sp\|P34972\|CNR2_HUMAN Cannabinoid receptor 2 OS=Homo sapiens GN=CNR2 PE=1 SV=1 | 309 | SGEIRSsAHHC | 0.11 | -0.109 | -1.271 | -0.423 |
| sp\|P34972\|CNR2_HUMAN Cannabinoid receptor 2 OS=Homo sapiens GN=CNR2 PE=1 SV=1 | 326 | CVRGLGsEAKE | 0.144 | 0.076 | -0.896 | -0.225 |
| sp\|P34972\|CNR2_HUMAN Cannabinoid receptor 2 OS=Homo sapiens GN=CNR2 PE=1 SV=1 | 335 | KEEAPRsSVTE | 0.071 | -0.35 | -1.562 | -0.614 |
| sp\|P34972\|CNR2_HUMAN Cannabinoid receptor 2 OS=Homo sapiens GN=CNR2 PE=1 SV=1 | 336 | EEAPRSsVTET | 0.17 | -0.076 | -0.827 | -0.244 |
| sp\|P34972\|CNR2_HUMAN Cannabinoid receptor 2 OS=Homo sapiens GN=CNR2 PE=1 SV=1 | 338 | APRSSVtETEA | 0.772 | 0.367 | 0.183 | 0.441 |
| sp\|P34972\|CNR2_HUMAN Cannabinoid receptor 2 OS=Homo sapiens GN=CNR2 PE=1 SV=1 | 340 | RSSVTEtEADG | 0.136 | -0.047 | -1.073 | -0.328 |
| sp\|P34972\|CNR2_HUMAN Cannabinoid receptor 2 OS=Homo sapiens GN=CNR2 PE=1 SV=1 | 347 | EADGKItPWPD | 0.057 | -0.466 | -1.408 | -0.606 |
| sp\|P34972\|CNR2_HUMAN Cannabinoid receptor 2 OS=Homo sapiens GN=CNR2 PE=1 SV=1 | 352 | ITPWPDsRDLD | 0.197 | -0.317 | -0.928 | -0.349 |
| sp\|P34972\|CNR2_HUMAN Cannabinoid receptor 2 OS=Homo sapiens GN=CNR2 PE=1 SV=1 | 358 | SRDLDLsDC-- | 0.076 | 0.078 | -1.541 | -0.462 |
| sp\|Q14330\|GPR18_HUMAN N-arachidonyl glycine receptor OS=Homo sapiens GN=GPR18 PE=1 SV=2 | 290 | ILYYIVsKQFQ | 0.35 | -0.017 | -0.182 | 0.05 |
| sp\|Q14330\|GPR18_HUMAN N-arachidonyl glycine receptor OS=Homo sapiens GN=GPR18 PE=1 SV=2 | 299 | FQARVIsVMLY | 0.75 | 0.974 | 0.619 | 0.781 |
| sp\|Q14330\|GPR18_HUMAN N-arachidonyl glycine receptor OS=Homo sapiens GN=GPR18 PE=1 SV=2 | 309 | YRNYLRsMRRK | 0.138 | 0.074 | -1.212 | -0.333 |
| sp\|Q14330\|GPR18_HUMAN N-arachidonyl glycine receptor OS=Homo sapiens GN=GPR18 PE=1 SV=2 | 314 | RSMRRKsFRSG | 0.837 | 1.056 | 0.97 | 0.954 |

| | | | | | | |
|---|---|---|---|---|---|---|
| sp\|Q14330\|GPR18_HUMAN N-arachidonyl glycine receptor OS=Homo sapiens GN=GPR18 PE=1 SV=2 | 317 | RRKSFRsGSLR | 0.147 | 0.079 | -1.028 | -0.267 |
| sp\|Q14330\|GPR18_HUMAN N-arachidonyl glycine receptor OS=Homo sapiens GN=GPR18 PE=1 SV=2 | 319 | KSFRSGsLRSL | 0.714 | 0.938 | 0.609 | 0.754 |
| sp\|Q14330\|GPR18_HUMAN N-arachidonyl glycine receptor OS=Homo sapiens GN=GPR18 PE=1 SV=2 | 322 | RSGSLRsLSNI | 0.131 | -0.07 | -1.163 | -0.367 |
| sp\|Q14330\|GPR18_HUMAN N-arachidonyl glycine receptor OS=Homo sapiens GN=GPR18 PE=1 SV=2 | 324 | GSLRSLsNINS | 0.6 | 0.969 | 0.239 | 0.603 |
| sp\|Q14330\|GPR18_HUMAN N-arachidonyl glycine receptor OS=Homo sapiens GN=GPR18 PE=1 SV=2 | 328 | SLSNINsEML- | 0.274 | 0.143 | -0.539 | -0.041 |
| sp\|Q9Y2T6\|GPR55_HUMAN G-protein coupled receptor 55 OS=Homo sapiens GN=GPR55 PE=1 SV=2 | 305 | IRAHRPsRVQL | 0.356 | 0.182 | -0.278 | 0.087 |
| sp\|Q9Y2T6\|GPR55_HUMAN G-protein coupled receptor 55 OS=Homo sapiens GN=GPR55 PE=1 SV=2 | 314 | QLVLQDtTISR | 0.079 | -0.116 | -1.3 | -0.446 |
| sp\|Q9Y2T6\|GPR55_HUMAN G-protein coupled receptor 55 OS=Homo sapiens GN=GPR55 PE=1 SV=2 | 315 | LVLQDTtISRG | 0.096 | -0.252 | -1.015 | -0.39 |
| sp\|Q9Y2T6\|GPR55_HUMAN G-protein coupled receptor 55 OS=Homo sapiens GN=GPR55 PE=1 SV=2 | 317 | LQDTTIsRG-- | 0.12 | 0.002 | -0.953 | -0.277 |
| sp\|Q8TDV5\|GP119_HUMAN Glucose-dependent insulinotropic receptor OS=Homo sapiens GN=GPR119 PE=1 | 302 | GVKKVLtSFLL | 0.301 | 0.085 | -0.527 | -0.047 |
| sp\|Q8TDV5\|GP119_HUMAN Glucose-dependent insulinotropic receptor OS=Homo sapiens GN=GPR119 PE=1 | 303 | VKKVLTsFLLF | 0.182 | 0.008 | -0.675 | -0.162 |
| sp\|Q8TDV5\|GP119_HUMAN Glucose-dependent insulinotropic receptor OS=Homo sapiens GN=GPR119 PE=1 | 309 | SFLLFLsARNC | 0.09 | -0.193 | -1.629 | -0.577 |
| sp\|Q8TDV5\|GP119_HUMAN Glucose-dependent insulinotropic receptor OS=Homo sapiens GN=GPR119 PE=1 | 321 | PERPREsSCHI | 0.35 | 0.125 | -0.55 | -0.025 |
| sp\|Q8TDV5\|GP119_HUMAN Glucose-dependent insulinotropic receptor OS=Homo sapiens GN=GPR119 PE=1 | 322 | ERPRESsCHIV | 0.485 | 0.98 | -0.266 | 0.4 |
| sp\|Q8TDV5\|GP119_HUMAN Glucose-dependent insulinotropic receptor OS=Homo sapiens GN=GPR119 PE=1 | 327 | SSCHIVtISSS | 0.057 | -0.346 | -1.418 | -0.569 |
| sp\|Q8TDV5\|GP119_HUMAN Glucose-dependent insulinotropic receptor OS=Homo sapiens GN=GPR119 PE=1 | 329 | CHIVTIsSSEF | 0.059 | -0.096 | -1.481 | -0.506 |
| sp\|Q8TDV5\|GP119_HUMAN Glucose-dependent insulinotropic receptor OS=Homo sapiens GN=GPR119 PE=1 | 330 | HIVTISsSEFD | 0.089 | -0.091 | -1.061 | -0.354 |
| sp\|Q8TDV5\|GP119_HUMAN Glucose-dependent insulinotropic receptor OS=Homo sapiens GN=GPR119 PE=1 | 331 | IVTISSsEFDG | 0.452 | 0.293 | 0.052 | 0.266 |
| sp\|P25105\|PTAFR_HUMAN Platelet-activating factor receptor OS=Homo sapiens GN=PTAFR PE=1 SV=1 | 297 | VIYCFLtKKFR | 0.098 | -0.167 | -1.148 | -0.406 |
| sp\|P25105\|PTAFR_HUMAN Platelet-activating factor receptor OS=Homo sapiens GN=PTAFR PE=1 SV=1 | 305 | KFRKHLtEKFY | 0.516 | 0.292 | -0.205 | 0.201 |
| sp\|P25105\|PTAFR_HUMAN Platelet-activating factor receptor OS=Homo sapiens GN=PTAFR PE=1 SV=1 | 310 | LTEKFYsMRSS | 0.191 | 0.007 | -0.838 | -0.213 |
| sp\|P25105\|PTAFR_HUMAN Platelet-activating factor receptor OS=Homo sapiens GN=PTAFR PE=1 SV=1 | 313 | KFYSMRsSRKC | 0.057 | -0.383 | -1.622 | -0.649 |
| sp\|P25105\|PTAFR_HUMAN Platelet-activating factor receptor OS=Homo sapiens GN=PTAFR PE=1 SV=1 | 314 | FYSMRSsRKCS | 0.198 | 0.014 | -0.895 | -0.228 |
| sp\|P25105\|PTAFR_HUMAN Platelet-activating factor receptor OS=Homo sapiens GN=PTAFR PE=1 SV=1 | 318 | RSSRKCsRATT | 0.405 | 0.536 | -0.374 | 0.189 |
| sp\|P25105\|PTAFR_HUMAN Platelet-activating factor receptor OS=Homo sapiens GN=PTAFR PE=1 SV=1 | 321 | RKCSRAtTDTV | 0.635 | 0.221 | -0.344 | 0.171 |
| sp\|P25105\|PTAFR_HUMAN Platelet-activating factor receptor OS=Homo sapiens GN=PTAFR PE=1 SV=1 | 322 | KCSRATtDTVT | 0.761 | 0.904 | 0.311 | 0.659 |
| sp\|P25105\|PTAFR_HUMAN Platelet-activating factor receptor OS=Homo sapiens GN=PTAFR PE=1 SV=1 | 324 | SRATTDtVTEV | 0.176 | 0.076 | -1.218 | -0.322 |
| sp\|P25105\|PTAFR_HUMAN Platelet-activating factor receptor OS=Homo sapiens GN=PTAFR PE=1 SV=1 | 326 | ATTDTVtEVVV | 0.294 | 0.068 | -0.953 | -0.197 |
| sp\|P25105\|PTAFR_HUMAN Platelet-activating factor receptor OS=Homo sapiens GN=PTAFR PE=1 SV=1 | 339 | NQIPGNsLKN- | 0.195 | 0.048 | -0.753 | -0.17 |
| sp\|Q13258\|PD2R_HUMAN Prostaglandin D2 receptor OS=Homo sapiens GN=PTGDR PE=2 SV=2 | 328 | IFIIFRsPVFR | 0.053 | -0.372 | -1.538 | -0.619 |
| sp\|Q13258\|PD2R_HUMAN Prostaglandin D2 receptor OS=Homo sapiens GN=PTGDR PE=2 SV=2 | 347 | RPLRYRsRCSN | 0.313 | 0.632 | -0.548 | 0.132 |
| sp\|Q13258\|PD2R_HUMAN Prostaglandin D2 receptor OS=Homo sapiens GN=PTGDR PE=2 SV=2 | 350 | RYRSRCsNSTN | 0.584 | 0.302 | -0.548 | 0.113 |
| sp\|Q13258\|PD2R_HUMAN Prostaglandin D2 receptor OS=Homo sapiens GN=PTGDR PE=2 SV=2 | 352 | RSRCSNsTNME | 0.718 | 0.371 | 0.026 | 0.372 |
| sp\|Q13258\|PD2R_HUMAN Prostaglandin D2 receptor OS=Homo sapiens GN=PTGDR PE=2 SV=2 | 353 | SRCSNStNMES | 0.108 | -0.038 | -1.377 | -0.436 |
| sp\|Q13258\|PD2R_HUMAN Prostaglandin D2 receptor OS=Homo sapiens GN=PTGDR PE=2 SV=2 | 357 | NSTNMEsSL-- | 0.075 | -0.142 | -1.236 | -0.434 |
| sp\|Q13258\|PD2R_HUMAN Prostaglandin D2 receptor OS=Homo sapiens GN=PTGDR PE=2 SV=2 | 358 | STNMESsL--- | 0.16 | -0.042 | -1.198 | -0.36 |
| sp\|Q9Y5Y4\|PD2R2_HUMAN Prostaglandin D2 receptor 2 OS=Homo sapiens GN=PTGDR2 PE=1 SV=3 | 331 | SVLVDDsELGG | 0.065 | -0.235 | -1.491 | -0.554 |
| sp\|Q9Y5Y4\|PD2R2_HUMAN Prostaglandin D2 receptor 2 OS=Homo sapiens GN=PTGDR2 PE=1 SV=3 | 338 | ELGGAGsSRRR | 0.145 | -0.151 | -0.997 | -0.334 |
| sp\|Q9Y5Y4\|PD2R2_HUMAN Prostaglandin D2 receptor 2 OS=Homo sapiens GN=PTGDR2 PE=1 SV=3 | 339 | LGGAGSsRRRR | 0.049 | -0.471 | -1.532 | -0.651 |
| sp\|Q9Y5Y4\|PD2R2_HUMAN Prostaglandin D2 receptor 2 OS=Homo sapiens GN=PTGDR2 PE=1 SV=3 | 344 | SSRRRRtSSTA | 0.614 | 0.954 | 0.107 | 0.558 |
| sp\|Q9Y5Y4\|PD2R2_HUMAN Prostaglandin D2 receptor 2 OS=Homo sapiens GN=PTGDR2 PE=1 SV=3 | 345 | SRRRRTsSTAR | 0.905 | 1.44 | 1.116 | 1.154 |
| sp\|Q9Y5Y4\|PD2R2_HUMAN Prostaglandin D2 receptor 2 OS=Homo sapiens GN=PTGDR2 PE=1 SV=3 | 346 | RRRRTSsTARS | 0.614 | 1.143 | 0.162 | 0.64 |
| sp\|Q9Y5Y4\|PD2R2_HUMAN Prostaglandin D2 receptor 2 OS=Homo sapiens GN=PTGDR2 PE=1 SV=3 | 347 | RRRTSStARSA | 0.492 | 0.518 | -0.167 | 0.281 |
| sp\|Q9Y5Y4\|PD2R2_HUMAN Prostaglandin D2 receptor 2 OS=Homo sapiens GN=PTGDR2 PE=1 SV=3 | 350 | TSSTARsASPL | 0.101 | -0.163 | -1.363 | -0.475 |
| sp\|Q9Y5Y4\|PD2R2_HUMAN Prostaglandin D2 receptor 2 OS=Homo sapiens GN=PTGDR2 PE=1 SV=3 | 352 | STARSAsPLAL | 0.592 | 0.809 | 0.289 | 0.563 |
| sp\|Q9Y5Y4\|PD2R2_HUMAN Prostaglandin D2 receptor 2 OS=Homo sapiens GN=PTGDR2 PE=1 SV=3 | 358 | SPLALCsRPEE | 0.384 | 0.193 | -0.792 | -0.072 |
| sp\|Q9Y5Y4\|PD2R2_HUMAN Prostaglandin D2 receptor 2 OS=Homo sapiens GN=PTGDR2 PE=1 SV=3 | 376 | LGWLLGsCAAS | 0.061 | -0.013 | -1.281 | -0.411 |
| sp\|Q9Y5Y4\|PD2R2_HUMAN Prostaglandin D2 receptor 2 OS=Homo sapiens GN=PTGDR2 PE=1 SV=3 | 380 | LGSCAAsPQTG | 0.212 | -0.289 | -0.836 | -0.304 |
| sp\|Q9Y5Y4\|PD2R2_HUMAN Prostaglandin D2 receptor 2 OS=Homo sapiens GN=PTGDR2 PE=1 SV=3 | 383 | CAASPQtGPLN | 0.408 | 0.305 | -0.036 | 0.226 |

| | | | | | | |
|---|---|---|---|---|---|---|
| sp\|Q9Y5Y4\|PD2R2_HUMAN Prostaglandin D2 receptor 2 OS=Homo sapiens GN=PTGDR2 PE=1 SV=3 | 391 | PLNRALsSTSS | 0.545 | 0.851 | 0.016 | 0.471 |
| sp\|Q9Y5Y4\|PD2R2_HUMAN Prostaglandin D2 receptor 2 OS=Homo sapiens GN=PTGDR2 PE=1 SV=3 | 392 | LNRALSsTSS- | 0.056 | -0.135 | -1.387 | -0.489 |
| sp\|Q9Y5Y4\|PD2R2_HUMAN Prostaglandin D2 receptor 2 OS=Homo sapiens GN=PTGDR2 PE=1 SV=3 | 393 | NRALSStSS-- | 0.186 | 0.366 | -0.654 | -0.034 |
| sp\|Q9Y5Y4\|PD2R2_HUMAN Prostaglandin D2 receptor 2 OS=Homo sapiens GN=PTGDR2 PE=1 SV=3 | 394 | RALSSTsS--- | 0.299 | 0.228 | -0.505 | 0.007 |
| sp\|Q9Y5Y4\|PD2R2_HUMAN Prostaglandin D2 receptor 2 OS=Homo sapiens GN=PTGDR2 PE=1 SV=3 | 395 | ALSSTSs---- | 0.118 | 0.032 | -1.089 | -0.313 |
| sp\|P34995\|PE2R1_HUMAN Prostaglandin E2 receptor EP1 subtype OS=Homo sapiens GN=PTGER1 PE=2 SV=3 | 382 | PAGLGLtPSAW | 0.021 | -0.471 | -1.864 | -0.771 |
| sp\|P34995\|PE2R1_HUMAN Prostaglandin E2 receptor EP1 subtype OS=Homo sapiens GN=PTGER1 PE=2 SV=3 | 384 | GLGLTPsAWEA | 0.331 | 0.135 | -0.714 | -0.083 |
| sp\|P34995\|PE2R1_HUMAN Prostaglandin E2 receptor EP1 subtype OS=Homo sapiens GN=PTGER1 PE=2 SV=3 | 389 | PSAWEAsSLRS | 0.14 | -0.278 | -1.263 | -0.467 |
| sp\|P34995\|PE2R1_HUMAN Prostaglandin E2 receptor EP1 subtype OS=Homo sapiens GN=PTGER1 PE=2 SV=3 | 390 | SAWEASsLRSS | 0.061 | -0.085 | -1.298 | -0.441 |
| sp\|P34995\|PE2R1_HUMAN Prostaglandin E2 receptor EP1 subtype OS=Homo sapiens GN=PTGER1 PE=2 SV=3 | 393 | EASSLRsSRHS | 0.028 | -0.441 | -1.84 | -0.751 |
| sp\|P34995\|PE2R1_HUMAN Prostaglandin E2 receptor EP1 subtype OS=Homo sapiens GN=PTGER1 PE=2 SV=3 | 394 | ASSLRSsRHSG | 0.118 | -0.192 | -1.319 | -0.464 |
| sp\|P34995\|PE2R1_HUMAN Prostaglandin E2 receptor EP1 subtype OS=Homo sapiens GN=PTGER1 PE=2 SV=3 | 397 | LRSSRHsGLSH | 0.121 | 0.123 | -1.052 | -0.269 |
| sp\|P34995\|PE2R1_HUMAN Prostaglandin E2 receptor EP1 subtype OS=Homo sapiens GN=PTGER1 PE=2 SV=3 | 400 | SRHSGLsHF-- | 0.057 | 0.065 | -1.415 | -0.431 |
| sp\|P43116\|PE2R2_HUMAN Prostaglandin E2 receptor EP2 subtype OS=Homo sapiens GN=PTGER2 PE=2 SV=2 | 328 | VLRLMRsVLCC | 0.252 | 0.161 | -0.912 | -0.166 |
| sp\|P43116\|PE2R2_HUMAN Prostaglandin E2 receptor EP2 subtype OS=Homo sapiens GN=PTGER2 PE=2 SV=2 | 335 | VLCCRIsLRTQ | 0.702 | 0.143 | -0.012 | 0.278 |
| sp\|P43116\|PE2R2_HUMAN Prostaglandin E2 receptor EP2 subtype OS=Homo sapiens GN=PTGER2 PE=2 SV=2 | 338 | CRISLRtQDAT | 0.153 | 0.133 | -0.988 | -0.234 |
| sp\|P43116\|PE2R2_HUMAN Prostaglandin E2 receptor EP2 subtype OS=Homo sapiens GN=PTGER2 PE=2 SV=2 | 342 | LRTQDAtQTSC | 0.163 | -0.001 | -1.089 | -0.309 |
| sp\|P43116\|PE2R2_HUMAN Prostaglandin E2 receptor EP2 subtype OS=Homo sapiens GN=PTGER2 PE=2 SV=2 | 344 | TQDATQtSCST | 0.028 | -0.343 | -1.584 | -0.633 |
| sp\|P43116\|PE2R2_HUMAN Prostaglandin E2 receptor EP2 subtype OS=Homo sapiens GN=PTGER2 PE=2 SV=2 | 345 | QDATQTsCSTQ | 0.049 | -0.246 | -1.512 | -0.57 |
| sp\|P43116\|PE2R2_HUMAN Prostaglandin E2 receptor EP2 subtype OS=Homo sapiens GN=PTGER2 PE=2 SV=2 | 347 | ATQTSCsTQSD | 0.434 | 0.151 | -0.562 | 0.008 |
| sp\|P43116\|PE2R2_HUMAN Prostaglandin E2 receptor EP2 subtype OS=Homo sapiens GN=PTGER2 PE=2 SV=2 | 348 | TQTSCStQSDA | 0.108 | -0.258 | -1.12 | -0.423 |
| sp\|P43116\|PE2R2_HUMAN Prostaglandin E2 receptor EP2 subtype OS=Homo sapiens GN=PTGER2 PE=2 SV=2 | 350 | TSCSTQsDASK | 0.063 | -0.255 | -1.464 | -0.552 |
| sp\|P43116\|PE2R2_HUMAN Prostaglandin E2 receptor EP2 subtype OS=Homo sapiens GN=PTGER2 PE=2 SV=2 | 353 | STQSDAsKQAD | 0.278 | -0.021 | -0.67 | -0.138 |
| sp\|P43115\|PE2R3_HUMAN Prostaglandin E2 receptor EP3 subtype OS=Homo sapiens GN=PTGER3 PE=1 SV=1 | 364 | CQIRYHtNNYA | 0.726 | 0.962 | 0.576 | 0.755 |
| sp\|P43115\|PE2R3_HUMAN Prostaglandin E2 receptor EP3 subtype OS=Homo sapiens GN=PTGER3 PE=1 SV=1 | 369 | HTNNYAsSSTS | 0.095 | -0.13 | -1.198 | -0.411 |
| sp\|P43115\|PE2R3_HUMAN Prostaglandin E2 receptor EP3 subtype OS=Homo sapiens GN=PTGER3 PE=1 SV=1 | 370 | TNNYASsSTSL | 0.074 | -0.191 | -1.235 | -0.451 |
| sp\|P43115\|PE2R3_HUMAN Prostaglandin E2 receptor EP3 subtype OS=Homo sapiens GN=PTGER3 PE=1 SV=1 | 371 | NNYASSsTSLP | 0.074 | -0.137 | -1.073 | -0.379 |
| sp\|P43115\|PE2R3_HUMAN Prostaglandin E2 receptor EP3 subtype OS=Homo sapiens GN=PTGER3 PE=1 SV=1 | 372 | NYASSStSLPC | 0.122 | -0.098 | -1.101 | -0.359 |
| sp\|P43115\|PE2R3_HUMAN Prostaglandin E2 receptor EP3 subtype OS=Homo sapiens GN=PTGER3 PE=1 SV=1 | 373 | YASSSTsLPCQ | 0.742 | 0.652 | 0.532 | 0.642 |
| sp\|P43115\|PE2R3_HUMAN Prostaglandin E2 receptor EP3 subtype OS=Homo sapiens GN=PTGER3 PE=1 SV=1 | 379 | SLPCQCsSTLM | 0.172 | -0.114 | -1.353 | -0.432 |
| sp\|P43115\|PE2R3_HUMAN Prostaglandin E2 receptor EP3 subtype OS=Homo sapiens GN=PTGER3 PE=1 SV=1 | 380 | LPCQCSsTLMW | 0.104 | -0.209 | -1.219 | -0.441 |
| sp\|P43115\|PE2R3_HUMAN Prostaglandin E2 receptor EP3 subtype OS=Homo sapiens GN=PTGER3 PE=1 SV=1 | 381 | PCQCSStLMWS | 0.579 | 0.332 | -0.07 | 0.28 |
| sp\|P43115\|PE2R3_HUMAN Prostaglandin E2 receptor EP3 subtype OS=Homo sapiens GN=PTGER3 PE=1 SV=1 | 385 | SSTLMWsDHLE | 0.179 | -0.211 | -1.14 | -0.391 |
| sp\|P35408\|PE2R4_HUMAN Prostaglandin E2 receptor EP4 subtype OS=Homo sapiens GN=PTGER4 PE=1 SV=1 | 335 | YILLRKtVLSK | 0.254 | 0.155 | -0.436 | -0.009 |
| sp\|P35408\|PE2R4_HUMAN Prostaglandin E2 receptor EP4 subtype OS=Homo sapiens GN=PTGER4 PE=1 SV=1 | 338 | LRKTVLsKAIE | 0.19 | 0.039 | -0.789 | -0.187 |
| sp\|P35408\|PE2R4_HUMAN Prostaglandin E2 receptor EP4 subtype OS=Homo sapiens GN=PTGER4 PE=1 SV=1 | 354 | FCRIGGsRRER | 0.288 | 0.092 | -0.827 | -0.149 |
| sp\|P35408\|PE2R4_HUMAN Prostaglandin E2 receptor EP4 subtype OS=Homo sapiens GN=PTGER4 PE=1 SV=1 | 359 | GSRRERsGQHC | 0.582 | 0.842 | -0.015 | 0.47 |
| sp\|P35408\|PE2R4_HUMAN Prostaglandin E2 receptor EP4 subtype OS=Homo sapiens GN=PTGER4 PE=1 SV=1 | 364 | RSGQHCsDSQR | 0.308 | -0.046 | -0.817 | -0.185 |
| sp\|P35408\|PE2R4_HUMAN Prostaglandin E2 receptor EP4 subtype OS=Homo sapiens GN=PTGER4 PE=1 SV=1 | 366 | GQHCSDsQRTS | 0.219 | -0.087 | -1.03 | -0.299 |
| sp\|P35408\|PE2R4_HUMAN Prostaglandin E2 receptor EP4 subtype OS=Homo sapiens GN=PTGER4 PE=1 SV=1 | 369 | CSDSQRtSSAM | 0.062 | -0.232 | -1.444 | -0.538 |
| sp\|P35408\|PE2R4_HUMAN Prostaglandin E2 receptor EP4 subtype OS=Homo sapiens GN=PTGER4 PE=1 SV=1 | 370 | SDSQRTsSAMS | 0.084 | -0.247 | -1.33 | -0.498 |
| sp\|P35408\|PE2R4_HUMAN Prostaglandin E2 receptor EP4 subtype OS=Homo sapiens GN=PTGER4 PE=1 SV=1 | 371 | DSQRTSsAMSG | 0.373 | 0.788 | -0.328 | 0.278 |
| sp\|P35408\|PE2R4_HUMAN Prostaglandin E2 receptor EP4 subtype OS=Homo sapiens GN=PTGER4 PE=1 SV=1 | 374 | RTSSAMsGHSR | 0.272 | 0.032 | -0.704 | -0.133 |
| sp\|P35408\|PE2R4_HUMAN Prostaglandin E2 receptor EP4 subtype OS=Homo sapiens GN=PTGER4 PE=1 SV=1 | 377 | SAMSGHsRSFI | 0.059 | -0.269 | -1.609 | -0.606 |
| sp\|P35408\|PE2R4_HUMAN Prostaglandin E2 receptor EP4 subtype OS=Homo sapiens GN=PTGER4 PE=1 SV=1 | 379 | MSGHSRsFISR | 0.202 | 0.181 | -0.443 | -0.02 |
| sp\|P35408\|PE2R4_HUMAN Prostaglandin E2 receptor EP4 subtype OS=Homo sapiens GN=PTGER4 PE=1 SV=1 | 382 | HSRSFIsRELK | 0.234 | 0 | -0.78 | -0.182 |
| sp\|P35408\|PE2R4_HUMAN Prostaglandin E2 receptor EP4 subtype OS=Homo sapiens GN=PTGER4 PE=1 SV=1 | 389 | RELKEIsSTSQ | 0.203 | -0.159 | -1.196 | -0.384 |
| sp\|P35408\|PE2R4_HUMAN Prostaglandin E2 receptor EP4 subtype OS=Homo sapiens GN=PTGER4 PE=1 SV=1 | 390 | ELKEISsTSQT | 0.07 | -0.166 | -0.955 | -0.35 |
| sp\|P35408\|PE2R4_HUMAN Prostaglandin E2 receptor EP4 subtype OS=Homo sapiens GN=PTGER4 PE=1 SV=1 | 391 | LKEISStSQTL | 0.341 | 0.172 | -0.219 | 0.098 |

| | | | | | | |
|---|---|---|---|---|---|---|
| sp\|P35408\|PE2R4_HUMAN Prostaglandin E2 receptor EP4 subtype OS=Homo sapiens GN=PTGER4 PE=1 SV=1 | 392 | KEISSTsQTLL | 0.255 | 0.08 | -0.865 | -0.177 |
| sp\|P35408\|PE2R4_HUMAN Prostaglandin E2 receptor EP4 subtype OS=Homo sapiens GN=PTGER4 PE=1 SV=1 | 394 | ISSTSQtLLPD | 0.459 | 0.116 | -0.028 | 0.182 |
| sp\|P35408\|PE2R4_HUMAN Prostaglandin E2 receptor EP4 subtype OS=Homo sapiens GN=PTGER4 PE=1 SV=1 | 400 | TLLPDLsLPDL | 0.738 | 0.592 | 0.566 | 0.632 |
| sp\|P35408\|PE2R4_HUMAN Prostaglandin E2 receptor EP4 subtype OS=Homo sapiens GN=PTGER4 PE=1 SV=1 | 405 | LSLPDLsENGL | 0.116 | -0.144 | -0.691 | -0.24 |
| sp\|P35408\|PE2R4_HUMAN Prostaglandin E2 receptor EP4 subtype OS=Homo sapiens GN=PTGER4 PE=1 SV=1 | 428 | GLAQEDtTSLR | 0.257 | -0.071 | -0.793 | -0.202 |
| sp\|P35408\|PE2R4_HUMAN Prostaglandin E2 receptor EP4 subtype OS=Homo sapiens GN=PTGER4 PE=1 SV=1 | 429 | LAQEDTtSLRT | 0.052 | -0.296 | -1.589 | -0.611 |
| sp\|P35408\|PE2R4_HUMAN Prostaglandin E2 receptor EP4 subtype OS=Homo sapiens GN=PTGER4 PE=1 SV=1 | 430 | AQEDTTsLRTL | 0.249 | -0.012 | -0.454 | -0.072 |
| sp\|P35408\|PE2R4_HUMAN Prostaglandin E2 receptor EP4 subtype OS=Homo sapiens GN=PTGER4 PE=1 SV=1 | 433 | DTTSLRtLRIS | 0.231 | -0.177 | -1.084 | -0.343 |
| sp\|P35408\|PE2R4_HUMAN Prostaglandin E2 receptor EP4 subtype OS=Homo sapiens GN=PTGER4 PE=1 SV=1 | 437 | LRTLRIsETSD | 0.461 | 0.381 | -0.144 | 0.233 |
| sp\|P35408\|PE2R4_HUMAN Prostaglandin E2 receptor EP4 subtype OS=Homo sapiens GN=PTGER4 PE=1 SV=1 | 439 | TLRISEtSDSS | 0.414 | 0.392 | -0.304 | 0.167 |
| sp\|P35408\|PE2R4_HUMAN Prostaglandin E2 receptor EP4 subtype OS=Homo sapiens GN=PTGER4 PE=1 SV=1 | 440 | LRISETsDSSQ | 0.074 | -0.021 | -1.444 | -0.464 |
| sp\|P35408\|PE2R4_HUMAN Prostaglandin E2 receptor EP4 subtype OS=Homo sapiens GN=PTGER4 PE=1 SV=1 | 442 | ISETSDsSQGQ | 0.295 | -0.123 | -0.645 | -0.158 |
| sp\|P35408\|PE2R4_HUMAN Prostaglandin E2 receptor EP4 subtype OS=Homo sapiens GN=PTGER4 PE=1 SV=1 | 443 | SETSDSsQGQD | 0.103 | -0.285 | -1.385 | -0.522 |
| sp\|P35408\|PE2R4_HUMAN Prostaglandin E2 receptor EP4 subtype OS=Homo sapiens GN=PTGER4 PE=1 SV=1 | 448 | SSQGQDsESVL | 0.061 | -0.119 | -1.502 | -0.52 |
| sp\|P35408\|PE2R4_HUMAN Prostaglandin E2 receptor EP4 subtype OS=Homo sapiens GN=PTGER4 PE=1 SV=1 | 450 | QGQDSEsVLLV | 0.249 | 0.167 | -0.843 | -0.142 |
| sp\|P35408\|PE2R4_HUMAN Prostaglandin E2 receptor EP4 subtype OS=Homo sapiens GN=PTGER4 PE=1 SV=1 | 460 | VDEAGGsGRAG | 0.059 | -0.402 | -1.275 | -0.539 |
| sp\|P35408\|PE2R4_HUMAN Prostaglandin E2 receptor EP4 subtype OS=Homo sapiens GN=PTGER4 PE=1 SV=1 | 470 | GPAPKGsSLQV | 0.059 | -0.319 | -1.672 | -0.644 |
| sp\|P35408\|PE2R4_HUMAN Prostaglandin E2 receptor EP4 subtype OS=Homo sapiens GN=PTGER4 PE=1 SV=1 | 471 | PAPKGSsLQVT | 0.167 | -0.112 | -0.78 | -0.242 |
| sp\|P35408\|PE2R4_HUMAN Prostaglandin E2 receptor EP4 subtype OS=Homo sapiens GN=PTGER4 PE=1 SV=1 | 475 | GSSLQVtFPSE | 0.546 | 0.397 | -0.165 | 0.259 |
| sp\|P35408\|PE2R4_HUMAN Prostaglandin E2 receptor EP4 subtype OS=Homo sapiens GN=PTGER4 PE=1 SV=1 | 478 | LQVTFPsETLN | 0.163 | -0.072 | -0.967 | -0.292 |
| sp\|P35408\|PE2R4_HUMAN Prostaglandin E2 receptor EP4 subtype OS=Homo sapiens GN=PTGER4 PE=1 SV=1 | 480 | VTFPSEtLNLS | 0.444 | 0.156 | -0.24 | 0.12 |
| sp\|P35408\|PE2R4_HUMAN Prostaglandin E2 receptor EP4 subtype OS=Homo sapiens GN=PTGER4 PE=1 SV=1 | 484 | SETLNLsEKCI | 0.137 | 0.001 | -0.907 | -0.256 |
| sp\|P43088\|PF2R_HUMAN Prostaglandin F2-alpha receptor OS=Homo sapiens GN=PTGFR PE=1 SV=1 | 320 | NLYKLAsQCCG | 0.287 | 0.011 | -0.914 | -0.205 |
| sp\|P43088\|PF2R_HUMAN Prostaglandin F2-alpha receptor OS=Homo sapiens GN=PTGFR PE=1 SV=1 | 329 | CGVHVIsLHIW | 0.462 | 0.077 | -0.202 | 0.112 |
| sp\|P43088\|PF2R_HUMAN Prostaglandin F2-alpha receptor OS=Homo sapiens GN=PTGFR PE=1 SV=1 | 336 | LHIWELsSIKN | 0.054 | -0.195 | -1.576 | -0.572 |
| sp\|P43088\|PF2R_HUMAN Prostaglandin F2-alpha receptor OS=Homo sapiens GN=PTGFR PE=1 SV=1 | 337 | HIWELSsIKNS | 0.034 | 0.022 | -1.478 | -0.474 |
| sp\|P43088\|PF2R_HUMAN Prostaglandin F2-alpha receptor OS=Homo sapiens GN=PTGFR PE=1 SV=1 | 341 | LSSIKNsLKVA | 0.211 | -0.045 | -0.61 | -0.148 |
| sp\|P43088\|PF2R_HUMAN Prostaglandin F2-alpha receptor OS=Homo sapiens GN=PTGFR PE=1 SV=1 | 348 | LKVAAIsESPV | 0.321 | 0.03 | -0.63 | -0.093 |
| sp\|P43088\|PF2R_HUMAN Prostaglandin F2-alpha receptor OS=Homo sapiens GN=PTGFR PE=1 SV=1 | 350 | VAAISEsPVAE | 0.122 | -0.197 | -0.956 | -0.344 |
| sp\|P43088\|PF2R_HUMAN Prostaglandin F2-alpha receptor OS=Homo sapiens GN=PTGFR PE=1 SV=1 | 356 | SPVAEKsAST- | 0.094 | -0.173 | -1.437 | -0.505 |
| sp\|P43088\|PF2R_HUMAN Prostaglandin F2-alpha receptor OS=Homo sapiens GN=PTGFR PE=1 SV=1 | 358 | VAEKSAsT--- | 0.544 | 0.31 | -0.158 | 0.232 |
| sp\|P43088\|PF2R_HUMAN Prostaglandin F2-alpha receptor OS=Homo sapiens GN=PTGFR PE=1 SV=1 | 359 | AEKSASt---- | 0.194 | 0.023 | -0.982 | -0.255 |
| sp\|P43119\|PI2R_HUMAN Prostacyclin receptor OS=Homo sapiens GN=PTGIR PE=1 SV=1 | 319 | GPAHGDsQTPL | 0.109 | -0.296 | -1.428 | -0.538 |
| sp\|P43119\|PI2R_HUMAN Prostacyclin receptor OS=Homo sapiens GN=PTGIR PE=1 SV=1 | 321 | AHGDSQtPLSQ | 0.046 | -0.284 | -1.818 | -0.685 |
| sp\|P43119\|PI2R_HUMAN Prostacyclin receptor OS=Homo sapiens GN=PTGIR PE=1 SV=1 | 324 | DSQTPLsQLAS | 0.079 | -0.246 | -1.517 | -0.561 |
| sp\|P43119\|PI2R_HUMAN Prostacyclin receptor OS=Homo sapiens GN=PTGIR PE=1 SV=1 | 328 | PLSQLAsGRRD | 0.372 | -0.112 | -0.551 | -0.097 |
| sp\|P43119\|PI2R_HUMAN Prostacyclin receptor OS=Homo sapiens GN=PTGIR PE=1 SV=1 | 337 | RDPRAPsAPVG | 0.858 | 1.318 | 0.775 | 0.984 |
| sp\|P43119\|PI2R_HUMAN Prostacyclin receptor OS=Homo sapiens GN=PTGIR PE=1 SV=1 | 345 | PVGKEGsCVPL | 0.121 | -0.034 | -1.121 | -0.345 |
| sp\|P43119\|PI2R_HUMAN Prostacyclin receptor OS=Homo sapiens GN=PTGIR PE=1 SV=1 | 350 | GSCVPLsAWGE | 0.133 | -0.197 | -1.199 | -0.421 |
| sp\|P43119\|PI2R_HUMAN Prostacyclin receptor OS=Homo sapiens GN=PTGIR PE=1 SV=1 | 363 | VEPLPPtQQSS | 0.035 | -0.423 | -1.914 | -0.767 |
| sp\|P43119\|PI2R_HUMAN Prostacyclin receptor OS=Homo sapiens GN=PTGIR PE=1 SV=1 | 366 | LPPTQQsSGSA | 0.054 | -0.231 | -1.439 | -0.539 |
| sp\|P43119\|PI2R_HUMAN Prostacyclin receptor OS=Homo sapiens GN=PTGIR PE=1 SV=1 | 367 | PPTQQSsGSAV | 0.08 | -0.168 | -1.253 | -0.447 |
| sp\|P43119\|PI2R_HUMAN Prostacyclin receptor OS=Homo sapiens GN=PTGIR PE=1 SV=1 | 369 | TQQSSGsAVGT | 0.141 | -0.005 | -0.798 | -0.221 |
| sp\|P43119\|PI2R_HUMAN Prostacyclin receptor OS=Homo sapiens GN=PTGIR PE=1 SV=1 | 373 | SGSAVGtSSKA | 0.047 | -0.425 | -1.769 | -0.716 |
| sp\|P43119\|PI2R_HUMAN Prostacyclin receptor OS=Homo sapiens GN=PTGIR PE=1 SV=1 | 374 | GSAVGTsSKAE | 0.094 | -0.181 | -1.018 | -0.368 |
| sp\|P43119\|PI2R_HUMAN Prostacyclin receptor OS=Homo sapiens GN=PTGIR PE=1 SV=1 | 375 | SAVGTSsKAEA | 0.048 | -0.259 | -1.464 | -0.558 |
| sp\|P43119\|PI2R_HUMAN Prostacyclin receptor OS=Homo sapiens GN=PTGIR PE=1 SV=1 | 380 | SSKAEAsVACS | 0.119 | -0.18 | -1.273 | -0.445 |
| sp\|P43119\|PI2R_HUMAN Prostacyclin receptor OS=Homo sapiens GN=PTGIR PE=1 SV=1 | 384 | EASVACsLC-- | 0.249 | 0.068 | -0.809 | -0.164 |
| sp\|P21731\|TA2R_HUMAN Thromboxane A2 receptor OS=Homo sapiens GN=TBXA2R PE=1 SV=3 | 324 | RLQPRLsTRPR | 0.345 | 0.088 | -0.435 | -0.001 |

| | | | | | | |
|---|---|---|---|---|---|---|
| sp\|P21731\|TA2R_HUMAN Thromboxane A2 receptor OS=Homo sapiens GN=TBXA2R PE=1 SV=3 | 325 | LQPRLStRPRS | 0.478 | 0.95 | 0.026 | 0.485 |
| sp\|P21731\|TA2R_HUMAN Thromboxane A2 receptor OS=Homo sapiens GN=TBXA2R PE=1 SV=3 | 329 | LSTRPRsLSLQ | 0.428 | 0.684 | 0.098 | 0.403 |
| sp\|P21731\|TA2R_HUMAN Thromboxane A2 receptor OS=Homo sapiens GN=TBXA2R PE=1 SV=3 | 331 | TRPRSLsLQPQ | 0.722 | 1.159 | 0.761 | 0.881 |
| sp\|P21731\|TA2R_HUMAN Thromboxane A2 receptor OS=Homo sapiens GN=TBXA2R PE=1 SV=3 | 337 | SLQPQLtQRSG | 0.055 | -0.212 | -1.614 | -0.59 |
| sp\|P21731\|TA2R_HUMAN Thromboxane A2 receptor OS=Homo sapiens GN=TBXA2R PE=1 SV=3 | 340 | PQLTQRsGLQ- | 0.064 | -0.163 | -1.375 | -0.491 |
| sp\|P48039\|MTR1A_HUMAN Melatonin receptor type 1A OS=Homo sapiens GN=MTNR1A PE=1 SV=1 | 312 | YRRIIVsLCTA | 0.65 | 0.424 | 0.339 | 0.471 |
| sp\|P48039\|MTR1A_HUMAN Melatonin receptor type 1A OS=Homo sapiens GN=MTNR1A PE=1 SV=1 | 315 | IIVSLCtARVF | 0.187 | -0.07 | -1.107 | -0.33 |
| sp\|P48039\|MTR1A_HUMAN Melatonin receptor type 1A OS=Homo sapiens GN=MTNR1A PE=1 SV=1 | 323 | RVFFVDsSNDV | 0.328 | 0.018 | -0.931 | -0.195 |
| sp\|P48039\|MTR1A_HUMAN Melatonin receptor type 1A OS=Homo sapiens GN=MTNR1A PE=1 SV=1 | 324 | VFFVDSsNDVA | 0.096 | -0.163 | -1.154 | -0.407 |
| sp\|P48039\|MTR1A_HUMAN Melatonin receptor type 1A OS=Homo sapiens GN=MTNR1A PE=1 SV=1 | 336 | RVKWKPsPLMT | 0.05 | -0.528 | -1.497 | -0.658 |
| sp\|P48039\|MTR1A_HUMAN Melatonin receptor type 1A OS=Homo sapiens GN=MTNR1A PE=1 SV=1 | 340 | KPSPLMtNNNV | 0.151 | -0.08 | -1.406 | -0.445 |
| sp\|P48039\|MTR1A_HUMAN Melatonin receptor type 1A OS=Homo sapiens GN=MTNR1A PE=1 SV=1 | 349 | NVVKVDsV--- | 0.271 | 0.106 | -0.828 | -0.15 |
| sp\|P49286\|MTR1B_HUMAN Melatonin receptor type 1B OS=Homo sapiens GN=MTNR1B PE=1 SV=1 | 337 | HCIQDAsKGSH | 0.204 | -0.025 | -0.861 | -0.227 |
| sp\|P49286\|MTR1B_HUMAN Melatonin receptor type 1B OS=Homo sapiens GN=MTNR1B PE=1 SV=1 | 340 | QDASKGsHAEG | 0.058 | -0.366 | -1.446 | -0.585 |
| sp\|P49286\|MTR1B_HUMAN Melatonin receptor type 1B OS=Homo sapiens GN=MTNR1B PE=1 SV=1 | 347 | HAEGLQsPAPP | 0.028 | -0.549 | -1.688 | -0.736 |
| sp\|P30542\|AA1R_HUMAN Adenosine receptor A1 OS=Homo sapiens GN=ADORA1 PE=1 SV=1 | 298 | IQKFRVtFLKI | 0.664 | 0.246 | 0.298 | 0.403 |
| sp\|P29274\|AA2AR_HUMAN Adenosine receptor A2a OS=Homo sapiens GN=ADORA2A PE=1 SV=2 | 298 | IREFRQtFRKI | 0.679 | 0.411 | 0.366 | 0.485 |
| sp\|P29274\|AA2AR_HUMAN Adenosine receptor A2a OS=Homo sapiens GN=ADORA2A PE=1 SV=2 | 305 | FRKIIRsHVLR | 0.301 | 0.342 | -0.386 | 0.086 |
| sp\|P29274\|AA2AR_HUMAN Adenosine receptor A2a OS=Homo sapiens GN=ADORA2A PE=1 SV=2 | 319 | PFKAAGtSARV | 0.231 | -0.157 | -1.033 | -0.32 |
| sp\|P29274\|AA2AR_HUMAN Adenosine receptor A2a OS=Homo sapiens GN=ADORA2A PE=1 SV=2 | 320 | FKAAGTsARVL | 0.317 | 0.075 | -0.453 | -0.02 |
| sp\|P29274\|AA2AR_HUMAN Adenosine receptor A2a OS=Homo sapiens GN=ADORA2A PE=1 SV=2 | 329 | VLAAHGsDGEQ | 0.404 | 0.021 | -0.58 | -0.052 |
| sp\|P29274\|AA2AR_HUMAN Adenosine receptor A2a OS=Homo sapiens GN=ADORA2A PE=1 SV=2 | 335 | SDGEQVsLRLN | 0.096 | -0.167 | -1.221 | -0.431 |
| sp\|P29274\|AA2AR_HUMAN Adenosine receptor A2a OS=Homo sapiens GN=ADORA2A PE=1 SV=2 | 350 | GVWANGsAPHP | 0.298 | 0.423 | -0.402 | 0.106 |
| sp\|P29274\|AA2AR_HUMAN Adenosine receptor A2a OS=Homo sapiens GN=ADORA2A PE=1 SV=2 | 367 | YALGLVsGGSA | 0.124 | -0.196 | -0.865 | -0.312 |
| sp\|P29274\|AA2AR_HUMAN Adenosine receptor A2a OS=Homo sapiens GN=ADORA2A PE=1 SV=2 | 370 | GLVSGGsAQES | 0.154 | -0.06 | -1.166 | -0.357 |
| sp\|P29274\|AA2AR_HUMAN Adenosine receptor A2a OS=Homo sapiens GN=ADORA2A PE=1 SV=2 | 374 | GGSAQEsQGNT | 0.151 | -0.155 | -1.365 | -0.456 |
| sp\|P29274\|AA2AR_HUMAN Adenosine receptor A2a OS=Homo sapiens GN=ADORA2A PE=1 SV=2 | 378 | QESQGNtGLPD | 0.107 | -0.237 | -1.171 | -0.434 |
| sp\|P29274\|AA2AR_HUMAN Adenosine receptor A2a OS=Homo sapiens GN=ADORA2A PE=1 SV=2 | 387 | PDVELLsHELK | 0.044 | -0.259 | -1.593 | -0.603 |
| sp\|P29274\|AA2AR_HUMAN Adenosine receptor A2a OS=Homo sapiens GN=ADORA2A PE=1 SV=2 | 412 | QDGAGVs---- | 0.083 | -0.128 | -1.188 | -0.411 |
| sp\|P29275\|AA2BR_HUMAN Adenosine receptor A2b OS=Homo sapiens GN=ADORA2B PE=2 SV=1 | 300 | NRDFRYtFHKI | 0.642 | 0.548 | 0.245 | 0.478 |
| sp\|P29275\|AA2BR_HUMAN Adenosine receptor A2b OS=Homo sapiens GN=ADORA2B PE=2 SV=1 | 306 | TFHKIIsRYLL | 0.222 | -0.044 | -0.592 | -0.138 |
| sp\|P29275\|AA2BR_HUMAN Adenosine receptor A2b OS=Homo sapiens GN=ADORA2B PE=2 SV=1 | 317 | CQADVKsGNGQ | 0.277 | -0.067 | -0.271 | -0.02 |
| sp\|P0DMS8\|AA3R_HUMAN Adenosine receptor A3 OS=Homo sapiens GN=ADORA3 PE=1 SV=1 | 292 | IKKFKEtYLLI | 0.302 | -0.004 | -0.534 | -0.079 |
| sp\|P0DMS8\|AA3R_HUMAN Adenosine receptor A3 OS=Homo sapiens GN=ADORA3 PE=1 SV=1 | 306 | CVVCHPsDSLD | 0.269 | 0.002 | -0.792 | -0.174 |
| sp\|P0DMS8\|AA3R_HUMAN Adenosine receptor A3 OS=Homo sapiens GN=ADORA3 PE=1 SV=1 | 308 | VCHPSDsLDTS | 0.391 | 0.105 | -0.438 | 0.019 |
| sp\|P0DMS8\|AA3R_HUMAN Adenosine receptor A3 OS=Homo sapiens GN=ADORA3 PE=1 SV=1 | 311 | PSDSLDtSIEK | 0.069 | -0.369 | -1.506 | -0.602 |
| sp\|P0DMS8\|AA3R_HUMAN Adenosine receptor A3 OS=Homo sapiens GN=ADORA3 PE=1 SV=1 | 312 | SDSLDTsIEKN | 0.048 | -0.324 | -1.673 | -0.65 |
| sp\|P0DMS8\|AA3R_HUMAN Adenosine receptor A3 OS=Homo sapiens GN=ADORA3 PE=1 SV=1 | 317 | TSIEKNsE--- | 0.122 | 0.096 | -1.139 | -0.307 |
| sp\|P47900\|P2RY1_HUMAN P2Y purinoceptor 1 OS=Homo sapiens GN=P2RY1 PE=1 SV=1 | 330 | YFLAGDtFRRR | 0.206 | -0.236 | -0.77 | -0.267 |
| sp\|P47900\|P2RY1_HUMAN P2Y purinoceptor 1 OS=Homo sapiens GN=P2RY1 PE=1 SV=1 | 336 | TFRRRLsRATR | 0.761 | 1.038 | 0.645 | 0.815 |
| sp\|P47900\|P2RY1_HUMAN P2Y purinoceptor 1 OS=Homo sapiens GN=P2RY1 PE=1 SV=1 | 339 | RRLSRAtRKAS | 0.403 | 0.295 | -0.379 | 0.106 |
| sp\|P47900\|P2RY1_HUMAN P2Y purinoceptor 1 OS=Homo sapiens GN=P2RY1 PE=1 SV=1 | 343 | RATRKAsRRSE | 0.409 | 0.596 | -0.24 | 0.255 |
| sp\|P47900\|P2RY1_HUMAN P2Y purinoceptor 1 OS=Homo sapiens GN=P2RY1 PE=1 SV=1 | 346 | RKASRRsEANL | 0.435 | 0.266 | -0.454 | 0.082 |
| sp\|P47900\|P2RY1_HUMAN P2Y purinoceptor 1 OS=Homo sapiens GN=P2RY1 PE=1 SV=1 | 352 | SEANLQsKSED | 0.078 | -0.188 | -1.143 | -0.418 |
| sp\|P47900\|P2RY1_HUMAN P2Y purinoceptor 1 OS=Homo sapiens GN=P2RY1 PE=1 SV=1 | 354 | ANLQSKsEDMT | 0.469 | 0.136 | 0.11 | 0.238 |
| sp\|P47900\|P2RY1_HUMAN P2Y purinoceptor 1 OS=Homo sapiens GN=P2RY1 PE=1 SV=1 | 358 | SKSEDMtLNIL | 0.447 | 0.19 | -0.186 | 0.15 |
| sp\|P47900\|P2RY1_HUMAN P2Y purinoceptor 1 OS=Homo sapiens GN=P2RY1 PE=1 SV=1 | 371 | FKQNGDtSL-- | 0.132 | 0.082 | -1.045 | -0.277 |
| sp\|P47900\|P2RY1_HUMAN P2Y purinoceptor 1 OS=Homo sapiens GN=P2RY1 PE=1 SV=1 | 372 | KQNGDTsL--- | 0.195 | 0.102 | -0.607 | -0.103 |
| sp\|P41231\|P2RY2_HUMAN P2Y purinoceptor 2 OS=Homo sapiens GN=P2RY2 PE=2 SV=4 | 324 | RDAKPPtGPSP | 0.243 | 0.155 | -0.416 | -0.006 |

| | | | | | | |
|---|---|---|---|---|---|---|
| sp\|P41231\|P2RY2_HUMAN P2Y purinoceptor 2 OS=Homo sapiens GN=P2RY2 PE=2 SV=4 | 327 | KPPTGPsPATP | 0.015 | -0.767 | -2.308 | -1.02 |
| sp\|P41231\|P2RY2_HUMAN P2Y purinoceptor 2 OS=Homo sapiens GN=P2RY2 PE=2 SV=4 | 330 | TGPSPAtPARR | 0.038 | -0.671 | -1.752 | -0.795 |
| sp\|P41231\|P2RY2_HUMAN P2Y purinoceptor 2 OS=Homo sapiens GN=P2RY2 PE=2 SV=4 | 341 | RLGLRRsDRTD | 0.472 | 0.195 | -0.515 | 0.051 |
| sp\|P41231\|P2RY2_HUMAN P2Y purinoceptor 2 OS=Homo sapiens GN=P2RY2 PE=2 SV=4 | 344 | LRRSDRtDMQR | 0.385 | 0.341 | -0.489 | 0.079 |
| sp\|P41231\|P2RY2_HUMAN P2Y purinoceptor 2 OS=Homo sapiens GN=P2RY2 PE=2 SV=4 | 355 | IEDVLGsSEDS | 0.056 | -0.273 | -1.684 | -0.634 |
| sp\|P41231\|P2RY2_HUMAN P2Y purinoceptor 2 OS=Homo sapiens GN=P2RY2 PE=2 SV=4 | 356 | EDVLGSsEDSR | 0.036 | -0.208 | -1.453 | -0.542 |
| sp\|P41231\|P2RY2_HUMAN P2Y purinoceptor 2 OS=Homo sapiens GN=P2RY2 PE=2 SV=4 | 359 | LGSSEDsRRTE | 0.083 | -0.49 | -1.535 | -0.647 |
| sp\|P41231\|P2RY2_HUMAN P2Y purinoceptor 2 OS=Homo sapiens GN=P2RY2 PE=2 SV=4 | 362 | SEDSRRtESTP | 0.062 | -0.155 | -1.653 | -0.582 |
| sp\|P41231\|P2RY2_HUMAN P2Y purinoceptor 2 OS=Homo sapiens GN=P2RY2 PE=2 SV=4 | 364 | DSRRTEsTPAG | 0.936 | 1.559 | 1.243 | 1.246 |
| sp\|P41231\|P2RY2_HUMAN P2Y purinoceptor 2 OS=Homo sapiens GN=P2RY2 PE=2 SV=4 | 365 | SRRTEStPAGS | 0.073 | -0.176 | -1.535 | -0.546 |
| sp\|P41231\|P2RY2_HUMAN P2Y purinoceptor 2 OS=Homo sapiens GN=P2RY2 PE=2 SV=4 | 369 | ESTPAGsENTK | 0.164 | -0.133 | -0.681 | -0.217 |
| sp\|P41231\|P2RY2_HUMAN P2Y purinoceptor 2 OS=Homo sapiens GN=P2RY2 PE=2 SV=4 | 372 | PAGSENtKDIR | 0.25 | -0.094 | -0.868 | -0.237 |
| sp\|P51582\|P2RY4_HUMAN P2Y purinoceptor 4 OS=Homo sapiens GN=P2RY4 PE=1 SV=1 | 330 | GKPQPRtAASS | 0.097 | -0.161 | -1.287 | -0.45 |
| sp\|P51582\|P2RY4_HUMAN P2Y purinoceptor 4 OS=Homo sapiens GN=P2RY4 PE=1 SV=1 | 333 | QPRTAAsSLAL | 0.417 | 0.287 | -0.386 | 0.106 |
| sp\|P51582\|P2RY4_HUMAN P2Y purinoceptor 4 OS=Homo sapiens GN=P2RY4 PE=1 SV=1 | 334 | PRTAASsLALV | 0.407 | 0.254 | -0.37 | 0.097 |
| sp\|P51582\|P2RY4_HUMAN P2Y purinoceptor 4 OS=Homo sapiens GN=P2RY4 PE=1 SV=1 | 339 | SSLALVsLPED | 0.761 | 0.414 | 0.297 | 0.491 |
| sp\|P51582\|P2RY4_HUMAN P2Y purinoceptor 4 OS=Homo sapiens GN=P2RY4 PE=1 SV=1 | 344 | VSLPEDsSCRW | 0.054 | -0.514 | -1.864 | -0.775 |
| sp\|P51582\|P2RY4_HUMAN P2Y purinoceptor 4 OS=Homo sapiens GN=P2RY4 PE=1 SV=1 | 345 | SLPEDSsCRWA | 0.064 | -0.18 | -1.35 | -0.489 |
| sp\|P51582\|P2RY4_HUMAN P2Y purinoceptor 4 OS=Homo sapiens GN=P2RY4 PE=1 SV=1 | 351 | SCRWAAtPQDS | 0.503 | 0.048 | -0.497 | 0.018 |
| sp\|P51582\|P2RY4_HUMAN P2Y purinoceptor 4 OS=Homo sapiens GN=P2RY4 PE=1 SV=1 | 355 | AATPQDsSCST | 0.03 | -0.501 | -1.999 | -0.823 |
| sp\|P51582\|P2RY4_HUMAN P2Y purinoceptor 4 OS=Homo sapiens GN=P2RY4 PE=1 SV=1 | 356 | ATPQDSsCSTP | 0.048 | -0.337 | -1.656 | -0.648 |
| sp\|P51582\|P2RY4_HUMAN P2Y purinoceptor 4 OS=Homo sapiens GN=P2RY4 PE=1 SV=1 | 358 | PQDSSCsTPRA | 0.714 | 0.498 | 0.127 | 0.446 |
| sp\|P51582\|P2RY4_HUMAN P2Y purinoceptor 4 OS=Homo sapiens GN=P2RY4 PE=1 SV=1 | 359 | QDSSCStPRAD | 0.066 | -0.534 | -1.275 | -0.581 |
| sp\|Q15077\|P2RY6_HUMAN P2Y purinoceptor 6 OS=Homo sapiens GN=P2RY6 PE=1 SV=1 | 320 | ELLQKLtAKWQ | 0.115 | -0.061 | -0.867 | -0.271 |
| sp\|Q96G91\|P2Y11_HUMAN P2Y purinoceptor 11 OS=Homo sapiens GN=P2RY11 PE=2 SV=2 | 341 | CPGYRDsWNPE | 0.238 | 0.06 | -0.598 | -0.1 |
| sp\|Q96G91\|P2Y11_HUMAN P2Y purinoceptor 11 OS=Homo sapiens GN=P2RY11 PE=2 SV=2 | 349 | NPEDAKsTGQA | 0.465 | 0.044 | -0.188 | 0.107 |
| sp\|Q96G91\|P2Y11_HUMAN P2Y purinoceptor 11 OS=Homo sapiens GN=P2RY11 PE=2 SV=2 | 350 | PEDAKStGQAL | 0.092 | -0.154 | -1.059 | -0.374 |
| sp\|Q96G91\|P2Y11_HUMAN P2Y purinoceptor 11 OS=Homo sapiens GN=P2RY11 PE=2 SV=2 | 359 | ALPLNAtAAPK | 0.203 | -0.125 | -1.023 | -0.315 |
| sp\|Q96G91\|P2Y11_HUMAN P2Y purinoceptor 11 OS=Homo sapiens GN=P2RY11 PE=2 SV=2 | 365 | TAAPKPsEPQS | 0.308 | 0.238 | -0.328 | 0.073 |
| sp\|Q96G91\|P2Y11_HUMAN P2Y purinoceptor 11 OS=Homo sapiens GN=P2RY11 PE=2 SV=2 | 369 | KPSEPQsRELS | 0.045 | -0.344 | -1.626 | -0.642 |
| sp\|Q96G91\|P2Y11_HUMAN P2Y purinoceptor 11 OS=Homo sapiens GN=P2RY11 PE=2 SV=2 | 373 | PQSRELsQ--- | 0.41 | 0.827 | -0.23 | 0.336 |
| sp\|Q9H244\|P2Y12_HUMAN P2Y purinoceptor 12 OS=Homo sapiens GN=P2RY12 PE=1 SV=1 | 340 | GDPNEEtPM-- | 0.038 | -0.405 | -1.907 | -0.758 |
| sp\|Q9BPV8\|P2Y13_HUMAN P2Y purinoceptor 13 OS=Homo sapiens GN=P2RY13 PE=2 SV=3 | 336 | CMQGRKtTASS | 0.188 | 0.226 | -0.533 | -0.04 |
| sp\|Q9BPV8\|P2Y13_HUMAN P2Y purinoceptor 13 OS=Homo sapiens GN=P2RY13 PE=2 SV=3 | 337 | MQGRKTtASSQ | 0.208 | 0.715 | -0.397 | 0.175 |
| sp\|Q9BPV8\|P2Y13_HUMAN P2Y purinoceptor 13 OS=Homo sapiens GN=P2RY13 PE=2 SV=3 | 339 | GRKTTAsSQEN | 0.402 | 0.327 | -0.453 | 0.092 |
| sp\|Q9BPV8\|P2Y13_HUMAN P2Y purinoceptor 13 OS=Homo sapiens GN=P2RY13 PE=2 SV=3 | 340 | RKTTASsQENH | 0.218 | -0.067 | -1.202 | -0.35 |
| sp\|Q9BPV8\|P2Y13_HUMAN P2Y purinoceptor 13 OS=Homo sapiens GN=P2RY13 PE=2 SV=3 | 345 | SSQENHsSQTD | 0.175 | -0.023 | -0.888 | -0.245 |
| sp\|Q9BPV8\|P2Y13_HUMAN P2Y purinoceptor 13 OS=Homo sapiens GN=P2RY13 PE=2 SV=3 | 346 | SQENHSsQTDN | 0.13 | -0.142 | -1.127 | -0.38 |
| sp\|Q9BPV8\|P2Y13_HUMAN P2Y purinoceptor 13 OS=Homo sapiens GN=P2RY13 PE=2 SV=3 | 348 | ENHSSQtDNIT | 0.194 | 0.009 | -0.585 | -0.127 |
| sp\|Q9BPV8\|P2Y13_HUMAN P2Y purinoceptor 13 OS=Homo sapiens GN=P2RY13 PE=2 SV=3 | 352 | SQTDNItLG-- | 0.372 | 0.177 | -0.267 | 0.094 |
| sp\|Q15391\|P2Y14_HUMAN P2Y purinoceptor 14 OS=Homo sapiens GN=P2RY14 PE=2 SV=1 | 330 | RIKRGNtTLES | 0.657 | 0.93 | 0.169 | 0.585 |
| sp\|Q15391\|P2Y14_HUMAN P2Y purinoceptor 14 OS=Homo sapiens GN=P2RY14 PE=2 SV=1 | 331 | IKRGNTtLEST | 0.55 | 0.274 | 0.263 | 0.362 |
| sp\|Q15391\|P2Y14_HUMAN P2Y purinoceptor 14 OS=Homo sapiens GN=P2RY14 PE=2 SV=1 | 334 | GNTTLEsTDTL | 0.15 | -0.154 | -1.079 | -0.361 |
| sp\|Q15391\|P2Y14_HUMAN P2Y purinoceptor 14 OS=Homo sapiens GN=P2RY14 PE=2 SV=1 | 335 | NTTLEStDTL- | 0.187 | -0.093 | -1.246 | -0.384 |
| sp\|Q15391\|P2Y14_HUMAN P2Y purinoceptor 14 OS=Homo sapiens GN=P2RY14 PE=2 SV=1 | 337 | TLESTDtL--- | 0.297 | 0.152 | -0.557 | -0.036 |
| sp\|Q99527\|GPER1_HUMAN G-protein coupled estrogen receptor 1 OS=Homo sapiens GN=GPER1 PE=1 SV=1 | 330 | YSFLGEtFRDK | 0.195 | -0.163 | -0.848 | -0.272 |
| sp\|Q99527\|GPER1_HUMAN G-protein coupled estrogen receptor 1 OS=Homo sapiens GN=GPER1 PE=1 SV=1 | 343 | LYIEQKtNLPA | 0.086 | -0.101 | -1.227 | -0.414 |
| sp\|Q99527\|GPER1_HUMAN G-protein coupled estrogen receptor 1 OS=Homo sapiens GN=GPER1 PE=1 SV=1 | 363 | KAVIPDsTEQS | 0.059 | -0.354 | -1.568 | -0.621 |
| sp\|Q99527\|GPER1_HUMAN G-protein coupled estrogen receptor 1 OS=Homo sapiens GN=GPER1 PE=1 SV=1 | 364 | AVIPDStEQSD | 0.07 | -0.14 | -1.184 | -0.418 |

| | | | | | | |
|---|---|---|---|---|---|---|
| sp\|Q99527\|GPER1_HUMAN G-protein coupled estrogen receptor 1 OS=Homo sapiens GN=GPER1 PE=1 SV=1 | 367 | PDSTEQsDVRF | 0.096 | -0.251 | -1.448 | -0.534 |
| sp\|Q99527\|GPER1_HUMAN G-protein coupled estrogen receptor 1 OS=Homo sapiens GN=GPER1 PE=1 SV=1 | 372 | QSDVRFsSAV- | 0.115 | -0.021 | -1.098 | -0.335 |
| sp\|Q99527\|GPER1_HUMAN G-protein coupled estrogen receptor 1 OS=Homo sapiens GN=GPER1 PE=1 SV=1 | 373 | SDVRFSsAV-- | 0.216 | 0.764 | -0.712 | 0.089 |
| sp\|Q9BXC0\|HCAR1_HUMAN Hydroxycarboxylic acid receptor 1 OS=Homo sapiens GN=HCAR1 PE=1 SV=1 | 282 | LVYYFSsPSFP | 0.014 | -0.597 | -2.019 | -0.867 |
| sp\|Q9BXC0\|HCAR1_HUMAN Hydroxycarboxylic acid receptor 1 OS=Homo sapiens GN=HCAR1 PE=1 SV=1 | 284 | YYFSSPsFPKF | 0.69 | 0.659 | 0.478 | 0.609 |
| sp\|Q9BXC0\|HCAR1_HUMAN Hydroxycarboxylic acid receptor 1 OS=Homo sapiens GN=HCAR1 PE=1 SV=1 | 296 | NKLKICsLKPK | 0.664 | 0.093 | 0.175 | 0.311 |
| sp\|Q9BXC0\|HCAR1_HUMAN Hydroxycarboxylic acid receptor 1 OS=Homo sapiens GN=HCAR1 PE=1 SV=1 | 305 | PKQPGHsKTQR | 0.454 | 0.096 | -0.227 | 0.108 |
| sp\|Q9BXC0\|HCAR1_HUMAN Hydroxycarboxylic acid receptor 1 OS=Homo sapiens GN=HCAR1 PE=1 SV=1 | 307 | QPGHSKtQRPE | 0.201 | -0.068 | -0.69 | -0.186 |
| sp\|Q9BXC0\|HCAR1_HUMAN Hydroxycarboxylic acid receptor 1 OS=Homo sapiens GN=HCAR1 PE=1 SV=1 | 316 | PEEMPIsNLGR | 0.136 | -0.291 | -1.053 | -0.403 |
| sp\|Q9BXC0\|HCAR1_HUMAN Hydroxycarboxylic acid receptor 1 OS=Homo sapiens GN=HCAR1 PE=1 SV=1 | 322 | SNLGRRsCISV | 0.068 | -0.02 | -1.373 | -0.442 |
| sp\|Q9BXC0\|HCAR1_HUMAN Hydroxycarboxylic acid receptor 1 OS=Homo sapiens GN=HCAR1 PE=1 SV=1 | 325 | GRRSCIsVANS | 0.562 | 0.422 | -0.324 | 0.22 |
| sp\|Q9BXC0\|HCAR1_HUMAN Hydroxycarboxylic acid receptor 1 OS=Homo sapiens GN=HCAR1 PE=1 SV=1 | 329 | CISVANsFQSQ | 0.327 | 0.141 | -0.478 | -0.003 |
| sp\|Q9BXC0\|HCAR1_HUMAN Hydroxycarboxylic acid receptor 1 OS=Homo sapiens GN=HCAR1 PE=1 SV=1 | 332 | VANSFQsQSDG | 0.105 | -0.257 | -1.498 | -0.55 |
| sp\|Q9BXC0\|HCAR1_HUMAN Hydroxycarboxylic acid receptor 1 OS=Homo sapiens GN=HCAR1 PE=1 SV=1 | 334 | NSFQSQsDGQW | 0.52 | 0.24 | 0.039 | 0.266 |
| sp\|Q8TDS4\|HCAR2_HUMAN Hydroxycarboxylic acid receptor 2 OS=Homo sapiens GN=HCAR2 PE=1 SV=1 | 298 | VVYYFSsPSFP | 0.016 | -0.626 | -2.096 | -0.902 |
| sp\|Q8TDS4\|HCAR2_HUMAN Hydroxycarboxylic acid receptor 2 OS=Homo sapiens GN=HCAR2 PE=1 SV=1 | 300 | YYFSSPsFPNF | 0.751 | 0.728 | 0.502 | 0.66 |
| sp\|Q8TDS4\|HCAR2_HUMAN Hydroxycarboxylic acid receptor 2 OS=Homo sapiens GN=HCAR2 PE=1 SV=1 | 306 | SFPNFFsTLIN | 0.08 | -0.147 | -1.299 | -0.455 |
| sp\|Q8TDS4\|HCAR2_HUMAN Hydroxycarboxylic acid receptor 2 OS=Homo sapiens GN=HCAR2 PE=1 SV=1 | 307 | FPNFFStLINR | 0.229 | 0.071 | -0.819 | -0.173 |
| sp\|Q8TDS4\|HCAR2_HUMAN Hydroxycarboxylic acid receptor 2 OS=Homo sapiens GN=HCAR2 PE=1 SV=1 | 318 | CLQRKMtGEPD | 0.745 | 1.024 | 0.78 | 0.85 |
| sp\|Q8TDS4\|HCAR2_HUMAN Hydroxycarboxylic acid receptor 2 OS=Homo sapiens GN=HCAR2 PE=1 SV=1 | 326 | EPDNNRsTSVE | 0.046 | -0.243 | -1.502 | -0.566 |
| sp\|Q8TDS4\|HCAR2_HUMAN Hydroxycarboxylic acid receptor 2 OS=Homo sapiens GN=HCAR2 PE=1 SV=1 | 327 | PDNNRStSVEL | 0.11 | -0.026 | -0.997 | -0.304 |
| sp\|Q8TDS4\|HCAR2_HUMAN Hydroxycarboxylic acid receptor 2 OS=Homo sapiens GN=HCAR2 PE=1 SV=1 | 328 | DNNRSTsVELT | 0.666 | 0.923 | 0.445 | 0.678 |
| sp\|Q8TDS4\|HCAR2_HUMAN Hydroxycarboxylic acid receptor 2 OS=Homo sapiens GN=HCAR2 PE=1 SV=1 | 332 | STSVELtGDPN | 0.047 | -0.316 | -1.69 | -0.653 |
| sp\|Q8TDS4\|HCAR2_HUMAN Hydroxycarboxylic acid receptor 2 OS=Homo sapiens GN=HCAR2 PE=1 SV=1 | 338 | TGDPNKtRGAP | 0.199 | -0.141 | -0.527 | -0.156 |
| sp\|Q8TDS4\|HCAR2_HUMAN Hydroxycarboxylic acid receptor 2 OS=Homo sapiens GN=HCAR2 PE=1 SV=1 | 349 | EALMANsGEPW | 0.156 | -0.125 | -0.893 | -0.287 |
| sp\|Q8TDS4\|HCAR2_HUMAN Hydroxycarboxylic acid receptor 2 OS=Homo sapiens GN=HCAR2 PE=1 SV=1 | 354 | NSGEPWsPSYL | 0.027 | -0.615 | -1.913 | -0.834 |
| sp\|Q8TDS4\|HCAR2_HUMAN Hydroxycarboxylic acid receptor 2 OS=Homo sapiens GN=HCAR2 PE=1 SV=1 | 356 | GEPWSPsYLGP | 0.142 | -0.133 | -0.97 | -0.32 |
| sp\|Q8TDS4\|HCAR2_HUMAN Hydroxycarboxylic acid receptor 2 OS=Homo sapiens GN=HCAR2 PE=1 SV=1 | 361 | PSYLGPtSP-- | 0.268 | 0.377 | -0.54 | 0.035 |
| sp\|Q8TDS4\|HCAR2_HUMAN Hydroxycarboxylic acid receptor 2 OS=Homo sapiens GN=HCAR2 PE=1 SV=1 | 362 | SYLGPTsP--- | 0.063 | -0.28 | -1.294 | -0.504 |
| sp\|P49019\|HCAR3_HUMAN Hydroxycarboxylic acid receptor 3 OS=Homo sapiens GN=HCAR3 PE=1 SV=3 | 298 | VVYYFSsPSFP | 0.016 | -0.626 | -2.096 | -0.902 |
| sp\|P49019\|HCAR3_HUMAN Hydroxycarboxylic acid receptor 3 OS=Homo sapiens GN=HCAR3 PE=1 SV=3 | 300 | YYFSSPsFPNF | 0.751 | 0.728 | 0.502 | 0.66 |
| sp\|P49019\|HCAR3_HUMAN Hydroxycarboxylic acid receptor 3 OS=Homo sapiens GN=HCAR3 PE=1 SV=3 | 306 | SFPNFFsTLIN | 0.08 | -0.147 | -1.299 | -0.455 |
| sp\|P49019\|HCAR3_HUMAN Hydroxycarboxylic acid receptor 3 OS=Homo sapiens GN=HCAR3 PE=1 SV=3 | 307 | FPNFFStLINR | 0.229 | 0.071 | -0.819 | -0.173 |
| sp\|P49019\|HCAR3_HUMAN Hydroxycarboxylic acid receptor 3 OS=Homo sapiens GN=HCAR3 PE=1 SV=3 | 318 | CLQRKItGEPD | 0.703 | 0.886 | 0.615 | 0.735 |
| sp\|P49019\|HCAR3_HUMAN Hydroxycarboxylic acid receptor 3 OS=Homo sapiens GN=HCAR3 PE=1 SV=3 | 326 | EPDNNRsTSVE | 0.046 | -0.243 | -1.502 | -0.566 |
| sp\|P49019\|HCAR3_HUMAN Hydroxycarboxylic acid receptor 3 OS=Homo sapiens GN=HCAR3 PE=1 SV=3 | 327 | PDNNRStSVEL | 0.11 | -0.026 | -0.997 | -0.304 |
| sp\|P49019\|HCAR3_HUMAN Hydroxycarboxylic acid receptor 3 OS=Homo sapiens GN=HCAR3 PE=1 SV=3 | 328 | DNNRSTsVELT | 0.666 | 0.923 | 0.445 | 0.678 |
| sp\|P49019\|HCAR3_HUMAN Hydroxycarboxylic acid receptor 3 OS=Homo sapiens GN=HCAR3 PE=1 SV=3 | 332 | STSVELtGDPN | 0.047 | -0.316 | -1.69 | -0.653 |
| sp\|P49019\|HCAR3_HUMAN Hydroxycarboxylic acid receptor 3 OS=Homo sapiens GN=HCAR3 PE=1 SV=3 | 338 | TGDPNKtRGAP | 0.199 | -0.141 | -0.527 | -0.156 |
| sp\|P49019\|HCAR3_HUMAN Hydroxycarboxylic acid receptor 3 OS=Homo sapiens GN=HCAR3 PE=1 SV=3 | 349 | EALIANsGEPW | 0.132 | -0.067 | -0.952 | -0.296 |
| sp\|P49019\|HCAR3_HUMAN Hydroxycarboxylic acid receptor 3 OS=Homo sapiens GN=HCAR3 PE=1 SV=3 | 354 | NSGEPWsPSYL | 0.027 | -0.615 | -1.913 | -0.834 |
| sp\|P49019\|HCAR3_HUMAN Hydroxycarboxylic acid receptor 3 OS=Homo sapiens GN=HCAR3 PE=1 SV=3 | 356 | GEPWSPsYLGP | 0.142 | -0.133 | -0.97 | -0.32 |
| sp\|P49019\|HCAR3_HUMAN Hydroxycarboxylic acid receptor 3 OS=Homo sapiens GN=HCAR3 PE=1 SV=3 | 361 | PSYLGPtSNNH | 0.064 | -0.302 | -1.553 | -0.597 |
| sp\|P49019\|HCAR3_HUMAN Hydroxycarboxylic acid receptor 3 OS=Homo sapiens GN=HCAR3 PE=1 SV=3 | 362 | SYLGPTsNNHS | 0.076 | -0.238 | -1.096 | -0.419 |
| sp\|P49019\|HCAR3_HUMAN Hydroxycarboxylic acid receptor 3 OS=Homo sapiens GN=HCAR3 PE=1 SV=3 | 366 | PTSNNHsKKGH | 0.202 | -0.136 | -0.684 | -0.206 |
| sp\|P49019\|HCAR3_HUMAN Hydroxycarboxylic acid receptor 3 OS=Homo sapiens GN=HCAR3 PE=1 SV=3 | 377 | CHQEPAsLEKQ | 0.189 | -0.016 | -0.955 | -0.261 |
| sp\|Q96P68\|OXGR1_HUMAN 2-oxoglutarate receptor 1 OS=Homo sapiens GN=OXGR1 PE=2 SV=1 | 306 | LLYVVVsDNFQ | 0.245 | -0.032 | -0.746 | -0.178 |
| sp\|Q96P68\|OXGR1_HUMAN 2-oxoglutarate receptor 1 OS=Homo sapiens GN=OXGR1 PE=2 SV=1 | 315 | FQQAVCsTVRC | 0.193 | -0.107 | -1.241 | -0.385 |
| sp\|Q96P68\|OXGR1_HUMAN 2-oxoglutarate receptor 1 OS=Homo sapiens GN=OXGR1 PE=2 SV=1 | 316 | QQAVCStVRCK | 0.099 | -0.187 | -0.897 | -0.328 |

| | | | | | | |
|---|---|---|---|---|---|---|
| sp\|Q96P68\|OXGR1_HUMAN 2-oxoglutarate receptor 1 OS=Homo sapiens GN=OXGR1 PE=2 SV=1 | 322 | TVRCKVsGNLE | 0.372 | 0.091 | -0.432 | 0.01 |
| sp\|Q96P68\|OXGR1_HUMAN 2-oxoglutarate receptor 1 OS=Homo sapiens GN=OXGR1 PE=2 SV=1 | 332 | EQAKKIsYSNN | 0.1 | -0.119 | -1.019 | -0.346 |
| sp\|Q96P68\|OXGR1_HUMAN 2-oxoglutarate receptor 1 OS=Homo sapiens GN=OXGR1 PE=2 SV=1 | 334 | AKKISYsNNP- | 0.55 | 0.317 | 0.065 | 0.311 |
| sp\|P08100\|OPSD_HUMAN Rhodopsin OS=Homo sapiens GN=RHO PE=1 SV=1 | 319 | FRNCMLtTICC | 0.125 | 0.097 | -1.148 | -0.309 |
| sp\|P08100\|OPSD_HUMAN Rhodopsin OS=Homo sapiens GN=RHO PE=1 SV=1 | 320 | RNCMLTtICCG | 0.103 | -0.249 | -1.233 | -0.46 |
| sp\|P08100\|OPSD_HUMAN Rhodopsin OS=Homo sapiens GN=RHO PE=1 SV=1 | 334 | LGDDEAsATVS | 0.186 | -0.067 | -1.219 | -0.367 |
| sp\|P08100\|OPSD_HUMAN Rhodopsin OS=Homo sapiens GN=RHO PE=1 SV=1 | 336 | DDEASAtVSKT | 0.192 | -0.061 | -0.824 | -0.231 |
| sp\|P08100\|OPSD_HUMAN Rhodopsin OS=Homo sapiens GN=RHO PE=1 SV=1 | 338 | EASATVsKTET | 0.187 | -0.176 | -0.805 | -0.265 |
| sp\|P08100\|OPSD_HUMAN Rhodopsin OS=Homo sapiens GN=RHO PE=1 SV=1 | 340 | SATVSKtETSQ | 0.192 | 0.087 | -0.675 | -0.132 |
| sp\|P08100\|OPSD_HUMAN Rhodopsin OS=Homo sapiens GN=RHO PE=1 SV=1 | 342 | TVSKTEtSQVA | 0.132 | -0.077 | -1.004 | -0.316 |
| sp\|P08100\|OPSD_HUMAN Rhodopsin OS=Homo sapiens GN=RHO PE=1 SV=1 | 343 | VSKTETsQVAP | 0.124 | -0.209 | -1.193 | -0.426 |
| sp\|Q9UHM6\|OPN4_HUMAN Melanopsin OS=Homo sapiens GN=OPN4 PE=1 SV=1 | 375 | GVLLGVsRRHS | 0.114 | -0.284 | -1.137 | -0.436 |
| sp\|Q9UHM6\|OPN4_HUMAN Melanopsin OS=Homo sapiens GN=OPN4 PE=1 SV=1 | 379 | GVSRRHsRPYP | 0.91 | 1.458 | 1.043 | 1.137 |
| sp\|Q9UHM6\|OPN4_HUMAN Melanopsin OS=Homo sapiens GN=OPN4 PE=1 SV=1 | 384 | HSRPYPsYRST | 0.128 | -0.098 | -0.806 | -0.259 |
| sp\|Q9UHM6\|OPN4_HUMAN Melanopsin OS=Homo sapiens GN=OPN4 PE=1 SV=1 | 387 | PYPSYRsTHRS | 0.133 | -0.187 | -1.438 | -0.497 |
| sp\|Q9UHM6\|OPN4_HUMAN Melanopsin OS=Homo sapiens GN=OPN4 PE=1 SV=1 | 388 | YPSYRStHRST | 0.121 | -0.129 | -0.878 | -0.295 |
| sp\|Q9UHM6\|OPN4_HUMAN Melanopsin OS=Homo sapiens GN=OPN4 PE=1 SV=1 | 391 | YRSTHRsTLTS | 0.114 | 0.078 | -1.224 | -0.344 |
| sp\|Q9UHM6\|OPN4_HUMAN Melanopsin OS=Homo sapiens GN=OPN4 PE=1 SV=1 | 392 | RSTHRStLTSH | 0.221 | -0.072 | -0.779 | -0.21 |
| sp\|Q9UHM6\|OPN4_HUMAN Melanopsin OS=Homo sapiens GN=OPN4 PE=1 SV=1 | 394 | THRSTLtSHTS | 0.095 | 0.021 | -1.368 | -0.417 |
| sp\|Q9UHM6\|OPN4_HUMAN Melanopsin OS=Homo sapiens GN=OPN4 PE=1 SV=1 | 395 | HRSTLTsHTSN | 0.158 | 0.117 | -0.931 | -0.219 |
| sp\|Q9UHM6\|OPN4_HUMAN Melanopsin OS=Homo sapiens GN=OPN4 PE=1 SV=1 | 397 | STLTSHtSNLS | 0.33 | 0.189 | -0.562 | -0.014 |
| sp\|Q9UHM6\|OPN4_HUMAN Melanopsin OS=Homo sapiens GN=OPN4 PE=1 SV=1 | 398 | TLTSHTsNLSW | 0.102 | -0.072 | -1.166 | -0.379 |
| sp\|Q9UHM6\|OPN4_HUMAN Melanopsin OS=Homo sapiens GN=OPN4 PE=1 SV=1 | 401 | SHTSNLsWISI | 0.054 | -0.115 | -1.337 | -0.466 |
| sp\|Q9UHM6\|OPN4_HUMAN Melanopsin OS=Homo sapiens GN=OPN4 PE=1 SV=1 | 404 | SNLSWIsIRRR | 0.108 | -0.373 | -1.148 | -0.471 |
| sp\|Q9UHM6\|OPN4_HUMAN Melanopsin OS=Homo sapiens GN=OPN4 PE=1 SV=1 | 411 | IRRRQEsLGSE | 0.858 | 1.313 | 0.906 | 1.026 |
| sp\|Q9UHM6\|OPN4_HUMAN Melanopsin OS=Homo sapiens GN=OPN4 PE=1 SV=1 | 414 | RQESLGsESEV | 0.088 | -0.182 | -1.457 | -0.517 |
| sp\|Q9UHM6\|OPN4_HUMAN Melanopsin OS=Homo sapiens GN=OPN4 PE=1 SV=1 | 416 | ESLGSEsEVGW | 0.193 | 0.1 | -0.517 | -0.075 |
| sp\|Q9UHM6\|OPN4_HUMAN Melanopsin OS=Homo sapiens GN=OPN4 PE=1 SV=1 | 421 | ESEVGWtHMEA | 0.112 | -0.251 | -1.071 | -0.403 |
| sp\|Q9UHM6\|OPN4_HUMAN Melanopsin OS=Homo sapiens GN=OPN4 PE=1 SV=1 | 439 | QQANGRsLYGQ | 0.082 | -0.152 | -0.784 | -0.285 |
| sp\|Q9UHM6\|OPN4_HUMAN Melanopsin OS=Homo sapiens GN=OPN4 PE=1 SV=1 | 463 | QGHEAEtPGKT | 0.052 | -0.434 | -1.6 | -0.661 |
| sp\|Q9UHM6\|OPN4_HUMAN Melanopsin OS=Homo sapiens GN=OPN4 PE=1 SV=1 | 467 | AETPGKtKGLI | 0.275 | -0.031 | -0.378 | -0.045 |
| sp\|Q9UHM6\|OPN4_HUMAN Melanopsin OS=Homo sapiens GN=OPN4 PE=1 SV=1 | 473 | TKGLIPsQDPR | 0.173 | -0.137 | -0.848 | -0.271 |
| sp\|Q9H1Y3\|OPN3_HUMAN Opsin-3 OS=Homo sapiens GN=OPN3 PE=1 SV=1 | 319 | IRKFRRsLLQL | 0.642 | 0.473 | 0.175 | 0.43 |
| sp\|Q9H1Y3\|OPN3_HUMAN Opsin-3 OS=Homo sapiens GN=OPN3 PE=1 SV=1 | 343 | DLPAAGsEMQI | 0.452 | 0.069 | -0.43 | 0.03 |
| sp\|Q9H1Y3\|OPN3_HUMAN Opsin-3 OS=Homo sapiens GN=OPN3 PE=1 SV=1 | 353 | IRPIVMsQKDG | 0.351 | 0.257 | -0.569 | 0.013 |
| sp\|Q9H1Y3\|OPN3_HUMAN Opsin-3 OS=Homo sapiens GN=OPN3 PE=1 SV=1 | 365 | RPKKKVtFNSS | 0.408 | 0.048 | -0.318 | 0.046 |
| sp\|Q9H1Y3\|OPN3_HUMAN Opsin-3 OS=Homo sapiens GN=OPN3 PE=1 SV=1 | 368 | KKVTFNsSSII | 0.321 | -0.034 | -0.861 | -0.191 |
| sp\|Q9H1Y3\|OPN3_HUMAN Opsin-3 OS=Homo sapiens GN=OPN3 PE=1 SV=1 | 369 | KVTFNSsSIIF | 0.083 | -0.171 | -1.019 | -0.369 |
| sp\|Q9H1Y3\|OPN3_HUMAN Opsin-3 OS=Homo sapiens GN=OPN3 PE=1 SV=1 | 370 | VTFNSSsIIFI | 0.144 | 0.002 | -0.533 | -0.129 |
| sp\|Q9H1Y3\|OPN3_HUMAN Opsin-3 OS=Homo sapiens GN=OPN3 PE=1 SV=1 | 376 | SIIFIItSDES | 0.201 | 0.025 | -0.819 | -0.198 |
| sp\|Q9H1Y3\|OPN3_HUMAN Opsin-3 OS=Homo sapiens GN=OPN3 PE=1 SV=1 | 377 | IIFIITsDESL | 0.183 | 0.089 | -0.658 | -0.129 |
| sp\|Q9H1Y3\|OPN3_HUMAN Opsin-3 OS=Homo sapiens GN=OPN3 PE=1 SV=1 | 380 | IITSDEsLSVD | 0.283 | -0.014 | -0.55 | -0.094 |
| sp\|Q9H1Y3\|OPN3_HUMAN Opsin-3 OS=Homo sapiens GN=OPN3 PE=1 SV=1 | 382 | TSDESLsVDDS | 0.189 | 0.081 | -0.67 | -0.133 |
| sp\|Q9H1Y3\|OPN3_HUMAN Opsin-3 OS=Homo sapiens GN=OPN3 PE=1 SV=1 | 386 | SLSVDDsDKTN | 0.115 | -0.172 | -1.379 | -0.479 |
| sp\|Q9H1Y3\|OPN3_HUMAN Opsin-3 OS=Homo sapiens GN=OPN3 PE=1 SV=1 | 389 | VDDSDKtNGSK | 0.069 | -0.325 | -1.312 | -0.523 |
| sp\|Q9H1Y3\|OPN3_HUMAN Opsin-3 OS=Homo sapiens GN=OPN3 PE=1 SV=1 | 392 | SDKTNGsKVDV | 0.12 | -0.202 | -1.255 | -0.446 |
| sp\|Q6U736\|OPN5_HUMAN Opsin-5 OS=Homo sapiens GN=OPN5 PE=1 SV=3 | 318 | KFACCQtGGLK | 0.385 | -0.043 | -0.322 | 0.007 |
| sp\|Q6U736\|OPN5_HUMAN Opsin-5 OS=Homo sapiens GN=OPN5 PE=1 SV=3 | 324 | TGGLKAtKKKS | 0.072 | -0.213 | -1.318 | -0.486 |
| sp\|Q6U736\|OPN5_HUMAN Opsin-5 OS=Homo sapiens GN=OPN5 PE=1 SV=3 | 328 | KATKKKsLEGF | 0.347 | -0.021 | -0.2 | 0.042 |

| | | | | | | |
|---|---|---|---|---|---|---|
| sp\|Q6U736\|OPN5_HUMAN Opsin-5 OS=Homo sapiens GN=OPN5 PE=1 SV=3 | 336 | EGFRLHtVTTV | 0.642 | 0.888 | 0.097 | 0.542 |
| sp\|Q6U736\|OPN5_HUMAN Opsin-5 OS=Homo sapiens GN=OPN5 PE=1 SV=3 | 338 | FRLHTVtTVRK | 0.27 | 0.142 | -0.656 | -0.081 |
| sp\|Q6U736\|OPN5_HUMAN Opsin-5 OS=Homo sapiens GN=OPN5 PE=1 SV=3 | 339 | RLHTVTtVRKS | 0.195 | -0.061 | -0.954 | -0.273 |
| sp\|Q6U736\|OPN5_HUMAN Opsin-5 OS=Homo sapiens GN=OPN5 PE=1 SV=3 | 343 | VTTVRKsSAVL | 0.248 | 0.076 | -0.594 | -0.09 |
| sp\|Q6U736\|OPN5_HUMAN Opsin-5 OS=Homo sapiens GN=OPN5 PE=1 SV=3 | 344 | TTVRKSsAVLE | 0.301 | 0.681 | -0.47 | 0.171 |
| sp\|P03999\|OPSB_HUMAN Short-wave-sensitive opsin 1 OS=Homo sapiens GN=OPN1SW PE=1 SV=1 | 324 | VCGKAMtDESD | 0.467 | 0.227 | -0.455 | 0.08 |
| sp\|P03999\|OPSB_HUMAN Short-wave-sensitive opsin 1 OS=Homo sapiens GN=OPN1SW PE=1 SV=1 | 327 | KAMTDEsDTCS | 0.125 | -0.148 | -1.5 | -0.508 |
| sp\|P03999\|OPSB_HUMAN Short-wave-sensitive opsin 1 OS=Homo sapiens GN=OPN1SW PE=1 SV=1 | 329 | MTDESDtCSSQ | 0.119 | 0.026 | -1.279 | -0.378 |
| sp\|P03999\|OPSB_HUMAN Short-wave-sensitive opsin 1 OS=Homo sapiens GN=OPN1SW PE=1 SV=1 | 331 | DESDTCsSQKT | 0.12 | -0.154 | -1.482 | -0.505 |
| sp\|P03999\|OPSB_HUMAN Short-wave-sensitive opsin 1 OS=Homo sapiens GN=OPN1SW PE=1 SV=1 | 332 | ESDTCSsQKTE | 0.076 | -0.352 | -1.497 | -0.591 |
| sp\|P03999\|OPSB_HUMAN Short-wave-sensitive opsin 1 OS=Homo sapiens GN=OPN1SW PE=1 SV=1 | 335 | TCSSQKtEVST | 0.093 | -0.081 | -1.118 | -0.369 |
| sp\|P03999\|OPSB_HUMAN Short-wave-sensitive opsin 1 OS=Homo sapiens GN=OPN1SW PE=1 SV=1 | 338 | SQKTEVsTVSS | 0.084 | -0.225 | -1.348 | -0.496 |
| sp\|P03999\|OPSB_HUMAN Short-wave-sensitive opsin 1 OS=Homo sapiens GN=OPN1SW PE=1 SV=1 | 339 | QKTEVStVSST | 0.063 | -0.215 | -1.332 | -0.495 |
| sp\|P03999\|OPSB_HUMAN Short-wave-sensitive opsin 1 OS=Homo sapiens GN=OPN1SW PE=1 SV=1 | 341 | TEVSTVsSTQV | 0.149 | -0.193 | -1.344 | -0.463 |
| sp\|P03999\|OPSB_HUMAN Short-wave-sensitive opsin 1 OS=Homo sapiens GN=OPN1SW PE=1 SV=1 | 342 | EVSTVSsTQVG | 0.087 | -0.147 | -1.21 | -0.423 |
| sp\|P03999\|OPSB_HUMAN Short-wave-sensitive opsin 1 OS=Homo sapiens GN=OPN1SW PE=1 SV=1 | 343 | VSTVSStQVGP | 0.083 | -0.169 | -1.168 | -0.418 |
| sp\|P04001\|OPSG_HUMAN Medium-wave-sensitive opsin 1 OS=Homo sapiens GN=OPN1MW PE=1 SV=1 | 313 | PAFFAKsATIY | 0.663 | 0.183 | -0.248 | 0.199 |
| sp\|P04001\|OPSG_HUMAN Medium-wave-sensitive opsin 1 OS=Homo sapiens GN=OPN1MW PE=1 SV=1 | 315 | FFAKSAtIYNP | 0.523 | 0.328 | 0.021 | 0.291 |
| sp\|P04001\|OPSG_HUMAN Medium-wave-sensitive opsin 1 OS=Homo sapiens GN=OPN1MW PE=1 SV=1 | 345 | KKVDDGsELSS | 0.072 | -0.133 | -1.479 | -0.513 |
| sp\|P04001\|OPSG_HUMAN Medium-wave-sensitive opsin 1 OS=Homo sapiens GN=OPN1MW PE=1 SV=1 | 348 | DDGSELsSASK | 0.019 | -0.508 | -2.176 | -0.888 |
| sp\|P04001\|OPSG_HUMAN Medium-wave-sensitive opsin 1 OS=Homo sapiens GN=OPN1MW PE=1 SV=1 | 349 | DGSELSsASKT | 0.038 | -0.345 | -1.923 | -0.743 |
| sp\|P04001\|OPSG_HUMAN Medium-wave-sensitive opsin 1 OS=Homo sapiens GN=OPN1MW PE=1 SV=1 | 351 | SELSSAsKTEV | 0.37 | 0.075 | -0.786 | -0.114 |
| sp\|P04001\|OPSG_HUMAN Medium-wave-sensitive opsin 1 OS=Homo sapiens GN=OPN1MW PE=1 SV=1 | 353 | LSSASKtEVSS | 0.231 | 0.117 | -0.359 | -0.004 |
| sp\|P04001\|OPSG_HUMAN Medium-wave-sensitive opsin 1 OS=Homo sapiens GN=OPN1MW PE=1 SV=1 | 356 | ASKTEVsSVSS | 0.125 | -0.268 | -1.353 | -0.499 |
| sp\|P04001\|OPSG_HUMAN Medium-wave-sensitive opsin 1 OS=Homo sapiens GN=OPN1MW PE=1 SV=1 | 357 | SKTEVSsVSSV | 0.061 | -0.204 | -1.687 | -0.61 |
| sp\|P04001\|OPSG_HUMAN Medium-wave-sensitive opsin 1 OS=Homo sapiens GN=OPN1MW PE=1 SV=1 | 359 | TEVSSVsSVSP | 0.117 | -0.078 | -1.112 | -0.358 |
| sp\|P04001\|OPSG_HUMAN Medium-wave-sensitive opsin 1 OS=Homo sapiens GN=OPN1MW PE=1 SV=1 | 360 | EVSSVSsVSPA | 0.041 | -0.343 | -1.478 | -0.593 |
| sp\|P04001\|OPSG_HUMAN Medium-wave-sensitive opsin 1 OS=Homo sapiens GN=OPN1MW PE=1 SV=1 | 362 | SSVSSVsPA-- | 0.115 | -0.209 | -1.202 | -0.432 |
| sp\|P04000\|OPSR_HUMAN Long-wave-sensitive opsin 1 OS=Homo sapiens GN=OPN1LW PE=1 SV=1 | 345 | KKVDDGsELSS | 0.072 | -0.133 | -1.479 | -0.513 |
| sp\|P04000\|OPSR_HUMAN Long-wave-sensitive opsin 1 OS=Homo sapiens GN=OPN1LW PE=1 SV=1 | 348 | DDGSELsSASK | 0.019 | -0.508 | -2.176 | -0.888 |
| sp\|P04000\|OPSR_HUMAN Long-wave-sensitive opsin 1 OS=Homo sapiens GN=OPN1LW PE=1 SV=1 | 349 | DGSELSsASKT | 0.038 | -0.345 | -1.923 | -0.743 |
| sp\|P04000\|OPSR_HUMAN Long-wave-sensitive opsin 1 OS=Homo sapiens GN=OPN1LW PE=1 SV=1 | 351 | SELSSAsKTEV | 0.37 | 0.075 | -0.786 | -0.114 |
| sp\|P04000\|OPSR_HUMAN Long-wave-sensitive opsin 1 OS=Homo sapiens GN=OPN1LW PE=1 SV=1 | 353 | LSSASKtEVSS | 0.231 | 0.117 | -0.359 | -0.004 |
| sp\|P04000\|OPSR_HUMAN Long-wave-sensitive opsin 1 OS=Homo sapiens GN=OPN1LW PE=1 SV=1 | 356 | ASKTEVsSVSS | 0.125 | -0.268 | -1.353 | -0.499 |
| sp\|P04000\|OPSR_HUMAN Long-wave-sensitive opsin 1 OS=Homo sapiens GN=OPN1LW PE=1 SV=1 | 357 | SKTEVSsVSSV | 0.061 | -0.204 | -1.687 | -0.61 |
| sp\|P04000\|OPSR_HUMAN Long-wave-sensitive opsin 1 OS=Homo sapiens GN=OPN1LW PE=1 SV=1 | 359 | TEVSSVsSVSP | 0.117 | -0.078 | -1.112 | -0.358 |
| sp\|P04000\|OPSR_HUMAN Long-wave-sensitive opsin 1 OS=Homo sapiens GN=OPN1LW PE=1 SV=1 | 360 | EVSSVSsVSPA | 0.041 | -0.343 | -1.478 | -0.593 |
| sp\|P04000\|OPSR_HUMAN Long-wave-sensitive opsin 1 OS=Homo sapiens GN=OPN1LW PE=1 SV=1 | 362 | SSVSSVsPA-- | 0.115 | -0.209 | -1.202 | -0.432 |
| sp\|P59534\|T2R39_HUMAN Taste receptor type 2 member 39 OS=Homo sapiens GN=TAS2R39 PE=2 SV=3 | 337 | LYPKEWtL--- | 0.466 | 0.171 | -0.386 | 0.084 |
| sp\|P59533\|T2R38_HUMAN Taste receptor type 2 member 38 OS=Homo sapiens GN=TAS2R38 PE=2 SV=3 | 329 | ADHKADsRTLC | 0.164 | -0.316 | -1.525 | -0.559 |
| sp\|P59533\|T2R38_HUMAN Taste receptor type 2 member 38 OS=Homo sapiens GN=TAS2R38 PE=2 SV=3 | 331 | HKADSRtLC-- | 0.553 | 0.45 | 0.054 | 0.352 |
| sp\|P46091\|GPR1_HUMAN G-protein coupled receptor 1 OS=Homo sapiens GN=GPR1 PE=1 SV=2 | 308 | ILYVLIsKKFQ | 0.099 | -0.178 | -1.039 | -0.373 |
| sp\|P46091\|GPR1_HUMAN G-protein coupled receptor 1 OS=Homo sapiens GN=GPR1 PE=1 SV=2 | 317 | FQARFRsSVAE | 0.479 | 0.862 | 0.109 | 0.483 |
| sp\|P46091\|GPR1_HUMAN G-protein coupled receptor 1 OS=Homo sapiens GN=GPR1 PE=1 SV=2 | 318 | QARFRSsVAEI | 0.518 | 0.296 | -0.148 | 0.222 |
| sp\|P46091\|GPR1_HUMAN G-protein coupled receptor 1 OS=Homo sapiens GN=GPR1 PE=1 SV=2 | 326 | AEILKYtLWEV | 0.353 | 0.122 | -0.785 | -0.103 |
| sp\|P46091\|GPR1_HUMAN G-protein coupled receptor 1 OS=Homo sapiens GN=GPR1 PE=1 SV=2 | 331 | YTLWEVsCSGT | 0.234 | -0.188 | -0.802 | -0.252 |
| sp\|P46091\|GPR1_HUMAN G-protein coupled receptor 1 OS=Homo sapiens GN=GPR1 PE=1 SV=2 | 333 | LWEVSCsGTVS | 0.283 | 0.122 | -0.639 | -0.078 |
| sp\|P46091\|GPR1_HUMAN G-protein coupled receptor 1 OS=Homo sapiens GN=GPR1 PE=1 SV=2 | 335 | EVSCSGtVSEQ | 0.145 | -0.02 | -0.952 | -0.276 |
| sp\|P46091\|GPR1_HUMAN G-protein coupled receptor 1 OS=Homo sapiens GN=GPR1 PE=1 SV=2 | 337 | SCSGTVsEQLR | 0.243 | 0.074 | -0.689 | -0.124 |

| | | | | | | |
|---|---|---|---|---|---|---|
| sp\|P46091\|GPR1_HUMAN G-protein coupled receptor 1 OS=Homo sapiens GN=GPR1 PE=1 SV=2 | 343 | SEQLRNsETKN | 0.374 | 0.195 | -0.887 | -0.106 |
| sp\|P46091\|GPR1_HUMAN G-protein coupled receptor 1 OS=Homo sapiens GN=GPR1 PE=1 SV=2 | 345 | QLRNSEtKNLC | 0.58 | 0.423 | 0.134 | 0.379 |
| sp\|P46091\|GPR1_HUMAN G-protein coupled receptor 1 OS=Homo sapiens GN=GPR1 PE=1 SV=2 | 353 | NLCLLEtAQ-- | 0.254 | 0.16 | -1.022 | -0.203 |
| sp\|P46089\|GPR3_HUMAN G-protein coupled receptor 3 OS=Homo sapiens GN=GPR3 PE=1 SV=1 | 316 | AVCCCCsSSKI | 0.28 | -0.094 | -1.033 | -0.282 |
| sp\|P46089\|GPR3_HUMAN G-protein coupled receptor 3 OS=Homo sapiens GN=GPR3 PE=1 SV=1 | 317 | VCCCCSsSKIP | 0.184 | -0.194 | -1.212 | -0.407 |
| sp\|P46089\|GPR3_HUMAN G-protein coupled receptor 3 OS=Homo sapiens GN=GPR3 PE=1 SV=1 | 318 | CCCCSSsKIPF | 0.313 | 0.114 | -0.395 | 0.011 |
| sp\|P46089\|GPR3_HUMAN G-protein coupled receptor 3 OS=Homo sapiens GN=GPR3 PE=1 SV=1 | 324 | SKIPFRsRSPS | 0.048 | -0.32 | -1.825 | -0.699 |
| sp\|P46089\|GPR3_HUMAN G-protein coupled receptor 3 OS=Homo sapiens GN=GPR3 PE=1 SV=1 | 326 | IPFRSRsPSDV | 0.568 | 0.728 | -0.073 | 0.408 |
| sp\|P46089\|GPR3_HUMAN G-protein coupled receptor 3 OS=Homo sapiens GN=GPR3 PE=1 SV=1 | 328 | FRSRSPsDV-- | 0.84 | 1.443 | 0.84 | 1.041 |
| sp\|P46093\|GPR4_HUMAN G-protein coupled receptor 4 OS=Homo sapiens GN=GPR4 PE=2 SV=2 | 295 | VNEGARsDVAK | 0.183 | -0.061 | -0.799 | -0.226 |
| sp\|P46093\|GPR4_HUMAN G-protein coupled receptor 4 OS=Homo sapiens GN=GPR4 PE=2 SV=2 | 310 | LLRFLAsDKPQ | 0.448 | 0.227 | -0.435 | 0.08 |
| sp\|P46093\|GPR4_HUMAN G-protein coupled receptor 4 OS=Homo sapiens GN=GPR4 PE=2 SV=2 | 320 | QEMANAsLTLE | 0.418 | 0.024 | -0.441 | 0 |
| sp\|P46093\|GPR4_HUMAN G-protein coupled receptor 4 OS=Homo sapiens GN=GPR4 PE=2 SV=2 | 322 | MANASLtLETP | 0.297 | 0.124 | -0.439 | -0.006 |
| sp\|P46093\|GPR4_HUMAN G-protein coupled receptor 4 OS=Homo sapiens GN=GPR4 PE=2 SV=2 | 325 | ASLTLEtPLTS | 0.027 | -0.633 | -2.139 | -0.915 |
| sp\|P46093\|GPR4_HUMAN G-protein coupled receptor 4 OS=Homo sapiens GN=GPR4 PE=2 SV=2 | 328 | TLETPLtSKRN | 0.114 | -0.254 | -1.207 | -0.449 |
| sp\|P46093\|GPR4_HUMAN G-protein coupled receptor 4 OS=Homo sapiens GN=GPR4 PE=2 SV=2 | 329 | LETPLTsKRNS | 0.091 | -0.215 | -1.201 | -0.442 |
| sp\|P46093\|GPR4_HUMAN G-protein coupled receptor 4 OS=Homo sapiens GN=GPR4 PE=2 SV=2 | 333 | LTSKRNsTAKA | 0.416 | 0.038 | -0.551 | -0.032 |
| sp\|P46093\|GPR4_HUMAN G-protein coupled receptor 4 OS=Homo sapiens GN=GPR4 PE=2 SV=2 | 334 | TSKRNStAKAM | 0.579 | 0.792 | 0.42 | 0.597 |
| sp\|P46093\|GPR4_HUMAN G-protein coupled receptor 4 OS=Homo sapiens GN=GPR4 PE=2 SV=2 | 339 | STAKAMtGSWA | 0.29 | 0.026 | -0.507 | -0.064 |
| sp\|P46093\|GPR4_HUMAN G-protein coupled receptor 4 OS=Homo sapiens GN=GPR4 PE=2 SV=2 | 341 | AKAMTGsWAAT | 0.141 | -0.043 | -0.77 | -0.224 |
| sp\|P46093\|GPR4_HUMAN G-protein coupled receptor 4 OS=Homo sapiens GN=GPR4 PE=2 SV=2 | 345 | TGSWAAtPPSQ | 0.32 | 0.081 | -0.437 | -0.012 |
| sp\|P46093\|GPR4_HUMAN G-protein coupled receptor 4 OS=Homo sapiens GN=GPR4 PE=2 SV=2 | 348 | WAATPPsQGDQ | 0.17 | -0.278 | -0.955 | -0.354 |
| sp\|P46095\|GPR6_HUMAN G-protein coupled receptor 6 OS=Homo sapiens GN=GPR6 PE=1 SV=1 | 350 | LCGCFQsKVPF | 0.134 | -0.121 | -1.104 | -0.364 |
| sp\|P46095\|GPR6_HUMAN G-protein coupled receptor 6 OS=Homo sapiens GN=GPR6 PE=1 SV=1 | 356 | SKVPFRsRSPS | 0.047 | -0.346 | -1.841 | -0.713 |
| sp\|P46095\|GPR6_HUMAN G-protein coupled receptor 6 OS=Homo sapiens GN=GPR6 PE=1 SV=1 | 358 | VPFRSRsPSEV | 0.405 | 0.654 | -0.404 | 0.218 |
| sp\|P46095\|GPR6_HUMAN G-protein coupled receptor 6 OS=Homo sapiens GN=GPR6 PE=1 SV=1 | 360 | FRSRSPsEV-- | 0.837 | 1.485 | 0.977 | 1.1 |
| sp\|P47775\|GPR12_HUMAN G-protein coupled receptor 12 OS=Homo sapiens GN=GPR12 PE=1 SV=1 | 322 | CCGCIPsSLAQ | 0.162 | -0.09 | -0.969 | -0.299 |
| sp\|P47775\|GPR12_HUMAN G-protein coupled receptor 12 OS=Homo sapiens GN=GPR12 PE=1 SV=1 | 323 | CGCIPSsLAQR | 0.213 | -0.123 | -0.473 | -0.128 |
| sp\|P47775\|GPR12_HUMAN G-protein coupled receptor 12 OS=Homo sapiens GN=GPR12 PE=1 SV=1 | 330 | LAQRARsPSDV | 0.418 | 0.612 | -0.377 | 0.218 |
| sp\|P47775\|GPR12_HUMAN G-protein coupled receptor 12 OS=Homo sapiens GN=GPR12 PE=1 SV=1 | 332 | QRARSPsDV-- | 0.807 | 1.372 | 0.85 | 1.01 |
| sp\|P49685\|GPR15_HUMAN G-protein coupled receptor 15 OS=Homo sapiens GN=GPR15 PE=2 SV=1 | 328 | KNYDFGsSTET | 0.071 | -0.341 | -1.6 | -0.623 |
| sp\|P49685\|GPR15_HUMAN G-protein coupled receptor 15 OS=Homo sapiens GN=GPR15 PE=2 SV=1 | 329 | NYDFGSsTETS | 0.071 | -0.214 | -1.422 | -0.522 |
| sp\|P49685\|GPR15_HUMAN G-protein coupled receptor 15 OS=Homo sapiens GN=GPR15 PE=2 SV=1 | 330 | YDFGSStETSD | 0.161 | 0.083 | -0.621 | -0.126 |
| sp\|P49685\|GPR15_HUMAN G-protein coupled receptor 15 OS=Homo sapiens GN=GPR15 PE=2 SV=1 | 332 | FGSSTEtSDSH | 0.047 | -0.252 | -1.611 | -0.605 |
| sp\|P49685\|GPR15_HUMAN G-protein coupled receptor 15 OS=Homo sapiens GN=GPR15 PE=2 SV=1 | 333 | GSSTETsDSHL | 0.132 | -0.197 | -1.223 | -0.429 |
| sp\|P49685\|GPR15_HUMAN G-protein coupled receptor 15 OS=Homo sapiens GN=GPR15 PE=2 SV=1 | 335 | STETSDsHLTK | 0.213 | -0.114 | -1.037 | -0.313 |
| sp\|P49685\|GPR15_HUMAN G-protein coupled receptor 15 OS=Homo sapiens GN=GPR15 PE=2 SV=1 | 338 | TSDSHLtKALS | 0.059 | -0.29 | -1.442 | -0.558 |
| sp\|P49685\|GPR15_HUMAN G-protein coupled receptor 15 OS=Homo sapiens GN=GPR15 PE=2 SV=1 | 342 | HLTKALsTFIH | 0.394 | 0.091 | -0.361 | 0.041 |
| sp\|P49685\|GPR15_HUMAN G-protein coupled receptor 15 OS=Homo sapiens GN=GPR15 PE=2 SV=1 | 343 | LTKALStFIHA | 0.118 | -0.191 | -0.759 | -0.277 |
| sp\|P49685\|GPR15_HUMAN G-protein coupled receptor 15 OS=Homo sapiens GN=GPR15 PE=2 SV=1 | 357 | ARRRKRsVSL- | 0.639 | 1.21 | 0.196 | 0.682 |
| sp\|P49685\|GPR15_HUMAN G-protein coupled receptor 15 OS=Homo sapiens GN=GPR15 PE=2 SV=1 | 359 | RRKRSVsL--- | 0.958 | 1.629 | 1.8 | 1.462 |
| sp\|Q13304\|GPR17_HUMAN Uracil nucleotide/cysteinyl leukotriene receptor OS=Homo sapiens GN=GPR17 P | 310 | ALANRItSCLT | 0.248 | -0.002 | -0.528 | -0.094 |
| sp\|Q13304\|GPR17_HUMAN Uracil nucleotide/cysteinyl leukotriene receptor OS=Homo sapiens GN=GPR17 P | 311 | LANRITsCLTS | 0.379 | 0.768 | -0.073 | 0.358 |
| sp\|Q13304\|GPR17_HUMAN Uracil nucleotide/cysteinyl leukotriene receptor OS=Homo sapiens GN=GPR17 P | 314 | RITSCLtSLNG | 0.167 | -0.008 | -1.08 | -0.307 |
| sp\|Q13304\|GPR17_HUMAN Uracil nucleotide/cysteinyl leukotriene receptor OS=Homo sapiens GN=GPR17 P | 315 | ITSCLTsLNGA | 0.442 | -0.087 | -0.261 | 0.031 |
| sp\|Q13304\|GPR17_HUMAN Uracil nucleotide/cysteinyl leukotriene receptor OS=Homo sapiens GN=GPR17 P | 351 | LKGPPPsFEGK | 0.17 | -0.218 | -0.599 | -0.216 |
| sp\|Q13304\|GPR17_HUMAN Uracil nucleotide/cysteinyl leukotriene receptor OS=Homo sapiens GN=GPR17 P | 356 | PSFEGKtNESS | 0.049 | -0.214 | -1.476 | -0.547 |
| sp\|Q13304\|GPR17_HUMAN Uracil nucleotide/cysteinyl leukotriene receptor OS=Homo sapiens GN=GPR17 P | 359 | EGKTNEsSLSA | 0.065 | -0.247 | -1.255 | -0.479 |
| sp\|Q13304\|GPR17_HUMAN Uracil nucleotide/cysteinyl leukotriene receptor OS=Homo sapiens GN=GPR17 P | 360 | GKTNESsLSAK | 0.343 | 0.032 | -0.3 | 0.025 |

| | | | | | | |
|---|---|---|---|---|---|---|
| sp\|Q13304\|GPR17_HUMAN Uracil nucleotide/cysteinyl leukotriene receptor OS=Homo sapiens GN=GPR17 P | 362 | TNESSLsAKSE | 0.092 | -0.123 | -1.081 | -0.371 |
| sp\|Q13304\|GPR17_HUMAN Uracil nucleotide/cysteinyl leukotriene receptor OS=Homo sapiens GN=GPR17 P | 365 | SSLSAKsEL-- | 0.192 | 0.098 | -0.754 | -0.155 |
| sp\|Q15760\|GPR19_HUMAN Probable G-protein coupled receptor 19 OS=Homo sapiens GN=GPR19 PE=2 SV= | 308 | EQDYKKsSLVF | 0.058 | -0.121 | -1.022 | -0.362 |
| sp\|Q15760\|GPR19_HUMAN Probable G-protein coupled receptor 19 OS=Homo sapiens GN=GPR19 PE=2 SV= | 309 | QDYKKSsLVFT | 0.066 | -0.248 | -1.12 | -0.434 |
| sp\|Q15760\|GPR19_HUMAN Probable G-protein coupled receptor 19 OS=Homo sapiens GN=GPR19 PE=2 SV= | 313 | KSSLVFtAITW | 0.119 | -0.102 | -1.101 | -0.361 |
| sp\|Q15760\|GPR19_HUMAN Probable G-protein coupled receptor 19 OS=Homo sapiens GN=GPR19 PE=2 SV= | 316 | LVFTAItWISF | 0.107 | 0.038 | -0.829 | -0.228 |
| sp\|Q15760\|GPR19_HUMAN Probable G-protein coupled receptor 19 OS=Homo sapiens GN=GPR19 PE=2 SV= | 319 | TAITWIsFSSS | 0.094 | -0.137 | -1.205 | -0.416 |
| sp\|Q15760\|GPR19_HUMAN Probable G-protein coupled receptor 19 OS=Homo sapiens GN=GPR19 PE=2 SV= | 321 | ITWISFsSSAS | 0.241 | 0.269 | -0.448 | 0.021 |
| sp\|Q15760\|GPR19_HUMAN Probable G-protein coupled receptor 19 OS=Homo sapiens GN=GPR19 PE=2 SV= | 322 | TWISFSsSASK | 0.033 | -0.352 | -1.845 | -0.721 |
| sp\|Q15760\|GPR19_HUMAN Probable G-protein coupled receptor 19 OS=Homo sapiens GN=GPR19 PE=2 SV= | 323 | WISFSSsASKP | 0.141 | 0.039 | -0.925 | -0.248 |
| sp\|Q15760\|GPR19_HUMAN Probable G-protein coupled receptor 19 OS=Homo sapiens GN=GPR19 PE=2 SV= | 325 | SFSSSAsKPTL | 0.682 | 0.605 | 0.401 | 0.563 |
| sp\|Q15760\|GPR19_HUMAN Probable G-protein coupled receptor 19 OS=Homo sapiens GN=GPR19 PE=2 SV= | 328 | SSASKPtLYSI | 0.108 | -0.218 | -0.956 | -0.355 |
| sp\|Q15760\|GPR19_HUMAN Probable G-protein coupled receptor 19 OS=Homo sapiens GN=GPR19 PE=2 SV= | 331 | SKPTLYsIYNA | 0.098 | -0.086 | -1.177 | -0.388 |
| sp\|Q15760\|GPR19_HUMAN Probable G-protein coupled receptor 19 OS=Homo sapiens GN=GPR19 PE=2 SV= | 344 | RRGMKEtFCMS | 0.106 | -0.049 | -1.265 | -0.403 |
| sp\|Q15760\|GPR19_HUMAN Probable G-protein coupled receptor 19 OS=Homo sapiens GN=GPR19 PE=2 SV= | 348 | KETFCMsSMKC | 0.233 | -0.034 | -1.057 | -0.286 |
| sp\|Q15760\|GPR19_HUMAN Probable G-protein coupled receptor 19 OS=Homo sapiens GN=GPR19 PE=2 SV= | 349 | ETFCMSsMKCY | 0.18 | -0.021 | -1.135 | -0.325 |
| sp\|Q15760\|GPR19_HUMAN Probable G-protein coupled receptor 19 OS=Homo sapiens GN=GPR19 PE=2 SV= | 355 | SMKCYRsNAYT | 0.077 | -0.148 | -1.633 | -0.568 |
| sp\|Q15760\|GPR19_HUMAN Probable G-protein coupled receptor 19 OS=Homo sapiens GN=GPR19 PE=2 SV= | 359 | YRSNAYtITTS | 0.215 | 0.202 | -0.363 | 0.018 |
| sp\|Q15760\|GPR19_HUMAN Probable G-protein coupled receptor 19 OS=Homo sapiens GN=GPR19 PE=2 SV= | 361 | SNAYTItTSSR | 0.044 | -0.243 | -1.319 | -0.506 |
| sp\|Q15760\|GPR19_HUMAN Probable G-protein coupled receptor 19 OS=Homo sapiens GN=GPR19 PE=2 SV= | 362 | NAYTITtSSRM | 0.079 | -0.3 | -1.29 | -0.504 |
| sp\|Q15760\|GPR19_HUMAN Probable G-protein coupled receptor 19 OS=Homo sapiens GN=GPR19 PE=2 SV= | 363 | AYTITTsSRMA | 0.129 | -0.159 | -0.992 | -0.341 |
| sp\|Q15760\|GPR19_HUMAN Probable G-protein coupled receptor 19 OS=Homo sapiens GN=GPR19 PE=2 SV= | 364 | YTITTSsRMAK | 0.095 | -0.149 | -1.23 | -0.428 |
| sp\|Q15760\|GPR19_HUMAN Probable G-protein coupled receptor 19 OS=Homo sapiens GN=GPR19 PE=2 SV= | 375 | KNYVGIsEIPS | 0.035 | -0.269 | -1.353 | -0.529 |
| sp\|Q15760\|GPR19_HUMAN Probable G-protein coupled receptor 19 OS=Homo sapiens GN=GPR19 PE=2 SV= | 379 | GISEIPsMAKT | 0.095 | -0.064 | -1.175 | -0.381 |
| sp\|Q15760\|GPR19_HUMAN Probable G-protein coupled receptor 19 OS=Homo sapiens GN=GPR19 PE=2 SV= | 383 | IPSMAKtITKD | 0.368 | -0.077 | -0.324 | -0.011 |
| sp\|Q15760\|GPR19_HUMAN Probable G-protein coupled receptor 19 OS=Homo sapiens GN=GPR19 PE=2 SV= | 385 | SMAKTItKDSI | 0.213 | 0.137 | -0.514 | -0.055 |
| sp\|Q15760\|GPR19_HUMAN Probable G-protein coupled receptor 19 OS=Homo sapiens GN=GPR19 PE=2 SV= | 388 | KTITKDsIYDS | 0.102 | -0.228 | -1.435 | -0.52 |
| sp\|Q15760\|GPR19_HUMAN Probable G-protein coupled receptor 19 OS=Homo sapiens GN=GPR19 PE=2 SV= | 392 | KDSIYDsFDRE | 0.153 | -0.246 | -1.179 | -0.424 |
| sp\|Q15760\|GPR19_HUMAN Probable G-protein coupled receptor 19 OS=Homo sapiens GN=GPR19 PE=2 SV= | 408 | LAWPINsNPPN | 0.387 | 0.438 | -0.279 | 0.182 |
| sp\|Q15760\|GPR19_HUMAN Probable G-protein coupled receptor 19 OS=Homo sapiens GN=GPR19 PE=2 SV= | 413 | NSNPPNtFV-- | 0.183 | 0.006 | -0.852 | -0.221 |
| sp\|Q99678\|GPR20_HUMAN G-protein coupled receptor 20 OS=Homo sapiens GN=GPR20 PE=1 SV=2 | 301 | IVYCFVtSGFQ | 0.223 | -0.173 | -0.962 | -0.304 |
| sp\|Q99678\|GPR20_HUMAN G-protein coupled receptor 20 OS=Homo sapiens GN=GPR20 PE=1 SV=2 | 302 | VYCFVTsGFQA | 0.301 | -0.085 | -0.566 | -0.117 |
| sp\|Q99678\|GPR20_HUMAN G-protein coupled receptor 20 OS=Homo sapiens GN=GPR20 PE=1 SV=2 | 307 | TSGFQAtVRGL | 0.148 | -0.15 | -0.979 | -0.327 |
| sp\|Q99678\|GPR20_HUMAN G-protein coupled receptor 20 OS=Homo sapiens GN=GPR20 PE=1 SV=2 | 321 | HGEREPsSGDV | 0.684 | 0.838 | 0.3 | 0.607 |
| sp\|Q99678\|GPR20_HUMAN G-protein coupled receptor 20 OS=Homo sapiens GN=GPR20 PE=1 SV=2 | 322 | GEREPSsGDVV | 0.188 | 0.07 | -1.032 | -0.258 |
| sp\|Q99678\|GPR20_HUMAN G-protein coupled receptor 20 OS=Homo sapiens GN=GPR20 PE=1 SV=2 | 327 | SSGDVVsMHRS | 0.146 | -0.146 | -1.484 | -0.495 |
| sp\|Q99678\|GPR20_HUMAN G-protein coupled receptor 20 OS=Homo sapiens GN=GPR20 PE=1 SV=2 | 331 | VVSMHRsSKGS | 0.082 | -0.24 | -1.296 | -0.485 |
| sp\|Q99678\|GPR20_HUMAN G-protein coupled receptor 20 OS=Homo sapiens GN=GPR20 PE=1 SV=2 | 332 | VSMHRSsKGSG | 0.169 | -0.035 | -0.741 | -0.202 |
| sp\|Q99678\|GPR20_HUMAN G-protein coupled receptor 20 OS=Homo sapiens GN=GPR20 PE=1 SV=2 | 335 | HRSSKGsGRHH | 0.043 | -0.271 | -1.489 | -0.572 |
| sp\|Q99678\|GPR20_HUMAN G-protein coupled receptor 20 OS=Homo sapiens GN=GPR20 PE=1 SV=2 | 342 | GRHHILsAGPH | 0.128 | 0.034 | -1.068 | -0.302 |
| sp\|Q99678\|GPR20_HUMAN G-protein coupled receptor 20 OS=Homo sapiens GN=GPR20 PE=1 SV=2 | 349 | AGPHALtQALA | 0.082 | -0.265 | -1.359 | -0.514 |
| sp\|Q99679\|GPR21_HUMAN Probable G-protein coupled receptor 21 OS=Homo sapiens GN=GPR21 PE=2 SV= | 309 | IYSLSNsVFQR | 0.638 | 0.26 | 0.136 | 0.345 |
| sp\|Q99679\|GPR21_HUMAN Probable G-protein coupled receptor 21 OS=Homo sapiens GN=GPR21 PE=2 SV= | 319 | RGLKRLsGAMC | 0.214 | -0.096 | -0.904 | -0.262 |
| sp\|Q99679\|GPR21_HUMAN Probable G-protein coupled receptor 21 OS=Homo sapiens GN=GPR21 PE=2 SV= | 324 | LSGAMCtSCAS | 0.1 | -0.294 | -1.249 | -0.481 |
| sp\|Q99679\|GPR21_HUMAN Probable G-protein coupled receptor 21 OS=Homo sapiens GN=GPR21 PE=2 SV= | 325 | SGAMCTsCASQ | 0.103 | -0.204 | -1.145 | -0.415 |
| sp\|Q99679\|GPR21_HUMAN Probable G-protein coupled receptor 21 OS=Homo sapiens GN=GPR21 PE=2 SV= | 328 | MCTSCAsQTTA | 0.495 | -0.007 | -0.658 | -0.057 |
| sp\|Q99679\|GPR21_HUMAN Probable G-protein coupled receptor 21 OS=Homo sapiens GN=GPR21 PE=2 SV= | 330 | TSCASQtTAND | 0.308 | 0.073 | -0.563 | -0.061 |
| sp\|Q99679\|GPR21_HUMAN Probable G-protein coupled receptor 21 OS=Homo sapiens GN=GPR21 PE=2 SV= | 331 | SCASQTtANDP | 0.155 | -0.054 | -1.086 | -0.328 |
| sp\|Q99679\|GPR21_HUMAN Probable G-protein coupled receptor 21 OS=Homo sapiens GN=GPR21 PE=2 SV= | 337 | TANDPYtVRSK | 0.068 | -0.268 | -1.226 | -0.475 |

| | | | | | | |
|---|---|---|---|---|---|---|
| sp\|Q99679\|GPR21_HUMAN Probable G-protein coupled receptor 21 OS=Homo sapiens GN=GPR21 PE=2 SV= | 340 | DPYTVRsKGPL | 0.158 | -0.111 | -1.119 | -0.357 |
| sp\|Q99680\|GPR22_HUMAN Probable G-protein coupled receptor 22 OS=Homo sapiens GN=GPR22 PE=2 SV= | 380 | FQKVLKsKMKK | 0.162 | -0.025 | -0.631 | -0.165 |
| sp\|Q99680\|GPR22_HUMAN Probable G-protein coupled receptor 22 OS=Homo sapiens GN=GPR22 PE=2 SV= | 388 | MKKRVVsIVEA | 0.901 | 1.003 | 1.08 | 0.995 |
| sp\|Q99680\|GPR22_HUMAN Probable G-protein coupled receptor 22 OS=Homo sapiens GN=GPR22 PE=2 SV= | 404 | NAVIHNsWIDP | 0.214 | 0.114 | -0.764 | -0.145 |
| sp\|Q99680\|GPR22_HUMAN Probable G-protein coupled receptor 22 OS=Homo sapiens GN=GPR22 PE=2 SV= | 415 | KRNKKItFEDS | 0.397 | 0.241 | -0.435 | 0.068 |
| sp\|Q99680\|GPR22_HUMAN Probable G-protein coupled receptor 22 OS=Homo sapiens GN=GPR22 PE=2 SV= | 419 | KITFEDsEIRE | 0.198 | -0.118 | -1.105 | -0.342 |
| sp\|Q99680\|GPR22_HUMAN Probable G-protein coupled receptor 22 OS=Homo sapiens GN=GPR22 PE=2 SV= | 432 | LVPQVVtD--- | 0.276 | 0.095 | -0.684 | -0.104 |
| sp\|O00155\|GPR25_HUMAN Probable G-protein coupled receptor 25 OS=Homo sapiens GN=GPR25 PE=1 SV= | 313 | YLLLDRsFRAR | 0.366 | 0.11 | -0.287 | 0.063 |
| sp\|O00155\|GPR25_HUMAN Probable G-protein coupled receptor 25 OS=Homo sapiens GN=GPR25 PE=1 SV= | 326 | DGACGRtGRLA | 0.112 | -0.279 | -1.161 | -0.443 |
| sp\|O00155\|GPR25_HUMAN Probable G-protein coupled receptor 25 OS=Homo sapiens GN=GPR25 PE=1 SV= | 334 | RLARRIsSASS | 0.69 | 0.963 | 0.43 | 0.694 |
| sp\|O00155\|GPR25_HUMAN Probable G-protein coupled receptor 25 OS=Homo sapiens GN=GPR25 PE=1 SV= | 335 | LARRISsASSL | 0.421 | 0.937 | 0.114 | 0.491 |
| sp\|O00155\|GPR25_HUMAN Probable G-protein coupled receptor 25 OS=Homo sapiens GN=GPR25 PE=1 SV= | 337 | RRISSAsSLSR | 0.245 | 0.343 | -0.64 | -0.017 |
| sp\|O00155\|GPR25_HUMAN Probable G-protein coupled receptor 25 OS=Homo sapiens GN=GPR25 PE=1 SV= | 338 | RISSASsLSRD | 0.311 | -0.015 | -0.632 | -0.112 |
| sp\|O00155\|GPR25_HUMAN Probable G-protein coupled receptor 25 OS=Homo sapiens GN=GPR25 PE=1 SV= | 340 | SSASSLsRDDS | 0.176 | -0.054 | -0.804 | -0.227 |
| sp\|O00155\|GPR25_HUMAN Probable G-protein coupled receptor 25 OS=Homo sapiens GN=GPR25 PE=1 SV= | 344 | SLSRDDsSVFR | 0.349 | 0.622 | -0.385 | 0.195 |
| sp\|O00155\|GPR25_HUMAN Probable G-protein coupled receptor 25 OS=Homo sapiens GN=GPR25 PE=1 SV= | 345 | LSRDDSsVFRC | 0.176 | -0.08 | -1.099 | -0.334 |
| sp\|O00155\|GPR25_HUMAN Probable G-protein coupled receptor 25 OS=Homo sapiens GN=GPR25 PE=1 SV= | 356 | RAQAANtASAS | 0.348 | 0.129 | -0.718 | -0.08 |
| sp\|O00155\|GPR25_HUMAN Probable G-protein coupled receptor 25 OS=Homo sapiens GN=GPR25 PE=1 SV= | 358 | QAANTAsASW- | 0.084 | -0.026 | -1.031 | -0.324 |
| sp\|O00155\|GPR25_HUMAN Probable G-protein coupled receptor 25 OS=Homo sapiens GN=GPR25 PE=1 SV= | 360 | ANTASAsW--- | 0.373 | 0.307 | -0.299 | 0.127 |
| sp\|Q8NDV2\|GPR26_HUMAN G-protein coupled receptor 26 OS=Homo sapiens GN=GPR26 PE=1 SV=1 | 304 | RHQYRKsCKEI | 0.669 | 0.441 | 0.096 | 0.402 |
| sp\|Q8NDV2\|GPR26_HUMAN G-protein coupled receptor 26 OS=Homo sapiens GN=GPR26 PE=1 SV=1 | 317 | RLLHRRsIHSS | 0.235 | 0.119 | -0.74 | -0.129 |
| sp\|Q8NDV2\|GPR26_HUMAN G-protein coupled receptor 26 OS=Homo sapiens GN=GPR26 PE=1 SV=1 | 320 | HRRSIHsSGLT | 0.491 | 0.547 | 0.093 | 0.377 |
| sp\|Q8NDV2\|GPR26_HUMAN G-protein coupled receptor 26 OS=Homo sapiens GN=GPR26 PE=1 SV=1 | 321 | RRSIHSsGLTG | 0.137 | 0.091 | -1.013 | -0.262 |
| sp\|Q8NDV2\|GPR26_HUMAN G-protein coupled receptor 26 OS=Homo sapiens GN=GPR26 PE=1 SV=1 | 324 | IHSSGLtGDSH | 0.028 | -0.44 | -1.939 | -0.784 |
| sp\|Q8NDV2\|GPR26_HUMAN G-protein coupled receptor 26 OS=Homo sapiens GN=GPR26 PE=1 SV=1 | 327 | SGLTGDsHSQN | 0.072 | -0.349 | -1.543 | -0.607 |
| sp\|Q8NDV2\|GPR26_HUMAN G-protein coupled receptor 26 OS=Homo sapiens GN=GPR26 PE=1 SV=1 | 329 | LTGDSHsQNIL | 0.468 | 0.284 | -0.371 | 0.127 |
| sp\|Q8NDV2\|GPR26_HUMAN G-protein coupled receptor 26 OS=Homo sapiens GN=GPR26 PE=1 SV=1 | 336 | QNILPVsE--- | 0.194 | 0.035 | -0.744 | -0.172 |
| sp\|Q9NS67\|GPR27_HUMAN Probable G-protein coupled receptor 27 OS=Homo sapiens GN=GPR27 PE=2 SV= | 358 | QFPCCQsPRTT | 0.106 | -0.481 | -1.139 | -0.505 |
| sp\|Q9NS67\|GPR27_HUMAN Probable G-protein coupled receptor 27 OS=Homo sapiens GN=GPR27 PE=2 SV= | 361 | CCQSPRtTQAT | 0.188 | -0.037 | -0.782 | -0.21 |
| sp\|Q9NS67\|GPR27_HUMAN Probable G-protein coupled receptor 27 OS=Homo sapiens GN=GPR27 PE=2 SV= | 362 | CQSPRTtQATH | 0.129 | -0.206 | -1.064 | -0.38 |
| sp\|Q9NS67\|GPR27_HUMAN Probable G-protein coupled receptor 27 OS=Homo sapiens GN=GPR27 PE=2 SV= | 365 | PRTTQAtHPCD | 0.751 | 0.805 | 0.324 | 0.627 |
| sp\|O00270\|GPR31_HUMAN 12-(S)-hydroxy-5,8,10,14-eicosatetraenoic acid receptor OS=Homo sapiens GN=( | 317 | DFNPRDsYS-- | 0.171 | -0.004 | -1.151 | -0.328 |
| sp\|O00270\|GPR31_HUMAN 12-(S)-hydroxy-5,8,10,14-eicosatetraenoic acid receptor OS=Homo sapiens GN=( | 319 | NPRDSYs---- | 0.604 | 0.624 | 0.087 | 0.438 |
| sp\|O75388\|GPR32_HUMAN Probable G-protein coupled receptor 32 OS=Homo sapiens GN=GPR32 PE=2 SV= | 301 | LLILQAsFALG | 0.29 | 0.14 | -0.623 | -0.064 |
| sp\|O75388\|GPR32_HUMAN Probable G-protein coupled receptor 32 OS=Homo sapiens GN=GPR32 PE=2 SV= | 309 | ALGCVNsSLNP | 0.167 | -0.053 | -1.37 | -0.419 |
| sp\|O75388\|GPR32_HUMAN Probable G-protein coupled receptor 32 OS=Homo sapiens GN=GPR32 PE=2 SV= | 310 | LGCVNSsLNPF | 0.145 | -0.107 | -0.558 | -0.173 |
| sp\|O75388\|GPR32_HUMAN Probable G-protein coupled receptor 32 OS=Homo sapiens GN=GPR32 PE=2 SV= | 330 | QEKFFQsLTSA | 0.314 | -0.01 | -0.559 | -0.085 |
| sp\|O75388\|GPR32_HUMAN Probable G-protein coupled receptor 32 OS=Homo sapiens GN=GPR32 PE=2 SV= | 332 | KFFQSLtSALA | 0.221 | 0.051 | -0.476 | -0.068 |
| sp\|O75388\|GPR32_HUMAN Probable G-protein coupled receptor 32 OS=Homo sapiens GN=GPR32 PE=2 SV= | 333 | FFQSLTsALAR | 0.214 | 0.114 | -0.703 | -0.125 |
| sp\|O75388\|GPR32_HUMAN Probable G-protein coupled receptor 32 OS=Homo sapiens GN=GPR32 PE=2 SV= | 346 | GEEEFLsSCPR | 0.056 | -0.31 | -1.658 | -0.637 |
| sp\|O75388\|GPR32_HUMAN Probable G-protein coupled receptor 32 OS=Homo sapiens GN=GPR32 PE=2 SV= | 347 | EEEFLSsCPRG | 0.349 | 0.327 | -0.639 | 0.012 |
| sp\|Q49SQ1\|GPR33_HUMAN Probable G-protein coupled receptor 33 OS=Homo sapiens GN=GPR33 PE=2 SV= | 313 | KKVFKKsILAL | 0.251 | 0.084 | -0.413 | -0.026 |
| sp\|Q49SQ1\|GPR33_HUMAN Probable G-protein coupled receptor 33 OS=Homo sapiens GN=GPR33 PE=2 SV= | 320 | ILALFEsTFSE | 0.176 | -0.108 | -0.966 | -0.299 |
| sp\|Q49SQ1\|GPR33_HUMAN Probable G-protein coupled receptor 33 OS=Homo sapiens GN=GPR33 PE=2 SV= | 321 | LALFEStFSED | 0.17 | -0.121 | -0.898 | -0.283 |
| sp\|Q49SQ1\|GPR33_HUMAN Probable G-protein coupled receptor 33 OS=Homo sapiens GN=GPR33 PE=2 SV= | 323 | LFESTFsEDSS | 0.111 | 0.025 | -0.708 | -0.191 |
| sp\|Q49SQ1\|GPR33_HUMAN Probable G-protein coupled receptor 33 OS=Homo sapiens GN=GPR33 PE=2 SV= | 326 | STFSEDsSVER | 0.098 | -0.297 | -1.681 | -0.627 |
| sp\|Q49SQ1\|GPR33_HUMAN Probable G-protein coupled receptor 33 OS=Homo sapiens GN=GPR33 PE=2 SV= | 327 | TFSEDSsVERT | 0.055 | -0.326 | -1.473 | -0.581 |
| sp\|Q49SQ1\|GPR33_HUMAN Probable G-protein coupled receptor 33 OS=Homo sapiens GN=GPR33 PE=2 SV= | 331 | DSSVERtQT-- | 0.088 | -0.167 | -1.809 | -0.629 |
| sp\|Q49SQ1\|GPR33_HUMAN Probable G-protein coupled receptor 33 OS=Homo sapiens GN=GPR33 PE=2 SV= | 333 | SVERTQt---- | 0.392 | 0.954 | -0.001 | 0.448 |

| | | | | | | |
|---|---|---|---|---|---|---|
| sp\|Q9UPC5\|GPR34_HUMAN Probable G-protein coupled receptor 34 OS=Homo sapiens GN=GPR34 PE=2 SV= | 308 | KEIVHKtNEIM | 0.18 | -0.052 | -1.073 | -0.315 |
| sp\|Q9UPC5\|GPR34_HUMAN Probable G-protein coupled receptor 34 OS=Homo sapiens GN=GPR34 PE=2 SV= | 316 | EIMLVLsSFNS | 0.082 | 0 | -1.287 | -0.402 |
| sp\|Q9UPC5\|GPR34_HUMAN Probable G-protein coupled receptor 34 OS=Homo sapiens GN=GPR34 PE=2 SV= | 317 | IMLVLSsFNSC | 0.055 | -0.102 | -1.309 | -0.452 |
| sp\|Q9UPC5\|GPR34_HUMAN Probable G-protein coupled receptor 34 OS=Homo sapiens GN=GPR34 PE=2 SV= | 320 | VLSSFNsCLDP | 0.245 | 0.012 | -0.948 | -0.23 |
| sp\|Q9UPC5\|GPR34_HUMAN Probable G-protein coupled receptor 34 OS=Homo sapiens GN=GPR34 PE=2 SV= | 331 | VMYFLMsSNIR | 0.184 | 0.136 | -0.723 | -0.134 |
| sp\|Q9UPC5\|GPR34_HUMAN Probable G-protein coupled receptor 34 OS=Homo sapiens GN=GPR34 PE=2 SV= | 332 | MYFLMSsNIRK | 0.151 | -0.183 | -1.148 | -0.393 |
| sp\|Q9UPC5\|GPR34_HUMAN Probable G-protein coupled receptor 34 OS=Homo sapiens GN=GPR34 PE=2 SV= | 351 | RFQGEPsRSES | 0.123 | -0.176 | -1.232 | -0.428 |
| sp\|Q9UPC5\|GPR34_HUMAN Probable G-protein coupled receptor 34 OS=Homo sapiens GN=GPR34 PE=2 SV= | 353 | QGEPSRsESTS | 0.14 | 0.02 | -0.778 | -0.206 |
| sp\|Q9UPC5\|GPR34_HUMAN Probable G-protein coupled receptor 34 OS=Homo sapiens GN=GPR34 PE=2 SV= | 355 | EPSRSEsTSEF | 0.488 | 0.843 | 0.07 | 0.467 |
| sp\|Q9UPC5\|GPR34_HUMAN Probable G-protein coupled receptor 34 OS=Homo sapiens GN=GPR34 PE=2 SV= | 356 | PSRSEStSEFK | 0.105 | -0.171 | -1.3 | -0.455 |
| sp\|Q9UPC5\|GPR34_HUMAN Probable G-protein coupled receptor 34 OS=Homo sapiens GN=GPR34 PE=2 SV= | 357 | SRSESTsEFKP | 0.164 | 0.292 | -0.664 | -0.069 |
| sp\|Q9UPC5\|GPR34_HUMAN Probable G-protein coupled receptor 34 OS=Homo sapiens GN=GPR34 PE=2 SV= | 364 | EFKPGYsLHDT | 0.348 | 0.101 | 0.017 | 0.155 |
| sp\|Q9UPC5\|GPR34_HUMAN Probable G-protein coupled receptor 34 OS=Homo sapiens GN=GPR34 PE=2 SV= | 368 | GYSLHDtSVAV | 0.293 | 0.024 | -1.037 | -0.24 |
| sp\|Q9UPC5\|GPR34_HUMAN Probable G-protein coupled receptor 34 OS=Homo sapiens GN=GPR34 PE=2 SV= | 369 | YSLHDTsVAVK | 0.117 | -0.215 | -0.923 | -0.34 |
| sp\|Q9UPC5\|GPR34_HUMAN Probable G-protein coupled receptor 34 OS=Homo sapiens GN=GPR34 PE=2 SV= | 376 | VAVKIQsSSKS | 0.088 | -0.211 | -1.203 | -0.442 |
| sp\|Q9UPC5\|GPR34_HUMAN Probable G-protein coupled receptor 34 OS=Homo sapiens GN=GPR34 PE=2 SV= | 377 | AVKIQSsSKST | 0.031 | -0.264 | -1.618 | -0.617 |
| sp\|Q9UPC5\|GPR34_HUMAN Probable G-protein coupled receptor 34 OS=Homo sapiens GN=GPR34 PE=2 SV= | 378 | VKIQSSsKST- | 0.306 | 0.118 | -0.371 | 0.018 |
| sp\|Q9UPC5\|GPR34_HUMAN Probable G-protein coupled receptor 34 OS=Homo sapiens GN=GPR34 PE=2 SV= | 380 | IQSSSKsT--- | 0.401 | 0.268 | -0.041 | 0.209 |
| sp\|Q9UPC5\|GPR34_HUMAN Probable G-protein coupled receptor 34 OS=Homo sapiens GN=GPR34 PE=2 SV= | 381 | QSSSKSt---- | 0.058 | -0.195 | -1.432 | -0.523 |
| sp\|Q9HC97\|GPR35_HUMAN G-protein coupled receptor 35 OS=Homo sapiens GN=GPR35 PE=2 SV=4 | 303 | AHKSQDsLCVT | 0.122 | -0.224 | -1.403 | -0.502 |
| sp\|Q9HC97\|GPR35_HUMAN G-protein coupled receptor 35 OS=Homo sapiens GN=GPR35 PE=2 SV=4 | 307 | QDSLCVtLA-- | 0.335 | 0.029 | -0.536 | -0.057 |
| sp\|O15354\|GPR37_HUMAN Prosaposin receptor GPR37 OS=Homo sapiens GN=GPR37 PE=1 SV=2 | 324 | VIFHELtKKWL | 0.123 | -0.058 | -0.899 | -0.278 |
| sp\|O15354\|GPR37_HUMAN Prosaposin receptor GPR37 OS=Homo sapiens GN=GPR37 PE=1 SV=2 | 333 | WLLEDFsCKIV | 0.223 | 0.064 | -0.773 | -0.162 |
| sp\|O15354\|GPR37_HUMAN Prosaposin receptor GPR37 OS=Homo sapiens GN=GPR37 PE=1 SV=2 | 344 | PYIEVAsLGVT | 0.447 | 0.19 | -0.326 | 0.104 |
| sp\|O15354\|GPR37_HUMAN Prosaposin receptor GPR37 OS=Homo sapiens GN=GPR37 PE=1 SV=2 | 348 | VASLGVtTFTL | 0.142 | -0.205 | -0.996 | -0.353 |
| sp\|O15354\|GPR37_HUMAN Prosaposin receptor GPR37 OS=Homo sapiens GN=GPR37 PE=1 SV=2 | 349 | ASLGVTtFTLC | 0.205 | -0.144 | -0.909 | -0.283 |
| sp\|O15354\|GPR37_HUMAN Prosaposin receptor GPR37 OS=Homo sapiens GN=GPR37 PE=1 SV=2 | 351 | LGVTTFtLCAL | 0.21 | 0.102 | -0.359 | -0.016 |
| sp\|O15354\|GPR37_HUMAN Prosaposin receptor GPR37 OS=Homo sapiens GN=GPR37 PE=1 SV=2 | 364 | DRFRAAtNVQM | 0.815 | 1.105 | 0.518 | 0.813 |
| sp\|O15354\|GPR37_HUMAN Prosaposin receptor GPR37 OS=Homo sapiens GN=GPR37 PE=1 SV=2 | 377 | EMIENCsSTTA | 0.121 | -0.036 | -1.176 | -0.364 |
| sp\|O15354\|GPR37_HUMAN Prosaposin receptor GPR37 OS=Homo sapiens GN=GPR37 PE=1 SV=2 | 378 | MIENCSsTTAK | 0.348 | 0.053 | -0.347 | 0.018 |
| sp\|O15354\|GPR37_HUMAN Prosaposin receptor GPR37 OS=Homo sapiens GN=GPR37 PE=1 SV=2 | 379 | IENCSStTAKL | 0.189 | 0 | -0.945 | -0.252 |
| sp\|O15354\|GPR37_HUMAN Prosaposin receptor GPR37 OS=Homo sapiens GN=GPR37 PE=1 SV=2 | 380 | ENCSSTtAKLA | 0.217 | 0.045 | -0.556 | -0.098 |
| sp\|O15354\|GPR37_HUMAN Prosaposin receptor GPR37 OS=Homo sapiens GN=GPR37 PE=1 SV=2 | 404 | VVLRQLsKEDL | 0.407 | 0.837 | 0.133 | 0.459 |
| sp\|O15354\|GPR37_HUMAN Prosaposin receptor GPR37 OS=Homo sapiens GN=GPR37 PE=1 SV=2 | 411 | KEDLGFsGRAP | 0.061 | -0.261 | -1.336 | -0.512 |
| sp\|O15354\|GPR37_HUMAN Prosaposin receptor GPR37 OS=Homo sapiens GN=GPR37 PE=1 SV=2 | 424 | RCIIKIsPDLP | 0.091 | -0.264 | -1.375 | -0.516 |
| sp\|O15354\|GPR37_HUMAN Prosaposin receptor GPR37 OS=Homo sapiens GN=GPR37 PE=1 SV=2 | 430 | SPDLPDtIYVL | 0.049 | -0.304 | -1.627 | -0.627 |
| sp\|O15354\|GPR37_HUMAN Prosaposin receptor GPR37 OS=Homo sapiens GN=GPR37 PE=1 SV=2 | 437 | IYVLALtYDSA | 0.159 | -0.047 | -0.813 | -0.234 |
| sp\|O15354\|GPR37_HUMAN Prosaposin receptor GPR37 OS=Homo sapiens GN=GPR37 PE=1 SV=2 | 440 | LALTYDsARLW | 0.154 | -0.174 | -1.155 | -0.392 |
| sp\|O15354\|GPR37_HUMAN Prosaposin receptor GPR37 OS=Homo sapiens GN=GPR37 PE=1 SV=2 | 455 | CYFCLPtLFTI | 0.557 | 0.091 | -0.087 | 0.187 |
| sp\|O15354\|GPR37_HUMAN Prosaposin receptor GPR37 OS=Homo sapiens GN=GPR37 PE=1 SV=2 | 458 | CLPTLFtITCS | 0.166 | -0.025 | -0.939 | -0.266 |
| sp\|O15354\|GPR37_HUMAN Prosaposin receptor GPR37 OS=Homo sapiens GN=GPR37 PE=1 SV=2 | 460 | PTLFTItCSLV | 0.196 | 0.05 | -1.051 | -0.268 |
| sp\|O15354\|GPR37_HUMAN Prosaposin receptor GPR37 OS=Homo sapiens GN=GPR37 PE=1 SV=2 | 462 | LFTITCsLVTA | 0.316 | 0.1 | -0.308 | 0.036 |
| sp\|O15354\|GPR37_HUMAN Prosaposin receptor GPR37 OS=Homo sapiens GN=GPR37 PE=1 SV=2 | 465 | ITCSLVtARKI | 0.354 | -0.142 | -0.787 | -0.192 |
| sp\|O15354\|GPR37_HUMAN Prosaposin receptor GPR37 OS=Homo sapiens GN=GPR37 PE=1 SV=2 | 477 | KAEKACtRGNK | 0.513 | -0.026 | -0.673 | -0.062 |
| sp\|O15354\|GPR37_HUMAN Prosaposin receptor GPR37 OS=Homo sapiens GN=GPR37 PE=1 SV=2 | 488 | RQIQLEsQMNC | 0.094 | -0.164 | -1.657 | -0.576 |
| sp\|O15354\|GPR37_HUMAN Prosaposin receptor GPR37 OS=Homo sapiens GN=GPR37 PE=1 SV=2 | 493 | ESQMNCtVVAL | 0.297 | 0.046 | -0.484 | -0.047 |
| sp\|O15354\|GPR37_HUMAN Prosaposin receptor GPR37 OS=Homo sapiens GN=GPR37 PE=1 SV=2 | 498 | CTVVALtILYG | 0.094 | -0.126 | -1.212 | -0.415 |
| sp\|O15354\|GPR37_HUMAN Prosaposin receptor GPR37 OS=Homo sapiens GN=GPR37 PE=1 SV=2 | 515 | NICNIVtAYMA | 0.296 | 0.02 | -0.488 | -0.057 |
| sp\|O15354\|GPR37_HUMAN Prosaposin receptor GPR37 OS=Homo sapiens GN=GPR37 PE=1 SV=2 | 520 | VTAYMAtGVSQ | 0.107 | -0.234 | -1.055 | -0.394 |

| | | | | | | |
|---|---|---|---|---|---|---|
| sp\|O15354\|GPR37_HUMAN Prosaposin receptor GPR37 OS=Homo sapiens GN=GPR37 PE=1 SV=2 | 523 | YMATGVsQQTM | 0.136 | -0.088 | -0.946 | -0.299 |
| sp\|O15354\|GPR37_HUMAN Prosaposin receptor GPR37 OS=Homo sapiens GN=GPR37 PE=1 SV=2 | 526 | TGVSQQtMDLL | 0.112 | 0.056 | -0.97 | -0.267 |
| sp\|O15354\|GPR37_HUMAN Prosaposin receptor GPR37 OS=Homo sapiens GN=GPR37 PE=1 SV=2 | 534 | DLLNIIsQFLL | 0.325 | 0.055 | -0.417 | -0.012 |
| sp\|O15354\|GPR37_HUMAN Prosaposin receptor GPR37 OS=Homo sapiens GN=GPR37 PE=1 SV=2 | 542 | FLLFFKsCVTP | 0.389 | 0.2 | -0.378 | 0.07 |
| sp\|O15354\|GPR37_HUMAN Prosaposin receptor GPR37 OS=Homo sapiens GN=GPR37 PE=1 SV=2 | 545 | FFKSCVtPVLL | 0.323 | -0.144 | -0.5 | -0.107 |
| sp\|O15354\|GPR37_HUMAN Prosaposin receptor GPR37 OS=Homo sapiens GN=GPR37 PE=1 SV=2 | 557 | CLCKPFsRAFM | 0.277 | -0.106 | -0.557 | -0.129 |
| sp\|O15354\|GPR37_HUMAN Prosaposin receptor GPR37 OS=Homo sapiens GN=GPR37 PE=1 SV=2 | 575 | EECIQKsSTVT | 0.121 | -0.081 | -1.153 | -0.371 |
| sp\|O15354\|GPR37_HUMAN Prosaposin receptor GPR37 OS=Homo sapiens GN=GPR37 PE=1 SV=2 | 576 | ECIQKSsTVTS | 0.068 | -0.169 | -1.368 | -0.49 |
| sp\|O15354\|GPR37_HUMAN Prosaposin receptor GPR37 OS=Homo sapiens GN=GPR37 PE=1 SV=2 | 577 | CIQKSStVTSD | 0.528 | 0.326 | 0.052 | 0.302 |
| sp\|O15354\|GPR37_HUMAN Prosaposin receptor GPR37 OS=Homo sapiens GN=GPR37 PE=1 SV=2 | 579 | QKSSTVtSDDN | 0.412 | 0.056 | -0.385 | 0.028 |
| sp\|O15354\|GPR37_HUMAN Prosaposin receptor GPR37 OS=Homo sapiens GN=GPR37 PE=1 SV=2 | 580 | KSSTVTsDDND | 0.286 | -0.047 | -0.875 | -0.212 |
| sp\|O15354\|GPR37_HUMAN Prosaposin receptor GPR37 OS=Homo sapiens GN=GPR37 PE=1 SV=2 | 588 | DNDNEYtTELE | 0.089 | -0.188 | -1.195 | -0.431 |
| sp\|O15354\|GPR37_HUMAN Prosaposin receptor GPR37 OS=Homo sapiens GN=GPR37 PE=1 SV=2 | 589 | NDNEYTtELEL | 0.096 | -0.041 | -1.149 | -0.365 |
| sp\|O15354\|GPR37_HUMAN Prosaposin receptor GPR37 OS=Homo sapiens GN=GPR37 PE=1 SV=2 | 594 | TTELELsPFST | 0.027 | -0.573 | -1.899 | -0.815 |
| sp\|O15354\|GPR37_HUMAN Prosaposin receptor GPR37 OS=Homo sapiens GN=GPR37 PE=1 SV=2 | 597 | LELSPFsTIRR | 0.089 | -0.257 | -1.047 | -0.405 |
| sp\|O15354\|GPR37_HUMAN Prosaposin receptor GPR37 OS=Homo sapiens GN=GPR37 PE=1 SV=2 | 598 | ELSPFStIRRE | 0.091 | -0.346 | -1.282 | -0.512 |
| sp\|O15354\|GPR37_HUMAN Prosaposin receptor GPR37 OS=Homo sapiens GN=GPR37 PE=1 SV=2 | 604 | TIRREMsTFAS | 0.862 | 1.273 | 0.899 | 1.011 |
| sp\|O15354\|GPR37_HUMAN Prosaposin receptor GPR37 OS=Homo sapiens GN=GPR37 PE=1 SV=2 | 605 | IRREMStFASV | 0.223 | 0.255 | -0.728 | -0.083 |
| sp\|O15354\|GPR37_HUMAN Prosaposin receptor GPR37 OS=Homo sapiens GN=GPR37 PE=1 SV=2 | 608 | EMSTFAsVGTH | 0.137 | -0.065 | -1.166 | -0.365 |
| sp\|O15354\|GPR37_HUMAN Prosaposin receptor GPR37 OS=Homo sapiens GN=GPR37 PE=1 SV=2 | 611 | TFASVGtHC-- | 0.108 | -0.145 | -1.157 | -0.398 |
| sp\|O60883\|ETBR2_HUMAN Prosaposin receptor GPR37L1 OS=Homo sapiens GN=GPR37L1 PE=1 SV=2 | 304 | LYSLVMtYQNA | 0.277 | 0.082 | -0.682 | -0.108 |
| sp\|O60883\|ETBR2_HUMAN Prosaposin receptor GPR37L1 OS=Homo sapiens GN=GPR37L1 PE=1 SV=2 | 325 | CLPILFtVTCQ | 0.203 | 0.05 | -0.833 | -0.193 |
| sp\|O60883\|ETBR2_HUMAN Prosaposin receptor GPR37L1 OS=Homo sapiens GN=GPR37L1 PE=1 SV=2 | 327 | PILFTVtCQLV | 0.42 | 0.242 | -0.475 | 0.062 |
| sp\|O60883\|ETBR2_HUMAN Prosaposin receptor GPR37L1 OS=Homo sapiens GN=GPR37L1 PE=1 SV=2 | 332 | VTCQLVtWRVR | 0.323 | -0.057 | -0.67 | -0.135 |
| sp\|O60883\|ETBR2_HUMAN Prosaposin receptor GPR37L1 OS=Homo sapiens GN=GPR37L1 PE=1 SV=2 | 343 | GPPGRKsECRA | 0.327 | 0.002 | -0.634 | -0.102 |
| sp\|O60883\|ETBR2_HUMAN Prosaposin receptor GPR37L1 OS=Homo sapiens GN=GPR37L1 PE=1 SV=2 | 348 | KSECRAsKHEQ | 0.503 | 0.022 | -0.541 | -0.005 |
| sp\|O60883\|ETBR2_HUMAN Prosaposin receptor GPR37L1 OS=Homo sapiens GN=GPR37L1 PE=1 SV=2 | 355 | KHEQCEsQLNS | 0.131 | -0.183 | -1.68 | -0.577 |
| sp\|O60883\|ETBR2_HUMAN Prosaposin receptor GPR37L1 OS=Homo sapiens GN=GPR37L1 PE=1 SV=2 | 359 | CESQLNsTVVG | 0.227 | -0.031 | -0.891 | -0.232 |
| sp\|O60883\|ETBR2_HUMAN Prosaposin receptor GPR37L1 OS=Homo sapiens GN=GPR37L1 PE=1 SV=2 | 360 | ESQLNStVVGL | 0.107 | -0.115 | -0.906 | -0.305 |
| sp\|O60883\|ETBR2_HUMAN Prosaposin receptor GPR37L1 OS=Homo sapiens GN=GPR37L1 PE=1 SV=2 | 365 | STVVGLtVVYA | 0.03 | -0.308 | -1.813 | -0.697 |
| sp\|O60883\|ETBR2_HUMAN Prosaposin receptor GPR37L1 OS=Homo sapiens GN=GPR37L1 PE=1 SV=2 | 372 | VVYAFCtLPEN | 0.731 | 0.529 | 0.141 | 0.467 |
| sp\|O60883\|ETBR2_HUMAN Prosaposin receptor GPR37L1 OS=Homo sapiens GN=GPR37L1 PE=1 SV=2 | 386 | IVVAYLsTELT | 0.092 | -0.167 | -1.085 | -0.387 |
| sp\|O60883\|ETBR2_HUMAN Prosaposin receptor GPR37L1 OS=Homo sapiens GN=GPR37L1 PE=1 SV=2 | 387 | VVAYLStELTR | 0.069 | -0.122 | -1.06 | -0.371 |
| sp\|O60883\|ETBR2_HUMAN Prosaposin receptor GPR37L1 OS=Homo sapiens GN=GPR37L1 PE=1 SV=2 | 390 | YLSTELtRQTL | 0.146 | -0.127 | -1.024 | -0.335 |
| sp\|O60883\|ETBR2_HUMAN Prosaposin receptor GPR37L1 OS=Homo sapiens GN=GPR37L1 PE=1 SV=2 | 393 | TELTRQtLDLL | 0.516 | 0.249 | -0.07 | 0.232 |
| sp\|O60883\|ETBR2_HUMAN Prosaposin receptor GPR37L1 OS=Homo sapiens GN=GPR37L1 PE=1 SV=2 | 404 | GLINQFsTFFK | 0.173 | 0.021 | -0.779 | -0.195 |
| sp\|O60883\|ETBR2_HUMAN Prosaposin receptor GPR37L1 OS=Homo sapiens GN=GPR37L1 PE=1 SV=2 | 405 | LINQFStFFKG | 0.199 | -0.003 | -0.717 | -0.174 |
| sp\|O60883\|ETBR2_HUMAN Prosaposin receptor GPR37L1 OS=Homo sapiens GN=GPR37L1 PE=1 SV=2 | 412 | FFKGAItPVLL | 0.239 | -0.054 | -0.473 | -0.096 |
| sp\|O60883\|ETBR2_HUMAN Prosaposin receptor GPR37L1 OS=Homo sapiens GN=GPR37L1 PE=1 SV=2 | 443 | EECGGAsEASA | 0.064 | -0.155 | -1.299 | -0.463 |
| sp\|O60883\|ETBR2_HUMAN Prosaposin receptor GPR37L1 OS=Homo sapiens GN=GPR37L1 PE=1 SV=2 | 446 | GGASEAsAANG | 0.235 | -0.05 | -1.069 | -0.295 |
| sp\|O60883\|ETBR2_HUMAN Prosaposin receptor GPR37L1 OS=Homo sapiens GN=GPR37L1 PE=1 SV=2 | 451 | ASAANGsDNKL | 0.168 | -0.173 | -0.889 | -0.298 |
| sp\|O60883\|ETBR2_HUMAN Prosaposin receptor GPR37L1 OS=Homo sapiens GN=GPR37L1 PE=1 SV=2 | 457 | SDNKLKtEVSS | 0.076 | -0.13 | -1.324 | -0.459 |
| sp\|O60883\|ETBR2_HUMAN Prosaposin receptor GPR37L1 OS=Homo sapiens GN=GPR37L1 PE=1 SV=2 | 460 | KLKTEVsSSIY | 0.412 | -0.058 | -0.781 | -0.142 |
| sp\|O60883\|ETBR2_HUMAN Prosaposin receptor GPR37L1 OS=Homo sapiens GN=GPR37L1 PE=1 SV=2 | 461 | LKTEVSsSIYF | 0.082 | -0.159 | -1.125 | -0.401 |
| sp\|O60883\|ETBR2_HUMAN Prosaposin receptor GPR37L1 OS=Homo sapiens GN=GPR37L1 PE=1 SV=2 | 462 | KTEVSSsIYFH | 0.065 | -0.216 | -1.378 | -0.51 |
| sp\|O60883\|ETBR2_HUMAN Prosaposin receptor GPR37L1 OS=Homo sapiens GN=GPR37L1 PE=1 SV=2 | 471 | FHKPREsPPLL | 0.506 | 0.387 | -0.333 | 0.187 |
| sp\|O60883\|ETBR2_HUMAN Prosaposin receptor GPR37L1 OS=Homo sapiens GN=GPR37L1 PE=1 SV=2 | 479 | PLLPLGtPC-- | 0.054 | -0.358 | -1.57 | -0.625 |
| sp\|O43194\|GPR39_HUMAN G-protein coupled receptor 39 OS=Homo sapiens GN=GPR39 PE=1 SV=1 | 315 | KPKHDWtRSYF | 0.074 | -0.385 | -1.492 | -0.601 |
| sp\|O43194\|GPR39_HUMAN G-protein coupled receptor 39 OS=Homo sapiens GN=GPR39 PE=1 SV=1 | 317 | KHDWTRsYFRA | 0.071 | -0.262 | -1.614 | -0.602 |

| | | | | | | |
|---|---|---|---|---|---|---|
| sp\|O43194\|GPR39_HUMAN G-protein coupled receptor 39 OS=Homo sapiens GN=GPR39 PE=1 SV=1 | 329 | MILLPFsETFF | 0.203 | 0.049 | -0.622 | -0.123 |
| sp\|O43194\|GPR39_HUMAN G-protein coupled receptor 39 OS=Homo sapiens GN=GPR39 PE=1 SV=1 | 331 | LLPFSEtFFYL | 0.492 | 0.286 | -0.115 | 0.221 |
| sp\|O43194\|GPR39_HUMAN G-protein coupled receptor 39 OS=Homo sapiens GN=GPR39 PE=1 SV=1 | 336 | ETFFYLsSVIN | 0.139 | -0.085 | -1.166 | -0.371 |
| sp\|O43194\|GPR39_HUMAN G-protein coupled receptor 39 OS=Homo sapiens GN=GPR39 PE=1 SV=1 | 337 | TFFYLSsVINP | 0.067 | -0.115 | -1.161 | -0.403 |
| sp\|O43194\|GPR39_HUMAN G-protein coupled receptor 39 OS=Homo sapiens GN=GPR39 PE=1 SV=1 | 345 | INPLLYtVSSQ | 0.037 | -0.302 | -1.56 | -0.608 |
| sp\|O43194\|GPR39_HUMAN G-protein coupled receptor 39 OS=Homo sapiens GN=GPR39 PE=1 SV=1 | 347 | PLLYTVsSQQF | 0.349 | 0.086 | -0.209 | 0.075 |
| sp\|O43194\|GPR39_HUMAN G-protein coupled receptor 39 OS=Homo sapiens GN=GPR39 PE=1 SV=1 | 348 | LLYTVSsQQFR | 0.114 | -0.156 | -1.179 | -0.407 |
| sp\|O43194\|GPR39_HUMAN G-protein coupled receptor 39 OS=Homo sapiens GN=GPR39 PE=1 SV=1 | 364 | VLCCRLsLQHA | 0.551 | 0.165 | -0.026 | 0.23 |
| sp\|O43194\|GPR39_HUMAN G-protein coupled receptor 39 OS=Homo sapiens GN=GPR39 PE=1 SV=1 | 380 | LRVHAHsTTDS | 0.474 | 0.364 | -0.26 | 0.193 |
| sp\|O43194\|GPR39_HUMAN G-protein coupled receptor 39 OS=Homo sapiens GN=GPR39 PE=1 SV=1 | 381 | RVHAHStTDSA | 0.09 | -0.131 | -1.186 | -0.409 |
| sp\|O43194\|GPR39_HUMAN G-protein coupled receptor 39 OS=Homo sapiens GN=GPR39 PE=1 SV=1 | 382 | VHAHSTtDSAR | 0.318 | 0.165 | -0.446 | 0.012 |
| sp\|O43194\|GPR39_HUMAN G-protein coupled receptor 39 OS=Homo sapiens GN=GPR39 PE=1 SV=1 | 384 | AHSTTDsARFV | 0.082 | -0.285 | -2.023 | -0.742 |
| sp\|O43194\|GPR39_HUMAN G-protein coupled receptor 39 OS=Homo sapiens GN=GPR39 PE=1 SV=1 | 396 | RPLLFAsRRQS | 0.187 | -0.22 | -1.174 | -0.402 |
| sp\|O43194\|GPR39_HUMAN G-protein coupled receptor 39 OS=Homo sapiens GN=GPR39 PE=1 SV=1 | 400 | FASRRQsSARR | 0.669 | 0.85 | 0.225 | 0.581 |
| sp\|O43194\|GPR39_HUMAN G-protein coupled receptor 39 OS=Homo sapiens GN=GPR39 PE=1 SV=1 | 401 | ASRRQSsARRT | 0.548 | 0.765 | -0.155 | 0.386 |
| sp\|O43194\|GPR39_HUMAN G-protein coupled receptor 39 OS=Homo sapiens GN=GPR39 PE=1 SV=1 | 405 | QSSARRtEKIF | 0.192 | -0.016 | -0.797 | -0.207 |
| sp\|O43194\|GPR39_HUMAN G-protein coupled receptor 39 OS=Homo sapiens GN=GPR39 PE=1 SV=1 | 411 | TEKIFLsTFQS | 0.1 | -0.166 | -1.202 | -0.423 |
| sp\|O43194\|GPR39_HUMAN G-protein coupled receptor 39 OS=Homo sapiens GN=GPR39 PE=1 SV=1 | 412 | EKIFLStFQSE | 0.136 | -0.035 | -0.915 | -0.271 |
| sp\|O43194\|GPR39_HUMAN G-protein coupled receptor 39 OS=Homo sapiens GN=GPR39 PE=1 SV=1 | 415 | FLSTFQsEAEP | 0.269 | 0.06 | -0.747 | -0.139 |
| sp\|O43194\|GPR39_HUMAN G-protein coupled receptor 39 OS=Homo sapiens GN=GPR39 PE=1 SV=1 | 421 | SEAEPQsKSQS | 0.043 | -0.338 | -1.5 | -0.598 |
| sp\|O43194\|GPR39_HUMAN G-protein coupled receptor 39 OS=Homo sapiens GN=GPR39 PE=1 SV=1 | 423 | AEPQSKsQSLS | 0.199 | 0.003 | -0.87 | -0.223 |
| sp\|O43194\|GPR39_HUMAN G-protein coupled receptor 39 OS=Homo sapiens GN=GPR39 PE=1 SV=1 | 425 | PQSKSQsLSLE | 0.54 | 0.245 | 0.172 | 0.319 |
| sp\|O43194\|GPR39_HUMAN G-protein coupled receptor 39 OS=Homo sapiens GN=GPR39 PE=1 SV=1 | 427 | SKSQSLsLESL | 0.341 | 0.216 | -0.155 | 0.134 |
| sp\|O43194\|GPR39_HUMAN G-protein coupled receptor 39 OS=Homo sapiens GN=GPR39 PE=1 SV=1 | 430 | QSLSLEsLEPN | 0.108 | -0.226 | -1.146 | -0.421 |
| sp\|O43194\|GPR39_HUMAN G-protein coupled receptor 39 OS=Homo sapiens GN=GPR39 PE=1 SV=1 | 435 | ESLEPNsGAKP | 0.039 | -0.375 | -1.547 | -0.628 |
| sp\|O43194\|GPR39_HUMAN G-protein coupled receptor 39 OS=Homo sapiens GN=GPR39 PE=1 SV=1 | 442 | GAKPANsAAEN | 0.469 | 0.092 | -0.603 | -0.014 |
| sp\|Q9Y5Y3\|GPR45_HUMAN Probable G-protein coupled receptor 45 OS=Homo sapiens GN=GPR45 PE=2 SV= | 290 | SVYSLLsVFSQ | 0.027 | -0.319 | -1.851 | -0.714 |
| sp\|Q9Y5Y3\|GPR45_HUMAN Probable G-protein coupled receptor 45 OS=Homo sapiens GN=GPR45 PE=2 SV= | 293 | SLLSVFsQRFY | 0.168 | -0.173 | -1.208 | -0.404 |
| sp\|Q9Y5Y3\|GPR45_HUMAN Probable G-protein coupled receptor 45 OS=Homo sapiens GN=GPR45 PE=2 SV= | 300 | QRFYCGsSFYA | 0.217 | 0.081 | -0.709 | -0.137 |
| sp\|Q9Y5Y3\|GPR45_HUMAN Probable G-protein coupled receptor 45 OS=Homo sapiens GN=GPR45 PE=2 SV= | 301 | RFYCGSsFYAT | 0.166 | -0.012 | -0.639 | -0.162 |
| sp\|Q9Y5Y3\|GPR45_HUMAN Probable G-protein coupled receptor 45 OS=Homo sapiens GN=GPR45 PE=2 SV= | 305 | GSSFYAtSTCV | 0.337 | -0.015 | -1.008 | -0.229 |
| sp\|Q9Y5Y3\|GPR45_HUMAN Probable G-protein coupled receptor 45 OS=Homo sapiens GN=GPR45 PE=2 SV= | 306 | SSFYATsTCVL | 0.146 | -0.073 | -0.941 | -0.289 |
| sp\|Q9Y5Y3\|GPR45_HUMAN Probable G-protein coupled receptor 45 OS=Homo sapiens GN=GPR45 PE=2 SV= | 307 | SFYATStCVLW | 0.058 | -0.038 | -1.292 | -0.424 |
| sp\|Q9Y5Y3\|GPR45_HUMAN Probable G-protein coupled receptor 45 OS=Homo sapiens GN=GPR45 PE=2 SV= | 313 | TCVLWLsYLKS | 0.058 | -0.158 | -1.62 | -0.573 |
| sp\|Q9Y5Y3\|GPR45_HUMAN Probable G-protein coupled receptor 45 OS=Homo sapiens GN=GPR45 PE=2 SV= | 317 | WLSYLKsVFNP | 0.261 | 0.056 | -0.425 | -0.036 |
| sp\|Q9Y5Y3\|GPR45_HUMAN Probable G-protein coupled receptor 45 OS=Homo sapiens GN=GPR45 PE=2 SV= | 342 | IELLPQtFQIL | 0.319 | -0.011 | -0.403 | -0.032 |
| sp\|Q9Y5Y3\|GPR45_HUMAN Probable G-protein coupled receptor 45 OS=Homo sapiens GN=GPR45 PE=2 SV= | 360 | RRRIQPsTVYV | 0.281 | 0.363 | -0.912 | -0.089 |
| sp\|Q9Y5Y3\|GPR45_HUMAN Probable G-protein coupled receptor 45 OS=Homo sapiens GN=GPR45 PE=2 SV= | 361 | RRIQPStVYVC | 0.131 | 0.085 | -0.952 | -0.245 |
| sp\|Q9Y5Y3\|GPR45_HUMAN Probable G-protein coupled receptor 45 OS=Homo sapiens GN=GPR45 PE=2 SV= | 370 | VCNENQsAV-- | 0.148 | 0.117 | -1.148 | -0.294 |
| sp\|Q13585\|MTR1L_HUMAN Melatonin-related receptor OS=Homo sapiens GN=GPR50 PE=1 SV=3 | 307 | FRREYWtIFHA | 0.349 | 0.357 | -0.2 | 0.169 |
| sp\|Q13585\|MTR1L_HUMAN Melatonin-related receptor OS=Homo sapiens GN=GPR50 PE=1 SV=3 | 320 | HPIIFFsGLIS | 0.079 | -0.071 | -1.27 | -0.421 |
| sp\|Q13585\|MTR1L_HUMAN Melatonin-related receptor OS=Homo sapiens GN=GPR50 PE=1 SV=3 | 324 | FFSGLIsDIRE | 0.174 | -0.093 | -0.917 | -0.279 |
| sp\|Q13585\|MTR1L_HUMAN Melatonin-related receptor OS=Homo sapiens GN=GPR50 PE=1 SV=3 | 334 | EMQEARtLARA | 0.156 | 0.089 | -0.918 | -0.224 |
| sp\|Q13585\|MTR1L_HUMAN Melatonin-related receptor OS=Homo sapiens GN=GPR50 PE=1 SV=3 | 361 | CPAVEEtPMNV | 0.065 | -0.405 | -1.899 | -0.746 |
| sp\|Q13585\|MTR1L_HUMAN Melatonin-related receptor OS=Homo sapiens GN=GPR50 PE=1 SV=3 | 383 | GHPDRAsGHPK | 0.268 | -0.008 | -1.073 | -0.271 |
| sp\|Q13585\|MTR1L_HUMAN Melatonin-related receptor OS=Homo sapiens GN=GPR50 PE=1 SV=3 | 390 | GHPKPHsRSSS | 0.091 | -0.186 | -1.581 | -0.559 |
| sp\|Q13585\|MTR1L_HUMAN Melatonin-related receptor OS=Homo sapiens GN=GPR50 PE=1 SV=3 | 392 | PKPHSRsSSAY | 0.343 | 0.193 | -0.472 | 0.021 |
| sp\|Q13585\|MTR1L_HUMAN Melatonin-related receptor OS=Homo sapiens GN=GPR50 PE=1 SV=3 | 393 | KPHSRSsSAYR | 0.067 | -0.253 | -1.562 | -0.583 |
| sp\|Q13585\|MTR1L_HUMAN Melatonin-related receptor OS=Homo sapiens GN=GPR50 PE=1 SV=3 | 394 | PHSRSSsAYRK | 0.656 | 0.899 | 0.034 | 0.53 |

| | | | | | | |
|---|---|---|---|---|---|---|
| sp\|Q13585\|MTR1L_HUMAN Melatonin-related receptor OS=Homo sapiens GN=GPR50 PE=1 SV=3 | 399 | SSAYRKsASTH | 0.173 | -0.077 | -0.887 | -0.264 |
| sp\|Q13585\|MTR1L_HUMAN Melatonin-related receptor OS=Homo sapiens GN=GPR50 PE=1 SV=3 | 401 | AYRKSAsTHHK | 0.791 | 0.433 | 0.315 | 0.513 |
| sp\|Q13585\|MTR1L_HUMAN Melatonin-related receptor OS=Homo sapiens GN=GPR50 PE=1 SV=3 | 402 | YRKSAStHHKS | 0.253 | 0.157 | -0.65 | -0.08 |
| sp\|Q13585\|MTR1L_HUMAN Melatonin-related receptor OS=Homo sapiens GN=GPR50 PE=1 SV=3 | 406 | ASTHHKsVFSH | 0.146 | -0.173 | -0.876 | -0.301 |
| sp\|Q13585\|MTR1L_HUMAN Melatonin-related receptor OS=Homo sapiens GN=GPR50 PE=1 SV=3 | 409 | HHKSVFsHSKA | 0.139 | -0.078 | -1.055 | -0.331 |
| sp\|Q13585\|MTR1L_HUMAN Melatonin-related receptor OS=Homo sapiens GN=GPR50 PE=1 SV=3 | 411 | KSVFSHsKAAS | 0.369 | 0.226 | -0.289 | 0.102 |
| sp\|Q13585\|MTR1L_HUMAN Melatonin-related receptor OS=Homo sapiens GN=GPR50 PE=1 SV=3 | 415 | SHSKAAsGHLK | 0.309 | -0.035 | -0.934 | -0.22 |
| sp\|Q13585\|MTR1L_HUMAN Melatonin-related receptor OS=Homo sapiens GN=GPR50 PE=1 SV=3 | 422 | GHLKPVsGHSK | 0.264 | -0.153 | -0.855 | -0.248 |
| sp\|Q13585\|MTR1L_HUMAN Melatonin-related receptor OS=Homo sapiens GN=GPR50 PE=1 SV=3 | 425 | KPVSGHsKPAS | 0.387 | 0.427 | -0.278 | 0.179 |
| sp\|Q13585\|MTR1L_HUMAN Melatonin-related receptor OS=Homo sapiens GN=GPR50 PE=1 SV=3 | 429 | GHSKPAsGHPK | 0.269 | -0.166 | -0.932 | -0.276 |
| sp\|Q13585\|MTR1L_HUMAN Melatonin-related receptor OS=Homo sapiens GN=GPR50 PE=1 SV=3 | 434 | ASGHPKsATVY | 0.223 | -0.111 | -0.858 | -0.249 |
| sp\|Q13585\|MTR1L_HUMAN Melatonin-related receptor OS=Homo sapiens GN=GPR50 PE=1 SV=3 | 436 | GHPKSAtVYPK | 0.449 | 0.167 | -0.501 | 0.038 |
| sp\|Q13585\|MTR1L_HUMAN Melatonin-related receptor OS=Homo sapiens GN=GPR50 PE=1 SV=3 | 443 | VYPKPAsVHFK | 0.234 | -0.163 | -0.829 | -0.253 |
| sp\|Q13585\|MTR1L_HUMAN Melatonin-related receptor OS=Homo sapiens GN=GPR50 PE=1 SV=3 | 450 | VHFKADsVHFK | 0.293 | -0.103 | -1.059 | -0.29 |
| sp\|Q13585\|MTR1L_HUMAN Melatonin-related receptor OS=Homo sapiens GN=GPR50 PE=1 SV=3 | 457 | VHFKGDsVHFK | 0.134 | -0.248 | -1.453 | -0.522 |
| sp\|Q13585\|MTR1L_HUMAN Melatonin-related receptor OS=Homo sapiens GN=GPR50 PE=1 SV=3 | 464 | VHFKPDsVHFK | 0.166 | -0.264 | -1.304 | -0.467 |
| sp\|Q13585\|MTR1L_HUMAN Melatonin-related receptor OS=Homo sapiens GN=GPR50 PE=1 SV=3 | 471 | VHFKPAsSNPK | 0.219 | -0.175 | -0.94 | -0.299 |
| sp\|Q13585\|MTR1L_HUMAN Melatonin-related receptor OS=Homo sapiens GN=GPR50 PE=1 SV=3 | 472 | HFKPASsNPKP | 0.402 | 0.405 | -0.115 | 0.231 |
| sp\|Q13585\|MTR1L_HUMAN Melatonin-related receptor OS=Homo sapiens GN=GPR50 PE=1 SV=3 | 478 | SNPKPItGHHV | 0.081 | -0.364 | -1.348 | -0.544 |
| sp\|Q13585\|MTR1L_HUMAN Melatonin-related receptor OS=Homo sapiens GN=GPR50 PE=1 SV=3 | 483 | ITGHHVsAGSH | 0.234 | -0.126 | -0.997 | -0.296 |
| sp\|Q13585\|MTR1L_HUMAN Melatonin-related receptor OS=Homo sapiens GN=GPR50 PE=1 SV=3 | 486 | HHVSAGsHSKS | 0.07 | -0.165 | -1.608 | -0.568 |
| sp\|Q13585\|MTR1L_HUMAN Melatonin-related receptor OS=Homo sapiens GN=GPR50 PE=1 SV=3 | 488 | VSAGSHsKSAF | 0.273 | 0.189 | -0.138 | 0.108 |
| sp\|Q13585\|MTR1L_HUMAN Melatonin-related receptor OS=Homo sapiens GN=GPR50 PE=1 SV=3 | 490 | AGSHSKsAFSA | 0.323 | 0.118 | -0.233 | 0.069 |
| sp\|Q13585\|MTR1L_HUMAN Melatonin-related receptor OS=Homo sapiens GN=GPR50 PE=1 SV=3 | 493 | HSKSAFsAATS | 0.201 | -0.01 | -0.715 | -0.175 |
| sp\|Q13585\|MTR1L_HUMAN Melatonin-related receptor OS=Homo sapiens GN=GPR50 PE=1 SV=3 | 496 | SAFSAAtSHPK | 0.156 | -0.202 | -1.216 | -0.421 |
| sp\|Q13585\|MTR1L_HUMAN Melatonin-related receptor OS=Homo sapiens GN=GPR50 PE=1 SV=3 | 497 | AFSAATsHPKP | 0.582 | 0.384 | 0.037 | 0.334 |
| sp\|Q13585\|MTR1L_HUMAN Melatonin-related receptor OS=Homo sapiens GN=GPR50 PE=1 SV=3 | 502 | TSHPKPtTGHI | 0.086 | -0.313 | -1.097 | -0.441 |
| sp\|Q13585\|MTR1L_HUMAN Melatonin-related receptor OS=Homo sapiens GN=GPR50 PE=1 SV=3 | 503 | SHPKPTtGHIK | 0.115 | -0.279 | -1.341 | -0.502 |
| sp\|Q13585\|MTR1L_HUMAN Melatonin-related receptor OS=Homo sapiens GN=GPR50 PE=1 SV=3 | 510 | GHIKPAtSHAE | 0.368 | 0.042 | -0.768 | -0.119 |
| sp\|Q13585\|MTR1L_HUMAN Melatonin-related receptor OS=Homo sapiens GN=GPR50 PE=1 SV=3 | 511 | HIKPATsHAEP | 0.301 | 0.072 | -0.376 | -0.001 |
| sp\|Q13585\|MTR1L_HUMAN Melatonin-related receptor OS=Homo sapiens GN=GPR50 PE=1 SV=3 | 516 | TSHAEPtTADY | 0.124 | -0.284 | -1.329 | -0.496 |
| sp\|Q13585\|MTR1L_HUMAN Melatonin-related receptor OS=Homo sapiens GN=GPR50 PE=1 SV=3 | 517 | SHAEPTtADYP | 0.057 | -0.237 | -1.657 | -0.612 |
| sp\|Q13585\|MTR1L_HUMAN Melatonin-related receptor OS=Homo sapiens GN=GPR50 PE=1 SV=3 | 525 | DYPKPAtTSHP | 0.13 | -0.26 | -1.186 | -0.439 |
| sp\|Q13585\|MTR1L_HUMAN Melatonin-related receptor OS=Homo sapiens GN=GPR50 PE=1 SV=3 | 526 | YPKPATtSHPK | 0.201 | -0.136 | -0.701 | -0.212 |
| sp\|Q13585\|MTR1L_HUMAN Melatonin-related receptor OS=Homo sapiens GN=GPR50 PE=1 SV=3 | 527 | PKPATTsHPKP | 0.471 | 0.422 | -0.103 | 0.263 |
| sp\|Q13585\|MTR1L_HUMAN Melatonin-related receptor OS=Homo sapiens GN=GPR50 PE=1 SV=3 | 532 | TSHPKPtAADN | 0.08 | -0.278 | -1.447 | -0.548 |
| sp\|Q13585\|MTR1L_HUMAN Melatonin-related receptor OS=Homo sapiens GN=GPR50 PE=1 SV=3 | 540 | ADNPELsASHC | 0.022 | -0.487 | -2.231 | -0.899 |
| sp\|Q13585\|MTR1L_HUMAN Melatonin-related receptor OS=Homo sapiens GN=GPR50 PE=1 SV=3 | 542 | NPELSAsHCPE | 0.302 | -0.014 | -0.657 | -0.123 |
| sp\|Q13585\|MTR1L_HUMAN Melatonin-related receptor OS=Homo sapiens GN=GPR50 PE=1 SV=3 | 555 | AIAHPVsDDSD | 0.308 | -0.038 | -0.464 | -0.065 |
| sp\|Q13585\|MTR1L_HUMAN Melatonin-related receptor OS=Homo sapiens GN=GPR50 PE=1 SV=3 | 558 | HPVSDDsDLPE | 0.073 | -0.291 | -1.632 | -0.617 |
| sp\|Q13585\|MTR1L_HUMAN Melatonin-related receptor OS=Homo sapiens GN=GPR50 PE=1 SV=3 | 563 | DSDLPEsASSP | 0.04 | -0.416 | -2.02 | -0.799 |
| sp\|Q13585\|MTR1L_HUMAN Melatonin-related receptor OS=Homo sapiens GN=GPR50 PE=1 SV=3 | 565 | DLPESAsSPAA | 0.781 | 0.73 | 0.444 | 0.652 |
| sp\|Q13585\|MTR1L_HUMAN Melatonin-related receptor OS=Homo sapiens GN=GPR50 PE=1 SV=3 | 566 | LPESASsPAAG | 0.07 | -0.427 | -1.279 | -0.545 |
| sp\|Q13585\|MTR1L_HUMAN Melatonin-related receptor OS=Homo sapiens GN=GPR50 PE=1 SV=3 | 572 | SPAAGPtKPAA | 0.314 | 0.237 | -0.247 | 0.101 |
| sp\|Q13585\|MTR1L_HUMAN Melatonin-related receptor OS=Homo sapiens GN=GPR50 PE=1 SV=3 | 577 | PTKPAAsQLES | 0.309 | -0.031 | -0.888 | -0.203 |
| sp\|Q13585\|MTR1L_HUMAN Melatonin-related receptor OS=Homo sapiens GN=GPR50 PE=1 SV=3 | 581 | AASQLEsDTIA | 0.207 | -0.251 | -1.216 | -0.42 |
| sp\|Q13585\|MTR1L_HUMAN Melatonin-related receptor OS=Homo sapiens GN=GPR50 PE=1 SV=3 | 583 | SQLESDtIADL | 0.137 | 0.001 | -0.811 | -0.224 |
| sp\|Q13585\|MTR1L_HUMAN Melatonin-related receptor OS=Homo sapiens GN=GPR50 PE=1 SV=3 | 591 | ADLPDPtVVTT | 0.055 | -0.389 | -1.362 | -0.565 |
| sp\|Q13585\|MTR1L_HUMAN Melatonin-related receptor OS=Homo sapiens GN=GPR50 PE=1 SV=3 | 594 | PDPTVVtTSTN | 0.104 | -0.276 | -1.367 | -0.513 |

| | | | | | | |
|---|---|---|---|---|---|---|
| sp\|Q13585\|MTR1L_HUMAN Melatonin-related receptor OS=Homo sapiens GN=GPR50 PE=1 SV=3 | 595 | DPTVVTtSTND | 0.197 | -0.101 | -1.143 | -0.349 |
| sp\|Q13585\|MTR1L_HUMAN Melatonin-related receptor OS=Homo sapiens GN=GPR50 PE=1 SV=3 | 596 | PTVVTTsTNDY | 0.247 | 0.077 | -0.675 | -0.117 |
| sp\|Q13585\|MTR1L_HUMAN Melatonin-related receptor OS=Homo sapiens GN=GPR50 PE=1 SV=3 | 597 | TVVTTStNDYH | 0.039 | -0.286 | -1.826 | -0.691 |
| sp\|Q9Y2T5\|GPR52_HUMAN G-protein coupled receptor 52 OS=Homo sapiens GN=GPR52 PE=2 SV=2 | 322 | IYSLSNsVFRL | 0.597 | 0.242 | -0.1 | 0.246 |
| sp\|Q9Y2T5\|GPR52_HUMAN G-protein coupled receptor 52 OS=Homo sapiens GN=GPR52 PE=2 SV=2 | 332 | LGLRRLsETMC | 0.615 | 0.849 | 0.234 | 0.566 |
| sp\|Q9Y2T5\|GPR52_HUMAN G-protein coupled receptor 52 OS=Homo sapiens GN=GPR52 PE=2 SV=2 | 334 | LRRLSEtMCTS | 0.437 | 0.558 | -0.356 | 0.213 |
| sp\|Q9Y2T5\|GPR52_HUMAN G-protein coupled receptor 52 OS=Homo sapiens GN=GPR52 PE=2 SV=2 | 337 | LSETMCtSCMC | 0.089 | -0.457 | -1.635 | -0.668 |
| sp\|Q9Y2T5\|GPR52_HUMAN G-protein coupled receptor 52 OS=Homo sapiens GN=GPR52 PE=2 SV=2 | 338 | SETMCTsCMCV | 0.243 | 0.007 | -1.098 | -0.283 |
| sp\|Q9Y2T5\|GPR52_HUMAN G-protein coupled receptor 52 OS=Homo sapiens GN=GPR52 PE=2 SV=2 | 358 | PRKRANsCSI- | 0.88 | 1.299 | 0.792 | 0.99 |
| sp\|Q9Y2T5\|GPR52_HUMAN G-protein coupled receptor 52 OS=Homo sapiens GN=GPR52 PE=2 SV=2 | 360 | KRANSCsI--- | 0.405 | 0.492 | -0.136 | 0.254 |
| sp\|Q9BZJ8\|GPR61_HUMAN Probable G-protein coupled receptor 61 OS=Homo sapiens GN=GPR61 PE=1 SV=: | 335 | IGYFCFtSNPF | 0.227 | -0.057 | -0.426 | -0.085 |
| sp\|Q9BZJ8\|GPR61_HUMAN Probable G-protein coupled receptor 61 OS=Homo sapiens GN=GPR61 PE=1 SV=: | 336 | GYFCFTsNPFF | 0.544 | 0.403 | -0.379 | 0.189 |
| sp\|Q9BZJ8\|GPR61_HUMAN Probable G-protein coupled receptor 61 OS=Homo sapiens GN=GPR61 PE=1 SV=: | 353 | QIRGELsKQFV | 0.197 | 0.115 | -0.892 | -0.193 |
| sp\|Q9BZJ8\|GPR61_HUMAN Probable G-protein coupled receptor 61 OS=Homo sapiens GN=GPR61 PE=1 SV=: | 372 | EELRLPsREGS | 0.238 | 0.524 | -0.489 | 0.091 |
| sp\|Q9BZJ8\|GPR61_HUMAN Probable G-protein coupled receptor 61 OS=Homo sapiens GN=GPR61 PE=1 SV=: | 376 | LPSREGsIEEN | 0.339 | 0.626 | -0.361 | 0.201 |
| sp\|Q9BZJ8\|GPR61_HUMAN Probable G-protein coupled receptor 61 OS=Homo sapiens GN=GPR61 PE=1 SV=: | 388 | LQFLQGtGCPS | 0.033 | -0.376 | -1.741 | -0.695 |
| sp\|Q9BZJ8\|GPR61_HUMAN Probable G-protein coupled receptor 61 OS=Homo sapiens GN=GPR61 PE=1 SV=: | 392 | QGTGCPsESWV | 0.165 | -0.072 | -0.808 | -0.238 |
| sp\|Q9BZJ8\|GPR61_HUMAN Probable G-protein coupled receptor 61 OS=Homo sapiens GN=GPR61 PE=1 SV=: | 394 | TGCPSEsWVSR | 0.145 | 0.082 | -0.788 | -0.187 |
| sp\|Q9BZJ8\|GPR61_HUMAN Probable G-protein coupled receptor 61 OS=Homo sapiens GN=GPR61 PE=1 SV=: | 397 | PSESWVsRPLP | 0.547 | 0.269 | -0.122 | 0.231 |
| sp\|Q9BZJ8\|GPR61_HUMAN Probable G-protein coupled receptor 61 OS=Homo sapiens GN=GPR61 PE=1 SV=: | 402 | VSRPLPsPKQE | 0.081 | -0.428 | -1.286 | -0.544 |
| sp\|Q9BZJ8\|GPR61_HUMAN Probable G-protein coupled receptor 61 OS=Homo sapiens GN=GPR61 PE=1 SV=: | 422 | GQIAEEtSEFL | 0.08 | -0.196 | -1.46 | -0.525 |
| sp\|Q9BZJ8\|GPR61_HUMAN Probable G-protein coupled receptor 61 OS=Homo sapiens GN=GPR61 PE=1 SV=: | 423 | QIAEETsEFLE | 0.154 | 0.025 | -0.793 | -0.205 |
| sp\|Q9BZJ8\|GPR61_HUMAN Probable G-protein coupled receptor 61 OS=Homo sapiens GN=GPR61 PE=1 SV=: | 431 | FLEQQLtSDII | 0.364 | 0.131 | -0.324 | 0.057 |
| sp\|Q9BZJ8\|GPR61_HUMAN Probable G-protein coupled receptor 61 OS=Homo sapiens GN=GPR61 PE=1 SV=: | 432 | LEQQLTsDIIM | 0.192 | -0.037 | -0.761 | -0.202 |
| sp\|Q9BZJ8\|GPR61_HUMAN Probable G-protein coupled receptor 61 OS=Homo sapiens GN=GPR61 PE=1 SV=: | 437 | TSDIIMsDSYL | 0.128 | -0.036 | -1.017 | -0.308 |
| sp\|Q9BZJ8\|GPR61_HUMAN Probable G-protein coupled receptor 61 OS=Homo sapiens GN=GPR61 PE=1 SV=: | 439 | DIIMSDsYLRP | 0.181 | -0.07 | -1.169 | -0.353 |
| sp\|Q9BZJ8\|GPR61_HUMAN Probable G-protein coupled receptor 61 OS=Homo sapiens GN=GPR61 PE=1 SV=: | 446 | YLRPAAsPRLE | 0.438 | 0.005 | -0.354 | 0.03 |
| sp\|Q9BZJ8\|GPR61_HUMAN Probable G-protein coupled receptor 61 OS=Homo sapiens GN=GPR61 PE=1 SV=: | 451 | ASPRLEs---- | 0.346 | 0.724 | -0.5 | 0.19 |
| sp\|Q9BZJ7\|GPR62_HUMAN Probable G-protein coupled receptor 62 OS=Homo sapiens GN=GPR62 PE=2 SV=: | 342 | GPAVGPsEAPE | 0.063 | -0.27 | -1.348 | -0.518 |
| sp\|Q9BZJ7\|GPR62_HUMAN Probable G-protein coupled receptor 62 OS=Homo sapiens GN=GPR62 PE=2 SV=: | 348 | SEAPEQtPELA | 0.039 | -0.515 | -1.719 | -0.732 |
| sp\|Q9BZJ7\|GPR62_HUMAN Probable G-protein coupled receptor 62 OS=Homo sapiens GN=GPR62 PE=2 SV=: | 356 | ELAGGRsPAYQ | 0.028 | -0.475 | -1.734 | -0.727 |
| sp\|Q9BZJ7\|GPR62_HUMAN Probable G-protein coupled receptor 62 OS=Homo sapiens GN=GPR62 PE=2 SV=: | 365 | YQGPPEsSLS- | 0.023 | -0.348 | -1.744 | -0.69 |
| sp\|Q9BZJ7\|GPR62_HUMAN Probable G-protein coupled receptor 62 OS=Homo sapiens GN=GPR62 PE=2 SV=: | 366 | QGPPESsLS-- | 0.067 | -0.138 | -1.247 | -0.439 |
| sp\|Q9BZJ7\|GPR62_HUMAN Probable G-protein coupled receptor 62 OS=Homo sapiens GN=GPR62 PE=2 SV=: | 368 | PPESSLs---- | 0.231 | 0.184 | -0.62 | -0.068 |
| sp\|Q9BZJ6\|GPR63_HUMAN Probable G-protein coupled receptor 63 OS=Homo sapiens GN=GPR63 PE=2 SV=: | 334 | APFTTYsLVAT | 0.214 | 0.109 | -0.485 | -0.054 |
| sp\|Q9BZJ6\|GPR63_HUMAN Probable G-protein coupled receptor 63 OS=Homo sapiens GN=GPR63 PE=2 SV=: | 338 | TYSLVAtFSKH | 0.159 | -0.16 | -1.249 | -0.417 |
| sp\|Q9BZJ6\|GPR63_HUMAN Probable G-protein coupled receptor 63 OS=Homo sapiens GN=GPR63 PE=2 SV=: | 340 | SLVATFsKHFY | 0.173 | 0.018 | -0.897 | -0.235 |
| sp\|Q9BZJ6\|GPR63_HUMAN Probable G-protein coupled receptor 63 OS=Homo sapiens GN=GPR63 PE=2 SV=: | 353 | HNFFEIsTWLL | 0.248 | 0.028 | -0.725 | -0.15 |
| sp\|Q9BZJ6\|GPR63_HUMAN Probable G-protein coupled receptor 63 OS=Homo sapiens GN=GPR63 PE=2 SV=: | 354 | NFFEIStWLLW | 0.067 | 0.002 | -1.099 | -0.343 |
| sp\|Q9BZJ6\|GPR63_HUMAN Probable G-protein coupled receptor 63 OS=Homo sapiens GN=GPR63 PE=2 SV=: | 364 | WLCYLKsALNP | 0.229 | 0.077 | -0.778 | -0.157 |
| sp\|Q9BZJ6\|GPR63_HUMAN Probable G-protein coupled receptor 63 OS=Homo sapiens GN=GPR63 PE=2 SV=: | 389 | LDMMPKsFKFL | 0.115 | -0.141 | -0.574 | -0.2 |
| sp\|Q9BZJ6\|GPR63_HUMAN Probable G-protein coupled receptor 63 OS=Homo sapiens GN=GPR63 PE=2 SV=: | 400 | PQLPGHtKRRI | 0.23 | -0.106 | -0.533 | -0.136 |
| sp\|Q9BZJ6\|GPR63_HUMAN Probable G-protein coupled receptor 63 OS=Homo sapiens GN=GPR63 PE=2 SV=: | 407 | KRRIRPsAVYV | 0.521 | 0.514 | -0.56 | 0.158 |
| sp\|Q9BZJ6\|GPR63_HUMAN Probable G-protein coupled receptor 63 OS=Homo sapiens GN=GPR63 PE=2 SV=: | 417 | VCGEHRtVV-- | 0.109 | 0.11 | -1.359 | -0.38 |
| sp\|Q15743\|OGR1_HUMAN Ovarian cancer G-protein coupled receptor 1 OS=Homo sapiens GN=GPR68 PE=1 | 342 | EEPELLtKLHP | 0.014 | -0.417 | -2.107 | -0.837 |
| sp\|Q15743\|OGR1_HUMAN Ovarian cancer G-protein coupled receptor 1 OS=Homo sapiens GN=GPR68 PE=1 | 350 | LHPAFQtPNSP | 0.025 | -0.533 | -1.954 | -0.821 |
| sp\|Q15743\|OGR1_HUMAN Ovarian cancer G-protein coupled receptor 1 OS=Homo sapiens GN=GPR68 PE=1 | 353 | AFQTPNsPGSG | 0.119 | -0.329 | -1.137 | -0.449 |
| sp\|Q15743\|OGR1_HUMAN Ovarian cancer G-protein coupled receptor 1 OS=Homo sapiens GN=GPR68 PE=1 | 356 | TPNSPGsGGFP | 0.033 | -0.495 | -1.8 | -0.754 |
| sp\|Q15743\|OGR1_HUMAN Ovarian cancer G-protein coupled receptor 1 OS=Homo sapiens GN=GPR68 PE=1 | 361 | GSGGFPtGRLA | 0.111 | -0.288 | -1.128 | -0.435 |

| | | | | | | |
|---|---|---|---|---|---|---|
| sp\|O95800\|GPR75_HUMAN Probable G-protein coupled receptor 75 OS=Homo sapiens GN=GPR75 PE=1 SV= | 380 | FIYSRNsAGLR | 0.572 | 0.381 | -0.195 | 0.253 |
| sp\|O95800\|GPR75_HUMAN Probable G-protein coupled receptor 75 OS=Homo sapiens GN=GPR75 PE=1 SV= | 405 | FCCKQKtRLRA | 0.337 | -0.003 | -0.849 | -0.172 |
| sp\|O95800\|GPR75_HUMAN Probable G-protein coupled receptor 75 OS=Homo sapiens GN=GPR75 PE=1 SV= | 422 | EVNRNKsSHHE | 0.481 | 0.763 | 0.272 | 0.505 |
| sp\|O95800\|GPR75_HUMAN Probable G-protein coupled receptor 75 OS=Homo sapiens GN=GPR75 PE=1 SV= | 423 | VNRNKSsHHET | 0.159 | -0.04 | -0.744 | -0.208 |
| sp\|O95800\|GPR75_HUMAN Probable G-protein coupled receptor 75 OS=Homo sapiens GN=GPR75 PE=1 SV= | 427 | KSSHHEtNSAY | 0.146 | -0.181 | -1.274 | -0.436 |
| sp\|O95800\|GPR75_HUMAN Probable G-protein coupled receptor 75 OS=Homo sapiens GN=GPR75 PE=1 SV= | 429 | SHHETNsAYML | 0.064 | -0.065 | -1.905 | -0.635 |
| sp\|O95800\|GPR75_HUMAN Probable G-protein coupled receptor 75 OS=Homo sapiens GN=GPR75 PE=1 SV= | 434 | NSAYMLsPKPQ | 0.039 | -0.583 | -1.408 | -0.651 |
| sp\|O95800\|GPR75_HUMAN Probable G-protein coupled receptor 75 OS=Homo sapiens GN=GPR75 PE=1 SV= | 449 | DQACGPsHSKE | 0.086 | -0.325 | -1.353 | -0.531 |
| sp\|O95800\|GPR75_HUMAN Probable G-protein coupled receptor 75 OS=Homo sapiens GN=GPR75 PE=1 SV= | 451 | ACGPSHsKESM | 0.243 | 0.17 | -0.598 | -0.062 |
| sp\|O95800\|GPR75_HUMAN Probable G-protein coupled receptor 75 OS=Homo sapiens GN=GPR75 PE=1 SV= | 454 | PSHSKEsMVSP | 0.026 | -0.287 | -1.949 | -0.737 |
| sp\|O95800\|GPR75_HUMAN Probable G-protein coupled receptor 75 OS=Homo sapiens GN=GPR75 PE=1 SV= | 457 | SKESMVsPKIS | 0.145 | -0.484 | -1.159 | -0.499 |
| sp\|O95800\|GPR75_HUMAN Probable G-protein coupled receptor 75 OS=Homo sapiens GN=GPR75 PE=1 SV= | 461 | MVSPKIsAGHQ | 0.137 | -0.108 | -0.928 | -0.3 |
| sp\|O95800\|GPR75_HUMAN Probable G-protein coupled receptor 75 OS=Homo sapiens GN=GPR75 PE=1 SV= | 470 | HQHCGQsSSTP | 0.031 | -0.292 | -1.542 | -0.601 |
| sp\|O95800\|GPR75_HUMAN Probable G-protein coupled receptor 75 OS=Homo sapiens GN=GPR75 PE=1 SV= | 471 | QHCGQSsSTPI | 0.086 | -0.202 | -1.497 | -0.538 |
| sp\|O95800\|GPR75_HUMAN Probable G-protein coupled receptor 75 OS=Homo sapiens GN=GPR75 PE=1 SV= | 472 | HCGQSSsTPIN | 0.687 | 0.705 | 0.333 | 0.575 |
| sp\|O95800\|GPR75_HUMAN Probable G-protein coupled receptor 75 OS=Homo sapiens GN=GPR75 PE=1 SV= | 473 | CGQSSStPINT | 0.097 | -0.174 | -0.895 | -0.324 |
| sp\|O95800\|GPR75_HUMAN Probable G-protein coupled receptor 75 OS=Homo sapiens GN=GPR75 PE=1 SV= | 477 | SSTPINtRIEP | 0.082 | -0.285 | -1.189 | -0.464 |
| sp\|O95800\|GPR75_HUMAN Probable G-protein coupled receptor 75 OS=Homo sapiens GN=GPR75 PE=1 SV= | 484 | RIEPYYsIYNS | 0.185 | 0.087 | -0.704 | -0.144 |
| sp\|O95800\|GPR75_HUMAN Probable G-protein coupled receptor 75 OS=Homo sapiens GN=GPR75 PE=1 SV= | 488 | YYSIYNsSPSQ | 0.445 | 0.43 | -0.333 | 0.181 |
| sp\|O95800\|GPR75_HUMAN Probable G-protein coupled receptor 75 OS=Homo sapiens GN=GPR75 PE=1 SV= | 489 | YSIYNSsPSQE | 0.042 | -0.521 | -1.448 | -0.642 |
| sp\|O95800\|GPR75_HUMAN Probable G-protein coupled receptor 75 OS=Homo sapiens GN=GPR75 PE=1 SV= | 491 | IYNSSPsQEES | 0.283 | 0.016 | -0.816 | -0.172 |
| sp\|O95800\|GPR75_HUMAN Probable G-protein coupled receptor 75 OS=Homo sapiens GN=GPR75 PE=1 SV= | 495 | SPSQEEsSPCN | 0.306 | 0.273 | -0.835 | -0.085 |
| sp\|O95800\|GPR75_HUMAN Probable G-protein coupled receptor 75 OS=Homo sapiens GN=GPR75 PE=1 SV= | 496 | PSQEESsPCNL | 0.033 | -0.459 | -1.95 | -0.792 |
| sp\|O95800\|GPR75_HUMAN Probable G-protein coupled receptor 75 OS=Homo sapiens GN=GPR75 PE=1 SV= | 505 | NLQPVNsFGFA | 0.501 | 0.141 | -0.223 | 0.14 |
| sp\|O95800\|GPR75_HUMAN Probable G-protein coupled receptor 75 OS=Homo sapiens GN=GPR75 PE=1 SV= | 511 | SFGFANsYIAM | 0.224 | 0.069 | -0.546 | -0.084 |
| sp\|O95800\|GPR75_HUMAN Probable G-protein coupled receptor 75 OS=Homo sapiens GN=GPR75 PE=1 SV= | 519 | IAMHYHtTNDL | 0.457 | 0.188 | -0.129 | 0.172 |
| sp\|O95800\|GPR75_HUMAN Probable G-protein coupled receptor 75 OS=Homo sapiens GN=GPR75 PE=1 SV= | 520 | AMHYHTtNDLV | 0.169 | 0.096 | -1.097 | -0.277 |
| sp\|O95800\|GPR75_HUMAN Probable G-protein coupled receptor 75 OS=Homo sapiens GN=GPR75 PE=1 SV= | 529 | LVQEYDsTSAK | 0.085 | -0.136 | -1.189 | -0.413 |
| sp\|O95800\|GPR75_HUMAN Probable G-protein coupled receptor 75 OS=Homo sapiens GN=GPR75 PE=1 SV= | 530 | VQEYDStSAKQ | 0.05 | -0.356 | -1.449 | -0.585 |
| sp\|O95800\|GPR75_HUMAN Probable G-protein coupled receptor 75 OS=Homo sapiens GN=GPR75 PE=1 SV= | 531 | QEYDSTsAKQI | 0.36 | 0.199 | -0.314 | 0.082 |
| sp\|O95800\|GPR75_HUMAN Probable G-protein coupled receptor 75 OS=Homo sapiens GN=GPR75 PE=1 SV= | 539 | KQIPVPsV--- | 0.112 | -0.021 | -1.024 | -0.311 |
| sp\|Q96P69\|GPR78_HUMAN G-protein coupled receptor 78 OS=Homo sapiens GN=GPR78 PE=1 SV=2 | 316 | HRLLKRtPRPA | 0.053 | -0.256 | -1.347 | -0.517 |
| sp\|Q96P69\|GPR78_HUMAN G-protein coupled receptor 78 OS=Homo sapiens GN=GPR78 PE=1 SV=2 | 321 | RTPRPAsTHDS | 0.675 | 0.792 | 0.138 | 0.535 |
| sp\|Q96P69\|GPR78_HUMAN G-protein coupled receptor 78 OS=Homo sapiens GN=GPR78 PE=1 SV=2 | 322 | TPRPAStHDSS | 0.196 | 0.069 | -0.765 | -0.167 |
| sp\|Q96P69\|GPR78_HUMAN G-protein coupled receptor 78 OS=Homo sapiens GN=GPR78 PE=1 SV=2 | 325 | PASTHDsSLDV | 0.188 | -0.151 | -1.371 | -0.445 |
| sp\|Q96P69\|GPR78_HUMAN G-protein coupled receptor 78 OS=Homo sapiens GN=GPR78 PE=1 SV=2 | 326 | ASTHDSsLDVA | 0.22 | -0.116 | -0.578 | -0.158 |
| sp\|Q96P69\|GPR78_HUMAN G-protein coupled receptor 78 OS=Homo sapiens GN=GPR78 PE=1 SV=2 | 340 | HQLLKRtPRPA | 0.033 | -0.498 | -1.503 | -0.656 |
| sp\|Q96P69\|GPR78_HUMAN G-protein coupled receptor 78 OS=Homo sapiens GN=GPR78 PE=1 SV=2 | 345 | RTPRPAsTHNG | 0.66 | 0.803 | -0.011 | 0.484 |
| sp\|Q96P69\|GPR78_HUMAN G-protein coupled receptor 78 OS=Homo sapiens GN=GPR78 PE=1 SV=2 | 346 | TPRPAStHNGS | 0.3 | 0.061 | -0.48 | -0.04 |
| sp\|Q96P69\|GPR78_HUMAN G-protein coupled receptor 78 OS=Homo sapiens GN=GPR78 PE=1 SV=2 | 350 | ASTHNGsVDTE | 0.156 | -0.241 | -0.902 | -0.329 |
| sp\|Q96P69\|GPR78_HUMAN G-protein coupled receptor 78 OS=Homo sapiens GN=GPR78 PE=1 SV=2 | 353 | HNGSVDtENDS | 0.149 | -0.091 | -0.9 | -0.281 |
| sp\|Q96P69\|GPR78_HUMAN G-protein coupled receptor 78 OS=Homo sapiens GN=GPR78 PE=1 SV=2 | 357 | VDTENDsCLQQ | 0.059 | -0.29 | -1.507 | -0.579 |
| sp\|Q96P69\|GPR78_HUMAN G-protein coupled receptor 78 OS=Homo sapiens GN=GPR78 PE=1 SV=2 | 362 | DSCLQQtH--- | 0.209 | 0.058 | -1.069 | -0.267 |
| sp\|Q96P67\|GPR82_HUMAN Probable G-protein coupled receptor 82 OS=Homo sapiens GN=GPR82 PE=2 SV= | 315 | FLLLDKtFKKT | 0.368 | 0.141 | -0.189 | 0.107 |
| sp\|Q96P67\|GPR82_HUMAN Probable G-protein coupled receptor 82 OS=Homo sapiens GN=GPR82 PE=2 SV= | 319 | DKTFKKtLYNL | 0.633 | 0.268 | 0.072 | 0.324 |
| sp\|Q96P67\|GPR82_HUMAN Probable G-protein coupled receptor 82 OS=Homo sapiens GN=GPR82 PE=2 SV= | 325 | TLYNLFtKSNS | 0.083 | -0.042 | -1.072 | -0.344 |
| sp\|Q96P67\|GPR82_HUMAN Probable G-protein coupled receptor 82 OS=Homo sapiens GN=GPR82 PE=2 SV= | 327 | YNLFTKsNSAH | 0.091 | -0.15 | -1.015 | -0.358 |
| sp\|Q96P67\|GPR82_HUMAN Probable G-protein coupled receptor 82 OS=Homo sapiens GN=GPR82 PE=2 SV= | 329 | LFTKSNsAHMQ | 0.626 | 0.273 | -0.047 | 0.284 |
| sp\|Q96P67\|GPR82_HUMAN Probable G-protein coupled receptor 82 OS=Homo sapiens GN=GPR82 PE=2 SV= | 334 | NSAHMQsYG-- | 0.182 | -0.031 | -0.626 | -0.158 |

| | | | | | | |
|---|---|---|---|---|---|---|
| sp\|Q9NYM4\|GPR83_HUMAN Probable G-protein coupled receptor 83 OS=Homo sapiens GN=GPR83 PE=2 SV | 361 | ELKALLsMCQR | 0.148 | -0.046 | -0.888 | -0.262 |
| sp\|Q9NYM4\|GPR83_HUMAN Probable G-protein coupled receptor 83 OS=Homo sapiens GN=GPR83 PE=2 SV | 376 | QEDRPPsPVPS | 0.126 | 0.243 | -0.819 | -0.15 |
| sp\|Q9NYM4\|GPR83_HUMAN Probable G-protein coupled receptor 83 OS=Homo sapiens GN=GPR83 PE=2 SV | 380 | PPSPVPsFRVA | 0.214 | -0.187 | -0.761 | -0.245 |
| sp\|Q9NYM4\|GPR83_HUMAN Probable G-protein coupled receptor 83 OS=Homo sapiens GN=GPR83 PE=2 SV | 386 | SFRVAWtEKND | 0.476 | 0.315 | -0.289 | 0.167 |
| sp\|Q9NYM4\|GPR83_HUMAN Probable G-protein coupled receptor 83 OS=Homo sapiens GN=GPR83 PE=2 SV | 403 | ANNLLPtSQLQ | 0.064 | -0.348 | -1.663 | -0.649 |
| sp\|Q9NYM4\|GPR83_HUMAN Probable G-protein coupled receptor 83 OS=Homo sapiens GN=GPR83 PE=2 SV | 404 | NNLLPTsQLQS | 0.063 | -0.34 | -1.385 | -0.554 |
| sp\|Q9NYM4\|GPR83_HUMAN Probable G-protein coupled receptor 83 OS=Homo sapiens GN=GPR83 PE=2 SV | 408 | PTSQLQsGKTD | 0.179 | -0.17 | -0.755 | -0.249 |
| sp\|Q9NYM4\|GPR83_HUMAN Probable G-protein coupled receptor 83 OS=Homo sapiens GN=GPR83 PE=2 SV | 411 | QLQSGKtDLSS | 0.11 | -0.033 | -1.052 | -0.325 |
| sp\|Q9NYM4\|GPR83_HUMAN Probable G-protein coupled receptor 83 OS=Homo sapiens GN=GPR83 PE=2 SV | 414 | SGKTDLsSVEP | 0.063 | -0.272 | -1.466 | -0.558 |
| sp\|Q9NYM4\|GPR83_HUMAN Probable G-protein coupled receptor 83 OS=Homo sapiens GN=GPR83 PE=2 SV | 415 | GKTDLSsVEPI | 0.261 | -0.008 | -0.684 | -0.144 |
| sp\|Q9NYM4\|GPR83_HUMAN Probable G-protein coupled receptor 83 OS=Homo sapiens GN=GPR83 PE=2 SV | 421 | SVEPIVtMS-- | 0.101 | 0.019 | -1.058 | -0.313 |
| sp\|Q9NYM4\|GPR83_HUMAN Probable G-protein coupled receptor 83 OS=Homo sapiens GN=GPR83 PE=2 SV | 423 | EPIVTMs---- | 0.092 | 0.207 | -1.037 | -0.246 |
| sp\|Q9NQS5\|GPR84_HUMAN G-protein coupled receptor 84 OS=Homo sapiens GN=GPR84 PE=2 SV=1 | 383 | FRQAYGsILKR | 0.14 | 0.134 | -0.929 | -0.218 |
| sp\|Q9NQS5\|GPR84_HUMAN G-protein coupled receptor 84 OS=Homo sapiens GN=GPR84 PE=2 SV=1 | 391 | LKRGPRsFHRL | 0.512 | 0.245 | -0.155 | 0.201 |
| sp\|P60893\|GPR85_HUMAN Probable G-protein coupled receptor 85 OS=Homo sapiens GN=GPR85 PE=1 SV=1 | 351 | ELRRCFsTTLL | 0.898 | 1.277 | 1.177 | 1.117 |
| sp\|P60893\|GPR85_HUMAN Probable G-protein coupled receptor 85 OS=Homo sapiens GN=GPR85 PE=1 SV=1 | 352 | LRRCFStTLLY | 0.331 | 0.339 | -0.703 | -0.011 |
| sp\|P60893\|GPR85_HUMAN Probable G-protein coupled receptor 85 OS=Homo sapiens GN=GPR85 PE=1 SV=1 | 353 | RRCFSTtLLYC | 0.551 | 0.446 | -0.176 | 0.274 |
| sp\|P60893\|GPR85_HUMAN Probable G-protein coupled receptor 85 OS=Homo sapiens GN=GPR85 PE=1 SV=1 | 360 | LLYCRKsRLPR | 0.384 | 0.098 | -0.338 | 0.048 |
| sp\|Q9BY21\|GPR87_HUMAN G-protein coupled receptor 87 OS=Homo sapiens GN=GPR87 PE=2 SV=1 | 320 | YFFMCRsFSRR | 0.279 | -0.068 | -0.682 | -0.157 |
| sp\|Q9BY21\|GPR87_HUMAN G-protein coupled receptor 87 OS=Homo sapiens GN=GPR87 PE=2 SV=1 | 322 | FMCRSFsRRLF | 0.733 | 1.151 | 0.662 | 0.849 |
| sp\|Q9BY21\|GPR87_HUMAN G-protein coupled receptor 87 OS=Homo sapiens GN=GPR87 PE=2 SV=1 | 329 | RRLFKKsNIRT | 0.211 | 0.096 | -0.696 | -0.13 |
| sp\|Q9BY21\|GPR87_HUMAN G-protein coupled receptor 87 OS=Homo sapiens GN=GPR87 PE=2 SV=1 | 333 | KKSNIRtRSES | 0.091 | -0.202 | -1.17 | -0.427 |
| sp\|Q9BY21\|GPR87_HUMAN G-protein coupled receptor 87 OS=Homo sapiens GN=GPR87 PE=2 SV=1 | 335 | SNIRTRsESIR | 0.267 | 0.754 | -0.407 | 0.205 |
| sp\|Q9BY21\|GPR87_HUMAN G-protein coupled receptor 87 OS=Homo sapiens GN=GPR87 PE=2 SV=1 | 337 | IRTRSEsIRSL | 0.727 | 1.135 | 0.783 | 0.882 |
| sp\|Q9BY21\|GPR87_HUMAN G-protein coupled receptor 87 OS=Homo sapiens GN=GPR87 PE=2 SV=1 | 340 | RSESIRsLQSV | 0.171 | -0.081 | -0.919 | -0.276 |
| sp\|Q9BY21\|GPR87_HUMAN G-protein coupled receptor 87 OS=Homo sapiens GN=GPR87 PE=2 SV=1 | 343 | SIRSLQsVRRS | 0.189 | -0.007 | -1.044 | -0.287 |
| sp\|Q9BY21\|GPR87_HUMAN G-protein coupled receptor 87 OS=Homo sapiens GN=GPR87 PE=2 SV=1 | 347 | LQSVRRsEVRI | 0.192 | 0.077 | -0.723 | -0.151 |
| sp\|Q9BY21\|GPR87_HUMAN G-protein coupled receptor 87 OS=Homo sapiens GN=GPR87 PE=2 SV=1 | 356 | RIYYDYtDV-- | 0.247 | 0.32 | -0.515 | 0.017 |
| sp\|Q9GZN0\|GPR88_HUMAN Probable G-protein coupled receptor 88 OS=Homo sapiens GN=GPR88 PE=2 SV= | 346 | NEEFRRsVRSV | 0.208 | 0.003 | -1.109 | -0.299 |
| sp\|Q9GZN0\|GPR88_HUMAN Probable G-protein coupled receptor 88 OS=Homo sapiens GN=GPR88 PE=2 SV= | 349 | FRRSVRsVLPG | 0.294 | 0.373 | -0.678 | -0.004 |
| sp\|Q9GZN0\|GPR88_HUMAN Probable G-protein coupled receptor 88 OS=Homo sapiens GN=GPR88 PE=2 SV= | 365 | AAAVAAtAVPA | 0.181 | -0.081 | -0.944 | -0.281 |
| sp\|Q9GZN0\|GPR88_HUMAN Probable G-protein coupled receptor 88 OS=Homo sapiens GN=GPR88 PE=2 SV= | 371 | TAVPAVsQAQL | 0.198 | -0.181 | -0.96 | -0.314 |
| sp\|Q9GZN0\|GPR88_HUMAN Probable G-protein coupled receptor 88 OS=Homo sapiens GN=GPR88 PE=2 SV= | 377 | SQAQLGtRAAG | 0.067 | -0.305 | -1.296 | -0.511 |
| sp\|Q96P66\|GP101_HUMAN Probable G-protein coupled receptor 101 OS=Homo sapiens GN=GPR101 PE=1 S | 309 | SEEVREsSTVA | 0.134 | -0.111 | -1.339 | -0.439 |
| sp\|Q96P66\|GP101_HUMAN Probable G-protein coupled receptor 101 OS=Homo sapiens GN=GPR101 PE=1 S | 310 | EEVRESsTVAS | 0.288 | 0.716 | -0.397 | 0.202 |
| sp\|Q96P66\|GP101_HUMAN Probable G-protein coupled receptor 101 OS=Homo sapiens GN=GPR101 PE=1 S | 311 | EVRESStVASD | 0.162 | 0.269 | -0.542 | -0.037 |
| sp\|Q96P66\|GP101_HUMAN Probable G-protein coupled receptor 101 OS=Homo sapiens GN=GPR101 PE=1 S | 314 | ESSTVAsDGSM | 0.18 | -0.113 | -0.995 | -0.309 |
| sp\|Q96P66\|GP101_HUMAN Probable G-protein coupled receptor 101 OS=Homo sapiens GN=GPR101 PE=1 S | 317 | TVASDGsMEGK | 0.066 | -0.244 | -1.344 | -0.507 |
| sp\|Q96P66\|GP101_HUMAN Probable G-protein coupled receptor 101 OS=Homo sapiens GN=GPR101 PE=1 S | 324 | MEGKEGsTKVE | 0.139 | -0.114 | -1.262 | -0.412 |
| sp\|Q96P66\|GP101_HUMAN Probable G-protein coupled receptor 101 OS=Homo sapiens GN=GPR101 PE=1 S | 325 | EGKEGStKVEE | 0.057 | -0.221 | -1.207 | -0.457 |
| sp\|Q96P66\|GP101_HUMAN Probable G-protein coupled receptor 101 OS=Homo sapiens GN=GPR101 PE=1 S | 331 | TKVEENsMKAD | 0.304 | 0.177 | -0.64 | -0.053 |
| sp\|Q96P66\|GP101_HUMAN Probable G-protein coupled receptor 101 OS=Homo sapiens GN=GPR101 PE=1 S | 339 | KADKGRtEVNQ | 0.102 | -0.109 | -1.469 | -0.492 |
| sp\|Q96P66\|GP101_HUMAN Probable G-protein coupled receptor 101 OS=Homo sapiens GN=GPR101 PE=1 S | 345 | TEVNQCsIDLG | 0.118 | -0.08 | -1.131 | -0.364 |
| sp\|Q96P66\|GP101_HUMAN Probable G-protein coupled receptor 101 OS=Homo sapiens GN=GPR101 PE=1 S | 363 | EDDINFsEDDV | 0.226 | 0.061 | -0.697 | -0.137 |
| sp\|Q96P66\|GP101_HUMAN Probable G-protein coupled receptor 101 OS=Homo sapiens GN=GPR101 PE=1 S | 375 | AVNIPEsLPPS | 0.363 | 0.309 | -0.387 | 0.095 |
| sp\|Q96P66\|GP101_HUMAN Probable G-protein coupled receptor 101 OS=Homo sapiens GN=GPR101 PE=1 S | 379 | PESLPPsRRNS | 0.09 | -0.348 | -1.512 | -0.59 |
| sp\|Q96P66\|GP101_HUMAN Probable G-protein coupled receptor 101 OS=Homo sapiens GN=GPR101 PE=1 S | 383 | PPSRRNsNSNP | 0.612 | 0.871 | -0.139 | 0.448 |
| sp\|Q96P66\|GP101_HUMAN Probable G-protein coupled receptor 101 OS=Homo sapiens GN=GPR101 PE=1 S | 385 | SRRNSNsNPPL | 0.84 | 1.086 | 0.7 | 0.875 |
| sp\|Q96P66\|GP101_HUMAN Probable G-protein coupled receptor 101 OS=Homo sapiens GN=GPR101 PE=1 S | 407 | IFIIIFsYVLS | 0.135 | 0.008 | -0.666 | -0.174 |

| | | | | | | |
|---|---|---|---|---|---|---|
| sp\|Q96P66\|GP101_HUMAN Probable G-protein coupled receptor 101 OS=Homo sapiens GN=GPR101 PE=1 S | 411 | IFSYVLsLGPY | 0.472 | 0.067 | -0.058 | 0.16 |
| sp\|Q96P66\|GP101_HUMAN Probable G-protein coupled receptor 101 OS=Homo sapiens GN=GPR101 PE=1 S | 429 | VWVDVEtQVPQ | 0.081 | -0.248 | -1.751 | -0.639 |
| sp\|Q96P66\|GP101_HUMAN Probable G-protein coupled receptor 101 OS=Homo sapiens GN=GPR101 PE=1 S | 437 | VPQWVItIIIW | 0.171 | -0.126 | -0.776 | -0.244 |
| sp\|Q96P66\|GP101_HUMAN Probable G-protein coupled receptor 101 OS=Homo sapiens GN=GPR101 PE=1 S | 460 | YGYMHKtIKKE | 0.153 | -0.111 | -0.588 | -0.182 |
| sp\|Q96P66\|GP101_HUMAN Probable G-protein coupled receptor 101 OS=Homo sapiens GN=GPR101 PE=1 S | 483 | KPPKEDsHPDL | 0.654 | 0.376 | -0.237 | 0.264 |
| sp\|Q96P66\|GP101_HUMAN Probable G-protein coupled receptor 101 OS=Homo sapiens GN=GPR101 PE=1 S | 490 | HPDLPGtEGGT | 0.096 | -0.185 | -0.994 | -0.361 |
| sp\|Q96P66\|GP101_HUMAN Probable G-protein coupled receptor 101 OS=Homo sapiens GN=GPR101 PE=1 S | 494 | PGTEGGtEGKI | 0.127 | -0.04 | -0.789 | -0.234 |
| sp\|Q96P66\|GP101_HUMAN Probable G-protein coupled receptor 101 OS=Homo sapiens GN=GPR101 PE=1 S | 501 | EGKIVPsYDSA | 0.098 | -0.168 | -0.904 | -0.325 |
| sp\|Q96P66\|GP101_HUMAN Probable G-protein coupled receptor 101 OS=Homo sapiens GN=GPR101 PE=1 S | 504 | IVPSYDsATFP | 0.082 | -0.34 | -1.618 | -0.625 |
| sp\|Q96P66\|GP101_HUMAN Probable G-protein coupled receptor 101 OS=Homo sapiens GN=GPR101 PE=1 S | 506 | PSYDSAtFP-- | 0.836 | 0.892 | 0.668 | 0.799 |
| sp\|Q9UNW8\|GP132_HUMAN Probable G-protein coupled receptor 132 OS=Homo sapiens GN=GPR132 PE=1 | 312 | IIYVLAtDHSR | 0.154 | 0.011 | -1.013 | -0.283 |
| sp\|Q9UNW8\|GP132_HUMAN Probable G-protein coupled receptor 132 OS=Homo sapiens GN=GPR132 PE=1 | 315 | VLATDHsRQEV | 0.376 | 0.044 | -0.714 | -0.098 |
| sp\|Q9UNW8\|GP132_HUMAN Probable G-protein coupled receptor 132 OS=Homo sapiens GN=GPR132 PE=1 | 320 | HSRQEVsRIHK | 0.319 | -0.01 | -0.371 | -0.021 |
| sp\|Q9UNW8\|GP132_HUMAN Probable G-protein coupled receptor 132 OS=Homo sapiens GN=GPR132 PE=1 | 330 | KGWKEWsMKTD | 0.269 | 0.087 | -0.701 | -0.115 |
| sp\|Q9UNW8\|GP132_HUMAN Probable G-protein coupled receptor 132 OS=Homo sapiens GN=GPR132 PE=1 | 333 | KEWSMKtDVTR | 0.2 | -0.016 | -0.842 | -0.219 |
| sp\|Q9UNW8\|GP132_HUMAN Probable G-protein coupled receptor 132 OS=Homo sapiens GN=GPR132 PE=1 | 336 | SMKTDVtRLTH | 0.083 | -0.27 | -1.599 | -0.595 |
| sp\|Q9UNW8\|GP132_HUMAN Probable G-protein coupled receptor 132 OS=Homo sapiens GN=GPR132 PE=1 | 339 | TDVTRLtHSRD | 0.113 | -0.181 | -1.252 | -0.44 |
| sp\|Q9UNW8\|GP132_HUMAN Probable G-protein coupled receptor 132 OS=Homo sapiens GN=GPR132 PE=1 | 341 | VTRLTHsRDTE | 0.38 | 0.204 | -0.585 | 0 |
| sp\|Q9UNW8\|GP132_HUMAN Probable G-protein coupled receptor 132 OS=Homo sapiens GN=GPR132 PE=1 | 344 | LTHSRDtEELQ | 0.16 | -0.061 | -1.142 | -0.348 |
| sp\|Q9UNW8\|GP132_HUMAN Probable G-protein coupled receptor 132 OS=Homo sapiens GN=GPR132 PE=1 | 349 | DTEELQsPVAL | 0.078 | -0.324 | -1.412 | -0.553 |
| sp\|Q9UNW8\|GP132_HUMAN Probable G-protein coupled receptor 132 OS=Homo sapiens GN=GPR132 PE=1 | 358 | ALADHYtFSRP | 0.289 | 0.039 | -0.646 | -0.106 |
| sp\|Q9UNW8\|GP132_HUMAN Probable G-protein coupled receptor 132 OS=Homo sapiens GN=GPR132 PE=1 | 360 | ADHYTFsRPVH | 0.154 | 0.223 | -0.857 | -0.16 |
| sp\|Q9UNW8\|GP132_HUMAN Probable G-protein coupled receptor 132 OS=Homo sapiens GN=GPR132 PE=1 | 368 | PVHPPGsPCPA | 0.018 | -0.739 | -1.96 | -0.894 |
| sp\|Q9UNW8\|GP132_HUMAN Probable G-protein coupled receptor 132 OS=Homo sapiens GN=GPR132 PE=1 | 379 | KRLIEEsC--- | 0.196 | 0.305 | -1.035 | -0.178 |
| sp\|Q8IZ08\|GP135_HUMAN Probable G-protein coupled receptor 135 OS=Homo sapiens GN=GPR135 PE=2 SV | 316 | RVRPVNtYARV | 0.316 | 0.071 | -1.013 | -0.209 |
| sp\|Q8IZ08\|GP135_HUMAN Probable G-protein coupled receptor 135 OS=Homo sapiens GN=GPR135 PE=2 SV | 325 | RVLRFFsEVRT | 0.586 | 0.862 | 0.178 | 0.542 |
| sp\|Q8IZ08\|GP135_HUMAN Probable G-protein coupled receptor 135 OS=Homo sapiens GN=GPR135 PE=2 SV | 329 | FFSEVRtATTV | 0.18 | -0.005 | -1.311 | -0.379 |
| sp\|Q8IZ08\|GP135_HUMAN Probable G-protein coupled receptor 135 OS=Homo sapiens GN=GPR135 PE=2 SV | 331 | SEVRTAtTVLI | 0.476 | 0.878 | -0.06 | 0.431 |
| sp\|Q8IZ08\|GP135_HUMAN Probable G-protein coupled receptor 135 OS=Homo sapiens GN=GPR135 PE=2 SV | 332 | EVRTATtVLIM | 0.349 | 0.228 | -0.37 | 0.069 |
| sp\|Q8IZ08\|GP135_HUMAN Probable G-protein coupled receptor 135 OS=Homo sapiens GN=GPR135 PE=2 SV | 361 | AARQAQtMQAP | 0.52 | 0.351 | -0.084 | 0.262 |
| sp\|Q8IZ08\|GP135_HUMAN Probable G-protein coupled receptor 135 OS=Homo sapiens GN=GPR135 PE=2 SV | 366 | QTMQAPsLLSV | 0.232 | 0.003 | -0.77 | -0.178 |
| sp\|Q8IZ08\|GP135_HUMAN Probable G-protein coupled receptor 135 OS=Homo sapiens GN=GPR135 PE=2 SV | 369 | QAPSLLsVVAV | 0.052 | -0.229 | -1.619 | -0.599 |
| sp\|Q8IZ08\|GP135_HUMAN Probable G-protein coupled receptor 135 OS=Homo sapiens GN=GPR135 PE=2 SV | 376 | VVAVWLtWANG | 0.071 | -0.027 | -1.196 | -0.384 |
| sp\|Q8IZ08\|GP135_HUMAN Probable G-protein coupled receptor 135 OS=Homo sapiens GN=GPR135 PE=2 SV | 395 | IRNPNIsMLLG | 0.248 | 0.206 | -0.68 | -0.075 |
| sp\|Q8IZ08\|GP135_HUMAN Probable G-protein coupled receptor 135 OS=Homo sapiens GN=GPR135 PE=2 SV | 408 | REEGYRtRNVD | 0.215 | -0.041 | -0.898 | -0.241 |
| sp\|Q8IZ08\|GP135_HUMAN Probable G-protein coupled receptor 135 OS=Homo sapiens GN=GPR135 PE=2 SV | 417 | VDAFLPsQGPG | 0.089 | -0.309 | -1.312 | -0.511 |
| sp\|Q8IZ08\|GP135_HUMAN Probable G-protein coupled receptor 135 OS=Homo sapiens GN=GPR135 PE=2 SV | 426 | PGLQARsRSRL | 0.165 | -0.151 | -0.983 | -0.323 |
| sp\|Q8IZ08\|GP135_HUMAN Probable G-protein coupled receptor 135 OS=Homo sapiens GN=GPR135 PE=2 SV | 428 | LQARSRsRLRN | 0.512 | 0.851 | 0.121 | 0.495 |
| sp\|Q8IZ08\|GP135_HUMAN Probable G-protein coupled receptor 135 OS=Homo sapiens GN=GPR135 PE=2 SV | 445 | GACNRMsSSNP | 0.218 | 0.119 | -0.89 | -0.184 |
| sp\|Q8IZ08\|GP135_HUMAN Probable G-protein coupled receptor 135 OS=Homo sapiens GN=GPR135 PE=2 SV | 446 | ACNRMSsSNPA | 0.47 | 0.62 | -0.129 | 0.32 |
| sp\|Q8IZ08\|GP135_HUMAN Probable G-protein coupled receptor 135 OS=Homo sapiens GN=GPR135 PE=2 SV | 447 | CNRMSSsNPAS | 0.789 | 0.775 | 0.66 | 0.741 |
| sp\|Q8IZ08\|GP135_HUMAN Probable G-protein coupled receptor 135 OS=Homo sapiens GN=GPR135 PE=2 SV | 451 | SSSNPAsGVAG | 0.093 | -0.223 | -0.976 | -0.369 |
| sp\|Q8IZ08\|GP135_HUMAN Probable G-protein coupled receptor 135 OS=Homo sapiens GN=GPR135 PE=2 SV | 479 | GPPEPVtAVTK | 0.115 | -0.231 | -1.269 | -0.462 |
| sp\|Q8IZ08\|GP135_HUMAN Probable G-protein coupled receptor 135 OS=Homo sapiens GN=GPR135 PE=2 SV | 482 | EPVTAVtKQPK | 0.249 | -0.13 | -0.67 | -0.184 |
| sp\|Q8IZ08\|GP135_HUMAN Probable G-protein coupled receptor 135 OS=Homo sapiens GN=GPR135 PE=2 SV | 487 | VTKQPKsEAGD | 0.39 | -0.068 | -0.09 | 0.077 |
| sp\|Q8IZ08\|GP135_HUMAN Probable G-protein coupled receptor 135 OS=Homo sapiens GN=GPR135 PE=2 SV | 492 | KSEAGDtSL-- | 0.059 | -0.274 | -1.663 | -0.626 |
| sp\|Q8IZ08\|GP135_HUMAN Probable G-protein coupled receptor 135 OS=Homo sapiens GN=GPR135 PE=2 SV | 493 | SEAGDTsL--- | 0.185 | 0.084 | -0.717 | -0.149 |
| sp\|Q6DWJ6\|GP139_HUMAN Probable G-protein coupled receptor 139 OS=Homo sapiens GN=GPR139 PE=2 S | 315 | QPVQFYtNHNF | 0.135 | -0.038 | -1.113 | -0.339 |
| sp\|Q6DWJ6\|GP139_HUMAN Probable G-protein coupled receptor 139 OS=Homo sapiens GN=GPR139 PE=2 S | 320 | YTNHNFsITSS | 0.098 | -0.181 | -1.111 | -0.398 |

| | | | | | | |
|---|---|---|---|---|---|---|
| sp\|Q6DWJ6\|GP139_HUMAN Probable G-protein coupled receptor 139 OS=Homo sapiens GN=GPR139 PE=2 S | 322 | NHNFSItSSPW | 0.169 | 0.036 | -1.083 | -0.293 |
| sp\|Q6DWJ6\|GP139_HUMAN Probable G-protein coupled receptor 139 OS=Homo sapiens GN=GPR139 PE=2 S | 323 | HNFSITsSPWI | 0.453 | 0.432 | 0.255 | 0.38 |
| sp\|Q6DWJ6\|GP139_HUMAN Probable G-protein coupled receptor 139 OS=Homo sapiens GN=GPR139 PE=2 S | 324 | NFSITSsPWIS | 0.066 | -0.335 | -1.412 | -0.56 |
| sp\|Q6DWJ6\|GP139_HUMAN Probable G-protein coupled receptor 139 OS=Homo sapiens GN=GPR139 PE=2 S | 328 | TSSPWIsPANS | 0.05 | -0.51 | -1.625 | -0.695 |
| sp\|Q6DWJ6\|GP139_HUMAN Probable G-protein coupled receptor 139 OS=Homo sapiens GN=GPR139 PE=2 S | 332 | WISPANsHCIK | 0.345 | -0.086 | -0.612 | -0.118 |
| sp\|Q6DWJ6\|GP139_HUMAN Probable G-protein coupled receptor 139 OS=Homo sapiens GN=GPR139 PE=2 S | 352 | GKPIKVsP--- | 0.171 | -0.147 | -1.05 | -0.342 |
| sp\|Q7Z601\|GP142_HUMAN Probable G-protein coupled receptor 142 OS=Homo sapiens GN=GPR142 PE=2 S' | 308 | DTDSPRtLDEV | 0.31 | -0.102 | -1.011 | -0.268 |
| sp\|Q7Z601\|GP142_HUMAN Probable G-protein coupled receptor 142 OS=Homo sapiens GN=GPR142 PE=2 S' | 320 | KWAHCLtVYFI | 0.171 | -0.065 | -0.696 | -0.197 |
| sp\|Q7Z601\|GP142_HUMAN Probable G-protein coupled receptor 142 OS=Homo sapiens GN=GPR142 PE=2 S' | 332 | CGVFLVtNSAI | 0.297 | -0.008 | -0.535 | -0.082 |
| sp\|Q7Z601\|GP142_HUMAN Probable G-protein coupled receptor 142 OS=Homo sapiens GN=GPR142 PE=2 S' | 334 | VFLVTNsAIIH | 0.11 | -0.079 | -1.137 | -0.369 |
| sp\|Q7Z601\|GP142_HUMAN Probable G-protein coupled receptor 142 OS=Homo sapiens GN=GPR142 PE=2 S' | 346 | LRRRGRsGLQP | 0.451 | 1.054 | 0.114 | 0.54 |
| sp\|Q7Z601\|GP142_HUMAN Probable G-protein coupled receptor 142 OS=Homo sapiens GN=GPR142 PE=2 S' | 355 | QPRVGKsTAIL | 0.187 | 0.084 | -0.692 | -0.14 |
| sp\|Q7Z601\|GP142_HUMAN Probable G-protein coupled receptor 142 OS=Homo sapiens GN=GPR142 PE=2 S' | 356 | PRVGKStAILL | 0.092 | 0.179 | -0.917 | -0.215 |
| sp\|Q7Z601\|GP142_HUMAN Probable G-protein coupled receptor 142 OS=Homo sapiens GN=GPR142 PE=2 S' | 363 | AILLGItTLFT | 0.062 | -0.244 | -1.444 | -0.542 |
| sp\|Q7Z601\|GP142_HUMAN Probable G-protein coupled receptor 142 OS=Homo sapiens GN=GPR142 PE=2 S' | 364 | ILLGITtLFTL | 0.526 | 0.18 | 0.344 | 0.35 |
| sp\|Q7Z601\|GP142_HUMAN Probable G-protein coupled receptor 142 OS=Homo sapiens GN=GPR142 PE=2 S' | 367 | GITTLFtLLWA | 0.317 | 0.129 | -0.384 | 0.021 |
| sp\|Q7Z601\|GP142_HUMAN Probable G-protein coupled receptor 142 OS=Homo sapiens GN=GPR142 PE=2 S' | 407 | MVAMLHtAANF | 0.174 | 0.093 | -0.941 | -0.225 |
| sp\|Q7Z601\|GP142_HUMAN Probable G-protein coupled receptor 142 OS=Homo sapiens GN=GPR142 PE=2 S' | 418 | GLYCFVsKTFR | 0.497 | -0.003 | -0.5 | -0.002 |
| sp\|Q7Z601\|GP142_HUMAN Probable G-protein coupled receptor 142 OS=Homo sapiens GN=GPR142 PE=2 S' | 420 | YCFVSKtFRAT | 0.578 | 0.334 | 0.289 | 0.4 |
| sp\|Q7Z601\|GP142_HUMAN Probable G-protein coupled receptor 142 OS=Homo sapiens GN=GPR142 PE=2 S' | 424 | SKTFRAtVRQV | 0.569 | 0.143 | -0.33 | 0.127 |
| sp\|Q7Z601\|GP142_HUMAN Probable G-protein coupled receptor 142 OS=Homo sapiens GN=GPR142 PE=2 S' | 437 | DAYLPCtLASQ | 0.139 | -0.224 | -1.234 | -0.44 |
| sp\|Q7Z601\|GP142_HUMAN Probable G-protein coupled receptor 142 OS=Homo sapiens GN=GPR142 PE=2 S' | 440 | LPCTLAsQPEG | 0.516 | 0.359 | -0.507 | 0.123 |
| sp\|Q7Z601\|GP142_HUMAN Probable G-protein coupled receptor 142 OS=Homo sapiens GN=GPR142 PE=2 S' | 458 | EPPGLPtGAEV | 0.039 | -0.356 | -1.782 | -0.7 |
| sp\|Q96CH1\|GP146_HUMAN Probable G-protein coupled receptor 146 OS=Homo sapiens GN=GPR146 PE=2 S | 322 | CGDRHCsPDHM | 0.568 | 0.574 | 0.176 | 0.439 |
| sp\|Q8TDV2\|GP148_HUMAN Probable G-protein coupled receptor 148 OS=Homo sapiens GN=GPR148 PE=2 S | 317 | LPRAMLtYLYL | 0.125 | -0.044 | -1.003 | -0.307 |
| sp\|Q8TDV2\|GP148_HUMAN Probable G-protein coupled receptor 148 OS=Homo sapiens GN=GPR148 PE=2 S | 337 | VRGHLPsRRHQ | 0.061 | -0.261 | -1.444 | -0.548 |
| sp\|Q8TDV2\|GP148_HUMAN Probable G-protein coupled receptor 148 OS=Homo sapiens GN=GPR148 PE=2 S | 345 | RHQAIFtIS-- | 0.121 | 0.083 | -1.021 | -0.272 |
| sp\|Q8TDV2\|GP148_HUMAN Probable G-protein coupled receptor 148 OS=Homo sapiens GN=GPR148 PE=2 S | 347 | QAIFTIs---- | 0.145 | 0.111 | -0.946 | -0.23 |
| sp\|O00398\|P2Y10_HUMAN Putative P2Y purinoceptor 10 OS=Homo sapiens GN=P2RY10 PE=2 SV=1 | 330 | TRSRLMsKESG | 0.5 | 1.034 | 0.203 | 0.579 |
| sp\|O00398\|P2Y10_HUMAN Putative P2Y purinoceptor 10 OS=Homo sapiens GN=P2RY10 PE=2 SV=1 | 333 | RLMSKEsGSSM | 0.058 | -0.199 | -1.415 | -0.519 |
| sp\|O00398\|P2Y10_HUMAN Putative P2Y purinoceptor 10 OS=Homo sapiens GN=P2RY10 PE=2 SV=1 | 335 | MSKESGsSMIG | 0.294 | 0.106 | -0.579 | -0.06 |
| sp\|O00398\|P2Y10_HUMAN Putative P2Y purinoceptor 10 OS=Homo sapiens GN=P2RY10 PE=2 SV=1 | 336 | SKESGSsMIG- | 0.074 | -0.143 | -1.235 | -0.435 |
| sp\|Q86SP6\|GP149_HUMAN Probable G-protein coupled receptor 149 OS=Homo sapiens GN=GPR149 PE=2 S' | 318 | ALILALtKVVL | 0.253 | 0.142 | -0.672 | -0.092 |
| sp\|Q86SP6\|GP149_HUMAN Probable G-protein coupled receptor 149 OS=Homo sapiens GN=GPR149 PE=2 S' | 340 | NVVGFQsLPLE | 0.589 | 0.535 | 0.174 | 0.433 |
| sp\|Q86SP6\|GP149_HUMAN Probable G-protein coupled receptor 149 OS=Homo sapiens GN=GPR149 PE=2 S' | 345 | QSLPLEtFSFL | 0.058 | -0.293 | -1.403 | -0.546 |
| sp\|Q86SP6\|GP149_HUMAN Probable G-protein coupled receptor 149 OS=Homo sapiens GN=GPR149 PE=2 S' | 347 | LPLETFsFLLT | 0.069 | -0.026 | -1.001 | -0.319 |
| sp\|Q86SP6\|GP149_HUMAN Probable G-protein coupled receptor 149 OS=Homo sapiens GN=GPR149 PE=2 S' | 351 | TFSFLLtLLAT | 0.14 | -0.049 | -0.704 | -0.204 |
| sp\|Q86SP6\|GP149_HUMAN Probable G-protein coupled receptor 149 OS=Homo sapiens GN=GPR149 PE=2 S' | 355 | LLTLLAtTVTP | 0.152 | -0.061 | -0.962 | -0.29 |
| sp\|Q86SP6\|GP149_HUMAN Probable G-protein coupled receptor 149 OS=Homo sapiens GN=GPR149 PE=2 S' | 356 | LTLLATtVTPV | 0.288 | -0.047 | -0.82 | -0.193 |
| sp\|Q86SP6\|GP149_HUMAN Probable G-protein coupled receptor 149 OS=Homo sapiens GN=GPR149 PE=2 S' | 358 | LLATTVtPVFV | 0.104 | -0.295 | -1.241 | -0.477 |
| sp\|Q86SP6\|GP149_HUMAN Probable G-protein coupled receptor 149 OS=Homo sapiens GN=GPR149 PE=2 S' | 364 | TPVFVLsKRWT | 0.092 | -0.236 | -1.133 | -0.426 |
| sp\|Q86SP6\|GP149_HUMAN Probable G-protein coupled receptor 149 OS=Homo sapiens GN=GPR149 PE=2 S' | 368 | VLSKRWtHLPC | 0.427 | 0.017 | -0.574 | -0.043 |
| sp\|Q86SP6\|GP149_HUMAN Probable G-protein coupled receptor 149 OS=Homo sapiens GN=GPR149 PE=2 S' | 387 | NAYAVAsDGKK | 0.263 | -0.08 | -0.866 | -0.228 |
| sp\|Q86SP6\|GP149_HUMAN Probable G-protein coupled receptor 149 OS=Homo sapiens GN=GPR149 PE=2 S' | 402 | GFEFNLsFQKS | 0.296 | 0.033 | -0.443 | -0.038 |
| sp\|Q86SP6\|GP149_HUMAN Probable G-protein coupled receptor 149 OS=Homo sapiens GN=GPR149 PE=2 S' | 406 | NLSFQKsYGIY | 0.494 | 0.148 | -0.269 | 0.124 |
| sp\|Q86SP6\|GP149_HUMAN Probable G-protein coupled receptor 149 OS=Homo sapiens GN=GPR149 PE=2 S' | 424 | YDDDENsIFYH | 0.072 | -0.325 | -1.668 | -0.64 |
| sp\|Q86SP6\|GP149_HUMAN Probable G-protein coupled receptor 149 OS=Homo sapiens GN=GPR149 PE=2 S' | 433 | YHNLMNsECET | 0.168 | -0.112 | -1.258 | -0.401 |
| sp\|Q86SP6\|GP149_HUMAN Probable G-protein coupled receptor 149 OS=Homo sapiens GN=GPR149 PE=2 S' | 437 | MNSECEtTKDP | 0.138 | -0.156 | -1.043 | -0.354 |
| sp\|Q86SP6\|GP149_HUMAN Probable G-protein coupled receptor 149 OS=Homo sapiens GN=GPR149 PE=2 S' | 438 | NSECETtKDPQ | 0.198 | -0.229 | -0.926 | -0.319 |

| | | | | | | |
|---|---|---|---|---|---|---|
| sp\|Q86SP6\|GP149_HUMAN Probable G-protein coupled receptor 149 OS=Homo sapiens GN=GPR149 PE=2 S' | 457 | AIKVEIsTTPS | 0.16 | -0.147 | -1.132 | -0.373 |
| sp\|Q86SP6\|GP149_HUMAN Probable G-protein coupled receptor 149 OS=Homo sapiens GN=GPR149 PE=2 S' | 458 | IKVEIStTPSL | 0.335 | 0.417 | -0.15 | 0.201 |
| sp\|Q86SP6\|GP149_HUMAN Probable G-protein coupled receptor 149 OS=Homo sapiens GN=GPR149 PE=2 S' | 459 | KVEISTtPSLD | 0.114 | -0.159 | -0.871 | -0.305 |
| sp\|Q86SP6\|GP149_HUMAN Probable G-protein coupled receptor 149 OS=Homo sapiens GN=GPR149 PE=2 S' | 461 | EISTTPsLDSS | 0.124 | 0.006 | -0.798 | -0.223 |
| sp\|Q86SP6\|GP149_HUMAN Probable G-protein coupled receptor 149 OS=Homo sapiens GN=GPR149 PE=2 S' | 464 | TTPSLDsSTQR | 0.076 | -0.411 | -1.661 | -0.665 |
| sp\|Q86SP6\|GP149_HUMAN Probable G-protein coupled receptor 149 OS=Homo sapiens GN=GPR149 PE=2 S' | 465 | TPSLDSsTQRG | 0.079 | -0.326 | -1.51 | -0.586 |
| sp\|Q86SP6\|GP149_HUMAN Probable G-protein coupled receptor 149 OS=Homo sapiens GN=GPR149 PE=2 S' | 466 | PSLDSStQRGI | 0.33 | -0.016 | -0.48 | -0.055 |
| sp\|Q86SP6\|GP149_HUMAN Probable G-protein coupled receptor 149 OS=Homo sapiens GN=GPR149 PE=2 S' | 474 | RGINKCtNTDI | 0.35 | 0.03 | -0.639 | -0.086 |
| sp\|Q86SP6\|GP149_HUMAN Probable G-protein coupled receptor 149 OS=Homo sapiens GN=GPR149 PE=2 S' | 476 | INKCTNtDITE | 0.277 | -0.045 | -0.652 | -0.14 |
| sp\|Q86SP6\|GP149_HUMAN Probable G-protein coupled receptor 149 OS=Homo sapiens GN=GPR149 PE=2 S' | 479 | CTNTDItEAKQ | 0.155 | -0.159 | -1.168 | -0.391 |
| sp\|Q86SP6\|GP149_HUMAN Probable G-protein coupled receptor 149 OS=Homo sapiens GN=GPR149 PE=2 S' | 485 | TEAKQDsNNKK | 0.135 | -0.221 | -1.27 | -0.452 |
| sp\|Q86SP6\|GP149_HUMAN Probable G-protein coupled receptor 149 OS=Homo sapiens GN=GPR149 PE=2 S' | 493 | NKKDAFsDKTG | 0.605 | 0.206 | -0.084 | 0.242 |
| sp\|Q86SP6\|GP149_HUMAN Probable G-protein coupled receptor 149 OS=Homo sapiens GN=GPR149 PE=2 S' | 496 | DAFSDKtGGDI | 0.466 | 0.002 | -0.27 | 0.066 |
| sp\|Q86SP6\|GP149_HUMAN Probable G-protein coupled receptor 149 OS=Homo sapiens GN=GPR149 PE=2 S' | 505 | DINYEEtTFSE | 0.123 | -0.135 | -1.381 | -0.464 |
| sp\|Q86SP6\|GP149_HUMAN Probable G-protein coupled receptor 149 OS=Homo sapiens GN=GPR149 PE=2 S' | 506 | INYEETtFSEG | 0.125 | -0.159 | -0.974 | -0.336 |
| sp\|Q86SP6\|GP149_HUMAN Probable G-protein coupled receptor 149 OS=Homo sapiens GN=GPR149 PE=2 S' | 508 | YEETTFsEGPE | 0.18 | 0.015 | -0.735 | -0.18 |
| sp\|Q86SP6\|GP149_HUMAN Probable G-protein coupled receptor 149 OS=Homo sapiens GN=GPR149 PE=2 S' | 516 | GPERRLsHEES | 0.717 | 0.932 | 0.32 | 0.656 |
| sp\|Q86SP6\|GP149_HUMAN Probable G-protein coupled receptor 149 OS=Homo sapiens GN=GPR149 PE=2 S' | 520 | RLSHEEsQKPD | 0.202 | -0.149 | -1.109 | -0.352 |
| sp\|Q86SP6\|GP149_HUMAN Probable G-protein coupled receptor 149 OS=Homo sapiens GN=GPR149 PE=2 S' | 526 | SQKPDLsDWEW | 0.127 | -0.096 | -1.071 | -0.347 |
| sp\|Q86SP6\|GP149_HUMAN Probable G-protein coupled receptor 149 OS=Homo sapiens GN=GPR149 PE=2 S' | 533 | DWEWCRsKSER | 0.318 | -0.078 | -0.728 | -0.163 |
| sp\|Q86SP6\|GP149_HUMAN Probable G-protein coupled receptor 149 OS=Homo sapiens GN=GPR149 PE=2 S' | 535 | EWCRSKsERTP | 0.759 | 1.088 | 0.974 | 0.94 |
| sp\|Q86SP6\|GP149_HUMAN Probable G-protein coupled receptor 149 OS=Homo sapiens GN=GPR149 PE=2 S' | 538 | RSKSERtPRQR | 0.09 | -0.502 | -1.409 | -0.607 |
| sp\|Q86SP6\|GP149_HUMAN Probable G-protein coupled receptor 149 OS=Homo sapiens GN=GPR149 PE=2 S' | 543 | RTPRQRsGYAL | 0.404 | 0.841 | -0.166 | 0.36 |
| sp\|Q86SP6\|GP149_HUMAN Probable G-protein coupled receptor 149 OS=Homo sapiens GN=GPR149 PE=2 S' | 557 | LCAFQGtVSLH | 0.071 | -0.216 | -1.553 | -0.566 |
| sp\|Q86SP6\|GP149_HUMAN Probable G-protein coupled receptor 149 OS=Homo sapiens GN=GPR149 PE=2 S' | 559 | AFQGTVsLHAP | 0.451 | 0.173 | -0.068 | 0.185 |
| sp\|Q86SP6\|GP149_HUMAN Probable G-protein coupled receptor 149 OS=Homo sapiens GN=GPR149 PE=2 S' | 564 | VSLHAPtGKTL | 0.149 | -0.208 | -0.782 | -0.28 |
| sp\|Q86SP6\|GP149_HUMAN Probable G-protein coupled receptor 149 OS=Homo sapiens GN=GPR149 PE=2 S' | 567 | HAPTGKtLSLS | 0.115 | -0.088 | -0.878 | -0.284 |
| sp\|Q86SP6\|GP149_HUMAN Probable G-protein coupled receptor 149 OS=Homo sapiens GN=GPR149 PE=2 S' | 569 | PTGKTLsLSTY | 0.237 | 0.03 | -0.708 | -0.147 |
| sp\|Q86SP6\|GP149_HUMAN Probable G-protein coupled receptor 149 OS=Homo sapiens GN=GPR149 PE=2 S' | 571 | GKTLSLsTYEV | 0.503 | 0.281 | -0.33 | 0.151 |
| sp\|Q86SP6\|GP149_HUMAN Probable G-protein coupled receptor 149 OS=Homo sapiens GN=GPR149 PE=2 S' | 572 | KTLSLStYEVS | 0.042 | -0.33 | -1.757 | -0.682 |
| sp\|Q86SP6\|GP149_HUMAN Probable G-protein coupled receptor 149 OS=Homo sapiens GN=GPR149 PE=2 S' | 576 | LSTYEVsAEGQ | 0.215 | -0.158 | -0.813 | -0.252 |
| sp\|Q86SP6\|GP149_HUMAN Probable G-protein coupled receptor 149 OS=Homo sapiens GN=GPR149 PE=2 S' | 583 | AEGQKItPASK | 0.029 | -0.618 | -1.89 | -0.826 |
| sp\|Q86SP6\|GP149_HUMAN Probable G-protein coupled receptor 149 OS=Homo sapiens GN=GPR149 PE=2 S' | 586 | QKITPAsKKIE | 0.277 | -0.093 | -0.629 | -0.148 |
| sp\|Q86SP6\|GP149_HUMAN Probable G-protein coupled receptor 149 OS=Homo sapiens GN=GPR149 PE=2 S' | 594 | KIEVYRsKSVG | 0.098 | -0.073 | -1.208 | -0.394 |
| sp\|Q86SP6\|GP149_HUMAN Probable G-protein coupled receptor 149 OS=Homo sapiens GN=GPR149 PE=2 S' | 596 | EVYRSKsVGHE | 0.742 | 1.083 | 1.07 | 0.965 |
| sp\|Q86SP6\|GP149_HUMAN Probable G-protein coupled receptor 149 OS=Homo sapiens GN=GPR149 PE=2 S' | 603 | VGHEPNsEDSS | 0.071 | -0.146 | -1.247 | -0.441 |
| sp\|Q86SP6\|GP149_HUMAN Probable G-protein coupled receptor 149 OS=Homo sapiens GN=GPR149 PE=2 S' | 606 | EPNSEDsSSTF | 0.031 | -0.448 | -2.03 | -0.816 |
| sp\|Q86SP6\|GP149_HUMAN Probable G-protein coupled receptor 149 OS=Homo sapiens GN=GPR149 PE=2 S' | 607 | PNSEDSsSTFV | 0.044 | -0.393 | -1.854 | -0.734 |
| sp\|Q86SP6\|GP149_HUMAN Probable G-protein coupled receptor 149 OS=Homo sapiens GN=GPR149 PE=2 S' | 608 | NSEDSSsTFVD | 0.298 | 0.098 | -0.444 | -0.016 |
| sp\|Q86SP6\|GP149_HUMAN Probable G-protein coupled receptor 149 OS=Homo sapiens GN=GPR149 PE=2 S' | 609 | SEDSSStFVDT | 0.185 | 0.023 | -0.83 | -0.207 |
| sp\|Q86SP6\|GP149_HUMAN Probable G-protein coupled receptor 149 OS=Homo sapiens GN=GPR149 PE=2 S' | 613 | SSTFVDtSVKI | 0.147 | -0.223 | -1.304 | -0.46 |
| sp\|Q86SP6\|GP149_HUMAN Probable G-protein coupled receptor 149 OS=Homo sapiens GN=GPR149 PE=2 S' | 614 | STFVDTsVKIH | 0.069 | -0.298 | -1.577 | -0.602 |
| sp\|Q86SP6\|GP149_HUMAN Probable G-protein coupled receptor 149 OS=Homo sapiens GN=GPR149 PE=2 S' | 633 | NEEALDtVSII | 0.155 | -0.229 | -1.181 | -0.418 |
| sp\|Q86SP6\|GP149_HUMAN Probable G-protein coupled receptor 149 OS=Homo sapiens GN=GPR149 PE=2 S' | 635 | EALDTVsIISN | 0.067 | -0.126 | -1.101 | -0.387 |
| sp\|Q86SP6\|GP149_HUMAN Probable G-protein coupled receptor 149 OS=Homo sapiens GN=GPR149 PE=2 S' | 638 | DTVSIIsNISQ | 0.076 | -0.276 | -1.431 | -0.544 |
| sp\|Q86SP6\|GP149_HUMAN Probable G-protein coupled receptor 149 OS=Homo sapiens GN=GPR149 PE=2 S' | 641 | SIISNIsQSST | 0.047 | -0.276 | -1.676 | -0.635 |
| sp\|Q86SP6\|GP149_HUMAN Probable G-protein coupled receptor 149 OS=Homo sapiens GN=GPR149 PE=2 S' | 643 | ISNISQsSTQV | 0.337 | 0.051 | -0.644 | -0.085 |
| sp\|Q86SP6\|GP149_HUMAN Probable G-protein coupled receptor 149 OS=Homo sapiens GN=GPR149 PE=2 S' | 644 | SNISQSsTQVR | 0.04 | -0.27 | -1.645 | -0.625 |
| sp\|Q86SP6\|GP149_HUMAN Probable G-protein coupled receptor 149 OS=Homo sapiens GN=GPR149 PE=2 S' | 645 | NISQSStQVRS | 0.21 | 0.014 | -0.881 | -0.219 |

| | | | | | | |
|---|---|---|---|---|---|---|
| sp\|Q86SP6\|GP149_HUMAN Probable G-protein coupled receptor 149 OS=Homo sapiens GN=GPR149 PE=2 S | 649 | SSTQVRsPSLR | 0.047 | -0.497 | -1.692 | -0.714 |
| sp\|Q86SP6\|GP149_HUMAN Probable G-protein coupled receptor 149 OS=Homo sapiens GN=GPR149 PE=2 S | 651 | TQVRSPsLRYS | 0.731 | 0.927 | 0.731 | 0.796 |
| sp\|Q86SP6\|GP149_HUMAN Probable G-protein coupled receptor 149 OS=Homo sapiens GN=GPR149 PE=2 S | 655 | SPSLRYsRKEN | 0.177 | -0.004 | -0.982 | -0.27 |
| sp\|Q86SP6\|GP149_HUMAN Probable G-protein coupled receptor 149 OS=Homo sapiens GN=GPR149 PE=2 S | 663 | KENRFVsCDLG | 0.734 | 0.9 | 0.182 | 0.605 |
| sp\|Q86SP6\|GP149_HUMAN Probable G-protein coupled receptor 149 OS=Homo sapiens GN=GPR149 PE=2 S | 669 | SCDLGEtASYS | 0.043 | -0.288 | -2.165 | -0.803 |
| sp\|Q86SP6\|GP149_HUMAN Probable G-protein coupled receptor 149 OS=Homo sapiens GN=GPR149 PE=2 S | 671 | DLGETAsYSLF | 0.072 | -0.051 | -1.329 | -0.436 |
| sp\|Q86SP6\|GP149_HUMAN Probable G-protein coupled receptor 149 OS=Homo sapiens GN=GPR149 PE=2 S | 673 | GETASYsLFLP | 0.526 | 0.32 | 0.185 | 0.344 |
| sp\|Q86SP6\|GP149_HUMAN Probable G-protein coupled receptor 149 OS=Homo sapiens GN=GPR149 PE=2 S | 678 | YSLFLPtSNPD | 0.134 | -0.218 | -0.892 | -0.325 |
| sp\|Q86SP6\|GP149_HUMAN Probable G-protein coupled receptor 149 OS=Homo sapiens GN=GPR149 PE=2 S | 679 | SLFLPTsNPDG | 0.615 | 0.461 | -0.063 | 0.338 |
| sp\|Q86SP6\|GP149_HUMAN Probable G-protein coupled receptor 149 OS=Homo sapiens GN=GPR149 PE=2 S | 688 | DGDINIsIPDT | 0.596 | 0.376 | 0.089 | 0.354 |
| sp\|Q86SP6\|GP149_HUMAN Probable G-protein coupled receptor 149 OS=Homo sapiens GN=GPR149 PE=2 S | 692 | NISIPDtVEAH | 0.118 | -0.181 | -1.151 | -0.405 |
| sp\|Q86SP6\|GP149_HUMAN Probable G-protein coupled receptor 149 OS=Homo sapiens GN=GPR149 PE=2 S | 700 | EAHRQNsKRQH | 0.341 | 0.568 | -0.385 | 0.175 |
| sp\|Q86SP6\|GP149_HUMAN Probable G-protein coupled receptor 149 OS=Homo sapiens GN=GPR149 PE=2 S | 728 | RKREEEsKGS- | 0.377 | 0.244 | -0.613 | 0.003 |
| sp\|Q86SP6\|GP149_HUMAN Probable G-protein coupled receptor 149 OS=Homo sapiens GN=GPR149 PE=2 S | 731 | EEESKGs---- | 0.059 | -0.149 | -1.487 | -0.526 |
| sp\|Q8NGU9\|GP150_HUMAN Probable G-protein coupled receptor 150 OS=Homo sapiens GN=GPR150 PE=3 S | 321 | RLAAAWsSGPA | 0.489 | 0.038 | -0.19 | 0.112 |
| sp\|Q8NGU9\|GP150_HUMAN Probable G-protein coupled receptor 150 OS=Homo sapiens GN=GPR150 PE=3 S | 322 | LAAAWSsGPAG | 0.448 | 0.37 | 0.088 | 0.302 |
| sp\|Q8NGU9\|GP150_HUMAN Probable G-protein coupled receptor 150 OS=Homo sapiens GN=GPR150 PE=3 S | 334 | WEGEGLsAALR | 0.033 | -0.247 | -1.713 | -0.642 |
| sp\|Q8NGU9\|GP150_HUMAN Probable G-protein coupled receptor 150 OS=Homo sapiens GN=GPR150 PE=3 S | 345 | VVAMANsALNP | 0.186 | 0 | -1.044 | -0.286 |
| sp\|Q8NGU9\|GP150_HUMAN Probable G-protein coupled receptor 150 OS=Homo sapiens GN=GPR150 PE=3 S | 372 | LRKRLGsLCCA | 0.631 | 0.996 | 0.445 | 0.691 |
| sp\|Q8NGU9\|GP150_HUMAN Probable G-protein coupled receptor 150 OS=Homo sapiens GN=GPR150 PE=3 S | 429 | PRPLPCsCESA | 0.126 | 0.082 | -1.099 | -0.297 |
| sp\|Q8NGU9\|GP150_HUMAN Probable G-protein coupled receptor 150 OS=Homo sapiens GN=GPR150 PE=3 S | 432 | LPCSCEsAF-- | 0.215 | 0.024 | -1.054 | -0.272 |
| sp\|Q8TDV0\|GP151_HUMAN Probable G-protein coupled receptor 151 OS=Homo sapiens GN=GPR151 PE=2 S | 310 | LIFLVMsEEFR | 0.27 | 0.181 | -0.61 | -0.053 |
| sp\|Q8TDV0\|GP151_HUMAN Probable G-protein coupled receptor 151 OS=Homo sapiens GN=GPR151 PE=2 S | 326 | VWKWMItKKPP | 0.185 | -0.234 | -0.611 | -0.22 |
| sp\|Q8TDV0\|GP151_HUMAN Probable G-protein coupled receptor 151 OS=Homo sapiens GN=GPR151 PE=2 S | 331 | ITKKPPtVSES | 0.273 | -0.168 | -0.65 | -0.182 |
| sp\|Q8TDV0\|GP151_HUMAN Probable G-protein coupled receptor 151 OS=Homo sapiens GN=GPR151 PE=2 S | 333 | KKPPTVsESQE | 0.183 | -0.115 | -0.834 | -0.255 |
| sp\|Q8TDV0\|GP151_HUMAN Probable G-protein coupled receptor 151 OS=Homo sapiens GN=GPR151 PE=2 S | 335 | PPTVSEsQETP | 0.124 | -0.058 | -1.168 | -0.367 |
| sp\|Q8TDV0\|GP151_HUMAN Probable G-protein coupled receptor 151 OS=Homo sapiens GN=GPR151 PE=2 S | 338 | VSESQEtPAGN | 0.038 | -0.648 | -1.918 | -0.843 |
| sp\|Q8TDV0\|GP151_HUMAN Probable G-protein coupled receptor 151 OS=Homo sapiens GN=GPR151 PE=2 S | 343 | ETPAGNsEGLP | 0.13 | -0.105 | -1.101 | -0.359 |
| sp\|Q8TDV0\|GP151_HUMAN Probable G-protein coupled receptor 151 OS=Homo sapiens GN=GPR151 PE=2 S | 352 | LPDKVPsPESP | 0.046 | -0.545 | -1.758 | -0.752 |
| sp\|Q8TDV0\|GP151_HUMAN Probable G-protein coupled receptor 151 OS=Homo sapiens GN=GPR151 PE=2 S | 355 | KVPSPEsPASI | 0.018 | -0.658 | -2.002 | -0.881 |
| sp\|Q8TDV0\|GP151_HUMAN Probable G-protein coupled receptor 151 OS=Homo sapiens GN=GPR151 PE=2 S | 358 | SPESPAsIPEK | 0.339 | 0.166 | -0.459 | 0.015 |
| sp\|Q8TDV0\|GP151_HUMAN Probable G-protein coupled receptor 151 OS=Homo sapiens GN=GPR151 PE=2 S | 366 | PEKEKPsSPSS | 0.169 | 0.244 | -0.811 | -0.133 |
| sp\|Q8TDV0\|GP151_HUMAN Probable G-protein coupled receptor 151 OS=Homo sapiens GN=GPR151 PE=2 S | 367 | EKEKPSsPSSG | 0.055 | -0.481 | -1.326 | -0.584 |
| sp\|Q8TDV0\|GP151_HUMAN Probable G-protein coupled receptor 151 OS=Homo sapiens GN=GPR151 PE=2 S | 369 | EKPSSPsSGKG | 0.275 | 0.053 | -0.525 | -0.066 |
| sp\|Q8TDV0\|GP151_HUMAN Probable G-protein coupled receptor 151 OS=Homo sapiens GN=GPR151 PE=2 S | 370 | KPSSPSsGKGK | 0.04 | -0.526 | -1.487 | -0.658 |
| sp\|Q8TDV0\|GP151_HUMAN Probable G-protein coupled receptor 151 OS=Homo sapiens GN=GPR151 PE=2 S | 375 | SSGKGKtEKAE | 0.179 | -0.039 | -0.646 | -0.169 |
| sp\|Q8TDV0\|GP151_HUMAN Probable G-protein coupled receptor 151 OS=Homo sapiens GN=GPR151 PE=2 S | 395 | FWHERDtVPSV | 0.392 | 0.558 | -0.569 | 0.127 |
| sp\|Q8TDV0\|GP151_HUMAN Probable G-protein coupled receptor 151 OS=Homo sapiens GN=GPR151 PE=2 S | 398 | ERDTVPsVQDN | 0.275 | 0.162 | -0.657 | -0.073 |
| sp\|Q8TDV0\|GP151_HUMAN Probable G-protein coupled receptor 151 OS=Homo sapiens GN=GPR151 PE=2 S | 414 | EHEDQEtGEGV | 0.062 | -0.24 | -1.852 | -0.677 |
| sp\|Q8TDT2\|GP152_HUMAN Probable G-protein coupled receptor 152 OS=Homo sapiens GN=GPR152 PE=2 S | 308 | LRTLLRsVLSS | 0.047 | 0.035 | -1.488 | -0.469 |
| sp\|Q8TDT2\|GP152_HUMAN Probable G-protein coupled receptor 152 OS=Homo sapiens GN=GPR152 PE=2 S | 311 | LLRSVLsSFAA | 0.388 | 0.212 | -0.236 | 0.121 |
| sp\|Q8TDT2\|GP152_HUMAN Probable G-protein coupled receptor 152 OS=Homo sapiens GN=GPR152 PE=2 S | 312 | LRSVLSsFAAA | 0.109 | 0.07 | -0.822 | -0.214 |
| sp\|Q8TDT2\|GP152_HUMAN Probable G-protein coupled receptor 152 OS=Homo sapiens GN=GPR152 PE=2 S | 324 | CEERPGsFTPT | 0.529 | 0.628 | 0.177 | 0.445 |
| sp\|Q8TDT2\|GP152_HUMAN Probable G-protein coupled receptor 152 OS=Homo sapiens GN=GPR152 PE=2 S | 326 | ERPGSFtPTEP | 0.136 | 0.024 | -0.804 | -0.215 |
| sp\|Q8TDT2\|GP152_HUMAN Probable G-protein coupled receptor 152 OS=Homo sapiens GN=GPR152 PE=2 S | 328 | PGSFTPtEPQT | 0.489 | 0.434 | 0.098 | 0.34 |
| sp\|Q8TDT2\|GP152_HUMAN Probable G-protein coupled receptor 152 OS=Homo sapiens GN=GPR152 PE=2 S | 332 | TPTEPQtQLDS | 0.061 | -0.244 | -1.46 | -0.548 |
| sp\|Q8TDT2\|GP152_HUMAN Probable G-protein coupled receptor 152 OS=Homo sapiens GN=GPR152 PE=2 S | 336 | PQTQLDsEGPT | 0.178 | -0.127 | -0.684 | -0.211 |
| sp\|Q8TDT2\|GP152_HUMAN Probable G-protein coupled receptor 152 OS=Homo sapiens GN=GPR152 PE=2 S | 340 | LDSEGPtLPEP | 0.231 | 0.228 | -0.431 | 0.009 |
| sp\|Q8TDT2\|GP152_HUMAN Probable G-protein coupled receptor 152 OS=Homo sapiens GN=GPR152 PE=2 S | 350 | PMAEAQsQMDP | 0.17 | 0.104 | -0.861 | -0.196 |

| | | | | | | |
|---|---|---|---|---|---|---|
| sp\|Q8TDT2\|GP152_HUMAN Probable G-protein coupled receptor 152 OS=Homo sapiens GN=GPR152 PE=2 S | 363 | QPQVNPtLQPR | 0.222 | -0.067 | -0.544 | -0.13 |
| sp\|Q8TDT2\|GP152_HUMAN Probable G-protein coupled receptor 152 OS=Homo sapiens GN=GPR152 PE=2 S | 368 | PTLQPRsDPTA | 0.584 | 0.355 | -0.042 | 0.299 |
| sp\|Q8TDT2\|GP152_HUMAN Probable G-protein coupled receptor 152 OS=Homo sapiens GN=GPR152 PE=2 S | 371 | QPRSDPtAQPQ | 0.176 | -0.062 | -1.019 | -0.302 |
| sp\|Q8TDT2\|GP152_HUMAN Probable G-protein coupled receptor 152 OS=Homo sapiens GN=GPR152 PE=2 S | 379 | QPQLNPtAQPQ | 0.136 | -0.158 | -1.124 | -0.382 |
| sp\|Q8TDT2\|GP152_HUMAN Probable G-protein coupled receptor 152 OS=Homo sapiens GN=GPR152 PE=2 S | 384 | PTAQPQsDPTA | 0.663 | 0.386 | 0.292 | 0.447 |
| sp\|Q8TDT2\|GP152_HUMAN Probable G-protein coupled receptor 152 OS=Homo sapiens GN=GPR152 PE=2 S | 387 | QPQSDPtAQPQ | 0.089 | -0.255 | -1.369 | -0.512 |
| sp\|Q8TDT2\|GP152_HUMAN Probable G-protein coupled receptor 152 OS=Homo sapiens GN=GPR152 PE=2 S | 400 | LMAQPQsDSVA | 0.116 | -0.028 | -0.743 | -0.218 |
| sp\|Q8TDT2\|GP152_HUMAN Probable G-protein coupled receptor 152 OS=Homo sapiens GN=GPR152 PE=2 S | 402 | AQPQSDsVAQP | 0.152 | -0.136 | -0.824 | -0.269 |
| sp\|Q8TDT2\|GP152_HUMAN Probable G-protein coupled receptor 152 OS=Homo sapiens GN=GPR152 PE=2 S | 410 | AQPQADtNVQT | 0.118 | -0.27 | -1.167 | -0.44 |
| sp\|Q8TDT2\|GP152_HUMAN Probable G-protein coupled receptor 152 OS=Homo sapiens GN=GPR152 PE=2 S | 414 | ADTNVQtPAPA | 0.035 | -0.589 | -1.526 | -0.693 |
| sp\|Q8TDT2\|GP152_HUMAN Probable G-protein coupled receptor 152 OS=Homo sapiens GN=GPR152 PE=2 S | 420 | TPAPAAsSVPS | 0.111 | -0.178 | -1.083 | -0.383 |
| sp\|Q8TDT2\|GP152_HUMAN Probable G-protein coupled receptor 152 OS=Homo sapiens GN=GPR152 PE=2 S | 421 | PAPAASsVPSP | 0.226 | 0.254 | -0.591 | -0.037 |
| sp\|Q8TDT2\|GP152_HUMAN Probable G-protein coupled receptor 152 OS=Homo sapiens GN=GPR152 PE=2 S | 424 | AASSVPsPCDE | 0.067 | -0.646 | -1.754 | -0.778 |
| sp\|Q8TDT2\|GP152_HUMAN Probable G-protein coupled receptor 152 OS=Homo sapiens GN=GPR152 PE=2 S | 430 | SPCDEAsPTPS | 0.06 | -0.518 | -1.992 | -0.817 |
| sp\|Q8TDT2\|GP152_HUMAN Probable G-protein coupled receptor 152 OS=Homo sapiens GN=GPR152 PE=2 S | 432 | CDEASPtPSSH | 0.042 | -0.508 | -1.536 | -0.667 |
| sp\|Q8TDT2\|GP152_HUMAN Probable G-protein coupled receptor 152 OS=Homo sapiens GN=GPR152 PE=2 S | 434 | EASPTPsSHPT | 0.041 | -0.347 | -1.445 | -0.584 |
| sp\|Q8TDT2\|GP152_HUMAN Probable G-protein coupled receptor 152 OS=Homo sapiens GN=GPR152 PE=2 S | 435 | ASPTPSsHPTP | 0.179 | 0.045 | -0.828 | -0.201 |
| sp\|Q8TDT2\|GP152_HUMAN Probable G-protein coupled receptor 152 OS=Homo sapiens GN=GPR152 PE=2 S | 438 | TPSSHPtPGAL | 0.109 | -0.338 | -1.093 | -0.441 |
| sp\|Q8TDT2\|GP152_HUMAN Probable G-protein coupled receptor 152 OS=Homo sapiens GN=GPR152 PE=2 S | 447 | ALEDPAtPPAS | 0.509 | 0.223 | -0.196 | 0.179 |
| sp\|Q8TDT2\|GP152_HUMAN Probable G-protein coupled receptor 152 OS=Homo sapiens GN=GPR152 PE=2 S | 451 | PATPPAsEGES | 0.287 | -0.034 | -0.533 | -0.093 |
| sp\|Q8TDT2\|GP152_HUMAN Probable G-protein coupled receptor 152 OS=Homo sapiens GN=GPR152 PE=2 S | 455 | PASEGEsPSST | 0.011 | -0.69 | -2.356 | -1.012 |
| sp\|Q8TDT2\|GP152_HUMAN Probable G-protein coupled receptor 152 OS=Homo sapiens GN=GPR152 PE=2 S | 457 | SEGESPsSTPP | 0.066 | -0.189 | -1.531 | -0.551 |
| sp\|Q8TDT2\|GP152_HUMAN Probable G-protein coupled receptor 152 OS=Homo sapiens GN=GPR152 PE=2 S | 458 | EGESPSsTPPE | 0.181 | 0.086 | -0.553 | -0.095 |
| sp\|Q8TDT2\|GP152_HUMAN Probable G-protein coupled receptor 152 OS=Homo sapiens GN=GPR152 PE=2 S | 459 | GESPSStPPEA | 0.437 | 0.251 | -0.236 | 0.151 |
| sp\|Q8TDT2\|GP152_HUMAN Probable G-protein coupled receptor 152 OS=Homo sapiens GN=GPR152 PE=2 S | 470 | APGAGPt---- | 0.065 | -0.166 | -1.436 | -0.512 |
| sp\|Q6NV75\|GP153_HUMAN Probable G-protein coupled receptor 153 OS=Homo sapiens GN=GPR153 PE=2 S | 324 | MANDEEsDDET | 0.258 | -0.051 | -1.192 | -0.328 |
| sp\|Q6NV75\|GP153_HUMAN Probable G-protein coupled receptor 153 OS=Homo sapiens GN=GPR153 PE=2 S | 328 | EESDDEtSLEG | 0.076 | -0.232 | -1.634 | -0.597 |
| sp\|Q6NV75\|GP153_HUMAN Probable G-protein coupled receptor 153 OS=Homo sapiens GN=GPR153 PE=2 S | 329 | ESDDETsLEGG | 0.199 | -0.128 | -0.817 | -0.249 |
| sp\|Q6NV75\|GP153_HUMAN Probable G-protein coupled receptor 153 OS=Homo sapiens GN=GPR153 PE=2 S | 335 | SLEGGIsPDLV | 0.089 | -0.322 | -1.334 | -0.522 |
| sp\|Q6NV75\|GP153_HUMAN Probable G-protein coupled receptor 153 OS=Homo sapiens GN=GPR153 PE=2 S | 343 | DLVLERsLDYG | 0.444 | 0.099 | -0.7 | -0.052 |
| sp\|Q6NV75\|GP153_HUMAN Probable G-protein coupled receptor 153 OS=Homo sapiens GN=GPR153 PE=2 S | 364 | MAKYEIsALEG | 0.356 | 0.066 | -0.667 | -0.082 |
| sp\|Q6NV75\|GP153_HUMAN Probable G-protein coupled receptor 153 OS=Homo sapiens GN=GPR153 PE=2 S | 392 | YLQVPPtRRFS | 0.077 | -0.295 | -1.238 | -0.485 |
| sp\|Q6NV75\|GP153_HUMAN Probable G-protein coupled receptor 153 OS=Homo sapiens GN=GPR153 PE=2 S | 396 | PPTRRFsHDDA | 0.893 | 1.123 | 1.191 | 1.069 |
| sp\|Q6NV75\|GP153_HUMAN Probable G-protein coupled receptor 153 OS=Homo sapiens GN=GPR153 PE=2 S | 417 | FLPRWGsGEDL | 0.673 | 0.966 | 0.424 | 0.688 |
| sp\|Q6NV75\|GP153_HUMAN Probable G-protein coupled receptor 153 OS=Homo sapiens GN=GPR153 PE=2 S | 439 | PERRRAsLLAF | 0.928 | 1.4 | 1.354 | 1.227 |
| sp\|Q6NV75\|GP153_HUMAN Probable G-protein coupled receptor 153 OS=Homo sapiens GN=GPR153 PE=2 S | 450 | AEDAPPsRARR | 0.073 | -0.521 | -1.687 | -0.712 |
| sp\|Q6NV75\|GP153_HUMAN Probable G-protein coupled receptor 153 OS=Homo sapiens GN=GPR153 PE=2 S | 456 | SRARRRsAESL | 0.528 | 1.169 | 0.091 | 0.596 |
| sp\|Q6NV75\|GP153_HUMAN Probable G-protein coupled receptor 153 OS=Homo sapiens GN=GPR153 PE=2 S | 459 | RRRSAEsLLSL | 0.503 | 0.511 | -0.201 | 0.271 |
| sp\|Q6NV75\|GP153_HUMAN Probable G-protein coupled receptor 153 OS=Homo sapiens GN=GPR153 PE=2 S | 462 | SAESLLsLRPS | 0.063 | -0.359 | -1.41 | -0.569 |
| sp\|Q6NV75\|GP153_HUMAN Probable G-protein coupled receptor 153 OS=Homo sapiens GN=GPR153 PE=2 S | 466 | LLSLRPsALDS | 0.36 | 0.158 | -0.592 | -0.025 |
| sp\|Q6NV75\|GP153_HUMAN Probable G-protein coupled receptor 153 OS=Homo sapiens GN=GPR153 PE=2 S | 470 | RPSALDsGPRG | 0.366 | 0.146 | -0.753 | -0.08 |
| sp\|Q6NV75\|GP153_HUMAN Probable G-protein coupled receptor 153 OS=Homo sapiens GN=GPR153 PE=2 S | 478 | PRGARDsPPGS | 0.445 | 0.364 | -0.303 | 0.169 |
| sp\|Q6NV75\|GP153_HUMAN Probable G-protein coupled receptor 153 OS=Homo sapiens GN=GPR153 PE=2 S | 482 | RDSPPGsPRRR | 0.031 | -0.758 | -1.847 | -0.858 |
| sp\|Q6NV75\|GP153_HUMAN Probable G-protein coupled receptor 153 OS=Homo sapiens GN=GPR153 PE=2 S | 493 | PGPGPRsASAS | 0.058 | -0.176 | -1.336 | -0.485 |
| sp\|Q6NV75\|GP153_HUMAN Probable G-protein coupled receptor 153 OS=Homo sapiens GN=GPR153 PE=2 S | 495 | PGPRSAsASLL | 0.734 | 1.079 | 0.554 | 0.789 |
| sp\|Q6NV75\|GP153_HUMAN Probable G-protein coupled receptor 153 OS=Homo sapiens GN=GPR153 PE=2 S | 497 | PRSASAsLLPD | 0.646 | 0.443 | 0.263 | 0.451 |
| sp\|Q6NV75\|GP153_HUMAN Probable G-protein coupled receptor 153 OS=Homo sapiens GN=GPR153 PE=2 S | 506 | PDAFALtAFEC | 0.174 | -0.053 | -0.948 | -0.276 |
| sp\|Q6NV75\|GP153_HUMAN Probable G-protein coupled receptor 153 OS=Homo sapiens GN=GPR153 PE=2 S | 539 | DPGEAPtPPSS | 0.119 | 0.009 | -1.173 | -0.348 |
| sp\|Q6NV75\|GP153_HUMAN Probable G-protein coupled receptor 153 OS=Homo sapiens GN=GPR153 PE=2 S | 542 | EAPTPPsSAQR | 0.048 | -0.442 | -1.477 | -0.624 |

| | | | | | | |
|---|---|---|---|---|---|---|
| sp\|Q6NV75\|GP153_HUMAN Probable G-protein coupled receptor 153 OS=Homo sapiens GN=GPR153 PE=2 S | 543 | APTPPSsAQRS | 0.066 | -0.364 | -1.517 | -0.605 |
| sp\|Q6NV75\|GP153_HUMAN Probable G-protein coupled receptor 153 OS=Homo sapiens GN=GPR153 PE=2 S | 547 | PSSAQRsPGPR | 0.062 | -0.459 | -1.496 | -0.631 |
| sp\|Q6NV75\|GP153_HUMAN Probable G-protein coupled receptor 153 OS=Homo sapiens GN=GPR153 PE=2 S | 553 | SPGPRPsAHSH | 0.057 | -0.233 | -1.727 | -0.634 |
| sp\|Q6NV75\|GP153_HUMAN Probable G-protein coupled receptor 153 OS=Homo sapiens GN=GPR153 PE=2 S | 556 | PRPSAHsHAGS | 0.303 | 0.183 | -0.61 | -0.041 |
| sp\|Q6NV75\|GP153_HUMAN Probable G-protein coupled receptor 153 OS=Homo sapiens GN=GPR153 PE=2 S | 560 | AHSHAGsLRPG | 0.24 | -0.159 | -0.916 | -0.278 |
| sp\|Q6NV75\|GP153_HUMAN Probable G-protein coupled receptor 153 OS=Homo sapiens GN=GPR153 PE=2 S | 566 | SLRPGLsASWG | 0.144 | 0.081 | -1.004 | -0.26 |
| sp\|Q6NV75\|GP153_HUMAN Probable G-protein coupled receptor 153 OS=Homo sapiens GN=GPR153 PE=2 S | 568 | RPGLSAsWGEP | 0.377 | 0.286 | -0.44 | 0.074 |
| sp\|Q6NV75\|GP153_HUMAN Probable G-protein coupled receptor 153 OS=Homo sapiens GN=GPR153 PE=2 S | 583 | AAGGGGsTSSF | 0.016 | -0.455 | -2.119 | -0.853 |
| sp\|Q6NV75\|GP153_HUMAN Probable G-protein coupled receptor 153 OS=Homo sapiens GN=GPR153 PE=2 S | 584 | AGGGGStSSFL | 0.022 | -0.398 | -1.858 | -0.745 |
| sp\|Q6NV75\|GP153_HUMAN Probable G-protein coupled receptor 153 OS=Homo sapiens GN=GPR153 PE=2 S | 585 | GGGGSTsSFLS | 0.223 | 0.143 | -0.46 | -0.031 |
| sp\|Q6NV75\|GP153_HUMAN Probable G-protein coupled receptor 153 OS=Homo sapiens GN=GPR153 PE=2 S | 586 | GGGSTSsFLSS | 0.046 | -0.166 | -1.49 | -0.537 |
| sp\|Q6NV75\|GP153_HUMAN Probable G-protein coupled receptor 153 OS=Homo sapiens GN=GPR153 PE=2 S | 589 | STSSFLsSPSE | 0.13 | 0.096 | -1.202 | -0.325 |
| sp\|Q6NV75\|GP153_HUMAN Probable G-protein coupled receptor 153 OS=Homo sapiens GN=GPR153 PE=2 S | 590 | TSSFLSsPSES | 0.026 | -0.615 | -2.029 | -0.873 |
| sp\|Q6NV75\|GP153_HUMAN Probable G-protein coupled receptor 153 OS=Homo sapiens GN=GPR153 PE=2 S | 592 | SFLSSPsESSG | 0.132 | -0.002 | -0.825 | -0.232 |
| sp\|Q6NV75\|GP153_HUMAN Probable G-protein coupled receptor 153 OS=Homo sapiens GN=GPR153 PE=2 S | 594 | LSSPSEsSGYA | 0.218 | -0.072 | -0.697 | -0.184 |
| sp\|Q6NV75\|GP153_HUMAN Probable G-protein coupled receptor 153 OS=Homo sapiens GN=GPR153 PE=2 S | 595 | SSPSESsGYAT | 0.035 | -0.416 | -1.752 | -0.711 |
| sp\|Q6NV75\|GP153_HUMAN Probable G-protein coupled receptor 153 OS=Homo sapiens GN=GPR153 PE=2 S | 599 | ESSGYAtLHSD | 0.276 | 0 | -0.454 | -0.059 |
| sp\|Q6NV75\|GP153_HUMAN Probable G-protein coupled receptor 153 OS=Homo sapiens GN=GPR153 PE=2 S | 602 | GYATLHsDSLG | 0.311 | 0.108 | -0.812 | -0.131 |
| sp\|Q6NV75\|GP153_HUMAN Probable G-protein coupled receptor 153 OS=Homo sapiens GN=GPR153 PE=2 S | 604 | ATLHSDsLGSA | 0.456 | 0.094 | -0.216 | 0.111 |
| sp\|Q6NV75\|GP153_HUMAN Probable G-protein coupled receptor 153 OS=Homo sapiens GN=GPR153 PE=2 S | 607 | HSDSLGsAS-- | 0.044 | -0.156 | -1.728 | -0.613 |
| sp\|Q6NV75\|GP153_HUMAN Probable G-protein coupled receptor 153 OS=Homo sapiens GN=GPR153 PE=2 S | 609 | DSLGSAs---- | 0.428 | 0.278 | -0.334 | 0.124 |
| sp\|Q9UJ42\|GP160_HUMAN Probable G-protein coupled receptor 160 OS=Homo sapiens GN=GPR160 PE=2 S' | 322 | CCFIPLtIPNL | 0.437 | 0.49 | -0.181 | 0.249 |
| sp\|Q9UJ42\|GP160_HUMAN Probable G-protein coupled receptor 160 OS=Homo sapiens GN=GPR160 PE=2 S' | 334 | QIEKPIsIMIC | 0.243 | -0.139 | -0.721 | -0.206 |
| sp\|Q8N6U8\|GP161_HUMAN G-protein coupled receptor 161 OS=Homo sapiens GN=GPR161 PE=2 SV=1 | 310 | SLETWAtWLSF | 0.12 | 0.026 | -1.08 | -0.311 |
| sp\|Q8N6U8\|GP161_HUMAN G-protein coupled receptor 161 OS=Homo sapiens GN=GPR161 PE=2 SV=1 | 313 | TWATWLsFASA | 0.097 | -0.109 | -0.929 | -0.314 |
| sp\|Q8N6U8\|GP161_HUMAN G-protein coupled receptor 161 OS=Homo sapiens GN=GPR161 PE=2 SV=1 | 316 | TWLSFAsAVCH | 0.116 | -0.111 | -1.428 | -0.474 |
| sp\|Q8N6U8\|GP161_HUMAN G-protein coupled receptor 161 OS=Homo sapiens GN=GPR161 PE=2 SV=1 | 330 | YGLWNKtVRKE | 0.305 | -0.119 | -0.226 | -0.013 |
| sp\|Q8N6U8\|GP161_HUMAN G-protein coupled receptor 161 OS=Homo sapiens GN=GPR161 PE=2 SV=1 | 355 | FVQRQRtSRLF | 0.36 | 0.85 | -0.215 | 0.332 |
| sp\|Q8N6U8\|GP161_HUMAN G-protein coupled receptor 161 OS=Homo sapiens GN=GPR161 PE=2 SV=1 | 356 | VQRQRTsRLFS | 0.245 | 0.102 | -0.595 | -0.083 |
| sp\|Q8N6U8\|GP161_HUMAN G-protein coupled receptor 161 OS=Homo sapiens GN=GPR161 PE=2 SV=1 | 360 | RTSRLFsISNR | 0.459 | 0.77 | -0.148 | 0.36 |
| sp\|Q8N6U8\|GP161_HUMAN G-protein coupled receptor 161 OS=Homo sapiens GN=GPR161 PE=2 SV=1 | 362 | SRLFSIsNRIT | 0.38 | 0.216 | -0.449 | 0.049 |
| sp\|Q8N6U8\|GP161_HUMAN G-protein coupled receptor 161 OS=Homo sapiens GN=GPR161 PE=2 SV=1 | 366 | SISNRItDLGL | 0.236 | 0.098 | -0.661 | -0.109 |
| sp\|Q8N6U8\|GP161_HUMAN G-protein coupled receptor 161 OS=Homo sapiens GN=GPR161 PE=2 SV=1 | 371 | ITDLGLsPHLT | 0.038 | -0.518 | -1.719 | -0.733 |
| sp\|Q8N6U8\|GP161_HUMAN G-protein coupled receptor 161 OS=Homo sapiens GN=GPR161 PE=2 SV=1 | 375 | GLSPHLtALMA | 0.185 | -0.049 | -1.01 | -0.291 |
| sp\|Q8N6U8\|GP161_HUMAN G-protein coupled receptor 161 OS=Homo sapiens GN=GPR161 PE=2 SV=1 | 387 | GQPLGHsSSTG | 0.079 | -0.136 | -1.319 | -0.459 |
| sp\|Q8N6U8\|GP161_HUMAN G-protein coupled receptor 161 OS=Homo sapiens GN=GPR161 PE=2 SV=1 | 388 | QPLGHSsSTGD | 0.138 | -0.192 | -0.961 | -0.338 |
| sp\|Q8N6U8\|GP161_HUMAN G-protein coupled receptor 161 OS=Homo sapiens GN=GPR161 PE=2 SV=1 | 389 | PLGHSSsTGDT | 0.433 | 0.233 | -0.074 | 0.197 |
| sp\|Q8N6U8\|GP161_HUMAN G-protein coupled receptor 161 OS=Homo sapiens GN=GPR161 PE=2 SV=1 | 390 | LGHSSStGDTG | 0.144 | -0.048 | -0.677 | -0.194 |
| sp\|Q8N6U8\|GP161_HUMAN G-protein coupled receptor 161 OS=Homo sapiens GN=GPR161 PE=2 SV=1 | 393 | SSSTGDtGFSC | 0.031 | -0.529 | -2.117 | -0.872 |
| sp\|Q8N6U8\|GP161_HUMAN G-protein coupled receptor 161 OS=Homo sapiens GN=GPR161 PE=2 SV=1 | 396 | TGDTGFsCSQD | 0.093 | -0.15 | -1.072 | -0.376 |
| sp\|Q8N6U8\|GP161_HUMAN G-protein coupled receptor 161 OS=Homo sapiens GN=GPR161 PE=2 SV=1 | 398 | DTGFSCsQDSG | 0.373 | 0.115 | -0.846 | -0.119 |
| sp\|Q8N6U8\|GP161_HUMAN G-protein coupled receptor 161 OS=Homo sapiens GN=GPR161 PE=2 SV=1 | 401 | FSCSQDsGTDM | 0.187 | -0.185 | -1.142 | -0.38 |
| sp\|Q8N6U8\|GP161_HUMAN G-protein coupled receptor 161 OS=Homo sapiens GN=GPR161 PE=2 SV=1 | 403 | CSQDSGtDMML | 0.367 | 0.164 | -0.563 | -0.011 |
| sp\|Q8N6U8\|GP161_HUMAN G-protein coupled receptor 161 OS=Homo sapiens GN=GPR161 PE=2 SV=1 | 412 | MLLEDYtSDDN | 0.39 | 0.246 | -0.239 | 0.132 |
| sp\|Q8N6U8\|GP161_HUMAN G-protein coupled receptor 161 OS=Homo sapiens GN=GPR161 PE=2 SV=1 | 413 | LLEDYTsDDNP | 0.36 | 0.117 | -0.585 | -0.036 |
| sp\|Q8N6U8\|GP161_HUMAN G-protein coupled receptor 161 OS=Homo sapiens GN=GPR161 PE=2 SV=1 | 419 | SDDNPPsHCTC | 0.029 | -0.566 | -1.882 | -0.806 |
| sp\|Q8N6U8\|GP161_HUMAN G-protein coupled receptor 161 OS=Homo sapiens GN=GPR161 PE=2 SV=1 | 422 | NPPSHCtCPPK | 0.499 | 0.349 | -0.472 | 0.125 |
| sp\|Q8N6U8\|GP161_HUMAN G-protein coupled receptor 161 OS=Homo sapiens GN=GPR161 PE=2 SV=1 | 429 | CPPKRRsSVTF | 0.194 | 0.004 | -0.91 | -0.237 |
| sp\|Q8N6U8\|GP161_HUMAN G-protein coupled receptor 161 OS=Homo sapiens GN=GPR161 PE=2 SV=1 | 430 | PPKRRSsVTFE | 0.679 | 0.836 | 0.292 | 0.602 |

| | | | | | | |
|---|---|---|---|---|---|---|
| sp\|Q8N6U8\|GP161_HUMAN G-protein coupled receptor 161 OS=Homo sapiens GN=GPR161 PE=2 SV=1 | 432 | KRRSSVtFEDE | 0.817 | 0.666 | 0.577 | 0.687 |
| sp\|Q8N6U8\|GP161_HUMAN G-protein coupled receptor 161 OS=Homo sapiens GN=GPR161 PE=2 SV=1 | 447 | KEAAKNsILHV | 0.07 | -0.264 | -1.509 | -0.568 |
| sp\|Q8N6U8\|GP161_HUMAN G-protein coupled receptor 161 OS=Homo sapiens GN=GPR161 PE=2 SV=1 | 458 | KAEVHKsLDSY | 0.38 | 0.085 | -0.337 | 0.043 |
| sp\|Q8N6U8\|GP161_HUMAN G-protein coupled receptor 161 OS=Homo sapiens GN=GPR161 PE=2 SV=1 | 461 | VHKSLDsYAAS | 0.064 | -0.299 | -1.65 | -0.628 |
| sp\|Q8N6U8\|GP161_HUMAN G-protein coupled receptor 161 OS=Homo sapiens GN=GPR161 PE=2 SV=1 | 465 | LDSYAAsLAKA | 0.264 | -0.032 | -0.42 | -0.063 |
| sp\|Q8N6U8\|GP161_HUMAN G-protein coupled receptor 161 OS=Homo sapiens GN=GPR161 PE=2 SV=1 | 490 | LPGVLVtARTV | 0.104 | -0.22 | -1.473 | -0.53 |
| sp\|Q8N6U8\|GP161_HUMAN G-protein coupled receptor 161 OS=Homo sapiens GN=GPR161 PE=2 SV=1 | 493 | VLVTARtVPGG | 0.673 | 0.539 | 0.128 | 0.447 |
| sp\|Q8N6U8\|GP161_HUMAN G-protein coupled receptor 161 OS=Homo sapiens GN=GPR161 PE=2 SV=1 | 505 | FGGRRGsRTLV | 0.622 | 0.872 | -0.042 | 0.484 |
| sp\|Q8N6U8\|GP161_HUMAN G-protein coupled receptor 161 OS=Homo sapiens GN=GPR161 PE=2 SV=1 | 507 | GRRGSRtLVSQ | 0.595 | 0.684 | 0.111 | 0.463 |
| sp\|Q8N6U8\|GP161_HUMAN G-protein coupled receptor 161 OS=Homo sapiens GN=GPR161 PE=2 SV=1 | 510 | GSRTLVsQRLQ | 0.385 | -0.012 | -0.85 | -0.159 |
| sp\|Q8N6U8\|GP161_HUMAN G-protein coupled receptor 161 OS=Homo sapiens GN=GPR161 PE=2 SV=1 | 517 | QRLQLQsIEEG | 0.183 | 0.112 | -0.606 | -0.104 |
| sp\|Q16538\|GP162_HUMAN Probable G-protein coupled receptor 162 OS=Homo sapiens GN=GPR162 PE=2 S | 313 | LAVLWCsMAQT | 0.151 | -0.061 | -1.097 | -0.336 |
| sp\|Q16538\|GP162_HUMAN Probable G-protein coupled receptor 162 OS=Homo sapiens GN=GPR162 PE=2 S | 317 | WCSMAQtLLLP | 0.311 | 0.017 | -0.474 | -0.049 |
| sp\|Q16538\|GP162_HUMAN Probable G-protein coupled receptor 162 OS=Homo sapiens GN=GPR162 PE=2 S | 322 | QTLLLPsFIWS | 0.112 | -0.127 | -0.964 | -0.326 |
| sp\|Q16538\|GP162_HUMAN Probable G-protein coupled receptor 162 OS=Homo sapiens GN=GPR162 PE=2 S | 326 | LPSFIWsCERY | 0.212 | -0.095 | -0.932 | -0.272 |
| sp\|Q16538\|GP162_HUMAN Probable G-protein coupled receptor 162 OS=Homo sapiens GN=GPR162 PE=2 S | 336 | YRADVRtVWEQ | 0.299 | 0.221 | -0.766 | -0.082 |
| sp\|Q16538\|GP162_HUMAN Probable G-protein coupled receptor 162 OS=Homo sapiens GN=GPR162 PE=2 S | 346 | QCVAIMsEEDG | 0.426 | 0.23 | -0.253 | 0.134 |
| sp\|Q16538\|GP162_HUMAN Probable G-protein coupled receptor 162 OS=Homo sapiens GN=GPR162 PE=2 S | 375 | FDANGAtGPGS | 0.26 | 0.285 | -0.254 | 0.097 |
| sp\|Q16538\|GP162_HUMAN Probable G-protein coupled receptor 162 OS=Homo sapiens GN=GPR162 PE=2 S | 379 | GATGPGsRDPA | 0.104 | -0.33 | -1.109 | -0.445 |
| sp\|Q16538\|GP162_HUMAN Probable G-protein coupled receptor 162 OS=Homo sapiens GN=GPR162 PE=2 S | 409 | YLQVPLsRRLS | 0.081 | -0.186 | -1.131 | -0.412 |
| sp\|Q16538\|GP162_HUMAN Probable G-protein coupled receptor 162 OS=Homo sapiens GN=GPR162 PE=2 S | 413 | PLSRRLsHDET | 0.846 | 1.065 | 0.901 | 0.937 |
| sp\|Q16538\|GP162_HUMAN Probable G-protein coupled receptor 162 OS=Homo sapiens GN=GPR162 PE=2 S | 417 | RLSHDEtNIFS | 0.097 | -0.185 | -1.317 | -0.468 |
| sp\|Q16538\|GP162_HUMAN Probable G-protein coupled receptor 162 OS=Homo sapiens GN=GPR162 PE=2 S | 421 | DETNIFsTPRE | 0.499 | 0.34 | -0.164 | 0.225 |
| sp\|Q16538\|GP162_HUMAN Probable G-protein coupled receptor 162 OS=Homo sapiens GN=GPR162 PE=2 S | 422 | ETNIFStPREP | 0.034 | -0.563 | -1.928 | -0.819 |
| sp\|Q16538\|GP162_HUMAN Probable G-protein coupled receptor 162 OS=Homo sapiens GN=GPR162 PE=2 S | 428 | TPREPGsFLHK | 0.088 | -0.112 | -1.073 | -0.366 |
| sp\|Q16538\|GP162_HUMAN Probable G-protein coupled receptor 162 OS=Homo sapiens GN=GPR162 PE=2 S | 434 | SFLHKWsSSDD | 0.128 | -0.202 | -0.999 | -0.358 |
| sp\|Q16538\|GP162_HUMAN Probable G-protein coupled receptor 162 OS=Homo sapiens GN=GPR162 PE=2 S | 435 | FLHKWSsSDDI | 0.557 | 0.257 | -0.02 | 0.265 |
| sp\|Q16538\|GP162_HUMAN Probable G-protein coupled receptor 162 OS=Homo sapiens GN=GPR162 PE=2 S | 436 | LHKWSSsDDIR | 0.498 | 0.19 | -0.222 | 0.155 |
| sp\|Q16538\|GP162_HUMAN Probable G-protein coupled receptor 162 OS=Homo sapiens GN=GPR162 PE=2 S | 446 | RVLPAQsRALG | 0.172 | -0.078 | -0.828 | -0.245 |
| sp\|Q16538\|GP162_HUMAN Probable G-protein coupled receptor 162 OS=Homo sapiens GN=GPR162 PE=2 S | 478 | EGGGLAsLRQF | 0.148 | -0.118 | -0.653 | -0.208 |
| sp\|Q16538\|GP162_HUMAN Probable G-protein coupled receptor 162 OS=Homo sapiens GN=GPR162 PE=2 S | 485 | LRQFLEsGVLG | 0.149 | 0.134 | -0.971 | -0.229 |
| sp\|Q16538\|GP162_HUMAN Probable G-protein coupled receptor 162 OS=Homo sapiens GN=GPR162 PE=2 S | 490 | ESGVLGsGGGP | 0.028 | -0.407 | -1.685 | -0.688 |
| sp\|Q16538\|GP162_HUMAN Probable G-protein coupled receptor 162 OS=Homo sapiens GN=GPR162 PE=2 S | 506 | FFREEItTFID | 0.522 | 0.27 | -0.224 | 0.189 |
| sp\|Q16538\|GP162_HUMAN Probable G-protein coupled receptor 162 OS=Homo sapiens GN=GPR162 PE=2 S | 507 | FREEITtFIDE | 0.343 | 0.331 | -0.067 | 0.202 |
| sp\|Q16538\|GP162_HUMAN Probable G-protein coupled receptor 162 OS=Homo sapiens GN=GPR162 PE=2 S | 512 | TTFIDEtPLPS | 0.03 | -0.555 | -2.063 | -0.863 |
| sp\|Q16538\|GP162_HUMAN Probable G-protein coupled receptor 162 OS=Homo sapiens GN=GPR162 PE=2 S | 516 | DETPLPsPTAS | 0.071 | -0.493 | -1.6 | -0.674 |
| sp\|Q16538\|GP162_HUMAN Probable G-protein coupled receptor 162 OS=Homo sapiens GN=GPR162 PE=2 S | 518 | TPLPSPtASPG | 0.141 | -0.084 | -0.921 | -0.288 |
| sp\|Q16538\|GP162_HUMAN Probable G-protein coupled receptor 162 OS=Homo sapiens GN=GPR162 PE=2 S | 520 | LPSPTAsPGHS | 0.045 | -0.435 | -1.402 | -0.597 |
| sp\|Q16538\|GP162_HUMAN Probable G-protein coupled receptor 162 OS=Homo sapiens GN=GPR162 PE=2 S | 524 | TASPGHsPRRP | 0.04 | -0.631 | -1.825 | -0.805 |
| sp\|Q16538\|GP162_HUMAN Probable G-protein coupled receptor 162 OS=Homo sapiens GN=GPR162 PE=2 S | 534 | PRPLGLsPRRL | 0.042 | -0.348 | -1.676 | -0.661 |
| sp\|Q16538\|GP162_HUMAN Probable G-protein coupled receptor 162 OS=Homo sapiens GN=GPR162 PE=2 S | 539 | LSPRRLsLGSP | 0.534 | 0.857 | 0.349 | 0.58 |
| sp\|Q16538\|GP162_HUMAN Probable G-protein coupled receptor 162 OS=Homo sapiens GN=GPR162 PE=2 S | 542 | RRLSLGsPESR | 0.036 | -0.302 | -1.707 | -0.658 |
| sp\|Q16538\|GP162_HUMAN Probable G-protein coupled receptor 162 OS=Homo sapiens GN=GPR162 PE=2 S | 545 | SLGSPEsRAVG | 0.062 | -0.324 | -1.616 | -0.626 |
| sp\|Q16538\|GP162_HUMAN Probable G-protein coupled receptor 162 OS=Homo sapiens GN=GPR162 PE=2 S | 555 | GLPLGLsAGRR | 0.196 | -0.047 | -1.091 | -0.314 |
| sp\|Q16538\|GP162_HUMAN Probable G-protein coupled receptor 162 OS=Homo sapiens GN=GPR162 PE=2 S | 561 | SAGRRCsLTGG | 0.825 | 0.928 | 0.514 | 0.756 |
| sp\|Q16538\|GP162_HUMAN Probable G-protein coupled receptor 162 OS=Homo sapiens GN=GPR162 PE=2 S | 563 | GRRCSLtGGEE | 0.757 | 0.63 | 0.265 | 0.551 |
| sp\|Q16538\|GP162_HUMAN Probable G-protein coupled receptor 162 OS=Homo sapiens GN=GPR162 PE=2 S | 568 | LTGGEEsARAW | 0.114 | -0.12 | -1.227 | -0.411 |
| sp\|Q16538\|GP162_HUMAN Probable G-protein coupled receptor 162 OS=Homo sapiens GN=GPR162 PE=2 S | 575 | ARAWGGsWGPG | 0.155 | 0.057 | -0.751 | -0.18 |
| sp\|Q16538\|GP162_HUMAN Probable G-protein coupled receptor 162 OS=Homo sapiens GN=GPR162 PE=2 S | 587 | PIFPQLtL--- | 0.219 | 0.218 | -0.633 | -0.065 |

| | | | | | | |
|---|---|---|---|---|---|---|
| sp\|Q9NS66\|GP173_HUMAN Probable G-protein coupled receptor 173 OS=Homo sapiens GN=GPR173 PE=2 S | 324 | PHRYLAtAVWM | 0.369 | 0.291 | -0.605 | 0.018 |
| sp\|Q9NS66\|GP173_HUMAN Probable G-protein coupled receptor 173 OS=Homo sapiens GN=GPR173 PE=2 S | 329 | ATAVWMsFAQA | 0.308 | 0.026 | -0.433 | -0.033 |
| sp\|Q9NS66\|GP173_HUMAN Probable G-protein coupled receptor 173 OS=Homo sapiens GN=GPR173 PE=2 S | 353 | LKKCLRtHAPC | 0.179 | -0.176 | -1.153 | -0.383 |
| sp\|Q9NS66\|GP173_HUMAN Probable G-protein coupled receptor 173 OS=Homo sapiens GN=GPR173 PE=2 S | 360 | HAPCWGtGGAP | 0.122 | -0.126 | -0.979 | -0.328 |
| sp\|Q9BXC1\|GP174_HUMAN Probable G-protein coupled receptor 174 OS=Homo sapiens GN=GPR174 PE=2 S | 311 | RQDLHDsIQLH | 0.11 | -0.21 | -1.311 | -0.47 |
| sp\|Q9BXC1\|GP174_HUMAN Probable G-protein coupled receptor 174 OS=Homo sapiens GN=GPR174 PE=2 S | 318 | IQLHAKsFVSN | 0.325 | 0.104 | -0.113 | 0.105 |
| sp\|Q9BXC1\|GP174_HUMAN Probable G-protein coupled receptor 174 OS=Homo sapiens GN=GPR174 PE=2 S | 321 | HAKSFVsNHTA | 0.294 | -0.129 | -0.775 | -0.203 |
| sp\|Q9BXC1\|GP174_HUMAN Probable G-protein coupled receptor 174 OS=Homo sapiens GN=GPR174 PE=2 S | 324 | SFVSNHtASTM | 0.142 | -0.067 | -1.029 | -0.318 |
| sp\|Q9BXC1\|GP174_HUMAN Probable G-protein coupled receptor 174 OS=Homo sapiens GN=GPR174 PE=2 S | 326 | VSNHTAsTMTP | 0.089 | -0.216 | -1.331 | -0.486 |
| sp\|Q9BXC1\|GP174_HUMAN Probable G-protein coupled receptor 174 OS=Homo sapiens GN=GPR174 PE=2 S | 327 | SNHTAStMTPE | 0.069 | -0.246 | -1.609 | -0.595 |
| sp\|Q9BXC1\|GP174_HUMAN Probable G-protein coupled receptor 174 OS=Homo sapiens GN=GPR174 PE=2 S | 329 | HTASTMtPELC | 0.061 | -0.254 | -1.359 | -0.517 |
| sp\|Q14439\|GP176_HUMAN G-protein coupled receptor 176 OS=Homo sapiens GN=GPR176 PE=2 SV=1 | 312 | VWLPKVsLLAN | 0.301 | 0.023 | -0.41 | -0.029 |
| sp\|Q14439\|GP176_HUMAN G-protein coupled receptor 176 OS=Homo sapiens GN=GPR176 PE=2 SV=1 | 322 | NPVLFLtVNKS | 0.078 | -0.153 | -1.362 | -0.479 |
| sp\|Q14439\|GP176_HUMAN G-protein coupled receptor 176 OS=Homo sapiens GN=GPR176 PE=2 SV=1 | 326 | FLTVNKsVRKC | 0.292 | 0.043 | -0.448 | -0.038 |
| sp\|Q14439\|GP176_HUMAN G-protein coupled receptor 176 OS=Homo sapiens GN=GPR176 PE=2 SV=1 | 334 | RKCLIGtLVQL | 0.465 | 0.128 | -0.196 | 0.132 |
| sp\|Q14439\|GP176_HUMAN G-protein coupled receptor 176 OS=Homo sapiens GN=GPR176 PE=2 SV=1 | 343 | QLHHRYsRRNV | 0.3 | 0.11 | -0.765 | -0.118 |
| sp\|Q14439\|GP176_HUMAN G-protein coupled receptor 176 OS=Homo sapiens GN=GPR176 PE=2 SV=1 | 349 | SRRNVVsTGSG | 0.415 | 0.363 | -0.318 | 0.153 |
| sp\|Q14439\|GP176_HUMAN G-protein coupled receptor 176 OS=Homo sapiens GN=GPR176 PE=2 SV=1 | 350 | RRNVVStGSGM | 0.074 | -0.067 | -1.301 | -0.431 |
| sp\|Q14439\|GP176_HUMAN G-protein coupled receptor 176 OS=Homo sapiens GN=GPR176 PE=2 SV=1 | 352 | NVVSTGsGMAE | 0.069 | -0.158 | -1.268 | -0.452 |
| sp\|Q14439\|GP176_HUMAN G-protein coupled receptor 176 OS=Homo sapiens GN=GPR176 PE=2 SV=1 | 358 | SGMAEAsLEPS | 0.156 | -0.111 | -0.97 | -0.308 |
| sp\|Q14439\|GP176_HUMAN G-protein coupled receptor 176 OS=Homo sapiens GN=GPR176 PE=2 SV=1 | 362 | EASLEPsIRSG | 0.053 | -0.382 | -1.525 | -0.618 |
| sp\|Q14439\|GP176_HUMAN G-protein coupled receptor 176 OS=Homo sapiens GN=GPR176 PE=2 SV=1 | 365 | LEPSIRsGSQL | 0.042 | -0.305 | -1.497 | -0.587 |
| sp\|Q14439\|GP176_HUMAN G-protein coupled receptor 176 OS=Homo sapiens GN=GPR176 PE=2 SV=1 | 367 | PSIRSGsQLLE | 0.532 | 0.863 | -0.039 | 0.452 |
| sp\|Q14439\|GP176_HUMAN G-protein coupled receptor 176 OS=Homo sapiens GN=GPR176 PE=2 SV=1 | 384 | QQIFKPtEDEE | 0.167 | -0.038 | -0.774 | -0.215 |
| sp\|Q14439\|GP176_HUMAN G-protein coupled receptor 176 OS=Homo sapiens GN=GPR176 PE=2 SV=1 | 390 | TEDEEEsEAKY | 0.065 | -0.211 | -1.786 | -0.644 |
| sp\|Q14439\|GP176_HUMAN G-protein coupled receptor 176 OS=Homo sapiens GN=GPR176 PE=2 SV=1 | 397 | EAKYIGsADFQ | 0.103 | -0.15 | -0.991 | -0.346 |
| sp\|Q14439\|GP176_HUMAN G-protein coupled receptor 176 OS=Homo sapiens GN=GPR176 PE=2 SV=1 | 407 | QAKEIFsTCLE | 0.086 | -0.182 | -1.042 | -0.379 |
| sp\|Q14439\|GP176_HUMAN G-protein coupled receptor 176 OS=Homo sapiens GN=GPR176 PE=2 SV=1 | 408 | AKEIFStCLEG | 0.216 | -0.012 | -0.998 | -0.265 |
| sp\|Q14439\|GP176_HUMAN G-protein coupled receptor 176 OS=Homo sapiens GN=GPR176 PE=2 SV=1 | 421 | GPQFAPsAPPL | 0.711 | 0.586 | 0.093 | 0.463 |
| sp\|Q14439\|GP176_HUMAN G-protein coupled receptor 176 OS=Homo sapiens GN=GPR176 PE=2 SV=1 | 426 | PSAPPLsTVDS | 0.082 | -0.244 | -1.018 | -0.393 |
| sp\|Q14439\|GP176_HUMAN G-protein coupled receptor 176 OS=Homo sapiens GN=GPR176 PE=2 SV=1 | 427 | SAPPLStVDSV | 0.028 | -0.369 | -2.038 | -0.793 |
| sp\|Q14439\|GP176_HUMAN G-protein coupled receptor 176 OS=Homo sapiens GN=GPR176 PE=2 SV=1 | 430 | PLSTVDsVSQV | 0.229 | -0.126 | -1.121 | -0.339 |
| sp\|Q14439\|GP176_HUMAN G-protein coupled receptor 176 OS=Homo sapiens GN=GPR176 PE=2 SV=1 | 432 | STVDSVsQVAP | 0.28 | 0.069 | -0.864 | -0.172 |
| sp\|Q14439\|GP176_HUMAN G-protein coupled receptor 176 OS=Homo sapiens GN=GPR176 PE=2 SV=1 | 444 | APVEPEtFPDK | 0.441 | 0.314 | -0.315 | 0.147 |
| sp\|Q14439\|GP176_HUMAN G-protein coupled receptor 176 OS=Homo sapiens GN=GPR176 PE=2 SV=1 | 450 | TFPDKYsLQFG | 0.166 | -0.023 | -0.649 | -0.169 |
| sp\|Q14439\|GP176_HUMAN G-protein coupled receptor 176 OS=Homo sapiens GN=GPR176 PE=2 SV=1 | 466 | LPPQWLsETRN | 0.158 | -0.135 | -1.053 | -0.343 |
| sp\|Q14439\|GP176_HUMAN G-protein coupled receptor 176 OS=Homo sapiens GN=GPR176 PE=2 SV=1 | 468 | PQWLSEtRNSK | 0.153 | 0.101 | -0.732 | -0.159 |
| sp\|Q14439\|GP176_HUMAN G-protein coupled receptor 176 OS=Homo sapiens GN=GPR176 PE=2 SV=1 | 471 | LSETRNsKKRL | 0.342 | -0.001 | -0.554 | -0.071 |
| sp\|Q14439\|GP176_HUMAN G-protein coupled receptor 176 OS=Homo sapiens GN=GPR176 PE=2 SV=1 | 482 | LPPLGNtPEEL | 0.045 | -0.404 | -1.666 | -0.675 |
| sp\|Q14439\|GP176_HUMAN G-protein coupled receptor 176 OS=Homo sapiens GN=GPR176 PE=2 SV=1 | 489 | PEELIQtKVPK | 0.151 | -0.153 | -0.806 | -0.269 |
| sp\|Q14439\|GP176_HUMAN G-protein coupled receptor 176 OS=Homo sapiens GN=GPR176 PE=2 SV=1 | 502 | RVERKMsRNNK | 0.639 | 0.892 | 0.27 | 0.6 |
| sp\|Q14439\|GP176_HUMAN G-protein coupled receptor 176 OS=Homo sapiens GN=GPR176 PE=2 SV=1 | 508 | SRNNKVsIFPK | 0.102 | -0.079 | -0.986 | -0.321 |
| sp\|Q14439\|GP176_HUMAN G-protein coupled receptor 176 OS=Homo sapiens GN=GPR176 PE=2 SV=1 | 515 | IFPKVDs---- | 0.312 | 0.032 | -0.719 | -0.125 |
| sp\|O15218\|GP182_HUMAN G-protein coupled receptor 182 OS=Homo sapiens GN=GPR182 PE=2 SV=1 | 309 | DVIDCFsMLHC | 0.134 | 0.002 | -1.295 | -0.386 |
| sp\|O15218\|GP182_HUMAN G-protein coupled receptor 182 OS=Homo sapiens GN=GPR182 PE=2 SV=1 | 324 | ILYNFLsPHFR | 0.074 | -0.358 | -1.197 | -0.494 |
| sp\|O15218\|GP182_HUMAN G-protein coupled receptor 182 OS=Homo sapiens GN=GPR182 PE=2 SV=1 | 344 | YLPKDQtKAGT | 0.221 | -0.116 | -0.568 | -0.154 |
| sp\|O15218\|GP182_HUMAN G-protein coupled receptor 182 OS=Homo sapiens GN=GPR182 PE=2 SV=1 | 348 | DQTKAGtCASS | 0.16 | -0.057 | -1.005 | -0.301 |
| sp\|O15218\|GP182_HUMAN G-protein coupled receptor 182 OS=Homo sapiens GN=GPR182 PE=2 SV=1 | 351 | KAGTCAsSSSC | 0.055 | -0.328 | -1.803 | -0.692 |
| sp\|O15218\|GP182_HUMAN G-protein coupled receptor 182 OS=Homo sapiens GN=GPR182 PE=2 SV=1 | 352 | AGTCASsSSCS | 0.103 | -0.168 | -1.278 | -0.448 |

| | | | | | | |
|---|---|---|---|---|---|---|
| sp\|O15218\|GP182_HUMAN G-protein coupled receptor 182 OS=Homo sapiens GN=GPR182 PE=2 SV=1 | 353 | GTCASSsSCST | 0.124 | -0.125 | -1.142 | -0.381 |
| sp\|O15218\|GP182_HUMAN G-protein coupled receptor 182 OS=Homo sapiens GN=GPR182 PE=2 SV=1 | 354 | TCASSSsCSTQ | 0.154 | 0.011 | -0.961 | -0.265 |
| sp\|O15218\|GP182_HUMAN G-protein coupled receptor 182 OS=Homo sapiens GN=GPR182 PE=2 SV=1 | 356 | ASSSSCsTQHS | 0.222 | -0.08 | -0.826 | -0.228 |
| sp\|O15218\|GP182_HUMAN G-protein coupled receptor 182 OS=Homo sapiens GN=GPR182 PE=2 SV=1 | 357 | SSSSCStQHSI | 0.084 | -0.314 | -1.498 | -0.576 |
| sp\|O15218\|GP182_HUMAN G-protein coupled receptor 182 OS=Homo sapiens GN=GPR182 PE=2 SV=1 | 360 | SCSTQHsIIIT | 0.116 | -0.084 | -1.236 | -0.401 |
| sp\|O15218\|GP182_HUMAN G-protein coupled receptor 182 OS=Homo sapiens GN=GPR182 PE=2 SV=1 | 364 | QHSIIItKGDS | 0.229 | -0.009 | -0.688 | -0.156 |
| sp\|O15218\|GP182_HUMAN G-protein coupled receptor 182 OS=Homo sapiens GN=GPR182 PE=2 SV=1 | 368 | IITKGDsQPAA | 0.661 | 0.446 | 0.144 | 0.417 |
| sp\|O15218\|GP182_HUMAN G-protein coupled receptor 182 OS=Homo sapiens GN=GPR182 PE=2 SV=1 | 380 | APHPEPsLSFQ | 0.059 | -0.405 | -1.668 | -0.671 |
| sp\|O15218\|GP182_HUMAN G-protein coupled receptor 182 OS=Homo sapiens GN=GPR182 PE=2 SV=1 | 382 | HPEPSLsFQAH | 0.264 | 0.13 | -0.294 | 0.033 |
| sp\|O15218\|GP182_HUMAN G-protein coupled receptor 182 OS=Homo sapiens GN=GPR182 PE=2 SV=1 | 392 | HHLLPNtSPIS | 0.436 | 0.406 | -0.445 | 0.132 |
| sp\|O15218\|GP182_HUMAN G-protein coupled receptor 182 OS=Homo sapiens GN=GPR182 PE=2 SV=1 | 393 | HLLPNTsPISP | 0.046 | -0.34 | -1.044 | -0.446 |
| sp\|O15218\|GP182_HUMAN G-protein coupled receptor 182 OS=Homo sapiens GN=GPR182 PE=2 SV=1 | 396 | PNTSPIsPTQP | 0.057 | -0.632 | -1.342 | -0.639 |
| sp\|O15218\|GP182_HUMAN G-protein coupled receptor 182 OS=Homo sapiens GN=GPR182 PE=2 SV=1 | 398 | TSPISPtQPLT | 0.388 | 0.391 | -0.315 | 0.155 |
| sp\|O15218\|GP182_HUMAN G-protein coupled receptor 182 OS=Homo sapiens GN=GPR182 PE=2 SV=1 | 402 | SPTQPLtPS-- | 0.024 | -0.48 | -1.795 | -0.75 |
| sp\|O15218\|GP182_HUMAN G-protein coupled receptor 182 OS=Homo sapiens GN=GPR182 PE=2 SV=1 | 404 | TQPLTPs---- | 0.061 | -0.043 | -1.27 | -0.417 |
| sp\|P32249\|GP183_HUMAN G-protein coupled receptor 183 OS=Homo sapiens GN=GPR183 PE=1 SV=3 | 293 | QISLHFtVCLM | 0.195 | 0.011 | -0.757 | -0.184 |
| sp\|P32249\|GP183_HUMAN G-protein coupled receptor 183 OS=Homo sapiens GN=GPR183 PE=1 SV=3 | 328 | MLKRQVsVSIS | 0.803 | 0.985 | 0.607 | 0.798 |
| sp\|P32249\|GP183_HUMAN G-protein coupled receptor 183 OS=Homo sapiens GN=GPR183 PE=1 SV=3 | 330 | KRQVSVsISSA | 0.197 | 0.235 | -0.558 | -0.042 |
| sp\|P32249\|GP183_HUMAN G-protein coupled receptor 183 OS=Homo sapiens GN=GPR183 PE=1 SV=3 | 332 | QVSVSIsSAVK | 0.133 | -0.009 | -0.833 | -0.236 |
| sp\|P32249\|GP183_HUMAN G-protein coupled receptor 183 OS=Homo sapiens GN=GPR183 PE=1 SV=3 | 333 | VSVSISsAVKS | 0.05 | -0.283 | -1.562 | -0.598 |
| sp\|P32249\|GP183_HUMAN G-protein coupled receptor 183 OS=Homo sapiens GN=GPR183 PE=1 SV=3 | 337 | ISSAVKsAPEE | 0.732 | 0.412 | 0.206 | 0.45 |
| sp\|P32249\|GP183_HUMAN G-protein coupled receptor 183 OS=Homo sapiens GN=GPR183 PE=1 SV=3 | 343 | SAPEENsREMT | 0.042 | -0.43 | -2.127 | -0.838 |
| sp\|P32249\|GP183_HUMAN G-protein coupled receptor 183 OS=Homo sapiens GN=GPR183 PE=1 SV=3 | 347 | ENSREMtETQM | 0.661 | 0.831 | 0.399 | 0.63 |
| sp\|P32249\|GP183_HUMAN G-protein coupled receptor 183 OS=Homo sapiens GN=GPR183 PE=1 SV=3 | 349 | SREMTEtQMMI | 0.134 | -0.004 | -1.236 | -0.369 |
| sp\|P32249\|GP183_HUMAN G-protein coupled receptor 183 OS=Homo sapiens GN=GPR183 PE=1 SV=3 | 355 | TQMMIHsKSSN | 0.082 | -0.056 | -0.874 | -0.283 |
| sp\|P32249\|GP183_HUMAN G-protein coupled receptor 183 OS=Homo sapiens GN=GPR183 PE=1 SV=3 | 357 | MMIHSKsSNGK | 0.466 | 0.336 | 0.11 | 0.304 |
| sp\|P32249\|GP183_HUMAN G-protein coupled receptor 183 OS=Homo sapiens GN=GPR183 PE=1 SV=3 | 358 | MIHSKSsNGK- | 0.085 | -0.089 | -1.398 | -0.467 |
| sp\|Q9BXB1\|LGR4_HUMAN Leucine-rich repeat-containing G-protein coupled receptor 4 OS=Homo sapiens GI | 820 | LLKRRVtKKSG | 0.86 | 1.065 | 1.105 | 1.01 |
| sp\|Q9BXB1\|LGR4_HUMAN Leucine-rich repeat-containing G-protein coupled receptor 4 OS=Homo sapiens GI | 823 | RRVTKKsGSVS | 0.125 | 0.1 | -0.966 | -0.247 |
| sp\|Q9BXB1\|LGR4_HUMAN Leucine-rich repeat-containing G-protein coupled receptor 4 OS=Homo sapiens GI | 825 | VTKKSGsVSVS | 0.318 | 0.094 | -0.548 | -0.045 |
| sp\|Q9BXB1\|LGR4_HUMAN Leucine-rich repeat-containing G-protein coupled receptor 4 OS=Homo sapiens GI | 827 | KKSGSVsVSIS | 0.456 | 0.127 | -0.233 | 0.117 |
| sp\|Q9BXB1\|LGR4_HUMAN Leucine-rich repeat-containing G-protein coupled receptor 4 OS=Homo sapiens GI | 829 | SGSVSVsISSQ | 0.083 | -0.149 | -1.152 | -0.406 |
| sp\|Q9BXB1\|LGR4_HUMAN Leucine-rich repeat-containing G-protein coupled receptor 4 OS=Homo sapiens GI | 831 | SVSVSIsSQGG | 0.172 | 0.002 | -0.739 | -0.188 |
| sp\|Q9BXB1\|LGR4_HUMAN Leucine-rich repeat-containing G-protein coupled receptor 4 OS=Homo sapiens GI | 832 | VSVSISsQGGC | 0.071 | -0.392 | -1.486 | -0.602 |
| sp\|Q9BXB1\|LGR4_HUMAN Leucine-rich repeat-containing G-protein coupled receptor 4 OS=Homo sapiens GI | 849 | YDCGMYsHLQG | 0.133 | -0.133 | -0.79 | -0.263 |
| sp\|Q9BXB1\|LGR4_HUMAN Leucine-rich repeat-containing G-protein coupled receptor 4 OS=Homo sapiens GI | 856 | HLQGNLtVCDC | 0.198 | 0.037 | -0.657 | -0.141 |
| sp\|Q9BXB1\|LGR4_HUMAN Leucine-rich repeat-containing G-protein coupled receptor 4 OS=Homo sapiens GI | 863 | VCDCCEsFLLT | 0.261 | -0.072 | -0.998 | -0.27 |
| sp\|Q9BXB1\|LGR4_HUMAN Leucine-rich repeat-containing G-protein coupled receptor 4 OS=Homo sapiens GI | 867 | CESFLLtKPVS | 0.345 | 0.373 | -0.42 | 0.099 |
| sp\|Q9BXB1\|LGR4_HUMAN Leucine-rich repeat-containing G-protein coupled receptor 4 OS=Homo sapiens GI | 871 | LLTKPVsCKHL | 0.417 | 0.086 | -0.019 | 0.161 |
| sp\|Q9BXB1\|LGR4_HUMAN Leucine-rich repeat-containing G-protein coupled receptor 4 OS=Homo sapiens GI | 878 | CKHLIKsHSCP | 0.243 | 0.033 | -0.493 | -0.072 |
| sp\|Q9BXB1\|LGR4_HUMAN Leucine-rich repeat-containing G-protein coupled receptor 4 OS=Homo sapiens GI | 880 | HLIKSHsCPAL | 0.939 | 1.251 | 1.5 | 1.23 |
| sp\|Q9BXB1\|LGR4_HUMAN Leucine-rich repeat-containing G-protein coupled receptor 4 OS=Homo sapiens GI | 888 | PALAVAsCQRP | 0.241 | -0.113 | -0.98 | -0.284 |
| sp\|Q9BXB1\|LGR4_HUMAN Leucine-rich repeat-containing G-protein coupled receptor 4 OS=Homo sapiens GI | 897 | RPEGYWsDCGT | 0.174 | -0.233 | -0.997 | -0.352 |
| sp\|Q9BXB1\|LGR4_HUMAN Leucine-rich repeat-containing G-protein coupled receptor 4 OS=Homo sapiens GI | 901 | YWSDCGtQSAH | 0.087 | -0.234 | -1.509 | -0.552 |
| sp\|Q9BXB1\|LGR4_HUMAN Leucine-rich repeat-containing G-protein coupled receptor 4 OS=Homo sapiens GI | 903 | SDCGTQsAHSD | 0.062 | -0.103 | -1.388 | -0.476 |
| sp\|Q9BXB1\|LGR4_HUMAN Leucine-rich repeat-containing G-protein coupled receptor 4 OS=Homo sapiens GI | 906 | GTQSAHsDYAD | 0.654 | 0.331 | -0.12 | 0.288 |
| sp\|Q9BXB1\|LGR4_HUMAN Leucine-rich repeat-containing G-protein coupled receptor 4 OS=Homo sapiens GI | 914 | YADEEDsFVSD | 0.084 | -0.205 | -1.417 | -0.513 |
| sp\|Q9BXB1\|LGR4_HUMAN Leucine-rich repeat-containing G-protein coupled receptor 4 OS=Homo sapiens GI | 917 | EEDSFVsDSSD | 0.102 | -0.23 | -1.427 | -0.518 |
| sp\|Q9BXB1\|LGR4_HUMAN Leucine-rich repeat-containing G-protein coupled receptor 4 OS=Homo sapiens GI | 919 | DSFVSDsSDQV | 0.288 | -0.005 | -0.939 | -0.219 |

| | | | | | | |
|---|---|---|---|---|---|---|
| sp\|Q9BXB1\|LGR4_HUMAN Leucine-rich repeat-containing G-protein coupled receptor 4 OS=Homo sapiens GI | 920 | SFVSDSsDQVQ | 0.08 | -0.212 | -1.447 | -0.526 |
| sp\|Q9BXB1\|LGR4_HUMAN Leucine-rich repeat-containing G-protein coupled receptor 4 OS=Homo sapiens GI | 934 | RACFYQsRGFP | 0.177 | -0.155 | -1.078 | -0.352 |
| sp\|O75473\|LGR5_HUMAN Leucine-rich repeat-containing G-protein coupled receptor 5 OS=Homo sapiens GI | 833 | FKEDLVsLRKQ | 0.556 | 0.043 | -0.31 | 0.096 |
| sp\|O75473\|LGR5_HUMAN Leucine-rich repeat-containing G-protein coupled receptor 5 OS=Homo sapiens GI | 838 | VSLRKQtYVWT | 0.278 | 0.62 | -0.066 | 0.277 |
| sp\|O75473\|LGR5_HUMAN Leucine-rich repeat-containing G-protein coupled receptor 5 OS=Homo sapiens GI | 842 | KQTYVWtRSKH | 0.059 | -0.386 | -1.518 | -0.615 |
| sp\|O75473\|LGR5_HUMAN Leucine-rich repeat-containing G-protein coupled receptor 5 OS=Homo sapiens GI | 844 | TYVWTRsKHPS | 0.11 | -0.103 | -1.102 | -0.365 |
| sp\|O75473\|LGR5_HUMAN Leucine-rich repeat-containing G-protein coupled receptor 5 OS=Homo sapiens GI | 848 | TRSKHPsLMSI | 0.528 | 0.289 | -0.041 | 0.259 |
| sp\|O75473\|LGR5_HUMAN Leucine-rich repeat-containing G-protein coupled receptor 5 OS=Homo sapiens GI | 851 | KHPSLMsINSD | 0.066 | -0.151 | -1.41 | -0.498 |
| sp\|O75473\|LGR5_HUMAN Leucine-rich repeat-containing G-protein coupled receptor 5 OS=Homo sapiens GI | 854 | SLMSINsDDVE | 0.304 | 0.102 | -0.669 | -0.088 |
| sp\|O75473\|LGR5_HUMAN Leucine-rich repeat-containing G-protein coupled receptor 5 OS=Homo sapiens GI | 861 | DDVEKQsCDST | 0.048 | -0.188 | -1.545 | -0.562 |
| sp\|O75473\|LGR5_HUMAN Leucine-rich repeat-containing G-protein coupled receptor 5 OS=Homo sapiens GI | 864 | EKQSCDsTQAL | 0.413 | 0.081 | -0.358 | 0.045 |
| sp\|O75473\|LGR5_HUMAN Leucine-rich repeat-containing G-protein coupled receptor 5 OS=Homo sapiens GI | 865 | KQSCDStQALV | 0.064 | -0.329 | -1.77 | -0.678 |
| sp\|O75473\|LGR5_HUMAN Leucine-rich repeat-containing G-protein coupled receptor 5 OS=Homo sapiens GI | 870 | STQALVtFTSS | 0.178 | -0.147 | -1.155 | -0.375 |
| sp\|O75473\|LGR5_HUMAN Leucine-rich repeat-containing G-protein coupled receptor 5 OS=Homo sapiens GI | 872 | QALVTFtSSSI | 0.042 | -0.154 | -1.318 | -0.477 |
| sp\|O75473\|LGR5_HUMAN Leucine-rich repeat-containing G-protein coupled receptor 5 OS=Homo sapiens GI | 873 | ALVTFTsSSIT | 0.133 | -0.178 | -1.228 | -0.424 |
| sp\|O75473\|LGR5_HUMAN Leucine-rich repeat-containing G-protein coupled receptor 5 OS=Homo sapiens GI | 874 | LVTFTSsSITY | 0.076 | -0.076 | -1.03 | -0.343 |
| sp\|O75473\|LGR5_HUMAN Leucine-rich repeat-containing G-protein coupled receptor 5 OS=Homo sapiens GI | 875 | VTFTSSsITYD | 0.276 | -0.018 | -0.729 | -0.157 |
| sp\|O75473\|LGR5_HUMAN Leucine-rich repeat-containing G-protein coupled receptor 5 OS=Homo sapiens GI | 877 | FTSSSItYDLP | 0.293 | 0.094 | -0.506 | -0.04 |
| sp\|O75473\|LGR5_HUMAN Leucine-rich repeat-containing G-protein coupled receptor 5 OS=Homo sapiens GI | 883 | TYDLPPsSVPS | 0.062 | -0.328 | -1.477 | -0.581 |
| sp\|O75473\|LGR5_HUMAN Leucine-rich repeat-containing G-protein coupled receptor 5 OS=Homo sapiens GI | 884 | YDLPPSsVPSP | 0.087 | 0.07 | -0.783 | -0.209 |
| sp\|O75473\|LGR5_HUMAN Leucine-rich repeat-containing G-protein coupled receptor 5 OS=Homo sapiens GI | 887 | PPSSVPsPAYP | 0.034 | -0.664 | -1.965 | -0.865 |
| sp\|O75473\|LGR5_HUMAN Leucine-rich repeat-containing G-protein coupled receptor 5 OS=Homo sapiens GI | 893 | SPAYPVtESCH | 0.084 | -0.236 | -1.221 | -0.458 |
| sp\|O75473\|LGR5_HUMAN Leucine-rich repeat-containing G-protein coupled receptor 5 OS=Homo sapiens GI | 895 | AYPVTEsCHLS | 0.076 | -0.045 | -1.506 | -0.492 |
| sp\|O75473\|LGR5_HUMAN Leucine-rich repeat-containing G-protein coupled receptor 5 OS=Homo sapiens GI | 899 | TESCHLsSVAF | 0.104 | -0.116 | -1.201 | -0.404 |
| sp\|O75473\|LGR5_HUMAN Leucine-rich repeat-containing G-protein coupled receptor 5 OS=Homo sapiens GI | 900 | ESCHLSsVAFV | 0.042 | -0.36 | -1.809 | -0.709 |
| sp\|Q9HBX8\|LGR6_HUMAN Leucine-rich repeat-containing G-protein coupled receptor 6 OS=Homo sapiens G | 848 | RPRAGDsGPLA | 0.576 | 0.469 | -0.104 | 0.314 |
| sp\|Q9HBX8\|LGR6_HUMAN Leucine-rich repeat-containing G-protein coupled receptor 6 OS=Homo sapiens G | 862 | AGELEKsSCDS | 0.183 | -0.187 | -0.987 | -0.33 |
| sp\|Q9HBX8\|LGR6_HUMAN Leucine-rich repeat-containing G-protein coupled receptor 6 OS=Homo sapiens G | 863 | GELEKSsCDST | 0.059 | -0.148 | -1.497 | -0.529 |
| sp\|Q9HBX8\|LGR6_HUMAN Leucine-rich repeat-containing G-protein coupled receptor 6 OS=Homo sapiens G | 866 | EKSSCDsTQAL | 0.35 | -0.007 | -0.46 | -0.039 |
| sp\|Q9HBX8\|LGR6_HUMAN Leucine-rich repeat-containing G-protein coupled receptor 6 OS=Homo sapiens G | 867 | KSSCDStQALV | 0.069 | -0.374 | -1.87 | -0.725 |
| sp\|Q9HBX8\|LGR6_HUMAN Leucine-rich repeat-containing G-protein coupled receptor 6 OS=Homo sapiens G | 874 | QALVAFsDVDL | 0.279 | 0.126 | -0.541 | -0.045 |
| sp\|Q9HBX8\|LGR6_HUMAN Leucine-rich repeat-containing G-protein coupled receptor 6 OS=Homo sapiens G | 883 | DLILEAsEAGR | 0.39 | 0.054 | -0.715 | -0.09 |
| sp\|Q9HBX8\|LGR6_HUMAN Leucine-rich repeat-containing G-protein coupled receptor 6 OS=Homo sapiens G | 893 | RPPGLEtYGFP | 0.045 | -0.337 | -1.785 | -0.692 |
| sp\|Q9HBX8\|LGR6_HUMAN Leucine-rich repeat-containing G-protein coupled receptor 6 OS=Homo sapiens G | 898 | ETYGFPsVTLI | 0.177 | -0.089 | -0.817 | -0.243 |
| sp\|Q9HBX8\|LGR6_HUMAN Leucine-rich repeat-containing G-protein coupled receptor 6 OS=Homo sapiens G | 900 | YGFPSVtLISC | 0.338 | 0.12 | -0.147 | 0.104 |
| sp\|Q9HBX8\|LGR6_HUMAN Leucine-rich repeat-containing G-protein coupled receptor 6 OS=Homo sapiens G | 903 | PSVTLIsCQQP | 0.145 | -0.146 | -1.076 | -0.359 |
| sp\|Q9HBX8\|LGR6_HUMAN Leucine-rich repeat-containing G-protein coupled receptor 6 OS=Homo sapiens G | 915 | APRLEGsHCVE | 0.181 | -0.084 | -1.308 | -0.404 |
| sp\|Q9HBX8\|LGR6_HUMAN Leucine-rich repeat-containing G-protein coupled receptor 6 OS=Homo sapiens G | 931 | FGNPQPsMDGE | 0.126 | -0.059 | -1.046 | -0.326 |
| sp\|Q9HBX8\|LGR6_HUMAN Leucine-rich repeat-containing G-protein coupled receptor 6 OS=Homo sapiens G | 943 | LLRAEGsTPAG | 0.79 | 0.707 | 0.425 | 0.641 |
| sp\|Q9HBX8\|LGR6_HUMAN Leucine-rich repeat-containing G-protein coupled receptor 6 OS=Homo sapiens G | 944 | LRAEGStPAGG | 0.027 | -0.371 | -1.557 | -0.634 |
| sp\|Q9HBX8\|LGR6_HUMAN Leucine-rich repeat-containing G-protein coupled receptor 6 OS=Homo sapiens G | 951 | PAGGGLsGGGG | 0.063 | -0.246 | -1.136 | -0.44 |
| sp\|Q9HBX8\|LGR6_HUMAN Leucine-rich repeat-containing G-protein coupled receptor 6 OS=Homo sapiens G | 959 | GGGFQPsGLAF | 0.1 | -0.122 | -1.147 | -0.39 |
| sp\|Q9HBX8\|LGR6_HUMAN Leucine-rich repeat-containing G-protein coupled receptor 6 OS=Homo sapiens G | 965 | SGLAFAsHV-- | 0.175 | 0.004 | -1.008 | -0.276 |
| sp\|P04201\|MAS_HUMAN Proto-oncogene Mas OS=Homo sapiens GN=MAS1 PE=1 SV=1 | 285 | IYFFVGsSKKK | 0.162 | -0.178 | -1.104 | -0.373 |
| sp\|P04201\|MAS_HUMAN Proto-oncogene Mas OS=Homo sapiens GN=MAS1 PE=1 SV=1 | 286 | YFFVGSsKKKR | 0.053 | -0.198 | -1.149 | -0.431 |
| sp\|P04201\|MAS_HUMAN Proto-oncogene Mas OS=Homo sapiens GN=MAS1 PE=1 SV=1 | 294 | KKRFKEsLKVV | 0.581 | 0.29 | -0.317 | 0.185 |
| sp\|P04201\|MAS_HUMAN Proto-oncogene Mas OS=Homo sapiens GN=MAS1 PE=1 SV=1 | 300 | SLKVVLtRAFK | 0.061 | -0.307 | -1.543 | -0.596 |
| sp\|P04201\|MAS_HUMAN Proto-oncogene Mas OS=Homo sapiens GN=MAS1 PE=1 SV=1 | 318 | QKDNCNtVTVE | 0.453 | 0.056 | -0.409 | 0.033 |
| sp\|P04201\|MAS_HUMAN Proto-oncogene Mas OS=Homo sapiens GN=MAS1 PE=1 SV=1 | 320 | DNCNTVtVETV | 0.182 | -0.113 | -1.021 | -0.317 |

| | | | | | | |
|---|---|---|---|---|---|---|
| sp\|P04201\|MAS_HUMAN Proto-oncogene Mas OS=Homo sapiens GN=MAS1 PE=1 SV=1 | 323 | NTVTVEtVV-- | 0.156 | 0.01 | -1.248 | -0.361 |
| sp\|P35410\|MAS1L_HUMAN Mas-related G-protein coupled receptor MRG OS=Homo sapiens GN=MAS1L PE= | 287 | SVAPLItDFKM | 0.083 | -0.194 | -1.209 | -0.44 |
| sp\|P35410\|MAS1L_HUMAN Mas-related G-protein coupled receptor MRG OS=Homo sapiens GN=MAS1L PE= | 294 | DFKMFVtTSYL | 0.335 | -0.115 | -0.74 | -0.173 |
| sp\|P35410\|MAS1L_HUMAN Mas-related G-protein coupled receptor MRG OS=Homo sapiens GN=MAS1L PE= | 295 | FKMFVTtSYLI | 0.436 | 0.23 | -0.213 | 0.151 |
| sp\|P35410\|MAS1L_HUMAN Mas-related G-protein coupled receptor MRG OS=Homo sapiens GN=MAS1L PE= | 296 | KMFVTTsYLIS | 0.04 | -0.055 | -1.546 | -0.52 |
| sp\|P35410\|MAS1L_HUMAN Mas-related G-protein coupled receptor MRG OS=Homo sapiens GN=MAS1L PE= | 300 | TTSYLIsLFLI | 0.403 | 0.049 | -0.21 | 0.081 |
| sp\|P35410\|MAS1L_HUMAN Mas-related G-protein coupled receptor MRG OS=Homo sapiens GN=MAS1L PE= | 307 | LFLIINsSANP | 0.115 | -0.074 | -0.966 | -0.308 |
| sp\|P35410\|MAS1L_HUMAN Mas-related G-protein coupled receptor MRG OS=Homo sapiens GN=MAS1L PE= | 308 | FLIINSsANPI | 0.349 | 0.182 | -0.346 | 0.062 |
| sp\|P35410\|MAS1L_HUMAN Mas-related G-protein coupled receptor MRG OS=Homo sapiens GN=MAS1L PE= | 319 | IYFFVGsLRKK | 0.368 | -0.075 | -0.544 | -0.084 |
| sp\|P35410\|MAS1L_HUMAN Mas-related G-protein coupled receptor MRG OS=Homo sapiens GN=MAS1L PE= | 328 | KKRLKEsLRVI | 0.662 | 0.282 | 0.029 | 0.324 |
| sp\|P35410\|MAS1L_HUMAN Mas-related G-protein coupled receptor MRG OS=Homo sapiens GN=MAS1L PE= | 360 | PMEQPHsTQHV | 0.211 | 0.066 | -0.642 | -0.122 |
| sp\|P35410\|MAS1L_HUMAN Mas-related G-protein coupled receptor MRG OS=Homo sapiens GN=MAS1L PE= | 361 | MEQPHStQHVE | 0.189 | -0.034 | -1.209 | -0.351 |
| sp\|P35410\|MAS1L_HUMAN Mas-related G-protein coupled receptor MRG OS=Homo sapiens GN=MAS1L PE= | 378 | HRVDVEt---- | 0.214 | 0.337 | -0.893 | -0.114 |
| sp\|Q8TDS7\|MRGRD_HUMAN Mas-related G-protein coupled receptor member D OS=Homo sapiens GN=MR( | 281 | IYFLVGsRRSH | 0.095 | -0.38 | -1.636 | -0.64 |
| sp\|Q8TDS7\|MRGRD_HUMAN Mas-related G-protein coupled receptor member D OS=Homo sapiens GN=MR( | 284 | LVGSRRsHRLP | 0.112 | -0.083 | -1.093 | -0.355 |
| sp\|Q8TDS7\|MRGRD_HUMAN Mas-related G-protein coupled receptor member D OS=Homo sapiens GN=MR( | 289 | RSHRLPtRSLG | 0.266 | 0.543 | -0.582 | 0.076 |
| sp\|Q8TDS7\|MRGRD_HUMAN Mas-related G-protein coupled receptor member D OS=Homo sapiens GN=MR( | 291 | HRLPTRsLGTV | 0.303 | 0.33 | -0.437 | 0.065 |
| sp\|Q8TDS7\|MRGRD_HUMAN Mas-related G-protein coupled receptor member D OS=Homo sapiens GN=MR( | 294 | PTRSLGtVLQQ | 0.159 | -0.06 | -1.153 | -0.351 |
| sp\|Q8TDS7\|MRGRD_HUMAN Mas-related G-protein coupled receptor member D OS=Homo sapiens GN=MR( | 311 | ELEGGEtPTVG | 0.075 | -0.362 | -1.343 | -0.543 |
| sp\|Q8TDS7\|MRGRD_HUMAN Mas-related G-protein coupled receptor member D OS=Homo sapiens GN=MR( | 313 | EGGETPtVGTN | 0.069 | -0.091 | -1.099 | -0.374 |
| sp\|Q8TDS7\|MRGRD_HUMAN Mas-related G-protein coupled receptor member D OS=Homo sapiens GN=MR( | 316 | ETPTVGtNEMG | 0.055 | -0.354 | -1.849 | -0.716 |
| sp\|Q96AM1\|MRGRF_HUMAN Mas-related G-protein coupled receptor member F OS=Homo sapiens GN=MR( | 299 | LAGRDKsQRLW | 0.45 | 0.705 | 0.007 | 0.387 |
| sp\|Q96AM1\|MRGRF_HUMAN Mas-related G-protein coupled receptor member F OS=Homo sapiens GN=MR( | 326 | LGEAGGsTPNT | 0.23 | 0.208 | -0.575 | -0.046 |
| sp\|Q96AM1\|MRGRF_HUMAN Mas-related G-protein coupled receptor member F OS=Homo sapiens GN=MR( | 327 | GEAGGStPNTV | 0.046 | -0.45 | -1.625 | -0.676 |
| sp\|Q96AM1\|MRGRF_HUMAN Mas-related G-protein coupled receptor member F OS=Homo sapiens GN=MR( | 330 | GGSTPNtVTME | 0.263 | -0.197 | -0.945 | -0.293 |
| sp\|Q96AM1\|MRGRF_HUMAN Mas-related G-protein coupled receptor member F OS=Homo sapiens GN=MR( | 332 | STPNTVtMEMQ | 0.062 | -0.164 | -1.534 | -0.545 |
| sp\|Q96AM1\|MRGRF_HUMAN Mas-related G-protein coupled receptor member F OS=Homo sapiens GN=MR( | 343 | CPPGNAs---- | 0.199 | 0.067 | -0.759 | -0.164 |
| sp\|Q86SM5\|MRGRG_HUMAN Mas-related G-protein coupled receptor member G OS=Homo sapiens GN=MR | 265 | KREPLRsVLRR | 0.089 | -0.059 | -1.384 | -0.451 |
| sp\|Q86SM5\|MRGRG_HUMAN Mas-related G-protein coupled receptor member G OS=Homo sapiens GN=MR | 283 | LGARGQsLPMG | 0.88 | 1.303 | 1.379 | 1.187 |
| sp\|Q96LB2\|MRGX1_HUMAN Mas-related G-protein coupled receptor member X1 OS=Homo sapiens GN=MR | 277 | IYFFVGsFRQR | 0.435 | -0.036 | -0.398 | 0 |
| sp\|Q96LB2\|MRGX1_HUMAN Mas-related G-protein coupled receptor member X1 OS=Homo sapiens GN=MR | 299 | RALQDAsEVDE | 0.399 | 0.058 | -0.445 | 0.004 |
| sp\|Q96LB2\|MRGX1_HUMAN Mas-related G-protein coupled receptor member X1 OS=Homo sapiens GN=MR | 316 | EEILELsGSRL | 0.041 | -0.292 | -1.836 | -0.696 |
| sp\|Q96LB2\|MRGX1_HUMAN Mas-related G-protein coupled receptor member X1 OS=Homo sapiens GN=MR | 318 | ILELSGsRLEQ | 0.315 | 0.036 | -0.668 | -0.106 |
| sp\|Q96LB1\|MRGX2_HUMAN Mas-related G-protein coupled receptor member X2 OS=Homo sapiens GN=MR | 284 | IYFFVGsFRKQ | 0.288 | -0.098 | -0.804 | -0.205 |
| sp\|Q96LB1\|MRGX2_HUMAN Mas-related G-protein coupled receptor member X2 OS=Homo sapiens GN=MR | 313 | IAEVDHsEGCF | 0.271 | 0.11 | -0.561 | -0.06 |
| sp\|Q96LB1\|MRGX2_HUMAN Mas-related G-protein coupled receptor member X2 OS=Homo sapiens GN=MR | 321 | GCFRQGtPEMS | 0.243 | 0.466 | -0.854 | -0.048 |
| sp\|Q96LB1\|MRGX2_HUMAN Mas-related G-protein coupled receptor member X2 OS=Homo sapiens GN=MR | 325 | QGTPEMsRSSL | 0.068 | -0.184 | -1.236 | -0.451 |
| sp\|Q96LB1\|MRGX2_HUMAN Mas-related G-protein coupled receptor member X2 OS=Homo sapiens GN=MR | 327 | TPEMSRsSLV- | 0.146 | 0.024 | -1.059 | -0.296 |
| sp\|Q96LB1\|MRGX2_HUMAN Mas-related G-protein coupled receptor member X2 OS=Homo sapiens GN=MR | 328 | PEMSRSsLV-- | 0.302 | 0.254 | -0.555 | 0 |
| sp\|Q96LB0\|MRGX3_HUMAN Mas-related G-protein coupled receptor member X3 OS=Homo sapiens GN=MR | 277 | IYFFVGsFRQR | 0.435 | -0.036 | -0.398 | 0 |
| sp\|Q96LB0\|MRGX3_HUMAN Mas-related G-protein coupled receptor member X3 OS=Homo sapiens GN=MR | 298 | QRALQDtPEVD | 0.076 | -0.176 | -1.376 | -0.492 |
| sp\|Q96LB0\|MRGX3_HUMAN Mas-related G-protein coupled receptor member X3 OS=Homo sapiens GN=MR | 312 | GWLPQEtLELS | 0.182 | 0.001 | -0.922 | -0.246 |
| sp\|Q96LB0\|MRGX3_HUMAN Mas-related G-protein coupled receptor member X3 OS=Homo sapiens GN=MR | 316 | QETLELsGSRL | 0.054 | -0.312 | -1.684 | -0.647 |
| sp\|Q96LB0\|MRGX3_HUMAN Mas-related G-protein coupled receptor member X3 OS=Homo sapiens GN=MR | 318 | TLELSGsRLEQ | 0.232 | 0.01 | -0.87 | -0.209 |
| sp\|Q96LA9\|MRGX4_HUMAN Mas-related G-protein coupled receptor member X4 OS=Homo sapiens GN=MR | 277 | IYFFVGsFRQR | 0.435 | -0.036 | -0.398 | 0 |
| sp\|Q96LA9\|MRGX4_HUMAN Mas-related G-protein coupled receptor member X4 OS=Homo sapiens GN=MR | 312 | GQLPEEsLELS | 0.186 | -0.081 | -0.873 | -0.256 |
| sp\|Q96LA9\|MRGX4_HUMAN Mas-related G-protein coupled receptor member X4 OS=Homo sapiens GN=MR | 316 | EESLELsGSRL | 0.04 | -0.347 | -1.824 | -0.71 |
| sp\|Q96LA9\|MRGX4_HUMAN Mas-related G-protein coupled receptor member X4 OS=Homo sapiens GN=MR | 318 | SLELSGsRLGP | 0.129 | -0.1 | -1.105 | -0.359 |
| sp\|Q86VZ1\|P2RY8_HUMAN P2Y purinoceptor 8 OS=Homo sapiens GN=P2RY8 PE=1 SV=1 | 297 | FVYYFAsREFQ | 0.096 | -0.125 | -1.268 | -0.432 |

| | | | | | | |
|---|---|---|---|---|---|---|
| sp\|Q86VZ1\|P2RY8_HUMAN P2Y purinoceptor 8 OS=Homo sapiens GN=P2RY8 PE=1 SV=1 | 317 | RRVPRDtLDTR | 0.613 | 0.367 | -0.002 | 0.326 |
| sp\|Q86VZ1\|P2RY8_HUMAN P2Y purinoceptor 8 OS=Homo sapiens GN=P2RY8 PE=1 SV=1 | 320 | PRDTLDtRRES | 0.133 | -0.125 | -1.385 | -0.459 |
| sp\|Q86VZ1\|P2RY8_HUMAN P2Y purinoceptor 8 OS=Homo sapiens GN=P2RY8 PE=1 SV=1 | 324 | LDTRREsLFSA | 0.653 | 0.894 | 0.591 | 0.713 |
| sp\|Q86VZ1\|P2RY8_HUMAN P2Y purinoceptor 8 OS=Homo sapiens GN=P2RY8 PE=1 SV=1 | 327 | RRESLFsARTT | 0.191 | 0.054 | -0.864 | -0.206 |
| sp\|Q86VZ1\|P2RY8_HUMAN P2Y purinoceptor 8 OS=Homo sapiens GN=P2RY8 PE=1 SV=1 | 330 | SLFSARtTSVR | 0.141 | -0.025 | -1.17 | -0.351 |
| sp\|Q86VZ1\|P2RY8_HUMAN P2Y purinoceptor 8 OS=Homo sapiens GN=P2RY8 PE=1 SV=1 | 331 | LFSARTtSVRS | 0.209 | -0.07 | -0.788 | -0.216 |
| sp\|Q86VZ1\|P2RY8_HUMAN P2Y purinoceptor 8 OS=Homo sapiens GN=P2RY8 PE=1 SV=1 | 332 | FSARTTsVRSE | 0.406 | 0.765 | 0.158 | 0.443 |
| sp\|Q86VZ1\|P2RY8_HUMAN P2Y purinoceptor 8 OS=Homo sapiens GN=P2RY8 PE=1 SV=1 | 335 | RTTSVRsEAGA | 0.173 | -0.138 | -1.021 | -0.329 |
| sp\|Q86VZ1\|P2RY8_HUMAN P2Y purinoceptor 8 OS=Homo sapiens GN=P2RY8 PE=1 SV=1 | 348 | EGMEGAtRPGL | 0.244 | 0.324 | -0.392 | 0.059 |
| sp\|Q86VZ1\|P2RY8_HUMAN P2Y purinoceptor 8 OS=Homo sapiens GN=P2RY8 PE=1 SV=1 | 357 | GLQRQEsVF-- | 0.748 | 1.137 | 0.372 | 0.752 |
| sp\|Q9P1P5\|TAAR2_HUMAN Trace amine-associated receptor 2 OS=Homo sapiens GN=TAAR2 PE=2 SV=2 | 336 | LLGKIFsSCFH | 0.103 | -0.197 | -1.051 | -0.382 |
| sp\|Q9P1P5\|TAAR2_HUMAN Trace amine-associated receptor 2 OS=Homo sapiens GN=TAAR2 PE=2 SV=2 | 337 | LGKIFSsCFHN | 0.123 | -0.053 | -0.862 | -0.264 |
| sp\|Q9P1P5\|TAAR2_HUMAN Trace amine-associated receptor 2 OS=Homo sapiens GN=TAAR2 PE=2 SV=2 | 342 | SSCFHNtILCM | 0.164 | -0.059 | -1.159 | -0.351 |
| sp\|Q9P1P5\|TAAR2_HUMAN Trace amine-associated receptor 2 OS=Homo sapiens GN=TAAR2 PE=2 SV=2 | 350 | LCMQKEsE--- | 0.202 | 0.176 | -0.806 | -0.143 |
| sp\|Q9P1P4\|TAAR3_HUMAN Putative trace amine-associated receptor 3 OS=Homo sapiens GN=TAAR3P PE=5 | 325 | AFKYIVsGKIF | 0.324 | -0.065 | -0.188 | 0.024 |
| sp\|Q9P1P4\|TAAR3_HUMAN Putative trace amine-associated receptor 3 OS=Homo sapiens GN=TAAR3P PE=5 | 330 | VSGKIFsSHSE | 0.119 | -0.162 | -0.972 | -0.338 |
| sp\|Q9P1P4\|TAAR3_HUMAN Putative trace amine-associated receptor 3 OS=Homo sapiens GN=TAAR3P PE=5 | 331 | SGKIFSsHSET | 0.076 | -0.24 | -1.33 | -0.498 |
| sp\|Q9P1P4\|TAAR3_HUMAN Putative trace amine-associated receptor 3 OS=Homo sapiens GN=TAAR3P PE=5 | 333 | KIFSSHsETAN | 0.644 | 0.473 | 0.072 | 0.396 |
| sp\|Q9P1P4\|TAAR3_HUMAN Putative trace amine-associated receptor 3 OS=Homo sapiens GN=TAAR3P PE=5 | 335 | FSSHSEtANLF | 0.301 | 0.163 | -0.475 | -0.004 |
| sp\|O14804\|TAAR5_HUMAN Trace amine-associated receptor 5 OS=Homo sapiens GN=TAAR5 PE=2 SV=2 | 319 | RKALKLtLSQK | 0.241 | -0.055 | -0.539 | -0.118 |
| sp\|O14804\|TAAR5_HUMAN Trace amine-associated receptor 5 OS=Homo sapiens GN=TAAR5 PE=2 SV=2 | 321 | ALKLTLsQKVF | 0.112 | -0.025 | -1.111 | -0.341 |
| sp\|O14804\|TAAR5_HUMAN Trace amine-associated receptor 5 OS=Homo sapiens GN=TAAR5 PE=2 SV=2 | 326 | LSQKVFsPQTR | 0.198 | -0.249 | -0.75 | -0.267 |
| sp\|O14804\|TAAR5_HUMAN Trace amine-associated receptor 5 OS=Homo sapiens GN=TAAR5 PE=2 SV=2 | 329 | KVFSPQtRTVD | 0.128 | -0.224 | -1.091 | -0.396 |
| sp\|O14804\|TAAR5_HUMAN Trace amine-associated receptor 5 OS=Homo sapiens GN=TAAR5 PE=2 SV=2 | 331 | FSPQTRtVDLY | 0.167 | 0.054 | -0.866 | -0.215 |
| sp\|Q96RI8\|TAAR6_HUMAN Trace amine-associated receptor 6 OS=Homo sapiens GN=TAAR6 PE=2 SV=1 | 327 | AIKVIVtGQVL | 0.296 | 0.056 | -0.378 | -0.009 |
| sp\|Q96RI8\|TAAR6_HUMAN Trace amine-associated receptor 6 OS=Homo sapiens GN=TAAR6 PE=2 SV=1 | 334 | GQVLKNsSATM | 0.12 | -0.155 | -1.188 | -0.408 |
| sp\|Q96RI8\|TAAR6_HUMAN Trace amine-associated receptor 6 OS=Homo sapiens GN=TAAR6 PE=2 SV=1 | 335 | QVLKNSsATMN | 0.218 | -0.082 | -0.889 | -0.251 |
| sp\|Q96RI8\|TAAR6_HUMAN Trace amine-associated receptor 6 OS=Homo sapiens GN=TAAR6 PE=2 SV=1 | 337 | LKNSSAtMNLF | 0.474 | 0.344 | -0.104 | 0.238 |
| sp\|Q96RI8\|TAAR6_HUMAN Trace amine-associated receptor 6 OS=Homo sapiens GN=TAAR6 PE=2 SV=1 | 342 | ATMNLFsEHI- | 0.209 | 0.056 | -0.834 | -0.19 |
| sp\|Q969N4\|TAAR8_HUMAN Trace amine-associated receptor 8 OS=Homo sapiens GN=TAAR8 PE=2 SV=1 | 326 | AIKLILsGDVL | 0.191 | 0.055 | -0.58 | -0.111 |
| sp\|Q969N4\|TAAR8_HUMAN Trace amine-associated receptor 8 OS=Homo sapiens GN=TAAR8 PE=2 SV=1 | 333 | GDVLKAsSSTI | 0.101 | -0.182 | -1.274 | -0.452 |
| sp\|Q969N4\|TAAR8_HUMAN Trace amine-associated receptor 8 OS=Homo sapiens GN=TAAR8 PE=2 SV=1 | 334 | DVLKASsSTIS | 0.306 | -0.067 | -0.855 | -0.205 |
| sp\|Q969N4\|TAAR8_HUMAN Trace amine-associated receptor 8 OS=Homo sapiens GN=TAAR8 PE=2 SV=1 | 335 | VLKASSsTISL | 0.23 | 0.155 | -0.31 | 0.025 |
| sp\|Q969N4\|TAAR8_HUMAN Trace amine-associated receptor 8 OS=Homo sapiens GN=TAAR8 PE=2 SV=1 | 336 | LKASSStISLF | 0.147 | 0.01 | -0.536 | -0.126 |
| sp\|Q969N4\|TAAR8_HUMAN Trace amine-associated receptor 8 OS=Homo sapiens GN=TAAR8 PE=2 SV=1 | 338 | ASSSTIsLFLE | 0.217 | -0.111 | -0.703 | -0.199 |
| sp\|Q96RI9\|TAAR9_HUMAN Trace amine-associated receptor 9 OS=Homo sapiens GN=TAAR9 PE=2 SV=1 | 327 | AIKLIVsGKVL | 0.316 | 0.047 | -0.375 | -0.004 |
| sp\|Q96RI9\|TAAR9_HUMAN Trace amine-associated receptor 9 OS=Homo sapiens GN=TAAR9 PE=2 SV=1 | 333 | SGKVLRtDSST | 0.031 | -0.297 | -1.92 | -0.729 |
| sp\|Q96RI9\|TAAR9_HUMAN Trace amine-associated receptor 9 OS=Homo sapiens GN=TAAR9 PE=2 SV=1 | 335 | KVLRTDsSTTN | 0.399 | 0.698 | -0.273 | 0.275 |
| sp\|Q96RI9\|TAAR9_HUMAN Trace amine-associated receptor 9 OS=Homo sapiens GN=TAAR9 PE=2 SV=1 | 336 | VLRTDSsTTNL | 0.364 | 0.193 | -0.649 | -0.031 |
| sp\|Q96RI9\|TAAR9_HUMAN Trace amine-associated receptor 9 OS=Homo sapiens GN=TAAR9 PE=2 SV=1 | 337 | LRTDSStTNLF | 0.271 | 0.353 | -0.248 | 0.125 |
| sp\|Q96RI9\|TAAR9_HUMAN Trace amine-associated receptor 9 OS=Homo sapiens GN=TAAR9 PE=2 SV=1 | 338 | RTDSSTtNLFS | 0.131 | -0.062 | -1.221 | -0.384 |
| sp\|Q96RI9\|TAAR9_HUMAN Trace amine-associated receptor 9 OS=Homo sapiens GN=TAAR9 PE=2 SV=1 | 342 | STTNLFsEEVE | 0.094 | -0.065 | -1.106 | -0.359 |
| sp\|Q96RI9\|TAAR9_HUMAN Trace amine-associated receptor 9 OS=Homo sapiens GN=TAAR9 PE=2 SV=1 | 347 | FSEEVEtD--- | 0.176 | 0.074 | -1.151 | -0.3 |
| sp\|P30988\|CALCR_HUMAN Calcitonin receptor OS=Homo sapiens GN=CALCR PE=1 SV=2 | 326 | NDNCWLsVETH | 0.068 | -0.292 | -1.558 | -0.594 |
| sp\|P30988\|CALCR_HUMAN Calcitonin receptor OS=Homo sapiens GN=CALCR PE=1 SV=2 | 329 | CWLSVEtHLLY | 0.225 | -0.044 | -0.973 | -0.264 |
| sp\|P30988\|CALCR_HUMAN Calcitonin receptor OS=Homo sapiens GN=CALCR PE=1 SV=2 | 359 | IVRVLVtKMRE | 0.339 | 0.032 | -0.65 | -0.093 |
| sp\|P30988\|CALCR_HUMAN Calcitonin receptor OS=Homo sapiens GN=CALCR PE=1 SV=2 | 364 | VTKMREtHEAE | 0.432 | 0.036 | -0.47 | -0.001 |
| sp\|P30988\|CALCR_HUMAN Calcitonin receptor OS=Homo sapiens GN=CALCR PE=1 SV=2 | 369 | ETHEAEsHMYL | 0.124 | -0.091 | -1.301 | -0.423 |
| sp\|P30988\|CALCR_HUMAN Calcitonin receptor OS=Homo sapiens GN=CALCR PE=1 SV=2 | 379 | LKAVKAtMILV | 0.175 | 0.11 | -0.827 | -0.181 |

| | | | | | | |
|---|---|---|---|---|---|---|
| sp\|P30988\|CALCR_HUMAN Calcitonin receptor OS=Homo sapiens GN=CALCR PE=1 SV=2 | 398 | VFPWRPsNKML | 0.192 | -0.124 | -0.815 | -0.249 |
| sp\|P30988\|CALCR_HUMAN Calcitonin receptor OS=Homo sapiens GN=CALCR PE=1 SV=2 | 412 | YDYVMHsLIHF | 0.097 | -0.067 | -0.63 | -0.2 |
| sp\|P30988\|CALCR_HUMAN Calcitonin receptor OS=Homo sapiens GN=CALCR PE=1 SV=2 | 423 | QGFFVAtIYCF | 0.172 | -0.012 | -0.841 | -0.227 |
| sp\|P30988\|CALCR_HUMAN Calcitonin receptor OS=Homo sapiens GN=CALCR PE=1 SV=2 | 434 | CNNEVQtTVKR | 0.09 | -0.157 | -1.194 | -0.42 |
| sp\|P30988\|CALCR_HUMAN Calcitonin receptor OS=Homo sapiens GN=CALCR PE=1 SV=2 | 435 | NNEVQTtVKRQ | 0.067 | -0.305 | -1.408 | -0.549 |
| sp\|P30988\|CALCR_HUMAN Calcitonin receptor OS=Homo sapiens GN=CALCR PE=1 SV=2 | 456 | RWGRRPsNRSA | 0.559 | 0.817 | 0.066 | 0.481 |
| sp\|P30988\|CALCR_HUMAN Calcitonin receptor OS=Homo sapiens GN=CALCR PE=1 SV=2 | 459 | RRPSNRsARAA | 0.228 | 0.122 | -0.787 | -0.146 |
| sp\|P30988\|CALCR_HUMAN Calcitonin receptor OS=Homo sapiens GN=CALCR PE=1 SV=2 | 493 | NNQGEEsAEII | 0.183 | -0.072 | -1.023 | -0.304 |
| sp\|P30988\|CALCR_HUMAN Calcitonin receptor OS=Homo sapiens GN=CALCR PE=1 SV=2 | 506 | NIIEQEsSA-- | 0.062 | 0.014 | -1.613 | -0.512 |
| sp\|P30988\|CALCR_HUMAN Calcitonin receptor OS=Homo sapiens GN=CALCR PE=1 SV=2 | 507 | IIEQESsA--- | 0.247 | 0.106 | -0.766 | -0.138 |
| sp\|Q16602\|CALRL_HUMAN Calcitonin gene-related peptide type 1 receptor OS=Homo sapiens GN=CALCRL P | 318 | IVRVLItKLKV | 0.163 | 0.071 | -1.026 | -0.264 |
| sp\|Q16602\|CALRL_HUMAN Calcitonin gene-related peptide type 1 receptor OS=Homo sapiens GN=CALCRL P | 323 | ITKLKVtHQAE | 0.494 | -0.013 | -0.28 | 0.067 |
| sp\|Q16602\|CALRL_HUMAN Calcitonin gene-related peptide type 1 receptor OS=Homo sapiens GN=CALCRL P | 328 | VTHQAEsNLYM | 0.121 | -0.227 | -1.419 | -0.508 |
| sp\|Q16602\|CALRL_HUMAN Calcitonin gene-related peptide type 1 receptor OS=Homo sapiens GN=CALCRL P | 338 | MKAVRAtLILV | 0.69 | 0.406 | 0.203 | 0.433 |
| sp\|Q16602\|CALRL_HUMAN Calcitonin gene-related peptide type 1 receptor OS=Homo sapiens GN=CALCRL P | 381 | FQGLLVsTIFC | 0.064 | -0.206 | -1.392 | -0.511 |
| sp\|Q16602\|CALRL_HUMAN Calcitonin gene-related peptide type 1 receptor OS=Homo sapiens GN=CALCRL P | 382 | QGLLVStIFCF | 0.076 | -0.162 | -1.091 | -0.392 |
| sp\|Q16602\|CALRL_HUMAN Calcitonin gene-related peptide type 1 receptor OS=Homo sapiens GN=CALCRL P | 409 | KIQFGNsFSNS | 0.205 | 0.101 | -0.995 | -0.23 |
| sp\|Q16602\|CALRL_HUMAN Calcitonin gene-related peptide type 1 receptor OS=Homo sapiens GN=CALCRL P | 411 | QFGNSFsNSEA | 0.19 | 0.096 | -0.472 | -0.062 |
| sp\|Q16602\|CALRL_HUMAN Calcitonin gene-related peptide type 1 receptor OS=Homo sapiens GN=CALCRL P | 413 | GNSFSNsEALR | 0.488 | 0.235 | -0.188 | 0.178 |
| sp\|Q16602\|CALRL_HUMAN Calcitonin gene-related peptide type 1 receptor OS=Homo sapiens GN=CALCRL P | 418 | NSEALRsASYT | 0.061 | -0.319 | -1.698 | -0.652 |
| sp\|Q16602\|CALRL_HUMAN Calcitonin gene-related peptide type 1 receptor OS=Homo sapiens GN=CALCRL P | 420 | EALRSAsYTVS | 0.694 | 0.975 | 0.474 | 0.714 |
| sp\|Q16602\|CALRL_HUMAN Calcitonin gene-related peptide type 1 receptor OS=Homo sapiens GN=CALCRL P | 422 | LRSASYtVSTI | 0.401 | 0.402 | 0.115 | 0.306 |
| sp\|Q16602\|CALRL_HUMAN Calcitonin gene-related peptide type 1 receptor OS=Homo sapiens GN=CALCRL P | 424 | SASYTVsTISD | 0.093 | -0.143 | -1.028 | -0.359 |
| sp\|Q16602\|CALRL_HUMAN Calcitonin gene-related peptide type 1 receptor OS=Homo sapiens GN=CALCRL P | 425 | ASYTVStISDG | 0.091 | -0.28 | -1.334 | -0.508 |
| sp\|Q16602\|CALRL_HUMAN Calcitonin gene-related peptide type 1 receptor OS=Homo sapiens GN=CALCRL P | 427 | YTVSTIsDGPG | 0.196 | -0.052 | -0.878 | -0.245 |
| sp\|Q16602\|CALRL_HUMAN Calcitonin gene-related peptide type 1 receptor OS=Homo sapiens GN=CALCRL P | 433 | SDGPGYsHDCP | 0.036 | -0.224 | -1.454 | -0.547 |
| sp\|Q16602\|CALRL_HUMAN Calcitonin gene-related peptide type 1 receptor OS=Homo sapiens GN=CALCRL P | 438 | YSHDCPsEHLN | 0.24 | -0.021 | -0.865 | -0.215 |
| sp\|Q16602\|CALRL_HUMAN Calcitonin gene-related peptide type 1 receptor OS=Homo sapiens GN=CALCRL P | 445 | EHLNGKsIHDI | 0.211 | 0.021 | -0.467 | -0.078 |
| sp\|P34998\|CRFR1_HUMAN Corticotropin-releasing factor receptor 1 OS=Homo sapiens GN=CRHR1 PE=1 SV= | 325 | IVRILMtKLRA | 0.301 | 0.179 | -0.543 | -0.021 |
| sp\|P34998\|CRFR1_HUMAN Corticotropin-releasing factor receptor 1 OS=Homo sapiens GN=CRHR1 PE=1 SV= | 330 | MTKLRAsTTSE | 0.465 | 0.134 | -0.557 | 0.014 |
| sp\|P34998\|CRFR1_HUMAN Corticotropin-releasing factor receptor 1 OS=Homo sapiens GN=CRHR1 PE=1 SV= | 331 | TKLRAStTSET | 0.595 | 0.783 | 0.266 | 0.548 |
| sp\|P34998\|CRFR1_HUMAN Corticotropin-releasing factor receptor 1 OS=Homo sapiens GN=CRHR1 PE=1 SV= | 332 | KLRASTtSETI | 0.688 | 0.45 | 0.329 | 0.489 |
| sp\|P34998\|CRFR1_HUMAN Corticotropin-releasing factor receptor 1 OS=Homo sapiens GN=CRHR1 PE=1 SV= | 333 | LRASTTsETIQ | 0.216 | 0.162 | -0.615 | -0.079 |
| sp\|P34998\|CRFR1_HUMAN Corticotropin-releasing factor receptor 1 OS=Homo sapiens GN=CRHR1 PE=1 SV= | 335 | ASTTSEtIQYR | 0.245 | -0.035 | -0.687 | -0.159 |
| sp\|P34998\|CRFR1_HUMAN Corticotropin-releasing factor receptor 1 OS=Homo sapiens GN=CRHR1 PE=1 SV= | 345 | RKAVKAtLVLL | 0.409 | 0.195 | -0.204 | 0.133 |
| sp\|P34998\|CRFR1_HUMAN Corticotropin-releasing factor receptor 1 OS=Homo sapiens GN=CRHR1 PE=1 SV= | 355 | LPLLGItYMLF | 0.068 | -0.212 | -1.229 | -0.458 |
| sp\|P34998\|CRFR1_HUMAN Corticotropin-releasing factor receptor 1 OS=Homo sapiens GN=CRHR1 PE=1 SV= | 369 | PGEDEVsRVVF | 0.205 | -0.152 | -0.996 | -0.314 |
| sp\|P34998\|CRFR1_HUMAN Corticotropin-releasing factor receptor 1 OS=Homo sapiens GN=CRHR1 PE=1 SV= | 378 | VFIYFNsFLES | 0.297 | 0.084 | -0.646 | -0.088 |
| sp\|P34998\|CRFR1_HUMAN Corticotropin-releasing factor receptor 1 OS=Homo sapiens GN=CRHR1 PE=1 SV= | 382 | FNSFLEsFQGF | 0.158 | -0.121 | -0.863 | -0.275 |
| sp\|P34998\|CRFR1_HUMAN Corticotropin-releasing factor receptor 1 OS=Homo sapiens GN=CRHR1 PE=1 SV= | 389 | FQGFFVsVFYC | 0.201 | -0.063 | -0.94 | -0.267 |
| sp\|P34998\|CRFR1_HUMAN Corticotropin-releasing factor receptor 1 OS=Homo sapiens GN=CRHR1 PE=1 SV= | 397 | FYCFLNsEVRS | 0.33 | 0.107 | -0.929 | -0.164 |
| sp\|P34998\|CRFR1_HUMAN Corticotropin-releasing factor receptor 1 OS=Homo sapiens GN=CRHR1 PE=1 SV= | 401 | LNSEVRsAIRK | 0.059 | -0.295 | -1.582 | -0.606 |
| sp\|P34998\|CRFR1_HUMAN Corticotropin-releasing factor receptor 1 OS=Homo sapiens GN=CRHR1 PE=1 SV= | 415 | RWQDKHsIRAR | 0.327 | 0.164 | -0.471 | 0.007 |
| sp\|P34998\|CRFR1_HUMAN Corticotropin-releasing factor receptor 1 OS=Homo sapiens GN=CRHR1 PE=1 SV= | 425 | RVARAMsIPTS | 0.912 | 1.504 | 1.411 | 1.276 |
| sp\|P34998\|CRFR1_HUMAN Corticotropin-releasing factor receptor 1 OS=Homo sapiens GN=CRHR1 PE=1 SV= | 428 | RAMSIPtSPTR | 0.404 | 0.317 | -0.207 | 0.171 |
| sp\|P34998\|CRFR1_HUMAN Corticotropin-releasing factor receptor 1 OS=Homo sapiens GN=CRHR1 PE=1 SV= | 429 | AMSIPTsPTRV | 0.057 | -0.45 | -1.795 | -0.729 |
| sp\|P34998\|CRFR1_HUMAN Corticotropin-releasing factor receptor 1 OS=Homo sapiens GN=CRHR1 PE=1 SV= | 431 | SIPTSPtRVSF | 0.081 | -0.102 | -1.346 | -0.456 |
| sp\|P34998\|CRFR1_HUMAN Corticotropin-releasing factor receptor 1 OS=Homo sapiens GN=CRHR1 PE=1 SV= | 434 | TSPTRVsFHSI | 0.408 | 0.058 | -0.391 | 0.025 |
| sp\|P34998\|CRFR1_HUMAN Corticotropin-releasing factor receptor 1 OS=Homo sapiens GN=CRHR1 PE=1 SV= | 437 | TRVSFHsIKQS | 0.154 | 0.089 | -0.915 | -0.224 |

| | | | | | | |
|---|---|---|---|---|---|---|
| sp\|P34998\|CRFR1_HUMAN Corticotropin-releasing factor receptor 1 OS=Homo sapiens GN=CRHR1 PE=1 SV= | 441 | FHSIKQsTAV- | 0.085 | -0.043 | -1.436 | -0.465 |
| sp\|P34998\|CRFR1_HUMAN Corticotropin-releasing factor receptor 1 OS=Homo sapiens GN=CRHR1 PE=1 SV= | 442 | HSIKQStAV-- | 0.13 | 0.129 | -1.107 | -0.283 |
| sp\|Q13324\|CRFR2_HUMAN Corticotropin-releasing factor receptor 2 OS=Homo sapiens GN=CRHR2 PE=1 SV= | 322 | LPLLGItYMLF | 0.068 | -0.212 | -1.229 | -0.458 |
| sp\|Q13324\|CRFR2_HUMAN Corticotropin-releasing factor receptor 2 OS=Homo sapiens GN=CRHR2 PE=1 SV= | 336 | PGEDDLsQIMF | 0.066 | -0.266 | -1.377 | -0.526 |
| sp\|Q13324\|CRFR2_HUMAN Corticotropin-releasing factor receptor 2 OS=Homo sapiens GN=CRHR2 PE=1 SV= | 345 | MFIYFNsFLQS | 0.384 | 0.163 | -0.424 | 0.041 |
| sp\|Q13324\|CRFR2_HUMAN Corticotropin-releasing factor receptor 2 OS=Homo sapiens GN=CRHR2 PE=1 SV= | 349 | FNSFLQsFQGF | 0.186 | -0.057 | -0.606 | -0.159 |
| sp\|Q13324\|CRFR2_HUMAN Corticotropin-releasing factor receptor 2 OS=Homo sapiens GN=CRHR2 PE=1 SV= | 356 | FQGFFVsVFYC | 0.201 | -0.063 | -0.94 | -0.267 |
| sp\|Q13324\|CRFR2_HUMAN Corticotropin-releasing factor receptor 2 OS=Homo sapiens GN=CRHR2 PE=1 SV= | 368 | FNGEVRsAVRK | 0.062 | -0.196 | -1.703 | -0.612 |
| sp\|Q13324\|CRFR2_HUMAN Corticotropin-releasing factor receptor 2 OS=Homo sapiens GN=CRHR2 PE=1 SV= | 382 | RWQDHHsLRVP | 0.54 | 0.296 | -0.256 | 0.193 |
| sp\|Q13324\|CRFR2_HUMAN Corticotropin-releasing factor receptor 2 OS=Homo sapiens GN=CRHR2 PE=1 SV= | 392 | PMARAMsIPTS | 0.894 | 1.586 | 1.422 | 1.301 |
| sp\|Q13324\|CRFR2_HUMAN Corticotropin-releasing factor receptor 2 OS=Homo sapiens GN=CRHR2 PE=1 SV= | 395 | RAMSIPtSPTR | 0.404 | 0.317 | -0.207 | 0.171 |
| sp\|Q13324\|CRFR2_HUMAN Corticotropin-releasing factor receptor 2 OS=Homo sapiens GN=CRHR2 PE=1 SV= | 396 | AMSIPTsPTRI | 0.083 | -0.393 | -1.349 | -0.553 |
| sp\|Q13324\|CRFR2_HUMAN Corticotropin-releasing factor receptor 2 OS=Homo sapiens GN=CRHR2 PE=1 SV= | 398 | SIPTSPtRISF | 0.077 | -0.103 | -1.251 | -0.426 |
| sp\|Q13324\|CRFR2_HUMAN Corticotropin-releasing factor receptor 2 OS=Homo sapiens GN=CRHR2 PE=1 SV= | 401 | TSPTRIsFHSI | 0.311 | 0.046 | -0.516 | -0.053 |
| sp\|Q13324\|CRFR2_HUMAN Corticotropin-releasing factor receptor 2 OS=Homo sapiens GN=CRHR2 PE=1 SV= | 404 | TRISFHsIKQT | 0.158 | 0.091 | -0.833 | -0.195 |
| sp\|Q13324\|CRFR2_HUMAN Corticotropin-releasing factor receptor 2 OS=Homo sapiens GN=CRHR2 PE=1 SV= | 408 | FHSIKQtAAV- | 0.098 | 0.015 | -1.435 | -0.441 |
| sp\|Q02643\|GHRHR_HUMAN Growth hormone-releasing hormone receptor OS=Homo sapiens GN=GHRHR PI | 317 | LEPAQGsLHTQ | 0.111 | -0.215 | -1.306 | -0.47 |
| sp\|Q02643\|GHRHR_HUMAN Growth hormone-releasing hormone receptor OS=Homo sapiens GN=GHRHR PI | 320 | AQGSLHtQSQY | 0.081 | -0.196 | -1.491 | -0.535 |
| sp\|Q02643\|GHRHR_HUMAN Growth hormone-releasing hormone receptor OS=Homo sapiens GN=GHRHR PI | 322 | GSLHTQsQYWR | 0.147 | -0.057 | -0.893 | -0.268 |
| sp\|Q02643\|GHRHR_HUMAN Growth hormone-releasing hormone receptor OS=Homo sapiens GN=GHRHR PI | 328 | SQYWRLsKSTL | 0.093 | -0.09 | -0.895 | -0.297 |
| sp\|Q02643\|GHRHR_HUMAN Growth hormone-releasing hormone receptor OS=Homo sapiens GN=GHRHR PI | 330 | YWRLSKsTLFL | 0.477 | 0.462 | 0.017 | 0.319 |
| sp\|Q02643\|GHRHR_HUMAN Growth hormone-releasing hormone receptor OS=Homo sapiens GN=GHRHR PI | 331 | WRLSKStLFLI | 0.285 | 0.157 | -0.243 | 0.066 |
| sp\|Q02643\|GHRHR_HUMAN Growth hormone-releasing hormone receptor OS=Homo sapiens GN=GHRHR PI | 366 | LELGLGsFQGF | 0.09 | -0.183 | -1.006 | -0.366 |
| sp\|Q02643\|GHRHR_HUMAN Growth hormone-releasing hormone receptor OS=Homo sapiens GN=GHRHR PI | 385 | LNQEVRtEISR | 0.054 | -0.083 | -1.239 | -0.423 |
| sp\|Q02643\|GHRHR_HUMAN Growth hormone-releasing hormone receptor OS=Homo sapiens GN=GHRHR PI | 388 | EVRTEIsRKWH | 0.147 | -0.074 | -1.101 | -0.343 |
| sp\|Q02643\|GHRHR_HUMAN Growth hormone-releasing hormone receptor OS=Homo sapiens GN=GHRHR PI | 404 | LLPAWRtRAKW | 0.082 | -0.169 | -1.337 | -0.475 |
| sp\|Q02643\|GHRHR_HUMAN Growth hormone-releasing hormone receptor OS=Homo sapiens GN=GHRHR PI | 409 | RTRAKWtTPSR | 0.654 | 0.551 | 0.02 | 0.408 |
| sp\|Q02643\|GHRHR_HUMAN Growth hormone-releasing hormone receptor OS=Homo sapiens GN=GHRHR PI | 410 | TRAKWTtPSRS | 0.121 | -0.198 | -1.085 | -0.387 |
| sp\|Q02643\|GHRHR_HUMAN Growth hormone-releasing hormone receptor OS=Homo sapiens GN=GHRHR PI | 412 | AKWTTPsRSAA | 0.145 | -0.038 | -0.964 | -0.286 |
| sp\|Q02643\|GHRHR_HUMAN Growth hormone-releasing hormone receptor OS=Homo sapiens GN=GHRHR PI | 414 | WTTPSRsAAKV | 0.192 | 0.024 | -1.038 | -0.274 |
| sp\|Q02643\|GHRHR_HUMAN Growth hormone-releasing hormone receptor OS=Homo sapiens GN=GHRHR PI | 420 | SAAKVLtSMC- | 0.126 | -0.099 | -1.253 | -0.409 |
| sp\|Q02643\|GHRHR_HUMAN Growth hormone-releasing hormone receptor OS=Homo sapiens GN=GHRHR PI | 421 | AAKVLTsMC-- | 0.083 | -0.02 | -1.38 | -0.439 |
| sp\|P48546\|GIPR_HUMAN Gastric inhibitory polypeptide receptor OS=Homo sapiens GN=GIPR PE=1 SV=1 | 323 | ILGILLsKLRT | 0.069 | -0.209 | -1.323 | -0.488 |
| sp\|P48546\|GIPR_HUMAN Gastric inhibitory polypeptide receptor OS=Homo sapiens GN=GIPR PE=1 SV=1 | 327 | LLSKLRtRQMR | 0.214 | -0.107 | -0.93 | -0.274 |
| sp\|P48546\|GIPR_HUMAN Gastric inhibitory polypeptide receptor OS=Homo sapiens GN=GIPR PE=1 SV=1 | 342 | LRLRLARsTLTL | 0.499 | 0.381 | -0.392 | 0.163 |
| sp\|P48546\|GIPR_HUMAN Gastric inhibitory polypeptide receptor OS=Homo sapiens GN=GIPR PE=1 SV=1 | 343 | LRLARStLTLV | 0.517 | 0.331 | -0.232 | 0.205 |
| sp\|P48546\|GIPR_HUMAN Gastric inhibitory polypeptide receptor OS=Homo sapiens GN=GIPR PE=1 SV=1 | 345 | LARSTLtLVPL | 0.196 | 0.164 | -0.501 | -0.047 |
| sp\|P48546\|GIPR_HUMAN Gastric inhibitory polypeptide receptor OS=Homo sapiens GN=GIPR PE=1 SV=1 | 361 | VVFAPVtEEQA | 0.225 | -0.139 | -0.574 | -0.163 |
| sp\|P48546\|GIPR_HUMAN Gastric inhibitory polypeptide receptor OS=Homo sapiens GN=GIPR PE=1 SV=1 | 381 | GFEIFLsSFQG | 0.19 | -0.082 | -0.807 | -0.233 |
| sp\|P48546\|GIPR_HUMAN Gastric inhibitory polypeptide receptor OS=Homo sapiens GN=GIPR PE=1 SV=1 | 382 | FEIFLSsFQGF | 0.145 | -0.025 | -0.941 | -0.274 |
| sp\|P48546\|GIPR_HUMAN Gastric inhibitory polypeptide receptor OS=Homo sapiens GN=GIPR PE=1 SV=1 | 389 | FQGFLVsVLYC | 0.109 | -0.132 | -1.305 | -0.443 |
| sp\|P48546\|GIPR_HUMAN Gastric inhibitory polypeptide receptor OS=Homo sapiens GN=GIPR PE=1 SV=1 | 401 | INKEVQsEIRR | 0.18 | -0.096 | -0.667 | -0.194 |
| sp\|P48546\|GIPR_HUMAN Gastric inhibitory polypeptide receptor OS=Homo sapiens GN=GIPR PE=1 SV=1 | 415 | HCRLRRsLGEE | 0.812 | 0.592 | 0.287 | 0.564 |
| sp\|P48546\|GIPR_HUMAN Gastric inhibitory polypeptide receptor OS=Homo sapiens GN=GIPR PE=1 SV=1 | 433 | AFRALPsGSGP | 0.127 | -0.167 | -1.002 | -0.347 |
| sp\|P48546\|GIPR_HUMAN Gastric inhibitory polypeptide receptor OS=Homo sapiens GN=GIPR PE=1 SV=1 | 435 | RALPSGsGPGE | 0.535 | 0.403 | 0.057 | 0.332 |
| sp\|P48546\|GIPR_HUMAN Gastric inhibitory polypeptide receptor OS=Homo sapiens GN=GIPR PE=1 SV=1 | 442 | GPGEVPtSRGL | 0.092 | -0.254 | -1.316 | -0.493 |
| sp\|P48546\|GIPR_HUMAN Gastric inhibitory polypeptide receptor OS=Homo sapiens GN=GIPR PE=1 SV=1 | 443 | PGEVPTsRGLS | 0.116 | -0.204 | -0.914 | -0.334 |
| sp\|P48546\|GIPR_HUMAN Gastric inhibitory polypeptide receptor OS=Homo sapiens GN=GIPR PE=1 SV=1 | 447 | PTSRGLsSGTL | 0.446 | 0.724 | -0.016 | 0.385 |
| sp\|P48546\|GIPR_HUMAN Gastric inhibitory polypeptide receptor OS=Homo sapiens GN=GIPR PE=1 SV=1 | 448 | TSRGLSsGTLP | 0.094 | -0.119 | -1.169 | -0.398 |

| | | | | | | |
|---|---|---|---|---|---|---|
| sp\|P48546\|GIPR_HUMAN Gastric inhibitory polypeptide receptor OS=Homo sapiens GN=GIPR PE=1 SV=1 | 450 | RGLSSGtLPGP | 0.713 | 0.554 | 0.511 | 0.593 |
| sp\|P48546\|GIPR_HUMAN Gastric inhibitory polypeptide receptor OS=Homo sapiens GN=GIPR PE=1 SV=1 | 459 | GPGNEAsRELE | 0.104 | -0.19 | -1.361 | -0.482 |
| sp\|P48546\|GIPR_HUMAN Gastric inhibitory polypeptide receptor OS=Homo sapiens GN=GIPR PE=1 SV=1 | 464 | ASRELEsYC-- | 0.089 | -0.022 | -1.487 | -0.473 |
| sp\|P43220\|GLP1R_HUMAN Glucagon-like peptide 1 receptor OS=Homo sapiens GN=GLP1R PE=1 SV=2 | 333 | VICIVVsKLKA | 0.223 | -0.058 | -0.845 | -0.227 |
| sp\|P43220\|GLP1R_HUMAN Glucagon-like peptide 1 receptor OS=Homo sapiens GN=GLP1R PE=1 SV=2 | 343 | ANLMCKtDIKC | 0.211 | -0.18 | -0.807 | -0.259 |
| sp\|P43220\|GLP1R_HUMAN Glucagon-like peptide 1 receptor OS=Homo sapiens GN=GLP1R PE=1 SV=2 | 352 | KCRLAKsTLTL | 0.549 | 0.335 | -0.24 | 0.215 |
| sp\|P43220\|GLP1R_HUMAN Glucagon-like peptide 1 receptor OS=Homo sapiens GN=GLP1R PE=1 SV=2 | 353 | CRLAKStLTLI | 0.432 | 0.227 | -0.084 | 0.192 |
| sp\|P43220\|GLP1R_HUMAN Glucagon-like peptide 1 receptor OS=Homo sapiens GN=GLP1R PE=1 SV=2 | 355 | LAKSTLtLIPL | 0.106 | -0.049 | -0.627 | -0.19 |
| sp\|P43220\|GLP1R_HUMAN Glucagon-like peptide 1 receptor OS=Homo sapiens GN=GLP1R PE=1 SV=2 | 362 | LIPLLGtHEVI | 0.108 | -0.07 | -1.149 | -0.37 |
| sp\|P43220\|GLP1R_HUMAN Glucagon-like peptide 1 receptor OS=Homo sapiens GN=GLP1R PE=1 SV=2 | 378 | DEHARGtLRFI | 0.331 | -0.052 | -0.695 | -0.139 |
| sp\|P43220\|GLP1R_HUMAN Glucagon-like peptide 1 receptor OS=Homo sapiens GN=GLP1R PE=1 SV=2 | 386 | RFIKLFtELSF | 0.151 | 0.087 | -0.82 | -0.194 |
| sp\|P43220\|GLP1R_HUMAN Glucagon-like peptide 1 receptor OS=Homo sapiens GN=GLP1R PE=1 SV=2 | 389 | KLFTELsFTSF | 0.144 | -0.074 | -1.083 | -0.338 |
| sp\|P43220\|GLP1R_HUMAN Glucagon-like peptide 1 receptor OS=Homo sapiens GN=GLP1R PE=1 SV=2 | 391 | FTELSFtSFQG | 0.477 | 0.224 | -0.156 | 0.182 |
| sp\|P43220\|GLP1R_HUMAN Glucagon-like peptide 1 receptor OS=Homo sapiens GN=GLP1R PE=1 SV=2 | 392 | TELSFTsFQGL | 0.217 | -0.086 | -0.698 | -0.189 |
| sp\|P43220\|GLP1R_HUMAN Glucagon-like peptide 1 receptor OS=Homo sapiens GN=GLP1R PE=1 SV=2 | 416 | QLEFRKsWERW | 0.503 | 0.285 | -0.131 | 0.219 |
| sp\|P43220\|GLP1R_HUMAN Glucagon-like peptide 1 receptor OS=Homo sapiens GN=GLP1R PE=1 SV=2 | 431 | LHIQRDsSMKP | 0.139 | -0.085 | -1.324 | -0.423 |
| sp\|P43220\|GLP1R_HUMAN Glucagon-like peptide 1 receptor OS=Homo sapiens GN=GLP1R PE=1 SV=2 | 432 | HIQRDSsMKPL | 0.448 | 0.998 | 0.211 | 0.552 |
| sp\|P43220\|GLP1R_HUMAN Glucagon-like peptide 1 receptor OS=Homo sapiens GN=GLP1R PE=1 SV=2 | 440 | KPLKCPtSSLS | 0.159 | -0.203 | -1.105 | -0.383 |
| sp\|P43220\|GLP1R_HUMAN Glucagon-like peptide 1 receptor OS=Homo sapiens GN=GLP1R PE=1 SV=2 | 441 | PLKCPTsSLSS | 0.14 | -0.11 | -0.948 | -0.306 |
| sp\|P43220\|GLP1R_HUMAN Glucagon-like peptide 1 receptor OS=Homo sapiens GN=GLP1R PE=1 SV=2 | 442 | LKCPTSsLSSG | 0.118 | -0.06 | -0.829 | -0.257 |
| sp\|P43220\|GLP1R_HUMAN Glucagon-like peptide 1 receptor OS=Homo sapiens GN=GLP1R PE=1 SV=2 | 444 | CPTSSLsSGAT | 0.27 | 0.114 | -0.271 | 0.038 |
| sp\|P43220\|GLP1R_HUMAN Glucagon-like peptide 1 receptor OS=Homo sapiens GN=GLP1R PE=1 SV=2 | 445 | PTSSLSsGATA | 0.055 | -0.405 | -1.519 | -0.623 |
| sp\|P43220\|GLP1R_HUMAN Glucagon-like peptide 1 receptor OS=Homo sapiens GN=GLP1R PE=1 SV=2 | 448 | SLSSGAtAGSS | 0.102 | -0.107 | -1.311 | -0.439 |
| sp\|P43220\|GLP1R_HUMAN Glucagon-like peptide 1 receptor OS=Homo sapiens GN=GLP1R PE=1 SV=2 | 451 | SGATAGsSMYT | 0.09 | -0.268 | -1.378 | -0.519 |
| sp\|P43220\|GLP1R_HUMAN Glucagon-like peptide 1 receptor OS=Homo sapiens GN=GLP1R PE=1 SV=2 | 452 | GATAGSsMYTA | 0.093 | -0.143 | -1.174 | -0.408 |
| sp\|P43220\|GLP1R_HUMAN Glucagon-like peptide 1 receptor OS=Homo sapiens GN=GLP1R PE=1 SV=2 | 455 | AGSSMYtATCQ | 0.166 | -0.137 | -1.026 | -0.332 |
| sp\|P43220\|GLP1R_HUMAN Glucagon-like peptide 1 receptor OS=Homo sapiens GN=GLP1R PE=1 SV=2 | 457 | SSMYTAtCQAS | 0.147 | 0.12 | -0.781 | -0.171 |
| sp\|P43220\|GLP1R_HUMAN Glucagon-like peptide 1 receptor OS=Homo sapiens GN=GLP1R PE=1 SV=2 | 461 | TATCQAsCS-- | 0.132 | 0.052 | -1.234 | -0.35 |
| sp\|P43220\|GLP1R_HUMAN Glucagon-like peptide 1 receptor OS=Homo sapiens GN=GLP1R PE=1 SV=2 | 463 | TCQASCs---- | 0.552 | 0.382 | -0.369 | 0.188 |
| sp\|O95838\|GLP2R_HUMAN Glucagon-like peptide 2 receptor OS=Homo sapiens GN=GLP2R PE=2 SV=1 | 328 | RAHLENtGCWT | 0.084 | -0.259 | -1.494 | -0.556 |
| sp\|O95838\|GLP2R_HUMAN Glucagon-like peptide 2 receptor OS=Homo sapiens GN=GLP2R PE=2 SV=1 | 332 | ENTGCWtTNGN | 0.21 | -0.154 | -0.544 | -0.163 |
| sp\|O95838\|GLP2R_HUMAN Glucagon-like peptide 2 receptor OS=Homo sapiens GN=GLP2R PE=2 SV=1 | 333 | NTGCWTtNGNK | 0.279 | -0.04 | -0.962 | -0.241 |
| sp\|O95838\|GLP2R_HUMAN Glucagon-like peptide 2 receptor OS=Homo sapiens GN=GLP2R PE=2 SV=1 | 352 | PMMLCVtVNFF | 0.266 | 0.117 | -0.501 | -0.039 |
| sp\|O95838\|GLP2R_HUMAN Glucagon-like peptide 2 receptor OS=Homo sapiens GN=GLP2R PE=2 SV=1 | 367 | ILKLLIsKLKA | 0.249 | -0.045 | -0.628 | -0.141 |
| sp\|O95838\|GLP2R_HUMAN Glucagon-like peptide 2 receptor OS=Homo sapiens GN=GLP2R PE=2 SV=1 | 386 | KYRLAKsTLVL | 0.55 | 0.389 | -0.132 | 0.269 |
| sp\|O95838\|GLP2R_HUMAN Glucagon-like peptide 2 receptor OS=Homo sapiens GN=GLP2R PE=2 SV=1 | 387 | YRLAKStLVLI | 0.268 | 0.211 | -0.28 | 0.066 |
| sp\|O95838\|GLP2R_HUMAN Glucagon-like peptide 2 receptor OS=Homo sapiens GN=GLP2R PE=2 SV=1 | 402 | VHEILFsFITD | 0.163 | -0.018 | -0.822 | -0.226 |
| sp\|O95838\|GLP2R_HUMAN Glucagon-like peptide 2 receptor OS=Homo sapiens GN=GLP2R PE=2 SV=1 | 405 | ILFSFItDDQV | 0.507 | 0.046 | -0.561 | -0.003 |
| sp\|O95838\|GLP2R_HUMAN Glucagon-like peptide 2 receptor OS=Homo sapiens GN=GLP2R PE=2 SV=1 | 423 | RLFIQLtLSSF | 0.1 | 0.038 | -1.007 | -0.29 |
| sp\|O95838\|GLP2R_HUMAN Glucagon-like peptide 2 receptor OS=Homo sapiens GN=GLP2R PE=2 SV=1 | 425 | FIQLTLsSFHG | 0.111 | 0.08 | -0.92 | -0.243 |
| sp\|O95838\|GLP2R_HUMAN Glucagon-like peptide 2 receptor OS=Homo sapiens GN=GLP2R PE=2 SV=1 | 426 | IQLTLSsFHGF | 0.128 | -0.172 | -0.795 | -0.28 |
| sp\|O95838\|GLP2R_HUMAN Glucagon-like peptide 2 receptor OS=Homo sapiens GN=GLP2R PE=2 SV=1 | 460 | FLLARHsGCRA | 0.498 | 0.147 | -0.314 | 0.11 |
| sp\|O95838\|GLP2R_HUMAN Glucagon-like peptide 2 receptor OS=Homo sapiens GN=GLP2R PE=2 SV=1 | 482 | KCPKKLsEGDG | 0.332 | 0.072 | -0.648 | -0.081 |
| sp\|O95838\|GLP2R_HUMAN Glucagon-like peptide 2 receptor OS=Homo sapiens GN=GLP2R PE=2 SV=1 | 496 | LRKLQPsLNSG | 0.277 | 0.226 | -0.356 | 0.049 |
| sp\|O95838\|GLP2R_HUMAN Glucagon-like peptide 2 receptor OS=Homo sapiens GN=GLP2R PE=2 SV=1 | 499 | LQPSLNsGRLL | 0.074 | -0.234 | -1.192 | -0.451 |
| sp\|O95838\|GLP2R_HUMAN Glucagon-like peptide 2 receptor OS=Homo sapiens GN=GLP2R PE=2 SV=1 | 528 | ARWPRGsSLSE | 0.071 | 0.126 | -1.327 | -0.377 |
| sp\|O95838\|GLP2R_HUMAN Glucagon-like peptide 2 receptor OS=Homo sapiens GN=GLP2R PE=2 SV=1 | 529 | RWPRGSsLSEC | 0.367 | 0.703 | -0.286 | 0.261 |
| sp\|O95838\|GLP2R_HUMAN Glucagon-like peptide 2 receptor OS=Homo sapiens GN=GLP2R PE=2 SV=1 | 531 | PRGSSLsECSE | 0.122 | 0.2 | -0.842 | -0.173 |
| sp\|O95838\|GLP2R_HUMAN Glucagon-like peptide 2 receptor OS=Homo sapiens GN=GLP2R PE=2 SV=1 | 534 | SSLSECsEGDV | 0.319 | -0.053 | -1.07 | -0.268 |

| | | | | | | |
|---|---|---|---|---|---|---|
| sp\|O95838\|GLP2R_HUMAN Glucagon-like peptide 2 receptor OS=Homo sapiens GN=GLP2R PE=2 SV=1 | 539 | CSEGDVtMANT | 0.194 | -0.072 | -0.855 | -0.244 |
| sp\|O95838\|GLP2R_HUMAN Glucagon-like peptide 2 receptor OS=Homo sapiens GN=GLP2R PE=2 SV=1 | 543 | DVTMANtMEEI | 0.516 | 0.224 | -0.337 | 0.134 |
| sp\|O95838\|GLP2R_HUMAN Glucagon-like peptide 2 receptor OS=Homo sapiens GN=GLP2R PE=2 SV=1 | 551 | EEILEEsEI-- | 0.096 | 0.062 | -1.342 | -0.395 |
| sp\|P47871\|GLR_HUMAN Glucagon receptor OS=Homo sapiens GN=GCGR PE=1 SV=1 | 341 | ARQMHHtDYKF | 0.377 | 0.342 | -0.57 | 0.05 |
| sp\|P47871\|GLR_HUMAN Glucagon receptor OS=Homo sapiens GN=GCGR PE=1 SV=1 | 350 | KFRLAKsTLTL | 0.526 | 0.313 | -0.073 | 0.255 |
| sp\|P47871\|GLR_HUMAN Glucagon receptor OS=Homo sapiens GN=GCGR PE=1 SV=1 | 351 | FRLAKStLTLI | 0.419 | 0.271 | -0.171 | 0.173 |
| sp\|P47871\|GLR_HUMAN Glucagon receptor OS=Homo sapiens GN=GCGR PE=1 SV=1 | 353 | LAKSTLtLIPL | 0.106 | -0.049 | -0.627 | -0.19 |
| sp\|P47871\|GLR_HUMAN Glucagon receptor OS=Homo sapiens GN=GCGR PE=1 SV=1 | 369 | VVFAFVtDEHA | 0.2 | -0.155 | -0.885 | -0.28 |
| sp\|P47871\|GLR_HUMAN Glucagon receptor OS=Homo sapiens GN=GCGR PE=1 SV=1 | 376 | DEHAQGtLRSA | 0.091 | -0.276 | -1.417 | -0.534 |
| sp\|P47871\|GLR_HUMAN Glucagon receptor OS=Homo sapiens GN=GCGR PE=1 SV=1 | 379 | AQGTLRsAKLF | 0.043 | -0.191 | -1.638 | -0.595 |
| sp\|P47871\|GLR_HUMAN Glucagon receptor OS=Homo sapiens GN=GCGR PE=1 SV=1 | 389 | FFDLFLsSFQG | 0.153 | -0.076 | -0.965 | -0.296 |
| sp\|P47871\|GLR_HUMAN Glucagon receptor OS=Homo sapiens GN=GCGR PE=1 SV=1 | 390 | FDLFLSsFQGL | 0.146 | -0.054 | -0.771 | -0.226 |
| sp\|P47871\|GLR_HUMAN Glucagon receptor OS=Homo sapiens GN=GCGR PE=1 SV=1 | 409 | LNKEVQsELRR | 0.116 | -0.128 | -0.996 | -0.336 |
| sp\|P47871\|GLR_HUMAN Glucagon receptor OS=Homo sapiens GN=GCGR PE=1 SV=1 | 430 | LWEERNtSNHR | 0.253 | 0.096 | -0.483 | -0.045 |
| sp\|P47871\|GLR_HUMAN Glucagon receptor OS=Homo sapiens GN=GCGR PE=1 SV=1 | 431 | WEERNTsNHRA | 0.624 | 0.652 | 0.114 | 0.463 |
| sp\|P47871\|GLR_HUMAN Glucagon receptor OS=Homo sapiens GN=GCGR PE=1 SV=1 | 436 | TSNHRAsSSPG | 0.155 | -0.137 | -1.103 | -0.362 |
| sp\|P47871\|GLR_HUMAN Glucagon receptor OS=Homo sapiens GN=GCGR PE=1 SV=1 | 437 | SNHRASsSPGH | 0.512 | 0.957 | 0.1 | 0.523 |
| sp\|P47871\|GLR_HUMAN Glucagon receptor OS=Homo sapiens GN=GCGR PE=1 SV=1 | 438 | NHRASSsPGHG | 0.262 | 0.026 | -0.634 | -0.115 |
| sp\|P47871\|GLR_HUMAN Glucagon receptor OS=Homo sapiens GN=GCGR PE=1 SV=1 | 445 | PGHGPPsKELQ | 0.072 | -0.273 | -1.125 | -0.442 |
| sp\|P47871\|GLR_HUMAN Glucagon receptor OS=Homo sapiens GN=GCGR PE=1 SV=1 | 456 | FGRGGGsQDSS | 0.099 | 0.033 | -1.132 | -0.333 |
| sp\|P47871\|GLR_HUMAN Glucagon receptor OS=Homo sapiens GN=GCGR PE=1 SV=1 | 459 | GGGSQDsSAET | 0.074 | -0.321 | -1.627 | -0.625 |
| sp\|P47871\|GLR_HUMAN Glucagon receptor OS=Homo sapiens GN=GCGR PE=1 SV=1 | 460 | GGSQDSsAETP | 0.117 | -0.21 | -1.171 | -0.421 |
| sp\|P47871\|GLR_HUMAN Glucagon receptor OS=Homo sapiens GN=GCGR PE=1 SV=1 | 463 | QDSSAEtPLAG | 0.051 | -0.458 | -1.558 | -0.655 |
| sp\|P47871\|GLR_HUMAN Glucagon receptor OS=Homo sapiens GN=GCGR PE=1 SV=1 | 475 | LPRLAEsPF-- | 0.211 | 0.04 | -0.904 | -0.218 |
| sp\|P47872\|SCTR_HUMAN Secretin receptor OS=Homo sapiens GN=SCTR PE=2 SV=2 | 326 | LMRKLRtQETR | 0.239 | 0.222 | -0.882 | -0.14 |
| sp\|P47872\|SCTR_HUMAN Secretin receptor OS=Homo sapiens GN=SCTR PE=2 SV=2 | 329 | KLRTQEtRGNE | 0.382 | 0.16 | -0.889 | -0.116 |
| sp\|P47872\|SCTR_HUMAN Secretin receptor OS=Homo sapiens GN=SCTR PE=2 SV=2 | 335 | TRGNEVsHYKR | 0.195 | 0.094 | -0.738 | -0.15 |
| sp\|P47872\|SCTR_HUMAN Secretin receptor OS=Homo sapiens GN=SCTR PE=2 SV=2 | 343 | YKRLARsTLLL | 0.457 | 0.37 | -0.331 | 0.165 |
| sp\|P47872\|SCTR_HUMAN Secretin receptor OS=Homo sapiens GN=SCTR PE=2 SV=2 | 344 | KRLARStLLLI | 0.456 | 0.348 | -0.092 | 0.237 |
| sp\|P47872\|SCTR_HUMAN Secretin receptor OS=Homo sapiens GN=SCTR PE=2 SV=2 | 361 | YIVFAFsPEDA | 0.238 | -0.069 | -0.593 | -0.141 |
| sp\|P47872\|SCTR_HUMAN Secretin receptor OS=Homo sapiens GN=SCTR PE=2 SV=2 | 378 | FELALGsFQGL | 0.19 | -0.057 | -0.811 | -0.226 |
| sp\|P47872\|SCTR_HUMAN Secretin receptor OS=Homo sapiens GN=SCTR PE=2 SV=2 | 418 | PLHPVAsFSNS | 0.24 | 0.046 | -0.881 | -0.198 |
| sp\|P47872\|SCTR_HUMAN Secretin receptor OS=Homo sapiens GN=SCTR PE=2 SV=2 | 420 | HPVASFsNSTK | 0.181 | 0.031 | -0.748 | -0.179 |
| sp\|P47872\|SCTR_HUMAN Secretin receptor OS=Homo sapiens GN=SCTR PE=2 SV=2 | 422 | VASFSNsTKAS | 0.358 | 0.151 | -0.394 | 0.038 |
| sp\|P47872\|SCTR_HUMAN Secretin receptor OS=Homo sapiens GN=SCTR PE=2 SV=2 | 423 | ASFSNStKASH | 0.039 | -0.481 | -1.759 | -0.734 |
| sp\|P47872\|SCTR_HUMAN Secretin receptor OS=Homo sapiens GN=SCTR PE=2 SV=2 | 426 | SNSTKAsHLEQ | 0.085 | -0.302 | -1.548 | -0.588 |
| sp\|P47872\|SCTR_HUMAN Secretin receptor OS=Homo sapiens GN=SCTR PE=2 SV=2 | 431 | ASHLEQsQGTC | 0.091 | -0.312 | -1.706 | -0.642 |
| sp\|P47872\|SCTR_HUMAN Secretin receptor OS=Homo sapiens GN=SCTR PE=2 SV=2 | 434 | LEQSQGtCRTS | 0.065 | -0.202 | -1.548 | -0.562 |
| sp\|P47872\|SCTR_HUMAN Secretin receptor OS=Homo sapiens GN=SCTR PE=2 SV=2 | 437 | SQGTCRtSII- | 0.066 | -0.164 | -1.418 | -0.505 |
| sp\|P47872\|SCTR_HUMAN Secretin receptor OS=Homo sapiens GN=SCTR PE=2 SV=2 | 438 | QGTCRTsII-- | 0.253 | 0.167 | -0.388 | 0.011 |
| sp\|Q03431\|PTH1R_HUMAN Parathyroid hormone/parathyroid hormone-related peptide receptor OS=Homo | 316 | IFMAFFsEKKY | 0.303 | 0.081 | -0.455 | -0.024 |
| sp\|Q03431\|PTH1R_HUMAN Parathyroid hormone/parathyroid hormone-related peptide receptor OS=Homo | 325 | KYLWGFtVFGW | 0.193 | -0.076 | -0.563 | -0.149 |
| sp\|Q03431\|PTH1R_HUMAN Parathyroid hormone/parathyroid hormone-related peptide receptor OS=Homo | 341 | FVAVWVsVRAT | 0.29 | 0.053 | -0.289 | 0.018 |
| sp\|Q03431\|PTH1R_HUMAN Parathyroid hormone/parathyroid hormone-related peptide receptor OS=Homo | 345 | WVSVRAtLANT | 0.28 | 0.099 | -0.522 | -0.048 |
| sp\|Q03431\|PTH1R_HUMAN Parathyroid hormone/parathyroid hormone-related peptide receptor OS=Homo | 349 | RATLANtGCWD | 0.305 | -0.014 | -0.632 | -0.114 |
| sp\|Q03431\|PTH1R_HUMAN Parathyroid hormone/parathyroid hormone-related peptide receptor OS=Homo | 355 | TGCWDLsSGNK | 0.149 | -0.187 | -1.032 | -0.357 |
| sp\|Q03431\|PTH1R_HUMAN Parathyroid hormone/parathyroid hormone-related peptide receptor OS=Homo | 356 | GCWDLSsGNKK | 0.108 | -0.041 | -1.262 | -0.398 |
| sp\|Q03431\|PTH1R_HUMAN Parathyroid hormone/parathyroid hormone-related peptide receptor OS=Homo | 370 | QVPILAsIVLN | 0.065 | -0.119 | -1.373 | -0.476 |
| sp\|Q03431\|PTH1R_HUMAN Parathyroid hormone/parathyroid hormone-related peptide receptor OS=Homo | 387 | IVRVLAtKLRE | 0.198 | 0.023 | -0.944 | -0.241 |

| | | | | | | |
|---|---|---|---|---|---|---|
| sp\|Q03431\|PTH1R_HUMAN Parathyroid hormone/parathyroid hormone-related peptide receptor OS=Homo | 392 | ATKLREtNAGR | 0.289 | -0.089 | -0.948 | -0.249 |
| sp\|Q03431\|PTH1R_HUMAN Parathyroid hormone/parathyroid hormone-related peptide receptor OS=Homo | 399 | NAGRCDtRQQY | 0.649 | 0.664 | 0.014 | 0.442 |
| sp\|Q03431\|PTH1R_HUMAN Parathyroid hormone/parathyroid hormone-related peptide receptor OS=Homo | 409 | YRKLLKsTLVL | 0.231 | 0.272 | -0.529 | -0.009 |
| sp\|Q03431\|PTH1R_HUMAN Parathyroid hormone/parathyroid hormone-related peptide receptor OS=Homo | 410 | RKLLKStLVLM | 0.286 | 0.058 | -0.462 | -0.039 |
| sp\|Q03431\|PTH1R_HUMAN Parathyroid hormone/parathyroid hormone-related peptide receptor OS=Homo | 427 | YIVFMAtPYTE | 0.137 | -0.295 | -1.093 | -0.417 |
| sp\|Q03431\|PTH1R_HUMAN Parathyroid hormone/parathyroid hormone-related peptide receptor OS=Homo | 430 | FMATPYtEVSG | 0.107 | 0.157 | -0.71 | -0.149 |
| sp\|Q03431\|PTH1R_HUMAN Parathyroid hormone/parathyroid hormone-related peptide receptor OS=Homo | 433 | TPYTEVsGTLW | 0.143 | -0.205 | -1.191 | -0.418 |
| sp\|Q03431\|PTH1R_HUMAN Parathyroid hormone/parathyroid hormone-related peptide receptor OS=Homo | 435 | YTEVSGtLWQV | 0.482 | 0.172 | -0.342 | 0.104 |
| sp\|Q03431\|PTH1R_HUMAN Parathyroid hormone/parathyroid hormone-related peptide receptor OS=Homo | 449 | YEMLFNsFQGF | 0.257 | 0.038 | -0.688 | -0.131 |
| sp\|Q03431\|PTH1R_HUMAN Parathyroid hormone/parathyroid hormone-related peptide receptor OS=Homo | 473 | QAEIKKsWSRW | 0.079 | -0.138 | -1.178 | -0.412 |
| sp\|Q03431\|PTH1R_HUMAN Parathyroid hormone/parathyroid hormone-related peptide receptor OS=Homo | 475 | EIKKSWsRWTL | 0.666 | 0.287 | 0.118 | 0.357 |
| sp\|Q03431\|PTH1R_HUMAN Parathyroid hormone/parathyroid hormone-related peptide receptor OS=Homo | 478 | KSWSRWtLALD | 0.381 | 0.123 | -0.367 | 0.046 |
| sp\|Q03431\|PTH1R_HUMAN Parathyroid hormone/parathyroid hormone-related peptide receptor OS=Homo | 489 | FKRKARsGSSS | 0.423 | 0.301 | -0.407 | 0.106 |
| sp\|Q03431\|PTH1R_HUMAN Parathyroid hormone/parathyroid hormone-related peptide receptor OS=Homo | 491 | RKARSGsSSYS | 0.673 | 0.936 | 0.347 | 0.652 |
| sp\|Q03431\|PTH1R_HUMAN Parathyroid hormone/parathyroid hormone-related peptide receptor OS=Homo | 492 | KARSGSsSYSY | 0.073 | -0.11 | -1.423 | -0.487 |
| sp\|Q03431\|PTH1R_HUMAN Parathyroid hormone/parathyroid hormone-related peptide receptor OS=Homo | 493 | ARSGSSsYSYG | 0.133 | 0.124 | -0.889 | -0.211 |
| sp\|Q03431\|PTH1R_HUMAN Parathyroid hormone/parathyroid hormone-related peptide receptor OS=Homo | 495 | SGSSSYsYGPM | 0.188 | 0.024 | -0.478 | -0.089 |
| sp\|Q03431\|PTH1R_HUMAN Parathyroid hormone/parathyroid hormone-related peptide receptor OS=Homo | 501 | SYGPMVsHTSV | 0.164 | -0.226 | -1.273 | -0.445 |
| sp\|Q03431\|PTH1R_HUMAN Parathyroid hormone/parathyroid hormone-related peptide receptor OS=Homo | 503 | GPMVSHtSVTN | 0.302 | 0.282 | -0.577 | 0.002 |
| sp\|Q03431\|PTH1R_HUMAN Parathyroid hormone/parathyroid hormone-related peptide receptor OS=Homo | 504 | PMVSHTsVTNV | 0.232 | 0.128 | -1.069 | -0.236 |
| sp\|Q03431\|PTH1R_HUMAN Parathyroid hormone/parathyroid hormone-related peptide receptor OS=Homo | 506 | VSHTSVtNVGP | 0.183 | -0.12 | -0.903 | -0.28 |
| sp\|Q03431\|PTH1R_HUMAN Parathyroid hormone/parathyroid hormone-related peptide receptor OS=Homo | 519 | GLGLPLsPRLL | 0.072 | -0.342 | -1.242 | -0.504 |
| sp\|Q03431\|PTH1R_HUMAN Parathyroid hormone/parathyroid hormone-related peptide receptor OS=Homo | 525 | SPRLLPtATTN | 0.204 | 0.019 | -1.232 | -0.336 |
| sp\|Q03431\|PTH1R_HUMAN Parathyroid hormone/parathyroid hormone-related peptide receptor OS=Homo | 527 | RLLPTAtTNGH | 0.231 | -0.039 | -0.675 | -0.161 |
| sp\|Q03431\|PTH1R_HUMAN Parathyroid hormone/parathyroid hormone-related peptide receptor OS=Homo | 528 | LLPTATtNGHP | 0.183 | -0.069 | -0.785 | -0.224 |
| sp\|Q03431\|PTH1R_HUMAN Parathyroid hormone/parathyroid hormone-related peptide receptor OS=Homo | 542 | GHAKPGtPALE | 0.082 | -0.485 | -1.591 | -0.665 |
| sp\|Q03431\|PTH1R_HUMAN Parathyroid hormone/parathyroid hormone-related peptide receptor OS=Homo | 547 | GTPALEtLETT | 0.171 | -0.197 | -1.151 | -0.392 |
| sp\|Q03431\|PTH1R_HUMAN Parathyroid hormone/parathyroid hormone-related peptide receptor OS=Homo | 550 | ALETLEtTPPA | 0.475 | 0.29 | -0.39 | 0.125 |
| sp\|Q03431\|PTH1R_HUMAN Parathyroid hormone/parathyroid hormone-related peptide receptor OS=Homo | 551 | LETLETtPPAM | 0.312 | 0.163 | -0.41 | 0.022 |
| sp\|Q03431\|PTH1R_HUMAN Parathyroid hormone/parathyroid hormone-related peptide receptor OS=Homo | 567 | DGFLNGsCSGL | 0.139 | -0.121 | -1.093 | -0.358 |
| sp\|Q03431\|PTH1R_HUMAN Parathyroid hormone/parathyroid hormone-related peptide receptor OS=Homo | 569 | FLNGSCsGLDE | 0.516 | 0.303 | -0.261 | 0.186 |
| sp\|Q03431\|PTH1R_HUMAN Parathyroid hormone/parathyroid hormone-related peptide receptor OS=Homo | 576 | GLDEEAsGPER | 0.669 | 0.518 | -0.044 | 0.381 |
| sp\|Q03431\|PTH1R_HUMAN Parathyroid hormone/parathyroid hormone-related peptide receptor OS=Homo | 591 | LQEEWEtVM-- | 0.112 | 0.043 | -0.958 | -0.268 |
| sp\|P49190\|PTH2R_HUMAN Parathyroid hormone 2 receptor OS=Homo sapiens GN=PTH2R PE=1 SV=1 | 336 | FILFLNtVRVL | 0.345 | 0.181 | -0.526 | 0 |
| sp\|P49190\|PTH2R_HUMAN Parathyroid hormone 2 receptor OS=Homo sapiens GN=PTH2R PE=1 SV=1 | 342 | TVRVLAtKIWE | 0.159 | 0.117 | -0.692 | -0.139 |
| sp\|P49190\|PTH2R_HUMAN Parathyroid hormone 2 receptor OS=Homo sapiens GN=PTH2R PE=1 SV=1 | 347 | ATKIWEtNAVG | 0.181 | -0.115 | -1.205 | -0.38 |
| sp\|P49190\|PTH2R_HUMAN Parathyroid hormone 2 receptor OS=Homo sapiens GN=PTH2R PE=1 SV=1 | 354 | NAVGHDtRKQY | 0.163 | -0.212 | -1.167 | -0.405 |
| sp\|P49190\|PTH2R_HUMAN Parathyroid hormone 2 receptor OS=Homo sapiens GN=PTH2R PE=1 SV=1 | 364 | YRKLAKsTLVL | 0.421 | 0.396 | -0.124 | 0.231 |
| sp\|P49190\|PTH2R_HUMAN Parathyroid hormone 2 receptor OS=Homo sapiens GN=PTH2R PE=1 SV=1 | 365 | RKLAKStLVLV | 0.325 | 0.042 | -0.618 | -0.084 |
| sp\|P49190\|PTH2R_HUMAN Parathyroid hormone 2 receptor OS=Homo sapiens GN=PTH2R PE=1 SV=1 | 385 | FVCLPHsFTGL | 0.404 | 0.164 | -0.318 | 0.083 |
| sp\|P49190\|PTH2R_HUMAN Parathyroid hormone 2 receptor OS=Homo sapiens GN=PTH2R PE=1 SV=1 | 387 | CLPHSFtGLGW | 0.288 | 0.167 | -0.24 | 0.072 |
| sp\|P49190\|PTH2R_HUMAN Parathyroid hormone 2 receptor OS=Homo sapiens GN=PTH2R PE=1 SV=1 | 403 | CELFFNsFQGF | 0.411 | 0.054 | -0.394 | 0.024 |
| sp\|P49190\|PTH2R_HUMAN Parathyroid hormone 2 receptor OS=Homo sapiens GN=PTH2R PE=1 SV=1 | 410 | FQGFFVsIIYC | 0.116 | -0.137 | -1.081 | -0.367 |
| sp\|P49190\|PTH2R_HUMAN Parathyroid hormone 2 receptor OS=Homo sapiens GN=PTH2R PE=1 SV=1 | 429 | EVKKMWsRWNL | 0.377 | 0.002 | -0.569 | -0.063 |
| sp\|P49190\|PTH2R_HUMAN Parathyroid hormone 2 receptor OS=Homo sapiens GN=PTH2R PE=1 SV=1 | 434 | WSRWNLsVDWK | 0.389 | 0.075 | -0.079 | 0.128 |
| sp\|P49190\|PTH2R_HUMAN Parathyroid hormone 2 receptor OS=Homo sapiens GN=PTH2R PE=1 SV=1 | 440 | SVDWKRtPPCG | 0.122 | 0.042 | -1.096 | -0.311 |
| sp\|P49190\|PTH2R_HUMAN Parathyroid hormone 2 receptor OS=Homo sapiens GN=PTH2R PE=1 SV=1 | 445 | RTPPCGsRRCG | 0.142 | -0.278 | -1.325 | -0.487 |
| sp\|P49190\|PTH2R_HUMAN Parathyroid hormone 2 receptor OS=Homo sapiens GN=PTH2R PE=1 SV=1 | 450 | GSRRCGsVLTT | 0.725 | 0.931 | 0.391 | 0.682 |
| sp\|P49190\|PTH2R_HUMAN Parathyroid hormone 2 receptor OS=Homo sapiens GN=PTH2R PE=1 SV=1 | 453 | RCGSVLtTVTH | 0.068 | -0.247 | -1.681 | -0.62 |

| | | | | | | |
|---|---|---|---|---|---|---|
| sp\|P49190\|PTH2R_HUMAN Parathyroid hormone 2 receptor OS=Homo sapiens GN=PTH2R PE=1 SV=1 | 454 | CGSVLTtVTHS | 0.09 | -0.21 | -1.054 | -0.391 |
| sp\|P49190\|PTH2R_HUMAN Parathyroid hormone 2 receptor OS=Homo sapiens GN=PTH2R PE=1 SV=1 | 456 | SVLTTVtHSTS | 0.087 | -0.184 | -1.287 | -0.461 |
| sp\|P49190\|PTH2R_HUMAN Parathyroid hormone 2 receptor OS=Homo sapiens GN=PTH2R PE=1 SV=1 | 458 | LTTVTHsTSSQ | 0.05 | -0.105 | -1.43 | -0.495 |
| sp\|P49190\|PTH2R_HUMAN Parathyroid hormone 2 receptor OS=Homo sapiens GN=PTH2R PE=1 SV=1 | 459 | TTVTHStSSQS | 0.064 | -0.288 | -1.598 | -0.607 |
| sp\|P49190\|PTH2R_HUMAN Parathyroid hormone 2 receptor OS=Homo sapiens GN=PTH2R PE=1 SV=1 | 460 | TVTHSTsSQSQ | 0.136 | 0.021 | -0.701 | -0.181 |
| sp\|P49190\|PTH2R_HUMAN Parathyroid hormone 2 receptor OS=Homo sapiens GN=PTH2R PE=1 SV=1 | 461 | VTHSTSsQSQV | 0.036 | -0.379 | -2.09 | -0.811 |
| sp\|P49190\|PTH2R_HUMAN Parathyroid hormone 2 receptor OS=Homo sapiens GN=PTH2R PE=1 SV=1 | 463 | HSTSSQsQVAA | 0.288 | 0.139 | -0.306 | 0.04 |
| sp\|P49190\|PTH2R_HUMAN Parathyroid hormone 2 receptor OS=Homo sapiens GN=PTH2R PE=1 SV=1 | 468 | QSQVAAsTRMV | 0.2 | -0.115 | -1.111 | -0.342 |
| sp\|P49190\|PTH2R_HUMAN Parathyroid hormone 2 receptor OS=Homo sapiens GN=PTH2R PE=1 SV=1 | 469 | SQVAAStRMVL | 0.084 | -0.155 | -1.247 | -0.439 |
| sp\|P49190\|PTH2R_HUMAN Parathyroid hormone 2 receptor OS=Homo sapiens GN=PTH2R PE=1 SV=1 | 475 | TRMVLIsGKAA | 0.107 | 0.091 | -0.786 | -0.196 |
| sp\|P49190\|PTH2R_HUMAN Parathyroid hormone 2 receptor OS=Homo sapiens GN=PTH2R PE=1 SV=1 | 483 | KAAKIAsRQPD | 0.311 | -0.128 | -0.5 | -0.106 |
| sp\|P49190\|PTH2R_HUMAN Parathyroid hormone 2 receptor OS=Homo sapiens GN=PTH2R PE=1 SV=1 | 488 | ASRQPDsHITL | 0.296 | -0.022 | -0.485 | -0.07 |
| sp\|P49190\|PTH2R_HUMAN Parathyroid hormone 2 receptor OS=Homo sapiens GN=PTH2R PE=1 SV=1 | 491 | QPDSHItLPGY | 0.734 | 0.453 | 0.241 | 0.476 |
| sp\|P49190\|PTH2R_HUMAN Parathyroid hormone 2 receptor OS=Homo sapiens GN=PTH2R PE=1 SV=1 | 498 | LPGYVWsNSEQ | 0.087 | -0.252 | -1.425 | -0.53 |
| sp\|P49190\|PTH2R_HUMAN Parathyroid hormone 2 receptor OS=Homo sapiens GN=PTH2R PE=1 SV=1 | 500 | GYVWSNsEQDC | 0.751 | 0.379 | 0.117 | 0.416 |
| sp\|P49190\|PTH2R_HUMAN Parathyroid hormone 2 receptor OS=Homo sapiens GN=PTH2R PE=1 SV=1 | 508 | QDCLPHsFHEE | 0.269 | -0.015 | -0.712 | -0.153 |
| sp\|P49190\|PTH2R_HUMAN Parathyroid hormone 2 receptor OS=Homo sapiens GN=PTH2R PE=1 SV=1 | 513 | HSFHEEtKEDS | 0.146 | -0.117 | -1.05 | -0.34 |
| sp\|P49190\|PTH2R_HUMAN Parathyroid hormone 2 receptor OS=Homo sapiens GN=PTH2R PE=1 SV=1 | 517 | EETKEDsGRQG | 0.196 | -0.264 | -1.015 | -0.361 |
| sp\|P49190\|PTH2R_HUMAN Parathyroid hormone 2 receptor OS=Homo sapiens GN=PTH2R PE=1 SV=1 | 530 | ILMEKPsRPME | 0.338 | 0.27 | -0.45 | 0.053 |
| sp\|P49190\|PTH2R_HUMAN Parathyroid hormone 2 receptor OS=Homo sapiens GN=PTH2R PE=1 SV=1 | 535 | PSRPMEsNPDT | 0.704 | 0.483 | 0.101 | 0.429 |
| sp\|P49190\|PTH2R_HUMAN Parathyroid hormone 2 receptor OS=Homo sapiens GN=PTH2R PE=1 SV=1 | 539 | MESNPDtEGCQ | 0.181 | -0.054 | -0.888 | -0.254 |
| sp\|P49190\|PTH2R_HUMAN Parathyroid hormone 2 receptor OS=Homo sapiens GN=PTH2R PE=1 SV=1 | 546 | EGCQGEtEDVL | 0.18 | 0.016 | -0.711 | -0.172 |
| sp\|P41586\|PACR_HUMAN Pituitary adenylate cyclase-activating polypeptide type I receptor OS=Homo sapie | 316 | KGPVVGsIMVN | 0.048 | -0.257 | -1.601 | -0.603 |
| sp\|P41586\|PACR_HUMAN Pituitary adenylate cyclase-activating polypeptide type I receptor OS=Homo sapie | 337 | LVQKLQsPDMG | 0.126 | -0.266 | -1.033 | -0.391 |
| sp\|P41586\|PACR_HUMAN Pituitary adenylate cyclase-activating polypeptide type I receptor OS=Homo sapie | 345 | DMGGNEsSIYL | 0.071 | -0.058 | -1.296 | -0.428 |
| sp\|P41586\|PACR_HUMAN Pituitary adenylate cyclase-activating polypeptide type I receptor OS=Homo sapie | 346 | MGGNESsIYLR | 0.071 | -0.123 | -1.031 | -0.361 |
| sp\|P41586\|PACR_HUMAN Pituitary adenylate cyclase-activating polypeptide type I receptor OS=Homo sapie | 354 | YLRLARsTLLL | 0.425 | 0.39 | -0.373 | 0.147 |
| sp\|P41586\|PACR_HUMAN Pituitary adenylate cyclase-activating polypeptide type I receptor OS=Homo sapie | 355 | LRLARStLLLI | 0.46 | 0.373 | 0.029 | 0.287 |
| sp\|P41586\|PACR_HUMAN Pituitary adenylate cyclase-activating polypeptide type I receptor OS=Homo sapie | 367 | LFGIHYtVFAF | 0.215 | 0.182 | -0.233 | 0.055 |
| sp\|P41586\|PACR_HUMAN Pituitary adenylate cyclase-activating polypeptide type I receptor OS=Homo sapie | 372 | YTVFAFsPENV | 0.15 | -0.166 | -1.296 | -0.437 |
| sp\|P41586\|PACR_HUMAN Pituitary adenylate cyclase-activating polypeptide type I receptor OS=Homo sapie | 377 | FSPENVsKRER | 0.253 | -0.129 | -0.645 | -0.174 |
| sp\|P41586\|PACR_HUMAN Pituitary adenylate cyclase-activating polypeptide type I receptor OS=Homo sapie | 390 | FELGLGsFQGF | 0.122 | -0.093 | -0.934 | -0.302 |
| sp\|P41586\|PACR_HUMAN Pituitary adenylate cyclase-activating polypeptide type I receptor OS=Homo sapie | 417 | IKRKWRsWKVN | 0.676 | 0.485 | 0.14 | 0.434 |
| sp\|P41586\|PACR_HUMAN Pituitary adenylate cyclase-activating polypeptide type I receptor OS=Homo sapie | 434 | FKHRHPsLASS | 0.687 | 0.921 | 0.403 | 0.67 |
| sp\|P41586\|PACR_HUMAN Pituitary adenylate cyclase-activating polypeptide type I receptor OS=Homo sapie | 437 | RHPSLAsSGVN | 0.133 | -0.082 | -1.485 | -0.478 |
| sp\|P41586\|PACR_HUMAN Pituitary adenylate cyclase-activating polypeptide type I receptor OS=Homo sapie | 438 | HPSLASsGVNG | 0.117 | -0.051 | -1.126 | -0.353 |
| sp\|P41586\|PACR_HUMAN Pituitary adenylate cyclase-activating polypeptide type I receptor OS=Homo sapie | 444 | SGVNGGtQLSI | 0.024 | -0.351 | -1.871 | -0.733 |
| sp\|P41586\|PACR_HUMAN Pituitary adenylate cyclase-activating polypeptide type I receptor OS=Homo sapie | 447 | NGGTQLsILSK | 0.026 | -0.329 | -1.791 | -0.698 |
| sp\|P41586\|PACR_HUMAN Pituitary adenylate cyclase-activating polypeptide type I receptor OS=Homo sapie | 450 | TQLSILsKSSS | 0.027 | -0.338 | -1.435 | -0.582 |
| sp\|P41586\|PACR_HUMAN Pituitary adenylate cyclase-activating polypeptide type I receptor OS=Homo sapie | 452 | LSILSKsSSQI | 0.326 | 0.132 | -0.192 | 0.089 |
| sp\|P41586\|PACR_HUMAN Pituitary adenylate cyclase-activating polypeptide type I receptor OS=Homo sapie | 453 | SILSKSsSQIR | 0.076 | -0.225 | -1.347 | -0.499 |
| sp\|P41586\|PACR_HUMAN Pituitary adenylate cyclase-activating polypeptide type I receptor OS=Homo sapie | 454 | ILSKSSsQIRM | 0.446 | 0.09 | -0.286 | 0.083 |
| sp\|P41586\|PACR_HUMAN Pituitary adenylate cyclase-activating polypeptide type I receptor OS=Homo sapie | 459 | SSQIRMsGLPA | 0.164 | 0.036 | -0.867 | -0.222 |
| sp\|P41586\|PACR_HUMAN Pituitary adenylate cyclase-activating polypeptide type I receptor OS=Homo sapie | 468 | PADNLAt---- | 0.141 | 0.023 | -1.029 | -0.288 |
| sp\|P32241\|VIPR1_HUMAN Vasoactive intestinal polypeptide receptor 1 OS=Homo sapiens GN=VIPR1 PE=1 S | 331 | PPDIRKsDSSP | 0.148 | 0.004 | -1 | -0.283 |
| sp\|P32241\|VIPR1_HUMAN Vasoactive intestinal polypeptide receptor 1 OS=Homo sapiens GN=VIPR1 PE=1 S | 333 | DIRKSDsSPYS | 0.897 | 0.874 | 0.556 | 0.776 |
| sp\|P32241\|VIPR1_HUMAN Vasoactive intestinal polypeptide receptor 1 OS=Homo sapiens GN=VIPR1 PE=1 S | 334 | IRKSDSsPYSR | 0.061 | -0.271 | -1.258 | -0.489 |
| sp\|P32241\|VIPR1_HUMAN Vasoactive intestinal polypeptide receptor 1 OS=Homo sapiens GN=VIPR1 PE=1 S | 337 | SDSSPYsRLAR | 0.049 | -0.321 | -1.37 | -0.547 |
| sp\|P32241\|VIPR1_HUMAN Vasoactive intestinal polypeptide receptor 1 OS=Homo sapiens GN=VIPR1 PE=1 S | 342 | YSRLARsTLLL | 0.264 | 0.229 | -0.69 | -0.066 |

| | | | | | | |
|---|---|---|---|---|---|---|
| sp\|P32241\|VIPR1_HUMAN Vasoactive intestinal polypeptide receptor 1 OS=Homo sapiens GN=VIPR1 PE=1 S\ | 343 | SRLARStLLLI | 0.375 | 0.321 | -0.257 | 0.146 |
| sp\|P32241\|VIPR1_HUMAN Vasoactive intestinal polypeptide receptor 1 OS=Homo sapiens GN=VIPR1 PE=1 S\ | 378 | FELVVGsFQGF | 0.141 | -0.075 | -0.938 | -0.291 |
| sp\|P32241\|VIPR1_HUMAN Vasoactive intestinal polypeptide receptor 1 OS=Homo sapiens GN=VIPR1 PE=1 S\ | 422 | PKYRHPsGGSN | 0.716 | 0.901 | 0.498 | 0.705 |
| sp\|P32241\|VIPR1_HUMAN Vasoactive intestinal polypeptide receptor 1 OS=Homo sapiens GN=VIPR1 PE=1 S\ | 425 | RHPSGGsNGAT | 0.056 | -0.25 | -1.674 | -0.623 |
| sp\|P32241\|VIPR1_HUMAN Vasoactive intestinal polypeptide receptor 1 OS=Homo sapiens GN=VIPR1 PE=1 S\ | 429 | GGSNGAtCSTQ | 0.096 | -0.145 | -1.144 | -0.398 |
| sp\|P32241\|VIPR1_HUMAN Vasoactive intestinal polypeptide receptor 1 OS=Homo sapiens GN=VIPR1 PE=1 S\ | 431 | SNGATCsTQVS | 0.061 | -0.195 | -1.516 | -0.55 |
| sp\|P32241\|VIPR1_HUMAN Vasoactive intestinal polypeptide receptor 1 OS=Homo sapiens GN=VIPR1 PE=1 S\ | 432 | NGATCStQVSM | 0.094 | -0.234 | -1.235 | -0.458 |
| sp\|P32241\|VIPR1_HUMAN Vasoactive intestinal polypeptide receptor 1 OS=Homo sapiens GN=VIPR1 PE=1 S\ | 435 | TCSTQVsMLTR | 0.182 | -0.018 | -1.158 | -0.331 |
| sp\|P32241\|VIPR1_HUMAN Vasoactive intestinal polypeptide receptor 1 OS=Homo sapiens GN=VIPR1 PE=1 S\ | 438 | TQVSMLtRVSP | 0.022 | -0.451 | -1.811 | -0.747 |
| sp\|P32241\|VIPR1_HUMAN Vasoactive intestinal polypeptide receptor 1 OS=Homo sapiens GN=VIPR1 PE=1 S\ | 441 | SMLTRVsPGAR | 0.29 | -0.014 | -0.594 | -0.106 |
| sp\|P32241\|VIPR1_HUMAN Vasoactive intestinal polypeptide receptor 1 OS=Homo sapiens GN=VIPR1 PE=1 S\ | 447 | SPGARRsSSFQ | 0.037 | -0.286 | -1.943 | -0.731 |
| sp\|P32241\|VIPR1_HUMAN Vasoactive intestinal polypeptide receptor 1 OS=Homo sapiens GN=VIPR1 PE=1 S\ | 448 | PGARRSsSFQA | 0.685 | 0.855 | 0.651 | 0.73 |
| sp\|P32241\|VIPR1_HUMAN Vasoactive intestinal polypeptide receptor 1 OS=Homo sapiens GN=VIPR1 PE=1 S\ | 449 | GARRSSsFQAE | 0.93 | 1.363 | 1.406 | 1.233 |
| sp\|P32241\|VIPR1_HUMAN Vasoactive intestinal polypeptide receptor 1 OS=Homo sapiens GN=VIPR1 PE=1 S\ | 455 | SFQAEVsLV-- | 0.399 | 0.15 | -0.482 | 0.022 |
| sp\|P41587\|VIPR2_HUMAN Vasoactive intestinal polypeptide receptor 2 OS=Homo sapiens GN=VIPR2 PE=1 S\ | 321 | VGGNDQsQYKR | 0.066 | -0.215 | -1.239 | -0.463 |
| sp\|P41587\|VIPR2_HUMAN Vasoactive intestinal polypeptide receptor 2 OS=Homo sapiens GN=VIPR2 PE=1 S\ | 329 | YKRLAKsTLLL | 0.658 | 0.451 | 0.216 | 0.442 |
| sp\|P41587\|VIPR2_HUMAN Vasoactive intestinal polypeptide receptor 2 OS=Homo sapiens GN=VIPR2 PE=1 S\ | 330 | KRLAKStLLLI | 0.22 | 0.14 | -0.548 | -0.063 |
| sp\|P41587\|VIPR2_HUMAN Vasoactive intestinal polypeptide receptor 2 OS=Homo sapiens GN=VIPR2 PE=1 S\ | 350 | FAVFPIsISSK | 0.062 | -0.271 | -1.288 | -0.499 |
| sp\|P41587\|VIPR2_HUMAN Vasoactive intestinal polypeptide receptor 2 OS=Homo sapiens GN=VIPR2 PE=1 S\ | 352 | VFPISIsSKYQ | 0.167 | -0.022 | -0.8 | -0.218 |
| sp\|P41587\|VIPR2_HUMAN Vasoactive intestinal polypeptide receptor 2 OS=Homo sapiens GN=VIPR2 PE=1 S\ | 353 | FPISISsKYQI | 0.152 | -0.065 | -0.698 | -0.204 |
| sp\|P41587\|VIPR2_HUMAN Vasoactive intestinal polypeptide receptor 2 OS=Homo sapiens GN=VIPR2 PE=1 S\ | 365 | FELCLGsFQGL | 0.226 | -0.03 | -0.822 | -0.209 |
| sp\|P41587\|VIPR2_HUMAN Vasoactive intestinal polypeptide receptor 2 OS=Homo sapiens GN=VIPR2 PE=1 S\ | 380 | LYCFLNsEVQC | 0.269 | 0.02 | -0.919 | -0.21 |
| sp\|P41587\|VIPR2_HUMAN Vasoactive intestinal polypeptide receptor 2 OS=Homo sapiens GN=VIPR2 PE=1 S\ | 392 | LKRKWRsRCPT | 0.385 | 0.081 | -0.471 | -0.002 |
| sp\|P41587\|VIPR2_HUMAN Vasoactive intestinal polypeptide receptor 2 OS=Homo sapiens GN=VIPR2 PE=1 S\ | 396 | WRSRCPtPSAS | 0.42 | 0.661 | 0.033 | 0.371 |
| sp\|P41587\|VIPR2_HUMAN Vasoactive intestinal polypeptide receptor 2 OS=Homo sapiens GN=VIPR2 PE=1 S\ | 398 | SRCPTPsASRD | 0.098 | -0.025 | -1.382 | -0.436 |
| sp\|P41587\|VIPR2_HUMAN Vasoactive intestinal polypeptide receptor 2 OS=Homo sapiens GN=VIPR2 PE=1 S\ | 400 | CPTPSAsRDYR | 0.422 | 0.098 | -0.251 | 0.09 |
| sp\|P41587\|VIPR2_HUMAN Vasoactive intestinal polypeptide receptor 2 OS=Homo sapiens GN=VIPR2 PE=1 S\ | 408 | DYRVCGsSFSR | 0.276 | 0.101 | -0.786 | -0.136 |
| sp\|P41587\|VIPR2_HUMAN Vasoactive intestinal polypeptide receptor 2 OS=Homo sapiens GN=VIPR2 PE=1 S\ | 409 | YRVCGSsFSRN | 0.115 | 0.005 | -1.191 | -0.357 |
| sp\|P41587\|VIPR2_HUMAN Vasoactive intestinal polypeptide receptor 2 OS=Homo sapiens GN=VIPR2 PE=1 S\ | 411 | VCGSSFsRNGS | 0.239 | 0.03 | -0.612 | -0.114 |
| sp\|P41587\|VIPR2_HUMAN Vasoactive intestinal polypeptide receptor 2 OS=Homo sapiens GN=VIPR2 PE=1 S\ | 415 | SFSRNGsEGAL | 0.697 | 0.938 | 0.605 | 0.747 |
| sp\|P41587\|VIPR2_HUMAN Vasoactive intestinal polypeptide receptor 2 OS=Homo sapiens GN=VIPR2 PE=1 S\ | 425 | LQFHRGsRAQS | 0.119 | -0.19 | -1.08 | -0.384 |
| sp\|P41587\|VIPR2_HUMAN Vasoactive intestinal polypeptide receptor 2 OS=Homo sapiens GN=VIPR2 PE=1 S\ | 429 | RGSRAQsFLQT | 0.699 | 0.897 | 0.659 | 0.752 |
| sp\|P41587\|VIPR2_HUMAN Vasoactive intestinal polypeptide receptor 2 OS=Homo sapiens GN=VIPR2 PE=1 S\ | 433 | AQSFLQtETSV | 0.109 | -0.113 | -1.27 | -0.425 |
| sp\|P41587\|VIPR2_HUMAN Vasoactive intestinal polypeptide receptor 2 OS=Homo sapiens GN=VIPR2 PE=1 S\ | 435 | SFLQTEtSVI- | 0.104 | -0.101 | -1.164 | -0.387 |
| sp\|P41587\|VIPR2_HUMAN Vasoactive intestinal polypeptide receptor 2 OS=Homo sapiens GN=VIPR2 PE=1 S\ | 436 | FLQTETsVI-- | 0.313 | 0.283 | -0.515 | 0.027 |
| sp\|O14514\|AGRB1_HUMAN Adhesion G protein-coupled receptor B1 OS=Homo sapiens GN=ADGRB1 PE=1 S | 320 | CGPAGRtSSRS | 0.037 | -0.404 | -1.837 | -0.735 |
| sp\|O14514\|AGRB1_HUMAN Adhesion G protein-coupled receptor B1 OS=Homo sapiens GN=ADGRB1 PE=1 S | 321 | GPAGRTsSRSQ | 0.157 | -0.081 | -0.883 | -0.269 |
| sp\|O14514\|AGRB1_HUMAN Adhesion G protein-coupled receptor B1 OS=Homo sapiens GN=ADGRB1 PE=1 S | 322 | PAGRTSsRSQS | 0.164 | 0.554 | -0.621 | 0.032 |
| sp\|O14514\|AGRB1_HUMAN Adhesion G protein-coupled receptor B1 OS=Homo sapiens GN=ADGRB1 PE=1 S | 324 | GRTSSRsQSLR | 0.266 | 0.292 | -0.711 | -0.051 |
| sp\|O14514\|AGRB1_HUMAN Adhesion G protein-coupled receptor B1 OS=Homo sapiens GN=ADGRB1 PE=1 S | 326 | TSSRSQsLRST | 0.704 | 0.895 | 0.771 | 0.79 |
| sp\|O14514\|AGRB1_HUMAN Adhesion G protein-coupled receptor B1 OS=Homo sapiens GN=ADGRB1 PE=1 S | 329 | RSQSLRsTDAR | 0.175 | -0.02 | -0.935 | -0.26 |
| sp\|O14514\|AGRB1_HUMAN Adhesion G protein-coupled receptor B1 OS=Homo sapiens GN=ADGRB1 PE=1 S | 330 | SQSLRStDARR | 0.123 | -0.15 | -1.279 | -0.435 |
| sp\|O14514\|AGRB1_HUMAN Adhesion G protein-coupled receptor B1 OS=Homo sapiens GN=ADGRB1 PE=1 S | 352 | GFPAPQtGDPA | 0.146 | -0.224 | -0.705 | -0.261 |
| sp\|O14514\|AGRB1_HUMAN Adhesion G protein-coupled receptor B1 OS=Homo sapiens GN=ADGRB1 PE=1 S | 361 | PAAEEWsPWSV | 0.054 | -0.453 | -1.823 | -0.741 |
| sp\|O14514\|AGRB1_HUMAN Adhesion G protein-coupled receptor B1 OS=Homo sapiens GN=ADGRB1 PE=1 S | 364 | EEWSPWsVCSS | 0.029 | -0.297 | -1.658 | -0.642 |
| sp\|O14514\|AGRB1_HUMAN Adhesion G protein-coupled receptor B1 OS=Homo sapiens GN=ADGRB1 PE=1 S | 367 | SPWSVCsSTCG | 0.119 | -0.074 | -1.508 | -0.488 |
| sp\|O14514\|AGRB1_HUMAN Adhesion G protein-coupled receptor B1 OS=Homo sapiens GN=ADGRB1 PE=1 S | 368 | PWSVCSsTCGE | 0.094 | -0.272 | -1.238 | -0.472 |
| sp\|O14514\|AGRB1_HUMAN Adhesion G protein-coupled receptor B1 OS=Homo sapiens GN=ADGRB1 PE=1 S | 369 | WSVCSStCGEG | 0.416 | 0.167 | -0.328 | 0.085 |
| sp\|O14514\|AGRB1_HUMAN Adhesion G protein-coupled receptor B1 OS=Homo sapiens GN=ADGRB1 PE=1 S | 376 | CGEGWQtRTRF | 0.2 | -0.18 | -0.756 | -0.245 |

| | | | | | | |
|---|---|---|---|---|---|---|
| sp\|O14514\|AGRB1_HUMAN Adhesion G protein-coupled receptor B1 OS=Homo sapiens GN=ADGRB1 PE=1 S' | 378 | EGWQTRtRFCV | 0.075 | 0.039 | -1.256 | -0.381 |
| sp\|O14514\|AGRB1_HUMAN Adhesion G protein-coupled receptor B1 OS=Homo sapiens GN=ADGRB1 PE=1 S' | 383 | RTRFCVsSSYS | 0.519 | 0.132 | -0.58 | 0.024 |
| sp\|O14514\|AGRB1_HUMAN Adhesion G protein-coupled receptor B1 OS=Homo sapiens GN=ADGRB1 PE=1 S' | 384 | TRFCVSsSYST | 0.081 | -0.039 | -1.361 | -0.44 |
| sp\|O14514\|AGRB1_HUMAN Adhesion G protein-coupled receptor B1 OS=Homo sapiens GN=ADGRB1 PE=1 S' | 385 | RFCVSSsYSTQ | 0.123 | -0.024 | -0.972 | -0.291 |
| sp\|O14514\|AGRB1_HUMAN Adhesion G protein-coupled receptor B1 OS=Homo sapiens GN=ADGRB1 PE=1 S' | 387 | CVSSSYsTQCS | 0.277 | 0.232 | -0.286 | 0.074 |
| sp\|O14514\|AGRB1_HUMAN Adhesion G protein-coupled receptor B1 OS=Homo sapiens GN=ADGRB1 PE=1 S' | 388 | VSSSYStQCSG | 0.04 | -0.475 | -1.868 | -0.768 |
| sp\|O14514\|AGRB1_HUMAN Adhesion G protein-coupled receptor B1 OS=Homo sapiens GN=ADGRB1 PE=1 S' | 391 | SYSTQCsGPLR | 0.451 | 0.372 | -0.514 | 0.103 |
| sp\|O14514\|AGRB1_HUMAN Adhesion G protein-coupled receptor B1 OS=Homo sapiens GN=ADGRB1 PE=1 S' | 403 | QRLCNNsAVCP | 0.291 | 0.234 | -0.747 | -0.074 |
| sp\|O14514\|AGRB1_HUMAN Adhesion G protein-coupled receptor B1 OS=Homo sapiens GN=ADGRB1 PE=1 S' | 416 | GAWDEWsPWSL | 0.086 | -0.246 | -1.557 | -0.572 |
| sp\|O14514\|AGRB1_HUMAN Adhesion G protein-coupled receptor B1 OS=Homo sapiens GN=ADGRB1 PE=1 S' | 419 | DEWSPWsLCSS | 0.088 | -0.212 | -1.322 | -0.482 |
| sp\|O14514\|AGRB1_HUMAN Adhesion G protein-coupled receptor B1 OS=Homo sapiens GN=ADGRB1 PE=1 S' | 422 | SPWSLCsSTCG | 0.082 | -0.116 | -1.675 | -0.57 |
| sp\|O14514\|AGRB1_HUMAN Adhesion G protein-coupled receptor B1 OS=Homo sapiens GN=ADGRB1 PE=1 S' | 423 | PWSLCSsTCGR | 0.15 | -0.198 | -0.996 | -0.348 |
| sp\|O14514\|AGRB1_HUMAN Adhesion G protein-coupled receptor B1 OS=Homo sapiens GN=ADGRB1 PE=1 S' | 424 | WSLCSStCGRG | 0.4 | 0.072 | -0.431 | 0.014 |
| sp\|O14514\|AGRB1_HUMAN Adhesion G protein-coupled receptor B1 OS=Homo sapiens GN=ADGRB1 PE=1 S' | 433 | RGFRDRtRTCR | 0.545 | 0.784 | -0.076 | 0.418 |
| sp\|O14514\|AGRB1_HUMAN Adhesion G protein-coupled receptor B1 OS=Homo sapiens GN=ADGRB1 PE=1 S' | 435 | FRDRTRtCRPP | 0.421 | 1.029 | -0.076 | 0.458 |
| sp\|O14514\|AGRB1_HUMAN Adhesion G protein-coupled receptor B1 OS=Homo sapiens GN=ADGRB1 PE=1 S' | 453 | EGPEKQtKFCN | 0.054 | -0.122 | -1.198 | -0.422 |
| sp\|O14514\|AGRB1_HUMAN Adhesion G protein-coupled receptor B1 OS=Homo sapiens GN=ADGRB1 PE=1 S' | 474 | GNWNEWsSWSA | 0.107 | -0.114 | -1.156 | -0.388 |
| sp\|O14514\|AGRB1_HUMAN Adhesion G protein-coupled receptor B1 OS=Homo sapiens GN=ADGRB1 PE=1 S' | 475 | NWNEWSsWSAC | 0.063 | -0.047 | -1.431 | -0.472 |
| sp\|O14514\|AGRB1_HUMAN Adhesion G protein-coupled receptor B1 OS=Homo sapiens GN=ADGRB1 PE=1 S' | 477 | NEWSSWsACSA | 0.142 | 0.053 | -1.003 | -0.269 |
| sp\|O14514\|AGRB1_HUMAN Adhesion G protein-coupled receptor B1 OS=Homo sapiens GN=ADGRB1 PE=1 S' | 480 | SSWSACsASCS | 0.102 | -0.014 | -1.505 | -0.472 |
| sp\|O14514\|AGRB1_HUMAN Adhesion G protein-coupled receptor B1 OS=Homo sapiens GN=ADGRB1 PE=1 S' | 482 | WSACSAsCSQG | 0.408 | 0.116 | -0.271 | 0.084 |
| sp\|O14514\|AGRB1_HUMAN Adhesion G protein-coupled receptor B1 OS=Homo sapiens GN=ADGRB1 PE=1 S' | 484 | ACSASCsQGRQ | 0.495 | 0.022 | -0.86 | -0.114 |
| sp\|O14514\|AGRB1_HUMAN Adhesion G protein-coupled receptor B1 OS=Homo sapiens GN=ADGRB1 PE=1 S' | 491 | QGRQQRtRECN | 0.181 | 0.123 | -0.948 | -0.215 |
| sp\|O14514\|AGRB1_HUMAN Adhesion G protein-coupled receptor B1 OS=Homo sapiens GN=ADGRB1 PE=1 S' | 498 | RECNGPsYGGA | 0.13 | -0.217 | -0.974 | -0.354 |
| sp\|O14514\|AGRB1_HUMAN Adhesion G protein-coupled receptor B1 OS=Homo sapiens GN=ADGRB1 PE=1 S' | 511 | QGHWVEtRDCF | 0.107 | -0.217 | -1.203 | -0.438 |
| sp\|O14514\|AGRB1_HUMAN Adhesion G protein-coupled receptor B1 OS=Homo sapiens GN=ADGRB1 PE=1 S' | 530 | KWQAWAsWGSC | 0.193 | 0.08 | -0.906 | -0.211 |
| sp\|O14514\|AGRB1_HUMAN Adhesion G protein-coupled receptor B1 OS=Homo sapiens GN=ADGRB1 PE=1 S' | 533 | AWASWGsCSVT | 0.078 | -0.142 | -1.289 | -0.451 |
| sp\|O14514\|AGRB1_HUMAN Adhesion G protein-coupled receptor B1 OS=Homo sapiens GN=ADGRB1 PE=1 S' | 535 | ASWGSCsVTCG | 0.392 | 0.269 | -0.461 | 0.067 |
| sp\|O14514\|AGRB1_HUMAN Adhesion G protein-coupled receptor B1 OS=Homo sapiens GN=ADGRB1 PE=1 S' | 537 | WGSCSVtCGAG | 0.717 | 0.313 | 0.369 | 0.466 |
| sp\|O14514\|AGRB1_HUMAN Adhesion G protein-coupled receptor B1 OS=Homo sapiens GN=ADGRB1 PE=1 S' | 542 | VTCGAGsQRRE | 0.149 | -0.325 | -1.449 | -0.542 |
| sp\|O14514\|AGRB1_HUMAN Adhesion G protein-coupled receptor B1 OS=Homo sapiens GN=ADGRB1 PE=1 S' | 550 | RRERVCsGPFF | 0.898 | 1.454 | 0.948 | 1.1 |
| sp\|O14514\|AGRB1_HUMAN Adhesion G protein-coupled receptor B1 OS=Homo sapiens GN=ADGRB1 PE=1 S' | 571 | EYRQCGtQRCP | 0.325 | 0.069 | -0.694 | -0.1 |
| sp\|O14514\|AGRB1_HUMAN Adhesion G protein-coupled receptor B1 OS=Homo sapiens GN=ADGRB1 PE=1 S' | 594 | AVIWKEtPAGE | 0.037 | -0.604 | -1.776 | -0.781 |
| sp\|O14514\|AGRB1_HUMAN Adhesion G protein-coupled receptor B1 OS=Homo sapiens GN=ADGRB1 PE=1 S' | 609 | RCPRNAtGLIL | 0.66 | 0.864 | 0.155 | 0.56 |
| sp\|O14514\|AGRB1_HUMAN Adhesion G protein-coupled receptor B1 OS=Homo sapiens GN=ADGRB1 PE=1 S' | 630 | AYWEPPtYIRC | 0.046 | -0.284 | -1.633 | -0.624 |
| sp\|O14514\|AGRB1_HUMAN Adhesion G protein-coupled receptor B1 OS=Homo sapiens GN=ADGRB1 PE=1 S' | 636 | TYIRCVsIDYR | 0.807 | 0.893 | 0.677 | 0.792 |
| sp\|O14514\|AGRB1_HUMAN Adhesion G protein-coupled receptor B1 OS=Homo sapiens GN=ADGRB1 PE=1 S' | 646 | RNIQMMtREHL | 0.17 | -0.087 | -0.702 | -0.206 |
| sp\|O14514\|AGRB1_HUMAN Adhesion G protein-coupled receptor B1 OS=Homo sapiens GN=ADGRB1 PE=1 S' | 663 | LPGEGVsEVIQ | 0.083 | -0.168 | -1.279 | -0.455 |
| sp\|O14514\|AGRB1_HUMAN Adhesion G protein-coupled receptor B1 OS=Homo sapiens GN=ADGRB1 PE=1 S' | 668 | VSEVIQtLVEI | 0.309 | 0.013 | -0.206 | 0.039 |
| sp\|O14514\|AGRB1_HUMAN Adhesion G protein-coupled receptor B1 OS=Homo sapiens GN=ADGRB1 PE=1 S' | 673 | QTLVEIsQDGT | 0.127 | -0.261 | -1.226 | -0.453 |
| sp\|O14514\|AGRB1_HUMAN Adhesion G protein-coupled receptor B1 OS=Homo sapiens GN=ADGRB1 PE=1 S' | 677 | EISQDGtSYSG | 0.068 | -0.194 | -1.282 | -0.469 |
| sp\|O14514\|AGRB1_HUMAN Adhesion G protein-coupled receptor B1 OS=Homo sapiens GN=ADGRB1 PE=1 S' | 678 | ISQDGTsYSGD | 0.104 | -0.236 | -1.021 | -0.384 |
| sp\|O14514\|AGRB1_HUMAN Adhesion G protein-coupled receptor B1 OS=Homo sapiens GN=ADGRB1 PE=1 S' | 680 | QDGTSYsGDLL | 0.181 | 0.152 | -0.43 | -0.032 |
| sp\|O14514\|AGRB1_HUMAN Adhesion G protein-coupled receptor B1 OS=Homo sapiens GN=ADGRB1 PE=1 S' | 685 | YSGDLLsTIDV | 0.073 | -0.156 | -1.43 | -0.504 |
| sp\|O14514\|AGRB1_HUMAN Adhesion G protein-coupled receptor B1 OS=Homo sapiens GN=ADGRB1 PE=1 S' | 686 | SGDLLStIDVL | 0.052 | -0.201 | -1.446 | -0.532 |
| sp\|O14514\|AGRB1_HUMAN Adhesion G protein-coupled receptor B1 OS=Homo sapiens GN=ADGRB1 PE=1 S' | 694 | DVLRNMtEIFR | 0.692 | 0.98 | 0.619 | 0.764 |
| sp\|O14514\|AGRB1_HUMAN Adhesion G protein-coupled receptor B1 OS=Homo sapiens GN=ADGRB1 PE=1 S' | 703 | FRRAYYsPTPG | 0.399 | 0.224 | -0.327 | 0.099 |
| sp\|O14514\|AGRB1_HUMAN Adhesion G protein-coupled receptor B1 OS=Homo sapiens GN=ADGRB1 PE=1 S' | 705 | RAYYSPtPGDV | 0.322 | -0.032 | -0.558 | -0.089 |
| sp\|O14514\|AGRB1_HUMAN Adhesion G protein-coupled receptor B1 OS=Homo sapiens GN=ADGRB1 PE=1 S' | 717 | NFVQILsNLLA | 0.101 | -0.126 | -0.905 | -0.31 |

| | | | | | | |
|---|---|---|---|---|---|---|
| sp\|O14514\|AGRB1_HUMAN Adhesion G protein-coupled receptor B1 OS=Homo sapiens GN=ADGRB1 PE=1 S' | 766 | RDAYQVtDNLV | 0.283 | 0.026 | -0.741 | -0.144 |
| sp\|O14514\|AGRB1_HUMAN Adhesion G protein-coupled receptor B1 OS=Homo sapiens GN=ADGRB1 PE=1 S' | 772 | TDNLVLsIHKL | 0.047 | -0.241 | -1.568 | -0.587 |
| sp\|O14514\|AGRB1_HUMAN Adhesion G protein-coupled receptor B1 OS=Homo sapiens GN=ADGRB1 PE=1 S' | 779 | IHKLPAsGATD | 0.206 | -0.176 | -0.882 | -0.284 |
| sp\|O14514\|AGRB1_HUMAN Adhesion G protein-coupled receptor B1 OS=Homo sapiens GN=ADGRB1 PE=1 S' | 782 | LPASGAtDISF | 0.051 | -0.217 | -1.287 | -0.484 |
| sp\|O14514\|AGRB1_HUMAN Adhesion G protein-coupled receptor B1 OS=Homo sapiens GN=ADGRB1 PE=1 S' | 785 | SGATDIsFPMK | 0.472 | 0.283 | -0.129 | 0.209 |
| sp\|O14514\|AGRB1_HUMAN Adhesion G protein-coupled receptor B1 OS=Homo sapiens GN=ADGRB1 PE=1 S' | 794 | MKGWRAtGDWA | 0.636 | 0.252 | 0.219 | 0.369 |
| sp\|O14514\|AGRB1_HUMAN Adhesion G protein-coupled receptor B1 OS=Homo sapiens GN=ADGRB1 PE=1 S' | 806 | VPEDRVtVSKS | 0.222 | -0.06 | -0.933 | -0.257 |
| sp\|O14514\|AGRB1_HUMAN Adhesion G protein-coupled receptor B1 OS=Homo sapiens GN=ADGRB1 PE=1 S' | 808 | EDRVTVsKSVF | 0.1 | 0.051 | -0.899 | -0.249 |
| sp\|O14514\|AGRB1_HUMAN Adhesion G protein-coupled receptor B1 OS=Homo sapiens GN=ADGRB1 PE=1 S' | 810 | RVTVSKsVFST | 0.228 | 0.206 | -0.217 | 0.072 |
| sp\|O14514\|AGRB1_HUMAN Adhesion G protein-coupled receptor B1 OS=Homo sapiens GN=ADGRB1 PE=1 S' | 813 | VSKSVFsTGLT | 0.203 | -0.123 | -0.714 | -0.211 |
| sp\|O14514\|AGRB1_HUMAN Adhesion G protein-coupled receptor B1 OS=Homo sapiens GN=ADGRB1 PE=1 S' | 814 | SKSVFStGLTE | 0.056 | -0.316 | -1.636 | -0.632 |
| sp\|O14514\|AGRB1_HUMAN Adhesion G protein-coupled receptor B1 OS=Homo sapiens GN=ADGRB1 PE=1 S' | 817 | VFSTGLtEADE | 0.107 | -0.169 | -1.039 | -0.367 |
| sp\|O14514\|AGRB1_HUMAN Adhesion G protein-coupled receptor B1 OS=Homo sapiens GN=ADGRB1 PE=1 S' | 823 | TEADEAsVFVV | 0.211 | -0.032 | -1.161 | -0.327 |
| sp\|O14514\|AGRB1_HUMAN Adhesion G protein-coupled receptor B1 OS=Homo sapiens GN=ADGRB1 PE=1 S' | 829 | SVFVVGtVLYR | 0.053 | -0.231 | -1.742 | -0.64 |
| sp\|O14514\|AGRB1_HUMAN Adhesion G protein-coupled receptor B1 OS=Homo sapiens GN=ADGRB1 PE=1 S' | 837 | LYRNLGsFLAL | 0.284 | 0.275 | -0.318 | 0.08 |
| sp\|O14514\|AGRB1_HUMAN Adhesion G protein-coupled receptor B1 OS=Homo sapiens GN=ADGRB1 PE=1 S' | 845 | LALQRNtTVLN | 0.377 | 0.134 | -0.45 | 0.02 |
| sp\|O14514\|AGRB1_HUMAN Adhesion G protein-coupled receptor B1 OS=Homo sapiens GN=ADGRB1 PE=1 S' | 846 | ALQRNTtVLNS | 0.69 | 0.964 | 0.298 | 0.651 |
| sp\|O14514\|AGRB1_HUMAN Adhesion G protein-coupled receptor B1 OS=Homo sapiens GN=ADGRB1 PE=1 S' | 850 | NTTVLNsKVIS | 0.141 | -0.108 | -1.158 | -0.375 |
| sp\|O14514\|AGRB1_HUMAN Adhesion G protein-coupled receptor B1 OS=Homo sapiens GN=ADGRB1 PE=1 S' | 854 | LNSKVIsVTVK | 0.303 | -0.131 | -0.618 | -0.149 |
| sp\|O14514\|AGRB1_HUMAN Adhesion G protein-coupled receptor B1 OS=Homo sapiens GN=ADGRB1 PE=1 S' | 856 | SKVISVtVKPP | 0.312 | 0.097 | -0.428 | -0.006 |
| sp\|O14514\|AGRB1_HUMAN Adhesion G protein-coupled receptor B1 OS=Homo sapiens GN=ADGRB1 PE=1 S' | 863 | VKPPPRsLRTP | 0.149 | -0.262 | -0.884 | -0.332 |
| sp\|O14514\|AGRB1_HUMAN Adhesion G protein-coupled receptor B1 OS=Homo sapiens GN=ADGRB1 PE=1 S' | 866 | PPRSLRtPLEI | 0.122 | -0.17 | -1.272 | -0.44 |
| sp\|O14514\|AGRB1_HUMAN Adhesion G protein-coupled receptor B1 OS=Homo sapiens GN=ADGRB1 PE=1 S' | 879 | AHMYNGtTNQT | 0.119 | -0.157 | -1.04 | -0.359 |
| sp\|O14514\|AGRB1_HUMAN Adhesion G protein-coupled receptor B1 OS=Homo sapiens GN=ADGRB1 PE=1 S' | 880 | HMYNGTtNQTC | 0.045 | -0.082 | -1.288 | -0.442 |
| sp\|O14514\|AGRB1_HUMAN Adhesion G protein-coupled receptor B1 OS=Homo sapiens GN=ADGRB1 PE=1 S' | 883 | NGTTNQtCILW | 0.183 | 0.056 | -0.541 | -0.101 |
| sp\|O14514\|AGRB1_HUMAN Adhesion G protein-coupled receptor B1 OS=Homo sapiens GN=ADGRB1 PE=1 S' | 890 | CILWDEtDVPS | 0.203 | -0.085 | -0.878 | -0.253 |
| sp\|O14514\|AGRB1_HUMAN Adhesion G protein-coupled receptor B1 OS=Homo sapiens GN=ADGRB1 PE=1 S' | 894 | DETDVPsSSAP | 0.113 | -0.223 | -1.428 | -0.513 |
| sp\|O14514\|AGRB1_HUMAN Adhesion G protein-coupled receptor B1 OS=Homo sapiens GN=ADGRB1 PE=1 S' | 895 | ETDVPSsSAPP | 0.019 | -0.509 | -1.957 | -0.816 |
| sp\|O14514\|AGRB1_HUMAN Adhesion G protein-coupled receptor B1 OS=Homo sapiens GN=ADGRB1 PE=1 S' | 896 | TDVPSSsAPPQ | 0.282 | 0.365 | -0.428 | 0.073 |
| sp\|O14514\|AGRB1_HUMAN Adhesion G protein-coupled receptor B1 OS=Homo sapiens GN=ADGRB1 PE=1 S' | 905 | PQLGPWsWRGC | 0.106 | -0.252 | -0.875 | -0.34 |
| sp\|O14514\|AGRB1_HUMAN Adhesion G protein-coupled receptor B1 OS=Homo sapiens GN=ADGRB1 PE=1 S' | 911 | SWRGCRtVPLD | 0.74 | 0.759 | 0.292 | 0.597 |
| sp\|O14514\|AGRB1_HUMAN Adhesion G protein-coupled receptor B1 OS=Homo sapiens GN=ADGRB1 PE=1 S' | 919 | PLDALRtRCLC | 0.08 | -0.284 | -1.682 | -0.629 |
| sp\|O14514\|AGRB1_HUMAN Adhesion G protein-coupled receptor B1 OS=Homo sapiens GN=ADGRB1 PE=1 S' | 927 | CLCDRLsTFAI | 0.64 | 0.349 | 0.155 | 0.381 |
| sp\|O14514\|AGRB1_HUMAN Adhesion G protein-coupled receptor B1 OS=Homo sapiens GN=ADGRB1 PE=1 S' | 928 | LCDRLStFAIL | 0.508 | 0.791 | -0.006 | 0.431 |
| sp\|O14514\|AGRB1_HUMAN Adhesion G protein-coupled receptor B1 OS=Homo sapiens GN=ADGRB1 PE=1 S' | 936 | AILAQLsADAN | 0.201 | 0.083 | -0.838 | -0.185 |
| sp\|O14514\|AGRB1_HUMAN Adhesion G protein-coupled receptor B1 OS=Homo sapiens GN=ADGRB1 PE=1 S' | 945 | ANMEKAtLPSV | 0.339 | 0.39 | -0.466 | 0.088 |
| sp\|O14514\|AGRB1_HUMAN Adhesion G protein-coupled receptor B1 OS=Homo sapiens GN=ADGRB1 PE=1 S' | 948 | EKATLPsVTLI | 0.3 | -0.028 | -0.504 | -0.077 |
| sp\|O14514\|AGRB1_HUMAN Adhesion G protein-coupled receptor B1 OS=Homo sapiens GN=ADGRB1 PE=1 S' | 950 | ATLPSVtLIVG | 0.616 | 0.21 | 0.165 | 0.33 |
| sp\|O14514\|AGRB1_HUMAN Adhesion G protein-coupled receptor B1 OS=Homo sapiens GN=ADGRB1 PE=1 S' | 958 | IVGCGVsSLTL | 0.114 | -0.181 | -1.13 | -0.399 |
| sp\|O14514\|AGRB1_HUMAN Adhesion G protein-coupled receptor B1 OS=Homo sapiens GN=ADGRB1 PE=1 S' | 959 | VGCGVSsLTLL | 0.247 | -0.022 | -0.587 | -0.121 |
| sp\|O14514\|AGRB1_HUMAN Adhesion G protein-coupled receptor B1 OS=Homo sapiens GN=ADGRB1 PE=1 S' | 961 | CGVSSLtLLML | 0.268 | 0.144 | -0.308 | 0.035 |
| sp\|O14514\|AGRB1_HUMAN Adhesion G protein-coupled receptor B1 OS=Homo sapiens GN=ADGRB1 PE=1 S' | 971 | LVIIYVsVWRY | 0.39 | 0.052 | -0.648 | -0.069 |
| sp\|O14514\|AGRB1_HUMAN Adhesion G protein-coupled receptor B1 OS=Homo sapiens GN=ADGRB1 PE=1 S' | 978 | VWRYIRsERSV | 0.254 | 0.206 | -0.649 | -0.063 |
| sp\|O14514\|AGRB1_HUMAN Adhesion G protein-coupled receptor B1 OS=Homo sapiens GN=ADGRB1 PE=1 S' | 981 | YIRSERsVILI | 0.321 | 0.273 | -0.409 | 0.062 |
| sp\|O14514\|AGRB1_HUMAN Adhesion G protein-coupled receptor B1 OS=Homo sapiens GN=ADGRB1 PE=1 S' | 990 | LINFCLsIISS | 0.072 | -0.093 | -1.108 | -0.376 |
| sp\|O14514\|AGRB1_HUMAN Adhesion G protein-coupled receptor B1 OS=Homo sapiens GN=ADGRB1 PE=1 S' | 993 | FCLSIIsSNAL | 0.353 | 0.134 | -0.258 | 0.076 |
| sp\|O14514\|AGRB1_HUMAN Adhesion G protein-coupled receptor B1 OS=Homo sapiens GN=ADGRB1 PE=1 S' | 994 | CLSIISsNALI | 0.201 | -0.011 | -0.607 | -0.139 |
| sp\|O14514\|AGRB1_HUMAN Adhesion G protein-coupled receptor B1 OS=Homo sapiens GN=ADGRB1 PE=1 S' | 1003 | LILIGQtQTRN | 0.126 | -0.134 | -1.249 | -0.419 |
| sp\|O14514\|AGRB1_HUMAN Adhesion G protein-coupled receptor B1 OS=Homo sapiens GN=ADGRB1 PE=1 S' | 1005 | LIGQTQtRNKV | 0.127 | -0.023 | -0.986 | -0.294 |

| | | | | | | | |
|---|---|---|---|---|---|---|---|
| sp\|O14514\|AGRB1_HUMAN Adhesion G protein-coupled receptor B1 OS=Homo sapiens GN=ADGRB1 PE=1 S` | 1012 | RNKVVCtLVAA | 0.441 | 0.087 | -0.282 | 0.082 |
| sp\|O14514\|AGRB1_HUMAN Adhesion G protein-coupled receptor B1 OS=Homo sapiens GN=ADGRB1 PE=1 S` | 1024 | LHFFFLsSFCW | 0.085 | -0.047 | -1.428 | -0.463 |
| sp\|O14514\|AGRB1_HUMAN Adhesion G protein-coupled receptor B1 OS=Homo sapiens GN=ADGRB1 PE=1 S` | 1025 | HFFFLSsFCWV | 0.145 | -0.024 | -0.945 | -0.275 |
| sp\|O14514\|AGRB1_HUMAN Adhesion G protein-coupled receptor B1 OS=Homo sapiens GN=ADGRB1 PE=1 S` | 1031 | SFCWVLtEAWQ | 0.129 | -0.148 | -1.055 | -0.358 |
| sp\|O14514\|AGRB1_HUMAN Adhesion G protein-coupled receptor B1 OS=Homo sapiens GN=ADGRB1 PE=1 S` | 1036 | LTEAWQsYMAV | 0.253 | -0.029 | -0.717 | -0.164 |
| sp\|O14514\|AGRB1_HUMAN Adhesion G protein-coupled receptor B1 OS=Homo sapiens GN=ADGRB1 PE=1 S` | 1041 | QSYMAVtGHLR | 0.313 | -0.087 | -0.483 | -0.086 |
| sp\|O14514\|AGRB1_HUMAN Adhesion G protein-coupled receptor B1 OS=Homo sapiens GN=ADGRB1 PE=1 S` | 1068 | ALVVAIsVGFT | 0.254 | 0.009 | -0.639 | -0.125 |
| sp\|O14514\|AGRB1_HUMAN Adhesion G protein-coupled receptor B1 OS=Homo sapiens GN=ADGRB1 PE=1 S` | 1072 | AISVGFtKAKG | 0.071 | -0.165 | -1.241 | -0.445 |
| sp\|O14514\|AGRB1_HUMAN Adhesion G protein-coupled receptor B1 OS=Homo sapiens GN=ADGRB1 PE=1 S` | 1078 | TKAKGYsTMNY | 0.394 | 0.146 | -0.382 | 0.053 |
| sp\|O14514\|AGRB1_HUMAN Adhesion G protein-coupled receptor B1 OS=Homo sapiens GN=ADGRB1 PE=1 S` | 1079 | KAKGYStMNYC | 0.106 | -0.117 | -1.216 | -0.409 |
| sp\|O14514\|AGRB1_HUMAN Adhesion G protein-coupled receptor B1 OS=Homo sapiens GN=ADGRB1 PE=1 S` | 1086 | MNYCWLsLEGG | 0.288 | 0 | -0.423 | -0.045 |
| sp\|O14514\|AGRB1_HUMAN Adhesion G protein-coupled receptor B1 OS=Homo sapiens GN=ADGRB1 PE=1 S` | 1119 | VFNKLVsKDGI | 0.518 | 0.007 | -0.064 | 0.154 |
| sp\|O14514\|AGRB1_HUMAN Adhesion G protein-coupled receptor B1 OS=Homo sapiens GN=ADGRB1 PE=1 S` | 1124 | VSKDGItDKKL | 0.142 | -0.133 | -1.014 | -0.335 |
| sp\|O14514\|AGRB1_HUMAN Adhesion G protein-coupled receptor B1 OS=Homo sapiens GN=ADGRB1 PE=1 S` | 1135 | KERAGAsLWSS | 0.36 | 0.163 | -0.613 | -0.03 |
| sp\|O14514\|AGRB1_HUMAN Adhesion G protein-coupled receptor B1 OS=Homo sapiens GN=ADGRB1 PE=1 S` | 1138 | AGASLWsSCVV | 0.076 | -0.345 | -1.611 | -0.627 |
| sp\|O14514\|AGRB1_HUMAN Adhesion G protein-coupled receptor B1 OS=Homo sapiens GN=ADGRB1 PE=1 S` | 1139 | GASLWSsCVVL | 0.159 | 0.048 | -1.011 | -0.268 |
| sp\|O14514\|AGRB1_HUMAN Adhesion G protein-coupled receptor B1 OS=Homo sapiens GN=ADGRB1 PE=1 S` | 1149 | LPLLALtWMSA | 0.089 | -0.042 | -1.049 | -0.334 |
| sp\|O14514\|AGRB1_HUMAN Adhesion G protein-coupled receptor B1 OS=Homo sapiens GN=ADGRB1 PE=1 S` | 1152 | LALTWMsAVLA | 0.257 | 0.094 | -0.617 | -0.089 |
| sp\|O14514\|AGRB1_HUMAN Adhesion G protein-coupled receptor B1 OS=Homo sapiens GN=ADGRB1 PE=1 S` | 1158 | SAVLAVtDRRS | 0.248 | -0.188 | -1.199 | -0.38 |
| sp\|O14514\|AGRB1_HUMAN Adhesion G protein-coupled receptor B1 OS=Homo sapiens GN=ADGRB1 PE=1 S` | 1162 | AVTDRRsALFQ | 0.11 | -0.028 | -1.446 | -0.455 |
| sp\|O14514\|AGRB1_HUMAN Adhesion G protein-coupled receptor B1 OS=Homo sapiens GN=ADGRB1 PE=1 S` | 1174 | LFAVFDsLEGF | 0.164 | -0.129 | -0.683 | -0.216 |
| sp\|O14514\|AGRB1_HUMAN Adhesion G protein-coupled receptor B1 OS=Homo sapiens GN=ADGRB1 PE=1 S` | 1210 | EEGNGDsGGSF | 0.023 | -0.334 | -1.645 | -0.652 |
| sp\|O14514\|AGRB1_HUMAN Adhesion G protein-coupled receptor B1 OS=Homo sapiens GN=ADGRB1 PE=1 S` | 1213 | NGDSGGsFQNG | 0.11 | -0.159 | -1.153 | -0.401 |
| sp\|O14514\|AGRB1_HUMAN Adhesion G protein-coupled receptor B1 OS=Homo sapiens GN=ADGRB1 PE=1 S` | 1223 | GHAQLMtDFEK | 0.39 | 0.107 | -0.623 | -0.042 |
| sp\|O14514\|AGRB1_HUMAN Adhesion G protein-coupled receptor B1 OS=Homo sapiens GN=ADGRB1 PE=1 S` | 1235 | VDLACRsVLNK | 0.097 | -0.21 | -1.339 | -0.484 |
| sp\|O14514\|AGRB1_HUMAN Adhesion G protein-coupled receptor B1 OS=Homo sapiens GN=ADGRB1 PE=1 S` | 1246 | DIAACRtATIT | 0.363 | -0.02 | -0.822 | -0.16 |
| sp\|O14514\|AGRB1_HUMAN Adhesion G protein-coupled receptor B1 OS=Homo sapiens GN=ADGRB1 PE=1 S` | 1248 | AACRTAtITGT | 0.515 | 0.679 | 0.009 | 0.401 |
| sp\|O14514\|AGRB1_HUMAN Adhesion G protein-coupled receptor B1 OS=Homo sapiens GN=ADGRB1 PE=1 S` | 1250 | CRTATItGTLK | 0.264 | 0.128 | -0.438 | -0.015 |
| sp\|O14514\|AGRB1_HUMAN Adhesion G protein-coupled receptor B1 OS=Homo sapiens GN=ADGRB1 PE=1 S` | 1252 | TATITGtLKRP | 0.077 | -0.21 | -1.211 | -0.448 |
| sp\|O14514\|AGRB1_HUMAN Adhesion G protein-coupled receptor B1 OS=Homo sapiens GN=ADGRB1 PE=1 S` | 1257 | GTLKRPsLPEE | 0.93 | 0.713 | 0.886 | 0.843 |
| sp\|O14514\|AGRB1_HUMAN Adhesion G protein-coupled receptor B1 OS=Homo sapiens GN=ADGRB1 PE=1 S` | 1274 | HAKGPPtNFNS | 0.102 | -0.154 | -1.065 | -0.372 |
| sp\|O14514\|AGRB1_HUMAN Adhesion G protein-coupled receptor B1 OS=Homo sapiens GN=ADGRB1 PE=1 S` | 1278 | PPTNFNsLPAN | 0.803 | 0.668 | 0.608 | 0.693 |
| sp\|O14514\|AGRB1_HUMAN Adhesion G protein-coupled receptor B1 OS=Homo sapiens GN=ADGRB1 PE=1 S` | 1284 | SLPANVsKLHL | 0.165 | -0.138 | -0.792 | -0.255 |
| sp\|O14514\|AGRB1_HUMAN Adhesion G protein-coupled receptor B1 OS=Homo sapiens GN=ADGRB1 PE=1 S` | 1291 | KLHLHGsPRYP | 0.048 | -0.494 | -1.784 | -0.743 |
| sp\|O14514\|AGRB1_HUMAN Adhesion G protein-coupled receptor B1 OS=Homo sapiens GN=ADGRB1 PE=1 S` | 1306 | PDFPNHsLTLK | 0.416 | 0.038 | -0.309 | 0.048 |
| sp\|O14514\|AGRB1_HUMAN Adhesion G protein-coupled receptor B1 OS=Homo sapiens GN=ADGRB1 PE=1 S` | 1308 | FPNHSLtLKRD | 0.399 | 0.168 | -0.289 | 0.093 |
| sp\|O14514\|AGRB1_HUMAN Adhesion G protein-coupled receptor B1 OS=Homo sapiens GN=ADGRB1 PE=1 S` | 1317 | RDKAPKsSFVG | 0.23 | -0.098 | -0.532 | -0.133 |
| sp\|O14514\|AGRB1_HUMAN Adhesion G protein-coupled receptor B1 OS=Homo sapiens GN=ADGRB1 PE=1 S` | 1318 | DKAPKSsFVGD | 0.25 | -0.091 | -0.507 | -0.116 |
| sp\|O14514\|AGRB1_HUMAN Adhesion G protein-coupled receptor B1 OS=Homo sapiens GN=ADGRB1 PE=1 S` | 1331 | IFKKLDsELSR | 0.242 | -0.039 | -0.679 | -0.159 |
| sp\|O14514\|AGRB1_HUMAN Adhesion G protein-coupled receptor B1 OS=Homo sapiens GN=ADGRB1 PE=1 S` | 1334 | KLDSELsRAQE | 0.075 | -0.357 | -1.547 | -0.61 |
| sp\|O14514\|AGRB1_HUMAN Adhesion G protein-coupled receptor B1 OS=Homo sapiens GN=ADGRB1 PE=1 S` | 1343 | QEKALDtSYVI | 0.145 | -0.151 | -1.111 | -0.372 |
| sp\|O14514\|AGRB1_HUMAN Adhesion G protein-coupled receptor B1 OS=Homo sapiens GN=ADGRB1 PE=1 S` | 1344 | EKALDTsYVIL | 0.197 | -0.023 | -0.639 | -0.155 |
| sp\|O14514\|AGRB1_HUMAN Adhesion G protein-coupled receptor B1 OS=Homo sapiens GN=ADGRB1 PE=1 S` | 1350 | SYVILPtATAT | 0.131 | -0.104 | -1.269 | -0.414 |
| sp\|O14514\|AGRB1_HUMAN Adhesion G protein-coupled receptor B1 OS=Homo sapiens GN=ADGRB1 PE=1 S` | 1352 | VILPTAtATLR | 0.236 | 0.065 | -0.74 | -0.146 |
| sp\|O14514\|AGRB1_HUMAN Adhesion G protein-coupled receptor B1 OS=Homo sapiens GN=ADGRB1 PE=1 S` | 1354 | LPTATAtLRPK | 0.239 | -0.111 | -0.51 | -0.127 |
| sp\|O14514\|AGRB1_HUMAN Adhesion G protein-coupled receptor B1 OS=Homo sapiens GN=ADGRB1 PE=1 S` | 1366 | KEEPKYsIHID | 0.147 | -0.14 | -0.87 | -0.288 |
| sp\|O14514\|AGRB1_HUMAN Adhesion G protein-coupled receptor B1 OS=Homo sapiens GN=ADGRB1 PE=1 S` | 1375 | IDQMPQtRLIH | 0.065 | -0.432 | -1.461 | -0.609 |
| sp\|O14514\|AGRB1_HUMAN Adhesion G protein-coupled receptor B1 OS=Homo sapiens GN=ADGRB1 PE=1 S` | 1381 | TRLIHLsTAPE | 0.093 | 0.005 | -1.111 | -0.338 |
| sp\|O14514\|AGRB1_HUMAN Adhesion G protein-coupled receptor B1 OS=Homo sapiens GN=ADGRB1 PE=1 S` | 1382 | RLIHLStAPEA | 0.582 | 0.523 | -0.141 | 0.321 |

| | | | | | | |
|---|---|---|---|---|---|---|
| sp\|O14514\|AGRB1_HUMAN Adhesion G protein-coupled receptor B1 OS=Homo sapiens GN=ADGRB1 PE=1 S' | 1387 | STAPEAsLPAR | 0.729 | 0.499 | 0.291 | 0.506 |
| sp\|O14514\|AGRB1_HUMAN Adhesion G protein-coupled receptor B1 OS=Homo sapiens GN=ADGRB1 PE=1 S' | 1392 | ASLPARsPPSR | 0.192 | 0.037 | -0.772 | -0.181 |
| sp\|O14514\|AGRB1_HUMAN Adhesion G protein-coupled receptor B1 OS=Homo sapiens GN=ADGRB1 PE=1 S' | 1395 | PARSPPsRQPP | 0.132 | -0.213 | -1.014 | -0.365 |
| sp\|O14514\|AGRB1_HUMAN Adhesion G protein-coupled receptor B1 OS=Homo sapiens GN=ADGRB1 PE=1 S' | 1400 | PSRQPPsGGPP | 0.239 | -0.091 | -0.471 | -0.108 |
| sp\|O14514\|AGRB1_HUMAN Adhesion G protein-coupled receptor B1 OS=Homo sapiens GN=ADGRB1 PE=1 S' | 1438 | LEPAPPsLGDP | 0.213 | -0.165 | -0.639 | -0.197 |
| sp\|O14514\|AGRB1_HUMAN Adhesion G protein-coupled receptor B1 OS=Homo sapiens GN=ADGRB1 PE=1 S' | 1452 | AAHPGPsTGPS | 0.044 | -0.403 | -1.61 | -0.656 |
| sp\|O14514\|AGRB1_HUMAN Adhesion G protein-coupled receptor B1 OS=Homo sapiens GN=ADGRB1 PE=1 S' | 1453 | AHPGPStGPST | 0.076 | 0.042 | -1.196 | -0.359 |
| sp\|O14514\|AGRB1_HUMAN Adhesion G protein-coupled receptor B1 OS=Homo sapiens GN=ADGRB1 PE=1 S' | 1456 | GPSTGPsTKNE | 0.089 | -0.258 | -1.491 | -0.553 |
| sp\|O14514\|AGRB1_HUMAN Adhesion G protein-coupled receptor B1 OS=Homo sapiens GN=ADGRB1 PE=1 S' | 1457 | PSTGPStKNEN | 0.141 | -0.161 | -0.673 | -0.231 |
| sp\|O14514\|AGRB1_HUMAN Adhesion G protein-coupled receptor B1 OS=Homo sapiens GN=ADGRB1 PE=1 S' | 1464 | KNENVAtLSVS | 0.167 | -0.114 | -0.801 | -0.249 |
| sp\|O14514\|AGRB1_HUMAN Adhesion G protein-coupled receptor B1 OS=Homo sapiens GN=ADGRB1 PE=1 S' | 1466 | ENVATLsVSSL | 0.023 | -0.242 | -1.558 | -0.592 |
| sp\|O14514\|AGRB1_HUMAN Adhesion G protein-coupled receptor B1 OS=Homo sapiens GN=ADGRB1 PE=1 S' | 1468 | VATLSVsSLER | 0.355 | 0.076 | -0.458 | -0.009 |
| sp\|O14514\|AGRB1_HUMAN Adhesion G protein-coupled receptor B1 OS=Homo sapiens GN=ADGRB1 PE=1 S' | 1469 | ATLSVSsLERR | 0.207 | -0.212 | -1.021 | -0.342 |
| sp\|O14514\|AGRB1_HUMAN Adhesion G protein-coupled receptor B1 OS=Homo sapiens GN=ADGRB1 PE=1 S' | 1475 | SLERRKsRYAE | 0.838 | 1.041 | 0.866 | 0.915 |
| sp\|O14514\|AGRB1_HUMAN Adhesion G protein-coupled receptor B1 OS=Homo sapiens GN=ADGRB1 PE=1 S' | 1488 | FEKIMHtRKRH | 0.187 | -0.158 | -1.19 | -0.387 |
| sp\|O14514\|AGRB1_HUMAN Adhesion G protein-coupled receptor B1 OS=Homo sapiens GN=ADGRB1 PE=1 S' | 1518 | EVLGPDsKPEK | 0.333 | 0.246 | -0.206 | 0.124 |
| sp\|O14514\|AGRB1_HUMAN Adhesion G protein-coupled receptor B1 OS=Homo sapiens GN=ADGRB1 PE=1 S' | 1525 | KPEKQQtPNKR | 0.102 | -0.35 | -1.168 | -0.472 |
| sp\|O14514\|AGRB1_HUMAN Adhesion G protein-coupled receptor B1 OS=Homo sapiens GN=ADGRB1 PE=1 S' | 1533 | NKRPWEsLRKA | 0.668 | 0.224 | 0.108 | 0.333 |
| sp\|O14514\|AGRB1_HUMAN Adhesion G protein-coupled receptor B1 OS=Homo sapiens GN=ADGRB1 PE=1 S' | 1540 | LRKAHGtPTWV | 0.165 | -0.132 | -1.097 | -0.355 |
| sp\|O14514\|AGRB1_HUMAN Adhesion G protein-coupled receptor B1 OS=Homo sapiens GN=ADGRB1 PE=1 S' | 1542 | KAHGTPtWVKK | 0.04 | -0.192 | -1.529 | -0.56 |
| sp\|O14514\|AGRB1_HUMAN Adhesion G protein-coupled receptor B1 OS=Homo sapiens GN=ADGRB1 PE=1 S' | 1554 | LEPLQPsPLEL | 0.03 | -0.468 | -1.954 | -0.797 |
| sp\|O14514\|AGRB1_HUMAN Adhesion G protein-coupled receptor B1 OS=Homo sapiens GN=ADGRB1 PE=1 S' | 1560 | SPLELRsVEWE | 0.036 | -0.243 | -1.792 | -0.666 |
| sp\|O14514\|AGRB1_HUMAN Adhesion G protein-coupled receptor B1 OS=Homo sapiens GN=ADGRB1 PE=1 S' | 1566 | SVEWERsGATI | 0.088 | -0.268 | -1.272 | -0.484 |
| sp\|O14514\|AGRB1_HUMAN Adhesion G protein-coupled receptor B1 OS=Homo sapiens GN=ADGRB1 PE=1 S' | 1569 | WERSGAtIPLV | 0.576 | 0.538 | -0.169 | 0.315 |
| sp\|O14514\|AGRB1_HUMAN Adhesion G protein-coupled receptor B1 OS=Homo sapiens GN=ADGRB1 PE=1 S' | 1582 | DIIDLQtEV-- | 0.217 | 0.236 | -0.982 | -0.176 |
| sp\|O60241\|AGRB2_HUMAN Adhesion G protein-coupled receptor B2 OS=Homo sapiens GN=ADGRB2 PE=1 S' | 316 | PAAEEWsPWSV | 0.054 | -0.453 | -1.823 | -0.741 |
| sp\|O60241\|AGRB2_HUMAN Adhesion G protein-coupled receptor B2 OS=Homo sapiens GN=ADGRB2 PE=1 S' | 319 | EEWSPWsVCSL | 0.037 | -0.228 | -1.463 | -0.551 |
| sp\|O60241\|AGRB2_HUMAN Adhesion G protein-coupled receptor B2 OS=Homo sapiens GN=ADGRB2 PE=1 S' | 322 | SPWSVCsLTCG | 0.277 | 0.065 | -0.972 | -0.21 |
| sp\|O60241\|AGRB2_HUMAN Adhesion G protein-coupled receptor B2 OS=Homo sapiens GN=ADGRB2 PE=1 S' | 324 | WSVCSLtCGQG | 0.382 | 0.147 | -0.29 | 0.08 |
| sp\|O60241\|AGRB2_HUMAN Adhesion G protein-coupled receptor B2 OS=Homo sapiens GN=ADGRB2 PE=1 S' | 333 | QGLQVRtRSCV | 0.092 | -0.194 | -1.448 | -0.517 |
| sp\|O60241\|AGRB2_HUMAN Adhesion G protein-coupled receptor B2 OS=Homo sapiens GN=ADGRB2 PE=1 S' | 335 | LQVRTRsCVSS | 0.167 | 0.833 | -0.415 | 0.195 |
| sp\|O60241\|AGRB2_HUMAN Adhesion G protein-coupled receptor B2 OS=Homo sapiens GN=ADGRB2 PE=1 S' | 338 | RTRSCVsSPYG | 0.88 | 0.641 | 0.396 | 0.639 |
| sp\|O60241\|AGRB2_HUMAN Adhesion G protein-coupled receptor B2 OS=Homo sapiens GN=ADGRB2 PE=1 S' | 339 | TRSCVSsPYGT | 0.069 | -0.335 | -1.42 | -0.562 |
| sp\|O60241\|AGRB2_HUMAN Adhesion G protein-coupled receptor B2 OS=Homo sapiens GN=ADGRB2 PE=1 S' | 343 | VSSPYGtLCSG | 0.084 | -0.329 | -1.201 | -0.482 |
| sp\|O60241\|AGRB2_HUMAN Adhesion G protein-coupled receptor B2 OS=Homo sapiens GN=ADGRB2 PE=1 S' | 346 | PYGTLCsGPLR | 0.532 | 0.431 | -0.275 | 0.229 |
| sp\|O60241\|AGRB2_HUMAN Adhesion G protein-coupled receptor B2 OS=Homo sapiens GN=ADGRB2 PE=1 S' | 352 | SGPLREtRPCN | 0.385 | 0.408 | -0.65 | 0.048 |
| sp\|O60241\|AGRB2_HUMAN Adhesion G protein-coupled receptor B2 OS=Homo sapiens GN=ADGRB2 PE=1 S' | 358 | TRPCNNsATCP | 0.272 | 0.149 | -0.979 | -0.186 |
| sp\|O60241\|AGRB2_HUMAN Adhesion G protein-coupled receptor B2 OS=Homo sapiens GN=ADGRB2 PE=1 S' | 360 | PCNNSAtCPVH | 0.755 | 0.757 | 0.214 | 0.575 |
| sp\|O60241\|AGRB2_HUMAN Adhesion G protein-coupled receptor B2 OS=Homo sapiens GN=ADGRB2 PE=1 S' | 372 | VWEEWGsWSLC | 0.057 | -0.154 | -1.571 | -0.556 |
| sp\|O60241\|AGRB2_HUMAN Adhesion G protein-coupled receptor B2 OS=Homo sapiens GN=ADGRB2 PE=1 S' | 374 | EEWGSWsLCSR | 0.212 | 0.182 | -0.457 | -0.021 |
| sp\|O60241\|AGRB2_HUMAN Adhesion G protein-coupled receptor B2 OS=Homo sapiens GN=ADGRB2 PE=1 S' | 377 | GSWSLCsRSCG | 0.099 | -0.129 | -1.548 | -0.526 |
| sp\|O60241\|AGRB2_HUMAN Adhesion G protein-coupled receptor B2 OS=Homo sapiens GN=ADGRB2 PE=1 S' | 379 | WSLCSRsCGRG | 0.441 | 0.126 | -0.457 | 0.037 |
| sp\|O60241\|AGRB2_HUMAN Adhesion G protein-coupled receptor B2 OS=Homo sapiens GN=ADGRB2 PE=1 S' | 384 | RSCGRGsRSRM | 0.14 | -0.22 | -1.305 | -0.462 |
| sp\|O60241\|AGRB2_HUMAN Adhesion G protein-coupled receptor B2 OS=Homo sapiens GN=ADGRB2 PE=1 S' | 386 | CGRGSRsRMRT | 0.406 | 0.225 | -0.334 | 0.099 |
| sp\|O60241\|AGRB2_HUMAN Adhesion G protein-coupled receptor B2 OS=Homo sapiens GN=ADGRB2 PE=1 S' | 390 | SRSRMRtCVPP | 0.326 | 0.86 | -0.336 | 0.283 |
| sp\|O60241\|AGRB2_HUMAN Adhesion G protein-coupled receptor B2 OS=Homo sapiens GN=ADGRB2 PE=1 S' | 408 | EGPELQtKLCS | 0.031 | -0.205 | -1.617 | -0.597 |
| sp\|O60241\|AGRB2_HUMAN Adhesion G protein-coupled receptor B2 OS=Homo sapiens GN=ADGRB2 PE=1 S' | 412 | LQTKLCsMAAC | 0.203 | 0.02 | -0.884 | -0.22 |
| sp\|O60241\|AGRB2_HUMAN Adhesion G protein-coupled receptor B2 OS=Homo sapiens GN=ADGRB2 PE=1 S' | 432 | GPWGPCsTSCA | 0.109 | -0.05 | -1.182 | -0.374 |
| sp\|O60241\|AGRB2_HUMAN Adhesion G protein-coupled receptor B2 OS=Homo sapiens GN=ADGRB2 PE=1 S' | 433 | PWGPCStSCAN | 0.107 | -0.14 | -1.089 | -0.374 |

| | | | | | | |
|---|---|---|---|---|---|---|
| sp\|O60241\|AGRB2_HUMAN Adhesion G protein-coupled receptor B2 OS=Homo sapiens GN=ADGRB2 PE=1 S' | 434 | WGPCSTsCANG | 0.283 | 0.137 | -0.565 | -0.048 |
| sp\|O60241\|AGRB2_HUMAN Adhesion G protein-coupled receptor B2 OS=Homo sapiens GN=ADGRB2 PE=1 S' | 439 | TSCANGtQQRS | 0.103 | -0.381 | -1.551 | -0.61 |
| sp\|O60241\|AGRB2_HUMAN Adhesion G protein-coupled receptor B2 OS=Homo sapiens GN=ADGRB2 PE=1 S' | 443 | NGTQQRsRKCS | 0.076 | -0.155 | -1.273 | -0.451 |
| sp\|O60241\|AGRB2_HUMAN Adhesion G protein-coupled receptor B2 OS=Homo sapiens GN=ADGRB2 PE=1 S' | 447 | QRSRKCsVAGP | 0.461 | 0.833 | -0.011 | 0.428 |
| sp\|O60241\|AGRB2_HUMAN Adhesion G protein-coupled receptor B2 OS=Homo sapiens GN=ADGRB2 PE=1 S' | 455 | AGPAWAtCTGA | 0.273 | -0.11 | -0.744 | -0.194 |
| sp\|O60241\|AGRB2_HUMAN Adhesion G protein-coupled receptor B2 OS=Homo sapiens GN=ADGRB2 PE=1 S' | 457 | PAWATCtGALT | 0.09 | -0.077 | -1.189 | -0.392 |
| sp\|O60241\|AGRB2_HUMAN Adhesion G protein-coupled receptor B2 OS=Homo sapiens GN=ADGRB2 PE=1 S' | 461 | TCTGALtDTRE | 0.263 | -0.076 | -1.001 | -0.271 |
| sp\|O60241\|AGRB2_HUMAN Adhesion G protein-coupled receptor B2 OS=Homo sapiens GN=ADGRB2 PE=1 S' | 463 | TGALTDtRECS | 0.062 | -0.217 | -1.492 | -0.549 |
| sp\|O60241\|AGRB2_HUMAN Adhesion G protein-coupled receptor B2 OS=Homo sapiens GN=ADGRB2 PE=1 S' | 467 | TDTRECsNLEC | 0.356 | 0.576 | -0.722 | 0.07 |
| sp\|O60241\|AGRB2_HUMAN Adhesion G protein-coupled receptor B2 OS=Homo sapiens GN=ADGRB2 PE=1 S' | 474 | NLECPAtDSKW | 0.239 | -0.046 | -0.867 | -0.225 |
| sp\|O60241\|AGRB2_HUMAN Adhesion G protein-coupled receptor B2 OS=Homo sapiens GN=ADGRB2 PE=1 S' | 476 | ECPATDsKWGP | 0.095 | -0.214 | -1.305 | -0.475 |
| sp\|O60241\|AGRB2_HUMAN Adhesion G protein-coupled receptor B2 OS=Homo sapiens GN=ADGRB2 PE=1 S' | 485 | GPWNAWsLCSK | 0.209 | 0.016 | -0.648 | -0.141 |
| sp\|O60241\|AGRB2_HUMAN Adhesion G protein-coupled receptor B2 OS=Homo sapiens GN=ADGRB2 PE=1 S' | 488 | NAWSLCsKTCD | 0.198 | -0.004 | -1.046 | -0.284 |
| sp\|O60241\|AGRB2_HUMAN Adhesion G protein-coupled receptor B2 OS=Homo sapiens GN=ADGRB2 PE=1 S' | 490 | WSLCSKtCDTG | 0.608 | 0.246 | 0.201 | 0.352 |
| sp\|O60241\|AGRB2_HUMAN Adhesion G protein-coupled receptor B2 OS=Homo sapiens GN=ADGRB2 PE=1 S' | 493 | CSKTCDtGWQR | 0.438 | -0.117 | -0.452 | -0.044 |
| sp\|O60241\|AGRB2_HUMAN Adhesion G protein-coupled receptor B2 OS=Homo sapiens GN=ADGRB2 PE=1 S' | 505 | FRMCQAtGTQG | 0.349 | 0.239 | -0.613 | -0.008 |
| sp\|O60241\|AGRB2_HUMAN Adhesion G protein-coupled receptor B2 OS=Homo sapiens GN=ADGRB2 PE=1 S' | 507 | MCQATGtQGYP | 0.151 | -0.035 | -1.27 | -0.385 |
| sp\|O60241\|AGRB2_HUMAN Adhesion G protein-coupled receptor B2 OS=Homo sapiens GN=ADGRB2 PE=1 S' | 515 | GYPCEGtGEEV | 0.16 | -0.179 | -1.531 | -0.517 |
| sp\|O60241\|AGRB2_HUMAN Adhesion G protein-coupled receptor B2 OS=Homo sapiens GN=ADGRB2 PE=1 S' | 523 | EEVKPCsEKRC | 0.188 | -0.18 | -1.225 | -0.406 |
| sp\|O60241\|AGRB2_HUMAN Adhesion G protein-coupled receptor B2 OS=Homo sapiens GN=ADGRB2 PE=1 S' | 543 | EYVMLMtWKKA | 0.135 | 0.047 | -0.841 | -0.22 |
| sp\|O60241\|AGRB2_HUMAN Adhesion G protein-coupled receptor B2 OS=Homo sapiens GN=ADGRB2 PE=1 S' | 562 | KCPPNAsGSAS | 0.111 | -0.175 | -1.159 | -0.408 |
| sp\|O60241\|AGRB2_HUMAN Adhesion G protein-coupled receptor B2 OS=Homo sapiens GN=ADGRB2 PE=1 S' | 564 | PPNASGsASRR | 0.187 | -0.054 | -1.112 | -0.326 |
| sp\|O60241\|AGRB2_HUMAN Adhesion G protein-coupled receptor B2 OS=Homo sapiens GN=ADGRB2 PE=1 S' | 566 | NASGSAsRRCL | 0.31 | 0.085 | -0.504 | -0.036 |
| sp\|O60241\|AGRB2_HUMAN Adhesion G protein-coupled receptor B2 OS=Homo sapiens GN=ADGRB2 PE=1 S' | 572 | SRRCLLsAQGV | 0.285 | 0.289 | -0.976 | -0.134 |
| sp\|O60241\|AGRB2_HUMAN Adhesion G protein-coupled receptor B2 OS=Homo sapiens GN=ADGRB2 PE=1 S' | 583 | AYWGLPsFARC | 0.101 | -0.158 | -1.39 | -0.482 |
| sp\|O60241\|AGRB2_HUMAN Adhesion G protein-coupled receptor B2 OS=Homo sapiens GN=ADGRB2 PE=1 S' | 589 | SFARCIsHEYR | 0.728 | 0.81 | 0.472 | 0.67 |
| sp\|O60241\|AGRB2_HUMAN Adhesion G protein-coupled receptor B2 OS=Homo sapiens GN=ADGRB2 PE=1 S' | 598 | YRYLYLsLREH | 0.236 | 0.1 | -0.649 | -0.104 |
| sp\|O60241\|AGRB2_HUMAN Adhesion G protein-coupled receptor B2 OS=Homo sapiens GN=ADGRB2 PE=1 S' | 616 | LAGEGMsQVVR | 0.06 | -0.092 | -1.422 | -0.485 |
| sp\|O60241\|AGRB2_HUMAN Adhesion G protein-coupled receptor B2 OS=Homo sapiens GN=ADGRB2 PE=1 S' | 621 | MSQVVRsLQEL | 0.367 | 0.169 | -0.477 | 0.02 |
| sp\|O60241\|AGRB2_HUMAN Adhesion G protein-coupled receptor B2 OS=Homo sapiens GN=ADGRB2 PE=1 S' | 630 | ELLARRtYYSG | 0.169 | 0.069 | -0.799 | -0.187 |
| sp\|O60241\|AGRB2_HUMAN Adhesion G protein-coupled receptor B2 OS=Homo sapiens GN=ADGRB2 PE=1 S' | 633 | ARRTYYsGDLL | 0.526 | 0.52 | 0.031 | 0.359 |
| sp\|O60241\|AGRB2_HUMAN Adhesion G protein-coupled receptor B2 OS=Homo sapiens GN=ADGRB2 PE=1 S' | 639 | SGDLLFsVDIL | 0.134 | -0.059 | -1.007 | -0.311 |
| sp\|O60241\|AGRB2_HUMAN Adhesion G protein-coupled receptor B2 OS=Homo sapiens GN=ADGRB2 PE=1 S' | 647 | DILRNVtDTFK | 0.83 | 0.815 | 0.448 | 0.698 |
| sp\|O60241\|AGRB2_HUMAN Adhesion G protein-coupled receptor B2 OS=Homo sapiens GN=ADGRB2 PE=1 S' | 649 | LRNVTDtFKRA | 0.12 | 0.038 | -1.054 | -0.299 |
| sp\|O60241\|AGRB2_HUMAN Adhesion G protein-coupled receptor B2 OS=Homo sapiens GN=ADGRB2 PE=1 S' | 654 | DTFKRAtYVPS | 0.427 | 0.061 | -0.692 | -0.068 |
| sp\|O60241\|AGRB2_HUMAN Adhesion G protein-coupled receptor B2 OS=Homo sapiens GN=ADGRB2 PE=1 S' | 658 | RATYVPsADDV | 0.405 | 0.076 | -0.636 | -0.052 |
| sp\|O60241\|AGRB2_HUMAN Adhesion G protein-coupled receptor B2 OS=Homo sapiens GN=ADGRB2 PE=1 S' | 670 | RFFQVVsFMVD | 0.687 | 0.196 | 0.116 | 0.333 |
| sp\|O60241\|AGRB2_HUMAN Adhesion G protein-coupled receptor B2 OS=Homo sapiens GN=ADGRB2 PE=1 S' | 688 | DDAQQVsPGSV | 0.087 | -0.43 | -1.447 | -0.597 |
| sp\|O60241\|AGRB2_HUMAN Adhesion G protein-coupled receptor B2 OS=Homo sapiens GN=ADGRB2 PE=1 S' | 691 | QQVSPGsVHLL | 0.068 | -0.205 | -1.127 | -0.421 |
| sp\|O60241\|AGRB2_HUMAN Adhesion G protein-coupled receptor B2 OS=Homo sapiens GN=ADGRB2 PE=1 S' | 714 | ALKAFQsSLIV | 0.283 | -0.049 | -0.995 | -0.254 |
| sp\|O60241\|AGRB2_HUMAN Adhesion G protein-coupled receptor B2 OS=Homo sapiens GN=ADGRB2 PE=1 S' | 715 | LKAFQSsLIVT | 0.21 | 0.052 | -0.378 | -0.039 |
| sp\|O60241\|AGRB2_HUMAN Adhesion G protein-coupled receptor B2 OS=Homo sapiens GN=ADGRB2 PE=1 S' | 719 | QSSLIVtDNLV | 0.303 | -0.055 | -0.71 | -0.154 |
| sp\|O60241\|AGRB2_HUMAN Adhesion G protein-coupled receptor B2 OS=Homo sapiens GN=ADGRB2 PE=1 S' | 725 | TDNLVIsIQRE | 0.089 | -0.332 | -1.454 | -0.566 |
| sp\|O60241\|AGRB2_HUMAN Adhesion G protein-coupled receptor B2 OS=Homo sapiens GN=ADGRB2 PE=1 S' | 732 | IQREPVsAVSS | 0.178 | 0.057 | -0.733 | -0.166 |
| sp\|O60241\|AGRB2_HUMAN Adhesion G protein-coupled receptor B2 OS=Homo sapiens GN=ADGRB2 PE=1 S' | 735 | EPVSAVsSDIT | 0.227 | -0.122 | -0.786 | -0.227 |
| sp\|O60241\|AGRB2_HUMAN Adhesion G protein-coupled receptor B2 OS=Homo sapiens GN=ADGRB2 PE=1 S' | 736 | PVSAVSsDITF | 0.093 | -0.165 | -1.096 | -0.389 |
| sp\|O60241\|AGRB2_HUMAN Adhesion G protein-coupled receptor B2 OS=Homo sapiens GN=ADGRB2 PE=1 S' | 739 | AVSSDItFPMR | 0.503 | 0.32 | -0.127 | 0.232 |
| sp\|O60241\|AGRB2_HUMAN Adhesion G protein-coupled receptor B2 OS=Homo sapiens GN=ADGRB2 PE=1 S' | 755 | KDWVRHsEDRL | 0.287 | 0.293 | -0.613 | -0.011 |
| sp\|O60241\|AGRB2_HUMAN Adhesion G protein-coupled receptor B2 OS=Homo sapiens GN=ADGRB2 PE=1 S' | 767 | LPKEVLsLSSP | 0.051 | -0.203 | -1.314 | -0.489 |

| | | | | | | |
|---|---|---|---|---|---|---|
| sp\|O60241\|AGRB2_HUMAN Adhesion G protein-coupled receptor B2 OS=Homo sapiens GN=ADGRB2 PE=1 S' | 769 | KEVLSLsSPGK | 0.451 | 0.443 | -0.213 | 0.227 |
| sp\|O60241\|AGRB2_HUMAN Adhesion G protein-coupled receptor B2 OS=Homo sapiens GN=ADGRB2 PE=1 S' | 770 | EVLSLSsPGKP | 0.019 | -0.561 | -1.895 | -0.812 |
| sp\|O60241\|AGRB2_HUMAN Adhesion G protein-coupled receptor B2 OS=Homo sapiens GN=ADGRB2 PE=1 S' | 776 | SPGKPAtSGAA | 0.216 | -0.094 | -0.746 | -0.208 |
| sp\|O60241\|AGRB2_HUMAN Adhesion G protein-coupled receptor B2 OS=Homo sapiens GN=ADGRB2 PE=1 S' | 777 | PGKPATsGAAG | 0.306 | -0.005 | -0.248 | 0.018 |
| sp\|O60241\|AGRB2_HUMAN Adhesion G protein-coupled receptor B2 OS=Homo sapiens GN=ADGRB2 PE=1 S' | 782 | TSGAAGsPGRG | 0.081 | -0.504 | -1.492 | -0.638 |
| sp\|O60241\|AGRB2_HUMAN Adhesion G protein-coupled receptor B2 OS=Homo sapiens GN=ADGRB2 PE=1 S' | 791 | RGRGPGtVPPG | 0.55 | 0.531 | 0.195 | 0.425 |
| sp\|O60241\|AGRB2_HUMAN Adhesion G protein-coupled receptor B2 OS=Homo sapiens GN=ADGRB2 PE=1 S' | 799 | PPGPGHsHQRL | 0.155 | -0.078 | -0.998 | -0.307 |
| sp\|O60241\|AGRB2_HUMAN Adhesion G protein-coupled receptor B2 OS=Homo sapiens GN=ADGRB2 PE=1 S' | 811 | PADPDEsSYFV | 0.054 | -0.344 | -1.818 | -0.703 |
| sp\|O60241\|AGRB2_HUMAN Adhesion G protein-coupled receptor B2 OS=Homo sapiens GN=ADGRB2 PE=1 S' | 812 | ADPDESsYFVI | 0.058 | -0.281 | -1.538 | -0.587 |
| sp\|O60241\|AGRB2_HUMAN Adhesion G protein-coupled receptor B2 OS=Homo sapiens GN=ADGRB2 PE=1 S' | 823 | GAVLYRtLGLI | 0.496 | 0.177 | -0.301 | 0.124 |
| sp\|O60241\|AGRB2_HUMAN Adhesion G protein-coupled receptor B2 OS=Homo sapiens GN=ADGRB2 PE=1 S' | 838 | RPPLAVtSRVM | 0.263 | -0.097 | -0.852 | -0.229 |
| sp\|O60241\|AGRB2_HUMAN Adhesion G protein-coupled receptor B2 OS=Homo sapiens GN=ADGRB2 PE=1 S' | 839 | PPLAVTsRVMT | 0.108 | -0.293 | -1.225 | -0.47 |
| sp\|O60241\|AGRB2_HUMAN Adhesion G protein-coupled receptor B2 OS=Homo sapiens GN=ADGRB2 PE=1 S' | 843 | VTSRVMtVTVR | 0.774 | 0.923 | 0.434 | 0.71 |
| sp\|O60241\|AGRB2_HUMAN Adhesion G protein-coupled receptor B2 OS=Homo sapiens GN=ADGRB2 PE=1 S' | 845 | SRVMTVtVRPP | 0.127 | -0.043 | -1.001 | -0.306 |
| sp\|O60241\|AGRB2_HUMAN Adhesion G protein-coupled receptor B2 OS=Homo sapiens GN=ADGRB2 PE=1 S' | 850 | VTVRPPtQPPA | 0.73 | 0.999 | 0.472 | 0.734 |
| sp\|O60241\|AGRB2_HUMAN Adhesion G protein-coupled receptor B2 OS=Homo sapiens GN=ADGRB2 PE=1 S' | 859 | PAEPLItVELS | 0.111 | -0.187 | -1.118 | -0.398 |
| sp\|O60241\|AGRB2_HUMAN Adhesion G protein-coupled receptor B2 OS=Homo sapiens GN=ADGRB2 PE=1 S' | 863 | LITVELsYIIN | 0.071 | -0.107 | -1.177 | -0.404 |
| sp\|O60241\|AGRB2_HUMAN Adhesion G protein-coupled receptor B2 OS=Homo sapiens GN=ADGRB2 PE=1 S' | 869 | SYIINGtTDPH | 0.076 | -0.25 | -1.503 | -0.559 |
| sp\|O60241\|AGRB2_HUMAN Adhesion G protein-coupled receptor B2 OS=Homo sapiens GN=ADGRB2 PE=1 S' | 870 | YIINGTtDPHC | 0.281 | 0.399 | -0.34 | 0.113 |
| sp\|O60241\|AGRB2_HUMAN Adhesion G protein-coupled receptor B2 OS=Homo sapiens GN=ADGRB2 PE=1 S' | 876 | TDPHCAsWDYS | 0.123 | -0.119 | -1.118 | -0.371 |
| sp\|O60241\|AGRB2_HUMAN Adhesion G protein-coupled receptor B2 OS=Homo sapiens GN=ADGRB2 PE=1 S' | 880 | CASWDYsRADA | 0.232 | -0.167 | -0.583 | -0.173 |
| sp\|O60241\|AGRB2_HUMAN Adhesion G protein-coupled receptor B2 OS=Homo sapiens GN=ADGRB2 PE=1 S' | 885 | YSRADAsSGDW | 0.481 | 0.208 | -0.222 | 0.156 |
| sp\|O60241\|AGRB2_HUMAN Adhesion G protein-coupled receptor B2 OS=Homo sapiens GN=ADGRB2 PE=1 S' | 886 | SRADASsGDWD | 0.225 | 0.173 | -0.557 | -0.053 |
| sp\|O60241\|AGRB2_HUMAN Adhesion G protein-coupled receptor B2 OS=Homo sapiens GN=ADGRB2 PE=1 S' | 891 | SSGDWDtENCQ | 0.112 | -0.106 | -1.298 | -0.431 |
| sp\|O60241\|AGRB2_HUMAN Adhesion G protein-coupled receptor B2 OS=Homo sapiens GN=ADGRB2 PE=1 S' | 896 | DTENCQtLETQ | 0.406 | -0.038 | -0.429 | -0.02 |
| sp\|O60241\|AGRB2_HUMAN Adhesion G protein-coupled receptor B2 OS=Homo sapiens GN=ADGRB2 PE=1 S' | 899 | NCQTLEtQAAH | 0.101 | -0.206 | -1.693 | -0.599 |
| sp\|O60241\|AGRB2_HUMAN Adhesion G protein-coupled receptor B2 OS=Homo sapiens GN=ADGRB2 PE=1 S' | 904 | ETQAAHtRCQC | 0.215 | -0.123 | -1.059 | -0.322 |
| sp\|O60241\|AGRB2_HUMAN Adhesion G protein-coupled receptor B2 OS=Homo sapiens GN=ADGRB2 PE=1 S' | 912 | CQCQHLsTFAV | 0.301 | 0.072 | -0.475 | -0.034 |
| sp\|O60241\|AGRB2_HUMAN Adhesion G protein-coupled receptor B2 OS=Homo sapiens GN=ADGRB2 PE=1 S' | 913 | QCQHLStFAVL | 0.139 | -0.001 | -1.001 | -0.288 |
| sp\|O60241\|AGRB2_HUMAN Adhesion G protein-coupled receptor B2 OS=Homo sapiens GN=ADGRB2 PE=1 S' | 925 | QPPKDLtLELA | 0.167 | -0.103 | -0.79 | -0.242 |
| sp\|O60241\|AGRB2_HUMAN Adhesion G protein-coupled receptor B2 OS=Homo sapiens GN=ADGRB2 PE=1 S' | 931 | TLELAGsPSVP | 0.061 | -0.384 | -1.505 | -0.609 |
| sp\|O60241\|AGRB2_HUMAN Adhesion G protein-coupled receptor B2 OS=Homo sapiens GN=ADGRB2 PE=1 S' | 933 | ELAGSPsVPLV | 0.75 | 0.736 | 0.551 | 0.679 |
| sp\|O60241\|AGRB2_HUMAN Adhesion G protein-coupled receptor B2 OS=Homo sapiens GN=ADGRB2 PE=1 S' | 943 | VIGCAVsCMAL | 0.503 | 0.259 | -0.214 | 0.183 |
| sp\|O60241\|AGRB2_HUMAN Adhesion G protein-coupled receptor B2 OS=Homo sapiens GN=ADGRB2 PE=1 S' | 949 | SCMALLtLLAI | 0.189 | 0.065 | -0.736 | -0.161 |
| sp\|O60241\|AGRB2_HUMAN Adhesion G protein-coupled receptor B2 OS=Homo sapiens GN=ADGRB2 PE=1 S' | 963 | FWRFIKsERSI | 0.64 | 0.454 | 0.401 | 0.498 |
| sp\|O60241\|AGRB2_HUMAN Adhesion G protein-coupled receptor B2 OS=Homo sapiens GN=ADGRB2 PE=1 S' | 966 | FIKSERsIILL | 0.138 | 0.057 | -0.86 | -0.222 |
| sp\|O60241\|AGRB2_HUMAN Adhesion G protein-coupled receptor B2 OS=Homo sapiens GN=ADGRB2 PE=1 S' | 975 | LLNFCLsILAS | 0.172 | 0.019 | -0.76 | -0.19 |
| sp\|O60241\|AGRB2_HUMAN Adhesion G protein-coupled receptor B2 OS=Homo sapiens GN=ADGRB2 PE=1 S' | 979 | CLSILAsNILI | 0.31 | 0.102 | -0.49 | -0.026 |
| sp\|O60241\|AGRB2_HUMAN Adhesion G protein-coupled receptor B2 OS=Homo sapiens GN=ADGRB2 PE=1 S' | 988 | LILVGQsRVLS | 0.055 | -0.148 | -1.294 | -0.462 |
| sp\|O60241\|AGRB2_HUMAN Adhesion G protein-coupled receptor B2 OS=Homo sapiens GN=ADGRB2 PE=1 S' | 992 | GQSRVLsKGVC | 0.535 | 0.783 | 0.095 | 0.471 |
| sp\|O60241\|AGRB2_HUMAN Adhesion G protein-coupled receptor B2 OS=Homo sapiens GN=ADGRB2 PE=1 S' | 997 | LSKGVCtMTAA | 0.358 | 0.077 | -0.497 | -0.021 |
| sp\|O60241\|AGRB2_HUMAN Adhesion G protein-coupled receptor B2 OS=Homo sapiens GN=ADGRB2 PE=1 S' | 999 | KGVCTMtAAFL | 0.126 | 0.017 | -1.093 | -0.317 |
| sp\|O60241\|AGRB2_HUMAN Adhesion G protein-coupled receptor B2 OS=Homo sapiens GN=ADGRB2 PE=1 S' | 1009 | LHFFFLsSFCW | 0.085 | -0.047 | -1.428 | -0.463 |
| sp\|O60241\|AGRB2_HUMAN Adhesion G protein-coupled receptor B2 OS=Homo sapiens GN=ADGRB2 PE=1 S' | 1010 | HFFFLSsFCWV | 0.145 | -0.024 | -0.945 | -0.275 |
| sp\|O60241\|AGRB2_HUMAN Adhesion G protein-coupled receptor B2 OS=Homo sapiens GN=ADGRB2 PE=1 S' | 1016 | SFCWVLtEAWQ | 0.129 | -0.148 | -1.055 | -0.358 |
| sp\|O60241\|AGRB2_HUMAN Adhesion G protein-coupled receptor B2 OS=Homo sapiens GN=ADGRB2 PE=1 S' | 1021 | LTEAWQsYLAV | 0.181 | -0.063 | -0.889 | -0.257 |
| sp\|O60241\|AGRB2_HUMAN Adhesion G protein-coupled receptor B2 OS=Homo sapiens GN=ADGRB2 PE=1 S' | 1031 | VIGRMRtRLVR | 0.335 | 0.71 | -0.327 | 0.239 |
| sp\|O60241\|AGRB2_HUMAN Adhesion G protein-coupled receptor B2 OS=Homo sapiens GN=ADGRB2 PE=1 S' | 1053 | ALVVAVsVGFT | 0.332 | 0.022 | -0.515 | -0.054 |
| sp\|O60241\|AGRB2_HUMAN Adhesion G protein-coupled receptor B2 OS=Homo sapiens GN=ADGRB2 PE=1 S' | 1057 | AVSVGFtRTKG | 0.058 | -0.277 | -1.511 | -0.577 |

| | | | | | | | |
|---|---|---|---|---|---|---|---|
| sp\|O60241\|AGRB2_HUMAN Adhesion G protein-coupled receptor B2 OS=Homo sapiens GN=ADGRB2 PE=1 S' | 1059 | SVGFTRtKGYG | 0.083 | -0.062 | -1.33 | -0.436 |
| sp\|O60241\|AGRB2_HUMAN Adhesion G protein-coupled receptor B2 OS=Homo sapiens GN=ADGRB2 PE=1 S' | 1064 | RTKGYGtSSYC | 0.086 | -0.266 | -1.554 | -0.578 |
| sp\|O60241\|AGRB2_HUMAN Adhesion G protein-coupled receptor B2 OS=Homo sapiens GN=ADGRB2 PE=1 S' | 1065 | TKGYGTsSYCW | 0.099 | -0.014 | -0.94 | -0.285 |
| sp\|O60241\|AGRB2_HUMAN Adhesion G protein-coupled receptor B2 OS=Homo sapiens GN=ADGRB2 PE=1 S' | 1066 | KGYGTSsYCWL | 0.033 | -0.224 | -1.344 | -0.512 |
| sp\|O60241\|AGRB2_HUMAN Adhesion G protein-coupled receptor B2 OS=Homo sapiens GN=ADGRB2 PE=1 S' | 1071 | SSYCWLsLEGG | 0.17 | -0.122 | -0.827 | -0.26 |
| sp\|O60241\|AGRB2_HUMAN Adhesion G protein-coupled receptor B2 OS=Homo sapiens GN=ADGRB2 PE=1 S' | 1109 | MARDGIsDKSK | 0.251 | 0.105 | -0.793 | -0.146 |
| sp\|O60241\|AGRB2_HUMAN Adhesion G protein-coupled receptor B2 OS=Homo sapiens GN=ADGRB2 PE=1 S' | 1112 | DGISDKsKKQR | 0.235 | -0.122 | -0.554 | -0.147 |
| sp\|O60241\|AGRB2_HUMAN Adhesion G protein-coupled receptor B2 OS=Homo sapiens GN=ADGRB2 PE=1 S' | 1119 | KKQRAGsERCP | 0.726 | 0.942 | 0.423 | 0.697 |
| sp\|O60241\|AGRB2_HUMAN Adhesion G protein-coupled receptor B2 OS=Homo sapiens GN=ADGRB2 PE=1 S' | 1126 | ERCPWAsLLLP | 0.28 | 0.228 | -0.497 | 0.004 |
| sp\|O60241\|AGRB2_HUMAN Adhesion G protein-coupled receptor B2 OS=Homo sapiens GN=ADGRB2 PE=1 S' | 1132 | SLLLPCsACGA | 0.185 | -0.191 | -1.081 | -0.362 |
| sp\|O60241\|AGRB2_HUMAN Adhesion G protein-coupled receptor B2 OS=Homo sapiens GN=ADGRB2 PE=1 S' | 1139 | ACGAVPsPLLS | 0.049 | -0.483 | -1.87 | -0.768 |
| sp\|O60241\|AGRB2_HUMAN Adhesion G protein-coupled receptor B2 OS=Homo sapiens GN=ADGRB2 PE=1 S' | 1143 | VPSPLLsSASA | 0.023 | -0.464 | -1.889 | -0.777 |
| sp\|O60241\|AGRB2_HUMAN Adhesion G protein-coupled receptor B2 OS=Homo sapiens GN=ADGRB2 PE=1 S' | 1144 | PSPLLSsASAR | 0.068 | -0.198 | -1.454 | -0.528 |
| sp\|O60241\|AGRB2_HUMAN Adhesion G protein-coupled receptor B2 OS=Homo sapiens GN=ADGRB2 PE=1 S' | 1146 | PLLSSAsARNA | 0.625 | 0.28 | -0.096 | 0.27 |
| sp\|O60241\|AGRB2_HUMAN Adhesion G protein-coupled receptor B2 OS=Homo sapiens GN=ADGRB2 PE=1 S' | 1153 | ARNAMAsLWSS | 0.355 | 0.155 | -0.607 | -0.032 |
| sp\|O60241\|AGRB2_HUMAN Adhesion G protein-coupled receptor B2 OS=Homo sapiens GN=ADGRB2 PE=1 S' | 1156 | AMASLWsSCVV | 0.06 | -0.221 | -1.714 | -0.625 |
| sp\|O60241\|AGRB2_HUMAN Adhesion G protein-coupled receptor B2 OS=Homo sapiens GN=ADGRB2 PE=1 S' | 1157 | MASLWSsCVVL | 0.169 | 0.091 | -0.919 | -0.22 |
| sp\|O60241\|AGRB2_HUMAN Adhesion G protein-coupled receptor B2 OS=Homo sapiens GN=ADGRB2 PE=1 S' | 1167 | LPLLALtWMSA | 0.089 | -0.042 | -1.049 | -0.334 |
| sp\|O60241\|AGRB2_HUMAN Adhesion G protein-coupled receptor B2 OS=Homo sapiens GN=ADGRB2 PE=1 S' | 1170 | LALTWMsAVLA | 0.257 | 0.094 | -0.617 | -0.089 |
| sp\|O60241\|AGRB2_HUMAN Adhesion G protein-coupled receptor B2 OS=Homo sapiens GN=ADGRB2 PE=1 S' | 1176 | SAVLAMtDRRS | 0.225 | -0.062 | -1.159 | -0.332 |
| sp\|O60241\|AGRB2_HUMAN Adhesion G protein-coupled receptor B2 OS=Homo sapiens GN=ADGRB2 PE=1 S' | 1180 | AMTDRRsVLFQ | 0.104 | 0.048 | -1.355 | -0.401 |
| sp\|O60241\|AGRB2_HUMAN Adhesion G protein-coupled receptor B2 OS=Homo sapiens GN=ADGRB2 PE=1 S' | 1192 | LFAVFNsAQGF | 0.19 | -0.03 | -0.723 | -0.188 |
| sp\|O60241\|AGRB2_HUMAN Adhesion G protein-coupled receptor B2 OS=Homo sapiens GN=ADGRB2 PE=1 S' | 1199 | AQGFVItAVHC | 0.096 | -0.193 | -1.351 | -0.483 |
| sp\|O60241\|AGRB2_HUMAN Adhesion G protein-coupled receptor B2 OS=Homo sapiens GN=ADGRB2 PE=1 S' | 1225 | VCRADEsEDSP | 0.244 | 0.109 | -0.833 | -0.16 |
| sp\|O60241\|AGRB2_HUMAN Adhesion G protein-coupled receptor B2 OS=Homo sapiens GN=ADGRB2 PE=1 S' | 1228 | ADESEDsPDSC | 0.026 | -0.741 | -2.305 | -1.007 |
| sp\|O60241\|AGRB2_HUMAN Adhesion G protein-coupled receptor B2 OS=Homo sapiens GN=ADGRB2 PE=1 S' | 1231 | SEDSPDsCKNG | 0.068 | -0.278 | -1.766 | -0.659 |
| sp\|O60241\|AGRB2_HUMAN Adhesion G protein-coupled receptor B2 OS=Homo sapiens GN=ADGRB2 PE=1 S' | 1241 | GQLQILsDFEK | 0.269 | -0.032 | -0.428 | -0.064 |
| sp\|O60241\|AGRB2_HUMAN Adhesion G protein-coupled receptor B2 OS=Homo sapiens GN=ADGRB2 PE=1 S' | 1253 | VDLACQtVLFK | 0.085 | -0.297 | -1.149 | -0.454 |
| sp\|O60241\|AGRB2_HUMAN Adhesion G protein-coupled receptor B2 OS=Homo sapiens GN=ADGRB2 PE=1 S' | 1261 | LFKEVNtCNPS | 0.239 | 0.066 | -0.6 | -0.098 |
| sp\|O60241\|AGRB2_HUMAN Adhesion G protein-coupled receptor B2 OS=Homo sapiens GN=ADGRB2 PE=1 S' | 1265 | VNTCNPsTITG | 0.14 | -0.263 | -0.845 | -0.323 |
| sp\|O60241\|AGRB2_HUMAN Adhesion G protein-coupled receptor B2 OS=Homo sapiens GN=ADGRB2 PE=1 S' | 1266 | NTCNPStITGT | 0.076 | -0.362 | -1.112 | -0.466 |
| sp\|O60241\|AGRB2_HUMAN Adhesion G protein-coupled receptor B2 OS=Homo sapiens GN=ADGRB2 PE=1 S' | 1268 | CNPSTItGTLS | 0.071 | -0.257 | -1.218 | -0.468 |
| sp\|O60241\|AGRB2_HUMAN Adhesion G protein-coupled receptor B2 OS=Homo sapiens GN=ADGRB2 PE=1 S' | 1270 | PSTITGtLSRL | 0.125 | -0.103 | -0.981 | -0.32 |
| sp\|O60241\|AGRB2_HUMAN Adhesion G protein-coupled receptor B2 OS=Homo sapiens GN=ADGRB2 PE=1 S' | 1272 | TITGTLsRLSL | 0.042 | -0.123 | -1.442 | -0.508 |
| sp\|O60241\|AGRB2_HUMAN Adhesion G protein-coupled receptor B2 OS=Homo sapiens GN=ADGRB2 PE=1 S' | 1275 | GTLSRLsLDED | 0.65 | 0.218 | 0.019 | 0.296 |
| sp\|O60241\|AGRB2_HUMAN Adhesion G protein-coupled receptor B2 OS=Homo sapiens GN=ADGRB2 PE=1 S' | 1284 | EDEEPKsCLVG | 0.076 | -0.121 | -1.083 | -0.376 |
| sp\|O60241\|AGRB2_HUMAN Adhesion G protein-coupled receptor B2 OS=Homo sapiens GN=ADGRB2 PE=1 S' | 1292 | LVGPEGsLSFS | 0.036 | -0.31 | -1.567 | -0.614 |
| sp\|O60241\|AGRB2_HUMAN Adhesion G protein-coupled receptor B2 OS=Homo sapiens GN=ADGRB2 PE=1 S' | 1294 | GPEGSLsFSPL | 0.218 | 0.117 | -0.464 | -0.043 |
| sp\|O60241\|AGRB2_HUMAN Adhesion G protein-coupled receptor B2 OS=Homo sapiens GN=ADGRB2 PE=1 S' | 1296 | EGSLSFsPLPG | 0.091 | -0.211 | -0.974 | -0.365 |
| sp\|O60241\|AGRB2_HUMAN Adhesion G protein-coupled receptor B2 OS=Homo sapiens GN=ADGRB2 PE=1 S' | 1309 | LVPMAAsPGLG | 0.149 | -0.253 | -0.93 | -0.345 |
| sp\|O60241\|AGRB2_HUMAN Adhesion G protein-coupled receptor B2 OS=Homo sapiens GN=ADGRB2 PE=1 S' | 1338 | LRQLDLtWLRP | 0.066 | 0.064 | -1.35 | -0.407 |
| sp\|O60241\|AGRB2_HUMAN Adhesion G protein-coupled receptor B2 OS=Homo sapiens GN=ADGRB2 PE=1 S' | 1343 | LTWLRPtEPGS | 0.633 | 0.643 | 0.139 | 0.472 |
| sp\|O60241\|AGRB2_HUMAN Adhesion G protein-coupled receptor B2 OS=Homo sapiens GN=ADGRB2 PE=1 S' | 1347 | RPTEPGsEGDY | 0.247 | 0.01 | -0.721 | -0.155 |
| sp\|O60241\|AGRB2_HUMAN Adhesion G protein-coupled receptor B2 OS=Homo sapiens GN=ADGRB2 PE=1 S' | 1358 | MVLPRRtLSLQ | 0.285 | 0.138 | -0.567 | -0.048 |
| sp\|O60241\|AGRB2_HUMAN Adhesion G protein-coupled receptor B2 OS=Homo sapiens GN=ADGRB2 PE=1 S' | 1360 | LPRRTLsLQPG | 0.731 | 1.09 | 0.772 | 0.864 |
| sp\|O60241\|AGRB2_HUMAN Adhesion G protein-coupled receptor B2 OS=Homo sapiens GN=ADGRB2 PE=1 S' | 1381 | RARPEGtPRRA | 0.12 | -0.422 | -1.442 | -0.581 |
| sp\|O60241\|AGRB2_HUMAN Adhesion G protein-coupled receptor B2 OS=Homo sapiens GN=ADGRB2 PE=1 S' | 1388 | PRRAAKtVAHT | 0.548 | 0.419 | 0.204 | 0.39 |
| sp\|O60241\|AGRB2_HUMAN Adhesion G protein-coupled receptor B2 OS=Homo sapiens GN=ADGRB2 PE=1 S' | 1392 | AKTVAHtEGYP | 0.481 | 0.221 | -0.289 | 0.138 |
| sp\|O60241\|AGRB2_HUMAN Adhesion G protein-coupled receptor B2 OS=Homo sapiens GN=ADGRB2 PE=1 S' | 1397 | HTEGYPsFLSV | 0.132 | -0.067 | -1.05 | -0.328 |

| | | | | | | | |
|---|---|---|---|---|---|---|---|
| sp\|O60241\|AGRB2_HUMAN Adhesion G protein-coupled receptor B2 OS=Homo sapiens GN=ADGRB2 PE=1 S' | 1400 | GYPSFLsVDHS | 0.101 | -0.163 | -1.21 | -0.424 |
| sp\|O60241\|AGRB2_HUMAN Adhesion G protein-coupled receptor B2 OS=Homo sapiens GN=ADGRB2 PE=1 S' | 1404 | FLSVDHsGLGL | 0.205 | 0.091 | -0.697 | -0.134 |
| sp\|O60241\|AGRB2_HUMAN Adhesion G protein-coupled receptor B2 OS=Homo sapiens GN=ADGRB2 PE=1 S' | 1414 | LGPAYGsLQNP | 0.144 | -0.136 | -0.925 | -0.306 |
| sp\|O60241\|AGRB2_HUMAN Adhesion G protein-coupled receptor B2 OS=Homo sapiens GN=ADGRB2 PE=1 S' | 1422 | QNPYGMtFQPP | 0.089 | -0.133 | -0.855 | -0.3 |
| sp\|O60241\|AGRB2_HUMAN Adhesion G protein-coupled receptor B2 OS=Homo sapiens GN=ADGRB2 PE=1 S' | 1429 | FQPPPPtPSAR | 0.036 | -0.51 | -1.393 | -0.622 |
| sp\|O60241\|AGRB2_HUMAN Adhesion G protein-coupled receptor B2 OS=Homo sapiens GN=ADGRB2 PE=1 S' | 1431 | PPPPTPsARQV | 0.088 | -0.244 | -1.432 | -0.529 |
| sp\|O60241\|AGRB2_HUMAN Adhesion G protein-coupled receptor B2 OS=Homo sapiens GN=ADGRB2 PE=1 S' | 1442 | PEPGERsRTMP | 0.051 | -0.388 | -1.969 | -0.769 |
| sp\|O60241\|AGRB2_HUMAN Adhesion G protein-coupled receptor B2 OS=Homo sapiens GN=ADGRB2 PE=1 S' | 1444 | PGERSRtMPRT | 0.903 | 1.518 | 1.216 | 1.212 |
| sp\|O60241\|AGRB2_HUMAN Adhesion G protein-coupled receptor B2 OS=Homo sapiens GN=ADGRB2 PE=1 S' | 1448 | SRTMPRtVPGS | 0.335 | 0.438 | -0.304 | 0.156 |
| sp\|O60241\|AGRB2_HUMAN Adhesion G protein-coupled receptor B2 OS=Homo sapiens GN=ADGRB2 PE=1 S' | 1452 | PRTVPGsTMKM | 0.085 | -0.029 | -1.078 | -0.341 |
| sp\|O60241\|AGRB2_HUMAN Adhesion G protein-coupled receptor B2 OS=Homo sapiens GN=ADGRB2 PE=1 S' | 1453 | RTVPGStMKMG | 0.062 | -0.187 | -1.497 | -0.541 |
| sp\|O60241\|AGRB2_HUMAN Adhesion G protein-coupled receptor B2 OS=Homo sapiens GN=ADGRB2 PE=1 S' | 1458 | STMKMGsLERK | 0.25 | -0.196 | -0.948 | -0.298 |
| sp\|O60241\|AGRB2_HUMAN Adhesion G protein-coupled receptor B2 OS=Homo sapiens GN=ADGRB2 PE=1 S' | 1467 | RKKLRYsDLDF | 0.662 | 0.4 | 0.091 | 0.384 |
| sp\|O60241\|AGRB2_HUMAN Adhesion G protein-coupled receptor B2 OS=Homo sapiens GN=ADGRB2 PE=1 S' | 1477 | FEKVMHtRKRH | 0.144 | -0.199 | -1.311 | -0.455 |
| sp\|O60241\|AGRB2_HUMAN Adhesion G protein-coupled receptor B2 OS=Homo sapiens GN=ADGRB2 PE=1 S' | 1482 | HTRKRHsELYH | 0.69 | 0.488 | -0.117 | 0.354 |
| sp\|O60241\|AGRB2_HUMAN Adhesion G protein-coupled receptor B2 OS=Homo sapiens GN=ADGRB2 PE=1 S' | 1494 | LNQKFHtFDRY | 0.599 | 0.175 | -0.2 | 0.191 |
| sp\|O60241\|AGRB2_HUMAN Adhesion G protein-coupled receptor B2 OS=Homo sapiens GN=ADGRB2 PE=1 S' | 1500 | TFDRYRsQSTA | 0.377 | 0.636 | -0.35 | 0.221 |
| sp\|O60241\|AGRB2_HUMAN Adhesion G protein-coupled receptor B2 OS=Homo sapiens GN=ADGRB2 PE=1 S' | 1502 | DRYRSQsTAKR | 0.747 | 1.194 | 0.656 | 0.866 |
| sp\|O60241\|AGRB2_HUMAN Adhesion G protein-coupled receptor B2 OS=Homo sapiens GN=ADGRB2 PE=1 S' | 1503 | RYRSQStAKRE | 0.236 | 0.063 | -1.073 | -0.258 |
| sp\|O60241\|AGRB2_HUMAN Adhesion G protein-coupled receptor B2 OS=Homo sapiens GN=ADGRB2 PE=1 S' | 1511 | KREKRWsVSSG | 0.358 | 0.224 | -0.472 | 0.037 |
| sp\|O60241\|AGRB2_HUMAN Adhesion G protein-coupled receptor B2 OS=Homo sapiens GN=ADGRB2 PE=1 S' | 1513 | EKRWSVsSGGA | 0.841 | 0.428 | 0.811 | 0.693 |
| sp\|O60241\|AGRB2_HUMAN Adhesion G protein-coupled receptor B2 OS=Homo sapiens GN=ADGRB2 PE=1 S' | 1514 | KRWSVSsGGAA | 0.171 | 0.177 | -0.694 | -0.115 |
| sp\|O60241\|AGRB2_HUMAN Adhesion G protein-coupled receptor B2 OS=Homo sapiens GN=ADGRB2 PE=1 S' | 1521 | GGAAERsVCTD | 0.175 | -0.184 | -1.013 | -0.341 |
| sp\|O60241\|AGRB2_HUMAN Adhesion G protein-coupled receptor B2 OS=Homo sapiens GN=ADGRB2 PE=1 S' | 1524 | AERSVCtDKPS | 0.308 | 0.033 | -1.047 | -0.235 |
| sp\|O60241\|AGRB2_HUMAN Adhesion G protein-coupled receptor B2 OS=Homo sapiens GN=ADGRB2 PE=1 S' | 1528 | VCTDKPsPGER | 0.137 | -0.336 | -1.183 | -0.461 |
| sp\|O60241\|AGRB2_HUMAN Adhesion G protein-coupled receptor B2 OS=Homo sapiens GN=ADGRB2 PE=1 S' | 1534 | SPGERPsLSQH | 0.083 | -0.204 | -1.368 | -0.496 |
| sp\|O60241\|AGRB2_HUMAN Adhesion G protein-coupled receptor B2 OS=Homo sapiens GN=ADGRB2 PE=1 S' | 1536 | GERPSLsQHRR | 0.486 | 0.252 | -0.534 | 0.068 |
| sp\|O60241\|AGRB2_HUMAN Adhesion G protein-coupled receptor B2 OS=Homo sapiens GN=ADGRB2 PE=1 S' | 1543 | QHRRHQsWSTF | 0.608 | 1.099 | 0.243 | 0.65 |
| sp\|O60241\|AGRB2_HUMAN Adhesion G protein-coupled receptor B2 OS=Homo sapiens GN=ADGRB2 PE=1 S' | 1545 | RRHQSWsTFKS | 0.344 | 0.318 | -0.348 | 0.105 |
| sp\|O60241\|AGRB2_HUMAN Adhesion G protein-coupled receptor B2 OS=Homo sapiens GN=ADGRB2 PE=1 S' | 1546 | RHQSWStFKSM | 0.113 | -0.045 | -1.119 | -0.35 |
| sp\|O60241\|AGRB2_HUMAN Adhesion G protein-coupled receptor B2 OS=Homo sapiens GN=ADGRB2 PE=1 S' | 1549 | SWSTFKsMTLG | 0.238 | 0.043 | -0.959 | -0.226 |
| sp\|O60241\|AGRB2_HUMAN Adhesion G protein-coupled receptor B2 OS=Homo sapiens GN=ADGRB2 PE=1 S' | 1551 | STFKSMtLGSL | 0.702 | 0.456 | 0.276 | 0.478 |
| sp\|O60241\|AGRB2_HUMAN Adhesion G protein-coupled receptor B2 OS=Homo sapiens GN=ADGRB2 PE=1 S' | 1554 | KSMTLGsLPPK | 0.373 | 0.247 | -0.395 | 0.075 |
| sp\|O60241\|AGRB2_HUMAN Adhesion G protein-coupled receptor B2 OS=Homo sapiens GN=ADGRB2 PE=1 S' | 1564 | KPRERLtLHRA | 0.473 | 0.243 | -0.369 | 0.116 |
| sp\|O60241\|AGRB2_HUMAN Adhesion G protein-coupled receptor B2 OS=Homo sapiens GN=ADGRB2 PE=1 S' | 1574 | AAAWEPtEPPD | 0.576 | 0.295 | -0.023 | 0.283 |
| sp\|O60241\|AGRB2_HUMAN Adhesion G protein-coupled receptor B2 OS=Homo sapiens GN=ADGRB2 PE=1 S' | 1583 | PDGDFQtEV-- | 0.115 | 0.078 | -1.148 | -0.318 |
| sp\|Q8IZP9\|AGRG2_HUMAN Adhesion G-protein coupled receptor G2 OS=Homo sapiens GN=ADGRG2 PE=1 S' | 322 | IDMPPQsETIS | 0.124 | -0.188 | -0.884 | -0.316 |
| sp\|Q8IZP9\|AGRG2_HUMAN Adhesion G-protein coupled receptor G2 OS=Homo sapiens GN=ADGRG2 PE=1 S' | 324 | MPPQSEtISSP | 0.079 | -0.096 | -1.16 | -0.392 |
| sp\|Q8IZP9\|AGRG2_HUMAN Adhesion G-protein coupled receptor G2 OS=Homo sapiens GN=ADGRG2 PE=1 S' | 326 | PQSETIsSPMP | 0.181 | 0.274 | -0.597 | -0.047 |
| sp\|Q8IZP9\|AGRG2_HUMAN Adhesion G-protein coupled receptor G2 OS=Homo sapiens GN=ADGRG2 PE=1 S' | 327 | QSETISsPMPQ | 0.038 | -0.619 | -1.653 | -0.745 |
| sp\|Q8IZP9\|AGRG2_HUMAN Adhesion G-protein coupled receptor G2 OS=Homo sapiens GN=ADGRG2 PE=1 S' | 332 | SSPMPQtHVSG | 0.052 | -0.366 | -1.479 | -0.598 |
| sp\|Q8IZP9\|AGRG2_HUMAN Adhesion G-protein coupled receptor G2 OS=Homo sapiens GN=ADGRG2 PE=1 S' | 335 | MPQTHVsGTPP | 0.293 | -0.085 | -0.813 | -0.202 |
| sp\|Q8IZP9\|AGRG2_HUMAN Adhesion G-protein coupled receptor G2 OS=Homo sapiens GN=ADGRG2 PE=1 S' | 337 | QTHVSGtPPPV | 0.188 | 0.144 | -0.922 | -0.197 |
| sp\|Q8IZP9\|AGRG2_HUMAN Adhesion G-protein coupled receptor G2 OS=Homo sapiens GN=ADGRG2 PE=1 S' | 344 | PPPVKAsFSSP | 0.037 | -0.261 | -1.646 | -0.623 |
| sp\|Q8IZP9\|AGRG2_HUMAN Adhesion G-protein coupled receptor G2 OS=Homo sapiens GN=ADGRG2 PE=1 S' | 346 | PVKASFsSPTV | 0.754 | 0.696 | 0.455 | 0.635 |
| sp\|Q8IZP9\|AGRG2_HUMAN Adhesion G-protein coupled receptor G2 OS=Homo sapiens GN=ADGRG2 PE=1 S' | 347 | VKASFSsPTVS | 0.078 | -0.451 | -1.452 | -0.608 |
| sp\|Q8IZP9\|AGRG2_HUMAN Adhesion G-protein coupled receptor G2 OS=Homo sapiens GN=ADGRG2 PE=1 S' | 349 | ASFSSPtVSAP | 0.178 | -0.061 | -0.799 | -0.227 |
| sp\|Q8IZP9\|AGRG2_HUMAN Adhesion G-protein coupled receptor G2 OS=Homo sapiens GN=ADGRG2 PE=1 S' | 351 | FSSPTVsAPAN | 0.63 | 0.568 | 0.157 | 0.452 |
| sp\|Q8IZP9\|AGRG2_HUMAN Adhesion G-protein coupled receptor G2 OS=Homo sapiens GN=ADGRG2 PE=1 S' | 358 | APANVNtTSAP | 0.109 | -0.154 | -1.079 | -0.375 |

| | | | | | | |
|---|---|---|---|---|---|---|
| sp\|Q8IZP9\|AGRG2_HUMAN Adhesion G-protein coupled receptor G2 OS=Homo sapiens GN=ADGRG2 PE=1 S' | 359 | PANVNTtSAPP | 0.052 | -0.325 | -1.478 | -0.584 |
| sp\|Q8IZP9\|AGRG2_HUMAN Adhesion G-protein coupled receptor G2 OS=Homo sapiens GN=ADGRG2 PE=1 S' | 360 | ANVNTTsAPPV | 0.273 | 0.345 | -0.563 | 0.018 |
| sp\|Q8IZP9\|AGRG2_HUMAN Adhesion G-protein coupled receptor G2 OS=Homo sapiens GN=ADGRG2 PE=1 S' | 366 | SAPPVQtDIVN | 0.076 | -0.17 | -1.418 | -0.504 |
| sp\|Q8IZP9\|AGRG2_HUMAN Adhesion G-protein coupled receptor G2 OS=Homo sapiens GN=ADGRG2 PE=1 S' | 371 | QTDIVNtSSIS | 0.114 | -0.187 | -1.477 | -0.517 |
| sp\|Q8IZP9\|AGRG2_HUMAN Adhesion G-protein coupled receptor G2 OS=Homo sapiens GN=ADGRG2 PE=1 S' | 372 | TDIVNTsSISD | 0.04 | -0.229 | -1.29 | -0.493 |
| sp\|Q8IZP9\|AGRG2_HUMAN Adhesion G-protein coupled receptor G2 OS=Homo sapiens GN=ADGRG2 PE=1 S' | 373 | DIVNTSsISDL | 0.084 | -0.025 | -1.035 | -0.325 |
| sp\|Q8IZP9\|AGRG2_HUMAN Adhesion G-protein coupled receptor G2 OS=Homo sapiens GN=ADGRG2 PE=1 S' | 375 | VNTSSIsDLEN | 0.294 | 0.054 | -0.586 | -0.079 |
| sp\|Q8IZP9\|AGRG2_HUMAN Adhesion G-protein coupled receptor G2 OS=Homo sapiens GN=ADGRG2 PE=1 S' | 389 | QMEKALsLGSL | 0.367 | 0.265 | -0.136 | 0.165 |
| sp\|Q8IZP9\|AGRG2_HUMAN Adhesion G-protein coupled receptor G2 OS=Homo sapiens GN=ADGRG2 PE=1 S' | 392 | KALSLGsLEPN | 0.09 | -0.26 | -1.294 | -0.488 |
| sp\|Q8IZP9\|AGRG2_HUMAN Adhesion G-protein coupled receptor G2 OS=Homo sapiens GN=ADGRG2 PE=1 S' | 406 | EMINQVsRLLH | 0.043 | -0.155 | -1.601 | -0.571 |
| sp\|Q8IZP9\|AGRG2_HUMAN Adhesion G-protein coupled receptor G2 OS=Homo sapiens GN=ADGRG2 PE=1 S' | 411 | VSRLLHsPPDM | 0.633 | 0.453 | 0.009 | 0.365 |
| sp\|Q8IZP9\|AGRG2_HUMAN Adhesion G-protein coupled receptor G2 OS=Homo sapiens GN=ADGRG2 PE=1 S' | 437 | GLQLNFsNTTI | 0.576 | 0.179 | -0.228 | 0.176 |
| sp\|Q8IZP9\|AGRG2_HUMAN Adhesion G-protein coupled receptor G2 OS=Homo sapiens GN=ADGRG2 PE=1 S' | 439 | QLNFSNtTISL | 0.347 | 0.301 | -0.298 | 0.117 |
| sp\|Q8IZP9\|AGRG2_HUMAN Adhesion G-protein coupled receptor G2 OS=Homo sapiens GN=ADGRG2 PE=1 S' | 440 | LNFSNTtISLT | 0.058 | -0.316 | -1.12 | -0.459 |
| sp\|Q8IZP9\|AGRG2_HUMAN Adhesion G-protein coupled receptor G2 OS=Homo sapiens GN=ADGRG2 PE=1 S' | 442 | FSNTTIsLTSP | 0.149 | -0.058 | -0.974 | -0.294 |
| sp\|Q8IZP9\|AGRG2_HUMAN Adhesion G-protein coupled receptor G2 OS=Homo sapiens GN=ADGRG2 PE=1 S' | 444 | NTTISLtSPSL | 0.445 | 0.596 | 0.002 | 0.348 |
| sp\|Q8IZP9\|AGRG2_HUMAN Adhesion G-protein coupled receptor G2 OS=Homo sapiens GN=ADGRG2 PE=1 S' | 445 | TTISLTsPSLA | 0.033 | -0.537 | -1.822 | -0.775 |
| sp\|Q8IZP9\|AGRG2_HUMAN Adhesion G-protein coupled receptor G2 OS=Homo sapiens GN=ADGRG2 PE=1 S' | 447 | ISLTSPsLALA | 0.459 | 0.065 | -0.038 | 0.162 |
| sp\|Q8IZP9\|AGRG2_HUMAN Adhesion G-protein coupled receptor G2 OS=Homo sapiens GN=ADGRG2 PE=1 S' | 458 | VIRVNAsSFNT | 0.335 | 0.224 | -0.528 | 0.01 |
| sp\|Q8IZP9\|AGRG2_HUMAN Adhesion G-protein coupled receptor G2 OS=Homo sapiens GN=ADGRG2 PE=1 S' | 459 | IRVNASsFNTT | 0.294 | 0.208 | -0.155 | 0.116 |
| sp\|Q8IZP9\|AGRG2_HUMAN Adhesion G-protein coupled receptor G2 OS=Homo sapiens GN=ADGRG2 PE=1 S' | 462 | NASSFNtTTFV | 0.149 | -0.249 | -1.544 | -0.548 |
| sp\|Q8IZP9\|AGRG2_HUMAN Adhesion G-protein coupled receptor G2 OS=Homo sapiens GN=ADGRG2 PE=1 S' | 463 | ASSFNTtTFVA | 0.212 | -0.142 | -0.719 | -0.216 |
| sp\|Q8IZP9\|AGRG2_HUMAN Adhesion G-protein coupled receptor G2 OS=Homo sapiens GN=ADGRG2 PE=1 S' | 464 | SSFNTTtFVAQ | 0.115 | -0.02 | -0.829 | -0.245 |
| sp\|Q8IZP9\|AGRG2_HUMAN Adhesion G-protein coupled receptor G2 OS=Homo sapiens GN=ADGRG2 PE=1 S' | 476 | PANLQVsLETQ | 0.267 | -0.07 | -0.869 | -0.224 |
| sp\|Q8IZP9\|AGRG2_HUMAN Adhesion G-protein coupled receptor G2 OS=Homo sapiens GN=ADGRG2 PE=1 S' | 479 | LQVSLEtQAPE | 0.035 | -0.43 | -1.87 | -0.755 |
| sp\|Q8IZP9\|AGRG2_HUMAN Adhesion G-protein coupled receptor G2 OS=Homo sapiens GN=ADGRG2 PE=1 S' | 485 | TQAPENsIGTI | 0.225 | -0.093 | -0.529 | -0.132 |
| sp\|Q8IZP9\|AGRG2_HUMAN Adhesion G-protein coupled receptor G2 OS=Homo sapiens GN=ADGRG2 PE=1 S' | 488 | PENSIGtITLP | 0.065 | -0.302 | -1.466 | -0.568 |
| sp\|Q8IZP9\|AGRG2_HUMAN Adhesion G-protein coupled receptor G2 OS=Homo sapiens GN=ADGRG2 PE=1 S' | 490 | NSIGTItLPSS | 0.39 | 0.476 | -0.062 | 0.268 |
| sp\|Q8IZP9\|AGRG2_HUMAN Adhesion G-protein coupled receptor G2 OS=Homo sapiens GN=ADGRG2 PE=1 S' | 493 | GTITLPsSLMN | 0.088 | -0.238 | -1.682 | -0.611 |
| sp\|Q8IZP9\|AGRG2_HUMAN Adhesion G-protein coupled receptor G2 OS=Homo sapiens GN=ADGRG2 PE=1 S' | 494 | TITLPSsLMNN | 0.212 | 0.028 | -0.742 | -0.167 |
| sp\|Q8IZP9\|AGRG2_HUMAN Adhesion G-protein coupled receptor G2 OS=Homo sapiens GN=ADGRG2 PE=1 S' | 508 | HDMELAsRVQF | 0.051 | -0.201 | -1.446 | -0.532 |
| sp\|Q8IZP9\|AGRG2_HUMAN Adhesion G-protein coupled receptor G2 OS=Homo sapiens GN=ADGRG2 PE=1 S' | 517 | QFNFFEtPALF | 0.05 | -0.444 | -1.662 | -0.685 |
| sp\|Q8IZP9\|AGRG2_HUMAN Adhesion G-protein coupled receptor G2 OS=Homo sapiens GN=ADGRG2 PE=1 S' | 525 | ALFQDPsLENL | 0.439 | 0.118 | -0.343 | 0.071 |
| sp\|Q8IZP9\|AGRG2_HUMAN Adhesion G-protein coupled receptor G2 OS=Homo sapiens GN=ADGRG2 PE=1 S' | 530 | PSLENLsLISY | 0.125 | -0.06 | -0.726 | -0.22 |
| sp\|Q8IZP9\|AGRG2_HUMAN Adhesion G-protein coupled receptor G2 OS=Homo sapiens GN=ADGRG2 PE=1 S' | 533 | ENLSLIsYVIS | 0.055 | -0.329 | -1.414 | -0.563 |
| sp\|Q8IZP9\|AGRG2_HUMAN Adhesion G-protein coupled receptor G2 OS=Homo sapiens GN=ADGRG2 PE=1 S' | 537 | LISYVIsSSVA | 0.147 | -0.071 | -0.845 | -0.256 |
| sp\|Q8IZP9\|AGRG2_HUMAN Adhesion G-protein coupled receptor G2 OS=Homo sapiens GN=ADGRG2 PE=1 S' | 538 | ISYVISsSVAN | 0.074 | -0.155 | -1.027 | -0.369 |
| sp\|Q8IZP9\|AGRG2_HUMAN Adhesion G-protein coupled receptor G2 OS=Homo sapiens GN=ADGRG2 PE=1 S' | 539 | SYVISSsVANL | 0.17 | 0.155 | -0.918 | -0.198 |
| sp\|Q8IZP9\|AGRG2_HUMAN Adhesion G-protein coupled receptor G2 OS=Homo sapiens GN=ADGRG2 PE=1 S' | 544 | SSVANLtVRNL | 0.092 | -0.201 | -1.231 | -0.447 |
| sp\|Q8IZP9\|AGRG2_HUMAN Adhesion G-protein coupled receptor G2 OS=Homo sapiens GN=ADGRG2 PE=1 S' | 549 | LTVRNLtRNVT | 0.427 | 0.686 | 0.034 | 0.382 |
| sp\|Q8IZP9\|AGRG2_HUMAN Adhesion G-protein coupled receptor G2 OS=Homo sapiens GN=ADGRG2 PE=1 S' | 553 | NLTRNVtVTLK | 0.881 | 0.934 | 0.968 | 0.928 |
| sp\|Q8IZP9\|AGRG2_HUMAN Adhesion G-protein coupled receptor G2 OS=Homo sapiens GN=ADGRG2 PE=1 S' | 555 | TRNVTVtLKHI | 0.277 | 0.228 | -0.264 | 0.08 |
| sp\|Q8IZP9\|AGRG2_HUMAN Adhesion G-protein coupled receptor G2 OS=Homo sapiens GN=ADGRG2 PE=1 S' | 562 | LKHINPsQDEL | 0.267 | -0.008 | -0.668 | -0.136 |
| sp\|Q8IZP9\|AGRG2_HUMAN Adhesion G-protein coupled receptor G2 OS=Homo sapiens GN=ADGRG2 PE=1 S' | 567 | PSQDELtVRCV | 0.151 | -0.09 | -1.304 | -0.414 |
| sp\|Q8IZP9\|AGRG2_HUMAN Adhesion G-protein coupled receptor G2 OS=Homo sapiens GN=ADGRG2 PE=1 S' | 585 | GGRGGWsDNGC | 0.347 | 0.074 | -0.528 | -0.036 |
| sp\|Q8IZP9\|AGRG2_HUMAN Adhesion G-protein coupled receptor G2 OS=Homo sapiens GN=ADGRG2 PE=1 S' | 590 | WSDNGCsVKDR | 0.157 | -0.157 | -0.792 | -0.264 |
| sp\|Q8IZP9\|AGRG2_HUMAN Adhesion G-protein coupled receptor G2 OS=Homo sapiens GN=ADGRG2 PE=1 S' | 599 | DRRLNEtICTC | 0.281 | 0.134 | -0.958 | -0.181 |
| sp\|Q8IZP9\|AGRG2_HUMAN Adhesion G-protein coupled receptor G2 OS=Homo sapiens GN=ADGRG2 PE=1 S' | 602 | LNETICtCSHL | 0.11 | -0.154 | -0.961 | -0.335 |
| sp\|Q8IZP9\|AGRG2_HUMAN Adhesion G-protein coupled receptor G2 OS=Homo sapiens GN=ADGRG2 PE=1 S' | 604 | ETICTCsHLTS | 0.15 | -0.081 | -1.336 | -0.422 |

| | | | | | | |
|---|---|---|---|---|---|---|
| sp\|Q8IZP9\|AGRG2_HUMAN Adhesion G-protein coupled receptor G2 OS=Homo sapiens GN=ADGRG2 PE=1 S' | 607 | CTCSHLtSFGV | 0.201 | -0.137 | -1.087 | -0.341 |
| sp\|Q8IZP9\|AGRG2_HUMAN Adhesion G-protein coupled receptor G2 OS=Homo sapiens GN=ADGRG2 PE=1 S' | 608 | TCSHLTsFGVL | 0.286 | 0.077 | -0.568 | -0.068 |
| sp\|Q8IZP9\|AGRG2_HUMAN Adhesion G-protein coupled receptor G2 OS=Homo sapiens GN=ADGRG2 PE=1 S' | 616 | GVLLDLsRTSV | 0.082 | -0.225 | -1.633 | -0.592 |
| sp\|Q8IZP9\|AGRG2_HUMAN Adhesion G-protein coupled receptor G2 OS=Homo sapiens GN=ADGRG2 PE=1 S' | 618 | LLDLSRtSVLP | 0.173 | 0.136 | -0.826 | -0.172 |
| sp\|Q8IZP9\|AGRG2_HUMAN Adhesion G-protein coupled receptor G2 OS=Homo sapiens GN=ADGRG2 PE=1 S' | 619 | LDLSRTsVLPA | 0.114 | -0.14 | -0.873 | -0.3 |
| sp\|Q8IZP9\|AGRG2_HUMAN Adhesion G-protein coupled receptor G2 OS=Homo sapiens GN=ADGRG2 PE=1 S' | 629 | AQMMALtFITY | 0.218 | 0.021 | -0.489 | -0.083 |
| sp\|Q8IZP9\|AGRG2_HUMAN Adhesion G-protein coupled receptor G2 OS=Homo sapiens GN=ADGRG2 PE=1 S' | 632 | MALTFItYIGC | 0.126 | -0.216 | -1.115 | -0.402 |
| sp\|Q8IZP9\|AGRG2_HUMAN Adhesion G-protein coupled receptor G2 OS=Homo sapiens GN=ADGRG2 PE=1 S' | 639 | YIGCGLsSIFL | 0.042 | -0.17 | -1.401 | -0.51 |
| sp\|Q8IZP9\|AGRG2_HUMAN Adhesion G-protein coupled receptor G2 OS=Homo sapiens GN=ADGRG2 PE=1 S' | 640 | IGCGLSsIFLS | 0.076 | -0.217 | -1.122 | -0.421 |
| sp\|Q8IZP9\|AGRG2_HUMAN Adhesion G-protein coupled receptor G2 OS=Homo sapiens GN=ADGRG2 PE=1 S' | 644 | LSSIFLsVTLV | 0.105 | -0.171 | -1.352 | -0.473 |
| sp\|Q8IZP9\|AGRG2_HUMAN Adhesion G-protein coupled receptor G2 OS=Homo sapiens GN=ADGRG2 PE=1 S' | 646 | SIFLSVtLVTY | 0.654 | 0.338 | 0.015 | 0.336 |
| sp\|Q8IZP9\|AGRG2_HUMAN Adhesion G-protein coupled receptor G2 OS=Homo sapiens GN=ADGRG2 PE=1 S' | 649 | LSVTLVtYIAF | 0.103 | -0.172 | -0.921 | -0.33 |
| sp\|Q8IZP9\|AGRG2_HUMAN Adhesion G-protein coupled receptor G2 OS=Homo sapiens GN=ADGRG2 PE=1 S' | 662 | IRRDYPsKILI | 0.606 | 0.462 | 0.203 | 0.424 |
| sp\|Q8IZP9\|AGRG2_HUMAN Adhesion G-protein coupled receptor G2 OS=Homo sapiens GN=ADGRG2 PE=1 S' | 683 | LVFLLDsWIAL | 0.101 | 0.041 | -0.959 | -0.272 |
| sp\|Q8IZP9\|AGRG2_HUMAN Adhesion G-protein coupled receptor G2 OS=Homo sapiens GN=ADGRG2 PE=1 S' | 696 | MQGLCIsVAVF | 0.172 | -0.002 | -0.73 | -0.187 |
| sp\|Q8IZP9\|AGRG2_HUMAN Adhesion G-protein coupled receptor G2 OS=Homo sapiens GN=ADGRG2 PE=1 S' | 708 | HYFLLVsFTWM | 0.429 | 0.114 | -0.309 | 0.078 |
| sp\|Q8IZP9\|AGRG2_HUMAN Adhesion G-protein coupled receptor G2 OS=Homo sapiens GN=ADGRG2 PE=1 S' | 710 | FLLVSFtWMGL | 0.487 | 0.466 | 0.141 | 0.365 |
| sp\|Q8IZP9\|AGRG2_HUMAN Adhesion G-protein coupled receptor G2 OS=Homo sapiens GN=ADGRG2 PE=1 S' | 729 | LVKVFNtYIRK | 0.109 | -0.174 | -1.109 | -0.391 |
| sp\|Q8IZP9\|AGRG2_HUMAN Adhesion G-protein coupled receptor G2 OS=Homo sapiens GN=ADGRG2 PE=1 S' | 751 | VPAVVVtIILT | 0.1 | -0.22 | -0.953 | -0.358 |
| sp\|Q8IZP9\|AGRG2_HUMAN Adhesion G-protein coupled receptor G2 OS=Homo sapiens GN=ADGRG2 PE=1 S' | 755 | VVTIILtISPD | 0.052 | -0.231 | -1.085 | -0.421 |
| sp\|Q8IZP9\|AGRG2_HUMAN Adhesion G-protein coupled receptor G2 OS=Homo sapiens GN=ADGRG2 PE=1 S' | 757 | TIILTIsPDNY | 0.132 | -0.112 | -1.213 | -0.398 |
| sp\|Q8IZP9\|AGRG2_HUMAN Adhesion G-protein coupled receptor G2 OS=Homo sapiens GN=ADGRG2 PE=1 S' | 765 | DNYGLGsYGKF | 0.044 | -0.324 | -1.555 | -0.612 |
| sp\|Q8IZP9\|AGRG2_HUMAN Adhesion G-protein coupled receptor G2 OS=Homo sapiens GN=ADGRG2 PE=1 S' | 773 | GKFPNGsPDDF | 0.223 | -0.247 | -0.752 | -0.259 |
| sp\|Q8IZP9\|AGRG2_HUMAN Adhesion G-protein coupled receptor G2 OS=Homo sapiens GN=ADGRG2 PE=1 S' | 789 | NAVFYItVVGY | 0.281 | -0.035 | -0.693 | -0.149 |
| sp\|Q8IZP9\|AGRG2_HUMAN Adhesion G-protein coupled receptor G2 OS=Homo sapiens GN=ADGRG2 PE=1 S' | 803 | IFLLNVsMFIV | 0.525 | 0.108 | -0.324 | 0.103 |
| sp\|Q8IZP9\|AGRG2_HUMAN Adhesion G-protein coupled receptor G2 OS=Homo sapiens GN=ADGRG2 PE=1 S' | 827 | LGAQRKtSIQD | 0.435 | 0.121 | 0.259 | 0.272 |
| sp\|Q8IZP9\|AGRG2_HUMAN Adhesion G-protein coupled receptor G2 OS=Homo sapiens GN=ADGRG2 PE=1 S' | 828 | GAQRKTsIQDL | 0.696 | 0.916 | 0.504 | 0.705 |
| sp\|Q8IZP9\|AGRG2_HUMAN Adhesion G-protein coupled receptor G2 OS=Homo sapiens GN=ADGRG2 PE=1 S' | 834 | SIQDLRsIAGL | 0.069 | -0.12 | -1.466 | -0.506 |
| sp\|Q8IZP9\|AGRG2_HUMAN Adhesion G-protein coupled receptor G2 OS=Homo sapiens GN=ADGRG2 PE=1 S' | 839 | RSIAGLtFLLG | 0.096 | -0.112 | -1.139 | -0.385 |
| sp\|Q8IZP9\|AGRG2_HUMAN Adhesion G-protein coupled receptor G2 OS=Homo sapiens GN=ADGRG2 PE=1 S' | 845 | TFLLGItWGFA | 0.132 | -0.081 | -0.791 | -0.247 |
| sp\|Q8IZP9\|AGRG2_HUMAN Adhesion G-protein coupled receptor G2 OS=Homo sapiens GN=ADGRG2 PE=1 S' | 859 | WGPVNVtFMYL | 0.231 | -0.081 | -0.575 | -0.142 |
| sp\|Q8IZP9\|AGRG2_HUMAN Adhesion G-protein coupled receptor G2 OS=Homo sapiens GN=ADGRG2 PE=1 S' | 869 | LFAIFNtLQGF | 0.387 | 0.071 | -0.142 | 0.105 |
| sp\|Q8IZP9\|AGRG2_HUMAN Adhesion G-protein coupled receptor G2 OS=Homo sapiens GN=ADGRG2 PE=1 S' | 905 | LRLAENsDWSK | 0.299 | 0.145 | -0.808 | -0.121 |
| sp\|Q8IZP9\|AGRG2_HUMAN Adhesion G-protein coupled receptor G2 OS=Homo sapiens GN=ADGRG2 PE=1 S' | 908 | AENSDWsKTAT | 0.158 | -0.253 | -1.157 | -0.417 |
| sp\|Q8IZP9\|AGRG2_HUMAN Adhesion G-protein coupled receptor G2 OS=Homo sapiens GN=ADGRG2 PE=1 S' | 910 | NSDWSKtATNG | 0.578 | 0.205 | -0.164 | 0.206 |
| sp\|Q8IZP9\|AGRG2_HUMAN Adhesion G-protein coupled receptor G2 OS=Homo sapiens GN=ADGRG2 PE=1 S' | 912 | DWSKTAtNGLK | 0.386 | 0.057 | -0.684 | -0.08 |
| sp\|Q8IZP9\|AGRG2_HUMAN Adhesion G-protein coupled receptor G2 OS=Homo sapiens GN=ADGRG2 PE=1 S' | 919 | NGLKKQtVNQG | 0.337 | -0.007 | -0.233 | 0.032 |
| sp\|Q8IZP9\|AGRG2_HUMAN Adhesion G-protein coupled receptor G2 OS=Homo sapiens GN=ADGRG2 PE=1 S' | 925 | TVNQGVsSSSN | 0.057 | -0.266 | -1.463 | -0.557 |
| sp\|Q8IZP9\|AGRG2_HUMAN Adhesion G-protein coupled receptor G2 OS=Homo sapiens GN=ADGRG2 PE=1 S' | 926 | VNQGVSsSSNS | 0.043 | -0.274 | -1.692 | -0.641 |
| sp\|Q8IZP9\|AGRG2_HUMAN Adhesion G-protein coupled receptor G2 OS=Homo sapiens GN=ADGRG2 PE=1 S' | 927 | NQGVSSsSNSL | 0.07 | 0.032 | -0.885 | -0.261 |
| sp\|Q8IZP9\|AGRG2_HUMAN Adhesion G-protein coupled receptor G2 OS=Homo sapiens GN=ADGRG2 PE=1 S' | 928 | QGVSSSsNSLQ | 0.078 | -0.125 | -1.241 | -0.429 |
| sp\|Q8IZP9\|AGRG2_HUMAN Adhesion G-protein coupled receptor G2 OS=Homo sapiens GN=ADGRG2 PE=1 S' | 930 | VSSSSNsLQSS | 0.328 | 0.084 | -0.4 | 0.004 |
| sp\|Q8IZP9\|AGRG2_HUMAN Adhesion G-protein coupled receptor G2 OS=Homo sapiens GN=ADGRG2 PE=1 S' | 933 | SSNSLQsSSNS | 0.032 | -0.36 | -2.109 | -0.812 |
| sp\|Q8IZP9\|AGRG2_HUMAN Adhesion G-protein coupled receptor G2 OS=Homo sapiens GN=ADGRG2 PE=1 S' | 934 | SNSLQSsSNST | 0.024 | -0.41 | -1.901 | -0.762 |
| sp\|Q8IZP9\|AGRG2_HUMAN Adhesion G-protein coupled receptor G2 OS=Homo sapiens GN=ADGRG2 PE=1 S' | 935 | NSLQSSsNSTN | 0.168 | -0.053 | -0.814 | -0.233 |
| sp\|Q8IZP9\|AGRG2_HUMAN Adhesion G-protein coupled receptor G2 OS=Homo sapiens GN=ADGRG2 PE=1 S' | 937 | LQSSSNsTNST | 0.181 | 0.032 | -0.524 | -0.104 |
| sp\|Q8IZP9\|AGRG2_HUMAN Adhesion G-protein coupled receptor G2 OS=Homo sapiens GN=ADGRG2 PE=1 S' | 938 | QSSSNStNSTT | 0.037 | -0.464 | -1.637 | -0.688 |
| sp\|Q8IZP9\|AGRG2_HUMAN Adhesion G-protein coupled receptor G2 OS=Homo sapiens GN=ADGRG2 PE=1 S' | 940 | SSNSTNsTTLL | 0.111 | -0.129 | -1.387 | -0.468 |
| sp\|Q8IZP9\|AGRG2_HUMAN Adhesion G-protein coupled receptor G2 OS=Homo sapiens GN=ADGRG2 PE=1 S' | 941 | SNSTNStTLLV | 0.047 | -0.339 | -1.78 | -0.691 |

| | | | | | | |
|---|---|---|---|---|---|---|
| sp\|Q8IZP9\|AGRG2_HUMAN Adhesion G-protein coupled receptor G2 OS=Homo sapiens GN=ADGRG2 PE=1 S' | 942 | NSTNSTtLLVN | 0.395 | 0.246 | -0.005 | 0.212 |
| sp\|Q8IZP9\|AGRG2_HUMAN Adhesion G-protein coupled receptor G2 OS=Homo sapiens GN=ADGRG2 PE=1 S' | 950 | LVNNDCsVHAS | 0.163 | -0.004 | -0.848 | -0.23 |
| sp\|Q8IZP9\|AGRG2_HUMAN Adhesion G-protein coupled receptor G2 OS=Homo sapiens GN=ADGRG2 PE=1 S' | 954 | DCSVHAsGNGN | 0.326 | -0.017 | -0.782 | -0.158 |
| sp\|Q8IZP9\|AGRG2_HUMAN Adhesion G-protein coupled receptor G2 OS=Homo sapiens GN=ADGRG2 PE=1 S' | 960 | SGNGNAsTERN | 0.119 | -0.186 | -1.281 | -0.449 |
| sp\|Q8IZP9\|AGRG2_HUMAN Adhesion G-protein coupled receptor G2 OS=Homo sapiens GN=ADGRG2 PE=1 S' | 961 | GNGNAStERNG | 0.165 | -0.04 | -0.845 | -0.24 |
| sp\|Q8IZP9\|AGRG2_HUMAN Adhesion G-protein coupled receptor G2 OS=Homo sapiens GN=ADGRG2 PE=1 S' | 967 | TERNGVsFSVQ | 0.246 | 0.082 | -0.664 | -0.112 |
| sp\|Q8IZP9\|AGRG2_HUMAN Adhesion G-protein coupled receptor G2 OS=Homo sapiens GN=ADGRG2 PE=1 S' | 969 | RNGVSFsVQNG | 0.253 | 0.21 | -0.502 | -0.013 |
| sp\|Q8IZP9\|AGRG2_HUMAN Adhesion G-protein coupled receptor G2 OS=Homo sapiens GN=ADGRG2 PE=1 S' | 981 | VCLHDFtGKQH | 0.115 | -0.265 | -1.114 | -0.421 |
| sp\|Q8IZP9\|AGRG2_HUMAN Adhesion G-protein coupled receptor G2 OS=Homo sapiens GN=ADGRG2 PE=1 S' | 993 | FNEKEDsCNGK | 0.329 | -0.106 | -0.664 | -0.147 |
| sp\|Q8IZP9\|AGRG2_HUMAN Adhesion G-protein coupled receptor G2 OS=Homo sapiens GN=ADGRG2 PE=1 S' | 1005 | RMALRRtSKRG | 0.187 | 0.114 | -1.002 | -0.234 |
| sp\|Q8IZP9\|AGRG2_HUMAN Adhesion G-protein coupled receptor G2 OS=Homo sapiens GN=ADGRG2 PE=1 S' | 1006 | MALRRTsKRGS | 0.78 | 0.898 | 0.735 | 0.804 |
| sp\|Q8IZP9\|AGRG2_HUMAN Adhesion G-protein coupled receptor G2 OS=Homo sapiens GN=ADGRG2 PE=1 S' | 1010 | RTSKRGsLHFI | 0.667 | 0.186 | -0.07 | 0.261 |
| sp\|O60242\|AGRB3_HUMAN Adhesion G protein-coupled receptor B3 OS=Homo sapiens GN=ADGRB3 PE=1 S' | 315 | QGSQVRtRTCV | 0.152 | -0.174 | -1.33 | -0.451 |
| sp\|O60242\|AGRB3_HUMAN Adhesion G protein-coupled receptor B3 OS=Homo sapiens GN=ADGRB3 PE=1 S' | 317 | SQVRTRtCVSP | 0.108 | 0.743 | -0.747 | 0.035 |
| sp\|O60242\|AGRB3_HUMAN Adhesion G protein-coupled receptor B3 OS=Homo sapiens GN=ADGRB3 PE=1 S' | 320 | RTRTCVsPYGT | 0.434 | -0.128 | -0.551 | -0.082 |
| sp\|O60242\|AGRB3_HUMAN Adhesion G protein-coupled receptor B3 OS=Homo sapiens GN=ADGRB3 PE=1 S' | 324 | CVSPYGtHCSG | 0.074 | -0.276 | -1.265 | -0.489 |
| sp\|O60242\|AGRB3_HUMAN Adhesion G protein-coupled receptor B3 OS=Homo sapiens GN=ADGRB3 PE=1 S' | 327 | PYGTHCsGPLR | 0.69 | 0.532 | 0.015 | 0.412 |
| sp\|O60242\|AGRB3_HUMAN Adhesion G protein-coupled receptor B3 OS=Homo sapiens GN=ADGRB3 PE=1 S' | 333 | SGPLREsRVCN | 0.098 | -0.093 | -1.489 | -0.495 |
| sp\|O60242\|AGRB3_HUMAN Adhesion G protein-coupled receptor B3 OS=Homo sapiens GN=ADGRB3 PE=1 S' | 339 | SRVCNNtALCP | 0.172 | 0.163 | -1.221 | -0.295 |
| sp\|O60242\|AGRB3_HUMAN Adhesion G protein-coupled receptor B3 OS=Homo sapiens GN=ADGRB3 PE=1 S' | 352 | GVWEEWsPWSL | 0.049 | -0.226 | -1.612 | -0.596 |
| sp\|O60242\|AGRB3_HUMAN Adhesion G protein-coupled receptor B3 OS=Homo sapiens GN=ADGRB3 PE=1 S' | 355 | EEWSPWsLCSF | 0.054 | -0.212 | -1.238 | -0.465 |
| sp\|O60242\|AGRB3_HUMAN Adhesion G protein-coupled receptor B3 OS=Homo sapiens GN=ADGRB3 PE=1 S' | 358 | SPWSLCsFTCG | 0.156 | 0.001 | -1.284 | -0.376 |
| sp\|O60242\|AGRB3_HUMAN Adhesion G protein-coupled receptor B3 OS=Homo sapiens GN=ADGRB3 PE=1 S' | 360 | WSLCSFtCGRG | 0.554 | 0.186 | -0.119 | 0.207 |
| sp\|O60242\|AGRB3_HUMAN Adhesion G protein-coupled receptor B3 OS=Homo sapiens GN=ADGRB3 PE=1 S' | 367 | CGRGQRtRTRS | 0.195 | -0.005 | -1.067 | -0.292 |
| sp\|O60242\|AGRB3_HUMAN Adhesion G protein-coupled receptor B3 OS=Homo sapiens GN=ADGRB3 PE=1 S' | 369 | RGQRTRtRSCT | 0.282 | 0.784 | -0.352 | 0.238 |
| sp\|O60242\|AGRB3_HUMAN Adhesion G protein-coupled receptor B3 OS=Homo sapiens GN=ADGRB3 PE=1 S' | 371 | QRTRTRsCTPP | 0.418 | 1.015 | -0.017 | 0.472 |
| sp\|O60242\|AGRB3_HUMAN Adhesion G protein-coupled receptor B3 OS=Homo sapiens GN=ADGRB3 PE=1 S' | 373 | TRTRSCtPPQY | 0.937 | 1.507 | 1.404 | 1.283 |
| sp\|O60242\|AGRB3_HUMAN Adhesion G protein-coupled receptor B3 OS=Homo sapiens GN=ADGRB3 PE=1 S' | 387 | PCEGPEtHHKP | 0.151 | -0.178 | -1.115 | -0.381 |
| sp\|O60242\|AGRB3_HUMAN Adhesion G protein-coupled receptor B3 OS=Homo sapiens GN=ADGRB3 PE=1 S' | 407 | GQWQEWsSWSQ | 0.133 | -0.057 | -1.108 | -0.344 |
| sp\|O60242\|AGRB3_HUMAN Adhesion G protein-coupled receptor B3 OS=Homo sapiens GN=ADGRB3 PE=1 S' | 408 | QWQEWSsWSQC | 0.058 | -0.093 | -1.43 | -0.488 |
| sp\|O60242\|AGRB3_HUMAN Adhesion G protein-coupled receptor B3 OS=Homo sapiens GN=ADGRB3 PE=1 S' | 410 | QEWSSWsQCSV | 0.093 | -0.039 | -1.479 | -0.475 |
| sp\|O60242\|AGRB3_HUMAN Adhesion G protein-coupled receptor B3 OS=Homo sapiens GN=ADGRB3 PE=1 S' | 413 | SSWSQCsVTCS | 0.08 | -0.078 | -1.588 | -0.529 |
| sp\|O60242\|AGRB3_HUMAN Adhesion G protein-coupled receptor B3 OS=Homo sapiens GN=ADGRB3 PE=1 S' | 415 | WSQCSVtCSNG | 0.491 | 0.213 | -0.351 | 0.118 |
| sp\|O60242\|AGRB3_HUMAN Adhesion G protein-coupled receptor B3 OS=Homo sapiens GN=ADGRB3 PE=1 S' | 417 | QCSVTCsNGTQ | 0.15 | -0.085 | -1.32 | -0.418 |
| sp\|O60242\|AGRB3_HUMAN Adhesion G protein-coupled receptor B3 OS=Homo sapiens GN=ADGRB3 PE=1 S' | 420 | VTCSNGtQQRS | 0.103 | -0.373 | -1.641 | -0.637 |
| sp\|O60242\|AGRB3_HUMAN Adhesion G protein-coupled receptor B3 OS=Homo sapiens GN=ADGRB3 PE=1 S' | 424 | NGTQQRsRQCT | 0.098 | -0.143 | -1.115 | -0.387 |
| sp\|O60242\|AGRB3_HUMAN Adhesion G protein-coupled receptor B3 OS=Homo sapiens GN=ADGRB3 PE=1 S' | 428 | QRSRQCtAAAH | 0.532 | 0.957 | -0.141 | 0.449 |
| sp\|O60242\|AGRB3_HUMAN Adhesion G protein-coupled receptor B3 OS=Homo sapiens GN=ADGRB3 PE=1 S' | 435 | AAAHGGsECRG | 0.068 | -0.385 | -1.553 | -0.623 |
| sp\|O60242\|AGRB3_HUMAN Adhesion G protein-coupled receptor B3 OS=Homo sapiens GN=ADGRB3 PE=1 S' | 444 | RGPWAEsRECY | 0.228 | -0.083 | -1.006 | -0.287 |
| sp\|O60242\|AGRB3_HUMAN Adhesion G protein-coupled receptor B3 OS=Homo sapiens GN=ADGRB3 PE=1 S' | 453 | CYNPECtANGQ | 0.33 | -0.07 | -0.894 | -0.211 |
| sp\|O60242\|AGRB3_HUMAN Adhesion G protein-coupled receptor B3 OS=Homo sapiens GN=ADGRB3 PE=1 S' | 465 | NQWGHWsGCSK | 0.058 | -0.171 | -1.172 | -0.428 |
| sp\|O60242\|AGRB3_HUMAN Adhesion G protein-coupled receptor B3 OS=Homo sapiens GN=ADGRB3 PE=1 S' | 468 | GHWSGCsKSCD | 0.114 | 0.001 | -1.384 | -0.423 |
| sp\|O60242\|AGRB3_HUMAN Adhesion G protein-coupled receptor B3 OS=Homo sapiens GN=ADGRB3 PE=1 S' | 470 | WSGCSKsCDGG | 0.514 | 0.207 | 0.079 | 0.267 |
| sp\|O60242\|AGRB3_HUMAN Adhesion G protein-coupled receptor B3 OS=Homo sapiens GN=ADGRB3 PE=1 S' | 481 | WERRIRtCQGA | 0.751 | 1.047 | 0.68 | 0.826 |
| sp\|O60242\|AGRB3_HUMAN Adhesion G protein-coupled receptor B3 OS=Homo sapiens GN=ADGRB3 PE=1 S' | 488 | CQGAVItGQQC | 0.139 | -0.221 | -0.891 | -0.324 |
| sp\|O60242\|AGRB3_HUMAN Adhesion G protein-coupled receptor B3 OS=Homo sapiens GN=ADGRB3 PE=1 S' | 495 | GQQCEGtGEEV | 0.162 | -0.126 | -1.335 | -0.433 |
| sp\|O60242\|AGRB3_HUMAN Adhesion G protein-coupled receptor B3 OS=Homo sapiens GN=ADGRB3 PE=1 S' | 521 | PEDYLMsMVWK | 0.183 | 0.125 | -0.863 | -0.185 |
| sp\|O60242\|AGRB3_HUMAN Adhesion G protein-coupled receptor B3 OS=Homo sapiens GN=ADGRB3 PE=1 S' | 527 | SMVWKRtPAGD | 0.035 | -0.455 | -1.787 | -0.736 |
| sp\|O60242\|AGRB3_HUMAN Adhesion G protein-coupled receptor B3 OS=Homo sapiens GN=ADGRB3 PE=1 S' | 542 | QCPLNAtGTTS | 0.173 | -0.155 | -1.129 | -0.37 |

| | | | | | | |
|---|---|---|---|---|---|---|
| sp\|O60242\|AGRB3_HUMAN Adhesion G protein-coupled receptor B3 OS=Homo sapiens GN=ADGRB3 PE=1 S' | 544 | PLNATGtTSRR | 0.112 | -0.164 | -1.295 | -0.449 |
| sp\|O60242\|AGRB3_HUMAN Adhesion G protein-coupled receptor B3 OS=Homo sapiens GN=ADGRB3 PE=1 S' | 545 | LNATGTtSRRC | 0.054 | -0.472 | -1.545 | -0.654 |
| sp\|O60242\|AGRB3_HUMAN Adhesion G protein-coupled receptor B3 OS=Homo sapiens GN=ADGRB3 PE=1 S' | 546 | NATGTTsRRCS | 0.081 | -0.17 | -1.147 | -0.412 |
| sp\|O60242\|AGRB3_HUMAN Adhesion G protein-coupled receptor B3 OS=Homo sapiens GN=ADGRB3 PE=1 S' | 550 | TTSRRCsLSLH | 0.762 | 0.862 | 0.247 | 0.624 |
| sp\|O60242\|AGRB3_HUMAN Adhesion G protein-coupled receptor B3 OS=Homo sapiens GN=ADGRB3 PE=1 S' | 552 | SRRCSLsLHGV | 0.732 | 0.625 | 0.124 | 0.494 |
| sp\|O60242\|AGRB3_HUMAN Adhesion G protein-coupled receptor B3 OS=Homo sapiens GN=ADGRB3 PE=1 S' | 563 | AFWEQPsFARC | 0.072 | -0.165 | -1.52 | -0.538 |
| sp\|O60242\|AGRB3_HUMAN Adhesion G protein-coupled receptor B3 OS=Homo sapiens GN=ADGRB3 PE=1 S' | 569 | SFARCIsNEYR | 0.641 | 0.757 | 0.2 | 0.533 |
| sp\|O60242\|AGRB3_HUMAN Adhesion G protein-coupled receptor B3 OS=Homo sapiens GN=ADGRB3 PE=1 S' | 578 | YRHLQHsIKEH | 0.1 | 0.107 | -1.174 | -0.322 |
| sp\|O60242\|AGRB3_HUMAN Adhesion G protein-coupled receptor B3 OS=Homo sapiens GN=ADGRB3 PE=1 S' | 596 | LAGDGMsQVTK | 0.08 | -0.172 | -1.411 | -0.501 |
| sp\|O60242\|AGRB3_HUMAN Adhesion G protein-coupled receptor B3 OS=Homo sapiens GN=ADGRB3 PE=1 S' | 599 | DGMSQVtKTLL | 0.253 | -0.054 | -0.78 | -0.194 |
| sp\|O60242\|AGRB3_HUMAN Adhesion G protein-coupled receptor B3 OS=Homo sapiens GN=ADGRB3 PE=1 S' | 601 | MSQVTKtLLDL | 0.4 | 0.284 | -0.083 | 0.2 |
| sp\|O60242\|AGRB3_HUMAN Adhesion G protein-coupled receptor B3 OS=Homo sapiens GN=ADGRB3 PE=1 S' | 606 | KTLLDLtQRKN | 0.076 | -0.303 | -1.627 | -0.618 |
| sp\|O60242\|AGRB3_HUMAN Adhesion G protein-coupled receptor B3 OS=Homo sapiens GN=ADGRB3 PE=1 S' | 619 | AGDLLMsVEIL | 0.178 | 0.005 | -0.932 | -0.25 |
| sp\|O60242\|AGRB3_HUMAN Adhesion G protein-coupled receptor B3 OS=Homo sapiens GN=ADGRB3 PE=1 S' | 627 | EILRNVtDTFK | 0.759 | 0.813 | 0.464 | 0.679 |
| sp\|O60242\|AGRB3_HUMAN Adhesion G protein-coupled receptor B3 OS=Homo sapiens GN=ADGRB3 PE=1 S' | 629 | LRNVTDtFKRA | 0.12 | 0.038 | -1.054 | -0.299 |
| sp\|O60242\|AGRB3_HUMAN Adhesion G protein-coupled receptor B3 OS=Homo sapiens GN=ADGRB3 PE=1 S' | 634 | DTFKRAsYIPA | 0.474 | 0.063 | -0.423 | 0.038 |
| sp\|O60242\|AGRB3_HUMAN Adhesion G protein-coupled receptor B3 OS=Homo sapiens GN=ADGRB3 PE=1 S' | 639 | ASYIPAsDGVQ | 0.187 | -0.109 | -0.994 | -0.305 |
| sp\|O60242\|AGRB3_HUMAN Adhesion G protein-coupled receptor B3 OS=Homo sapiens GN=ADGRB3 PE=1 S' | 650 | NFFQIVsNLLD | 0.35 | 0 | -0.364 | -0.005 |
| sp\|O60242\|AGRB3_HUMAN Adhesion G protein-coupled receptor B3 OS=Homo sapiens GN=ADGRB3 PE=1 S' | 671 | QQIYPGsIELM | 0.055 | -0.232 | -1.072 | -0.416 |
| sp\|O60242\|AGRB3_HUMAN Adhesion G protein-coupled receptor B3 OS=Homo sapiens GN=ADGRB3 PE=1 S' | 695 | MMDFQNsYLMT | 0.09 | -0.007 | -1.398 | -0.438 |
| sp\|O60242\|AGRB3_HUMAN Adhesion G protein-coupled receptor B3 OS=Homo sapiens GN=ADGRB3 PE=1 S' | 699 | QNSYLMtGNVV | 0.147 | -0.043 | -0.875 | -0.257 |
| sp\|O60242\|AGRB3_HUMAN Adhesion G protein-coupled receptor B3 OS=Homo sapiens GN=ADGRB3 PE=1 S' | 705 | TGNVVAsIQKL | 0.101 | -0.118 | -1.118 | -0.378 |
| sp\|O60242\|AGRB3_HUMAN Adhesion G protein-coupled receptor B3 OS=Homo sapiens GN=ADGRB3 PE=1 S' | 713 | QKLPAAsVLTD | 0.397 | 0.066 | -0.286 | 0.059 |
| sp\|O60242\|AGRB3_HUMAN Adhesion G protein-coupled receptor B3 OS=Homo sapiens GN=ADGRB3 PE=1 S' | 716 | PAASVLtDINF | 0.101 | -0.122 | -1.192 | -0.404 |
| sp\|O60242\|AGRB3_HUMAN Adhesion G protein-coupled receptor B3 OS=Homo sapiens GN=ADGRB3 PE=1 S' | 735 | VDWARNsEDRV | 0.359 | 0.164 | -0.782 | -0.086 |
| sp\|O60242\|AGRB3_HUMAN Adhesion G protein-coupled receptor B3 OS=Homo sapiens GN=ADGRB3 PE=1 S' | 744 | RVVIPKsIFTP | 0.111 | -0.122 | -0.785 | -0.265 |
| sp\|O60242\|AGRB3_HUMAN Adhesion G protein-coupled receptor B3 OS=Homo sapiens GN=ADGRB3 PE=1 S' | 747 | IPKSIFtPVSS | 0.047 | -0.427 | -1.301 | -0.56 |
| sp\|O60242\|AGRB3_HUMAN Adhesion G protein-coupled receptor B3 OS=Homo sapiens GN=ADGRB3 PE=1 S' | 750 | SIFTPVsSKEL | 0.188 | -0.098 | -0.907 | -0.272 |
| sp\|O60242\|AGRB3_HUMAN Adhesion G protein-coupled receptor B3 OS=Homo sapiens GN=ADGRB3 PE=1 S' | 751 | IFTPVSsKELD | 0.195 | -0.111 | -0.563 | -0.16 |
| sp\|O60242\|AGRB3_HUMAN Adhesion G protein-coupled receptor B3 OS=Homo sapiens GN=ADGRB3 PE=1 S' | 757 | SKELDEsSVFV | 0.094 | -0.238 | -1.658 | -0.601 |
| sp\|O60242\|AGRB3_HUMAN Adhesion G protein-coupled receptor B3 OS=Homo sapiens GN=ADGRB3 PE=1 S' | 758 | KELDESsVFVL | 0.115 | -0.101 | -1.257 | -0.414 |
| sp\|O60242\|AGRB3_HUMAN Adhesion G protein-coupled receptor B3 OS=Homo sapiens GN=ADGRB3 PE=1 S' | 776 | LDLILPtLRNY | 0.177 | -0.12 | -0.911 | -0.285 |
| sp\|O60242\|AGRB3_HUMAN Adhesion G protein-coupled receptor B3 OS=Homo sapiens GN=ADGRB3 PE=1 S' | 781 | PTLRNYtVINS | 0.647 | 0.952 | 0.505 | 0.701 |
| sp\|O60242\|AGRB3_HUMAN Adhesion G protein-coupled receptor B3 OS=Homo sapiens GN=ADGRB3 PE=1 S' | 785 | NYTVINsKIIV | 0.258 | 0.037 | -0.636 | -0.114 |
| sp\|O60242\|AGRB3_HUMAN Adhesion G protein-coupled receptor B3 OS=Homo sapiens GN=ADGRB3 PE=1 S' | 791 | SKIIVVtIRPE | 0.225 | -0.175 | -0.893 | -0.281 |
| sp\|O60242\|AGRB3_HUMAN Adhesion G protein-coupled receptor B3 OS=Homo sapiens GN=ADGRB3 PE=1 S' | 798 | IRPEPKtTDSF | 0.09 | 0.03 | -0.793 | -0.224 |
| sp\|O60242\|AGRB3_HUMAN Adhesion G protein-coupled receptor B3 OS=Homo sapiens GN=ADGRB3 PE=1 S' | 799 | RPEPKTtDSFL | 0.075 | -0.22 | -1.345 | -0.497 |
| sp\|O60242\|AGRB3_HUMAN Adhesion G protein-coupled receptor B3 OS=Homo sapiens GN=ADGRB3 PE=1 S' | 801 | EPKTTDsFLEI | 0.202 | -0.004 | -0.755 | -0.186 |
| sp\|O60242\|AGRB3_HUMAN Adhesion G protein-coupled receptor B3 OS=Homo sapiens GN=ADGRB3 PE=1 S' | 814 | AHLANGtLNPY | 0.299 | -0.095 | -0.727 | -0.174 |
| sp\|O60242\|AGRB3_HUMAN Adhesion G protein-coupled receptor B3 OS=Homo sapiens GN=ADGRB3 PE=1 S' | 825 | CVLWDDsKTNE | 0.233 | -0.161 | -0.861 | -0.263 |
| sp\|O60242\|AGRB3_HUMAN Adhesion G protein-coupled receptor B3 OS=Homo sapiens GN=ADGRB3 PE=1 S' | 827 | LWDDSKtNESL | 0.224 | 0.195 | -0.57 | -0.05 |
| sp\|O60242\|AGRB3_HUMAN Adhesion G protein-coupled receptor B3 OS=Homo sapiens GN=ADGRB3 PE=1 S' | 830 | DSKTNEsLGTW | 0.476 | -0.009 | -0.384 | 0.028 |
| sp\|O60242\|AGRB3_HUMAN Adhesion G protein-coupled receptor B3 OS=Homo sapiens GN=ADGRB3 PE=1 S' | 833 | TNESLGtWSTQ | 0.031 | -0.416 | -1.825 | -0.737 |
| sp\|O60242\|AGRB3_HUMAN Adhesion G protein-coupled receptor B3 OS=Homo sapiens GN=ADGRB3 PE=1 S' | 835 | ESLGTWsTQGC | 0.076 | -0.251 | -1.149 | -0.441 |
| sp\|O60242\|AGRB3_HUMAN Adhesion G protein-coupled receptor B3 OS=Homo sapiens GN=ADGRB3 PE=1 S' | 836 | SLGTWStQGCK | 0.133 | -0.076 | -1.23 | -0.391 |
| sp\|O60242\|AGRB3_HUMAN Adhesion G protein-coupled receptor B3 OS=Homo sapiens GN=ADGRB3 PE=1 S' | 841 | STQGCKtVLTD | 0.295 | 0.014 | -0.576 | -0.089 |
| sp\|O60242\|AGRB3_HUMAN Adhesion G protein-coupled receptor B3 OS=Homo sapiens GN=ADGRB3 PE=1 S' | 844 | GCKTVLtDASH | 0.135 | -0.17 | -1.5 | -0.512 |
| sp\|O60242\|AGRB3_HUMAN Adhesion G protein-coupled receptor B3 OS=Homo sapiens GN=ADGRB3 PE=1 S' | 847 | TVLTDAsHTKC | 0.152 | -0.199 | -1.271 | -0.439 |
| sp\|O60242\|AGRB3_HUMAN Adhesion G protein-coupled receptor B3 OS=Homo sapiens GN=ADGRB3 PE=1 S' | 849 | LTDASHtKCLC | 0.275 | 0.058 | -0.685 | -0.117 |

| | | | | | | |
|---|---|---|---|---|---|---|
| sp\|O60242\|AGRB3_HUMAN Adhesion G protein-coupled receptor B3 OS=Homo sapiens GN=ADGRB3 PE=1 S' | 857 | CLCDRLsTFAI | 0.64 | 0.349 | 0.155 | 0.381 |
| sp\|O60242\|AGRB3_HUMAN Adhesion G protein-coupled receptor B3 OS=Homo sapiens GN=ADGRB3 PE=1 S' | 858 | LCDRLStFAIL | 0.508 | 0.791 | -0.006 | 0.431 |
| sp\|O60242\|AGRB3_HUMAN Adhesion G protein-coupled receptor B3 OS=Homo sapiens GN=ADGRB3 PE=1 S' | 873 | REIIMEsSGTP | 0.124 | -0.171 | -1.328 | -0.458 |
| sp\|O60242\|AGRB3_HUMAN Adhesion G protein-coupled receptor B3 OS=Homo sapiens GN=ADGRB3 PE=1 S' | 874 | EIIMESsGTPS | 0.067 | -0.257 | -1.428 | -0.539 |
| sp\|O60242\|AGRB3_HUMAN Adhesion G protein-coupled receptor B3 OS=Homo sapiens GN=ADGRB3 PE=1 S' | 876 | IMESSGtPSVT | 0.062 | -0.251 | -1.263 | -0.484 |
| sp\|O60242\|AGRB3_HUMAN Adhesion G protein-coupled receptor B3 OS=Homo sapiens GN=ADGRB3 PE=1 S' | 878 | ESSGTPsVTLI | 0.131 | -0.104 | -0.793 | -0.255 |
| sp\|O60242\|AGRB3_HUMAN Adhesion G protein-coupled receptor B3 OS=Homo sapiens GN=ADGRB3 PE=1 S' | 880 | SGTPSVtLIVG | 0.498 | 0.2 | 0.136 | 0.278 |
| sp\|O60242\|AGRB3_HUMAN Adhesion G protein-coupled receptor B3 OS=Homo sapiens GN=ADGRB3 PE=1 S' | 885 | VTLIVGsGLSC | 0.037 | -0.379 | -1.935 | -0.759 |
| sp\|O60242\|AGRB3_HUMAN Adhesion G protein-coupled receptor B3 OS=Homo sapiens GN=ADGRB3 PE=1 S' | 888 | IVGSGLsCLAL | 0.056 | -0.072 | -1.215 | -0.41 |
| sp\|O60242\|AGRB3_HUMAN Adhesion G protein-coupled receptor B3 OS=Homo sapiens GN=ADGRB3 PE=1 S' | 894 | SCLALItLAVV | 0.227 | -0.061 | -1.078 | -0.304 |
| sp\|O60242\|AGRB3_HUMAN Adhesion G protein-coupled receptor B3 OS=Homo sapiens GN=ADGRB3 PE=1 S' | 908 | LWRYIRsERSI | 0.34 | 0.292 | -0.126 | 0.169 |
| sp\|O60242\|AGRB3_HUMAN Adhesion G protein-coupled receptor B3 OS=Homo sapiens GN=ADGRB3 PE=1 S' | 911 | YIRSERsIILI | 0.255 | 0.213 | -0.501 | -0.011 |
| sp\|O60242\|AGRB3_HUMAN Adhesion G protein-coupled receptor B3 OS=Homo sapiens GN=ADGRB3 PE=1 S' | 920 | LINFCLsIISS | 0.072 | -0.093 | -1.108 | -0.376 |
| sp\|O60242\|AGRB3_HUMAN Adhesion G protein-coupled receptor B3 OS=Homo sapiens GN=ADGRB3 PE=1 S' | 923 | FCLSIIsSNIL | 0.335 | 0.059 | -0.447 | -0.018 |
| sp\|O60242\|AGRB3_HUMAN Adhesion G protein-coupled receptor B3 OS=Homo sapiens GN=ADGRB3 PE=1 S' | 924 | CLSIISsNILI | 0.21 | 0.044 | -0.439 | -0.062 |
| sp\|O60242\|AGRB3_HUMAN Adhesion G protein-coupled receptor B3 OS=Homo sapiens GN=ADGRB3 PE=1 S' | 933 | LILVGQtQTHN | 0.073 | -0.16 | -1.249 | -0.445 |
| sp\|O60242\|AGRB3_HUMAN Adhesion G protein-coupled receptor B3 OS=Homo sapiens GN=ADGRB3 PE=1 S' | 935 | LVGQTQtHNKS | 0.099 | -0.028 | -0.812 | -0.247 |
| sp\|O60242\|AGRB3_HUMAN Adhesion G protein-coupled receptor B3 OS=Homo sapiens GN=ADGRB3 PE=1 S' | 939 | TQTHNKsICTT | 0.115 | -0.254 | -0.648 | -0.262 |
| sp\|O60242\|AGRB3_HUMAN Adhesion G protein-coupled receptor B3 OS=Homo sapiens GN=ADGRB3 PE=1 S' | 942 | HNKSICtTTTA | 0.24 | -0.149 | -0.646 | -0.185 |
| sp\|O60242\|AGRB3_HUMAN Adhesion G protein-coupled receptor B3 OS=Homo sapiens GN=ADGRB3 PE=1 S' | 943 | NKSICTtTTAF | 0.413 | 0.075 | -0.265 | 0.074 |
| sp\|O60242\|AGRB3_HUMAN Adhesion G protein-coupled receptor B3 OS=Homo sapiens GN=ADGRB3 PE=1 S' | 944 | KSICTTtTAFL | 0.074 | -0.193 | -1.374 | -0.498 |
| sp\|O60242\|AGRB3_HUMAN Adhesion G protein-coupled receptor B3 OS=Homo sapiens GN=ADGRB3 PE=1 S' | 945 | SICTTTtAFLH | 0.106 | -0.053 | -1.354 | -0.434 |
| sp\|O60242\|AGRB3_HUMAN Adhesion G protein-coupled receptor B3 OS=Homo sapiens GN=ADGRB3 PE=1 S' | 955 | HFFFLAsFCWV | 0.281 | 0.082 | -0.668 | -0.102 |
| sp\|O60242\|AGRB3_HUMAN Adhesion G protein-coupled receptor B3 OS=Homo sapiens GN=ADGRB3 PE=1 S' | 961 | SFCWVLtEAWQ | 0.129 | -0.148 | -1.055 | -0.358 |
| sp\|O60242\|AGRB3_HUMAN Adhesion G protein-coupled receptor B3 OS=Homo sapiens GN=ADGRB3 PE=1 S' | 966 | LTEAWQsYMAV | 0.253 | -0.029 | -0.717 | -0.164 |
| sp\|O60242\|AGRB3_HUMAN Adhesion G protein-coupled receptor B3 OS=Homo sapiens GN=ADGRB3 PE=1 S' | 971 | QSYMAVtGKIR | 0.279 | -0.15 | -0.511 | -0.127 |
| sp\|O60242\|AGRB3_HUMAN Adhesion G protein-coupled receptor B3 OS=Homo sapiens GN=ADGRB3 PE=1 S' | 976 | VTGKIRtRLIR | 0.137 | -0.187 | -1.186 | -0.412 |
| sp\|O60242\|AGRB3_HUMAN Adhesion G protein-coupled receptor B3 OS=Homo sapiens GN=ADGRB3 PE=1 S' | 997 | PALVVAtSVGF | 0.116 | -0.17 | -1.115 | -0.39 |
| sp\|O60242\|AGRB3_HUMAN Adhesion G protein-coupled receptor B3 OS=Homo sapiens GN=ADGRB3 PE=1 S' | 998 | ALVVATsVGFT | 0.225 | 0.013 | -0.656 | -0.139 |
| sp\|O60242\|AGRB3_HUMAN Adhesion G protein-coupled receptor B3 OS=Homo sapiens GN=ADGRB3 PE=1 S' | 1002 | ATSVGFtRTKG | 0.074 | -0.304 | -1.531 | -0.587 |
| sp\|O60242\|AGRB3_HUMAN Adhesion G protein-coupled receptor B3 OS=Homo sapiens GN=ADGRB3 PE=1 S' | 1004 | SVGFTRtKGYG | 0.083 | -0.062 | -1.33 | -0.436 |
| sp\|O60242\|AGRB3_HUMAN Adhesion G protein-coupled receptor B3 OS=Homo sapiens GN=ADGRB3 PE=1 S' | 1009 | RTKGYGtDHYC | 0.221 | -0.105 | -1.185 | -0.356 |
| sp\|O60242\|AGRB3_HUMAN Adhesion G protein-coupled receptor B3 OS=Homo sapiens GN=ADGRB3 PE=1 S' | 1016 | DHYCWLsLEGG | 0.273 | -0.024 | -0.854 | -0.202 |
| sp\|O60242\|AGRB3_HUMAN Adhesion G protein-coupled receptor B3 OS=Homo sapiens GN=ADGRB3 PE=1 S' | 1049 | VFNKLVsRDGI | 0.447 | -0.073 | -0.322 | 0.017 |
| sp\|O60242\|AGRB3_HUMAN Adhesion G protein-coupled receptor B3 OS=Homo sapiens GN=ADGRB3 PE=1 S' | 1066 | HRAGQMsEPHS | 0.521 | 0.856 | 0.373 | 0.583 |
| sp\|O60242\|AGRB3_HUMAN Adhesion G protein-coupled receptor B3 OS=Homo sapiens GN=ADGRB3 PE=1 S' | 1070 | QMSEPHsGLTL | 0.062 | -0.028 | -1.19 | -0.385 |
| sp\|O60242\|AGRB3_HUMAN Adhesion G protein-coupled receptor B3 OS=Homo sapiens GN=ADGRB3 PE=1 S' | 1073 | EPHSGLtLKCA | 0.053 | -0.207 | -1.248 | -0.467 |
| sp\|O60242\|AGRB3_HUMAN Adhesion G protein-coupled receptor B3 OS=Homo sapiens GN=ADGRB3 PE=1 S' | 1083 | AKCGVVsTTAL | 0.564 | 0.095 | -0.238 | 0.14 |
| sp\|O60242\|AGRB3_HUMAN Adhesion G protein-coupled receptor B3 OS=Homo sapiens GN=ADGRB3 PE=1 S' | 1084 | KCGVVStTALS | 0.05 | -0.235 | -1.761 | -0.649 |
| sp\|O60242\|AGRB3_HUMAN Adhesion G protein-coupled receptor B3 OS=Homo sapiens GN=ADGRB3 PE=1 S' | 1085 | CGVVSTtALSA | 0.156 | 0.112 | -0.687 | -0.14 |
| sp\|O60242\|AGRB3_HUMAN Adhesion G protein-coupled receptor B3 OS=Homo sapiens GN=ADGRB3 PE=1 S' | 1088 | VSTTALsATTA | 0.208 | -0.144 | -0.919 | -0.285 |
| sp\|O60242\|AGRB3_HUMAN Adhesion G protein-coupled receptor B3 OS=Homo sapiens GN=ADGRB3 PE=1 S' | 1090 | TTALSAtTASN | 0.214 | 0.061 | -0.781 | -0.169 |
| sp\|O60242\|AGRB3_HUMAN Adhesion G protein-coupled receptor B3 OS=Homo sapiens GN=ADGRB3 PE=1 S' | 1091 | TALSATtASNA | 0.147 | -0.108 | -1.114 | -0.358 |
| sp\|O60242\|AGRB3_HUMAN Adhesion G protein-coupled receptor B3 OS=Homo sapiens GN=ADGRB3 PE=1 S' | 1093 | LSATTAsNAMA | 0.095 | -0.249 | -1.244 | -0.466 |
| sp\|O60242\|AGRB3_HUMAN Adhesion G protein-coupled receptor B3 OS=Homo sapiens GN=ADGRB3 PE=1 S' | 1098 | ASNAMAsLWSS | 0.254 | -0.132 | -0.863 | -0.247 |
| sp\|O60242\|AGRB3_HUMAN Adhesion G protein-coupled receptor B3 OS=Homo sapiens GN=ADGRB3 PE=1 S' | 1101 | AMASLWsSCVV | 0.06 | -0.221 | -1.714 | -0.625 |
| sp\|O60242\|AGRB3_HUMAN Adhesion G protein-coupled receptor B3 OS=Homo sapiens GN=ADGRB3 PE=1 S' | 1102 | MASLWSsCVVL | 0.169 | 0.091 | -0.919 | -0.22 |
| sp\|O60242\|AGRB3_HUMAN Adhesion G protein-coupled receptor B3 OS=Homo sapiens GN=ADGRB3 PE=1 S' | 1112 | LPLLALtWMSA | 0.089 | -0.042 | -1.049 | -0.334 |
| sp\|O60242\|AGRB3_HUMAN Adhesion G protein-coupled receptor B3 OS=Homo sapiens GN=ADGRB3 PE=1 S' | 1115 | LALTWMsAVLA | 0.257 | 0.094 | -0.617 | -0.089 |

| | | | | | | |
|---|---|---|---|---|---|---|
| sp\|O60242\|AGRB3_HUMAN Adhesion G protein-coupled receptor B3 OS=Homo sapiens GN=ADGRB3 PE=1 S' | 1121 | SAVLAMtDKRS | 0.19 | -0.025 | -1.183 | -0.339 |
| sp\|O60242\|AGRB3_HUMAN Adhesion G protein-coupled receptor B3 OS=Homo sapiens GN=ADGRB3 PE=1 S' | 1125 | AMTDKRsILFQ | 0.032 | -0.22 | -1.902 | -0.697 |
| sp\|O60242\|AGRB3_HUMAN Adhesion G protein-coupled receptor B3 OS=Homo sapiens GN=ADGRB3 PE=1 S' | 1137 | LFAVFDsLQGF | 0.21 | -0.105 | -0.535 | -0.143 |
| sp\|O60242\|AGRB3_HUMAN Adhesion G protein-coupled receptor B3 OS=Homo sapiens GN=ADGRB3 PE=1 S' | 1173 | DPINADsSSSF | 0.052 | -0.256 | -1.617 | -0.607 |
| sp\|O60242\|AGRB3_HUMAN Adhesion G protein-coupled receptor B3 OS=Homo sapiens GN=ADGRB3 PE=1 S' | 1174 | PINADSsSSFP | 0.039 | -0.339 | -1.759 | -0.686 |
| sp\|O60242\|AGRB3_HUMAN Adhesion G protein-coupled receptor B3 OS=Homo sapiens GN=ADGRB3 PE=1 S' | 1175 | INADSSsSFPN | 0.189 | -0.035 | -0.612 | -0.153 |
| sp\|O60242\|AGRB3_HUMAN Adhesion G protein-coupled receptor B3 OS=Homo sapiens GN=ADGRB3 PE=1 S' | 1176 | NADSSSsFPNG | 0.638 | 0.599 | 0.12 | 0.452 |
| sp\|O60242\|AGRB3_HUMAN Adhesion G protein-coupled receptor B3 OS=Homo sapiens GN=ADGRB3 PE=1 S' | 1186 | GHAQIMtDFEK | 0.474 | 0.155 | -0.295 | 0.111 |
| sp\|O60242\|AGRB3_HUMAN Adhesion G protein-coupled receptor B3 OS=Homo sapiens GN=ADGRB3 PE=1 S' | 1198 | VDIACRsVLHK | 0.064 | -0.286 | -1.381 | -0.534 |
| sp\|O60242\|AGRB3_HUMAN Adhesion G protein-coupled receptor B3 OS=Homo sapiens GN=ADGRB3 PE=1 S' | 1211 | GPCRAAtITGT | 0.75 | 0.812 | 0.423 | 0.662 |
| sp\|O60242\|AGRB3_HUMAN Adhesion G protein-coupled receptor B3 OS=Homo sapiens GN=ADGRB3 PE=1 S' | 1213 | CRAATItGTLS | 0.219 | 0.141 | -0.527 | -0.056 |
| sp\|O60242\|AGRB3_HUMAN Adhesion G protein-coupled receptor B3 OS=Homo sapiens GN=ADGRB3 PE=1 S' | 1215 | AATITGtLSRI | 0.145 | -0.134 | -0.996 | -0.328 |
| sp\|O60242\|AGRB3_HUMAN Adhesion G protein-coupled receptor B3 OS=Homo sapiens GN=ADGRB3 PE=1 S' | 1217 | TITGTLsRISL | 0.047 | -0.095 | -1.22 | -0.423 |
| sp\|O60242\|AGRB3_HUMAN Adhesion G protein-coupled receptor B3 OS=Homo sapiens GN=ADGRB3 PE=1 S' | 1220 | GTLSRIsLNDD | 0.818 | 0.291 | 0.436 | 0.515 |
| sp\|O60242\|AGRB3_HUMAN Adhesion G protein-coupled receptor B3 OS=Homo sapiens GN=ADGRB3 PE=1 S' | 1230 | DEEEKGtNPEG | 0.297 | 0.235 | -0.908 | -0.125 |
| sp\|O60242\|AGRB3_HUMAN Adhesion G protein-coupled receptor B3 OS=Homo sapiens GN=ADGRB3 PE=1 S' | 1236 | TNPEGLsYSTL | 0.014 | -0.452 | -2.002 | -0.813 |
| sp\|O60242\|AGRB3_HUMAN Adhesion G protein-coupled receptor B3 OS=Homo sapiens GN=ADGRB3 PE=1 S' | 1238 | PEGLSYsTLPG | 0.183 | 0.139 | -0.711 | -0.13 |
| sp\|O60242\|AGRB3_HUMAN Adhesion G protein-coupled receptor B3 OS=Homo sapiens GN=ADGRB3 PE=1 S' | 1239 | EGLSYStLPGN | 0.539 | 0.387 | 0.185 | 0.37 |
| sp\|O60242\|AGRB3_HUMAN Adhesion G protein-coupled receptor B3 OS=Homo sapiens GN=ADGRB3 PE=1 S' | 1246 | LPGNVIsKVII | 0.167 | -0.078 | -0.659 | -0.19 |
| sp\|O60242\|AGRB3_HUMAN Adhesion G protein-coupled receptor B3 OS=Homo sapiens GN=ADGRB3 PE=1 S' | 1254 | VIIQQPtGLHM | 0.087 | -0.201 | -1.171 | -0.428 |
| sp\|O60242\|AGRB3_HUMAN Adhesion G protein-coupled receptor B3 OS=Homo sapiens GN=ADGRB3 PE=1 S' | 1261 | GLHMPMsMNEL | 0.451 | 0.271 | -0.215 | 0.169 |
| sp\|O60242\|AGRB3_HUMAN Adhesion G protein-coupled receptor B3 OS=Homo sapiens GN=ADGRB3 PE=1 S' | 1266 | MSMNELsNPCL | 0.34 | 0.479 | -0.359 | 0.153 |
| sp\|O60242\|AGRB3_HUMAN Adhesion G protein-coupled receptor B3 OS=Homo sapiens GN=ADGRB3 PE=1 S' | 1275 | CLKKENsELRR | 0.663 | 0.188 | -0.19 | 0.22 |
| sp\|O60242\|AGRB3_HUMAN Adhesion G protein-coupled receptor B3 OS=Homo sapiens GN=ADGRB3 PE=1 S' | 1280 | NSELRRtVYLC | 0.19 | -0.007 | -1.046 | -0.288 |
| sp\|O60242\|AGRB3_HUMAN Adhesion G protein-coupled receptor B3 OS=Homo sapiens GN=ADGRB3 PE=1 S' | 1285 | RTVYLCtDDNL | 0.384 | 0.146 | -0.863 | -0.111 |
| sp\|O60242\|AGRB3_HUMAN Adhesion G protein-coupled receptor B3 OS=Homo sapiens GN=ADGRB3 PE=1 S' | 1306 | QERMMEsDYIV | 0.394 | 0.062 | -0.926 | -0.157 |
| sp\|O60242\|AGRB3_HUMAN Adhesion G protein-coupled receptor B3 OS=Homo sapiens GN=ADGRB3 PE=1 S' | 1314 | YIVMPRsSVNN | 0.092 | -0.076 | -1.304 | -0.429 |
| sp\|O60242\|AGRB3_HUMAN Adhesion G protein-coupled receptor B3 OS=Homo sapiens GN=ADGRB3 PE=1 S' | 1315 | IVMPRSsVNNQ | 0.205 | 0.07 | -0.676 | -0.134 |
| sp\|O60242\|AGRB3_HUMAN Adhesion G protein-coupled receptor B3 OS=Homo sapiens GN=ADGRB3 PE=1 S' | 1321 | SVNNQPsMKEE | 0.049 | -0.128 | -1.531 | -0.537 |
| sp\|O60242\|AGRB3_HUMAN Adhesion G protein-coupled receptor B3 OS=Homo sapiens GN=ADGRB3 PE=1 S' | 1326 | PSMKEEsKMNI | 0.353 | 0.031 | -0.686 | -0.101 |
| sp\|O60242\|AGRB3_HUMAN Adhesion G protein-coupled receptor B3 OS=Homo sapiens GN=ADGRB3 PE=1 S' | 1334 | MNIGMEtLPHE | 0.544 | 0.376 | 0.111 | 0.344 |
| sp\|O60242\|AGRB3_HUMAN Adhesion G protein-coupled receptor B3 OS=Homo sapiens GN=ADGRB3 PE=1 S' | 1382 | LPFEPRtAVKN | 0.053 | -0.162 | -1.643 | -0.584 |
| sp\|O60242\|AGRB3_HUMAN Adhesion G protein-coupled receptor B3 OS=Homo sapiens GN=ADGRB3 PE=1 S' | 1390 | VKNFMAsELDD | 0.541 | 0.132 | -0.258 | 0.138 |
| sp\|O60242\|AGRB3_HUMAN Adhesion G protein-coupled receptor B3 OS=Homo sapiens GN=ADGRB3 PE=1 S' | 1399 | DDNAGLsRSET | 0.029 | -0.487 | -1.981 | -0.813 |
| sp\|O60242\|AGRB3_HUMAN Adhesion G protein-coupled receptor B3 OS=Homo sapiens GN=ADGRB3 PE=1 S' | 1401 | NAGLSRsETGS | 0.221 | 0.091 | -0.798 | -0.162 |
| sp\|O60242\|AGRB3_HUMAN Adhesion G protein-coupled receptor B3 OS=Homo sapiens GN=ADGRB3 PE=1 S' | 1403 | GLSRSEtGSTI | 0.832 | 1.041 | 0.796 | 0.89 |
| sp\|O60242\|AGRB3_HUMAN Adhesion G protein-coupled receptor B3 OS=Homo sapiens GN=ADGRB3 PE=1 S' | 1405 | SRSETGsTISM | 0.032 | -0.062 | -1.635 | -0.555 |
| sp\|O60242\|AGRB3_HUMAN Adhesion G protein-coupled receptor B3 OS=Homo sapiens GN=ADGRB3 PE=1 S' | 1406 | RSETGStISMS | 0.04 | -0.431 | -1.763 | -0.718 |
| sp\|O60242\|AGRB3_HUMAN Adhesion G protein-coupled receptor B3 OS=Homo sapiens GN=ADGRB3 PE=1 S' | 1408 | ETGSTIsMSSL | 0.037 | -0.094 | -1.556 | -0.538 |
| sp\|O60242\|AGRB3_HUMAN Adhesion G protein-coupled receptor B3 OS=Homo sapiens GN=ADGRB3 PE=1 S' | 1410 | GSTISMsSLER | 0.41 | 0.283 | -0.237 | 0.152 |
| sp\|O60242\|AGRB3_HUMAN Adhesion G protein-coupled receptor B3 OS=Homo sapiens GN=ADGRB3 PE=1 S' | 1411 | STISMSsLERR | 0.132 | -0.26 | -1.233 | -0.454 |
| sp\|O60242\|AGRB3_HUMAN Adhesion G protein-coupled receptor B3 OS=Homo sapiens GN=ADGRB3 PE=1 S' | 1417 | SLERRKsRYSD | 0.765 | 0.986 | 0.661 | 0.804 |
| sp\|O60242\|AGRB3_HUMAN Adhesion G protein-coupled receptor B3 OS=Homo sapiens GN=ADGRB3 PE=1 S' | 1420 | RRKSRYsDLDF | 0.538 | 0.487 | -0.059 | 0.322 |
| sp\|O60242\|AGRB3_HUMAN Adhesion G protein-coupled receptor B3 OS=Homo sapiens GN=ADGRB3 PE=1 S' | 1430 | FEKVMHtRKRH | 0.144 | -0.199 | -1.311 | -0.455 |
| sp\|O60242\|AGRB3_HUMAN Adhesion G protein-coupled receptor B3 OS=Homo sapiens GN=ADGRB3 PE=1 S' | 1447 | LNQKFQtLDRF | 0.416 | 0.018 | -0.287 | 0.049 |
| sp\|O60242\|AGRB3_HUMAN Adhesion G protein-coupled receptor B3 OS=Homo sapiens GN=ADGRB3 PE=1 S' | 1457 | FRDIPNtSSME | 0.116 | 0.013 | -1.215 | -0.362 |
| sp\|O60242\|AGRB3_HUMAN Adhesion G protein-coupled receptor B3 OS=Homo sapiens GN=ADGRB3 PE=1 S' | 1458 | RDIPNTsSMEN | 0.146 | -0.115 | -1.019 | -0.329 |
| sp\|O60242\|AGRB3_HUMAN Adhesion G protein-coupled receptor B3 OS=Homo sapiens GN=ADGRB3 PE=1 S' | 1459 | DIPNTSsMENP | 0.045 | -0.008 | -1.572 | -0.512 |
| sp\|O60242\|AGRB3_HUMAN Adhesion G protein-coupled receptor B3 OS=Homo sapiens GN=ADGRB3 PE=1 S' | 1472 | NKNPWDtFKNP | 0.259 | -0.033 | -0.818 | -0.197 |

| | | | | | | |
|---|---|---|---|---|---|---|
| sp\|O60242\|AGRB3_HUMAN Adhesion G protein-coupled receptor B3 OS=Homo sapiens GN=ADGRB3 PE=1 S' | 1477 | DTFKNPsEYPH | 0.285 | -0.15 | -0.924 | -0.263 |
| sp\|O60242\|AGRB3_HUMAN Adhesion G protein-coupled receptor B3 OS=Homo sapiens GN=ADGRB3 PE=1 S' | 1483 | SEYPHYtTINV | 0.089 | -0.026 | -1.352 | -0.43 |
| sp\|O60242\|AGRB3_HUMAN Adhesion G protein-coupled receptor B3 OS=Homo sapiens GN=ADGRB3 PE=1 S' | 1484 | EYPHYTtINVL | 0.152 | -0.015 | -0.653 | -0.172 |
| sp\|O60242\|AGRB3_HUMAN Adhesion G protein-coupled receptor B3 OS=Homo sapiens GN=ADGRB3 PE=1 S' | 1490 | TINVLDtEAKD | 0.083 | -0.145 | -1.466 | -0.509 |
| sp\|O60242\|AGRB3_HUMAN Adhesion G protein-coupled receptor B3 OS=Homo sapiens GN=ADGRB3 PE=1 S' | 1520 | QEGDFQtEV-- | 0.123 | 0.104 | -1.199 | -0.324 |
| sp\|P48960\|CD97_HUMAN CD97 antigen OS=Homo sapiens GN=CD97 PE=1 SV=4 | 328 | VRHLIAtQLLS | 0.098 | 0.051 | -1.3 | -0.384 |
| sp\|P48960\|CD97_HUMAN CD97 antigen OS=Homo sapiens GN=CD97 PE=1 SV=4 | 332 | IATQLLsNLED | 0.129 | -0.194 | -1.082 | -0.382 |
| sp\|P48960\|CD97_HUMAN CD97 antigen OS=Homo sapiens GN=CD97 PE=1 SV=4 | 344 | MRILAKsLPKG | 0.891 | 1.014 | 1.026 | 0.977 |
| sp\|P48960\|CD97_HUMAN CD97 antigen OS=Homo sapiens GN=CD97 PE=1 SV=4 | 351 | LPKGPFtYISP | 0.038 | -0.255 | -1.109 | -0.442 |
| sp\|P48960\|CD97_HUMAN CD97 antigen OS=Homo sapiens GN=CD97 PE=1 SV=4 | 354 | GPFTYIsPSNT | 0.083 | -0.394 | -1.591 | -0.634 |
| sp\|P48960\|CD97_HUMAN CD97 antigen OS=Homo sapiens GN=CD97 PE=1 SV=4 | 356 | FTYISPsNTEL | 0.395 | 0.196 | -0.521 | 0.023 |
| sp\|P48960\|CD97_HUMAN CD97 antigen OS=Homo sapiens GN=CD97 PE=1 SV=4 | 358 | YISPSNtELTL | 0.386 | 0.3 | -0.22 | 0.155 |
| sp\|P48960\|CD97_HUMAN CD97 antigen OS=Homo sapiens GN=CD97 PE=1 SV=4 | 361 | PSNTELtLMIQ | 0.215 | -0.15 | -1.074 | -0.336 |
| sp\|P48960\|CD97_HUMAN CD97 antigen OS=Homo sapiens GN=CD97 PE=1 SV=4 | 373 | RGDKNVtMGQS | 0.562 | 0.125 | -0.181 | 0.169 |
| sp\|P48960\|CD97_HUMAN CD97 antigen OS=Homo sapiens GN=CD97 PE=1 SV=4 | 377 | NVTMGQsSARM | 0.074 | -0.305 | -1.257 | -0.496 |
| sp\|P48960\|CD97_HUMAN CD97 antigen OS=Homo sapiens GN=CD97 PE=1 SV=4 | 378 | VTMGQSsARMK | 0.067 | -0.275 | -1.509 | -0.572 |
| sp\|P48960\|CD97_HUMAN CD97 antigen OS=Homo sapiens GN=CD97 PE=1 SV=4 | 403 | AVAGILsIQNM | 0.078 | -0.122 | -0.911 | -0.318 |
| sp\|P48960\|CD97_HUMAN CD97 antigen OS=Homo sapiens GN=CD97 PE=1 SV=4 | 408 | LSIQNMtTLLA | 0.204 | 0.002 | -0.575 | -0.123 |
| sp\|P48960\|CD97_HUMAN CD97 antigen OS=Homo sapiens GN=CD97 PE=1 SV=4 | 409 | SIQNMTtLLAN | 0.273 | 0.135 | -0.368 | 0.013 |
| sp\|P48960\|CD97_HUMAN CD97 antigen OS=Homo sapiens GN=CD97 PE=1 SV=4 | 415 | TLLANAsLNLH | 0.5 | 0.057 | -0.123 | 0.145 |
| sp\|P48960\|CD97_HUMAN CD97 antigen OS=Homo sapiens GN=CD97 PE=1 SV=4 | 420 | ASLNLHsKKQA | 0.154 | -0.138 | -0.712 | -0.232 |
| sp\|P48960\|CD97_HUMAN CD97 antigen OS=Homo sapiens GN=CD97 PE=1 SV=4 | 432 | LEEIYEsSIRG | 0.105 | -0.218 | -1.307 | -0.473 |
| sp\|P48960\|CD97_HUMAN CD97 antigen OS=Homo sapiens GN=CD97 PE=1 SV=4 | 433 | EEIYESsIRGV | 0.071 | -0.274 | -1.451 | -0.551 |
| sp\|P48960\|CD97_HUMAN CD97 antigen OS=Homo sapiens GN=CD97 PE=1 SV=4 | 443 | VQLRRLsAVNS | 0.529 | 0.949 | 0.077 | 0.518 |
| sp\|P48960\|CD97_HUMAN CD97 antigen OS=Homo sapiens GN=CD97 PE=1 SV=4 | 447 | RLSAVNsIFLS | 0.337 | 0.043 | -0.601 | -0.074 |
| sp\|P48960\|CD97_HUMAN CD97 antigen OS=Homo sapiens GN=CD97 PE=1 SV=4 | 451 | VNSIFLsHNNT | 0.087 | -0.214 | -1.23 | -0.452 |
| sp\|P48960\|CD97_HUMAN CD97 antigen OS=Homo sapiens GN=CD97 PE=1 SV=4 | 455 | FLSHNNtKELN | 0.422 | 0.16 | -0.336 | 0.082 |
| sp\|P48960\|CD97_HUMAN CD97 antigen OS=Homo sapiens GN=CD97 PE=1 SV=4 | 460 | NTKELNsPILF | 0.061 | -0.339 | -1.423 | -0.567 |
| sp\|P48960\|CD97_HUMAN CD97 antigen OS=Homo sapiens GN=CD97 PE=1 SV=4 | 467 | PILFAFsHLES | 0.399 | 0.186 | -0.392 | 0.064 |
| sp\|P48960\|CD97_HUMAN CD97 antigen OS=Homo sapiens GN=CD97 PE=1 SV=4 | 471 | AFSHLEsSDGE | 0.116 | -0.28 | -1.183 | -0.449 |
| sp\|P48960\|CD97_HUMAN CD97 antigen OS=Homo sapiens GN=CD97 PE=1 SV=4 | 472 | FSHLESsDGEA | 0.179 | -0.088 | -1.135 | -0.348 |
| sp\|P48960\|CD97_HUMAN CD97 antigen OS=Homo sapiens GN=CD97 PE=1 SV=4 | 500 | LCAFWKsDSDR | 0.484 | 0.182 | -0.129 | 0.179 |
| sp\|P48960\|CD97_HUMAN CD97 antigen OS=Homo sapiens GN=CD97 PE=1 SV=4 | 502 | AFWKSDsDRGG | 0.573 | 0.203 | -0.141 | 0.212 |
| sp\|P48960\|CD97_HUMAN CD97 antigen OS=Homo sapiens GN=CD97 PE=1 SV=4 | 510 | RGGHWAtEGCQ | 0.332 | 0.146 | -0.505 | -0.009 |
| sp\|P48960\|CD97_HUMAN CD97 antigen OS=Homo sapiens GN=CD97 PE=1 SV=4 | 518 | GCQVLGsKNGS | 0.136 | -0.093 | -1.124 | -0.36 |
| sp\|P48960\|CD97_HUMAN CD97 antigen OS=Homo sapiens GN=CD97 PE=1 SV=4 | 522 | LGSKNGsTTCQ | 0.201 | -0.154 | -0.888 | -0.28 |
| sp\|P48960\|CD97_HUMAN CD97 antigen OS=Homo sapiens GN=CD97 PE=1 SV=4 | 523 | GSKNGStTCQC | 0.058 | -0.378 | -1.433 | -0.584 |
| sp\|P48960\|CD97_HUMAN CD97 antigen OS=Homo sapiens GN=CD97 PE=1 SV=4 | 524 | SKNGSTtCQCS | 0.346 | 0.299 | -0.424 | 0.074 |
| sp\|P48960\|CD97_HUMAN CD97 antigen OS=Homo sapiens GN=CD97 PE=1 SV=4 | 528 | STTCQCsHLSS | 0.085 | -0.208 | -1.735 | -0.619 |
| sp\|P48960\|CD97_HUMAN CD97 antigen OS=Homo sapiens GN=CD97 PE=1 SV=4 | 531 | CQCSHLsSFAI | 0.259 | 0.041 | -0.385 | -0.028 |
| sp\|P48960\|CD97_HUMAN CD97 antigen OS=Homo sapiens GN=CD97 PE=1 SV=4 | 532 | QCSHLSsFAIL | 0.141 | -0.122 | -1.067 | -0.349 |
| sp\|P48960\|CD97_HUMAN CD97 antigen OS=Homo sapiens GN=CD97 PE=1 SV=4 | 548 | VEDWKLtLITR | 0.125 | -0.176 | -0.82 | -0.29 |
| sp\|P48960\|CD97_HUMAN CD97 antigen OS=Homo sapiens GN=CD97 PE=1 SV=4 | 551 | WKLTLItRVGL | 0.21 | -0.153 | -0.744 | -0.229 |
| sp\|P48960\|CD97_HUMAN CD97 antigen OS=Homo sapiens GN=CD97 PE=1 SV=4 | 558 | RVGLALsLFCL | 0.272 | 0.216 | -0.445 | 0.014 |
| sp\|P48960\|CD97_HUMAN CD97 antigen OS=Homo sapiens GN=CD97 PE=1 SV=4 | 568 | LLLCILtFLLV | 0.242 | 0.066 | -0.654 | -0.115 |
| sp\|P48960\|CD97_HUMAN CD97 antigen OS=Homo sapiens GN=CD97 PE=1 SV=4 | 578 | VRPIQGsRTTI | 0.1 | -0.06 | -1.312 | -0.424 |
| sp\|P48960\|CD97_HUMAN CD97 antigen OS=Homo sapiens GN=CD97 PE=1 SV=4 | 580 | PIQGSRtTIHL | 0.219 | 0.285 | -0.304 | 0.067 |
| sp\|P48960\|CD97_HUMAN CD97 antigen OS=Homo sapiens GN=CD97 PE=1 SV=4 | 581 | IQGSRTtIHLH | 0.139 | -0.114 | -0.87 | -0.282 |
| sp\|P48960\|CD97_HUMAN CD97 antigen OS=Homo sapiens GN=CD97 PE=1 SV=4 | 594 | ICLFVGsTIFL | 0.162 | -0.108 | -1.011 | -0.319 |

| | | | | | | |
|---|---|---|---|---|---|---|
| sp\|P48960\|CD97_HUMAN CD97 antigen OS=Homo sapiens GN=CD97 PE=1 SV=4 | 595 | CLFVGStIFLA | 0.103 | -0.089 | -0.841 | -0.276 |
| sp\|P48960\|CD97_HUMAN CD97 antigen OS=Homo sapiens GN=CD97 PE=1 SV=4 | 631 | AAFCWMsLEGL | 0.514 | 0.155 | -0.21 | 0.153 |
| sp\|P48960\|CD97_HUMAN CD97 antigen OS=Homo sapiens GN=CD97 PE=1 SV=4 | 651 | FQGQGLsTRWL | 0.066 | -0.123 | -0.932 | -0.33 |
| sp\|P48960\|CD97_HUMAN CD97 antigen OS=Homo sapiens GN=CD97 PE=1 SV=4 | 652 | QGQGLStRWLC | 0.073 | -0.235 | -1.5 | -0.554 |
| sp\|P48960\|CD97_HUMAN CD97 antigen OS=Homo sapiens GN=CD97 PE=1 SV=4 | 670 | LLIVGVsAAIY | 0.259 | 0.009 | -0.87 | -0.201 |
| sp\|P48960\|CD97_HUMAN CD97 antigen OS=Homo sapiens GN=CD97 PE=1 SV=4 | 675 | VSAAIYsKGYG | 0.226 | -0.076 | -0.356 | -0.069 |
| sp\|P48960\|CD97_HUMAN CD97 antigen OS=Homo sapiens GN=CD97 PE=1 SV=4 | 695 | EQGFLWsFLGP | 0.066 | -0.214 | -1.165 | -0.438 |
| sp\|P48960\|CD97_HUMAN CD97 antigen OS=Homo sapiens GN=CD97 PE=1 SV=4 | 701 | SFLGPVtFIIL | 0.261 | -0.036 | -0.309 | -0.028 |
| sp\|P48960\|CD97_HUMAN CD97 antigen OS=Homo sapiens GN=CD97 PE=1 SV=4 | 713 | NAVIFVtTVWK | 0.176 | -0.133 | -0.996 | -0.318 |
| sp\|P48960\|CD97_HUMAN CD97 antigen OS=Homo sapiens GN=CD97 PE=1 SV=4 | 714 | AVIFVTtVWKL | 0.22 | 0.058 | -0.84 | -0.187 |
| sp\|P48960\|CD97_HUMAN CD97 antigen OS=Homo sapiens GN=CD97 PE=1 SV=4 | 719 | TTVWKLtQKFS | 0.038 | -0.395 | -1.787 | -0.715 |
| sp\|P48960\|CD97_HUMAN CD97 antigen OS=Homo sapiens GN=CD97 PE=1 SV=4 | 723 | KLTQKFsEINP | 0.201 | 0.086 | -0.565 | -0.093 |
| sp\|P48960\|CD97_HUMAN CD97 antigen OS=Homo sapiens GN=CD97 PE=1 SV=4 | 739 | KKARALtITAI | 0.829 | 0.991 | 1.023 | 0.948 |
| sp\|P48960\|CD97_HUMAN CD97 antigen OS=Homo sapiens GN=CD97 PE=1 SV=4 | 741 | ARALTItAIAQ | 0.206 | 0.238 | -0.633 | -0.063 |
| sp\|P48960\|CD97_HUMAN CD97 antigen OS=Homo sapiens GN=CD97 PE=1 SV=4 | 752 | LFLLGCtWVFG | 0.113 | -0.058 | -1.06 | -0.335 |
| sp\|P48960\|CD97_HUMAN CD97 antigen OS=Homo sapiens GN=CD97 PE=1 SV=4 | 764 | FIFDDRsLVLT | 0.254 | 0.158 | -0.704 | -0.097 |
| sp\|P48960\|CD97_HUMAN CD97 antigen OS=Homo sapiens GN=CD97 PE=1 SV=4 | 768 | DRSLVLtYVFT | 0.074 | -0.083 | -1.434 | -0.481 |
| sp\|P48960\|CD97_HUMAN CD97 antigen OS=Homo sapiens GN=CD97 PE=1 SV=4 | 772 | VLTYVFtILNC | 0.161 | -0.001 | -0.911 | -0.25 |
| sp\|P48960\|CD97_HUMAN CD97 antigen OS=Homo sapiens GN=CD97 PE=1 SV=4 | 808 | CLVAGGsKYSE | 0.084 | -0.171 | -1.108 | -0.398 |
| sp\|P48960\|CD97_HUMAN CD97 antigen OS=Homo sapiens GN=CD97 PE=1 SV=4 | 811 | AGGSKYsEFTS | 0.081 | -0.114 | -1.01 | -0.348 |
| sp\|P48960\|CD97_HUMAN CD97 antigen OS=Homo sapiens GN=CD97 PE=1 SV=4 | 814 | SKYSEFtSTTS | 0.123 | -0.188 | -1.269 | -0.445 |
| sp\|P48960\|CD97_HUMAN CD97 antigen OS=Homo sapiens GN=CD97 PE=1 SV=4 | 815 | KYSEFTsTTSG | 0.106 | -0.175 | -1.339 | -0.469 |
| sp\|P48960\|CD97_HUMAN CD97 antigen OS=Homo sapiens GN=CD97 PE=1 SV=4 | 816 | YSEFTStTSGT | 0.065 | -0.298 | -1.29 | -0.508 |
| sp\|P48960\|CD97_HUMAN CD97 antigen OS=Homo sapiens GN=CD97 PE=1 SV=4 | 817 | SEFTSTtSGTG | 0.284 | 0.084 | -0.695 | -0.109 |
| sp\|P48960\|CD97_HUMAN CD97 antigen OS=Homo sapiens GN=CD97 PE=1 SV=4 | 818 | EFTSTTsGTGH | 0.076 | -0.276 | -1.075 | -0.425 |
| sp\|P48960\|CD97_HUMAN CD97 antigen OS=Homo sapiens GN=CD97 PE=1 SV=4 | 820 | TSTTSGtGHNQ | 0.175 | -0.045 | -0.973 | -0.281 |
| sp\|P48960\|CD97_HUMAN CD97 antigen OS=Homo sapiens GN=CD97 PE=1 SV=4 | 825 | GTGHNQtRALR | 0.163 | -0.167 | -0.992 | -0.332 |
| sp\|P48960\|CD97_HUMAN CD97 antigen OS=Homo sapiens GN=CD97 PE=1 SV=4 | 831 | TRALRAsESGI | 0.491 | 0.373 | -0.064 | 0.267 |
| sp\|P48960\|CD97_HUMAN CD97 antigen OS=Homo sapiens GN=CD97 PE=1 SV=4 | 833 | ALRASEsGI-- | 0.567 | 0.489 | -0.002 | 0.351 |
| sp\|Q9NYQ6\|CELR1_HUMAN Cadherin EGF LAG seven-pass G-type receptor 1 OS=Homo sapiens GN=CELSR1 I | 315 | SVLDREtKETH | 0.105 | -0.145 | -1.373 | -0.471 |
| sp\|Q9NYQ6\|CELR1_HUMAN Cadherin EGF LAG seven-pass G-type receptor 1 OS=Homo sapiens GN=CELSR1 I | 318 | DRETKEtHVLR | 0.224 | 0.071 | -0.972 | -0.226 |
| sp\|Q9NYQ6\|CELR1_HUMAN Cadherin EGF LAG seven-pass G-type receptor 1 OS=Homo sapiens GN=CELSR1 I | 329 | VKAVDYsTPPR | 0.498 | 0.451 | 0.19 | 0.38 |
| sp\|Q9NYQ6\|CELR1_HUMAN Cadherin EGF LAG seven-pass G-type receptor 1 OS=Homo sapiens GN=CELSR1 I | 330 | KAVDYStPPRS | 0.156 | -0.063 | -1.219 | -0.375 |
| sp\|Q9NYQ6\|CELR1_HUMAN Cadherin EGF LAG seven-pass G-type receptor 1 OS=Homo sapiens GN=CELSR1 I | 334 | YSTPPRsATTY | 0.139 | -0.201 | -1.112 | -0.391 |
| sp\|Q9NYQ6\|CELR1_HUMAN Cadherin EGF LAG seven-pass G-type receptor 1 OS=Homo sapiens GN=CELSR1 I | 336 | TPPRSAtTYIT | 0.646 | 0.903 | 0.319 | 0.623 |
| sp\|Q9NYQ6\|CELR1_HUMAN Cadherin EGF LAG seven-pass G-type receptor 1 OS=Homo sapiens GN=CELSR1 I | 337 | PPRSATtYITV | 0.335 | 0.139 | -0.585 | -0.037 |
| sp\|Q9NYQ6\|CELR1_HUMAN Cadherin EGF LAG seven-pass G-type receptor 1 OS=Homo sapiens GN=CELSR1 I | 340 | SATTYItVLVK | 0.116 | -0.181 | -1.202 | -0.422 |
| sp\|Q9NYQ6\|CELR1_HUMAN Cadherin EGF LAG seven-pass G-type receptor 1 OS=Homo sapiens GN=CELSR1 I | 346 | TVLVKDtNDHS | 0.041 | -0.318 | -1.62 | -0.632 |
| sp\|Q9NYQ6\|CELR1_HUMAN Cadherin EGF LAG seven-pass G-type receptor 1 OS=Homo sapiens GN=CELSR1 I | 350 | KDTNDHsPVFE | 0.035 | -0.478 | -1.609 | -0.684 |
| sp\|Q9NYQ6\|CELR1_HUMAN Cadherin EGF LAG seven-pass G-type receptor 1 OS=Homo sapiens GN=CELSR1 I | 356 | SPVFEQsEYRE | 0.114 | -0.168 | -1.433 | -0.496 |
| sp\|Q9NYQ6\|CELR1_HUMAN Cadherin EGF LAG seven-pass G-type receptor 1 OS=Homo sapiens GN=CELSR1 I | 374 | VGYEVLtIRAS | 0.058 | -0.232 | -1.2 | -0.458 |
| sp\|Q9NYQ6\|CELR1_HUMAN Cadherin EGF LAG seven-pass G-type receptor 1 OS=Homo sapiens GN=CELSR1 I | 378 | VLTIRAsDRDS | 0.641 | 0.25 | -0.123 | 0.256 |
| sp\|Q9NYQ6\|CELR1_HUMAN Cadherin EGF LAG seven-pass G-type receptor 1 OS=Homo sapiens GN=CELSR1 I | 382 | RASDRDsPINA | 0.132 | -0.271 | -1.322 | -0.487 |
| sp\|Q9NYQ6\|CELR1_HUMAN Cadherin EGF LAG seven-pass G-type receptor 1 OS=Homo sapiens GN=CELSR1 I | 405 | VFQLNEsSGVV | 0.261 | -0.001 | -0.929 | -0.223 |
| sp\|Q9NYQ6\|CELR1_HUMAN Cadherin EGF LAG seven-pass G-type receptor 1 OS=Homo sapiens GN=CELSR1 I | 406 | FQLNESsGVVS | 0.052 | -0.146 | -1.21 | -0.435 |
| sp\|Q9NYQ6\|CELR1_HUMAN Cadherin EGF LAG seven-pass G-type receptor 1 OS=Homo sapiens GN=CELSR1 I | 410 | ESSGVVsTRAV | 0.228 | -0.149 | -0.823 | -0.248 |
| sp\|Q9NYQ6\|CELR1_HUMAN Cadherin EGF LAG seven-pass G-type receptor 1 OS=Homo sapiens GN=CELSR1 I | 411 | SSGVVStRAVL | 0.033 | -0.36 | -1.938 | -0.755 |
| sp\|Q9NYQ6\|CELR1_HUMAN Cadherin EGF LAG seven-pass G-type receptor 1 OS=Homo sapiens GN=CELSR1 I | 440 | RNPGPLsATAT | 0.075 | -0.23 | -1.167 | -0.441 |
| sp\|Q9NYQ6\|CELR1_HUMAN Cadherin EGF LAG seven-pass G-type receptor 1 OS=Homo sapiens GN=CELSR1 I | 442 | PGPLSAtATVY | 0.51 | 0.255 | -0.426 | 0.113 |

| | | | | | | |
|---|---|---|---|---|---|---|
| sp\|Q9NYQ6\|CELR1_HUMAN Cadherin EGF LAG seven-pass G-type receptor 1 OS=Homo sapiens GN=CELSR1 I | 444 | PLSATAtVYIE | 0.327 | 0.071 | -0.528 | -0.043 |
| sp\|Q9NYQ6\|CELR1_HUMAN Cadherin EGF LAG seven-pass G-type receptor 1 OS=Homo sapiens GN=CELSR1 I | 460 | DNYPQFsEQNY | 0.186 | 0.006 | -0.991 | -0.266 |
| sp\|Q9NYQ6\|CELR1_HUMAN Cadherin EGF LAG seven-pass G-type receptor 1 OS=Homo sapiens GN=CELSR1 I | 476 | EDVGLNtAVLR | 0.083 | -0.083 | -1.305 | -0.435 |
| sp\|Q9NYQ6\|CELR1_HUMAN Cadherin EGF LAG seven-pass G-type receptor 1 OS=Homo sapiens GN=CELSR1 I | 484 | VLRVQAtDRDQ | 0.526 | 0.26 | -0.424 | 0.121 |
| sp\|Q9NYQ6\|CELR1_HUMAN Cadherin EGF LAG seven-pass G-type receptor 1 OS=Homo sapiens GN=CELSR1 I | 497 | NAAIHYsILSG | 0.083 | -0.052 | -0.976 | -0.315 |
| sp\|Q9NYQ6\|CELR1_HUMAN Cadherin EGF LAG seven-pass G-type receptor 1 OS=Homo sapiens GN=CELSR1 I | 500 | IHYSILsGNVA | 0.084 | -0.182 | -1.023 | -0.374 |
| sp\|Q9NYQ6\|CELR1_HUMAN Cadherin EGF LAG seven-pass G-type receptor 1 OS=Homo sapiens GN=CELSR1 I | 511 | GQFYLHsLSGI | 0.419 | 0.174 | -0.079 | 0.171 |
| sp\|Q9NYQ6\|CELR1_HUMAN Cadherin EGF LAG seven-pass G-type receptor 1 OS=Homo sapiens GN=CELSR1 I | 513 | FYLHSLsGILD | 0.307 | 0.231 | -0.126 | 0.137 |
| sp\|Q9NYQ6\|CELR1_HUMAN Cadherin EGF LAG seven-pass G-type receptor 1 OS=Homo sapiens GN=CELSR1 I | 531 | EDVQKYsLSIK | 0.121 | -0.133 | -0.744 | -0.252 |
| sp\|Q9NYQ6\|CELR1_HUMAN Cadherin EGF LAG seven-pass G-type receptor 1 OS=Homo sapiens GN=CELSR1 I | 533 | VQKYSLsIKAQ | 0.202 | 0.115 | -0.163 | 0.051 |
| sp\|Q9NYQ6\|CELR1_HUMAN Cadherin EGF LAG seven-pass G-type receptor 1 OS=Homo sapiens GN=CELSR1 I | 547 | RPPLINsSGVV | 0.209 | -0.006 | -1.115 | -0.304 |
| sp\|Q9NYQ6\|CELR1_HUMAN Cadherin EGF LAG seven-pass G-type receptor 1 OS=Homo sapiens GN=CELSR1 I | 548 | PPLINSsGVVS | 0.073 | -0.166 | -1.194 | -0.429 |
| sp\|Q9NYQ6\|CELR1_HUMAN Cadherin EGF LAG seven-pass G-type receptor 1 OS=Homo sapiens GN=CELSR1 I | 552 | NSSGVVsVQVL | 0.278 | -0.025 | -0.548 | -0.098 |
| sp\|Q9NYQ6\|CELR1_HUMAN Cadherin EGF LAG seven-pass G-type receptor 1 OS=Homo sapiens GN=CELSR1 I | 567 | NEPIFVsSPFQ | 0.313 | 0.219 | -0.791 | -0.086 |
| sp\|Q9NYQ6\|CELR1_HUMAN Cadherin EGF LAG seven-pass G-type receptor 1 OS=Homo sapiens GN=CELSR1 I | 568 | EPIFVSsPFQA | 0.054 | -0.446 | -1.429 | -0.607 |
| sp\|Q9NYQ6\|CELR1_HUMAN Cadherin EGF LAG seven-pass G-type receptor 1 OS=Homo sapiens GN=CELSR1 I | 573 | SSPFQAtVLEN | 0.093 | -0.144 | -1.513 | -0.521 |
| sp\|Q9NYQ6\|CELR1_HUMAN Cadherin EGF LAG seven-pass G-type receptor 1 OS=Homo sapiens GN=CELSR1 I | 594 | QAVDADsGENA | 0.144 | -0.151 | -1.285 | -0.431 |
| sp\|Q9NYQ6\|CELR1_HUMAN Cadherin EGF LAG seven-pass G-type receptor 1 OS=Homo sapiens GN=CELSR1 I | 607 | HYRLVDtASTF | 0.27 | 0.157 | -0.845 | -0.139 |
| sp\|Q9NYQ6\|CELR1_HUMAN Cadherin EGF LAG seven-pass G-type receptor 1 OS=Homo sapiens GN=CELSR1 I | 609 | RLVDTAsTFLG | 0.334 | 0.192 | -0.593 | -0.022 |
| sp\|Q9NYQ6\|CELR1_HUMAN Cadherin EGF LAG seven-pass G-type receptor 1 OS=Homo sapiens GN=CELSR1 I | 610 | LVDTAStFLGG | 0.15 | -0.083 | -0.863 | -0.265 |
| sp\|Q9NYQ6\|CELR1_HUMAN Cadherin EGF LAG seven-pass G-type receptor 1 OS=Homo sapiens GN=CELSR1 I | 616 | TFLGGGsAGPK | 0.089 | -0.214 | -1.074 | -0.4 |
| sp\|Q9NYQ6\|CELR1_HUMAN Cadherin EGF LAG seven-pass G-type receptor 1 OS=Homo sapiens GN=CELSR1 I | 625 | PKNPAPtPDFP | 0.112 | -0.382 | -1.144 | -0.471 |
| sp\|Q9NYQ6\|CELR1_HUMAN Cadherin EGF LAG seven-pass G-type receptor 1 OS=Homo sapiens GN=CELSR1 I | 635 | PFQIHNsSGWI | 0.571 | 0.275 | -0.02 | 0.275 |
| sp\|Q9NYQ6\|CELR1_HUMAN Cadherin EGF LAG seven-pass G-type receptor 1 OS=Homo sapiens GN=CELSR1 I | 636 | FQIHNSsGWIT | 0.138 | -0.101 | -0.831 | -0.265 |
| sp\|Q9NYQ6\|CELR1_HUMAN Cadherin EGF LAG seven-pass G-type receptor 1 OS=Homo sapiens GN=CELSR1 I | 640 | NSSGWItVCAE | 0.186 | -0.135 | -0.71 | -0.22 |
| sp\|Q9NYQ6\|CELR1_HUMAN Cadherin EGF LAG seven-pass G-type receptor 1 OS=Homo sapiens GN=CELSR1 I | 654 | EEVEHYsFGVE | 0.228 | 0.147 | -0.635 | -0.087 |
| sp\|Q9NYQ6\|CELR1_HUMAN Cadherin EGF LAG seven-pass G-type receptor 1 OS=Homo sapiens GN=CELSR1 I | 664 | EAVDHGsPPMS | 0.143 | -0.003 | -1.159 | -0.34 |
| sp\|Q9NYQ6\|CELR1_HUMAN Cadherin EGF LAG seven-pass G-type receptor 1 OS=Homo sapiens GN=CELSR1 I | 668 | HGSPPMsSSTS | 0.067 | -0.188 | -1.046 | -0.389 |
| sp\|Q9NYQ6\|CELR1_HUMAN Cadherin EGF LAG seven-pass G-type receptor 1 OS=Homo sapiens GN=CELSR1 I | 669 | GSPPMSsSTSV | 0.05 | -0.401 | -1.783 | -0.711 |
| sp\|Q9NYQ6\|CELR1_HUMAN Cadherin EGF LAG seven-pass G-type receptor 1 OS=Homo sapiens GN=CELSR1 I | 670 | SPPMSSsTSVS | 0.051 | -0.173 | -1.511 | -0.544 |
| sp\|Q9NYQ6\|CELR1_HUMAN Cadherin EGF LAG seven-pass G-type receptor 1 OS=Homo sapiens GN=CELSR1 I | 671 | PPMSSStSVSI | 0.127 | 0.047 | -0.817 | -0.214 |
| sp\|Q9NYQ6\|CELR1_HUMAN Cadherin EGF LAG seven-pass G-type receptor 1 OS=Homo sapiens GN=CELSR1 I | 672 | PMSSSTsVSIT | 0.193 | 0.121 | -0.602 | -0.096 |
| sp\|Q9NYQ6\|CELR1_HUMAN Cadherin EGF LAG seven-pass G-type receptor 1 OS=Homo sapiens GN=CELSR1 I | 674 | SSSTSVsITVL | 0.318 | 0.031 | -0.556 | -0.069 |
| sp\|Q9NYQ6\|CELR1_HUMAN Cadherin EGF LAG seven-pass G-type receptor 1 OS=Homo sapiens GN=CELSR1 I | 676 | STSVSItVLDV | 0.255 | 0.09 | -0.899 | -0.185 |
| sp\|Q9NYQ6\|CELR1_HUMAN Cadherin EGF LAG seven-pass G-type receptor 1 OS=Homo sapiens GN=CELSR1 I | 688 | DNDPVFtQPTY | 0.498 | 0.271 | -0.502 | 0.089 |
| sp\|Q9NYQ6\|CELR1_HUMAN Cadherin EGF LAG seven-pass G-type receptor 1 OS=Homo sapiens GN=CELSR1 I | 691 | PVFTQPtYELR | 0.086 | -0.123 | -1.219 | -0.419 |
| sp\|Q9NYQ6\|CELR1_HUMAN Cadherin EGF LAG seven-pass G-type receptor 1 OS=Homo sapiens GN=CELSR1 I | 704 | EDAAVGsSVLT | 0.038 | -0.342 | -1.499 | -0.601 |
| sp\|Q9NYQ6\|CELR1_HUMAN Cadherin EGF LAG seven-pass G-type receptor 1 OS=Homo sapiens GN=CELSR1 I | 705 | DAAVGSsVLTL | 0.044 | -0.263 | -1.524 | -0.581 |
| sp\|Q9NYQ6\|CELR1_HUMAN Cadherin EGF LAG seven-pass G-type receptor 1 OS=Homo sapiens GN=CELSR1 I | 708 | VGSSVLtLQAR | 0.254 | -0.042 | -0.41 | -0.066 |
| sp\|Q9NYQ6\|CELR1_HUMAN Cadherin EGF LAG seven-pass G-type receptor 1 OS=Homo sapiens GN=CELSR1 I | 718 | RDRDANsVITY | 0.569 | 0.324 | -0.255 | 0.213 |
| sp\|Q9NYQ6\|CELR1_HUMAN Cadherin EGF LAG seven-pass G-type receptor 1 OS=Homo sapiens GN=CELSR1 I | 721 | DANSVItYQLT | 0.132 | -0.252 | -1.283 | -0.468 |
| sp\|Q9NYQ6\|CELR1_HUMAN Cadherin EGF LAG seven-pass G-type receptor 1 OS=Homo sapiens GN=CELSR1 I | 725 | VITYQLtGGNT | 0.118 | -0.024 | -0.943 | -0.283 |
| sp\|Q9NYQ6\|CELR1_HUMAN Cadherin EGF LAG seven-pass G-type receptor 1 OS=Homo sapiens GN=CELSR1 I | 729 | QLTGGNtRNRF | 0.164 | -0.124 | -0.863 | -0.274 |
| sp\|Q9NYQ6\|CELR1_HUMAN Cadherin EGF LAG seven-pass G-type receptor 1 OS=Homo sapiens GN=CELSR1 I | 736 | RNRFALsSQRG | 0.404 | 0.13 | -0.541 | -0.002 |
| sp\|Q9NYQ6\|CELR1_HUMAN Cadherin EGF LAG seven-pass G-type receptor 1 OS=Homo sapiens GN=CELSR1 I | 737 | NRFALSsQRGG | 0.133 | -0.094 | -1.173 | -0.378 |
| sp\|Q9NYQ6\|CELR1_HUMAN Cadherin EGF LAG seven-pass G-type receptor 1 OS=Homo sapiens GN=CELSR1 I | 745 | RGGGLItLALP | 0.129 | -0.099 | -0.876 | -0.282 |
| sp\|Q9NYQ6\|CELR1_HUMAN Cadherin EGF LAG seven-pass G-type receptor 1 OS=Homo sapiens GN=CELSR1 I | 763 | QYVLAVtASDG | 0.467 | 0.117 | -0.515 | 0.023 |
| sp\|Q9NYQ6\|CELR1_HUMAN Cadherin EGF LAG seven-pass G-type receptor 1 OS=Homo sapiens GN=CELSR1 I | 765 | VLAVTAsDGTR | 0.32 | 0.131 | -0.444 | 0.002 |
| sp\|Q9NYQ6\|CELR1_HUMAN Cadherin EGF LAG seven-pass G-type receptor 1 OS=Homo sapiens GN=CELSR1 I | 768 | VTASDGtRSHT | 0.038 | -0.54 | -1.762 | -0.755 |

| | | | | | | |
|---|---|---|---|---|---|---|
| sp\|Q9NYQ6\|CELR1_HUMAN Cadherin EGF LAG seven-pass G-type receptor 1 OS=Homo sapiens GN=CELSR1 I | 770 | ASDGTRsHTAH | 0.088 | -0.212 | -1.412 | -0.512 |
| sp\|Q9NYQ6\|CELR1_HUMAN Cadherin EGF LAG seven-pass G-type receptor 1 OS=Homo sapiens GN=CELSR1 I | 772 | DGTRSHtAHVL | 0.889 | 1.306 | 1.038 | 1.078 |
| sp\|Q9NYQ6\|CELR1_HUMAN Cadherin EGF LAG seven-pass G-type receptor 1 OS=Homo sapiens GN=CELSR1 I | 780 | HVLINVtDANT | 0.218 | 0.019 | -0.716 | -0.16 |
| sp\|Q9NYQ6\|CELR1_HUMAN Cadherin EGF LAG seven-pass G-type receptor 1 OS=Homo sapiens GN=CELSR1 I | 784 | NVTDANtHRPV | 0.371 | 0.012 | -0.736 | -0.118 |
| sp\|Q9NYQ6\|CELR1_HUMAN Cadherin EGF LAG seven-pass G-type receptor 1 OS=Homo sapiens GN=CELSR1 I | 791 | HRPVFQsSHYT | 0.083 | 0.047 | -1.216 | -0.362 |
| sp\|Q9NYQ6\|CELR1_HUMAN Cadherin EGF LAG seven-pass G-type receptor 1 OS=Homo sapiens GN=CELSR1 I | 792 | RPVFQSsHYTV | 0.11 | -0.102 | -1.445 | -0.479 |
| sp\|Q9NYQ6\|CELR1_HUMAN Cadherin EGF LAG seven-pass G-type receptor 1 OS=Homo sapiens GN=CELSR1 I | 795 | FQSSHYtVSVS | 0.115 | 0.024 | -0.86 | -0.24 |
| sp\|Q9NYQ6\|CELR1_HUMAN Cadherin EGF LAG seven-pass G-type receptor 1 OS=Homo sapiens GN=CELSR1 I | 797 | SSHYTVsVSED | 0.126 | -0.125 | -1.015 | -0.338 |
| sp\|Q9NYQ6\|CELR1_HUMAN Cadherin EGF LAG seven-pass G-type receptor 1 OS=Homo sapiens GN=CELSR1 I | 799 | HYTVSVsEDRP | 0.493 | 0.262 | -0.056 | 0.233 |
| sp\|Q9NYQ6\|CELR1_HUMAN Cadherin EGF LAG seven-pass G-type receptor 1 OS=Homo sapiens GN=CELSR1 I | 806 | EDRPVGtSIAT | 0.088 | -0.059 | -0.941 | -0.304 |
| sp\|Q9NYQ6\|CELR1_HUMAN Cadherin EGF LAG seven-pass G-type receptor 1 OS=Homo sapiens GN=CELSR1 I | 807 | DRPVGTsIATL | 0.058 | -0.065 | -1.313 | -0.44 |
| sp\|Q9NYQ6\|CELR1_HUMAN Cadherin EGF LAG seven-pass G-type receptor 1 OS=Homo sapiens GN=CELSR1 I | 810 | VGTSIAtLSAN | 0.291 | 0.019 | -0.266 | 0.015 |
| sp\|Q9NYQ6\|CELR1_HUMAN Cadherin EGF LAG seven-pass G-type receptor 1 OS=Homo sapiens GN=CELSR1 I | 812 | TSIATLsANDE | 0.137 | -0.062 | -0.992 | -0.306 |
| sp\|Q9NYQ6\|CELR1_HUMAN Cadherin EGF LAG seven-pass G-type receptor 1 OS=Homo sapiens GN=CELSR1 I | 818 | SANDEDtGENA | 0.065 | -0.324 | -1.952 | -0.737 |
| sp\|Q9NYQ6\|CELR1_HUMAN Cadherin EGF LAG seven-pass G-type receptor 1 OS=Homo sapiens GN=CELSR1 I | 825 | GENARItYVIQ | 0.29 | -0.019 | -0.943 | -0.224 |
| sp\|Q9NYQ6\|CELR1_HUMAN Cadherin EGF LAG seven-pass G-type receptor 1 OS=Homo sapiens GN=CELSR1 I | 841 | FRIDPDsGTMY | 0.216 | 0.044 | -1.063 | -0.268 |
| sp\|Q9NYQ6\|CELR1_HUMAN Cadherin EGF LAG seven-pass G-type receptor 1 OS=Homo sapiens GN=CELSR1 I | 843 | IDPDSGtMYTM | 0.133 | 0.025 | -0.959 | -0.267 |
| sp\|Q9NYQ6\|CELR1_HUMAN Cadherin EGF LAG seven-pass G-type receptor 1 OS=Homo sapiens GN=CELSR1 I | 846 | DSGTMYtMMEL | 0.214 | 0.039 | -0.864 | -0.204 |
| sp\|Q9NYQ6\|CELR1_HUMAN Cadherin EGF LAG seven-pass G-type receptor 1 OS=Homo sapiens GN=CELSR1 I | 859 | ENQVAYtLTIM | 0.362 | 0.114 | -0.191 | 0.095 |
| sp\|Q9NYQ6\|CELR1_HUMAN Cadherin EGF LAG seven-pass G-type receptor 1 OS=Homo sapiens GN=CELSR1 I | 861 | QVAYTLtIMAQ | 0.07 | -0.049 | -0.859 | -0.279 |
| sp\|Q9NYQ6\|CELR1_HUMAN Cadherin EGF LAG seven-pass G-type receptor 1 OS=Homo sapiens GN=CELSR1 I | 873 | NGIPQKsDTTT | 0.189 | -0.089 | -0.825 | -0.242 |
| sp\|Q9NYQ6\|CELR1_HUMAN Cadherin EGF LAG seven-pass G-type receptor 1 OS=Homo sapiens GN=CELSR1 I | 875 | IPQKSDtTTLE | 0.531 | 0.116 | -0.349 | 0.099 |
| sp\|Q9NYQ6\|CELR1_HUMAN Cadherin EGF LAG seven-pass G-type receptor 1 OS=Homo sapiens GN=CELSR1 I | 876 | PQKSDTtTLEI | 0.213 | -0.06 | -0.578 | -0.142 |
| sp\|Q9NYQ6\|CELR1_HUMAN Cadherin EGF LAG seven-pass G-type receptor 1 OS=Homo sapiens GN=CELSR1 I | 877 | QKSDTTtLEIL | 0.39 | 0.16 | -0.257 | 0.098 |
| sp\|Q9NYQ6\|CELR1_HUMAN Cadherin EGF LAG seven-pass G-type receptor 1 OS=Homo sapiens GN=CELSR1 I | 900 | WDFYQGsIFED | 0.09 | -0.19 | -1.013 | -0.371 |
| sp\|Q9NYQ6\|CELR1_HUMAN Cadherin EGF LAG seven-pass G-type receptor 1 OS=Homo sapiens GN=CELSR1 I | 908 | FEDAPPsTSIL | 0.097 | -0.238 | -1.272 | -0.471 |
| sp\|Q9NYQ6\|CELR1_HUMAN Cadherin EGF LAG seven-pass G-type receptor 1 OS=Homo sapiens GN=CELSR1 I | 909 | EDAPPStSILQ | 0.023 | -0.429 | -1.503 | -0.636 |
| sp\|Q9NYQ6\|CELR1_HUMAN Cadherin EGF LAG seven-pass G-type receptor 1 OS=Homo sapiens GN=CELSR1 I | 910 | DAPPSTsILQV | 0.157 | -0.087 | -1.047 | -0.326 |
| sp\|Q9NYQ6\|CELR1_HUMAN Cadherin EGF LAG seven-pass G-type receptor 1 OS=Homo sapiens GN=CELSR1 I | 915 | TSILQVsATDR | 0.318 | -0.015 | -0.851 | -0.183 |
| sp\|Q9NYQ6\|CELR1_HUMAN Cadherin EGF LAG seven-pass G-type receptor 1 OS=Homo sapiens GN=CELSR1 I | 917 | ILQVSAtDRDS | 0.703 | 0.356 | 0.104 | 0.388 |
| sp\|Q9NYQ6\|CELR1_HUMAN Cadherin EGF LAG seven-pass G-type receptor 1 OS=Homo sapiens GN=CELSR1 I | 921 | SATDRDsGPNG | 0.527 | 0.436 | -0.434 | 0.176 |
| sp\|Q9NYQ6\|CELR1_HUMAN Cadherin EGF LAG seven-pass G-type receptor 1 OS=Homo sapiens GN=CELSR1 I | 930 | NGRLLYtFQGG | 0.469 | 0.281 | -0.005 | 0.248 |
| sp\|Q9NYQ6\|CELR1_HUMAN Cadherin EGF LAG seven-pass G-type receptor 1 OS=Homo sapiens GN=CELSR1 I | 946 | DFYIEPtSGVI | 0.281 | -0.02 | -0.731 | -0.157 |
| sp\|Q9NYQ6\|CELR1_HUMAN Cadherin EGF LAG seven-pass G-type receptor 1 OS=Homo sapiens GN=CELSR1 I | 947 | FYIEPTsGVIR | 0.14 | -0.039 | -0.87 | -0.256 |
| sp\|Q9NYQ6\|CELR1_HUMAN Cadherin EGF LAG seven-pass G-type receptor 1 OS=Homo sapiens GN=CELSR1 I | 952 | TSGVIRtQRRL | 0.046 | -0.344 | -1.69 | -0.663 |
| sp\|Q9NYQ6\|CELR1_HUMAN Cadherin EGF LAG seven-pass G-type receptor 1 OS=Homo sapiens GN=CELSR1 I | 975 | LAVDRGsPTPL | 0.096 | -0.337 | -1.403 | -0.548 |
| sp\|Q9NYQ6\|CELR1_HUMAN Cadherin EGF LAG seven-pass G-type receptor 1 OS=Homo sapiens GN=CELSR1 I | 977 | VDRGSPtPLSA | 0.086 | -0.157 | -1.01 | -0.36 |
| sp\|Q9NYQ6\|CELR1_HUMAN Cadherin EGF LAG seven-pass G-type receptor 1 OS=Homo sapiens GN=CELSR1 I | 980 | GSPTPLsASVE | 0.063 | -0.303 | -1.648 | -0.629 |
| sp\|Q9NYQ6\|CELR1_HUMAN Cadherin EGF LAG seven-pass G-type receptor 1 OS=Homo sapiens GN=CELSR1 I | 982 | PTPLSAsVEIQ | 0.364 | 0.114 | -0.638 | -0.053 |
| sp\|Q9NYQ6\|CELR1_HUMAN Cadherin EGF LAG seven-pass G-type receptor 1 OS=Homo sapiens GN=CELSR1 I | 988 | SVEIQVtILDI | 0.156 | -0.052 | -0.874 | -0.257 |
| sp\|Q9NYQ6\|CELR1_HUMAN Cadherin EGF LAG seven-pass G-type receptor 1 OS=Homo sapiens GN=CELSR1 I | 1016 | ENNPVGsVVAK | 0.072 | -0.258 | -1.241 | -0.476 |
| sp\|Q9NYQ6\|CELR1_HUMAN Cadherin EGF LAG seven-pass G-type receptor 1 OS=Homo sapiens GN=CELSR1 I | 1077 | VLVVQAtSAPL | 0.093 | -0.096 | -1.246 | -0.416 |
| sp\|Q9NYQ6\|CELR1_HUMAN Cadherin EGF LAG seven-pass G-type receptor 1 OS=Homo sapiens GN=CELSR1 I | 1078 | LVVQATsAPLV | 0.638 | 0.599 | 0.073 | 0.437 |
| sp\|Q9NYQ6\|CELR1_HUMAN Cadherin EGF LAG seven-pass G-type receptor 1 OS=Homo sapiens GN=CELSR1 I | 1083 | TSAPLVsRATV | 0.094 | -0.392 | -1.435 | -0.578 |
| sp\|Q9NYQ6\|CELR1_HUMAN Cadherin EGF LAG seven-pass G-type receptor 1 OS=Homo sapiens GN=CELSR1 I | 1086 | PLVSRAtVHIL | 0.633 | 0.29 | -0.119 | 0.268 |
| sp\|Q9NYQ6\|CELR1_HUMAN Cadherin EGF LAG seven-pass G-type receptor 1 OS=Homo sapiens GN=CELSR1 I | 1113 | LFNNYVtNKSN | 0.139 | -0.109 | -0.897 | -0.289 |
| sp\|Q9NYQ6\|CELR1_HUMAN Cadherin EGF LAG seven-pass G-type receptor 1 OS=Homo sapiens GN=CELSR1 I | 1116 | NYVTNKsNSFP | 0.107 | -0.193 | -1.174 | -0.42 |
| sp\|Q9NYQ6\|CELR1_HUMAN Cadherin EGF LAG seven-pass G-type receptor 1 OS=Homo sapiens GN=CELSR1 I | 1118 | VTNKSNsFPTG | 0.911 | 0.774 | 0.779 | 0.821 |
| sp\|Q9NYQ6\|CELR1_HUMAN Cadherin EGF LAG seven-pass G-type receptor 1 OS=Homo sapiens GN=CELSR1 I | 1121 | KSNSFPtGVIG | 0.091 | -0.345 | -1.481 | -0.578 |

| | | | | | | |
|---|---|---|---|---|---|---|
| sp\|Q9NYQ6\|CELR1_HUMAN Cadherin EGF LAG seven-pass G-type receptor 1 OS=Homo sapiens GN=CELSR1 I | 1135 | AHDPDVsDSLN | 0.139 | -0.196 | -1.485 | -0.514 |
| sp\|Q9NYQ6\|CELR1_HUMAN Cadherin EGF LAG seven-pass G-type receptor 1 OS=Homo sapiens GN=CELSR1 I | 1137 | DPDVSDsLNYT | 0.333 | 0.03 | -0.612 | -0.083 |
| sp\|Q9NYQ6\|CELR1_HUMAN Cadherin EGF LAG seven-pass G-type receptor 1 OS=Homo sapiens GN=CELSR1 I | 1141 | SDSLNYtFVQG | 0.149 | -0.087 | -0.788 | -0.242 |
| sp\|Q9NYQ6\|CELR1_HUMAN Cadherin EGF LAG seven-pass G-type receptor 1 OS=Homo sapiens GN=CELSR1 I | 1157 | LLLDPAtGELQ | 0.181 | -0.083 | -0.799 | -0.234 |
| sp\|Q9NYQ6\|CELR1_HUMAN Cadherin EGF LAG seven-pass G-type receptor 1 OS=Homo sapiens GN=CELSR1 I | 1163 | TGELQLsRDLD | 0.11 | -0.166 | -1.106 | -0.387 |
| sp\|Q9NYQ6\|CELR1_HUMAN Cadherin EGF LAG seven-pass G-type receptor 1 OS=Homo sapiens GN=CELSR1 I | 1179 | EALMEVsVSDG | 0.244 | -0.157 | -0.792 | -0.235 |
| sp\|Q9NYQ6\|CELR1_HUMAN Cadherin EGF LAG seven-pass G-type receptor 1 OS=Homo sapiens GN=CELSR1 I | 1181 | LMEVSVsDGIH | 0.409 | 0.21 | -0.401 | 0.073 |
| sp\|Q9NYQ6\|CELR1_HUMAN Cadherin EGF LAG seven-pass G-type receptor 1 OS=Homo sapiens GN=CELSR1 I | 1186 | VSDGIHsVTAF | 0.209 | 0.009 | -0.521 | -0.101 |
| sp\|Q9NYQ6\|CELR1_HUMAN Cadherin EGF LAG seven-pass G-type receptor 1 OS=Homo sapiens GN=CELSR1 I | 1188 | DGIHSVtAFCT | 0.49 | 0.213 | -0.258 | 0.148 |
| sp\|Q9NYQ6\|CELR1_HUMAN Cadherin EGF LAG seven-pass G-type receptor 1 OS=Homo sapiens GN=CELSR1 I | 1192 | SVTAFCtLRVT | 0.246 | -0.103 | -0.823 | -0.227 |
| sp\|Q9NYQ6\|CELR1_HUMAN Cadherin EGF LAG seven-pass G-type receptor 1 OS=Homo sapiens GN=CELSR1 I | 1196 | FCTLRVtIITD | 0.545 | 0.22 | -0.078 | 0.229 |
| sp\|Q9NYQ6\|CELR1_HUMAN Cadherin EGF LAG seven-pass G-type receptor 1 OS=Homo sapiens GN=CELSR1 I | 1199 | LRVTIItDDML | 0.306 | 0.211 | -0.466 | 0.017 |
| sp\|Q9NYQ6\|CELR1_HUMAN Cadherin EGF LAG seven-pass G-type receptor 1 OS=Homo sapiens GN=CELSR1 I | 1204 | ITDDMLtNSIT | 0.089 | -0.352 | -1.522 | -0.595 |
| sp\|Q9NYQ6\|CELR1_HUMAN Cadherin EGF LAG seven-pass G-type receptor 1 OS=Homo sapiens GN=CELSR1 I | 1206 | DDMLTNsITVR | 0.141 | -0.043 | -1.116 | -0.339 |
| sp\|Q9NYQ6\|CELR1_HUMAN Cadherin EGF LAG seven-pass G-type receptor 1 OS=Homo sapiens GN=CELSR1 I | 1208 | MLTNSItVRLE | 0.594 | 0.304 | 0.287 | 0.395 |
| sp\|Q9NYQ6\|CELR1_HUMAN Cadherin EGF LAG seven-pass G-type receptor 1 OS=Homo sapiens GN=CELSR1 I | 1215 | VRLENMsQEKF | 0.124 | 0.143 | -0.969 | -0.234 |
| sp\|Q9NYQ6\|CELR1_HUMAN Cadherin EGF LAG seven-pass G-type receptor 1 OS=Homo sapiens GN=CELSR1 I | 1221 | SQEKFLsPLLA | 0.047 | -0.467 | -1.575 | -0.665 |
| sp\|Q9NYQ6\|CELR1_HUMAN Cadherin EGF LAG seven-pass G-type receptor 1 OS=Homo sapiens GN=CELSR1 I | 1236 | GVAAVLsTTKD | 0.181 | -0.11 | -0.926 | -0.285 |
| sp\|Q9NYQ6\|CELR1_HUMAN Cadherin EGF LAG seven-pass G-type receptor 1 OS=Homo sapiens GN=CELSR1 I | 1237 | VAAVLStTKDD | 0.086 | -0.221 | -1.158 | -0.431 |
| sp\|Q9NYQ6\|CELR1_HUMAN Cadherin EGF LAG seven-pass G-type receptor 1 OS=Homo sapiens GN=CELSR1 I | 1238 | AAVLSTtKDDV | 0.47 | 0.195 | -0.365 | 0.1 |
| sp\|Q9NYQ6\|CELR1_HUMAN Cadherin EGF LAG seven-pass G-type receptor 1 OS=Homo sapiens GN=CELSR1 I | 1251 | FNVQNDtDVSS | 0.102 | -0.161 | -1.196 | -0.418 |
| sp\|Q9NYQ6\|CELR1_HUMAN Cadherin EGF LAG seven-pass G-type receptor 1 OS=Homo sapiens GN=CELSR1 I | 1254 | QNDTDVsSNIL | 0.186 | -0.2 | -0.861 | -0.292 |
| sp\|Q9NYQ6\|CELR1_HUMAN Cadherin EGF LAG seven-pass G-type receptor 1 OS=Homo sapiens GN=CELSR1 I | 1255 | NDTDVSsNILN | 0.067 | -0.193 | -1.411 | -0.512 |
| sp\|Q9NYQ6\|CELR1_HUMAN Cadherin EGF LAG seven-pass G-type receptor 1 OS=Homo sapiens GN=CELSR1 I | 1261 | SNILNVtFSAL | 0.23 | -0.026 | -0.632 | -0.143 |
| sp\|Q9NYQ6\|CELR1_HUMAN Cadherin EGF LAG seven-pass G-type receptor 1 OS=Homo sapiens GN=CELSR1 I | 1263 | ILNVTFsALLP | 0.1 | 0.049 | -1.106 | -0.319 |
| sp\|Q9NYQ6\|CELR1_HUMAN Cadherin EGF LAG seven-pass G-type receptor 1 OS=Homo sapiens GN=CELSR1 I | 1277 | RGQFFPsEDLQ | 0.31 | 0.072 | -0.635 | -0.084 |
| sp\|Q9NYQ6\|CELR1_HUMAN Cadherin EGF LAG seven-pass G-type receptor 1 OS=Homo sapiens GN=CELSR1 I | 1289 | QIYLNRtLLTT | 0.152 | -0.008 | -0.814 | -0.223 |
| sp\|Q9NYQ6\|CELR1_HUMAN Cadherin EGF LAG seven-pass G-type receptor 1 OS=Homo sapiens GN=CELSR1 I | 1292 | LNRTLLtTIST | 0.057 | -0.116 | -1.206 | -0.422 |
| sp\|Q9NYQ6\|CELR1_HUMAN Cadherin EGF LAG seven-pass G-type receptor 1 OS=Homo sapiens GN=CELSR1 I | 1293 | NRTLLTtISTQ | 0.086 | -0.031 | -1.144 | -0.363 |
| sp\|Q9NYQ6\|CELR1_HUMAN Cadherin EGF LAG seven-pass G-type receptor 1 OS=Homo sapiens GN=CELSR1 I | 1295 | TLLTTIsTQRV | 0.235 | -0.044 | -0.984 | -0.264 |
| sp\|Q9NYQ6\|CELR1_HUMAN Cadherin EGF LAG seven-pass G-type receptor 1 OS=Homo sapiens GN=CELSR1 I | 1296 | LLTTIStQRVL | 0.172 | -0.072 | -0.785 | -0.228 |
| sp\|Q9NYQ6\|CELR1_HUMAN Cadherin EGF LAG seven-pass G-type receptor 1 OS=Homo sapiens GN=CELSR1 I | 1320 | NYMKCVsVLRF | 0.467 | 0.04 | -0.418 | 0.03 |
| sp\|Q9NYQ6\|CELR1_HUMAN Cadherin EGF LAG seven-pass G-type receptor 1 OS=Homo sapiens GN=CELSR1 I | 1326 | SVLRFDsSAPF | 0.188 | 0.504 | -0.699 | -0.002 |
| sp\|Q9NYQ6\|CELR1_HUMAN Cadherin EGF LAG seven-pass G-type receptor 1 OS=Homo sapiens GN=CELSR1 I | 1327 | VLRFDSsAPFL | 0.671 | 0.699 | 0.164 | 0.511 |
| sp\|Q9NYQ6\|CELR1_HUMAN Cadherin EGF LAG seven-pass G-type receptor 1 OS=Homo sapiens GN=CELSR1 I | 1332 | SSAPFLsSTTV | 0.062 | -0.362 | -1.721 | -0.674 |
| sp\|Q9NYQ6\|CELR1_HUMAN Cadherin EGF LAG seven-pass G-type receptor 1 OS=Homo sapiens GN=CELSR1 I | 1333 | SAPFLSsTTVL | 0.051 | -0.26 | -1.73 | -0.646 |
| sp\|Q9NYQ6\|CELR1_HUMAN Cadherin EGF LAG seven-pass G-type receptor 1 OS=Homo sapiens GN=CELSR1 I | 1334 | APFLSStTVLF | 0.139 | 0.026 | -0.988 | -0.274 |
| sp\|Q9NYQ6\|CELR1_HUMAN Cadherin EGF LAG seven-pass G-type receptor 1 OS=Homo sapiens GN=CELSR1 I | 1335 | PFLSSTtVLFR | 0.259 | 0.097 | -0.351 | 0.002 |
| sp\|Q9NYQ6\|CELR1_HUMAN Cadherin EGF LAG seven-pass G-type receptor 1 OS=Homo sapiens GN=CELSR1 I | 1356 | RCPPGFtGDYC | 0.083 | -0.21 | -1.417 | -0.515 |
| sp\|Q9NYQ6\|CELR1_HUMAN Cadherin EGF LAG seven-pass G-type receptor 1 OS=Homo sapiens GN=CELSR1 I | 1362 | TGDYCEtEIDL | 0.299 | 0.075 | -0.39 | -0.005 |
| sp\|Q9NYQ6\|CELR1_HUMAN Cadherin EGF LAG seven-pass G-type receptor 1 OS=Homo sapiens GN=CELSR1 I | 1369 | EIDLCYsDPCG | 0.696 | 0.692 | 0.204 | 0.531 |
| sp\|Q9NYQ6\|CELR1_HUMAN Cadherin EGF LAG seven-pass G-type receptor 1 OS=Homo sapiens GN=CELSR1 I | 1380 | ANGRCRsREGG | 0.394 | 0.575 | -0.282 | 0.229 |
| sp\|Q9NYQ6\|CELR1_HUMAN Cadherin EGF LAG seven-pass G-type receptor 1 OS=Homo sapiens GN=CELSR1 I | 1386 | SREGGYtCECF | 0.08 | 0.213 | -0.965 | -0.224 |
| sp\|Q9NYQ6\|CELR1_HUMAN Cadherin EGF LAG seven-pass G-type receptor 1 OS=Homo sapiens GN=CELSR1 I | 1394 | ECFEDFtGEHC | 0.058 | -0.204 | -1.472 | -0.539 |
| sp\|Q9NYQ6\|CELR1_HUMAN Cadherin EGF LAG seven-pass G-type receptor 1 OS=Homo sapiens GN=CELSR1 I | 1404 | CEVDARsGRCA | 0.196 | -0.046 | -0.979 | -0.276 |
| sp\|Q9NYQ6\|CELR1_HUMAN Cadherin EGF LAG seven-pass G-type receptor 1 OS=Homo sapiens GN=CELSR1 I | 1417 | VCKNGGtCVNL | 0.112 | 0.017 | -1.126 | -0.332 |
| sp\|Q9NYQ6\|CELR1_HUMAN Cadherin EGF LAG seven-pass G-type receptor 1 OS=Homo sapiens GN=CELSR1 I | 1443 | RPYCEVtTRSF | 0.135 | -0.228 | -1.221 | -0.438 |
| sp\|Q9NYQ6\|CELR1_HUMAN Cadherin EGF LAG seven-pass G-type receptor 1 OS=Homo sapiens GN=CELSR1 I | 1444 | PYCEVTtRSFP | 0.072 | -0.277 | -1.585 | -0.597 |
| sp\|Q9NYQ6\|CELR1_HUMAN Cadherin EGF LAG seven-pass G-type receptor 1 OS=Homo sapiens GN=CELSR1 I | 1446 | CEVTTRsFPPQ | 0.39 | 0.435 | -0.394 | 0.144 |

| | | | | | | |
|---|---|---|---|---|---|---|
| sp\|Q9NYQ6\|CELR1_HUMAN Cadherin EGF LAG seven-pass G-type receptor 1 OS=Homo sapiens GN=CELSR1 I | 1451 | RSFPPQsFVTF | 0.139 | -0.14 | -0.751 | -0.251 |
| sp\|Q9NYQ6\|CELR1_HUMAN Cadherin EGF LAG seven-pass G-type receptor 1 OS=Homo sapiens GN=CELSR1 I | 1454 | PPQSFVtFRGL | 0.411 | -0.053 | -0.438 | -0.027 |
| sp\|Q9NYQ6\|CELR1_HUMAN Cadherin EGF LAG seven-pass G-type receptor 1 OS=Homo sapiens GN=CELSR1 I | 1465 | RQRFHFtISLT | 0.291 | 0.219 | -0.371 | 0.046 |
| sp\|Q9NYQ6\|CELR1_HUMAN Cadherin EGF LAG seven-pass G-type receptor 1 OS=Homo sapiens GN=CELSR1 I | 1467 | RFHFTIsLTFA | 0.297 | 0.031 | -0.484 | -0.052 |
| sp\|Q9NYQ6\|CELR1_HUMAN Cadherin EGF LAG seven-pass G-type receptor 1 OS=Homo sapiens GN=CELSR1 I | 1469 | HFTISLtFATQ | 0.266 | 0.193 | -0.214 | 0.082 |
| sp\|Q9NYQ6\|CELR1_HUMAN Cadherin EGF LAG seven-pass G-type receptor 1 OS=Homo sapiens GN=CELSR1 I | 1472 | ISLTFAtQERN | 0.196 | -0.233 | -1.312 | -0.45 |
| sp\|Q9NYQ6\|CELR1_HUMAN Cadherin EGF LAG seven-pass G-type receptor 1 OS=Homo sapiens GN=CELSR1 I | 1504 | DEQVQLtFSAG | 0.111 | -0.015 | -1.208 | -0.371 |
| sp\|Q9NYQ6\|CELR1_HUMAN Cadherin EGF LAG seven-pass G-type receptor 1 OS=Homo sapiens GN=CELSR1 I | 1506 | QVQLTFsAGET | 0.181 | 0.138 | -0.728 | -0.136 |
| sp\|Q9NYQ6\|CELR1_HUMAN Cadherin EGF LAG seven-pass G-type receptor 1 OS=Homo sapiens GN=CELSR1 I | 1510 | TFSAGEtTTTV | 0.109 | -0.282 | -1.411 | -0.528 |
| sp\|Q9NYQ6\|CELR1_HUMAN Cadherin EGF LAG seven-pass G-type receptor 1 OS=Homo sapiens GN=CELSR1 I | 1511 | FSAGETtTTVA | 0.184 | -0.091 | -0.816 | -0.241 |
| sp\|Q9NYQ6\|CELR1_HUMAN Cadherin EGF LAG seven-pass G-type receptor 1 OS=Homo sapiens GN=CELSR1 I | 1512 | SAGETTtTVAP | 0.036 | -0.175 | -1.601 | -0.58 |
| sp\|Q9NYQ6\|CELR1_HUMAN Cadherin EGF LAG seven-pass G-type receptor 1 OS=Homo sapiens GN=CELSR1 I | 1513 | AGETTTtVAPK | 0.098 | -0.246 | -1.08 | -0.409 |
| sp\|Q9NYQ6\|CELR1_HUMAN Cadherin EGF LAG seven-pass G-type receptor 1 OS=Homo sapiens GN=CELSR1 I | 1520 | VAPKVPsGVSD | 0.115 | -0.269 | -1.219 | -0.458 |
| sp\|Q9NYQ6\|CELR1_HUMAN Cadherin EGF LAG seven-pass G-type receptor 1 OS=Homo sapiens GN=CELSR1 I | 1523 | KVPSGVsDGRW | 0.138 | -0.221 | -1.295 | -0.459 |
| sp\|Q9NYQ6\|CELR1_HUMAN Cadherin EGF LAG seven-pass G-type receptor 1 OS=Homo sapiens GN=CELSR1 I | 1529 | SDGRWHsVQVQ | 0.46 | 0.851 | -0.05 | 0.42 |
| sp\|Q9NYQ6\|CELR1_HUMAN Cadherin EGF LAG seven-pass G-type receptor 1 OS=Homo sapiens GN=CELSR1 I | 1550 | GLPHGPsGEKM | 0.089 | -0.217 | -1.183 | -0.437 |
| sp\|Q9NYQ6\|CELR1_HUMAN Cadherin EGF LAG seven-pass G-type receptor 1 OS=Homo sapiens GN=CELSR1 I | 1558 | EKMAVVtVDDC | 0.514 | 0.084 | -0.256 | 0.114 |
| sp\|Q9NYQ6\|CELR1_HUMAN Cadherin EGF LAG seven-pass G-type receptor 1 OS=Homo sapiens GN=CELSR1 I | 1564 | TVDDCDtTMAV | 0.2 | -0.082 | -1.193 | -0.358 |
| sp\|Q9NYQ6\|CELR1_HUMAN Cadherin EGF LAG seven-pass G-type receptor 1 OS=Homo sapiens GN=CELSR1 I | 1565 | VDDCDTtMAVR | 0.092 | -0.159 | -1.32 | -0.462 |
| sp\|Q9NYQ6\|CELR1_HUMAN Cadherin EGF LAG seven-pass G-type receptor 1 OS=Homo sapiens GN=CELSR1 I | 1578 | KDIGNYsCAAQ | 0.103 | -0.02 | -0.886 | -0.268 |
| sp\|Q9NYQ6\|CELR1_HUMAN Cadherin EGF LAG seven-pass G-type receptor 1 OS=Homo sapiens GN=CELSR1 I | 1584 | SCAAQGtQTGS | 0.073 | -0.318 | -1.75 | -0.665 |
| sp\|Q9NYQ6\|CELR1_HUMAN Cadherin EGF LAG seven-pass G-type receptor 1 OS=Homo sapiens GN=CELSR1 I | 1586 | AAQGTQtGSKK | 0.051 | -0.22 | -1.376 | -0.515 |
| sp\|Q9NYQ6\|CELR1_HUMAN Cadherin EGF LAG seven-pass G-type receptor 1 OS=Homo sapiens GN=CELSR1 I | 1588 | QGTQTGsKKSL | 0.069 | -0.147 | -0.946 | -0.341 |
| sp\|Q9NYQ6\|CELR1_HUMAN Cadherin EGF LAG seven-pass G-type receptor 1 OS=Homo sapiens GN=CELSR1 I | 1591 | QTGSKKsLDLT | 0.224 | -0.045 | -0.498 | -0.106 |
| sp\|Q9NYQ6\|CELR1_HUMAN Cadherin EGF LAG seven-pass G-type receptor 1 OS=Homo sapiens GN=CELSR1 I | 1595 | KKSLDLtGPLL | 0.436 | 0.396 | -0.186 | 0.215 |
| sp\|Q9NYQ6\|CELR1_HUMAN Cadherin EGF LAG seven-pass G-type receptor 1 OS=Homo sapiens GN=CELSR1 I | 1625 | GCMRNLsVDGK | 0.722 | 0.894 | 0.529 | 0.715 |
| sp\|Q9NYQ6\|CELR1_HUMAN Cadherin EGF LAG seven-pass G-type receptor 1 OS=Homo sapiens GN=CELSR1 I | 1642 | FIANNGtREGC | 0.099 | -0.183 | -1.066 | -0.383 |
| sp\|Q9NYQ6\|CELR1_HUMAN Cadherin EGF LAG seven-pass G-type receptor 1 OS=Homo sapiens GN=CELSR1 I | 1663 | RCQNGGtCVNR | 0.141 | 0.082 | -1.146 | -0.308 |
| sp\|Q9NYQ6\|CELR1_HUMAN Cadherin EGF LAG seven-pass G-type receptor 1 OS=Homo sapiens GN=CELSR1 I | 1695 | PHPQLFsGESV | 0.054 | -0.194 | -1.686 | -0.609 |
| sp\|Q9NYQ6\|CELR1_HUMAN Cadherin EGF LAG seven-pass G-type receptor 1 OS=Homo sapiens GN=CELSR1 I | 1698 | QLFSGEsVVSW | 0.062 | -0.135 | -1.371 | -0.481 |
| sp\|Q9NYQ6\|CELR1_HUMAN Cadherin EGF LAG seven-pass G-type receptor 1 OS=Homo sapiens GN=CELSR1 I | 1701 | SGESVVsWSDL | 0.205 | -0.064 | -0.842 | -0.234 |
| sp\|Q9NYQ6\|CELR1_HUMAN Cadherin EGF LAG seven-pass G-type receptor 1 OS=Homo sapiens GN=CELSR1 I | 1703 | ESVVSWsDLNI | 0.245 | 0.146 | -0.711 | -0.107 |
| sp\|Q9NYQ6\|CELR1_HUMAN Cadherin EGF LAG seven-pass G-type receptor 1 OS=Homo sapiens GN=CELSR1 I | 1710 | DLNIIIsVPWY | 0.758 | 0.617 | 0.337 | 0.571 |
| sp\|Q9NYQ6\|CELR1_HUMAN Cadherin EGF LAG seven-pass G-type receptor 1 OS=Homo sapiens GN=CELSR1 I | 1721 | LGLMFRtRKED | 0.147 | -0.207 | -1.004 | -0.355 |
| sp\|Q9NYQ6\|CELR1_HUMAN Cadherin EGF LAG seven-pass G-type receptor 1 OS=Homo sapiens GN=CELSR1 I | 1726 | RTRKEDsVLME | 0.417 | 0.054 | -0.882 | -0.137 |
| sp\|Q9NYQ6\|CELR1_HUMAN Cadherin EGF LAG seven-pass G-type receptor 1 OS=Homo sapiens GN=CELSR1 I | 1732 | SVLMEAtSGGP | 0.11 | -0.243 | -1.214 | -0.449 |
| sp\|Q9NYQ6\|CELR1_HUMAN Cadherin EGF LAG seven-pass G-type receptor 1 OS=Homo sapiens GN=CELSR1 I | 1733 | VLMEATsGGPT | 0.203 | 0.006 | -0.551 | -0.114 |
| sp\|Q9NYQ6\|CELR1_HUMAN Cadherin EGF LAG seven-pass G-type receptor 1 OS=Homo sapiens GN=CELSR1 I | 1737 | ATSGGPtSFRL | 0.084 | -0.325 | -1.407 | -0.549 |
| sp\|Q9NYQ6\|CELR1_HUMAN Cadherin EGF LAG seven-pass G-type receptor 1 OS=Homo sapiens GN=CELSR1 I | 1738 | TSGGPTsFRLQ | 0.099 | -0.256 | -0.935 | -0.364 |
| sp\|Q9NYQ6\|CELR1_HUMAN Cadherin EGF LAG seven-pass G-type receptor 1 OS=Homo sapiens GN=CELSR1 I | 1753 | YLQFEVsHGPS | 0.52 | 0.099 | -0.316 | 0.101 |
| sp\|Q9NYQ6\|CELR1_HUMAN Cadherin EGF LAG seven-pass G-type receptor 1 OS=Homo sapiens GN=CELSR1 I | 1757 | EVSHGPsDVES | 0.065 | -0.204 | -1.338 | -0.492 |
| sp\|Q9NYQ6\|CELR1_HUMAN Cadherin EGF LAG seven-pass G-type receptor 1 OS=Homo sapiens GN=CELSR1 I | 1761 | GPSDVEsVMLS | 0.163 | -0.102 | -1.301 | -0.413 |
| sp\|Q9NYQ6\|CELR1_HUMAN Cadherin EGF LAG seven-pass G-type receptor 1 OS=Homo sapiens GN=CELSR1 I | 1765 | VESVMLsGLRV | 0.038 | -0.418 | -1.969 | -0.783 |
| sp\|Q9NYQ6\|CELR1_HUMAN Cadherin EGF LAG seven-pass G-type receptor 1 OS=Homo sapiens GN=CELSR1 I | 1770 | LSGLRVtDGEW | 0.476 | 0.153 | -0.382 | 0.082 |
| sp\|Q9NYQ6\|CELR1_HUMAN Cadherin EGF LAG seven-pass G-type receptor 1 OS=Homo sapiens GN=CELSR1 I | 1788 | KNVKEDsEMKH | 0.115 | -0.291 | -1.524 | -0.567 |
| sp\|Q9NYQ6\|CELR1_HUMAN Cadherin EGF LAG seven-pass G-type receptor 1 OS=Homo sapiens GN=CELSR1 I | 1795 | EMKHLVtMTLD | 0.275 | 0.164 | -0.607 | -0.056 |
| sp\|Q9NYQ6\|CELR1_HUMAN Cadherin EGF LAG seven-pass G-type receptor 1 OS=Homo sapiens GN=CELSR1 I | 1797 | KHLVTMtLDYG | 0.248 | 0.162 | -0.693 | -0.094 |
| sp\|Q9NYQ6\|CELR1_HUMAN Cadherin EGF LAG seven-pass G-type receptor 1 OS=Homo sapiens GN=CELSR1 I | 1817 | GMLPGLtVRSV | 0.049 | -0.15 | -1.509 | -0.537 |
| sp\|Q9NYQ6\|CELR1_HUMAN Cadherin EGF LAG seven-pass G-type receptor 1 OS=Homo sapiens GN=CELSR1 I | 1820 | PGLTVRsVVVG | 0.167 | -0.028 | -0.962 | -0.274 |

| | | | | | | |
|---|---|---|---|---|---|---|
| sp\|Q9NYQ6\|CELR1_HUMAN Cadherin EGF LAG seven-pass G-type receptor 1 OS=Homo sapiens GN=CELSR1 I | 1827 | VVVGGAsEDKV | 0.115 | -0.016 | -1.115 | -0.339 |
| sp\|Q9NYQ6\|CELR1_HUMAN Cadherin EGF LAG seven-pass G-type receptor 1 OS=Homo sapiens GN=CELSR1 I | 1832 | ASEDKVsVRRG | 0.239 | -0.299 | -1.036 | -0.365 |
| sp\|Q9NYQ6\|CELR1_HUMAN Cadherin EGF LAG seven-pass G-type receptor 1 OS=Homo sapiens GN=CELSR1 I | 1849 | GVRMGGtPTNV | 0.126 | -0.197 | -1.495 | -0.522 |
| sp\|Q9NYQ6\|CELR1_HUMAN Cadherin EGF LAG seven-pass G-type receptor 1 OS=Homo sapiens GN=CELSR1 I | 1851 | RMGGTPtNVAT | 0.063 | 0.027 | -1.201 | -0.37 |
| sp\|Q9NYQ6\|CELR1_HUMAN Cadherin EGF LAG seven-pass G-type receptor 1 OS=Homo sapiens GN=CELSR1 I | 1855 | TPTNVAtLNMN | 0.319 | 0.008 | -0.411 | -0.028 |
| sp\|Q9NYQ6\|CELR1_HUMAN Cadherin EGF LAG seven-pass G-type receptor 1 OS=Homo sapiens GN=CELSR1 I | 1877 | DVDDPCtSSPC | 0.068 | -0.372 | -1.868 | -0.724 |
| sp\|Q9NYQ6\|CELR1_HUMAN Cadherin EGF LAG seven-pass G-type receptor 1 OS=Homo sapiens GN=CELSR1 I | 1878 | VDDPCTsSPCP | 0.257 | 0.217 | -0.568 | -0.031 |
| sp\|Q9NYQ6\|CELR1_HUMAN Cadherin EGF LAG seven-pass G-type receptor 1 OS=Homo sapiens GN=CELSR1 I | 1879 | DDPCTSsPCPP | 0.015 | -0.76 | -2.433 | -1.059 |
| sp\|Q9NYQ6\|CELR1_HUMAN Cadherin EGF LAG seven-pass G-type receptor 1 OS=Homo sapiens GN=CELSR1 I | 1885 | SPCPPNsRCHD | 0.068 | -0.433 | -1.524 | -0.63 |
| sp\|Q9NYQ6\|CELR1_HUMAN Cadherin EGF LAG seven-pass G-type receptor 1 OS=Homo sapiens GN=CELSR1 I | 1895 | DAWEDYsCVCD | 0.137 | 0.186 | -0.972 | -0.216 |
| sp\|Q9NYQ6\|CELR1_HUMAN Cadherin EGF LAG seven-pass G-type receptor 1 OS=Homo sapiens GN=CELSR1 I | 1925 | MGACVRsPGSP | 0.073 | -0.354 | -1.393 | -0.558 |
| sp\|Q9NYQ6\|CELR1_HUMAN Cadherin EGF LAG seven-pass G-type receptor 1 OS=Homo sapiens GN=CELSR1 I | 1928 | CVRSPGsPQGY | 0.144 | -0.254 | -0.901 | -0.337 |
| sp\|Q9NYQ6\|CELR1_HUMAN Cadherin EGF LAG seven-pass G-type receptor 1 OS=Homo sapiens GN=CELSR1 I | 1939 | VCECGPsHYGP | 0.138 | -0.268 | -1.159 | -0.43 |
| sp\|Q9NYQ6\|CELR1_HUMAN Cadherin EGF LAG seven-pass G-type receptor 1 OS=Homo sapiens GN=CELSR1 I | 1971 | PCHCAVsKGFD | 0.548 | 0.07 | -0.329 | 0.096 |
| sp\|Q9NYQ6\|CELR1_HUMAN Cadherin EGF LAG seven-pass G-type receptor 1 OS=Homo sapiens GN=CELSR1 I | 1981 | DPDCNKtNGQC | 0.339 | -0.157 | -0.905 | -0.241 |
| sp\|Q9NYQ6\|CELR1_HUMAN Cadherin EGF LAG seven-pass G-type receptor 1 OS=Homo sapiens GN=CELSR1 I | 1999 | KLLAQDtCLPC | 0.09 | -0.186 | -1.529 | -0.542 |
| sp\|Q9NYQ6\|CELR1_HUMAN Cadherin EGF LAG seven-pass G-type receptor 1 OS=Homo sapiens GN=CELSR1 I | 2010 | DCFPHGsHSRT | 0.155 | -0.246 | -1.451 | -0.514 |
| sp\|Q9NYQ6\|CELR1_HUMAN Cadherin EGF LAG seven-pass G-type receptor 1 OS=Homo sapiens GN=CELSR1 I | 2012 | FPHGSHsRTCD | 0.397 | 0.285 | -0.419 | 0.088 |
| sp\|Q9NYQ6\|CELR1_HUMAN Cadherin EGF LAG seven-pass G-type receptor 1 OS=Homo sapiens GN=CELSR1 I | 2014 | HGSHSRtCDMA | 0.283 | 0.19 | -0.353 | 0.04 |
| sp\|Q9NYQ6\|CELR1_HUMAN Cadherin EGF LAG seven-pass G-type receptor 1 OS=Homo sapiens GN=CELSR1 I | 2019 | RTCDMAtGQCA | 0.339 | -0.037 | -0.776 | -0.158 |
| sp\|Q9NYQ6\|CELR1_HUMAN Cadherin EGF LAG seven-pass G-type receptor 1 OS=Homo sapiens GN=CELSR1 I | 2044 | NPFAEVtTLGC | 0.14 | -0.289 | -1.358 | -0.502 |
| sp\|Q9NYQ6\|CELR1_HUMAN Cadherin EGF LAG seven-pass G-type receptor 1 OS=Homo sapiens GN=CELSR1 I | 2045 | PFAEVTtLGCE | 0.416 | 0.143 | -0.151 | 0.136 |
| sp\|Q9NYQ6\|CELR1_HUMAN Cadherin EGF LAG seven-pass G-type receptor 1 OS=Homo sapiens GN=CELSR1 I | 2068 | GIWWPQtKFGQ | 0.248 | 0.017 | -0.412 | -0.049 |
| sp\|Q9NYQ6\|CELR1_HUMAN Cadherin EGF LAG seven-pass G-type receptor 1 OS=Homo sapiens GN=CELSR1 I | 2082 | VPCPKGsVGNA | 0.102 | -0.213 | -1.283 | -0.465 |
| sp\|Q9NYQ6\|CELR1_HUMAN Cadherin EGF LAG seven-pass G-type receptor 1 OS=Homo sapiens GN=CELSR1 I | 2091 | NAVRHCsGEKG | 0.584 | 0.781 | -0.058 | 0.436 |
| sp\|Q9NYQ6\|CELR1_HUMAN Cadherin EGF LAG seven-pass G-type receptor 1 OS=Homo sapiens GN=CELSR1 I | 2105 | PELFNCtTISF | 0.136 | -0.087 | -0.981 | -0.311 |
| sp\|Q9NYQ6\|CELR1_HUMAN Cadherin EGF LAG seven-pass G-type receptor 1 OS=Homo sapiens GN=CELSR1 I | 2106 | ELFNCTtISFV | 0.123 | -0.094 | -0.965 | -0.312 |
| sp\|Q9NYQ6\|CELR1_HUMAN Cadherin EGF LAG seven-pass G-type receptor 1 OS=Homo sapiens GN=CELSR1 I | 2108 | FNCTTIsFVDL | 0.328 | 0.152 | -0.414 | 0.022 |
| sp\|Q9NYQ6\|CELR1_HUMAN Cadherin EGF LAG seven-pass G-type receptor 1 OS=Homo sapiens GN=CELSR1 I | 2120 | AMNEKLsRNET | 0.041 | -0.196 | -1.636 | -0.597 |
| sp\|Q9NYQ6\|CELR1_HUMAN Cadherin EGF LAG seven-pass G-type receptor 1 OS=Homo sapiens GN=CELSR1 I | 2124 | KLSRNEtQVDG | 0.732 | 0.84 | 0.225 | 0.599 |
| sp\|Q9NYQ6\|CELR1_HUMAN Cadherin EGF LAG seven-pass G-type receptor 1 OS=Homo sapiens GN=CELSR1 I | 2140 | LVRALRsATQH | 0.179 | -0.006 | -1.157 | -0.328 |
| sp\|Q9NYQ6\|CELR1_HUMAN Cadherin EGF LAG seven-pass G-type receptor 1 OS=Homo sapiens GN=CELSR1 I | 2142 | RALRSAtQHTG | 0.849 | 1.038 | 0.613 | 0.833 |
| sp\|Q9NYQ6\|CELR1_HUMAN Cadherin EGF LAG seven-pass G-type receptor 1 OS=Homo sapiens GN=CELSR1 I | 2145 | RSATQHtGTLF | 0.17 | -0.038 | -0.933 | -0.267 |
| sp\|Q9NYQ6\|CELR1_HUMAN Cadherin EGF LAG seven-pass G-type receptor 1 OS=Homo sapiens GN=CELSR1 I | 2147 | ATQHTGtLFGN | 0.229 | -0.028 | -0.705 | -0.168 |
| sp\|Q9NYQ6\|CELR1_HUMAN Cadherin EGF LAG seven-pass G-type receptor 1 OS=Homo sapiens GN=CELSR1 I | 2155 | FGNDVRtAYQL | 0.183 | 0.062 | -1.084 | -0.28 |
| sp\|Q9NYQ6\|CELR1_HUMAN Cadherin EGF LAG seven-pass G-type receptor 1 OS=Homo sapiens GN=CELSR1 I | 2168 | HVLQHEsWQQG | 0.276 | 0.116 | -0.43 | -0.013 |
| sp\|Q9NYQ6\|CELR1_HUMAN Cadherin EGF LAG seven-pass G-type receptor 1 OS=Homo sapiens GN=CELSR1 I | 2178 | GFDLAAtQDAD | 0.581 | 0.196 | -0.218 | 0.186 |
| sp\|Q9NYQ6\|CELR1_HUMAN Cadherin EGF LAG seven-pass G-type receptor 1 OS=Homo sapiens GN=CELSR1 I | 2190 | HEDVIHsGSAL | 0.106 | 0.066 | -0.805 | -0.211 |
| sp\|Q9NYQ6\|CELR1_HUMAN Cadherin EGF LAG seven-pass G-type receptor 1 OS=Homo sapiens GN=CELSR1 I | 2192 | DVIHSGsALLA | 0.189 | 0.099 | -0.919 | -0.21 |
| sp\|Q9NYQ6\|CELR1_HUMAN Cadherin EGF LAG seven-pass G-type receptor 1 OS=Homo sapiens GN=CELSR1 I | 2199 | ALLAPAtRAAW | 0.217 | -0.115 | -0.724 | -0.207 |
| sp\|Q9NYQ6\|CELR1_HUMAN Cadherin EGF LAG seven-pass G-type receptor 1 OS=Homo sapiens GN=CELSR1 I | 2209 | WEQIQRsEGGT | 0.127 | -0.02 | -1.016 | -0.303 |
| sp\|Q9NYQ6\|CELR1_HUMAN Cadherin EGF LAG seven-pass G-type receptor 1 OS=Homo sapiens GN=CELSR1 I | 2213 | QRSEGGtAQLL | 0.07 | 0.075 | -1.235 | -0.363 |
| sp\|Q9NYQ6\|CELR1_HUMAN Cadherin EGF LAG seven-pass G-type receptor 1 OS=Homo sapiens GN=CELSR1 I | 2225 | RLEGYFsNVAR | 0.409 | 0.182 | -0.26 | 0.11 |
| sp\|Q9NYQ6\|CELR1_HUMAN Cadherin EGF LAG seven-pass G-type receptor 1 OS=Homo sapiens GN=CELSR1 I | 2234 | ARNVRRtYLRP | 0.08 | 0.026 | -1.585 | -0.493 |
| sp\|Q9NYQ6\|CELR1_HUMAN Cadherin EGF LAG seven-pass G-type receptor 1 OS=Homo sapiens GN=CELSR1 I | 2243 | RPFVIVtANMI | 0.343 | 0.031 | -0.489 | -0.038 |
| sp\|Q9NYQ6\|CELR1_HUMAN Cadherin EGF LAG seven-pass G-type receptor 1 OS=Homo sapiens GN=CELSR1 I | 2259 | FDKFNFtGARV | 0.188 | -0.125 | -0.993 | -0.31 |
| sp\|Q9NYQ6\|CELR1_HUMAN Cadherin EGF LAG seven-pass G-type receptor 1 OS=Homo sapiens GN=CELSR1 I | 2268 | RVPRFDtIHEE | 0.484 | 0.685 | -0.262 | 0.302 |
| sp\|Q9NYQ6\|CELR1_HUMAN Cadherin EGF LAG seven-pass G-type receptor 1 OS=Homo sapiens GN=CELSR1 I | 2279 | FPRELEsSVSF | 0.061 | 0.02 | -1.485 | -0.468 |
| sp\|Q9NYQ6\|CELR1_HUMAN Cadherin EGF LAG seven-pass G-type receptor 1 OS=Homo sapiens GN=CELSR1 I | 2280 | PRELESsVSFP | 0.059 | -0.104 | -1.467 | -0.504 |

| | | | | | | | |
|---|---|---|---|---|---|---|---|
| sp\|Q9NYQ6\|CELR1_HUMAN Cadherin EGF LAG seven-pass G-type receptor 1 OS=Homo sapiens GN=CELSR1 I | 2282 | ELESSVsFPAD | 0.923 | 0.878 | 1.403 | 1.068 |
| sp\|Q9NYQ6\|CELR1_HUMAN Cadherin EGF LAG seven-pass G-type receptor 1 OS=Homo sapiens GN=CELSR1 I | 2306 | RPAGRRtTPQT | 0.536 | 0.504 | 0.011 | 0.35 |
| sp\|Q9NYQ6\|CELR1_HUMAN Cadherin EGF LAG seven-pass G-type receptor 1 OS=Homo sapiens GN=CELSR1 I | 2307 | PAGRRTtPQTT | 0.449 | 0.595 | 0.046 | 0.363 |
| sp\|Q9NYQ6\|CELR1_HUMAN Cadherin EGF LAG seven-pass G-type receptor 1 OS=Homo sapiens GN=CELSR1 I | 2310 | RRTTPQtTRPG | 0.206 | 0.017 | -0.644 | -0.14 |
| sp\|Q9NYQ6\|CELR1_HUMAN Cadherin EGF LAG seven-pass G-type receptor 1 OS=Homo sapiens GN=CELSR1 I | 2311 | RTTPQTtRPGP | 0.332 | 0.228 | -0.466 | 0.031 |
| sp\|Q9NYQ6\|CELR1_HUMAN Cadherin EGF LAG seven-pass G-type receptor 1 OS=Homo sapiens GN=CELSR1 I | 2317 | TRPGPGtEREA | 0.112 | -0.058 | -0.934 | -0.293 |
| sp\|Q9NYQ6\|CELR1_HUMAN Cadherin EGF LAG seven-pass G-type receptor 1 OS=Homo sapiens GN=CELSR1 I | 2324 | EREAPIsRRRR | 0.183 | -0.173 | -0.854 | -0.281 |
| sp\|Q9NYQ6\|CELR1_HUMAN Cadherin EGF LAG seven-pass G-type receptor 1 OS=Homo sapiens GN=CELSR1 I | 2346 | LVIIYRtLGQL | 0.309 | 0.163 | -0.372 | 0.033 |
| sp\|Q9NYQ6\|CELR1_HUMAN Cadherin EGF LAG seven-pass G-type receptor 1 OS=Homo sapiens GN=CELSR1 I | 2361 | YDPDRRsLRLP | 0.163 | -0.045 | -0.94 | -0.274 |
| sp\|Q9NYQ6\|CELR1_HUMAN Cadherin EGF LAG seven-pass G-type receptor 1 OS=Homo sapiens GN=CELSR1 I | 2372 | HRPIINtPMVS | 0.084 | -0.031 | -1.133 | -0.36 |
| sp\|Q9NYQ6\|CELR1_HUMAN Cadherin EGF LAG seven-pass G-type receptor 1 OS=Homo sapiens GN=CELSR1 I | 2376 | INTPMVsTLVY | 0.236 | -0.204 | -0.733 | -0.234 |
| sp\|Q9NYQ6\|CELR1_HUMAN Cadherin EGF LAG seven-pass G-type receptor 1 OS=Homo sapiens GN=CELSR1 I | 2377 | NTPMVStLVYS | 0.132 | -0.201 | -1.185 | -0.418 |
| sp\|Q9NYQ6\|CELR1_HUMAN Cadherin EGF LAG seven-pass G-type receptor 1 OS=Homo sapiens GN=CELSR1 I | 2381 | VSTLVYsEGAP | 0.241 | 0.08 | -0.408 | -0.029 |
| sp\|Q9NYQ6\|CELR1_HUMAN Cadherin EGF LAG seven-pass G-type receptor 1 OS=Homo sapiens GN=CELSR1 I | 2407 | LEVEERtKPVC | 0.187 | 0.293 | -1.024 | -0.181 |
| sp\|Q9NYQ6\|CELR1_HUMAN Cadherin EGF LAG seven-pass G-type receptor 1 OS=Homo sapiens GN=CELSR1 I | 2417 | CVFWNHsLAVG | 0.552 | 0.205 | 0.071 | 0.276 |
| sp\|Q9NYQ6\|CELR1_HUMAN Cadherin EGF LAG seven-pass G-type receptor 1 OS=Homo sapiens GN=CELSR1 I | 2423 | SLAVGGtGGWS | 0.055 | -0.191 | -1.269 | -0.468 |
| sp\|Q9NYQ6\|CELR1_HUMAN Cadherin EGF LAG seven-pass G-type receptor 1 OS=Homo sapiens GN=CELSR1 I | 2427 | GGTGGWsARGC | 0.138 | -0.253 | -1.072 | -0.396 |
| sp\|Q9NYQ6\|CELR1_HUMAN Cadherin EGF LAG seven-pass G-type receptor 1 OS=Homo sapiens GN=CELSR1 I | 2435 | RGCELLsRNRT | 0.066 | -0.306 | -1.531 | -0.59 |
| sp\|Q9NYQ6\|CELR1_HUMAN Cadherin EGF LAG seven-pass G-type receptor 1 OS=Homo sapiens GN=CELSR1 I | 2439 | LLSRNRtHVAC | 0.671 | 0.893 | 0.35 | 0.638 |
| sp\|Q9NYQ6\|CELR1_HUMAN Cadherin EGF LAG seven-pass G-type receptor 1 OS=Homo sapiens GN=CELSR1 I | 2446 | HVACQCsHTAS | 0.287 | 0.066 | -0.717 | -0.121 |
| sp\|Q9NYQ6\|CELR1_HUMAN Cadherin EGF LAG seven-pass G-type receptor 1 OS=Homo sapiens GN=CELSR1 I | 2448 | ACQCSHtASFA | 0.465 | 0.256 | -0.6 | 0.04 |
| sp\|Q9NYQ6\|CELR1_HUMAN Cadherin EGF LAG seven-pass G-type receptor 1 OS=Homo sapiens GN=CELSR1 I | 2450 | QCSHTAsFAVL | 0.233 | 0.095 | -0.695 | -0.122 |
| sp\|Q9NYQ6\|CELR1_HUMAN Cadherin EGF LAG seven-pass G-type receptor 1 OS=Homo sapiens GN=CELSR1 I | 2458 | AVLMDIsRREN | 0.18 | -0.262 | -1.025 | -0.369 |
| sp\|Q9NYQ6\|CELR1_HUMAN Cadherin EGF LAG seven-pass G-type receptor 1 OS=Homo sapiens GN=CELSR1 I | 2472 | LPLKIVtYAAV | 0.309 | -0.051 | -0.511 | -0.084 |
| sp\|Q9NYQ6\|CELR1_HUMAN Cadherin EGF LAG seven-pass G-type receptor 1 OS=Homo sapiens GN=CELSR1 I | 2477 | VTYAAVsLSLA | 0.428 | -0.02 | -0.312 | 0.032 |
| sp\|Q9NYQ6\|CELR1_HUMAN Cadherin EGF LAG seven-pass G-type receptor 1 OS=Homo sapiens GN=CELSR1 I | 2479 | YAAVSLsLAAL | 0.277 | 0.225 | -0.074 | 0.143 |
| sp\|Q9NYQ6\|CELR1_HUMAN Cadherin EGF LAG seven-pass G-type receptor 1 OS=Homo sapiens GN=CELSR1 I | 2491 | VAFVLLsLVRM | 0.084 | -0.218 | -1.287 | -0.474 |
| sp\|Q9NYQ6\|CELR1_HUMAN Cadherin EGF LAG seven-pass G-type receptor 1 OS=Homo sapiens GN=CELSR1 I | 2498 | LVRMLRsNLHS | 0.076 | -0.057 | -1.383 | -0.455 |
| sp\|Q9NYQ6\|CELR1_HUMAN Cadherin EGF LAG seven-pass G-type receptor 1 OS=Homo sapiens GN=CELSR1 I | 2502 | LRSNLHsIHKH | 0.101 | 0.054 | -1.056 | -0.3 |
| sp\|Q9NYQ6\|CELR1_HUMAN Cadherin EGF LAG seven-pass G-type receptor 1 OS=Homo sapiens GN=CELSR1 I | 2514 | AVALFLsQLVF | 0.047 | -0.217 | -1.701 | -0.624 |
| sp\|Q9NYQ6\|CELR1_HUMAN Cadherin EGF LAG seven-pass G-type receptor 1 OS=Homo sapiens GN=CELSR1 I | 2525 | VIGINQtENPF | 0.148 | 0.034 | -0.591 | -0.136 |
| sp\|Q9NYQ6\|CELR1_HUMAN Cadherin EGF LAG seven-pass G-type receptor 1 OS=Homo sapiens GN=CELSR1 I | 2532 | ENPFLCtVVAI | 0.161 | -0.055 | -0.858 | -0.251 |
| sp\|Q9NYQ6\|CELR1_HUMAN Cadherin EGF LAG seven-pass G-type receptor 1 OS=Homo sapiens GN=CELSR1 I | 2544 | LHYIYMsTFAW | 0.197 | 0.181 | -0.676 | -0.099 |
| sp\|Q9NYQ6\|CELR1_HUMAN Cadherin EGF LAG seven-pass G-type receptor 1 OS=Homo sapiens GN=CELSR1 I | 2545 | HYIYMStFAWT | 0.156 | -0.011 | -0.551 | -0.135 |
| sp\|Q9NYQ6\|CELR1_HUMAN Cadherin EGF LAG seven-pass G-type receptor 1 OS=Homo sapiens GN=CELSR1 I | 2549 | MSTFAWtLVES | 0.563 | 0.154 | -0.1 | 0.206 |
| sp\|Q9NYQ6\|CELR1_HUMAN Cadherin EGF LAG seven-pass G-type receptor 1 OS=Homo sapiens GN=CELSR1 I | 2553 | AWTLVEsLHVY | 0.431 | 0.101 | -0.637 | -0.035 |
| sp\|Q9NYQ6\|CELR1_HUMAN Cadherin EGF LAG seven-pass G-type receptor 1 OS=Homo sapiens GN=CELSR1 I | 2561 | HVYRMLtEVRN | 0.411 | 0.823 | 0.041 | 0.425 |
| sp\|Q9NYQ6\|CELR1_HUMAN Cadherin EGF LAG seven-pass G-type receptor 1 OS=Homo sapiens GN=CELSR1 I | 2568 | EVRNIDtGPMR | 0.483 | 0.508 | 0.14 | 0.377 |
| sp\|Q9NYQ6\|CELR1_HUMAN Cadherin EGF LAG seven-pass G-type receptor 1 OS=Homo sapiens GN=CELSR1 I | 2586 | GIPAIVtGLAV | 0.257 | -0.009 | -0.738 | -0.163 |
| sp\|Q9NYQ6\|CELR1_HUMAN Cadherin EGF LAG seven-pass G-type receptor 1 OS=Homo sapiens GN=CELSR1 I | 2606 | PDFCWLsLQDT | 0.353 | 0.026 | -0.378 | 0 |
| sp\|Q9NYQ6\|CELR1_HUMAN Cadherin EGF LAG seven-pass G-type receptor 1 OS=Homo sapiens GN=CELSR1 I | 2610 | WLSLQDtLIWS | 0.2 | -0.011 | -0.646 | -0.152 |
| sp\|Q9NYQ6\|CELR1_HUMAN Cadherin EGF LAG seven-pass G-type receptor 1 OS=Homo sapiens GN=CELSR1 I | 2614 | QDTLIWsFAGP | 0.098 | -0.269 | -0.91 | -0.36 |
| sp\|Q9NYQ6\|CELR1_HUMAN Cadherin EGF LAG seven-pass G-type receptor 1 OS=Homo sapiens GN=CELSR1 I | 2627 | AVIIINtVTSV | 0.137 | -0.024 | -1.199 | -0.362 |
| sp\|Q9NYQ6\|CELR1_HUMAN Cadherin EGF LAG seven-pass G-type receptor 1 OS=Homo sapiens GN=CELSR1 I | 2629 | IIINTVtSVLS | 0.154 | 0.041 | -0.761 | -0.189 |
| sp\|Q9NYQ6\|CELR1_HUMAN Cadherin EGF LAG seven-pass G-type receptor 1 OS=Homo sapiens GN=CELSR1 I | 2630 | IINTVTsVLSA | 0.142 | -0.092 | -1.062 | -0.337 |
| sp\|Q9NYQ6\|CELR1_HUMAN Cadherin EGF LAG seven-pass G-type receptor 1 OS=Homo sapiens GN=CELSR1 I | 2633 | TVTSVLsAKVS | 0.069 | -0.147 | -1.369 | -0.482 |
| sp\|Q9NYQ6\|CELR1_HUMAN Cadherin EGF LAG seven-pass G-type receptor 1 OS=Homo sapiens GN=CELSR1 I | 2637 | VLSAKVsCQRK | 0.344 | -0.114 | -0.7 | -0.157 |
| sp\|Q9NYQ6\|CELR1_HUMAN Cadherin EGF LAG seven-pass G-type receptor 1 OS=Homo sapiens GN=CELSR1 I | 2652 | GKKGIVsLLRT | 0.614 | 0.073 | 0.06 | 0.249 |
| sp\|Q9NYQ6\|CELR1_HUMAN Cadherin EGF LAG seven-pass G-type receptor 1 OS=Homo sapiens GN=CELSR1 I | 2656 | IVSLLRtAFLL | 0.114 | -0.023 | -1.156 | -0.355 |

| | | | | | | |
|---|---|---|---|---|---|---|
| sp\|Q9NYQ6\|CELR1_HUMAN Cadherin EGF LAG seven-pass G-type receptor 1 OS=Homo sapiens GN=CELSR1 I | 2665 | LLLLLIsATWL | 0.296 | 0.085 | -0.609 | -0.076 |
| sp\|Q9NYQ6\|CELR1_HUMAN Cadherin EGF LAG seven-pass G-type receptor 1 OS=Homo sapiens GN=CELSR1 I | 2667 | LLLISAtWLLG | 0.434 | 0.399 | -0.099 | 0.245 |
| sp\|Q9NYQ6\|CELR1_HUMAN Cadherin EGF LAG seven-pass G-type receptor 1 OS=Homo sapiens GN=CELSR1 I | 2681 | VNRDALsFHYL | 0.428 | 0.233 | -0.351 | 0.103 |
| sp\|Q9NYQ6\|CELR1_HUMAN Cadherin EGF LAG seven-pass G-type receptor 1 OS=Homo sapiens GN=CELSR1 I | 2690 | YLFAIFsGLQG | 0.221 | -0.003 | -0.437 | -0.073 |
| sp\|Q9NYQ6\|CELR1_HUMAN Cadherin EGF LAG seven-pass G-type receptor 1 OS=Homo sapiens GN=CELSR1 I | 2726 | KLHLEDsATTR | 0.2 | -0.13 | -1.302 | -0.411 |
| sp\|Q9NYQ6\|CELR1_HUMAN Cadherin EGF LAG seven-pass G-type receptor 1 OS=Homo sapiens GN=CELSR1 I | 2728 | HLEDSAtTRAT | 0.587 | 0.317 | 0.181 | 0.362 |
| sp\|Q9NYQ6\|CELR1_HUMAN Cadherin EGF LAG seven-pass G-type receptor 1 OS=Homo sapiens GN=CELSR1 I | 2729 | LEDSATtRATL | 0.116 | -0.213 | -1.173 | -0.423 |
| sp\|Q9NYQ6\|CELR1_HUMAN Cadherin EGF LAG seven-pass G-type receptor 1 OS=Homo sapiens GN=CELSR1 I | 2732 | SATTRAtLLTR | 0.374 | 0.082 | -0.517 | -0.02 |
| sp\|Q9NYQ6\|CELR1_HUMAN Cadherin EGF LAG seven-pass G-type receptor 1 OS=Homo sapiens GN=CELSR1 I | 2735 | TRATLLtRSLN | 0.067 | -0.074 | -1.453 | -0.487 |
| sp\|Q9NYQ6\|CELR1_HUMAN Cadherin EGF LAG seven-pass G-type receptor 1 OS=Homo sapiens GN=CELSR1 I | 2737 | ATLLTRsLNCN | 0.256 | 0.112 | -0.752 | -0.128 |
| sp\|Q9NYQ6\|CELR1_HUMAN Cadherin EGF LAG seven-pass G-type receptor 1 OS=Homo sapiens GN=CELSR1 I | 2742 | RSLNCNtTFGD | 0.415 | -0.01 | -0.269 | 0.045 |
| sp\|Q9NYQ6\|CELR1_HUMAN Cadherin EGF LAG seven-pass G-type receptor 1 OS=Homo sapiens GN=CELSR1 I | 2743 | SLNCNTtFGDG | 0.623 | 0.174 | -0.12 | 0.226 |
| sp\|Q9NYQ6\|CELR1_HUMAN Cadherin EGF LAG seven-pass G-type receptor 1 OS=Homo sapiens GN=CELSR1 I | 2753 | GPDMLRtDLGE | 0.092 | -0.252 | -1.61 | -0.59 |
| sp\|Q9NYQ6\|CELR1_HUMAN Cadherin EGF LAG seven-pass G-type receptor 1 OS=Homo sapiens GN=CELSR1 I | 2758 | RTDLGEsTASL | 0.052 | -0.251 | -1.73 | -0.643 |
| sp\|Q9NYQ6\|CELR1_HUMAN Cadherin EGF LAG seven-pass G-type receptor 1 OS=Homo sapiens GN=CELSR1 I | 2759 | TDLGEStASLD | 0.048 | -0.261 | -1.515 | -0.576 |
| sp\|Q9NYQ6\|CELR1_HUMAN Cadherin EGF LAG seven-pass G-type receptor 1 OS=Homo sapiens GN=CELSR1 I | 2761 | LGESTAsLDSI | 0.284 | 0.077 | -0.207 | 0.051 |
| sp\|Q9NYQ6\|CELR1_HUMAN Cadherin EGF LAG seven-pass G-type receptor 1 OS=Homo sapiens GN=CELSR1 I | 2764 | STASLDsIVRD | 0.068 | -0.404 | -1.671 | -0.669 |
| sp\|Q9NYQ6\|CELR1_HUMAN Cadherin EGF LAG seven-pass G-type receptor 1 OS=Homo sapiens GN=CELSR1 I | 2777 | IQKLGVsSGLV | 0.293 | -0.054 | -0.651 | -0.137 |
| sp\|Q9NYQ6\|CELR1_HUMAN Cadherin EGF LAG seven-pass G-type receptor 1 OS=Homo sapiens GN=CELSR1 I | 2778 | QKLGVSsGLVR | 0.118 | -0.104 | -0.92 | -0.302 |
| sp\|Q9NYQ6\|CELR1_HUMAN Cadherin EGF LAG seven-pass G-type receptor 1 OS=Homo sapiens GN=CELSR1 I | 2784 | SGLVRGsHGEP | 0.163 | -0.057 | -0.95 | -0.281 |
| sp\|Q9NYQ6\|CELR1_HUMAN Cadherin EGF LAG seven-pass G-type receptor 1 OS=Homo sapiens GN=CELSR1 I | 2791 | HGEPDAsLMPR | 0.308 | -0.014 | -0.28 | 0.005 |
| sp\|Q9NYQ6\|CELR1_HUMAN Cadherin EGF LAG seven-pass G-type receptor 1 OS=Homo sapiens GN=CELSR1 I | 2796 | ASLMPRsCKDP | 0.107 | -0.227 | -1.139 | -0.42 |
| sp\|Q9NYQ6\|CELR1_HUMAN Cadherin EGF LAG seven-pass G-type receptor 1 OS=Homo sapiens GN=CELSR1 I | 2805 | DPPGHDsDSDS | 0.142 | -0.177 | -1.455 | -0.497 |
| sp\|Q9NYQ6\|CELR1_HUMAN Cadherin EGF LAG seven-pass G-type receptor 1 OS=Homo sapiens GN=CELSR1 I | 2807 | PGHDSDsDSEL | 0.28 | 0.109 | -0.776 | -0.129 |
| sp\|Q9NYQ6\|CELR1_HUMAN Cadherin EGF LAG seven-pass G-type receptor 1 OS=Homo sapiens GN=CELSR1 I | 2809 | HDSDSDsELSL | 0.114 | 0.088 | -0.948 | -0.249 |
| sp\|Q9NYQ6\|CELR1_HUMAN Cadherin EGF LAG seven-pass G-type receptor 1 OS=Homo sapiens GN=CELSR1 I | 2812 | DSDSELsLDEQ | 0.222 | -0.179 | -1.04 | -0.332 |
| sp\|Q9NYQ6\|CELR1_HUMAN Cadherin EGF LAG seven-pass G-type receptor 1 OS=Homo sapiens GN=CELSR1 I | 2817 | LSLDEQsSSYA | 0.074 | -0.292 | -1.409 | -0.542 |
| sp\|Q9NYQ6\|CELR1_HUMAN Cadherin EGF LAG seven-pass G-type receptor 1 OS=Homo sapiens GN=CELSR1 I | 2818 | SLDEQSsSYAS | 0.047 | -0.127 | -1.594 | -0.558 |
| sp\|Q9NYQ6\|CELR1_HUMAN Cadherin EGF LAG seven-pass G-type receptor 1 OS=Homo sapiens GN=CELSR1 I | 2819 | LDEQSSsYASS | 0.057 | -0.178 | -1.085 | -0.402 |
| sp\|Q9NYQ6\|CELR1_HUMAN Cadherin EGF LAG seven-pass G-type receptor 1 OS=Homo sapiens GN=CELSR1 I | 2822 | QSSSYAsSHSS | 0.081 | -0.253 | -1.374 | -0.515 |
| sp\|Q9NYQ6\|CELR1_HUMAN Cadherin EGF LAG seven-pass G-type receptor 1 OS=Homo sapiens GN=CELSR1 I | 2823 | SSSYASsHSSD | 0.086 | -0.224 | -1.23 | -0.456 |
| sp\|Q9NYQ6\|CELR1_HUMAN Cadherin EGF LAG seven-pass G-type receptor 1 OS=Homo sapiens GN=CELSR1 I | 2825 | SYASSHsSDSE | 0.245 | 0.157 | -0.585 | -0.061 |
| sp\|Q9NYQ6\|CELR1_HUMAN Cadherin EGF LAG seven-pass G-type receptor 1 OS=Homo sapiens GN=CELSR1 I | 2826 | YASSHSsDSED | 0.139 | -0.166 | -1.149 | -0.392 |
| sp\|Q9NYQ6\|CELR1_HUMAN Cadherin EGF LAG seven-pass G-type receptor 1 OS=Homo sapiens GN=CELSR1 I | 2828 | SSHSSDsEDDG | 0.297 | 0.065 | -0.665 | -0.101 |
| sp\|Q9NYQ6\|CELR1_HUMAN Cadherin EGF LAG seven-pass G-type receptor 1 OS=Homo sapiens GN=CELSR1 I | 2848 | ARGAVHsTPKG | 0.603 | 0.679 | -0.077 | 0.402 |
| sp\|Q9NYQ6\|CELR1_HUMAN Cadherin EGF LAG seven-pass G-type receptor 1 OS=Homo sapiens GN=CELSR1 I | 2849 | RGAVHStPKGD | 0.073 | -0.391 | -1.125 | -0.481 |
| sp\|Q9NYQ6\|CELR1_HUMAN Cadherin EGF LAG seven-pass G-type receptor 1 OS=Homo sapiens GN=CELSR1 I | 2867 | AGWPDQsLAES | 0.162 | -0.024 | -0.737 | -0.2 |
| sp\|Q9NYQ6\|CELR1_HUMAN Cadherin EGF LAG seven-pass G-type receptor 1 OS=Homo sapiens GN=CELSR1 I | 2871 | DQSLAEsDSED | 0.27 | -0.052 | -0.956 | -0.246 |
| sp\|Q9NYQ6\|CELR1_HUMAN Cadherin EGF LAG seven-pass G-type receptor 1 OS=Homo sapiens GN=CELSR1 I | 2873 | SLAESDsEDPS | 0.257 | 0.15 | -0.57 | -0.054 |
| sp\|Q9NYQ6\|CELR1_HUMAN Cadherin EGF LAG seven-pass G-type receptor 1 OS=Homo sapiens GN=CELSR1 I | 2877 | SDSEDPsGKPR | 0.032 | -0.473 | -1.748 | -0.73 |
| sp\|Q9NYQ6\|CELR1_HUMAN Cadherin EGF LAG seven-pass G-type receptor 1 OS=Homo sapiens GN=CELSR1 I | 2886 | PRLKVEtKVSV | 0.254 | 0.16 | -0.868 | -0.151 |
| sp\|Q9NYQ6\|CELR1_HUMAN Cadherin EGF LAG seven-pass G-type receptor 1 OS=Homo sapiens GN=CELSR1 I | 2889 | KVETKVsVELH | 0.117 | -0.245 | -1.267 | -0.465 |
| sp\|Q9NYQ6\|CELR1_HUMAN Cadherin EGF LAG seven-pass G-type receptor 1 OS=Homo sapiens GN=CELSR1 I | 2899 | HREEQGsHRGE | 0.107 | -0.018 | -1.043 | -0.318 |
| sp\|Q9NYQ6\|CELR1_HUMAN Cadherin EGF LAG seven-pass G-type receptor 1 OS=Homo sapiens GN=CELSR1 I | 2910 | YPPDQEsGGAA | 0.109 | -0.141 | -1.186 | -0.406 |
| sp\|Q9NYQ6\|CELR1_HUMAN Cadherin EGF LAG seven-pass G-type receptor 1 OS=Homo sapiens GN=CELSR1 I | 2918 | GAARLAsSQPP | 0.465 | 0.676 | -0.101 | 0.347 |
| sp\|Q9NYQ6\|CELR1_HUMAN Cadherin EGF LAG seven-pass G-type receptor 1 OS=Homo sapiens GN=CELSR1 I | 2919 | AARLASsQPPE | 0.601 | 0.482 | -0.281 | 0.267 |
| sp\|Q9NYQ6\|CELR1_HUMAN Cadherin EGF LAG seven-pass G-type receptor 1 OS=Homo sapiens GN=CELSR1 I | 2934 | ILKNKVtYPPP | 0.588 | 0.373 | 0.269 | 0.41 |
| sp\|Q9NYQ6\|CELR1_HUMAN Cadherin EGF LAG seven-pass G-type receptor 1 OS=Homo sapiens GN=CELSR1 I | 2940 | TYPPPLtLTEQ | 0.15 | -0.21 | -0.935 | -0.332 |
| sp\|Q9NYQ6\|CELR1_HUMAN Cadherin EGF LAG seven-pass G-type receptor 1 OS=Homo sapiens GN=CELSR1 I | 2942 | PPPLTLtEQTL | 0.091 | -0.024 | -1.111 | -0.348 |

| | | | | | | |
|---|---|---|---|---|---|---|
| sp\|Q9NYQ6\|CELR1_HUMAN Cadherin EGF LAG seven-pass G-type receptor 1 OS=Homo sapiens GN=CELSR1 I | 2945 | LTLTEQtLKGR | 0.305 | -0.077 | -0.463 | -0.078 |
| sp\|Q9NYQ6\|CELR1_HUMAN Cadherin EGF LAG seven-pass G-type receptor 1 OS=Homo sapiens GN=CELSR1 I | 2960 | LADCEQsPTSS | 0.042 | -0.542 | -1.873 | -0.791 |
| sp\|Q9NYQ6\|CELR1_HUMAN Cadherin EGF LAG seven-pass G-type receptor 1 OS=Homo sapiens GN=CELSR1 I | 2962 | DCEQSPtSSRT | 0.298 | -0.081 | -0.82 | -0.201 |
| sp\|Q9NYQ6\|CELR1_HUMAN Cadherin EGF LAG seven-pass G-type receptor 1 OS=Homo sapiens GN=CELSR1 I | 2963 | CEQSPTsSRTS | 0.115 | -0.263 | -1.088 | -0.412 |
| sp\|Q9NYQ6\|CELR1_HUMAN Cadherin EGF LAG seven-pass G-type receptor 1 OS=Homo sapiens GN=CELSR1 I | 2964 | EQSPTSsRTSS | 0.022 | -0.371 | -1.721 | -0.69 |
| sp\|Q9NYQ6\|CELR1_HUMAN Cadherin EGF LAG seven-pass G-type receptor 1 OS=Homo sapiens GN=CELSR1 I | 2966 | SPTSSRtSSLG | 0.088 | -0.067 | -1.281 | -0.42 |
| sp\|Q9NYQ6\|CELR1_HUMAN Cadherin EGF LAG seven-pass G-type receptor 1 OS=Homo sapiens GN=CELSR1 I | 2967 | PTSSRTsSLGS | 0.174 | -0.112 | -0.993 | -0.31 |
| sp\|Q9NYQ6\|CELR1_HUMAN Cadherin EGF LAG seven-pass G-type receptor 1 OS=Homo sapiens GN=CELSR1 I | 2968 | TSSRTSsLGSG | 0.458 | 0.785 | 0.221 | 0.488 |
| sp\|Q9NYQ6\|CELR1_HUMAN Cadherin EGF LAG seven-pass G-type receptor 1 OS=Homo sapiens GN=CELSR1 I | 2971 | RTSSLGsGGPD | 0.124 | -0.273 | -1.232 | -0.46 |
| sp\|Q9NYQ6\|CELR1_HUMAN Cadherin EGF LAG seven-pass G-type receptor 1 OS=Homo sapiens GN=CELSR1 I | 2979 | GPDCAItVKSP | 0.162 | -0.12 | -1.063 | -0.34 |
| sp\|Q9NYQ6\|CELR1_HUMAN Cadherin EGF LAG seven-pass G-type receptor 1 OS=Homo sapiens GN=CELSR1 I | 2982 | CAITVKsPGRE | 0.2 | -0.343 | -1.033 | -0.392 |
| sp\|Q9NYQ6\|CELR1_HUMAN Cadherin EGF LAG seven-pass G-type receptor 1 OS=Homo sapiens GN=CELSR1 I | 3001 | VAMNVRtGSAQ | 0.056 | -0.207 | -1.357 | -0.503 |
| sp\|Q9NYQ6\|CELR1_HUMAN Cadherin EGF LAG seven-pass G-type receptor 1 OS=Homo sapiens GN=CELSR1 I | 3003 | MNVRTGsAQAD | 0.591 | 0.941 | 0.342 | 0.625 |
| sp\|Q9NYQ6\|CELR1_HUMAN Cadherin EGF LAG seven-pass G-type receptor 1 OS=Homo sapiens GN=CELSR1 I | 3009 | SAQADGsDSEK | 0.08 | -0.3 | -1.62 | -0.613 |
| sp\|Q9NYQ6\|CELR1_HUMAN Cadherin EGF LAG seven-pass G-type receptor 1 OS=Homo sapiens GN=CELSR1 I | 3011 | QADGSDsEKP- | 0.176 | 0.024 | -0.781 | -0.194 |
| sp\|Q9HCU4\|CELR2_HUMAN Cadherin EGF LAG seven-pass G-type receptor 2 OS=Homo sapiens GN=CELSR2 I | 332 | YRLLEGsGGSP | 0.071 | -0.046 | -1.278 | -0.418 |
| sp\|Q9HCU4\|CELR2_HUMAN Cadherin EGF LAG seven-pass G-type receptor 2 OS=Homo sapiens GN=CELSR2 I | 335 | LEGSGGsPSEV | 0.017 | -0.638 | -2.38 | -1 |
| sp\|Q9HCU4\|CELR2_HUMAN Cadherin EGF LAG seven-pass G-type receptor 2 OS=Homo sapiens GN=CELSR2 I | 337 | GSGGSPsEVFE | 0.186 | 0.038 | -0.731 | -0.169 |
| sp\|Q9HCU4\|CELR2_HUMAN Cadherin EGF LAG seven-pass G-type receptor 2 OS=Homo sapiens GN=CELSR2 I | 346 | FEIDPRsGVIR | 0.111 | -0.096 | -1.285 | -0.423 |
| sp\|Q9HCU4\|CELR2_HUMAN Cadherin EGF LAG seven-pass G-type receptor 2 OS=Homo sapiens GN=CELSR2 I | 351 | RSGVIRtRGPV | 0.078 | -0.216 | -1.499 | -0.546 |
| sp\|Q9HCU4\|CELR2_HUMAN Cadherin EGF LAG seven-pass G-type receptor 2 OS=Homo sapiens GN=CELSR2 I | 362 | DREEVEsYQLT | 0.136 | 0.012 | -1.159 | -0.337 |
| sp\|Q9HCU4\|CELR2_HUMAN Cadherin EGF LAG seven-pass G-type receptor 2 OS=Homo sapiens GN=CELSR2 I | 366 | VESYQLtVEAS | 0.074 | -0.103 | -1.211 | -0.413 |
| sp\|Q9HCU4\|CELR2_HUMAN Cadherin EGF LAG seven-pass G-type receptor 2 OS=Homo sapiens GN=CELSR2 I | 370 | QLTVEAsDQGR | 0.33 | 0.033 | -0.571 | -0.069 |
| sp\|Q9HCU4\|CELR2_HUMAN Cadherin EGF LAG seven-pass G-type receptor 2 OS=Homo sapiens GN=CELSR2 I | 380 | RDPGPRsTTAA | 0.074 | -0.239 | -1.238 | -0.468 |
| sp\|Q9HCU4\|CELR2_HUMAN Cadherin EGF LAG seven-pass G-type receptor 2 OS=Homo sapiens GN=CELSR2 I | 381 | DPGPRStTAAV | 0.138 | -0.087 | -1.263 | -0.404 |
| sp\|Q9HCU4\|CELR2_HUMAN Cadherin EGF LAG seven-pass G-type receptor 2 OS=Homo sapiens GN=CELSR2 I | 382 | PGPRSTtAAVF | 0.632 | 0.998 | 0.469 | 0.7 |
| sp\|Q9HCU4\|CELR2_HUMAN Cadherin EGF LAG seven-pass G-type receptor 2 OS=Homo sapiens GN=CELSR2 I | 388 | TAAVFLsVEDD | 0.125 | -0.117 | -0.997 | -0.33 |
| sp\|Q9HCU4\|CELR2_HUMAN Cadherin EGF LAG seven-pass G-type receptor 2 OS=Homo sapiens GN=CELSR2 I | 400 | DNAPQFsEKRY | 0.17 | -0.142 | -1.011 | -0.328 |
| sp\|Q9HCU4\|CELR2_HUMAN Cadherin EGF LAG seven-pass G-type receptor 2 OS=Homo sapiens GN=CELSR2 I | 413 | QVREDVtPGAP | 0.181 | -0.09 | -0.734 | -0.214 |
| sp\|Q9HCU4\|CELR2_HUMAN Cadherin EGF LAG seven-pass G-type receptor 2 OS=Homo sapiens GN=CELSR2 I | 422 | APVLRVtASDR | 0.42 | 0.093 | -0.658 | -0.048 |
| sp\|Q9HCU4\|CELR2_HUMAN Cadherin EGF LAG seven-pass G-type receptor 2 OS=Homo sapiens GN=CELSR2 I | 424 | VLRVTAsDRDK | 0.574 | 0.304 | -0.181 | 0.232 |
| sp\|Q9HCU4\|CELR2_HUMAN Cadherin EGF LAG seven-pass G-type receptor 2 OS=Homo sapiens GN=CELSR2 I | 430 | SDRDKGsNAVV | 0.068 | -0.137 | -1.878 | -0.649 |
| sp\|Q9HCU4\|CELR2_HUMAN Cadherin EGF LAG seven-pass G-type receptor 2 OS=Homo sapiens GN=CELSR2 I | 437 | NAVVHYsIMSG | 0.083 | -0.06 | -1.09 | -0.356 |
| sp\|Q9HCU4\|CELR2_HUMAN Cadherin EGF LAG seven-pass G-type receptor 2 OS=Homo sapiens GN=CELSR2 I | 440 | VHYSIMsGNAR | 0.194 | 0.044 | -0.545 | -0.102 |
| sp\|Q9HCU4\|CELR2_HUMAN Cadherin EGF LAG seven-pass G-type receptor 2 OS=Homo sapiens GN=CELSR2 I | 453 | FYLDAQtGALD | 0.348 | 0.122 | -0.419 | 0.017 |
| sp\|Q9HCU4\|CELR2_HUMAN Cadherin EGF LAG seven-pass G-type receptor 2 OS=Homo sapiens GN=CELSR2 I | 460 | GALDVVsPLDY | 0.276 | -0.254 | -1.057 | -0.345 |
| sp\|Q9HCU4\|CELR2_HUMAN Cadherin EGF LAG seven-pass G-type receptor 2 OS=Homo sapiens GN=CELSR2 I | 466 | SPLDYEtTKEY | 0.152 | -0.148 | -1.334 | -0.443 |
| sp\|Q9HCU4\|CELR2_HUMAN Cadherin EGF LAG seven-pass G-type receptor 2 OS=Homo sapiens GN=CELSR2 I | 467 | PLDYETtKEYT | 0.206 | -0.055 | -0.744 | -0.198 |
| sp\|Q9HCU4\|CELR2_HUMAN Cadherin EGF LAG seven-pass G-type receptor 2 OS=Homo sapiens GN=CELSR2 I | 471 | ETTKEYtLRVR | 0.484 | 0.11 | -0.088 | 0.169 |
| sp\|Q9HCU4\|CELR2_HUMAN Cadherin EGF LAG seven-pass G-type receptor 2 OS=Homo sapiens GN=CELSR2 I | 485 | GGRPPLsNVSG | 0.117 | -0.033 | -0.997 | -0.304 |
| sp\|Q9HCU4\|CELR2_HUMAN Cadherin EGF LAG seven-pass G-type receptor 2 OS=Homo sapiens GN=CELSR2 I | 488 | PPLSNVsGLVT | 0.147 | -0.212 | -0.927 | -0.331 |
| sp\|Q9HCU4\|CELR2_HUMAN Cadherin EGF LAG seven-pass G-type receptor 2 OS=Homo sapiens GN=CELSR2 I | 492 | NVSGLVtVQVL | 0.191 | -0.001 | -0.685 | -0.165 |
| sp\|Q9HCU4\|CELR2_HUMAN Cadherin EGF LAG seven-pass G-type receptor 2 OS=Homo sapiens GN=CELSR2 I | 507 | NAPIFVsTPFQ | 0.335 | 0.218 | -0.672 | -0.04 |
| sp\|Q9HCU4\|CELR2_HUMAN Cadherin EGF LAG seven-pass G-type receptor 2 OS=Homo sapiens GN=CELSR2 I | 508 | APIFVStPFQA | 0.071 | -0.468 | -1.475 | -0.624 |
| sp\|Q9HCU4\|CELR2_HUMAN Cadherin EGF LAG seven-pass G-type receptor 2 OS=Homo sapiens GN=CELSR2 I | 513 | STPFQAtVLES | 0.083 | -0.151 | -1.607 | -0.558 |
| sp\|Q9HCU4\|CELR2_HUMAN Cadherin EGF LAG seven-pass G-type receptor 2 OS=Homo sapiens GN=CELSR2 I | 517 | QATVLEsVPLG | 0.28 | 0.317 | -0.562 | 0.012 |
| sp\|Q9HCU4\|CELR2_HUMAN Cadherin EGF LAG seven-pass G-type receptor 2 OS=Homo sapiens GN=CELSR2 I | 554 | GHDFPFtINNG | 0.191 | -0.02 | -0.964 | -0.264 |
| sp\|Q9HCU4\|CELR2_HUMAN Cadherin EGF LAG seven-pass G-type receptor 2 OS=Homo sapiens GN=CELSR2 I | 559 | FTINNGtGWIS | 0.171 | -0.077 | -0.98 | -0.295 |
| sp\|Q9HCU4\|CELR2_HUMAN Cadherin EGF LAG seven-pass G-type receptor 2 OS=Homo sapiens GN=CELSR2 I | 563 | NGTGWIsVAAE | 0.24 | -0.013 | -0.401 | -0.058 |

| | | | | | | |
|---|---|---|---|---|---|---|
| sp\|Q9HCU4\|CELR2_HUMAN Cadherin EGF LAG seven-pass G-type receptor 2 OS=Homo sapiens GN=CELSR2 I | 577 | EEVDFYsFGVE | 0.253 | 0.106 | -0.712 | -0.118 |
| sp\|Q9HCU4\|CELR2_HUMAN Cadherin EGF LAG seven-pass G-type receptor 2 OS=Homo sapiens GN=CELSR2 I | 587 | EARDHGtPALT | 0.082 | -0.253 | -1.384 | -0.518 |
| sp\|Q9HCU4\|CELR2_HUMAN Cadherin EGF LAG seven-pass G-type receptor 2 OS=Homo sapiens GN=CELSR2 I | 591 | HGTPALtASAS | 0.123 | 0.009 | -0.777 | -0.215 |
| sp\|Q9HCU4\|CELR2_HUMAN Cadherin EGF LAG seven-pass G-type receptor 2 OS=Homo sapiens GN=CELSR2 I | 593 | TPALTAsASVS | 0.075 | -0.068 | -1.378 | -0.457 |
| sp\|Q9HCU4\|CELR2_HUMAN Cadherin EGF LAG seven-pass G-type receptor 2 OS=Homo sapiens GN=CELSR2 I | 595 | ALTASAsVSVT | 0.412 | 0.188 | -0.244 | 0.119 |
| sp\|Q9HCU4\|CELR2_HUMAN Cadherin EGF LAG seven-pass G-type receptor 2 OS=Homo sapiens GN=CELSR2 I | 597 | TASASVsVTVL | 0.465 | 0.123 | -0.236 | 0.117 |
| sp\|Q9HCU4\|CELR2_HUMAN Cadherin EGF LAG seven-pass G-type receptor 2 OS=Homo sapiens GN=CELSR2 I | 599 | SASVSVtVLDV | 0.283 | 0.077 | -0.819 | -0.153 |
| sp\|Q9HCU4\|CELR2_HUMAN Cadherin EGF LAG seven-pass G-type receptor 2 OS=Homo sapiens GN=CELSR2 I | 609 | VNDNNPtFTQP | 0.128 | -0.292 | -0.819 | -0.328 |
| sp\|Q9HCU4\|CELR2_HUMAN Cadherin EGF LAG seven-pass G-type receptor 2 OS=Homo sapiens GN=CELSR2 I | 611 | DNNPTFtQPEY | 0.462 | 0.371 | -0.485 | 0.116 |
| sp\|Q9HCU4\|CELR2_HUMAN Cadherin EGF LAG seven-pass G-type receptor 2 OS=Homo sapiens GN=CELSR2 I | 616 | FTQPEYtVRLN | 0.255 | 0.057 | -0.704 | -0.131 |
| sp\|Q9HCU4\|CELR2_HUMAN Cadherin EGF LAG seven-pass G-type receptor 2 OS=Homo sapiens GN=CELSR2 I | 627 | EDAAVGtSVVT | 0.037 | -0.33 | -1.515 | -0.603 |
| sp\|Q9HCU4\|CELR2_HUMAN Cadherin EGF LAG seven-pass G-type receptor 2 OS=Homo sapiens GN=CELSR2 I | 628 | DAAVGTsVVTV | 0.082 | -0.203 | -1.395 | -0.505 |
| sp\|Q9HCU4\|CELR2_HUMAN Cadherin EGF LAG seven-pass G-type receptor 2 OS=Homo sapiens GN=CELSR2 I | 631 | VGTSVVtVSAV | 0.238 | -0.115 | -0.819 | -0.232 |
| sp\|Q9HCU4\|CELR2_HUMAN Cadherin EGF LAG seven-pass G-type receptor 2 OS=Homo sapiens GN=CELSR2 I | 633 | TSVVTVsAVDR | 0.182 | -0.01 | -0.893 | -0.24 |
| sp\|Q9HCU4\|CELR2_HUMAN Cadherin EGF LAG seven-pass G-type receptor 2 OS=Homo sapiens GN=CELSR2 I | 641 | VDRDAHsVITY | 0.49 | 0.325 | -0.233 | 0.194 |
| sp\|Q9HCU4\|CELR2_HUMAN Cadherin EGF LAG seven-pass G-type receptor 2 OS=Homo sapiens GN=CELSR2 I | 644 | DAHSVItYQIT | 0.123 | -0.296 | -1.323 | -0.499 |
| sp\|Q9HCU4\|CELR2_HUMAN Cadherin EGF LAG seven-pass G-type receptor 2 OS=Homo sapiens GN=CELSR2 I | 648 | VITYQItSGNT | 0.189 | 0.032 | -0.767 | -0.182 |
| sp\|Q9HCU4\|CELR2_HUMAN Cadherin EGF LAG seven-pass G-type receptor 2 OS=Homo sapiens GN=CELSR2 I | 649 | ITYQITsGNTR | 0.265 | -0.101 | -0.257 | -0.031 |
| sp\|Q9HCU4\|CELR2_HUMAN Cadherin EGF LAG seven-pass G-type receptor 2 OS=Homo sapiens GN=CELSR2 I | 652 | QITSGNtRNRF | 0.122 | -0.201 | -1.128 | -0.402 |
| sp\|Q9HCU4\|CELR2_HUMAN Cadherin EGF LAG seven-pass G-type receptor 2 OS=Homo sapiens GN=CELSR2 I | 657 | NTRNRFsITSQ | 0.358 | 0.23 | -0.372 | 0.072 |
| sp\|Q9HCU4\|CELR2_HUMAN Cadherin EGF LAG seven-pass G-type receptor 2 OS=Homo sapiens GN=CELSR2 I | 659 | RNRFSItSQSG | 0.513 | 0.345 | -0.065 | 0.264 |
| sp\|Q9HCU4\|CELR2_HUMAN Cadherin EGF LAG seven-pass G-type receptor 2 OS=Homo sapiens GN=CELSR2 I | 660 | NRFSITsQSGG | 0.144 | -0.006 | -0.924 | -0.262 |
| sp\|Q9HCU4\|CELR2_HUMAN Cadherin EGF LAG seven-pass G-type receptor 2 OS=Homo sapiens GN=CELSR2 I | 662 | FSITSQsGGGL | 0.401 | 0.214 | -0.13 | 0.162 |
| sp\|Q9HCU4\|CELR2_HUMAN Cadherin EGF LAG seven-pass G-type receptor 2 OS=Homo sapiens GN=CELSR2 I | 668 | SGGGLVsLALP | 0.101 | -0.188 | -1.061 | -0.383 |
| sp\|Q9HCU4\|CELR2_HUMAN Cadherin EGF LAG seven-pass G-type receptor 2 OS=Homo sapiens GN=CELSR2 I | 686 | QYVLAVtASDG | 0.467 | 0.117 | -0.515 | 0.023 |
| sp\|Q9HCU4\|CELR2_HUMAN Cadherin EGF LAG seven-pass G-type receptor 2 OS=Homo sapiens GN=CELSR2 I | 688 | VLAVTAsDGTR | 0.32 | 0.131 | -0.444 | 0.002 |
| sp\|Q9HCU4\|CELR2_HUMAN Cadherin EGF LAG seven-pass G-type receptor 2 OS=Homo sapiens GN=CELSR2 I | 691 | VTASDGtRQDT | 0.113 | -0.339 | -1.227 | -0.484 |
| sp\|Q9HCU4\|CELR2_HUMAN Cadherin EGF LAG seven-pass G-type receptor 2 OS=Homo sapiens GN=CELSR2 I | 695 | DGTRQDtAQIV | 0.636 | 0.776 | -0.214 | 0.399 |
| sp\|Q9HCU4\|CELR2_HUMAN Cadherin EGF LAG seven-pass G-type receptor 2 OS=Homo sapiens GN=CELSR2 I | 703 | QIVVNVtDANT | 0.197 | -0.029 | -0.955 | -0.262 |
| sp\|Q9HCU4\|CELR2_HUMAN Cadherin EGF LAG seven-pass G-type receptor 2 OS=Homo sapiens GN=CELSR2 I | 707 | NVTDANtHRPV | 0.371 | 0.012 | -0.736 | -0.118 |
| sp\|Q9HCU4\|CELR2_HUMAN Cadherin EGF LAG seven-pass G-type receptor 2 OS=Homo sapiens GN=CELSR2 I | 714 | HRPVFQsSHYT | 0.083 | 0.047 | -1.216 | -0.362 |
| sp\|Q9HCU4\|CELR2_HUMAN Cadherin EGF LAG seven-pass G-type receptor 2 OS=Homo sapiens GN=CELSR2 I | 715 | RPVFQSsHYTV | 0.11 | -0.102 | -1.445 | -0.479 |
| sp\|Q9HCU4\|CELR2_HUMAN Cadherin EGF LAG seven-pass G-type receptor 2 OS=Homo sapiens GN=CELSR2 I | 718 | FQSSHYtVNVN | 0.266 | 0.165 | -0.372 | 0.02 |
| sp\|Q9HCU4\|CELR2_HUMAN Cadherin EGF LAG seven-pass G-type receptor 2 OS=Homo sapiens GN=CELSR2 I | 729 | EDRPAGtTVVL | 0.16 | 0.096 | -0.819 | -0.188 |
| sp\|Q9HCU4\|CELR2_HUMAN Cadherin EGF LAG seven-pass G-type receptor 2 OS=Homo sapiens GN=CELSR2 I | 730 | DRPAGTtVVLI | 0.184 | 0.106 | -0.767 | -0.159 |
| sp\|Q9HCU4\|CELR2_HUMAN Cadherin EGF LAG seven-pass G-type receptor 2 OS=Homo sapiens GN=CELSR2 I | 735 | TTVVLIsATDE | 0.177 | -0.123 | -1.228 | -0.391 |
| sp\|Q9HCU4\|CELR2_HUMAN Cadherin EGF LAG seven-pass G-type receptor 2 OS=Homo sapiens GN=CELSR2 I | 737 | VVLISAtDEDT | 0.403 | 0.237 | -0.274 | 0.122 |
| sp\|Q9HCU4\|CELR2_HUMAN Cadherin EGF LAG seven-pass G-type receptor 2 OS=Homo sapiens GN=CELSR2 I | 741 | SATDEDtGENA | 0.077 | -0.299 | -1.73 | -0.651 |
| sp\|Q9HCU4\|CELR2_HUMAN Cadherin EGF LAG seven-pass G-type receptor 2 OS=Homo sapiens GN=CELSR2 I | 748 | GENARItYFME | 0.287 | -0.054 | -0.892 | -0.22 |
| sp\|Q9HCU4\|CELR2_HUMAN Cadherin EGF LAG seven-pass G-type receptor 2 OS=Homo sapiens GN=CELSR2 I | 754 | TYFMEDsIPQF | 0.421 | 0.237 | -0.394 | 0.088 |
| sp\|Q9HCU4\|CELR2_HUMAN Cadherin EGF LAG seven-pass G-type receptor 2 OS=Homo sapiens GN=CELSR2 I | 764 | FRIDADtGAVT | 0.198 | 0.164 | -0.93 | -0.189 |
| sp\|Q9HCU4\|CELR2_HUMAN Cadherin EGF LAG seven-pass G-type receptor 2 OS=Homo sapiens GN=CELSR2 I | 768 | ADTGAVtTQAE | 0.302 | -0.048 | -0.462 | -0.069 |
| sp\|Q9HCU4\|CELR2_HUMAN Cadherin EGF LAG seven-pass G-type receptor 2 OS=Homo sapiens GN=CELSR2 I | 769 | DTGAVTtQAEL | 0.174 | -0.182 | -1.307 | -0.438 |
| sp\|Q9HCU4\|CELR2_HUMAN Cadherin EGF LAG seven-pass G-type receptor 2 OS=Homo sapiens GN=CELSR2 I | 780 | DYEDQVsYTLA | 0.339 | -0.083 | -0.865 | -0.203 |
| sp\|Q9HCU4\|CELR2_HUMAN Cadherin EGF LAG seven-pass G-type receptor 2 OS=Homo sapiens GN=CELSR2 I | 782 | EDQVSYtLAIT | 0.234 | 0.173 | -0.264 | 0.048 |
| sp\|Q9HCU4\|CELR2_HUMAN Cadherin EGF LAG seven-pass G-type receptor 2 OS=Homo sapiens GN=CELSR2 I | 786 | SYTLAItARDN | 0.447 | 0.094 | -0.55 | -0.003 |
| sp\|Q9HCU4\|CELR2_HUMAN Cadherin EGF LAG seven-pass G-type receptor 2 OS=Homo sapiens GN=CELSR2 I | 796 | NGIPQKsDTTY | 0.291 | 0 | -0.718 | -0.142 |
| sp\|Q9HCU4\|CELR2_HUMAN Cadherin EGF LAG seven-pass G-type receptor 2 OS=Homo sapiens GN=CELSR2 I | 798 | IPQKSDtTYLE | 0.463 | 0.139 | -0.362 | 0.08 |
| sp\|Q9HCU4\|CELR2_HUMAN Cadherin EGF LAG seven-pass G-type receptor 2 OS=Homo sapiens GN=CELSR2 I | 799 | PQKSDTtYLEI | 0.177 | -0.09 | -0.631 | -0.181 |

| | | | | | | |
|---|---|---|---|---|---|---|
| sp\|Q9HCU4\|CELR2_HUMAN Cadherin EGF LAG seven-pass G-type receptor 2 OS=Homo sapiens GN=CELSR2 I | 819 | PQFLRDsYQGS | 0.206 | -0.049 | -0.795 | -0.213 |
| sp\|Q9HCU4\|CELR2_HUMAN Cadherin EGF LAG seven-pass G-type receptor 2 OS=Homo sapiens GN=CELSR2 I | 823 | RDSYQGsVYED | 0.114 | -0.108 | -1.066 | -0.353 |
| sp\|Q9HCU4\|CELR2_HUMAN Cadherin EGF LAG seven-pass G-type receptor 2 OS=Homo sapiens GN=CELSR2 I | 832 | EDVPPFtSVLQ | 0.036 | -0.316 | -1.451 | -0.577 |
| sp\|Q9HCU4\|CELR2_HUMAN Cadherin EGF LAG seven-pass G-type receptor 2 OS=Homo sapiens GN=CELSR2 I | 833 | DVPPFTsVLQI | 0.133 | -0.158 | -1.037 | -0.354 |
| sp\|Q9HCU4\|CELR2_HUMAN Cadherin EGF LAG seven-pass G-type receptor 2 OS=Homo sapiens GN=CELSR2 I | 838 | TSVLQIsATDR | 0.231 | -0.054 | -0.992 | -0.272 |
| sp\|Q9HCU4\|CELR2_HUMAN Cadherin EGF LAG seven-pass G-type receptor 2 OS=Homo sapiens GN=CELSR2 I | 840 | VLQISAtDRDS | 0.685 | 0.365 | 0.04 | 0.363 |
| sp\|Q9HCU4\|CELR2_HUMAN Cadherin EGF LAG seven-pass G-type receptor 2 OS=Homo sapiens GN=CELSR2 I | 844 | SATDRDsGLNG | 0.142 | -0.094 | -1.4 | -0.451 |
| sp\|Q9HCU4\|CELR2_HUMAN Cadherin EGF LAG seven-pass G-type receptor 2 OS=Homo sapiens GN=CELSR2 I | 853 | NGRVFYtFQGG | 0.453 | 0.281 | -0.008 | 0.242 |
| sp\|Q9HCU4\|CELR2_HUMAN Cadherin EGF LAG seven-pass G-type receptor 2 OS=Homo sapiens GN=CELSR2 I | 868 | GDFIVEsTSGI | 0.127 | -0.181 | -1.193 | -0.416 |
| sp\|Q9HCU4\|CELR2_HUMAN Cadherin EGF LAG seven-pass G-type receptor 2 OS=Homo sapiens GN=CELSR2 I | 869 | DFIVEStSGIV | 0.152 | -0.129 | -1.404 | -0.46 |
| sp\|Q9HCU4\|CELR2_HUMAN Cadherin EGF LAG seven-pass G-type receptor 2 OS=Homo sapiens GN=CELSR2 I | 870 | FIVESTsGIVR | 0.244 | 0.302 | -0.298 | 0.083 |
| sp\|Q9HCU4\|CELR2_HUMAN Cadherin EGF LAG seven-pass G-type receptor 2 OS=Homo sapiens GN=CELSR2 I | 875 | TSGIVRtLRRL | 0.149 | -0.156 | -1.077 | -0.361 |
| sp\|Q9HCU4\|CELR2_HUMAN Cadherin EGF LAG seven-pass G-type receptor 2 OS=Homo sapiens GN=CELSR2 I | 903 | GMPPARtPMEV | 0.088 | -0.21 | -1.64 | -0.587 |
| sp\|Q9HCU4\|CELR2_HUMAN Cadherin EGF LAG seven-pass G-type receptor 2 OS=Homo sapiens GN=CELSR2 I | 908 | RTPMEVtVTVL | 0.333 | -0.081 | -0.87 | -0.206 |
| sp\|Q9HCU4\|CELR2_HUMAN Cadherin EGF LAG seven-pass G-type receptor 2 OS=Homo sapiens GN=CELSR2 I | 910 | PMEVTVtVLDV | 0.19 | 0.138 | -0.9 | -0.191 |
| sp\|Q9HCU4\|CELR2_HUMAN Cadherin EGF LAG seven-pass G-type receptor 2 OS=Homo sapiens GN=CELSR2 I | 934 | VFVEENsPIGL | 0.068 | -0.359 | -1.322 | -0.538 |
| sp\|Q9HCU4\|CELR2_HUMAN Cadherin EGF LAG seven-pass G-type receptor 2 OS=Homo sapiens GN=CELSR2 I | 944 | LAVARVtATDP | 0.468 | 0.057 | -0.492 | 0.011 |
| sp\|Q9HCU4\|CELR2_HUMAN Cadherin EGF LAG seven-pass G-type receptor 2 OS=Homo sapiens GN=CELSR2 I | 946 | VARVTAtDPDE | 0.744 | 0.726 | 0.192 | 0.554 |
| sp\|Q9HCU4\|CELR2_HUMAN Cadherin EGF LAG seven-pass G-type receptor 2 OS=Homo sapiens GN=CELSR2 I | 952 | TDPDEGtNAQI | 0.048 | -0.402 | -1.786 | -0.713 |
| sp\|Q9HCU4\|CELR2_HUMAN Cadherin EGF LAG seven-pass G-type receptor 2 OS=Homo sapiens GN=CELSR2 I | 975 | FQLDIFsGELT | 0.118 | -0.019 | -0.663 | -0.188 |
| sp\|Q9HCU4\|CELR2_HUMAN Cadherin EGF LAG seven-pass G-type receptor 2 OS=Homo sapiens GN=CELSR2 I | 979 | IFSGELtALVD | 0.134 | -0.072 | -0.991 | -0.31 |
| sp\|Q9HCU4\|CELR2_HUMAN Cadherin EGF LAG seven-pass G-type receptor 2 OS=Homo sapiens GN=CELSR2 I | 999 | VLVIQAtSAPL | 0.115 | -0.055 | -1.126 | -0.355 |
| sp\|Q9HCU4\|CELR2_HUMAN Cadherin EGF LAG seven-pass G-type receptor 2 OS=Homo sapiens GN=CELSR2 I | 1000 | LVIQATsAPLV | 0.65 | 0.625 | 0.089 | 0.455 |
| sp\|Q9HCU4\|CELR2_HUMAN Cadherin EGF LAG seven-pass G-type receptor 2 OS=Homo sapiens GN=CELSR2 I | 1005 | TSAPLVsRATV | 0.094 | -0.392 | -1.435 | -0.578 |
| sp\|Q9HCU4\|CELR2_HUMAN Cadherin EGF LAG seven-pass G-type receptor 2 OS=Homo sapiens GN=CELSR2 I | 1008 | PLVSRAtVHVR | 0.595 | 0.29 | -0.147 | 0.246 |
| sp\|Q9HCU4\|CELR2_HUMAN Cadherin EGF LAG seven-pass G-type receptor 2 OS=Homo sapiens GN=CELSR2 I | 1035 | LFNNYVtNRSS | 0.132 | -0.193 | -0.976 | -0.346 |
| sp\|Q9HCU4\|CELR2_HUMAN Cadherin EGF LAG seven-pass G-type receptor 2 OS=Homo sapiens GN=CELSR2 I | 1038 | NYVTNRsSSFP | 0.055 | -0.272 | -1.656 | -0.624 |
| sp\|Q9HCU4\|CELR2_HUMAN Cadherin EGF LAG seven-pass G-type receptor 2 OS=Homo sapiens GN=CELSR2 I | 1039 | YVTNRSsSFPG | 0.122 | -0.02 | -0.669 | -0.189 |
| sp\|Q9HCU4\|CELR2_HUMAN Cadherin EGF LAG seven-pass G-type receptor 2 OS=Homo sapiens GN=CELSR2 I | 1040 | VTNRSSsFPGG | 0.93 | 1.456 | 1.416 | 1.267 |
| sp\|Q9HCU4\|CELR2_HUMAN Cadherin EGF LAG seven-pass G-type receptor 2 OS=Homo sapiens GN=CELSR2 I | 1057 | AHDPDIsDSLT | 0.08 | -0.28 | -1.647 | -0.616 |
| sp\|Q9HCU4\|CELR2_HUMAN Cadherin EGF LAG seven-pass G-type receptor 2 OS=Homo sapiens GN=CELSR2 I | 1059 | DPDISDsLTYS | 0.402 | 0.05 | -0.733 | -0.094 |
| sp\|Q9HCU4\|CELR2_HUMAN Cadherin EGF LAG seven-pass G-type receptor 2 OS=Homo sapiens GN=CELSR2 I | 1061 | DISDSLtYSFE | 0.129 | -0.043 | -1.165 | -0.36 |
| sp\|Q9HCU4\|CELR2_HUMAN Cadherin EGF LAG seven-pass G-type receptor 2 OS=Homo sapiens GN=CELSR2 I | 1063 | SDSLTYsFERG | 0.084 | -0.117 | -1.219 | -0.417 |
| sp\|Q9HCU4\|CELR2_HUMAN Cadherin EGF LAG seven-pass G-type receptor 2 OS=Homo sapiens GN=CELSR2 I | 1071 | ERGNELsLVLL | 0.128 | 0.212 | -0.676 | -0.112 |
| sp\|Q9HCU4\|CELR2_HUMAN Cadherin EGF LAG seven-pass G-type receptor 2 OS=Homo sapiens GN=CELSR2 I | 1078 | LVLLNAsTGEL | 0.328 | 0.086 | -0.401 | 0.004 |
| sp\|Q9HCU4\|CELR2_HUMAN Cadherin EGF LAG seven-pass G-type receptor 2 OS=Homo sapiens GN=CELSR2 I | 1079 | VLLNAStGELK | 0.153 | -0.074 | -0.665 | -0.195 |
| sp\|Q9HCU4\|CELR2_HUMAN Cadherin EGF LAG seven-pass G-type receptor 2 OS=Homo sapiens GN=CELSR2 I | 1085 | TGELKLsRALD | 0.075 | -0.303 | -1.347 | -0.525 |
| sp\|Q9HCU4\|CELR2_HUMAN Cadherin EGF LAG seven-pass G-type receptor 2 OS=Homo sapiens GN=CELSR2 I | 1099 | PLEAIMsVLVS | 0.323 | 0.163 | -0.347 | 0.046 |
| sp\|Q9HCU4\|CELR2_HUMAN Cadherin EGF LAG seven-pass G-type receptor 2 OS=Homo sapiens GN=CELSR2 I | 1103 | IMSVLVsDGVH | 0.168 | -0.04 | -1.101 | -0.324 |
| sp\|Q9HCU4\|CELR2_HUMAN Cadherin EGF LAG seven-pass G-type receptor 2 OS=Homo sapiens GN=CELSR2 I | 1108 | VSDGVHsVTAQ | 0.247 | -0.023 | -0.763 | -0.18 |
| sp\|Q9HCU4\|CELR2_HUMAN Cadherin EGF LAG seven-pass G-type receptor 2 OS=Homo sapiens GN=CELSR2 I | 1110 | DGVHSVtAQCA | 0.552 | 0.215 | -0.173 | 0.198 |
| sp\|Q9HCU4\|CELR2_HUMAN Cadherin EGF LAG seven-pass G-type receptor 2 OS=Homo sapiens GN=CELSR2 I | 1118 | QCALRVtIITD | 0.432 | 0.11 | -0.192 | 0.117 |
| sp\|Q9HCU4\|CELR2_HUMAN Cadherin EGF LAG seven-pass G-type receptor 2 OS=Homo sapiens GN=CELSR2 I | 1121 | LRVTIItDEML | 0.22 | 0.159 | -0.686 | -0.102 |
| sp\|Q9HCU4\|CELR2_HUMAN Cadherin EGF LAG seven-pass G-type receptor 2 OS=Homo sapiens GN=CELSR2 I | 1126 | ITDEMLtHSIT | 0.077 | -0.333 | -1.368 | -0.541 |
| sp\|Q9HCU4\|CELR2_HUMAN Cadherin EGF LAG seven-pass G-type receptor 2 OS=Homo sapiens GN=CELSR2 I | 1128 | DEMLTHsITLR | 0.21 | 0.094 | -0.917 | -0.204 |
| sp\|Q9HCU4\|CELR2_HUMAN Cadherin EGF LAG seven-pass G-type receptor 2 OS=Homo sapiens GN=CELSR2 I | 1130 | MLTHSItLRLE | 0.775 | 0.37 | 0.588 | 0.578 |
| sp\|Q9HCU4\|CELR2_HUMAN Cadherin EGF LAG seven-pass G-type receptor 2 OS=Homo sapiens GN=CELSR2 I | 1137 | LRLEDMsPERF | 0.064 | -0.151 | -1.288 | -0.458 |
| sp\|Q9HCU4\|CELR2_HUMAN Cadherin EGF LAG seven-pass G-type receptor 2 OS=Homo sapiens GN=CELSR2 I | 1143 | SPERFLsPLLG | 0.137 | 0.36 | -0.913 | -0.139 |
| sp\|Q9HCU4\|CELR2_HUMAN Cadherin EGF LAG seven-pass G-type receptor 2 OS=Homo sapiens GN=CELSR2 I | 1156 | IQAVAAtLATP | 0.25 | -0.025 | -0.36 | -0.045 |

| | | | | | | |
|---|---|---|---|---|---|---|
| sp\|Q9HCU4\|CELR2_HUMAN Cadherin EGF LAG seven-pass G-type receptor 2 OS=Homo sapiens GN=CELSR2 I | 1159 | VAATLAtPPDH | 0.273 | -0.007 | -0.815 | -0.183 |
| sp\|Q9HCU4\|CELR2_HUMAN Cadherin EGF LAG seven-pass G-type receptor 2 OS=Homo sapiens GN=CELSR2 I | 1173 | FNVQRDtDAPG | 0.262 | -0.039 | -0.801 | -0.193 |
| sp\|Q9HCU4\|CELR2_HUMAN Cadherin EGF LAG seven-pass G-type receptor 2 OS=Homo sapiens GN=CELSR2 I | 1184 | GHILNVsLSVG | 0.432 | 0.094 | -0.627 | -0.034 |
| sp\|Q9HCU4\|CELR2_HUMAN Cadherin EGF LAG seven-pass G-type receptor 2 OS=Homo sapiens GN=CELSR2 I | 1186 | ILNVSLsVGQP | 0.318 | 0.167 | -0.297 | 0.063 |
| sp\|Q9HCU4\|CELR2_HUMAN Cadherin EGF LAG seven-pass G-type receptor 2 OS=Homo sapiens GN=CELSR2 I | 1202 | GPPFLPsEDLQ | 0.147 | -0.093 | -1.143 | -0.363 |
| sp\|Q9HCU4\|CELR2_HUMAN Cadherin EGF LAG seven-pass G-type receptor 2 OS=Homo sapiens GN=CELSR2 I | 1214 | RLYLNRsLLTA | 0.329 | 0.1 | -0.481 | -0.017 |
| sp\|Q9HCU4\|CELR2_HUMAN Cadherin EGF LAG seven-pass G-type receptor 2 OS=Homo sapiens GN=CELSR2 I | 1217 | LNRSLLtAISA | 0.07 | -0.068 | -1.139 | -0.379 |
| sp\|Q9HCU4\|CELR2_HUMAN Cadherin EGF LAG seven-pass G-type receptor 2 OS=Homo sapiens GN=CELSR2 I | 1220 | SLLTAIsAQRV | 0.404 | 0.031 | -0.9 | -0.155 |
| sp\|Q9HCU4\|CELR2_HUMAN Cadherin EGF LAG seven-pass G-type receptor 2 OS=Homo sapiens GN=CELSR2 I | 1245 | NYMRCVsVLRF | 0.748 | 0.862 | 0.418 | 0.676 |
| sp\|Q9HCU4\|CELR2_HUMAN Cadherin EGF LAG seven-pass G-type receptor 2 OS=Homo sapiens GN=CELSR2 I | 1251 | SVLRFDsSAPF | 0.188 | 0.504 | -0.699 | -0.002 |
| sp\|Q9HCU4\|CELR2_HUMAN Cadherin EGF LAG seven-pass G-type receptor 2 OS=Homo sapiens GN=CELSR2 I | 1252 | VLRFDSsAPFI | 0.745 | 0.716 | 0.355 | 0.605 |
| sp\|Q9HCU4\|CELR2_HUMAN Cadherin EGF LAG seven-pass G-type receptor 2 OS=Homo sapiens GN=CELSR2 I | 1258 | SAPFIAsSSVL | 0.066 | -0.177 | -1.407 | -0.506 |
| sp\|Q9HCU4\|CELR2_HUMAN Cadherin EGF LAG seven-pass G-type receptor 2 OS=Homo sapiens GN=CELSR2 I | 1259 | APFIASsSVLF | 0.084 | -0.127 | -1.317 | -0.453 |
| sp\|Q9HCU4\|CELR2_HUMAN Cadherin EGF LAG seven-pass G-type receptor 2 OS=Homo sapiens GN=CELSR2 I | 1260 | PFIASSsVLFR | 0.188 | 0.077 | -0.594 | -0.11 |
| sp\|Q9HCU4\|CELR2_HUMAN Cadherin EGF LAG seven-pass G-type receptor 2 OS=Homo sapiens GN=CELSR2 I | 1281 | RCPPGFtGDYC | 0.083 | -0.21 | -1.417 | -0.515 |
| sp\|Q9HCU4\|CELR2_HUMAN Cadherin EGF LAG seven-pass G-type receptor 2 OS=Homo sapiens GN=CELSR2 I | 1287 | TGDYCEtEVDL | 0.32 | 0.076 | -0.484 | -0.029 |
| sp\|Q9HCU4\|CELR2_HUMAN Cadherin EGF LAG seven-pass G-type receptor 2 OS=Homo sapiens GN=CELSR2 I | 1294 | EVDLCYsRPCG | 0.481 | 0.495 | -0.117 | 0.286 |
| sp\|Q9HCU4\|CELR2_HUMAN Cadherin EGF LAG seven-pass G-type receptor 2 OS=Homo sapiens GN=CELSR2 I | 1305 | PHGRCRsREGG | 0.48 | 0.71 | -0.317 | 0.291 |
| sp\|Q9HCU4\|CELR2_HUMAN Cadherin EGF LAG seven-pass G-type receptor 2 OS=Homo sapiens GN=CELSR2 I | 1311 | SREGGYtCLCR | 0.096 | 0.226 | -0.946 | -0.208 |
| sp\|Q9HCU4\|CELR2_HUMAN Cadherin EGF LAG seven-pass G-type receptor 2 OS=Homo sapiens GN=CELSR2 I | 1319 | LCRDGYtGEHC | 0.155 | 0.075 | -0.904 | -0.225 |
| sp\|Q9HCU4\|CELR2_HUMAN Cadherin EGF LAG seven-pass G-type receptor 2 OS=Homo sapiens GN=CELSR2 I | 1326 | GEHCEVsARSG | 0.216 | -0.16 | -1.299 | -0.414 |
| sp\|Q9HCU4\|CELR2_HUMAN Cadherin EGF LAG seven-pass G-type receptor 2 OS=Homo sapiens GN=CELSR2 I | 1329 | CEVSARsGRCT | 0.126 | -0.142 | -1.128 | -0.381 |
| sp\|Q9HCU4\|CELR2_HUMAN Cadherin EGF LAG seven-pass G-type receptor 2 OS=Homo sapiens GN=CELSR2 I | 1333 | ARSGRCtPGVC | 0.235 | 0 | -0.996 | -0.254 |
| sp\|Q9HCU4\|CELR2_HUMAN Cadherin EGF LAG seven-pass G-type receptor 2 OS=Homo sapiens GN=CELSR2 I | 1342 | VCKNGGtCVNL | 0.112 | 0.017 | -1.126 | -0.332 |
| sp\|Q9HCU4\|CELR2_HUMAN Cadherin EGF LAG seven-pass G-type receptor 2 OS=Homo sapiens GN=CELSR2 I | 1357 | FKCDCPsGDFE | 0.389 | -0.018 | -0.599 | -0.076 |
| sp\|Q9HCU4\|CELR2_HUMAN Cadherin EGF LAG seven-pass G-type receptor 2 OS=Homo sapiens GN=CELSR2 I | 1368 | KPYCQVtTRSF | 0.078 | -0.276 | -1.4 | -0.533 |
| sp\|Q9HCU4\|CELR2_HUMAN Cadherin EGF LAG seven-pass G-type receptor 2 OS=Homo sapiens GN=CELSR2 I | 1369 | PYCQVTtRSFP | 0.126 | -0.246 | -1.223 | -0.448 |
| sp\|Q9HCU4\|CELR2_HUMAN Cadherin EGF LAG seven-pass G-type receptor 2 OS=Homo sapiens GN=CELSR2 I | 1371 | CQVTTRsFPAH | 0.424 | 0.5 | -0.104 | 0.273 |
| sp\|Q9HCU4\|CELR2_HUMAN Cadherin EGF LAG seven-pass G-type receptor 2 OS=Homo sapiens GN=CELSR2 I | 1376 | RSFPAHsFITF | 0.349 | 0.137 | -0.282 | 0.068 |
| sp\|Q9HCU4\|CELR2_HUMAN Cadherin EGF LAG seven-pass G-type receptor 2 OS=Homo sapiens GN=CELSR2 I | 1379 | PAHSFItFRGL | 0.21 | -0.171 | -0.802 | -0.254 |
| sp\|Q9HCU4\|CELR2_HUMAN Cadherin EGF LAG seven-pass G-type receptor 2 OS=Homo sapiens GN=CELSR2 I | 1390 | RQRFHFtLALS | 0.594 | 0.394 | 0.084 | 0.357 |
| sp\|Q9HCU4\|CELR2_HUMAN Cadherin EGF LAG seven-pass G-type receptor 2 OS=Homo sapiens GN=CELSR2 I | 1394 | HFTLALsFATK | 0.235 | 0.033 | -0.404 | -0.045 |
| sp\|Q9HCU4\|CELR2_HUMAN Cadherin EGF LAG seven-pass G-type receptor 2 OS=Homo sapiens GN=CELSR2 I | 1397 | LALSFAtKERD | 0.187 | -0.205 | -0.998 | -0.339 |
| sp\|Q9HCU4\|CELR2_HUMAN Cadherin EGF LAG seven-pass G-type receptor 2 OS=Homo sapiens GN=CELSR2 I | 1429 | QEQVQLtFSAG | 0.08 | -0.022 | -1.176 | -0.373 |
| sp\|Q9HCU4\|CELR2_HUMAN Cadherin EGF LAG seven-pass G-type receptor 2 OS=Homo sapiens GN=CELSR2 I | 1431 | QVQLTFsAGES | 0.185 | 0.162 | -0.794 | -0.149 |
| sp\|Q9HCU4\|CELR2_HUMAN Cadherin EGF LAG seven-pass G-type receptor 2 OS=Homo sapiens GN=CELSR2 I | 1435 | TFSAGEsTTTV | 0.109 | -0.282 | -1.411 | -0.528 |
| sp\|Q9HCU4\|CELR2_HUMAN Cadherin EGF LAG seven-pass G-type receptor 2 OS=Homo sapiens GN=CELSR2 I | 1436 | FSAGEStTTVS | 0.092 | -0.165 | -1.248 | -0.44 |
| sp\|Q9HCU4\|CELR2_HUMAN Cadherin EGF LAG seven-pass G-type receptor 2 OS=Homo sapiens GN=CELSR2 I | 1437 | SAGESTtTVSP | 0.046 | -0.103 | -1.474 | -0.51 |
| sp\|Q9HCU4\|CELR2_HUMAN Cadherin EGF LAG seven-pass G-type receptor 2 OS=Homo sapiens GN=CELSR2 I | 1438 | AGESTTtVSPF | 0.061 | -0.266 | -1.236 | -0.48 |
| sp\|Q9HCU4\|CELR2_HUMAN Cadherin EGF LAG seven-pass G-type receptor 2 OS=Homo sapiens GN=CELSR2 I | 1440 | ESTTTVsPFVP | 0.062 | -0.404 | -1.298 | -0.547 |
| sp\|Q9HCU4\|CELR2_HUMAN Cadherin EGF LAG seven-pass G-type receptor 2 OS=Homo sapiens GN=CELSR2 I | 1448 | FVPGGVsDGQW | 0.194 | 0.002 | -0.753 | -0.186 |
| sp\|Q9HCU4\|CELR2_HUMAN Cadherin EGF LAG seven-pass G-type receptor 2 OS=Homo sapiens GN=CELSR2 I | 1454 | SDGQWHtVQLK | 0.154 | -0.035 | -0.832 | -0.238 |
| sp\|Q9HCU4\|CELR2_HUMAN Cadherin EGF LAG seven-pass G-type receptor 2 OS=Homo sapiens GN=CELSR2 I | 1468 | KPLLGQtGLPQ | 0.037 | -0.359 | -1.649 | -0.657 |
| sp\|Q9HCU4\|CELR2_HUMAN Cadherin EGF LAG seven-pass G-type receptor 2 OS=Homo sapiens GN=CELSR2 I | 1475 | GLPQGPsEQKV | 0.222 | -0.016 | -0.899 | -0.231 |
| sp\|Q9HCU4\|CELR2_HUMAN Cadherin EGF LAG seven-pass G-type receptor 2 OS=Homo sapiens GN=CELSR2 I | 1483 | QKVAVVtVDGC | 0.413 | -0.07 | -0.484 | -0.047 |
| sp\|Q9HCU4\|CELR2_HUMAN Cadherin EGF LAG seven-pass G-type receptor 2 OS=Homo sapiens GN=CELSR2 I | 1489 | TVDGCDtGVAL | 0.13 | -0.095 | -0.906 | -0.29 |
| sp\|Q9HCU4\|CELR2_HUMAN Cadherin EGF LAG seven-pass G-type receptor 2 OS=Homo sapiens GN=CELSR2 I | 1497 | VALRFGsVLGN | 0.357 | 0.616 | -0.304 | 0.223 |
| sp\|Q9HCU4\|CELR2_HUMAN Cadherin EGF LAG seven-pass G-type receptor 2 OS=Homo sapiens GN=CELSR2 I | 1503 | SVLGNYsCAAQ | 0.114 | 0.026 | -0.778 | -0.213 |
| sp\|Q9HCU4\|CELR2_HUMAN Cadherin EGF LAG seven-pass G-type receptor 2 OS=Homo sapiens GN=CELSR2 I | 1509 | SCAAQGtQGGS | 0.084 | -0.253 | -1.566 | -0.578 |

| | | | | | | |
|---|---|---|---|---|---|---|
| sp\|Q9HCU4\|CELR2_HUMAN Cadherin EGF LAG seven-pass G-type receptor 2 OS=Homo sapiens GN=CELSR2 I | 1513 | QGTQGGsKKSL | 0.055 | -0.245 | -1.068 | -0.419 |
| sp\|Q9HCU4\|CELR2_HUMAN Cadherin EGF LAG seven-pass G-type receptor 2 OS=Homo sapiens GN=CELSR2 I | 1516 | QGGSKKsLDLT | 0.196 | -0.042 | -0.394 | -0.08 |
| sp\|Q9HCU4\|CELR2_HUMAN Cadherin EGF LAG seven-pass G-type receptor 2 OS=Homo sapiens GN=CELSR2 I | 1520 | KKSLDLtGPLL | 0.436 | 0.396 | -0.186 | 0.215 |
| sp\|Q9HCU4\|CELR2_HUMAN Cadherin EGF LAG seven-pass G-type receptor 2 OS=Homo sapiens GN=CELSR2 I | 1534 | VPDLPEsFPVR | 0.434 | 0.331 | -0.309 | 0.152 |
| sp\|Q9HCU4\|CELR2_HUMAN Cadherin EGF LAG seven-pass G-type receptor 2 OS=Homo sapiens GN=CELSR2 I | 1553 | RNLQVDsRHID | 0.288 | -0.208 | -0.851 | -0.257 |
| sp\|Q9HCU4\|CELR2_HUMAN Cadherin EGF LAG seven-pass G-type receptor 2 OS=Homo sapiens GN=CELSR2 I | 1567 | FIANNGtVPGC | 0.443 | 0.429 | 0.019 | 0.297 |
| sp\|Q9HCU4\|CELR2_HUMAN Cadherin EGF LAG seven-pass G-type receptor 2 OS=Homo sapiens GN=CELSR2 I | 1580 | KKNVCDsNTCH | 0.178 | -0.218 | -1.501 | -0.514 |
| sp\|Q9HCU4\|CELR2_HUMAN Cadherin EGF LAG seven-pass G-type receptor 2 OS=Homo sapiens GN=CELSR2 I | 1582 | NVCDSNtCHNG | 0.576 | 0.409 | -0.39 | 0.198 |
| sp\|Q9HCU4\|CELR2_HUMAN Cadherin EGF LAG seven-pass G-type receptor 2 OS=Homo sapiens GN=CELSR2 I | 1588 | TCHNGGtCVNQ | 0.048 | -0.147 | -1.739 | -0.613 |
| sp\|Q9HCU4\|CELR2_HUMAN Cadherin EGF LAG seven-pass G-type receptor 2 OS=Homo sapiens GN=CELSR2 I | 1597 | NQWDAFsCECP | 0.158 | 0.244 | -0.771 | -0.123 |
| sp\|Q9HCU4\|CELR2_HUMAN Cadherin EGF LAG seven-pass G-type receptor 2 OS=Homo sapiens GN=CELSR2 I | 1608 | LGFGGKsCAQE | 0.129 | -0.095 | -0.721 | -0.229 |
| sp\|Q9HCU4\|CELR2_HUMAN Cadherin EGF LAG seven-pass G-type receptor 2 OS=Homo sapiens GN=CELSR2 I | 1622 | PQHFLGsSLVA | 0.045 | -0.24 | -1.574 | -0.59 |
| sp\|Q9HCU4\|CELR2_HUMAN Cadherin EGF LAG seven-pass G-type receptor 2 OS=Homo sapiens GN=CELSR2 I | 1623 | QHFLGSsLVAW | 0.105 | -0.007 | -1.091 | -0.331 |
| sp\|Q9HCU4\|CELR2_HUMAN Cadherin EGF LAG seven-pass G-type receptor 2 OS=Homo sapiens GN=CELSR2 I | 1631 | VAWHGLsLPIS | 0.34 | 0.422 | -0.25 | 0.171 |
| sp\|Q9HCU4\|CELR2_HUMAN Cadherin EGF LAG seven-pass G-type receptor 2 OS=Homo sapiens GN=CELSR2 I | 1635 | GLSLPIsQPWY | 0.718 | 0.486 | 0.071 | 0.425 |
| sp\|Q9HCU4\|CELR2_HUMAN Cadherin EGF LAG seven-pass G-type receptor 2 OS=Homo sapiens GN=CELSR2 I | 1641 | SQPWYLsLMFR | 0.113 | -0.193 | -0.948 | -0.343 |
| sp\|Q9HCU4\|CELR2_HUMAN Cadherin EGF LAG seven-pass G-type receptor 2 OS=Homo sapiens GN=CELSR2 I | 1646 | LSLMFRtRQAD | 0.177 | -0.163 | -0.868 | -0.285 |
| sp\|Q9HCU4\|CELR2_HUMAN Cadherin EGF LAG seven-pass G-type receptor 2 OS=Homo sapiens GN=CELSR2 I | 1658 | VLLQAItRGRS | 0.46 | -0.035 | -0.413 | 0.004 |
| sp\|Q9HCU4\|CELR2_HUMAN Cadherin EGF LAG seven-pass G-type receptor 2 OS=Homo sapiens GN=CELSR2 I | 1662 | AITRGRsTITL | 0.373 | 0.804 | -0.03 | 0.382 |
| sp\|Q9HCU4\|CELR2_HUMAN Cadherin EGF LAG seven-pass G-type receptor 2 OS=Homo sapiens GN=CELSR2 I | 1663 | ITRGRStITLQ | 0.414 | 0.175 | -0.4 | 0.063 |
| sp\|Q9HCU4\|CELR2_HUMAN Cadherin EGF LAG seven-pass G-type receptor 2 OS=Homo sapiens GN=CELSR2 I | 1665 | RGRSTItLQLR | 0.581 | 0.342 | 0.144 | 0.356 |
| sp\|Q9HCU4\|CELR2_HUMAN Cadherin EGF LAG seven-pass G-type receptor 2 OS=Homo sapiens GN=CELSR2 I | 1676 | EGHVMLsVEGT | 0.036 | -0.331 | -1.358 | -0.551 |
| sp\|Q9HCU4\|CELR2_HUMAN Cadherin EGF LAG seven-pass G-type receptor 2 OS=Homo sapiens GN=CELSR2 I | 1680 | MLSVEGtGLQA | 0.11 | -0.165 | -1.157 | -0.404 |
| sp\|Q9HCU4\|CELR2_HUMAN Cadherin EGF LAG seven-pass G-type receptor 2 OS=Homo sapiens GN=CELSR2 I | 1685 | GTGLQAsSLRL | 0.111 | -0.123 | -1.546 | -0.519 |
| sp\|Q9HCU4\|CELR2_HUMAN Cadherin EGF LAG seven-pass G-type receptor 2 OS=Homo sapiens GN=CELSR2 I | 1686 | TGLQASsLRLE | 0.328 | -0.059 | -0.269 | 0 |
| sp\|Q9HCU4\|CELR2_HUMAN Cadherin EGF LAG seven-pass G-type receptor 2 OS=Homo sapiens GN=CELSR2 I | 1709 | QLALGAsGGPG | 0.215 | -0.047 | -0.611 | -0.148 |
| sp\|Q9HCU4\|CELR2_HUMAN Cadherin EGF LAG seven-pass G-type receptor 2 OS=Homo sapiens GN=CELSR2 I | 1718 | PGHAILsFDYG | 0.181 | -0.064 | -0.618 | -0.167 |
| sp\|Q9HCU4\|CELR2_HUMAN Cadherin EGF LAG seven-pass G-type receptor 2 OS=Homo sapiens GN=CELSR2 I | 1740 | LHGLHLsNITV | 0.063 | -0.144 | -1.739 | -0.607 |
| sp\|Q9HCU4\|CELR2_HUMAN Cadherin EGF LAG seven-pass G-type receptor 2 OS=Homo sapiens GN=CELSR2 I | 1743 | LHLSNItVGGI | 0.326 | -0.041 | -0.412 | -0.042 |
| sp\|Q9HCU4\|CELR2_HUMAN Cadherin EGF LAG seven-pass G-type receptor 2 OS=Homo sapiens GN=CELSR2 I | 1768 | LQGVRVsDTPE | 0.256 | -0.025 | -0.717 | -0.162 |
| sp\|Q9HCU4\|CELR2_HUMAN Cadherin EGF LAG seven-pass G-type receptor 2 OS=Homo sapiens GN=CELSR2 I | 1770 | GVRVSDtPEGV | 0.243 | 0.027 | -0.882 | -0.204 |
| sp\|Q9HCU4\|CELR2_HUMAN Cadherin EGF LAG seven-pass G-type receptor 2 OS=Homo sapiens GN=CELSR2 I | 1776 | TPEGVNsLDPS | 0.289 | -0.028 | -0.576 | -0.105 |
| sp\|Q9HCU4\|CELR2_HUMAN Cadherin EGF LAG seven-pass G-type receptor 2 OS=Homo sapiens GN=CELSR2 I | 1780 | VNSLDPsHGES | 0.156 | -0.241 | -1.025 | -0.37 |
| sp\|Q9HCU4\|CELR2_HUMAN Cadherin EGF LAG seven-pass G-type receptor 2 OS=Homo sapiens GN=CELSR2 I | 1784 | DPSHGEsINVE | 0.08 | -0.286 | -1.374 | -0.527 |
| sp\|Q9HCU4\|CELR2_HUMAN Cadherin EGF LAG seven-pass G-type receptor 2 OS=Homo sapiens GN=CELSR2 I | 1792 | NVEQGCsLPDP | 0.73 | 0.507 | 0.374 | 0.537 |
| sp\|Q9HCU4\|CELR2_HUMAN Cadherin EGF LAG seven-pass G-type receptor 2 OS=Homo sapiens GN=CELSR2 I | 1799 | LPDPCDsNPCP | 0.313 | 0.213 | -0.735 | -0.07 |
| sp\|Q9HCU4\|CELR2_HUMAN Cadherin EGF LAG seven-pass G-type receptor 2 OS=Homo sapiens GN=CELSR2 I | 1806 | NPCPANsYCSN | 0.119 | -0.165 | -1.274 | -0.44 |
| sp\|Q9HCU4\|CELR2_HUMAN Cadherin EGF LAG seven-pass G-type receptor 2 OS=Homo sapiens GN=CELSR2 I | 1809 | PANSYCsNDWD | 0.307 | -0.063 | -0.889 | -0.215 |
| sp\|Q9HCU4\|CELR2_HUMAN Cadherin EGF LAG seven-pass G-type receptor 2 OS=Homo sapiens GN=CELSR2 I | 1814 | CSNDWDsYSCS | 0.087 | -0.2 | -1.471 | -0.528 |
| sp\|Q9HCU4\|CELR2_HUMAN Cadherin EGF LAG seven-pass G-type receptor 2 OS=Homo sapiens GN=CELSR2 I | 1816 | NDWDSYsCSCD | 0.225 | 0.387 | -0.467 | 0.048 |
| sp\|Q9HCU4\|CELR2_HUMAN Cadherin EGF LAG seven-pass G-type receptor 2 OS=Homo sapiens GN=CELSR2 I | 1818 | WDSYSCsCDPG | 0.369 | 0.149 | -0.28 | 0.079 |
| sp\|Q9HCU4\|CELR2_HUMAN Cadherin EGF LAG seven-pass G-type receptor 2 OS=Homo sapiens GN=CELSR2 I | 1829 | YYGDNCtNVCD | 0.271 | 0.052 | -0.92 | -0.199 |
| sp\|Q9HCU4\|CELR2_HUMAN Cadherin EGF LAG seven-pass G-type receptor 2 OS=Homo sapiens GN=CELSR2 I | 1841 | NPCEHQsVCTR | 0.136 | -0.119 | -1.074 | -0.352 |
| sp\|Q9HCU4\|CELR2_HUMAN Cadherin EGF LAG seven-pass G-type receptor 2 OS=Homo sapiens GN=CELSR2 I | 1844 | EHQSVCtRKPS | 0.087 | -0.247 | -1.671 | -0.61 |
| sp\|Q9HCU4\|CELR2_HUMAN Cadherin EGF LAG seven-pass G-type receptor 2 OS=Homo sapiens GN=CELSR2 I | 1848 | VCTRKPsAPHG | 0.822 | 1.232 | 0.776 | 0.943 |
| sp\|Q9HCU4\|CELR2_HUMAN Cadherin EGF LAG seven-pass G-type receptor 2 OS=Homo sapiens GN=CELSR2 I | 1854 | SAPHGYtCECP | 0.035 | -0.167 | -1.624 | -0.585 |
| sp\|Q9HCU4\|CELR2_HUMAN Cadherin EGF LAG seven-pass G-type receptor 2 OS=Homo sapiens GN=CELSR2 I | 1868 | LGPYCEtRIDQ | 0.154 | -0.184 | -0.906 | -0.312 |
| sp\|Q9HCU4\|CELR2_HUMAN Cadherin EGF LAG seven-pass G-type receptor 2 OS=Homo sapiens GN=CELSR2 I | 1883 | GWWGHPtCGPC | 0.211 | 0.14 | -0.853 | -0.167 |
| sp\|Q9HCU4\|CELR2_HUMAN Cadherin EGF LAG seven-pass G-type receptor 2 OS=Homo sapiens GN=CELSR2 I | 1892 | PCNCDVsKGFD | 0.472 | -0.021 | -0.55 | -0.033 |

| | | | | | | |
|---|---|---|---|---|---|---|
| sp\|Q9HCU4\|CELR2_HUMAN Cadherin EGF LAG seven-pass G-type receptor 2 OS=Homo sapiens GN=CELSR2 I | 1902 | DPDCNKtSGEC | 0.352 | -0.11 | -0.849 | -0.202 |
| sp\|Q9HCU4\|CELR2_HUMAN Cadherin EGF LAG seven-pass G-type receptor 2 OS=Homo sapiens GN=CELSR2 I | 1903 | PDCNKTsGECH | 0.045 | -0.295 | -1.485 | -0.578 |
| sp\|Q9HCU4\|CELR2_HUMAN Cadherin EGF LAG seven-pass G-type receptor 2 OS=Homo sapiens GN=CELSR2 I | 1918 | HYRPPGsPTCL | 0.143 | -0.116 | -0.903 | -0.292 |
| sp\|Q9HCU4\|CELR2_HUMAN Cadherin EGF LAG seven-pass G-type receptor 2 OS=Homo sapiens GN=CELSR2 I | 1920 | RPPGSPtCLLC | 0.136 | 0.048 | -1.105 | -0.307 |
| sp\|Q9HCU4\|CELR2_HUMAN Cadherin EGF LAG seven-pass G-type receptor 2 OS=Homo sapiens GN=CELSR2 I | 1929 | LCDCYPtGSLS | 0.118 | -0.211 | -1.322 | -0.472 |
| sp\|Q9HCU4\|CELR2_HUMAN Cadherin EGF LAG seven-pass G-type receptor 2 OS=Homo sapiens GN=CELSR2 I | 1931 | DCYPTGsLSRV | 0.139 | -0.15 | -1.442 | -0.484 |
| sp\|Q9HCU4\|CELR2_HUMAN Cadherin EGF LAG seven-pass G-type receptor 2 OS=Homo sapiens GN=CELSR2 I | 1933 | YPTGSLsRVCD | 0.174 | 0.098 | -0.607 | -0.112 |
| sp\|Q9HCU4\|CELR2_HUMAN Cadherin EGF LAG seven-pass G-type receptor 2 OS=Homo sapiens GN=CELSR2 I | 1965 | NPFAEVtTNGC | 0.224 | -0.229 | -0.997 | -0.334 |
| sp\|Q9HCU4\|CELR2_HUMAN Cadherin EGF LAG seven-pass G-type receptor 2 OS=Homo sapiens GN=CELSR2 I | 1966 | PFAEVTtNGCE | 0.214 | 0.002 | -0.752 | -0.179 |
| sp\|Q9HCU4\|CELR2_HUMAN Cadherin EGF LAG seven-pass G-type receptor 2 OS=Homo sapiens GN=CELSR2 I | 1975 | CEVNYDsCPRA | 0.532 | 0.378 | -0.255 | 0.218 |
| sp\|Q9HCU4\|CELR2_HUMAN Cadherin EGF LAG seven-pass G-type receptor 2 OS=Homo sapiens GN=CELSR2 I | 1989 | GIWWPRtRFGL | 0.182 | -0.002 | -0.749 | -0.19 |
| sp\|Q9HCU4\|CELR2_HUMAN Cadherin EGF LAG seven-pass G-type receptor 2 OS=Homo sapiens GN=CELSR2 I | 2003 | APCPKGsFGTA | 0.145 | -0.221 | -1.035 | -0.37 |
| sp\|Q9HCU4\|CELR2_HUMAN Cadherin EGF LAG seven-pass G-type receptor 2 OS=Homo sapiens GN=CELSR2 I | 2006 | PKGSFGtAVRH | 0.102 | -0.275 | -1.652 | -0.608 |
| sp\|Q9HCU4\|CELR2_HUMAN Cadherin EGF LAG seven-pass G-type receptor 2 OS=Homo sapiens GN=CELSR2 I | 2026 | PNLFNCtSITF | 0.165 | -0.149 | -0.799 | -0.261 |
| sp\|Q9HCU4\|CELR2_HUMAN Cadherin EGF LAG seven-pass G-type receptor 2 OS=Homo sapiens GN=CELSR2 I | 2027 | NLFNCTsITFS | 0.22 | -0.054 | -0.632 | -0.155 |
| sp\|Q9HCU4\|CELR2_HUMAN Cadherin EGF LAG seven-pass G-type receptor 2 OS=Homo sapiens GN=CELSR2 I | 2029 | FNCTSItFSEL | 0.483 | 0.238 | -0.159 | 0.187 |
| sp\|Q9HCU4\|CELR2_HUMAN Cadherin EGF LAG seven-pass G-type receptor 2 OS=Homo sapiens GN=CELSR2 I | 2031 | CTSITFsELKG | 0.142 | 0.06 | -0.885 | -0.228 |
| sp\|Q9HCU4\|CELR2_HUMAN Cadherin EGF LAG seven-pass G-type receptor 2 OS=Homo sapiens GN=CELSR2 I | 2045 | RLQRNEsGLDS | 0.733 | 0.943 | 0.377 | 0.684 |
| sp\|Q9HCU4\|CELR2_HUMAN Cadherin EGF LAG seven-pass G-type receptor 2 OS=Homo sapiens GN=CELSR2 I | 2049 | NESGLDsGRSQ | 0.045 | -0.433 | -1.794 | -0.727 |
| sp\|Q9HCU4\|CELR2_HUMAN Cadherin EGF LAG seven-pass G-type receptor 2 OS=Homo sapiens GN=CELSR2 I | 2052 | GLDSGRsQQLA | 0.134 | -0.121 | -1.263 | -0.417 |
| sp\|Q9HCU4\|CELR2_HUMAN Cadherin EGF LAG seven-pass G-type receptor 2 OS=Homo sapiens GN=CELSR2 I | 2063 | LLLRNAtQHTA | 0.776 | 0.884 | 0.596 | 0.752 |
| sp\|Q9HCU4\|CELR2_HUMAN Cadherin EGF LAG seven-pass G-type receptor 2 OS=Homo sapiens GN=CELSR2 I | 2066 | RNATQHtAGYF | 0.206 | 0.043 | -0.893 | -0.215 |
| sp\|Q9HCU4\|CELR2_HUMAN Cadherin EGF LAG seven-pass G-type receptor 2 OS=Homo sapiens GN=CELSR2 I | 2072 | TAGYFGsDVKV | 0.071 | -0.199 | -1.709 | -0.612 |
| sp\|Q9HCU4\|CELR2_HUMAN Cadherin EGF LAG seven-pass G-type receptor 2 OS=Homo sapiens GN=CELSR2 I | 2082 | VAYQLAtRLLA | 0.1 | -0.256 | -1.28 | -0.479 |
| sp\|Q9HCU4\|CELR2_HUMAN Cadherin EGF LAG seven-pass G-type receptor 2 OS=Homo sapiens GN=CELSR2 I | 2089 | RLLAHEsTQRG | 0.414 | -0.001 | -0.69 | -0.092 |
| sp\|Q9HCU4\|CELR2_HUMAN Cadherin EGF LAG seven-pass G-type receptor 2 OS=Homo sapiens GN=CELSR2 I | 2090 | LLAHEStQRGF | 0.11 | -0.264 | -1.059 | -0.404 |
| sp\|Q9HCU4\|CELR2_HUMAN Cadherin EGF LAG seven-pass G-type receptor 2 OS=Homo sapiens GN=CELSR2 I | 2097 | QRGFGLsATQD | 0.144 | 0.086 | -0.945 | -0.238 |
| sp\|Q9HCU4\|CELR2_HUMAN Cadherin EGF LAG seven-pass G-type receptor 2 OS=Homo sapiens GN=CELSR2 I | 2099 | GFGLSAtQDVH | 0.369 | 0.161 | -0.657 | -0.042 |
| sp\|Q9HCU4\|CELR2_HUMAN Cadherin EGF LAG seven-pass G-type receptor 2 OS=Homo sapiens GN=CELSR2 I | 2105 | TQDVHFtENLL | 0.178 | 0.116 | -0.55 | -0.085 |
| sp\|Q9HCU4\|CELR2_HUMAN Cadherin EGF LAG seven-pass G-type receptor 2 OS=Homo sapiens GN=CELSR2 I | 2113 | NLLRVGsALLD | 0.67 | 0.91 | 0.215 | 0.598 |
| sp\|Q9HCU4\|CELR2_HUMAN Cadherin EGF LAG seven-pass G-type receptor 2 OS=Homo sapiens GN=CELSR2 I | 2118 | GSALLDtANKR | 0.173 | -0.138 | -1.126 | -0.364 |
| sp\|Q9HCU4\|CELR2_HUMAN Cadherin EGF LAG seven-pass G-type receptor 2 OS=Homo sapiens GN=CELSR2 I | 2130 | WELIQQtEGGT | 0.15 | -0.049 | -0.742 | -0.214 |
| sp\|Q9HCU4\|CELR2_HUMAN Cadherin EGF LAG seven-pass G-type receptor 2 OS=Homo sapiens GN=CELSR2 I | 2134 | QQTEGGtAWLL | 0.065 | -0.091 | -1.259 | -0.428 |
| sp\|Q9HCU4\|CELR2_HUMAN Cadherin EGF LAG seven-pass G-type receptor 2 OS=Homo sapiens GN=CELSR2 I | 2146 | HYEAYAsALAQ | 0.315 | 0.106 | -0.587 | -0.055 |
| sp\|Q9HCU4\|CELR2_HUMAN Cadherin EGF LAG seven-pass G-type receptor 2 OS=Homo sapiens GN=CELSR2 I | 2155 | AQNMRHtYLSP | 0.089 | -0.058 | -1.195 | -0.388 |
| sp\|Q9HCU4\|CELR2_HUMAN Cadherin EGF LAG seven-pass G-type receptor 2 OS=Homo sapiens GN=CELSR2 I | 2158 | MRHTYLsPFTI | 0.141 | -0.067 | -0.929 | -0.285 |
| sp\|Q9HCU4\|CELR2_HUMAN Cadherin EGF LAG seven-pass G-type receptor 2 OS=Homo sapiens GN=CELSR2 I | 2161 | TYLSPFtIVTP | 0.07 | -0.259 | -1.071 | -0.42 |
| sp\|Q9HCU4\|CELR2_HUMAN Cadherin EGF LAG seven-pass G-type receptor 2 OS=Homo sapiens GN=CELSR2 I | 2164 | SPFTIVtPNIV | 0.129 | -0.397 | -1.343 | -0.537 |
| sp\|Q9HCU4\|CELR2_HUMAN Cadherin EGF LAG seven-pass G-type receptor 2 OS=Homo sapiens GN=CELSR2 I | 2170 | TPNIVIsVVRL | 0.131 | -0.139 | -1.252 | -0.42 |
| sp\|Q9HCU4\|CELR2_HUMAN Cadherin EGF LAG seven-pass G-type receptor 2 OS=Homo sapiens GN=CELSR2 I | 2200 | QPPDLEtTVIL | 0.067 | -0.227 | -1.671 | -0.61 |
| sp\|Q9HCU4\|CELR2_HUMAN Cadherin EGF LAG seven-pass G-type receptor 2 OS=Homo sapiens GN=CELSR2 I | 2201 | PPDLETtVILP | 0.085 | -0.162 | -1.231 | -0.436 |
| sp\|Q9HCU4\|CELR2_HUMAN Cadherin EGF LAG seven-pass G-type receptor 2 OS=Homo sapiens GN=CELSR2 I | 2207 | TVILPEsVFRE | 0.084 | -0.259 | -1.379 | -0.518 |
| sp\|Q9HCU4\|CELR2_HUMAN Cadherin EGF LAG seven-pass G-type receptor 2 OS=Homo sapiens GN=CELSR2 I | 2212 | ESVFREtPPVV | 0.391 | 0.254 | -0.669 | -0.008 |
| sp\|Q9HCU4\|CELR2_HUMAN Cadherin EGF LAG seven-pass G-type receptor 2 OS=Homo sapiens GN=CELSR2 I | 2241 | RRHPELsQGEA | 0.164 | 0.039 | -1.13 | -0.309 |
| sp\|Q9HCU4\|CELR2_HUMAN Cadherin EGF LAG seven-pass G-type receptor 2 OS=Homo sapiens GN=CELSR2 I | 2248 | QGEAVAsVIIY | 0.303 | -0.044 | -0.553 | -0.098 |
| sp\|Q9HCU4\|CELR2_HUMAN Cadherin EGF LAG seven-pass G-type receptor 2 OS=Homo sapiens GN=CELSR2 I | 2254 | SVIIYRtLAGL | 0.121 | -0.017 | -0.99 | -0.295 |
| sp\|Q9HCU4\|CELR2_HUMAN Cadherin EGF LAG seven-pass G-type receptor 2 OS=Homo sapiens GN=CELSR2 I | 2269 | YDPDKRsLRVP | 0.059 | -0.241 | -1.412 | -0.531 |
| sp\|Q9HCU4\|CELR2_HUMAN Cadherin EGF LAG seven-pass G-type receptor 2 OS=Homo sapiens GN=CELSR2 I | 2280 | KRPIINtPVVS | 0.066 | -0.131 | -1.386 | -0.484 |
| sp\|Q9HCU4\|CELR2_HUMAN Cadherin EGF LAG seven-pass G-type receptor 2 OS=Homo sapiens GN=CELSR2 I | 2284 | INTPVVsISVH | 0.098 | -0.37 | -1.159 | -0.477 |

| | | | | | | |
|---|---|---|---|---|---|---|
| sp\|Q9HCU4\|CELR2_HUMAN Cadherin EGF LAG seven-pass G-type receptor 2 OS=Homo sapiens GN=CELSR2 I | 2286 | TPVVSIsVHDD | 0.428 | 0.205 | -0.245 | 0.129 |
| sp\|Q9HCU4\|CELR2_HUMAN Cadherin EGF LAG seven-pass G-type receptor 2 OS=Homo sapiens GN=CELSR2 I | 2303 | ALDKPVtVQFR | 0.426 | -0.059 | -0.364 | 0.001 |
| sp\|Q9HCU4\|CELR2_HUMAN Cadherin EGF LAG seven-pass G-type receptor 2 OS=Homo sapiens GN=CELSR2 I | 2311 | QFRLLEtEERT | 0.232 | 0.065 | -0.898 | -0.2 |
| sp\|Q9HCU4\|CELR2_HUMAN Cadherin EGF LAG seven-pass G-type receptor 2 OS=Homo sapiens GN=CELSR2 I | 2315 | LETEERtKPIC | 0.251 | 0.272 | -0.835 | -0.104 |
| sp\|Q9HCU4\|CELR2_HUMAN Cadherin EGF LAG seven-pass G-type receptor 2 OS=Homo sapiens GN=CELSR2 I | 2325 | CVFWNHsILVS | 0.216 | 0.057 | -0.582 | -0.103 |
| sp\|Q9HCU4\|CELR2_HUMAN Cadherin EGF LAG seven-pass G-type receptor 2 OS=Homo sapiens GN=CELSR2 I | 2329 | NHSILVsGTGG | 0.185 | -0.208 | -1.165 | -0.396 |
| sp\|Q9HCU4\|CELR2_HUMAN Cadherin EGF LAG seven-pass G-type receptor 2 OS=Homo sapiens GN=CELSR2 I | 2331 | SILVSGtGGWS | 0.136 | 0.078 | -0.786 | -0.191 |
| sp\|Q9HCU4\|CELR2_HUMAN Cadherin EGF LAG seven-pass G-type receptor 2 OS=Homo sapiens GN=CELSR2 I | 2335 | SGTGGWsARGC | 0.07 | -0.357 | -1.402 | -0.563 |
| sp\|Q9HCU4\|CELR2_HUMAN Cadherin EGF LAG seven-pass G-type receptor 2 OS=Homo sapiens GN=CELSR2 I | 2347 | VVFRNEsHVSC | 0.329 | 0.665 | -0.371 | 0.208 |
| sp\|Q9HCU4\|CELR2_HUMAN Cadherin EGF LAG seven-pass G-type receptor 2 OS=Homo sapiens GN=CELSR2 I | 2350 | RNESHVsCQCN | 0.402 | 0.068 | -0.579 | -0.036 |
| sp\|Q9HCU4\|CELR2_HUMAN Cadherin EGF LAG seven-pass G-type receptor 2 OS=Homo sapiens GN=CELSR2 I | 2357 | CQCNHMtSFAV | 0.385 | 0.217 | -0.253 | 0.116 |
| sp\|Q9HCU4\|CELR2_HUMAN Cadherin EGF LAG seven-pass G-type receptor 2 OS=Homo sapiens GN=CELSR2 I | 2358 | QCNHMTsFAVL | 0.234 | -0.012 | -0.718 | -0.165 |
| sp\|Q9HCU4\|CELR2_HUMAN Cadherin EGF LAG seven-pass G-type receptor 2 OS=Homo sapiens GN=CELSR2 I | 2366 | AVLMDVsRREN | 0.249 | -0.249 | -0.9 | -0.3 |
| sp\|Q9HCU4\|CELR2_HUMAN Cadherin EGF LAG seven-pass G-type receptor 2 OS=Homo sapiens GN=CELSR2 I | 2378 | EILPLKtLTYV | 0.336 | 0.037 | -0.555 | -0.061 |
| sp\|Q9HCU4\|CELR2_HUMAN Cadherin EGF LAG seven-pass G-type receptor 2 OS=Homo sapiens GN=CELSR2 I | 2380 | LPLKTLtYVAL | 0.115 | 0.02 | -0.748 | -0.204 |
| sp\|Q9HCU4\|CELR2_HUMAN Cadherin EGF LAG seven-pass G-type receptor 2 OS=Homo sapiens GN=CELSR2 I | 2387 | YVALGVtLAAL | 0.29 | 0.071 | -0.219 | 0.047 |
| sp\|Q9HCU4\|CELR2_HUMAN Cadherin EGF LAG seven-pass G-type receptor 2 OS=Homo sapiens GN=CELSR2 I | 2394 | LAALLLtFFFL | 0.087 | -0.134 | -1.022 | -0.356 |
| sp\|Q9HCU4\|CELR2_HUMAN Cadherin EGF LAG seven-pass G-type receptor 2 OS=Homo sapiens GN=CELSR2 I | 2399 | LTFFFLtLLRI | 0.27 | -0.037 | -0.759 | -0.175 |
| sp\|Q9HCU4\|CELR2_HUMAN Cadherin EGF LAG seven-pass G-type receptor 2 OS=Homo sapiens GN=CELSR2 I | 2406 | LLRILRsNQHG | 0.243 | 0.171 | -0.734 | -0.107 |
| sp\|Q9HCU4\|CELR2_HUMAN Cadherin EGF LAG seven-pass G-type receptor 2 OS=Homo sapiens GN=CELSR2 I | 2416 | GIRRNLtAALG | 0.819 | 1.174 | 0.647 | 0.88 |
| sp\|Q9HCU4\|CELR2_HUMAN Cadherin EGF LAG seven-pass G-type receptor 2 OS=Homo sapiens GN=CELSR2 I | 2440 | DLPFACtVIAI | 0.622 | 0.256 | -0.04 | 0.279 |
| sp\|Q9HCU4\|CELR2_HUMAN Cadherin EGF LAG seven-pass G-type receptor 2 OS=Homo sapiens GN=CELSR2 I | 2453 | HFLYLCtFSWA | 0.255 | 0.06 | -0.403 | -0.029 |
| sp\|Q9HCU4\|CELR2_HUMAN Cadherin EGF LAG seven-pass G-type receptor 2 OS=Homo sapiens GN=CELSR2 I | 2455 | LYLCTFsWALL | 0.177 | 0.154 | -0.636 | -0.102 |
| sp\|Q9HCU4\|CELR2_HUMAN Cadherin EGF LAG seven-pass G-type receptor 2 OS=Homo sapiens GN=CELSR2 I | 2469 | HLYRALtEVRD | 0.739 | 1.058 | 0.684 | 0.827 |
| sp\|Q9HCU4\|CELR2_HUMAN Cadherin EGF LAG seven-pass G-type receptor 2 OS=Homo sapiens GN=CELSR2 I | 2476 | EVRDVNtGPMR | 0.705 | 0.652 | 0.123 | 0.493 |
| sp\|Q9HCU4\|CELR2_HUMAN Cadherin EGF LAG seven-pass G-type receptor 2 OS=Homo sapiens GN=CELSR2 I | 2494 | GVPAFItGLAV | 0.13 | -0.117 | -1.241 | -0.409 |
| sp\|Q9HCU4\|CELR2_HUMAN Cadherin EGF LAG seven-pass G-type receptor 2 OS=Homo sapiens GN=CELSR2 I | 2514 | PDFCWLsIYDT | 0.138 | -0.122 | -0.914 | -0.299 |
| sp\|Q9HCU4\|CELR2_HUMAN Cadherin EGF LAG seven-pass G-type receptor 2 OS=Homo sapiens GN=CELSR2 I | 2518 | WLSIYDtLIWS | 0.29 | 0.033 | -0.365 | -0.014 |
| sp\|Q9HCU4\|CELR2_HUMAN Cadherin EGF LAG seven-pass G-type receptor 2 OS=Homo sapiens GN=CELSR2 I | 2522 | YDTLIWsFAGP | 0.107 | -0.242 | -0.834 | -0.323 |
| sp\|Q9HCU4\|CELR2_HUMAN Cadherin EGF LAG seven-pass G-type receptor 2 OS=Homo sapiens GN=CELSR2 I | 2532 | PVAFAVsMSVF | 0.28 | 0.151 | -0.532 | -0.034 |
| sp\|Q9HCU4\|CELR2_HUMAN Cadherin EGF LAG seven-pass G-type receptor 2 OS=Homo sapiens GN=CELSR2 I | 2534 | AFAVSMsVFLY | 0.52 | 0.365 | 0.103 | 0.329 |
| sp\|Q9HCU4\|CELR2_HUMAN Cadherin EGF LAG seven-pass G-type receptor 2 OS=Homo sapiens GN=CELSR2 I | 2545 | ILAARAsCAAQ | 0.599 | 0.259 | 0.008 | 0.289 |
| sp\|Q9HCU4\|CELR2_HUMAN Cadherin EGF LAG seven-pass G-type receptor 2 OS=Homo sapiens GN=CELSR2 I | 2560 | EKKGPVsGLQP | 0.183 | -0.184 | -0.575 | -0.192 |
| sp\|Q9HCU4\|CELR2_HUMAN Cadherin EGF LAG seven-pass G-type receptor 2 OS=Homo sapiens GN=CELSR2 I | 2565 | VSGLQPsFAVL | 0.091 | -0.148 | -1.23 | -0.429 |
| sp\|Q9HCU4\|CELR2_HUMAN Cadherin EGF LAG seven-pass G-type receptor 2 OS=Homo sapiens GN=CELSR2 I | 2573 | AVLLLLsATWL | 0.099 | -0.082 | -1.258 | -0.414 |
| sp\|Q9HCU4\|CELR2_HUMAN Cadherin EGF LAG seven-pass G-type receptor 2 OS=Homo sapiens GN=CELSR2 I | 2575 | LLLLSAtWLLA | 0.431 | 0.355 | -0.104 | 0.227 |
| sp\|Q9HCU4\|CELR2_HUMAN Cadherin EGF LAG seven-pass G-type receptor 2 OS=Homo sapiens GN=CELSR2 I | 2582 | WLLALLsVNSD | 0.138 | -0.073 | -0.667 | -0.201 |
| sp\|Q9HCU4\|CELR2_HUMAN Cadherin EGF LAG seven-pass G-type receptor 2 OS=Homo sapiens GN=CELSR2 I | 2585 | ALLSVNsDTLL | 0.474 | 0.09 | -0.627 | -0.021 |
| sp\|Q9HCU4\|CELR2_HUMAN Cadherin EGF LAG seven-pass G-type receptor 2 OS=Homo sapiens GN=CELSR2 I | 2587 | LSVNSDtLLFH | 0.126 | -0.105 | -0.899 | -0.293 |
| sp\|Q9HCU4\|CELR2_HUMAN Cadherin EGF LAG seven-pass G-type receptor 2 OS=Homo sapiens GN=CELSR2 I | 2596 | FHYLFAtCNCI | 0.348 | 0.258 | -0.714 | -0.036 |
| sp\|Q9HCU4\|CELR2_HUMAN Cadherin EGF LAG seven-pass G-type receptor 2 OS=Homo sapiens GN=CELSR2 I | 2608 | GPFIFLsYVVL | 0.091 | -0.05 | -1.305 | -0.421 |
| sp\|Q9HCU4\|CELR2_HUMAN Cadherin EGF LAG seven-pass G-type receptor 2 OS=Homo sapiens GN=CELSR2 I | 2613 | LSYVVLsKEVR | 0.062 | -0.17 | -1.195 | -0.434 |
| sp\|Q9HCU4\|CELR2_HUMAN Cadherin EGF LAG seven-pass G-type receptor 2 OS=Homo sapiens GN=CELSR2 I | 2625 | ALKLACsRKPS | 0.348 | -0.036 | -0.761 | -0.15 |
| sp\|Q9HCU4\|CELR2_HUMAN Cadherin EGF LAG seven-pass G-type receptor 2 OS=Homo sapiens GN=CELSR2 I | 2629 | ACSRKPsPDPA | 0.316 | 0.376 | -0.389 | 0.101 |
| sp\|Q9HCU4\|CELR2_HUMAN Cadherin EGF LAG seven-pass G-type receptor 2 OS=Homo sapiens GN=CELSR2 I | 2635 | SPDPALtTKST | 0.035 | -0.336 | -1.685 | -0.662 |
| sp\|Q9HCU4\|CELR2_HUMAN Cadherin EGF LAG seven-pass G-type receptor 2 OS=Homo sapiens GN=CELSR2 I | 2636 | PDPALTtKSTL | 0.055 | -0.303 | -1.375 | -0.541 |
| sp\|Q9HCU4\|CELR2_HUMAN Cadherin EGF LAG seven-pass G-type receptor 2 OS=Homo sapiens GN=CELSR2 I | 2638 | PALTTKsTLTS | 0.115 | -0.093 | -1.002 | -0.327 |
| sp\|Q9HCU4\|CELR2_HUMAN Cadherin EGF LAG seven-pass G-type receptor 2 OS=Homo sapiens GN=CELSR2 I | 2639 | ALTTKStLTSS | 0.157 | -0.103 | -0.941 | -0.296 |
| sp\|Q9HCU4\|CELR2_HUMAN Cadherin EGF LAG seven-pass G-type receptor 2 OS=Homo sapiens GN=CELSR2 I | 2641 | TTKSTLtSSYN | 0.053 | -0.249 | -1.599 | -0.598 |

| | | | | | | |
|---|---|---|---|---|---|---|
| sp\|Q9HCU4\|CELR2_HUMAN Cadherin EGF LAG seven-pass G-type receptor 2 OS=Homo sapiens GN=CELSR2 I | 2642 | TKSTLTsSYNC | 0.124 | -0.155 | -1.372 | -0.468 |
| sp\|Q9HCU4\|CELR2_HUMAN Cadherin EGF LAG seven-pass G-type receptor 2 OS=Homo sapiens GN=CELSR2 I | 2643 | KSTLTSsYNCP | 0.047 | -0.202 | -1.369 | -0.508 |
| sp\|Q9HCU4\|CELR2_HUMAN Cadherin EGF LAG seven-pass G-type receptor 2 OS=Homo sapiens GN=CELSR2 I | 2648 | SSYNCPsPYAD | 0.081 | -0.39 | -1.088 | -0.466 |
| sp\|Q9HCU4\|CELR2_HUMAN Cadherin EGF LAG seven-pass G-type receptor 2 OS=Homo sapiens GN=CELSR2 I | 2662 | YQPYGDsAGSL | 0.059 | -0.139 | -1.21 | -0.43 |
| sp\|Q9HCU4\|CELR2_HUMAN Cadherin EGF LAG seven-pass G-type receptor 2 OS=Homo sapiens GN=CELSR2 I | 2665 | YGDSAGsLHST | 0.149 | -0.126 | -0.833 | -0.27 |
| sp\|Q9HCU4\|CELR2_HUMAN Cadherin EGF LAG seven-pass G-type receptor 2 OS=Homo sapiens GN=CELSR2 I | 2668 | SAGSLHsTSRS | 0.038 | -0.33 | -2.004 | -0.765 |
| sp\|Q9HCU4\|CELR2_HUMAN Cadherin EGF LAG seven-pass G-type receptor 2 OS=Homo sapiens GN=CELSR2 I | 2669 | AGSLHStSRSG | 0.077 | -0.285 | -1.388 | -0.532 |
| sp\|Q9HCU4\|CELR2_HUMAN Cadherin EGF LAG seven-pass G-type receptor 2 OS=Homo sapiens GN=CELSR2 I | 2670 | GSLHSTsRSGK | 0.237 | -0.102 | -0.59 | -0.152 |
| sp\|Q9HCU4\|CELR2_HUMAN Cadherin EGF LAG seven-pass G-type receptor 2 OS=Homo sapiens GN=CELSR2 I | 2672 | LHSTSRsGKSQ | 0.081 | -0.078 | -1.366 | -0.454 |
| sp\|Q9HCU4\|CELR2_HUMAN Cadherin EGF LAG seven-pass G-type receptor 2 OS=Homo sapiens GN=CELSR2 I | 2675 | TSRSGKsQPSY | 0.532 | 0.491 | -0.163 | 0.287 |
| sp\|Q9HCU4\|CELR2_HUMAN Cadherin EGF LAG seven-pass G-type receptor 2 OS=Homo sapiens GN=CELSR2 I | 2678 | SGKSQPsYIPF | 0.039 | -0.347 | -1.437 | -0.582 |
| sp\|Q9HCU4\|CELR2_HUMAN Cadherin EGF LAG seven-pass G-type receptor 2 OS=Homo sapiens GN=CELSR2 I | 2688 | FLLREEsALNP | 0.571 | 0.918 | -0.222 | 0.422 |
| sp\|Q9HCU4\|CELR2_HUMAN Cadherin EGF LAG seven-pass G-type receptor 2 OS=Homo sapiens GN=CELSR2 I | 2704 | GLGDPGsLFLE | 0.295 | 0.014 | -0.572 | -0.088 |
| sp\|Q9HCU4\|CELR2_HUMAN Cadherin EGF LAG seven-pass G-type receptor 2 OS=Homo sapiens GN=CELSR2 I | 2718 | QQHDPDtDSDS | 0.077 | -0.265 | -1.474 | -0.554 |
| sp\|Q9HCU4\|CELR2_HUMAN Cadherin EGF LAG seven-pass G-type receptor 2 OS=Homo sapiens GN=CELSR2 I | 2720 | HDPDTDsDSDL | 0.074 | -0.099 | -1.408 | -0.478 |
| sp\|Q9HCU4\|CELR2_HUMAN Cadherin EGF LAG seven-pass G-type receptor 2 OS=Homo sapiens GN=CELSR2 I | 2722 | PDTDSDsDLSL | 0.153 | 0.049 | -0.995 | -0.264 |
| sp\|Q9HCU4\|CELR2_HUMAN Cadherin EGF LAG seven-pass G-type receptor 2 OS=Homo sapiens GN=CELSR2 I | 2725 | DSDSDLsLEDD | 0.261 | -0.112 | -0.735 | -0.195 |
| sp\|Q9HCU4\|CELR2_HUMAN Cadherin EGF LAG seven-pass G-type receptor 2 OS=Homo sapiens GN=CELSR2 I | 2731 | SLEDDQsGSYA | 0.107 | -0.208 | -1.223 | -0.441 |
| sp\|Q9HCU4\|CELR2_HUMAN Cadherin EGF LAG seven-pass G-type receptor 2 OS=Homo sapiens GN=CELSR2 I | 2733 | EDDQSGsYAST | 0.042 | -0.237 | -1.296 | -0.497 |
| sp\|Q9HCU4\|CELR2_HUMAN Cadherin EGF LAG seven-pass G-type receptor 2 OS=Homo sapiens GN=CELSR2 I | 2736 | QSGSYAsTHSS | 0.075 | -0.223 | -1.398 | -0.515 |
| sp\|Q9HCU4\|CELR2_HUMAN Cadherin EGF LAG seven-pass G-type receptor 2 OS=Homo sapiens GN=CELSR2 I | 2737 | SGSYAStHSSD | 0.094 | -0.181 | -1.116 | -0.401 |
| sp\|Q9HCU4\|CELR2_HUMAN Cadherin EGF LAG seven-pass G-type receptor 2 OS=Homo sapiens GN=CELSR2 I | 2739 | SYASTHsSDSE | 0.104 | -0.035 | -1.139 | -0.357 |
| sp\|Q9HCU4\|CELR2_HUMAN Cadherin EGF LAG seven-pass G-type receptor 2 OS=Homo sapiens GN=CELSR2 I | 2740 | YASTHSsDSEE | 0.123 | -0.194 | -1.326 | -0.466 |
| sp\|Q9HCU4\|CELR2_HUMAN Cadherin EGF LAG seven-pass G-type receptor 2 OS=Homo sapiens GN=CELSR2 I | 2742 | STHSSDsEEEE | 0.179 | -0.032 | -1.122 | -0.325 |
| sp\|Q9HCU4\|CELR2_HUMAN Cadherin EGF LAG seven-pass G-type receptor 2 OS=Homo sapiens GN=CELSR2 I | 2763 | GEQGWDsLLGP | 0.223 | -0.045 | -0.821 | -0.214 |
| sp\|Q9HCU4\|CELR2_HUMAN Cadherin EGF LAG seven-pass G-type receptor 2 OS=Homo sapiens GN=CELSR2 I | 2776 | ERLPLHsTPKD | 0.53 | 0.681 | 0.052 | 0.421 |
| sp\|Q9HCU4\|CELR2_HUMAN Cadherin EGF LAG seven-pass G-type receptor 2 OS=Homo sapiens GN=CELSR2 I | 2777 | RLPLHStPKDG | 0.149 | -0.221 | -1.091 | -0.388 |
| sp\|Q9HCU4\|CELR2_HUMAN Cadherin EGF LAG seven-pass G-type receptor 2 OS=Homo sapiens GN=CELSR2 I | 2796 | WPGDFGtTAKE | 0.047 | -0.369 | -1.838 | -0.72 |
| sp\|Q9HCU4\|CELR2_HUMAN Cadherin EGF LAG seven-pass G-type receptor 2 OS=Homo sapiens GN=CELSR2 I | 2797 | PGDFGTtAKES | 0.127 | -0.094 | -1.025 | -0.331 |
| sp\|Q9HCU4\|CELR2_HUMAN Cadherin EGF LAG seven-pass G-type receptor 2 OS=Homo sapiens GN=CELSR2 I | 2801 | GTTAKEsSGNG | 0.225 | -0.088 | -1.095 | -0.319 |
| sp\|Q9HCU4\|CELR2_HUMAN Cadherin EGF LAG seven-pass G-type receptor 2 OS=Homo sapiens GN=CELSR2 I | 2802 | TTAKESsGNGA | 0.189 | -0.236 | -0.76 | -0.269 |
| sp\|Q9HCU4\|CELR2_HUMAN Cadherin EGF LAG seven-pass G-type receptor 2 OS=Homo sapiens GN=CELSR2 I | 2819 | ENGDALsREGS | 0.05 | -0.289 | -1.461 | -0.567 |
| sp\|Q9HCU4\|CELR2_HUMAN Cadherin EGF LAG seven-pass G-type receptor 2 OS=Homo sapiens GN=CELSR2 I | 2823 | ALSREGsLGPL | 0.771 | 0.885 | 0.549 | 0.735 |
| sp\|Q9HCU4\|CELR2_HUMAN Cadherin EGF LAG seven-pass G-type receptor 2 OS=Homo sapiens GN=CELSR2 I | 2830 | LGPLPGsSAQP | 0.033 | -0.478 | -1.675 | -0.707 |
| sp\|Q9HCU4\|CELR2_HUMAN Cadherin EGF LAG seven-pass G-type receptor 2 OS=Homo sapiens GN=CELSR2 I | 2831 | GPLPGSsAQPH | 0.048 | -0.355 | -1.645 | -0.651 |
| sp\|Q9HCU4\|CELR2_HUMAN Cadherin EGF LAG seven-pass G-type receptor 2 OS=Homo sapiens GN=CELSR2 I | 2846 | KKKCLPtISEK | 0.208 | -0.207 | -0.93 | -0.31 |
| sp\|Q9HCU4\|CELR2_HUMAN Cadherin EGF LAG seven-pass G-type receptor 2 OS=Homo sapiens GN=CELSR2 I | 2848 | KCLPTIsEKSS | 0.09 | -0.07 | -1.136 | -0.372 |
| sp\|Q9HCU4\|CELR2_HUMAN Cadherin EGF LAG seven-pass G-type receptor 2 OS=Homo sapiens GN=CELSR2 I | 2851 | PTISEKsSLLR | 0.168 | -0.093 | -1.044 | -0.323 |
| sp\|Q9HCU4\|CELR2_HUMAN Cadherin EGF LAG seven-pass G-type receptor 2 OS=Homo sapiens GN=CELSR2 I | 2852 | TISEKSsLLRL | 0.078 | -0.134 | -1.249 | -0.435 |
| sp\|Q9HCU4\|CELR2_HUMAN Cadherin EGF LAG seven-pass G-type receptor 2 OS=Homo sapiens GN=CELSR2 I | 2862 | LPLEQCtGSSR | 0.042 | -0.265 | -1.693 | -0.639 |
| sp\|Q9HCU4\|CELR2_HUMAN Cadherin EGF LAG seven-pass G-type receptor 2 OS=Homo sapiens GN=CELSR2 I | 2864 | LEQCTGsSRGS | 0.075 | -0.242 | -1.404 | -0.524 |
| sp\|Q9HCU4\|CELR2_HUMAN Cadherin EGF LAG seven-pass G-type receptor 2 OS=Homo sapiens GN=CELSR2 I | 2865 | EQCTGSsRGSS | 0.031 | -0.333 | -1.644 | -0.649 |
| sp\|Q9HCU4\|CELR2_HUMAN Cadherin EGF LAG seven-pass G-type receptor 2 OS=Homo sapiens GN=CELSR2 I | 2868 | TGSSRGsSASE | 0.064 | -0.29 | -1.487 | -0.571 |
| sp\|Q9HCU4\|CELR2_HUMAN Cadherin EGF LAG seven-pass G-type receptor 2 OS=Homo sapiens GN=CELSR2 I | 2869 | GSSRGSsASEG | 0.367 | 0.656 | -0.347 | 0.225 |
| sp\|Q9HCU4\|CELR2_HUMAN Cadherin EGF LAG seven-pass G-type receptor 2 OS=Homo sapiens GN=CELSR2 I | 2871 | SRGSSAsEGSR | 0.301 | 0.391 | -0.402 | 0.097 |
| sp\|Q9HCU4\|CELR2_HUMAN Cadherin EGF LAG seven-pass G-type receptor 2 OS=Homo sapiens GN=CELSR2 I | 2874 | SSASEGsRGGP | 0.055 | -0.488 | -1.697 | -0.71 |
| sp\|Q9HCU4\|CELR2_HUMAN Cadherin EGF LAG seven-pass G-type receptor 2 OS=Homo sapiens GN=CELSR2 I | 2887 | RPPPRQsLQEQ | 0.41 | 0.101 | -0.39 | 0.04 |
| sp\|Q9HCU4\|CELR2_HUMAN Cadherin EGF LAG seven-pass G-type receptor 2 OS=Homo sapiens GN=CELSR2 I | 2901 | VMPIAMsIKAG | 0.185 | 0.207 | -0.539 | -0.049 |
| sp\|Q9HCU4\|CELR2_HUMAN Cadherin EGF LAG seven-pass G-type receptor 2 OS=Homo sapiens GN=CELSR2 I | 2906 | MSIKAGtVDED | 0.564 | 0.169 | -0.162 | 0.19 |

| | | | | | | |
|---|---|---|---|---|---|---|
| sp\|Q9HCU4\|CELR2_HUMAN Cadherin EGF LAG seven-pass G-type receptor 2 OS=Homo sapiens GN=CELSR2 I | 2911 | GTVDEDsSGSE | 0.132 | -0.211 | -1.551 | -0.543 |
| sp\|Q9HCU4\|CELR2_HUMAN Cadherin EGF LAG seven-pass G-type receptor 2 OS=Homo sapiens GN=CELSR2 I | 2912 | TVDEDSsGSEF | 0.026 | -0.352 | -1.783 | -0.703 |
| sp\|Q9HCU4\|CELR2_HUMAN Cadherin EGF LAG seven-pass G-type receptor 2 OS=Homo sapiens GN=CELSR2 I | 2914 | DEDSSGsEFLF | 0.216 | 0.055 | -0.854 | -0.194 |
| sp\|Q9NYQ7\|CELR3_HUMAN Cadherin EGF LAG seven-pass G-type receptor 3 OS=Homo sapiens GN=CELSR3 I | 332 | PQYNYQtLVPE | 0.187 | -0.011 | -0.371 | -0.065 |
| sp\|Q9NYQ7\|CELR3_HUMAN Cadherin EGF LAG seven-pass G-type receptor 3 OS=Homo sapiens GN=CELSR3 I | 342 | ENEAAGtAVLR | 0.136 | -0.127 | -0.978 | -0.323 |
| sp\|Q9NYQ7\|CELR3_HUMAN Cadherin EGF LAG seven-pass G-type receptor 3 OS=Homo sapiens GN=CELSR3 I | 363 | AGRLVYsLAAL | 0.585 | 0.386 | 0.192 | 0.388 |
| sp\|Q9NYQ7\|CELR3_HUMAN Cadherin EGF LAG seven-pass G-type receptor 3 OS=Homo sapiens GN=CELSR3 I | 370 | LAALMNsRSLE | 0.114 | -0.273 | -1.233 | -0.464 |
| sp\|Q9NYQ7\|CELR3_HUMAN Cadherin EGF LAG seven-pass G-type receptor 3 OS=Homo sapiens GN=CELSR3 I | 372 | ALMNSRsLELF | 0.381 | 0.344 | -0.05 | 0.225 |
| sp\|Q9NYQ7\|CELR3_HUMAN Cadherin EGF LAG seven-pass G-type receptor 3 OS=Homo sapiens GN=CELSR3 I | 377 | RSLELFsIDPQ | 0.077 | -0.196 | -1.173 | -0.431 |
| sp\|Q9NYQ7\|CELR3_HUMAN Cadherin EGF LAG seven-pass G-type receptor 3 OS=Homo sapiens GN=CELSR3 I | 382 | FSIDPQsGLIR | 0.119 | -0.132 | -1.044 | -0.352 |
| sp\|Q9NYQ7\|CELR3_HUMAN Cadherin EGF LAG seven-pass G-type receptor 3 OS=Homo sapiens GN=CELSR3 I | 387 | QSGLIRtAAAL | 0.088 | -0.061 | -1.128 | -0.367 |
| sp\|Q9NYQ7\|CELR3_HUMAN Cadherin EGF LAG seven-pass G-type receptor 3 OS=Homo sapiens GN=CELSR3 I | 395 | AALDREsMERH | 0.16 | -0.111 | -1.523 | -0.491 |
| sp\|Q9NYQ7\|CELR3_HUMAN Cadherin EGF LAG seven-pass G-type receptor 3 OS=Homo sapiens GN=CELSR3 I | 404 | RHYLRVtAQDH | 0.468 | 0.147 | -0.762 | -0.049 |
| sp\|Q9NYQ7\|CELR3_HUMAN Cadherin EGF LAG seven-pass G-type receptor 3 OS=Homo sapiens GN=CELSR3 I | 410 | TAQDHGsPRLS | 0.062 | -0.441 | -1.68 | -0.686 |
| sp\|Q9NYQ7\|CELR3_HUMAN Cadherin EGF LAG seven-pass G-type receptor 3 OS=Homo sapiens GN=CELSR3 I | 414 | HGSPRLsATTM | 0.208 | 0.011 | -0.665 | -0.149 |
| sp\|Q9NYQ7\|CELR3_HUMAN Cadherin EGF LAG seven-pass G-type receptor 3 OS=Homo sapiens GN=CELSR3 I | 416 | SPRLSAtTMVA | 0.498 | 0.359 | -0.334 | 0.174 |
| sp\|Q9NYQ7\|CELR3_HUMAN Cadherin EGF LAG seven-pass G-type receptor 3 OS=Homo sapiens GN=CELSR3 I | 417 | PRLSATtMVAV | 0.395 | 0.407 | -0.419 | 0.128 |
| sp\|Q9NYQ7\|CELR3_HUMAN Cadherin EGF LAG seven-pass G-type receptor 3 OS=Homo sapiens GN=CELSR3 I | 422 | TTMVAVtVADR | 0.357 | 0.039 | -0.487 | -0.03 |
| sp\|Q9NYQ7\|CELR3_HUMAN Cadherin EGF LAG seven-pass G-type receptor 3 OS=Homo sapiens GN=CELSR3 I | 430 | ADRNDHsPVFE | 0.081 | -0.239 | -1.278 | -0.479 |
| sp\|Q9NYQ7\|CELR3_HUMAN Cadherin EGF LAG seven-pass G-type receptor 3 OS=Homo sapiens GN=CELSR3 I | 441 | QAQYREtLREN | 0.539 | 0.173 | -0.193 | 0.173 |
| sp\|Q9NYQ7\|CELR3_HUMAN Cadherin EGF LAG seven-pass G-type receptor 3 OS=Homo sapiens GN=CELSR3 I | 458 | ILQLRAtDGDA | 0.843 | 0.428 | 0.363 | 0.545 |
| sp\|Q9NYQ7\|CELR3_HUMAN Cadherin EGF LAG seven-pass G-type receptor 3 OS=Homo sapiens GN=CELSR3 I | 492 | FEIDPRsGLIS | 0.065 | -0.161 | -1.615 | -0.57 |
| sp\|Q9NYQ7\|CELR3_HUMAN Cadherin EGF LAG seven-pass G-type receptor 3 OS=Homo sapiens GN=CELSR3 I | 496 | PRSGLIsTSGR | 0.136 | 0.009 | -0.856 | -0.237 |
| sp\|Q9NYQ7\|CELR3_HUMAN Cadherin EGF LAG seven-pass G-type receptor 3 OS=Homo sapiens GN=CELSR3 I | 497 | RSGLIStSGRV | 0.107 | -0.215 | -1.412 | -0.507 |
| sp\|Q9NYQ7\|CELR3_HUMAN Cadherin EGF LAG seven-pass G-type receptor 3 OS=Homo sapiens GN=CELSR3 I | 498 | SGLISTsGRVD | 0.25 | 0.061 | -0.397 | -0.029 |
| sp\|Q9NYQ7\|CELR3_HUMAN Cadherin EGF LAG seven-pass G-type receptor 3 OS=Homo sapiens GN=CELSR3 I | 508 | DREHMEsYELV | 0.185 | -0.01 | -1.18 | -0.335 |
| sp\|Q9NYQ7\|CELR3_HUMAN Cadherin EGF LAG seven-pass G-type receptor 3 OS=Homo sapiens GN=CELSR3 I | 516 | ELVVEAsDQGQ | 0.218 | -0.031 | -0.954 | -0.256 |
| sp\|Q9NYQ7\|CELR3_HUMAN Cadherin EGF LAG seven-pass G-type receptor 3 OS=Homo sapiens GN=CELSR3 I | 526 | QEPGPRsATVR | 0.076 | -0.182 | -1.455 | -0.52 |
| sp\|Q9NYQ7\|CELR3_HUMAN Cadherin EGF LAG seven-pass G-type receptor 3 OS=Homo sapiens GN=CELSR3 I | 528 | PGPRSAtVRVH | 0.722 | 0.911 | 0.477 | 0.703 |
| sp\|Q9NYQ7\|CELR3_HUMAN Cadherin EGF LAG seven-pass G-type receptor 3 OS=Homo sapiens GN=CELSR3 I | 534 | TVRVHItVLDE | 0.329 | 0.22 | -0.514 | 0.012 |
| sp\|Q9NYQ7\|CELR3_HUMAN Cadherin EGF LAG seven-pass G-type receptor 3 OS=Homo sapiens GN=CELSR3 I | 546 | DNAPQFsEKRY | 0.17 | -0.142 | -1.011 | -0.328 |
| sp\|Q9NYQ7\|CELR3_HUMAN Cadherin EGF LAG seven-pass G-type receptor 3 OS=Homo sapiens GN=CELSR3 I | 562 | EDVRPHtVVLR | 0.422 | 0.822 | 0.163 | 0.469 |
| sp\|Q9NYQ7\|CELR3_HUMAN Cadherin EGF LAG seven-pass G-type receptor 3 OS=Homo sapiens GN=CELSR3 I | 568 | TVVLRVtATDR | 0.551 | 0.193 | -0.341 | 0.134 |
| sp\|Q9NYQ7\|CELR3_HUMAN Cadherin EGF LAG seven-pass G-type receptor 3 OS=Homo sapiens GN=CELSR3 I | 570 | VLRVTAtDRDK | 0.574 | 0.304 | -0.181 | 0.232 |
| sp\|Q9NYQ7\|CELR3_HUMAN Cadherin EGF LAG seven-pass G-type receptor 3 OS=Homo sapiens GN=CELSR3 I | 586 | VHYNIIsGNSR | 0.07 | -0.163 | -0.967 | -0.353 |
| sp\|Q9NYQ7\|CELR3_HUMAN Cadherin EGF LAG seven-pass G-type receptor 3 OS=Homo sapiens GN=CELSR3 I | 589 | NIISGNsRGHF | 0.122 | -0.128 | -1.085 | -0.364 |
| sp\|Q9NYQ7\|CELR3_HUMAN Cadherin EGF LAG seven-pass G-type receptor 3 OS=Homo sapiens GN=CELSR3 I | 597 | GHFAIDsLTGE | 0.359 | -0.102 | -0.738 | -0.16 |
| sp\|Q9NYQ7\|CELR3_HUMAN Cadherin EGF LAG seven-pass G-type receptor 3 OS=Homo sapiens GN=CELSR3 I | 599 | FAIDSLtGEIQ | 0.189 | 0.089 | -0.883 | -0.202 |
| sp\|Q9NYQ7\|CELR3_HUMAN Cadherin EGF LAG seven-pass G-type receptor 3 OS=Homo sapiens GN=CELSR3 I | 631 | AGRPPLsNNTG | 0.187 | -0.061 | -0.732 | -0.202 |
| sp\|Q9NYQ7\|CELR3_HUMAN Cadherin EGF LAG seven-pass G-type receptor 3 OS=Homo sapiens GN=CELSR3 I | 634 | PPLSNNtGLAS | 0.159 | -0.103 | -0.902 | -0.282 |
| sp\|Q9NYQ7\|CELR3_HUMAN Cadherin EGF LAG seven-pass G-type receptor 3 OS=Homo sapiens GN=CELSR3 I | 638 | NNTGLAsIQVV | 0.116 | -0.127 | -1.078 | -0.363 |
| sp\|Q9NYQ7\|CELR3_HUMAN Cadherin EGF LAG seven-pass G-type receptor 3 OS=Homo sapiens GN=CELSR3 I | 653 | HIPIFVsTPFQ | 0.407 | 0.402 | -0.369 | 0.147 |
| sp\|Q9NYQ7\|CELR3_HUMAN Cadherin EGF LAG seven-pass G-type receptor 3 OS=Homo sapiens GN=CELSR3 I | 654 | IPIFVStPFQV | 0.088 | -0.411 | -1.484 | -0.602 |
| sp\|Q9NYQ7\|CELR3_HUMAN Cadherin EGF LAG seven-pass G-type receptor 3 OS=Homo sapiens GN=CELSR3 I | 659 | STPFQVsVLEN | 0.133 | -0.13 | -1.382 | -0.46 |
| sp\|Q9NYQ7\|CELR3_HUMAN Cadherin EGF LAG seven-pass G-type receptor 3 OS=Homo sapiens GN=CELSR3 I | 669 | NAPLGHsVIHI | 0.112 | -0.067 | -0.868 | -0.274 |
| sp\|Q9NYQ7\|CELR3_HUMAN Cadherin EGF LAG seven-pass G-type receptor 3 OS=Homo sapiens GN=CELSR3 I | 689 | NARLEYsLTGV | 0.592 | 0.248 | -0.268 | 0.191 |
| sp\|Q9NYQ7\|CELR3_HUMAN Cadherin EGF LAG seven-pass G-type receptor 3 OS=Homo sapiens GN=CELSR3 I | 691 | RLEYSLtGVAP | 0.34 | 0.253 | -0.092 | 0.167 |
| sp\|Q9NYQ7\|CELR3_HUMAN Cadherin EGF LAG seven-pass G-type receptor 3 OS=Homo sapiens GN=CELSR3 I | 697 | TGVAPDtPFVI | 0.075 | -0.482 | -1.232 | -0.546 |
| sp\|Q9NYQ7\|CELR3_HUMAN Cadherin EGF LAG seven-pass G-type receptor 3 OS=Homo sapiens GN=CELSR3 I | 703 | TPFVINsATGW | 0.168 | -0.072 | -1.042 | -0.315 |

| | | | | | | |
|---|---|---|---|---|---|---|
| sp\|Q9NYQ7\|CELR3_HUMAN Cadherin EGF LAG seven-pass G-type receptor 3 OS=Homo sapiens GN=CELSR3 I | 705 | FVINSAtGWVS | 0.347 | 0.322 | -0.313 | 0.119 |
| sp\|Q9NYQ7\|CELR3_HUMAN Cadherin EGF LAG seven-pass G-type receptor 3 OS=Homo sapiens GN=CELSR3 I | 709 | SATGWVsVSGP | 0.108 | -0.244 | -1.052 | -0.396 |
| sp\|Q9NYQ7\|CELR3_HUMAN Cadherin EGF LAG seven-pass G-type receptor 3 OS=Homo sapiens GN=CELSR3 I | 711 | TGWVSVsGPLD | 0.687 | 0.72 | 0.551 | 0.653 |
| sp\|Q9NYQ7\|CELR3_HUMAN Cadherin EGF LAG seven-pass G-type receptor 3 OS=Homo sapiens GN=CELSR3 I | 718 | GPLDREsVEHY | 0.298 | 0.06 | -0.809 | -0.15 |
| sp\|Q9NYQ7\|CELR3_HUMAN Cadherin EGF LAG seven-pass G-type receptor 3 OS=Homo sapiens GN=CELSR3 I | 733 | EARDHGsPPLS | 0.383 | 0.328 | -0.537 | 0.058 |
| sp\|Q9NYQ7\|CELR3_HUMAN Cadherin EGF LAG seven-pass G-type receptor 3 OS=Homo sapiens GN=CELSR3 I | 737 | HGSPPLsASAS | 0.055 | -0.19 | -1.147 | -0.427 |
| sp\|Q9NYQ7\|CELR3_HUMAN Cadherin EGF LAG seven-pass G-type receptor 3 OS=Homo sapiens GN=CELSR3 I | 739 | SPPLSAsASVT | 0.117 | 0.011 | -1.25 | -0.374 |
| sp\|Q9NYQ7\|CELR3_HUMAN Cadherin EGF LAG seven-pass G-type receptor 3 OS=Homo sapiens GN=CELSR3 I | 741 | PLSASAsVTVT | 0.599 | 0.259 | 0.011 | 0.29 |
| sp\|Q9NYQ7\|CELR3_HUMAN Cadherin EGF LAG seven-pass G-type receptor 3 OS=Homo sapiens GN=CELSR3 I | 743 | SASASVtVTVL | 0.398 | 0.094 | -0.427 | 0.022 |
| sp\|Q9NYQ7\|CELR3_HUMAN Cadherin EGF LAG seven-pass G-type receptor 3 OS=Homo sapiens GN=CELSR3 I | 745 | SASVTVtVLDV | 0.117 | -0.115 | -1.372 | -0.457 |
| sp\|Q9NYQ7\|CELR3_HUMAN Cadherin EGF LAG seven-pass G-type receptor 3 OS=Homo sapiens GN=CELSR3 I | 757 | DNRPEFtMKEY | 0.402 | 0.205 | -0.61 | -0.001 |
| sp\|Q9NYQ7\|CELR3_HUMAN Cadherin EGF LAG seven-pass G-type receptor 3 OS=Homo sapiens GN=CELSR3 I | 773 | EDAAVGtSVVS | 0.04 | -0.306 | -1.581 | -0.616 |
| sp\|Q9NYQ7\|CELR3_HUMAN Cadherin EGF LAG seven-pass G-type receptor 3 OS=Homo sapiens GN=CELSR3 I | 774 | DAAVGTsVVSV | 0.053 | -0.224 | -1.588 | -0.586 |
| sp\|Q9NYQ7\|CELR3_HUMAN Cadherin EGF LAG seven-pass G-type receptor 3 OS=Homo sapiens GN=CELSR3 I | 777 | VGTSVVsVTAV | 0.357 | -0.065 | -0.61 | -0.106 |
| sp\|Q9NYQ7\|CELR3_HUMAN Cadherin EGF LAG seven-pass G-type receptor 3 OS=Homo sapiens GN=CELSR3 I | 779 | TSVVSVtAVDR | 0.414 | 0.181 | -0.34 | 0.085 |
| sp\|Q9NYQ7\|CELR3_HUMAN Cadherin EGF LAG seven-pass G-type receptor 3 OS=Homo sapiens GN=CELSR3 I | 787 | VDRDANsAISY | 0.351 | 0.247 | -0.659 | -0.02 |
| sp\|Q9NYQ7\|CELR3_HUMAN Cadherin EGF LAG seven-pass G-type receptor 3 OS=Homo sapiens GN=CELSR3 I | 790 | DANSAIsYQIT | 0.219 | -0.189 | -1.026 | -0.332 |
| sp\|Q9NYQ7\|CELR3_HUMAN Cadherin EGF LAG seven-pass G-type receptor 3 OS=Homo sapiens GN=CELSR3 I | 794 | AISYQItGGNT | 0.181 | -0.021 | -0.863 | -0.234 |
| sp\|Q9NYQ7\|CELR3_HUMAN Cadherin EGF LAG seven-pass G-type receptor 3 OS=Homo sapiens GN=CELSR3 I | 798 | QITGGNtRNRF | 0.125 | -0.145 | -1.007 | -0.342 |
| sp\|Q9NYQ7\|CELR3_HUMAN Cadherin EGF LAG seven-pass G-type receptor 3 OS=Homo sapiens GN=CELSR3 I | 805 | RNRFAIsTQGG | 0.611 | 0.226 | -0.012 | 0.275 |
| sp\|Q9NYQ7\|CELR3_HUMAN Cadherin EGF LAG seven-pass G-type receptor 3 OS=Homo sapiens GN=CELSR3 I | 806 | NRFAIStQGGV | 0.21 | 0.059 | -0.888 | -0.206 |
| sp\|Q9NYQ7\|CELR3_HUMAN Cadherin EGF LAG seven-pass G-type receptor 3 OS=Homo sapiens GN=CELSR3 I | 814 | GGVGLVtLALP | 0.23 | -0.064 | -0.662 | -0.165 |
| sp\|Q9NYQ7\|CELR3_HUMAN Cadherin EGF LAG seven-pass G-type receptor 3 OS=Homo sapiens GN=CELSR3 I | 832 | YFKLVLtASDR | 0.227 | 0.031 | -0.673 | -0.138 |
| sp\|Q9NYQ7\|CELR3_HUMAN Cadherin EGF LAG seven-pass G-type receptor 3 OS=Homo sapiens GN=CELSR3 I | 834 | KLVLTAsDRAL | 0.359 | 0.181 | -0.506 | 0.011 |
| sp\|Q9NYQ7\|CELR3_HUMAN Cadherin EGF LAG seven-pass G-type receptor 3 OS=Homo sapiens GN=CELSR3 I | 849 | YVHINItDANT | 0.103 | -0.086 | -1.156 | -0.38 |
| sp\|Q9NYQ7\|CELR3_HUMAN Cadherin EGF LAG seven-pass G-type receptor 3 OS=Homo sapiens GN=CELSR3 I | 853 | NITDANtHRPV | 0.471 | 0.086 | -0.593 | -0.012 |
| sp\|Q9NYQ7\|CELR3_HUMAN Cadherin EGF LAG seven-pass G-type receptor 3 OS=Homo sapiens GN=CELSR3 I | 860 | HRPVFQsAHYS | 0.112 | 0.151 | -1.207 | -0.315 |
| sp\|Q9NYQ7\|CELR3_HUMAN Cadherin EGF LAG seven-pass G-type receptor 3 OS=Homo sapiens GN=CELSR3 I | 864 | FQSAHYsVSVN | 0.184 | 0.097 | -0.64 | -0.12 |
| sp\|Q9NYQ7\|CELR3_HUMAN Cadherin EGF LAG seven-pass G-type receptor 3 OS=Homo sapiens GN=CELSR3 I | 866 | SAHYSVsVNED | 0.451 | 0.174 | -0.112 | 0.171 |
| sp\|Q9NYQ7\|CELR3_HUMAN Cadherin EGF LAG seven-pass G-type receptor 3 OS=Homo sapiens GN=CELSR3 I | 875 | EDRPMGsTIVV | 0.101 | -0.079 | -1.161 | -0.38 |
| sp\|Q9NYQ7\|CELR3_HUMAN Cadherin EGF LAG seven-pass G-type receptor 3 OS=Homo sapiens GN=CELSR3 I | 876 | DRPMGStIVVI | 0.095 | -0.024 | -1.119 | -0.349 |
| sp\|Q9NYQ7\|CELR3_HUMAN Cadherin EGF LAG seven-pass G-type receptor 3 OS=Homo sapiens GN=CELSR3 I | 881 | STIVVIsASDD | 0.169 | -0.068 | -1.224 | -0.374 |
| sp\|Q9NYQ7\|CELR3_HUMAN Cadherin EGF LAG seven-pass G-type receptor 3 OS=Homo sapiens GN=CELSR3 I | 883 | IVVISAsDDDV | 0.634 | 0.372 | -0.113 | 0.298 |
| sp\|Q9NYQ7\|CELR3_HUMAN Cadherin EGF LAG seven-pass G-type receptor 3 OS=Homo sapiens GN=CELSR3 I | 894 | GENARItYLLE | 0.223 | -0.018 | -1.021 | -0.272 |
| sp\|Q9NYQ7\|CELR3_HUMAN Cadherin EGF LAG seven-pass G-type receptor 3 OS=Homo sapiens GN=CELSR3 I | 910 | FRIDADsGAIT | 0.234 | 0.132 | -0.894 | -0.176 |
| sp\|Q9NYQ7\|CELR3_HUMAN Cadherin EGF LAG seven-pass G-type receptor 3 OS=Homo sapiens GN=CELSR3 I | 914 | ADSGAItLQAP | 0.29 | -0.01 | -0.352 | -0.024 |
| sp\|Q9NYQ7\|CELR3_HUMAN Cadherin EGF LAG seven-pass G-type receptor 3 OS=Homo sapiens GN=CELSR3 I | 926 | DYEDQVtYTLA | 0.339 | -0.083 | -0.865 | -0.203 |
| sp\|Q9NYQ7\|CELR3_HUMAN Cadherin EGF LAG seven-pass G-type receptor 3 OS=Homo sapiens GN=CELSR3 I | 928 | EDQVTYtLAIT | 0.086 | -0.018 | -0.818 | -0.25 |
| sp\|Q9NYQ7\|CELR3_HUMAN Cadherin EGF LAG seven-pass G-type receptor 3 OS=Homo sapiens GN=CELSR3 I | 932 | TYTLAItARDN | 0.515 | 0.123 | -0.358 | 0.093 |
| sp\|Q9NYQ7\|CELR3_HUMAN Cadherin EGF LAG seven-pass G-type receptor 3 OS=Homo sapiens GN=CELSR3 I | 944 | IPQKADtTYVE | 0.375 | 0.006 | -0.659 | -0.093 |
| sp\|Q9NYQ7\|CELR3_HUMAN Cadherin EGF LAG seven-pass G-type receptor 3 OS=Homo sapiens GN=CELSR3 I | 945 | PQKADTtYVEV | 0.199 | -0.091 | -0.833 | -0.242 |
| sp\|Q9NYQ7\|CELR3_HUMAN Cadherin EGF LAG seven-pass G-type receptor 3 OS=Homo sapiens GN=CELSR3 I | 964 | APQFVAsHYTG | 0.334 | -0.004 | -0.801 | -0.157 |
| sp\|Q9NYQ7\|CELR3_HUMAN Cadherin EGF LAG seven-pass G-type receptor 3 OS=Homo sapiens GN=CELSR3 I | 967 | FVASHYtGLVS | 0.109 | 0.041 | -0.883 | -0.244 |
| sp\|Q9NYQ7\|CELR3_HUMAN Cadherin EGF LAG seven-pass G-type receptor 3 OS=Homo sapiens GN=CELSR3 I | 971 | HYTGLVsEDAP | 0.332 | 0.163 | -0.193 | 0.101 |
| sp\|Q9NYQ7\|CELR3_HUMAN Cadherin EGF LAG seven-pass G-type receptor 3 OS=Homo sapiens GN=CELSR3 I | 978 | EDAPPFtSVLQ | 0.04 | -0.314 | -1.285 | -0.52 |
| sp\|Q9NYQ7\|CELR3_HUMAN Cadherin EGF LAG seven-pass G-type receptor 3 OS=Homo sapiens GN=CELSR3 I | 979 | DAPPFTsVLQI | 0.143 | -0.211 | -1.101 | -0.39 |
| sp\|Q9NYQ7\|CELR3_HUMAN Cadherin EGF LAG seven-pass G-type receptor 3 OS=Homo sapiens GN=CELSR3 I | 984 | TSVLQIsATDR | 0.231 | -0.054 | -0.992 | -0.272 |
| sp\|Q9NYQ7\|CELR3_HUMAN Cadherin EGF LAG seven-pass G-type receptor 3 OS=Homo sapiens GN=CELSR3 I | 986 | VLQISAtDRDA | 0.735 | 0.368 | 0.215 | 0.439 |
| sp\|Q9NYQ7\|CELR3_HUMAN Cadherin EGF LAG seven-pass G-type receptor 3 OS=Homo sapiens GN=CELSR3 I | 999 | NGRVQYtFQNG | 0.381 | 0.373 | -0.248 | 0.169 |

| | | | | | | | |
|---|---|---|---|---|---|---|---|
| sp\|Q9NYQ7\|CELR3_HUMAN Cadherin EGF LAG seven-pass G-type receptor 3 OS=Homo sapiens GN=CELSR3 I | 1011 | DGDGDFtIEPT | 0.081 | -0.264 | -1.154 | -0.446 |
| sp\|Q9NYQ7\|CELR3_HUMAN Cadherin EGF LAG seven-pass G-type receptor 3 OS=Homo sapiens GN=CELSR3 I | 1015 | DFTIEPtSGIV | 0.289 | -0.096 | -1.021 | -0.276 |
| sp\|Q9NYQ7\|CELR3_HUMAN Cadherin EGF LAG seven-pass G-type receptor 3 OS=Homo sapiens GN=CELSR3 I | 1016 | FTIEPTsGIVR | 0.092 | -0.078 | -0.971 | -0.319 |
| sp\|Q9NYQ7\|CELR3_HUMAN Cadherin EGF LAG seven-pass G-type receptor 3 OS=Homo sapiens GN=CELSR3 I | 1021 | TSGIVRtVRRL | 0.083 | -0.238 | -1.429 | -0.528 |
| sp\|Q9NYQ7\|CELR3_HUMAN Cadherin EGF LAG seven-pass G-type receptor 3 OS=Homo sapiens GN=CELSR3 I | 1031 | LDREAVsVYEL | 0.429 | 0.261 | -0.217 | 0.158 |
| sp\|Q9NYQ7\|CELR3_HUMAN Cadherin EGF LAG seven-pass G-type receptor 3 OS=Homo sapiens GN=CELSR3 I | 1036 | VSVYELtAYAV | 0.123 | -0.069 | -1.237 | -0.394 |
| sp\|Q9NYQ7\|CELR3_HUMAN Cadherin EGF LAG seven-pass G-type receptor 3 OS=Homo sapiens GN=CELSR3 I | 1049 | GVPPLRtPVSI | 0.025 | -0.451 | -1.894 | -0.773 |
| sp\|Q9NYQ7\|CELR3_HUMAN Cadherin EGF LAG seven-pass G-type receptor 3 OS=Homo sapiens GN=CELSR3 I | 1052 | PLRTPVsIQVM | 0.597 | 0.225 | 0.076 | 0.299 |
| sp\|Q9NYQ7\|CELR3_HUMAN Cadherin EGF LAG seven-pass G-type receptor 3 OS=Homo sapiens GN=CELSR3 I | 1080 | VRVKENsIVGS | 0.256 | 0.102 | -0.781 | -0.141 |
| sp\|Q9NYQ7\|CELR3_HUMAN Cadherin EGF LAG seven-pass G-type receptor 3 OS=Homo sapiens GN=CELSR3 I | 1084 | ENSIVGsVVAQ | 0.069 | -0.204 | -1.245 | -0.46 |
| sp\|Q9NYQ7\|CELR3_HUMAN Cadherin EGF LAG seven-pass G-type receptor 3 OS=Homo sapiens GN=CELSR3 I | 1090 | SVVAQItAVDP | 0.086 | -0.109 | -1.391 | -0.471 |
| sp\|Q9NYQ7\|CELR3_HUMAN Cadherin EGF LAG seven-pass G-type receptor 3 OS=Homo sapiens GN=CELSR3 I | 1121 | FQMDIFsGELT | 0.108 | 0.023 | -0.694 | -0.188 |
| sp\|Q9NYQ7\|CELR3_HUMAN Cadherin EGF LAG seven-pass G-type receptor 3 OS=Homo sapiens GN=CELSR3 I | 1125 | IFSGELtALID | 0.16 | -0.104 | -0.955 | -0.3 |
| sp\|Q9NYQ7\|CELR3_HUMAN Cadherin EGF LAG seven-pass G-type receptor 3 OS=Homo sapiens GN=CELSR3 I | 1145 | VIVVQAtSAPL | 0.08 | -0.117 | -1.39 | -0.476 |
| sp\|Q9NYQ7\|CELR3_HUMAN Cadherin EGF LAG seven-pass G-type receptor 3 OS=Homo sapiens GN=CELSR3 I | 1146 | IVVQATsAPLV | 0.714 | 0.602 | 0.18 | 0.499 |
| sp\|Q9NYQ7\|CELR3_HUMAN Cadherin EGF LAG seven-pass G-type receptor 3 OS=Homo sapiens GN=CELSR3 I | 1151 | TSAPLVsRATV | 0.094 | -0.392 | -1.435 | -0.578 |
| sp\|Q9NYQ7\|CELR3_HUMAN Cadherin EGF LAG seven-pass G-type receptor 3 OS=Homo sapiens GN=CELSR3 I | 1154 | PLVSRAtVHVR | 0.595 | 0.29 | -0.147 | 0.246 |
| sp\|Q9NYQ7\|CELR3_HUMAN Cadherin EGF LAG seven-pass G-type receptor 3 OS=Homo sapiens GN=CELSR3 I | 1166 | VDQNDNsPVLN | 0.06 | -0.363 | -1.461 | -0.588 |
| sp\|Q9NYQ7\|CELR3_HUMAN Cadherin EGF LAG seven-pass G-type receptor 3 OS=Homo sapiens GN=CELSR3 I | 1181 | LFNNYVsNRSD | 0.204 | -0.14 | -0.698 | -0.211 |
| sp\|Q9NYQ7\|CELR3_HUMAN Cadherin EGF LAG seven-pass G-type receptor 3 OS=Homo sapiens GN=CELSR3 I | 1184 | NYVSNRsDTFP | 0.126 | -0.183 | -1.364 | -0.474 |
| sp\|Q9NYQ7\|CELR3_HUMAN Cadherin EGF LAG seven-pass G-type receptor 3 OS=Homo sapiens GN=CELSR3 I | 1186 | VSNRSDtFPSG | 0.899 | 1.4 | 1.14 | 1.146 |
| sp\|Q9NYQ7\|CELR3_HUMAN Cadherin EGF LAG seven-pass G-type receptor 3 OS=Homo sapiens GN=CELSR3 I | 1189 | RSDTFPsGIIG | 0.131 | -0.25 | -1.174 | -0.431 |
| sp\|Q9NYQ7\|CELR3_HUMAN Cadherin EGF LAG seven-pass G-type receptor 3 OS=Homo sapiens GN=CELSR3 I | 1203 | AYDPDVsDHLF | 0.272 | -0.095 | -0.883 | -0.235 |
| sp\|Q9NYQ7\|CELR3_HUMAN Cadherin EGF LAG seven-pass G-type receptor 3 OS=Homo sapiens GN=CELSR3 I | 1209 | SDHLFYsFERG | 0.079 | -0.178 | -1.413 | -0.504 |
| sp\|Q9NYQ7\|CELR3_HUMAN Cadherin EGF LAG seven-pass G-type receptor 3 OS=Homo sapiens GN=CELSR3 I | 1224 | LLVVNQtSGEL | 0.279 | 0.109 | -0.417 | -0.01 |
| sp\|Q9NYQ7\|CELR3_HUMAN Cadherin EGF LAG seven-pass G-type receptor 3 OS=Homo sapiens GN=CELSR3 I | 1225 | LVVNQTsGELR | 0.062 | -0.102 | -1.037 | -0.359 |
| sp\|Q9NYQ7\|CELR3_HUMAN Cadherin EGF LAG seven-pass G-type receptor 3 OS=Homo sapiens GN=CELSR3 I | 1231 | SGELRLsRKLD | 0.147 | -0.09 | -0.964 | -0.302 |
| sp\|Q9NYQ7\|CELR3_HUMAN Cadherin EGF LAG seven-pass G-type receptor 3 OS=Homo sapiens GN=CELSR3 I | 1243 | NRPLVAsMLVT | 0.143 | 0.214 | -1.13 | -0.258 |
| sp\|Q9NYQ7\|CELR3_HUMAN Cadherin EGF LAG seven-pass G-type receptor 3 OS=Homo sapiens GN=CELSR3 I | 1247 | VASMLVtVTDG | 0.341 | -0.166 | -0.715 | -0.18 |
| sp\|Q9NYQ7\|CELR3_HUMAN Cadherin EGF LAG seven-pass G-type receptor 3 OS=Homo sapiens GN=CELSR3 I | 1249 | SMLVTVtDGLH | 0.105 | 0 | -1.255 | -0.383 |
| sp\|Q9NYQ7\|CELR3_HUMAN Cadherin EGF LAG seven-pass G-type receptor 3 OS=Homo sapiens GN=CELSR3 I | 1254 | VTDGLHsVTAQ | 0.205 | -0.025 | -0.92 | -0.247 |
| sp\|Q9NYQ7\|CELR3_HUMAN Cadherin EGF LAG seven-pass G-type receptor 3 OS=Homo sapiens GN=CELSR3 I | 1256 | DGLHSVtAQCV | 0.6 | 0.241 | -0.29 | 0.184 |
| sp\|Q9NYQ7\|CELR3_HUMAN Cadherin EGF LAG seven-pass G-type receptor 3 OS=Homo sapiens GN=CELSR3 I | 1267 | LRVVIItEELL | 0.18 | 0.274 | -0.499 | -0.015 |
| sp\|Q9NYQ7\|CELR3_HUMAN Cadherin EGF LAG seven-pass G-type receptor 3 OS=Homo sapiens GN=CELSR3 I | 1274 | EELLANsLTVR | 0.524 | 0.171 | -0.294 | 0.134 |
| sp\|Q9NYQ7\|CELR3_HUMAN Cadherin EGF LAG seven-pass G-type receptor 3 OS=Homo sapiens GN=CELSR3 I | 1276 | LLANSLtVRLE | 0.304 | 0.157 | -0.081 | 0.127 |
| sp\|Q9NYQ7\|CELR3_HUMAN Cadherin EGF LAG seven-pass G-type receptor 3 OS=Homo sapiens GN=CELSR3 I | 1289 | WQERFLsPLLG | 0.182 | 0.412 | -0.443 | 0.05 |
| sp\|Q9NYQ7\|CELR3_HUMAN Cadherin EGF LAG seven-pass G-type receptor 3 OS=Homo sapiens GN=CELSR3 I | 1305 | VAAVLAtPAED | 0.068 | -0.465 | -1.497 | -0.631 |
| sp\|Q9NYQ7\|CELR3_HUMAN Cadherin EGF LAG seven-pass G-type receptor 3 OS=Homo sapiens GN=CELSR3 I | 1319 | FNIQNDtDVGG | 0.241 | -0.098 | -0.764 | -0.207 |
| sp\|Q9NYQ7\|CELR3_HUMAN Cadherin EGF LAG seven-pass G-type receptor 3 OS=Homo sapiens GN=CELSR3 I | 1324 | DTDVGGtVLNV | 0.05 | -0.254 | -2.074 | -0.759 |
| sp\|Q9NYQ7\|CELR3_HUMAN Cadherin EGF LAG seven-pass G-type receptor 3 OS=Homo sapiens GN=CELSR3 I | 1329 | GTVLNVsFSAL | 0.478 | 0.115 | -0.29 | 0.101 |
| sp\|Q9NYQ7\|CELR3_HUMAN Cadherin EGF LAG seven-pass G-type receptor 3 OS=Homo sapiens GN=CELSR3 I | 1331 | VLNVSFsALAP | 0.254 | 0.263 | -0.529 | -0.004 |
| sp\|Q9NYQ7\|CELR3_HUMAN Cadherin EGF LAG seven-pass G-type receptor 3 OS=Homo sapiens GN=CELSR3 I | 1348 | AAGPWFsSEEL | 0.111 | -0.101 | -1.049 | -0.346 |
| sp\|Q9NYQ7\|CELR3_HUMAN Cadherin EGF LAG seven-pass G-type receptor 3 OS=Homo sapiens GN=CELSR3 I | 1349 | AGPWFSsEELQ | 0.089 | -0.234 | -1.302 | -0.482 |
| sp\|Q9NYQ7\|CELR3_HUMAN Cadherin EGF LAG seven-pass G-type receptor 3 OS=Homo sapiens GN=CELSR3 I | 1367 | AALAARsLLDV | 0.372 | 0.036 | -0.748 | -0.113 |
| sp\|Q9NYQ7\|CELR3_HUMAN Cadherin EGF LAG seven-pass G-type receptor 3 OS=Homo sapiens GN=CELSR3 I | 1392 | NYMKCVsVLRF | 0.467 | 0.04 | -0.418 | 0.03 |
| sp\|Q9NYQ7\|CELR3_HUMAN Cadherin EGF LAG seven-pass G-type receptor 3 OS=Homo sapiens GN=CELSR3 I | 1398 | SVLRFDsSAPF | 0.188 | 0.504 | -0.699 | -0.002 |
| sp\|Q9NYQ7\|CELR3_HUMAN Cadherin EGF LAG seven-pass G-type receptor 3 OS=Homo sapiens GN=CELSR3 I | 1399 | VLRFDSsAPFL | 0.671 | 0.699 | 0.164 | 0.511 |
| sp\|Q9NYQ7\|CELR3_HUMAN Cadherin EGF LAG seven-pass G-type receptor 3 OS=Homo sapiens GN=CELSR3 I | 1405 | SAPFLAsASTL | 0.066 | -0.201 | -1.668 | -0.601 |
| sp\|Q9NYQ7\|CELR3_HUMAN Cadherin EGF LAG seven-pass G-type receptor 3 OS=Homo sapiens GN=CELSR3 I | 1407 | PFLASAsTLFR | 0.34 | 0.123 | -0.326 | 0.046 |

| | | | | | | | |
|---|---|---|---|---|---|---|---|
| sp\|Q9NYQ7\|CELR3_HUMAN Cadherin EGF LAG seven-pass G-type receptor 3 OS=Homo sapiens GN=CELSR3 I | 1408 | FLASAStLFRP | 0.384 | 0.06 | -0.333 | 0.037 |
| sp\|Q9NYQ7\|CELR3_HUMAN Cadherin EGF LAG seven-pass G-type receptor 3 OS=Homo sapiens GN=CELSR3 I | 1428 | RCPPGFtGDFC | 0.063 | -0.249 | -1.491 | -0.559 |
| sp\|Q9NYQ7\|CELR3_HUMAN Cadherin EGF LAG seven-pass G-type receptor 3 OS=Homo sapiens GN=CELSR3 I | 1434 | TGDFCEtELDL | 0.279 | 0.038 | -0.703 | -0.129 |
| sp\|Q9NYQ7\|CELR3_HUMAN Cadherin EGF LAG seven-pass G-type receptor 3 OS=Homo sapiens GN=CELSR3 I | 1441 | ELDLCYsNPCR | 0.652 | 0.638 | 0.202 | 0.497 |
| sp\|Q9NYQ7\|CELR3_HUMAN Cadherin EGF LAG seven-pass G-type receptor 3 OS=Homo sapiens GN=CELSR3 I | 1458 | RREGGYtCVCR | 0.218 | 0.358 | -0.509 | 0.022 |
| sp\|Q9NYQ7\|CELR3_HUMAN Cadherin EGF LAG seven-pass G-type receptor 3 OS=Homo sapiens GN=CELSR3 I | 1466 | VCRPRFtGEDC | 0.524 | 0.292 | -0.306 | 0.17 |
| sp\|Q9NYQ7\|CELR3_HUMAN Cadherin EGF LAG seven-pass G-type receptor 3 OS=Homo sapiens GN=CELSR3 I | 1474 | EDCELDtEAGR | 0.049 | -0.305 | -1.563 | -0.606 |
| sp\|Q9NYQ7\|CELR3_HUMAN Cadherin EGF LAG seven-pass G-type receptor 3 OS=Homo sapiens GN=CELSR3 I | 1489 | VCRNGGtCTDA | 0.345 | 0.171 | -0.563 | -0.016 |
| sp\|Q9NYQ7\|CELR3_HUMAN Cadherin EGF LAG seven-pass G-type receptor 3 OS=Homo sapiens GN=CELSR3 I | 1491 | RNGGTCtDAPN | 0.105 | -0.119 | -1.32 | -0.445 |
| sp\|Q9NYQ7\|CELR3_HUMAN Cadherin EGF LAG seven-pass G-type receptor 3 OS=Homo sapiens GN=CELSR3 I | 1519 | CEVAARsFPPS | 0.593 | 0.492 | -0.037 | 0.349 |
| sp\|Q9NYQ7\|CELR3_HUMAN Cadherin EGF LAG seven-pass G-type receptor 3 OS=Homo sapiens GN=CELSR3 I | 1523 | ARSFPPsSFVM | 0.163 | -0.014 | -0.839 | -0.23 |
| sp\|Q9NYQ7\|CELR3_HUMAN Cadherin EGF LAG seven-pass G-type receptor 3 OS=Homo sapiens GN=CELSR3 I | 1524 | RSFPPSsFVMF | 0.084 | -0.259 | -1.13 | -0.435 |
| sp\|Q9NYQ7\|CELR3_HUMAN Cadherin EGF LAG seven-pass G-type receptor 3 OS=Homo sapiens GN=CELSR3 I | 1538 | RQRFHLtLSLS | 0.4 | 0.297 | -0.276 | 0.14 |
| sp\|Q9NYQ7\|CELR3_HUMAN Cadherin EGF LAG seven-pass G-type receptor 3 OS=Homo sapiens GN=CELSR3 I | 1540 | RFHLTLsLSFA | 0.085 | -0.089 | -1.061 | -0.355 |
| sp\|Q9NYQ7\|CELR3_HUMAN Cadherin EGF LAG seven-pass G-type receptor 3 OS=Homo sapiens GN=CELSR3 I | 1542 | HLTLSLsFATV | 0.411 | 0.3 | -0.205 | 0.169 |
| sp\|Q9NYQ7\|CELR3_HUMAN Cadherin EGF LAG seven-pass G-type receptor 3 OS=Homo sapiens GN=CELSR3 I | 1545 | LSLSFAtVQQS | 0.168 | -0.174 | -0.888 | -0.298 |
| sp\|Q9NYQ7\|CELR3_HUMAN Cadherin EGF LAG seven-pass G-type receptor 3 OS=Homo sapiens GN=CELSR3 I | 1549 | FATVQQsGLLF | 0.05 | -0.114 | -1.343 | -0.469 |
| sp\|Q9NYQ7\|CELR3_HUMAN Cadherin EGF LAG seven-pass G-type receptor 3 OS=Homo sapiens GN=CELSR3 I | 1577 | AGQVRLtYSTG | 0.091 | -0.09 | -1.148 | -0.382 |
| sp\|Q9NYQ7\|CELR3_HUMAN Cadherin EGF LAG seven-pass G-type receptor 3 OS=Homo sapiens GN=CELSR3 I | 1579 | QVRLTYsTGES | 0.292 | 0.333 | -0.367 | 0.086 |
| sp\|Q9NYQ7\|CELR3_HUMAN Cadherin EGF LAG seven-pass G-type receptor 3 OS=Homo sapiens GN=CELSR3 I | 1580 | VRLTYStGESN | 0.079 | -0.02 | -1.211 | -0.384 |
| sp\|Q9NYQ7\|CELR3_HUMAN Cadherin EGF LAG seven-pass G-type receptor 3 OS=Homo sapiens GN=CELSR3 I | 1583 | TYSTGEsNTVV | 0.107 | -0.219 | -1.68 | -0.597 |
| sp\|Q9NYQ7\|CELR3_HUMAN Cadherin EGF LAG seven-pass G-type receptor 3 OS=Homo sapiens GN=CELSR3 I | 1585 | STGESNtVVSP | 0.089 | 0.008 | -1.311 | -0.405 |
| sp\|Q9NYQ7\|CELR3_HUMAN Cadherin EGF LAG seven-pass G-type receptor 3 OS=Homo sapiens GN=CELSR3 I | 1588 | ESNTVVsPTVP | 0.081 | -0.491 | -1.571 | -0.66 |
| sp\|Q9NYQ7\|CELR3_HUMAN Cadherin EGF LAG seven-pass G-type receptor 3 OS=Homo sapiens GN=CELSR3 I | 1590 | NTVVSPtVPGG | 0.63 | 0.55 | 0.231 | 0.47 |
| sp\|Q9NYQ7\|CELR3_HUMAN Cadherin EGF LAG seven-pass G-type receptor 3 OS=Homo sapiens GN=CELSR3 I | 1596 | TVPGGLsDGQW | 0.058 | -0.167 | -1.288 | -0.466 |
| sp\|Q9NYQ7\|CELR3_HUMAN Cadherin EGF LAG seven-pass G-type receptor 3 OS=Homo sapiens GN=CELSR3 I | 1602 | SDGQWHtVHLR | 0.182 | 0.03 | -0.777 | -0.188 |
| sp\|Q9NYQ7\|CELR3_HUMAN Cadherin EGF LAG seven-pass G-type receptor 3 OS=Homo sapiens GN=CELSR3 I | 1613 | YYNKPRtDALG | 0.235 | -0.038 | -0.934 | -0.246 |
| sp\|Q9NYQ7\|CELR3_HUMAN Cadherin EGF LAG seven-pass G-type receptor 3 OS=Homo sapiens GN=CELSR3 I | 1623 | GGAQGPsKDKV | 0.187 | -0.132 | -0.788 | -0.244 |
| sp\|Q9NYQ7\|CELR3_HUMAN Cadherin EGF LAG seven-pass G-type receptor 3 OS=Homo sapiens GN=CELSR3 I | 1631 | DKVAVLsVDDC | 0.36 | -0.012 | -0.728 | -0.127 |
| sp\|Q9NYQ7\|CELR3_HUMAN Cadherin EGF LAG seven-pass G-type receptor 3 OS=Homo sapiens GN=CELSR3 I | 1651 | AEIGNYsCAAA | 0.186 | 0.071 | -0.637 | -0.127 |
| sp\|Q9NYQ7\|CELR3_HUMAN Cadherin EGF LAG seven-pass G-type receptor 3 OS=Homo sapiens GN=CELSR3 I | 1659 | AAAGVQtSSKK | 0.069 | -0.301 | -1.375 | -0.536 |
| sp\|Q9NYQ7\|CELR3_HUMAN Cadherin EGF LAG seven-pass G-type receptor 3 OS=Homo sapiens GN=CELSR3 I | 1660 | AAGVQTsSKKS | 0.028 | -0.304 | -1.855 | -0.71 |
| sp\|Q9NYQ7\|CELR3_HUMAN Cadherin EGF LAG seven-pass G-type receptor 3 OS=Homo sapiens GN=CELSR3 I | 1661 | AGVQTSsKKSL | 0.06 | -0.164 | -1.115 | -0.406 |
| sp\|Q9NYQ7\|CELR3_HUMAN Cadherin EGF LAG seven-pass G-type receptor 3 OS=Homo sapiens GN=CELSR3 I | 1664 | QTSSKKsLDLT | 0.274 | -0.053 | -0.401 | -0.06 |
| sp\|Q9NYQ7\|CELR3_HUMAN Cadherin EGF LAG seven-pass G-type receptor 3 OS=Homo sapiens GN=CELSR3 I | 1668 | KKSLDLtGPLL | 0.436 | 0.396 | -0.186 | 0.215 |
| sp\|Q9NYQ7\|CELR3_HUMAN Cadherin EGF LAG seven-pass G-type receptor 3 OS=Homo sapiens GN=CELSR3 I | 1686 | PENFPVsHKDF | 0.319 | -0.074 | -0.604 | -0.12 |
| sp\|Q9NYQ7\|CELR3_HUMAN Cadherin EGF LAG seven-pass G-type receptor 3 OS=Homo sapiens GN=CELSR3 I | 1715 | FVANNGtMAGC | 0.074 | -0.112 | -1.123 | -0.387 |
| sp\|Q9NYQ7\|CELR3_HUMAN Cadherin EGF LAG seven-pass G-type receptor 3 OS=Homo sapiens GN=CELSR3 I | 1728 | KLHFCDsGPCK | 0.596 | 0.406 | -0.17 | 0.277 |
| sp\|Q9NYQ7\|CELR3_HUMAN Cadherin EGF LAG seven-pass G-type receptor 3 OS=Homo sapiens GN=CELSR3 I | 1734 | SGPCKNsGFCS | 0.074 | -0.206 | -1.464 | -0.532 |
| sp\|Q9NYQ7\|CELR3_HUMAN Cadherin EGF LAG seven-pass G-type receptor 3 OS=Homo sapiens GN=CELSR3 I | 1738 | KNSGFCsERWG | 0.195 | -0.154 | -0.929 | -0.296 |
| sp\|Q9NYQ7\|CELR3_HUMAN Cadherin EGF LAG seven-pass G-type receptor 3 OS=Homo sapiens GN=CELSR3 I | 1743 | CSERWGsFSCD | 0.677 | 0.852 | 0.478 | 0.669 |
| sp\|Q9NYQ7\|CELR3_HUMAN Cadherin EGF LAG seven-pass G-type receptor 3 OS=Homo sapiens GN=CELSR3 I | 1745 | ERWGSFsCDCP | 0.3 | 0.653 | -0.099 | 0.285 |
| sp\|Q9NYQ7\|CELR3_HUMAN Cadherin EGF LAG seven-pass G-type receptor 3 OS=Homo sapiens GN=CELSR3 I | 1760 | GKDCQLtMAHP | 0.097 | -0.09 | -1.328 | -0.44 |
| sp\|Q9NYQ7\|CELR3_HUMAN Cadherin EGF LAG seven-pass G-type receptor 3 OS=Homo sapiens GN=CELSR3 I | 1772 | HFRGNGtLSWN | 0.397 | 0.287 | -0.004 | 0.227 |
| sp\|Q9NYQ7\|CELR3_HUMAN Cadherin EGF LAG seven-pass G-type receptor 3 OS=Homo sapiens GN=CELSR3 I | 1774 | RGNGTLsWNFG | 0.077 | -0.007 | -1.057 | -0.329 |
| sp\|Q9NYQ7\|CELR3_HUMAN Cadherin EGF LAG seven-pass G-type receptor 3 OS=Homo sapiens GN=CELSR3 I | 1779 | LSWNFGsDMAV | 0.126 | 0.01 | -1.153 | -0.339 |
| sp\|Q9NYQ7\|CELR3_HUMAN Cadherin EGF LAG seven-pass G-type receptor 3 OS=Homo sapiens GN=CELSR3 I | 1784 | GSDMAVsVPWY | 0.833 | 0.524 | 0.436 | 0.598 |
| sp\|Q9NYQ7\|CELR3_HUMAN Cadherin EGF LAG seven-pass G-type receptor 3 OS=Homo sapiens GN=CELSR3 I | 1795 | LGLAFRtRATQ | 0.068 | -0.354 | -1.5 | -0.595 |
| sp\|Q9NYQ7\|CELR3_HUMAN Cadherin EGF LAG seven-pass G-type receptor 3 OS=Homo sapiens GN=CELSR3 I | 1798 | AFRTRAtQGVL | 0.725 | 0.438 | -0.035 | 0.376 |

| | | | | | | |
|---|---|---|---|---|---|---|
| sp\|Q9NYQ7\|CELR3_HUMAN Cadherin EGF LAG seven-pass G-type receptor 3 OS=Homo sapiens GN=CELSR3 I | 1811 | VQAGPHsTLLC | 0.076 | -0.156 | -1.099 | -0.393 |
| sp\|Q9NYQ7\|CELR3_HUMAN Cadherin EGF LAG seven-pass G-type receptor 3 OS=Homo sapiens GN=CELSR3 I | 1812 | QAGPHStLLCQ | 0.086 | -0.132 | -1.203 | -0.416 |
| sp\|Q9NYQ7\|CELR3_HUMAN Cadherin EGF LAG seven-pass G-type receptor 3 OS=Homo sapiens GN=CELSR3 I | 1823 | LDRGLLsVTVT | 0.107 | -0.007 | -0.975 | -0.292 |
| sp\|Q9NYQ7\|CELR3_HUMAN Cadherin EGF LAG seven-pass G-type receptor 3 OS=Homo sapiens GN=CELSR3 I | 1825 | RGLLSVtVTRG | 0.62 | 0.147 | -0.139 | 0.209 |
| sp\|Q9NYQ7\|CELR3_HUMAN Cadherin EGF LAG seven-pass G-type receptor 3 OS=Homo sapiens GN=CELSR3 I | 1827 | LLSVTVtRGSG | 0.173 | -0.044 | -0.8 | -0.224 |
| sp\|Q9NYQ7\|CELR3_HUMAN Cadherin EGF LAG seven-pass G-type receptor 3 OS=Homo sapiens GN=CELSR3 I | 1830 | VTVTRGsGRAS | 0.181 | -0.117 | -0.978 | -0.305 |
| sp\|Q9NYQ7\|CELR3_HUMAN Cadherin EGF LAG seven-pass G-type receptor 3 OS=Homo sapiens GN=CELSR3 I | 1834 | RGSGRAsHLLL | 0.366 | 0.18 | -0.416 | 0.043 |
| sp\|Q9NYQ7\|CELR3_HUMAN Cadherin EGF LAG seven-pass G-type receptor 3 OS=Homo sapiens GN=CELSR3 I | 1842 | LLLDQVtVSDG | 0.368 | 0.077 | -0.491 | -0.015 |
| sp\|Q9NYQ7\|CELR3_HUMAN Cadherin EGF LAG seven-pass G-type receptor 3 OS=Homo sapiens GN=CELSR3 I | 1844 | LDQVTVsDGRW | 0.158 | -0.045 | -1.008 | -0.298 |
| sp\|Q9NYQ7\|CELR3_HUMAN Cadherin EGF LAG seven-pass G-type receptor 3 OS=Homo sapiens GN=CELSR3 I | 1871 | HHVLMVsLDFS | 0.291 | -0.001 | -0.683 | -0.131 |
| sp\|Q9NYQ7\|CELR3_HUMAN Cadherin EGF LAG seven-pass G-type receptor 3 OS=Homo sapiens GN=CELSR3 I | 1875 | MVSLDFsLFQD | 0.454 | 0.139 | -0.061 | 0.177 |
| sp\|Q9NYQ7\|CELR3_HUMAN Cadherin EGF LAG seven-pass G-type receptor 3 OS=Homo sapiens GN=CELSR3 I | 1880 | FSLFQDtMAVG | 0.13 | -0.006 | -1.234 | -0.37 |
| sp\|Q9NYQ7\|CELR3_HUMAN Cadherin EGF LAG seven-pass G-type receptor 3 OS=Homo sapiens GN=CELSR3 I | 1885 | DTMAVGsELQG | 0.175 | -0.123 | -1.156 | -0.368 |
| sp\|Q9NYQ7\|CELR3_HUMAN Cadherin EGF LAG seven-pass G-type receptor 3 OS=Homo sapiens GN=CELSR3 I | 1904 | GGLPPGsAEEA | 0.126 | -0.209 | -1.018 | -0.367 |
| sp\|Q9NYQ7\|CELR3_HUMAN Cadherin EGF LAG seven-pass G-type receptor 3 OS=Homo sapiens GN=CELSR3 I | 1923 | QGVWLGsTPSG | 0.216 | 0.208 | -0.634 | -0.07 |
| sp\|Q9NYQ7\|CELR3_HUMAN Cadherin EGF LAG seven-pass G-type receptor 3 OS=Homo sapiens GN=CELSR3 I | 1924 | GVWLGStPSGS | 0.019 | -0.443 | -1.946 | -0.79 |
| sp\|Q9NYQ7\|CELR3_HUMAN Cadherin EGF LAG seven-pass G-type receptor 3 OS=Homo sapiens GN=CELSR3 I | 1926 | WLGSTPsGSPA | 0.055 | -0.285 | -1.211 | -0.48 |
| sp\|Q9NYQ7\|CELR3_HUMAN Cadherin EGF LAG seven-pass G-type receptor 3 OS=Homo sapiens GN=CELSR3 I | 1928 | GSTPSGsPALL | 0.108 | -0.253 | -1.017 | -0.387 |
| sp\|Q9NYQ7\|CELR3_HUMAN Cadherin EGF LAG seven-pass G-type receptor 3 OS=Homo sapiens GN=CELSR3 I | 1935 | PALLPPsHRVN | 0.18 | -0.211 | -0.923 | -0.318 |
| sp\|Q9NYQ7\|CELR3_HUMAN Cadherin EGF LAG seven-pass G-type receptor 3 OS=Homo sapiens GN=CELSR3 I | 1947 | EPGCVVtNACA | 0.143 | -0.193 | -1.275 | -0.442 |
| sp\|Q9NYQ7\|CELR3_HUMAN Cadherin EGF LAG seven-pass G-type receptor 3 OS=Homo sapiens GN=CELSR3 I | 1952 | VTNACAsGPCP | 0.548 | 0.349 | -0.294 | 0.201 |
| sp\|Q9NYQ7\|CELR3_HUMAN Cadherin EGF LAG seven-pass G-type receptor 3 OS=Homo sapiens GN=CELSR3 I | 1967 | CRDLWQtFSCT | 0.253 | 0.258 | -0.482 | 0.01 |
| sp\|Q9NYQ7\|CELR3_HUMAN Cadherin EGF LAG seven-pass G-type receptor 3 OS=Homo sapiens GN=CELSR3 I | 1969 | DLWQTFsCTCQ | 0.333 | 0.309 | -0.561 | 0.027 |
| sp\|Q9NYQ7\|CELR3_HUMAN Cadherin EGF LAG seven-pass G-type receptor 3 OS=Homo sapiens GN=CELSR3 I | 1971 | WQTFSCtCQPG | 0.469 | 0.23 | -0.078 | 0.207 |
| sp\|Q9NYQ7\|CELR3_HUMAN Cadherin EGF LAG seven-pass G-type receptor 3 OS=Homo sapiens GN=CELSR3 I | 1995 | PCQNQGsCRHL | 0.118 | -0.009 | -1.003 | -0.298 |
| sp\|Q9NYQ7\|CELR3_HUMAN Cadherin EGF LAG seven-pass G-type receptor 3 OS=Homo sapiens GN=CELSR3 I | 2007 | GAPHGYtCDCV | 0.142 | 0.054 | -1.087 | -0.297 |
| sp\|Q9NYQ7\|CELR3_HUMAN Cadherin EGF LAG seven-pass G-type receptor 3 OS=Homo sapiens GN=CELSR3 I | 2034 | PRGWWGsPTCG | 0.137 | -0.126 | -1.074 | -0.354 |
| sp\|Q9NYQ7\|CELR3_HUMAN Cadherin EGF LAG seven-pass G-type receptor 3 OS=Homo sapiens GN=CELSR3 I | 2036 | GWWGSPtCGPC | 0.308 | 0.308 | -0.459 | 0.052 |
| sp\|Q9NYQ7\|CELR3_HUMAN Cadherin EGF LAG seven-pass G-type receptor 3 OS=Homo sapiens GN=CELSR3 I | 2055 | DPNCNKtNGQC | 0.343 | -0.14 | -0.929 | -0.242 |
| sp\|Q9NYQ7\|CELR3_HUMAN Cadherin EGF LAG seven-pass G-type receptor 3 OS=Homo sapiens GN=CELSR3 I | 2071 | HYRPRGsDSCL | 0.422 | 0.4 | -0.366 | 0.152 |
| sp\|Q9NYQ7\|CELR3_HUMAN Cadherin EGF LAG seven-pass G-type receptor 3 OS=Homo sapiens GN=CELSR3 I | 2073 | RPRGSDsCLPC | 0.315 | 0.266 | -0.708 | -0.042 |
| sp\|Q9NYQ7\|CELR3_HUMAN Cadherin EGF LAG seven-pass G-type receptor 3 OS=Homo sapiens GN=CELSR3 I | 2084 | DCYPVGsTSRS | 0.076 | -0.332 | -1.81 | -0.689 |
| sp\|Q9NYQ7\|CELR3_HUMAN Cadherin EGF LAG seven-pass G-type receptor 3 OS=Homo sapiens GN=CELSR3 I | 2085 | CYPVGStSRSC | 0.025 | -0.403 | -1.899 | -0.759 |
| sp\|Q9NYQ7\|CELR3_HUMAN Cadherin EGF LAG seven-pass G-type receptor 3 OS=Homo sapiens GN=CELSR3 I | 2086 | YPVGSTsRSCA | 0.143 | 0.02 | -0.786 | -0.208 |
| sp\|Q9NYQ7\|CELR3_HUMAN Cadherin EGF LAG seven-pass G-type receptor 3 OS=Homo sapiens GN=CELSR3 I | 2088 | VGSTSRsCAPH | 0.122 | -0.096 | -1.18 | -0.385 |
| sp\|Q9NYQ7\|CELR3_HUMAN Cadherin EGF LAG seven-pass G-type receptor 3 OS=Homo sapiens GN=CELSR3 I | 2093 | RSCAPHsGQCP | 0.18 | -0.091 | -0.882 | -0.264 |
| sp\|Q9NYQ7\|CELR3_HUMAN Cadherin EGF LAG seven-pass G-type receptor 3 OS=Homo sapiens GN=CELSR3 I | 2109 | LGRQCNsCDSP | 0.469 | 0.281 | -0.139 | 0.204 |
| sp\|Q9NYQ7\|CELR3_HUMAN Cadherin EGF LAG seven-pass G-type receptor 3 OS=Homo sapiens GN=CELSR3 I | 2112 | QCNSCDsPFAE | 0.139 | -0.383 | -1.315 | -0.52 |
| sp\|Q9NYQ7\|CELR3_HUMAN Cadherin EGF LAG seven-pass G-type receptor 3 OS=Homo sapiens GN=CELSR3 I | 2118 | SPFAEVtASGC | 0.105 | -0.329 | -1.659 | -0.628 |
| sp\|Q9NYQ7\|CELR3_HUMAN Cadherin EGF LAG seven-pass G-type receptor 3 OS=Homo sapiens GN=CELSR3 I | 2120 | FAEVTAsGCRV | 0.084 | -0.204 | -1.55 | -0.557 |
| sp\|Q9NYQ7\|CELR3_HUMAN Cadherin EGF LAG seven-pass G-type receptor 3 OS=Homo sapiens GN=CELSR3 I | 2132 | YDACPKsLRSG | 0.228 | -0.152 | -0.382 | -0.102 |
| sp\|Q9NYQ7\|CELR3_HUMAN Cadherin EGF LAG seven-pass G-type receptor 3 OS=Homo sapiens GN=CELSR3 I | 2135 | CPKSLRsGVWW | 0.075 | -0.15 | -1.211 | -0.429 |
| sp\|Q9NYQ7\|CELR3_HUMAN Cadherin EGF LAG seven-pass G-type receptor 3 OS=Homo sapiens GN=CELSR3 I | 2142 | GVWWPQtKFGV | 0.21 | -0.006 | -0.604 | -0.133 |
| sp\|Q9NYQ7\|CELR3_HUMAN Cadherin EGF LAG seven-pass G-type receptor 3 OS=Homo sapiens GN=CELSR3 I | 2149 | KFGVLAtVPCP | 0.265 | 0.392 | -0.538 | 0.04 |
| sp\|Q9NYQ7\|CELR3_HUMAN Cadherin EGF LAG seven-pass G-type receptor 3 OS=Homo sapiens GN=CELSR3 I | 2179 | PDLFNCtSPAF | 0.589 | 0.443 | 0.058 | 0.363 |
| sp\|Q9NYQ7\|CELR3_HUMAN Cadherin EGF LAG seven-pass G-type receptor 3 OS=Homo sapiens GN=CELSR3 I | 2180 | DLFNCTsPAFR | 0.143 | -0.324 | -1.012 | -0.398 |
| sp\|Q9NYQ7\|CELR3_HUMAN Cadherin EGF LAG seven-pass G-type receptor 3 OS=Homo sapiens GN=CELSR3 I | 2187 | PAFRELsLLLD | 0.641 | 0.861 | 0.278 | 0.593 |
| sp\|Q9NYQ7\|CELR3_HUMAN Cadherin EGF LAG seven-pass G-type receptor 3 OS=Homo sapiens GN=CELSR3 I | 2198 | GLELNKtALDT | 0.532 | 0.173 | -0.178 | 0.176 |
| sp\|Q9NYQ7\|CELR3_HUMAN Cadherin EGF LAG seven-pass G-type receptor 3 OS=Homo sapiens GN=CELSR3 I | 2202 | NKTALDtMEAK | 0.254 | 0.01 | -0.743 | -0.16 |

| | | | | | | |
|---|---|---|---|---|---|---|
| sp\|Q9NYQ7\|CELR3_HUMAN Cadherin EGF LAG seven-pass G-type receptor 3 OS=Homo sapiens GN=CELSR3 I | 2216 | QRLREVtGHTD | 0.782 | 0.984 | 0.606 | 0.791 |
| sp\|Q9NYQ7\|CELR3_HUMAN Cadherin EGF LAG seven-pass G-type receptor 3 OS=Homo sapiens GN=CELSR3 I | 2219 | REVTGHtDHYF | 0.186 | 0.016 | -1.213 | -0.337 |
| sp\|Q9NYQ7\|CELR3_HUMAN Cadherin EGF LAG seven-pass G-type receptor 3 OS=Homo sapiens GN=CELSR3 I | 2224 | HTDHYFsQDVR | 0.236 | 0.028 | -0.738 | -0.158 |
| sp\|Q9NYQ7\|CELR3_HUMAN Cadherin EGF LAG seven-pass G-type receptor 3 OS=Homo sapiens GN=CELSR3 I | 2230 | SQDVRVtARLL | 0.224 | 0.027 | -0.799 | -0.183 |
| sp\|Q9NYQ7\|CELR3_HUMAN Cadherin EGF LAG seven-pass G-type receptor 3 OS=Homo sapiens GN=CELSR3 I | 2242 | HLLAFEsHQQG | 0.383 | 0.024 | -0.46 | -0.018 |
| sp\|Q9NYQ7\|CELR3_HUMAN Cadherin EGF LAG seven-pass G-type receptor 3 OS=Homo sapiens GN=CELSR3 I | 2250 | QQGFGLtATQD | 0.094 | -0.157 | -1.101 | -0.388 |
| sp\|Q9NYQ7\|CELR3_HUMAN Cadherin EGF LAG seven-pass G-type receptor 3 OS=Homo sapiens GN=CELSR3 I | 2252 | GFGLTAtQDAH | 0.188 | 0.013 | -0.986 | -0.262 |
| sp\|Q9NYQ7\|CELR3_HUMAN Cadherin EGF LAG seven-pass G-type receptor 3 OS=Homo sapiens GN=CELSR3 I | 2266 | NLLWAGsALLA | 0.311 | 0.038 | -0.589 | -0.08 |
| sp\|Q9NYQ7\|CELR3_HUMAN Cadherin EGF LAG seven-pass G-type receptor 3 OS=Homo sapiens GN=CELSR3 I | 2273 | ALLAPEtGDLW | 0.177 | -0.125 | -0.805 | -0.251 |
| sp\|Q9NYQ7\|CELR3_HUMAN Cadherin EGF LAG seven-pass G-type receptor 3 OS=Homo sapiens GN=CELSR3 I | 2288 | QRAPGGsPGSA | 0.032 | -0.351 | -1.447 | -0.589 |
| sp\|Q9NYQ7\|CELR3_HUMAN Cadherin EGF LAG seven-pass G-type receptor 3 OS=Homo sapiens GN=CELSR3 I | 2291 | PGGSPGsAGLV | 0.106 | -0.173 | -1.286 | -0.451 |
| sp\|Q9NYQ7\|CELR3_HUMAN Cadherin EGF LAG seven-pass G-type receptor 3 OS=Homo sapiens GN=CELSR3 I | 2304 | LEEYAAtLARN | 0.451 | 0.049 | -0.382 | 0.039 |
| sp\|Q9NYQ7\|CELR3_HUMAN Cadherin EGF LAG seven-pass G-type receptor 3 OS=Homo sapiens GN=CELSR3 I | 2312 | ARNMELtYLNP | 0.053 | -0.099 | -1.752 | -0.599 |
| sp\|Q9NYQ7\|CELR3_HUMAN Cadherin EGF LAG seven-pass G-type receptor 3 OS=Homo sapiens GN=CELSR3 I | 2321 | NPMGLVtPNIM | 0.111 | -0.356 | -1.071 | -0.439 |
| sp\|Q9NYQ7\|CELR3_HUMAN Cadherin EGF LAG seven-pass G-type receptor 3 OS=Homo sapiens GN=CELSR3 I | 2327 | TPNIMLsIDRM | 0.07 | -0.308 | -1.352 | -0.53 |
| sp\|Q9NYQ7\|CELR3_HUMAN Cadherin EGF LAG seven-pass G-type receptor 3 OS=Homo sapiens GN=CELSR3 I | 2335 | DRMEHPsSPRG | 0.548 | 0.572 | -0.336 | 0.261 |
| sp\|Q9NYQ7\|CELR3_HUMAN Cadherin EGF LAG seven-pass G-type receptor 3 OS=Homo sapiens GN=CELSR3 I | 2336 | RMEHPSsPRGA | 0.051 | -0.503 | -1.322 | -0.591 |
| sp\|Q9NYQ7\|CELR3_HUMAN Cadherin EGF LAG seven-pass G-type receptor 3 OS=Homo sapiens GN=CELSR3 I | 2348 | RYPRYHsNLFR | 0.574 | 0.88 | -0.045 | 0.47 |
| sp\|Q9NYQ7\|CELR3_HUMAN Cadherin EGF LAG seven-pass G-type receptor 3 OS=Homo sapiens GN=CELSR3 I | 2361 | DAWDPHtHVLL | 0.24 | 0.142 | -0.82 | -0.146 |
| sp\|Q9NYQ7\|CELR3_HUMAN Cadherin EGF LAG seven-pass G-type receptor 3 OS=Homo sapiens GN=CELSR3 I | 2367 | THVLLPsQSPR | 0.046 | -0.362 | -2.05 | -0.789 |
| sp\|Q9NYQ7\|CELR3_HUMAN Cadherin EGF LAG seven-pass G-type receptor 3 OS=Homo sapiens GN=CELSR3 I | 2369 | VLLPSQsPRPS | 0.161 | -0.231 | -0.687 | -0.252 |
| sp\|Q9NYQ7\|CELR3_HUMAN Cadherin EGF LAG seven-pass G-type receptor 3 OS=Homo sapiens GN=CELSR3 I | 2373 | SQSPRPsPSEV | 0.052 | -0.444 | -1.667 | -0.686 |
| sp\|Q9NYQ7\|CELR3_HUMAN Cadherin EGF LAG seven-pass G-type receptor 3 OS=Homo sapiens GN=CELSR3 I | 2375 | SPRPSPsEVLP | 0.278 | 0.244 | -0.53 | -0.003 |
| sp\|Q9NYQ7\|CELR3_HUMAN Cadherin EGF LAG seven-pass G-type receptor 3 OS=Homo sapiens GN=CELSR3 I | 2380 | PSEVLPtSSSI | 0.051 | -0.323 | -1.492 | -0.588 |
| sp\|Q9NYQ7\|CELR3_HUMAN Cadherin EGF LAG seven-pass G-type receptor 3 OS=Homo sapiens GN=CELSR3 I | 2381 | SEVLPTsSSIE | 0.052 | -0.355 | -1.734 | -0.679 |
| sp\|Q9NYQ7\|CELR3_HUMAN Cadherin EGF LAG seven-pass G-type receptor 3 OS=Homo sapiens GN=CELSR3 I | 2382 | EVLPTSsSIEN | 0.043 | -0.145 | -1.242 | -0.448 |
| sp\|Q9NYQ7\|CELR3_HUMAN Cadherin EGF LAG seven-pass G-type receptor 3 OS=Homo sapiens GN=CELSR3 I | 2383 | VLPTSSsIENS | 0.146 | 0.053 | -0.948 | -0.25 |
| sp\|Q9NYQ7\|CELR3_HUMAN Cadherin EGF LAG seven-pass G-type receptor 3 OS=Homo sapiens GN=CELSR3 I | 2387 | SSSIENsTTSS | 0.069 | -0.275 | -1.728 | -0.645 |
| sp\|Q9NYQ7\|CELR3_HUMAN Cadherin EGF LAG seven-pass G-type receptor 3 OS=Homo sapiens GN=CELSR3 I | 2388 | SSIENStTSSV | 0.024 | -0.36 | -2.137 | -0.824 |
| sp\|Q9NYQ7\|CELR3_HUMAN Cadherin EGF LAG seven-pass G-type receptor 3 OS=Homo sapiens GN=CELSR3 I | 2389 | SIENSTtSSVV | 0.162 | 0.106 | -0.87 | -0.201 |
| sp\|Q9NYQ7\|CELR3_HUMAN Cadherin EGF LAG seven-pass G-type receptor 3 OS=Homo sapiens GN=CELSR3 I | 2390 | IENSTTsSVVP | 0.059 | -0.189 | -1.474 | -0.535 |
| sp\|Q9NYQ7\|CELR3_HUMAN Cadherin EGF LAG seven-pass G-type receptor 3 OS=Homo sapiens GN=CELSR3 I | 2391 | ENSTTSsVVPP | 0.027 | -0.338 | -1.588 | -0.633 |
| sp\|Q9NYQ7\|CELR3_HUMAN Cadherin EGF LAG seven-pass G-type receptor 3 OS=Homo sapiens GN=CELSR3 I | 2406 | EPEPGIsIIIL | 0.065 | -0.241 | -1.024 | -0.4 |
| sp\|Q9NYQ7\|CELR3_HUMAN Cadherin EGF LAG seven-pass G-type receptor 3 OS=Homo sapiens GN=CELSR3 I | 2415 | ILLVYRtLGGL | 0.508 | 0.174 | -0.013 | 0.223 |
| sp\|Q9NYQ7\|CELR3_HUMAN Cadherin EGF LAG seven-pass G-type receptor 3 OS=Homo sapiens GN=CELSR3 I | 2441 | QNPVMNsPVVS | 0.028 | -0.527 | -1.82 | -0.773 |
| sp\|Q9NYQ7\|CELR3_HUMAN Cadherin EGF LAG seven-pass G-type receptor 3 OS=Homo sapiens GN=CELSR3 I | 2445 | MNSPVVsVAVF | 0.175 | -0.155 | -0.823 | -0.268 |
| sp\|Q9NYQ7\|CELR3_HUMAN Cadherin EGF LAG seven-pass G-type receptor 3 OS=Homo sapiens GN=CELSR3 I | 2461 | LRGILEsPISL | 0.022 | -0.227 | -1.702 | -0.636 |
| sp\|Q9NYQ7\|CELR3_HUMAN Cadherin EGF LAG seven-pass G-type receptor 3 OS=Homo sapiens GN=CELSR3 I | 2464 | ILESPIsLEFR | 0.348 | -0.082 | -0.26 | 0.002 |
| sp\|Q9NYQ7\|CELR3_HUMAN Cadherin EGF LAG seven-pass G-type receptor 3 OS=Homo sapiens GN=CELSR3 I | 2472 | EFRLLQtANRS | 0.295 | 0.159 | -0.666 | -0.071 |
| sp\|Q9NYQ7\|CELR3_HUMAN Cadherin EGF LAG seven-pass G-type receptor 3 OS=Homo sapiens GN=CELSR3 I | 2476 | LQTANRsKAIC | 0.099 | -0.246 | -1.084 | -0.41 |
| sp\|Q9NYQ7\|CELR3_HUMAN Cadherin EGF LAG seven-pass G-type receptor 3 OS=Homo sapiens GN=CELSR3 I | 2496 | EQHGVWtARDC | 0.115 | -0.19 | -1.17 | -0.415 |
| sp\|Q9NYQ7\|CELR3_HUMAN Cadherin EGF LAG seven-pass G-type receptor 3 OS=Homo sapiens GN=CELSR3 I | 2508 | LVHRNGsHARC | 0.267 | 0.52 | -0.563 | 0.075 |
| sp\|Q9NYQ7\|CELR3_HUMAN Cadherin EGF LAG seven-pass G-type receptor 3 OS=Homo sapiens GN=CELSR3 I | 2515 | HARCRCsRTGT | 0.611 | 0.195 | -0.329 | 0.159 |
| sp\|Q9NYQ7\|CELR3_HUMAN Cadherin EGF LAG seven-pass G-type receptor 3 OS=Homo sapiens GN=CELSR3 I | 2517 | RCRCSRtGTFG | 0.615 | 0.366 | -0.339 | 0.214 |
| sp\|Q9NYQ7\|CELR3_HUMAN Cadherin EGF LAG seven-pass G-type receptor 3 OS=Homo sapiens GN=CELSR3 I | 2519 | RCSRTGtFGVL | 0.73 | 1.049 | 0.459 | 0.746 |
| sp\|Q9NYQ7\|CELR3_HUMAN Cadherin EGF LAG seven-pass G-type receptor 3 OS=Homo sapiens GN=CELSR3 I | 2527 | GVLMDAsPRER | 0.17 | -0.36 | -0.989 | -0.393 |
| sp\|Q9NYQ7\|CELR3_HUMAN Cadherin EGF LAG seven-pass G-type receptor 3 OS=Homo sapiens GN=CELSR3 I | 2543 | ELLAVFtHVVV | 0.311 | 0.091 | -0.666 | -0.088 |
| sp\|Q9NYQ7\|CELR3_HUMAN Cadherin EGF LAG seven-pass G-type receptor 3 OS=Homo sapiens GN=CELSR3 I | 2550 | HVVVAVsVAAL | 0.255 | 0.158 | -0.37 | 0.014 |
| sp\|Q9NYQ7\|CELR3_HUMAN Cadherin EGF LAG seven-pass G-type receptor 3 OS=Homo sapiens GN=CELSR3 I | 2557 | VAALVLtAAIL | 0.111 | -0.155 | -1.242 | -0.429 |

| | | | | | | | |
|---|---|---|---|---|---|---|---|
| sp\|Q9NYQ7\|CELR3_HUMAN Cadherin EGF LAG seven-pass G-type receptor 3 OS=Homo sapiens GN=CELSR3 I | 2563 | TAAILLsLRSL | 0.076 | -0.164 | -1.036 | -0.375 |
| sp\|Q9NYQ7\|CELR3_HUMAN Cadherin EGF LAG seven-pass G-type receptor 3 OS=Homo sapiens GN=CELSR3 I | 2566 | ILLSLRsLKSN | 0.186 | -0.019 | -0.774 | -0.202 |
| sp\|Q9NYQ7\|CELR3_HUMAN Cadherin EGF LAG seven-pass G-type receptor 3 OS=Homo sapiens GN=CELSR3 I | 2569 | SLRSLKsNVRG | 0.338 | 0.096 | -0.801 | -0.122 |
| sp\|Q9NYQ7\|CELR3_HUMAN Cadherin EGF LAG seven-pass G-type receptor 3 OS=Homo sapiens GN=CELSR3 I | 2596 | LLGIHRtHNQL | 0.306 | 0.14 | -0.478 | -0.011 |
| sp\|Q9NYQ7\|CELR3_HUMAN Cadherin EGF LAG seven-pass G-type receptor 3 OS=Homo sapiens GN=CELSR3 I | 2603 | HNQLVCtAVAI | 0.371 | 0.175 | -0.501 | 0.015 |
| sp\|Q9NYQ7\|CELR3_HUMAN Cadherin EGF LAG seven-pass G-type receptor 3 OS=Homo sapiens GN=CELSR3 I | 2615 | LHYFFLsTFAW | 0.101 | -0.022 | -1.152 | -0.358 |
| sp\|Q9NYQ7\|CELR3_HUMAN Cadherin EGF LAG seven-pass G-type receptor 3 OS=Homo sapiens GN=CELSR3 I | 2616 | HYFFLStFAWL | 0.174 | 0.075 | -0.685 | -0.145 |
| sp\|Q9NYQ7\|CELR3_HUMAN Cadherin EGF LAG seven-pass G-type receptor 3 OS=Homo sapiens GN=CELSR3 I | 2677 | PDFCWIsVHEP | 0.277 | -0.061 | -0.791 | -0.192 |
| sp\|Q9NYQ7\|CELR3_HUMAN Cadherin EGF LAG seven-pass G-type receptor 3 OS=Homo sapiens GN=CELSR3 I | 2685 | HEPLIWsFAGP | 0.103 | -0.189 | -0.993 | -0.36 |
| sp\|Q9NYQ7\|CELR3_HUMAN Cadherin EGF LAG seven-pass G-type receptor 3 OS=Homo sapiens GN=CELSR3 I | 2699 | VIVMNGtMFLL | 0.166 | 0.052 | -0.814 | -0.199 |
| sp\|Q9NYQ7\|CELR3_HUMAN Cadherin EGF LAG seven-pass G-type receptor 3 OS=Homo sapiens GN=CELSR3 I | 2707 | FLLAARtSCST | 0.125 | -0.065 | -1.051 | -0.33 |
| sp\|Q9NYQ7\|CELR3_HUMAN Cadherin EGF LAG seven-pass G-type receptor 3 OS=Homo sapiens GN=CELSR3 I | 2708 | LLAARTsCSTG | 0.383 | 0.17 | -0.261 | 0.097 |
| sp\|Q9NYQ7\|CELR3_HUMAN Cadherin EGF LAG seven-pass G-type receptor 3 OS=Homo sapiens GN=CELSR3 I | 2710 | AARTSCsTGQR | 0.736 | 0.362 | 0.05 | 0.383 |
| sp\|Q9NYQ7\|CELR3_HUMAN Cadherin EGF LAG seven-pass G-type receptor 3 OS=Homo sapiens GN=CELSR3 I | 2711 | ARTSCStGQRE | 0.194 | -0.094 | -0.997 | -0.299 |
| sp\|Q9NYQ7\|CELR3_HUMAN Cadherin EGF LAG seven-pass G-type receptor 3 OS=Homo sapiens GN=CELSR3 I | 2719 | QREAKKtSALT | 0.129 | 0 | -0.768 | -0.213 |
| sp\|Q9NYQ7\|CELR3_HUMAN Cadherin EGF LAG seven-pass G-type receptor 3 OS=Homo sapiens GN=CELSR3 I | 2720 | REAKKTsALTL | 0.208 | 0.011 | -0.867 | -0.216 |
| sp\|Q9NYQ7\|CELR3_HUMAN Cadherin EGF LAG seven-pass G-type receptor 3 OS=Homo sapiens GN=CELSR3 I | 2723 | KKTSALtLRSS | 0.255 | -0.047 | -0.544 | -0.112 |
| sp\|Q9NYQ7\|CELR3_HUMAN Cadherin EGF LAG seven-pass G-type receptor 3 OS=Homo sapiens GN=CELSR3 I | 2726 | SALTLRsSFLL | 0.057 | -0.204 | -1.601 | -0.583 |
| sp\|Q9NYQ7\|CELR3_HUMAN Cadherin EGF LAG seven-pass G-type receptor 3 OS=Homo sapiens GN=CELSR3 I | 2727 | ALTLRSsFLLL | 0.355 | 0.221 | -0.399 | 0.059 |
| sp\|Q9NYQ7\|CELR3_HUMAN Cadherin EGF LAG seven-pass G-type receptor 3 OS=Homo sapiens GN=CELSR3 I | 2735 | LLLLLVsASWL | 0.245 | 0.048 | -0.693 | -0.133 |
| sp\|Q9NYQ7\|CELR3_HUMAN Cadherin EGF LAG seven-pass G-type receptor 3 OS=Homo sapiens GN=CELSR3 I | 2737 | LLLVSAsWLFG | 0.267 | 0.266 | -0.423 | 0.037 |
| sp\|Q9NYQ7\|CELR3_HUMAN Cadherin EGF LAG seven-pass G-type receptor 3 OS=Homo sapiens GN=CELSR3 I | 2748 | LLAVNHsILAF | 0.195 | 0.13 | -0.368 | -0.014 |
| sp\|Q9NYQ7\|CELR3_HUMAN Cadherin EGF LAG seven-pass G-type receptor 3 OS=Homo sapiens GN=CELSR3 I | 2810 | PGAYNNtALFE | 0.194 | -0.05 | -0.772 | -0.209 |
| sp\|Q9NYQ7\|CELR3_HUMAN Cadherin EGF LAG seven-pass G-type receptor 3 OS=Homo sapiens GN=CELSR3 I | 2816 | TALFEEsGLIR | 0.099 | -0.26 | -1.393 | -0.518 |
| sp\|Q9NYQ7\|CELR3_HUMAN Cadherin EGF LAG seven-pass G-type receptor 3 OS=Homo sapiens GN=CELSR3 I | 2822 | SGLIRItLGAS | 0.52 | 0.241 | 0.022 | 0.261 |
| sp\|Q9NYQ7\|CELR3_HUMAN Cadherin EGF LAG seven-pass G-type receptor 3 OS=Homo sapiens GN=CELSR3 I | 2826 | RITLGAsTVSS | 0.094 | -0.036 | -1.217 | -0.386 |
| sp\|Q9NYQ7\|CELR3_HUMAN Cadherin EGF LAG seven-pass G-type receptor 3 OS=Homo sapiens GN=CELSR3 I | 2827 | ITLGAStVSSV | 0.098 | -0.158 | -1.24 | -0.433 |
| sp\|Q9NYQ7\|CELR3_HUMAN Cadherin EGF LAG seven-pass G-type receptor 3 OS=Homo sapiens GN=CELSR3 I | 2829 | LGASTVsSVSS | 0.068 | -0.201 | -1.164 | -0.432 |
| sp\|Q9NYQ7\|CELR3_HUMAN Cadherin EGF LAG seven-pass G-type receptor 3 OS=Homo sapiens GN=CELSR3 I | 2830 | GASTVSsVSSA | 0.078 | -0.292 | -1.518 | -0.577 |
| sp\|Q9NYQ7\|CELR3_HUMAN Cadherin EGF LAG seven-pass G-type receptor 3 OS=Homo sapiens GN=CELSR3 I | 2832 | STVSSVsSARS | 0.183 | -0.15 | -1.19 | -0.386 |
| sp\|Q9NYQ7\|CELR3_HUMAN Cadherin EGF LAG seven-pass G-type receptor 3 OS=Homo sapiens GN=CELSR3 I | 2833 | TVSSVSsARSG | 0.06 | -0.293 | -1.544 | -0.592 |
| sp\|Q9NYQ7\|CELR3_HUMAN Cadherin EGF LAG seven-pass G-type receptor 3 OS=Homo sapiens GN=CELSR3 I | 2836 | SVSSARsGRTQ | 0.067 | -0.311 | -1.504 | -0.583 |
| sp\|Q9NYQ7\|CELR3_HUMAN Cadherin EGF LAG seven-pass G-type receptor 3 OS=Homo sapiens GN=CELSR3 I | 2839 | SARSGRtQDQD | 0.154 | -0.051 | -1.185 | -0.361 |
| sp\|Q9NYQ7\|CELR3_HUMAN Cadherin EGF LAG seven-pass G-type receptor 3 OS=Homo sapiens GN=CELSR3 I | 2844 | RTQDQDsQRGR | 0.18 | -0.197 | -1.36 | -0.459 |
| sp\|Q9NYQ7\|CELR3_HUMAN Cadherin EGF LAG seven-pass G-type receptor 3 OS=Homo sapiens GN=CELSR3 I | 2849 | DSQRGRsYLRD | 0.356 | 0.615 | -0.531 | 0.147 |
| sp\|Q9NYQ7\|CELR3_HUMAN Cadherin EGF LAG seven-pass G-type receptor 3 OS=Homo sapiens GN=CELSR3 I | 2861 | VLVRHGsAADH | 0.614 | 0.8 | -0.069 | 0.448 |
| sp\|Q9NYQ7\|CELR3_HUMAN Cadherin EGF LAG seven-pass G-type receptor 3 OS=Homo sapiens GN=CELSR3 I | 2866 | GSAADHtDHSL | 0.281 | 0.053 | -0.719 | -0.128 |
| sp\|Q9NYQ7\|CELR3_HUMAN Cadherin EGF LAG seven-pass G-type receptor 3 OS=Homo sapiens GN=CELSR3 I | 2869 | ADHTDHsLQAH | 0.192 | -0.075 | -0.897 | -0.26 |
| sp\|Q9NYQ7\|CELR3_HUMAN Cadherin EGF LAG seven-pass G-type receptor 3 OS=Homo sapiens GN=CELSR3 I | 2877 | QAHAGPtDLDV | 0.085 | -0.24 | -1.584 | -0.58 |
| sp\|Q9NYQ7\|CELR3_HUMAN Cadherin EGF LAG seven-pass G-type receptor 3 OS=Homo sapiens GN=CELSR3 I | 2892 | RDAGADsDSDS | 0.176 | -0.093 | -1.013 | -0.31 |
| sp\|Q9NYQ7\|CELR3_HUMAN Cadherin EGF LAG seven-pass G-type receptor 3 OS=Homo sapiens GN=CELSR3 I | 2894 | AGADSDsDSDL | 0.378 | 0.13 | -0.506 | 0.001 |
| sp\|Q9NYQ7\|CELR3_HUMAN Cadherin EGF LAG seven-pass G-type receptor 3 OS=Homo sapiens GN=CELSR3 I | 2896 | ADSDSDsDLSL | 0.115 | -0.052 | -1.291 | -0.409 |
| sp\|Q9NYQ7\|CELR3_HUMAN Cadherin EGF LAG seven-pass G-type receptor 3 OS=Homo sapiens GN=CELSR3 I | 2899 | DSDSDLsLEEE | 0.16 | -0.218 | -1.103 | -0.387 |
| sp\|Q9NYQ7\|CELR3_HUMAN Cadherin EGF LAG seven-pass G-type receptor 3 OS=Homo sapiens GN=CELSR3 I | 2905 | SLEEERsLSIP | 0.106 | -0.129 | -1.322 | -0.448 |
| sp\|Q9NYQ7\|CELR3_HUMAN Cadherin EGF LAG seven-pass G-type receptor 3 OS=Homo sapiens GN=CELSR3 I | 2907 | EEERSLsIPSS | 0.723 | 1.346 | 0.83 | 0.966 |
| sp\|Q9NYQ7\|CELR3_HUMAN Cadherin EGF LAG seven-pass G-type receptor 3 OS=Homo sapiens GN=CELSR3 I | 2910 | RSLSIPsSESE | 0.07 | -0.318 | -1.331 | -0.526 |
| sp\|Q9NYQ7\|CELR3_HUMAN Cadherin EGF LAG seven-pass G-type receptor 3 OS=Homo sapiens GN=CELSR3 I | 2911 | SLSIPSsESED | 0.111 | -0.109 | -1.024 | -0.341 |
| sp\|Q9NYQ7\|CELR3_HUMAN Cadherin EGF LAG seven-pass G-type receptor 3 OS=Homo sapiens GN=CELSR3 I | 2913 | SIPSSEsEDNG | 0.27 | 0.198 | -0.793 | -0.108 |
| sp\|Q9NYQ7\|CELR3_HUMAN Cadherin EGF LAG seven-pass G-type receptor 3 OS=Homo sapiens GN=CELSR3 I | 2919 | SEDNGRtRGRF | 0.033 | -0.383 | -1.924 | -0.758 |

| | | | | | | |
|---|---|---|---|---|---|---|
| sp\|Q9NYQ7\|CELR3_HUMAN Cadherin EGF LAG seven-pass G-type receptor 3 OS=Homo sapiens GN=CELSR3 I | 2933 | LCRAAQsERLL | 0.649 | 0.389 | 0.102 | 0.38 |
| sp\|Q9NYQ7\|CELR3_HUMAN Cadherin EGF LAG seven-pass G-type receptor 3 OS=Homo sapiens GN=CELSR3 I | 2938 | QSERLLtHPKD | 0.768 | 1.162 | 0.721 | 0.884 |
| sp\|Q9NYQ7\|CELR3_HUMAN Cadherin EGF LAG seven-pass G-type receptor 3 OS=Homo sapiens GN=CELSR3 I | 2950 | DGNDLLsYWPA | 0.092 | -0.248 | -1.471 | -0.542 |
| sp\|Q9NYQ7\|CELR3_HUMAN Cadherin EGF LAG seven-pass G-type receptor 3 OS=Homo sapiens GN=CELSR3 I | 2967 | APCALQtWGSE | 0.102 | -0.126 | -1.254 | -0.426 |
| sp\|Q9NYQ7\|CELR3_HUMAN Cadherin EGF LAG seven-pass G-type receptor 3 OS=Homo sapiens GN=CELSR3 I | 2970 | ALQTWGsERRL | 0.334 | 0.007 | -0.744 | -0.134 |
| sp\|Q9NYQ7\|CELR3_HUMAN Cadherin EGF LAG seven-pass G-type receptor 3 OS=Homo sapiens GN=CELSR3 I | 2978 | RRLGLDtSKDA | 0.189 | 0.07 | -0.826 | -0.189 |
| sp\|Q9NYQ7\|CELR3_HUMAN Cadherin EGF LAG seven-pass G-type receptor 3 OS=Homo sapiens GN=CELSR3 I | 2979 | RLGLDTsKDAA | 0.356 | 0.124 | -0.257 | 0.074 |
| sp\|Q9NYQ7\|CELR3_HUMAN Cadherin EGF LAG seven-pass G-type receptor 3 OS=Homo sapiens GN=CELSR3 I | 2993 | QPDPALtSGDE | 0.163 | -0.121 | -0.924 | -0.294 |
| sp\|Q9NYQ7\|CELR3_HUMAN Cadherin EGF LAG seven-pass G-type receptor 3 OS=Homo sapiens GN=CELSR3 I | 2994 | PDPALTsGDET | 0.076 | -0.29 | -1.208 | -0.474 |
| sp\|Q9NYQ7\|CELR3_HUMAN Cadherin EGF LAG seven-pass G-type receptor 3 OS=Homo sapiens GN=CELSR3 I | 2998 | LTSGDEtSLGR | 0.077 | -0.297 | -1.353 | -0.524 |
| sp\|Q9NYQ7\|CELR3_HUMAN Cadherin EGF LAG seven-pass G-type receptor 3 OS=Homo sapiens GN=CELSR3 I | 2999 | TSGDETsLGRA | 0.262 | -0.136 | -0.802 | -0.225 |
| sp\|Q9NYQ7\|CELR3_HUMAN Cadherin EGF LAG seven-pass G-type receptor 3 OS=Homo sapiens GN=CELSR3 I | 3023 | YPLVPQtRGAP | 0.089 | -0.199 | -0.95 | -0.353 |
| sp\|Q9NYQ7\|CELR3_HUMAN Cadherin EGF LAG seven-pass G-type receptor 3 OS=Homo sapiens GN=CELSR3 I | 3030 | RGAPELsWCRA | 0.085 | -0.268 | -1.305 | -0.496 |
| sp\|Q9NYQ7\|CELR3_HUMAN Cadherin EGF LAG seven-pass G-type receptor 3 OS=Homo sapiens GN=CELSR3 I | 3036 | SWCRAAtLGHR | 0.859 | 1.039 | 0.908 | 0.935 |
| sp\|Q9NYQ7\|CELR3_HUMAN Cadherin EGF LAG seven-pass G-type receptor 3 OS=Homo sapiens GN=CELSR3 I | 3046 | RAVPAAsYGRI | 0.404 | -0.004 | -0.547 | -0.049 |
| sp\|Q9NYQ7\|CELR3_HUMAN Cadherin EGF LAG seven-pass G-type receptor 3 OS=Homo sapiens GN=CELSR3 I | 3056 | IYAGGGtGSLS | 0.053 | -0.278 | -1.31 | -0.512 |
| sp\|Q9NYQ7\|CELR3_HUMAN Cadherin EGF LAG seven-pass G-type receptor 3 OS=Homo sapiens GN=CELSR3 I | 3058 | AGGGTGsLSQP | 0.055 | -0.227 | -1.224 | -0.465 |
| sp\|Q9NYQ7\|CELR3_HUMAN Cadherin EGF LAG seven-pass G-type receptor 3 OS=Homo sapiens GN=CELSR3 I | 3060 | GGTGSLsQPAS | 0.621 | 0.657 | 0.32 | 0.533 |
| sp\|Q9NYQ7\|CELR3_HUMAN Cadherin EGF LAG seven-pass G-type receptor 3 OS=Homo sapiens GN=CELSR3 I | 3064 | SLSQPAsRYSS | 0.097 | -0.224 | -1.201 | -0.443 |
| sp\|Q9NYQ7\|CELR3_HUMAN Cadherin EGF LAG seven-pass G-type receptor 3 OS=Homo sapiens GN=CELSR3 I | 3067 | QPASRYsSREQ | 0.208 | -0.051 | -0.703 | -0.182 |
| sp\|Q9NYQ7\|CELR3_HUMAN Cadherin EGF LAG seven-pass G-type receptor 3 OS=Homo sapiens GN=CELSR3 I | 3068 | PASRYSsREQL | 0.441 | 0.654 | -0.06 | 0.345 |
| sp\|Q9NYQ7\|CELR3_HUMAN Cadherin EGF LAG seven-pass G-type receptor 3 OS=Homo sapiens GN=CELSR3 I | 3081 | LLRRQLsRERL | 0.634 | 0.995 | 0.234 | 0.621 |
| sp\|Q9NYQ7\|CELR3_HUMAN Cadherin EGF LAG seven-pass G-type receptor 3 OS=Homo sapiens GN=CELSR3 I | 3097 | PVLRPLsRPGS | 0.676 | 1.084 | 0.661 | 0.807 |
| sp\|Q9NYQ7\|CELR3_HUMAN Cadherin EGF LAG seven-pass G-type receptor 3 OS=Homo sapiens GN=CELSR3 I | 3101 | PLSRPGsQECM | 0.495 | 0.738 | -0.042 | 0.397 |
| sp\|Q9NYQ7\|CELR3_HUMAN Cadherin EGF LAG seven-pass G-type receptor 3 OS=Homo sapiens GN=CELSR3 I | 3119 | EPKDRGsTLPR | 0.135 | -0.07 | -1.063 | -0.333 |
| sp\|Q9NYQ7\|CELR3_HUMAN Cadherin EGF LAG seven-pass G-type receptor 3 OS=Homo sapiens GN=CELSR3 I | 3120 | PKDRGStLPRR | 0.904 | 1.308 | 1.226 | 1.146 |
| sp\|Q9NYQ7\|CELR3_HUMAN Cadherin EGF LAG seven-pass G-type receptor 3 OS=Homo sapiens GN=CELSR3 I | 3140 | MAGRFGsRDAL | 0.554 | 0.823 | 0.082 | 0.486 |
| sp\|Q9NYQ7\|CELR3_HUMAN Cadherin EGF LAG seven-pass G-type receptor 3 OS=Homo sapiens GN=CELSR3 I | 3154 | APREWLsTLPP | 0.077 | -0.062 | -1.358 | -0.448 |
| sp\|Q9NYQ7\|CELR3_HUMAN Cadherin EGF LAG seven-pass G-type receptor 3 OS=Homo sapiens GN=CELSR3 I | 3155 | PREWLStLPPP | 0.561 | 0.531 | 0.172 | 0.421 |
| sp\|Q9NYQ7\|CELR3_HUMAN Cadherin EGF LAG seven-pass G-type receptor 3 OS=Homo sapiens GN=CELSR3 I | 3162 | LPPPRRtRDLD | 0.155 | -0.066 | -0.979 | -0.297 |
| sp\|Q9NYQ7\|CELR3_HUMAN Cadherin EGF LAG seven-pass G-type receptor 3 OS=Homo sapiens GN=CELSR3 I | 3175 | PPPLPLsPQRQ | 0.033 | -0.647 | -1.977 | -0.864 |
| sp\|Q9NYQ7\|CELR3_HUMAN Cadherin EGF LAG seven-pass G-type receptor 3 OS=Homo sapiens GN=CELSR3 I | 3181 | SPQRQLsRDPL | 0.247 | 0.678 | -0.467 | 0.153 |
| sp\|Q9NYQ7\|CELR3_HUMAN Cadherin EGF LAG seven-pass G-type receptor 3 OS=Homo sapiens GN=CELSR3 I | 3188 | RDPLLPsRPLD | 0.259 | 0.195 | -0.765 | -0.104 |
| sp\|Q9NYQ7\|CELR3_HUMAN Cadherin EGF LAG seven-pass G-type receptor 3 OS=Homo sapiens GN=CELSR3 I | 3193 | PSRPLDsLSRS | 0.244 | -0.078 | -0.956 | -0.263 |
| sp\|Q9NYQ7\|CELR3_HUMAN Cadherin EGF LAG seven-pass G-type receptor 3 OS=Homo sapiens GN=CELSR3 I | 3195 | RPLDSLsRSSN | 0.101 | -0.032 | -1.229 | -0.387 |
| sp\|Q9NYQ7\|CELR3_HUMAN Cadherin EGF LAG seven-pass G-type receptor 3 OS=Homo sapiens GN=CELSR3 I | 3197 | LDSLSRsSNSR | 0.102 | -0.015 | -0.944 | -0.286 |
| sp\|Q9NYQ7\|CELR3_HUMAN Cadherin EGF LAG seven-pass G-type receptor 3 OS=Homo sapiens GN=CELSR3 I | 3198 | DSLSRSsNSRE | 0.119 | -0.308 | -1.554 | -0.581 |
| sp\|Q9NYQ7\|CELR3_HUMAN Cadherin EGF LAG seven-pass G-type receptor 3 OS=Homo sapiens GN=CELSR3 I | 3200 | LSRSSNsREQL | 0.502 | 0.278 | -0.133 | 0.216 |
| sp\|Q9NYQ7\|CELR3_HUMAN Cadherin EGF LAG seven-pass G-type receptor 3 OS=Homo sapiens GN=CELSR3 I | 3209 | QLDQVPsRHPS | 0.179 | -0.203 | -1.006 | -0.343 |
| sp\|Q9NYQ7\|CELR3_HUMAN Cadherin EGF LAG seven-pass G-type receptor 3 OS=Homo sapiens GN=CELSR3 I | 3213 | VPSRHPsREAL | 0.533 | 0.742 | 0.094 | 0.456 |
| sp\|Q9NYQ7\|CELR3_HUMAN Cadherin EGF LAG seven-pass G-type receptor 3 OS=Homo sapiens GN=CELSR3 I | 3230 | LRAREDsVSGP | 0.39 | 0.779 | -0.082 | 0.362 |
| sp\|Q9NYQ7\|CELR3_HUMAN Cadherin EGF LAG seven-pass G-type receptor 3 OS=Homo sapiens GN=CELSR3 I | 3232 | AREDSVsGPSH | 0.672 | 0.654 | 0.119 | 0.482 |
| sp\|Q9NYQ7\|CELR3_HUMAN Cadherin EGF LAG seven-pass G-type receptor 3 OS=Homo sapiens GN=CELSR3 I | 3235 | DSVSGPsHGPS | 0.099 | -0.328 | -1.339 | -0.523 |
| sp\|Q9NYQ7\|CELR3_HUMAN Cadherin EGF LAG seven-pass G-type receptor 3 OS=Homo sapiens GN=CELSR3 I | 3239 | GPSHGPsTEQL | 0.103 | -0.232 | -1.169 | -0.433 |
| sp\|Q9NYQ7\|CELR3_HUMAN Cadherin EGF LAG seven-pass G-type receptor 3 OS=Homo sapiens GN=CELSR3 I | 3240 | PSHGPStEQLD | 0.101 | -0.139 | -0.871 | -0.303 |
| sp\|Q9NYQ7\|CELR3_HUMAN Cadherin EGF LAG seven-pass G-type receptor 3 OS=Homo sapiens GN=CELSR3 I | 3247 | EQLDILsSILA | 0.053 | -0.156 | -0.943 | -0.349 |
| sp\|Q9NYQ7\|CELR3_HUMAN Cadherin EGF LAG seven-pass G-type receptor 3 OS=Homo sapiens GN=CELSR3 I | 3248 | QLDILSsILAS | 0.059 | -0.124 | -1.296 | -0.454 |
| sp\|Q9NYQ7\|CELR3_HUMAN Cadherin EGF LAG seven-pass G-type receptor 3 OS=Homo sapiens GN=CELSR3 I | 3252 | LSSILAsFNSS | 0.109 | -0.106 | -0.974 | -0.324 |
| sp\|Q9NYQ7\|CELR3_HUMAN Cadherin EGF LAG seven-pass G-type receptor 3 OS=Homo sapiens GN=CELSR3 I | 3255 | ILASFNsSALS | 0.222 | -0.08 | -0.881 | -0.246 |

| | | | | | | |
|---|---|---|---|---|---|---|
| sp\|Q9NYQ7\|CELR3_HUMAN Cadherin EGF LAG seven-pass G-type receptor 3 OS=Homo sapiens GN=CELSR3 I | 3256 | LASFNSsALSS | 0.052 | -0.234 | -1.54 | -0.574 |
| sp\|Q9NYQ7\|CELR3_HUMAN Cadherin EGF LAG seven-pass G-type receptor 3 OS=Homo sapiens GN=CELSR3 I | 3259 | FNSSALsSVQS | 0.086 | -0.193 | -1.182 | -0.43 |
| sp\|Q9NYQ7\|CELR3_HUMAN Cadherin EGF LAG seven-pass G-type receptor 3 OS=Homo sapiens GN=CELSR3 I | 3260 | NSSALSsVQSS | 0.05 | -0.322 | -1.515 | -0.596 |
| sp\|Q9NYQ7\|CELR3_HUMAN Cadherin EGF LAG seven-pass G-type receptor 3 OS=Homo sapiens GN=CELSR3 I | 3263 | ALSSVQsSSTP | 0.09 | -0.254 | -1.324 | -0.496 |
| sp\|Q9NYQ7\|CELR3_HUMAN Cadherin EGF LAG seven-pass G-type receptor 3 OS=Homo sapiens GN=CELSR3 I | 3264 | LSSVQSsSTPL | 0.044 | -0.321 | -1.604 | -0.627 |
| sp\|Q9NYQ7\|CELR3_HUMAN Cadherin EGF LAG seven-pass G-type receptor 3 OS=Homo sapiens GN=CELSR3 I | 3265 | SSVQSSsTPLG | 0.494 | 0.499 | -0.005 | 0.329 |
| sp\|Q9NYQ7\|CELR3_HUMAN Cadherin EGF LAG seven-pass G-type receptor 3 OS=Homo sapiens GN=CELSR3 I | 3266 | SVQSSStPLGP | 0.041 | -0.376 | -1.607 | -0.647 |
| sp\|Q9NYQ7\|CELR3_HUMAN Cadherin EGF LAG seven-pass G-type receptor 3 OS=Homo sapiens GN=CELSR3 I | 3272 | TPLGPHtTATP | 0.072 | -0.234 | -1.19 | -0.451 |
| sp\|Q9NYQ7\|CELR3_HUMAN Cadherin EGF LAG seven-pass G-type receptor 3 OS=Homo sapiens GN=CELSR3 I | 3273 | PLGPHTtATPS | 0.253 | 0.003 | -0.8 | -0.181 |
| sp\|Q9NYQ7\|CELR3_HUMAN Cadherin EGF LAG seven-pass G-type receptor 3 OS=Homo sapiens GN=CELSR3 I | 3275 | GPHTTAtPSAT | 0.056 | -0.368 | -1.599 | -0.637 |
| sp\|Q9NYQ7\|CELR3_HUMAN Cadherin EGF LAG seven-pass G-type receptor 3 OS=Homo sapiens GN=CELSR3 I | 3277 | HTTATPsATAS | 0.195 | 0.014 | -0.79 | -0.194 |
| sp\|Q9NYQ7\|CELR3_HUMAN Cadherin EGF LAG seven-pass G-type receptor 3 OS=Homo sapiens GN=CELSR3 I | 3279 | TATPSAtASVL | 0.251 | 0.14 | -0.611 | -0.073 |
| sp\|Q9NYQ7\|CELR3_HUMAN Cadherin EGF LAG seven-pass G-type receptor 3 OS=Homo sapiens GN=CELSR3 I | 3281 | TPSATAsVLGP | 0.077 | -0.23 | -1.301 | -0.485 |
| sp\|Q9NYQ7\|CELR3_HUMAN Cadherin EGF LAG seven-pass G-type receptor 3 OS=Homo sapiens GN=CELSR3 I | 3286 | ASVLGPsTPRS | 0.225 | 0.108 | -0.946 | -0.204 |
| sp\|Q9NYQ7\|CELR3_HUMAN Cadherin EGF LAG seven-pass G-type receptor 3 OS=Homo sapiens GN=CELSR3 I | 3287 | SVLGPStPRSA | 0.017 | -0.675 | -1.866 | -0.841 |
| sp\|Q9NYQ7\|CELR3_HUMAN Cadherin EGF LAG seven-pass G-type receptor 3 OS=Homo sapiens GN=CELSR3 I | 3290 | GPSTPRsATSH | 0.062 | -0.331 | -1.84 | -0.703 |
| sp\|Q9NYQ7\|CELR3_HUMAN Cadherin EGF LAG seven-pass G-type receptor 3 OS=Homo sapiens GN=CELSR3 I | 3292 | STPRSAtSHSI | 0.667 | 0.949 | 0.251 | 0.622 |
| sp\|Q9NYQ7\|CELR3_HUMAN Cadherin EGF LAG seven-pass G-type receptor 3 OS=Homo sapiens GN=CELSR3 I | 3293 | TPRSATsHSIS | 0.305 | 0.075 | -0.688 | -0.103 |
| sp\|Q9NYQ7\|CELR3_HUMAN Cadherin EGF LAG seven-pass G-type receptor 3 OS=Homo sapiens GN=CELSR3 I | 3295 | RSATSHsISEL | 0.381 | 0.227 | -0.258 | 0.117 |
| sp\|Q9NYQ7\|CELR3_HUMAN Cadherin EGF LAG seven-pass G-type receptor 3 OS=Homo sapiens GN=CELSR3 I | 3297 | ATSHSIsELSP | 0.155 | 0.003 | -0.887 | -0.243 |
| sp\|Q9NYQ7\|CELR3_HUMAN Cadherin EGF LAG seven-pass G-type receptor 3 OS=Homo sapiens GN=CELSR3 I | 3300 | HSISELsPDSE | 0.031 | -0.499 | -1.817 | -0.762 |
| sp\|Q9NYQ7\|CELR3_HUMAN Cadherin EGF LAG seven-pass G-type receptor 3 OS=Homo sapiens GN=CELSR3 I | 3303 | SELSPDsEVPR | 0.065 | -0.321 | -1.468 | -0.575 |
| sp\|Q9NYQ7\|CELR3_HUMAN Cadherin EGF LAG seven-pass G-type receptor 3 OS=Homo sapiens GN=CELSR3 I | 3308 | DSEVPRsEGHS | 0.092 | -0.213 | -1.186 | -0.436 |
| sp\|Q9NYQ7\|CELR3_HUMAN Cadherin EGF LAG seven-pass G-type receptor 3 OS=Homo sapiens GN=CELSR3 I | 3312 | PRSEGHs---- | 0.185 | 0.357 | -0.789 | -0.082 |
| sp\|Q9HBW9\|AGRL4_HUMAN Adhesion G protein-coupled receptor L4 OS=Homo sapiens GN=ADGRL4 PE=1 S | 325 | PQNYDNsEEEE | 0.26 | 0.051 | -0.659 | -0.116 |
| sp\|Q9HBW9\|AGRL4_HUMAN Adhesion G protein-coupled receptor L4 OS=Homo sapiens GN=ADGRL4 PE=1 S | 333 | EEERVIsSVIS | 0.488 | 0.708 | -0.139 | 0.352 |
| sp\|Q9HBW9\|AGRL4_HUMAN Adhesion G protein-coupled receptor L4 OS=Homo sapiens GN=ADGRL4 PE=1 S | 334 | EERVISsVISV | 0.067 | 0.045 | -1.15 | -0.346 |
| sp\|Q9HBW9\|AGRL4_HUMAN Adhesion G protein-coupled receptor L4 OS=Homo sapiens GN=ADGRL4 PE=1 S | 337 | VISSVIsVSMS | 0.111 | -0.232 | -1.3 | -0.474 |
| sp\|Q9HBW9\|AGRL4_HUMAN Adhesion G protein-coupled receptor L4 OS=Homo sapiens GN=ADGRL4 PE=1 S | 339 | SSVISVsMSSN | 0.135 | 0.096 | -1.053 | -0.274 |
| sp\|Q9HBW9\|AGRL4_HUMAN Adhesion G protein-coupled receptor L4 OS=Homo sapiens GN=ADGRL4 PE=1 S | 341 | VISVSMsSNPP | 0.232 | 0.17 | -0.453 | -0.017 |
| sp\|Q9HBW9\|AGRL4_HUMAN Adhesion G protein-coupled receptor L4 OS=Homo sapiens GN=ADGRL4 PE=1 S | 342 | ISVSMSsNPPT | 0.227 | 0.056 | -0.667 | -0.128 |
| sp\|Q9HBW9\|AGRL4_HUMAN Adhesion G protein-coupled receptor L4 OS=Homo sapiens GN=ADGRL4 PE=1 S | 346 | MSSNPPtLYEL | 0.268 | -0.029 | -0.31 | -0.024 |
| sp\|Q9HBW9\|AGRL4_HUMAN Adhesion G protein-coupled receptor L4 OS=Homo sapiens GN=ADGRL4 PE=1 S | 354 | YELEKItFTLS | 0.135 | -0.085 | -1.013 | -0.321 |
| sp\|Q9HBW9\|AGRL4_HUMAN Adhesion G protein-coupled receptor L4 OS=Homo sapiens GN=ADGRL4 PE=1 S | 356 | LEKITFtLSHR | 0.158 | 0.039 | -0.524 | -0.109 |
| sp\|Q9HBW9\|AGRL4_HUMAN Adhesion G protein-coupled receptor L4 OS=Homo sapiens GN=ADGRL4 PE=1 S | 358 | KITFTLsHRKV | 0.134 | -0.048 | -1.134 | -0.349 |
| sp\|Q9HBW9\|AGRL4_HUMAN Adhesion G protein-coupled receptor L4 OS=Homo sapiens GN=ADGRL4 PE=1 S | 363 | LSHRKVtDRYR | 0.547 | 0.639 | 0.022 | 0.403 |
| sp\|Q9HBW9\|AGRL4_HUMAN Adhesion G protein-coupled receptor L4 OS=Homo sapiens GN=ADGRL4 PE=1 S | 368 | VTDRYRsLCAF | 0.617 | 0.801 | 0.23 | 0.549 |
| sp\|Q9HBW9\|AGRL4_HUMAN Adhesion G protein-coupled receptor L4 OS=Homo sapiens GN=ADGRL4 PE=1 S | 376 | CAFWNYsPDTM | 0.201 | -0.267 | -0.523 | -0.196 |
| sp\|Q9HBW9\|AGRL4_HUMAN Adhesion G protein-coupled receptor L4 OS=Homo sapiens GN=ADGRL4 PE=1 S | 379 | WNYSPDtMNGS | 0.066 | -0.321 | -1.187 | -0.481 |
| sp\|Q9HBW9\|AGRL4_HUMAN Adhesion G protein-coupled receptor L4 OS=Homo sapiens GN=ADGRL4 PE=1 S | 383 | PDTMNGsWSSE | 0.042 | -0.292 | -1.433 | -0.561 |
| sp\|Q9HBW9\|AGRL4_HUMAN Adhesion G protein-coupled receptor L4 OS=Homo sapiens GN=ADGRL4 PE=1 S | 385 | TMNGSWsSEGC | 0.127 | 0.029 | -1.016 | -0.287 |
| sp\|Q9HBW9\|AGRL4_HUMAN Adhesion G protein-coupled receptor L4 OS=Homo sapiens GN=ADGRL4 PE=1 S | 386 | MNGSWSsEGCE | 0.156 | -0.009 | -0.851 | -0.235 |
| sp\|Q9HBW9\|AGRL4_HUMAN Adhesion G protein-coupled receptor L4 OS=Homo sapiens GN=ADGRL4 PE=1 S | 392 | SEGCELtYSNE | 0.032 | -0.349 | -2.202 | -0.84 |
| sp\|Q9HBW9\|AGRL4_HUMAN Adhesion G protein-coupled receptor L4 OS=Homo sapiens GN=ADGRL4 PE=1 S | 394 | GCELTYsNETH | 0.138 | -0.05 | -1.253 | -0.388 |
| sp\|Q9HBW9\|AGRL4_HUMAN Adhesion G protein-coupled receptor L4 OS=Homo sapiens GN=ADGRL4 PE=1 S | 397 | LTYSNEtHTSC | 0.085 | -0.309 | -1.488 | -0.571 |
| sp\|Q9HBW9\|AGRL4_HUMAN Adhesion G protein-coupled receptor L4 OS=Homo sapiens GN=ADGRL4 PE=1 S | 399 | YSNETHtSCRC | 0.053 | -0.257 | -1.824 | -0.676 |
| sp\|Q9HBW9\|AGRL4_HUMAN Adhesion G protein-coupled receptor L4 OS=Homo sapiens GN=ADGRL4 PE=1 S | 400 | SNETHTsCRCN | 0.172 | -0.079 | -1.055 | -0.321 |
| sp\|Q9HBW9\|AGRL4_HUMAN Adhesion G protein-coupled receptor L4 OS=Homo sapiens GN=ADGRL4 PE=1 S | 407 | CRCNHLtHFAI | 0.506 | 0.387 | 0.147 | 0.347 |
| sp\|Q9HBW9\|AGRL4_HUMAN Adhesion G protein-coupled receptor L4 OS=Homo sapiens GN=ADGRL4 PE=1 S | 414 | HFAILMsSGPS | 0.168 | 0.077 | -0.597 | -0.117 |

| | | | | | | |
|---|---|---|---|---|---|---|
| sp\|Q9HBW9\|AGRL4_HUMAN Adhesion G protein-coupled receptor L4 OS=Homo sapiens GN=ADGRL4 PE=1 S | 415 | FAILMSsGPSI | 0.318 | 0.349 | -0.355 | 0.104 |
| sp\|Q9HBW9\|AGRL4_HUMAN Adhesion G protein-coupled receptor L4 OS=Homo sapiens GN=ADGRL4 PE=1 S | 418 | LMSSGPsIGIK | 0.075 | -0.21 | -1.123 | -0.419 |
| sp\|Q9HBW9\|AGRL4_HUMAN Adhesion G protein-coupled receptor L4 OS=Homo sapiens GN=ADGRL4 PE=1 S | 428 | KDYNILtRITQ | 0.027 | -0.385 | -1.452 | -0.603 |
| sp\|Q9HBW9\|AGRL4_HUMAN Adhesion G protein-coupled receptor L4 OS=Homo sapiens GN=ADGRL4 PE=1 S | 431 | NILTRItQLGI | 0.374 | 0.103 | -0.471 | 0.002 |
| sp\|Q9HBW9\|AGRL4_HUMAN Adhesion G protein-coupled receptor L4 OS=Homo sapiens GN=ADGRL4 PE=1 S | 438 | QLGIIIsLICL | 0.303 | 0.218 | -0.097 | 0.141 |
| sp\|Q9HBW9\|AGRL4_HUMAN Adhesion G protein-coupled receptor L4 OS=Homo sapiens GN=ADGRL4 PE=1 S | 448 | LAICIFtFWFF | 0.212 | -0.003 | -0.629 | -0.14 |
| sp\|Q9HBW9\|AGRL4_HUMAN Adhesion G protein-coupled receptor L4 OS=Homo sapiens GN=ADGRL4 PE=1 S | 453 | FTFWFFsEIQS | 0.295 | 0.064 | -0.526 | -0.056 |
| sp\|Q9HBW9\|AGRL4_HUMAN Adhesion G protein-coupled receptor L4 OS=Homo sapiens GN=ADGRL4 PE=1 S | 457 | FFSEIQsTRTT | 0.125 | -0.124 | -0.729 | -0.243 |
| sp\|Q9HBW9\|AGRL4_HUMAN Adhesion G protein-coupled receptor L4 OS=Homo sapiens GN=ADGRL4 PE=1 S | 458 | FSEIQStRTTI | 0.128 | -0.172 | -1.126 | -0.39 |
| sp\|Q9HBW9\|AGRL4_HUMAN Adhesion G protein-coupled receptor L4 OS=Homo sapiens GN=ADGRL4 PE=1 S | 460 | EIQSTRtTIHK | 0.051 | -0.091 | -1.197 | -0.412 |
| sp\|Q9HBW9\|AGRL4_HUMAN Adhesion G protein-coupled receptor L4 OS=Homo sapiens GN=ADGRL4 PE=1 S | 461 | IQSTRTtIHKN | 0.256 | 0.011 | -0.564 | -0.099 |
| sp\|Q9HBW9\|AGRL4_HUMAN Adhesion G protein-coupled receptor L4 OS=Homo sapiens GN=ADGRL4 PE=1 S | 469 | HKNLCCsLFLA | 0.692 | 0.237 | 0.07 | 0.333 |
| sp\|Q9HBW9\|AGRL4_HUMAN Adhesion G protein-coupled receptor L4 OS=Homo sapiens GN=ADGRL4 PE=1 S | 483 | FLVGINtNTNK | 0.372 | 0.126 | -0.665 | -0.056 |
| sp\|Q9HBW9\|AGRL4_HUMAN Adhesion G protein-coupled receptor L4 OS=Homo sapiens GN=ADGRL4 PE=1 S | 485 | VGINTNtNKLF | 0.078 | -0.066 | -1.09 | -0.359 |
| sp\|Q9HBW9\|AGRL4_HUMAN Adhesion G protein-coupled receptor L4 OS=Homo sapiens GN=ADGRL4 PE=1 S | 491 | TNKLFCsIIAG | 0.194 | -0.089 | -0.745 | -0.213 |
| sp\|Q9HBW9\|AGRL4_HUMAN Adhesion G protein-coupled receptor L4 OS=Homo sapiens GN=ADGRL4 PE=1 S | 540 | YIFGYLsPAVV | 0.058 | -0.287 | -1.512 | -0.58 |
| sp\|Q9HBW9\|AGRL4_HUMAN Adhesion G protein-coupled receptor L4 OS=Homo sapiens GN=ADGRL4 PE=1 S | 548 | AVVVGFsAALG | 0.057 | -0.119 | -1.414 | -0.492 |
| sp\|Q9HBW9\|AGRL4_HUMAN Adhesion G protein-coupled receptor L4 OS=Homo sapiens GN=ADGRL4 PE=1 S | 558 | GYRYYGtTKVC | 0.35 | 0.185 | -0.632 | -0.032 |
| sp\|Q9HBW9\|AGRL4_HUMAN Adhesion G protein-coupled receptor L4 OS=Homo sapiens GN=ADGRL4 PE=1 S | 559 | YRYYGTtKVCW | 0.11 | 0.215 | -0.664 | -0.113 |
| sp\|Q9HBW9\|AGRL4_HUMAN Adhesion G protein-coupled receptor L4 OS=Homo sapiens GN=ADGRL4 PE=1 S | 565 | TKVCWLsTENN | 0.221 | 0.033 | -0.974 | -0.24 |
| sp\|Q9HBW9\|AGRL4_HUMAN Adhesion G protein-coupled receptor L4 OS=Homo sapiens GN=ADGRL4 PE=1 S | 566 | KVCWLStENNF | 0.119 | -0.075 | -1.076 | -0.344 |
| sp\|Q9HBW9\|AGRL4_HUMAN Adhesion G protein-coupled receptor L4 OS=Homo sapiens GN=ADGRL4 PE=1 S | 573 | ENNFIWsFIGP | 0.11 | -0.198 | -0.733 | -0.274 |
| sp\|Q9HBW9\|AGRL4_HUMAN Adhesion G protein-coupled receptor L4 OS=Homo sapiens GN=ADGRL4 PE=1 S | 600 | YKVFRHtAGLK | 0.722 | 0.419 | 0.102 | 0.414 |
| sp\|Q9HBW9\|AGRL4_HUMAN Adhesion G protein-coupled receptor L4 OS=Homo sapiens GN=ADGRL4 PE=1 S | 608 | GLKPEVsCFEN | 0.639 | 0.188 | -0.186 | 0.214 |
| sp\|Q9HBW9\|AGRL4_HUMAN Adhesion G protein-coupled receptor L4 OS=Homo sapiens GN=ADGRL4 PE=1 S | 615 | CFENIRsCARG | 0.178 | -0.058 | -0.706 | -0.195 |
| sp\|Q9HBW9\|AGRL4_HUMAN Adhesion G protein-coupled receptor L4 OS=Homo sapiens GN=ADGRL4 PE=1 S | 629 | LLFLLGtTWIF | 0.169 | -0.055 | -1.064 | -0.317 |
| sp\|Q9HBW9\|AGRL4_HUMAN Adhesion G protein-coupled receptor L4 OS=Homo sapiens GN=ADGRL4 PE=1 S | 630 | LFLLGTtWIFG | 0.078 | -0.076 | -0.88 | -0.293 |
| sp\|Q9HBW9\|AGRL4_HUMAN Adhesion G protein-coupled receptor L4 OS=Homo sapiens GN=ADGRL4 PE=1 S | 642 | LHVVHAsVVTA | 0.144 | -0.033 | -1.13 | -0.34 |
| sp\|Q9HBW9\|AGRL4_HUMAN Adhesion G protein-coupled receptor L4 OS=Homo sapiens GN=ADGRL4 PE=1 S | 645 | VHASVVtAYLF | 0.176 | -0.102 | -1.139 | -0.355 |
| sp\|Q9HBW9\|AGRL4_HUMAN Adhesion G protein-coupled receptor L4 OS=Homo sapiens GN=ADGRL4 PE=1 S | 650 | VTAYLFtVSNA | 0.121 | -0.096 | -0.983 | -0.319 |
| sp\|Q9HBW9\|AGRL4_HUMAN Adhesion G protein-coupled receptor L4 OS=Homo sapiens GN=ADGRL4 PE=1 S | 652 | AYLFTVsNAFQ | 0.148 | -0.184 | -1.24 | -0.425 |
| sp\|Q9HBW9\|AGRL4_HUMAN Adhesion G protein-coupled receptor L4 OS=Homo sapiens GN=ADGRL4 PE=1 S | 668 | LFLCVLsRKIQ | 0.136 | -0.238 | -1.098 | -0.4 |
| sp\|Q14246\|AGRE1_HUMAN Adhesion G protein-coupled receptor E1 OS=Homo sapiens GN=ADGRE1 PE=2 SV | 340 | QQCQEGtAVKP | 0.063 | -0.236 | -1.487 | -0.553 |
| sp\|Q14246\|AGRE1_HUMAN Adhesion G protein-coupled receptor E1 OS=Homo sapiens GN=ADGRE1 PE=2 SV | 348 | VKPAYVsFCAQ | 0.431 | -0.06 | -0.389 | -0.006 |
| sp\|Q14246\|AGRE1_HUMAN Adhesion G protein-coupled receptor E1 OS=Homo sapiens GN=ADGRE1 PE=2 SV | 358 | QINNIFsVLDK | 0.172 | 0.043 | -0.542 | -0.109 |
| sp\|Q14246\|AGRE1_HUMAN Adhesion G protein-coupled receptor E1 OS=Homo sapiens GN=ADGRE1 PE=2 SV | 368 | KVCENKtTVVS | 0.129 | -0.036 | -0.972 | -0.293 |
| sp\|Q14246\|AGRE1_HUMAN Adhesion G protein-coupled receptor E1 OS=Homo sapiens GN=ADGRE1 PE=2 SV | 369 | VCENKTtVVSL | 0.088 | -0.09 | -1.018 | -0.34 |
| sp\|Q14246\|AGRE1_HUMAN Adhesion G protein-coupled receptor E1 OS=Homo sapiens GN=ADGRE1 PE=2 SV | 372 | NKTTVVsLKNT | 0.589 | 0.104 | -0.103 | 0.197 |
| sp\|Q14246\|AGRE1_HUMAN Adhesion G protein-coupled receptor E1 OS=Homo sapiens GN=ADGRE1 PE=2 SV | 376 | VVSLKNtTESF | 0.064 | -0.204 | -1.507 | -0.549 |
| sp\|Q14246\|AGRE1_HUMAN Adhesion G protein-coupled receptor E1 OS=Homo sapiens GN=ADGRE1 PE=2 SV | 377 | VSLKNTtESFV | 0.186 | -0.139 | -0.925 | -0.293 |
| sp\|Q14246\|AGRE1_HUMAN Adhesion G protein-coupled receptor E1 OS=Homo sapiens GN=ADGRE1 PE=2 SV | 379 | LKNTTEsFVPV | 0.19 | -0.004 | -1.04 | -0.285 |
| sp\|Q14246\|AGRE1_HUMAN Adhesion G protein-coupled receptor E1 OS=Homo sapiens GN=ADGRE1 PE=2 SV | 388 | PVLKQIsTWTK | 0.325 | -0.012 | -0.622 | -0.103 |
| sp\|Q14246\|AGRE1_HUMAN Adhesion G protein-coupled receptor E1 OS=Homo sapiens GN=ADGRE1 PE=2 SV | 389 | VLKQIStWTKF | 0.205 | 0.017 | -0.49 | -0.089 |
| sp\|Q14246\|AGRE1_HUMAN Adhesion G protein-coupled receptor E1 OS=Homo sapiens GN=ADGRE1 PE=2 SV | 391 | KQISTWtKFTK | 0.097 | -0.202 | -0.996 | -0.367 |
| sp\|Q14246\|AGRE1_HUMAN Adhesion G protein-coupled receptor E1 OS=Homo sapiens GN=ADGRE1 PE=2 SV | 394 | STWTKFtKEET | 0.08 | -0.085 | -1.253 | -0.419 |
| sp\|Q14246\|AGRE1_HUMAN Adhesion G protein-coupled receptor E1 OS=Homo sapiens GN=ADGRE1 PE=2 SV | 398 | KFTKEEtSSLA | 0.169 | -0.179 | -1.068 | -0.359 |
| sp\|Q14246\|AGRE1_HUMAN Adhesion G protein-coupled receptor E1 OS=Homo sapiens GN=ADGRE1 PE=2 SV | 399 | FTKEETsSLAT | 0.123 | -0.054 | -1.055 | -0.329 |
| sp\|Q14246\|AGRE1_HUMAN Adhesion G protein-coupled receptor E1 OS=Homo sapiens GN=ADGRE1 PE=2 SV | 400 | TKEETSsLATV | 0.152 | -0.052 | -0.946 | -0.282 |
| sp\|Q14246\|AGRE1_HUMAN Adhesion G protein-coupled receptor E1 OS=Homo sapiens GN=ADGRE1 PE=2 SV | 403 | ETSSLAtVFLE | 0.129 | -0.17 | -1.128 | -0.39 |

| | | | | | | |
|---|---|---|---|---|---|---|
| sp\|Q14246\|AGRE1_HUMAN Adhesion G protein-coupled receptor E1 OS=Homo sapiens GN=ADGRE1 PE=2 SV | 408 | ATVFLEsVESM | 0.076 | -0.225 | -1.569 | -0.573 |
| sp\|Q14246\|AGRE1_HUMAN Adhesion G protein-coupled receptor E1 OS=Homo sapiens GN=ADGRE1 PE=2 SV | 411 | FLESVEsMTLA | 0.342 | 0.097 | -0.752 | -0.104 |
| sp\|Q14246\|AGRE1_HUMAN Adhesion G protein-coupled receptor E1 OS=Homo sapiens GN=ADGRE1 PE=2 SV | 413 | ESVESMtLASF | 0.169 | 0.187 | -0.469 | -0.038 |
| sp\|Q14246\|AGRE1_HUMAN Adhesion G protein-coupled receptor E1 OS=Homo sapiens GN=ADGRE1 PE=2 SV | 416 | ESMTLAsFWKP | 0.139 | -0.074 | -1.064 | -0.333 |
| sp\|Q14246\|AGRE1_HUMAN Adhesion G protein-coupled receptor E1 OS=Homo sapiens GN=ADGRE1 PE=2 SV | 421 | ASFWKPsANIT | 0.171 | -0.244 | -0.984 | -0.352 |
| sp\|Q14246\|AGRE1_HUMAN Adhesion G protein-coupled receptor E1 OS=Homo sapiens GN=ADGRE1 PE=2 SV | 425 | KPSANItPAVR | 0.073 | -0.491 | -1.356 | -0.591 |
| sp\|Q14246\|AGRE1_HUMAN Adhesion G protein-coupled receptor E1 OS=Homo sapiens GN=ADGRE1 PE=2 SV | 430 | ITPAVRtEYLD | 0.206 | -0.051 | -0.93 | -0.258 |
| sp\|Q14246\|AGRE1_HUMAN Adhesion G protein-coupled receptor E1 OS=Homo sapiens GN=ADGRE1 PE=2 SV | 437 | EYLDIEsKVIN | 0.196 | -0.036 | -0.84 | -0.227 |
| sp\|Q14246\|AGRE1_HUMAN Adhesion G protein-coupled receptor E1 OS=Homo sapiens GN=ADGRE1 PE=2 SV | 445 | VINKECsEENV | 0.405 | 0.133 | -1.075 | -0.179 |
| sp\|Q14246\|AGRE1_HUMAN Adhesion G protein-coupled receptor E1 OS=Homo sapiens GN=ADGRE1 PE=2 SV | 450 | CSEENVtLDLV | 0.527 | 0.066 | -0.157 | 0.145 |
| sp\|Q14246\|AGRE1_HUMAN Adhesion G protein-coupled receptor E1 OS=Homo sapiens GN=ADGRE1 PE=2 SV | 465 | KMKIGCsTIEE | 0.124 | -0.004 | -1.077 | -0.319 |
| sp\|Q14246\|AGRE1_HUMAN Adhesion G protein-coupled receptor E1 OS=Homo sapiens GN=ADGRE1 PE=2 SV | 466 | MKIGCStIEES | 0.262 | 0.048 | -0.616 | -0.102 |
| sp\|Q14246\|AGRE1_HUMAN Adhesion G protein-coupled receptor E1 OS=Homo sapiens GN=ADGRE1 PE=2 SV | 470 | CSTIEEsESTE | 0.122 | -0.182 | -1.19 | -0.417 |
| sp\|Q14246\|AGRE1_HUMAN Adhesion G protein-coupled receptor E1 OS=Homo sapiens GN=ADGRE1 PE=2 SV | 472 | TIEESEsTETT | 0.157 | 0.036 | -0.851 | -0.219 |
| sp\|Q14246\|AGRE1_HUMAN Adhesion G protein-coupled receptor E1 OS=Homo sapiens GN=ADGRE1 PE=2 SV | 473 | IEESEStETTG | 0.172 | -0.212 | -1.126 | -0.389 |
| sp\|Q14246\|AGRE1_HUMAN Adhesion G protein-coupled receptor E1 OS=Homo sapiens GN=ADGRE1 PE=2 SV | 475 | ESESTEtTGVA | 0.106 | -0.172 | -1.106 | -0.391 |
| sp\|Q14246\|AGRE1_HUMAN Adhesion G protein-coupled receptor E1 OS=Homo sapiens GN=ADGRE1 PE=2 SV | 476 | SESTETtGVAF | 0.073 | -0.222 | -1.411 | -0.52 |
| sp\|Q14246\|AGRE1_HUMAN Adhesion G protein-coupled receptor E1 OS=Homo sapiens GN=ADGRE1 PE=2 SV | 482 | TGVAFVsFVGM | 0.276 | -0.117 | -0.529 | -0.123 |
| sp\|Q14246\|AGRE1_HUMAN Adhesion G protein-coupled receptor E1 OS=Homo sapiens GN=ADGRE1 PE=2 SV | 488 | SFVGMEsVLNE | 0.077 | -0.193 | -1.452 | -0.523 |
| sp\|Q14246\|AGRE1_HUMAN Adhesion G protein-coupled receptor E1 OS=Homo sapiens GN=ADGRE1 PE=2 SV | 503 | DHQAPLtTSEI | 0.122 | -0.17 | -1.273 | -0.44 |
| sp\|Q14246\|AGRE1_HUMAN Adhesion G protein-coupled receptor E1 OS=Homo sapiens GN=ADGRE1 PE=2 SV | 504 | HQAPLTtSEIK | 0.077 | -0.199 | -0.976 | -0.366 |
| sp\|Q14246\|AGRE1_HUMAN Adhesion G protein-coupled receptor E1 OS=Homo sapiens GN=ADGRE1 PE=2 SV | 505 | QAPLTTsEIKL | 0.063 | -0.045 | -1.123 | -0.368 |
| sp\|Q14246\|AGRE1_HUMAN Adhesion G protein-coupled receptor E1 OS=Homo sapiens GN=ADGRE1 PE=2 SV | 513 | IKLKMNsRVVG | 0.591 | 0.037 | -0.233 | 0.132 |
| sp\|Q14246\|AGRE1_HUMAN Adhesion G protein-coupled receptor E1 OS=Homo sapiens GN=ADGRE1 PE=2 SV | 521 | VVGGIMtGEKK | 0.078 | -0.101 | -0.956 | -0.326 |
| sp\|Q14246\|AGRE1_HUMAN Adhesion G protein-coupled receptor E1 OS=Homo sapiens GN=ADGRE1 PE=2 SV | 529 | EKKDGFsDPII | 0.791 | 0.642 | 0.525 | 0.653 |
| sp\|Q14246\|AGRE1_HUMAN Adhesion G protein-coupled receptor E1 OS=Homo sapiens GN=ADGRE1 PE=2 SV | 535 | SDPIIYtLENI | 0.134 | 0.031 | -0.723 | -0.186 |
| sp\|Q14246\|AGRE1_HUMAN Adhesion G protein-coupled receptor E1 OS=Homo sapiens GN=ADGRE1 PE=2 SV | 552 | ERPICVsWSTD | 0.251 | 0.185 | -0.598 | -0.054 |
| sp\|Q14246\|AGRE1_HUMAN Adhesion G protein-coupled receptor E1 OS=Homo sapiens GN=ADGRE1 PE=2 SV | 554 | PICVSWsTDVK | 0.437 | 0.233 | -0.268 | 0.134 |
| sp\|Q14246\|AGRE1_HUMAN Adhesion G protein-coupled receptor E1 OS=Homo sapiens GN=ADGRE1 PE=2 SV | 555 | ICVSWStDVKG | 0.181 | -0.068 | -1.105 | -0.331 |
| sp\|Q14246\|AGRE1_HUMAN Adhesion G protein-coupled receptor E1 OS=Homo sapiens GN=ADGRE1 PE=2 SV | 563 | VKGGRWtSFGC | 0.261 | -0.053 | -0.68 | -0.157 |
| sp\|Q14246\|AGRE1_HUMAN Adhesion G protein-coupled receptor E1 OS=Homo sapiens GN=ADGRE1 PE=2 SV | 564 | KGGRWTsFGCV | 0.728 | 0.987 | 0.487 | 0.734 |
| sp\|Q14246\|AGRE1_HUMAN Adhesion G protein-coupled receptor E1 OS=Homo sapiens GN=ADGRE1 PE=2 SV | 573 | CVILEAsETYT | 0.235 | -0.001 | -0.936 | -0.234 |
| sp\|Q14246\|AGRE1_HUMAN Adhesion G protein-coupled receptor E1 OS=Homo sapiens GN=ADGRE1 PE=2 SV | 575 | ILEASEtYTIC | 0.482 | 0.039 | -0.462 | 0.02 |
| sp\|Q14246\|AGRE1_HUMAN Adhesion G protein-coupled receptor E1 OS=Homo sapiens GN=ADGRE1 PE=2 SV | 577 | EASETYtICSC | 0.019 | -0.308 | -1.808 | -0.699 |
| sp\|Q14246\|AGRE1_HUMAN Adhesion G protein-coupled receptor E1 OS=Homo sapiens GN=ADGRE1 PE=2 SV | 580 | ETYTICsCNQM | 0.257 | -0.011 | -0.625 | -0.126 |
| sp\|Q14246\|AGRE1_HUMAN Adhesion G protein-coupled receptor E1 OS=Homo sapiens GN=ADGRE1 PE=2 SV | 593 | LAVIMAsGELT | 0.096 | -0.202 | -1.097 | -0.401 |
| sp\|Q14246\|AGRE1_HUMAN Adhesion G protein-coupled receptor E1 OS=Homo sapiens GN=ADGRE1 PE=2 SV | 597 | MASGELtMDFS | 0.079 | -0.077 | -1.337 | -0.445 |
| sp\|Q14246\|AGRE1_HUMAN Adhesion G protein-coupled receptor E1 OS=Homo sapiens GN=ADGRE1 PE=2 SV | 601 | ELTMDFsLYII | 0.552 | 0.18 | 0.193 | 0.308 |
| sp\|Q14246\|AGRE1_HUMAN Adhesion G protein-coupled receptor E1 OS=Homo sapiens GN=ADGRE1 PE=2 SV | 606 | FSLYIIsHVGI | 0.372 | 0.039 | -0.066 | 0.115 |
| sp\|Q14246\|AGRE1_HUMAN Adhesion G protein-coupled receptor E1 OS=Homo sapiens GN=ADGRE1 PE=2 SV | 613 | HVGIIIsLVCL | 0.201 | 0.204 | -0.334 | 0.024 |
| sp\|Q14246\|AGRE1_HUMAN Adhesion G protein-coupled receptor E1 OS=Homo sapiens GN=ADGRE1 PE=2 SV | 623 | LVLAIAtFLLC | 0.153 | -0.051 | -0.772 | -0.223 |
| sp\|Q14246\|AGRE1_HUMAN Adhesion G protein-coupled receptor E1 OS=Homo sapiens GN=ADGRE1 PE=2 SV | 629 | TFLLCRsIRNH | 0.127 | -0.212 | -1.241 | -0.442 |
| sp\|Q14246\|AGRE1_HUMAN Adhesion G protein-coupled receptor E1 OS=Homo sapiens GN=ADGRE1 PE=2 SV | 635 | SIRNHNtYLHL | 0.229 | 0.232 | -0.624 | -0.054 |
| sp\|Q14246\|AGRE1_HUMAN Adhesion G protein-coupled receptor E1 OS=Homo sapiens GN=ADGRE1 PE=2 SV | 650 | CLLLAKtLFLA | 0.758 | 0.341 | 0.664 | 0.588 |
| sp\|Q14246\|AGRE1_HUMAN Adhesion G protein-coupled receptor E1 OS=Homo sapiens GN=ADGRE1 PE=2 SV | 659 | LAGIHKtDNKM | 0.229 | 0.038 | -0.585 | -0.106 |
| sp\|Q14246\|AGRE1_HUMAN Adhesion G protein-coupled receptor E1 OS=Homo sapiens GN=ADGRE1 PE=2 SV | 704 | KVVNYFsSRNI | 0.169 | -0.017 | -0.759 | -0.202 |
| sp\|Q14246\|AGRE1_HUMAN Adhesion G protein-coupled receptor E1 OS=Homo sapiens GN=ADGRE1 PE=2 SV | 705 | VVNYFSsRNIK | 0.104 | -0.25 | -1.211 | -0.452 |
| sp\|Q14246\|AGRE1_HUMAN Adhesion G protein-coupled receptor E1 OS=Homo sapiens GN=ADGRE1 PE=2 SV | 728 | MLVVVIsASVQ | 0.205 | 0.065 | -1.015 | -0.248 |
| sp\|Q14246\|AGRE1_HUMAN Adhesion G protein-coupled receptor E1 OS=Homo sapiens GN=ADGRE1 PE=2 SV | 730 | VVVISAsVQPQ | 0.244 | 0.123 | -0.544 | -0.059 |

| | | | | | | |
|---|---|---|---|---|---|---|
| sp\|Q14246\|AGRE1_HUMAN Adhesion G protein-coupled receptor E1 OS=Homo sapiens GN=ADGRE1 PE=2 SV | 746 | NRCWLNtETGF | 0.398 | 0.176 | -0.496 | 0.026 |
| sp\|Q14246\|AGRE1_HUMAN Adhesion G protein-coupled receptor E1 OS=Homo sapiens GN=ADGRE1 PE=2 SV | 748 | CWLNTEtGFIW | 0.122 | -0.022 | -0.783 | -0.228 |
| sp\|Q14246\|AGRE1_HUMAN Adhesion G protein-coupled receptor E1 OS=Homo sapiens GN=ADGRE1 PE=2 SV | 753 | ETGFIWsFLGP | 0.115 | -0.17 | -0.927 | -0.327 |
| sp\|Q14246\|AGRE1_HUMAN Adhesion G protein-coupled receptor E1 OS=Homo sapiens GN=ADGRE1 PE=2 SV | 760 | FLGPVCtVIVI | 0.373 | 0.18 | -0.392 | 0.054 |
| sp\|Q14246\|AGRE1_HUMAN Adhesion G protein-coupled receptor E1 OS=Homo sapiens GN=ADGRE1 PE=2 SV | 766 | TVIVINsLLLT | 0.135 | 0.019 | -0.787 | -0.211 |
| sp\|Q14246\|AGRE1_HUMAN Adhesion G protein-coupled receptor E1 OS=Homo sapiens GN=ADGRE1 PE=2 SV | 770 | INSLLLtWTLW | 0.077 | -0.155 | -1.266 | -0.448 |
| sp\|Q14246\|AGRE1_HUMAN Adhesion G protein-coupled receptor E1 OS=Homo sapiens GN=ADGRE1 PE=2 SV | 772 | SLLLTWtLWIL | 0.464 | 0.18 | -0.33 | 0.105 |
| sp\|Q14246\|AGRE1_HUMAN Adhesion G protein-coupled receptor E1 OS=Homo sapiens GN=ADGRE1 PE=2 SV | 781 | ILRQRLsSVNA | 0.625 | 0.398 | 0.027 | 0.35 |
| sp\|Q14246\|AGRE1_HUMAN Adhesion G protein-coupled receptor E1 OS=Homo sapiens GN=ADGRE1 PE=2 SV | 782 | LRQRLSsVNAE | 0.603 | 1.079 | 0.509 | 0.73 |
| sp\|Q14246\|AGRE1_HUMAN Adhesion G protein-coupled receptor E1 OS=Homo sapiens GN=ADGRE1 PE=2 SV | 788 | SVNAEVsTLKD | 0.102 | -0.224 | -1.438 | -0.52 |
| sp\|Q14246\|AGRE1_HUMAN Adhesion G protein-coupled receptor E1 OS=Homo sapiens GN=ADGRE1 PE=2 SV | 789 | VNAEVStLKDT | 0.127 | -0.173 | -0.754 | -0.267 |
| sp\|Q14246\|AGRE1_HUMAN Adhesion G protein-coupled receptor E1 OS=Homo sapiens GN=ADGRE1 PE=2 SV | 793 | VSTLKDtRLLT | 0.063 | -0.415 | -1.709 | -0.687 |
| sp\|Q14246\|AGRE1_HUMAN Adhesion G protein-coupled receptor E1 OS=Homo sapiens GN=ADGRE1 PE=2 SV | 797 | KDTRLLtFKAF | 0.324 | 0.733 | 0.072 | 0.376 |
| sp\|Q14246\|AGRE1_HUMAN Adhesion G protein-coupled receptor E1 OS=Homo sapiens GN=ADGRE1 PE=2 SV | 810 | LFILGCsWVLG | 0.153 | 0.059 | -0.957 | -0.248 |
| sp\|Q14246\|AGRE1_HUMAN Adhesion G protein-coupled receptor E1 OS=Homo sapiens GN=ADGRE1 PE=2 SV | 830 | VMAYLFtIINS | 0.064 | 0.03 | -1.001 | -0.302 |
| sp\|Q14246\|AGRE1_HUMAN Adhesion G protein-coupled receptor E1 OS=Homo sapiens GN=ADGRE1 PE=2 SV | 834 | LFTIINsLQGA | 0.474 | 0.102 | 0.185 | 0.254 |
| sp\|Q14246\|AGRE1_HUMAN Adhesion G protein-coupled receptor E1 OS=Homo sapiens GN=ADGRE1 PE=2 SV | 860 | EYKRWItGKTK | 0.666 | 0.804 | 0.586 | 0.685 |
| sp\|Q14246\|AGRE1_HUMAN Adhesion G protein-coupled receptor E1 OS=Homo sapiens GN=ADGRE1 PE=2 SV | 863 | RWITGKtKPSS | 0.374 | 0.458 | -0.226 | 0.202 |
| sp\|Q14246\|AGRE1_HUMAN Adhesion G protein-coupled receptor E1 OS=Homo sapiens GN=ADGRE1 PE=2 SV | 866 | TGKTKPsSQSQ | 0.069 | -0.312 | -1.355 | -0.533 |
| sp\|Q14246\|AGRE1_HUMAN Adhesion G protein-coupled receptor E1 OS=Homo sapiens GN=ADGRE1 PE=2 SV | 867 | GKTKPSsQSQT | 0.215 | -0.195 | -0.825 | -0.268 |
| sp\|Q14246\|AGRE1_HUMAN Adhesion G protein-coupled receptor E1 OS=Homo sapiens GN=ADGRE1 PE=2 SV | 869 | TKPSSQsQTSR | 0.232 | 0.018 | -0.755 | -0.168 |
| sp\|Q14246\|AGRE1_HUMAN Adhesion G protein-coupled receptor E1 OS=Homo sapiens GN=ADGRE1 PE=2 SV | 871 | PSSQSQtSRIL | 0.415 | 0.074 | -0.163 | 0.109 |
| sp\|Q14246\|AGRE1_HUMAN Adhesion G protein-coupled receptor E1 OS=Homo sapiens GN=ADGRE1 PE=2 SV | 872 | SSQSQTsRILL | 0.064 | -0.204 | -1.411 | -0.517 |
| sp\|Q14246\|AGRE1_HUMAN Adhesion G protein-coupled receptor E1 OS=Homo sapiens GN=ADGRE1 PE=2 SV | 877 | TSRILLsSMPS | 0.081 | -0.08 | -1.308 | -0.436 |
| sp\|Q14246\|AGRE1_HUMAN Adhesion G protein-coupled receptor E1 OS=Homo sapiens GN=ADGRE1 PE=2 SV | 878 | SRILLSsMPSA | 0.18 | 0.566 | -0.827 | -0.027 |
| sp\|Q14246\|AGRE1_HUMAN Adhesion G protein-coupled receptor E1 OS=Homo sapiens GN=ADGRE1 PE=2 SV | 881 | LLSSMPsASKT | 0.091 | -0.26 | -1.203 | -0.457 |
| sp\|Q14246\|AGRE1_HUMAN Adhesion G protein-coupled receptor E1 OS=Homo sapiens GN=ADGRE1 PE=2 SV | 883 | SSMPSAsKTG- | 0.292 | 0.046 | -0.54 | -0.067 |
| sp\|Q14246\|AGRE1_HUMAN Adhesion G protein-coupled receptor E1 OS=Homo sapiens GN=ADGRE1 PE=2 SV | 885 | MPSASKtG--- | 0.47 | 0.327 | -0.054 | 0.248 |
| sp\|Q9UHX3\|AGRE2_HUMAN Adhesion G protein-coupled receptor E2 OS=Homo sapiens GN=ADGRE2 PE=1 S | 320 | APGDLEtLPRL | 0.46 | 0.338 | -0.553 | 0.082 |
| sp\|Q9UHX3\|AGRE2_HUMAN Adhesion G protein-coupled receptor E2 OS=Homo sapiens GN=ADGRE2 PE=1 S | 331 | QQHCVAsHLLD | 0.195 | -0.067 | -0.933 | -0.268 |
| sp\|Q9UHX3\|AGRE2_HUMAN Adhesion G protein-coupled receptor E2 OS=Homo sapiens GN=ADGRE2 PE=1 S | 345 | DVLRGLsKNLS | 0.364 | 0.733 | -0.019 | 0.359 |
| sp\|Q9UHX3\|AGRE2_HUMAN Adhesion G protein-coupled receptor E2 OS=Homo sapiens GN=ADGRE2 PE=1 S | 349 | GLSKNLsNGLL | 0.497 | 0.152 | -0.282 | 0.122 |
| sp\|Q9UHX3\|AGRE2_HUMAN Adhesion G protein-coupled receptor E2 OS=Homo sapiens GN=ADGRE2 PE=1 S | 356 | NGLLNFsYPAG | 0.628 | 0.517 | 0.375 | 0.507 |
| sp\|Q9UHX3\|AGRE2_HUMAN Adhesion G protein-coupled receptor E2 OS=Homo sapiens GN=ADGRE2 PE=1 S | 361 | FSYPAGtELSL | 0.08 | -0.04 | -1.12 | -0.36 |
| sp\|Q9UHX3\|AGRE2_HUMAN Adhesion G protein-coupled receptor E2 OS=Homo sapiens GN=ADGRE2 PE=1 S | 364 | PAGTELsLEVQ | 0.115 | -0.126 | -1.201 | -0.404 |
| sp\|Q9UHX3\|AGRE2_HUMAN Adhesion G protein-coupled receptor E2 OS=Homo sapiens GN=ADGRE2 PE=1 S | 374 | QKQVDRsVTLR | 0.214 | 0.025 | -0.83 | -0.197 |
| sp\|Q9UHX3\|AGRE2_HUMAN Adhesion G protein-coupled receptor E2 OS=Homo sapiens GN=ADGRE2 PE=1 S | 376 | QVDRSVtLRQN | 0.894 | 1.063 | 1.218 | 1.058 |
| sp\|Q9UHX3\|AGRE2_HUMAN Adhesion G protein-coupled receptor E2 OS=Homo sapiens GN=ADGRE2 PE=1 S | 394 | WNQAQKsGDPG | 0.158 | -0.145 | -0.624 | -0.204 |
| sp\|Q9UHX3\|AGRE2_HUMAN Adhesion G protein-coupled receptor E2 OS=Homo sapiens GN=ADGRE2 PE=1 S | 400 | SGDPGPsVVGL | 0.051 | -0.323 | -1.357 | -0.543 |
| sp\|Q9UHX3\|AGRE2_HUMAN Adhesion G protein-coupled receptor E2 OS=Homo sapiens GN=ADGRE2 PE=1 S | 406 | SVVGLVsIPGM | 0.294 | 0.26 | -0.352 | 0.067 |
| sp\|Q9UHX3\|AGRE2_HUMAN Adhesion G protein-coupled receptor E2 OS=Homo sapiens GN=ADGRE2 PE=1 S | 432 | QMLLHEtHQGL | 0.215 | 0.072 | -0.761 | -0.158 |
| sp\|Q9UHX3\|AGRE2_HUMAN Adhesion G protein-coupled receptor E2 OS=Homo sapiens GN=ADGRE2 PE=1 S | 441 | GLLQDGsPILL | 0.124 | -0.247 | -0.903 | -0.342 |
| sp\|Q9UHX3\|AGRE2_HUMAN Adhesion G protein-coupled receptor E2 OS=Homo sapiens GN=ADGRE2 PE=1 S | 446 | GSPILLsDVIS | 0.075 | -0.192 | -1.599 | -0.572 |
| sp\|Q9UHX3\|AGRE2_HUMAN Adhesion G protein-coupled receptor E2 OS=Homo sapiens GN=ADGRE2 PE=1 S | 450 | LLSDVIsAFLS | 0.3 | 0.064 | -0.711 | -0.116 |
| sp\|Q9UHX3\|AGRE2_HUMAN Adhesion G protein-coupled receptor E2 OS=Homo sapiens GN=ADGRE2 PE=1 S | 454 | VISAFLsNNDT | 0.155 | -0.127 | -0.959 | -0.31 |
| sp\|Q9UHX3\|AGRE2_HUMAN Adhesion G protein-coupled receptor E2 OS=Homo sapiens GN=ADGRE2 PE=1 S | 458 | FLSNNDtQNLS | 0.238 | -0.013 | -0.741 | -0.172 |
| sp\|Q9UHX3\|AGRE2_HUMAN Adhesion G protein-coupled receptor E2 OS=Homo sapiens GN=ADGRE2 PE=1 S | 462 | NDTQNLsSPVT | 0.294 | 0.278 | -0.227 | 0.115 |
| sp\|Q9UHX3\|AGRE2_HUMAN Adhesion G protein-coupled receptor E2 OS=Homo sapiens GN=ADGRE2 PE=1 S | 463 | DTQNLSsPVTF | 0.035 | -0.483 | -1.77 | -0.739 |
| sp\|Q9UHX3\|AGRE2_HUMAN Adhesion G protein-coupled receptor E2 OS=Homo sapiens GN=ADGRE2 PE=1 S | 466 | NLSSPVtFTFS | 0.294 | -0.139 | -0.616 | -0.154 |

| | | | | | | |
|---|---|---|---|---|---|---|
| sp\|Q9UHX3\|AGRE2_HUMAN Adhesion G protein-coupled receptor E2 OS=Homo sapiens GN=ADGRE2 PE=1 S | 468 | SSPVTFtFSHR | 0.041 | -0.213 | -1.387 | -0.52 |
| sp\|Q9UHX3\|AGRE2_HUMAN Adhesion G protein-coupled receptor E2 OS=Homo sapiens GN=ADGRE2 PE=1 S | 470 | PVTFTFsHRSV | 0.144 | 0.006 | -0.907 | -0.252 |
| sp\|Q9UHX3\|AGRE2_HUMAN Adhesion G protein-coupled receptor E2 OS=Homo sapiens GN=ADGRE2 PE=1 S | 473 | FTFSHRsVIPR | 0.167 | -0.016 | -0.944 | -0.264 |
| sp\|Q9UHX3\|AGRE2_HUMAN Adhesion G protein-coupled receptor E2 OS=Homo sapiens GN=ADGRE2 PE=1 S | 497 | GCGHWAtTGCS | 0.342 | 0.14 | -0.711 | -0.076 |
| sp\|Q9UHX3\|AGRE2_HUMAN Adhesion G protein-coupled receptor E2 OS=Homo sapiens GN=ADGRE2 PE=1 S | 498 | CGHWATtGCST | 0.103 | -0.254 | -0.92 | -0.357 |
| sp\|Q9UHX3\|AGRE2_HUMAN Adhesion G protein-coupled receptor E2 OS=Homo sapiens GN=ADGRE2 PE=1 S | 501 | WATTGCsTIGT | 0.091 | -0.297 | -1.108 | -0.438 |
| sp\|Q9UHX3\|AGRE2_HUMAN Adhesion G protein-coupled receptor E2 OS=Homo sapiens GN=ADGRE2 PE=1 S | 502 | ATTGCStIGTR | 0.215 | -0.144 | -0.703 | -0.211 |
| sp\|Q9UHX3\|AGRE2_HUMAN Adhesion G protein-coupled receptor E2 OS=Homo sapiens GN=ADGRE2 PE=1 S | 505 | GCSTIGtRDTS | 0.167 | -0.178 | -1.155 | -0.389 |
| sp\|Q9UHX3\|AGRE2_HUMAN Adhesion G protein-coupled receptor E2 OS=Homo sapiens GN=ADGRE2 PE=1 S | 508 | TIGTRDtSTIC | 0.209 | -0.098 | -1.28 | -0.39 |
| sp\|Q9UHX3\|AGRE2_HUMAN Adhesion G protein-coupled receptor E2 OS=Homo sapiens GN=ADGRE2 PE=1 S | 509 | IGTRDTsTICR | 0.578 | 0.826 | 0.535 | 0.646 |
| sp\|Q9UHX3\|AGRE2_HUMAN Adhesion G protein-coupled receptor E2 OS=Homo sapiens GN=ADGRE2 PE=1 S | 510 | GTRDTStICRC | 0.133 | -0.088 | -1.418 | -0.458 |
| sp\|Q9UHX3\|AGRE2_HUMAN Adhesion G protein-coupled receptor E2 OS=Homo sapiens GN=ADGRE2 PE=1 S | 515 | STICRCtHLSS | 0.187 | -0.024 | -1.374 | -0.404 |
| sp\|Q9UHX3\|AGRE2_HUMAN Adhesion G protein-coupled receptor E2 OS=Homo sapiens GN=ADGRE2 PE=1 S | 518 | CRCTHLsSFAV | 0.338 | 0.264 | -0.631 | -0.01 |
| sp\|Q9UHX3\|AGRE2_HUMAN Adhesion G protein-coupled receptor E2 OS=Homo sapiens GN=ADGRE2 PE=1 S | 519 | RCTHLSsFAVL | 0.174 | 0.011 | -0.898 | -0.238 |
| sp\|Q9UHX3\|AGRE2_HUMAN Adhesion G protein-coupled receptor E2 OS=Homo sapiens GN=ADGRE2 PE=1 S | 537 | EEDPVLtVITY | 0.073 | -0.19 | -1.274 | -0.464 |
| sp\|Q9UHX3\|AGRE2_HUMAN Adhesion G protein-coupled receptor E2 OS=Homo sapiens GN=ADGRE2 PE=1 S | 540 | PVLTVItYMGL | 0.192 | -0.087 | -0.767 | -0.221 |
| sp\|Q9UHX3\|AGRE2_HUMAN Adhesion G protein-coupled receptor E2 OS=Homo sapiens GN=ADGRE2 PE=1 S | 545 | ITYMGLsVSLL | 0.064 | -0.216 | -1.209 | -0.454 |
| sp\|Q9UHX3\|AGRE2_HUMAN Adhesion G protein-coupled receptor E2 OS=Homo sapiens GN=ADGRE2 PE=1 S | 547 | YMGLSVsLLCL | 0.416 | 0.441 | -0.091 | 0.255 |
| sp\|Q9UHX3\|AGRE2_HUMAN Adhesion G protein-coupled receptor E2 OS=Homo sapiens GN=ADGRE2 PE=1 S | 557 | LLLAALtFLLC | 0.222 | 0.038 | -0.654 | -0.131 |
| sp\|Q9UHX3\|AGRE2_HUMAN Adhesion G protein-coupled receptor E2 OS=Homo sapiens GN=ADGRE2 PE=1 S | 567 | CKAIQNtSTSL | 0.243 | 0.07 | -0.698 | -0.128 |
| sp\|Q9UHX3\|AGRE2_HUMAN Adhesion G protein-coupled receptor E2 OS=Homo sapiens GN=ADGRE2 PE=1 S | 568 | KAIQNTsTSLH | 0.082 | -0.275 | -1.304 | -0.499 |
| sp\|Q9UHX3\|AGRE2_HUMAN Adhesion G protein-coupled receptor E2 OS=Homo sapiens GN=ADGRE2 PE=1 S | 569 | AIQNTStSLHL | 0.049 | -0.075 | -1.25 | -0.425 |
| sp\|Q9UHX3\|AGRE2_HUMAN Adhesion G protein-coupled receptor E2 OS=Homo sapiens GN=ADGRE2 PE=1 S | 570 | IQNTSTsLHLQ | 0.514 | 0.212 | -0.016 | 0.237 |
| sp\|Q9UHX3\|AGRE2_HUMAN Adhesion G protein-coupled receptor E2 OS=Homo sapiens GN=ADGRE2 PE=1 S | 576 | SLHLQLsLCLF | 0.071 | -0.104 | -1.313 | -0.449 |
| sp\|Q9UHX3\|AGRE2_HUMAN Adhesion G protein-coupled receptor E2 OS=Homo sapiens GN=ADGRE2 PE=1 S | 593 | LVAIDQtGHKV | 0.091 | -0.113 | -1.137 | -0.386 |
| sp\|Q9UHX3\|AGRE2_HUMAN Adhesion G protein-coupled receptor E2 OS=Homo sapiens GN=ADGRE2 PE=1 S | 600 | GHKVLCsIIAG | 0.184 | 0.005 | -1.002 | -0.271 |
| sp\|Q9UHX3\|AGRE2_HUMAN Adhesion G protein-coupled receptor E2 OS=Homo sapiens GN=ADGRE2 PE=1 S | 605 | CSIIAGtLHYL | 0.369 | 0.096 | -0.459 | 0.002 |
| sp\|Q9UHX3\|AGRE2_HUMAN Adhesion G protein-coupled receptor E2 OS=Homo sapiens GN=ADGRE2 PE=1 S | 613 | HYLYLAtLTWM | 0.476 | 0.18 | -0.02 | 0.212 |
| sp\|Q9UHX3\|AGRE2_HUMAN Adhesion G protein-coupled receptor E2 OS=Homo sapiens GN=ADGRE2 PE=1 S | 615 | LYLATLtWMLL | 0.114 | 0.098 | -0.79 | -0.193 |
| sp\|Q9UHX3\|AGRE2_HUMAN Adhesion G protein-coupled receptor E2 OS=Homo sapiens GN=ADGRE2 PE=1 S | 627 | ALYLFLtARNL | 0.191 | 0.017 | -1.123 | -0.305 |
| sp\|Q9UHX3\|AGRE2_HUMAN Adhesion G protein-coupled receptor E2 OS=Homo sapiens GN=ADGRE2 PE=1 S | 632 | LTARNLtVVNY | 0.598 | 0.86 | 0.216 | 0.558 |
| sp\|Q9UHX3\|AGRE2_HUMAN Adhesion G protein-coupled receptor E2 OS=Homo sapiens GN=ADGRE2 PE=1 S | 637 | LTVVNYsSINR | 0.107 | -0.01 | -0.893 | -0.265 |
| sp\|Q9UHX3\|AGRE2_HUMAN Adhesion G protein-coupled receptor E2 OS=Homo sapiens GN=ADGRE2 PE=1 S | 638 | TVVNYSsINRF | 0.053 | -0.242 | -1.166 | -0.452 |
| sp\|Q9UHX3\|AGRE2_HUMAN Adhesion G protein-coupled receptor E2 OS=Homo sapiens GN=ADGRE2 PE=1 S | 658 | YGVPAVtVAIS | 0.249 | -0.09 | -0.631 | -0.157 |
| sp\|Q9UHX3\|AGRE2_HUMAN Adhesion G protein-coupled receptor E2 OS=Homo sapiens GN=ADGRE2 PE=1 S | 662 | AVTVAIsAASR | 0.115 | -0.071 | -1.075 | -0.344 |
| sp\|Q9UHX3\|AGRE2_HUMAN Adhesion G protein-coupled receptor E2 OS=Homo sapiens GN=ADGRE2 PE=1 S | 665 | VAISAAsRPHL | 0.454 | 0.349 | -0.233 | 0.19 |
| sp\|Q9UHX3\|AGRE2_HUMAN Adhesion G protein-coupled receptor E2 OS=Homo sapiens GN=ADGRE2 PE=1 S | 672 | RPHLYGtPSRC | 0.026 | -0.651 | -2.398 | -1.008 |
| sp\|Q9UHX3\|AGRE2_HUMAN Adhesion G protein-coupled receptor E2 OS=Homo sapiens GN=ADGRE2 PE=1 S | 674 | HLYGTPsRCWL | 0.088 | -0.041 | -0.876 | -0.276 |
| sp\|Q9UHX3\|AGRE2_HUMAN Adhesion G protein-coupled receptor E2 OS=Homo sapiens GN=ADGRE2 PE=1 S | 697 | PVCAIFsVNLV | 0.28 | 0.077 | -0.503 | -0.049 |
| sp\|Q9UHX3\|AGRE2_HUMAN Adhesion G protein-coupled receptor E2 OS=Homo sapiens GN=ADGRE2 PE=1 S | 706 | LVLFLVtLWIL | 0.525 | 0.135 | -0.144 | 0.172 |
| sp\|Q9UHX3\|AGRE2_HUMAN Adhesion G protein-coupled receptor E2 OS=Homo sapiens GN=ADGRE2 PE=1 S | 715 | ILKNRLsSLNS | 0.247 | 0.14 | -0.61 | -0.074 |
| sp\|Q9UHX3\|AGRE2_HUMAN Adhesion G protein-coupled receptor E2 OS=Homo sapiens GN=ADGRE2 PE=1 S | 716 | LKNRLSsLNSE | 0.592 | 0.823 | 0.338 | 0.584 |
| sp\|Q9UHX3\|AGRE2_HUMAN Adhesion G protein-coupled receptor E2 OS=Homo sapiens GN=ADGRE2 PE=1 S | 719 | RLSSLNsEVST | 0.156 | -0.005 | -1.009 | -0.286 |
| sp\|Q9UHX3\|AGRE2_HUMAN Adhesion G protein-coupled receptor E2 OS=Homo sapiens GN=ADGRE2 PE=1 S | 722 | SLNSEVsTLRN | 0.172 | -0.21 | -1.479 | -0.506 |
| sp\|Q9UHX3\|AGRE2_HUMAN Adhesion G protein-coupled receptor E2 OS=Homo sapiens GN=ADGRE2 PE=1 S | 723 | LNSEVStLRNT | 0.093 | -0.233 | -1.097 | -0.412 |
| sp\|Q9UHX3\|AGRE2_HUMAN Adhesion G protein-coupled receptor E2 OS=Homo sapiens GN=ADGRE2 PE=1 S | 727 | VSTLRNtRMLA | 0.323 | -0.012 | -0.7 | -0.13 |
| sp\|Q9UHX3\|AGRE2_HUMAN Adhesion G protein-coupled receptor E2 OS=Homo sapiens GN=ADGRE2 PE=1 S | 735 | MLAFKAtAQLF | 0.384 | 0.203 | -0.365 | 0.074 |
| sp\|Q9UHX3\|AGRE2_HUMAN Adhesion G protein-coupled receptor E2 OS=Homo sapiens GN=ADGRE2 PE=1 S | 744 | LFILGCtWCLG | 0.134 | -0.036 | -1.102 | -0.335 |
| sp\|Q9UHX3\|AGRE2_HUMAN Adhesion G protein-coupled receptor E2 OS=Homo sapiens GN=ADGRE2 PE=1 S | 764 | VMAYLFtIINS | 0.064 | 0.03 | -1.001 | -0.302 |

| | | | | | | |
|---|---|---|---|---|---|---|
| sp\|Q9UHX3\|AGRE2_HUMAN Adhesion G protein-coupled receptor E2 OS=Homo sapiens GN=ADGRE2 PE=1 S | 768 | LFTIINsLQGV | 0.478 | 0.127 | -0.049 | 0.185 |
| sp\|Q9UHX3\|AGRE2_HUMAN Adhesion G protein-coupled receptor E2 OS=Homo sapiens GN=ADGRE2 PE=1 S | 782 | LVYCLLsQQVR | 0.083 | -0.148 | -1.353 | -0.473 |
| sp\|Q9UHX3\|AGRE2_HUMAN Adhesion G protein-coupled receptor E2 OS=Homo sapiens GN=ADGRE2 PE=1 S | 793 | EQYGKWsKGIR | 0.135 | -0.119 | -0.656 | -0.213 |
| sp\|Q9UHX3\|AGRE2_HUMAN Adhesion G protein-coupled receptor E2 OS=Homo sapiens GN=ADGRE2 PE=1 S | 801 | GIRKLKtESEM | 0.693 | 0.43 | 0.153 | 0.425 |
| sp\|Q9UHX3\|AGRE2_HUMAN Adhesion G protein-coupled receptor E2 OS=Homo sapiens GN=ADGRE2 PE=1 S | 803 | RKLKTEsEMHT | 0.333 | 0.093 | -0.434 | -0.003 |
| sp\|Q9UHX3\|AGRE2_HUMAN Adhesion G protein-coupled receptor E2 OS=Homo sapiens GN=ADGRE2 PE=1 S | 807 | TESEMHtLSSS | 0.085 | -0.114 | -1.202 | -0.41 |
| sp\|Q9UHX3\|AGRE2_HUMAN Adhesion G protein-coupled receptor E2 OS=Homo sapiens GN=ADGRE2 PE=1 S | 809 | SEMHTLsSSAK | 0.038 | -0.203 | -1.598 | -0.588 |
| sp\|Q9UHX3\|AGRE2_HUMAN Adhesion G protein-coupled receptor E2 OS=Homo sapiens GN=ADGRE2 PE=1 S | 810 | EMHTLSsSAKA | 0.022 | -0.278 | -1.931 | -0.729 |
| sp\|Q9UHX3\|AGRE2_HUMAN Adhesion G protein-coupled receptor E2 OS=Homo sapiens GN=ADGRE2 PE=1 S | 811 | MHTLSSsAKAD | 0.388 | 0.312 | -0.355 | 0.115 |
| sp\|Q9UHX3\|AGRE2_HUMAN Adhesion G protein-coupled receptor E2 OS=Homo sapiens GN=ADGRE2 PE=1 S | 816 | SSAKADtSKPS | 0.148 | -0.255 | -1.156 | -0.421 |
| sp\|Q9UHX3\|AGRE2_HUMAN Adhesion G protein-coupled receptor E2 OS=Homo sapiens GN=ADGRE2 PE=1 S | 817 | SAKADTsKPST | 0.275 | 0.215 | -0.423 | 0.022 |
| sp\|Q9UHX3\|AGRE2_HUMAN Adhesion G protein-coupled receptor E2 OS=Homo sapiens GN=ADGRE2 PE=1 S | 820 | ADTSKPsTVN- | 0.054 | -0.286 | -1.639 | -0.624 |
| sp\|Q9UHX3\|AGRE2_HUMAN Adhesion G protein-coupled receptor E2 OS=Homo sapiens GN=ADGRE2 PE=1 S | 821 | DTSKPStVN-- | 0.25 | -0.022 | -0.762 | -0.178 |
| sp\|Q9BY15\|AGRE3_HUMAN Adhesion G protein-coupled receptor E3 OS=Homo sapiens GN=ADGRE3 PE=2 S\ | 317 | GQGSQWsRDGC | 0.092 | -0.311 | -1.422 | -0.547 |
| sp\|Q9BY15\|AGRE3_HUMAN Adhesion G protein-coupled receptor E3 OS=Homo sapiens GN=ADGRE3 PE=2 S\ | 329 | LIHVNKsHTMC | 0.171 | -0.113 | -0.942 | -0.295 |
| sp\|Q9BY15\|AGRE3_HUMAN Adhesion G protein-coupled receptor E3 OS=Homo sapiens GN=ADGRE3 PE=2 S\ | 331 | HVNKSHtMCNC | 0.393 | 0.399 | -0.574 | 0.073 |
| sp\|Q9BY15\|AGRE3_HUMAN Adhesion G protein-coupled receptor E3 OS=Homo sapiens GN=ADGRE3 PE=2 S\ | 336 | HTMCNCsHLSS | 0.173 | -0.042 | -1.108 | -0.326 |
| sp\|Q9BY15\|AGRE3_HUMAN Adhesion G protein-coupled receptor E3 OS=Homo sapiens GN=ADGRE3 PE=2 S\ | 339 | CNCSHLsSFAV | 0.18 | -0.085 | -0.949 | -0.285 |
| sp\|Q9BY15\|AGRE3_HUMAN Adhesion G protein-coupled receptor E3 OS=Homo sapiens GN=ADGRE3 PE=2 S\ | 340 | NCSHLSsFAVL | 0.138 | -0.067 | -1.053 | -0.327 |
| sp\|Q9BY15\|AGRE3_HUMAN Adhesion G protein-coupled receptor E3 OS=Homo sapiens GN=ADGRE3 PE=2 S\ | 348 | AVLMALtSQEE | 0.174 | -0.128 | -0.916 | -0.29 |
| sp\|Q9BY15\|AGRE3_HUMAN Adhesion G protein-coupled receptor E3 OS=Homo sapiens GN=ADGRE3 PE=2 S\ | 349 | VLMALTsQEED | 0.227 | -0.032 | -0.855 | -0.22 |
| sp\|Q9BY15\|AGRE3_HUMAN Adhesion G protein-coupled receptor E3 OS=Homo sapiens GN=ADGRE3 PE=2 S\ | 357 | EEDPVLtVITY | 0.073 | -0.19 | -1.274 | -0.464 |
| sp\|Q9BY15\|AGRE3_HUMAN Adhesion G protein-coupled receptor E3 OS=Homo sapiens GN=ADGRE3 PE=2 S\ | 360 | PVLTVItYVGL | 0.163 | -0.093 | -0.811 | -0.247 |
| sp\|Q9BY15\|AGRE3_HUMAN Adhesion G protein-coupled receptor E3 OS=Homo sapiens GN=ADGRE3 PE=2 S\ | 365 | ITYVGLsVSLL | 0.042 | -0.2 | -1.388 | -0.515 |
| sp\|Q9BY15\|AGRE3_HUMAN Adhesion G protein-coupled receptor E3 OS=Homo sapiens GN=ADGRE3 PE=2 S\ | 367 | YVGLSVsLLCL | 0.433 | 0.341 | -0.072 | 0.234 |
| sp\|Q9BY15\|AGRE3_HUMAN Adhesion G protein-coupled receptor E3 OS=Homo sapiens GN=ADGRE3 PE=2 S\ | 377 | LLLAALtFLLC | 0.222 | 0.038 | -0.654 | -0.131 |
| sp\|Q9BY15\|AGRE3_HUMAN Adhesion G protein-coupled receptor E3 OS=Homo sapiens GN=ADGRE3 PE=2 S\ | 387 | CKAIRNtSTSL | 0.505 | 0.238 | -0.202 | 0.18 |
| sp\|Q9BY15\|AGRE3_HUMAN Adhesion G protein-coupled receptor E3 OS=Homo sapiens GN=ADGRE3 PE=2 S\ | 388 | KAIRNTsTSLH | 0.322 | 0.599 | -0.392 | 0.176 |
| sp\|Q9BY15\|AGRE3_HUMAN Adhesion G protein-coupled receptor E3 OS=Homo sapiens GN=ADGRE3 PE=2 S\ | 389 | AIRNTStSLHL | 0.093 | 0.118 | -0.901 | -0.23 |
| sp\|Q9BY15\|AGRE3_HUMAN Adhesion G protein-coupled receptor E3 OS=Homo sapiens GN=ADGRE3 PE=2 S\ | 390 | IRNTSTsLHLQ | 0.642 | 0.455 | 0.14 | 0.412 |
| sp\|Q9BY15\|AGRE3_HUMAN Adhesion G protein-coupled receptor E3 OS=Homo sapiens GN=ADGRE3 PE=2 S\ | 396 | SLHLQLsLCLF | 0.071 | -0.104 | -1.313 | -0.449 |
| sp\|Q9BY15\|AGRE3_HUMAN Adhesion G protein-coupled receptor E3 OS=Homo sapiens GN=ADGRE3 PE=2 S\ | 413 | LVGIDRtEPKV | 0.24 | 0.445 | -0.715 | -0.01 |
| sp\|Q9BY15\|AGRE3_HUMAN Adhesion G protein-coupled receptor E3 OS=Homo sapiens GN=ADGRE3 PE=2 S\ | 420 | EPKVLCsIIAG | 0.109 | -0.081 | -0.949 | -0.307 |
| sp\|Q9BY15\|AGRE3_HUMAN Adhesion G protein-coupled receptor E3 OS=Homo sapiens GN=ADGRE3 PE=2 S\ | 435 | LYLAAFtWMLL | 0.356 | 0.234 | -0.235 | 0.118 |
| sp\|Q9BY15\|AGRE3_HUMAN Adhesion G protein-coupled receptor E3 OS=Homo sapiens GN=ADGRE3 PE=2 S\ | 447 | GVHLFLtARNL | 0.111 | -0.034 | -1.409 | -0.444 |
| sp\|Q9BY15\|AGRE3_HUMAN Adhesion G protein-coupled receptor E3 OS=Homo sapiens GN=ADGRE3 PE=2 S\ | 452 | LTARNLtVVNY | 0.598 | 0.86 | 0.216 | 0.558 |
| sp\|Q9BY15\|AGRE3_HUMAN Adhesion G protein-coupled receptor E3 OS=Homo sapiens GN=ADGRE3 PE=2 S\ | 457 | LTVVNYsSINR | 0.107 | -0.01 | -0.893 | -0.265 |
| sp\|Q9BY15\|AGRE3_HUMAN Adhesion G protein-coupled receptor E3 OS=Homo sapiens GN=ADGRE3 PE=2 S\ | 458 | TVVNYSsINRL | 0.069 | -0.177 | -1.039 | -0.382 |
| sp\|Q9BY15\|AGRE3_HUMAN Adhesion G protein-coupled receptor E3 OS=Homo sapiens GN=ADGRE3 PE=2 S\ | 478 | YGVPAVtVAIS | 0.249 | -0.09 | -0.631 | -0.157 |
| sp\|Q9BY15\|AGRE3_HUMAN Adhesion G protein-coupled receptor E3 OS=Homo sapiens GN=ADGRE3 PE=2 S\ | 482 | AVTVAIsAASW | 0.095 | -0.066 | -1.201 | -0.391 |
| sp\|Q9BY15\|AGRE3_HUMAN Adhesion G protein-coupled receptor E3 OS=Homo sapiens GN=ADGRE3 PE=2 S\ | 485 | VAISAAsWPHL | 0.501 | 0.519 | -0.022 | 0.333 |
| sp\|Q9BY15\|AGRE3_HUMAN Adhesion G protein-coupled receptor E3 OS=Homo sapiens GN=ADGRE3 PE=2 S\ | 492 | WPHLYGtADRC | 0.081 | -0.288 | -1.681 | -0.629 |
| sp\|Q9BY15\|AGRE3_HUMAN Adhesion G protein-coupled receptor E3 OS=Homo sapiens GN=ADGRE3 PE=2 S\ | 507 | DQGFMWsFLGP | 0.14 | -0.22 | -0.957 | -0.346 |
| sp\|Q9BY15\|AGRE3_HUMAN Adhesion G protein-coupled receptor E3 OS=Homo sapiens GN=ADGRE3 PE=2 S\ | 517 | PVCAIFsANLV | 0.29 | 0.1 | -0.612 | -0.074 |
| sp\|Q9BY15\|AGRE3_HUMAN Adhesion G protein-coupled receptor E3 OS=Homo sapiens GN=ADGRE3 PE=2 S\ | 535 | ILKRKLsSLNS | 0.472 | 0.82 | -0.038 | 0.418 |
| sp\|Q9BY15\|AGRE3_HUMAN Adhesion G protein-coupled receptor E3 OS=Homo sapiens GN=ADGRE3 PE=2 S\ | 536 | LKRKLSsLNSE | 0.558 | 0.268 | 0.051 | 0.292 |
| sp\|Q9BY15\|AGRE3_HUMAN Adhesion G protein-coupled receptor E3 OS=Homo sapiens GN=ADGRE3 PE=2 S\ | 539 | KLSSLNsEVST | 0.116 | -0.081 | -1.155 | -0.373 |
| sp\|Q9BY15\|AGRE3_HUMAN Adhesion G protein-coupled receptor E3 OS=Homo sapiens GN=ADGRE3 PE=2 S\ | 542 | SLNSEVsTIQN | 0.201 | -0.13 | -1.029 | -0.319 |
| sp\|Q9BY15\|AGRE3_HUMAN Adhesion G protein-coupled receptor E3 OS=Homo sapiens GN=ADGRE3 PE=2 S\ | 543 | LNSEVStIQNT | 0.045 | -0.303 | -1.471 | -0.576 |

| | | | | | | |
|---|---|---|---|---|---|---|
| sp\|Q9BY15\|AGRE3_HUMAN Adhesion G protein-coupled receptor E3 OS=Homo sapiens GN=ADGRE3 PE=2 S\ | 547 | VSTIQNtRMLA | 0.135 | -0.165 | -1.173 | -0.401 |
| sp\|Q9BY15\|AGRE3_HUMAN Adhesion G protein-coupled receptor E3 OS=Homo sapiens GN=ADGRE3 PE=2 S\ | 555 | MLAFKAtAQLF | 0.384 | 0.203 | -0.365 | 0.074 |
| sp\|Q9BY15\|AGRE3_HUMAN Adhesion G protein-coupled receptor E3 OS=Homo sapiens GN=ADGRE3 PE=2 S\ | 564 | LFILGCtWCLG | 0.134 | -0.036 | -1.102 | -0.335 |
| sp\|Q9BY15\|AGRE3_HUMAN Adhesion G protein-coupled receptor E3 OS=Homo sapiens GN=ADGRE3 PE=2 S\ | 584 | VMAYLFtIINS | 0.064 | 0.03 | -1.001 | -0.302 |
| sp\|Q9BY15\|AGRE3_HUMAN Adhesion G protein-coupled receptor E3 OS=Homo sapiens GN=ADGRE3 PE=2 S\ | 588 | LFTIINsLQGF | 0.396 | 0.102 | 0.079 | 0.192 |
| sp\|Q9BY15\|AGRE3_HUMAN Adhesion G protein-coupled receptor E3 OS=Homo sapiens GN=ADGRE3 PE=2 S\ | 602 | LVYCLLsQQVQ | 0.063 | -0.206 | -1.568 | -0.57 |
| sp\|Q9BY15\|AGRE3_HUMAN Adhesion G protein-coupled receptor E3 OS=Homo sapiens GN=ADGRE3 PE=2 S\ | 619 | FREIVKsKSES | 0.302 | 0.25 | -0.447 | 0.035 |
| sp\|Q9BY15\|AGRE3_HUMAN Adhesion G protein-coupled receptor E3 OS=Homo sapiens GN=ADGRE3 PE=2 S\ | 621 | EIVKSKsESET | 0.528 | 0.353 | 0.141 | 0.341 |
| sp\|Q9BY15\|AGRE3_HUMAN Adhesion G protein-coupled receptor E3 OS=Homo sapiens GN=ADGRE3 PE=2 S\ | 623 | VKSKSEsETYT | 0.607 | 0.181 | -0.141 | 0.216 |
| sp\|Q9BY15\|AGRE3_HUMAN Adhesion G protein-coupled receptor E3 OS=Homo sapiens GN=ADGRE3 PE=2 S\ | 625 | SKSESEtYTLS | 0.189 | 0.025 | -0.968 | -0.251 |
| sp\|Q9BY15\|AGRE3_HUMAN Adhesion G protein-coupled receptor E3 OS=Homo sapiens GN=ADGRE3 PE=2 S\ | 627 | SESETYtLSSK | 0.043 | -0.13 | -1.381 | -0.489 |
| sp\|Q9BY15\|AGRE3_HUMAN Adhesion G protein-coupled receptor E3 OS=Homo sapiens GN=ADGRE3 PE=2 S\ | 629 | SETYTLsSKMG | 0.049 | -0.213 | -1.489 | -0.551 |
| sp\|Q9BY15\|AGRE3_HUMAN Adhesion G protein-coupled receptor E3 OS=Homo sapiens GN=ADGRE3 PE=2 S\ | 630 | ETYTLSsKMGP | 0.046 | -0.33 | -1.495 | -0.593 |
| sp\|Q9BY15\|AGRE3_HUMAN Adhesion G protein-coupled receptor E3 OS=Homo sapiens GN=ADGRE3 PE=2 S\ | 636 | SKMGPDsKPSE | 0.283 | 0.259 | -0.436 | 0.035 |
| sp\|Q9BY15\|AGRE3_HUMAN Adhesion G protein-coupled receptor E3 OS=Homo sapiens GN=ADGRE3 PE=2 S\ | 639 | GPDSKPsEGDV | 0.251 | -0.053 | -0.997 | -0.266 |
| sp\|Q86SQ3\|AGRE4_HUMAN Putative adhesion G protein-coupled receptor E4P OS=Homo sapiens GN=ADGR | 316 | AVIIAVsAIVG | 0.301 | 0.127 | -0.644 | -0.072 |
| sp\|Q86SQ3\|AGRE4_HUMAN Putative adhesion G protein-coupled receptor E4P OS=Homo sapiens GN=ADGR | 326 | GPQNYGtFTCW | 0.241 | 0.07 | -0.758 | -0.149 |
| sp\|Q86SQ3\|AGRE4_HUMAN Putative adhesion G protein-coupled receptor E4P OS=Homo sapiens GN=ADGR | 328 | QNYGTFtCWLK | 0.114 | 0.021 | -0.83 | -0.232 |
| sp\|Q86SQ3\|AGRE4_HUMAN Putative adhesion G protein-coupled receptor E4P OS=Homo sapiens GN=ADGR | 340 | DKGFIWsFMGP | 0.372 | -0.031 | -0.422 | -0.027 |
| sp\|Q86SQ3\|AGRE4_HUMAN Putative adhesion G protein-coupled receptor E4P OS=Homo sapiens GN=ADGR | 365 | VLWILRsKLSS | 0.04 | -0.022 | -1.542 | -0.508 |
| sp\|Q86SQ3\|AGRE4_HUMAN Putative adhesion G protein-coupled receptor E4P OS=Homo sapiens GN=ADGR | 368 | ILRSKLsSLNK | 0.209 | 0.07 | -0.911 | -0.211 |
| sp\|Q86SQ3\|AGRE4_HUMAN Putative adhesion G protein-coupled receptor E4P OS=Homo sapiens GN=ADGR | 369 | LRSKLSsLNKE | 0.287 | 0.152 | -0.418 | 0.007 |
| sp\|Q86SQ3\|AGRE4_HUMAN Putative adhesion G protein-coupled receptor E4P OS=Homo sapiens GN=ADGR | 375 | SLNKEVsTIQD | 0.429 | -0.005 | -0.494 | -0.023 |
| sp\|Q86SQ3\|AGRE4_HUMAN Putative adhesion G protein-coupled receptor E4P OS=Homo sapiens GN=ADGR | 376 | LNKEVStIQDT | 0.093 | -0.213 | -0.934 | -0.351 |
| sp\|Q86SQ3\|AGRE4_HUMAN Putative adhesion G protein-coupled receptor E4P OS=Homo sapiens GN=ADGR | 380 | VSTIQDtRVMT | 0.06 | -0.41 | -1.74 | -0.697 |
| sp\|Q86SQ3\|AGRE4_HUMAN Putative adhesion G protein-coupled receptor E4P OS=Homo sapiens GN=ADGR | 384 | QDTRVMtFKAI | 0.796 | 1.054 | 1.03 | 0.96 |
| sp\|Q86SQ3\|AGRE4_HUMAN Putative adhesion G protein-coupled receptor E4P OS=Homo sapiens GN=ADGR | 389 | MTFKAIsQLFI | 0.455 | 0.075 | -0.566 | -0.012 |
| sp\|Q86SQ3\|AGRE4_HUMAN Putative adhesion G protein-coupled receptor E4P OS=Homo sapiens GN=ADGR | 397 | LFILGCsWGLG | 0.237 | 0.11 | -0.715 | -0.123 |
| sp\|Q86SQ3\|AGRE4_HUMAN Putative adhesion G protein-coupled receptor E4P OS=Homo sapiens GN=ADGR | 411 | VEEVGKtIGSI | 0.103 | -0.129 | -0.815 | -0.28 |
| sp\|Q86SQ3\|AGRE4_HUMAN Putative adhesion G protein-coupled receptor E4P OS=Homo sapiens GN=ADGR | 414 | VGKTIGsIIAY | 0.159 | -0.094 | -0.564 | -0.166 |
| sp\|Q86SQ3\|AGRE4_HUMAN Putative adhesion G protein-coupled receptor E4P OS=Homo sapiens GN=ADGR | 419 | GSIIAYsFTII | 0.597 | 0.255 | 0.025 | 0.292 |
| sp\|Q86SQ3\|AGRE4_HUMAN Putative adhesion G protein-coupled receptor E4P OS=Homo sapiens GN=ADGR | 421 | IIAYSFtIINT | 0.331 | 0.282 | 0.095 | 0.236 |
| sp\|Q86SQ3\|AGRE4_HUMAN Putative adhesion G protein-coupled receptor E4P OS=Homo sapiens GN=ADGR | 425 | SFTIINtLQGV | 0.394 | 0.075 | -0.334 | 0.045 |
| sp\|Q86SQ3\|AGRE4_HUMAN Putative adhesion G protein-coupled receptor E4P OS=Homo sapiens GN=ADGR | 448 | VRLIILsVISL | 0.07 | 0.127 | -0.774 | -0.192 |
| sp\|Q86SQ3\|AGRE4_HUMAN Putative adhesion G protein-coupled receptor E4P OS=Homo sapiens GN=ADGR | 451 | IILSVIsLVPK | 0.359 | -0.042 | -0.36 | -0.014 |
| sp\|Q86SQ3\|AGRE4_HUMAN Putative adhesion G protein-coupled receptor E4P OS=Homo sapiens GN=ADGR | 456 | ISLVPKsN--- | 0.157 | -0.044 | -0.764 | -0.217 |
| sp\|Q9Y653\|AGRG1_HUMAN Adhesion G-protein coupled receptor G1 OS=Homo sapiens GN=ADGRG1 PE=1 S | 320 | GIVVQNtKVAN | 0.237 | 0.176 | -0.8 | -0.129 |
| sp\|Q9Y653\|AGRG1_HUMAN Adhesion G-protein coupled receptor G1 OS=Homo sapiens GN=ADGRG1 PE=1 S | 326 | TKVANLtEPVV | 0.628 | 0.537 | 0.005 | 0.39 |
| sp\|Q9Y653\|AGRG1_HUMAN Adhesion G-protein coupled receptor G1 OS=Homo sapiens GN=ADGRG1 PE=1 S | 332 | TEPVVLtFQHQ | 0.055 | -0.253 | -1.451 | -0.55 |
| sp\|Q9Y653\|AGRG1_HUMAN Adhesion G-protein coupled receptor G1 OS=Homo sapiens GN=ADGRG1 PE=1 S | 343 | LQPKNVtLQCV | 0.528 | 0.106 | -0.127 | 0.169 |
| sp\|Q9Y653\|AGRG1_HUMAN Adhesion G-protein coupled receptor G1 OS=Homo sapiens GN=ADGRG1 PE=1 S | 354 | FWVEDPtLSSP | 0.063 | -0.126 | -1.308 | -0.457 |
| sp\|Q9Y653\|AGRG1_HUMAN Adhesion G-protein coupled receptor G1 OS=Homo sapiens GN=ADGRG1 PE=1 S | 356 | VEDPTLsSPGH | 0.116 | 0.092 | -1.053 | -0.282 |
| sp\|Q9Y653\|AGRG1_HUMAN Adhesion G-protein coupled receptor G1 OS=Homo sapiens GN=ADGRG1 PE=1 S | 357 | EDPTLSsPGHW | 0.014 | -0.635 | -2.065 | -0.895 |
| sp\|Q9Y653\|AGRG1_HUMAN Adhesion G-protein coupled receptor G1 OS=Homo sapiens GN=ADGRG1 PE=1 S | 362 | SSPGHWsSAGC | 0.051 | -0.425 | -1.739 | -0.704 |
| sp\|Q9Y653\|AGRG1_HUMAN Adhesion G-protein coupled receptor G1 OS=Homo sapiens GN=ADGRG1 PE=1 S | 363 | SPGHWSsAGCE | 0.084 | -0.112 | -1.382 | -0.47 |
| sp\|Q9Y653\|AGRG1_HUMAN Adhesion G-protein coupled receptor G1 OS=Homo sapiens GN=ADGRG1 PE=1 S | 368 | SSAGCEtVRRE | 0.137 | -0.34 | -1.237 | -0.48 |
| sp\|Q9Y653\|AGRG1_HUMAN Adhesion G-protein coupled receptor G1 OS=Homo sapiens GN=ADGRG1 PE=1 S | 373 | ETVRREtQTSC | 0.441 | 0.716 | -0.545 | 0.204 |
| sp\|Q9Y653\|AGRG1_HUMAN Adhesion G-protein coupled receptor G1 OS=Homo sapiens GN=ADGRG1 PE=1 S | 375 | VRRETQtSCFC | 0.088 | 0.135 | -1.28 | -0.352 |
| sp\|Q9Y653\|AGRG1_HUMAN Adhesion G-protein coupled receptor G1 OS=Homo sapiens GN=ADGRG1 PE=1 S | 376 | RRETQTsCFCN | 0.272 | 0.339 | -0.682 | -0.024 |

| | | | | | | |
|---|---|---|---|---|---|---|
| sp\|Q9Y653\|AGRG1_HUMAN Adhesion G-protein coupled receptor G1 OS=Homo sapiens GN=ADGRG1 PE=1 S | 383 | CFCNHLtYFAV | 0.255 | 0.073 | -0.507 | -0.06 |
| sp\|Q9Y653\|AGRG1_HUMAN Adhesion G-protein coupled receptor G1 OS=Homo sapiens GN=ADGRG1 PE=1 S | 391 | FAVLMVsSVEV | 0.259 | -0.073 | -1.058 | -0.291 |
| sp\|Q9Y653\|AGRG1_HUMAN Adhesion G-protein coupled receptor G1 OS=Homo sapiens GN=ADGRG1 PE=1 S | 392 | AVLMVSsVEVD | 0.133 | -0.13 | -0.996 | -0.331 |
| sp\|Q9Y653\|AGRG1_HUMAN Adhesion G-protein coupled receptor G1 OS=Homo sapiens GN=ADGRG1 PE=1 S | 404 | VHKHYLsLLSY | 0.147 | -0.044 | -1.028 | -0.308 |
| sp\|Q9Y653\|AGRG1_HUMAN Adhesion G-protein coupled receptor G1 OS=Homo sapiens GN=ADGRG1 PE=1 S | 407 | HYLSLLsYVGC | 0.052 | -0.252 | -1.436 | -0.545 |
| sp\|Q9Y653\|AGRG1_HUMAN Adhesion G-protein coupled receptor G1 OS=Homo sapiens GN=ADGRG1 PE=1 S | 414 | YVGCVVsALAC | 0.188 | -0.044 | -1.096 | -0.317 |
| sp\|Q9Y653\|AGRG1_HUMAN Adhesion G-protein coupled receptor G1 OS=Homo sapiens GN=ADGRG1 PE=1 S | 421 | ALACLVtIAAY | 0.407 | -0.011 | -0.485 | -0.03 |
| sp\|Q9Y653\|AGRG1_HUMAN Adhesion G-protein coupled receptor G1 OS=Homo sapiens GN=ADGRG1 PE=1 S | 428 | IAAYLCsRVPL | 0.146 | -0.188 | -1.02 | -0.354 |
| sp\|Q9Y653\|AGRG1_HUMAN Adhesion G-protein coupled receptor G1 OS=Homo sapiens GN=ADGRG1 PE=1 S | 442 | RKPRDYtIKVH | 0.495 | 0.788 | 0.131 | 0.471 |
| sp\|Q9Y653\|AGRG1_HUMAN Adhesion G-protein coupled receptor G1 OS=Homo sapiens GN=ADGRG1 PE=1 S | 458 | AVFLLDtSFLL | 0.091 | -0.154 | -1.397 | -0.487 |
| sp\|Q9Y653\|AGRG1_HUMAN Adhesion G-protein coupled receptor G1 OS=Homo sapiens GN=ADGRG1 PE=1 S | 459 | VFLLDTsFLLS | 0.158 | -0.052 | -0.788 | -0.227 |
| sp\|Q9Y653\|AGRG1_HUMAN Adhesion G-protein coupled receptor G1 OS=Homo sapiens GN=ADGRG1 PE=1 S | 463 | DTSFLLsEPVA | 0.502 | 0.414 | -0.344 | 0.191 |
| sp\|Q9Y653\|AGRG1_HUMAN Adhesion G-protein coupled receptor G1 OS=Homo sapiens GN=ADGRG1 PE=1 S | 469 | SEPVALtGSEA | 0.039 | -0.301 | -1.728 | -0.663 |
| sp\|Q9Y653\|AGRG1_HUMAN Adhesion G-protein coupled receptor G1 OS=Homo sapiens GN=ADGRG1 PE=1 S | 471 | PVALTGsEAGC | 0.066 | -0.16 | -1.287 | -0.46 |
| sp\|Q9Y653\|AGRG1_HUMAN Adhesion G-protein coupled receptor G1 OS=Homo sapiens GN=ADGRG1 PE=1 S | 478 | EAGCRAsAIFL | 0.186 | 0.065 | -0.889 | -0.213 |
| sp\|Q9Y653\|AGRG1_HUMAN Adhesion G-protein coupled receptor G1 OS=Homo sapiens GN=ADGRG1 PE=1 S | 485 | AIFLHFsLLTC | 0.3 | 0.085 | -0.746 | -0.12 |
| sp\|Q9Y653\|AGRG1_HUMAN Adhesion G-protein coupled receptor G1 OS=Homo sapiens GN=ADGRG1 PE=1 S | 488 | LHFSLLtCLSW | 0.032 | -0.178 | -1.993 | -0.713 |
| sp\|Q9Y653\|AGRG1_HUMAN Adhesion G-protein coupled receptor G1 OS=Homo sapiens GN=ADGRG1 PE=1 S | 491 | SLLTCLsWMGL | 0.225 | 0.048 | -0.715 | -0.147 |
| sp\|Q9Y653\|AGRG1_HUMAN Adhesion G-protein coupled receptor G1 OS=Homo sapiens GN=ADGRG1 PE=1 S | 510 | VVEVFGtYVPG | 0.043 | -0.317 | -1.595 | -0.623 |
| sp\|Q9Y653\|AGRG1_HUMAN Adhesion G-protein coupled receptor G1 OS=Homo sapiens GN=ADGRG1 PE=1 S | 520 | GYLLKLsAMGW | 0.154 | -0.035 | -1.066 | -0.316 |
| sp\|Q9Y653\|AGRG1_HUMAN Adhesion G-protein coupled receptor G1 OS=Homo sapiens GN=ADGRG1 PE=1 S | 532 | FPIFLVtLVAL | 0.474 | 0.229 | -0.214 | 0.163 |
| sp\|Q9Y653\|AGRG1_HUMAN Adhesion G-protein coupled receptor G1 OS=Homo sapiens GN=ADGRG1 PE=1 S | 552 | ILAVHRtPEGV | 0.112 | -0.279 | -1.259 | -0.475 |
| sp\|Q9Y653\|AGRG1_HUMAN Adhesion G-protein coupled receptor G1 OS=Homo sapiens GN=ADGRG1 PE=1 S | 560 | EGVIYPsMCWI | 0.128 | -0.022 | -0.852 | -0.249 |
| sp\|Q9Y653\|AGRG1_HUMAN Adhesion G-protein coupled receptor G1 OS=Homo sapiens GN=ADGRG1 PE=1 S | 567 | MCWIRDsLVSY | 0.474 | 0.386 | -0.43 | 0.143 |
| sp\|Q9Y653\|AGRG1_HUMAN Adhesion G-protein coupled receptor G1 OS=Homo sapiens GN=ADGRG1 PE=1 S | 570 | IRDSLVsYITN | 0.173 | -0.005 | -0.846 | -0.226 |
| sp\|Q9Y653\|AGRG1_HUMAN Adhesion G-protein coupled receptor G1 OS=Homo sapiens GN=ADGRG1 PE=1 S | 573 | SLVSYItNLGL | 0.138 | -0.122 | -1.144 | -0.376 |
| sp\|Q9Y653\|AGRG1_HUMAN Adhesion G-protein coupled receptor G1 OS=Homo sapiens GN=ADGRG1 PE=1 S | 579 | TNLGLFsLVFL | 0.105 | -0.099 | -0.791 | -0.262 |
| sp\|Q9Y653\|AGRG1_HUMAN Adhesion G-protein coupled receptor G1 OS=Homo sapiens GN=ADGRG1 PE=1 S | 591 | NMAMLAtMVVQ | 0.119 | 0.1 | -1.108 | -0.296 |
| sp\|Q9Y653\|AGRG1_HUMAN Adhesion G-protein coupled receptor G1 OS=Homo sapiens GN=ADGRG1 PE=1 S | 603 | LRLRPHtQKWS | 0.55 | 1.02 | 0.351 | 0.64 |
| sp\|Q9Y653\|AGRG1_HUMAN Adhesion G-protein coupled receptor G1 OS=Homo sapiens GN=ADGRG1 PE=1 S | 607 | PHTQKWsHVLT | 0.164 | -0.126 | -0.972 | -0.311 |
| sp\|Q9Y653\|AGRG1_HUMAN Adhesion G-protein coupled receptor G1 OS=Homo sapiens GN=ADGRG1 PE=1 S | 611 | KWSHVLtLLGL | 0.152 | -0.073 | -0.805 | -0.242 |
| sp\|Q9Y653\|AGRG1_HUMAN Adhesion G-protein coupled receptor G1 OS=Homo sapiens GN=ADGRG1 PE=1 S | 616 | LTLLGLsLVLG | 0.138 | -0.064 | -0.85 | -0.259 |
| sp\|Q9Y653\|AGRG1_HUMAN Adhesion G-protein coupled receptor G1 OS=Homo sapiens GN=ADGRG1 PE=1 S | 629 | WALIFFsFASG | 0.121 | -0.105 | -0.912 | -0.299 |
| sp\|Q9Y653\|AGRG1_HUMAN Adhesion G-protein coupled receptor G1 OS=Homo sapiens GN=ADGRG1 PE=1 S | 632 | IFFSFAsGTFQ | 0.181 | -0.234 | -1.064 | -0.372 |
| sp\|Q9Y653\|AGRG1_HUMAN Adhesion G-protein coupled receptor G1 OS=Homo sapiens GN=ADGRG1 PE=1 S | 634 | FSFASGtFQLV | 0.433 | 0.214 | -0.393 | 0.085 |
| sp\|Q9Y653\|AGRG1_HUMAN Adhesion G-protein coupled receptor G1 OS=Homo sapiens GN=ADGRG1 PE=1 S | 644 | VVLYLFsIITS | 0.063 | -0.138 | -0.948 | -0.341 |
| sp\|Q9Y653\|AGRG1_HUMAN Adhesion G-protein coupled receptor G1 OS=Homo sapiens GN=ADGRG1 PE=1 S | 647 | YLFSIItSFQG | 0.287 | -0.021 | -0.389 | -0.041 |
| sp\|Q9Y653\|AGRG1_HUMAN Adhesion G-protein coupled receptor G1 OS=Homo sapiens GN=ADGRG1 PE=1 S | 648 | LFSIITsFQGF | 0.224 | -0.011 | -0.198 | 0.005 |
| sp\|Q9Y653\|AGRG1_HUMAN Adhesion G-protein coupled receptor G1 OS=Homo sapiens GN=ADGRG1 PE=1 S | 660 | IFIWYWsMRLQ | 0.367 | -0.023 | -0.52 | -0.059 |
| sp\|Q9Y653\|AGRG1_HUMAN Adhesion G-protein coupled receptor G1 OS=Homo sapiens GN=ADGRG1 PE=1 S | 670 | QARGGPsPLKS | 0.04 | -0.367 | -1.647 | -0.658 |
| sp\|Q9Y653\|AGRG1_HUMAN Adhesion G-protein coupled receptor G1 OS=Homo sapiens GN=ADGRG1 PE=1 S | 674 | GPSPLKsNSDS | 0.13 | -0.176 | -1.178 | -0.408 |
| sp\|Q9Y653\|AGRG1_HUMAN Adhesion G-protein coupled receptor G1 OS=Homo sapiens GN=ADGRG1 PE=1 S | 676 | SPLKSNsDSAR | 0.501 | 0.245 | -0.282 | 0.155 |
| sp\|Q9Y653\|AGRG1_HUMAN Adhesion G-protein coupled receptor G1 OS=Homo sapiens GN=ADGRG1 PE=1 S | 678 | LKSNSDsARLP | 0.314 | 0.067 | -0.427 | -0.015 |
| sp\|Q9Y653\|AGRG1_HUMAN Adhesion G-protein coupled receptor G1 OS=Homo sapiens GN=ADGRG1 PE=1 S | 684 | SARLPIsSGST | 0.129 | -0.073 | -1.026 | -0.323 |
| sp\|Q9Y653\|AGRG1_HUMAN Adhesion G-protein coupled receptor G1 OS=Homo sapiens GN=ADGRG1 PE=1 S | 685 | ARLPISsGSTS | 0.053 | -0.146 | -1.22 | -0.438 |
| sp\|Q9Y653\|AGRG1_HUMAN Adhesion G-protein coupled receptor G1 OS=Homo sapiens GN=ADGRG1 PE=1 S | 687 | LPISSGsTSSS | 0.048 | -0.14 | -1.451 | -0.514 |
| sp\|Q9Y653\|AGRG1_HUMAN Adhesion G-protein coupled receptor G1 OS=Homo sapiens GN=ADGRG1 PE=1 S | 688 | PISSGStSSSR | 0.032 | -0.308 | -1.657 | -0.644 |
| sp\|Q9Y653\|AGRG1_HUMAN Adhesion G-protein coupled receptor G1 OS=Homo sapiens GN=ADGRG1 PE=1 S | 689 | ISSGSTsSSRI | 0.257 | -0.038 | -0.491 | -0.091 |
| sp\|Q9Y653\|AGRG1_HUMAN Adhesion G-protein coupled receptor G1 OS=Homo sapiens GN=ADGRG1 PE=1 S | 690 | SSGSTSsSRI- | 0.038 | -0.358 | -1.804 | -0.708 |

| | | | | | | |
|---|---|---|---|---|---|---|
| sp\|Q9Y653\|AGRG1_HUMAN Adhesion G-protein coupled receptor G1 OS=Homo sapiens GN=ADGRG1 PE=1 S | 691 | SGSTSSsRI-- | 0.117 | 0.023 | -1.02 | -0.293 |
| sp\|Q86Y34\|AGRG3_HUMAN Adhesion G protein-coupled receptor G3 OS=Homo sapiens GN=ADGRG3 PE=1 S | 315 | HVALGGsLFLL | 0.14 | 0.045 | -0.585 | -0.133 |
| sp\|Q86Y34\|AGRG3_HUMAN Adhesion G protein-coupled receptor G3 OS=Homo sapiens GN=ADGRG3 PE=1 S | 329 | FLVNVGsGSKG | 0.096 | -0.094 | -1.135 | -0.378 |
| sp\|Q86Y34\|AGRG3_HUMAN Adhesion G protein-coupled receptor G3 OS=Homo sapiens GN=ADGRG3 PE=1 S | 331 | VNVGSGsKGSD | 0.169 | -0.005 | -0.581 | -0.139 |
| sp\|Q86Y34\|AGRG3_HUMAN Adhesion G protein-coupled receptor G3 OS=Homo sapiens GN=ADGRG3 PE=1 S | 334 | GSGSKGsDAAC | 0.079 | -0.299 | -1.667 | -0.629 |
| sp\|Q86Y34\|AGRG3_HUMAN Adhesion G protein-coupled receptor G3 OS=Homo sapiens GN=ADGRG3 PE=1 S | 354 | FLLCAFtWMGL | 0.567 | 0.342 | 0.017 | 0.309 |
| sp\|Q86Y34\|AGRG3_HUMAN Adhesion G protein-coupled receptor G3 OS=Homo sapiens GN=ADGRG3 PE=1 S | 373 | AVRVFNtYFGH | 0.184 | -0.017 | -1.041 | -0.291 |
| sp\|Q86Y34\|AGRG3_HUMAN Adhesion G protein-coupled receptor G3 OS=Homo sapiens GN=ADGRG3 PE=1 S | 383 | HYFLKLsLVGW | 0.17 | 0.056 | -0.656 | -0.143 |
| sp\|Q86Y34\|AGRG3_HUMAN Adhesion G protein-coupled receptor G3 OS=Homo sapiens GN=ADGRG3 PE=1 S | 397 | ALMVIGtGSAN | 0.089 | -0.07 | -1.007 | -0.329 |
| sp\|Q86Y34\|AGRG3_HUMAN Adhesion G protein-coupled receptor G3 OS=Homo sapiens GN=ADGRG3 PE=1 S | 399 | MVIGTGsANSY | 0.092 | 0.081 | -1.102 | -0.31 |
| sp\|Q86Y34\|AGRG3_HUMAN Adhesion G protein-coupled receptor G3 OS=Homo sapiens GN=ADGRG3 PE=1 S | 402 | GTGSANsYGLY | 0.427 | 0.084 | -0.667 | -0.052 |
| sp\|Q86Y34\|AGRG3_HUMAN Adhesion G protein-coupled receptor G3 OS=Homo sapiens GN=ADGRG3 PE=1 S | 407 | NSYGLYtIRDR | 0.135 | -0.103 | -0.656 | -0.208 |
| sp\|Q86Y34\|AGRG3_HUMAN Adhesion G protein-coupled receptor G3 OS=Homo sapiens GN=ADGRG3 PE=1 S | 415 | RDRENRtSLEL | 0.146 | 0.113 | -1.055 | -0.265 |
| sp\|Q86Y34\|AGRG3_HUMAN Adhesion G protein-coupled receptor G3 OS=Homo sapiens GN=ADGRG3 PE=1 S | 416 | DRENRTsLELC | 0.494 | 0.338 | -0.17 | 0.221 |
| sp\|Q86Y34\|AGRG3_HUMAN Adhesion G protein-coupled receptor G3 OS=Homo sapiens GN=ADGRG3 PE=1 S | 426 | CWFREGtTMYA | 0.537 | 0.738 | -0.098 | 0.392 |
| sp\|Q86Y34\|AGRG3_HUMAN Adhesion G protein-coupled receptor G3 OS=Homo sapiens GN=ADGRG3 PE=1 S | 427 | WFREGTtMYAL | 0.256 | 0.345 | -0.276 | 0.108 |
| sp\|Q86Y34\|AGRG3_HUMAN Adhesion G protein-coupled receptor G3 OS=Homo sapiens GN=ADGRG3 PE=1 S | 434 | MYALYItVHGY | 0.549 | 0.138 | -0.213 | 0.158 |
| sp\|Q86Y34\|AGRG3_HUMAN Adhesion G protein-coupled receptor G3 OS=Homo sapiens GN=ADGRG3 PE=1 S | 442 | HGYFLItFLFG | 0.13 | -0.047 | -0.862 | -0.26 |
| sp\|Q86Y34\|AGRG3_HUMAN Adhesion G protein-coupled receptor G3 OS=Homo sapiens GN=ADGRG3 PE=1 S | 459 | VVWKIFtLSRA | 0.283 | 0.084 | -0.347 | 0.007 |
| sp\|Q86Y34\|AGRG3_HUMAN Adhesion G protein-coupled receptor G3 OS=Homo sapiens GN=ADGRG3 PE=1 S | 461 | WKIFTLsRATA | 0.114 | -0.147 | -0.993 | -0.342 |
| sp\|Q86Y34\|AGRG3_HUMAN Adhesion G protein-coupled receptor G3 OS=Homo sapiens GN=ADGRG3 PE=1 S | 464 | FTLSRAtAVKE | 0.339 | 0.124 | -0.781 | -0.106 |
| sp\|Q86Y34\|AGRG3_HUMAN Adhesion G protein-coupled receptor G3 OS=Homo sapiens GN=ADGRG3 PE=1 S | 478 | NRKKVLtLLGL | 0.476 | 0.303 | -0.045 | 0.245 |
| sp\|Q86Y34\|AGRG3_HUMAN Adhesion G protein-coupled receptor G3 OS=Homo sapiens GN=ADGRG3 PE=1 S | 483 | LTLLGLsSLVG | 0.049 | -0.221 | -1.53 | -0.567 |
| sp\|Q86Y34\|AGRG3_HUMAN Adhesion G protein-coupled receptor G3 OS=Homo sapiens GN=ADGRG3 PE=1 S | 484 | TLLGLSsLVGV | 0.194 | -0.038 | -0.776 | -0.207 |
| sp\|Q86Y34\|AGRG3_HUMAN Adhesion G protein-coupled receptor G3 OS=Homo sapiens GN=ADGRG3 PE=1 S | 489 | SSLVGVtWGLA | 0.114 | -0.121 | -0.985 | -0.331 |
| sp\|Q86Y34\|AGRG3_HUMAN Adhesion G protein-coupled receptor G3 OS=Homo sapiens GN=ADGRG3 PE=1 S | 496 | WGLAIFtPLGL | 0.073 | -0.402 | -0.906 | -0.412 |
| sp\|Q86Y34\|AGRG3_HUMAN Adhesion G protein-coupled receptor G3 OS=Homo sapiens GN=ADGRG3 PE=1 S | 501 | FTPLGLsTVYI | 0.071 | -0.15 | -1.394 | -0.491 |
| sp\|Q86Y34\|AGRG3_HUMAN Adhesion G protein-coupled receptor G3 OS=Homo sapiens GN=ADGRG3 PE=1 S | 502 | TPLGLStVYIF | 0.055 | -0.235 | -1.35 | -0.51 |
| sp\|Q86Y34\|AGRG3_HUMAN Adhesion G protein-coupled receptor G3 OS=Homo sapiens GN=ADGRG3 PE=1 S | 511 | IFALFNsLQGV | 0.578 | 0.085 | -0.187 | 0.159 |
| sp\|Q86Y34\|AGRG3_HUMAN Adhesion G protein-coupled receptor G3 OS=Homo sapiens GN=ADGRG3 PE=1 S | 522 | FICCWFtILYL | 0.268 | 0.146 | -0.684 | -0.09 |
| sp\|Q86Y34\|AGRG3_HUMAN Adhesion G protein-coupled receptor G3 OS=Homo sapiens GN=ADGRG3 PE=1 S | 528 | TILYLPsQSTT | 0.063 | -0.263 | -1.398 | -0.533 |
| sp\|Q86Y34\|AGRG3_HUMAN Adhesion G protein-coupled receptor G3 OS=Homo sapiens GN=ADGRG3 PE=1 S | 530 | LYLPSQsTTVS | 0.326 | 0.15 | -0.353 | 0.041 |
| sp\|Q86Y34\|AGRG3_HUMAN Adhesion G protein-coupled receptor G3 OS=Homo sapiens GN=ADGRG3 PE=1 S | 531 | YLPSQStTVSS | 0.031 | -0.229 | -1.696 | -0.631 |
| sp\|Q86Y34\|AGRG3_HUMAN Adhesion G protein-coupled receptor G3 OS=Homo sapiens GN=ADGRG3 PE=1 S | 532 | LPSQSTtVSSS | 0.125 | -0.011 | -0.743 | -0.21 |
| sp\|Q86Y34\|AGRG3_HUMAN Adhesion G protein-coupled receptor G3 OS=Homo sapiens GN=ADGRG3 PE=1 S | 534 | SQSTTVsSSTA | 0.065 | -0.284 | -1.34 | -0.52 |
| sp\|Q86Y34\|AGRG3_HUMAN Adhesion G protein-coupled receptor G3 OS=Homo sapiens GN=ADGRG3 PE=1 S | 535 | QSTTVSsSTAR | 0.158 | -0.182 | -0.98 | -0.335 |
| sp\|Q86Y34\|AGRG3_HUMAN Adhesion G protein-coupled receptor G3 OS=Homo sapiens GN=ADGRG3 PE=1 S | 536 | STTVSSsTARL | 0.1 | -0.097 | -1.218 | -0.405 |
| sp\|Q86Y34\|AGRG3_HUMAN Adhesion G protein-coupled receptor G3 OS=Homo sapiens GN=ADGRG3 PE=1 S | 537 | TTVSSStARLD | 0.248 | 0.022 | -0.735 | -0.155 |
| sp\|Q86Y34\|AGRG3_HUMAN Adhesion G protein-coupled receptor G3 OS=Homo sapiens GN=ADGRG3 PE=1 S | 545 | RLDQAHsASQE | 0.509 | 0.199 | -0.343 | 0.122 |
| sp\|Q86Y34\|AGRG3_HUMAN Adhesion G protein-coupled receptor G3 OS=Homo sapiens GN=ADGRG3 PE=1 S | 547 | DQAHSAsQE-- | 0.326 | 0.232 | -0.522 | 0.012 |
| sp\|Q8WXG9\|GPR98_HUMAN G-protein coupled receptor 98 OS=Homo sapiens GN=GPR98 PE=1 SV=2 | 329 | IDLQPNtTVVF | 0.129 | -0.178 | -0.839 | -0.296 |
| sp\|Q8WXG9\|GPR98_HUMAN G-protein coupled receptor 98 OS=Homo sapiens GN=GPR98 PE=1 SV=2 | 330 | DLQPNTtVVFP | 0.187 | -0.071 | -0.825 | -0.236 |
| sp\|Q8WXG9\|GPR98_HUMAN G-protein coupled receptor 98 OS=Homo sapiens GN=GPR98 PE=1 SV=2 | 340 | PPFIHEsHLKF | 0.117 | -0.091 | -1.308 | -0.427 |
| sp\|Q8WXG9\|GPR98_HUMAN G-protein coupled receptor 98 OS=Homo sapiens GN=GPR98 PE=1 SV=2 | 350 | FQIVDDtIPEI | 0.428 | 0.436 | -0.131 | 0.244 |
| sp\|Q8WXG9\|GPR98_HUMAN G-protein coupled receptor 98 OS=Homo sapiens GN=GPR98 PE=1 SV=2 | 357 | IPEIAEsFHIM | 0.434 | 0.021 | -0.444 | 0.004 |
| sp\|Q8WXG9\|GPR98_HUMAN G-protein coupled receptor 98 OS=Homo sapiens GN=GPR98 PE=1 SV=2 | 366 | IMLLKDtLQGD | 0.295 | 0.019 | -0.443 | -0.043 |
| sp\|Q8WXG9\|GPR98_HUMAN G-protein coupled receptor 98 OS=Homo sapiens GN=GPR98 PE=1 SV=2 | 375 | GDAVLIsPSVV | 0.032 | -0.493 | -1.967 | -0.809 |
| sp\|Q8WXG9\|GPR98_HUMAN G-protein coupled receptor 98 OS=Homo sapiens GN=GPR98 PE=1 SV=2 | 377 | AVLISPsVVQV | 0.25 | 0.094 | -0.66 | -0.105 |
| sp\|Q8WXG9\|GPR98_HUMAN G-protein coupled receptor 98 OS=Homo sapiens GN=GPR98 PE=1 SV=2 | 382 | PSVVQVtIKPN | 0.088 | -0.213 | -1.191 | -0.439 |

| | | | | | | |
|---|---|---|---|---|---|---|
| sp\|Q8WXG9\|GPR98_HUMAN G-protein coupled receptor 98 OS=Homo sapiens GN=GPR98 PE=1 SV=2 | 394 | KPYGVLsFNSV | 0.08 | -0.113 | -1.236 | -0.423 |
| sp\|Q8WXG9\|GPR98_HUMAN G-protein coupled receptor 98 OS=Homo sapiens GN=GPR98 PE=1 SV=2 | 397 | GVLSFNsVLFE | 0.133 | -0.147 | -1.296 | -0.437 |
| sp\|Q8WXG9\|GPR98_HUMAN G-protein coupled receptor 98 OS=Homo sapiens GN=GPR98 PE=1 SV=2 | 403 | SVLFERtVIID | 0.115 | -0.087 | -1.138 | -0.37 |
| sp\|Q8WXG9\|GPR98_HUMAN G-protein coupled receptor 98 OS=Homo sapiens GN=GPR98 PE=1 SV=2 | 412 | IDEDRIsRYEE | 0.293 | -0.086 | -0.758 | -0.184 |
| sp\|Q8WXG9\|GPR98_HUMAN G-protein coupled receptor 98 OS=Homo sapiens GN=GPR98 PE=1 SV=2 | 418 | SRYEEItVVRN | 0.097 | 0.015 | -1.404 | -0.431 |
| sp\|Q8WXG9\|GPR98_HUMAN G-protein coupled receptor 98 OS=Homo sapiens GN=GPR98 PE=1 SV=2 | 425 | VVRNGGtHGNV | 0.208 | 0.126 | -0.908 | -0.191 |
| sp\|Q8WXG9\|GPR98_HUMAN G-protein coupled receptor 98 OS=Homo sapiens GN=GPR98 PE=1 SV=2 | 430 | GTHGNVsANWV | 0.375 | 0.036 | -0.676 | -0.088 |
| sp\|Q8WXG9\|GPR98_HUMAN G-protein coupled receptor 98 OS=Homo sapiens GN=GPR98 PE=1 SV=2 | 436 | SANWVLtRNST | 0.053 | -0.393 | -1.659 | -0.666 |
| sp\|Q8WXG9\|GPR98_HUMAN G-protein coupled receptor 98 OS=Homo sapiens GN=GPR98 PE=1 SV=2 | 439 | WVLTRNsTDPS | 0.287 | 0.056 | -0.572 | -0.076 |
| sp\|Q8WXG9\|GPR98_HUMAN G-protein coupled receptor 98 OS=Homo sapiens GN=GPR98 PE=1 SV=2 | 440 | VLTRNStDPSP | 0.811 | 1.266 | 0.856 | 0.978 |
| sp\|Q8WXG9\|GPR98_HUMAN G-protein coupled receptor 98 OS=Homo sapiens GN=GPR98 PE=1 SV=2 | 443 | RNSTDPsPVTA | 0.054 | -0.58 | -1.556 | -0.694 |
| sp\|Q8WXG9\|GPR98_HUMAN G-protein coupled receptor 98 OS=Homo sapiens GN=GPR98 PE=1 SV=2 | 446 | TDPSPVtADIR | 0.137 | -0.27 | -1.061 | -0.398 |
| sp\|Q8WXG9\|GPR98_HUMAN G-protein coupled receptor 98 OS=Homo sapiens GN=GPR98 PE=1 SV=2 | 452 | TADIRPsSGVL | 0.211 | 0.035 | -0.813 | -0.189 |
| sp\|Q8WXG9\|GPR98_HUMAN G-protein coupled receptor 98 OS=Homo sapiens GN=GPR98 PE=1 SV=2 | 453 | ADIRPSsGVLH | 0.13 | 0.425 | -0.767 | -0.071 |
| sp\|Q8WXG9\|GPR98_HUMAN G-protein coupled receptor 98 OS=Homo sapiens GN=GPR98 PE=1 SV=2 | 466 | QGQMLAtIPLT | 0.392 | 0.348 | -0.164 | 0.192 |
| sp\|Q8WXG9\|GPR98_HUMAN G-protein coupled receptor 98 OS=Homo sapiens GN=GPR98 PE=1 SV=2 | 470 | LATIPLtVVDD | 0.135 | -0.096 | -0.718 | -0.226 |
| sp\|Q8WXG9\|GPR98_HUMAN G-protein coupled receptor 98 OS=Homo sapiens GN=GPR98 PE=1 SV=2 | 491 | LQILPHtIRGG | 0.139 | -0.134 | -0.684 | -0.226 |
| sp\|Q8WXG9\|GPR98_HUMAN G-protein coupled receptor 98 OS=Homo sapiens GN=GPR98 PE=1 SV=2 | 499 | RGGAEVsEPAE | 0.72 | 0.509 | 0.234 | 0.488 |
| sp\|Q8WXG9\|GPR98_HUMAN G-protein coupled receptor 98 OS=Homo sapiens GN=GPR98 PE=1 SV=2 | 511 | LFYIQDsDDVY | 0.194 | 0.038 | -0.931 | -0.233 |
| sp\|Q8WXG9\|GPR98_HUMAN G-protein coupled receptor 98 OS=Homo sapiens GN=GPR98 PE=1 SV=2 | 519 | DVYGLItFFPM | 0.152 | -0.087 | -0.769 | -0.235 |
| sp\|Q8WXG9\|GPR98_HUMAN G-protein coupled receptor 98 OS=Homo sapiens GN=GPR98 PE=1 SV=2 | 530 | ENQKIEsSPGE | 0.472 | 0.306 | -0.087 | 0.23 |
| sp\|Q8WXG9\|GPR98_HUMAN G-protein coupled receptor 98 OS=Homo sapiens GN=GPR98 PE=1 SV=2 | 531 | NQKIESsPGER | 0.098 | -0.323 | -1.078 | -0.434 |
| sp\|Q8WXG9\|GPR98_HUMAN G-protein coupled receptor 98 OS=Homo sapiens GN=GPR98 PE=1 SV=2 | 538 | PGERYLsLSFT | 0.485 | 0.711 | 0.259 | 0.485 |
| sp\|Q8WXG9\|GPR98_HUMAN G-protein coupled receptor 98 OS=Homo sapiens GN=GPR98 PE=1 SV=2 | 540 | ERYLSLsFTRL | 0.359 | 0.372 | -0.303 | 0.143 |
| sp\|Q8WXG9\|GPR98_HUMAN G-protein coupled receptor 98 OS=Homo sapiens GN=GPR98 PE=1 SV=2 | 542 | YLSLSFtRLGG | 0.34 | 0.149 | -0.327 | 0.054 |
| sp\|Q8WXG9\|GPR98_HUMAN G-protein coupled receptor 98 OS=Homo sapiens GN=GPR98 PE=1 SV=2 | 547 | FTRLGGtKGDV | 0.451 | 0.228 | -0.595 | 0.028 |
| sp\|Q8WXG9\|GPR98_HUMAN G-protein coupled receptor 98 OS=Homo sapiens GN=GPR98 PE=1 SV=2 | 556 | DVRLLYsVLYI | 0.298 | 0.247 | -0.584 | -0.013 |
| sp\|Q8WXG9\|GPR98_HUMAN G-protein coupled receptor 98 OS=Homo sapiens GN=GPR98 PE=1 SV=2 | 578 | EGILNIsRRND | 0.198 | -0.129 | -0.865 | -0.265 |
| sp\|Q8WXG9\|GPR98_HUMAN G-protein coupled receptor 98 OS=Homo sapiens GN=GPR98 PE=1 SV=2 | 590 | IFPEQKtQVTT | 0.083 | -0.182 | -1.18 | -0.426 |
| sp\|Q8WXG9\|GPR98_HUMAN G-protein coupled receptor 98 OS=Homo sapiens GN=GPR98 PE=1 SV=2 | 593 | EQKTQVtTKLP | 0.104 | -0.147 | -0.952 | -0.332 |
| sp\|Q8WXG9\|GPR98_HUMAN G-protein coupled receptor 98 OS=Homo sapiens GN=GPR98 PE=1 SV=2 | 594 | QKTQVTtKLPI | 0.343 | -0.001 | -0.21 | 0.044 |
| sp\|Q8WXG9\|GPR98_HUMAN G-protein coupled receptor 98 OS=Homo sapiens GN=GPR98 PE=1 SV=2 | 616 | FLVQLEtVELL | 0.23 | 0.115 | -0.714 | -0.123 |
| sp\|Q8WXG9\|GPR98_HUMAN G-protein coupled receptor 98 OS=Homo sapiens GN=GPR98 PE=1 SV=2 | 630 | PLIPPIsPRFG | 0.092 | -0.452 | -1.135 | -0.498 |
| sp\|Q8WXG9\|GPR98_HUMAN G-protein coupled receptor 98 OS=Homo sapiens GN=GPR98 PE=1 SV=2 | 640 | GEICNIsLLVT | 0.366 | 0.042 | -0.616 | -0.069 |
| sp\|Q8WXG9\|GPR98_HUMAN G-protein coupled receptor 98 OS=Homo sapiens GN=GPR98 PE=1 SV=2 | 644 | NISLLVtPAIA | 0.125 | -0.401 | -1.273 | -0.516 |
| sp\|Q8WXG9\|GPR98_HUMAN G-protein coupled receptor 98 OS=Homo sapiens GN=GPR98 PE=1 SV=2 | 656 | GEIGFLsNLPI | 0.084 | -0.136 | -1.421 | -0.491 |
| sp\|Q8WXG9\|GPR98_HUMAN G-protein coupled receptor 98 OS=Homo sapiens GN=GPR98 PE=1 SV=2 | 682 | PLHRDGtDGQA | 0.715 | 0.843 | 0.364 | 0.641 |
| sp\|Q8WXG9\|GPR98_HUMAN G-protein coupled receptor 98 OS=Homo sapiens GN=GPR98 PE=1 SV=2 | 687 | GTDGQAtVYWS | 0.153 | -0.017 | -1.056 | -0.307 |
| sp\|Q8WXG9\|GPR98_HUMAN G-protein coupled receptor 98 OS=Homo sapiens GN=GPR98 PE=1 SV=2 | 691 | QATVYWsLKPS | 0.152 | -0.154 | -0.783 | -0.262 |
| sp\|Q8WXG9\|GPR98_HUMAN G-protein coupled receptor 98 OS=Homo sapiens GN=GPR98 PE=1 SV=2 | 695 | YWSLKPsGFNS | 0.077 | -0.168 | -1.301 | -0.464 |
| sp\|Q8WXG9\|GPR98_HUMAN G-protein coupled receptor 98 OS=Homo sapiens GN=GPR98 PE=1 SV=2 | 699 | KPSGFNsKAVT | 0.103 | -0.202 | -1.242 | -0.447 |
| sp\|Q8WXG9\|GPR98_HUMAN G-protein coupled receptor 98 OS=Homo sapiens GN=GPR98 PE=1 SV=2 | 703 | FNSKAVtPDDI | 0.592 | -0.058 | 0.003 | 0.179 |
| sp\|Q8WXG9\|GPR98_HUMAN G-protein coupled receptor 98 OS=Homo sapiens GN=GPR98 PE=1 SV=2 | 713 | IGPFNGsVLFL | 0.086 | -0.224 | -1.172 | -0.437 |
| sp\|Q8WXG9\|GPR98_HUMAN G-protein coupled receptor 98 OS=Homo sapiens GN=GPR98 PE=1 SV=2 | 718 | GSVLFLsGQSD | 0.093 | -0.188 | -1.275 | -0.457 |
| sp\|Q8WXG9\|GPR98_HUMAN G-protein coupled receptor 98 OS=Homo sapiens GN=GPR98 PE=1 SV=2 | 721 | LFLSGQsDTTI | 0.227 | -0.08 | -0.586 | -0.146 |
| sp\|Q8WXG9\|GPR98_HUMAN G-protein coupled receptor 98 OS=Homo sapiens GN=GPR98 PE=1 SV=2 | 723 | LSGQSDtTINI | 0.287 | 0.094 | -0.419 | -0.013 |
| sp\|Q8WXG9\|GPR98_HUMAN G-protein coupled receptor 98 OS=Homo sapiens GN=GPR98 PE=1 SV=2 | 724 | SGQSDTtINIT | 0.104 | -0.226 | -1.004 | -0.375 |
| sp\|Q8WXG9\|GPR98_HUMAN G-protein coupled receptor 98 OS=Homo sapiens GN=GPR98 PE=1 SV=2 | 728 | DTTINItIKGD | 0.274 | -0.136 | -0.557 | -0.14 |
| sp\|Q8WXG9\|GPR98_HUMAN G-protein coupled receptor 98 OS=Homo sapiens GN=GPR98 PE=1 SV=2 | 740 | IPEMNEtVTLS | 0.214 | -0.201 | -0.892 | -0.293 |

| | | | | | | |
|---|---|---|---|---|---|---|
| sp\|Q8WXG9\|GPR98_HUMAN G-protein coupled receptor 98 OS=Homo sapiens GN=GPR98 PE=1 SV=2 | 742 | EMNETVtLSLD | 0.141 | 0.13 | -0.771 | -0.167 |
| sp\|Q8WXG9\|GPR98_HUMAN G-protein coupled receptor 98 OS=Homo sapiens GN=GPR98 PE=1 SV=2 | 744 | NETVTLsLDRV | 0.149 | -0.027 | -1.064 | -0.314 |
| sp\|Q8WXG9\|GPR98_HUMAN G-protein coupled receptor 98 OS=Homo sapiens GN=GPR98 PE=1 SV=2 | 757 | ENQVLKsGYTS | 0.056 | -0.191 | -1.156 | -0.43 |
| sp\|Q8WXG9\|GPR98_HUMAN G-protein coupled receptor 98 OS=Homo sapiens GN=GPR98 PE=1 SV=2 | 760 | VLKSGYtSRDL | 0.275 | 0.019 | -0.393 | -0.033 |
| sp\|Q8WXG9\|GPR98_HUMAN G-protein coupled receptor 98 OS=Homo sapiens GN=GPR98 PE=1 SV=2 | 761 | LKSGYTsRDLI | 0.387 | 0.046 | -0.186 | 0.082 |
| sp\|Q8WXG9\|GPR98_HUMAN G-protein coupled receptor 98 OS=Homo sapiens GN=GPR98 PE=1 SV=2 | 779 | GGVFEFsPASR | 0.068 | -0.374 | -1.445 | -0.584 |
| sp\|Q8WXG9\|GPR98_HUMAN G-protein coupled receptor 98 OS=Homo sapiens GN=GPR98 PE=1 SV=2 | 782 | FEFSPAsRGPY | 0.23 | -0.126 | -0.98 | -0.292 |
| sp\|Q8WXG9\|GPR98_HUMAN G-protein coupled receptor 98 OS=Homo sapiens GN=GPR98 PE=1 SV=2 | 793 | VIKEGEsVELH | 0.062 | -0.197 | -1.487 | -0.541 |
| sp\|Q8WXG9\|GPR98_HUMAN G-protein coupled receptor 98 OS=Homo sapiens GN=GPR98 PE=1 SV=2 | 801 | ELHIIRsRGSL | 0.078 | -0.037 | -1.097 | -0.352 |
| sp\|Q8WXG9\|GPR98_HUMAN G-protein coupled receptor 98 OS=Homo sapiens GN=GPR98 PE=1 SV=2 | 804 | IIRSRGsLVKQ | 0.542 | 0.321 | -0.148 | 0.238 |
| sp\|Q8WXG9\|GPR98_HUMAN G-protein coupled receptor 98 OS=Homo sapiens GN=GPR98 PE=1 SV=2 | 819 | RVEPRDsNEFY | 0.184 | -0.079 | -1.145 | -0.347 |
| sp\|Q8WXG9\|GPR98_HUMAN G-protein coupled receptor 98 OS=Homo sapiens GN=GPR98 PE=1 SV=2 | 826 | NEFYGNtGVLE | 0.127 | -0.098 | -1.113 | -0.361 |
| sp\|Q8WXG9\|GPR98_HUMAN G-protein coupled receptor 98 OS=Homo sapiens GN=GPR98 PE=1 SV=2 | 841 | EREIVItLLAR | 0.398 | 0.313 | -0.125 | 0.195 |
| sp\|Q8WXG9\|GPR98_HUMAN G-protein coupled receptor 98 OS=Homo sapiens GN=GPR98 PE=1 SV=2 | 861 | HYWVVLsSHGE | 0.094 | 0.004 | -1.096 | -0.333 |
| sp\|Q8WXG9\|GPR98_HUMAN G-protein coupled receptor 98 OS=Homo sapiens GN=GPR98 PE=1 SV=2 | 862 | YWVVLSsHGER | 0.128 | -0.05 | -0.998 | -0.307 |
| sp\|Q8WXG9\|GPR98_HUMAN G-protein coupled receptor 98 OS=Homo sapiens GN=GPR98 PE=1 SV=2 | 868 | SHGEREsKLGS | 0.058 | -0.16 | -1.79 | -0.631 |
| sp\|Q8WXG9\|GPR98_HUMAN G-protein coupled receptor 98 OS=Homo sapiens GN=GPR98 PE=1 SV=2 | 872 | RESKLGsATIV | 0.255 | -0.099 | -1.378 | -0.407 |
| sp\|Q8WXG9\|GPR98_HUMAN G-protein coupled receptor 98 OS=Homo sapiens GN=GPR98 PE=1 SV=2 | 874 | SKLGSAtIVNI | 0.466 | 0.289 | -0.11 | 0.215 |
| sp\|Q8WXG9\|GPR98_HUMAN G-protein coupled receptor 98 OS=Homo sapiens GN=GPR98 PE=1 SV=2 | 879 | ATIVNItILKN | 0.082 | -0.186 | -1.363 | -0.489 |
| sp\|Q8WXG9\|GPR98_HUMAN G-protein coupled receptor 98 OS=Homo sapiens GN=GPR98 PE=1 SV=2 | 894 | GIIEFVsDGLI | 0.525 | 0.235 | -0.354 | 0.135 |
| sp\|Q8WXG9\|GPR98_HUMAN G-protein coupled receptor 98 OS=Homo sapiens GN=GPR98 PE=1 SV=2 | 904 | IVMINEsKGDA | 0.34 | 0.048 | -0.313 | 0.025 |
| sp\|Q8WXG9\|GPR98_HUMAN G-protein coupled receptor 98 OS=Homo sapiens GN=GPR98 PE=1 SV=2 | 911 | KGDAIYsAVYD | 0.167 | -0.046 | -0.751 | -0.21 |
| sp\|Q8WXG9\|GPR98_HUMAN G-protein coupled receptor 98 OS=Homo sapiens GN=GPR98 PE=1 SV=2 | 927 | GNFGDVsVSWV | 0.18 | -0.126 | -0.982 | -0.309 |
| sp\|Q8WXG9\|GPR98_HUMAN G-protein coupled receptor 98 OS=Homo sapiens GN=GPR98 PE=1 SV=2 | 929 | FGDVSVsWVVS | 0.291 | 0.281 | -0.437 | 0.045 |
| sp\|Q8WXG9\|GPR98_HUMAN G-protein coupled receptor 98 OS=Homo sapiens GN=GPR98 PE=1 SV=2 | 933 | SVSWVVsPDFT | 0.079 | -0.521 | -1.369 | -0.604 |
| sp\|Q8WXG9\|GPR98_HUMAN G-protein coupled receptor 98 OS=Homo sapiens GN=GPR98 PE=1 SV=2 | 937 | VVSPDFtQDVF | 0.085 | -0.16 | -1.139 | -0.405 |
| sp\|Q8WXG9\|GPR98_HUMAN G-protein coupled receptor 98 OS=Homo sapiens GN=GPR98 PE=1 SV=2 | 946 | VFPVQGtVVFG | 0.038 | -0.285 | -1.687 | -0.645 |
| sp\|Q8WXG9\|GPR98_HUMAN G-protein coupled receptor 98 OS=Homo sapiens GN=GPR98 PE=1 SV=2 | 955 | FGDQEFsKNIT | 0.27 | -0.03 | -0.459 | -0.073 |
| sp\|Q8WXG9\|GPR98_HUMAN G-protein coupled receptor 98 OS=Homo sapiens GN=GPR98 PE=1 SV=2 | 959 | EFSKNItIYSL | 0.182 | -0.042 | -0.493 | -0.118 |
| sp\|Q8WXG9\|GPR98_HUMAN G-protein coupled receptor 98 OS=Homo sapiens GN=GPR98 PE=1 SV=2 | 962 | KNITIYsLPDE | 0.702 | 0.548 | 0.514 | 0.588 |
| sp\|Q8WXG9\|GPR98_HUMAN G-protein coupled receptor 98 OS=Homo sapiens GN=GPR98 PE=1 SV=2 | 975 | EEMEEFtVILL | 0.071 | 0.015 | -1.08 | -0.331 |
| sp\|Q8WXG9\|GPR98_HUMAN G-protein coupled receptor 98 OS=Homo sapiens GN=GPR98 PE=1 SV=2 | 982 | VILLNGtGGAK | 0.214 | -0.061 | -0.595 | -0.147 |
| sp\|Q8WXG9\|GPR98_HUMAN G-protein coupled receptor 98 OS=Homo sapiens GN=GPR98 PE=1 SV=2 | 991 | AKVGNRtTATL | 0.167 | -0.078 | -0.927 | -0.279 |
| sp\|Q8WXG9\|GPR98_HUMAN G-protein coupled receptor 98 OS=Homo sapiens GN=GPR98 PE=1 SV=2 | 992 | KVGNRTtATLR | 0.223 | 0.109 | -0.656 | -0.108 |
| sp\|Q8WXG9\|GPR98_HUMAN G-protein coupled receptor 98 OS=Homo sapiens GN=GPR98 PE=1 SV=2 | 994 | GNRTTAtLRIR | 0.656 | 0.259 | 0.028 | 0.314 |
| sp\|Q8WXG9\|GPR98_HUMAN G-protein coupled receptor 98 OS=Homo sapiens GN=GPR98 PE=1 SV=2 | 1019 | RVQEGEtANFT | 0.058 | -0.132 | -1.467 | -0.514 |
| sp\|Q8WXG9\|GPR98_HUMAN G-protein coupled receptor 98 OS=Homo sapiens GN=GPR98 PE=1 SV=2 | 1023 | GETANFtVLRN | 0.231 | -0.069 | -0.898 | -0.245 |
| sp\|Q8WXG9\|GPR98_HUMAN G-protein coupled receptor 98 OS=Homo sapiens GN=GPR98 PE=1 SV=2 | 1029 | TVLRNGsVDVT | 0.468 | 0.768 | 0.212 | 0.483 |
| sp\|Q8WXG9\|GPR98_HUMAN G-protein coupled receptor 98 OS=Homo sapiens GN=GPR98 PE=1 SV=2 | 1033 | NGSVDVtCMVQ | 0.172 | -0.077 | -0.974 | -0.293 |
| sp\|Q8WXG9\|GPR98_HUMAN G-protein coupled receptor 98 OS=Homo sapiens GN=GPR98 PE=1 SV=2 | 1040 | CMVQYAtKDGK | 0.368 | 0.101 | -0.206 | 0.088 |
| sp\|Q8WXG9\|GPR98_HUMAN G-protein coupled receptor 98 OS=Homo sapiens GN=GPR98 PE=1 SV=2 | 1046 | TKDGKAtARER | 0.293 | -0.033 | -0.698 | -0.146 |
| sp\|Q8WXG9\|GPR98_HUMAN G-protein coupled receptor 98 OS=Homo sapiens GN=GPR98 PE=1 SV=2 | 1060 | PVEKGEtLIFE | 0.189 | -0.099 | -0.702 | -0.204 |
| sp\|Q8WXG9\|GPR98_HUMAN G-protein coupled receptor 98 OS=Homo sapiens GN=GPR98 PE=1 SV=2 | 1067 | LIFEVGsRQQS | 0.094 | -0.185 | -1.326 | -0.472 |
| sp\|Q8WXG9\|GPR98_HUMAN G-protein coupled receptor 98 OS=Homo sapiens GN=GPR98 PE=1 SV=2 | 1071 | VGSRQQsISIF | 0.261 | 0.621 | -0.202 | 0.227 |
| sp\|Q8WXG9\|GPR98_HUMAN G-protein coupled receptor 98 OS=Homo sapiens GN=GPR98 PE=1 SV=2 | 1073 | SRQQSIsIFVN | 0.437 | 0.426 | -0.09 | 0.258 |
| sp\|Q8WXG9\|GPR98_HUMAN G-protein coupled receptor 98 OS=Homo sapiens GN=GPR98 PE=1 SV=2 | 1084 | EDGIPEtDEPF | 0.03 | -0.344 | -1.703 | -0.672 |
| sp\|Q8WXG9\|GPR98_HUMAN G-protein coupled receptor 98 OS=Homo sapiens GN=GPR98 PE=1 SV=2 | 1095 | YIILLNsTGDT | 0.315 | 0.119 | -0.656 | -0.074 |
| sp\|Q8WXG9\|GPR98_HUMAN G-protein coupled receptor 98 OS=Homo sapiens GN=GPR98 PE=1 SV=2 | 1096 | IILLNStGDTV | 0.21 | -0.075 | -0.795 | -0.22 |
| sp\|Q8WXG9\|GPR98_HUMAN G-protein coupled receptor 98 OS=Homo sapiens GN=GPR98 PE=1 SV=2 | 1099 | LNSTGDtVVYQ | 0.051 | -0.405 | -1.658 | -0.671 |

| | | | | | | |
|---|---|---|---|---|---|---|
| sp\|Q8WXG9\|GPR98_HUMAN G-protein coupled receptor 98 OS=Homo sapiens GN=GPR98 PE=1 SV=2 | 1108 | YQYGVAtVIIE | 0.145 | -0.052 | -0.686 | -0.198 |
| sp\|Q8WXG9\|GPR98_HUMAN G-protein coupled receptor 98 OS=Homo sapiens GN=GPR98 PE=1 SV=2 | 1122 | DPNGIFsLEPI | 0.292 | 0.024 | -0.379 | -0.021 |
| sp\|Q8WXG9\|GPR98_HUMAN G-protein coupled receptor 98 OS=Homo sapiens GN=GPR98 PE=1 SV=2 | 1135 | AVEEGKtNAFW | 0.042 | -0.274 | -1.542 | -0.591 |
| sp\|Q8WXG9\|GPR98_HUMAN G-protein coupled receptor 98 OS=Homo sapiens GN=GPR98 PE=1 SV=2 | 1149 | HRGYFGsVSVS | 0.07 | 0.113 | -1.155 | -0.324 |
| sp\|Q8WXG9\|GPR98_HUMAN G-protein coupled receptor 98 OS=Homo sapiens GN=GPR98 PE=1 SV=2 | 1151 | GYFGSVsVSWQ | 0.466 | 0.218 | -0.186 | 0.166 |
| sp\|Q8WXG9\|GPR98_HUMAN G-protein coupled receptor 98 OS=Homo sapiens GN=GPR98 PE=1 SV=2 | 1153 | FGSVSVsWQLF | 0.362 | 0.288 | -0.141 | 0.17 |
| sp\|Q8WXG9\|GPR98_HUMAN G-protein coupled receptor 98 OS=Homo sapiens GN=GPR98 PE=1 SV=2 | 1161 | QLFQNDsALQP | 0.205 | -0.096 | -0.869 | -0.253 |
| sp\|Q8WXG9\|GPR98_HUMAN G-protein coupled receptor 98 OS=Homo sapiens GN=GPR98 PE=1 SV=2 | 1172 | GQEFYEtSGTV | 0.288 | -0.05 | -0.905 | -0.222 |
| sp\|Q8WXG9\|GPR98_HUMAN G-protein coupled receptor 98 OS=Homo sapiens GN=GPR98 PE=1 SV=2 | 1173 | QEFYETsGTVN | 0.164 | -0.088 | -1.057 | -0.327 |
| sp\|Q8WXG9\|GPR98_HUMAN G-protein coupled receptor 98 OS=Homo sapiens GN=GPR98 PE=1 SV=2 | 1175 | FYETSGtVNFM | 0.34 | 0.151 | -0.338 | 0.051 |
| sp\|Q8WXG9\|GPR98_HUMAN G-protein coupled receptor 98 OS=Homo sapiens GN=GPR98 PE=1 SV=2 | 1211 | LKLVNIsGGSP | 0.147 | -0.115 | -0.685 | -0.218 |
| sp\|Q8WXG9\|GPR98_HUMAN G-protein coupled receptor 98 OS=Homo sapiens GN=GPR98 PE=1 SV=2 | 1214 | VNISGGsPGPG | 0.027 | -0.64 | -1.833 | -0.815 |
| sp\|Q8WXG9\|GPR98_HUMAN G-protein coupled receptor 98 OS=Homo sapiens GN=GPR98 PE=1 SV=2 | 1224 | GGQLAEtNLQV | 0.236 | -0.025 | -1.205 | -0.331 |
| sp\|Q8WXG9\|GPR98_HUMAN G-protein coupled receptor 98 OS=Homo sapiens GN=GPR98 PE=1 SV=2 | 1229 | ETNLQVtVMVP | 0.124 | -0.111 | -1.273 | -0.42 |
| sp\|Q8WXG9\|GPR98_HUMAN G-protein coupled receptor 98 OS=Homo sapiens GN=GPR98 PE=1 SV=2 | 1259 | VAEDVLsEDDM | 0.263 | -0.014 | -0.68 | -0.144 |
| sp\|Q8WXG9\|GPR98_HUMAN G-protein coupled receptor 98 OS=Homo sapiens GN=GPR98 PE=1 SV=2 | 1264 | LSEDDMsYITN | 0.14 | -0.075 | -0.844 | -0.26 |
| sp\|Q8WXG9\|GPR98_HUMAN G-protein coupled receptor 98 OS=Homo sapiens GN=GPR98 PE=1 SV=2 | 1267 | DDMSYItNFTI | 0.2 | -0.149 | -0.921 | -0.29 |
| sp\|Q8WXG9\|GPR98_HUMAN G-protein coupled receptor 98 OS=Homo sapiens GN=GPR98 PE=1 SV=2 | 1270 | SYITNFtILRQ | 0.097 | -0.203 | -1.393 | -0.5 |
| sp\|Q8WXG9\|GPR98_HUMAN G-protein coupled receptor 98 OS=Homo sapiens GN=GPR98 PE=1 SV=2 | 1289 | LGWEILsSEFP | 0.025 | -0.21 | -1.56 | -0.582 |
| sp\|Q8WXG9\|GPR98_HUMAN G-protein coupled receptor 98 OS=Homo sapiens GN=GPR98 PE=1 SV=2 | 1290 | GWEILSsEFPA | 0.13 | -0.069 | -0.941 | -0.293 |
| sp\|Q8WXG9\|GPR98_HUMAN G-protein coupled receptor 98 OS=Homo sapiens GN=GPR98 PE=1 SV=2 | 1310 | LVGIFPtTVHL | 0.052 | -0.172 | -1.285 | -0.468 |
| sp\|Q8WXG9\|GPR98_HUMAN G-protein coupled receptor 98 OS=Homo sapiens GN=GPR98 PE=1 SV=2 | 1311 | VGIFPTtVHLQ | 0.16 | -0.119 | -0.851 | -0.27 |
| sp\|Q8WXG9\|GPR98_HUMAN G-protein coupled receptor 98 OS=Homo sapiens GN=GPR98 PE=1 SV=2 | 1323 | HMRRHHsGTDA | 0.901 | 1.414 | 1.198 | 1.171 |
| sp\|Q8WXG9\|GPR98_HUMAN G-protein coupled receptor 98 OS=Homo sapiens GN=GPR98 PE=1 SV=2 | 1325 | RRHHSGtDALY | 0.328 | 0.339 | -0.621 | 0.015 |
| sp\|Q8WXG9\|GPR98_HUMAN G-protein coupled receptor 98 OS=Homo sapiens GN=GPR98 PE=1 SV=2 | 1331 | TDALYFtGLEG | 0.096 | -0.163 | -1.056 | -0.374 |
| sp\|Q8WXG9\|GPR98_HUMAN G-protein coupled receptor 98 OS=Homo sapiens GN=GPR98 PE=1 SV=2 | 1339 | LEGAFGtVNPK | 0.078 | -0.289 | -1.304 | -0.505 |
| sp\|Q8WXG9\|GPR98_HUMAN G-protein coupled receptor 98 OS=Homo sapiens GN=GPR98 PE=1 SV=2 | 1347 | NPKYHPsRNNT | 0.232 | -0.077 | -0.738 | -0.194 |
| sp\|Q8WXG9\|GPR98_HUMAN G-protein coupled receptor 98 OS=Homo sapiens GN=GPR98 PE=1 SV=2 | 1351 | HPSRNNtIANF | 0.47 | 0.799 | 0.069 | 0.446 |
| sp\|Q8WXG9\|GPR98_HUMAN G-protein coupled receptor 98 OS=Homo sapiens GN=GPR98 PE=1 SV=2 | 1356 | NTIANFtFSAW | 0.275 | 0.076 | -0.461 | -0.037 |
| sp\|Q8WXG9\|GPR98_HUMAN G-protein coupled receptor 98 OS=Homo sapiens GN=GPR98 PE=1 SV=2 | 1358 | IANFTFsAWVM | 0.253 | 0.099 | -0.77 | -0.139 |
| sp\|Q8WXG9\|GPR98_HUMAN G-protein coupled receptor 98 OS=Homo sapiens GN=GPR98 PE=1 SV=2 | 1367 | VMPNANtNGFI | 0.202 | 0.081 | -0.758 | -0.158 |
| sp\|Q8WXG9\|GPR98_HUMAN G-protein coupled receptor 98 OS=Homo sapiens GN=GPR98 PE=1 SV=2 | 1380 | KDDGNGsIYYG | 0.05 | -0.351 | -1.502 | -0.601 |
| sp\|Q8WXG9\|GPR98_HUMAN G-protein coupled receptor 98 OS=Homo sapiens GN=GPR98 PE=1 SV=2 | 1389 | YGVKIQtNESH | 0.093 | -0.198 | -1.162 | -0.422 |
| sp\|Q8WXG9\|GPR98_HUMAN G-protein coupled receptor 98 OS=Homo sapiens GN=GPR98 PE=1 SV=2 | 1392 | KIQTNEsHVTL | 0.249 | 0.017 | -0.825 | -0.186 |
| sp\|Q8WXG9\|GPR98_HUMAN G-protein coupled receptor 98 OS=Homo sapiens GN=GPR98 PE=1 SV=2 | 1395 | TNESHVtLSLH | 0.209 | -0.235 | -0.874 | -0.3 |
| sp\|Q8WXG9\|GPR98_HUMAN G-protein coupled receptor 98 OS=Homo sapiens GN=GPR98 PE=1 SV=2 | 1397 | ESHVTLsLHYK | 0.061 | -0.163 | -1.245 | -0.449 |
| sp\|Q8WXG9\|GPR98_HUMAN G-protein coupled receptor 98 OS=Homo sapiens GN=GPR98 PE=1 SV=2 | 1402 | LSLHYKtLGSN | 0.389 | 0.068 | -0.076 | 0.127 |
| sp\|Q8WXG9\|GPR98_HUMAN G-protein coupled receptor 98 OS=Homo sapiens GN=GPR98 PE=1 SV=2 | 1405 | HYKTLGsNATY | 0.139 | -0.131 | -1.231 | -0.408 |
| sp\|Q8WXG9\|GPR98_HUMAN G-protein coupled receptor 98 OS=Homo sapiens GN=GPR98 PE=1 SV=2 | 1408 | TLGSNAtYIAK | 0.192 | -0.045 | -0.578 | -0.144 |
| sp\|Q8WXG9\|GPR98_HUMAN G-protein coupled receptor 98 OS=Homo sapiens GN=GPR98 PE=1 SV=2 | 1413 | ATYIAKtTVMK | 0.225 | -0.077 | -0.802 | -0.218 |
| sp\|Q8WXG9\|GPR98_HUMAN G-protein coupled receptor 98 OS=Homo sapiens GN=GPR98 PE=1 SV=2 | 1414 | TYIAKTtVMKY | 0.164 | -0.052 | -0.978 | -0.289 |
| sp\|Q8WXG9\|GPR98_HUMAN G-protein coupled receptor 98 OS=Homo sapiens GN=GPR98 PE=1 SV=2 | 1422 | MKYLEEsVWLH | 0.285 | -0.004 | -1.007 | -0.242 |
| sp\|Q8WXG9\|GPR98_HUMAN G-protein coupled receptor 98 OS=Homo sapiens GN=GPR98 PE=1 SV=2 | 1451 | MPRGIKsLKGE | 0.627 | 0.314 | 0.454 | 0.465 |
| sp\|Q8WXG9\|GPR98_HUMAN G-protein coupled receptor 98 OS=Homo sapiens GN=GPR98 PE=1 SV=2 | 1458 | LKGEAItDGPG | 0.366 | 0.094 | -0.424 | 0.012 |
| sp\|Q8WXG9\|GPR98_HUMAN G-protein coupled receptor 98 OS=Homo sapiens GN=GPR98 PE=1 SV=2 | 1477 | NGNDRFtGLMQ | 0.15 | -0.056 | -1.078 | -0.328 |
| sp\|Q8WXG9\|GPR98_HUMAN G-protein coupled receptor 98 OS=Homo sapiens GN=GPR98 PE=1 SV=2 | 1485 | LMQDVRsYERK | 0.083 | -0.08 | -1.491 | -0.496 |
| sp\|Q8WXG9\|GPR98_HUMAN G-protein coupled receptor 98 OS=Homo sapiens GN=GPR98 PE=1 SV=2 | 1491 | SYERKLtLEEI | 0.661 | 0.884 | 0.473 | 0.673 |
| sp\|Q8WXG9\|GPR98_HUMAN G-protein coupled receptor 98 OS=Homo sapiens GN=GPR98 PE=1 SV=2 | 1505 | HAMPAKsDLHP | 0.133 | -0.018 | -0.728 | -0.204 |
| sp\|Q8WXG9\|GPR98_HUMAN G-protein coupled receptor 98 OS=Homo sapiens GN=GPR98 PE=1 SV=2 | 1511 | SDLHPIsGYLE | 0.06 | -0.37 | -1.343 | -0.551 |

| | | | | | | |
|---|---|---|---|---|---|---|
| sp\|Q8WXG9\|GPR98_HUMAN G-protein coupled receptor 98 OS=Homo sapiens GN=GPR98 PE=1 SV=2 | 1521 | EFRQGEtNKSF | 0.098 | -0.021 | -0.986 | -0.303 |
| sp\|Q8WXG9\|GPR98_HUMAN G-protein coupled receptor 98 OS=Homo sapiens GN=GPR98 PE=1 SV=2 | 1524 | QGETNKsFIIS | 0.319 | -0.005 | -0.192 | 0.041 |
| sp\|Q8WXG9\|GPR98_HUMAN G-protein coupled receptor 98 OS=Homo sapiens GN=GPR98 PE=1 SV=2 | 1528 | NKSFIIsARDD | 0.609 | 0.11 | 0.017 | 0.245 |
| sp\|Q8WXG9\|GPR98_HUMAN G-protein coupled receptor 98 OS=Homo sapiens GN=GPR98 PE=1 SV=2 | 1547 | FILKLVsVYGG | 0.482 | 0.129 | -0.208 | 0.134 |
| sp\|Q8WXG9\|GPR98_HUMAN G-protein coupled receptor 98 OS=Homo sapiens GN=GPR98 PE=1 SV=2 | 1555 | YGGARIsEENT | 0.243 | 0.104 | -0.591 | -0.081 |
| sp\|Q8WXG9\|GPR98_HUMAN G-protein coupled receptor 98 OS=Homo sapiens GN=GPR98 PE=1 SV=2 | 1559 | RISEENtTARL | 0.167 | -0.07 | -1.282 | -0.395 |
| sp\|Q8WXG9\|GPR98_HUMAN G-protein coupled receptor 98 OS=Homo sapiens GN=GPR98 PE=1 SV=2 | 1560 | ISEENTtARLT | 0.164 | -0.197 | -0.824 | -0.286 |
| sp\|Q8WXG9\|GPR98_HUMAN G-protein coupled receptor 98 OS=Homo sapiens GN=GPR98 PE=1 SV=2 | 1564 | NTTARLtIQKS | 0.156 | -0.057 | -0.856 | -0.252 |
| sp\|Q8WXG9\|GPR98_HUMAN G-protein coupled receptor 98 OS=Homo sapiens GN=GPR98 PE=1 SV=2 | 1568 | RLTIQKsDNAN | 0.54 | 0.335 | 0.005 | 0.293 |
| sp\|Q8WXG9\|GPR98_HUMAN G-protein coupled receptor 98 OS=Homo sapiens GN=GPR98 PE=1 SV=2 | 1578 | NGLFGFtGACI | 0.139 | -0.055 | -0.686 | -0.201 |
| sp\|Q8WXG9\|GPR98_HUMAN G-protein coupled receptor 98 OS=Homo sapiens GN=GPR98 PE=1 SV=2 | 1590 | EIAEEGsTISC | 0.028 | -0.265 | -1.774 | -0.67 |
| sp\|Q8WXG9\|GPR98_HUMAN G-protein coupled receptor 98 OS=Homo sapiens GN=GPR98 PE=1 SV=2 | 1591 | IAEEGStISCV | 0.032 | -0.328 | -1.786 | -0.694 |
| sp\|Q8WXG9\|GPR98_HUMAN G-protein coupled receptor 98 OS=Homo sapiens GN=GPR98 PE=1 SV=2 | 1593 | EEGSTIsCVVE | 0.069 | -0.063 | -1.375 | -0.456 |
| sp\|Q8WXG9\|GPR98_HUMAN G-protein coupled receptor 98 OS=Homo sapiens GN=GPR98 PE=1 SV=2 | 1599 | SCVVERtRGAL | 0.114 | -0.058 | -1.417 | -0.454 |
| sp\|Q8WXG9\|GPR98_HUMAN G-protein coupled receptor 98 OS=Homo sapiens GN=GPR98 PE=1 SV=2 | 1611 | YVHVFYtISQI | 0.066 | -0.107 | -1.013 | -0.351 |
| sp\|Q8WXG9\|GPR98_HUMAN G-protein coupled receptor 98 OS=Homo sapiens GN=GPR98 PE=1 SV=2 | 1613 | HVFYTIsQIET | 0.104 | 0.022 | -0.809 | -0.228 |
| sp\|Q8WXG9\|GPR98_HUMAN G-protein coupled receptor 98 OS=Homo sapiens GN=GPR98 PE=1 SV=2 | 1617 | TISQIEtDGIN | 0.349 | 0.039 | -0.544 | -0.052 |
| sp\|Q8WXG9\|GPR98_HUMAN G-protein coupled receptor 98 OS=Homo sapiens GN=GPR98 PE=1 SV=2 | 1631 | DDFANAsGTIT | 0.221 | -0.246 | -0.988 | -0.338 |
| sp\|Q8WXG9\|GPR98_HUMAN G-protein coupled receptor 98 OS=Homo sapiens GN=GPR98 PE=1 SV=2 | 1633 | FANASGtITFL | 0.171 | -0.005 | -0.89 | -0.241 |
| sp\|Q8WXG9\|GPR98_HUMAN G-protein coupled receptor 98 OS=Homo sapiens GN=GPR98 PE=1 SV=2 | 1635 | NASGTItFLPW | 0.111 | -0.087 | -0.985 | -0.32 |
| sp\|Q8WXG9\|GPR98_HUMAN G-protein coupled receptor 98 OS=Homo sapiens GN=GPR98 PE=1 SV=2 | 1642 | FLPWQRsEVLN | 0.197 | 0.143 | -0.9 | -0.187 |
| sp\|Q8WXG9\|GPR98_HUMAN G-protein coupled receptor 98 OS=Homo sapiens GN=GPR98 PE=1 SV=2 | 1664 | NEYFRVtLVSA | 0.442 | 0.155 | -0.25 | 0.116 |
| sp\|Q8WXG9\|GPR98_HUMAN G-protein coupled receptor 98 OS=Homo sapiens GN=GPR98 PE=1 SV=2 | 1667 | FRVTLVsAIPG | 0.247 | 0.194 | -0.743 | -0.101 |
| sp\|Q8WXG9\|GPR98_HUMAN G-protein coupled receptor 98 OS=Homo sapiens GN=GPR98 PE=1 SV=2 | 1677 | GDGKLGsTPTS | 0.274 | 0.245 | -0.719 | -0.067 |
| sp\|Q8WXG9\|GPR98_HUMAN G-protein coupled receptor 98 OS=Homo sapiens GN=GPR98 PE=1 SV=2 | 1678 | DGKLGStPTSG | 0.046 | -0.519 | -1.695 | -0.723 |
| sp\|Q8WXG9\|GPR98_HUMAN G-protein coupled receptor 98 OS=Homo sapiens GN=GPR98 PE=1 SV=2 | 1680 | KLGSTPtSGAS | 0.135 | -0.033 | -0.921 | -0.273 |
| sp\|Q8WXG9\|GPR98_HUMAN G-protein coupled receptor 98 OS=Homo sapiens GN=GPR98 PE=1 SV=2 | 1681 | LGSTPTsGASI | 0.084 | -0.3 | -0.989 | -0.402 |
| sp\|Q8WXG9\|GPR98_HUMAN G-protein coupled receptor 98 OS=Homo sapiens GN=GPR98 PE=1 SV=2 | 1684 | TPTSGAsIDPE | 0.078 | -0.292 | -1.191 | -0.468 |
| sp\|Q8WXG9\|GPR98_HUMAN G-protein coupled receptor 98 OS=Homo sapiens GN=GPR98 PE=1 SV=2 | 1691 | IDPEKEtTDIT | 0.046 | -0.356 | -1.59 | -0.633 |
| sp\|Q8WXG9\|GPR98_HUMAN G-protein coupled receptor 98 OS=Homo sapiens GN=GPR98 PE=1 SV=2 | 1692 | DPEKETtDITI | 0.411 | -0.02 | -0.548 | -0.052 |
| sp\|Q8WXG9\|GPR98_HUMAN G-protein coupled receptor 98 OS=Homo sapiens GN=GPR98 PE=1 SV=2 | 1695 | KETTDItIKAS | 0.116 | -0.173 | -1.018 | -0.358 |
| sp\|Q8WXG9\|GPR98_HUMAN G-protein coupled receptor 98 OS=Homo sapiens GN=GPR98 PE=1 SV=2 | 1699 | DITIKAsDHPY | 0.35 | 0.071 | -0.818 | -0.132 |
| sp\|Q8WXG9\|GPR98_HUMAN G-protein coupled receptor 98 OS=Homo sapiens GN=GPR98 PE=1 SV=2 | 1709 | YGLLQFsTGLP | 0.124 | -0.038 | -0.874 | -0.263 |
| sp\|Q8WXG9\|GPR98_HUMAN G-protein coupled receptor 98 OS=Homo sapiens GN=GPR98 PE=1 SV=2 | 1710 | GLLQFStGLPP | 0.11 | -0.188 | -1.088 | -0.389 |
| sp\|Q8WXG9\|GPR98_HUMAN G-protein coupled receptor 98 OS=Homo sapiens GN=GPR98 PE=1 SV=2 | 1721 | QPKDAMtLPAS | 0.867 | 0.805 | 0.859 | 0.844 |
| sp\|Q8WXG9\|GPR98_HUMAN G-protein coupled receptor 98 OS=Homo sapiens GN=GPR98 PE=1 SV=2 | 1725 | AMTLPAsSVPH | 0.062 | -0.229 | -1.503 | -0.557 |
| sp\|Q8WXG9\|GPR98_HUMAN G-protein coupled receptor 98 OS=Homo sapiens GN=GPR98 PE=1 SV=2 | 1726 | MTLPASsVPHI | 0.637 | 0.491 | 0.333 | 0.487 |
| sp\|Q8WXG9\|GPR98_HUMAN G-protein coupled receptor 98 OS=Homo sapiens GN=GPR98 PE=1 SV=2 | 1731 | SSVPHItVEEE | 0.141 | -0.178 | -1.138 | -0.392 |
| sp\|Q8WXG9\|GPR98_HUMAN G-protein coupled receptor 98 OS=Homo sapiens GN=GPR98 PE=1 SV=2 | 1754 | GLLGRVtAEFR | 0.548 | 0.227 | -0.195 | 0.193 |
| sp\|Q8WXG9\|GPR98_HUMAN G-protein coupled receptor 98 OS=Homo sapiens GN=GPR98 PE=1 SV=2 | 1759 | VTAEFRtVSLT | 0.055 | -0.24 | -1.571 | -0.585 |
| sp\|Q8WXG9\|GPR98_HUMAN G-protein coupled receptor 98 OS=Homo sapiens GN=GPR98 PE=1 SV=2 | 1761 | AEFRTVsLTAF | 0.824 | 0.971 | 0.71 | 0.835 |
| sp\|Q8WXG9\|GPR98_HUMAN G-protein coupled receptor 98 OS=Homo sapiens GN=GPR98 PE=1 SV=2 | 1763 | FRTVSLtAFSP | 0.145 | 0.365 | -0.656 | -0.049 |
| sp\|Q8WXG9\|GPR98_HUMAN G-protein coupled receptor 98 OS=Homo sapiens GN=GPR98 PE=1 SV=2 | 1766 | VSLTAFsPEDY | 0.204 | -0.242 | -0.873 | -0.304 |
| sp\|Q8WXG9\|GPR98_HUMAN G-protein coupled receptor 98 OS=Homo sapiens GN=GPR98 PE=1 SV=2 | 1776 | YQNVAGtLEFQ | 0.095 | -0.132 | -1.087 | -0.375 |
| sp\|Q8WXG9\|GPR98_HUMAN G-protein coupled receptor 98 OS=Homo sapiens GN=GPR98 PE=1 SV=2 | 1793 | YIFINItDNSI | 0.333 | 0.154 | -0.298 | 0.063 |
| sp\|Q8WXG9\|GPR98_HUMAN G-protein coupled receptor 98 OS=Homo sapiens GN=GPR98 PE=1 SV=2 | 1796 | INITDNsIPEL | 0.557 | 0.404 | -0.039 | 0.307 |
| sp\|Q8WXG9\|GPR98_HUMAN G-protein coupled receptor 98 OS=Homo sapiens GN=GPR98 PE=1 SV=2 | 1803 | IPELEKsFKVE | 0.325 | -0.032 | -0.511 | -0.073 |
| sp\|Q8WXG9\|GPR98_HUMAN G-protein coupled receptor 98 OS=Homo sapiens GN=GPR98 PE=1 SV=2 | 1824 | LFRVDGsGSGD | 0.147 | -0.052 | -0.774 | -0.226 |
| sp\|Q8WXG9\|GPR98_HUMAN G-protein coupled receptor 98 OS=Homo sapiens GN=GPR98 PE=1 SV=2 | 1826 | RVDGSGsGDGD | 0.26 | 0.082 | -0.39 | -0.016 |

| | | | | | | |
|---|---|---|---|---|---|---|
| sp\|Q8WXG9\|GPR98_HUMAN G-protein coupled receptor 98 OS=Homo sapiens GN=GPR98 PE=1 SV=2 | 1837 | MEFFLPtIHKR | 0.157 | -0.084 | -1.079 | -0.335 |
| sp\|Q8WXG9\|GPR98_HUMAN G-protein coupled receptor 98 OS=Homo sapiens GN=GPR98 PE=1 SV=2 | 1843 | TIHKRAsLGVA | 0.732 | 0.372 | 0.266 | 0.457 |
| sp\|Q8WXG9\|GPR98_HUMAN G-protein coupled receptor 98 OS=Homo sapiens GN=GPR98 PE=1 SV=2 | 1848 | ASLGVAsQILV | 0.138 | -0.15 | -1.275 | -0.429 |
| sp\|Q8WXG9\|GPR98_HUMAN G-protein coupled receptor 98 OS=Homo sapiens GN=GPR98 PE=1 SV=2 | 1853 | ASQILVtIAAS | 0.128 | -0.16 | -1.045 | -0.359 |
| sp\|Q8WXG9\|GPR98_HUMAN G-protein coupled receptor 98 OS=Homo sapiens GN=GPR98 PE=1 SV=2 | 1857 | LVTIAAsDHAH | 0.315 | 0.103 | -0.589 | -0.057 |
| sp\|Q8WXG9\|GPR98_HUMAN G-protein coupled receptor 98 OS=Homo sapiens GN=GPR98 PE=1 SV=2 | 1867 | HGVFEFsPESL | 0.052 | -0.277 | -1.347 | -0.524 |
| sp\|Q8WXG9\|GPR98_HUMAN G-protein coupled receptor 98 OS=Homo sapiens GN=GPR98 PE=1 SV=2 | 1870 | FEFSPEsLFVS | 0.202 | -0.043 | -0.872 | -0.238 |
| sp\|Q8WXG9\|GPR98_HUMAN G-protein coupled receptor 98 OS=Homo sapiens GN=GPR98 PE=1 SV=2 | 1874 | PESLFVsGTEP | 0.23 | -0.193 | -1.085 | -0.349 |
| sp\|Q8WXG9\|GPR98_HUMAN G-protein coupled receptor 98 OS=Homo sapiens GN=GPR98 PE=1 SV=2 | 1876 | SLFVSGtEPED | 0.74 | 0.756 | 0.367 | 0.621 |
| sp\|Q8WXG9\|GPR98_HUMAN G-protein coupled receptor 98 OS=Homo sapiens GN=GPR98 PE=1 SV=2 | 1883 | EPEDGYsTVTL | 0.08 | -0.092 | -1.083 | -0.365 |
| sp\|Q8WXG9\|GPR98_HUMAN G-protein coupled receptor 98 OS=Homo sapiens GN=GPR98 PE=1 SV=2 | 1884 | PEDGYStVTLN | 0.14 | -0.092 | -1.103 | -0.352 |
| sp\|Q8WXG9\|GPR98_HUMAN G-protein coupled receptor 98 OS=Homo sapiens GN=GPR98 PE=1 SV=2 | 1886 | DGYSTVtLNVI | 0.504 | 0.124 | -0.028 | 0.2 |
| sp\|Q8WXG9\|GPR98_HUMAN G-protein coupled receptor 98 OS=Homo sapiens GN=GPR98 PE=1 SV=2 | 1895 | VIRHHGtLSPV | 0.376 | 0.182 | -0.575 | -0.006 |
| sp\|Q8WXG9\|GPR98_HUMAN G-protein coupled receptor 98 OS=Homo sapiens GN=GPR98 PE=1 SV=2 | 1897 | RHHGTLsPVTL | 0.025 | -0.345 | -1.91 | -0.743 |
| sp\|Q8WXG9\|GPR98_HUMAN G-protein coupled receptor 98 OS=Homo sapiens GN=GPR98 PE=1 SV=2 | 1900 | GTLSPVtLHWN | 0.516 | 0.005 | -0.232 | 0.096 |
| sp\|Q8WXG9\|GPR98_HUMAN G-protein coupled receptor 98 OS=Homo sapiens GN=GPR98 PE=1 SV=2 | 1907 | LHWNIDsDPDG | 0.525 | 0.571 | -0.105 | 0.33 |
| sp\|Q8WXG9\|GPR98_HUMAN G-protein coupled receptor 98 OS=Homo sapiens GN=GPR98 PE=1 SV=2 | 1916 | DGDLAFtSGNI | 0.432 | 0.128 | -0.491 | 0.023 |
| sp\|Q8WXG9\|GPR98_HUMAN G-protein coupled receptor 98 OS=Homo sapiens GN=GPR98 PE=1 SV=2 | 1917 | GDLAFTsGNIT | 0.158 | -0.22 | -0.879 | -0.314 |
| sp\|Q8WXG9\|GPR98_HUMAN G-protein coupled receptor 98 OS=Homo sapiens GN=GPR98 PE=1 SV=2 | 1921 | FTSGNItFEIG | 0.401 | 0.052 | -0.375 | 0.026 |
| sp\|Q8WXG9\|GPR98_HUMAN G-protein coupled receptor 98 OS=Homo sapiens GN=GPR98 PE=1 SV=2 | 1927 | TFEIGQtSANI | 0.113 | -0.095 | -0.953 | -0.312 |
| sp\|Q8WXG9\|GPR98_HUMAN G-protein coupled receptor 98 OS=Homo sapiens GN=GPR98 PE=1 SV=2 | 1928 | FEIGQTsANIT | 0.154 | 0.042 | -0.975 | -0.26 |
| sp\|Q8WXG9\|GPR98_HUMAN G-protein coupled receptor 98 OS=Homo sapiens GN=GPR98 PE=1 SV=2 | 1932 | QTSANItVEIL | 0.253 | -0.101 | -0.674 | -0.174 |
| sp\|Q8WXG9\|GPR98_HUMAN G-protein coupled receptor 98 OS=Homo sapiens GN=GPR98 PE=1 SV=2 | 1948 | ELDKAFsVSVL | 0.385 | 0.217 | -0.268 | 0.111 |
| sp\|Q8WXG9\|GPR98_HUMAN G-protein coupled receptor 98 OS=Homo sapiens GN=GPR98 PE=1 SV=2 | 1950 | DKAFSVsVLSV | 0.544 | 0.229 | -0.285 | 0.163 |
| sp\|Q8WXG9\|GPR98_HUMAN G-protein coupled receptor 98 OS=Homo sapiens GN=GPR98 PE=1 SV=2 | 1953 | FSVSVLsVSSG | 0.059 | -0.221 | -1.516 | -0.559 |
| sp\|Q8WXG9\|GPR98_HUMAN G-protein coupled receptor 98 OS=Homo sapiens GN=GPR98 PE=1 SV=2 | 1955 | VSVLSVsSGSL | 0.298 | 0.095 | -0.482 | -0.03 |
| sp\|Q8WXG9\|GPR98_HUMAN G-protein coupled receptor 98 OS=Homo sapiens GN=GPR98 PE=1 SV=2 | 1956 | SVLSVSsGSLG | 0.044 | -0.326 | -1.653 | -0.645 |
| sp\|Q8WXG9\|GPR98_HUMAN G-protein coupled receptor 98 OS=Homo sapiens GN=GPR98 PE=1 SV=2 | 1958 | LSVSSGsLGAH | 0.331 | 0.071 | -0.323 | 0.026 |
| sp\|Q8WXG9\|GPR98_HUMAN G-protein coupled receptor 98 OS=Homo sapiens GN=GPR98 PE=1 SV=2 | 1966 | GAHINAtLTVL | 0.435 | 0.12 | -0.432 | 0.041 |
| sp\|Q8WXG9\|GPR98_HUMAN G-protein coupled receptor 98 OS=Homo sapiens GN=GPR98 PE=1 SV=2 | 1968 | HINATLtVLAS | 0.083 | 0.069 | -1.021 | -0.29 |
| sp\|Q8WXG9\|GPR98_HUMAN G-protein coupled receptor 98 OS=Homo sapiens GN=GPR98 PE=1 SV=2 | 1972 | TLTVLAsDDPY | 0.3 | 0.057 | -0.729 | -0.124 |
| sp\|Q8WXG9\|GPR98_HUMAN G-protein coupled receptor 98 OS=Homo sapiens GN=GPR98 PE=1 SV=2 | 1982 | YGIFIFsEKNR | 0.24 | 0.154 | -0.391 | 0.001 |
| sp\|Q8WXG9\|GPR98_HUMAN G-protein coupled receptor 98 OS=Homo sapiens GN=GPR98 PE=1 SV=2 | 1994 | VKVEEAtQNIT | 0.213 | -0.106 | -1.04 | -0.311 |
| sp\|Q8WXG9\|GPR98_HUMAN G-protein coupled receptor 98 OS=Homo sapiens GN=GPR98 PE=1 SV=2 | 1998 | EATQNItLSII | 0.435 | 0.001 | -0.04 | 0.132 |
| sp\|Q8WXG9\|GPR98_HUMAN G-protein coupled receptor 98 OS=Homo sapiens GN=GPR98 PE=1 SV=2 | 2000 | TQNITLsIIRL | 0.034 | -0.183 | -1.373 | -0.507 |
| sp\|Q8WXG9\|GPR98_HUMAN G-protein coupled receptor 98 OS=Homo sapiens GN=GPR98 PE=1 SV=2 | 2014 | MGKVLVsYATL | 0.171 | -0.088 | -0.778 | -0.232 |
| sp\|Q8WXG9\|GPR98_HUMAN G-protein coupled receptor 98 OS=Homo sapiens GN=GPR98 PE=1 SV=2 | 2017 | VLVSYAtLDDM | 0.659 | 0.202 | 0.129 | 0.33 |
| sp\|Q8WXG9\|GPR98_HUMAN G-protein coupled receptor 98 OS=Homo sapiens GN=GPR98 PE=1 SV=2 | 2035 | PNLARAtQGRD | 0.51 | 0.003 | -0.477 | 0.012 |
| sp\|Q8WXG9\|GPR98_HUMAN G-protein coupled receptor 98 OS=Homo sapiens GN=GPR98 PE=1 SV=2 | 2044 | RDYIPAsGFAL | 0.127 | -0.056 | -0.716 | -0.215 |
| sp\|Q8WXG9\|GPR98_HUMAN G-protein coupled receptor 98 OS=Homo sapiens GN=GPR98 PE=1 SV=2 | 2054 | LFGANQsEATI | 0.238 | -0.008 | -0.338 | -0.036 |
| sp\|Q8WXG9\|GPR98_HUMAN G-protein coupled receptor 98 OS=Homo sapiens GN=GPR98 PE=1 SV=2 | 2057 | ANQSEAtIAIS | 0.093 | -0.3 | -1.411 | -0.539 |
| sp\|Q8WXG9\|GPR98_HUMAN G-protein coupled receptor 98 OS=Homo sapiens GN=GPR98 PE=1 SV=2 | 2061 | EATIAIsILDD | 0.239 | 0.011 | -0.519 | -0.09 |
| sp\|Q8WXG9\|GPR98_HUMAN G-protein coupled receptor 98 OS=Homo sapiens GN=GPR98 PE=1 SV=2 | 2071 | DDEPERsESVF | 0.059 | -0.252 | -1.637 | -0.61 |
| sp\|Q8WXG9\|GPR98_HUMAN G-protein coupled receptor 98 OS=Homo sapiens GN=GPR98 PE=1 SV=2 | 2073 | EPERSEsVFIE | 0.726 | 0.931 | 0.52 | 0.726 |
| sp\|Q8WXG9\|GPR98_HUMAN G-protein coupled receptor 98 OS=Homo sapiens GN=GPR98 PE=1 SV=2 | 2081 | FIELLNsTLVA | 0.189 | 0.067 | -0.981 | -0.242 |
| sp\|Q8WXG9\|GPR98_HUMAN G-protein coupled receptor 98 OS=Homo sapiens GN=GPR98 PE=1 SV=2 | 2082 | IELLNStLVAK | 0.309 | -0.019 | -0.396 | -0.035 |
| sp\|Q8WXG9\|GPR98_HUMAN G-protein coupled receptor 98 OS=Homo sapiens GN=GPR98 PE=1 SV=2 | 2089 | LVAKVQsRSIP | 0.138 | -0.194 | -1.002 | -0.353 |
| sp\|Q8WXG9\|GPR98_HUMAN G-protein coupled receptor 98 OS=Homo sapiens GN=GPR98 PE=1 SV=2 | 2091 | AKVQSRsIPNS | 0.717 | 0.685 | 0.252 | 0.551 |
| sp\|Q8WXG9\|GPR98_HUMAN G-protein coupled receptor 98 OS=Homo sapiens GN=GPR98 PE=1 SV=2 | 2095 | SRSIPNsPRLG | 0.092 | -0.251 | -1.315 | -0.491 |

| | | | | | | |
|---|---|---|---|---|---|---|
| sp\|Q8WXG9\|GPR98_HUMAN G-protein coupled receptor 98 OS=Homo sapiens GN=GPR98 PE=1 SV=2 | 2104 | LGPKVEtIAQL | 0.141 | -0.205 | -0.972 | -0.345 |
| sp\|Q8WXG9\|GPR98_HUMAN G-protein coupled receptor 98 OS=Homo sapiens GN=GPR98 PE=1 SV=2 | 2119 | NDDAFGtLQLS | 0.128 | -0.194 | -1.111 | -0.392 |
| sp\|Q8WXG9\|GPR98_HUMAN G-protein coupled receptor 98 OS=Homo sapiens GN=GPR98 PE=1 SV=2 | 2123 | FGTLQLsAPIV | 0.447 | 0.511 | -0.465 | 0.164 |
| sp\|Q8WXG9\|GPR98_HUMAN G-protein coupled receptor 98 OS=Homo sapiens GN=GPR98 PE=1 SV=2 | 2141 | GPIINVtRTGG | 0.34 | -0.128 | -0.752 | -0.18 |
| sp\|Q8WXG9\|GPR98_HUMAN G-protein coupled receptor 98 OS=Homo sapiens GN=GPR98 PE=1 SV=2 | 2143 | IINVTRtGGAF | 0.115 | 0.051 | -0.9 | -0.245 |
| sp\|Q8WXG9\|GPR98_HUMAN G-protein coupled receptor 98 OS=Homo sapiens GN=GPR98 PE=1 SV=2 | 2151 | GAFADVsVKFK | 0.214 | -0.218 | -0.912 | -0.305 |
| sp\|Q8WXG9\|GPR98_HUMAN G-protein coupled receptor 98 OS=Homo sapiens GN=GPR98 PE=1 SV=2 | 2160 | FKAVPItAIAG | 0.319 | 0.125 | -0.242 | 0.067 |
| sp\|Q8WXG9\|GPR98_HUMAN G-protein coupled receptor 98 OS=Homo sapiens GN=GPR98 PE=1 SV=2 | 2168 | IAGEDYsIASS | 0.035 | -0.274 | -1.515 | -0.585 |
| sp\|Q8WXG9\|GPR98_HUMAN G-protein coupled receptor 98 OS=Homo sapiens GN=GPR98 PE=1 SV=2 | 2171 | EDYSIAsSDVV | 0.089 | -0.142 | -1.2 | -0.418 |
| sp\|Q8WXG9\|GPR98_HUMAN G-protein coupled receptor 98 OS=Homo sapiens GN=GPR98 PE=1 SV=2 | 2172 | DYSIASsDVVL | 0.263 | 0.093 | -0.866 | -0.17 |
| sp\|Q8WXG9\|GPR98_HUMAN G-protein coupled receptor 98 OS=Homo sapiens GN=GPR98 PE=1 SV=2 | 2181 | VLLEGEtSKAV | 0.098 | -0.108 | -1.271 | -0.427 |
| sp\|Q8WXG9\|GPR98_HUMAN G-protein coupled receptor 98 OS=Homo sapiens GN=GPR98 PE=1 SV=2 | 2182 | LLEGETsKAVP | 0.134 | -0.105 | -0.839 | -0.27 |
| sp\|Q8WXG9\|GPR98_HUMAN G-protein coupled receptor 98 OS=Homo sapiens GN=GPR98 PE=1 SV=2 | 2200 | YPELEEsFLVQ | 0.112 | -0.151 | -1.357 | -0.465 |
| sp\|Q8WXG9\|GPR98_HUMAN G-protein coupled receptor 98 OS=Homo sapiens GN=GPR98 PE=1 SV=2 | 2209 | VQLMNEtTGGA | 0.193 | -0.224 | -0.628 | -0.22 |
| sp\|Q8WXG9\|GPR98_HUMAN G-protein coupled receptor 98 OS=Homo sapiens GN=GPR98 PE=1 SV=2 | 2210 | QLMNETtGGAR | 0.288 | 0.113 | -0.224 | 0.059 |
| sp\|Q8WXG9\|GPR98_HUMAN G-protein coupled receptor 98 OS=Homo sapiens GN=GPR98 PE=1 SV=2 | 2219 | ARLGALtEAVI | 0.283 | 0.285 | -0.384 | 0.061 |
| sp\|Q8WXG9\|GPR98_HUMAN G-protein coupled receptor 98 OS=Homo sapiens GN=GPR98 PE=1 SV=2 | 2228 | VIIIEAsDDPY | 0.324 | 0.094 | -0.835 | -0.139 |
| sp\|Q8WXG9\|GPR98_HUMAN G-protein coupled receptor 98 OS=Homo sapiens GN=GPR98 PE=1 SV=2 | 2240 | LFGFQItKLIV | 0.139 | -0.07 | -1.102 | -0.344 |
| sp\|Q8WXG9\|GPR98_HUMAN G-protein coupled receptor 98 OS=Homo sapiens GN=GPR98 PE=1 SV=2 | 2251 | EEPEFNsVKVN | 0.081 | -0.087 | -1.449 | -0.485 |
| sp\|Q8WXG9\|GPR98_HUMAN G-protein coupled receptor 98 OS=Homo sapiens GN=GPR98 PE=1 SV=2 | 2262 | LPIIRNsGTLG | 0.32 | 0.111 | -0.676 | -0.082 |
| sp\|Q8WXG9\|GPR98_HUMAN G-protein coupled receptor 98 OS=Homo sapiens GN=GPR98 PE=1 SV=2 | 2264 | IIRNSGtLGNV | 0.795 | 0.61 | 0.423 | 0.609 |
| sp\|Q8WXG9\|GPR98_HUMAN G-protein coupled receptor 98 OS=Homo sapiens GN=GPR98 PE=1 SV=2 | 2269 | GTLGNVtVQWV | 0.452 | 0.036 | -0.417 | 0.024 |
| sp\|Q8WXG9\|GPR98_HUMAN G-protein coupled receptor 98 OS=Homo sapiens GN=GPR98 PE=1 SV=2 | 2275 | TVQWVAtINGQ | 0.226 | -0.108 | -0.632 | -0.171 |
| sp\|Q8WXG9\|GPR98_HUMAN G-protein coupled receptor 98 OS=Homo sapiens GN=GPR98 PE=1 SV=2 | 2282 | INGQLAtGDLR | 0.178 | -0.144 | -0.698 | -0.221 |
| sp\|Q8WXG9\|GPR98_HUMAN G-protein coupled receptor 98 OS=Homo sapiens GN=GPR98 PE=1 SV=2 | 2289 | GDLRVVsGNVT | 0.663 | 0.749 | 0.379 | 0.597 |
| sp\|Q8WXG9\|GPR98_HUMAN G-protein coupled receptor 98 OS=Homo sapiens GN=GPR98 PE=1 SV=2 | 2293 | VVSGNVtFAPG | 0.229 | -0.128 | -0.542 | -0.147 |
| sp\|Q8WXG9\|GPR98_HUMAN G-protein coupled receptor 98 OS=Homo sapiens GN=GPR98 PE=1 SV=2 | 2299 | TFAPGEtIQTL | 0.08 | -0.23 | -0.977 | -0.376 |
| sp\|Q8WXG9\|GPR98_HUMAN G-protein coupled receptor 98 OS=Homo sapiens GN=GPR98 PE=1 SV=2 | 2302 | PGETIQtLLLE | 0.269 | -0.008 | -0.376 | -0.038 |
| sp\|Q8WXG9\|GPR98_HUMAN G-protein coupled receptor 98 OS=Homo sapiens GN=GPR98 PE=1 SV=2 | 2324 | VIQVQLtDASG | 0.059 | -0.09 | -1.554 | -0.528 |
| sp\|Q8WXG9\|GPR98_HUMAN G-protein coupled receptor 98 OS=Homo sapiens GN=GPR98 PE=1 SV=2 | 2327 | VQLTDAsGGGT | 0.166 | -0.218 | -0.752 | -0.268 |
| sp\|Q8WXG9\|GPR98_HUMAN G-protein coupled receptor 98 OS=Homo sapiens GN=GPR98 PE=1 SV=2 | 2331 | DASGGGtIGLD | 0.099 | -0.234 | -1.152 | -0.429 |
| sp\|Q8WXG9\|GPR98_HUMAN G-protein coupled receptor 98 OS=Homo sapiens GN=GPR98 PE=1 SV=2 | 2351 | NDDPYGtVAFA | 0.044 | -0.421 | -1.594 | -0.657 |
| sp\|Q8WXG9\|GPR98_HUMAN G-protein coupled receptor 98 OS=Homo sapiens GN=GPR98 PE=1 SV=2 | 2368 | QEPLERsSCAN | 0.055 | -0.244 | -1.804 | -0.664 |
| sp\|Q8WXG9\|GPR98_HUMAN G-protein coupled receptor 98 OS=Homo sapiens GN=GPR98 PE=1 SV=2 | 2369 | EPLERSsCANI | 0.135 | 0.088 | -0.982 | -0.253 |
| sp\|Q8WXG9\|GPR98_HUMAN G-protein coupled receptor 98 OS=Homo sapiens GN=GPR98 PE=1 SV=2 | 2374 | SSCANItVRRS | 0.152 | -0.332 | -1.189 | -0.456 |
| sp\|Q8WXG9\|GPR98_HUMAN G-protein coupled receptor 98 OS=Homo sapiens GN=GPR98 PE=1 SV=2 | 2378 | NITVRRsGGHF | 0.168 | 0.077 | -0.681 | -0.145 |
| sp\|Q8WXG9\|GPR98_HUMAN G-protein coupled receptor 98 OS=Homo sapiens GN=GPR98 PE=1 SV=2 | 2390 | RLLLFYsTSDI | 0.436 | 0.199 | -0.259 | 0.125 |
| sp\|Q8WXG9\|GPR98_HUMAN G-protein coupled receptor 98 OS=Homo sapiens GN=GPR98 PE=1 SV=2 | 2391 | LLLFYStSDID | 0.352 | 0.048 | -0.385 | 0.005 |
| sp\|Q8WXG9\|GPR98_HUMAN G-protein coupled receptor 98 OS=Homo sapiens GN=GPR98 PE=1 SV=2 | 2392 | LLFYSTsDIDV | 0.64 | 0.428 | 0.193 | 0.42 |
| sp\|Q8WXG9\|GPR98_HUMAN G-protein coupled receptor 98 OS=Homo sapiens GN=GPR98 PE=1 SV=2 | 2409 | EGQDLLsYYES | 0.056 | -0.145 | -1.397 | -0.495 |
| sp\|Q8WXG9\|GPR98_HUMAN G-protein coupled receptor 98 OS=Homo sapiens GN=GPR98 PE=1 SV=2 | 2413 | LLSYYEsPIQG | 0.13 | -0.288 | -0.854 | -0.337 |
| sp\|Q8WXG9\|GPR98_HUMAN G-protein coupled receptor 98 OS=Homo sapiens GN=GPR98 PE=1 SV=2 | 2425 | PDPLWRtWMNV | 0.1 | -0.01 | -1.593 | -0.501 |
| sp\|Q8WXG9\|GPR98_HUMAN G-protein coupled receptor 98 OS=Homo sapiens GN=GPR98 PE=1 SV=2 | 2430 | RTWMNVsAVGE | 0.363 | 0.019 | -0.63 | -0.083 |
| sp\|Q8WXG9\|GPR98_HUMAN G-protein coupled receptor 98 OS=Homo sapiens GN=GPR98 PE=1 SV=2 | 2438 | VGEPLYtCATL | 0.101 | -0.093 | -0.9 | -0.297 |
| sp\|Q8WXG9\|GPR98_HUMAN G-protein coupled receptor 98 OS=Homo sapiens GN=GPR98 PE=1 SV=2 | 2441 | PLYTCAtLCLK | 0.548 | 0.096 | -0.165 | 0.16 |
| sp\|Q8WXG9\|GPR98_HUMAN G-protein coupled receptor 98 OS=Homo sapiens GN=GPR98 PE=1 SV=2 | 2450 | LKEQACsAFSF | 0.413 | 0.1 | -0.352 | 0.054 |
| sp\|Q8WXG9\|GPR98_HUMAN G-protein coupled receptor 98 OS=Homo sapiens GN=GPR98 PE=1 SV=2 | 2453 | QACSAFsFFSA | 0.22 | -0.019 | -0.584 | -0.128 |
| sp\|Q8WXG9\|GPR98_HUMAN G-protein coupled receptor 98 OS=Homo sapiens GN=GPR98 PE=1 SV=2 | 2456 | SAFSFFsASEG | 0.107 | -0.163 | -1.472 | -0.509 |
| sp\|Q8WXG9\|GPR98_HUMAN G-protein coupled receptor 98 OS=Homo sapiens GN=GPR98 PE=1 SV=2 | 2458 | FSFFSAsEGPQ | 0.564 | 0.306 | -0.072 | 0.266 |

| | | | | | | |
|---|---|---|---|---|---|---|
| sp\|Q8WXG9\|GPR98_HUMAN G-protein coupled receptor 98 OS=Homo sapiens GN=GPR98 PE=1 SV=2 | 2467 | PQCFWMtSWIS | 0.418 | 0.165 | -0.45 | 0.044 |
| sp\|Q8WXG9\|GPR98_HUMAN G-protein coupled receptor 98 OS=Homo sapiens GN=GPR98 PE=1 SV=2 | 2468 | QCFWMTsWISP | 0.105 | -0.1 | -0.945 | -0.313 |
| sp\|Q8WXG9\|GPR98_HUMAN G-protein coupled receptor 98 OS=Homo sapiens GN=GPR98 PE=1 SV=2 | 2471 | WMTSWIsPAVN | 0.068 | -0.274 | -1.23 | -0.479 |
| sp\|Q8WXG9\|GPR98_HUMAN G-protein coupled receptor 98 OS=Homo sapiens GN=GPR98 PE=1 SV=2 | 2477 | SPAVNNsDFWT | 0.135 | -0.102 | -0.993 | -0.32 |
| sp\|Q8WXG9\|GPR98_HUMAN G-protein coupled receptor 98 OS=Homo sapiens GN=GPR98 PE=1 SV=2 | 2481 | NNSDFWtYRKN | 0.102 | -0.316 | -1.356 | -0.523 |
| sp\|Q8WXG9\|GPR98_HUMAN G-protein coupled receptor 98 OS=Homo sapiens GN=GPR98 PE=1 SV=2 | 2487 | TYRKNMtRVAS | 0.646 | 0.381 | 0.078 | 0.368 |
| sp\|Q8WXG9\|GPR98_HUMAN G-protein coupled receptor 98 OS=Homo sapiens GN=GPR98 PE=1 SV=2 | 2491 | NMTRVAsLFSG | 0.735 | 1.058 | 0.659 | 0.817 |
| sp\|Q8WXG9\|GPR98_HUMAN G-protein coupled receptor 98 OS=Homo sapiens GN=GPR98 PE=1 SV=2 | 2494 | RVASLFsGQAV | 0.155 | -0.021 | -0.869 | -0.245 |
| sp\|Q8WXG9\|GPR98_HUMAN G-protein coupled receptor 98 OS=Homo sapiens GN=GPR98 PE=1 SV=2 | 2501 | GQAVAGsDYEP | 0.15 | -0.045 | -0.96 | -0.285 |
| sp\|Q8WXG9\|GPR98_HUMAN G-protein coupled receptor 98 OS=Homo sapiens GN=GPR98 PE=1 SV=2 | 2507 | SDYEPVtRQWA | 0.056 | -0.373 | -1.346 | -0.554 |
| sp\|Q8WXG9\|GPR98_HUMAN G-protein coupled receptor 98 OS=Homo sapiens GN=GPR98 PE=1 SV=2 | 2523 | DEFANLtVSIL | 0.153 | -0.145 | -1.14 | -0.377 |
| sp\|Q8WXG9\|GPR98_HUMAN G-protein coupled receptor 98 OS=Homo sapiens GN=GPR98 PE=1 SV=2 | 2525 | FANLTVsILPD | 0.129 | -0.086 | -1.05 | -0.336 |
| sp\|Q8WXG9\|GPR98_HUMAN G-protein coupled receptor 98 OS=Homo sapiens GN=GPR98 PE=1 SV=2 | 2537 | FPEMDEsFLIS | 0.167 | -0.129 | -1.086 | -0.349 |
| sp\|Q8WXG9\|GPR98_HUMAN G-protein coupled receptor 98 OS=Homo sapiens GN=GPR98 PE=1 SV=2 | 2541 | DESFLIsLLEV | 0.316 | -0.037 | -1.052 | -0.258 |
| sp\|Q8WXG9\|GPR98_HUMAN G-protein coupled receptor 98 OS=Homo sapiens GN=GPR98 PE=1 SV=2 | 2551 | VHLMNIsASLK | 0.141 | -0.192 | -1.187 | -0.413 |
| sp\|Q8WXG9\|GPR98_HUMAN G-protein coupled receptor 98 OS=Homo sapiens GN=GPR98 PE=1 SV=2 | 2553 | LMNISAsLKNQ | 0.392 | 0.383 | -0.32 | 0.152 |
| sp\|Q8WXG9\|GPR98_HUMAN G-protein coupled receptor 98 OS=Homo sapiens GN=GPR98 PE=1 SV=2 | 2559 | SLKNQPtIGQP | 0.12 | -0.128 | -0.819 | -0.276 |
| sp\|Q8WXG9\|GPR98_HUMAN G-protein coupled receptor 98 OS=Homo sapiens GN=GPR98 PE=1 SV=2 | 2566 | IGQPNIsTVVI | 0.322 | 0.006 | -0.307 | 0.007 |
| sp\|Q8WXG9\|GPR98_HUMAN G-protein coupled receptor 98 OS=Homo sapiens GN=GPR98 PE=1 SV=2 | 2567 | GQPNIStVVIA | 0.085 | -0.161 | -0.872 | -0.316 |
| sp\|Q8WXG9\|GPR98_HUMAN G-protein coupled receptor 98 OS=Homo sapiens GN=GPR98 PE=1 SV=2 | 2586 | FVIYNIsPNTS | 0.123 | -0.21 | -0.849 | -0.312 |
| sp\|Q8WXG9\|GPR98_HUMAN G-protein coupled receptor 98 OS=Homo sapiens GN=GPR98 PE=1 SV=2 | 2589 | YNISPNtSEDG | 0.145 | -0.166 | -0.972 | -0.331 |
| sp\|Q8WXG9\|GPR98_HUMAN G-protein coupled receptor 98 OS=Homo sapiens GN=GPR98 PE=1 SV=2 | 2590 | NISPNTsEDGL | 0.33 | 0.077 | -0.213 | 0.065 |
| sp\|Q8WXG9\|GPR98_HUMAN G-protein coupled receptor 98 OS=Homo sapiens GN=GPR98 PE=1 SV=2 | 2604 | VQEQPQtLVEL | 0.289 | -0.01 | -0.133 | 0.049 |
| sp\|Q8WXG9\|GPR98_HUMAN G-protein coupled receptor 98 OS=Homo sapiens GN=GPR98 PE=1 SV=2 | 2613 | ELMIHRtGGSL | 0.151 | 0.129 | -0.8 | -0.173 |
| sp\|Q8WXG9\|GPR98_HUMAN G-protein coupled receptor 98 OS=Homo sapiens GN=GPR98 PE=1 SV=2 | 2616 | IHRTGGsLGQV | 0.45 | 0.15 | -0.607 | -0.002 |
| sp\|Q8WXG9\|GPR98_HUMAN G-protein coupled receptor 98 OS=Homo sapiens GN=GPR98 PE=1 SV=2 | 2630 | WRVVGGtATEG | 0.109 | 0.027 | -1.215 | -0.36 |
| sp\|Q8WXG9\|GPR98_HUMAN G-protein coupled receptor 98 OS=Homo sapiens GN=GPR98 PE=1 SV=2 | 2632 | VVGGTAtEGLD | 0.189 | 0.14 | -0.56 | -0.077 |
| sp\|Q8WXG9\|GPR98_HUMAN G-protein coupled receptor 98 OS=Homo sapiens GN=GPR98 PE=1 SV=2 | 2645 | GAGEILtFAEG | 0.111 | -0.09 | -1.013 | -0.331 |
| sp\|Q8WXG9\|GPR98_HUMAN G-protein coupled receptor 98 OS=Homo sapiens GN=GPR98 PE=1 SV=2 | 2651 | TFAEGEtKKTV | 0.064 | -0.238 | -1.347 | -0.507 |
| sp\|Q8WXG9\|GPR98_HUMAN G-protein coupled receptor 98 OS=Homo sapiens GN=GPR98 PE=1 SV=2 | 2654 | EGETKKtVILT | 0.11 | -0.117 | -0.647 | -0.218 |
| sp\|Q8WXG9\|GPR98_HUMAN G-protein coupled receptor 98 OS=Homo sapiens GN=GPR98 PE=1 SV=2 | 2658 | KKTVILtILDD | 0.16 | -0.04 | -0.616 | -0.165 |
| sp\|Q8WXG9\|GPR98_HUMAN G-protein coupled receptor 98 OS=Homo sapiens GN=GPR98 PE=1 SV=2 | 2663 | LTILDDsEPED | 0.614 | 0.458 | -0.134 | 0.313 |
| sp\|Q8WXG9\|GPR98_HUMAN G-protein coupled receptor 98 OS=Homo sapiens GN=GPR98 PE=1 SV=2 | 2670 | EPEDDEsIIVS | 0.052 | -0.232 | -1.431 | -0.537 |
| sp\|Q8WXG9\|GPR98_HUMAN G-protein coupled receptor 98 OS=Homo sapiens GN=GPR98 PE=1 SV=2 | 2674 | DESIIVsLVYT | 0.32 | -0.069 | -0.628 | -0.126 |
| sp\|Q8WXG9\|GPR98_HUMAN G-protein coupled receptor 98 OS=Homo sapiens GN=GPR98 PE=1 SV=2 | 2678 | IVSLVYtEGGS | 0.271 | 0.061 | -0.438 | -0.035 |
| sp\|Q8WXG9\|GPR98_HUMAN G-protein coupled receptor 98 OS=Homo sapiens GN=GPR98 PE=1 SV=2 | 2682 | VYTEGGsRILP | 0.039 | -0.329 | -1.628 | -0.639 |
| sp\|Q8WXG9\|GPR98_HUMAN G-protein coupled receptor 98 OS=Homo sapiens GN=GPR98 PE=1 SV=2 | 2687 | GSRILPsSDTV | 0.284 | 0.072 | -0.91 | -0.185 |
| sp\|Q8WXG9\|GPR98_HUMAN G-protein coupled receptor 98 OS=Homo sapiens GN=GPR98 PE=1 SV=2 | 2688 | SRILPSsDTVR | 0.128 | 0.056 | -1.192 | -0.336 |
| sp\|Q8WXG9\|GPR98_HUMAN G-protein coupled receptor 98 OS=Homo sapiens GN=GPR98 PE=1 SV=2 | 2690 | ILPSSDtVRVN | 0.395 | 0.082 | -0.449 | 0.009 |
| sp\|Q8WXG9\|GPR98_HUMAN G-protein coupled receptor 98 OS=Homo sapiens GN=GPR98 PE=1 SV=2 | 2706 | NVAGIVsFQTA | 0.348 | 0.017 | -0.042 | 0.108 |
| sp\|Q8WXG9\|GPR98_HUMAN G-protein coupled receptor 98 OS=Homo sapiens GN=GPR98 PE=1 SV=2 | 2709 | GIVSFQtASRS | 0.133 | -0.111 | -1.43 | -0.469 |
| sp\|Q8WXG9\|GPR98_HUMAN G-protein coupled receptor 98 OS=Homo sapiens GN=GPR98 PE=1 SV=2 | 2711 | VSFQTAsRSVI | 0.171 | -0.11 | -0.906 | -0.282 |
| sp\|Q8WXG9\|GPR98_HUMAN G-protein coupled receptor 98 OS=Homo sapiens GN=GPR98 PE=1 SV=2 | 2713 | FQTASRsVIGH | 0.174 | 0.059 | -0.53 | -0.099 |
| sp\|Q8WXG9\|GPR98_HUMAN G-protein coupled receptor 98 OS=Homo sapiens GN=GPR98 PE=1 SV=2 | 2729 | QFHVIRtFPGR | 0.396 | 0.429 | 0.022 | 0.282 |
| sp\|Q8WXG9\|GPR98_HUMAN G-protein coupled receptor 98 OS=Homo sapiens GN=GPR98 PE=1 SV=2 | 2737 | PGRGNVtVNWK | 0.61 | 0.238 | 0.318 | 0.389 |
| sp\|Q8WXG9\|GPR98_HUMAN G-protein coupled receptor 98 OS=Homo sapiens GN=GPR98 PE=1 SV=2 | 2755 | LNFANFsGQLF | 0.121 | -0.151 | -0.681 | -0.237 |
| sp\|Q8WXG9\|GPR98_HUMAN G-protein coupled receptor 98 OS=Homo sapiens GN=GPR98 PE=1 SV=2 | 2764 | LFFPEGsLNTT | 0.206 | -0.128 | -0.593 | -0.172 |
| sp\|Q8WXG9\|GPR98_HUMAN G-protein coupled receptor 98 OS=Homo sapiens GN=GPR98 PE=1 SV=2 | 2767 | PEGSLNtTLFV | 0.06 | -0.239 | -1.906 | -0.695 |
| sp\|Q8WXG9\|GPR98_HUMAN G-protein coupled receptor 98 OS=Homo sapiens GN=GPR98 PE=1 SV=2 | 2768 | EGSLNTtLFVH | 0.215 | -0.083 | -0.588 | -0.152 |

| | | | | | | |
|---|---|---|---|---|---|---|
| sp\|Q8WXG9\|GPR98_HUMAN G-protein coupled receptor 98 OS=Homo sapiens GN=GPR98 PE=1 SV=2 | 2794 | ILYDVRtQGVP | 0.221 | -0.001 | -1.058 | -0.279 |
| sp\|Q8WXG9\|GPR98_HUMAN G-protein coupled receptor 98 OS=Homo sapiens GN=GPR98 PE=1 SV=2 | 2815 | GYAAVLtVEAS | 0.226 | 0.019 | -0.683 | -0.146 |
| sp\|Q8WXG9\|GPR98_HUMAN G-protein coupled receptor 98 OS=Homo sapiens GN=GPR98 PE=1 SV=2 | 2819 | VLTVEAsDEPH | 0.145 | -0.151 | -1.261 | -0.422 |
| sp\|Q8WXG9\|GPR98_HUMAN G-protein coupled receptor 98 OS=Homo sapiens GN=GPR98 PE=1 SV=2 | 2831 | VLNFALsSRFV | 0.176 | -0.107 | -1.175 | -0.369 |
| sp\|Q8WXG9\|GPR98_HUMAN G-protein coupled receptor 98 OS=Homo sapiens GN=GPR98 PE=1 SV=2 | 2832 | LNFALSsRFVL | 0.064 | -0.269 | -1.317 | -0.507 |
| sp\|Q8WXG9\|GPR98_HUMAN G-protein coupled receptor 98 OS=Homo sapiens GN=GPR98 PE=1 SV=2 | 2843 | LQEANItIQLF | 0.146 | -0.152 | -0.529 | -0.178 |
| sp\|Q8WXG9\|GPR98_HUMAN G-protein coupled receptor 98 OS=Homo sapiens GN=GPR98 PE=1 SV=2 | 2854 | INREFGsLGAI | 0.583 | 0.275 | 0.154 | 0.337 |
| sp\|Q8WXG9\|GPR98_HUMAN G-protein coupled receptor 98 OS=Homo sapiens GN=GPR98 PE=1 SV=2 | 2861 | LGAINVtYTTV | 0.265 | -0.131 | -0.69 | -0.185 |
| sp\|Q8WXG9\|GPR98_HUMAN G-protein coupled receptor 98 OS=Homo sapiens GN=GPR98 PE=1 SV=2 | 2863 | AINVTYtTVPG | 0.081 | -0.001 | -1.15 | -0.357 |
| sp\|Q8WXG9\|GPR98_HUMAN G-protein coupled receptor 98 OS=Homo sapiens GN=GPR98 PE=1 SV=2 | 2864 | INVTYTtVPGM | 0.526 | 0.321 | 0.126 | 0.324 |
| sp\|Q8WXG9\|GPR98_HUMAN G-protein coupled receptor 98 OS=Homo sapiens GN=GPR98 PE=1 SV=2 | 2870 | TVPGMLsLKNQ | 0.082 | -0.162 | -1.119 | -0.4 |
| sp\|Q8WXG9\|GPR98_HUMAN G-protein coupled receptor 98 OS=Homo sapiens GN=GPR98 PE=1 SV=2 | 2875 | LSLKNQtVGNL | 0.424 | 0.113 | -0.181 | 0.119 |
| sp\|Q8WXG9\|GPR98_HUMAN G-protein coupled receptor 98 OS=Homo sapiens GN=GPR98 PE=1 SV=2 | 2900 | ILEEGEtAAAI | 0.222 | 0.013 | -0.687 | -0.151 |
| sp\|Q8WXG9\|GPR98_HUMAN G-protein coupled receptor 98 OS=Homo sapiens GN=GPR98 PE=1 SV=2 | 2907 | AAAINItILED | 0.175 | -0.14 | -0.78 | -0.248 |
| sp\|Q8WXG9\|GPR98_HUMAN G-protein coupled receptor 98 OS=Homo sapiens GN=GPR98 PE=1 SV=2 | 2925 | YFLVNLtYVGL | 0.081 | -0.129 | -0.822 | -0.29 |
| sp\|Q8WXG9\|GPR98_HUMAN G-protein coupled receptor 98 OS=Homo sapiens GN=GPR98 PE=1 SV=2 | 2930 | LTYVGLtMAAS | 0.034 | -0.121 | -1.49 | -0.526 |
| sp\|Q8WXG9\|GPR98_HUMAN G-protein coupled receptor 98 OS=Homo sapiens GN=GPR98 PE=1 SV=2 | 2934 | GLTMAAsTSFP | 0.25 | -0.041 | -0.76 | -0.184 |
| sp\|Q8WXG9\|GPR98_HUMAN G-protein coupled receptor 98 OS=Homo sapiens GN=GPR98 PE=1 SV=2 | 2935 | LTMAAStSFPP | 0.09 | -0.206 | -1.123 | -0.413 |
| sp\|Q8WXG9\|GPR98_HUMAN G-protein coupled receptor 98 OS=Homo sapiens GN=GPR98 PE=1 SV=2 | 2936 | TMAASTsFPPR | 0.718 | 0.753 | 0.736 | 0.736 |
| sp\|Q8WXG9\|GPR98_HUMAN G-protein coupled receptor 98 OS=Homo sapiens GN=GPR98 PE=1 SV=2 | 2943 | FPPRLDsEGLT | 0.486 | 0.786 | -0.114 | 0.386 |
| sp\|Q8WXG9\|GPR98_HUMAN G-protein coupled receptor 98 OS=Homo sapiens GN=GPR98 PE=1 SV=2 | 2947 | LDSEGLtAQVI | 0.047 | -0.168 | -1.327 | -0.483 |
| sp\|Q8WXG9\|GPR98_HUMAN G-protein coupled receptor 98 OS=Homo sapiens GN=GPR98 PE=1 SV=2 | 2967 | VIEWQQsRFEV | 0.215 | -0.087 | -0.938 | -0.27 |
| sp\|Q8WXG9\|GPR98_HUMAN G-protein coupled receptor 98 OS=Homo sapiens GN=GPR98 PE=1 SV=2 | 2974 | RFEVNEtHGSL | 0.215 | -0.006 | -0.677 | -0.156 |
| sp\|Q8WXG9\|GPR98_HUMAN G-protein coupled receptor 98 OS=Homo sapiens GN=GPR98 PE=1 SV=2 | 2977 | VNETHGsLTLV | 0.277 | -0.124 | -0.914 | -0.254 |
| sp\|Q8WXG9\|GPR98_HUMAN G-protein coupled receptor 98 OS=Homo sapiens GN=GPR98 PE=1 SV=2 | 2979 | ETHGSLtLVAQ | 0.238 | 0.178 | -0.379 | 0.012 |
| sp\|Q8WXG9\|GPR98_HUMAN G-protein coupled receptor 98 OS=Homo sapiens GN=GPR98 PE=1 SV=2 | 2985 | TLVAQRsREPL | 0.08 | -0.147 | -1.388 | -0.485 |
| sp\|Q8WXG9\|GPR98_HUMAN G-protein coupled receptor 98 OS=Homo sapiens GN=GPR98 PE=1 SV=2 | 2993 | EPLGHVsLFVY | 0.481 | 0.142 | -0.119 | 0.168 |
| sp\|Q8WXG9\|GPR98_HUMAN G-protein coupled receptor 98 OS=Homo sapiens GN=GPR98 PE=1 SV=2 | 3012 | GLDYIFtPMIL | 0.25 | -0.097 | -0.557 | -0.135 |
| sp\|Q8WXG9\|GPR98_HUMAN G-protein coupled receptor 98 OS=Homo sapiens GN=GPR98 PE=1 SV=2 | 3048 | KFQLILtNPSP | 0.201 | 0.312 | -0.597 | -0.028 |
| sp\|Q8WXG9\|GPR98_HUMAN G-protein coupled receptor 98 OS=Homo sapiens GN=GPR98 PE=1 SV=2 | 3051 | LILTNPsPGLE | 0.133 | -0.318 | -0.951 | -0.379 |
| sp\|Q8WXG9\|GPR98_HUMAN G-protein coupled receptor 98 OS=Homo sapiens GN=GPR98 PE=1 SV=2 | 3060 | LELGKNtIALI | 0.131 | -0.103 | -0.884 | -0.285 |
| sp\|Q8WXG9\|GPR98_HUMAN G-protein coupled receptor 98 OS=Homo sapiens GN=GPR98 PE=1 SV=2 | 3077 | DGPGVLsFNNS | 0.143 | -0.064 | -1.029 | -0.317 |
| sp\|Q8WXG9\|GPR98_HUMAN G-protein coupled receptor 98 OS=Homo sapiens GN=GPR98 PE=1 SV=2 | 3081 | VLSFNNsEHFF | 0.352 | 0.072 | -0.509 | -0.028 |
| sp\|Q8WXG9\|GPR98_HUMAN G-protein coupled receptor 98 OS=Homo sapiens GN=GPR98 PE=1 SV=2 | 3090 | FFLREPtALYV | 0.579 | 0.822 | -0.216 | 0.395 |
| sp\|Q8WXG9\|GPR98_HUMAN G-protein coupled receptor 98 OS=Homo sapiens GN=GPR98 PE=1 SV=2 | 3097 | ALYVQEsVAVL | 0.084 | -0.04 | -1.279 | -0.412 |
| sp\|Q8WXG9\|GPR98_HUMAN G-protein coupled receptor 98 OS=Homo sapiens GN=GPR98 PE=1 SV=2 | 3114 | AQGLFGtVTVQ | 0.073 | -0.237 | -1.534 | -0.566 |
| sp\|Q8WXG9\|GPR98_HUMAN G-protein coupled receptor 98 OS=Homo sapiens GN=GPR98 PE=1 SV=2 | 3116 | GLFGTVtVQFI | 0.441 | 0.172 | -0.172 | 0.147 |
| sp\|Q8WXG9\|GPR98_HUMAN G-protein coupled receptor 98 OS=Homo sapiens GN=GPR98 PE=1 SV=2 | 3122 | TVQFIVtEVNS | 0.266 | 0.122 | -0.636 | -0.083 |
| sp\|Q8WXG9\|GPR98_HUMAN G-protein coupled receptor 98 OS=Homo sapiens GN=GPR98 PE=1 SV=2 | 3126 | IVTEVNsSNES | 0.198 | -0.024 | -0.829 | -0.218 |
| sp\|Q8WXG9\|GPR98_HUMAN G-protein coupled receptor 98 OS=Homo sapiens GN=GPR98 PE=1 SV=2 | 3127 | VTEVNSsNESK | 0.046 | -0.36 | -1.627 | -0.647 |
| sp\|Q8WXG9\|GPR98_HUMAN G-protein coupled receptor 98 OS=Homo sapiens GN=GPR98 PE=1 SV=2 | 3130 | VNSSNEsKDLT | 0.104 | -0.301 | -1.017 | -0.405 |
| sp\|Q8WXG9\|GPR98_HUMAN G-protein coupled receptor 98 OS=Homo sapiens GN=GPR98 PE=1 SV=2 | 3134 | NESKDLtPSKG | 0.061 | -0.495 | -1.707 | -0.714 |
| sp\|Q8WXG9\|GPR98_HUMAN G-protein coupled receptor 98 OS=Homo sapiens GN=GPR98 PE=1 SV=2 | 3136 | SKDLTPsKGYI | 0.245 | -0.019 | -0.718 | -0.164 |
| sp\|Q8WXG9\|GPR98_HUMAN G-protein coupled receptor 98 OS=Homo sapiens GN=GPR98 PE=1 SV=2 | 3154 | FKALQIsAILD | 0.353 | 0.187 | -0.389 | 0.05 |
| sp\|Q8WXG9\|GPR98_HUMAN G-protein coupled receptor 98 OS=Homo sapiens GN=GPR98 PE=1 SV=2 | 3159 | ISAILDtEPEM | 0.521 | 0.362 | -0.098 | 0.262 |
| sp\|Q8WXG9\|GPR98_HUMAN G-protein coupled receptor 98 OS=Homo sapiens GN=GPR98 PE=1 SV=2 | 3170 | DEYFVCtLFNP | 0.396 | 0.048 | -0.857 | -0.138 |
| sp\|Q8WXG9\|GPR98_HUMAN G-protein coupled receptor 98 OS=Homo sapiens GN=GPR98 PE=1 SV=2 | 3175 | CTLFNPtGGAR | 0.432 | 0.017 | -0.128 | 0.107 |
| sp\|Q8WXG9\|GPR98_HUMAN G-protein coupled receptor 98 OS=Homo sapiens GN=GPR98 PE=1 SV=2 | 3186 | LGVHVQtLITV | 0.23 | -0.01 | -0.552 | -0.111 |
| sp\|Q8WXG9\|GPR98_HUMAN G-protein coupled receptor 98 OS=Homo sapiens GN=GPR98 PE=1 SV=2 | 3189 | HVQTLItVLQN | 0.132 | -0.005 | -0.97 | -0.281 |

| | | | | | | |
|---|---|---|---|---|---|---|
| sp\|Q8WXG9\|GPR98_HUMAN G-protein coupled receptor 98 OS=Homo sapiens GN=GPR98 PE=1 SV=2 | 3201 | APLGLFsISAV | 0.075 | -0.16 | -1.287 | -0.457 |
| sp\|Q8WXG9\|GPR98_HUMAN G-protein coupled receptor 98 OS=Homo sapiens GN=GPR98 PE=1 SV=2 | 3203 | LGLFSIsAVEN | 0.463 | 0.26 | -0.165 | 0.186 |
| sp\|Q8WXG9\|GPR98_HUMAN G-protein coupled receptor 98 OS=Homo sapiens GN=GPR98 PE=1 SV=2 | 3210 | AVENRAtSIDI | 0.409 | 0.176 | -0.106 | 0.16 |
| sp\|Q8WXG9\|GPR98_HUMAN G-protein coupled receptor 98 OS=Homo sapiens GN=GPR98 PE=1 SV=2 | 3211 | VENRATsIDIE | 0.66 | 0.798 | 0.252 | 0.57 |
| sp\|Q8WXG9\|GPR98_HUMAN G-protein coupled receptor 98 OS=Homo sapiens GN=GPR98 PE=1 SV=2 | 3220 | IEEANRtVYLN | 0.198 | -0.07 | -0.871 | -0.248 |
| sp\|Q8WXG9\|GPR98_HUMAN G-protein coupled receptor 98 OS=Homo sapiens GN=GPR98 PE=1 SV=2 | 3226 | TVYLNVsRTNG | 0.227 | -0.115 | -0.964 | -0.284 |
| sp\|Q8WXG9\|GPR98_HUMAN G-protein coupled receptor 98 OS=Homo sapiens GN=GPR98 PE=1 SV=2 | 3228 | YLNVSRtNGID | 0.434 | 0.262 | -0.405 | 0.097 |
| sp\|Q8WXG9\|GPR98_HUMAN G-protein coupled receptor 98 OS=Homo sapiens GN=GPR98 PE=1 SV=2 | 3236 | GIDLAVsVQWE | 0.507 | 0.145 | -0.292 | 0.12 |
| sp\|Q8WXG9\|GPR98_HUMAN G-protein coupled receptor 98 OS=Homo sapiens GN=GPR98 PE=1 SV=2 | 3241 | VSVQWEtVSET | 0.128 | -0.216 | -1.073 | -0.387 |
| sp\|Q8WXG9\|GPR98_HUMAN G-protein coupled receptor 98 OS=Homo sapiens GN=GPR98 PE=1 SV=2 | 3243 | VQWETVsETAF | 0.14 | 0.15 | -0.616 | -0.109 |
| sp\|Q8WXG9\|GPR98_HUMAN G-protein coupled receptor 98 OS=Homo sapiens GN=GPR98 PE=1 SV=2 | 3245 | WETVSEtAFGM | 0.231 | 0.052 | -0.591 | -0.103 |
| sp\|Q8WXG9\|GPR98_HUMAN G-protein coupled receptor 98 OS=Homo sapiens GN=GPR98 PE=1 SV=2 | 3257 | GMDVVFsVFQS | 0.142 | 0.065 | -0.928 | -0.24 |
| sp\|Q8WXG9\|GPR98_HUMAN G-protein coupled receptor 98 OS=Homo sapiens GN=GPR98 PE=1 SV=2 | 3261 | VFSVFQsFLDE | 0.201 | -0.042 | -0.747 | -0.196 |
| sp\|Q8WXG9\|GPR98_HUMAN G-protein coupled receptor 98 OS=Homo sapiens GN=GPR98 PE=1 SV=2 | 3266 | QSFLDEsASGW | 0.082 | -0.245 | -1.527 | -0.563 |
| sp\|Q8WXG9\|GPR98_HUMAN G-protein coupled receptor 98 OS=Homo sapiens GN=GPR98 PE=1 SV=2 | 3268 | FLDESAsGWCF | 0.429 | 0.365 | -0.28 | 0.171 |
| sp\|Q8WXG9\|GPR98_HUMAN G-protein coupled receptor 98 OS=Homo sapiens GN=GPR98 PE=1 SV=2 | 3274 | SGWCFFtLENL | 0.246 | 0.186 | -0.737 | -0.102 |
| sp\|Q8WXG9\|GPR98_HUMAN G-protein coupled receptor 98 OS=Homo sapiens GN=GPR98 PE=1 SV=2 | 3287 | GIMLRKsSVTV | 0.449 | 0.276 | -0.468 | 0.086 |
| sp\|Q8WXG9\|GPR98_HUMAN G-protein coupled receptor 98 OS=Homo sapiens GN=GPR98 PE=1 SV=2 | 3288 | IMLRKSsVTVY | 0.527 | 0.886 | 0.118 | 0.51 |
| sp\|Q8WXG9\|GPR98_HUMAN G-protein coupled receptor 98 OS=Homo sapiens GN=GPR98 PE=1 SV=2 | 3290 | LRKSSVtVYRW | 0.531 | 0.353 | -0.02 | 0.288 |
| sp\|Q8WXG9\|GPR98_HUMAN G-protein coupled receptor 98 OS=Homo sapiens GN=GPR98 PE=1 SV=2 | 3311 | NIENPKtCEAF | 0.222 | 0.12 | -0.203 | 0.046 |
| sp\|Q8WXG9\|GPR98_HUMAN G-protein coupled receptor 98 OS=Homo sapiens GN=GPR98 PE=1 SV=2 | 3320 | AFNIGFsPYFV | 0.038 | -0.431 | -1.812 | -0.735 |
| sp\|Q8WXG9\|GPR98_HUMAN G-protein coupled receptor 98 OS=Homo sapiens GN=GPR98 PE=1 SV=2 | 3326 | SPYFVItHEER | 0.192 | -0.094 | -1.002 | -0.301 |
| sp\|Q8WXG9\|GPR98_HUMAN G-protein coupled receptor 98 OS=Homo sapiens GN=GPR98 PE=1 SV=2 | 3336 | RNEEKPsLNSV | 0.114 | -0.156 | -1.052 | -0.365 |
| sp\|Q8WXG9\|GPR98_HUMAN G-protein coupled receptor 98 OS=Homo sapiens GN=GPR98 PE=1 SV=2 | 3339 | EKPSLNsVFTF | 0.147 | -0.11 | -0.965 | -0.309 |
| sp\|Q8WXG9\|GPR98_HUMAN G-protein coupled receptor 98 OS=Homo sapiens GN=GPR98 PE=1 SV=2 | 3342 | SLNSVFtFTSG | 0.213 | -0.017 | -0.928 | -0.244 |
| sp\|Q8WXG9\|GPR98_HUMAN G-protein coupled receptor 98 OS=Homo sapiens GN=GPR98 PE=1 SV=2 | 3344 | NSVFTFtSGFK | 0.124 | -0.111 | -1.007 | -0.331 |
| sp\|Q8WXG9\|GPR98_HUMAN G-protein coupled receptor 98 OS=Homo sapiens GN=GPR98 PE=1 SV=2 | 3345 | SVFTFTsGFKL | 0.084 | -0.145 | -1.272 | -0.444 |
| sp\|Q8WXG9\|GPR98_HUMAN G-protein coupled receptor 98 OS=Homo sapiens GN=GPR98 PE=1 SV=2 | 3354 | KLFLVQtIIIL | 0.231 | 0.045 | -0.616 | -0.113 |
| sp\|Q8WXG9\|GPR98_HUMAN G-protein coupled receptor 98 OS=Homo sapiens GN=GPR98 PE=1 SV=2 | 3360 | TIIILEsSQVR | 0.1 | -0.038 | -1.263 | -0.4 |
| sp\|Q8WXG9\|GPR98_HUMAN G-protein coupled receptor 98 OS=Homo sapiens GN=GPR98 PE=1 SV=2 | 3361 | IIILESsQVRY | 0.145 | -0.145 | -1.491 | -0.497 |
| sp\|Q8WXG9\|GPR98_HUMAN G-protein coupled receptor 98 OS=Homo sapiens GN=GPR98 PE=1 SV=2 | 3367 | SQVRYFtSDSQ | 0.306 | 0.726 | -0.194 | 0.279 |
| sp\|Q8WXG9\|GPR98_HUMAN G-protein coupled receptor 98 OS=Homo sapiens GN=GPR98 PE=1 SV=2 | 3368 | QVRYFTsDSQD | 0.358 | 0.19 | -0.376 | 0.057 |
| sp\|Q8WXG9\|GPR98_HUMAN G-protein coupled receptor 98 OS=Homo sapiens GN=GPR98 PE=1 SV=2 | 3370 | RYFTSDsQDYL | 0.45 | 0.178 | -0.618 | 0.003 |
| sp\|Q8WXG9\|GPR98_HUMAN G-protein coupled receptor 98 OS=Homo sapiens GN=GPR98 PE=1 SV=2 | 3378 | DYLIIAsQRDD | 0.486 | 0.022 | -0.455 | 0.018 |
| sp\|Q8WXG9\|GPR98_HUMAN G-protein coupled receptor 98 OS=Homo sapiens GN=GPR98 PE=1 SV=2 | 3383 | ASQRDDsELTQ | 0.436 | 0.651 | -0.33 | 0.252 |
| sp\|Q8WXG9\|GPR98_HUMAN G-protein coupled receptor 98 OS=Homo sapiens GN=GPR98 PE=1 SV=2 | 3386 | RDDSELtQVFR | 0.034 | -0.39 | -1.971 | -0.776 |
| sp\|Q8WXG9\|GPR98_HUMAN G-protein coupled receptor 98 OS=Homo sapiens GN=GPR98 PE=1 SV=2 | 3395 | FRWNGGsFVLH | 0.089 | 0.198 | -1.032 | -0.248 |
| sp\|Q8WXG9\|GPR98_HUMAN G-protein coupled receptor 98 OS=Homo sapiens GN=GPR98 PE=1 SV=2 | 3409 | PVRGVLtVALF | 0.161 | 0.139 | -0.696 | -0.132 |
| sp\|Q8WXG9\|GPR98_HUMAN G-protein coupled receptor 98 OS=Homo sapiens GN=GPR98 PE=1 SV=2 | 3418 | LFNKGGsVFLA | 0.156 | -0.105 | -0.762 | -0.237 |
| sp\|Q8WXG9\|GPR98_HUMAN G-protein coupled receptor 98 OS=Homo sapiens GN=GPR98 PE=1 SV=2 | 3424 | SVFLAIsQANA | 0.138 | -0.097 | -1.342 | -0.434 |
| sp\|Q8WXG9\|GPR98_HUMAN G-protein coupled receptor 98 OS=Homo sapiens GN=GPR98 PE=1 SV=2 | 3432 | ANARLNsLLFR | 0.549 | 0.726 | 0.142 | 0.472 |
| sp\|Q8WXG9\|GPR98_HUMAN G-protein coupled receptor 98 OS=Homo sapiens GN=GPR98 PE=1 SV=2 | 3438 | SLLFRWsGSGF | 0.218 | -0.042 | -0.704 | -0.176 |
| sp\|Q8WXG9\|GPR98_HUMAN G-protein coupled receptor 98 OS=Homo sapiens GN=GPR98 PE=1 SV=2 | 3440 | LFRWSGsGFIN | 0.605 | 0.323 | 0.192 | 0.373 |
| sp\|Q8WXG9\|GPR98_HUMAN G-protein coupled receptor 98 OS=Homo sapiens GN=GPR98 PE=1 SV=2 | 3451 | FQEVPVsGTTE | 0.159 | -0.223 | -0.801 | -0.288 |
| sp\|Q8WXG9\|GPR98_HUMAN G-protein coupled receptor 98 OS=Homo sapiens GN=GPR98 PE=1 SV=2 | 3453 | EVPVSGtTEVE | 0.061 | -0.034 | -1.24 | -0.404 |
| sp\|Q8WXG9\|GPR98_HUMAN G-protein coupled receptor 98 OS=Homo sapiens GN=GPR98 PE=1 SV=2 | 3454 | VPVSGTtEVEA | 0.093 | -0.171 | -1.13 | -0.403 |
| sp\|Q8WXG9\|GPR98_HUMAN G-protein coupled receptor 98 OS=Homo sapiens GN=GPR98 PE=1 SV=2 | 3460 | TEVEALsSAND | 0.078 | -0.119 | -1.479 | -0.507 |
| sp\|Q8WXG9\|GPR98_HUMAN G-protein coupled receptor 98 OS=Homo sapiens GN=GPR98 PE=1 SV=2 | 3461 | EVEALSsANDI | 0.196 | -0.023 | -0.674 | -0.167 |
| sp\|Q8WXG9\|GPR98_HUMAN G-protein coupled receptor 98 OS=Homo sapiens GN=GPR98 PE=1 SV=2 | 3480 | FLGDQNsIDIF | 0.261 | 0.137 | -0.74 | -0.114 |

| | | | | | | |
|---|---|---|---|---|---|---|
| sp\|Q8WXG9\|GPR98_HUMAN G-protein coupled receptor 98 OS=Homo sapiens GN=GPR98 PE=1 SV=2 | 3491 | IWEMGQsSFRY | 0.198 | -0.185 | -0.902 | -0.296 |
| sp\|Q8WXG9\|GPR98_HUMAN G-protein coupled receptor 98 OS=Homo sapiens GN=GPR98 PE=1 SV=2 | 3492 | WEMGQSsFRYF | 0.073 | -0.185 | -1.2 | -0.437 |
| sp\|Q8WXG9\|GPR98_HUMAN G-protein coupled receptor 98 OS=Homo sapiens GN=GPR98 PE=1 SV=2 | 3498 | SFRYFQsVDFA | 0.32 | 0.168 | -0.348 | 0.047 |
| sp\|Q8WXG9\|GPR98_HUMAN G-protein coupled receptor 98 OS=Homo sapiens GN=GPR98 PE=1 SV=2 | 3509 | AVNRIHsFTPA | 0.719 | 0.907 | 0.54 | 0.722 |
| sp\|Q8WXG9\|GPR98_HUMAN G-protein coupled receptor 98 OS=Homo sapiens GN=GPR98 PE=1 SV=2 | 3511 | NRIHSFtPASG | 0.131 | 0.067 | -0.789 | -0.197 |
| sp\|Q8WXG9\|GPR98_HUMAN G-protein coupled receptor 98 OS=Homo sapiens GN=GPR98 PE=1 SV=2 | 3514 | HSFTPAsGIAH | 0.107 | -0.195 | -0.942 | -0.343 |
| sp\|Q8WXG9\|GPR98_HUMAN G-protein coupled receptor 98 OS=Homo sapiens GN=GPR98 PE=1 SV=2 | 3527 | LIGQDMsALYC | 0.158 | 0.011 | -1.137 | -0.323 |
| sp\|Q8WXG9\|GPR98_HUMAN G-protein coupled receptor 98 OS=Homo sapiens GN=GPR98 PE=1 SV=2 | 3534 | ALYCWNsERNQ | 0.481 | 0.121 | -0.615 | -0.004 |
| sp\|Q8WXG9\|GPR98_HUMAN G-protein coupled receptor 98 OS=Homo sapiens GN=GPR98 PE=1 SV=2 | 3540 | SERNQFsFVLE | 0.211 | 0.204 | -0.713 | -0.099 |
| sp\|Q8WXG9\|GPR98_HUMAN G-protein coupled receptor 98 OS=Homo sapiens GN=GPR98 PE=1 SV=2 | 3547 | FVLEVPsAYDV | 0.195 | 0.066 | -1.065 | -0.268 |
| sp\|Q8WXG9\|GPR98_HUMAN G-protein coupled receptor 98 OS=Homo sapiens GN=GPR98 PE=1 SV=2 | 3553 | SAYDVAsVTVK | 0.167 | -0.13 | -1.233 | -0.399 |
| sp\|Q8WXG9\|GPR98_HUMAN G-protein coupled receptor 98 OS=Homo sapiens GN=GPR98 PE=1 SV=2 | 3555 | YDVASVtVKSL | 0.2 | 0.062 | -0.47 | -0.069 |
| sp\|Q8WXG9\|GPR98_HUMAN G-protein coupled receptor 98 OS=Homo sapiens GN=GPR98 PE=1 SV=2 | 3558 | ASVTVKsLNSS | 0.25 | -0.049 | -0.59 | -0.13 |
| sp\|Q8WXG9\|GPR98_HUMAN G-protein coupled receptor 98 OS=Homo sapiens GN=GPR98 PE=1 SV=2 | 3561 | TVKSLNsSKNL | 0.12 | -0.061 | -1.164 | -0.368 |
| sp\|Q8WXG9\|GPR98_HUMAN G-protein coupled receptor 98 OS=Homo sapiens GN=GPR98 PE=1 SV=2 | 3562 | VKSLNSsKNLI | 0.337 | 0.001 | -0.249 | 0.03 |
| sp\|Q8WXG9\|GPR98_HUMAN G-protein coupled receptor 98 OS=Homo sapiens GN=GPR98 PE=1 SV=2 | 3573 | ALVGAHsHIYE | 0.398 | 0.161 | -0.413 | 0.049 |
| sp\|Q8WXG9\|GPR98_HUMAN G-protein coupled receptor 98 OS=Homo sapiens GN=GPR98 PE=1 SV=2 | 3582 | YELAYIsSHSD | 0.175 | -0.117 | -0.897 | -0.28 |
| sp\|Q8WXG9\|GPR98_HUMAN G-protein coupled receptor 98 OS=Homo sapiens GN=GPR98 PE=1 SV=2 | 3583 | ELAYISsHSDF | 0.173 | -0.002 | -0.431 | -0.087 |
| sp\|Q8WXG9\|GPR98_HUMAN G-protein coupled receptor 98 OS=Homo sapiens GN=GPR98 PE=1 SV=2 | 3585 | AYISSHsDFIP | 0.493 | 0.27 | -0.371 | 0.131 |
| sp\|Q8WXG9\|GPR98_HUMAN G-protein coupled receptor 98 OS=Homo sapiens GN=GPR98 PE=1 SV=2 | 3590 | HSDFIPsSGEL | 0.206 | -0.035 | -0.599 | -0.143 |
| sp\|Q8WXG9\|GPR98_HUMAN G-protein coupled receptor 98 OS=Homo sapiens GN=GPR98 PE=1 SV=2 | 3591 | SDFIPSsGELI | 0.047 | -0.303 | -1.44 | -0.565 |
| sp\|Q8WXG9\|GPR98_HUMAN G-protein coupled receptor 98 OS=Homo sapiens GN=GPR98 PE=1 SV=2 | 3604 | PGEREAtIAVN | 0.59 | 0.775 | 0.141 | 0.502 |
| sp\|Q8WXG9\|GPR98_HUMAN G-protein coupled receptor 98 OS=Homo sapiens GN=GPR98 PE=1 SV=2 | 3613 | VNILDDtVPEK | 0.376 | 0.229 | -0.494 | 0.037 |
| sp\|Q8WXG9\|GPR98_HUMAN G-protein coupled receptor 98 OS=Homo sapiens GN=GPR98 PE=1 SV=2 | 3620 | VPEKEEsFKVQ | 0.231 | -0.129 | -0.969 | -0.289 |
| sp\|Q8WXG9\|GPR98_HUMAN G-protein coupled receptor 98 OS=Homo sapiens GN=GPR98 PE=1 SV=2 | 3639 | EIGINDsVTIT | 0.147 | -0.119 | -1.011 | -0.328 |
| sp\|Q8WXG9\|GPR98_HUMAN G-protein coupled receptor 98 OS=Homo sapiens GN=GPR98 PE=1 SV=2 | 3641 | GINDSVtITIL | 0.648 | 0.288 | -0.118 | 0.273 |
| sp\|Q8WXG9\|GPR98_HUMAN G-protein coupled receptor 98 OS=Homo sapiens GN=GPR98 PE=1 SV=2 | 3643 | NDSVTItILSN | 0.031 | -0.261 | -1.668 | -0.633 |
| sp\|Q8WXG9\|GPR98_HUMAN G-protein coupled receptor 98 OS=Homo sapiens GN=GPR98 PE=1 SV=2 | 3646 | VTITILsNDDA | 0.184 | -0.102 | -0.889 | -0.269 |
| sp\|Q8WXG9\|GPR98_HUMAN G-protein coupled receptor 98 OS=Homo sapiens GN=GPR98 PE=1 SV=2 | 3660 | VAFAQNsLYKQ | 0.201 | -0.07 | -0.978 | -0.282 |
| sp\|Q8WXG9\|GPR98_HUMAN G-protein coupled receptor 98 OS=Homo sapiens GN=GPR98 PE=1 SV=2 | 3672 | EEMEQDsLVTL | 0.089 | -0.044 | -1.199 | -0.385 |
| sp\|Q8WXG9\|GPR98_HUMAN G-protein coupled receptor 98 OS=Homo sapiens GN=GPR98 PE=1 SV=2 | 3675 | EQDSLVtLNVE | 0.235 | -0.081 | -0.564 | -0.137 |
| sp\|Q8WXG9\|GPR98_HUMAN G-protein coupled receptor 98 OS=Homo sapiens GN=GPR98 PE=1 SV=2 | 3684 | VERLKGtYGRI | 0.236 | 0 | -0.909 | -0.224 |
| sp\|Q8WXG9\|GPR98_HUMAN G-protein coupled receptor 98 OS=Homo sapiens GN=GPR98 PE=1 SV=2 | 3689 | GTYGRItIAWE | 0.239 | 0.004 | -0.636 | -0.131 |
| sp\|Q8WXG9\|GPR98_HUMAN G-protein coupled receptor 98 OS=Homo sapiens GN=GPR98 PE=1 SV=2 | 3697 | AWEADGsISDI | 0.144 | -0.194 | -0.952 | -0.334 |
| sp\|Q8WXG9\|GPR98_HUMAN G-protein coupled receptor 98 OS=Homo sapiens GN=GPR98 PE=1 SV=2 | 3699 | EADGSIsDIFP | 0.115 | -0.024 | -0.858 | -0.256 |
| sp\|Q8WXG9\|GPR98_HUMAN G-protein coupled receptor 98 OS=Homo sapiens GN=GPR98 PE=1 SV=2 | 3704 | ISDIFPtSGVI | 0.225 | -0.111 | -0.792 | -0.226 |
| sp\|Q8WXG9\|GPR98_HUMAN G-protein coupled receptor 98 OS=Homo sapiens GN=GPR98 PE=1 SV=2 | 3705 | SDIFPTsGVIL | 0.064 | -0.239 | -1.294 | -0.49 |
| sp\|Q8WXG9\|GPR98_HUMAN G-protein coupled receptor 98 OS=Homo sapiens GN=GPR98 PE=1 SV=2 | 3711 | SGVILFtEGQV | 0.148 | -0.004 | -1.038 | -0.298 |
| sp\|Q8WXG9\|GPR98_HUMAN G-protein coupled receptor 98 OS=Homo sapiens GN=GPR98 PE=1 SV=2 | 3717 | TEGQVLsTITL | 0.075 | -0.167 | -1.184 | -0.425 |
| sp\|Q8WXG9\|GPR98_HUMAN G-protein coupled receptor 98 OS=Homo sapiens GN=GPR98 PE=1 SV=2 | 3718 | EGQVLStITLT | 0.04 | -0.253 | -1.417 | -0.543 |
| sp\|Q8WXG9\|GPR98_HUMAN G-protein coupled receptor 98 OS=Homo sapiens GN=GPR98 PE=1 SV=2 | 3720 | QVLSTItLTIL | 0.278 | 0.037 | -0.45 | -0.045 |
| sp\|Q8WXG9\|GPR98_HUMAN G-protein coupled receptor 98 OS=Homo sapiens GN=GPR98 PE=1 SV=2 | 3722 | LSTITLtILAD | 0.069 | -0.076 | -0.92 | -0.309 |
| sp\|Q8WXG9\|GPR98_HUMAN G-protein coupled receptor 98 OS=Homo sapiens GN=GPR98 PE=1 SV=2 | 3732 | DNIPELsEVVI | 0.141 | -0.089 | -1.089 | -0.346 |
| sp\|Q8WXG9\|GPR98_HUMAN G-protein coupled receptor 98 OS=Homo sapiens GN=GPR98 PE=1 SV=2 | 3738 | SEVVIVtLTRI | 0.339 | -0.074 | -0.634 | -0.123 |
| sp\|Q8WXG9\|GPR98_HUMAN G-protein coupled receptor 98 OS=Homo sapiens GN=GPR98 PE=1 SV=2 | 3740 | VVIVTLtRITT | 0.026 | -0.254 | -1.59 | -0.606 |
| sp\|Q8WXG9\|GPR98_HUMAN G-protein coupled receptor 98 OS=Homo sapiens GN=GPR98 PE=1 SV=2 | 3743 | VTLTRItTEGV | 0.312 | -0.041 | -0.844 | -0.191 |
| sp\|Q8WXG9\|GPR98_HUMAN G-protein coupled receptor 98 OS=Homo sapiens GN=GPR98 PE=1 SV=2 | 3744 | TLTRITtEGVE | 0.797 | 1.04 | 0.947 | 0.928 |
| sp\|Q8WXG9\|GPR98_HUMAN G-protein coupled receptor 98 OS=Homo sapiens GN=GPR98 PE=1 SV=2 | 3750 | TEGVEDsYKGA | 0.042 | -0.407 | -1.741 | -0.702 |
| sp\|Q8WXG9\|GPR98_HUMAN G-protein coupled receptor 98 OS=Homo sapiens GN=GPR98 PE=1 SV=2 | 3755 | DSYKGAtIDQD | 0.318 | -0.09 | -0.526 | -0.099 |

| | | | | | | |
|---|---|---|---|---|---|---|
| sp\|Q8WXG9\|GPR98_HUMAN G-protein coupled receptor 98 OS=Homo sapiens GN=GPR98 PE=1 SV=2 | 3761 | TIDQDRsKSVI | 0.146 | -0.056 | -0.916 | -0.275 |
| sp\|Q8WXG9\|GPR98_HUMAN G-protein coupled receptor 98 OS=Homo sapiens GN=GPR98 PE=1 SV=2 | 3763 | DQDRSKsVITT | 0.747 | 1.004 | 0.931 | 0.894 |
| sp\|Q8WXG9\|GPR98_HUMAN G-protein coupled receptor 98 OS=Homo sapiens GN=GPR98 PE=1 SV=2 | 3766 | RSKSVItTLPN | 0.169 | -0.174 | -1.064 | -0.356 |
| sp\|Q8WXG9\|GPR98_HUMAN G-protein coupled receptor 98 OS=Homo sapiens GN=GPR98 PE=1 SV=2 | 3767 | SKSVITtLPND | 0.695 | 0.592 | 0.427 | 0.571 |
| sp\|Q8WXG9\|GPR98_HUMAN G-protein coupled receptor 98 OS=Homo sapiens GN=GPR98 PE=1 SV=2 | 3772 | TTLPNDsPFGL | 0.085 | -0.513 | -1.246 | -0.558 |
| sp\|Q8WXG9\|GPR98_HUMAN G-protein coupled receptor 98 OS=Homo sapiens GN=GPR98 PE=1 SV=2 | 3783 | VGWRAAsVFIR | 0.788 | 1.048 | 0.775 | 0.87 |
| sp\|Q8WXG9\|GPR98_HUMAN G-protein coupled receptor 98 OS=Homo sapiens GN=GPR98 PE=1 SV=2 | 3795 | AEPKENtTTLQ | 0.241 | -0.156 | -1.309 | -0.408 |
| sp\|Q8WXG9\|GPR98_HUMAN G-protein coupled receptor 98 OS=Homo sapiens GN=GPR98 PE=1 SV=2 | 3796 | EPKENTtTLQL | 0.099 | -0.109 | -0.922 | -0.311 |
| sp\|Q8WXG9\|GPR98_HUMAN G-protein coupled receptor 98 OS=Homo sapiens GN=GPR98 PE=1 SV=2 | 3797 | PKENTTtLQLQ | 0.405 | 0.155 | -0.01 | 0.183 |
| sp\|Q8WXG9\|GPR98_HUMAN G-protein coupled receptor 98 OS=Homo sapiens GN=GPR98 PE=1 SV=2 | 3831 | HVDNQAtENED | 0.244 | 0.164 | -0.409 | 0 |
| sp\|Q8WXG9\|GPR98_HUMAN G-protein coupled receptor 98 OS=Homo sapiens GN=GPR98 PE=1 SV=2 | 3841 | DYVLQEtIIIM | 0.126 | -0.132 | -1.25 | -0.419 |
| sp\|Q8WXG9\|GPR98_HUMAN G-protein coupled receptor 98 OS=Homo sapiens GN=GPR98 PE=1 SV=2 | 3857 | EAHAEVsILPD | 0.087 | -0.31 | -1.279 | -0.501 |
| sp\|Q8WXG9\|GPR98_HUMAN G-protein coupled receptor 98 OS=Homo sapiens GN=GPR98 PE=1 SV=2 | 3873 | EEGFIVtITEV | 0.222 | -0.098 | -0.939 | -0.272 |
| sp\|Q8WXG9\|GPR98_HUMAN G-protein coupled receptor 98 OS=Homo sapiens GN=GPR98 PE=1 SV=2 | 3875 | GFIVTItEVNL | 0.197 | 0.217 | -0.751 | -0.112 |
| sp\|Q8WXG9\|GPR98_HUMAN G-protein coupled receptor 98 OS=Homo sapiens GN=GPR98 PE=1 SV=2 | 3882 | EVNLVNsDFST | 0.106 | -0.07 | -1.269 | -0.411 |
| sp\|Q8WXG9\|GPR98_HUMAN G-protein coupled receptor 98 OS=Homo sapiens GN=GPR98 PE=1 SV=2 | 3885 | LVNSDFsTGQP | 0.1 | -0.17 | -1.032 | -0.367 |
| sp\|Q8WXG9\|GPR98_HUMAN G-protein coupled receptor 98 OS=Homo sapiens GN=GPR98 PE=1 SV=2 | 3886 | VNSDFStGQPS | 0.047 | -0.398 | -1.567 | -0.639 |
| sp\|Q8WXG9\|GPR98_HUMAN G-protein coupled receptor 98 OS=Homo sapiens GN=GPR98 PE=1 SV=2 | 3890 | FSTGQPsVRRP | 0.087 | -0.273 | -1.244 | -0.477 |
| sp\|Q8WXG9\|GPR98_HUMAN G-protein coupled receptor 98 OS=Homo sapiens GN=GPR98 PE=1 SV=2 | 3918 | IFMFHVtRGAG | 0.623 | 0.157 | 0.09 | 0.29 |
| sp\|Q8WXG9\|GPR98_HUMAN G-protein coupled receptor 98 OS=Homo sapiens GN=GPR98 PE=1 SV=2 | 3926 | GAGEVItAYEV | 0.177 | -0.029 | -1.343 | -0.398 |
| sp\|Q8WXG9\|GPR98_HUMAN G-protein coupled receptor 98 OS=Homo sapiens GN=GPR98 PE=1 SV=2 | 3947 | VVRLAGsFGAV | 0.584 | 0.374 | -0.136 | 0.274 |
| sp\|Q8WXG9\|GPR98_HUMAN G-protein coupled receptor 98 OS=Homo sapiens GN=GPR98 PE=1 SV=2 | 3958 | NVYWKAsPDSA | 0.06 | -0.397 | -1.237 | -0.525 |
| sp\|Q8WXG9\|GPR98_HUMAN G-protein coupled receptor 98 OS=Homo sapiens GN=GPR98 PE=1 SV=2 | 3961 | WKASPDsAGLE | 0.258 | -0.148 | -0.676 | -0.189 |
| sp\|Q8WXG9\|GPR98_HUMAN G-protein coupled receptor 98 OS=Homo sapiens GN=GPR98 PE=1 SV=2 | 3970 | LEDFKPsHGIL | 0.209 | -0.094 | -0.877 | -0.254 |
| sp\|Q8WXG9\|GPR98_HUMAN G-protein coupled receptor 98 OS=Homo sapiens GN=GPR98 PE=1 SV=2 | 3982 | FADKQVtAMIE | 0.386 | 0.02 | -0.808 | -0.134 |
| sp\|Q8WXG9\|GPR98_HUMAN G-protein coupled receptor 98 OS=Homo sapiens GN=GPR98 PE=1 SV=2 | 3988 | TAMIEItIIDD | 0.16 | -0.064 | -0.754 | -0.219 |
| sp\|Q8WXG9\|GPR98_HUMAN G-protein coupled receptor 98 OS=Homo sapiens GN=GPR98 PE=1 SV=2 | 3998 | DAEFELtETFN | 0.173 | -0.165 | -1.306 | -0.433 |
| sp\|Q8WXG9\|GPR98_HUMAN G-protein coupled receptor 98 OS=Homo sapiens GN=GPR98 PE=1 SV=2 | 4000 | EFELTEtFNIS | 0.205 | 0.016 | -0.653 | -0.144 |
| sp\|Q8WXG9\|GPR98_HUMAN G-protein coupled receptor 98 OS=Homo sapiens GN=GPR98 PE=1 SV=2 | 4004 | TETFNIsLISV | 0.231 | -0.011 | -0.659 | -0.146 |
| sp\|Q8WXG9\|GPR98_HUMAN G-protein coupled receptor 98 OS=Homo sapiens GN=GPR98 PE=1 SV=2 | 4007 | FNISLIsVAGG | 0.108 | -0.207 | -1.104 | -0.401 |
| sp\|Q8WXG9\|GPR98_HUMAN G-protein coupled receptor 98 OS=Homo sapiens GN=GPR98 PE=1 SV=2 | 4021 | GDDVVVtVVIP | 0.133 | -0.198 | -1.225 | -0.43 |
| sp\|Q8WXG9\|GPR98_HUMAN G-protein coupled receptor 98 OS=Homo sapiens GN=GPR98 PE=1 SV=2 | 4029 | VIPQNDsPFGV | 0.1 | -0.456 | -1.347 | -0.568 |
| sp\|Q8WXG9\|GPR98_HUMAN G-protein coupled receptor 98 OS=Homo sapiens GN=GPR98 PE=1 SV=2 | 4040 | FGFEEKtVMID | 0.321 | 0.086 | -0.5 | -0.031 |
| sp\|Q8WXG9\|GPR98_HUMAN G-protein coupled receptor 98 OS=Homo sapiens GN=GPR98 PE=1 SV=2 | 4046 | TVMIDEsLSSD | 0.08 | -0.086 | -1.091 | -0.366 |
| sp\|Q8WXG9\|GPR98_HUMAN G-protein coupled receptor 98 OS=Homo sapiens GN=GPR98 PE=1 SV=2 | 4048 | MIDESLsSDDP | 0.257 | 0.244 | -0.496 | 0.002 |
| sp\|Q8WXG9\|GPR98_HUMAN G-protein coupled receptor 98 OS=Homo sapiens GN=GPR98 PE=1 SV=2 | 4049 | IDESLSsDDPD | 0.112 | -0.283 | -1.144 | -0.438 |
| sp\|Q8WXG9\|GPR98_HUMAN G-protein coupled receptor 98 OS=Homo sapiens GN=GPR98 PE=1 SV=2 | 4054 | SSDDPDsYVTL | 0.046 | -0.384 | -1.703 | -0.68 |
| sp\|Q8WXG9\|GPR98_HUMAN G-protein coupled receptor 98 OS=Homo sapiens GN=GPR98 PE=1 SV=2 | 4057 | DPDSYVtLTVV | 0.499 | -0.044 | -0.768 | -0.104 |
| sp\|Q8WXG9\|GPR98_HUMAN G-protein coupled receptor 98 OS=Homo sapiens GN=GPR98 PE=1 SV=2 | 4059 | DSYVTLtVVRS | 0.043 | -0.245 | -1.685 | -0.629 |
| sp\|Q8WXG9\|GPR98_HUMAN G-protein coupled receptor 98 OS=Homo sapiens GN=GPR98 PE=1 SV=2 | 4063 | TLTVVRsPGGK | 0.102 | -0.345 | -1.181 | -0.475 |
| sp\|Q8WXG9\|GPR98_HUMAN G-protein coupled receptor 98 OS=Homo sapiens GN=GPR98 PE=1 SV=2 | 4069 | SPGGKGtVRLE | 0.042 | -0.366 | -1.737 | -0.687 |
| sp\|Q8WXG9\|GPR98_HUMAN G-protein coupled receptor 98 OS=Homo sapiens GN=GPR98 PE=1 SV=2 | 4075 | TVRLEWtIDEK | 0.331 | 0.075 | -0.6 | -0.065 |
| sp\|Q8WXG9\|GPR98_HUMAN G-protein coupled receptor 98 OS=Homo sapiens GN=GPR98 PE=1 SV=2 | 4085 | KAKHNLsPLNG | 0.055 | -0.427 | -1.54 | -0.637 |
| sp\|Q8WXG9\|GPR98_HUMAN G-protein coupled receptor 98 OS=Homo sapiens GN=GPR98 PE=1 SV=2 | 4090 | LSPLNGtLHFD | 0.159 | -0.18 | -0.865 | -0.295 |
| sp\|Q8WXG9\|GPR98_HUMAN G-protein coupled receptor 98 OS=Homo sapiens GN=GPR98 PE=1 SV=2 | 4096 | TLHFDEtESQK | 0.138 | -0.131 | -1.088 | -0.36 |
| sp\|Q8WXG9\|GPR98_HUMAN G-protein coupled receptor 98 OS=Homo sapiens GN=GPR98 PE=1 SV=2 | 4098 | HFDETEsQKTI | 0.082 | -0.07 | -1.135 | -0.374 |
| sp\|Q8WXG9\|GPR98_HUMAN G-protein coupled receptor 98 OS=Homo sapiens GN=GPR98 PE=1 SV=2 | 4101 | ETESQKtIVLH | 0.073 | -0.247 | -1.28 | -0.485 |
| sp\|Q8WXG9\|GPR98_HUMAN G-protein coupled receptor 98 OS=Homo sapiens GN=GPR98 PE=1 SV=2 | 4106 | KTIVLHtLQDT | 0.336 | 0.11 | -0.574 | -0.043 |
| sp\|Q8WXG9\|GPR98_HUMAN G-protein coupled receptor 98 OS=Homo sapiens GN=GPR98 PE=1 SV=2 | 4110 | LHTLQDtVLEE | 0.091 | -0.178 | -1.621 | -0.569 |

| | | | | | | |
|---|---|---|---|---|---|---|
| sp\|Q8WXG9\|GPR98_HUMAN G-protein coupled receptor 98 OS=Homo sapiens GN=GPR98 PE=1 SV=2 | 4119 | EEDRRFtIQLI | 0.719 | 1.014 | 0.668 | 0.8 |
| sp\|Q8WXG9\|GPR98_HUMAN G-protein coupled receptor 98 OS=Homo sapiens GN=GPR98 PE=1 SV=2 | 4124 | FTIQLIsIDEV | 0.295 | 0.021 | -0.809 | -0.164 |
| sp\|Q8WXG9\|GPR98_HUMAN G-protein coupled receptor 98 OS=Homo sapiens GN=GPR98 PE=1 SV=2 | 4131 | IDEVEIsPVKG | 0.041 | -0.539 | -1.668 | -0.722 |
| sp\|Q8WXG9\|GPR98_HUMAN G-protein coupled receptor 98 OS=Homo sapiens GN=GPR98 PE=1 SV=2 | 4136 | ISPVKGsASII | 0.066 | -0.297 | -1.504 | -0.578 |
| sp\|Q8WXG9\|GPR98_HUMAN G-protein coupled receptor 98 OS=Homo sapiens GN=GPR98 PE=1 SV=2 | 4138 | PVKGSAsIIIR | 0.414 | 0.243 | 0.079 | 0.245 |
| sp\|Q8WXG9\|GPR98_HUMAN G-protein coupled receptor 98 OS=Homo sapiens GN=GPR98 PE=1 SV=2 | 4148 | RGDKRAsGEVG | 0.488 | 0.171 | -0.33 | 0.11 |
| sp\|Q8WXG9\|GPR98_HUMAN G-protein coupled receptor 98 OS=Homo sapiens GN=GPR98 PE=1 SV=2 | 4156 | EVGIAPsSRHI | 0.098 | -0.145 | -0.859 | -0.302 |
| sp\|Q8WXG9\|GPR98_HUMAN G-protein coupled receptor 98 OS=Homo sapiens GN=GPR98 PE=1 SV=2 | 4157 | VGIAPSsRHIL | 0.101 | -0.283 | -1.171 | -0.451 |
| sp\|Q8WXG9\|GPR98_HUMAN G-protein coupled receptor 98 OS=Homo sapiens GN=GPR98 PE=1 SV=2 | 4166 | ILIGEPsAKYN | 0.22 | -0.025 | -0.941 | -0.249 |
| sp\|Q8WXG9\|GPR98_HUMAN G-protein coupled receptor 98 OS=Homo sapiens GN=GPR98 PE=1 SV=2 | 4172 | SAKYNGtAIIS | 0.113 | -0.147 | -1.089 | -0.374 |
| sp\|Q8WXG9\|GPR98_HUMAN G-protein coupled receptor 98 OS=Homo sapiens GN=GPR98 PE=1 SV=2 | 4176 | NGTAIIsLVRG | 0.363 | -0.05 | -0.254 | 0.02 |
| sp\|Q8WXG9\|GPR98_HUMAN G-protein coupled receptor 98 OS=Homo sapiens GN=GPR98 PE=1 SV=2 | 4188 | GILGEVtVFWR | 0.417 | 0.102 | -0.27 | 0.083 |
| sp\|Q8WXG9\|GPR98_HUMAN G-protein coupled receptor 98 OS=Homo sapiens GN=GPR98 PE=1 SV=2 | 4197 | WRIFPPsVGEF | 0.241 | 0.124 | -0.451 | -0.029 |
| sp\|Q8WXG9\|GPR98_HUMAN G-protein coupled receptor 98 OS=Homo sapiens GN=GPR98 PE=1 SV=2 | 4204 | VGEFAEtSGKL | 0.251 | -0.031 | -0.72 | -0.167 |
| sp\|Q8WXG9\|GPR98_HUMAN G-protein coupled receptor 98 OS=Homo sapiens GN=GPR98 PE=1 SV=2 | 4205 | GEFAETsGKLT | 0.133 | -0.185 | -1.136 | -0.396 |
| sp\|Q8WXG9\|GPR98_HUMAN G-protein coupled receptor 98 OS=Homo sapiens GN=GPR98 PE=1 SV=2 | 4209 | ETSGKLtMRDE | 0.091 | -0.138 | -1.173 | -0.407 |
| sp\|Q8WXG9\|GPR98_HUMAN G-protein coupled receptor 98 OS=Homo sapiens GN=GPR98 PE=1 SV=2 | 4215 | TMRDEQsAVIV | 0.322 | 0.305 | -0.988 | -0.12 |
| sp\|Q8WXG9\|GPR98_HUMAN G-protein coupled receptor 98 OS=Homo sapiens GN=GPR98 PE=1 SV=2 | 4233 | DIPEEKsFYEF | 0.228 | 0.049 | -0.772 | -0.165 |
| sp\|Q8WXG9\|GPR98_HUMAN G-protein coupled receptor 98 OS=Homo sapiens GN=GPR98 PE=1 SV=2 | 4240 | FYEFQLtAVSE | 0.106 | -0.003 | -1.283 | -0.393 |
| sp\|Q8WXG9\|GPR98_HUMAN G-protein coupled receptor 98 OS=Homo sapiens GN=GPR98 PE=1 SV=2 | 4243 | FQLTAVsEGGV | 0.584 | 0.175 | -0.13 | 0.21 |
| sp\|Q8WXG9\|GPR98_HUMAN G-protein coupled receptor 98 OS=Homo sapiens GN=GPR98 PE=1 SV=2 | 4249 | SEGGVLsESSS | 0.024 | -0.263 | -1.979 | -0.739 |
| sp\|Q8WXG9\|GPR98_HUMAN G-protein coupled receptor 98 OS=Homo sapiens GN=GPR98 PE=1 SV=2 | 4251 | GGVLSEsSSTA | 0.198 | 0 | -0.864 | -0.222 |
| sp\|Q8WXG9\|GPR98_HUMAN G-protein coupled receptor 98 OS=Homo sapiens GN=GPR98 PE=1 SV=2 | 4252 | GVLSESsSTAN | 0.145 | -0.126 | -1.159 | -0.38 |
| sp\|Q8WXG9\|GPR98_HUMAN G-protein coupled receptor 98 OS=Homo sapiens GN=GPR98 PE=1 SV=2 | 4253 | VLSESSsTANI | 0.234 | 0.145 | -0.604 | -0.075 |
| sp\|Q8WXG9\|GPR98_HUMAN G-protein coupled receptor 98 OS=Homo sapiens GN=GPR98 PE=1 SV=2 | 4254 | LSESSStANIT | 0.227 | -0.016 | -0.563 | -0.117 |
| sp\|Q8WXG9\|GPR98_HUMAN G-protein coupled receptor 98 OS=Homo sapiens GN=GPR98 PE=1 SV=2 | 4258 | SSTANItVVAS | 0.16 | -0.127 | -0.783 | -0.25 |
| sp\|Q8WXG9\|GPR98_HUMAN G-protein coupled receptor 98 OS=Homo sapiens GN=GPR98 PE=1 SV=2 | 4262 | NITVVAsDSPY | 0.228 | 0.008 | -0.977 | -0.247 |
| sp\|Q8WXG9\|GPR98_HUMAN G-protein coupled receptor 98 OS=Homo sapiens GN=GPR98 PE=1 SV=2 | 4264 | TVVASDsPYGR | 0.112 | -0.279 | -1.015 | -0.394 |
| sp\|Q8WXG9\|GPR98_HUMAN G-protein coupled receptor 98 OS=Homo sapiens GN=GPR98 PE=1 SV=2 | 4272 | YGRFAFsHEQL | 0.587 | 0.368 | 0.196 | 0.384 |
| sp\|Q8WXG9\|GPR98_HUMAN G-protein coupled receptor 98 OS=Homo sapiens GN=GPR98 PE=1 SV=2 | 4279 | HEQLRVsEAQR | 0.508 | 0.225 | -0.22 | 0.171 |
| sp\|Q8WXG9\|GPR98_HUMAN G-protein coupled receptor 98 OS=Homo sapiens GN=GPR98 PE=1 SV=2 | 4287 | AQRVNItIIRS | 0.169 | -0.02 | -0.728 | -0.193 |
| sp\|Q8WXG9\|GPR98_HUMAN G-protein coupled receptor 98 OS=Homo sapiens GN=GPR98 PE=1 SV=2 | 4291 | NITIIRsSGDF | 0.212 | 0.101 | -0.555 | -0.081 |
| sp\|Q8WXG9\|GPR98_HUMAN G-protein coupled receptor 98 OS=Homo sapiens GN=GPR98 PE=1 SV=2 | 4292 | ITIIRSsGDFG | 0.198 | -0.025 | -0.823 | -0.217 |
| sp\|Q8WXG9\|GPR98_HUMAN G-protein coupled receptor 98 OS=Homo sapiens GN=GPR98 PE=1 SV=2 | 4304 | VRLWYKtMSGT | 0.277 | 0.151 | -0.365 | 0.021 |
| sp\|Q8WXG9\|GPR98_HUMAN G-protein coupled receptor 98 OS=Homo sapiens GN=GPR98 PE=1 SV=2 | 4306 | LWYKTMsGTAE | 0.317 | 0.177 | -0.317 | 0.059 |
| sp\|Q8WXG9\|GPR98_HUMAN G-protein coupled receptor 98 OS=Homo sapiens GN=GPR98 PE=1 SV=2 | 4308 | YKTMSGtAEAG | 0.493 | 0.262 | -0.028 | 0.242 |
| sp\|Q8WXG9\|GPR98_HUMAN G-protein coupled receptor 98 OS=Homo sapiens GN=GPR98 PE=1 SV=2 | 4332 | AGEMRKsLHVE | 0.653 | 0.176 | 0.173 | 0.334 |
| sp\|Q8WXG9\|GPR98_HUMAN G-protein coupled receptor 98 OS=Homo sapiens GN=GPR98 PE=1 SV=2 | 4350 | EGPEEFsLTIT | 0.153 | -0.123 | -0.9 | -0.29 |
| sp\|Q8WXG9\|GPR98_HUMAN G-protein coupled receptor 98 OS=Homo sapiens GN=GPR98 PE=1 SV=2 | 4352 | PEEFSLtITKV | 0.248 | 0.064 | -0.824 | -0.171 |
| sp\|Q8WXG9\|GPR98_HUMAN G-protein coupled receptor 98 OS=Homo sapiens GN=GPR98 PE=1 SV=2 | 4354 | EFSLTItKVEL | 0.161 | 0.027 | -0.657 | -0.156 |
| sp\|Q8WXG9\|GPR98_HUMAN G-protein coupled receptor 98 OS=Homo sapiens GN=GPR98 PE=1 SV=2 | 4366 | GRGYDFtIQEN | 0.262 | 0.258 | -0.442 | 0.026 |
| sp\|Q8WXG9\|GPR98_HUMAN G-protein coupled receptor 98 OS=Homo sapiens GN=GPR98 PE=1 SV=2 | 4384 | PEIGNIsIVRI | 0.213 | -0.082 | -0.739 | -0.203 |
| sp\|Q8WXG9\|GPR98_HUMAN G-protein coupled receptor 98 OS=Homo sapiens GN=GPR98 PE=1 SV=2 | 4407 | EFDPKYtAFEV | 0.148 | -0.012 | -0.935 | -0.266 |
| sp\|Q8WXG9\|GPR98_HUMAN G-protein coupled receptor 98 OS=Homo sapiens GN=GPR98 PE=1 SV=2 | 4428 | VVRLHGtYGYV | 0.276 | 0.118 | -0.959 | -0.188 |
| sp\|Q8WXG9\|GPR98_HUMAN G-protein coupled receptor 98 OS=Homo sapiens GN=GPR98 PE=1 SV=2 | 4433 | GTYGYVtADFI | 0.42 | 0.064 | -0.424 | 0.02 |
| sp\|Q8WXG9\|GPR98_HUMAN G-protein coupled receptor 98 OS=Homo sapiens GN=GPR98 PE=1 SV=2 | 4438 | VTADFIsQSSS | 0.062 | -0.306 | -1.736 | -0.66 |
| sp\|Q8WXG9\|GPR98_HUMAN G-protein coupled receptor 98 OS=Homo sapiens GN=GPR98 PE=1 SV=2 | 4440 | ADFISQsSSAS | 0.127 | 0.039 | -0.873 | -0.236 |
| sp\|Q8WXG9\|GPR98_HUMAN G-protein coupled receptor 98 OS=Homo sapiens GN=GPR98 PE=1 SV=2 | 4441 | DFISQSsSASP | 0.028 | -0.352 | -1.927 | -0.75 |
| sp\|Q8WXG9\|GPR98_HUMAN G-protein coupled receptor 98 OS=Homo sapiens GN=GPR98 PE=1 SV=2 | 4442 | FISQSSsASPG | 0.267 | 0.173 | -0.519 | -0.026 |

| | | | | | | |
|---|---|---|---|---|---|---|
| sp\|Q8WXG9\|GPR98_HUMAN G-protein coupled receptor 98 OS=Homo sapiens GN=GPR98 PE=1 SV=2 | 4444 | SQSSSAsPGGV | 0.157 | -0.233 | -1.006 | -0.361 |
| sp\|Q8WXG9\|GPR98_HUMAN G-protein coupled receptor 98 OS=Homo sapiens GN=GPR98 PE=1 SV=2 | 4455 | DYILHGsTVTF | 0.153 | -0.077 | -1.25 | -0.391 |
| sp\|Q8WXG9\|GPR98_HUMAN G-protein coupled receptor 98 OS=Homo sapiens GN=GPR98 PE=1 SV=2 | 4456 | YILHGStVTFQ | 0.075 | -0.236 | -1.292 | -0.484 |
| sp\|Q8WXG9\|GPR98_HUMAN G-protein coupled receptor 98 OS=Homo sapiens GN=GPR98 PE=1 SV=2 | 4458 | LHGSTVtFQHG | 0.151 | -0.066 | -0.885 | -0.267 |
| sp\|Q8WXG9\|GPR98_HUMAN G-protein coupled receptor 98 OS=Homo sapiens GN=GPR98 PE=1 SV=2 | 4466 | QHGQNLsFINI | 0.175 | 0.042 | -0.708 | -0.164 |
| sp\|Q8WXG9\|GPR98_HUMAN G-protein coupled receptor 98 OS=Homo sapiens GN=GPR98 PE=1 SV=2 | 4471 | LSFINIsIIDD | 0.242 | -0.037 | -0.357 | -0.051 |
| sp\|Q8WXG9\|GPR98_HUMAN G-protein coupled receptor 98 OS=Homo sapiens GN=GPR98 PE=1 SV=2 | 4478 | IIDDNEsEFEE | 0.353 | 0.034 | -0.638 | -0.084 |
| sp\|Q8WXG9\|GPR98_HUMAN G-protein coupled receptor 98 OS=Homo sapiens GN=GPR98 PE=1 SV=2 | 4489 | PIEILLtGATG | 0.08 | -0.162 | -1.186 | -0.423 |
| sp\|Q8WXG9\|GPR98_HUMAN G-protein coupled receptor 98 OS=Homo sapiens GN=GPR98 PE=1 SV=2 | 4492 | ILLTGAtGGAV | 0.41 | 0.07 | -0.423 | 0.019 |
| sp\|Q8WXG9\|GPR98_HUMAN G-protein coupled receptor 98 OS=Homo sapiens GN=GPR98 PE=1 SV=2 | 4503 | LGRHLVsRIII | 0.407 | 0.092 | -0.197 | 0.101 |
| sp\|Q8WXG9\|GPR98_HUMAN G-protein coupled receptor 98 OS=Homo sapiens GN=GPR98 PE=1 SV=2 | 4510 | RIIIAKsDSPF | 0.27 | 0.152 | -0.583 | -0.054 |
| sp\|Q8WXG9\|GPR98_HUMAN G-protein coupled receptor 98 OS=Homo sapiens GN=GPR98 PE=1 SV=2 | 4512 | IIAKSDsPFGV | 0.392 | -0.089 | -0.424 | -0.04 |
| sp\|Q8WXG9\|GPR98_HUMAN G-protein coupled receptor 98 OS=Homo sapiens GN=GPR98 PE=1 SV=2 | 4523 | IRFLNQsKISI | 0.264 | 0.215 | -0.224 | 0.085 |
| sp\|Q8WXG9\|GPR98_HUMAN G-protein coupled receptor 98 OS=Homo sapiens GN=GPR98 PE=1 SV=2 | 4526 | LNQSKIsIANP | 0.047 | -0.315 | -1.461 | -0.576 |
| sp\|Q8WXG9\|GPR98_HUMAN G-protein coupled receptor 98 OS=Homo sapiens GN=GPR98 PE=1 SV=2 | 4532 | SIANPNsTMIL | 0.168 | -0.035 | -0.777 | -0.215 |
| sp\|Q8WXG9\|GPR98_HUMAN G-protein coupled receptor 98 OS=Homo sapiens GN=GPR98 PE=1 SV=2 | 4533 | IANPNStMILS | 0.076 | -0.139 | -1.18 | -0.414 |
| sp\|Q8WXG9\|GPR98_HUMAN G-protein coupled receptor 98 OS=Homo sapiens GN=GPR98 PE=1 SV=2 | 4537 | NSTMILsLVLE | 0.194 | -0.099 | -0.53 | -0.145 |
| sp\|Q8WXG9\|GPR98_HUMAN G-protein coupled receptor 98 OS=Homo sapiens GN=GPR98 PE=1 SV=2 | 4543 | SLVLERtGGLL | 0.147 | -0.004 | -1.161 | -0.339 |
| sp\|Q8WXG9\|GPR98_HUMAN G-protein coupled receptor 98 OS=Homo sapiens GN=GPR98 PE=1 SV=2 | 4556 | IQVNWEtVGPN | 0.188 | -0.046 | -0.607 | -0.155 |
| sp\|Q8WXG9\|GPR98_HUMAN G-protein coupled receptor 98 OS=Homo sapiens GN=GPR98 PE=1 SV=2 | 4561 | ETVGPNsQEAL | 0.134 | -0.064 | -0.998 | -0.309 |
| sp\|Q8WXG9\|GPR98_HUMAN G-protein coupled receptor 98 OS=Homo sapiens GN=GPR98 PE=1 SV=2 | 4577 | DIADPVsGLFY | 0.196 | -0.157 | -0.995 | -0.319 |
| sp\|Q8WXG9\|GPR98_HUMAN G-protein coupled receptor 98 OS=Homo sapiens GN=GPR98 PE=1 SV=2 | 4591 | GEGGVRtIILT | 0.068 | -0.149 | -1.348 | -0.476 |
| sp\|Q8WXG9\|GPR98_HUMAN G-protein coupled receptor 98 OS=Homo sapiens GN=GPR98 PE=1 SV=2 | 4595 | VRTIILtIYPH | 0.058 | -0.084 | -1.134 | -0.387 |
| sp\|Q8WXG9\|GPR98_HUMAN G-protein coupled receptor 98 OS=Homo sapiens GN=GPR98 PE=1 SV=2 | 4607 | EIEVEEtFIIK | 0.146 | -0.085 | -0.944 | -0.294 |
| sp\|Q8WXG9\|GPR98_HUMAN G-protein coupled receptor 98 OS=Homo sapiens GN=GPR98 PE=1 SV=2 | 4623 | GEAKLDsRAKD | 0.203 | -0.236 | -1.252 | -0.428 |
| sp\|Q8WXG9\|GPR98_HUMAN G-protein coupled receptor 98 OS=Homo sapiens GN=GPR98 PE=1 SV=2 | 4629 | SRAKDVtLTIQ | 0.627 | 0.188 | -0.135 | 0.227 |
| sp\|Q8WXG9\|GPR98_HUMAN G-protein coupled receptor 98 OS=Homo sapiens GN=GPR98 PE=1 SV=2 | 4631 | AKDVTLtIQEF | 0.082 | -0.104 | -1.098 | -0.373 |
| sp\|Q8WXG9\|GPR98_HUMAN G-protein coupled receptor 98 OS=Homo sapiens GN=GPR98 PE=1 SV=2 | 4648 | VQFAPEtLSKK | 0.096 | -0.283 | -1.03 | -0.406 |
| sp\|Q8WXG9\|GPR98_HUMAN G-protein coupled receptor 98 OS=Homo sapiens GN=GPR98 PE=1 SV=2 | 4650 | FAPETLsKKTY | 0.05 | -0.122 | -1.371 | -0.481 |
| sp\|Q8WXG9\|GPR98_HUMAN G-protein coupled receptor 98 OS=Homo sapiens GN=GPR98 PE=1 SV=2 | 4653 | ETLSKKtYSEP | 0.066 | -0.281 | -1.262 | -0.492 |
| sp\|Q8WXG9\|GPR98_HUMAN G-protein coupled receptor 98 OS=Homo sapiens GN=GPR98 PE=1 SV=2 | 4655 | LSKKTYsEPLA | 0.706 | 0.67 | 0.664 | 0.68 |
| sp\|Q8WXG9\|GPR98_HUMAN G-protein coupled receptor 98 OS=Homo sapiens GN=GPR98 PE=1 SV=2 | 4667 | EGPLLItFFVR | 0.151 | -0.057 | -0.805 | -0.237 |
| sp\|Q8WXG9\|GPR98_HUMAN G-protein coupled receptor 98 OS=Homo sapiens GN=GPR98 PE=1 SV=2 | 4676 | VRRVKGtFGEI | 0.482 | 0.444 | -0.078 | 0.283 |
| sp\|Q8WXG9\|GPR98_HUMAN G-protein coupled receptor 98 OS=Homo sapiens GN=GPR98 PE=1 SV=2 | 4687 | MVYWELsSEFD | 0.082 | -0.161 | -1.172 | -0.417 |
| sp\|Q8WXG9\|GPR98_HUMAN G-protein coupled receptor 98 OS=Homo sapiens GN=GPR98 PE=1 SV=2 | 4688 | VYWELSsEFDI | 0.175 | 0.156 | -0.684 | -0.118 |
| sp\|Q8WXG9\|GPR98_HUMAN G-protein coupled receptor 98 OS=Homo sapiens GN=GPR98 PE=1 SV=2 | 4693 | SSEFDItEDFL | 0.177 | -0.105 | -0.893 | -0.274 |
| sp\|Q8WXG9\|GPR98_HUMAN G-protein coupled receptor 98 OS=Homo sapiens GN=GPR98 PE=1 SV=2 | 4698 | ITEDFLsTSGF | 0.087 | -0.288 | -1.328 | -0.51 |
| sp\|Q8WXG9\|GPR98_HUMAN G-protein coupled receptor 98 OS=Homo sapiens GN=GPR98 PE=1 SV=2 | 4699 | TEDFLStSGFF | 0.049 | -0.307 | -1.7 | -0.653 |
| sp\|Q8WXG9\|GPR98_HUMAN G-protein coupled receptor 98 OS=Homo sapiens GN=GPR98 PE=1 SV=2 | 4700 | EDFLSTsGFFT | 0.097 | -0.074 | -0.836 | -0.271 |
| sp\|Q8WXG9\|GPR98_HUMAN G-protein coupled receptor 98 OS=Homo sapiens GN=GPR98 PE=1 SV=2 | 4704 | STSGFFtIADG | 0.128 | -0.165 | -1.103 | -0.38 |
| sp\|Q8WXG9\|GPR98_HUMAN G-protein coupled receptor 98 OS=Homo sapiens GN=GPR98 PE=1 SV=2 | 4710 | TIADGEsEASF | 0.05 | -0.151 | -1.415 | -0.505 |
| sp\|Q8WXG9\|GPR98_HUMAN G-protein coupled receptor 98 OS=Homo sapiens GN=GPR98 PE=1 SV=2 | 4713 | DGESEAsFDVH | 0.284 | -0.123 | -0.914 | -0.251 |
| sp\|Q8WXG9\|GPR98_HUMAN G-protein coupled receptor 98 OS=Homo sapiens GN=GPR98 PE=1 SV=2 | 4736 | YVIQLVsVEGG | 0.212 | -0.065 | -0.638 | -0.164 |
| sp\|Q8WXG9\|GPR98_HUMAN G-protein coupled receptor 98 OS=Homo sapiens GN=GPR98 PE=1 SV=2 | 4748 | ELDLEKsITWF | 0.207 | -0.029 | -0.671 | -0.164 |
| sp\|Q8WXG9\|GPR98_HUMAN G-protein coupled receptor 98 OS=Homo sapiens GN=GPR98 PE=1 SV=2 | 4750 | DLEKSItWFSV | 0.643 | 0.361 | -0.064 | 0.313 |
| sp\|Q8WXG9\|GPR98_HUMAN G-protein coupled receptor 98 OS=Homo sapiens GN=GPR98 PE=1 SV=2 | 4753 | KSITWFsVYAN | 0.237 | 0.073 | -0.641 | -0.11 |
| sp\|Q8WXG9\|GPR98_HUMAN G-protein coupled receptor 98 OS=Homo sapiens GN=GPR98 PE=1 SV=2 | 4768 | GVFALYsDRQS | 0.199 | -0.047 | -0.851 | -0.233 |
| sp\|Q8WXG9\|GPR98_HUMAN G-protein coupled receptor 98 OS=Homo sapiens GN=GPR98 PE=1 SV=2 | 4772 | LYSDRQsILIG | 0.288 | 0.076 | -0.618 | -0.085 |
| sp\|Q8WXG9\|GPR98_HUMAN G-protein coupled receptor 98 OS=Homo sapiens GN=GPR98 PE=1 SV=2 | 4782 | GQNLIRsIQIN | 0.146 | -0.058 | -0.979 | -0.297 |

| | | | | | | |
|---|---|---|---|---|---|---|
| sp\|Q8WXG9\|GPR98_HUMAN G-protein coupled receptor 98 OS=Homo sapiens GN=GPR98 PE=1 SV=2 | 4788 | SIQINItRLAG | 0.181 | 0.009 | -0.883 | -0.231 |
| sp\|Q8WXG9\|GPR98_HUMAN G-protein coupled receptor 98 OS=Homo sapiens GN=GPR98 PE=1 SV=2 | 4793 | ITRLAGtFGDV | 0.771 | 0.369 | 0.018 | 0.386 |
| sp\|Q8WXG9\|GPR98_HUMAN G-protein coupled receptor 98 OS=Homo sapiens GN=GPR98 PE=1 SV=2 | 4804 | AVGLRIsSDHK | 0.172 | -0.052 | -0.83 | -0.237 |
| sp\|Q8WXG9\|GPR98_HUMAN G-protein coupled receptor 98 OS=Homo sapiens GN=GPR98 PE=1 SV=2 | 4805 | VGLRISsDHKE | 0.468 | 0.707 | 0.066 | 0.414 |
| sp\|Q8WXG9\|GPR98_HUMAN G-protein coupled receptor 98 OS=Homo sapiens GN=GPR98 PE=1 SV=2 | 4814 | KEQPIVtENAE | 0.382 | 0.058 | -0.28 | 0.053 |
| sp\|Q8WXG9\|GPR98_HUMAN G-protein coupled receptor 98 OS=Homo sapiens GN=GPR98 PE=1 SV=2 | 4828 | VVKDGAtYKVD | 0.143 | -0.071 | -0.891 | -0.273 |
| sp\|Q8WXG9\|GPR98_HUMAN G-protein coupled receptor 98 OS=Homo sapiens GN=GPR98 PE=1 SV=2 | 4843 | KNQVFLsLGSN | 0.112 | -0.074 | -1.028 | -0.33 |
| sp\|Q8WXG9\|GPR98_HUMAN G-protein coupled receptor 98 OS=Homo sapiens GN=GPR98 PE=1 SV=2 | 4846 | VFLSLGsNFTL | 0.081 | -0.253 | -1.274 | -0.482 |
| sp\|Q8WXG9\|GPR98_HUMAN G-protein coupled receptor 98 OS=Homo sapiens GN=GPR98 PE=1 SV=2 | 4849 | SLGSNFtLQLV | 0.324 | 0.092 | -0.547 | -0.044 |
| sp\|Q8WXG9\|GPR98_HUMAN G-protein coupled receptor 98 OS=Homo sapiens GN=GPR98 PE=1 SV=2 | 4854 | FTLQLVtVMLV | 0.377 | 0.028 | -0.707 | -0.101 |
| sp\|Q8WXG9\|GPR98_HUMAN G-protein coupled receptor 98 OS=Homo sapiens GN=GPR98 PE=1 SV=2 | 4867 | RFYGMPtILQE | 0.106 | -0.232 | -0.91 | -0.345 |
| sp\|Q8WXG9\|GPR98_HUMAN G-protein coupled receptor 98 OS=Homo sapiens GN=GPR98 PE=1 SV=2 | 4874 | ILQEAKsAVLP | 0.387 | 0.221 | -0.327 | 0.094 |
| sp\|Q8WXG9\|GPR98_HUMAN G-protein coupled receptor 98 OS=Homo sapiens GN=GPR98 PE=1 SV=2 | 4880 | SAVLPVsEKAA | 0.178 | -0.119 | -0.78 | -0.24 |
| sp\|Q8WXG9\|GPR98_HUMAN G-protein coupled receptor 98 OS=Homo sapiens GN=GPR98 PE=1 SV=2 | 4886 | SEKAANsQVGF | 0.199 | -0.113 | -1.084 | -0.333 |
| sp\|Q8WXG9\|GPR98_HUMAN G-protein coupled receptor 98 OS=Homo sapiens GN=GPR98 PE=1 SV=2 | 4892 | SQVGFEsTAFQ | 0.033 | -0.391 | -1.94 | -0.766 |
| sp\|Q8WXG9\|GPR98_HUMAN G-protein coupled receptor 98 OS=Homo sapiens GN=GPR98 PE=1 SV=2 | 4893 | QVGFEStAFQL | 0.094 | -0.09 | -1.171 | -0.389 |
| sp\|Q8WXG9\|GPR98_HUMAN G-protein coupled receptor 98 OS=Homo sapiens GN=GPR98 PE=1 SV=2 | 4901 | FQLMNItAGTS | 0.31 | 0.015 | -0.448 | -0.041 |
| sp\|Q8WXG9\|GPR98_HUMAN G-protein coupled receptor 98 OS=Homo sapiens GN=GPR98 PE=1 SV=2 | 4904 | MNITAGtSHVM | 0.184 | -0.046 | -0.978 | -0.28 |
| sp\|Q8WXG9\|GPR98_HUMAN G-protein coupled receptor 98 OS=Homo sapiens GN=GPR98 PE=1 SV=2 | 4905 | NITAGTsHVMI | 0.217 | -0.04 | -0.601 | -0.141 |
| sp\|Q8WXG9\|GPR98_HUMAN G-protein coupled receptor 98 OS=Homo sapiens GN=GPR98 PE=1 SV=2 | 4910 | TSHVMIsRRGT | 0.061 | -0.5 | -1.404 | -0.614 |
| sp\|Q8WXG9\|GPR98_HUMAN G-protein coupled receptor 98 OS=Homo sapiens GN=GPR98 PE=1 SV=2 | 4914 | MISRRGtYGAL | 0.829 | 1.164 | 0.899 | 0.964 |
| sp\|Q8WXG9\|GPR98_HUMAN G-protein coupled receptor 98 OS=Homo sapiens GN=GPR98 PE=1 SV=2 | 4919 | GTYGALsVAWT | 0.128 | -0.069 | -0.875 | -0.272 |
| sp\|Q8WXG9\|GPR98_HUMAN G-protein coupled receptor 98 OS=Homo sapiens GN=GPR98 PE=1 SV=2 | 4923 | ALSVAWtTGYA | 0.347 | -0.023 | -0.611 | -0.096 |
| sp\|Q8WXG9\|GPR98_HUMAN G-protein coupled receptor 98 OS=Homo sapiens GN=GPR98 PE=1 SV=2 | 4924 | LSVAWTtGYAP | 0.122 | -0.136 | -0.783 | -0.266 |
| sp\|Q8WXG9\|GPR98_HUMAN G-protein coupled receptor 98 OS=Homo sapiens GN=GPR98 PE=1 SV=2 | 4942 | IVVGNMtPTLG | 0.163 | -0.193 | -0.801 | -0.277 |
| sp\|Q8WXG9\|GPR98_HUMAN G-protein coupled receptor 98 OS=Homo sapiens GN=GPR98 PE=1 SV=2 | 4944 | VGNMTPtLGSL | 0.17 | -0.043 | -0.644 | -0.172 |
| sp\|Q8WXG9\|GPR98_HUMAN G-protein coupled receptor 98 OS=Homo sapiens GN=GPR98 PE=1 SV=2 | 4947 | MTPTLGsLSFS | 0.074 | -0.253 | -1.564 | -0.581 |
| sp\|Q8WXG9\|GPR98_HUMAN G-protein coupled receptor 98 OS=Homo sapiens GN=GPR98 PE=1 SV=2 | 4949 | PTLGSLsFSHG | 0.224 | 0.092 | -0.391 | -0.025 |
| sp\|Q8WXG9\|GPR98_HUMAN G-protein coupled receptor 98 OS=Homo sapiens GN=GPR98 PE=1 SV=2 | 4951 | LGSLSFsHGEQ | 0.478 | 0.202 | -0.081 | 0.2 |
| sp\|Q8WXG9\|GPR98_HUMAN G-protein coupled receptor 98 OS=Homo sapiens GN=GPR98 PE=1 SV=2 | 4963 | KGVFLWtFPSP | 0.318 | 0.312 | -0.423 | 0.069 |
| sp\|Q8WXG9\|GPR98_HUMAN G-protein coupled receptor 98 OS=Homo sapiens GN=GPR98 PE=1 SV=2 | 4966 | FLWTFPsPGWP | 0.13 | -0.138 | -1.057 | -0.355 |
| sp\|Q8WXG9\|GPR98_HUMAN G-protein coupled receptor 98 OS=Homo sapiens GN=GPR98 PE=1 SV=2 | 4978 | AFVLHLsGVQS | 0.1 | -0.147 | -1.143 | -0.397 |
| sp\|Q8WXG9\|GPR98_HUMAN G-protein coupled receptor 98 OS=Homo sapiens GN=GPR98 PE=1 SV=2 | 4982 | HLSGVQsSAPG | 0.154 | -0.054 | -0.775 | -0.225 |
| sp\|Q8WXG9\|GPR98_HUMAN G-protein coupled receptor 98 OS=Homo sapiens GN=GPR98 PE=1 SV=2 | 4983 | LSGVQSsAPGG | 0.181 | 0.258 | -0.76 | -0.107 |
| sp\|Q8WXG9\|GPR98_HUMAN G-protein coupled receptor 98 OS=Homo sapiens GN=GPR98 PE=1 SV=2 | 4992 | GGAQLRsGFIV | 0.187 | -0.115 | -1.002 | -0.31 |
| sp\|Q8WXG9\|GPR98_HUMAN G-protein coupled receptor 98 OS=Homo sapiens GN=GPR98 PE=1 SV=2 | 5008 | MGVFQFsTSSR | 0.09 | -0.037 | -1.111 | -0.353 |
| sp\|Q8WXG9\|GPR98_HUMAN G-protein coupled receptor 98 OS=Homo sapiens GN=GPR98 PE=1 SV=2 | 5009 | GVFQFStSSRN | 0.101 | -0.217 | -1.455 | -0.524 |
| sp\|Q8WXG9\|GPR98_HUMAN G-protein coupled receptor 98 OS=Homo sapiens GN=GPR98 PE=1 SV=2 | 5010 | VFQFSTsSRNI | 0.508 | 0.26 | -0.007 | 0.254 |
| sp\|Q8WXG9\|GPR98_HUMAN G-protein coupled receptor 98 OS=Homo sapiens GN=GPR98 PE=1 SV=2 | 5011 | FQFSTSsRNII | 0.139 | -0.056 | -0.796 | -0.238 |
| sp\|Q8WXG9\|GPR98_HUMAN G-protein coupled receptor 98 OS=Homo sapiens GN=GPR98 PE=1 SV=2 | 5017 | SRNIIVsEDTQ | 0.279 | 0.179 | -0.62 | -0.054 |
| sp\|Q8WXG9\|GPR98_HUMAN G-protein coupled receptor 98 OS=Homo sapiens GN=GPR98 PE=1 SV=2 | 5020 | IIVSEDtQMIR | 0.197 | -0.194 | -1.298 | -0.432 |
| sp\|Q8WXG9\|GPR98_HUMAN G-protein coupled receptor 98 OS=Homo sapiens GN=GPR98 PE=1 SV=2 | 5035 | RLFGFHsDLIK | 0.438 | 0.182 | -0.621 | 0 |
| sp\|Q8WXG9\|GPR98_HUMAN G-protein coupled receptor 98 OS=Homo sapiens GN=GPR98 PE=1 SV=2 | 5041 | SDLIKVsYQTT | 0.057 | -0.336 | -1.352 | -0.544 |
| sp\|Q8WXG9\|GPR98_HUMAN G-protein coupled receptor 98 OS=Homo sapiens GN=GPR98 PE=1 SV=2 | 5044 | IKVSYQtTAGS | 0.218 | -0.142 | -0.727 | -0.217 |
| sp\|Q8WXG9\|GPR98_HUMAN G-protein coupled receptor 98 OS=Homo sapiens GN=GPR98 PE=1 SV=2 | 5045 | KVSYQTtAGSA | 0.101 | -0.057 | -1.023 | -0.326 |
| sp\|Q8WXG9\|GPR98_HUMAN G-protein coupled receptor 98 OS=Homo sapiens GN=GPR98 PE=1 SV=2 | 5048 | YQTTAGsAKPL | 0.131 | -0.063 | -0.848 | -0.26 |
| sp\|Q8WXG9\|GPR98_HUMAN G-protein coupled receptor 98 OS=Homo sapiens GN=GPR98 PE=1 SV=2 | 5070 | FFQKFQtEVDF | 0.533 | 0.297 | -0.074 | 0.252 |
| sp\|Q8WXG9\|GPR98_HUMAN G-protein coupled receptor 98 OS=Homo sapiens GN=GPR98 PE=1 SV=2 | 5077 | EVDFEItIIND | 0.115 | -0.075 | -1.005 | -0.322 |
| sp\|Q8WXG9\|GPR98_HUMAN G-protein coupled receptor 98 OS=Homo sapiens GN=GPR98 PE=1 SV=2 | 5084 | IINDQLsEIEE | 0.141 | 0.028 | -1.004 | -0.278 |

| | | | | | | |
|---|---|---|---|---|---|---|
| sp\|Q8WXG9\|GPR98_HUMAN G-protein coupled receptor 98 OS=Homo sapiens GN=GPR98 PE=1 SV=2 | 5095 | FFYINLtSVEI | 0.199 | 0.073 | -0.557 | -0.095 |
| sp\|Q8WXG9\|GPR98_HUMAN G-protein coupled receptor 98 OS=Homo sapiens GN=GPR98 PE=1 SV=2 | 5096 | FYINLTsVEIR | 0.201 | 0.074 | -0.679 | -0.135 |
| sp\|Q8WXG9\|GPR98_HUMAN G-protein coupled receptor 98 OS=Homo sapiens GN=GPR98 PE=1 SV=2 | 5110 | KFDVNWsPRLN | 0.081 | -0.457 | -1.318 | -0.565 |
| sp\|Q8WXG9\|GPR98_HUMAN G-protein coupled receptor 98 OS=Homo sapiens GN=GPR98 PE=1 SV=2 | 5118 | RLNLDFsVAVI | 0.328 | 0.138 | -0.52 | -0.018 |
| sp\|Q8WXG9\|GPR98_HUMAN G-protein coupled receptor 98 OS=Homo sapiens GN=GPR98 PE=1 SV=2 | 5123 | FSVAVItILDN | 0.207 | -0.053 | -0.859 | -0.235 |
| sp\|Q8WXG9\|GPR98_HUMAN G-protein coupled receptor 98 OS=Homo sapiens GN=GPR98 PE=1 SV=2 | 5136 | LAGMDIsFPET | 0.5 | 0.342 | 0.014 | 0.285 |
| sp\|Q8WXG9\|GPR98_HUMAN G-protein coupled receptor 98 OS=Homo sapiens GN=GPR98 PE=1 SV=2 | 5140 | DISFPEtTVAV | 0.211 | -0.054 | -1.088 | -0.31 |
| sp\|Q8WXG9\|GPR98_HUMAN G-protein coupled receptor 98 OS=Homo sapiens GN=GPR98 PE=1 SV=2 | 5141 | ISFPETtVAVA | 0.144 | -0.217 | -0.961 | -0.345 |
| sp\|Q8WXG9\|GPR98_HUMAN G-protein coupled receptor 98 OS=Homo sapiens GN=GPR98 PE=1 SV=2 | 5148 | VAVAVDtTLIP | 0.082 | -0.364 | -1.673 | -0.652 |
| sp\|Q8WXG9\|GPR98_HUMAN G-protein coupled receptor 98 OS=Homo sapiens GN=GPR98 PE=1 SV=2 | 5149 | AVAVDTtLIPV | 0.125 | -0.095 | -0.858 | -0.276 |
| sp\|Q8WXG9\|GPR98_HUMAN G-protein coupled receptor 98 OS=Homo sapiens GN=GPR98 PE=1 SV=2 | 5155 | TLIPVEtESTT | 0.124 | -0.12 | -1.135 | -0.377 |
| sp\|Q8WXG9\|GPR98_HUMAN G-protein coupled receptor 98 OS=Homo sapiens GN=GPR98 PE=1 SV=2 | 5157 | IPVETEsTTYL | 0.083 | -0.156 | -1.453 | -0.509 |
| sp\|Q8WXG9\|GPR98_HUMAN G-protein coupled receptor 98 OS=Homo sapiens GN=GPR98 PE=1 SV=2 | 5158 | PVETEStTYLS | 0.081 | -0.178 | -1.361 | -0.486 |
| sp\|Q8WXG9\|GPR98_HUMAN G-protein coupled receptor 98 OS=Homo sapiens GN=GPR98 PE=1 SV=2 | 5159 | VETESTtYLST | 0.067 | -0.069 | -1.157 | -0.386 |
| sp\|Q8WXG9\|GPR98_HUMAN G-protein coupled receptor 98 OS=Homo sapiens GN=GPR98 PE=1 SV=2 | 5162 | ESTTYLsTSKT | 0.043 | -0.32 | -1.485 | -0.587 |
| sp\|Q8WXG9\|GPR98_HUMAN G-protein coupled receptor 98 OS=Homo sapiens GN=GPR98 PE=1 SV=2 | 5163 | STTYLStSKTT | 0.042 | -0.334 | -1.532 | -0.608 |
| sp\|Q8WXG9\|GPR98_HUMAN G-protein coupled receptor 98 OS=Homo sapiens GN=GPR98 PE=1 SV=2 | 5164 | TTYLSTsKTTT | 0.279 | 0.028 | -0.513 | -0.069 |
| sp\|Q8WXG9\|GPR98_HUMAN G-protein coupled receptor 98 OS=Homo sapiens GN=GPR98 PE=1 SV=2 | 5166 | YLSTSKtTTIL | 0.666 | 0.308 | 0.203 | 0.392 |
| sp\|Q8WXG9\|GPR98_HUMAN G-protein coupled receptor 98 OS=Homo sapiens GN=GPR98 PE=1 SV=2 | 5167 | LSTSKTtTILQ | 0.07 | -0.263 | -1.147 | -0.447 |
| sp\|Q8WXG9\|GPR98_HUMAN G-protein coupled receptor 98 OS=Homo sapiens GN=GPR98 PE=1 SV=2 | 5168 | STSKTTtILQP | 0.088 | -0.224 | -1.276 | -0.471 |
| sp\|Q8WXG9\|GPR98_HUMAN G-protein coupled receptor 98 OS=Homo sapiens GN=GPR98 PE=1 SV=2 | 5173 | TTILQPtNVVA | 0.077 | -0.183 | -1.555 | -0.554 |
| sp\|Q8WXG9\|GPR98_HUMAN G-protein coupled receptor 98 OS=Homo sapiens GN=GPR98 PE=1 SV=2 | 5180 | NVVAIVtEATG | 0.234 | -0.053 | -0.567 | -0.129 |
| sp\|Q8WXG9\|GPR98_HUMAN G-protein coupled receptor 98 OS=Homo sapiens GN=GPR98 PE=1 SV=2 | 5183 | AIVTEAtGVSA | 0.109 | -0.152 | -1.318 | -0.454 |
| sp\|Q8WXG9\|GPR98_HUMAN G-protein coupled receptor 98 OS=Homo sapiens GN=GPR98 PE=1 SV=2 | 5186 | TEATGVsAIPE | 0.112 | -0.233 | -1.169 | -0.43 |
| sp\|Q8WXG9\|GPR98_HUMAN G-protein coupled receptor 98 OS=Homo sapiens GN=GPR98 PE=1 SV=2 | 5194 | IPEKLVtLHGT | 0.583 | -0.053 | -0.096 | 0.145 |
| sp\|Q8WXG9\|GPR98_HUMAN G-protein coupled receptor 98 OS=Homo sapiens GN=GPR98 PE=1 SV=2 | 5198 | LVTLHGtPAVS | 0.039 | -0.409 | -1.651 | -0.674 |
| sp\|Q8WXG9\|GPR98_HUMAN G-protein coupled receptor 98 OS=Homo sapiens GN=GPR98 PE=1 SV=2 | 5202 | HGTPAVsEKPD | 0.385 | 0.069 | -0.036 | 0.139 |
| sp\|Q8WXG9\|GPR98_HUMAN G-protein coupled receptor 98 OS=Homo sapiens GN=GPR98 PE=1 SV=2 | 5209 | EKPDVAtVTAN | 0.402 | 0.1 | -0.566 | -0.021 |
| sp\|Q8WXG9\|GPR98_HUMAN G-protein coupled receptor 98 OS=Homo sapiens GN=GPR98 PE=1 SV=2 | 5211 | PDVATVtANVS | 0.168 | -0.031 | -0.904 | -0.256 |
| sp\|Q8WXG9\|GPR98_HUMAN G-protein coupled receptor 98 OS=Homo sapiens GN=GPR98 PE=1 SV=2 | 5215 | TVTANVsIHGT | 0.213 | -0.197 | -0.538 | -0.174 |
| sp\|Q8WXG9\|GPR98_HUMAN G-protein coupled receptor 98 OS=Homo sapiens GN=GPR98 PE=1 SV=2 | 5219 | NVSIHGtFSLG | 0.134 | -0.041 | -0.994 | -0.3 |
| sp\|Q8WXG9\|GPR98_HUMAN G-protein coupled receptor 98 OS=Homo sapiens GN=GPR98 PE=1 SV=2 | 5221 | SIHGTFsLGPS | 0.135 | 0.075 | -0.737 | -0.176 |
| sp\|Q8WXG9\|GPR98_HUMAN G-protein coupled receptor 98 OS=Homo sapiens GN=GPR98 PE=1 SV=2 | 5225 | TFSLGPsIVYI | 0.091 | -0.227 | -1.055 | -0.397 |
| sp\|Q8WXG9\|GPR98_HUMAN G-protein coupled receptor 98 OS=Homo sapiens GN=GPR98 PE=1 SV=2 | 5237 | EEMKNGtFNTA | 0.298 | -0.005 | -0.411 | -0.039 |
| sp\|Q8WXG9\|GPR98_HUMAN G-protein coupled receptor 98 OS=Homo sapiens GN=GPR98 PE=1 SV=2 | 5240 | KNGTFNtAEVL | 0.128 | -0.072 | -1.321 | -0.422 |
| sp\|Q8WXG9\|GPR98_HUMAN G-protein coupled receptor 98 OS=Homo sapiens GN=GPR98 PE=1 SV=2 | 5248 | EVLIRRtGGFT | 0.099 | -0.026 | -0.963 | -0.297 |
| sp\|Q8WXG9\|GPR98_HUMAN G-protein coupled receptor 98 OS=Homo sapiens GN=GPR98 PE=1 SV=2 | 5252 | RRTGGFtGNVS | 0.158 | 0.188 | -0.594 | -0.083 |
| sp\|Q8WXG9\|GPR98_HUMAN G-protein coupled receptor 98 OS=Homo sapiens GN=GPR98 PE=1 SV=2 | 5256 | GFTGNVsITVK | 0.418 | -0.032 | -0.245 | 0.047 |
| sp\|Q8WXG9\|GPR98_HUMAN G-protein coupled receptor 98 OS=Homo sapiens GN=GPR98 PE=1 SV=2 | 5258 | TGNVSItVKTF | 0.16 | 0.031 | -0.623 | -0.144 |
| sp\|Q8WXG9\|GPR98_HUMAN G-protein coupled receptor 98 OS=Homo sapiens GN=GPR98 PE=1 SV=2 | 5261 | VSITVKtFGER | 0.484 | 0.08 | -0.198 | 0.122 |
| sp\|Q8WXG9\|GPR98_HUMAN G-protein coupled receptor 98 OS=Homo sapiens GN=GPR98 PE=1 SV=2 | 5283 | RGIYGIsNLTW | 0.088 | -0.111 | -1.194 | -0.406 |
| sp\|Q8WXG9\|GPR98_HUMAN G-protein coupled receptor 98 OS=Homo sapiens GN=GPR98 PE=1 SV=2 | 5286 | YGISNLtWAVE | 0.071 | -0.113 | -1.125 | -0.389 |
| sp\|Q8WXG9\|GPR98_HUMAN G-protein coupled receptor 98 OS=Homo sapiens GN=GPR98 PE=1 SV=2 | 5298 | EDFEEQtLTLI | 0.2 | -0.02 | -0.704 | -0.175 |
| sp\|Q8WXG9\|GPR98_HUMAN G-protein coupled receptor 98 OS=Homo sapiens GN=GPR98 PE=1 SV=2 | 5300 | FEEQTLtLIFL | 0.146 | 0.059 | -0.586 | -0.127 |
| sp\|Q8WXG9\|GPR98_HUMAN G-protein coupled receptor 98 OS=Homo sapiens GN=GPR98 PE=1 SV=2 | 5313 | ERERKVsVQIL | 0.762 | 1.064 | 0.717 | 0.848 |
| sp\|Q8WXG9\|GPR98_HUMAN G-protein coupled receptor 98 OS=Homo sapiens GN=GPR98 PE=1 SV=2 | 5333 | FFYVFLtNPQG | 0.369 | 0.413 | -0.353 | 0.143 |
| sp\|Q8WXG9\|GPR98_HUMAN G-protein coupled receptor 98 OS=Homo sapiens GN=GPR98 PE=1 SV=2 | 5348 | VEEKDDtGFAA | 0.263 | -0.172 | -0.727 | -0.212 |
| sp\|Q8WXG9\|GPR98_HUMAN G-protein coupled receptor 98 OS=Homo sapiens GN=GPR98 PE=1 SV=2 | 5359 | FAMVIItGSDL | 0.125 | -0.007 | -0.768 | -0.217 |
| sp\|Q8WXG9\|GPR98_HUMAN G-protein coupled receptor 98 OS=Homo sapiens GN=GPR98 PE=1 SV=2 | 5361 | MVIITGsDLHN | 0.066 | 0.005 | -1.361 | -0.43 |

| | | | | | | |
|---|---|---|---|---|---|---|
| sp\|Q8WXG9\|GPR98_HUMAN G-protein coupled receptor 98 OS=Homo sapiens GN=GPR98 PE=1 SV=2 | 5371 | NGIIGFsEESQ | 0.058 | -0.069 | -1.216 | -0.409 |
| sp\|Q8WXG9\|GPR98_HUMAN G-protein coupled receptor 98 OS=Homo sapiens GN=GPR98 PE=1 SV=2 | 5374 | IGFSEEsQSGL | 0.094 | -0.315 | -1.459 | -0.56 |
| sp\|Q8WXG9\|GPR98_HUMAN G-protein coupled receptor 98 OS=Homo sapiens GN=GPR98 PE=1 SV=2 | 5376 | FSEESQsGLEL | 0.216 | 0.157 | -0.515 | -0.047 |
| sp\|Q8WXG9\|GPR98_HUMAN G-protein coupled receptor 98 OS=Homo sapiens GN=GPR98 PE=1 SV=2 | 5394 | RLHLIVtRQPN | 0.303 | -0.037 | -0.661 | -0.132 |
| sp\|Q8WXG9\|GPR98_HUMAN G-protein coupled receptor 98 OS=Homo sapiens GN=GPR98 PE=1 SV=2 | 5411 | KVFWRVtLNKT | 0.58 | 0.141 | 0.08 | 0.267 |
| sp\|Q8WXG9\|GPR98_HUMAN G-protein coupled receptor 98 OS=Homo sapiens GN=GPR98 PE=1 SV=2 | 5415 | RVTLNKtVVVL | 0.307 | 0.167 | -0.296 | 0.059 |
| sp\|Q8WXG9\|GPR98_HUMAN G-protein coupled receptor 98 OS=Homo sapiens GN=GPR98 PE=1 SV=2 | 5432 | LVEELQsVSGT | 0.049 | -0.257 | -1.29 | -0.499 |
| sp\|Q8WXG9\|GPR98_HUMAN G-protein coupled receptor 98 OS=Homo sapiens GN=GPR98 PE=1 SV=2 | 5434 | EELQSVsGTTT | 0.389 | 0.014 | -0.257 | 0.049 |
| sp\|Q8WXG9\|GPR98_HUMAN G-protein coupled receptor 98 OS=Homo sapiens GN=GPR98 PE=1 SV=2 | 5436 | LQSVSGtTTCT | 0.121 | -0.019 | -0.872 | -0.257 |
| sp\|Q8WXG9\|GPR98_HUMAN G-protein coupled receptor 98 OS=Homo sapiens GN=GPR98 PE=1 SV=2 | 5437 | QSVSGTtTCTM | 0.049 | -0.403 | -1.475 | -0.61 |
| sp\|Q8WXG9\|GPR98_HUMAN G-protein coupled receptor 98 OS=Homo sapiens GN=GPR98 PE=1 SV=2 | 5438 | SVSGTTtCTMG | 0.094 | -0.088 | -1.245 | -0.413 |
| sp\|Q8WXG9\|GPR98_HUMAN G-protein coupled receptor 98 OS=Homo sapiens GN=GPR98 PE=1 SV=2 | 5440 | SGTTTCtMGQT | 0.164 | -0.001 | -0.961 | -0.266 |
| sp\|Q8WXG9\|GPR98_HUMAN G-protein coupled receptor 98 OS=Homo sapiens GN=GPR98 PE=1 SV=2 | 5444 | TCTMGQtKCFI | 0.123 | -0.227 | -0.984 | -0.363 |
| sp\|Q8WXG9\|GPR98_HUMAN G-protein coupled receptor 98 OS=Homo sapiens GN=GPR98 PE=1 SV=2 | 5449 | QTKCFIsIELK | 0.221 | -0.157 | -0.886 | -0.274 |
| sp\|Q8WXG9\|GPR98_HUMAN G-protein coupled receptor 98 OS=Homo sapiens GN=GPR98 PE=1 SV=2 | 5472 | VELYEAtAGAA | 0.36 | 0.067 | -0.522 | -0.032 |
| sp\|Q8WXG9\|GPR98_HUMAN G-protein coupled receptor 98 OS=Homo sapiens GN=GPR98 PE=1 SV=2 | 5480 | GAAINNsARFA | 0.254 | -0.097 | -0.808 | -0.217 |
| sp\|Q8WXG9\|GPR98_HUMAN G-protein coupled receptor 98 OS=Homo sapiens GN=GPR98 PE=1 SV=2 | 5491 | QIKILEsDESQ | 0.084 | -0.094 | -1.42 | -0.477 |
| sp\|Q8WXG9\|GPR98_HUMAN G-protein coupled receptor 98 OS=Homo sapiens GN=GPR98 PE=1 SV=2 | 5494 | ILESDEsQSLV | 0.146 | -0.208 | -1.419 | -0.494 |
| sp\|Q8WXG9\|GPR98_HUMAN G-protein coupled receptor 98 OS=Homo sapiens GN=GPR98 PE=1 SV=2 | 5496 | ESDESQsLVYF | 0.184 | 0.103 | -0.533 | -0.082 |
| sp\|Q8WXG9\|GPR98_HUMAN G-protein coupled receptor 98 OS=Homo sapiens GN=GPR98 PE=1 SV=2 | 5501 | QSLVYFsVGSR | 0.129 | -0.061 | -0.759 | -0.23 |
| sp\|Q8WXG9\|GPR98_HUMAN G-protein coupled receptor 98 OS=Homo sapiens GN=GPR98 PE=1 SV=2 | 5504 | VYFSVGsRLAV | 0.135 | -0.2 | -1.503 | -0.523 |
| sp\|Q8WXG9\|GPR98_HUMAN G-protein coupled receptor 98 OS=Homo sapiens GN=GPR98 PE=1 SV=2 | 5514 | VAHKKAtLISL | 0.171 | -0.031 | -0.708 | -0.189 |
| sp\|Q8WXG9\|GPR98_HUMAN G-protein coupled receptor 98 OS=Homo sapiens GN=GPR98 PE=1 SV=2 | 5517 | KKATLIsLQVA | 0.468 | 0.053 | -0.203 | 0.106 |
| sp\|Q8WXG9\|GPR98_HUMAN G-protein coupled receptor 98 OS=Homo sapiens GN=GPR98 PE=1 SV=2 | 5524 | LQVARDsGTGL | 0.22 | -0.097 | -0.682 | -0.186 |
| sp\|Q8WXG9\|GPR98_HUMAN G-protein coupled receptor 98 OS=Homo sapiens GN=GPR98 PE=1 SV=2 | 5526 | VARDSGtGLMM | 0.249 | 0.116 | -0.743 | -0.126 |
| sp\|Q8WXG9\|GPR98_HUMAN G-protein coupled receptor 98 OS=Homo sapiens GN=GPR98 PE=1 SV=2 | 5531 | GTGLMMsVNFS | 0.205 | -0.018 | -0.848 | -0.22 |
| sp\|Q8WXG9\|GPR98_HUMAN G-protein coupled receptor 98 OS=Homo sapiens GN=GPR98 PE=1 SV=2 | 5535 | MMSVNFsTQEL | 0.23 | 0.196 | -0.538 | -0.037 |
| sp\|Q8WXG9\|GPR98_HUMAN G-protein coupled receptor 98 OS=Homo sapiens GN=GPR98 PE=1 SV=2 | 5536 | MSVNFStQELR | 0.088 | -0.153 | -1.284 | -0.45 |
| sp\|Q8WXG9\|GPR98_HUMAN G-protein coupled receptor 98 OS=Homo sapiens GN=GPR98 PE=1 SV=2 | 5541 | STQELRsAETI | 0.08 | -0.089 | -1.582 | -0.53 |
| sp\|Q8WXG9\|GPR98_HUMAN G-protein coupled receptor 98 OS=Homo sapiens GN=GPR98 PE=1 SV=2 | 5544 | ELRSAEtIGRT | 0.441 | 0.145 | -0.392 | 0.065 |
| sp\|Q8WXG9\|GPR98_HUMAN G-protein coupled receptor 98 OS=Homo sapiens GN=GPR98 PE=1 SV=2 | 5548 | AETIGRtIISP | 0.021 | -0.32 | -1.825 | -0.708 |
| sp\|Q8WXG9\|GPR98_HUMAN G-protein coupled receptor 98 OS=Homo sapiens GN=GPR98 PE=1 SV=2 | 5551 | IGRTIIsPAIS | 0.206 | -0.214 | -0.741 | -0.25 |
| sp\|Q8WXG9\|GPR98_HUMAN G-protein coupled receptor 98 OS=Homo sapiens GN=GPR98 PE=1 SV=2 | 5555 | IISPAIsGKDF | 0.311 | -0.013 | -0.33 | -0.011 |
| sp\|Q8WXG9\|GPR98_HUMAN G-protein coupled receptor 98 OS=Homo sapiens GN=GPR98 PE=1 SV=2 | 5562 | GKDFVItEGTL | 0.621 | 0.214 | -0.109 | 0.242 |
| sp\|Q8WXG9\|GPR98_HUMAN G-protein coupled receptor 98 OS=Homo sapiens GN=GPR98 PE=1 SV=2 | 5565 | FVITEGtLVFE | 0.121 | -0.088 | -1.215 | -0.394 |
| sp\|Q8WXG9\|GPR98_HUMAN G-protein coupled receptor 98 OS=Homo sapiens GN=GPR98 PE=1 SV=2 | 5574 | FEPGQRsTVLD | 0.067 | -0.051 | -1.452 | -0.479 |
| sp\|Q8WXG9\|GPR98_HUMAN G-protein coupled receptor 98 OS=Homo sapiens GN=GPR98 PE=1 SV=2 | 5575 | EPGQRStVLDV | 0.164 | 0.024 | -0.975 | -0.262 |
| sp\|Q8WXG9\|GPR98_HUMAN G-protein coupled receptor 98 OS=Homo sapiens GN=GPR98 PE=1 SV=2 | 5582 | VLDVILtPETG | 0.046 | -0.404 | -1.412 | -0.59 |
| sp\|Q8WXG9\|GPR98_HUMAN G-protein coupled receptor 98 OS=Homo sapiens GN=GPR98 PE=1 SV=2 | 5585 | VILTPEtGSLN | 0.081 | -0.234 | -1.314 | -0.489 |
| sp\|Q8WXG9\|GPR98_HUMAN G-protein coupled receptor 98 OS=Homo sapiens GN=GPR98 PE=1 SV=2 | 5587 | LTPETGsLNSF | 0.046 | -0.147 | -1.252 | -0.451 |
| sp\|Q8WXG9\|GPR98_HUMAN G-protein coupled receptor 98 OS=Homo sapiens GN=GPR98 PE=1 SV=2 | 5590 | ETGSLNsFPKR | 0.443 | 0.404 | -0.321 | 0.175 |
| sp\|Q8WXG9\|GPR98_HUMAN G-protein coupled receptor 98 OS=Homo sapiens GN=GPR98 PE=1 SV=2 | 5614 | IDKVYGtANIT | 0.138 | -0.177 | -0.97 | -0.336 |
| sp\|Q8WXG9\|GPR98_HUMAN G-protein coupled receptor 98 OS=Homo sapiens GN=GPR98 PE=1 SV=2 | 5618 | YGTANItLVSD | 0.304 | 0.001 | -0.161 | 0.048 |
| sp\|Q8WXG9\|GPR98_HUMAN G-protein coupled receptor 98 OS=Homo sapiens GN=GPR98 PE=1 SV=2 | 5621 | ANITLVsDADS | 0.191 | -0.182 | -1.181 | -0.391 |
| sp\|Q8WXG9\|GPR98_HUMAN G-protein coupled receptor 98 OS=Homo sapiens GN=GPR98 PE=1 SV=2 | 5625 | LVSDADsQAIW | 0.13 | -0.17 | -1.356 | -0.465 |
| sp\|Q8WXG9\|GPR98_HUMAN G-protein coupled receptor 98 OS=Homo sapiens GN=GPR98 PE=1 SV=2 | 5650 | LNRVLHtISMK | 0.102 | -0.044 | -1.053 | -0.332 |
| sp\|Q8WXG9\|GPR98_HUMAN G-protein coupled receptor 98 OS=Homo sapiens GN=GPR98 PE=1 SV=2 | 5652 | RVLHTIsMKVA | 0.155 | 0.1 | -0.76 | -0.168 |
| sp\|Q8WXG9\|GPR98_HUMAN G-protein coupled receptor 98 OS=Homo sapiens GN=GPR98 PE=1 SV=2 | 5657 | ISMKVAtENTD | 0.588 | 0.132 | -0.007 | 0.238 |
| sp\|Q8WXG9\|GPR98_HUMAN G-protein coupled receptor 98 OS=Homo sapiens GN=GPR98 PE=1 SV=2 | 5660 | KVATENtDEQL | 0.216 | -0.035 | -0.966 | -0.262 |

| | | | | | | |
|---|---|---|---|---|---|---|
| sp\|Q8WXG9\|GPR98_HUMAN G-protein coupled receptor 98 OS=Homo sapiens GN=GPR98 PE=1 SV=2 | 5665 | NTDEQLsAMMH | 0.039 | -0.31 | -2.062 | -0.778 |
| sp\|Q8WXG9\|GPR98_HUMAN G-protein coupled receptor 98 OS=Homo sapiens GN=GPR98 PE=1 SV=2 | 5675 | HLIEKItTEGK | 0.123 | -0.056 | -0.89 | -0.274 |
| sp\|Q8WXG9\|GPR98_HUMAN G-protein coupled receptor 98 OS=Homo sapiens GN=GPR98 PE=1 SV=2 | 5676 | LIEKITtEGKI | 0.539 | 0.237 | 0.238 | 0.338 |
| sp\|Q8WXG9\|GPR98_HUMAN G-protein coupled receptor 98 OS=Homo sapiens GN=GPR98 PE=1 SV=2 | 5684 | GKIQAFsVASR | 0.409 | 0.157 | -0.24 | 0.109 |
| sp\|Q8WXG9\|GPR98_HUMAN G-protein coupled receptor 98 OS=Homo sapiens GN=GPR98 PE=1 SV=2 | 5687 | QAFSVAsRTLF | 0.153 | -0.225 | -1.26 | -0.444 |
| sp\|Q8WXG9\|GPR98_HUMAN G-protein coupled receptor 98 OS=Homo sapiens GN=GPR98 PE=1 SV=2 | 5689 | FSVASRtLFYE | 0.429 | 0.189 | -0.303 | 0.105 |
| sp\|Q8WXG9\|GPR98_HUMAN G-protein coupled receptor 98 OS=Homo sapiens GN=GPR98 PE=1 SV=2 | 5697 | FYEILCsLINP | 0.313 | 0.133 | -0.682 | -0.079 |
| sp\|Q8WXG9\|GPR98_HUMAN G-protein coupled receptor 98 OS=Homo sapiens GN=GPR98 PE=1 SV=2 | 5706 | NPKRKDtRGFS | 0.377 | 0.57 | -0.339 | 0.203 |
| sp\|Q8WXG9\|GPR98_HUMAN G-protein coupled receptor 98 OS=Homo sapiens GN=GPR98 PE=1 SV=2 | 5710 | KDTRGFsHFAE | 0.486 | 0.755 | 0.27 | 0.504 |
| sp\|Q8WXG9\|GPR98_HUMAN G-protein coupled receptor 98 OS=Homo sapiens GN=GPR98 PE=1 SV=2 | 5716 | SHFAEVtENFA | 0.183 | -0.148 | -1.178 | -0.381 |
| sp\|Q8WXG9\|GPR98_HUMAN G-protein coupled receptor 98 OS=Homo sapiens GN=GPR98 PE=1 SV=2 | 5722 | TENFAFsLLTN | 0.325 | 0.1 | -0.626 | -0.067 |
| sp\|Q8WXG9\|GPR98_HUMAN G-protein coupled receptor 98 OS=Homo sapiens GN=GPR98 PE=1 SV=2 | 5725 | FAFSLLtNVTC | 0.046 | -0.285 | -1.858 | -0.699 |
| sp\|Q8WXG9\|GPR98_HUMAN G-protein coupled receptor 98 OS=Homo sapiens GN=GPR98 PE=1 SV=2 | 5728 | SLLTNVtCGSP | 0.26 | 0.003 | -0.706 | -0.148 |
| sp\|Q8WXG9\|GPR98_HUMAN G-protein coupled receptor 98 OS=Homo sapiens GN=GPR98 PE=1 SV=2 | 5731 | TNVTCGsPGEK | 0.09 | -0.502 | -1.378 | -0.597 |
| sp\|Q8WXG9\|GPR98_HUMAN G-protein coupled receptor 98 OS=Homo sapiens GN=GPR98 PE=1 SV=2 | 5736 | GSPGEKsKTIL | 0.28 | -0.081 | -0.687 | -0.163 |
| sp\|Q8WXG9\|GPR98_HUMAN G-protein coupled receptor 98 OS=Homo sapiens GN=GPR98 PE=1 SV=2 | 5738 | PGEKSKtILDS | 0.581 | 0.258 | 0.256 | 0.365 |
| sp\|Q8WXG9\|GPR98_HUMAN G-protein coupled receptor 98 OS=Homo sapiens GN=GPR98 PE=1 SV=2 | 5742 | SKTILDsCPYL | 0.474 | 0.459 | -0.404 | 0.176 |
| sp\|Q8WXG9\|GPR98_HUMAN G-protein coupled receptor 98 OS=Homo sapiens GN=GPR98 PE=1 SV=2 | 5747 | DSCPYLsILAL | 0.11 | -0.148 | -1.071 | -0.37 |
| sp\|Q8WXG9\|GPR98_HUMAN G-protein coupled receptor 98 OS=Homo sapiens GN=GPR98 PE=1 SV=2 | 5790 | EIMAGKsTCKL | 0.088 | -0.07 | -0.928 | -0.303 |
| sp\|Q8WXG9\|GPR98_HUMAN G-protein coupled receptor 98 OS=Homo sapiens GN=GPR98 PE=1 SV=2 | 5791 | IMAGKStCKLV | 0.077 | 0.009 | -1.165 | -0.36 |
| sp\|Q8WXG9\|GPR98_HUMAN G-protein coupled receptor 98 OS=Homo sapiens GN=GPR98 PE=1 SV=2 | 5798 | CKLVQFtEYSS | 0.134 | 0.092 | -0.764 | -0.179 |
| sp\|Q8WXG9\|GPR98_HUMAN G-protein coupled receptor 98 OS=Homo sapiens GN=GPR98 PE=1 SV=2 | 5801 | VQFTEYsSQQW | 0.116 | -0.094 | -0.92 | -0.299 |
| sp\|Q8WXG9\|GPR98_HUMAN G-protein coupled receptor 98 OS=Homo sapiens GN=GPR98 PE=1 SV=2 | 5802 | QFTEYSsQQWF | 0.075 | -0.147 | -1.093 | -0.388 |
| sp\|Q8WXG9\|GPR98_HUMAN G-protein coupled receptor 98 OS=Homo sapiens GN=GPR98 PE=1 SV=2 | 5808 | SQQWFIsGNNL | 0.192 | -0.046 | -0.795 | -0.216 |
| sp\|Q8WXG9\|GPR98_HUMAN G-protein coupled receptor 98 OS=Homo sapiens GN=GPR98 PE=1 SV=2 | 5814 | SGNNLPtLKNK | 0.099 | -0.183 | -1.126 | -0.403 |
| sp\|Q8WXG9\|GPR98_HUMAN G-protein coupled receptor 98 OS=Homo sapiens GN=GPR98 PE=1 SV=2 | 5821 | LKNKVLsLSVK | 0.374 | 0.039 | -0.439 | -0.009 |
| sp\|Q8WXG9\|GPR98_HUMAN G-protein coupled receptor 98 OS=Homo sapiens GN=GPR98 PE=1 SV=2 | 5823 | NKVLSLsVKGQ | 0.294 | 0.121 | -0.383 | 0.011 |
| sp\|Q8WXG9\|GPR98_HUMAN G-protein coupled receptor 98 OS=Homo sapiens GN=GPR98 PE=1 SV=2 | 5828 | LSVKGQsSQLL | 0.145 | -0.074 | -0.793 | -0.241 |
| sp\|Q8WXG9\|GPR98_HUMAN G-protein coupled receptor 98 OS=Homo sapiens GN=GPR98 PE=1 SV=2 | 5829 | SVKGQSsQLLT | 0.031 | -0.262 | -1.808 | -0.68 |
| sp\|Q8WXG9\|GPR98_HUMAN G-protein coupled receptor 98 OS=Homo sapiens GN=GPR98 PE=1 SV=2 | 5833 | QSSQLLtNDNE | 0.085 | -0.235 | -1.379 | -0.51 |
| sp\|Q8WXG9\|GPR98_HUMAN G-protein coupled receptor 98 OS=Homo sapiens GN=GPR98 PE=1 SV=2 | 5853 | PRIIPQtSLCL | 0.12 | 0.199 | -0.902 | -0.194 |
| sp\|Q8WXG9\|GPR98_HUMAN G-protein coupled receptor 98 OS=Homo sapiens GN=GPR98 PE=1 SV=2 | 5854 | RIIPQTsLCLL | 0.225 | 0.101 | -0.636 | -0.103 |
| sp\|Q8WXG9\|GPR98_HUMAN G-protein coupled receptor 98 OS=Homo sapiens GN=GPR98 PE=1 SV=2 | 5865 | WNQAAAsWLSD | 0.173 | 0.013 | -0.723 | -0.179 |
| sp\|Q8WXG9\|GPR98_HUMAN G-protein coupled receptor 98 OS=Homo sapiens GN=GPR98 PE=1 SV=2 | 5868 | AAASWLsDSQF | 0.079 | -0.26 | -1.285 | -0.489 |
| sp\|Q8WXG9\|GPR98_HUMAN G-protein coupled receptor 98 OS=Homo sapiens GN=GPR98 PE=1 SV=2 | 5870 | ASWLSDsQFCK | 0.218 | 0.072 | -0.984 | -0.231 |
| sp\|Q8WXG9\|GPR98_HUMAN G-protein coupled receptor 98 OS=Homo sapiens GN=GPR98 PE=1 SV=2 | 5879 | CKVVEEtADYV | 0.271 | 0.005 | -1.157 | -0.294 |
| sp\|Q8WXG9\|GPR98_HUMAN G-protein coupled receptor 98 OS=Homo sapiens GN=GPR98 PE=1 SV=2 | 5888 | YVECACsHMSV | 0.312 | 0.017 | -0.934 | -0.202 |
| sp\|Q8WXG9\|GPR98_HUMAN G-protein coupled receptor 98 OS=Homo sapiens GN=GPR98 PE=1 SV=2 | 5891 | CACSHMsVYAV | 0.433 | 0.188 | -0.472 | 0.05 |
| sp\|Q8WXG9\|GPR98_HUMAN G-protein coupled receptor 98 OS=Homo sapiens GN=GPR98 PE=1 SV=2 | 5899 | YAVYARtDNLS | 0.209 | 0.072 | -0.77 | -0.163 |
| sp\|Q8WXG9\|GPR98_HUMAN G-protein coupled receptor 98 OS=Homo sapiens GN=GPR98 PE=1 SV=2 | 5903 | ARTDNLsSYNE | 0.15 | 0.093 | -1.079 | -0.279 |
| sp\|Q8WXG9\|GPR98_HUMAN G-protein coupled receptor 98 OS=Homo sapiens GN=GPR98 PE=1 SV=2 | 5904 | RTDNLSsYNEA | 0.103 | -0.198 | -1.119 | -0.405 |
| sp\|Q8WXG9\|GPR98_HUMAN G-protein coupled receptor 98 OS=Homo sapiens GN=GPR98 PE=1 SV=2 | 5911 | YNEAFFtSGFI | 0.188 | -0.147 | -0.694 | -0.218 |
| sp\|Q8WXG9\|GPR98_HUMAN G-protein coupled receptor 98 OS=Homo sapiens GN=GPR98 PE=1 SV=2 | 5912 | NEAFFTsGFIC | 0.169 | -0.172 | -1.071 | -0.358 |
| sp\|Q8WXG9\|GPR98_HUMAN G-protein coupled receptor 98 OS=Homo sapiens GN=GPR98 PE=1 SV=2 | 5918 | SGFICIsGLCL | 0.124 | -0.053 | -1.033 | -0.321 |
| sp\|Q8WXG9\|GPR98_HUMAN G-protein coupled receptor 98 OS=Homo sapiens GN=GPR98 PE=1 SV=2 | 5926 | LCLAVLsHIFC | 0.096 | -0.224 | -1.305 | -0.478 |
| sp\|Q8WXG9\|GPR98_HUMAN G-protein coupled receptor 98 OS=Homo sapiens GN=GPR98 PE=1 SV=2 | 5934 | IFCARYsMFAA | 0.601 | 0.361 | 0.275 | 0.412 |
| sp\|Q8WXG9\|GPR98_HUMAN G-protein coupled receptor 98 OS=Homo sapiens GN=GPR98 PE=1 SV=2 | 5942 | FAAKLLtHMMA | 0.198 | -0.11 | -0.88 | -0.264 |
| sp\|Q8WXG9\|GPR98_HUMAN G-protein coupled receptor 98 OS=Homo sapiens GN=GPR98 PE=1 SV=2 | 5948 | THMMAAsLGTQ | 0.466 | 0.101 | -0.435 | 0.044 |
| sp\|Q8WXG9\|GPR98_HUMAN G-protein coupled receptor 98 OS=Homo sapiens GN=GPR98 PE=1 SV=2 | 5951 | MAASLGtQILF | 0.055 | -0.253 | -1.488 | -0.562 |

| | | | | | | |
|---|---|---|---|---|---|---|
| sp\|Q8WXG9\|GPR98_HUMAN G-protein coupled receptor 98 OS=Homo sapiens GN=GPR98 PE=1 SV=2 | 5958 | QILFLAsAYAS | 0.238 | 0.129 | -0.746 | -0.126 |
| sp\|Q8WXG9\|GPR98_HUMAN G-protein coupled receptor 98 OS=Homo sapiens GN=GPR98 PE=1 SV=2 | 5962 | LASAYAsPQLA | 0.119 | -0.392 | -1.076 | -0.45 |
| sp\|Q8WXG9\|GPR98_HUMAN G-protein coupled receptor 98 OS=Homo sapiens GN=GPR98 PE=1 SV=2 | 5969 | PQLAEEsCSAM | 0.141 | -0.084 | -0.948 | -0.297 |
| sp\|Q8WXG9\|GPR98_HUMAN G-protein coupled receptor 98 OS=Homo sapiens GN=GPR98 PE=1 SV=2 | 5971 | LAEESCsAMAA | 0.391 | 0.198 | -0.396 | 0.064 |
| sp\|Q8WXG9\|GPR98_HUMAN G-protein coupled receptor 98 OS=Homo sapiens GN=GPR98 PE=1 SV=2 | 5977 | SAMAAVtHYLY | 0.36 | 0.021 | -0.645 | -0.088 |
| sp\|Q8WXG9\|GPR98_HUMAN G-protein coupled receptor 98 OS=Homo sapiens GN=GPR98 PE=1 SV=2 | 5986 | LYLCQFsWMLI | 0.295 | 0.188 | -0.456 | 0.009 |
| sp\|Q8WXG9\|GPR98_HUMAN G-protein coupled receptor 98 OS=Homo sapiens GN=GPR98 PE=1 SV=2 | 5992 | SWMLIQsVNFW | 0.095 | -0.021 | -0.944 | -0.29 |
| sp\|Q8WXG9\|GPR98_HUMAN G-protein coupled receptor 98 OS=Homo sapiens GN=GPR98 PE=1 SV=2 | 6006 | VMNDEHtERRY | 0.287 | 0.094 | -1.088 | -0.236 |
| sp\|Q8WXG9\|GPR98_HUMAN G-protein coupled receptor 98 OS=Homo sapiens GN=GPR98 PE=1 SV=2 | 6017 | LLFFLLsWGLP | 0.15 | 0.09 | -0.814 | -0.191 |
| sp\|Q8WXG9\|GPR98_HUMAN G-protein coupled receptor 98 OS=Homo sapiens GN=GPR98 PE=1 SV=2 | 6039 | KGIYHQsMSQI | 0.238 | 0.113 | -0.541 | -0.063 |
| sp\|Q8WXG9\|GPR98_HUMAN G-protein coupled receptor 98 OS=Homo sapiens GN=GPR98 PE=1 SV=2 | 6041 | IYHQSMsQIYG | 0.453 | 0.241 | -0.2 | 0.165 |
| sp\|Q8WXG9\|GPR98_HUMAN G-protein coupled receptor 98 OS=Homo sapiens GN=GPR98 PE=1 SV=2 | 6063 | VYAALFtAALV | 0.157 | -0.063 | -1.128 | -0.345 |
| sp\|Q8WXG9\|GPR98_HUMAN G-protein coupled receptor 98 OS=Homo sapiens GN=GPR98 PE=1 SV=2 | 6070 | AALVPLtCLVV | 0.047 | -0.202 | -1.697 | -0.617 |
| sp\|Q8WXG9\|GPR98_HUMAN G-protein coupled receptor 98 OS=Homo sapiens GN=GPR98 PE=1 SV=2 | 6100 | DVFRGRtNAAE | 0.35 | 0.703 | -0.439 | 0.205 |
| sp\|Q8WXG9\|GPR98_HUMAN G-protein coupled receptor 98 OS=Homo sapiens GN=GPR98 PE=1 SV=2 | 6116 | YLFALIsVTWL | 0.317 | 0.065 | -0.46 | -0.026 |
| sp\|Q8WXG9\|GPR98_HUMAN G-protein coupled receptor 98 OS=Homo sapiens GN=GPR98 PE=1 SV=2 | 6118 | FALISVtWLWG | 0.412 | 0.315 | -0.194 | 0.178 |
| sp\|Q8WXG9\|GPR98_HUMAN G-protein coupled receptor 98 OS=Homo sapiens GN=GPR98 PE=1 SV=2 | 6142 | LFVIFNsLQGL | 0.434 | 0.135 | -0.181 | 0.129 |
| sp\|Q8WXG9\|GPR98_HUMAN G-protein coupled receptor 98 OS=Homo sapiens GN=GPR98 PE=1 SV=2 | 6166 | CCPMKAsYTVE | 0.187 | -0.145 | -1.095 | -0.351 |
| sp\|Q8WXG9\|GPR98_HUMAN G-protein coupled receptor 98 OS=Homo sapiens GN=GPR98 PE=1 SV=2 | 6168 | PMKASYtVEMN | 0.377 | 0.362 | -0.106 | 0.211 |
| sp\|Q8WXG9\|GPR98_HUMAN G-protein coupled receptor 98 OS=Homo sapiens GN=GPR98 PE=1 SV=2 | 6178 | NGHPGPsTAFF | 0.03 | -0.454 | -1.718 | -0.714 |
| sp\|Q8WXG9\|GPR98_HUMAN G-protein coupled receptor 98 OS=Homo sapiens GN=GPR98 PE=1 SV=2 | 6179 | GHPGPStAFFT | 0.046 | -0.3 | -1.71 | -0.655 |
| sp\|Q8WXG9\|GPR98_HUMAN G-protein coupled receptor 98 OS=Homo sapiens GN=GPR98 PE=1 SV=2 | 6183 | PSTAFFtPGSG | 0.095 | -0.361 | -1.125 | -0.464 |
| sp\|Q8WXG9\|GPR98_HUMAN G-protein coupled receptor 98 OS=Homo sapiens GN=GPR98 PE=1 SV=2 | 6186 | AFFTPGsGMPP | 0.06 | -0.389 | -1.428 | -0.586 |
| sp\|Q8WXG9\|GPR98_HUMAN G-protein coupled receptor 98 OS=Homo sapiens GN=GPR98 PE=1 SV=2 | 6196 | PAGGEIsKSTQ | 0.075 | -0.28 | -1.355 | -0.52 |
| sp\|Q8WXG9\|GPR98_HUMAN G-protein coupled receptor 98 OS=Homo sapiens GN=GPR98 PE=1 SV=2 | 6198 | GGEISKsTQNL | 0.564 | 0.349 | 0.092 | 0.335 |
| sp\|Q8WXG9\|GPR98_HUMAN G-protein coupled receptor 98 OS=Homo sapiens GN=GPR98 PE=1 SV=2 | 6199 | GEISKStQNLI | 0.125 | -0.128 | -1.231 | -0.411 |
| sp\|Q8WXG9\|GPR98_HUMAN G-protein coupled receptor 98 OS=Homo sapiens GN=GPR98 PE=1 SV=2 | 6217 | PDWERAsFQQG | 0.345 | 0.245 | -0.336 | 0.085 |
| sp\|Q8WXG9\|GPR98_HUMAN G-protein coupled receptor 98 OS=Homo sapiens GN=GPR98 PE=1 SV=2 | 6222 | ASFQQGsQASP | 0.033 | -0.433 | -2.02 | -0.807 |
| sp\|Q8WXG9\|GPR98_HUMAN G-protein coupled receptor 98 OS=Homo sapiens GN=GPR98 PE=1 SV=2 | 6225 | QQGSQAsPDLK | 0.05 | -0.418 | -1.47 | -0.613 |
| sp\|Q8WXG9\|GPR98_HUMAN G-protein coupled receptor 98 OS=Homo sapiens GN=GPR98 PE=1 SV=2 | 6231 | SPDLKPsPQNG | 0.037 | -0.521 | -2.018 | -0.834 |
| sp\|Q8WXG9\|GPR98_HUMAN G-protein coupled receptor 98 OS=Homo sapiens GN=GPR98 PE=1 SV=2 | 6237 | SPQNGAtFPSS | 0.247 | 0.379 | -0.5 | 0.042 |
| sp\|Q8WXG9\|GPR98_HUMAN G-protein coupled receptor 98 OS=Homo sapiens GN=GPR98 PE=1 SV=2 | 6240 | NGATFPsSGGY | 0.237 | -0.156 | -0.762 | -0.227 |
| sp\|Q8WXG9\|GPR98_HUMAN G-protein coupled receptor 98 OS=Homo sapiens GN=GPR98 PE=1 SV=2 | 6241 | GATFPSsGGYG | 0.166 | -0.195 | -0.927 | -0.319 |
| sp\|Q8WXG9\|GPR98_HUMAN G-protein coupled receptor 98 OS=Homo sapiens GN=GPR98 PE=1 SV=2 | 6248 | GGYGQGsLIAD | 0.215 | 0.078 | -0.37 | -0.026 |
| sp\|Q8WXG9\|GPR98_HUMAN G-protein coupled receptor 98 OS=Homo sapiens GN=GPR98 PE=1 SV=2 | 6255 | LIADEEsQEFD | 0.115 | -0.163 | -1.394 | -0.481 |
| sp\|Q8WXG9\|GPR98_HUMAN G-protein coupled receptor 98 OS=Homo sapiens GN=GPR98 PE=1 SV=2 | 6267 | LIFALKtGAGL | 0.167 | -0.078 | -0.644 | -0.185 |
| sp\|Q8WXG9\|GPR98_HUMAN G-protein coupled receptor 98 OS=Homo sapiens GN=GPR98 PE=1 SV=2 | 6272 | KTGAGLsVSDN | 0.064 | -0.23 | -1.446 | -0.537 |
| sp\|Q8WXG9\|GPR98_HUMAN G-protein coupled receptor 98 OS=Homo sapiens GN=GPR98 PE=1 SV=2 | 6274 | GAGLSVsDNES | 0.558 | 0.224 | -0.274 | 0.169 |
| sp\|Q8WXG9\|GPR98_HUMAN G-protein coupled receptor 98 OS=Homo sapiens GN=GPR98 PE=1 SV=2 | 6278 | SVSDNEsGQGS | 0.074 | -0.276 | -1.37 | -0.524 |
| sp\|Q8WXG9\|GPR98_HUMAN G-protein coupled receptor 98 OS=Homo sapiens GN=GPR98 PE=1 SV=2 | 6282 | NESGQGsQEGG | 0.064 | -0.293 | -1.669 | -0.633 |
| sp\|Q8WXG9\|GPR98_HUMAN G-protein coupled receptor 98 OS=Homo sapiens GN=GPR98 PE=1 SV=2 | 6287 | GSQEGGtLTDS | 0.204 | -0.055 | -0.903 | -0.251 |
| sp\|Q8WXG9\|GPR98_HUMAN G-protein coupled receptor 98 OS=Homo sapiens GN=GPR98 PE=1 SV=2 | 6289 | QEGGTLtDSQI | 0.069 | -0.117 | -1.259 | -0.436 |
| sp\|Q8WXG9\|GPR98_HUMAN G-protein coupled receptor 98 OS=Homo sapiens GN=GPR98 PE=1 SV=2 | 6291 | GGTLTDsQIVE | 0.111 | -0.124 | -1.257 | -0.423 |
| sp\|Q8WXG9\|GPR98_HUMAN G-protein coupled receptor 98 OS=Homo sapiens GN=GPR98 PE=1 SV=2 | 6304 | RIPIADtHL-- | 0.198 | 0.14 | -1.037 | -0.233 |
| sp\|Q5T601\|AGRF1_HUMAN Adhesion G-protein coupled receptor F1 OS=Homo sapiens GN=ADGRF1 PE=1 S\ | 319 | MIVGNAtEAAV | 0.405 | 0.251 | -0.386 | 0.09 |
| sp\|Q5T601\|AGRF1_HUMAN Adhesion G-protein coupled receptor F1 OS=Homo sapiens GN=ADGRF1 PE=1 S\ | 324 | ATEAAVsSFVQ | 0.385 | -0.107 | -0.713 | -0.145 |
| sp\|Q5T601\|AGRF1_HUMAN Adhesion G-protein coupled receptor F1 OS=Homo sapiens GN=ADGRF1 PE=1 S\ | 325 | TEAAVSsFVQN | 0.16 | -0.132 | -0.929 | -0.3 |
| sp\|Q5T601\|AGRF1_HUMAN Adhesion G-protein coupled receptor F1 OS=Homo sapiens GN=ADGRF1 PE=1 S\ | 331 | SFVQNLsVIIR | 0.141 | -0.092 | -0.67 | -0.207 |
| sp\|Q5T601\|AGRF1_HUMAN Adhesion G-protein coupled receptor F1 OS=Homo sapiens GN=ADGRF1 PE=1 S\ | 339 | IIRQNPsTTVG | 0.612 | 0.23 | 0.007 | 0.283 |

| | | | | | | |
|---|---|---|---|---|---|---|
| sp\|Q5T601\|AGRF1_HUMAN Adhesion G-protein coupled receptor F1 OS=Homo sapiens GN=ADGRF1 PE=1 S\ | 340 | IRQNPStTVGN | 0.129 | 0.035 | -0.717 | -0.184 |
| sp\|Q5T601\|AGRF1_HUMAN Adhesion G-protein coupled receptor F1 OS=Homo sapiens GN=ADGRF1 PE=1 S\ | 341 | RQNPSTtVGNL | 0.403 | 0.288 | -0.186 | 0.168 |
| sp\|Q5T601\|AGRF1_HUMAN Adhesion G-protein coupled receptor F1 OS=Homo sapiens GN=ADGRF1 PE=1 S\ | 347 | TVGNLAsVVSI | 0.064 | -0.08 | -1.093 | -0.37 |
| sp\|Q5T601\|AGRF1_HUMAN Adhesion G-protein coupled receptor F1 OS=Homo sapiens GN=ADGRF1 PE=1 S\ | 350 | NLASVVsILSN | 0.172 | -0.092 | -0.863 | -0.261 |
| sp\|Q5T601\|AGRF1_HUMAN Adhesion G-protein coupled receptor F1 OS=Homo sapiens GN=ADGRF1 PE=1 S\ | 353 | SVVSILsNISS | 0.02 | -0.318 | -1.842 | -0.713 |
| sp\|Q5T601\|AGRF1_HUMAN Adhesion G-protein coupled receptor F1 OS=Homo sapiens GN=ADGRF1 PE=1 S\ | 356 | SILSNIsSLSL | 0.067 | -0.16 | -1.303 | -0.465 |
| sp\|Q5T601\|AGRF1_HUMAN Adhesion G-protein coupled receptor F1 OS=Homo sapiens GN=ADGRF1 PE=1 S\ | 357 | ILSNISsLSLA | 0.232 | -0.024 | -0.249 | -0.014 |
| sp\|Q5T601\|AGRF1_HUMAN Adhesion G-protein coupled receptor F1 OS=Homo sapiens GN=ADGRF1 PE=1 S\ | 359 | SNISSLsLASH | 0.064 | -0.17 | -1.277 | -0.461 |
| sp\|Q5T601\|AGRF1_HUMAN Adhesion G-protein coupled receptor F1 OS=Homo sapiens GN=ADGRF1 PE=1 S\ | 362 | SSLSLAsHFRV | 0.125 | -0.284 | -1.523 | -0.561 |
| sp\|Q5T601\|AGRF1_HUMAN Adhesion G-protein coupled receptor F1 OS=Homo sapiens GN=ADGRF1 PE=1 S\ | 367 | ASHFRVsNSTM | 0.217 | -0.133 | -1.007 | -0.308 |
| sp\|Q5T601\|AGRF1_HUMAN Adhesion G-protein coupled receptor F1 OS=Homo sapiens GN=ADGRF1 PE=1 S\ | 369 | HFRVSNsTMED | 0.727 | 0.58 | 0.394 | 0.567 |
| sp\|Q5T601\|AGRF1_HUMAN Adhesion G-protein coupled receptor F1 OS=Homo sapiens GN=ADGRF1 PE=1 S\ | 370 | FRVSNStMEDV | 0.235 | 0.296 | -0.88 | -0.116 |
| sp\|Q5T601\|AGRF1_HUMAN Adhesion G-protein coupled receptor F1 OS=Homo sapiens GN=ADGRF1 PE=1 S\ | 376 | TMEDVIsIADN | 0.179 | 0.002 | -0.921 | -0.247 |
| sp\|Q5T601\|AGRF1_HUMAN Adhesion G-protein coupled receptor F1 OS=Homo sapiens GN=ADGRF1 PE=1 S\ | 384 | ADNILNsASVT | 0.057 | -0.214 | -1.774 | -0.644 |
| sp\|Q5T601\|AGRF1_HUMAN Adhesion G-protein coupled receptor F1 OS=Homo sapiens GN=ADGRF1 PE=1 S\ | 386 | NILNSAsVTNW | 0.465 | 0.35 | -0.13 | 0.228 |
| sp\|Q5T601\|AGRF1_HUMAN Adhesion G-protein coupled receptor F1 OS=Homo sapiens GN=ADGRF1 PE=1 S\ | 388 | LNSASVtNWTV | 0.431 | 0.019 | -0.552 | -0.034 |
| sp\|Q5T601\|AGRF1_HUMAN Adhesion G-protein coupled receptor F1 OS=Homo sapiens GN=ADGRF1 PE=1 S\ | 391 | ASVTNWtVLLR | 0.151 | -0.187 | -1.044 | -0.36 |
| sp\|Q5T601\|AGRF1_HUMAN Adhesion G-protein coupled receptor F1 OS=Homo sapiens GN=ADGRF1 PE=1 S\ | 401 | REEKYAsSRLL | 0.434 | 0.033 | -0.537 | -0.023 |
| sp\|Q5T601\|AGRF1_HUMAN Adhesion G-protein coupled receptor F1 OS=Homo sapiens GN=ADGRF1 PE=1 S\ | 402 | EEKYASsRLLE | 0.111 | -0.135 | -1.104 | -0.376 |
| sp\|Q5T601\|AGRF1_HUMAN Adhesion G-protein coupled receptor F1 OS=Homo sapiens GN=ADGRF1 PE=1 S\ | 407 | SSRLLEtLENI | 0.35 | 0.181 | -0.708 | -0.059 |
| sp\|Q5T601\|AGRF1_HUMAN Adhesion G-protein coupled receptor F1 OS=Homo sapiens GN=ADGRF1 PE=1 S\ | 412 | ETLENIsTLVP | 0.073 | -0.188 | -1.3 | -0.472 |
| sp\|Q5T601\|AGRF1_HUMAN Adhesion G-protein coupled receptor F1 OS=Homo sapiens GN=ADGRF1 PE=1 S\ | 413 | TLENIStLVPP | 0.143 | -0.086 | -0.516 | -0.153 |
| sp\|Q5T601\|AGRF1_HUMAN Adhesion G-protein coupled receptor F1 OS=Homo sapiens GN=ADGRF1 PE=1 S\ | 418 | STLVPPtALPL | 0.049 | -0.302 | -1.617 | -0.623 |
| sp\|Q5T601\|AGRF1_HUMAN Adhesion G-protein coupled receptor F1 OS=Homo sapiens GN=ADGRF1 PE=1 S\ | 425 | ALPLNFsRKFI | 0.183 | -0.118 | -0.833 | -0.256 |
| sp\|Q5T601\|AGRF1_HUMAN Adhesion G-protein coupled receptor F1 OS=Homo sapiens GN=ADGRF1 PE=1 S\ | 439 | GIPVNKsQLKR | 0.136 | -0.044 | -1.053 | -0.32 |
| sp\|Q5T601\|AGRF1_HUMAN Adhesion G-protein coupled receptor F1 OS=Homo sapiens GN=ADGRF1 PE=1 S\ | 446 | QLKRGYsYQIK | 0.648 | 0.886 | 0.64 | 0.725 |
| sp\|Q5T601\|AGRF1_HUMAN Adhesion G-protein coupled receptor F1 OS=Homo sapiens GN=ADGRF1 PE=1 S\ | 456 | KMCPQNtSIPI | 0.106 | -0.016 | -1.086 | -0.332 |
| sp\|Q5T601\|AGRF1_HUMAN Adhesion G-protein coupled receptor F1 OS=Homo sapiens GN=ADGRF1 PE=1 S\ | 457 | MCPQNTsIPIR | 0.698 | 0.506 | 0.333 | 0.512 |
| sp\|Q5T601\|AGRF1_HUMAN Adhesion G-protein coupled receptor F1 OS=Homo sapiens GN=ADGRF1 PE=1 S\ | 468 | GRVLIGsDQFQ | 0.159 | 0.073 | -1.077 | -0.282 |
| sp\|Q5T601\|AGRF1_HUMAN Adhesion G-protein coupled receptor F1 OS=Homo sapiens GN=ADGRF1 PE=1 S\ | 474 | SDQFQRsLPET | 0.382 | 0.431 | -0.411 | 0.134 |
| sp\|Q5T601\|AGRF1_HUMAN Adhesion G-protein coupled receptor F1 OS=Homo sapiens GN=ADGRF1 PE=1 S\ | 478 | QRSLPEtIISM | 0.053 | -0.104 | -1.184 | -0.412 |
| sp\|Q5T601\|AGRF1_HUMAN Adhesion G-protein coupled receptor F1 OS=Homo sapiens GN=ADGRF1 PE=1 S\ | 481 | LPETIIsMASL | 0.086 | -0.083 | -1.047 | -0.348 |
| sp\|Q5T601\|AGRF1_HUMAN Adhesion G-protein coupled receptor F1 OS=Homo sapiens GN=ADGRF1 PE=1 S\ | 484 | TIISMAsLTLG | 0.435 | 0.059 | -0.41 | 0.028 |
| sp\|Q5T601\|AGRF1_HUMAN Adhesion G-protein coupled receptor F1 OS=Homo sapiens GN=ADGRF1 PE=1 S\ | 486 | ISMASLtLGNI | 0.641 | 0.327 | 0.326 | 0.431 |
| sp\|Q5T601\|AGRF1_HUMAN Adhesion G-protein coupled receptor F1 OS=Homo sapiens GN=ADGRF1 PE=1 S\ | 494 | GNILPVsKNGN | 0.284 | -0.132 | -0.522 | -0.123 |
| sp\|Q5T601\|AGRF1_HUMAN Adhesion G-protein coupled receptor F1 OS=Homo sapiens GN=ADGRF1 PE=1 S\ | 507 | VNGPVIsTVIQ | 0.081 | -0.319 | -1.342 | -0.527 |
| sp\|Q5T601\|AGRF1_HUMAN Adhesion G-protein coupled receptor F1 OS=Homo sapiens GN=ADGRF1 PE=1 S\ | 508 | NGPVIStVIQN | 0.062 | -0.199 | -1.063 | -0.4 |
| sp\|Q5T601\|AGRF1_HUMAN Adhesion G-protein coupled receptor F1 OS=Homo sapiens GN=ADGRF1 PE=1 S\ | 514 | TVIQNYsINEV | 0.228 | 0.054 | -0.49 | -0.069 |
| sp\|Q5T601\|AGRF1_HUMAN Adhesion G-protein coupled receptor F1 OS=Homo sapiens GN=ADGRF1 PE=1 S\ | 523 | EVFLFFsKIES | 0.111 | -0.022 | -0.925 | -0.279 |
| sp\|Q5T601\|AGRF1_HUMAN Adhesion G-protein coupled receptor F1 OS=Homo sapiens GN=ADGRF1 PE=1 S\ | 527 | FFSKIEsNLSQ | 0.117 | -0.118 | -1.152 | -0.384 |
| sp\|Q5T601\|AGRF1_HUMAN Adhesion G-protein coupled receptor F1 OS=Homo sapiens GN=ADGRF1 PE=1 S\ | 530 | KIESNLsQPHC | 0.248 | 0.196 | -0.71 | -0.089 |
| sp\|Q5T601\|AGRF1_HUMAN Adhesion G-protein coupled receptor F1 OS=Homo sapiens GN=ADGRF1 PE=1 S\ | 540 | CVFWDFsHLQW | 0.212 | -0.027 | -0.59 | -0.135 |
| sp\|Q5T601\|AGRF1_HUMAN Adhesion G-protein coupled receptor F1 OS=Homo sapiens GN=ADGRF1 PE=1 S\ | 555 | CHLVNEtQDIV | 0.183 | -0.107 | -1.379 | -0.434 |
| sp\|Q5T601\|AGRF1_HUMAN Adhesion G-protein coupled receptor F1 OS=Homo sapiens GN=ADGRF1 PE=1 S\ | 560 | ETQDIVtCQCT | 0.299 | 0.093 | -0.591 | -0.066 |
| sp\|Q5T601\|AGRF1_HUMAN Adhesion G-protein coupled receptor F1 OS=Homo sapiens GN=ADGRF1 PE=1 S\ | 564 | IVTCQCtHLTS | 0.179 | -0.104 | -1.129 | -0.351 |
| sp\|Q5T601\|AGRF1_HUMAN Adhesion G-protein coupled receptor F1 OS=Homo sapiens GN=ADGRF1 PE=1 S\ | 567 | CQCTHLtSFSI | 0.146 | -0.041 | -0.766 | -0.22 |
| sp\|Q5T601\|AGRF1_HUMAN Adhesion G-protein coupled receptor F1 OS=Homo sapiens GN=ADGRF1 PE=1 S\ | 568 | QCTHLTsFSIL | 0.203 | -0.018 | -0.763 | -0.193 |
| sp\|Q5T601\|AGRF1_HUMAN Adhesion G-protein coupled receptor F1 OS=Homo sapiens GN=ADGRF1 PE=1 S\ | 570 | THLTSFsILMS | 0.124 | -0.002 | -1.094 | -0.324 |
| sp\|Q5T601\|AGRF1_HUMAN Adhesion G-protein coupled receptor F1 OS=Homo sapiens GN=ADGRF1 PE=1 S\ | 574 | SFSILMsPFVP | 0.05 | -0.316 | -1.465 | -0.577 |

| | | | | | | |
|---|---|---|---|---|---|---|
| sp\|Q5T601\|AGRF1_HUMAN Adhesion G-protein coupled receptor F1 OS=Homo sapiens GN=ADGRF1 PE=1 S\ | 579 | MSPFVPsTIFP | 0.077 | -0.276 | -1.372 | -0.524 |
| sp\|Q5T601\|AGRF1_HUMAN Adhesion G-protein coupled receptor F1 OS=Homo sapiens GN=ADGRF1 PE=1 S\ | 580 | SPFVPStIFPV | 0.031 | -0.391 | -1.824 | -0.728 |
| sp\|Q5T601\|AGRF1_HUMAN Adhesion G-protein coupled receptor F1 OS=Homo sapiens GN=ADGRF1 PE=1 S\ | 589 | PVVKWItYVGL | 0.28 | 0.038 | -0.456 | -0.046 |
| sp\|Q5T601\|AGRF1_HUMAN Adhesion G-protein coupled receptor F1 OS=Homo sapiens GN=ADGRF1 PE=1 S\ | 596 | YVGLGIsIGSL | 0.056 | -0.12 | -1.132 | -0.399 |
| sp\|Q5T601\|AGRF1_HUMAN Adhesion G-protein coupled receptor F1 OS=Homo sapiens GN=ADGRF1 PE=1 S\ | 599 | LGISIGsLILC | 0.099 | -0.142 | -0.86 | -0.301 |
| sp\|Q5T601\|AGRF1_HUMAN Adhesion G-protein coupled receptor F1 OS=Homo sapiens GN=ADGRF1 PE=1 S\ | 617 | WKQIKKsQTSH | 0.151 | -0.145 | -1.073 | -0.356 |
| sp\|Q5T601\|AGRF1_HUMAN Adhesion G-protein coupled receptor F1 OS=Homo sapiens GN=ADGRF1 PE=1 S\ | 619 | QIKKSQtSHTR | 0.62 | 0.317 | 0.195 | 0.377 |
| sp\|Q5T601\|AGRF1_HUMAN Adhesion G-protein coupled receptor F1 OS=Homo sapiens GN=ADGRF1 PE=1 S\ | 620 | IKKSQTsHTRR | 0.451 | -0.067 | -0.47 | -0.029 |
| sp\|Q5T601\|AGRF1_HUMAN Adhesion G-protein coupled receptor F1 OS=Homo sapiens GN=ADGRF1 PE=1 S\ | 622 | KSQTSHtRRIC | 0.376 | 0.031 | -0.738 | -0.11 |
| sp\|Q5T601\|AGRF1_HUMAN Adhesion G-protein coupled receptor F1 OS=Homo sapiens GN=ADGRF1 PE=1 S\ | 633 | MVNIALsLLIA | 0.278 | 0.123 | -0.551 | -0.05 |
| sp\|Q5T601\|AGRF1_HUMAN Adhesion G-protein coupled receptor F1 OS=Homo sapiens GN=ADGRF1 PE=1 S\ | 646 | WFIVGAtVDTT | 0.123 | -0.098 | -0.804 | -0.26 |
| sp\|Q5T601\|AGRF1_HUMAN Adhesion G-protein coupled receptor F1 OS=Homo sapiens GN=ADGRF1 PE=1 S\ | 649 | VGATVDtTVNP | 0.108 | -0.248 | -1.431 | -0.524 |
| sp\|Q5T601\|AGRF1_HUMAN Adhesion G-protein coupled receptor F1 OS=Homo sapiens GN=ADGRF1 PE=1 S\ | 650 | GATVDTtVNPS | 0.131 | -0.138 | -0.927 | -0.311 |
| sp\|Q5T601\|AGRF1_HUMAN Adhesion G-protein coupled receptor F1 OS=Homo sapiens GN=ADGRF1 PE=1 S\ | 654 | DTTVNPsGVCT | 0.104 | -0.212 | -1.178 | -0.429 |
| sp\|Q5T601\|AGRF1_HUMAN Adhesion G-protein coupled receptor F1 OS=Homo sapiens GN=ADGRF1 PE=1 S\ | 658 | NPSGVCtAAVF | 0.135 | -0.11 | -1.276 | -0.417 |
| sp\|Q5T601\|AGRF1_HUMAN Adhesion G-protein coupled receptor F1 OS=Homo sapiens GN=ADGRF1 PE=1 S\ | 664 | TAAVFFtHFFY | 0.135 | -0.137 | -1.043 | -0.348 |
| sp\|Q5T601\|AGRF1_HUMAN Adhesion G-protein coupled receptor F1 OS=Homo sapiens GN=ADGRF1 PE=1 S\ | 670 | THFFYLsLFFW | 0.158 | -0.018 | -0.993 | -0.284 |
| sp\|Q5T601\|AGRF1_HUMAN Adhesion G-protein coupled receptor F1 OS=Homo sapiens GN=ADGRF1 PE=1 S\ | 714 | GCPLIIsVITI | 0.294 | 0.039 | -0.488 | -0.052 |
| sp\|Q5T601\|AGRF1_HUMAN Adhesion G-protein coupled receptor F1 OS=Homo sapiens GN=ADGRF1 PE=1 S\ | 717 | LIISVItIAVT | 0.106 | -0.134 | -1.057 | -0.362 |
| sp\|Q5T601\|AGRF1_HUMAN Adhesion G-protein coupled receptor F1 OS=Homo sapiens GN=ADGRF1 PE=1 S\ | 721 | VITIAVtQPSN | 0.621 | 0.53 | -0.026 | 0.375 |
| sp\|Q5T601\|AGRF1_HUMAN Adhesion G-protein coupled receptor F1 OS=Homo sapiens GN=ADGRF1 PE=1 S\ | 724 | IAVTQPsNTYK | 0.113 | -0.334 | -1.545 | -0.589 |
| sp\|Q5T601\|AGRF1_HUMAN Adhesion G-protein coupled receptor F1 OS=Homo sapiens GN=ADGRF1 PE=1 S\ | 726 | VTQPSNtYKRK | 0.316 | -0.016 | -0.686 | -0.129 |
| sp\|Q5T601\|AGRF1_HUMAN Adhesion G-protein coupled receptor F1 OS=Homo sapiens GN=ADGRF1 PE=1 S\ | 738 | VCWLNWsNGSK | 0.168 | -0.039 | -1.063 | -0.311 |
| sp\|Q5T601\|AGRF1_HUMAN Adhesion G-protein coupled receptor F1 OS=Homo sapiens GN=ADGRF1 PE=1 S\ | 741 | LNWSNGsKPLL | 0.296 | 0.392 | -0.189 | 0.166 |
| sp\|Q5T601\|AGRF1_HUMAN Adhesion G-protein coupled receptor F1 OS=Homo sapiens GN=ADGRF1 PE=1 S\ | 767 | VVLLVLtKLWR | 0.081 | -0.134 | -1.096 | -0.383 |
| sp\|Q5T601\|AGRF1_HUMAN Adhesion G-protein coupled receptor F1 OS=Homo sapiens GN=ADGRF1 PE=1 S\ | 773 | TKLWRPtVGER | 0.686 | 0.173 | 0.191 | 0.35 |
| sp\|Q5T601\|AGRF1_HUMAN Adhesion G-protein coupled receptor F1 OS=Homo sapiens GN=ADGRF1 PE=1 S\ | 779 | TVGERLsRDDK | 0.128 | -0.035 | -0.981 | -0.296 |
| sp\|Q5T601\|AGRF1_HUMAN Adhesion G-protein coupled receptor F1 OS=Homo sapiens GN=ADGRF1 PE=1 S\ | 785 | SRDDKAtIIRV | 0.091 | -0.034 | -1.512 | -0.485 |
| sp\|Q5T601\|AGRF1_HUMAN Adhesion G-protein coupled receptor F1 OS=Homo sapiens GN=ADGRF1 PE=1 S\ | 792 | IIRVGKsLLIL | 0.535 | 0.362 | 0.109 | 0.335 |
| sp\|Q5T601\|AGRF1_HUMAN Adhesion G-protein coupled receptor F1 OS=Homo sapiens GN=ADGRF1 PE=1 S\ | 797 | KSLLILtPLLG | 0.038 | -0.48 | -1.604 | -0.682 |
| sp\|Q5T601\|AGRF1_HUMAN Adhesion G-protein coupled receptor F1 OS=Homo sapiens GN=ADGRF1 PE=1 S\ | 803 | TPLLGLtWGFG | 0.058 | -0.163 | -1.345 | -0.483 |
| sp\|Q5T601\|AGRF1_HUMAN Adhesion G-protein coupled receptor F1 OS=Homo sapiens GN=ADGRF1 PE=1 S\ | 810 | WGFGIGtIVDS | 0.096 | -0.165 | -0.849 | -0.306 |
| sp\|Q5T601\|AGRF1_HUMAN Adhesion G-protein coupled receptor F1 OS=Homo sapiens GN=ADGRF1 PE=1 S\ | 814 | IGTIVDsQNLA | 0.207 | -0.16 | -0.862 | -0.272 |
| sp\|Q5T601\|AGRF1_HUMAN Adhesion G-protein coupled receptor F1 OS=Homo sapiens GN=ADGRF1 PE=1 S\ | 843 | FGILLDsKLRQ | 0.081 | -0.242 | -1.614 | -0.592 |
| sp\|Q5T601\|AGRF1_HUMAN Adhesion G-protein coupled receptor F1 OS=Homo sapiens GN=ADGRF1 PE=1 S\ | 854 | LLFNKLsALSS | 0.044 | -0.101 | -1.4 | -0.486 |
| sp\|Q5T601\|AGRF1_HUMAN Adhesion G-protein coupled receptor F1 OS=Homo sapiens GN=ADGRF1 PE=1 S\ | 857 | NKLSALsSWKQ | 0.227 | -0.051 | -0.85 | -0.225 |
| sp\|Q5T601\|AGRF1_HUMAN Adhesion G-protein coupled receptor F1 OS=Homo sapiens GN=ADGRF1 PE=1 S\ | 858 | KLSALSsWKQT | 0.092 | -0.167 | -1.084 | -0.386 |
| sp\|Q5T601\|AGRF1_HUMAN Adhesion G-protein coupled receptor F1 OS=Homo sapiens GN=ADGRF1 PE=1 S\ | 862 | LSSWKQtEKQN | 0.153 | -0.15 | -0.695 | -0.231 |
| sp\|Q5T601\|AGRF1_HUMAN Adhesion G-protein coupled receptor F1 OS=Homo sapiens GN=ADGRF1 PE=1 S\ | 867 | QTEKQNsSDLS | 0.27 | -0.007 | -0.852 | -0.196 |
| sp\|Q5T601\|AGRF1_HUMAN Adhesion G-protein coupled receptor F1 OS=Homo sapiens GN=ADGRF1 PE=1 S\ | 868 | TEKQNSsDLSA | 0.101 | -0.186 | -1.111 | -0.399 |
| sp\|Q5T601\|AGRF1_HUMAN Adhesion G-protein coupled receptor F1 OS=Homo sapiens GN=ADGRF1 PE=1 S\ | 871 | QNSSDLsAKPK | 0.045 | -0.407 | -1.51 | -0.624 |
| sp\|Q5T601\|AGRF1_HUMAN Adhesion G-protein coupled receptor F1 OS=Homo sapiens GN=ADGRF1 PE=1 S\ | 877 | SAKPKFsKPFN | 0.273 | 0.265 | -0.457 | 0.027 |
| sp\|Q5T601\|AGRF1_HUMAN Adhesion G-protein coupled receptor F1 OS=Homo sapiens GN=ADGRF1 PE=1 S\ | 892 | KGHYAFsHTGD | 0.335 | 0.003 | -0.327 | 0.004 |
| sp\|Q5T601\|AGRF1_HUMAN Adhesion G-protein coupled receptor F1 OS=Homo sapiens GN=ADGRF1 PE=1 S\ | 894 | HYAFSHtGDSS | 0.386 | 0.363 | -0.029 | 0.24 |
| sp\|Q5T601\|AGRF1_HUMAN Adhesion G-protein coupled receptor F1 OS=Homo sapiens GN=ADGRF1 PE=1 S\ | 897 | FSHTGDsSDNI | 0.128 | -0.131 | -1.279 | -0.427 |
| sp\|Q5T601\|AGRF1_HUMAN Adhesion G-protein coupled receptor F1 OS=Homo sapiens GN=ADGRF1 PE=1 S\ | 898 | SHTGDSsDNIM | 0.107 | -0.167 | -1.323 | -0.461 |
| sp\|Q5T601\|AGRF1_HUMAN Adhesion G-protein coupled receptor F1 OS=Homo sapiens GN=ADGRF1 PE=1 S\ | 904 | SDNIMLtQFVS | 0.034 | -0.338 | -1.917 | -0.74 |
| sp\|Q5T601\|AGRF1_HUMAN Adhesion G-protein coupled receptor F1 OS=Homo sapiens GN=ADGRF1 PE=1 S\ | 908 | MLTQFVsNE-- | 0.511 | 0.229 | -0.344 | 0.132 |
| sp\|Q8IZF7\|AGRF2_HUMAN Adhesion G-protein coupled receptor F2 OS=Homo sapiens GN=ADGRF2 PE=2 SV | 315 | SMRINDtSNEV | 0.259 | 0.199 | -0.938 | -0.16 |

| | | | | | | |
|---|---|---|---|---|---|---|
| sp\|Q8IZF7\|AGRF2_HUMAN Adhesion G-protein coupled receptor F2 OS=Homo sapiens GN=ADGRF2 PE=2 SV | 316 | MRINDTsNEVT | 0.144 | 0.177 | -0.825 | -0.168 |
| sp\|Q8IZF7\|AGRF2_HUMAN Adhesion G-protein coupled receptor F2 OS=Homo sapiens GN=ADGRF2 PE=2 SV | 320 | DTSNEVtGRVL | 0.251 | -0.184 | -0.886 | -0.273 |
| sp\|Q8IZF7\|AGRF2_HUMAN Adhesion G-protein coupled receptor F2 OS=Homo sapiens GN=ADGRF2 PE=2 SV | 326 | TGRVLIsRDEL | 0.268 | 0.102 | -0.626 | -0.085 |
| sp\|Q8IZF7\|AGRF2_HUMAN Adhesion G-protein coupled receptor F2 OS=Homo sapiens GN=ADGRF2 PE=2 SV | 335 | ELRKVPsPSQV | 0.306 | -0.091 | -0.807 | -0.197 |
| sp\|Q8IZF7\|AGRF2_HUMAN Adhesion G-protein coupled receptor F2 OS=Homo sapiens GN=ADGRF2 PE=2 SV | 337 | RKVPSPsQVIS | 0.371 | 0.1 | -0.602 | -0.044 |
| sp\|Q8IZF7\|AGRF2_HUMAN Adhesion G-protein coupled receptor F2 OS=Homo sapiens GN=ADGRF2 PE=2 SV | 341 | SPSQVIsIAFP | 0.055 | -0.413 | -1.576 | -0.645 |
| sp\|Q8IZF7\|AGRF2_HUMAN Adhesion G-protein coupled receptor F2 OS=Homo sapiens GN=ADGRF2 PE=2 SV | 346 | ISIAFPtIGAI | 0.318 | -0.06 | -0.366 | -0.036 |
| sp\|Q8IZF7\|AGRF2_HUMAN Adhesion G-protein coupled receptor F2 OS=Homo sapiens GN=ADGRF2 PE=2 SV | 354 | GAILEAsLLEN | 0.381 | 0.084 | -0.808 | -0.114 |
| sp\|Q8IZF7\|AGRF2_HUMAN Adhesion G-protein coupled receptor F2 OS=Homo sapiens GN=ADGRF2 PE=2 SV | 360 | SLLENVtVNGL | 0.342 | 0.029 | -0.309 | 0.021 |
| sp\|Q8IZF7\|AGRF2_HUMAN Adhesion G-protein coupled receptor F2 OS=Homo sapiens GN=ADGRF2 PE=2 SV | 367 | VNGLVLsAILP | 0.044 | -0.247 | -1.556 | -0.586 |
| sp\|Q8IZF7\|AGRF2_HUMAN Adhesion G-protein coupled receptor F2 OS=Homo sapiens GN=ADGRF2 PE=2 SV | 378 | KELKRIsLIFE | 0.48 | 0.096 | -0.224 | 0.117 |
| sp\|Q8IZF7\|AGRF2_HUMAN Adhesion G-protein coupled receptor F2 OS=Homo sapiens GN=ADGRF2 PE=2 SV | 385 | LIFEKIsKSEE | 0.106 | -0.119 | -1.111 | -0.375 |
| sp\|Q8IZF7\|AGRF2_HUMAN Adhesion G-protein coupled receptor F2 OS=Homo sapiens GN=ADGRF2 PE=2 SV | 387 | FEKISKsEERR | 0.578 | 0.359 | 0.057 | 0.331 |
| sp\|Q8IZF7\|AGRF2_HUMAN Adhesion G-protein coupled receptor F2 OS=Homo sapiens GN=ADGRF2 PE=2 SV | 392 | KSEERRtQCVG | 0.103 | -0.151 | -1.491 | -0.513 |
| sp\|Q8IZF7\|AGRF2_HUMAN Adhesion G-protein coupled receptor F2 OS=Homo sapiens GN=ADGRF2 PE=2 SV | 399 | QCVGWHsVENR | 0.305 | 0.219 | -0.641 | -0.039 |
| sp\|Q8IZF7\|AGRF2_HUMAN Adhesion G-protein coupled receptor F2 OS=Homo sapiens GN=ADGRF2 PE=2 SV | 416 | KMIQENsQQAV | 0.247 | 0.106 | -1.098 | -0.248 |
| sp\|Q8IZF7\|AGRF2_HUMAN Adhesion G-protein coupled receptor F2 OS=Homo sapiens GN=ADGRF2 PE=2 SV | 426 | VCKCRPsKLFT | 0.263 | -0.065 | -0.831 | -0.211 |
| sp\|Q8IZF7\|AGRF2_HUMAN Adhesion G-protein coupled receptor F2 OS=Homo sapiens GN=ADGRF2 PE=2 SV | 430 | RPSKLFtSFSI | 0.194 | -0.03 | -0.774 | -0.203 |
| sp\|Q8IZF7\|AGRF2_HUMAN Adhesion G-protein coupled receptor F2 OS=Homo sapiens GN=ADGRF2 PE=2 SV | 431 | PSKLFTsFSIL | 0.293 | -0.007 | -0.591 | -0.102 |
| sp\|Q8IZF7\|AGRF2_HUMAN Adhesion G-protein coupled receptor F2 OS=Homo sapiens GN=ADGRF2 PE=2 SV | 433 | KLFTSFsILMS | 0.248 | 0.126 | -0.629 | -0.085 |
| sp\|Q8IZF7\|AGRF2_HUMAN Adhesion G-protein coupled receptor F2 OS=Homo sapiens GN=ADGRF2 PE=2 SV | 437 | SFSILMsPHIL | 0.099 | -0.232 | -1.24 | -0.458 |
| sp\|Q8IZF7\|AGRF2_HUMAN Adhesion G-protein coupled receptor F2 OS=Homo sapiens GN=ADGRF2 PE=2 SV | 443 | SPHILEsLILT | 0.051 | -0.2 | -1.456 | -0.535 |
| sp\|Q8IZF7\|AGRF2_HUMAN Adhesion G-protein coupled receptor F2 OS=Homo sapiens GN=ADGRF2 PE=2 SV | 447 | LESLILtYITY | 0.069 | -0.186 | -1.142 | -0.42 |
| sp\|Q8IZF7\|AGRF2_HUMAN Adhesion G-protein coupled receptor F2 OS=Homo sapiens GN=ADGRF2 PE=2 SV | 450 | LILTYItYVGL | 0.198 | -0.03 | -0.628 | -0.153 |
| sp\|Q8IZF7\|AGRF2_HUMAN Adhesion G-protein coupled receptor F2 OS=Homo sapiens GN=ADGRF2 PE=2 SV | 457 | YVGLGIsICSL | 0.033 | -0.266 | -1.519 | -0.584 |
| sp\|Q8IZF7\|AGRF2_HUMAN Adhesion G-protein coupled receptor F2 OS=Homo sapiens GN=ADGRF2 PE=2 SV | 460 | LGISICsLILC | 0.203 | -0.043 | -0.642 | -0.161 |
| sp\|Q8IZF7\|AGRF2_HUMAN Adhesion G-protein coupled receptor F2 OS=Homo sapiens GN=ADGRF2 PE=2 SV | 466 | SLILCLsIEVL | 0.136 | 0.025 | -0.997 | -0.279 |
| sp\|Q8IZF7\|AGRF2_HUMAN Adhesion G-protein coupled receptor F2 OS=Homo sapiens GN=ADGRF2 PE=2 SV | 473 | IEVLVWsQVTK | 0.159 | -0.245 | -1.353 | -0.48 |
| sp\|Q8IZF7\|AGRF2_HUMAN Adhesion G-protein coupled receptor F2 OS=Homo sapiens GN=ADGRF2 PE=2 SV | 476 | LVWSQVtKTEI | 0.255 | 0.068 | -0.555 | -0.077 |
| sp\|Q8IZF7\|AGRF2_HUMAN Adhesion G-protein coupled receptor F2 OS=Homo sapiens GN=ADGRF2 PE=2 SV | 478 | WSQVTKtEITY | 0.186 | 0.08 | -0.487 | -0.074 |
| sp\|Q8IZF7\|AGRF2_HUMAN Adhesion G-protein coupled receptor F2 OS=Homo sapiens GN=ADGRF2 PE=2 SV | 481 | VTKTEItYLRH | 0.094 | -0.399 | -1.679 | -0.661 |
| sp\|Q8IZF7\|AGRF2_HUMAN Adhesion G-protein coupled receptor F2 OS=Homo sapiens GN=ADGRF2 PE=2 SV | 494 | IVNIAAtLLMA | 0.339 | 0.055 | -0.496 | -0.034 |
| sp\|Q8IZF7\|AGRF2_HUMAN Adhesion G-protein coupled receptor F2 OS=Homo sapiens GN=ADGRF2 PE=2 SV | 506 | VWFIVAsFLSG | 0.177 | 0.027 | -0.965 | -0.254 |
| sp\|Q8IZF7\|AGRF2_HUMAN Adhesion G-protein coupled receptor F2 OS=Homo sapiens GN=ADGRF2 PE=2 SV | 509 | IVASFLsGPIT | 0.308 | 0.228 | -0.337 | 0.066 |
| sp\|Q8IZF7\|AGRF2_HUMAN Adhesion G-protein coupled receptor F2 OS=Homo sapiens GN=ADGRF2 PE=2 SV | 513 | FLSGPItHHKG | 0.327 | 0.031 | -0.462 | -0.035 |
| sp\|Q8IZF7\|AGRF2_HUMAN Adhesion G-protein coupled receptor F2 OS=Homo sapiens GN=ADGRF2 PE=2 SV | 522 | KGCVAAtFFVH | 0.267 | 0 | -0.751 | -0.161 |
| sp\|Q8IZF7\|AGRF2_HUMAN Adhesion G-protein coupled receptor F2 OS=Homo sapiens GN=ADGRF2 PE=2 SV | 531 | VHFFYLsVFFW | 0.11 | -0.106 | -1.327 | -0.441 |
| sp\|Q8IZF7\|AGRF2_HUMAN Adhesion G-protein coupled receptor F2 OS=Homo sapiens GN=ADGRF2 PE=2 SV | 553 | IMIVFHtLPKS | 0.595 | 0.713 | 0.056 | 0.455 |
| sp\|Q8IZF7\|AGRF2_HUMAN Adhesion G-protein coupled receptor F2 OS=Homo sapiens GN=ADGRF2 PE=2 SV | 557 | FHTLPKsVLVA | 0.167 | 0.012 | -0.816 | -0.212 |
| sp\|Q8IZF7\|AGRF2_HUMAN Adhesion G-protein coupled receptor F2 OS=Homo sapiens GN=ADGRF2 PE=2 SV | 562 | KSVLVAsLFSV | 0.225 | -0.042 | -0.984 | -0.267 |
| sp\|Q8IZF7\|AGRF2_HUMAN Adhesion G-protein coupled receptor F2 OS=Homo sapiens GN=ADGRF2 PE=2 SV | 565 | LVASLFsVGYG | 0.137 | -0.069 | -0.797 | -0.243 |
| sp\|Q8IZF7\|AGRF2_HUMAN Adhesion G-protein coupled receptor F2 OS=Homo sapiens GN=ADGRF2 PE=2 SV | 578 | LAIAAItVAAT | 0.221 | -0.049 | -0.594 | -0.141 |
| sp\|Q8IZF7\|AGRF2_HUMAN Adhesion G-protein coupled receptor F2 OS=Homo sapiens GN=ADGRF2 PE=2 SV | 582 | AITVAAtEPGK | 0.719 | 0.582 | 0.332 | 0.544 |
| sp\|Q8IZF7\|AGRF2_HUMAN Adhesion G-protein coupled receptor F2 OS=Homo sapiens GN=ADGRF2 PE=2 SV | 601 | WLNWDMtKALL | 0.327 | 0.081 | -0.328 | 0.027 |
| sp\|Q8IZF7\|AGRF2_HUMAN Adhesion G-protein coupled receptor F2 OS=Homo sapiens GN=ADGRF2 PE=2 SV | 621 | VVVNLItVTLV | 0.114 | -0.11 | -1.179 | -0.392 |
| sp\|Q8IZF7\|AGRF2_HUMAN Adhesion G-protein coupled receptor F2 OS=Homo sapiens GN=ADGRF2 PE=2 SV | 623 | VNLITVtLVIV | 0.289 | -0.012 | -0.637 | -0.12 |
| sp\|Q8IZF7\|AGRF2_HUMAN Adhesion G-protein coupled receptor F2 OS=Homo sapiens GN=ADGRF2 PE=2 SV | 629 | TLVIVKtQRAA | 0.356 | 0.035 | -0.441 | -0.017 |
| sp\|Q8IZF7\|AGRF2_HUMAN Adhesion G-protein coupled receptor F2 OS=Homo sapiens GN=ADGRF2 PE=2 SV | 637 | RAAIGNsMFQE | 0.185 | 0.008 | -0.894 | -0.234 |
| sp\|Q8IZF7\|AGRF2_HUMAN Adhesion G-protein coupled receptor F2 OS=Homo sapiens GN=ADGRF2 PE=2 SV | 649 | RAIVRIsKNIA | 0.386 | 0.1 | -0.39 | 0.032 |

| | | | | | | |
|---|---|---|---|---|---|---|
| sp\|Q8IZF7\|AGRF2_HUMAN Adhesion G-protein coupled receptor F2 OS=Homo sapiens GN=ADGRF2 PE=2 SV | 656 | KNIAILtPLLG | 0.028 | -0.511 | -1.655 | -0.713 |
| sp\|Q8IZF7\|AGRF2_HUMAN Adhesion G-protein coupled receptor F2 OS=Homo sapiens GN=ADGRF2 PE=2 SV | 662 | TPLLGLtWGFG | 0.058 | -0.163 | -1.345 | -0.483 |
| sp\|Q8IZF7\|AGRF2_HUMAN Adhesion G-protein coupled receptor F2 OS=Homo sapiens GN=ADGRF2 PE=2 SV | 669 | WGFGVAtVIDD | 0.352 | 0.059 | -0.226 | 0.062 |
| sp\|Q8IZF7\|AGRF2_HUMAN Adhesion G-protein coupled receptor F2 OS=Homo sapiens GN=ADGRF2 PE=2 SV | 675 | TVIDDRsLAFH | 0.059 | -0.24 | -1.601 | -0.594 |
| sp\|Q8IZF7\|AGRF2_HUMAN Adhesion G-protein coupled receptor F2 OS=Homo sapiens GN=ADGRF2 PE=2 SV | 683 | AFHIIFsLLNA | 0.211 | 0.057 | -0.58 | -0.104 |
| sp\|Q8IZF7\|AGRF2_HUMAN Adhesion G-protein coupled receptor F2 OS=Homo sapiens GN=ADGRF2 PE=2 SV | 691 | LNAFQVsPDAS | 0.117 | -0.315 | -0.898 | -0.365 |
| sp\|Q8IZF7\|AGRF2_HUMAN Adhesion G-protein coupled receptor F2 OS=Homo sapiens GN=ADGRF2 PE=2 SV | 695 | QVSPDAsDQVQ | 0.144 | -0.112 | -1.061 | -0.343 |
| sp\|Q8IZF7\|AGRF2_HUMAN Adhesion G-protein coupled receptor F2 OS=Homo sapiens GN=ADGRF2 PE=2 SV | 700 | ASDQVQsERIH | 0.186 | -0.26 | -1.05 | -0.375 |
| sp\|Q8IZF6\|AGRG4_HUMAN Adhesion G-protein coupled receptor G4 OS=Homo sapiens GN=ADGRG4 PE=2 SV | 319 | FAVDVLsTSSA | 0.052 | -0.204 | -1.604 | -0.585 |
| sp\|Q8IZF6\|AGRG4_HUMAN Adhesion G-protein coupled receptor G4 OS=Homo sapiens GN=ADGRG4 PE=2 SV | 320 | AVDVLStSSAI | 0.048 | -0.223 | -1.496 | -0.557 |
| sp\|Q8IZF6\|AGRG4_HUMAN Adhesion G-protein coupled receptor G4 OS=Homo sapiens GN=ADGRG4 PE=2 SV | 321 | VDVLSTsSAIS | 0.12 | -0.076 | -1.038 | -0.331 |
| sp\|Q8IZF6\|AGRG4_HUMAN Adhesion G-protein coupled receptor G4 OS=Homo sapiens GN=ADGRG4 PE=2 SV | 322 | DVLSTSsAISL | 0.048 | -0.114 | -1.418 | -0.495 |
| sp\|Q8IZF6\|AGRG4_HUMAN Adhesion G-protein coupled receptor G4 OS=Homo sapiens GN=ADGRG4 PE=2 SV | 325 | STSSAIsLPTQ | 0.681 | 0.386 | 0.014 | 0.36 |
| sp\|Q8IZF6\|AGRG4_HUMAN Adhesion G-protein coupled receptor G4 OS=Homo sapiens GN=ADGRG4 PE=2 SV | 328 | SAISLPtQSIS | 0.033 | -0.442 | -2.183 | -0.864 |
| sp\|Q8IZF6\|AGRG4_HUMAN Adhesion G-protein coupled receptor G4 OS=Homo sapiens GN=ADGRG4 PE=2 SV | 330 | ISLPTQsISID | 0.107 | -0.202 | -0.842 | -0.312 |
| sp\|Q8IZF6\|AGRG4_HUMAN Adhesion G-protein coupled receptor G4 OS=Homo sapiens GN=ADGRG4 PE=2 SV | 332 | LPTQSIsIDNT | 0.338 | 0.134 | -0.158 | 0.105 |
| sp\|Q8IZF6\|AGRG4_HUMAN Adhesion G-protein coupled receptor G4 OS=Homo sapiens GN=ADGRG4 PE=2 SV | 336 | SISIDNtTNSM | 0.13 | -0.075 | -1.125 | -0.357 |
| sp\|Q8IZF6\|AGRG4_HUMAN Adhesion G-protein coupled receptor G4 OS=Homo sapiens GN=ADGRG4 PE=2 SV | 337 | ISIDNTtNSMK | 0.114 | -0.288 | -1.268 | -0.481 |
| sp\|Q8IZF6\|AGRG4_HUMAN Adhesion G-protein coupled receptor G4 OS=Homo sapiens GN=ADGRG4 PE=2 SV | 339 | IDNTTNsMKKT | 0.079 | -0.088 | -1.341 | -0.45 |
| sp\|Q8IZF6\|AGRG4_HUMAN Adhesion G-protein coupled receptor G4 OS=Homo sapiens GN=ADGRG4 PE=2 SV | 343 | TNSMKKtKSPS | 0.058 | -0.374 | -1.173 | -0.496 |
| sp\|Q8IZF6\|AGRG4_HUMAN Adhesion G-protein coupled receptor G4 OS=Homo sapiens GN=ADGRG4 PE=2 SV | 345 | SMKKTKsPSSE | 0.056 | -0.244 | -1.348 | -0.512 |
| sp\|Q8IZF6\|AGRG4_HUMAN Adhesion G-protein coupled receptor G4 OS=Homo sapiens GN=ADGRG4 PE=2 SV | 347 | KKTKSPsSEST | 0.325 | 0.085 | -0.358 | 0.017 |
| sp\|Q8IZF6\|AGRG4_HUMAN Adhesion G-protein coupled receptor G4 OS=Homo sapiens GN=ADGRG4 PE=2 SV | 348 | KTKSPSsESTK | 0.08 | -0.331 | -1.257 | -0.503 |
| sp\|Q8IZF6\|AGRG4_HUMAN Adhesion G-protein coupled receptor G4 OS=Homo sapiens GN=ADGRG4 PE=2 SV | 350 | KSPSSEsTKTT | 0.095 | -0.2 | -1.165 | -0.423 |
| sp\|Q8IZF6\|AGRG4_HUMAN Adhesion G-protein coupled receptor G4 OS=Homo sapiens GN=ADGRG4 PE=2 SV | 351 | SPSSEStKTTK | 0.058 | -0.438 | -1.689 | -0.69 |
| sp\|Q8IZF6\|AGRG4_HUMAN Adhesion G-protein coupled receptor G4 OS=Homo sapiens GN=ADGRG4 PE=2 SV | 353 | SSESTKtTKMV | 0.085 | -0.241 | -1.359 | -0.505 |
| sp\|Q8IZF6\|AGRG4_HUMAN Adhesion G-protein coupled receptor G4 OS=Homo sapiens GN=ADGRG4 PE=2 SV | 354 | SESTKTtKMVE | 0.082 | -0.24 | -1.457 | -0.538 |
| sp\|Q8IZF6\|AGRG4_HUMAN Adhesion G-protein coupled receptor G4 OS=Homo sapiens GN=ADGRG4 PE=2 SV | 362 | MVEAMAtEIFQ | 0.19 | -0.064 | -0.714 | -0.196 |
| sp\|Q8IZF6\|AGRG4_HUMAN Adhesion G-protein coupled receptor G4 OS=Homo sapiens GN=ADGRG4 PE=2 SV | 369 | EIFQPPtPSNF | 0.042 | -0.435 | -1.486 | -0.626 |
| sp\|Q8IZF6\|AGRG4_HUMAN Adhesion G-protein coupled receptor G4 OS=Homo sapiens GN=ADGRG4 PE=2 SV | 371 | FQPPTPsNFLS | 0.059 | -0.162 | -1.324 | -0.476 |
| sp\|Q8IZF6\|AGRG4_HUMAN Adhesion G-protein coupled receptor G4 OS=Homo sapiens GN=ADGRG4 PE=2 SV | 375 | TPSNFLsTSRF | 0.034 | -0.397 | -1.765 | -0.709 |
| sp\|Q8IZF6\|AGRG4_HUMAN Adhesion G-protein coupled receptor G4 OS=Homo sapiens GN=ADGRG4 PE=2 SV | 376 | PSNFLStSRFT | 0.047 | -0.408 | -1.67 | -0.677 |
| sp\|Q8IZF6\|AGRG4_HUMAN Adhesion G-protein coupled receptor G4 OS=Homo sapiens GN=ADGRG4 PE=2 SV | 377 | SNFLSTsRFTK | 0.174 | -0.095 | -0.83 | -0.25 |
| sp\|Q8IZF6\|AGRG4_HUMAN Adhesion G-protein coupled receptor G4 OS=Homo sapiens GN=ADGRG4 PE=2 SV | 380 | LSTSRFtKNSV | 0.21 | 0.016 | -0.628 | -0.134 |
| sp\|Q8IZF6\|AGRG4_HUMAN Adhesion G-protein coupled receptor G4 OS=Homo sapiens GN=ADGRG4 PE=2 SV | 383 | SRFTKNsVVST | 0.098 | 0.038 | -1.332 | -0.399 |
| sp\|Q8IZF6\|AGRG4_HUMAN Adhesion G-protein coupled receptor G4 OS=Homo sapiens GN=ADGRG4 PE=2 SV | 386 | TKNSVVsTTSA | 0.255 | -0.166 | -0.907 | -0.273 |
| sp\|Q8IZF6\|AGRG4_HUMAN Adhesion G-protein coupled receptor G4 OS=Homo sapiens GN=ADGRG4 PE=2 SV | 387 | KNSVVStTSAI | 0.072 | -0.245 | -1.234 | -0.469 |
| sp\|Q8IZF6\|AGRG4_HUMAN Adhesion G-protein coupled receptor G4 OS=Homo sapiens GN=ADGRG4 PE=2 SV | 388 | NSVVSTtSAIK | 0.17 | -0.052 | -0.821 | -0.234 |
| sp\|Q8IZF6\|AGRG4_HUMAN Adhesion G-protein coupled receptor G4 OS=Homo sapiens GN=ADGRG4 PE=2 SV | 389 | SVVSTTsAIKS | 0.041 | -0.14 | -1.585 | -0.561 |
| sp\|Q8IZF6\|AGRG4_HUMAN Adhesion G-protein coupled receptor G4 OS=Homo sapiens GN=ADGRG4 PE=2 SV | 393 | TTSAIKsQSAV | 0.182 | -0.135 | -0.968 | -0.307 |
| sp\|Q8IZF6\|AGRG4_HUMAN Adhesion G-protein coupled receptor G4 OS=Homo sapiens GN=ADGRG4 PE=2 SV | 395 | SAIKSQsAVTK | 0.32 | 0.136 | -0.602 | -0.049 |
| sp\|Q8IZF6\|AGRG4_HUMAN Adhesion G-protein coupled receptor G4 OS=Homo sapiens GN=ADGRG4 PE=2 SV | 398 | KSQSAVtKTTS | 0.325 | -0.124 | -0.702 | -0.167 |
| sp\|Q8IZF6\|AGRG4_HUMAN Adhesion G-protein coupled receptor G4 OS=Homo sapiens GN=ADGRG4 PE=2 SV | 400 | QSAVTKtTSLF | 0.059 | -0.142 | -1.053 | -0.379 |
| sp\|Q8IZF6\|AGRG4_HUMAN Adhesion G-protein coupled receptor G4 OS=Homo sapiens GN=ADGRG4 PE=2 SV | 401 | SAVTKTtSLFS | 0.031 | -0.395 | -2.063 | -0.809 |
| sp\|Q8IZF6\|AGRG4_HUMAN Adhesion G-protein coupled receptor G4 OS=Homo sapiens GN=ADGRG4 PE=2 SV | 402 | AVTKTTsLFST | 0.21 | 0.041 | -0.414 | -0.054 |
| sp\|Q8IZF6\|AGRG4_HUMAN Adhesion G-protein coupled receptor G4 OS=Homo sapiens GN=ADGRG4 PE=2 SV | 405 | KTTSLFsTIES | 0.097 | -0.175 | -1.184 | -0.421 |
| sp\|Q8IZF6\|AGRG4_HUMAN Adhesion G-protein coupled receptor G4 OS=Homo sapiens GN=ADGRG4 PE=2 SV | 406 | TTSLFStIEST | 0.037 | -0.367 | -1.743 | -0.691 |
| sp\|Q8IZF6\|AGRG4_HUMAN Adhesion G-protein coupled receptor G4 OS=Homo sapiens GN=ADGRG4 PE=2 SV | 409 | LFSTIEsTSMS | 0.065 | -0.291 | -1.339 | -0.522 |
| sp\|Q8IZF6\|AGRG4_HUMAN Adhesion G-protein coupled receptor G4 OS=Homo sapiens GN=ADGRG4 PE=2 SV | 410 | FSTIEStSMST | 0.048 | -0.242 | -1.533 | -0.576 |

| | | | | | | |
|---|---|---|---|---|---|---|
| sp\|Q8IZF6\|AGRG4_HUMAN Adhesion G-protein coupled receptor G4 OS=Homo sapiens GN=ADGRG4 PE=2 S\' | 411 | STIESTsMSTT | 0.098 | 0.066 | -1.163 | -0.333 |
| sp\|Q8IZF6\|AGRG4_HUMAN Adhesion G-protein coupled receptor G4 OS=Homo sapiens GN=ADGRG4 PE=2 S\' | 413 | IESTSMsTTPC | 0.368 | 0.071 | -0.633 | -0.065 |
| sp\|Q8IZF6\|AGRG4_HUMAN Adhesion G-protein coupled receptor G4 OS=Homo sapiens GN=ADGRG4 PE=2 S\' | 414 | ESTSMStTPCL | 0.28 | 0.279 | -0.374 | 0.062 |
| sp\|Q8IZF6\|AGRG4_HUMAN Adhesion G-protein coupled receptor G4 OS=Homo sapiens GN=ADGRG4 PE=2 S\' | 415 | STSMSTtPCLK | 0.098 | -0.392 | -1.252 | -0.515 |
| sp\|Q8IZF6\|AGRG4_HUMAN Adhesion G-protein coupled receptor G4 OS=Homo sapiens GN=ADGRG4 PE=2 S\' | 422 | PCLKQKsTNTG | 0.511 | 0.134 | -0.194 | 0.15 |
| sp\|Q8IZF6\|AGRG4_HUMAN Adhesion G-protein coupled receptor G4 OS=Homo sapiens GN=ADGRG4 PE=2 S\' | 423 | CLKQKStNTGA | 0.295 | -0.105 | -0.486 | -0.099 |
| sp\|Q8IZF6\|AGRG4_HUMAN Adhesion G-protein coupled receptor G4 OS=Homo sapiens GN=ADGRG4 PE=2 S\' | 425 | KQKSTNtGALP | 0.072 | -0.187 | -1.172 | -0.429 |
| sp\|Q8IZF6\|AGRG4_HUMAN Adhesion G-protein coupled receptor G4 OS=Homo sapiens GN=ADGRG4 PE=2 S\' | 431 | TGALPIsTAGQ | 0.091 | -0.344 | -1.06 | -0.438 |
| sp\|Q8IZF6\|AGRG4_HUMAN Adhesion G-protein coupled receptor G4 OS=Homo sapiens GN=ADGRG4 PE=2 S\' | 432 | GALPIStAGQE | 0.184 | -0.141 | -0.809 | -0.255 |
| sp\|Q8IZF6\|AGRG4_HUMAN Adhesion G-protein coupled receptor G4 OS=Homo sapiens GN=ADGRG4 PE=2 S\' | 440 | GQEFIEsTAAG | 0.215 | -0.047 | -0.631 | -0.154 |
| sp\|Q8IZF6\|AGRG4_HUMAN Adhesion G-protein coupled receptor G4 OS=Homo sapiens GN=ADGRG4 PE=2 S\' | 441 | QEFIEStAAGT | 0.058 | -0.274 | -1.61 | -0.609 |
| sp\|Q8IZF6\|AGRG4_HUMAN Adhesion G-protein coupled receptor G4 OS=Homo sapiens GN=ADGRG4 PE=2 S\' | 445 | ESTAAGtVPWF | 0.404 | 0.366 | -0.044 | 0.242 |
| sp\|Q8IZF6\|AGRG4_HUMAN Adhesion G-protein coupled receptor G4 OS=Homo sapiens GN=ADGRG4 PE=2 S\' | 450 | GTVPWFtVEKT | 0.158 | -0.059 | -0.902 | -0.268 |
| sp\|Q8IZF6\|AGRG4_HUMAN Adhesion G-protein coupled receptor G4 OS=Homo sapiens GN=ADGRG4 PE=2 S\' | 454 | WFTVEKtSPAS | 0.531 | 0.467 | 0.216 | 0.405 |
| sp\|Q8IZF6\|AGRG4_HUMAN Adhesion G-protein coupled receptor G4 OS=Homo sapiens GN=ADGRG4 PE=2 S\' | 455 | FTVEKTsPAST | 0.021 | -0.505 | -1.956 | -0.813 |
| sp\|Q8IZF6\|AGRG4_HUMAN Adhesion G-protein coupled receptor G4 OS=Homo sapiens GN=ADGRG4 PE=2 S\' | 458 | EKTSPAsTHVG | 0.291 | -0.034 | -0.577 | -0.107 |
| sp\|Q8IZF6\|AGRG4_HUMAN Adhesion G-protein coupled receptor G4 OS=Homo sapiens GN=ADGRG4 PE=2 S\' | 459 | KTSPAStHVGT | 0.126 | -0.259 | -1.062 | -0.398 |
| sp\|Q8IZF6\|AGRG4_HUMAN Adhesion G-protein coupled receptor G4 OS=Homo sapiens GN=ADGRG4 PE=2 S\' | 463 | ASTHVGtASSF | 0.053 | -0.335 | -1.707 | -0.663 |
| sp\|Q8IZF6\|AGRG4_HUMAN Adhesion G-protein coupled receptor G4 OS=Homo sapiens GN=ADGRG4 PE=2 S\' | 465 | THVGTAsSFPP | 0.06 | -0.172 | -1.531 | -0.548 |
| sp\|Q8IZF6\|AGRG4_HUMAN Adhesion G-protein coupled receptor G4 OS=Homo sapiens GN=ADGRG4 PE=2 S\' | 466 | HVGTASsFPPE | 0.416 | 0.469 | -0.055 | 0.277 |
| sp\|Q8IZF6\|AGRG4_HUMAN Adhesion G-protein coupled receptor G4 OS=Homo sapiens GN=ADGRG4 PE=2 S\' | 475 | PEPVLIsTAAP | 0.054 | -0.246 | -1.532 | -0.575 |
| sp\|Q8IZF6\|AGRG4_HUMAN Adhesion G-protein coupled receptor G4 OS=Homo sapiens GN=ADGRG4 PE=2 S\' | 476 | EPVLIStAAPV | 0.047 | -0.238 | -1.649 | -0.613 |
| sp\|Q8IZF6\|AGRG4_HUMAN Adhesion G-protein coupled receptor G4 OS=Homo sapiens GN=ADGRG4 PE=2 S\' | 482 | TAAPVDsVFPR | 0.121 | -0.281 | -1.053 | -0.404 |
| sp\|Q8IZF6\|AGRG4_HUMAN Adhesion G-protein coupled receptor G4 OS=Homo sapiens GN=ADGRG4 PE=2 S\' | 489 | VFPRNQtAFPL | 0.585 | 0.823 | 0.379 | 0.596 |
| sp\|Q8IZF6\|AGRG4_HUMAN Adhesion G-protein coupled receptor G4 OS=Homo sapiens GN=ADGRG4 PE=2 S\' | 495 | TAFPLAtTDMK | 0.119 | -0.255 | -1.241 | -0.459 |
| sp\|Q8IZF6\|AGRG4_HUMAN Adhesion G-protein coupled receptor G4 OS=Homo sapiens GN=ADGRG4 PE=2 S\' | 496 | AFPLATtDMKI | 0.314 | 0.041 | -0.637 | -0.094 |
| sp\|Q8IZF6\|AGRG4_HUMAN Adhesion G-protein coupled receptor G4 OS=Homo sapiens GN=ADGRG4 PE=2 S\' | 503 | DMKIAFtVHSL | 0.275 | 0.259 | -0.532 | 0.001 |
| sp\|Q8IZF6\|AGRG4_HUMAN Adhesion G-protein coupled receptor G4 OS=Homo sapiens GN=ADGRG4 PE=2 S\' | 506 | IAFTVHsLTLP | 0.447 | 0.066 | -0.469 | 0.015 |
| sp\|Q8IZF6\|AGRG4_HUMAN Adhesion G-protein coupled receptor G4 OS=Homo sapiens GN=ADGRG4 PE=2 S\' | 508 | FTVHSLtLPTR | 0.804 | 0.754 | 0.71 | 0.756 |
| sp\|Q8IZF6\|AGRG4_HUMAN Adhesion G-protein coupled receptor G4 OS=Homo sapiens GN=ADGRG4 PE=2 S\' | 511 | HSLTLPtRLIE | 0.082 | -0.331 | -1.48 | -0.576 |
| sp\|Q8IZF6\|AGRG4_HUMAN Adhesion G-protein coupled receptor G4 OS=Homo sapiens GN=ADGRG4 PE=2 S\' | 516 | PTRLIEtTPAP | 0.72 | 0.653 | 0.239 | 0.537 |
| sp\|Q8IZF6\|AGRG4_HUMAN Adhesion G-protein coupled receptor G4 OS=Homo sapiens GN=ADGRG4 PE=2 S\' | 517 | TRLIETtPAPR | 0.06 | -0.269 | -1.343 | -0.517 |
| sp\|Q8IZF6\|AGRG4_HUMAN Adhesion G-protein coupled receptor G4 OS=Homo sapiens GN=ADGRG4 PE=2 S\' | 522 | TTPAPRtAETE | 0.061 | -0.32 | -1.629 | -0.629 |
| sp\|Q8IZF6\|AGRG4_HUMAN Adhesion G-protein coupled receptor G4 OS=Homo sapiens GN=ADGRG4 PE=2 S\' | 525 | APRTAEtELTS | 0.299 | 0.134 | -0.885 | -0.151 |
| sp\|Q8IZF6\|AGRG4_HUMAN Adhesion G-protein coupled receptor G4 OS=Homo sapiens GN=ADGRG4 PE=2 S\' | 528 | TAETELtSTNF | 0.074 | -0.28 | -1.642 | -0.616 |
| sp\|Q8IZF6\|AGRG4_HUMAN Adhesion G-protein coupled receptor G4 OS=Homo sapiens GN=ADGRG4 PE=2 S\' | 529 | AETELTsTNFQ | 0.064 | -0.274 | -1.533 | -0.581 |
| sp\|Q8IZF6\|AGRG4_HUMAN Adhesion G-protein coupled receptor G4 OS=Homo sapiens GN=ADGRG4 PE=2 S\' | 530 | ETELTStNFQD | 0.108 | -0.158 | -1.108 | -0.386 |
| sp\|Q8IZF6\|AGRG4_HUMAN Adhesion G-protein coupled receptor G4 OS=Homo sapiens GN=ADGRG4 PE=2 S\' | 536 | TNFQDVsLPRV | 0.766 | 0.364 | 0.151 | 0.427 |
| sp\|Q8IZF6\|AGRG4_HUMAN Adhesion G-protein coupled receptor G4 OS=Homo sapiens GN=ADGRG4 PE=2 S\' | 545 | RVEDAMsTSMS | 0.205 | 0.043 | -0.924 | -0.225 |
| sp\|Q8IZF6\|AGRG4_HUMAN Adhesion G-protein coupled receptor G4 OS=Homo sapiens GN=ADGRG4 PE=2 S\' | 546 | VEDAMStSMSK | 0.045 | -0.425 | -1.69 | -0.69 |
| sp\|Q8IZF6\|AGRG4_HUMAN Adhesion G-protein coupled receptor G4 OS=Homo sapiens GN=ADGRG4 PE=2 S\' | 547 | EDAMSTsMSKE | 0.107 | 0.008 | -0.857 | -0.247 |
| sp\|Q8IZF6\|AGRG4_HUMAN Adhesion G-protein coupled receptor G4 OS=Homo sapiens GN=ADGRG4 PE=2 S\' | 549 | AMSTSMsKETS | 0.276 | 0.264 | -0.495 | 0.015 |
| sp\|Q8IZF6\|AGRG4_HUMAN Adhesion G-protein coupled receptor G4 OS=Homo sapiens GN=ADGRG4 PE=2 S\' | 552 | TSMSKEtSSKT | 0.028 | -0.437 | -1.934 | -0.781 |
| sp\|Q8IZF6\|AGRG4_HUMAN Adhesion G-protein coupled receptor G4 OS=Homo sapiens GN=ADGRG4 PE=2 S\' | 553 | SMSKETsSKTF | 0.087 | -0.119 | -1.26 | -0.431 |
| sp\|Q8IZF6\|AGRG4_HUMAN Adhesion G-protein coupled receptor G4 OS=Homo sapiens GN=ADGRG4 PE=2 S\' | 554 | MSKETSsKTFS | 0.077 | -0.163 | -1.246 | -0.444 |
| sp\|Q8IZF6\|AGRG4_HUMAN Adhesion G-protein coupled receptor G4 OS=Homo sapiens GN=ADGRG4 PE=2 S\' | 556 | KETSSKtFSFL | 0.287 | 0.118 | -0.37 | 0.012 |
| sp\|Q8IZF6\|AGRG4_HUMAN Adhesion G-protein coupled receptor G4 OS=Homo sapiens GN=ADGRG4 PE=2 S\' | 558 | TSSKTFsFLTS | 0.163 | -0.036 | -0.78 | -0.218 |
| sp\|Q8IZF6\|AGRG4_HUMAN Adhesion G-protein coupled receptor G4 OS=Homo sapiens GN=ADGRG4 PE=2 S\' | 561 | KTFSFLtSFSF | 0.046 | -0.303 | -1.681 | -0.646 |
| sp\|Q8IZF6\|AGRG4_HUMAN Adhesion G-protein coupled receptor G4 OS=Homo sapiens GN=ADGRG4 PE=2 S\' | 562 | TFSFLTsFSFT | 0.096 | -0.206 | -1.058 | -0.389 |

| | | | | | | |
|---|---|---|---|---|---|---|
| sp\|Q8IZF6\|AGRG4_HUMAN Adhesion G-protein coupled receptor G4 OS=Homo sapiens GN=ADGRG4 PE=2 S' | 564 | SFLTSFsFTGT | 0.412 | 0.138 | -0.091 | 0.153 |
| sp\|Q8IZF6\|AGRG4_HUMAN Adhesion G-protein coupled receptor G4 OS=Homo sapiens GN=ADGRG4 PE=2 S' | 566 | LTSFSFtGTES | 0.384 | 0.119 | -0.373 | 0.043 |
| sp\|Q8IZF6\|AGRG4_HUMAN Adhesion G-protein coupled receptor G4 OS=Homo sapiens GN=ADGRG4 PE=2 S' | 568 | SFSFTGtESVQ | 0.061 | -0.132 | -1.436 | -0.502 |
| sp\|Q8IZF6\|AGRG4_HUMAN Adhesion G-protein coupled receptor G4 OS=Homo sapiens GN=ADGRG4 PE=2 S' | 570 | SFTGTEsVQTV | 0.101 | -0.079 | -1.161 | -0.38 |
| sp\|Q8IZF6\|AGRG4_HUMAN Adhesion G-protein coupled receptor G4 OS=Homo sapiens GN=ADGRG4 PE=2 S' | 573 | GTESVQtVIDA | 0.327 | -0.03 | -0.509 | -0.071 |
| sp\|Q8IZF6\|AGRG4_HUMAN Adhesion G-protein coupled receptor G4 OS=Homo sapiens GN=ADGRG4 PE=2 S' | 580 | VIDAEAtRTAL | 0.273 | -0.054 | -0.865 | -0.215 |
| sp\|Q8IZF6\|AGRG4_HUMAN Adhesion G-protein coupled receptor G4 OS=Homo sapiens GN=ADGRG4 PE=2 S' | 582 | DAEATRtALTP | 0.063 | -0.213 | -1.666 | -0.605 |
| sp\|Q8IZF6\|AGRG4_HUMAN Adhesion G-protein coupled receptor G4 OS=Homo sapiens GN=ADGRG4 PE=2 S' | 585 | ATRTALtPEIT | 0.175 | -0.194 | -1.111 | -0.377 |
| sp\|Q8IZF6\|AGRG4_HUMAN Adhesion G-protein coupled receptor G4 OS=Homo sapiens GN=ADGRG4 PE=2 S' | 589 | ALTPEItLAST | 0.202 | -0.122 | -0.726 | -0.215 |
| sp\|Q8IZF6\|AGRG4_HUMAN Adhesion G-protein coupled receptor G4 OS=Homo sapiens GN=ADGRG4 PE=2 S' | 592 | PEITLAsTVAE | 0.17 | -0.034 | -1.109 | -0.324 |
| sp\|Q8IZF6\|AGRG4_HUMAN Adhesion G-protein coupled receptor G4 OS=Homo sapiens GN=ADGRG4 PE=2 S' | 593 | EITLAStVAET | 0.147 | -0.03 | -0.823 | -0.235 |
| sp\|Q8IZF6\|AGRG4_HUMAN Adhesion G-protein coupled receptor G4 OS=Homo sapiens GN=ADGRG4 PE=2 S' | 597 | ASTVAEtMLSS | 0.053 | -0.128 | -1.609 | -0.561 |
| sp\|Q8IZF6\|AGRG4_HUMAN Adhesion G-protein coupled receptor G4 OS=Homo sapiens GN=ADGRG4 PE=2 S' | 600 | VAETMLsSTIT | 0.102 | -0.373 | -1.313 | -0.528 |
| sp\|Q8IZF6\|AGRG4_HUMAN Adhesion G-protein coupled receptor G4 OS=Homo sapiens GN=ADGRG4 PE=2 S' | 601 | AETMLSsTITG | 0.068 | -0.294 | -1.374 | -0.533 |
| sp\|Q8IZF6\|AGRG4_HUMAN Adhesion G-protein coupled receptor G4 OS=Homo sapiens GN=ADGRG4 PE=2 S' | 602 | ETMLSStITGR | 0.199 | 0.023 | -0.604 | -0.127 |
| sp\|Q8IZF6\|AGRG4_HUMAN Adhesion G-protein coupled receptor G4 OS=Homo sapiens GN=ADGRG4 PE=2 S' | 604 | MLSSTItGRVY | 0.304 | 0.039 | -0.575 | -0.077 |
| sp\|Q8IZF6\|AGRG4_HUMAN Adhesion G-protein coupled receptor G4 OS=Homo sapiens GN=ADGRG4 PE=2 S' | 609 | ITGRVYtQNTP | 0.535 | 0.75 | 0.089 | 0.458 |
| sp\|Q8IZF6\|AGRG4_HUMAN Adhesion G-protein coupled receptor G4 OS=Homo sapiens GN=ADGRG4 PE=2 S' | 612 | RVYTQNtPTAD | 0.152 | -0.188 | -1.092 | -0.376 |
| sp\|Q8IZF6\|AGRG4_HUMAN Adhesion G-protein coupled receptor G4 OS=Homo sapiens GN=ADGRG4 PE=2 S' | 614 | YTQNTPtADGH | 0.113 | -0.118 | -1.039 | -0.348 |
| sp\|Q8IZF6\|AGRG4_HUMAN Adhesion G-protein coupled receptor G4 OS=Homo sapiens GN=ADGRG4 PE=2 S' | 621 | ADGHLLtLMST | 0.036 | -0.304 | -1.509 | -0.592 |
| sp\|Q8IZF6\|AGRG4_HUMAN Adhesion G-protein coupled receptor G4 OS=Homo sapiens GN=ADGRG4 PE=2 S' | 624 | HLLTLMsTRSA | 0.192 | 0.037 | -0.652 | -0.141 |
| sp\|Q8IZF6\|AGRG4_HUMAN Adhesion G-protein coupled receptor G4 OS=Homo sapiens GN=ADGRG4 PE=2 S' | 625 | LLTLMStRSAS | 0.108 | -0.171 | -1.009 | -0.357 |
| sp\|Q8IZF6\|AGRG4_HUMAN Adhesion G-protein coupled receptor G4 OS=Homo sapiens GN=ADGRG4 PE=2 S' | 627 | TLMSTRsASTS | 0.063 | -0.053 | -1.442 | -0.477 |
| sp\|Q8IZF6\|AGRG4_HUMAN Adhesion G-protein coupled receptor G4 OS=Homo sapiens GN=ADGRG4 PE=2 S' | 629 | MSTRSAsTSKA | 0.748 | 0.999 | 0.655 | 0.801 |
| sp\|Q8IZF6\|AGRG4_HUMAN Adhesion G-protein coupled receptor G4 OS=Homo sapiens GN=ADGRG4 PE=2 S' | 630 | STRSAStSKAP | 0.168 | 0.021 | -0.941 | -0.251 |
| sp\|Q8IZF6\|AGRG4_HUMAN Adhesion G-protein coupled receptor G4 OS=Homo sapiens GN=ADGRG4 PE=2 S' | 631 | TRSASTsKAPE | 0.261 | 0.156 | -0.398 | 0.006 |
| sp\|Q8IZF6\|AGRG4_HUMAN Adhesion G-protein coupled receptor G4 OS=Homo sapiens GN=ADGRG4 PE=2 S' | 636 | TSKAPEsGPTS | 0.311 | 0.134 | -0.428 | 0.006 |
| sp\|Q8IZF6\|AGRG4_HUMAN Adhesion G-protein coupled receptor G4 OS=Homo sapiens GN=ADGRG4 PE=2 S' | 639 | APESGPtSTTD | 0.094 | -0.377 | -1.366 | -0.55 |
| sp\|Q8IZF6\|AGRG4_HUMAN Adhesion G-protein coupled receptor G4 OS=Homo sapiens GN=ADGRG4 PE=2 S' | 640 | PESGPTsTTDE | 0.202 | -0.165 | -0.832 | -0.265 |
| sp\|Q8IZF6\|AGRG4_HUMAN Adhesion G-protein coupled receptor G4 OS=Homo sapiens GN=ADGRG4 PE=2 S' | 641 | ESGPTStTDEA | 0.055 | -0.213 | -1.201 | -0.453 |
| sp\|Q8IZF6\|AGRG4_HUMAN Adhesion G-protein coupled receptor G4 OS=Homo sapiens GN=ADGRG4 PE=2 S' | 642 | SGPTSTtDEAA | 0.25 | 0.097 | -0.577 | -0.077 |
| sp\|Q8IZF6\|AGRG4_HUMAN Adhesion G-protein coupled receptor G4 OS=Homo sapiens GN=ADGRG4 PE=2 S' | 650 | EAAHLFsSNET | 0.095 | -0.169 | -0.917 | -0.33 |
| sp\|Q8IZF6\|AGRG4_HUMAN Adhesion G-protein coupled receptor G4 OS=Homo sapiens GN=ADGRG4 PE=2 S' | 651 | AAHLFSsNETI | 0.071 | -0.276 | -1.612 | -0.606 |
| sp\|Q8IZF6\|AGRG4_HUMAN Adhesion G-protein coupled receptor G4 OS=Homo sapiens GN=ADGRG4 PE=2 S' | 654 | LFSSNEtIWTS | 0.143 | -0.206 | -0.955 | -0.339 |
| sp\|Q8IZF6\|AGRG4_HUMAN Adhesion G-protein coupled receptor G4 OS=Homo sapiens GN=ADGRG4 PE=2 S' | 657 | SNETIWtSRPD | 0.101 | -0.388 | -1.106 | -0.464 |
| sp\|Q8IZF6\|AGRG4_HUMAN Adhesion G-protein coupled receptor G4 OS=Homo sapiens GN=ADGRG4 PE=2 S' | 658 | NETIWTsRPDQ | 0.584 | 0.436 | -0.063 | 0.319 |
| sp\|Q8IZF6\|AGRG4_HUMAN Adhesion G-protein coupled receptor G4 OS=Homo sapiens GN=ADGRG4 PE=2 S' | 667 | DQALLAsMNTT | 0.173 | -0.022 | -0.88 | -0.243 |
| sp\|Q8IZF6\|AGRG4_HUMAN Adhesion G-protein coupled receptor G4 OS=Homo sapiens GN=ADGRG4 PE=2 S' | 670 | LLASMNtTTIL | 0.408 | -0.006 | -0.439 | -0.012 |
| sp\|Q8IZF6\|AGRG4_HUMAN Adhesion G-protein coupled receptor G4 OS=Homo sapiens GN=ADGRG4 PE=2 S' | 671 | LASMNTtTILT | 0.109 | -0.223 | -0.82 | -0.311 |
| sp\|Q8IZF6\|AGRG4_HUMAN Adhesion G-protein coupled receptor G4 OS=Homo sapiens GN=ADGRG4 PE=2 S' | 672 | ASMNTTtILTF | 0.04 | -0.197 | -1.219 | -0.459 |
| sp\|Q8IZF6\|AGRG4_HUMAN Adhesion G-protein coupled receptor G4 OS=Homo sapiens GN=ADGRG4 PE=2 S' | 675 | NTTTILtFVPN | 0.157 | -0.076 | -0.788 | -0.236 |
| sp\|Q8IZF6\|AGRG4_HUMAN Adhesion G-protein coupled receptor G4 OS=Homo sapiens GN=ADGRG4 PE=2 S' | 683 | VPNENFtSAFH | 0.031 | -0.405 | -1.872 | -0.749 |
| sp\|Q8IZF6\|AGRG4_HUMAN Adhesion G-protein coupled receptor G4 OS=Homo sapiens GN=ADGRG4 PE=2 S' | 684 | PNENFTsAFHE | 0.125 | -0.189 | -0.859 | -0.308 |
| sp\|Q8IZF6\|AGRG4_HUMAN Adhesion G-protein coupled receptor G4 OS=Homo sapiens GN=ADGRG4 PE=2 S' | 690 | SAFHENtTYTE | 0.124 | -0.217 | -1.441 | -0.511 |
| sp\|Q8IZF6\|AGRG4_HUMAN Adhesion G-protein coupled receptor G4 OS=Homo sapiens GN=ADGRG4 PE=2 S' | 691 | AFHENTtYTEY | 0.18 | -0.116 | -0.963 | -0.3 |
| sp\|Q8IZF6\|AGRG4_HUMAN Adhesion G-protein coupled receptor G4 OS=Homo sapiens GN=ADGRG4 PE=2 S' | 693 | HENTTYtEYLS | 0.109 | 0.146 | -0.944 | -0.23 |
| sp\|Q8IZF6\|AGRG4_HUMAN Adhesion G-protein coupled receptor G4 OS=Homo sapiens GN=ADGRG4 PE=2 S' | 697 | TYTEYLsATTN | 0.149 | -0.049 | -1.107 | -0.336 |
| sp\|Q8IZF6\|AGRG4_HUMAN Adhesion G-protein coupled receptor G4 OS=Homo sapiens GN=ADGRG4 PE=2 S' | 699 | TEYLSAtTNIT | 0.318 | 0.116 | -0.5 | -0.022 |
| sp\|Q8IZF6\|AGRG4_HUMAN Adhesion G-protein coupled receptor G4 OS=Homo sapiens GN=ADGRG4 PE=2 S' | 700 | EYLSATtNITP | 0.12 | -0.123 | -0.921 | -0.308 |

| | | | | | | |
|---|---|---|---|---|---|---|
| sp\|Q8IZF6\|AGRG4_HUMAN Adhesion G-protein coupled receptor G4 OS=Homo sapiens GN=ADGRG4 PE=2 S' | 703 | SATTNItPLKA | 0.047 | -0.537 | -1.646 | -0.712 |
| sp\|Q8IZF6\|AGRG4_HUMAN Adhesion G-protein coupled receptor G4 OS=Homo sapiens GN=ADGRG4 PE=2 S' | 708 | ITPLKAsPEGK | 0.067 | -0.539 | -1.551 | -0.674 |
| sp\|Q8IZF6\|AGRG4_HUMAN Adhesion G-protein coupled receptor G4 OS=Homo sapiens GN=ADGRG4 PE=2 S' | 714 | SPEGKGtTAND | 0.056 | -0.301 | -1.692 | -0.646 |
| sp\|Q8IZF6\|AGRG4_HUMAN Adhesion G-protein coupled receptor G4 OS=Homo sapiens GN=ADGRG4 PE=2 S' | 715 | PEGKGTtANDA | 0.314 | 0.038 | -0.544 | -0.064 |
| sp\|Q8IZF6\|AGRG4_HUMAN Adhesion G-protein coupled receptor G4 OS=Homo sapiens GN=ADGRG4 PE=2 S' | 720 | TTANDAtTARY | 0.159 | -0.216 | -1.103 | -0.387 |
| sp\|Q8IZF6\|AGRG4_HUMAN Adhesion G-protein coupled receptor G4 OS=Homo sapiens GN=ADGRG4 PE=2 S' | 721 | TANDATtARYT | 0.172 | -0.153 | -1.173 | -0.385 |
| sp\|Q8IZF6\|AGRG4_HUMAN Adhesion G-protein coupled receptor G4 OS=Homo sapiens GN=ADGRG4 PE=2 S' | 725 | ATTARYtTAVS | 0.243 | 0.04 | -0.721 | -0.146 |
| sp\|Q8IZF6\|AGRG4_HUMAN Adhesion G-protein coupled receptor G4 OS=Homo sapiens GN=ADGRG4 PE=2 S' | 726 | TTARYTtAVSK | 0.475 | 0.747 | -0.001 | 0.407 |
| sp\|Q8IZF6\|AGRG4_HUMAN Adhesion G-protein coupled receptor G4 OS=Homo sapiens GN=ADGRG4 PE=2 S' | 729 | RYTTAVsKLTS | 0.398 | 0.035 | -0.507 | -0.025 |
| sp\|Q8IZF6\|AGRG4_HUMAN Adhesion G-protein coupled receptor G4 OS=Homo sapiens GN=ADGRG4 PE=2 S' | 732 | TAVSKLtSPWF | 0.122 | 0.174 | -0.841 | -0.182 |
| sp\|Q8IZF6\|AGRG4_HUMAN Adhesion G-protein coupled receptor G4 OS=Homo sapiens GN=ADGRG4 PE=2 S' | 733 | AVSKLTsPWFA | 0.094 | -0.429 | -1.362 | -0.566 |
| sp\|Q8IZF6\|AGRG4_HUMAN Adhesion G-protein coupled receptor G4 OS=Homo sapiens GN=ADGRG4 PE=2 S' | 740 | PWFANFsIVSG | 0.138 | -0.036 | -0.767 | -0.222 |
| sp\|Q8IZF6\|AGRG4_HUMAN Adhesion G-protein coupled receptor G4 OS=Homo sapiens GN=ADGRG4 PE=2 S' | 743 | ANFSIVsGTTS | 0.151 | -0.314 | -0.995 | -0.386 |
| sp\|Q8IZF6\|AGRG4_HUMAN Adhesion G-protein coupled receptor G4 OS=Homo sapiens GN=ADGRG4 PE=2 S' | 745 | FSIVSGtTSIT | 0.112 | -0.019 | -1.071 | -0.326 |
| sp\|Q8IZF6\|AGRG4_HUMAN Adhesion G-protein coupled receptor G4 OS=Homo sapiens GN=ADGRG4 PE=2 S' | 746 | SIVSGTtSITN | 0.051 | -0.197 | -1.427 | -0.524 |
| sp\|Q8IZF6\|AGRG4_HUMAN Adhesion G-protein coupled receptor G4 OS=Homo sapiens GN=ADGRG4 PE=2 S' | 747 | IVSGTTsITNM | 0.123 | -0.073 | -0.872 | -0.274 |
| sp\|Q8IZF6\|AGRG4_HUMAN Adhesion G-protein coupled receptor G4 OS=Homo sapiens GN=ADGRG4 PE=2 S' | 749 | SGTTSItNMPE | 0.213 | -0.045 | -0.818 | -0.217 |
| sp\|Q8IZF6\|AGRG4_HUMAN Adhesion G-protein coupled receptor G4 OS=Homo sapiens GN=ADGRG4 PE=2 S' | 757 | MPEFKLtTLLL | 0.089 | -0.07 | -1.174 | -0.385 |
| sp\|Q8IZF6\|AGRG4_HUMAN Adhesion G-protein coupled receptor G4 OS=Homo sapiens GN=ADGRG4 PE=2 S' | 758 | PEFKLTtLLLK | 0.331 | 0.028 | -0.587 | -0.076 |
| sp\|Q8IZF6\|AGRG4_HUMAN Adhesion G-protein coupled receptor G4 OS=Homo sapiens GN=ADGRG4 PE=2 S' | 763 | TTLLLKtIPMS | 0.378 | 0.29 | -0.365 | 0.101 |
| sp\|Q8IZF6\|AGRG4_HUMAN Adhesion G-protein coupled receptor G4 OS=Homo sapiens GN=ADGRG4 PE=2 S' | 767 | LKTIPMsTKPA | 0.237 | -0.013 | -0.366 | -0.047 |
| sp\|Q8IZF6\|AGRG4_HUMAN Adhesion G-protein coupled receptor G4 OS=Homo sapiens GN=ADGRG4 PE=2 S' | 768 | KTIPMStKPAN | 0.445 | 0.333 | -0.211 | 0.189 |
| sp\|Q8IZF6\|AGRG4_HUMAN Adhesion G-protein coupled receptor G4 OS=Homo sapiens GN=ADGRG4 PE=2 S' | 777 | ANELPLtPRET | 0.034 | -0.688 | -1.634 | -0.763 |
| sp\|Q8IZF6\|AGRG4_HUMAN Adhesion G-protein coupled receptor G4 OS=Homo sapiens GN=ADGRG4 PE=2 S' | 781 | PLTPREtVVPS | 0.291 | 0.076 | -0.571 | -0.068 |
| sp\|Q8IZF6\|AGRG4_HUMAN Adhesion G-protein coupled receptor G4 OS=Homo sapiens GN=ADGRG4 PE=2 S' | 785 | RETVVPsVDII | 0.306 | 0.005 | -0.618 | -0.102 |
| sp\|Q8IZF6\|AGRG4_HUMAN Adhesion G-protein coupled receptor G4 OS=Homo sapiens GN=ADGRG4 PE=2 S' | 790 | PSVDIIsTLAC | 0.133 | -0.123 | -1.052 | -0.347 |
| sp\|Q8IZF6\|AGRG4_HUMAN Adhesion G-protein coupled receptor G4 OS=Homo sapiens GN=ADGRG4 PE=2 S' | 791 | SVDIIStLACI | 0.11 | -0.033 | -0.84 | -0.254 |
| sp\|Q8IZF6\|AGRG4_HUMAN Adhesion G-protein coupled receptor G4 OS=Homo sapiens GN=ADGRG4 PE=2 S' | 800 | CIQPNFsTEES | 0.27 | 0.089 | -0.49 | -0.044 |
| sp\|Q8IZF6\|AGRG4_HUMAN Adhesion G-protein coupled receptor G4 OS=Homo sapiens GN=ADGRG4 PE=2 S' | 801 | IQPNFStEESA | 0.043 | -0.223 | -1.212 | -0.464 |
| sp\|Q8IZF6\|AGRG4_HUMAN Adhesion G-protein coupled receptor G4 OS=Homo sapiens GN=ADGRG4 PE=2 S' | 804 | NFSTEEsASET | 0.119 | -0.205 | -1.376 | -0.487 |
| sp\|Q8IZF6\|AGRG4_HUMAN Adhesion G-protein coupled receptor G4 OS=Homo sapiens GN=ADGRG4 PE=2 S' | 806 | STEESAsETTQ | 0.31 | 0.086 | -0.749 | -0.118 |
| sp\|Q8IZF6\|AGRG4_HUMAN Adhesion G-protein coupled receptor G4 OS=Homo sapiens GN=ADGRG4 PE=2 S' | 808 | EESASEtTQTE | 0.208 | -0.038 | -0.835 | -0.222 |
| sp\|Q8IZF6\|AGRG4_HUMAN Adhesion G-protein coupled receptor G4 OS=Homo sapiens GN=ADGRG4 PE=2 S' | 809 | ESASETtQTEI | 0.192 | -0.204 | -0.991 | -0.334 |
| sp\|Q8IZF6\|AGRG4_HUMAN Adhesion G-protein coupled receptor G4 OS=Homo sapiens GN=ADGRG4 PE=2 S' | 811 | ASETTQtEING | 0.151 | -0.007 | -0.916 | -0.257 |
| sp\|Q8IZF6\|AGRG4_HUMAN Adhesion G-protein coupled receptor G4 OS=Homo sapiens GN=ADGRG4 PE=2 S' | 822 | AIVFGGtTTPV | 0.097 | -0.211 | -1.575 | -0.563 |
| sp\|Q8IZF6\|AGRG4_HUMAN Adhesion G-protein coupled receptor G4 OS=Homo sapiens GN=ADGRG4 PE=2 S' | 823 | IVFGGTtTPVP | 0.271 | 0.353 | -0.314 | 0.103 |
| sp\|Q8IZF6\|AGRG4_HUMAN Adhesion G-protein coupled receptor G4 OS=Homo sapiens GN=ADGRG4 PE=2 S' | 824 | VFGGTTtPVPK | 0.035 | -0.442 | -1.415 | -0.607 |
| sp\|Q8IZF6\|AGRG4_HUMAN Adhesion G-protein coupled receptor G4 OS=Homo sapiens GN=ADGRG4 PE=2 S' | 829 | TTPVPKsATTQ | 0.11 | -0.249 | -1.248 | -0.462 |
| sp\|Q8IZF6\|AGRG4_HUMAN Adhesion G-protein coupled receptor G4 OS=Homo sapiens GN=ADGRG4 PE=2 S' | 831 | PVPKSAtTQRL | 0.507 | 0.209 | -0.249 | 0.156 |
| sp\|Q8IZF6\|AGRG4_HUMAN Adhesion G-protein coupled receptor G4 OS=Homo sapiens GN=ADGRG4 PE=2 S' | 832 | VPKSATtQRLN | 0.224 | -0.137 | -0.913 | -0.275 |
| sp\|Q8IZF6\|AGRG4_HUMAN Adhesion G-protein coupled receptor G4 OS=Homo sapiens GN=ADGRG4 PE=2 S' | 838 | TQRLNAtVTRK | 0.448 | 0.076 | -0.449 | 0.025 |
| sp\|Q8IZF6\|AGRG4_HUMAN Adhesion G-protein coupled receptor G4 OS=Homo sapiens GN=ADGRG4 PE=2 S' | 840 | RLNATVtRKEA | 0.355 | -0.017 | -0.616 | -0.093 |
| sp\|Q8IZF6\|AGRG4_HUMAN Adhesion G-protein coupled receptor G4 OS=Homo sapiens GN=ADGRG4 PE=2 S' | 845 | VTRKEAtSHYL | 0.569 | 0.193 | -0.515 | 0.082 |
| sp\|Q8IZF6\|AGRG4_HUMAN Adhesion G-protein coupled receptor G4 OS=Homo sapiens GN=ADGRG4 PE=2 S' | 846 | TRKEATsHYLM | 0.314 | 0.269 | -0.334 | 0.083 |
| sp\|Q8IZF6\|AGRG4_HUMAN Adhesion G-protein coupled receptor G4 OS=Homo sapiens GN=ADGRG4 PE=2 S' | 853 | HYLMRKsTIAA | 0.524 | 0.272 | 0.319 | 0.372 |
| sp\|Q8IZF6\|AGRG4_HUMAN Adhesion G-protein coupled receptor G4 OS=Homo sapiens GN=ADGRG4 PE=2 S' | 854 | YLMRKStIAAV | 0.429 | 0.839 | 0.075 | 0.448 |
| sp\|Q8IZF6\|AGRG4_HUMAN Adhesion G-protein coupled receptor G4 OS=Homo sapiens GN=ADGRG4 PE=2 S' | 862 | AAVAEVsPFST | 0.058 | -0.569 | -1.643 | -0.718 |
| sp\|Q8IZF6\|AGRG4_HUMAN Adhesion G-protein coupled receptor G4 OS=Homo sapiens GN=ADGRG4 PE=2 S' | 865 | AEVSPFsTMLE | 0.093 | -0.237 | -1.309 | -0.484 |
| sp\|Q8IZF6\|AGRG4_HUMAN Adhesion G-protein coupled receptor G4 OS=Homo sapiens GN=ADGRG4 PE=2 S' | 866 | EVSPFStMLEV | 0.057 | -0.126 | -1.656 | -0.575 |

| | | | | | | |
|---|---|---|---|---|---|---|
| sp\|Q8IZF6\|AGRG4_HUMAN Adhesion G-protein coupled receptor G4 OS=Homo sapiens GN=ADGRG4 PE=2 S\ | 871 | STMLEVtDESA | 0.132 | -0.158 | -1.391 | -0.472 |
| sp\|Q8IZF6\|AGRG4_HUMAN Adhesion G-protein coupled receptor G4 OS=Homo sapiens GN=ADGRG4 PE=2 S\ | 874 | LEVTDEsAQRV | 0.124 | -0.204 | -1.684 | -0.588 |
| sp\|Q8IZF6\|AGRG4_HUMAN Adhesion G-protein coupled receptor G4 OS=Homo sapiens GN=ADGRG4 PE=2 S\ | 879 | ESAQRVtASVT | 0.293 | 0.009 | -0.508 | -0.069 |
| sp\|Q8IZF6\|AGRG4_HUMAN Adhesion G-protein coupled receptor G4 OS=Homo sapiens GN=ADGRG4 PE=2 S\ | 881 | AQRVTAsVTVS | 0.229 | 0.201 | -0.71 | -0.093 |
| sp\|Q8IZF6\|AGRG4_HUMAN Adhesion G-protein coupled receptor G4 OS=Homo sapiens GN=ADGRG4 PE=2 S\ | 883 | RVTASVtVSSF | 0.229 | 0.097 | -0.465 | -0.046 |
| sp\|Q8IZF6\|AGRG4_HUMAN Adhesion G-protein coupled receptor G4 OS=Homo sapiens GN=ADGRG4 PE=2 S\ | 885 | TASVTVsSFPD | 0.113 | -0.195 | -1.037 | -0.373 |
| sp\|Q8IZF6\|AGRG4_HUMAN Adhesion G-protein coupled receptor G4 OS=Homo sapiens GN=ADGRG4 PE=2 S\ | 886 | ASVTVSsFPDI | 0.677 | 0.46 | 0.152 | 0.43 |
| sp\|Q8IZF6\|AGRG4_HUMAN Adhesion G-protein coupled receptor G4 OS=Homo sapiens GN=ADGRG4 PE=2 S\ | 894 | PDIEKLsTPLD | 0.187 | 0.318 | -0.642 | -0.046 |
| sp\|Q8IZF6\|AGRG4_HUMAN Adhesion G-protein coupled receptor G4 OS=Homo sapiens GN=ADGRG4 PE=2 S\ | 895 | DIEKLStPLDN | 0.144 | -0.285 | -1.3 | -0.48 |
| sp\|Q8IZF6\|AGRG4_HUMAN Adhesion G-protein coupled receptor G4 OS=Homo sapiens GN=ADGRG4 PE=2 S\ | 901 | TPLDNKtATTE | 0.297 | -0.078 | -0.739 | -0.173 |
| sp\|Q8IZF6\|AGRG4_HUMAN Adhesion G-protein coupled receptor G4 OS=Homo sapiens GN=ADGRG4 PE=2 S\ | 903 | LDNKTAtTEVR | 0.148 | -0.022 | -0.95 | -0.275 |
| sp\|Q8IZF6\|AGRG4_HUMAN Adhesion G-protein coupled receptor G4 OS=Homo sapiens GN=ADGRG4 PE=2 S\ | 904 | DNKTATtEVRE | 0.346 | -0.078 | -0.744 | -0.159 |
| sp\|Q8IZF6\|AGRG4_HUMAN Adhesion G-protein coupled receptor G4 OS=Homo sapiens GN=ADGRG4 PE=2 S\ | 909 | TTEVREsWLLT | 0.115 | -0.01 | -1.173 | -0.356 |
| sp\|Q8IZF6\|AGRG4_HUMAN Adhesion G-protein coupled receptor G4 OS=Homo sapiens GN=ADGRG4 PE=2 S\ | 913 | RESWLLtKLVK | 0.083 | -0.225 | -1.331 | -0.491 |
| sp\|Q8IZF6\|AGRG4_HUMAN Adhesion G-protein coupled receptor G4 OS=Homo sapiens GN=ADGRG4 PE=2 S\ | 918 | LTKLVKtTPRS | 0.656 | 0.407 | -0.009 | 0.351 |
| sp\|Q8IZF6\|AGRG4_HUMAN Adhesion G-protein coupled receptor G4 OS=Homo sapiens GN=ADGRG4 PE=2 S\ | 919 | TKLVKTtPRSS | 0.037 | -0.497 | -1.549 | -0.67 |
| sp\|Q8IZF6\|AGRG4_HUMAN Adhesion G-protein coupled receptor G4 OS=Homo sapiens GN=ADGRG4 PE=2 S\ | 922 | VKTTPRsSYNE | 0.14 | -0.137 | -1.151 | -0.383 |
| sp\|Q8IZF6\|AGRG4_HUMAN Adhesion G-protein coupled receptor G4 OS=Homo sapiens GN=ADGRG4 PE=2 S\ | 923 | KTTPRSsYNEM | 0.216 | -0.068 | -0.686 | -0.179 |
| sp\|Q8IZF6\|AGRG4_HUMAN Adhesion G-protein coupled receptor G4 OS=Homo sapiens GN=ADGRG4 PE=2 S\ | 928 | SSYNEMtEMFN | 0.101 | -0.05 | -1.159 | -0.369 |
| sp\|Q8IZF6\|AGRG4_HUMAN Adhesion G-protein coupled receptor G4 OS=Homo sapiens GN=ADGRG4 PE=2 S\ | 936 | MFNFNHtYVAH | 0.32 | 0.12 | -0.534 | -0.031 |
| sp\|Q8IZF6\|AGRG4_HUMAN Adhesion G-protein coupled receptor G4 OS=Homo sapiens GN=ADGRG4 PE=2 S\ | 942 | TYVAHWtSETS | 0.157 | -0.145 | -1.108 | -0.365 |
| sp\|Q8IZF6\|AGRG4_HUMAN Adhesion G-protein coupled receptor G4 OS=Homo sapiens GN=ADGRG4 PE=2 S\ | 943 | YVAHWTsETSE | 0.152 | -0.026 | -0.681 | -0.185 |
| sp\|Q8IZF6\|AGRG4_HUMAN Adhesion G-protein coupled receptor G4 OS=Homo sapiens GN=ADGRG4 PE=2 S\ | 945 | AHWTSEtSEGI | 0.225 | 0.142 | -0.884 | -0.172 |
| sp\|Q8IZF6\|AGRG4_HUMAN Adhesion G-protein coupled receptor G4 OS=Homo sapiens GN=ADGRG4 PE=2 S\ | 946 | HWTSETsEGIS | 0.256 | 0.067 | -0.612 | -0.096 |
| sp\|Q8IZF6\|AGRG4_HUMAN Adhesion G-protein coupled receptor G4 OS=Homo sapiens GN=ADGRG4 PE=2 S\ | 950 | ETSEGIsAGSP | 0.046 | -0.224 | -1.532 | -0.57 |
| sp\|Q8IZF6\|AGRG4_HUMAN Adhesion G-protein coupled receptor G4 OS=Homo sapiens GN=ADGRG4 PE=2 S\ | 953 | EGISAGsPTSG | 0.044 | -0.461 | -1.619 | -0.679 |
| sp\|Q8IZF6\|AGRG4_HUMAN Adhesion G-protein coupled receptor G4 OS=Homo sapiens GN=ADGRG4 PE=2 S\ | 955 | ISAGSPtSGST | 0.192 | -0.04 | -0.46 | -0.103 |
| sp\|Q8IZF6\|AGRG4_HUMAN Adhesion G-protein coupled receptor G4 OS=Homo sapiens GN=ADGRG4 PE=2 S\ | 956 | SAGSPTsGSTH | 0.021 | -0.576 | -2.029 | -0.861 |
| sp\|Q8IZF6\|AGRG4_HUMAN Adhesion G-protein coupled receptor G4 OS=Homo sapiens GN=ADGRG4 PE=2 S\ | 958 | GSPTSGsTHIF | 0.217 | -0.025 | -0.909 | -0.239 |
| sp\|Q8IZF6\|AGRG4_HUMAN Adhesion G-protein coupled receptor G4 OS=Homo sapiens GN=ADGRG4 PE=2 S\ | 959 | SPTSGStHIFG | 0.034 | -0.389 | -1.68 | -0.678 |
| sp\|Q8IZF6\|AGRG4_HUMAN Adhesion G-protein coupled receptor G4 OS=Homo sapiens GN=ADGRG4 PE=2 S\ | 969 | GEPLGAsTTRI | 0.201 | -0.163 | -1.245 | -0.402 |
| sp\|Q8IZF6\|AGRG4_HUMAN Adhesion G-protein coupled receptor G4 OS=Homo sapiens GN=ADGRG4 PE=2 S\ | 970 | EPLGAStTRIS | 0.099 | -0.2 | -1.079 | -0.393 |
| sp\|Q8IZF6\|AGRG4_HUMAN Adhesion G-protein coupled receptor G4 OS=Homo sapiens GN=ADGRG4 PE=2 S\ | 971 | PLGASTtRISE | 0.206 | 0.078 | -0.512 | -0.076 |
| sp\|Q8IZF6\|AGRG4_HUMAN Adhesion G-protein coupled receptor G4 OS=Homo sapiens GN=ADGRG4 PE=2 S\ | 974 | ASTTRIsETSF | 0.245 | -0.007 | -0.73 | -0.164 |
| sp\|Q8IZF6\|AGRG4_HUMAN Adhesion G-protein coupled receptor G4 OS=Homo sapiens GN=ADGRG4 PE=2 S\ | 976 | TTRISEtSFST | 0.262 | 0.218 | -0.61 | -0.043 |
| sp\|Q8IZF6\|AGRG4_HUMAN Adhesion G-protein coupled receptor G4 OS=Homo sapiens GN=ADGRG4 PE=2 S\ | 977 | TRISETsFSTT | 0.125 | 0.015 | -1.004 | -0.288 |
| sp\|Q8IZF6\|AGRG4_HUMAN Adhesion G-protein coupled receptor G4 OS=Homo sapiens GN=ADGRG4 PE=2 S\ | 979 | ISETSFsTTPT | 0.333 | -0.018 | -0.379 | -0.021 |
| sp\|Q8IZF6\|AGRG4_HUMAN Adhesion G-protein coupled receptor G4 OS=Homo sapiens GN=ADGRG4 PE=2 S\ | 980 | SETSFStTPTD | 0.27 | 0.217 | -0.712 | -0.075 |
| sp\|Q8IZF6\|AGRG4_HUMAN Adhesion G-protein coupled receptor G4 OS=Homo sapiens GN=ADGRG4 PE=2 S\ | 981 | ETSFSTtPTDR | 0.325 | -0.09 | -0.496 | -0.087 |
| sp\|Q8IZF6\|AGRG4_HUMAN Adhesion G-protein coupled receptor G4 OS=Homo sapiens GN=ADGRG4 PE=2 S\ | 983 | SFSTTPtDRTA | 0.121 | -0.23 | -1.131 | -0.413 |
| sp\|Q8IZF6\|AGRG4_HUMAN Adhesion G-protein coupled receptor G4 OS=Homo sapiens GN=ADGRG4 PE=2 S\ | 986 | TTPTDRtATSL | 0.066 | -0.21 | -1.722 | -0.622 |
| sp\|Q8IZF6\|AGRG4_HUMAN Adhesion G-protein coupled receptor G4 OS=Homo sapiens GN=ADGRG4 PE=2 S\ | 988 | PTDRTAtSLSD | 0.399 | 0.786 | -0.202 | 0.328 |
| sp\|Q8IZF6\|AGRG4_HUMAN Adhesion G-protein coupled receptor G4 OS=Homo sapiens GN=ADGRG4 PE=2 S\ | 989 | TDRTATsLSDG | 0.547 | 0.246 | -0.065 | 0.243 |
| sp\|Q8IZF6\|AGRG4_HUMAN Adhesion G-protein coupled receptor G4 OS=Homo sapiens GN=ADGRG4 PE=2 S\ | 991 | RTATSLsDGIL | 0.538 | 0.26 | -0.213 | 0.195 |
| sp\|Q8IZF6\|AGRG4_HUMAN Adhesion G-protein coupled receptor G4 OS=Homo sapiens GN=ADGRG4 PE=2 S\ | 1000 | ILPPQPtAAHS | 0.07 | -0.24 | -1.325 | -0.498 |
| sp\|Q8IZF6\|AGRG4_HUMAN Adhesion G-protein coupled receptor G4 OS=Homo sapiens GN=ADGRG4 PE=2 S\ | 1004 | QPTAAHsSATP | 0.153 | -0.103 | -0.949 | -0.3 |
| sp\|Q8IZF6\|AGRG4_HUMAN Adhesion G-protein coupled receptor G4 OS=Homo sapiens GN=ADGRG4 PE=2 S\ | 1005 | PTAAHSsATPV | 0.245 | -0.117 | -1.13 | -0.334 |
| sp\|Q8IZF6\|AGRG4_HUMAN Adhesion G-protein coupled receptor G4 OS=Homo sapiens GN=ADGRG4 PE=2 S\ | 1007 | AAHSSAtPVPV | 0.092 | -0.314 | -1.496 | -0.573 |
| sp\|Q8IZF6\|AGRG4_HUMAN Adhesion G-protein coupled receptor G4 OS=Homo sapiens GN=ADGRG4 PE=2 S\ | 1012 | ATPVPVtHMFS | 0.089 | -0.346 | -1.441 | -0.566 |

| | | | | | | |
|---|---|---|---|---|---|---|
| sp\|Q8IZF6\|AGRG4_HUMAN Adhesion G-protein coupled receptor G4 OS=Homo sapiens GN=ADGRG4 PE=2 S' | 1016 | PVTHMFsLPVN | 0.718 | 0.611 | 0.595 | 0.641 |
| sp\|Q8IZF6\|AGRG4_HUMAN Adhesion G-protein coupled receptor G4 OS=Homo sapiens GN=ADGRG4 PE=2 S' | 1022 | SLPVNGsSVVA | 0.056 | -0.205 | -1.473 | -0.541 |
| sp\|Q8IZF6\|AGRG4_HUMAN Adhesion G-protein coupled receptor G4 OS=Homo sapiens GN=ADGRG4 PE=2 S' | 1023 | LPVNGSsVVAE | 0.05 | -0.186 | -1.184 | -0.44 |
| sp\|Q8IZF6\|AGRG4_HUMAN Adhesion G-protein coupled receptor G4 OS=Homo sapiens GN=ADGRG4 PE=2 S' | 1029 | SVVAEEtEVTM | 0.078 | -0.183 | -1.451 | -0.519 |
| sp\|Q8IZF6\|AGRG4_HUMAN Adhesion G-protein coupled receptor G4 OS=Homo sapiens GN=ADGRG4 PE=2 S' | 1032 | AEETEVtMSEP | 0.156 | -0.171 | -1.409 | -0.475 |
| sp\|Q8IZF6\|AGRG4_HUMAN Adhesion G-protein coupled receptor G4 OS=Homo sapiens GN=ADGRG4 PE=2 S' | 1034 | ETEVTMsEPST | 0.298 | 0.528 | -0.23 | 0.199 |
| sp\|Q8IZF6\|AGRG4_HUMAN Adhesion G-protein coupled receptor G4 OS=Homo sapiens GN=ADGRG4 PE=2 S' | 1037 | VTMSEPsTLAR | 0.108 | -0.188 | -1.217 | -0.432 |
| sp\|Q8IZF6\|AGRG4_HUMAN Adhesion G-protein coupled receptor G4 OS=Homo sapiens GN=ADGRG4 PE=2 S' | 1038 | TMSEPStLARA | 0.059 | -0.231 | -1.262 | -0.478 |
| sp\|Q8IZF6\|AGRG4_HUMAN Adhesion G-protein coupled receptor G4 OS=Homo sapiens GN=ADGRG4 PE=2 S' | 1044 | TLARAFsTSVL | 0.711 | 1.019 | 0.646 | 0.792 |
| sp\|Q8IZF6\|AGRG4_HUMAN Adhesion G-protein coupled receptor G4 OS=Homo sapiens GN=ADGRG4 PE=2 S' | 1045 | LARAFStSVLS | 0.13 | -0.019 | -1.102 | -0.33 |
| sp\|Q8IZF6\|AGRG4_HUMAN Adhesion G-protein coupled receptor G4 OS=Homo sapiens GN=ADGRG4 PE=2 S' | 1046 | ARAFSTsVLSD | 0.325 | 0.365 | -0.221 | 0.156 |
| sp\|Q8IZF6\|AGRG4_HUMAN Adhesion G-protein coupled receptor G4 OS=Homo sapiens GN=ADGRG4 PE=2 S' | 1049 | FSTSVLsDVSN | 0.097 | -0.113 | -1.323 | -0.446 |
| sp\|Q8IZF6\|AGRG4_HUMAN Adhesion G-protein coupled receptor G4 OS=Homo sapiens GN=ADGRG4 PE=2 S' | 1052 | SVLSDVsNLSS | 0.044 | -0.331 | -1.781 | -0.689 |
| sp\|Q8IZF6\|AGRG4_HUMAN Adhesion G-protein coupled receptor G4 OS=Homo sapiens GN=ADGRG4 PE=2 S' | 1055 | SDVSNLsSTTM | 0.042 | -0.396 | -1.631 | -0.662 |
| sp\|Q8IZF6\|AGRG4_HUMAN Adhesion G-protein coupled receptor G4 OS=Homo sapiens GN=ADGRG4 PE=2 S' | 1056 | DVSNLSsTTMT | 0.053 | -0.348 | -1.564 | -0.62 |
| sp\|Q8IZF6\|AGRG4_HUMAN Adhesion G-protein coupled receptor G4 OS=Homo sapiens GN=ADGRG4 PE=2 S' | 1057 | VSNLSStTMTT | 0.132 | -0.094 | -1.012 | -0.325 |
| sp\|Q8IZF6\|AGRG4_HUMAN Adhesion G-protein coupled receptor G4 OS=Homo sapiens GN=ADGRG4 PE=2 S' | 1058 | SNLSSTtMTTA | 0.209 | 0.018 | -0.693 | -0.155 |
| sp\|Q8IZF6\|AGRG4_HUMAN Adhesion G-protein coupled receptor G4 OS=Homo sapiens GN=ADGRG4 PE=2 S' | 1060 | LSSTTMtTALV | 0.123 | -0.052 | -1.174 | -0.368 |
| sp\|Q8IZF6\|AGRG4_HUMAN Adhesion G-protein coupled receptor G4 OS=Homo sapiens GN=ADGRG4 PE=2 S' | 1061 | SSTTMTtALVP | 0.067 | -0.255 | -1.479 | -0.556 |
| sp\|Q8IZF6\|AGRG4_HUMAN Adhesion G-protein coupled receptor G4 OS=Homo sapiens GN=ADGRG4 PE=2 S' | 1070 | VPPLDQtASTT | 0.052 | -0.304 | -1.626 | -0.626 |
| sp\|Q8IZF6\|AGRG4_HUMAN Adhesion G-protein coupled receptor G4 OS=Homo sapiens GN=ADGRG4 PE=2 S' | 1072 | PLDQTAsTTIV | 0.428 | 0.09 | -0.65 | -0.044 |
| sp\|Q8IZF6\|AGRG4_HUMAN Adhesion G-protein coupled receptor G4 OS=Homo sapiens GN=ADGRG4 PE=2 S' | 1073 | LDQTAStTIVI | 0.133 | -0.031 | -0.781 | -0.226 |
| sp\|Q8IZF6\|AGRG4_HUMAN Adhesion G-protein coupled receptor G4 OS=Homo sapiens GN=ADGRG4 PE=2 S' | 1074 | DQTASTtIVIV | 0.335 | 0.085 | -0.48 | -0.02 |
| sp\|Q8IZF6\|AGRG4_HUMAN Adhesion G-protein coupled receptor G4 OS=Homo sapiens GN=ADGRG4 PE=2 S' | 1080 | TIVIVPtHGDL | 0.365 | 0.117 | -0.469 | 0.004 |
| sp\|Q8IZF6\|AGRG4_HUMAN Adhesion G-protein coupled receptor G4 OS=Homo sapiens GN=ADGRG4 PE=2 S' | 1087 | HGDLIRtTSEA | 0.087 | -0.131 | -1.103 | -0.382 |
| sp\|Q8IZF6\|AGRG4_HUMAN Adhesion G-protein coupled receptor G4 OS=Homo sapiens GN=ADGRG4 PE=2 S' | 1088 | GDLIRTtSEAT | 0.197 | 0.049 | -0.644 | -0.133 |
| sp\|Q8IZF6\|AGRG4_HUMAN Adhesion G-protein coupled receptor G4 OS=Homo sapiens GN=ADGRG4 PE=2 S' | 1089 | DLIRTTsEATV | 0.683 | 0.982 | 0.181 | 0.615 |
| sp\|Q8IZF6\|AGRG4_HUMAN Adhesion G-protein coupled receptor G4 OS=Homo sapiens GN=ADGRG4 PE=2 S' | 1092 | RTTSEAtVISV | 0.141 | -0.106 | -1.256 | -0.407 |
| sp\|Q8IZF6\|AGRG4_HUMAN Adhesion G-protein coupled receptor G4 OS=Homo sapiens GN=ADGRG4 PE=2 S' | 1095 | SEATVIsVRKT | 0.113 | -0.276 | -1.285 | -0.483 |
| sp\|Q8IZF6\|AGRG4_HUMAN Adhesion G-protein coupled receptor G4 OS=Homo sapiens GN=ADGRG4 PE=2 S' | 1099 | VISVRKtSMAV | 0.358 | 0.202 | -0.468 | 0.031 |
| sp\|Q8IZF6\|AGRG4_HUMAN Adhesion G-protein coupled receptor G4 OS=Homo sapiens GN=ADGRG4 PE=2 S' | 1100 | ISVRKTsMAVP | 0.344 | 0.734 | -0.19 | 0.296 |
| sp\|Q8IZF6\|AGRG4_HUMAN Adhesion G-protein coupled receptor G4 OS=Homo sapiens GN=ADGRG4 PE=2 S' | 1105 | TSMAVPsLTET | 0.277 | -0.139 | -0.642 | -0.168 |
| sp\|Q8IZF6\|AGRG4_HUMAN Adhesion G-protein coupled receptor G4 OS=Homo sapiens GN=ADGRG4 PE=2 S' | 1107 | MAVPSLtETPF | 0.219 | 0.101 | -0.573 | -0.084 |
| sp\|Q8IZF6\|AGRG4_HUMAN Adhesion G-protein coupled receptor G4 OS=Homo sapiens GN=ADGRG4 PE=2 S' | 1109 | VPSLTEtPFHS | 0.027 | -0.534 | -1.861 | -0.789 |
| sp\|Q8IZF6\|AGRG4_HUMAN Adhesion G-protein coupled receptor G4 OS=Homo sapiens GN=ADGRG4 PE=2 S' | 1113 | TETPFHsLRLS | 0.298 | 0.001 | -0.635 | -0.112 |
| sp\|Q8IZF6\|AGRG4_HUMAN Adhesion G-protein coupled receptor G4 OS=Homo sapiens GN=ADGRG4 PE=2 S' | 1117 | FHSLRLsTPVT | 0.476 | 0.568 | -0.344 | 0.233 |
| sp\|Q8IZF6\|AGRG4_HUMAN Adhesion G-protein coupled receptor G4 OS=Homo sapiens GN=ADGRG4 PE=2 S' | 1118 | HSLRLStPVTA | 0.167 | 0.379 | -0.483 | 0.021 |
| sp\|Q8IZF6\|AGRG4_HUMAN Adhesion G-protein coupled receptor G4 OS=Homo sapiens GN=ADGRG4 PE=2 S' | 1121 | RLSTPVtAKAE | 0.417 | 0.028 | -0.4 | 0.015 |
| sp\|Q8IZF6\|AGRG4_HUMAN Adhesion G-protein coupled receptor G4 OS=Homo sapiens GN=ADGRG4 PE=2 S' | 1126 | VTAKAEtTLFS | 0.165 | -0.162 | -1.149 | -0.382 |
| sp\|Q8IZF6\|AGRG4_HUMAN Adhesion G-protein coupled receptor G4 OS=Homo sapiens GN=ADGRG4 PE=2 S' | 1127 | TAKAETtLFST | 0.188 | -0.141 | -0.688 | -0.214 |
| sp\|Q8IZF6\|AGRG4_HUMAN Adhesion G-protein coupled receptor G4 OS=Homo sapiens GN=ADGRG4 PE=2 S' | 1130 | AETTLFsTSVD | 0.108 | -0.144 | -1.264 | -0.433 |
| sp\|Q8IZF6\|AGRG4_HUMAN Adhesion G-protein coupled receptor G4 OS=Homo sapiens GN=ADGRG4 PE=2 S' | 1131 | ETTLFStSVDT | 0.092 | -0.187 | -1.262 | -0.452 |
| sp\|Q8IZF6\|AGRG4_HUMAN Adhesion G-protein coupled receptor G4 OS=Homo sapiens GN=ADGRG4 PE=2 S' | 1132 | TTLFSTsVDTV | 0.464 | 0.19 | -0.301 | 0.118 |
| sp\|Q8IZF6\|AGRG4_HUMAN Adhesion G-protein coupled receptor G4 OS=Homo sapiens GN=ADGRG4 PE=2 S' | 1135 | FSTSVDtVTPS | 0.146 | -0.24 | -1.213 | -0.436 |
| sp\|Q8IZF6\|AGRG4_HUMAN Adhesion G-protein coupled receptor G4 OS=Homo sapiens GN=ADGRG4 PE=2 S' | 1137 | TSVDTVtPSTH | 0.034 | -0.587 | -1.926 | -0.826 |
| sp\|Q8IZF6\|AGRG4_HUMAN Adhesion G-protein coupled receptor G4 OS=Homo sapiens GN=ADGRG4 PE=2 S' | 1139 | VDTVTPsTHTL | 0.056 | -0.203 | -1.277 | -0.475 |
| sp\|Q8IZF6\|AGRG4_HUMAN Adhesion G-protein coupled receptor G4 OS=Homo sapiens GN=ADGRG4 PE=2 S' | 1140 | DTVTPStHTLV | 0.168 | -0.236 | -1.425 | -0.498 |
| sp\|Q8IZF6\|AGRG4_HUMAN Adhesion G-protein coupled receptor G4 OS=Homo sapiens GN=ADGRG4 PE=2 S' | 1142 | VTPSTHtLVCS | 0.168 | 0.064 | -0.931 | -0.233 |
| sp\|Q8IZF6\|AGRG4_HUMAN Adhesion G-protein coupled receptor G4 OS=Homo sapiens GN=ADGRG4 PE=2 S' | 1146 | THTLVCsKPPP | 0.409 | 0.309 | -0.618 | 0.033 |

| | | | | | | | |
|---|---|---|---|---|---|---|---|
| sp\|Q8IZF6\|AGRG4_HUMAN Adhesion G-protein coupled receptor G4 OS=Homo sapiens GN=ADGRG4 PE=2 S' | 1157 | DNIPPAsSTHV | 0.103 | -0.328 | -1.402 | -0.542 |
| sp\|Q8IZF6\|AGRG4_HUMAN Adhesion G-protein coupled receptor G4 OS=Homo sapiens GN=ADGRG4 PE=2 S' | 1158 | NIPPASsTHVI | 0.211 | 0.026 | -0.727 | -0.163 |
| sp\|Q8IZF6\|AGRG4_HUMAN Adhesion G-protein coupled receptor G4 OS=Homo sapiens GN=ADGRG4 PE=2 S' | 1159 | IPPASStHVIS | 0.197 | -0.046 | -0.81 | -0.22 |
| sp\|Q8IZF6\|AGRG4_HUMAN Adhesion G-protein coupled receptor G4 OS=Homo sapiens GN=ADGRG4 PE=2 S' | 1163 | SSTHVIsTTST | 0.101 | -0.315 | -1.352 | -0.522 |
| sp\|Q8IZF6\|AGRG4_HUMAN Adhesion G-protein coupled receptor G4 OS=Homo sapiens GN=ADGRG4 PE=2 S' | 1164 | STHVIStTSTP | 0.019 | -0.432 | -2.031 | -0.815 |
| sp\|Q8IZF6\|AGRG4_HUMAN Adhesion G-protein coupled receptor G4 OS=Homo sapiens GN=ADGRG4 PE=2 S' | 1165 | THVISTtSTPE | 0.175 | 0.002 | -1.03 | -0.284 |
| sp\|Q8IZF6\|AGRG4_HUMAN Adhesion G-protein coupled receptor G4 OS=Homo sapiens GN=ADGRG4 PE=2 S' | 1166 | HVISTTsTPEA | 0.371 | 0.502 | -0.095 | 0.259 |
| sp\|Q8IZF6\|AGRG4_HUMAN Adhesion G-protein coupled receptor G4 OS=Homo sapiens GN=ADGRG4 PE=2 S' | 1167 | VISTTStPEAT | 0.052 | -0.371 | -1.472 | -0.597 |
| sp\|Q8IZF6\|AGRG4_HUMAN Adhesion G-protein coupled receptor G4 OS=Homo sapiens GN=ADGRG4 PE=2 S' | 1171 | TSTPEAtQPIS | 0.424 | 0.232 | -0.587 | 0.023 |
| sp\|Q8IZF6\|AGRG4_HUMAN Adhesion G-protein coupled receptor G4 OS=Homo sapiens GN=ADGRG4 PE=2 S' | 1175 | EATQPIsQVEE | 0.13 | -0.245 | -1.004 | -0.373 |
| sp\|Q8IZF6\|AGRG4_HUMAN Adhesion G-protein coupled receptor G4 OS=Homo sapiens GN=ADGRG4 PE=2 S' | 1180 | ISQVEEtSTYA | 0.117 | -0.281 | -1.427 | -0.53 |
| sp\|Q8IZF6\|AGRG4_HUMAN Adhesion G-protein coupled receptor G4 OS=Homo sapiens GN=ADGRG4 PE=2 S' | 1181 | SQVEETsTYAL | 0.071 | -0.078 | -1.206 | -0.404 |
| sp\|Q8IZF6\|AGRG4_HUMAN Adhesion G-protein coupled receptor G4 OS=Homo sapiens GN=ADGRG4 PE=2 S' | 1182 | QVEETStYALS | 0.027 | -0.247 | -1.669 | -0.63 |
| sp\|Q8IZF6\|AGRG4_HUMAN Adhesion G-protein coupled receptor G4 OS=Homo sapiens GN=ADGRG4 PE=2 S' | 1186 | TSTYALsFPYT | 0.525 | 0.433 | 0.164 | 0.374 |
| sp\|Q8IZF6\|AGRG4_HUMAN Adhesion G-protein coupled receptor G4 OS=Homo sapiens GN=ADGRG4 PE=2 S' | 1190 | ALSFPYtFSGG | 0.282 | -0.009 | -0.37 | -0.032 |
| sp\|Q8IZF6\|AGRG4_HUMAN Adhesion G-protein coupled receptor G4 OS=Homo sapiens GN=ADGRG4 PE=2 S' | 1192 | SFPYTFsGGGV | 0.112 | -0.067 | -0.941 | -0.299 |
| sp\|Q8IZF6\|AGRG4_HUMAN Adhesion G-protein coupled receptor G4 OS=Homo sapiens GN=ADGRG4 PE=2 S' | 1199 | GGGVVAsLATG | 0.261 | -0.014 | -0.679 | -0.144 |
| sp\|Q8IZF6\|AGRG4_HUMAN Adhesion G-protein coupled receptor G4 OS=Homo sapiens GN=ADGRG4 PE=2 S' | 1202 | VVASLAtGTTE | 0.106 | -0.275 | -1.201 | -0.457 |
| sp\|Q8IZF6\|AGRG4_HUMAN Adhesion G-protein coupled receptor G4 OS=Homo sapiens GN=ADGRG4 PE=2 S' | 1204 | ASLATGtTETS | 0.053 | -0.305 | -1.516 | -0.589 |
| sp\|Q8IZF6\|AGRG4_HUMAN Adhesion G-protein coupled receptor G4 OS=Homo sapiens GN=ADGRG4 PE=2 S' | 1205 | SLATGTtETSV | 0.124 | -0.072 | -1.202 | -0.383 |
| sp\|Q8IZF6\|AGRG4_HUMAN Adhesion G-protein coupled receptor G4 OS=Homo sapiens GN=ADGRG4 PE=2 S' | 1207 | ATGTTEtSVVD | 0.084 | -0.147 | -1.494 | -0.519 |
| sp\|Q8IZF6\|AGRG4_HUMAN Adhesion G-protein coupled receptor G4 OS=Homo sapiens GN=ADGRG4 PE=2 S' | 1208 | TGTTETsVVDE | 0.201 | -0.102 | -0.807 | -0.236 |
| sp\|Q8IZF6\|AGRG4_HUMAN Adhesion G-protein coupled receptor G4 OS=Homo sapiens GN=ADGRG4 PE=2 S' | 1213 | TSVVDEtTPSH | 0.106 | 0.063 | -1.271 | -0.367 |
| sp\|Q8IZF6\|AGRG4_HUMAN Adhesion G-protein coupled receptor G4 OS=Homo sapiens GN=ADGRG4 PE=2 S' | 1214 | SVVDETtPSHI | 0.033 | -0.484 | -1.769 | -0.74 |
| sp\|Q8IZF6\|AGRG4_HUMAN Adhesion G-protein coupled receptor G4 OS=Homo sapiens GN=ADGRG4 PE=2 S' | 1216 | VDETTPsHISA | 0.054 | -0.301 | -1.298 | -0.515 |
| sp\|Q8IZF6\|AGRG4_HUMAN Adhesion G-protein coupled receptor G4 OS=Homo sapiens GN=ADGRG4 PE=2 S' | 1219 | TTPSHIsANKL | 0.174 | -0.077 | -1.038 | -0.314 |
| sp\|Q8IZF6\|AGRG4_HUMAN Adhesion G-protein coupled receptor G4 OS=Homo sapiens GN=ADGRG4 PE=2 S' | 1224 | ISANKLtTSVN | 0.055 | -0.246 | -1.233 | -0.475 |
| sp\|Q8IZF6\|AGRG4_HUMAN Adhesion G-protein coupled receptor G4 OS=Homo sapiens GN=ADGRG4 PE=2 S' | 1225 | SANKLTtSVNS | 0.081 | -0.177 | -1.636 | -0.577 |
| sp\|Q8IZF6\|AGRG4_HUMAN Adhesion G-protein coupled receptor G4 OS=Homo sapiens GN=ADGRG4 PE=2 S' | 1226 | ANKLTTsVNSH | 0.073 | -0.214 | -1.19 | -0.444 |
| sp\|Q8IZF6\|AGRG4_HUMAN Adhesion G-protein coupled receptor G4 OS=Homo sapiens GN=ADGRG4 PE=2 S' | 1229 | LTTSVNsHISS | 0.125 | -0.139 | -1.078 | -0.364 |
| sp\|Q8IZF6\|AGRG4_HUMAN Adhesion G-protein coupled receptor G4 OS=Homo sapiens GN=ADGRG4 PE=2 S' | 1232 | SVNSHIsSSAT | 0.07 | -0.202 | -1.394 | -0.509 |
| sp\|Q8IZF6\|AGRG4_HUMAN Adhesion G-protein coupled receptor G4 OS=Homo sapiens GN=ADGRG4 PE=2 S' | 1233 | VNSHISsSATY | 0.061 | -0.37 | -1.304 | -0.538 |
| sp\|Q8IZF6\|AGRG4_HUMAN Adhesion G-protein coupled receptor G4 OS=Homo sapiens GN=ADGRG4 PE=2 S' | 1234 | NSHISSsATYR | 0.193 | 0.027 | -0.93 | -0.237 |
| sp\|Q8IZF6\|AGRG4_HUMAN Adhesion G-protein coupled receptor G4 OS=Homo sapiens GN=ADGRG4 PE=2 S' | 1236 | HISSSAtYRVH | 0.244 | 0.07 | -0.626 | -0.104 |
| sp\|Q8IZF6\|AGRG4_HUMAN Adhesion G-protein coupled receptor G4 OS=Homo sapiens GN=ADGRG4 PE=2 S' | 1241 | ATYRVHtPVSI | 0.424 | 0.623 | -0.19 | 0.286 |
| sp\|Q8IZF6\|AGRG4_HUMAN Adhesion G-protein coupled receptor G4 OS=Homo sapiens GN=ADGRG4 PE=2 S' | 1244 | RVHTPVsIQLV | 0.155 | -0.14 | -1.058 | -0.348 |
| sp\|Q8IZF6\|AGRG4_HUMAN Adhesion G-protein coupled receptor G4 OS=Homo sapiens GN=ADGRG4 PE=2 S' | 1249 | VSIQLVtSTSV | 0.133 | -0.246 | -1.354 | -0.489 |
| sp\|Q8IZF6\|AGRG4_HUMAN Adhesion G-protein coupled receptor G4 OS=Homo sapiens GN=ADGRG4 PE=2 S' | 1250 | SIQLVTsTSVL | 0.124 | 0.02 | -1.166 | -0.341 |
| sp\|Q8IZF6\|AGRG4_HUMAN Adhesion G-protein coupled receptor G4 OS=Homo sapiens GN=ADGRG4 PE=2 S' | 1251 | IQLVTStSVLS | 0.037 | -0.181 | -1.349 | -0.498 |
| sp\|Q8IZF6\|AGRG4_HUMAN Adhesion G-protein coupled receptor G4 OS=Homo sapiens GN=ADGRG4 PE=2 S' | 1252 | QLVTSTsVLSS | 0.182 | 0.153 | -0.67 | -0.112 |
| sp\|Q8IZF6\|AGRG4_HUMAN Adhesion G-protein coupled receptor G4 OS=Homo sapiens GN=ADGRG4 PE=2 S' | 1255 | TSTSVLsSDKD | 0.107 | -0.234 | -1.147 | -0.425 |
| sp\|Q8IZF6\|AGRG4_HUMAN Adhesion G-protein coupled receptor G4 OS=Homo sapiens GN=ADGRG4 PE=2 S' | 1256 | STSVLSsDKDQ | 0.065 | -0.269 | -1.695 | -0.633 |
| sp\|Q8IZF6\|AGRG4_HUMAN Adhesion G-protein coupled receptor G4 OS=Homo sapiens GN=ADGRG4 PE=2 S' | 1262 | SDKDQMtISLG | 0.072 | -0.109 | -1.342 | -0.46 |
| sp\|Q8IZF6\|AGRG4_HUMAN Adhesion G-protein coupled receptor G4 OS=Homo sapiens GN=ADGRG4 PE=2 S' | 1264 | KDQMTIsLGKT | 0.192 | -0.055 | -0.64 | -0.168 |
| sp\|Q8IZF6\|AGRG4_HUMAN Adhesion G-protein coupled receptor G4 OS=Homo sapiens GN=ADGRG4 PE=2 S' | 1268 | TISLGKtPRTM | 0.079 | -0.418 | -1.14 | -0.493 |
| sp\|Q8IZF6\|AGRG4_HUMAN Adhesion G-protein coupled receptor G4 OS=Homo sapiens GN=ADGRG4 PE=2 S' | 1271 | LGKTPRtMEVT | 0.093 | -0.075 | -1.069 | -0.35 |
| sp\|Q8IZF6\|AGRG4_HUMAN Adhesion G-protein coupled receptor G4 OS=Homo sapiens GN=ADGRG4 PE=2 S' | 1275 | PRTMEVtEMSP | 0.258 | 0.115 | -0.685 | -0.104 |
| sp\|Q8IZF6\|AGRG4_HUMAN Adhesion G-protein coupled receptor G4 OS=Homo sapiens GN=ADGRG4 PE=2 S' | 1278 | MEVTEMsPSKN | 0.075 | -0.27 | -1.639 | -0.611 |
| sp\|Q8IZF6\|AGRG4_HUMAN Adhesion G-protein coupled receptor G4 OS=Homo sapiens GN=ADGRG4 PE=2 S' | 1280 | VTEMSPsKNSF | 0.221 | -0.045 | -0.474 | -0.099 |

| | | | | | | |
|---|---|---|---|---|---|---|
| sp\|Q8IZF6\|AGRG4_HUMAN Adhesion G-protein coupled receptor G4 OS=Homo sapiens GN=ADGRG4 PE=2 S' | 1283 | MSPSKNsFISY | 0.135 | -0.071 | -1.004 | -0.313 |
| sp\|Q8IZF6\|AGRG4_HUMAN Adhesion G-protein coupled receptor G4 OS=Homo sapiens GN=ADGRG4 PE=2 S' | 1286 | SKNSFIsYSRG | 0.117 | -0.3 | -1.527 | -0.57 |
| sp\|Q8IZF6\|AGRG4_HUMAN Adhesion G-protein coupled receptor G4 OS=Homo sapiens GN=ADGRG4 PE=2 S' | 1288 | NSFISYsRGTP | 0.257 | 0.109 | -0.427 | -0.02 |
| sp\|Q8IZF6\|AGRG4_HUMAN Adhesion G-protein coupled receptor G4 OS=Homo sapiens GN=ADGRG4 PE=2 S' | 1291 | ISYSRGtPSLE | 0.057 | -0.474 | -1.534 | -0.65 |
| sp\|Q8IZF6\|AGRG4_HUMAN Adhesion G-protein coupled receptor G4 OS=Homo sapiens GN=ADGRG4 PE=2 S' | 1293 | YSRGTPsLEMT | 0.232 | 0.098 | -0.43 | -0.033 |
| sp\|Q8IZF6\|AGRG4_HUMAN Adhesion G-protein coupled receptor G4 OS=Homo sapiens GN=ADGRG4 PE=2 S' | 1297 | TPSLEMtDTGF | 0.197 | -0.094 | -1.061 | -0.319 |
| sp\|Q8IZF6\|AGRG4_HUMAN Adhesion G-protein coupled receptor G4 OS=Homo sapiens GN=ADGRG4 PE=2 S' | 1299 | SLEMTDtGFPE | 0.108 | -0.247 | -1.167 | -0.435 |
| sp\|Q8IZF6\|AGRG4_HUMAN Adhesion G-protein coupled receptor G4 OS=Homo sapiens GN=ADGRG4 PE=2 S' | 1304 | DTGFPEtTKIS | 0.111 | -0.272 | -1.434 | -0.532 |
| sp\|Q8IZF6\|AGRG4_HUMAN Adhesion G-protein coupled receptor G4 OS=Homo sapiens GN=ADGRG4 PE=2 S' | 1305 | TGFPETtKISS | 0.053 | -0.249 | -1.233 | -0.476 |
| sp\|Q8IZF6\|AGRG4_HUMAN Adhesion G-protein coupled receptor G4 OS=Homo sapiens GN=ADGRG4 PE=2 S' | 1308 | PETTKIsSHQT | 0.141 | -0.202 | -1.053 | -0.371 |
| sp\|Q8IZF6\|AGRG4_HUMAN Adhesion G-protein coupled receptor G4 OS=Homo sapiens GN=ADGRG4 PE=2 S' | 1309 | ETTKISsHQTH | 0.17 | -0.198 | -0.851 | -0.293 |
| sp\|Q8IZF6\|AGRG4_HUMAN Adhesion G-protein coupled receptor G4 OS=Homo sapiens GN=ADGRG4 PE=2 S' | 1312 | KISSHQtHSPS | 0.119 | -0.154 | -1.152 | -0.396 |
| sp\|Q8IZF6\|AGRG4_HUMAN Adhesion G-protein coupled receptor G4 OS=Homo sapiens GN=ADGRG4 PE=2 S' | 1314 | SSHQTHsPSEI | 0.067 | -0.309 | -1.358 | -0.533 |
| sp\|Q8IZF6\|AGRG4_HUMAN Adhesion G-protein coupled receptor G4 OS=Homo sapiens GN=ADGRG4 PE=2 S' | 1316 | HQTHSPsEIPL | 0.232 | 0.21 | -0.016 | 0.142 |
| sp\|Q8IZF6\|AGRG4_HUMAN Adhesion G-protein coupled receptor G4 OS=Homo sapiens GN=ADGRG4 PE=2 S' | 1322 | SEIPLGtPSDG | 0.026 | -0.571 | -2.168 | -0.904 |
| sp\|Q8IZF6\|AGRG4_HUMAN Adhesion G-protein coupled receptor G4 OS=Homo sapiens GN=ADGRG4 PE=2 S' | 1324 | IPLGTPsDGNL | 0.211 | 0.048 | -0.75 | -0.164 |
| sp\|Q8IZF6\|AGRG4_HUMAN Adhesion G-protein coupled receptor G4 OS=Homo sapiens GN=ADGRG4 PE=2 S' | 1330 | SDGNLAsSPTS | 0.117 | 0.171 | -0.992 | -0.235 |
| sp\|Q8IZF6\|AGRG4_HUMAN Adhesion G-protein coupled receptor G4 OS=Homo sapiens GN=ADGRG4 PE=2 S' | 1331 | DGNLASsPTSG | 0.076 | -0.429 | -1.629 | -0.661 |
| sp\|Q8IZF6\|AGRG4_HUMAN Adhesion G-protein coupled receptor G4 OS=Homo sapiens GN=ADGRG4 PE=2 S' | 1333 | NLASSPtSGST | 0.263 | 0.075 | -0.378 | -0.013 |
| sp\|Q8IZF6\|AGRG4_HUMAN Adhesion G-protein coupled receptor G4 OS=Homo sapiens GN=ADGRG4 PE=2 S' | 1334 | LASSPTsGSTQ | 0.045 | -0.46 | -1.464 | -0.626 |
| sp\|Q8IZF6\|AGRG4_HUMAN Adhesion G-protein coupled receptor G4 OS=Homo sapiens GN=ADGRG4 PE=2 S' | 1336 | SSPTSGsTQIT | 0.11 | -0.162 | -1.197 | -0.416 |
| sp\|Q8IZF6\|AGRG4_HUMAN Adhesion G-protein coupled receptor G4 OS=Homo sapiens GN=ADGRG4 PE=2 S' | 1337 | SPTSGStQITP | 0.019 | -0.476 | -2.029 | -0.829 |
| sp\|Q8IZF6\|AGRG4_HUMAN Adhesion G-protein coupled receptor G4 OS=Homo sapiens GN=ADGRG4 PE=2 S' | 1340 | SGSTQItPTLT | 0.053 | -0.528 | -1.733 | -0.736 |
| sp\|Q8IZF6\|AGRG4_HUMAN Adhesion G-protein coupled receptor G4 OS=Homo sapiens GN=ADGRG4 PE=2 S' | 1342 | STQITPtLTSS | 0.107 | -0.073 | -1.196 | -0.387 |
| sp\|Q8IZF6\|AGRG4_HUMAN Adhesion G-protein coupled receptor G4 OS=Homo sapiens GN=ADGRG4 PE=2 S' | 1344 | QITPTLtSSNT | 0.041 | -0.154 | -1.46 | -0.524 |
| sp\|Q8IZF6\|AGRG4_HUMAN Adhesion G-protein coupled receptor G4 OS=Homo sapiens GN=ADGRG4 PE=2 S' | 1345 | ITPTLTsSNTV | 0.128 | -0.23 | -1.322 | -0.475 |
| sp\|Q8IZF6\|AGRG4_HUMAN Adhesion G-protein coupled receptor G4 OS=Homo sapiens GN=ADGRG4 PE=2 S' | 1346 | TPTLTSsNTVG | 0.073 | -0.165 | -1.442 | -0.511 |
| sp\|Q8IZF6\|AGRG4_HUMAN Adhesion G-protein coupled receptor G4 OS=Homo sapiens GN=ADGRG4 PE=2 S' | 1348 | TLTSSNtVGVH | 0.5 | 0.228 | -0.215 | 0.171 |
| sp\|Q8IZF6\|AGRG4_HUMAN Adhesion G-protein coupled receptor G4 OS=Homo sapiens GN=ADGRG4 PE=2 S' | 1357 | VHIPEMsTSLG | 0.105 | -0.084 | -1.431 | -0.47 |
| sp\|Q8IZF6\|AGRG4_HUMAN Adhesion G-protein coupled receptor G4 OS=Homo sapiens GN=ADGRG4 PE=2 S' | 1358 | HIPEMStSLGK | 0.041 | -0.274 | -1.467 | -0.567 |
| sp\|Q8IZF6\|AGRG4_HUMAN Adhesion G-protein coupled receptor G4 OS=Homo sapiens GN=ADGRG4 PE=2 S' | 1359 | IPEMSTsLGKT | 0.552 | 0.15 | 0.195 | 0.299 |
| sp\|Q8IZF6\|AGRG4_HUMAN Adhesion G-protein coupled receptor G4 OS=Homo sapiens GN=ADGRG4 PE=2 S' | 1363 | STSLGKtALPS | 0.067 | -0.231 | -1.561 | -0.575 |
| sp\|Q8IZF6\|AGRG4_HUMAN Adhesion G-protein coupled receptor G4 OS=Homo sapiens GN=ADGRG4 PE=2 S' | 1367 | GKTALPsQALT | 0.169 | -0.196 | -1.083 | -0.37 |
| sp\|Q8IZF6\|AGRG4_HUMAN Adhesion G-protein coupled receptor G4 OS=Homo sapiens GN=ADGRG4 PE=2 S' | 1371 | LPSQALtITTF | 0.128 | -0.189 | -0.871 | -0.311 |
| sp\|Q8IZF6\|AGRG4_HUMAN Adhesion G-protein coupled receptor G4 OS=Homo sapiens GN=ADGRG4 PE=2 S' | 1373 | SQALTItTFLC | 0.072 | -0.155 | -1.253 | -0.445 |
| sp\|Q8IZF6\|AGRG4_HUMAN Adhesion G-protein coupled receptor G4 OS=Homo sapiens GN=ADGRG4 PE=2 S' | 1374 | QALTITtFLCP | 0.115 | -0.097 | -0.914 | -0.299 |
| sp\|Q8IZF6\|AGRG4_HUMAN Adhesion G-protein coupled receptor G4 OS=Homo sapiens GN=ADGRG4 PE=2 S' | 1382 | LCPEKEsTSAL | 0.054 | -0.153 | -1.528 | -0.542 |
| sp\|Q8IZF6\|AGRG4_HUMAN Adhesion G-protein coupled receptor G4 OS=Homo sapiens GN=ADGRG4 PE=2 S' | 1383 | CPEKEStSALP | 0.102 | -0.255 | -1.263 | -0.472 |
| sp\|Q8IZF6\|AGRG4_HUMAN Adhesion G-protein coupled receptor G4 OS=Homo sapiens GN=ADGRG4 PE=2 S' | 1384 | PEKESTsALPA | 0.233 | 0.127 | -0.622 | -0.087 |
| sp\|Q8IZF6\|AGRG4_HUMAN Adhesion G-protein coupled receptor G4 OS=Homo sapiens GN=ADGRG4 PE=2 S' | 1390 | SALPAYtPRTV | 0.076 | -0.422 | -1.399 | -0.582 |
| sp\|Q8IZF6\|AGRG4_HUMAN Adhesion G-protein coupled receptor G4 OS=Homo sapiens GN=ADGRG4 PE=2 S' | 1393 | PAYTPRtVEMI | 0.106 | -0.185 | -1.112 | -0.397 |
| sp\|Q8IZF6\|AGRG4_HUMAN Adhesion G-protein coupled receptor G4 OS=Homo sapiens GN=ADGRG4 PE=2 S' | 1400 | VEMIVNsTYVT | 0.138 | -0.032 | -1.158 | -0.351 |
| sp\|Q8IZF6\|AGRG4_HUMAN Adhesion G-protein coupled receptor G4 OS=Homo sapiens GN=ADGRG4 PE=2 S' | 1401 | EMIVNStYVTH | 0.025 | -0.218 | -1.724 | -0.639 |
| sp\|Q8IZF6\|AGRG4_HUMAN Adhesion G-protein coupled receptor G4 OS=Homo sapiens GN=ADGRG4 PE=2 S' | 1404 | VNSTYVtHSVS | 0.153 | -0.241 | -1.08 | -0.389 |
| sp\|Q8IZF6\|AGRG4_HUMAN Adhesion G-protein coupled receptor G4 OS=Homo sapiens GN=ADGRG4 PE=2 S' | 1406 | STYVTHsVSYG | 0.065 | -0.069 | -1.47 | -0.491 |
| sp\|Q8IZF6\|AGRG4_HUMAN Adhesion G-protein coupled receptor G4 OS=Homo sapiens GN=ADGRG4 PE=2 S' | 1408 | YVTHSVsYGQD | 0.538 | 0.196 | 0.246 | 0.327 |
| sp\|Q8IZF6\|AGRG4_HUMAN Adhesion G-protein coupled receptor G4 OS=Homo sapiens GN=ADGRG4 PE=2 S' | 1413 | VSYGQDtSFVD | 0.082 | -0.215 | -1.279 | -0.471 |
| sp\|Q8IZF6\|AGRG4_HUMAN Adhesion G-protein coupled receptor G4 OS=Homo sapiens GN=ADGRG4 PE=2 S' | 1414 | SYGQDTsFVDT | 0.242 | -0.019 | -0.624 | -0.134 |
| sp\|Q8IZF6\|AGRG4_HUMAN Adhesion G-protein coupled receptor G4 OS=Homo sapiens GN=ADGRG4 PE=2 S' | 1418 | DTSFVDtTTSS | 0.147 | -0.272 | -1.562 | -0.562 |

| | | | | | | |
|---|---|---|---|---|---|---|
| sp\|Q8IZF6\|AGRG4_HUMAN Adhesion G-protein coupled receptor G4 OS=Homo sapiens GN=ADGRG4 PE=2 S' | 1419 | TSFVDTtTSSS | 0.036 | -0.337 | -1.746 | -0.682 |
| sp\|Q8IZF6\|AGRG4_HUMAN Adhesion G-protein coupled receptor G4 OS=Homo sapiens GN=ADGRG4 PE=2 S' | 1420 | SFVDTTtSSST | 0.031 | -0.236 | -1.68 | -0.628 |
| sp\|Q8IZF6\|AGRG4_HUMAN Adhesion G-protein coupled receptor G4 OS=Homo sapiens GN=ADGRG4 PE=2 S' | 1421 | FVDTTTsSSTR | 0.072 | -0.097 | -1.24 | -0.422 |
| sp\|Q8IZF6\|AGRG4_HUMAN Adhesion G-protein coupled receptor G4 OS=Homo sapiens GN=ADGRG4 PE=2 S' | 1422 | VDTTTSsSTRI | 0.081 | -0.293 | -1.371 | -0.528 |
| sp\|Q8IZF6\|AGRG4_HUMAN Adhesion G-protein coupled receptor G4 OS=Homo sapiens GN=ADGRG4 PE=2 S' | 1423 | DTTTSSsTRIS | 0.305 | -0.042 | -0.792 | -0.176 |
| sp\|Q8IZF6\|AGRG4_HUMAN Adhesion G-protein coupled receptor G4 OS=Homo sapiens GN=ADGRG4 PE=2 S' | 1424 | TTTSSStRISN | 0.099 | -0.107 | -1.074 | -0.361 |
| sp\|Q8IZF6\|AGRG4_HUMAN Adhesion G-protein coupled receptor G4 OS=Homo sapiens GN=ADGRG4 PE=2 S' | 1427 | SSSTRIsNPMD | 0.578 | 0.361 | -0.286 | 0.218 |
| sp\|Q8IZF6\|AGRG4_HUMAN Adhesion G-protein coupled receptor G4 OS=Homo sapiens GN=ADGRG4 PE=2 S' | 1434 | NPMDINtTFSH | 0.091 | -0.148 | -1.291 | -0.449 |
| sp\|Q8IZF6\|AGRG4_HUMAN Adhesion G-protein coupled receptor G4 OS=Homo sapiens GN=ADGRG4 PE=2 S' | 1435 | PMDINTtFSHL | 0.126 | 0.066 | -0.698 | -0.169 |
| sp\|Q8IZF6\|AGRG4_HUMAN Adhesion G-protein coupled receptor G4 OS=Homo sapiens GN=ADGRG4 PE=2 S' | 1437 | DINTTFsHLHS | 0.108 | -0.042 | -1.219 | -0.384 |
| sp\|Q8IZF6\|AGRG4_HUMAN Adhesion G-protein coupled receptor G4 OS=Homo sapiens GN=ADGRG4 PE=2 S' | 1441 | TFSHLHsLRTQ | 0.308 | -0.048 | -0.512 | -0.084 |
| sp\|Q8IZF6\|AGRG4_HUMAN Adhesion G-protein coupled receptor G4 OS=Homo sapiens GN=ADGRG4 PE=2 S' | 1444 | HLHSLRtQPEV | 0.377 | 0.426 | -0.793 | 0.003 |
| sp\|Q8IZF6\|AGRG4_HUMAN Adhesion G-protein coupled receptor G4 OS=Homo sapiens GN=ADGRG4 PE=2 S' | 1449 | RTQPEVtSVAS | 0.267 | -0.043 | -0.891 | -0.222 |
| sp\|Q8IZF6\|AGRG4_HUMAN Adhesion G-protein coupled receptor G4 OS=Homo sapiens GN=ADGRG4 PE=2 S' | 1450 | TQPEVTsVASF | 0.034 | -0.288 | -1.577 | -0.61 |
| sp\|Q8IZF6\|AGRG4_HUMAN Adhesion G-protein coupled receptor G4 OS=Homo sapiens GN=ADGRG4 PE=2 S' | 1453 | EVTSVAsFISE | 0.107 | -0.08 | -0.878 | -0.284 |
| sp\|Q8IZF6\|AGRG4_HUMAN Adhesion G-protein coupled receptor G4 OS=Homo sapiens GN=ADGRG4 PE=2 S' | 1456 | SVASFIsESTQ | 0.075 | -0.239 | -1.401 | -0.522 |
| sp\|Q8IZF6\|AGRG4_HUMAN Adhesion G-protein coupled receptor G4 OS=Homo sapiens GN=ADGRG4 PE=2 S' | 1458 | ASFISEsTQTF | 0.206 | 0.011 | -0.793 | -0.192 |
| sp\|Q8IZF6\|AGRG4_HUMAN Adhesion G-protein coupled receptor G4 OS=Homo sapiens GN=ADGRG4 PE=2 S' | 1459 | SFISEStQTFP | 0.035 | -0.427 | -2.065 | -0.819 |
| sp\|Q8IZF6\|AGRG4_HUMAN Adhesion G-protein coupled receptor G4 OS=Homo sapiens GN=ADGRG4 PE=2 S' | 1461 | ISESTQtFPES | 0.565 | 0.435 | 0.139 | 0.38 |
| sp\|Q8IZF6\|AGRG4_HUMAN Adhesion G-protein coupled receptor G4 OS=Homo sapiens GN=ADGRG4 PE=2 S' | 1465 | TQTFPEsLSLS | 0.111 | -0.159 | -0.88 | -0.309 |
| sp\|Q8IZF6\|AGRG4_HUMAN Adhesion G-protein coupled receptor G4 OS=Homo sapiens GN=ADGRG4 PE=2 S' | 1467 | TFPESLsLSTA | 0.154 | 0.034 | -0.579 | -0.13 |
| sp\|Q8IZF6\|AGRG4_HUMAN Adhesion G-protein coupled receptor G4 OS=Homo sapiens GN=ADGRG4 PE=2 S' | 1469 | PESLSLsTAGL | 0.179 | 0.028 | -0.757 | -0.183 |
| sp\|Q8IZF6\|AGRG4_HUMAN Adhesion G-protein coupled receptor G4 OS=Homo sapiens GN=ADGRG4 PE=2 S' | 1470 | ESLSLStAGLY | 0.106 | -0.149 | -1.22 | -0.421 |
| sp\|Q8IZF6\|AGRG4_HUMAN Adhesion G-protein coupled receptor G4 OS=Homo sapiens GN=ADGRG4 PE=2 S' | 1479 | LYNDGFtVLSD | 0.087 | -0.065 | -1.177 | -0.385 |
| sp\|Q8IZF6\|AGRG4_HUMAN Adhesion G-protein coupled receptor G4 OS=Homo sapiens GN=ADGRG4 PE=2 S' | 1482 | DGFTVLsDRIT | 0.203 | -0.202 | -1.14 | -0.38 |
| sp\|Q8IZF6\|AGRG4_HUMAN Adhesion G-protein coupled receptor G4 OS=Homo sapiens GN=ADGRG4 PE=2 S' | 1486 | VLSDRItTAFS | 0.227 | -0.023 | -0.878 | -0.225 |
| sp\|Q8IZF6\|AGRG4_HUMAN Adhesion G-protein coupled receptor G4 OS=Homo sapiens GN=ADGRG4 PE=2 S' | 1487 | LSDRITtAFSV | 0.409 | 0.747 | -0.079 | 0.359 |
| sp\|Q8IZF6\|AGRG4_HUMAN Adhesion G-protein coupled receptor G4 OS=Homo sapiens GN=ADGRG4 PE=2 S' | 1490 | RITTAFsVPNV | 0.782 | 0.749 | 0.211 | 0.581 |
| sp\|Q8IZF6\|AGRG4_HUMAN Adhesion G-protein coupled receptor G4 OS=Homo sapiens GN=ADGRG4 PE=2 S' | 1496 | SVPNVPtMLPR | 0.041 | -0.203 | -1.592 | -0.585 |
| sp\|Q8IZF6\|AGRG4_HUMAN Adhesion G-protein coupled receptor G4 OS=Homo sapiens GN=ADGRG4 PE=2 S' | 1502 | TMLPREsSMAT | 0.15 | 0.069 | -0.842 | -0.208 |
| sp\|Q8IZF6\|AGRG4_HUMAN Adhesion G-protein coupled receptor G4 OS=Homo sapiens GN=ADGRG4 PE=2 S' | 1503 | MLPRESsMATS | 0.445 | 0.852 | -0.204 | 0.364 |
| sp\|Q8IZF6\|AGRG4_HUMAN Adhesion G-protein coupled receptor G4 OS=Homo sapiens GN=ADGRG4 PE=2 S' | 1506 | RESSMAtSTPI | 0.247 | -0.186 | -0.923 | -0.287 |
| sp\|Q8IZF6\|AGRG4_HUMAN Adhesion G-protein coupled receptor G4 OS=Homo sapiens GN=ADGRG4 PE=2 S' | 1507 | ESSMATsTPIY | 0.659 | 0.405 | 0.097 | 0.387 |
| sp\|Q8IZF6\|AGRG4_HUMAN Adhesion G-protein coupled receptor G4 OS=Homo sapiens GN=ADGRG4 PE=2 S' | 1508 | SSMATStPIYQ | 0.021 | -0.571 | -2.032 | -0.861 |
| sp\|Q8IZF6\|AGRG4_HUMAN Adhesion G-protein coupled receptor G4 OS=Homo sapiens GN=ADGRG4 PE=2 S' | 1514 | TPIYQMsSLPV | 0.079 | -0.055 | -1.455 | -0.477 |
| sp\|Q8IZF6\|AGRG4_HUMAN Adhesion G-protein coupled receptor G4 OS=Homo sapiens GN=ADGRG4 PE=2 S' | 1515 | PIYQMSsLPVN | 0.703 | 0.587 | 0.471 | 0.587 |
| sp\|Q8IZF6\|AGRG4_HUMAN Adhesion G-protein coupled receptor G4 OS=Homo sapiens GN=ADGRG4 PE=2 S' | 1521 | SLPVNVtAFTS | 0.183 | -0.079 | -0.992 | -0.296 |
| sp\|Q8IZF6\|AGRG4_HUMAN Adhesion G-protein coupled receptor G4 OS=Homo sapiens GN=ADGRG4 PE=2 S' | 1524 | VNVTAFtSKKV | 0.113 | -0.154 | -1.238 | -0.426 |
| sp\|Q8IZF6\|AGRG4_HUMAN Adhesion G-protein coupled receptor G4 OS=Homo sapiens GN=ADGRG4 PE=2 S' | 1525 | NVTAFTsKKVS | 0.128 | -0.099 | -0.878 | -0.283 |
| sp\|Q8IZF6\|AGRG4_HUMAN Adhesion G-protein coupled receptor G4 OS=Homo sapiens GN=ADGRG4 PE=2 S' | 1529 | FTSKKVsDTPP | 0.363 | -0.095 | -0.789 | -0.174 |
| sp\|Q8IZF6\|AGRG4_HUMAN Adhesion G-protein coupled receptor G4 OS=Homo sapiens GN=ADGRG4 PE=2 S' | 1531 | SKKVSDtPPIV | 0.591 | 0.337 | -0.274 | 0.218 |
| sp\|Q8IZF6\|AGRG4_HUMAN Adhesion G-protein coupled receptor G4 OS=Homo sapiens GN=ADGRG4 PE=2 S' | 1537 | TPPIVItKSSK | 0.044 | -0.333 | -1.586 | -0.625 |
| sp\|Q8IZF6\|AGRG4_HUMAN Adhesion G-protein coupled receptor G4 OS=Homo sapiens GN=ADGRG4 PE=2 S' | 1539 | PIVITKsSKTM | 0.154 | 0.068 | -0.62 | -0.133 |
| sp\|Q8IZF6\|AGRG4_HUMAN Adhesion G-protein coupled receptor G4 OS=Homo sapiens GN=ADGRG4 PE=2 S' | 1540 | IVITKSsKTMH | 0.065 | -0.363 | -1.586 | -0.628 |
| sp\|Q8IZF6\|AGRG4_HUMAN Adhesion G-protein coupled receptor G4 OS=Homo sapiens GN=ADGRG4 PE=2 S' | 1542 | ITKSSKtMHPG | 0.621 | 0.274 | 0.09 | 0.328 |
| sp\|Q8IZF6\|AGRG4_HUMAN Adhesion G-protein coupled receptor G4 OS=Homo sapiens GN=ADGRG4 PE=2 S' | 1550 | HPGCLKsPCTA | 0.054 | -0.469 | -1.439 | -0.618 |
| sp\|Q8IZF6\|AGRG4_HUMAN Adhesion G-protein coupled receptor G4 OS=Homo sapiens GN=ADGRG4 PE=2 S' | 1553 | CLKSPCtATSG | 0.371 | -0.006 | -0.619 | -0.085 |
| sp\|Q8IZF6\|AGRG4_HUMAN Adhesion G-protein coupled receptor G4 OS=Homo sapiens GN=ADGRG4 PE=2 S' | 1555 | KSPCTAtSGPM | 0.115 | -0.196 | -1.171 | -0.417 |
| sp\|Q8IZF6\|AGRG4_HUMAN Adhesion G-protein coupled receptor G4 OS=Homo sapiens GN=ADGRG4 PE=2 S' | 1556 | SPCTATsGPMS | 0.371 | 0.283 | -0.569 | 0.028 |

| | | | | | | | |
|---|---|---|---|---|---|---|---|
| sp\|Q8IZF6\|AGRG4_HUMAN Adhesion G-protein coupled receptor G4 OS=Homo sapiens GN=ADGRG4 PE=2 SV | 1560 | ATSGPMsEMSS | 0.115 | -0.072 | -1.026 | -0.328 |
| sp\|Q8IZF6\|AGRG4_HUMAN Adhesion G-protein coupled receptor G4 OS=Homo sapiens GN=ADGRG4 PE=2 SV | 1563 | GPMSEMsSIPV | 0.126 | -0.059 | -1.253 | -0.395 |
| sp\|Q8IZF6\|AGRG4_HUMAN Adhesion G-protein coupled receptor G4 OS=Homo sapiens GN=ADGRG4 PE=2 SV | 1564 | PMSEMSsIPVN | 0.237 | 0.413 | -0.499 | 0.05 |
| sp\|Q8IZF6\|AGRG4_HUMAN Adhesion G-protein coupled receptor G4 OS=Homo sapiens GN=ADGRG4 PE=2 SV | 1570 | SIPVNNsAFTP | 0.098 | -0.096 | -1.316 | -0.438 |
| sp\|Q8IZF6\|AGRG4_HUMAN Adhesion G-protein coupled receptor G4 OS=Homo sapiens GN=ADGRG4 PE=2 SV | 1573 | VNNSAFtPATV | 0.057 | -0.49 | -1.64 | -0.691 |
| sp\|Q8IZF6\|AGRG4_HUMAN Adhesion G-protein coupled receptor G4 OS=Homo sapiens GN=ADGRG4 PE=2 SV | 1576 | SAFTPAtVSSD | 0.081 | -0.268 | -1.435 | -0.541 |
| sp\|Q8IZF6\|AGRG4_HUMAN Adhesion G-protein coupled receptor G4 OS=Homo sapiens GN=ADGRG4 PE=2 SV | 1578 | FTPATVsSDTS | 0.178 | -0.084 | -0.978 | -0.295 |
| sp\|Q8IZF6\|AGRG4_HUMAN Adhesion G-protein coupled receptor G4 OS=Homo sapiens GN=ADGRG4 PE=2 SV | 1579 | TPATVSsDTST | 0.081 | -0.286 | -1.496 | -0.567 |
| sp\|Q8IZF6\|AGRG4_HUMAN Adhesion G-protein coupled receptor G4 OS=Homo sapiens GN=ADGRG4 PE=2 SV | 1581 | ATVSSDtSTRV | 0.253 | -0.149 | -1.357 | -0.418 |
| sp\|Q8IZF6\|AGRG4_HUMAN Adhesion G-protein coupled receptor G4 OS=Homo sapiens GN=ADGRG4 PE=2 SV | 1582 | TVSSDTsTRVG | 0.1 | -0.237 | -1.13 | -0.422 |
| sp\|Q8IZF6\|AGRG4_HUMAN Adhesion G-protein coupled receptor G4 OS=Homo sapiens GN=ADGRG4 PE=2 SV | 1583 | VSSDTStRVGL | 0.064 | -0.288 | -1.446 | -0.557 |
| sp\|Q8IZF6\|AGRG4_HUMAN Adhesion G-protein coupled receptor G4 OS=Homo sapiens GN=ADGRG4 PE=2 SV | 1589 | TRVGLFsTLLS | 0.073 | 0.088 | -1.216 | -0.352 |
| sp\|Q8IZF6\|AGRG4_HUMAN Adhesion G-protein coupled receptor G4 OS=Homo sapiens GN=ADGRG4 PE=2 SV | 1590 | RVGLFStLLSS | 0.058 | -0.112 | -1.44 | -0.498 |
| sp\|Q8IZF6\|AGRG4_HUMAN Adhesion G-protein coupled receptor G4 OS=Homo sapiens GN=ADGRG4 PE=2 SV | 1593 | LFSTLLsSVTP | 0.043 | -0.297 | -1.491 | -0.582 |
| sp\|Q8IZF6\|AGRG4_HUMAN Adhesion G-protein coupled receptor G4 OS=Homo sapiens GN=ADGRG4 PE=2 SV | 1594 | FSTLLSsVTPR | 0.112 | -0.174 | -1.123 | -0.395 |
| sp\|Q8IZF6\|AGRG4_HUMAN Adhesion G-protein coupled receptor G4 OS=Homo sapiens GN=ADGRG4 PE=2 SV | 1596 | TLLSSVtPRTT | 0.285 | -0.228 | -0.5 | -0.148 |
| sp\|Q8IZF6\|AGRG4_HUMAN Adhesion G-protein coupled receptor G4 OS=Homo sapiens GN=ADGRG4 PE=2 SV | 1599 | SSVTPRtTMTM | 0.056 | -0.339 | -1.605 | -0.629 |
| sp\|Q8IZF6\|AGRG4_HUMAN Adhesion G-protein coupled receptor G4 OS=Homo sapiens GN=ADGRG4 PE=2 SV | 1600 | SVTPRTtMTMQ | 0.158 | -0.004 | -1.042 | -0.296 |
| sp\|Q8IZF6\|AGRG4_HUMAN Adhesion G-protein coupled receptor G4 OS=Homo sapiens GN=ADGRG4 PE=2 SV | 1602 | TPRTTMtMQTS | 0.294 | 0.35 | -0.6 | 0.015 |
| sp\|Q8IZF6\|AGRG4_HUMAN Adhesion G-protein coupled receptor G4 OS=Homo sapiens GN=ADGRG4 PE=2 SV | 1605 | TTMTMQtSTLD | 0.203 | -0.128 | -0.89 | -0.272 |
| sp\|Q8IZF6\|AGRG4_HUMAN Adhesion G-protein coupled receptor G4 OS=Homo sapiens GN=ADGRG4 PE=2 SV | 1606 | TMTMQTsTLDV | 0.121 | 0.003 | -1.212 | -0.363 |
| sp\|Q8IZF6\|AGRG4_HUMAN Adhesion G-protein coupled receptor G4 OS=Homo sapiens GN=ADGRG4 PE=2 SV | 1607 | MTMQTStLDVT | 0.286 | 0.15 | -0.315 | 0.04 |
| sp\|Q8IZF6\|AGRG4_HUMAN Adhesion G-protein coupled receptor G4 OS=Homo sapiens GN=ADGRG4 PE=2 SV | 1611 | TSTLDVtPVIY | 0.141 | -0.411 | -1.214 | -0.495 |
| sp\|Q8IZF6\|AGRG4_HUMAN Adhesion G-protein coupled receptor G4 OS=Homo sapiens GN=ADGRG4 PE=2 SV | 1619 | VIYAGAtSKNK | 0.104 | -0.136 | -1.185 | -0.406 |
| sp\|Q8IZF6\|AGRG4_HUMAN Adhesion G-protein coupled receptor G4 OS=Homo sapiens GN=ADGRG4 PE=2 SV | 1620 | IYAGATsKNKM | 0.361 | 0.059 | -0.056 | 0.121 |
| sp\|Q8IZF6\|AGRG4_HUMAN Adhesion G-protein coupled receptor G4 OS=Homo sapiens GN=ADGRG4 PE=2 SV | 1626 | SKNKMVsSAFT | 0.217 | -0.246 | -0.98 | -0.336 |
| sp\|Q8IZF6\|AGRG4_HUMAN Adhesion G-protein coupled receptor G4 OS=Homo sapiens GN=ADGRG4 PE=2 SV | 1627 | KNKMVSsAFTT | 0.117 | -0.265 | -1.08 | -0.409 |
| sp\|Q8IZF6\|AGRG4_HUMAN Adhesion G-protein coupled receptor G4 OS=Homo sapiens GN=ADGRG4 PE=2 SV | 1630 | MVSSAFtTEMI | 0.264 | 0.051 | -0.54 | -0.075 |
| sp\|Q8IZF6\|AGRG4_HUMAN Adhesion G-protein coupled receptor G4 OS=Homo sapiens GN=ADGRG4 PE=2 SV | 1631 | VSSAFTtEMIE | 0.2 | -0.179 | -0.952 | -0.31 |
| sp\|Q8IZF6\|AGRG4_HUMAN Adhesion G-protein coupled receptor G4 OS=Homo sapiens GN=ADGRG4 PE=2 SV | 1638 | EMIEAPsRITP | 0.046 | -0.113 | -1.375 | -0.481 |
| sp\|Q8IZF6\|AGRG4_HUMAN Adhesion G-protein coupled receptor G4 OS=Homo sapiens GN=ADGRG4 PE=2 SV | 1641 | EAPSRItPTTF | 0.074 | -0.426 | -1.399 | -0.584 |
| sp\|Q8IZF6\|AGRG4_HUMAN Adhesion G-protein coupled receptor G4 OS=Homo sapiens GN=ADGRG4 PE=2 SV | 1643 | PSRITPtTFLS | 0.211 | 0.139 | -0.676 | -0.109 |
| sp\|Q8IZF6\|AGRG4_HUMAN Adhesion G-protein coupled receptor G4 OS=Homo sapiens GN=ADGRG4 PE=2 SV | 1644 | SRITPTtFLSP | 0.061 | -0.02 | -1.309 | -0.423 |
| sp\|Q8IZF6\|AGRG4_HUMAN Adhesion G-protein coupled receptor G4 OS=Homo sapiens GN=ADGRG4 PE=2 SV | 1647 | TPTTFLsPTEP | 0.049 | -0.541 | -1.802 | -0.765 |
| sp\|Q8IZF6\|AGRG4_HUMAN Adhesion G-protein coupled receptor G4 OS=Homo sapiens GN=ADGRG4 PE=2 SV | 1649 | TTFLSPtEPTL | 0.749 | 0.677 | 0.361 | 0.596 |
| sp\|Q8IZF6\|AGRG4_HUMAN Adhesion G-protein coupled receptor G4 OS=Homo sapiens GN=ADGRG4 PE=2 SV | 1652 | LSPTEPtLPFV | 0.391 | 0.23 | -0.572 | 0.016 |
| sp\|Q8IZF6\|AGRG4_HUMAN Adhesion G-protein coupled receptor G4 OS=Homo sapiens GN=ADGRG4 PE=2 SV | 1658 | TLPFVKtVPTT | 0.658 | 0.494 | 0.256 | 0.469 |
| sp\|Q8IZF6\|AGRG4_HUMAN Adhesion G-protein coupled receptor G4 OS=Homo sapiens GN=ADGRG4 PE=2 SV | 1661 | FVKTVPtTIMA | 0.163 | -0.11 | -0.859 | -0.269 |
| sp\|Q8IZF6\|AGRG4_HUMAN Adhesion G-protein coupled receptor G4 OS=Homo sapiens GN=ADGRG4 PE=2 SV | 1662 | VKTVPTtIMAG | 0.208 | -0.058 | -0.452 | -0.101 |
| sp\|Q8IZF6\|AGRG4_HUMAN Adhesion G-protein coupled receptor G4 OS=Homo sapiens GN=ADGRG4 PE=2 SV | 1669 | IMAGIVtPFVG | 0.151 | -0.177 | -0.618 | -0.215 |
| sp\|Q8IZF6\|AGRG4_HUMAN Adhesion G-protein coupled receptor G4 OS=Homo sapiens GN=ADGRG4 PE=2 SV | 1674 | VTPFVGtTAFS | 0.051 | -0.395 | -1.869 | -0.738 |
| sp\|Q8IZF6\|AGRG4_HUMAN Adhesion G-protein coupled receptor G4 OS=Homo sapiens GN=ADGRG4 PE=2 SV | 1675 | TPFVGTtAFSP | 0.032 | -0.249 | -1.665 | -0.627 |
| sp\|Q8IZF6\|AGRG4_HUMAN Adhesion G-protein coupled receptor G4 OS=Homo sapiens GN=ADGRG4 PE=2 SV | 1678 | VGTTAFsPLSS | 0.052 | -0.383 | -1.429 | -0.587 |
| sp\|Q8IZF6\|AGRG4_HUMAN Adhesion G-protein coupled receptor G4 OS=Homo sapiens GN=ADGRG4 PE=2 SV | 1681 | TAFSPLsSKST | 0.027 | -0.45 | -1.736 | -0.72 |
| sp\|Q8IZF6\|AGRG4_HUMAN Adhesion G-protein coupled receptor G4 OS=Homo sapiens GN=ADGRG4 PE=2 SV | 1682 | AFSPLSsKSTG | 0.056 | -0.343 | -1.364 | -0.55 |
| sp\|Q8IZF6\|AGRG4_HUMAN Adhesion G-protein coupled receptor G4 OS=Homo sapiens GN=ADGRG4 PE=2 SV | 1684 | SPLSSKsTGAI | 0.523 | 0.248 | 0.136 | 0.302 |
| sp\|Q8IZF6\|AGRG4_HUMAN Adhesion G-protein coupled receptor G4 OS=Homo sapiens GN=ADGRG4 PE=2 SV | 1685 | PLSSKStGAIS | 0.08 | -0.269 | -1.299 | -0.496 |
| sp\|Q8IZF6\|AGRG4_HUMAN Adhesion G-protein coupled receptor G4 OS=Homo sapiens GN=ADGRG4 PE=2 SV | 1689 | KSTGAIsSIPK | 0.131 | -0.203 | -0.81 | -0.294 |
| sp\|Q8IZF6\|AGRG4_HUMAN Adhesion G-protein coupled receptor G4 OS=Homo sapiens GN=ADGRG4 PE=2 SV | 1690 | STGAISsIPKT | 0.139 | 0.131 | -0.79 | -0.173 |

| | | | | | | | |
|---|---|---|---|---|---|---|---|
| sp\|Q8IZF6\|AGRG4_HUMAN Adhesion G-protein coupled receptor G4 OS=Homo sapiens GN=ADGRG4 PE=2 S' | 1694 | ISSIPKtTFSP | 0.082 | -0.261 | -0.917 | -0.365 |
| sp\|Q8IZF6\|AGRG4_HUMAN Adhesion G-protein coupled receptor G4 OS=Homo sapiens GN=ADGRG4 PE=2 S' | 1695 | SSIPKTtFSPF | 0.046 | -0.312 | -1.44 | -0.569 |
| sp\|Q8IZF6\|AGRG4_HUMAN Adhesion G-protein coupled receptor G4 OS=Homo sapiens GN=ADGRG4 PE=2 S' | 1697 | IPKTTFsPFLS | 0.087 | -0.284 | -1.091 | -0.429 |
| sp\|Q8IZF6\|AGRG4_HUMAN Adhesion G-protein coupled receptor G4 OS=Homo sapiens GN=ADGRG4 PE=2 S' | 1701 | TFSPFLsATQQ | 0.117 | -0.241 | -1.226 | -0.45 |
| sp\|Q8IZF6\|AGRG4_HUMAN Adhesion G-protein coupled receptor G4 OS=Homo sapiens GN=ADGRG4 PE=2 S' | 1703 | SPFLSAtQQSS | 0.138 | 0.019 | -1.219 | -0.354 |
| sp\|Q8IZF6\|AGRG4_HUMAN Adhesion G-protein coupled receptor G4 OS=Homo sapiens GN=ADGRG4 PE=2 S' | 1706 | LSATQQsSQAD | 0.168 | -0.059 | -0.66 | -0.184 |
| sp\|Q8IZF6\|AGRG4_HUMAN Adhesion G-protein coupled receptor G4 OS=Homo sapiens GN=ADGRG4 PE=2 S' | 1707 | SATQQSsQADE | 0.073 | -0.303 | -1.574 | -0.601 |
| sp\|Q8IZF6\|AGRG4_HUMAN Adhesion G-protein coupled receptor G4 OS=Homo sapiens GN=ADGRG4 PE=2 S' | 1713 | SQADEAtTLGI | 0.134 | -0.146 | -1.049 | -0.354 |
| sp\|Q8IZF6\|AGRG4_HUMAN Adhesion G-protein coupled receptor G4 OS=Homo sapiens GN=ADGRG4 PE=2 S' | 1714 | QADEATtLGIL | 0.382 | 0.103 | -0.317 | 0.056 |
| sp\|Q8IZF6\|AGRG4_HUMAN Adhesion G-protein coupled receptor G4 OS=Homo sapiens GN=ADGRG4 PE=2 S' | 1719 | TTLGILsGITN | 0.066 | -0.203 | -1.002 | -0.38 |
| sp\|Q8IZF6\|AGRG4_HUMAN Adhesion G-protein coupled receptor G4 OS=Homo sapiens GN=ADGRG4 PE=2 S' | 1722 | GILSGItNRSL | 0.106 | -0.156 | -1.154 | -0.401 |
| sp\|Q8IZF6\|AGRG4_HUMAN Adhesion G-protein coupled receptor G4 OS=Homo sapiens GN=ADGRG4 PE=2 S' | 1725 | SGITNRsLSTV | 0.126 | -0.122 | -1.27 | -0.422 |
| sp\|Q8IZF6\|AGRG4_HUMAN Adhesion G-protein coupled receptor G4 OS=Homo sapiens GN=ADGRG4 PE=2 S' | 1727 | ITNRSLsTVNS | 0.643 | 0.97 | 0.167 | 0.593 |
| sp\|Q8IZF6\|AGRG4_HUMAN Adhesion G-protein coupled receptor G4 OS=Homo sapiens GN=ADGRG4 PE=2 S' | 1728 | TNRSLStVNSG | 0.098 | -0.078 | -1.033 | -0.338 |
| sp\|Q8IZF6\|AGRG4_HUMAN Adhesion G-protein coupled receptor G4 OS=Homo sapiens GN=ADGRG4 PE=2 S' | 1731 | SLSTVNsGTGV | 0.28 | -0.117 | -1.111 | -0.316 |
| sp\|Q8IZF6\|AGRG4_HUMAN Adhesion G-protein coupled receptor G4 OS=Homo sapiens GN=ADGRG4 PE=2 S' | 1733 | STVNSGtGVAL | 0.133 | 0.069 | -0.751 | -0.183 |
| sp\|Q8IZF6\|AGRG4_HUMAN Adhesion G-protein coupled receptor G4 OS=Homo sapiens GN=ADGRG4 PE=2 S' | 1738 | GTGVALtDTYS | 0.155 | -0.079 | -1.333 | -0.419 |
| sp\|Q8IZF6\|AGRG4_HUMAN Adhesion G-protein coupled receptor G4 OS=Homo sapiens GN=ADGRG4 PE=2 S' | 1740 | GVALTDtYSRI | 0.109 | -0.173 | -1.258 | -0.441 |
| sp\|Q8IZF6\|AGRG4_HUMAN Adhesion G-protein coupled receptor G4 OS=Homo sapiens GN=ADGRG4 PE=2 S' | 1742 | ALTDTYsRITV | 0.172 | 0.04 | -0.882 | -0.223 |
| sp\|Q8IZF6\|AGRG4_HUMAN Adhesion G-protein coupled receptor G4 OS=Homo sapiens GN=ADGRG4 PE=2 S' | 1745 | DTYSRItVPEN | 0.795 | 0.573 | 0.15 | 0.506 |
| sp\|Q8IZF6\|AGRG4_HUMAN Adhesion G-protein coupled receptor G4 OS=Homo sapiens GN=ADGRG4 PE=2 S' | 1752 | VPENMLsPTHA | 0.038 | -0.632 | -1.445 | -0.68 |
| sp\|Q8IZF6\|AGRG4_HUMAN Adhesion G-protein coupled receptor G4 OS=Homo sapiens GN=ADGRG4 PE=2 S' | 1754 | ENMLSPtHADS | 0.219 | 0.049 | -0.568 | -0.1 |
| sp\|Q8IZF6\|AGRG4_HUMAN Adhesion G-protein coupled receptor G4 OS=Homo sapiens GN=ADGRG4 PE=2 S' | 1758 | SPTHADsLHTS | 0.256 | -0.093 | -0.827 | -0.221 |
| sp\|Q8IZF6\|AGRG4_HUMAN Adhesion G-protein coupled receptor G4 OS=Homo sapiens GN=ADGRG4 PE=2 S' | 1761 | HADSLHtSFNI | 0.18 | 0.037 | -0.936 | -0.24 |
| sp\|Q8IZF6\|AGRG4_HUMAN Adhesion G-protein coupled receptor G4 OS=Homo sapiens GN=ADGRG4 PE=2 S' | 1762 | ADSLHTsFNIQ | 0.256 | -0.092 | -0.76 | -0.199 |
| sp\|Q8IZF6\|AGRG4_HUMAN Adhesion G-protein coupled receptor G4 OS=Homo sapiens GN=ADGRG4 PE=2 S' | 1768 | SFNIQVsPSLT | 0.037 | -0.467 | -1.766 | -0.732 |
| sp\|Q8IZF6\|AGRG4_HUMAN Adhesion G-protein coupled receptor G4 OS=Homo sapiens GN=ADGRG4 PE=2 S' | 1770 | NIQVSPsLTSF | 0.335 | 0.245 | -0.252 | 0.109 |
| sp\|Q8IZF6\|AGRG4_HUMAN Adhesion G-protein coupled receptor G4 OS=Homo sapiens GN=ADGRG4 PE=2 S' | 1772 | QVSPSLtSFKS | 0.085 | -0.045 | -0.97 | -0.31 |
| sp\|Q8IZF6\|AGRG4_HUMAN Adhesion G-protein coupled receptor G4 OS=Homo sapiens GN=ADGRG4 PE=2 S' | 1773 | VSPSLTsFKSA | 0.057 | -0.321 | -1.318 | -0.527 |
| sp\|Q8IZF6\|AGRG4_HUMAN Adhesion G-protein coupled receptor G4 OS=Homo sapiens GN=ADGRG4 PE=2 S' | 1776 | SLTSFKsASGP | 0.139 | -0.139 | -1.039 | -0.346 |
| sp\|Q8IZF6\|AGRG4_HUMAN Adhesion G-protein coupled receptor G4 OS=Homo sapiens GN=ADGRG4 PE=2 S' | 1778 | TSFKSAsGPTK | 0.799 | 0.573 | 0.526 | 0.633 |
| sp\|Q8IZF6\|AGRG4_HUMAN Adhesion G-protein coupled receptor G4 OS=Homo sapiens GN=ADGRG4 PE=2 S' | 1781 | KSASGPtKNVK | 0.095 | -0.322 | -1.047 | -0.425 |
| sp\|Q8IZF6\|AGRG4_HUMAN Adhesion G-protein coupled receptor G4 OS=Homo sapiens GN=ADGRG4 PE=2 S' | 1786 | PTKNVKtTTNC | 0.344 | -0.014 | -0.701 | -0.124 |
| sp\|Q8IZF6\|AGRG4_HUMAN Adhesion G-protein coupled receptor G4 OS=Homo sapiens GN=ADGRG4 PE=2 S' | 1787 | TKNVKTtTNCF | 0.116 | -0.05 | -0.964 | -0.299 |
| sp\|Q8IZF6\|AGRG4_HUMAN Adhesion G-protein coupled receptor G4 OS=Homo sapiens GN=ADGRG4 PE=2 S' | 1788 | KNVKTTtNCFS | 0.057 | -0.31 | -1.561 | -0.605 |
| sp\|Q8IZF6\|AGRG4_HUMAN Adhesion G-protein coupled receptor G4 OS=Homo sapiens GN=ADGRG4 PE=2 S' | 1792 | TTTNCFsSNTR | 0.265 | -0.044 | -0.422 | -0.067 |
| sp\|Q8IZF6\|AGRG4_HUMAN Adhesion G-protein coupled receptor G4 OS=Homo sapiens GN=ADGRG4 PE=2 S' | 1793 | TTNCFSsNTRK | 0.123 | -0.386 | -1.783 | -0.682 |
| sp\|Q8IZF6\|AGRG4_HUMAN Adhesion G-protein coupled receptor G4 OS=Homo sapiens GN=ADGRG4 PE=2 S' | 1795 | NCFSSNtRKMT | 0.321 | 0.026 | -0.759 | -0.137 |
| sp\|Q8IZF6\|AGRG4_HUMAN Adhesion G-protein coupled receptor G4 OS=Homo sapiens GN=ADGRG4 PE=2 S' | 1799 | SNTRKMtSLLE | 0.282 | 0.695 | -0.3 | 0.226 |
| sp\|Q8IZF6\|AGRG4_HUMAN Adhesion G-protein coupled receptor G4 OS=Homo sapiens GN=ADGRG4 PE=2 S' | 1800 | NTRKMTsLLEK | 0.722 | 0.238 | 0.131 | 0.364 |
| sp\|Q8IZF6\|AGRG4_HUMAN Adhesion G-protein coupled receptor G4 OS=Homo sapiens GN=ADGRG4 PE=2 S' | 1805 | TSLLEKtSLTN | 0.114 | -0.187 | -1.177 | -0.417 |
| sp\|Q8IZF6\|AGRG4_HUMAN Adhesion G-protein coupled receptor G4 OS=Homo sapiens GN=ADGRG4 PE=2 S' | 1806 | SLLEKTsLTNY | 0.301 | 0.087 | -0.725 | -0.112 |
| sp\|Q8IZF6\|AGRG4_HUMAN Adhesion G-protein coupled receptor G4 OS=Homo sapiens GN=ADGRG4 PE=2 S' | 1808 | LEKTSLtNYAT | 0.223 | 0.108 | -0.566 | -0.078 |
| sp\|Q8IZF6\|AGRG4_HUMAN Adhesion G-protein coupled receptor G4 OS=Homo sapiens GN=ADGRG4 PE=2 S' | 1812 | SLTNYAtSLNT | 0.134 | -0.027 | -1.036 | -0.31 |
| sp\|Q8IZF6\|AGRG4_HUMAN Adhesion G-protein coupled receptor G4 OS=Homo sapiens GN=ADGRG4 PE=2 S' | 1813 | LTNYATsLNTP | 0.39 | 0.066 | -0.193 | 0.088 |
| sp\|Q8IZF6\|AGRG4_HUMAN Adhesion G-protein coupled receptor G4 OS=Homo sapiens GN=ADGRG4 PE=2 S' | 1816 | YATSLNtPVSY | 0.049 | -0.428 | -1.677 | -0.685 |
| sp\|Q8IZF6\|AGRG4_HUMAN Adhesion G-protein coupled receptor G4 OS=Homo sapiens GN=ADGRG4 PE=2 S' | 1819 | SLNTPVsYPPW | 0.424 | 0.297 | -0.336 | 0.128 |
| sp\|Q8IZF6\|AGRG4_HUMAN Adhesion G-protein coupled receptor G4 OS=Homo sapiens GN=ADGRG4 PE=2 S' | 1824 | VSYPPWtPSSA | 0.021 | -0.745 | -1.853 | -0.859 |
| sp\|Q8IZF6\|AGRG4_HUMAN Adhesion G-protein coupled receptor G4 OS=Homo sapiens GN=ADGRG4 PE=2 S' | 1826 | YPPWTPsSATL | 0.055 | -0.27 | -1.313 | -0.509 |

| | | | | | | |
|---|---|---|---|---|---|---|
| sp\|Q8IZF6\|AGRG4_HUMAN Adhesion G-protein coupled receptor G4 OS=Homo sapiens GN=ADGRG4 PE=2 S' | 1827 | PPWTPSsATLP | 0.07 | -0.139 | -1.402 | -0.49 |
| sp\|Q8IZF6\|AGRG4_HUMAN Adhesion G-protein coupled receptor G4 OS=Homo sapiens GN=ADGRG4 PE=2 S' | 1829 | WTPSSAtLPSL | 0.725 | 0.638 | 0.464 | 0.609 |
| sp\|Q8IZF6\|AGRG4_HUMAN Adhesion G-protein coupled receptor G4 OS=Homo sapiens GN=ADGRG4 PE=2 S' | 1832 | SSATLPsLTSF | 0.083 | -0.281 | -1.26 | -0.486 |
| sp\|Q8IZF6\|AGRG4_HUMAN Adhesion G-protein coupled receptor G4 OS=Homo sapiens GN=ADGRG4 PE=2 S' | 1834 | ATLPSLtSFVY | 0.259 | 0.061 | -0.673 | -0.118 |
| sp\|Q8IZF6\|AGRG4_HUMAN Adhesion G-protein coupled receptor G4 OS=Homo sapiens GN=ADGRG4 PE=2 S' | 1835 | TLPSLTsFVYS | 0.111 | -0.124 | -1.15 | -0.388 |
| sp\|Q8IZF6\|AGRG4_HUMAN Adhesion G-protein coupled receptor G4 OS=Homo sapiens GN=ADGRG4 PE=2 S' | 1839 | LTSFVYsPHST | 0.068 | -0.368 | -1.329 | -0.543 |
| sp\|Q8IZF6\|AGRG4_HUMAN Adhesion G-protein coupled receptor G4 OS=Homo sapiens GN=ADGRG4 PE=2 S' | 1842 | FVYSPHsTEAE | 0.155 | 0.037 | -0.748 | -0.185 |
| sp\|Q8IZF6\|AGRG4_HUMAN Adhesion G-protein coupled receptor G4 OS=Homo sapiens GN=ADGRG4 PE=2 S' | 1843 | VYSPHStEAEI | 0.247 | -0.029 | -0.66 | -0.147 |
| sp\|Q8IZF6\|AGRG4_HUMAN Adhesion G-protein coupled receptor G4 OS=Homo sapiens GN=ADGRG4 PE=2 S' | 1848 | STEAEIsTPKT | 0.348 | 0.19 | -0.644 | -0.035 |
| sp\|Q8IZF6\|AGRG4_HUMAN Adhesion G-protein coupled receptor G4 OS=Homo sapiens GN=ADGRG4 PE=2 S' | 1849 | TEAEIStPKTS | 0.022 | -0.555 | -1.776 | -0.77 |
| sp\|Q8IZF6\|AGRG4_HUMAN Adhesion G-protein coupled receptor G4 OS=Homo sapiens GN=ADGRG4 PE=2 S' | 1852 | EISTPKtSPPP | 0.326 | 0.291 | -0.192 | 0.142 |
| sp\|Q8IZF6\|AGRG4_HUMAN Adhesion G-protein coupled receptor G4 OS=Homo sapiens GN=ADGRG4 PE=2 S' | 1853 | ISTPKTsPPPT | 0.172 | -0.103 | -0.593 | -0.175 |
| sp\|Q8IZF6\|AGRG4_HUMAN Adhesion G-protein coupled receptor G4 OS=Homo sapiens GN=ADGRG4 PE=2 S' | 1857 | KTSPPPtSQMV | 0.079 | -0.413 | -1.561 | -0.632 |
| sp\|Q8IZF6\|AGRG4_HUMAN Adhesion G-protein coupled receptor G4 OS=Homo sapiens GN=ADGRG4 PE=2 S' | 1858 | TSPPPTsQMVE | 0.054 | -0.389 | -1.543 | -0.626 |
| sp\|Q8IZF6\|AGRG4_HUMAN Adhesion G-protein coupled receptor G4 OS=Homo sapiens GN=ADGRG4 PE=2 S' | 1868 | EFPVLGtRMTS | 0.029 | -0.377 | -1.851 | -0.733 |
| sp\|Q8IZF6\|AGRG4_HUMAN Adhesion G-protein coupled receptor G4 OS=Homo sapiens GN=ADGRG4 PE=2 S' | 1871 | VLGTRMtSSNT | 0.246 | 0.155 | -0.796 | -0.132 |
| sp\|Q8IZF6\|AGRG4_HUMAN Adhesion G-protein coupled receptor G4 OS=Homo sapiens GN=ADGRG4 PE=2 S' | 1872 | LGTRMTsSNTQ | 0.543 | 0.683 | 0.385 | 0.537 |
| sp\|Q8IZF6\|AGRG4_HUMAN Adhesion G-protein coupled receptor G4 OS=Homo sapiens GN=ADGRG4 PE=2 S' | 1873 | GTRMTSsNTQP | 0.241 | 0.004 | -0.955 | -0.237 |
| sp\|Q8IZF6\|AGRG4_HUMAN Adhesion G-protein coupled receptor G4 OS=Homo sapiens GN=ADGRG4 PE=2 S' | 1875 | RMTSSNtQPLL | 0.751 | 0.835 | 0.299 | 0.628 |
| sp\|Q8IZF6\|AGRG4_HUMAN Adhesion G-protein coupled receptor G4 OS=Homo sapiens GN=ADGRG4 PE=2 S' | 1881 | TQPLLMtSWNI | 0.173 | 0.05 | -0.975 | -0.251 |
| sp\|Q8IZF6\|AGRG4_HUMAN Adhesion G-protein coupled receptor G4 OS=Homo sapiens GN=ADGRG4 PE=2 S' | 1882 | QPLLMTsWNIP | 0.144 | -0.111 | -0.853 | -0.273 |
| sp\|Q8IZF6\|AGRG4_HUMAN Adhesion G-protein coupled receptor G4 OS=Homo sapiens GN=ADGRG4 PE=2 S' | 1887 | TSWNIPtAEGS | 0.067 | -0.128 | -1.056 | -0.372 |
| sp\|Q8IZF6\|AGRG4_HUMAN Adhesion G-protein coupled receptor G4 OS=Homo sapiens GN=ADGRG4 PE=2 S' | 1891 | IPTAEGsQFPI | 0.156 | -0.272 | -1.114 | -0.41 |
| sp\|Q8IZF6\|AGRG4_HUMAN Adhesion G-protein coupled receptor G4 OS=Homo sapiens GN=ADGRG4 PE=2 S' | 1896 | GSQFPIsTTIN | 0.337 | -0.076 | -0.723 | -0.154 |
| sp\|Q8IZF6\|AGRG4_HUMAN Adhesion G-protein coupled receptor G4 OS=Homo sapiens GN=ADGRG4 PE=2 S' | 1897 | SQFPIStTINV | 0.048 | -0.2 | -1.488 | -0.547 |
| sp\|Q8IZF6\|AGRG4_HUMAN Adhesion G-protein coupled receptor G4 OS=Homo sapiens GN=ADGRG4 PE=2 S' | 1898 | QFPISTtINVP | 0.177 | 0.097 | -0.435 | -0.054 |
| sp\|Q8IZF6\|AGRG4_HUMAN Adhesion G-protein coupled receptor G4 OS=Homo sapiens GN=ADGRG4 PE=2 S' | 1903 | TTINVPtSNEM | 0.15 | -0.148 | -0.912 | -0.303 |
| sp\|Q8IZF6\|AGRG4_HUMAN Adhesion G-protein coupled receptor G4 OS=Homo sapiens GN=ADGRG4 PE=2 S' | 1904 | TINVPTsNEME | 0.061 | -0.27 | -1.541 | -0.583 |
| sp\|Q8IZF6\|AGRG4_HUMAN Adhesion G-protein coupled receptor G4 OS=Homo sapiens GN=ADGRG4 PE=2 S' | 1909 | TSNEMEtETLH | 0.071 | -0.318 | -1.642 | -0.63 |
| sp\|Q8IZF6\|AGRG4_HUMAN Adhesion G-protein coupled receptor G4 OS=Homo sapiens GN=ADGRG4 PE=2 S' | 1911 | NEMETEtLHLV | 0.161 | 0.076 | -1.135 | -0.299 |
| sp\|Q8IZF6\|AGRG4_HUMAN Adhesion G-protein coupled receptor G4 OS=Homo sapiens GN=ADGRG4 PE=2 S' | 1920 | LVPGPLsTFTA | 0.048 | -0.292 | -1.183 | -0.476 |
| sp\|Q8IZF6\|AGRG4_HUMAN Adhesion G-protein coupled receptor G4 OS=Homo sapiens GN=ADGRG4 PE=2 S' | 1921 | VPGPLStFTAS | 0.076 | -0.205 | -1.244 | -0.458 |
| sp\|Q8IZF6\|AGRG4_HUMAN Adhesion G-protein coupled receptor G4 OS=Homo sapiens GN=ADGRG4 PE=2 S' | 1923 | GPLSTFtASQT | 0.094 | -0.122 | -1.156 | -0.395 |
| sp\|Q8IZF6\|AGRG4_HUMAN Adhesion G-protein coupled receptor G4 OS=Homo sapiens GN=ADGRG4 PE=2 S' | 1925 | LSTFTAsQTGL | 0.199 | -0.077 | -0.847 | -0.242 |
| sp\|Q8IZF6\|AGRG4_HUMAN Adhesion G-protein coupled receptor G4 OS=Homo sapiens GN=ADGRG4 PE=2 S' | 1927 | TFTASQtGLVS | 0.205 | 0.088 | -0.476 | -0.061 |
| sp\|Q8IZF6\|AGRG4_HUMAN Adhesion G-protein coupled receptor G4 OS=Homo sapiens GN=ADGRG4 PE=2 S' | 1931 | SQTGLVsKDVM | 0.155 | -0.099 | -0.704 | -0.216 |
| sp\|Q8IZF6\|AGRG4_HUMAN Adhesion G-protein coupled receptor G4 OS=Homo sapiens GN=ADGRG4 PE=2 S' | 1938 | KDVMAMsSIPM | 0.122 | -0.113 | -0.866 | -0.286 |
| sp\|Q8IZF6\|AGRG4_HUMAN Adhesion G-protein coupled receptor G4 OS=Homo sapiens GN=ADGRG4 PE=2 S' | 1939 | DVMAMSsIPMS | 0.262 | 0.202 | -0.597 | -0.044 |
| sp\|Q8IZF6\|AGRG4_HUMAN Adhesion G-protein coupled receptor G4 OS=Homo sapiens GN=ADGRG4 PE=2 S' | 1943 | MSSIPMsGILP | 0.103 | -0.082 | -0.805 | -0.261 |
| sp\|Q8IZF6\|AGRG4_HUMAN Adhesion G-protein coupled receptor G4 OS=Homo sapiens GN=ADGRG4 PE=2 S' | 1952 | LPNHGLsENPS | 0.051 | -0.217 | -1.308 | -0.491 |
| sp\|Q8IZF6\|AGRG4_HUMAN Adhesion G-protein coupled receptor G4 OS=Homo sapiens GN=ADGRG4 PE=2 S' | 1956 | GLSENPsLSTS | 0.257 | -0.033 | -0.636 | -0.137 |
| sp\|Q8IZF6\|AGRG4_HUMAN Adhesion G-protein coupled receptor G4 OS=Homo sapiens GN=ADGRG4 PE=2 S' | 1958 | SENPSLsTSLR | 0.081 | -0.075 | -1.295 | -0.43 |
| sp\|Q8IZF6\|AGRG4_HUMAN Adhesion G-protein coupled receptor G4 OS=Homo sapiens GN=ADGRG4 PE=2 S' | 1959 | ENPSLStSLRA | 0.02 | -0.546 | -2.102 | -0.876 |
| sp\|Q8IZF6\|AGRG4_HUMAN Adhesion G-protein coupled receptor G4 OS=Homo sapiens GN=ADGRG4 PE=2 S' | 1960 | NPSLSTsLRAI | 0.659 | 0.287 | 0.398 | 0.448 |
| sp\|Q8IZF6\|AGRG4_HUMAN Adhesion G-protein coupled receptor G4 OS=Homo sapiens GN=ADGRG4 PE=2 S' | 1965 | TSLRAItSTLA | 0.693 | 0.76 | 0.375 | 0.609 |
| sp\|Q8IZF6\|AGRG4_HUMAN Adhesion G-protein coupled receptor G4 OS=Homo sapiens GN=ADGRG4 PE=2 S' | 1966 | SLRAITsTLAD | 0.439 | 0.271 | -0.111 | 0.2 |
| sp\|Q8IZF6\|AGRG4_HUMAN Adhesion G-protein coupled receptor G4 OS=Homo sapiens GN=ADGRG4 PE=2 S' | 1967 | LRAITStLADV | 0.228 | 0.258 | -0.531 | -0.015 |
| sp\|Q8IZF6\|AGRG4_HUMAN Adhesion G-protein coupled receptor G4 OS=Homo sapiens GN=ADGRG4 PE=2 S' | 1974 | LADVKHtFEKM | 0.11 | -0.053 | -1.041 | -0.328 |
| sp\|Q8IZF6\|AGRG4_HUMAN Adhesion G-protein coupled receptor G4 OS=Homo sapiens GN=ADGRG4 PE=2 S' | 1979 | HTFEKMtTSVT | 0.077 | -0.029 | -1.216 | -0.389 |

| | | | | | | | |
|---|---|---|---|---|---|---|---|
| sp\|Q8IZF6\|AGRG4_HUMAN Adhesion G-protein coupled receptor G4 OS=Homo sapiens GN=ADGRG4 PE=2 S' | 1980 | TFEKMTtSVTP | 0.182 | -0.186 | -0.73 | -0.245 |
| sp\|Q8IZF6\|AGRG4_HUMAN Adhesion G-protein coupled receptor G4 OS=Homo sapiens GN=ADGRG4 PE=2 S' | 1981 | FEKMTTsVTPG | 0.237 | -0.005 | -0.672 | -0.147 |
| sp\|Q8IZF6\|AGRG4_HUMAN Adhesion G-protein coupled receptor G4 OS=Homo sapiens GN=ADGRG4 PE=2 S' | 1983 | KMTTSVtPGTT | 0.241 | -0.068 | -0.58 | -0.136 |
| sp\|Q8IZF6\|AGRG4_HUMAN Adhesion G-protein coupled receptor G4 OS=Homo sapiens GN=ADGRG4 PE=2 S' | 1986 | TSVTPGtTLPS | 0.036 | -0.454 | -1.829 | -0.749 |
| sp\|Q8IZF6\|AGRG4_HUMAN Adhesion G-protein coupled receptor G4 OS=Homo sapiens GN=ADGRG4 PE=2 S' | 1987 | SVTPGTtLPSI | 0.36 | 0.403 | 0.01 | 0.258 |
| sp\|Q8IZF6\|AGRG4_HUMAN Adhesion G-protein coupled receptor G4 OS=Homo sapiens GN=ADGRG4 PE=2 S' | 1990 | PGTTLPsILSG | 0.058 | -0.273 | -1.361 | -0.525 |
| sp\|Q8IZF6\|AGRG4_HUMAN Adhesion G-protein coupled receptor G4 OS=Homo sapiens GN=ADGRG4 PE=2 S' | 1993 | TLPSILsGATS | 0.048 | -0.298 | -1.338 | -0.529 |
| sp\|Q8IZF6\|AGRG4_HUMAN Adhesion G-protein coupled receptor G4 OS=Homo sapiens GN=ADGRG4 PE=2 S' | 1996 | SILSGAtSGSV | 0.079 | -0.15 | -1.499 | -0.523 |
| sp\|Q8IZF6\|AGRG4_HUMAN Adhesion G-protein coupled receptor G4 OS=Homo sapiens GN=ADGRG4 PE=2 S' | 1997 | ILSGATsGSVI | 0.333 | 0.07 | -0.233 | 0.057 |
| sp\|Q8IZF6\|AGRG4_HUMAN Adhesion G-protein coupled receptor G4 OS=Homo sapiens GN=ADGRG4 PE=2 S' | 1999 | SGATSGsVISK | 0.099 | -0.082 | -0.914 | -0.299 |
| sp\|Q8IZF6\|AGRG4_HUMAN Adhesion G-protein coupled receptor G4 OS=Homo sapiens GN=ADGRG4 PE=2 S' | 2002 | TSGSVIsKSPI | 0.106 | -0.292 | -1.118 | -0.435 |
| sp\|Q8IZF6\|AGRG4_HUMAN Adhesion G-protein coupled receptor G4 OS=Homo sapiens GN=ADGRG4 PE=2 S' | 2004 | GSVISKsPILT | 0.172 | -0.087 | -0.553 | -0.156 |
| sp\|Q8IZF6\|AGRG4_HUMAN Adhesion G-protein coupled receptor G4 OS=Homo sapiens GN=ADGRG4 PE=2 S' | 2008 | SKSPILtWLLS | 0.067 | -0.105 | -1.205 | -0.414 |
| sp\|Q8IZF6\|AGRG4_HUMAN Adhesion G-protein coupled receptor G4 OS=Homo sapiens GN=ADGRG4 PE=2 S' | 2012 | ILTWLLsSLPS | 0.096 | -0.206 | -1.102 | -0.404 |
| sp\|Q8IZF6\|AGRG4_HUMAN Adhesion G-protein coupled receptor G4 OS=Homo sapiens GN=ADGRG4 PE=2 S' | 2013 | LTWLLSsLPSG | 0.339 | 0.484 | -0.319 | 0.168 |
| sp\|Q8IZF6\|AGRG4_HUMAN Adhesion G-protein coupled receptor G4 OS=Homo sapiens GN=ADGRG4 PE=2 S' | 2016 | LLSSLPsGSPP | 0.045 | -0.388 | -1.496 | -0.613 |
| sp\|Q8IZF6\|AGRG4_HUMAN Adhesion G-protein coupled receptor G4 OS=Homo sapiens GN=ADGRG4 PE=2 S' | 2018 | SSLPSGsPPAT | 0.252 | 0.152 | -0.39 | 0.005 |
| sp\|Q8IZF6\|AGRG4_HUMAN Adhesion G-protein coupled receptor G4 OS=Homo sapiens GN=ADGRG4 PE=2 S' | 2022 | SGSPPAtVSNA | 0.078 | -0.281 | -1.306 | -0.503 |
| sp\|Q8IZF6\|AGRG4_HUMAN Adhesion G-protein coupled receptor G4 OS=Homo sapiens GN=ADGRG4 PE=2 S' | 2024 | SPPATVsNAPH | 0.036 | -0.469 | -1.998 | -0.81 |
| sp\|Q8IZF6\|AGRG4_HUMAN Adhesion G-protein coupled receptor G4 OS=Homo sapiens GN=ADGRG4 PE=2 S' | 2031 | NAPHVMtSSTV | 0.1 | -0.166 | -1.444 | -0.503 |
| sp\|Q8IZF6\|AGRG4_HUMAN Adhesion G-protein coupled receptor G4 OS=Homo sapiens GN=ADGRG4 PE=2 S' | 2032 | APHVMTsSTVE | 0.071 | -0.304 | -1.527 | -0.587 |
| sp\|Q8IZF6\|AGRG4_HUMAN Adhesion G-protein coupled receptor G4 OS=Homo sapiens GN=ADGRG4 PE=2 S' | 2033 | PHVMTSsTVEV | 0.085 | -0.109 | -1.601 | -0.542 |
| sp\|Q8IZF6\|AGRG4_HUMAN Adhesion G-protein coupled receptor G4 OS=Homo sapiens GN=ADGRG4 PE=2 S' | 2034 | HVMTSStVEVS | 0.141 | 0.205 | -0.667 | -0.107 |
| sp\|Q8IZF6\|AGRG4_HUMAN Adhesion G-protein coupled receptor G4 OS=Homo sapiens GN=ADGRG4 PE=2 S' | 2038 | SSTVEVsKSTF | 0.066 | -0.331 | -1.403 | -0.556 |
| sp\|Q8IZF6\|AGRG4_HUMAN Adhesion G-protein coupled receptor G4 OS=Homo sapiens GN=ADGRG4 PE=2 S' | 2040 | TVEVSKsTFLT | 0.24 | 0.138 | -0.267 | 0.037 |
| sp\|Q8IZF6\|AGRG4_HUMAN Adhesion G-protein coupled receptor G4 OS=Homo sapiens GN=ADGRG4 PE=2 S' | 2041 | VEVSKStFLTS | 0.049 | -0.292 | -1.632 | -0.625 |
| sp\|Q8IZF6\|AGRG4_HUMAN Adhesion G-protein coupled receptor G4 OS=Homo sapiens GN=ADGRG4 PE=2 S' | 2044 | SKSTFLtSDMI | 0.173 | -0.14 | -1.036 | -0.334 |
| sp\|Q8IZF6\|AGRG4_HUMAN Adhesion G-protein coupled receptor G4 OS=Homo sapiens GN=ADGRG4 PE=2 S' | 2045 | KSTFLTsDMIS | 0.179 | -0.134 | -1.105 | -0.353 |
| sp\|Q8IZF6\|AGRG4_HUMAN Adhesion G-protein coupled receptor G4 OS=Homo sapiens GN=ADGRG4 PE=2 S' | 2049 | LTSDMIsAHPF | 0.189 | -0.17 | -1.028 | -0.336 |
| sp\|Q8IZF6\|AGRG4_HUMAN Adhesion G-protein coupled receptor G4 OS=Homo sapiens GN=ADGRG4 PE=2 S' | 2054 | ISAHPFtNLTT | 0.087 | -0.318 | -1.045 | -0.425 |
| sp\|Q8IZF6\|AGRG4_HUMAN Adhesion G-protein coupled receptor G4 OS=Homo sapiens GN=ADGRG4 PE=2 S' | 2057 | HPFTNLtTLPS | 0.054 | -0.202 | -1.396 | -0.515 |
| sp\|Q8IZF6\|AGRG4_HUMAN Adhesion G-protein coupled receptor G4 OS=Homo sapiens GN=ADGRG4 PE=2 S' | 2058 | PFTNLTtLPSA | 0.561 | 0.496 | 0.444 | 0.5 |
| sp\|Q8IZF6\|AGRG4_HUMAN Adhesion G-protein coupled receptor G4 OS=Homo sapiens GN=ADGRG4 PE=2 S' | 2061 | NLTTLPsATMS | 0.196 | -0.122 | -1.133 | -0.353 |
| sp\|Q8IZF6\|AGRG4_HUMAN Adhesion G-protein coupled receptor G4 OS=Homo sapiens GN=ADGRG4 PE=2 S' | 2063 | TTLPSAtMSTI | 0.359 | 0.183 | -0.394 | 0.049 |
| sp\|Q8IZF6\|AGRG4_HUMAN Adhesion G-protein coupled receptor G4 OS=Homo sapiens GN=ADGRG4 PE=2 S' | 2065 | LPSATMsTILT | 0.097 | -0.039 | -0.864 | -0.269 |
| sp\|Q8IZF6\|AGRG4_HUMAN Adhesion G-protein coupled receptor G4 OS=Homo sapiens GN=ADGRG4 PE=2 S' | 2066 | PSATMStILTR | 0.076 | -0.317 | -1.124 | -0.455 |
| sp\|Q8IZF6\|AGRG4_HUMAN Adhesion G-protein coupled receptor G4 OS=Homo sapiens GN=ADGRG4 PE=2 S' | 2069 | TMSTILtRTIP | 0.071 | -0.232 | -1.429 | -0.53 |
| sp\|Q8IZF6\|AGRG4_HUMAN Adhesion G-protein coupled receptor G4 OS=Homo sapiens GN=ADGRG4 PE=2 S' | 2071 | STILTRtIPTP | 0.147 | 0.282 | -1.002 | -0.191 |
| sp\|Q8IZF6\|AGRG4_HUMAN Adhesion G-protein coupled receptor G4 OS=Homo sapiens GN=ADGRG4 PE=2 S' | 2074 | LTRTIPtPTLG | 0.218 | -0.186 | -0.832 | -0.267 |
| sp\|Q8IZF6\|AGRG4_HUMAN Adhesion G-protein coupled receptor G4 OS=Homo sapiens GN=ADGRG4 PE=2 S' | 2076 | RTIPTPtLGGI | 0.423 | 0.082 | -0.183 | 0.107 |
| sp\|Q8IZF6\|AGRG4_HUMAN Adhesion G-protein coupled receptor G4 OS=Homo sapiens GN=ADGRG4 PE=2 S' | 2081 | PTLGGItTGFP | 0.097 | -0.221 | -1.105 | -0.41 |
| sp\|Q8IZF6\|AGRG4_HUMAN Adhesion G-protein coupled receptor G4 OS=Homo sapiens GN=ADGRG4 PE=2 S' | 2082 | TLGGITtGFPT | 0.102 | -0.145 | -0.7 | -0.248 |
| sp\|Q8IZF6\|AGRG4_HUMAN Adhesion G-protein coupled receptor G4 OS=Homo sapiens GN=ADGRG4 PE=2 S' | 2086 | ITTGFPtSLPM | 0.097 | -0.286 | -1.19 | -0.46 |
| sp\|Q8IZF6\|AGRG4_HUMAN Adhesion G-protein coupled receptor G4 OS=Homo sapiens GN=ADGRG4 PE=2 S' | 2087 | TTGFPTsLPMS | 0.495 | 0.34 | -0.09 | 0.248 |
| sp\|Q8IZF6\|AGRG4_HUMAN Adhesion G-protein coupled receptor G4 OS=Homo sapiens GN=ADGRG4 PE=2 S' | 2091 | PTSLPMsINVT | 0.194 | -0.052 | -0.644 | -0.167 |
| sp\|Q8IZF6\|AGRG4_HUMAN Adhesion G-protein coupled receptor G4 OS=Homo sapiens GN=ADGRG4 PE=2 S' | 2095 | PMSINVtDDIV | 0.454 | 0.158 | -0.576 | 0.012 |
| sp\|Q8IZF6\|AGRG4_HUMAN Adhesion G-protein coupled receptor G4 OS=Homo sapiens GN=ADGRG4 PE=2 S' | 2102 | DDIVYIsTHPE | 0.106 | -0.228 | -1.344 | -0.489 |
| sp\|Q8IZF6\|AGRG4_HUMAN Adhesion G-protein coupled receptor G4 OS=Homo sapiens GN=ADGRG4 PE=2 S' | 2103 | DIVYIStHPEA | 0.643 | 0.501 | 0.193 | 0.446 |
| sp\|Q8IZF6\|AGRG4_HUMAN Adhesion G-protein coupled receptor G4 OS=Homo sapiens GN=ADGRG4 PE=2 S' | 2108 | STHPEAsSRTT | 0.064 | -0.398 | -1.68 | -0.671 |

| | | | | | | | |
|---|---|---|---|---|---|---|---|
| sp\|Q8IZF6\|AGRG4_HUMAN Adhesion G-protein coupled receptor G4 OS=Homo sapiens GN=ADGRG4 PE=2 S\ | 2109 | THPEASsRTTI | 0.094 | -0.228 | -1.514 | -0.549 |
| sp\|Q8IZF6\|AGRG4_HUMAN Adhesion G-protein coupled receptor G4 OS=Homo sapiens GN=ADGRG4 PE=2 S\ | 2111 | PEASSRtTITA | 0.195 | 0.037 | -0.642 | -0.137 |
| sp\|Q8IZF6\|AGRG4_HUMAN Adhesion G-protein coupled receptor G4 OS=Homo sapiens GN=ADGRG4 PE=2 S\ | 2112 | EASSRTtITAN | 0.24 | -0.002 | -0.615 | -0.126 |
| sp\|Q8IZF6\|AGRG4_HUMAN Adhesion G-protein coupled receptor G4 OS=Homo sapiens GN=ADGRG4 PE=2 S\ | 2114 | SSRTTItANPR | 0.257 | 0.117 | -0.683 | -0.103 |
| sp\|Q8IZF6\|AGRG4_HUMAN Adhesion G-protein coupled receptor G4 OS=Homo sapiens GN=ADGRG4 PE=2 S\ | 2119 | ITANPRtVSHP | 0.044 | -0.345 | -1.233 | -0.511 |
| sp\|Q8IZF6\|AGRG4_HUMAN Adhesion G-protein coupled receptor G4 OS=Homo sapiens GN=ADGRG4 PE=2 S\ | 2121 | ANPRTVsHPSS | 0.721 | 1.135 | 0.529 | 0.795 |
| sp\|Q8IZF6\|AGRG4_HUMAN Adhesion G-protein coupled receptor G4 OS=Homo sapiens GN=ADGRG4 PE=2 S\ | 2124 | RTVSHPsSFSR | 0.128 | -0.157 | -1.175 | -0.401 |
| sp\|Q8IZF6\|AGRG4_HUMAN Adhesion G-protein coupled receptor G4 OS=Homo sapiens GN=ADGRG4 PE=2 S\ | 2125 | TVSHPSsFSRK | 0.058 | -0.385 | -1.343 | -0.557 |
| sp\|Q8IZF6\|AGRG4_HUMAN Adhesion G-protein coupled receptor G4 OS=Homo sapiens GN=ADGRG4 PE=2 S\ | 2127 | SHPSSFsRKTM | 0.096 | -0.114 | -1.268 | -0.429 |
| sp\|Q8IZF6\|AGRG4_HUMAN Adhesion G-protein coupled receptor G4 OS=Homo sapiens GN=ADGRG4 PE=2 S\ | 2130 | SSFSRKtMSPS | 0.125 | -0.026 | -1.088 | -0.33 |
| sp\|Q8IZF6\|AGRG4_HUMAN Adhesion G-protein coupled receptor G4 OS=Homo sapiens GN=ADGRG4 PE=2 S\ | 2132 | FSRKTMsPSTT | 0.224 | 0.024 | -0.662 | -0.138 |
| sp\|Q8IZF6\|AGRG4_HUMAN Adhesion G-protein coupled receptor G4 OS=Homo sapiens GN=ADGRG4 PE=2 S\ | 2134 | RKTMSPsTTDH | 0.605 | 0.117 | -0.143 | 0.193 |
| sp\|Q8IZF6\|AGRG4_HUMAN Adhesion G-protein coupled receptor G4 OS=Homo sapiens GN=ADGRG4 PE=2 S\ | 2135 | KTMSPStTDHT | 0.053 | -0.356 | -1.322 | -0.542 |
| sp\|Q8IZF6\|AGRG4_HUMAN Adhesion G-protein coupled receptor G4 OS=Homo sapiens GN=ADGRG4 PE=2 S\ | 2136 | TMSPSTtDHTL | 0.297 | 0.245 | -0.422 | 0.04 |
| sp\|Q8IZF6\|AGRG4_HUMAN Adhesion G-protein coupled receptor G4 OS=Homo sapiens GN=ADGRG4 PE=2 S\ | 2139 | PSTTDHtLSVG | 0.346 | 0.072 | -0.468 | -0.017 |
| sp\|Q8IZF6\|AGRG4_HUMAN Adhesion G-protein coupled receptor G4 OS=Homo sapiens GN=ADGRG4 PE=2 S\ | 2141 | TTDHTLsVGAM | 0.139 | -0.039 | -0.808 | -0.236 |
| sp\|Q8IZF6\|AGRG4_HUMAN Adhesion G-protein coupled receptor G4 OS=Homo sapiens GN=ADGRG4 PE=2 S\ | 2149 | GAMPLPsSTIT | 0.105 | -0.278 | -1.332 | -0.502 |
| sp\|Q8IZF6\|AGRG4_HUMAN Adhesion G-protein coupled receptor G4 OS=Homo sapiens GN=ADGRG4 PE=2 S\ | 2150 | AMPLPSsTITS | 0.028 | -0.259 | -1.647 | -0.626 |
| sp\|Q8IZF6\|AGRG4_HUMAN Adhesion G-protein coupled receptor G4 OS=Homo sapiens GN=ADGRG4 PE=2 S\ | 2151 | MPLPSStITSS | 0.116 | -0.031 | -0.881 | -0.265 |
| sp\|Q8IZF6\|AGRG4_HUMAN Adhesion G-protein coupled receptor G4 OS=Homo sapiens GN=ADGRG4 PE=2 S\ | 2153 | LPSSTItSSWN | 0.054 | -0.217 | -1.363 | -0.509 |
| sp\|Q8IZF6\|AGRG4_HUMAN Adhesion G-protein coupled receptor G4 OS=Homo sapiens GN=ADGRG4 PE=2 S\ | 2154 | PSSTITsSWNR | 0.224 | -0.066 | -0.805 | -0.216 |
| sp\|Q8IZF6\|AGRG4_HUMAN Adhesion G-protein coupled receptor G4 OS=Homo sapiens GN=ADGRG4 PE=2 S\ | 2155 | SSTITSsWNRI | 0.091 | -0.081 | -1.074 | -0.355 |
| sp\|Q8IZF6\|AGRG4_HUMAN Adhesion G-protein coupled receptor G4 OS=Homo sapiens GN=ADGRG4 PE=2 S\ | 2161 | SWNRIPtASSP | 0.156 | 0.604 | -0.716 | 0.015 |
| sp\|Q8IZF6\|AGRG4_HUMAN Adhesion G-protein coupled receptor G4 OS=Homo sapiens GN=ADGRG4 PE=2 S\ | 2163 | NRIPTAsSPST | 0.295 | 0.587 | -0.337 | 0.182 |
| sp\|Q8IZF6\|AGRG4_HUMAN Adhesion G-protein coupled receptor G4 OS=Homo sapiens GN=ADGRG4 PE=2 S\ | 2164 | RIPTASsPSTL | 0.06 | -0.334 | -1.502 | -0.592 |
| sp\|Q8IZF6\|AGRG4_HUMAN Adhesion G-protein coupled receptor G4 OS=Homo sapiens GN=ADGRG4 PE=2 S\ | 2166 | PTASSPsTLII | 0.377 | 0.069 | -0.364 | 0.027 |
| sp\|Q8IZF6\|AGRG4_HUMAN Adhesion G-protein coupled receptor G4 OS=Homo sapiens GN=ADGRG4 PE=2 S\ | 2167 | TASSPStLIIP | 0.076 | -0.333 | -1.095 | -0.451 |
| sp\|Q8IZF6\|AGRG4_HUMAN Adhesion G-protein coupled receptor G4 OS=Homo sapiens GN=ADGRG4 PE=2 S\ | 2174 | LIIPKPtLDSL | 0.156 | 0.02 | -0.616 | -0.147 |
| sp\|Q8IZF6\|AGRG4_HUMAN Adhesion G-protein coupled receptor G4 OS=Homo sapiens GN=ADGRG4 PE=2 S\ | 2177 | PKPTLDsLLNI | 0.34 | 0.022 | -0.753 | -0.13 |
| sp\|Q8IZF6\|AGRG4_HUMAN Adhesion G-protein coupled receptor G4 OS=Homo sapiens GN=ADGRG4 PE=2 S\ | 2183 | SLLNIMtTTST | 0.137 | -0.003 | -0.692 | -0.186 |
| sp\|Q8IZF6\|AGRG4_HUMAN Adhesion G-protein coupled receptor G4 OS=Homo sapiens GN=ADGRG4 PE=2 S\ | 2184 | LLNIMTtTSTV | 0.148 | -0.113 | -1.118 | -0.361 |
| sp\|Q8IZF6\|AGRG4_HUMAN Adhesion G-protein coupled receptor G4 OS=Homo sapiens GN=ADGRG4 PE=2 S\ | 2185 | LNIMTTtSTVP | 0.065 | -0.211 | -1.245 | -0.464 |
| sp\|Q8IZF6\|AGRG4_HUMAN Adhesion G-protein coupled receptor G4 OS=Homo sapiens GN=ADGRG4 PE=2 S\ | 2186 | NIMTTTsTVPG | 0.134 | 0.023 | -0.923 | -0.255 |
| sp\|Q8IZF6\|AGRG4_HUMAN Adhesion G-protein coupled receptor G4 OS=Homo sapiens GN=ADGRG4 PE=2 S\ | 2187 | IMTTTStVPGA | 0.351 | 0.457 | -0.069 | 0.246 |
| sp\|Q8IZF6\|AGRG4_HUMAN Adhesion G-protein coupled receptor G4 OS=Homo sapiens GN=ADGRG4 PE=2 S\ | 2192 | STVPGAsFPLI | 0.54 | 0.442 | -0.071 | 0.304 |
| sp\|Q8IZF6\|AGRG4_HUMAN Adhesion G-protein coupled receptor G4 OS=Homo sapiens GN=ADGRG4 PE=2 S\ | 2197 | ASFPLIsTGVT | 0.125 | -0.215 | -1.109 | -0.4 |
| sp\|Q8IZF6\|AGRG4_HUMAN Adhesion G-protein coupled receptor G4 OS=Homo sapiens GN=ADGRG4 PE=2 S\ | 2198 | SFPLIStGVTY | 0.057 | -0.244 | -1.348 | -0.512 |
| sp\|Q8IZF6\|AGRG4_HUMAN Adhesion G-protein coupled receptor G4 OS=Homo sapiens GN=ADGRG4 PE=2 S\ | 2201 | LISTGVtYPFT | 0.302 | 0.246 | -0.38 | 0.056 |
| sp\|Q8IZF6\|AGRG4_HUMAN Adhesion G-protein coupled receptor G4 OS=Homo sapiens GN=ADGRG4 PE=2 S\ | 2205 | GVTYPFtATVS | 0.216 | 0.042 | -0.68 | -0.141 |
| sp\|Q8IZF6\|AGRG4_HUMAN Adhesion G-protein coupled receptor G4 OS=Homo sapiens GN=ADGRG4 PE=2 S\ | 2207 | TYPFTAtVSSP | 0.063 | -0.149 | -1.468 | -0.518 |
| sp\|Q8IZF6\|AGRG4_HUMAN Adhesion G-protein coupled receptor G4 OS=Homo sapiens GN=ADGRG4 PE=2 S\ | 2209 | PFTATVsSPIS | 0.634 | 0.459 | 0.191 | 0.428 |
| sp\|Q8IZF6\|AGRG4_HUMAN Adhesion G-protein coupled receptor G4 OS=Homo sapiens GN=ADGRG4 PE=2 S\ | 2210 | FTATVSsPISS | 0.033 | -0.445 | -1.7 | -0.704 |
| sp\|Q8IZF6\|AGRG4_HUMAN Adhesion G-protein coupled receptor G4 OS=Homo sapiens GN=ADGRG4 PE=2 S\ | 2213 | TVSSPIsSFFE | 0.061 | -0.383 | -1.362 | -0.561 |
| sp\|Q8IZF6\|AGRG4_HUMAN Adhesion G-protein coupled receptor G4 OS=Homo sapiens GN=ADGRG4 PE=2 S\ | 2214 | VSSPISsFFET | 0.124 | -0.234 | -0.767 | -0.292 |
| sp\|Q8IZF6\|AGRG4_HUMAN Adhesion G-protein coupled receptor G4 OS=Homo sapiens GN=ADGRG4 PE=2 S\ | 2218 | ISSFFEtTWLD | 0.293 | -0.103 | -0.859 | -0.223 |
| sp\|Q8IZF6\|AGRG4_HUMAN Adhesion G-protein coupled receptor G4 OS=Homo sapiens GN=ADGRG4 PE=2 S\ | 2219 | SSFFETtWLDS | 0.12 | -0.067 | -1.276 | -0.408 |
| sp\|Q8IZF6\|AGRG4_HUMAN Adhesion G-protein coupled receptor G4 OS=Homo sapiens GN=ADGRG4 PE=2 S\ | 2223 | ETTWLDsTPSF | 0.229 | 0.19 | -0.652 | -0.078 |
| sp\|Q8IZF6\|AGRG4_HUMAN Adhesion G-protein coupled receptor G4 OS=Homo sapiens GN=ADGRG4 PE=2 S\ | 2224 | TTWLDStPSFL | 0.02 | -0.477 | -2.041 | -0.833 |
| sp\|Q8IZF6\|AGRG4_HUMAN Adhesion G-protein coupled receptor G4 OS=Homo sapiens GN=ADGRG4 PE=2 S\ | 2226 | WLDSTPsFLST | 0.074 | -0.148 | -1.086 | -0.387 |

| | | | | | | |
|---|---|---|---|---|---|---|
| sp\|Q8IZF6\|AGRG4_HUMAN Adhesion G-protein coupled receptor G4 OS=Homo sapiens GN=ADGRG4 PE=2 S' | 2229 | STPSFLsTEAS | 0.044 | -0.291 | -1.773 | -0.673 |
| sp\|Q8IZF6\|AGRG4_HUMAN Adhesion G-protein coupled receptor G4 OS=Homo sapiens GN=ADGRG4 PE=2 S' | 2230 | TPSFLStEAST | 0.037 | -0.31 | -1.668 | -0.647 |
| sp\|Q8IZF6\|AGRG4_HUMAN Adhesion G-protein coupled receptor G4 OS=Homo sapiens GN=ADGRG4 PE=2 S' | 2233 | FLSTEAsTSPT | 0.164 | -0.106 | -1.078 | -0.34 |
| sp\|Q8IZF6\|AGRG4_HUMAN Adhesion G-protein coupled receptor G4 OS=Homo sapiens GN=ADGRG4 PE=2 S' | 2234 | LSTEAStSPTA | 0.305 | 0.292 | -0.289 | 0.103 |
| sp\|Q8IZF6\|AGRG4_HUMAN Adhesion G-protein coupled receptor G4 OS=Homo sapiens GN=ADGRG4 PE=2 S' | 2235 | STEASTsPTAT | 0.191 | -0.231 | -0.804 | -0.281 |
| sp\|Q8IZF6\|AGRG4_HUMAN Adhesion G-protein coupled receptor G4 OS=Homo sapiens GN=ADGRG4 PE=2 S' | 2237 | EASTSPtATKS | 0.192 | -0.025 | -0.979 | -0.271 |
| sp\|Q8IZF6\|AGRG4_HUMAN Adhesion G-protein coupled receptor G4 OS=Homo sapiens GN=ADGRG4 PE=2 S' | 2239 | STSPTAtKSTV | 0.071 | -0.236 | -1.566 | -0.577 |
| sp\|Q8IZF6\|AGRG4_HUMAN Adhesion G-protein coupled receptor G4 OS=Homo sapiens GN=ADGRG4 PE=2 S' | 2241 | SPTATKsTVSF | 0.049 | -0.159 | -1.251 | -0.454 |
| sp\|Q8IZF6\|AGRG4_HUMAN Adhesion G-protein coupled receptor G4 OS=Homo sapiens GN=ADGRG4 PE=2 S' | 2242 | PTATKStVSFY | 0.07 | -0.267 | -1.438 | -0.545 |
| sp\|Q8IZF6\|AGRG4_HUMAN Adhesion G-protein coupled receptor G4 OS=Homo sapiens GN=ADGRG4 PE=2 S' | 2244 | ATKSTVsFYNV | 0.367 | 0.049 | -0.807 | -0.13 |
| sp\|Q8IZF6\|AGRG4_HUMAN Adhesion G-protein coupled receptor G4 OS=Homo sapiens GN=ADGRG4 PE=2 S' | 2251 | FYNVEMsFSVF | 0.194 | 0.159 | -0.868 | -0.172 |
| sp\|Q8IZF6\|AGRG4_HUMAN Adhesion G-protein coupled receptor G4 OS=Homo sapiens GN=ADGRG4 PE=2 S' | 2253 | NVEMSFsVFVE | 0.383 | 0.197 | -0.123 | 0.152 |
| sp\|Q8IZF6\|AGRG4_HUMAN Adhesion G-protein coupled receptor G4 OS=Homo sapiens GN=ADGRG4 PE=2 S' | 2264 | EPRIPItSVIN | 0.214 | 0.06 | -0.776 | -0.167 |
| sp\|Q8IZF6\|AGRG4_HUMAN Adhesion G-protein coupled receptor G4 OS=Homo sapiens GN=ADGRG4 PE=2 S' | 2265 | PRIPITsVINE | 0.19 | 0.201 | -0.559 | -0.056 |
| sp\|Q8IZF6\|AGRG4_HUMAN Adhesion G-protein coupled receptor G4 OS=Homo sapiens GN=ADGRG4 PE=2 S' | 2271 | SVINEFtENSL | 0.077 | 0.028 | -1.003 | -0.299 |
| sp\|Q8IZF6\|AGRG4_HUMAN Adhesion G-protein coupled receptor G4 OS=Homo sapiens GN=ADGRG4 PE=2 S' | 2274 | NEFTENsLNSI | 0.4 | 0.087 | -0.493 | -0.002 |
| sp\|Q8IZF6\|AGRG4_HUMAN Adhesion G-protein coupled receptor G4 OS=Homo sapiens GN=ADGRG4 PE=2 S' | 2277 | TENSLNsIFQN | 0.108 | -0.202 | -1.336 | -0.477 |
| sp\|Q8IZF6\|AGRG4_HUMAN Adhesion G-protein coupled receptor G4 OS=Homo sapiens GN=ADGRG4 PE=2 S' | 2282 | NSIFQNsEFSL | 0.172 | 0.077 | -0.92 | -0.224 |
| sp\|Q8IZF6\|AGRG4_HUMAN Adhesion G-protein coupled receptor G4 OS=Homo sapiens GN=ADGRG4 PE=2 S' | 2285 | FQNSEFsLATL | 0.211 | 0.011 | -0.672 | -0.15 |
| sp\|Q8IZF6\|AGRG4_HUMAN Adhesion G-protein coupled receptor G4 OS=Homo sapiens GN=ADGRG4 PE=2 S' | 2288 | SEFSLAtLETQ | 0.135 | -0.164 | -1.29 | -0.44 |
| sp\|Q8IZF6\|AGRG4_HUMAN Adhesion G-protein coupled receptor G4 OS=Homo sapiens GN=ADGRG4 PE=2 S' | 2291 | SLATLEtQIKS | 0.057 | -0.23 | -1.667 | -0.613 |
| sp\|Q8IZF6\|AGRG4_HUMAN Adhesion G-protein coupled receptor G4 OS=Homo sapiens GN=ADGRG4 PE=2 S' | 2295 | LETQIKsRDIS | 0.275 | -0.065 | -0.376 | -0.055 |
| sp\|Q8IZF6\|AGRG4_HUMAN Adhesion G-protein coupled receptor G4 OS=Homo sapiens GN=ADGRG4 PE=2 S' | 2299 | IKSRDIsEEEM | 0.824 | 0.919 | 0.802 | 0.848 |
| sp\|Q8IZF6\|AGRG4_HUMAN Adhesion G-protein coupled receptor G4 OS=Homo sapiens GN=ADGRG4 PE=2 S' | 2320 | EGQEMAtISYV | 0.074 | -0.202 | -1.411 | -0.513 |
| sp\|Q8IZF6\|AGRG4_HUMAN Adhesion G-protein coupled receptor G4 OS=Homo sapiens GN=ADGRG4 PE=2 S' | 2322 | QEMATIsYVPY | 0.093 | -0.116 | -1.174 | -0.399 |
| sp\|Q8IZF6\|AGRG4_HUMAN Adhesion G-protein coupled receptor G4 OS=Homo sapiens GN=ADGRG4 PE=2 S' | 2327 | ISYVPYsCVCQ | 0.082 | -0.062 | -1.001 | -0.327 |
| sp\|Q8IZF6\|AGRG4_HUMAN Adhesion G-protein coupled receptor G4 OS=Homo sapiens GN=ADGRG4 PE=2 S' | 2337 | QVIIKAsSSLA | 0.06 | -0.144 | -1.352 | -0.479 |
| sp\|Q8IZF6\|AGRG4_HUMAN Adhesion G-protein coupled receptor G4 OS=Homo sapiens GN=ADGRG4 PE=2 S' | 2338 | VIIKASsSLAS | 0.212 | 0.068 | -0.798 | -0.173 |
| sp\|Q8IZF6\|AGRG4_HUMAN Adhesion G-protein coupled receptor G4 OS=Homo sapiens GN=ADGRG4 PE=2 S' | 2339 | IIKASSsLASS | 0.339 | 0.16 | -0.17 | 0.11 |
| sp\|Q8IZF6\|AGRG4_HUMAN Adhesion G-protein coupled receptor G4 OS=Homo sapiens GN=ADGRG4 PE=2 S' | 2342 | ASSSLAsSELM | 0.091 | -0.288 | -1.415 | -0.537 |
| sp\|Q8IZF6\|AGRG4_HUMAN Adhesion G-protein coupled receptor G4 OS=Homo sapiens GN=ADGRG4 PE=2 S' | 2343 | SSSLASsELMR | 0.09 | -0.19 | -1.364 | -0.488 |
| sp\|Q8IZF6\|AGRG4_HUMAN Adhesion G-protein coupled receptor G4 OS=Homo sapiens GN=ADGRG4 PE=2 S' | 2351 | LMRKIKsKIHG | 0.456 | 0.37 | 0.354 | 0.393 |
| sp\|Q8IZF6\|AGRG4_HUMAN Adhesion G-protein coupled receptor G4 OS=Homo sapiens GN=ADGRG4 PE=2 S' | 2358 | KIHGNFtHGNF | 0.213 | 0.084 | -0.666 | -0.123 |
| sp\|Q8IZF6\|AGRG4_HUMAN Adhesion G-protein coupled receptor G4 OS=Homo sapiens GN=ADGRG4 PE=2 S' | 2363 | FTHGNFtQDQL | 0.234 | 0.025 | -0.703 | -0.148 |
| sp\|Q8IZF6\|AGRG4_HUMAN Adhesion G-protein coupled receptor G4 OS=Homo sapiens GN=ADGRG4 PE=2 S' | 2368 | FTQDQLtLLVN | 0.202 | 0.146 | -0.977 | -0.21 |
| sp\|Q8IZF6\|AGRG4_HUMAN Adhesion G-protein coupled receptor G4 OS=Homo sapiens GN=ADGRG4 PE=2 S' | 2391 | NCKADEtASKY | 0.202 | -0.083 | -1.227 | -0.369 |
| sp\|Q8IZF6\|AGRG4_HUMAN Adhesion G-protein coupled receptor G4 OS=Homo sapiens GN=ADGRG4 PE=2 S' | 2393 | KADETAsKYKG | 0.076 | -0.132 | -1.352 | -0.469 |
| sp\|Q8IZF6\|AGRG4_HUMAN Adhesion G-protein coupled receptor G4 OS=Homo sapiens GN=ADGRG4 PE=2 S' | 2398 | ASKYKGtYKWL | 0.079 | -0.21 | -1.03 | -0.387 |
| sp\|Q8IZF6\|AGRG4_HUMAN Adhesion G-protein coupled receptor G4 OS=Homo sapiens GN=ADGRG4 PE=2 S' | 2404 | TYKWLLtNPTE | 0.447 | 0.292 | -0.328 | 0.137 |
| sp\|Q8IZF6\|AGRG4_HUMAN Adhesion G-protein coupled receptor G4 OS=Homo sapiens GN=ADGRG4 PE=2 S' | 2407 | WLLTNPtETAQ | 0.416 | 0.029 | -0.28 | 0.055 |
| sp\|Q8IZF6\|AGRG4_HUMAN Adhesion G-protein coupled receptor G4 OS=Homo sapiens GN=ADGRG4 PE=2 S' | 2409 | LTNPTEtAQTR | 0.112 | -0.122 | -1.233 | -0.414 |
| sp\|Q8IZF6\|AGRG4_HUMAN Adhesion G-protein coupled receptor G4 OS=Homo sapiens GN=ADGRG4 PE=2 S' | 2412 | PTETAQtRCIK | 0.257 | -0.217 | -0.941 | -0.3 |
| sp\|Q8IZF6\|AGRG4_HUMAN Adhesion G-protein coupled receptor G4 OS=Homo sapiens GN=ADGRG4 PE=2 S' | 2423 | NEDGNAtRFCS | 0.142 | -0.113 | -1.106 | -0.359 |
| sp\|Q8IZF6\|AGRG4_HUMAN Adhesion G-protein coupled receptor G4 OS=Homo sapiens GN=ADGRG4 PE=2 S' | 2427 | NATRFCsISIN | 0.555 | 0.713 | -0.119 | 0.383 |
| sp\|Q8IZF6\|AGRG4_HUMAN Adhesion G-protein coupled receptor G4 OS=Homo sapiens GN=ADGRG4 PE=2 S' | 2429 | TRFCSIsINTG | 0.522 | 0.351 | -0.003 | 0.29 |
| sp\|Q8IZF6\|AGRG4_HUMAN Adhesion G-protein coupled receptor G4 OS=Homo sapiens GN=ADGRG4 PE=2 S' | 2432 | CSISINtGKSQ | 0.095 | -0.217 | -1.104 | -0.409 |
| sp\|Q8IZF6\|AGRG4_HUMAN Adhesion G-protein coupled receptor G4 OS=Homo sapiens GN=ADGRG4 PE=2 S' | 2435 | SINTGKsQWEK | 0.19 | -0.094 | -1.182 | -0.362 |
| sp\|Q8IZF6\|AGRG4_HUMAN Adhesion G-protein coupled receptor G4 OS=Homo sapiens GN=ADGRG4 PE=2 S' | 2462 | VDLANItISDE | 0.116 | -0.29 | -0.945 | -0.373 |
| sp\|Q8IZF6\|AGRG4_HUMAN Adhesion G-protein coupled receptor G4 OS=Homo sapiens GN=ADGRG4 PE=2 S' | 2464 | LANITIsDENA | 0.118 | -0.026 | -1.201 | -0.37 |

| | | | | | | |
|---|---|---|---|---|---|---|
| sp\|Q8IZF6\|AGRG4_HUMAN Adhesion G-protein coupled receptor G4 OS=Homo sapiens GN=ADGRG4 PE=2 S' | 2482 | LNLINEsPALG | 0.045 | -0.496 | -1.456 | -0.636 |
| sp\|Q8IZF6\|AGRG4_HUMAN Adhesion G-protein coupled receptor G4 OS=Homo sapiens GN=ADGRG4 PE=2 S' | 2490 | ALGKEEtKIIV | 0.289 | -0.041 | -0.966 | -0.239 |
| sp\|Q8IZF6\|AGRG4_HUMAN Adhesion G-protein coupled receptor G4 OS=Homo sapiens GN=ADGRG4 PE=2 S' | 2495 | ETKIIVsKISD | 0.206 | -0.041 | -0.399 | -0.078 |
| sp\|Q8IZF6\|AGRG4_HUMAN Adhesion G-protein coupled receptor G4 OS=Homo sapiens GN=ADGRG4 PE=2 S' | 2498 | IIVSKIsDISQ | 0.103 | -0.144 | -1.165 | -0.402 |
| sp\|Q8IZF6\|AGRG4_HUMAN Adhesion G-protein coupled receptor G4 OS=Homo sapiens GN=ADGRG4 PE=2 S' | 2501 | SKISDIsQCDE | 0.17 | -0.222 | -1.275 | -0.442 |
| sp\|Q8IZF6\|AGRG4_HUMAN Adhesion G-protein coupled receptor G4 OS=Homo sapiens GN=ADGRG4 PE=2 S' | 2507 | SQCDEIsMNLT | 0.135 | -0.041 | -1.138 | -0.348 |
| sp\|Q8IZF6\|AGRG4_HUMAN Adhesion G-protein coupled receptor G4 OS=Homo sapiens GN=ADGRG4 PE=2 S' | 2511 | EISMNLtHVML | 0.128 | -0.104 | -0.884 | -0.287 |
| sp\|Q8IZF6\|AGRG4_HUMAN Adhesion G-protein coupled receptor G4 OS=Homo sapiens GN=ADGRG4 PE=2 S' | 2528 | LEKQNNsASDL | 0.41 | 0.105 | -0.39 | 0.042 |
| sp\|Q8IZF6\|AGRG4_HUMAN Adhesion G-protein coupled receptor G4 OS=Homo sapiens GN=ADGRG4 PE=2 S' | 2530 | KQNNSAsDLHE | 0.171 | 0.052 | -0.708 | -0.162 |
| sp\|Q8IZF6\|AGRG4_HUMAN Adhesion G-protein coupled receptor G4 OS=Homo sapiens GN=ADGRG4 PE=2 S' | 2536 | SDLHEIsNEIL | 0.075 | -0.275 | -1.499 | -0.566 |
| sp\|Q8IZF6\|AGRG4_HUMAN Adhesion G-protein coupled receptor G4 OS=Homo sapiens GN=ADGRG4 PE=2 S' | 2546 | LRIIERtGHKM | 0.099 | 0.085 | -1.209 | -0.342 |
| sp\|Q8IZF6\|AGRG4_HUMAN Adhesion G-protein coupled receptor G4 OS=Homo sapiens GN=ADGRG4 PE=2 S' | 2553 | GHKMEFsGQIA | 0.3 | -0.077 | -0.799 | -0.192 |
| sp\|Q8IZF6\|AGRG4_HUMAN Adhesion G-protein coupled receptor G4 OS=Homo sapiens GN=ADGRG4 PE=2 S' | 2560 | GQIANLtVAGL | 0.131 | -0.11 | -0.778 | -0.252 |
| sp\|Q8IZF6\|AGRG4_HUMAN Adhesion G-protein coupled receptor G4 OS=Homo sapiens GN=ADGRG4 PE=2 S' | 2574 | VLRGDHtFDGM | 0.684 | 0.422 | 0.404 | 0.503 |
| sp\|Q8IZF6\|AGRG4_HUMAN Adhesion G-protein coupled receptor G4 OS=Homo sapiens GN=ADGRG4 PE=2 S' | 2581 | FDGMAFsIHSY | 0.138 | -0.029 | -0.909 | -0.267 |
| sp\|Q8IZF6\|AGRG4_HUMAN Adhesion G-protein coupled receptor G4 OS=Homo sapiens GN=ADGRG4 PE=2 S' | 2584 | MAFSIHsYEEG | 0.264 | 0.058 | -0.652 | -0.11 |
| sp\|Q8IZF6\|AGRG4_HUMAN Adhesion G-protein coupled receptor G4 OS=Homo sapiens GN=ADGRG4 PE=2 S' | 2589 | HSYEEGtDPEI | 0.418 | 0.425 | -0.32 | 0.174 |
| sp\|Q8IZF6\|AGRG4_HUMAN Adhesion G-protein coupled receptor G4 OS=Homo sapiens GN=ADGRG4 PE=2 S' | 2606 | VGGILAsIYLP | 0.061 | -0.199 | -1.351 | -0.496 |
| sp\|Q8IZF6\|AGRG4_HUMAN Adhesion G-protein coupled receptor G4 OS=Homo sapiens GN=ADGRG4 PE=2 S' | 2612 | SIYLPKsLTER | 0.393 | 0.071 | -0.261 | 0.068 |
| sp\|Q8IZF6\|AGRG4_HUMAN Adhesion G-protein coupled receptor G4 OS=Homo sapiens GN=ADGRG4 PE=2 S' | 2614 | YLPKSLtERIP | 0.432 | 0.194 | -0.174 | 0.151 |
| sp\|Q8IZF6\|AGRG4_HUMAN Adhesion G-protein coupled receptor G4 OS=Homo sapiens GN=ADGRG4 PE=2 S' | 2620 | TERIPLsNLQT | 0.083 | -0.106 | -1.259 | -0.427 |
| sp\|Q8IZF6\|AGRG4_HUMAN Adhesion G-protein coupled receptor G4 OS=Homo sapiens GN=ADGRG4 PE=2 S' | 2624 | PLSNLQtILFN | 0.081 | -0.115 | -1.064 | -0.366 |
| sp\|Q8IZF6\|AGRG4_HUMAN Adhesion G-protein coupled receptor G4 OS=Homo sapiens GN=ADGRG4 PE=2 S' | 2633 | FNFFGQtSLFK | 0.057 | -0.246 | -1.423 | -0.537 |
| sp\|Q8IZF6\|AGRG4_HUMAN Adhesion G-protein coupled receptor G4 OS=Homo sapiens GN=ADGRG4 PE=2 S' | 2634 | NFFGQTsLFKT | 0.203 | 0.027 | -0.464 | -0.078 |
| sp\|Q8IZF6\|AGRG4_HUMAN Adhesion G-protein coupled receptor G4 OS=Homo sapiens GN=ADGRG4 PE=2 S' | 2638 | QTSLFKtKNVT | 0.237 | -0.066 | -0.623 | -0.151 |
| sp\|Q8IZF6\|AGRG4_HUMAN Adhesion G-protein coupled receptor G4 OS=Homo sapiens GN=ADGRG4 PE=2 S' | 2642 | FKTKNVtKALT | 0.616 | 0.103 | 0.108 | 0.276 |
| sp\|Q8IZF6\|AGRG4_HUMAN Adhesion G-protein coupled receptor G4 OS=Homo sapiens GN=ADGRG4 PE=2 S' | 2646 | NVTKALtTYVV | 0.26 | 0.089 | -0.746 | -0.132 |
| sp\|Q8IZF6\|AGRG4_HUMAN Adhesion G-protein coupled receptor G4 OS=Homo sapiens GN=ADGRG4 PE=2 S' | 2647 | VTKALTtYVVS | 0.109 | -0.186 | -1.186 | -0.421 |
| sp\|Q8IZF6\|AGRG4_HUMAN Adhesion G-protein coupled receptor G4 OS=Homo sapiens GN=ADGRG4 PE=2 S' | 2651 | LTTYVVsASIS | 0.256 | -0.074 | -0.823 | -0.214 |
| sp\|Q8IZF6\|AGRG4_HUMAN Adhesion G-protein coupled receptor G4 OS=Homo sapiens GN=ADGRG4 PE=2 S' | 2653 | TYVVSAsISDD | 0.346 | 0.187 | -0.352 | 0.06 |
| sp\|Q8IZF6\|AGRG4_HUMAN Adhesion G-protein coupled receptor G4 OS=Homo sapiens GN=ADGRG4 PE=2 S' | 2655 | VVSASIsDDMF | 0.313 | 0.076 | -0.438 | -0.016 |
| sp\|Q8IZF6\|AGRG4_HUMAN Adhesion G-protein coupled receptor G4 OS=Homo sapiens GN=ADGRG4 PE=2 S' | 2670 | ADPVVItLQHI | 0.159 | -0.154 | -0.745 | -0.247 |
| sp\|Q8IZF6\|AGRG4_HUMAN Adhesion G-protein coupled receptor G4 OS=Homo sapiens GN=ADGRG4 PE=2 S' | 2701 | GLGGWNsSGCK | 0.415 | 0.182 | -0.44 | 0.052 |
| sp\|Q8IZF6\|AGRG4_HUMAN Adhesion G-protein coupled receptor G4 OS=Homo sapiens GN=ADGRG4 PE=2 S' | 2702 | LGGWNSsGCKV | 0.057 | -0.359 | -1.399 | -0.567 |
| sp\|Q8IZF6\|AGRG4_HUMAN Adhesion G-protein coupled receptor G4 OS=Homo sapiens GN=ADGRG4 PE=2 S' | 2709 | GCKVKEtNVNY | 0.189 | -0.002 | -1.386 | -0.4 |
| sp\|Q8IZF6\|AGRG4_HUMAN Adhesion G-protein coupled receptor G4 OS=Homo sapiens GN=ADGRG4 PE=2 S' | 2714 | ETNVNYtICQC | 0.061 | -0.277 | -1.362 | -0.526 |
| sp\|Q8IZF6\|AGRG4_HUMAN Adhesion G-protein coupled receptor G4 OS=Homo sapiens GN=ADGRG4 PE=2 S' | 2722 | CQCDHLtHFGV | 0.283 | -0.012 | -0.749 | -0.159 |
| sp\|Q8IZF6\|AGRG4_HUMAN Adhesion G-protein coupled receptor G4 OS=Homo sapiens GN=ADGRG4 PE=2 S' | 2731 | GVLMDLsRSTV | 0.073 | -0.296 | -1.566 | -0.596 |
| sp\|Q8IZF6\|AGRG4_HUMAN Adhesion G-protein coupled receptor G4 OS=Homo sapiens GN=ADGRG4 PE=2 S' | 2733 | LMDLSRsTVDS | 0.18 | 0.246 | -0.814 | -0.129 |
| sp\|Q8IZF6\|AGRG4_HUMAN Adhesion G-protein coupled receptor G4 OS=Homo sapiens GN=ADGRG4 PE=2 S' | 2734 | MDLSRStVDSV | 0.134 | -0.011 | -1.027 | -0.301 |
| sp\|Q8IZF6\|AGRG4_HUMAN Adhesion G-protein coupled receptor G4 OS=Homo sapiens GN=ADGRG4 PE=2 S' | 2737 | SRSTVDsVNEQ | 0.192 | 0.004 | -1.096 | -0.3 |
| sp\|Q8IZF6\|AGRG4_HUMAN Adhesion G-protein coupled receptor G4 OS=Homo sapiens GN=ADGRG4 PE=2 S' | 2747 | QILALItYTGC | 0.126 | -0.245 | -1.157 | -0.425 |
| sp\|Q8IZF6\|AGRG4_HUMAN Adhesion G-protein coupled receptor G4 OS=Homo sapiens GN=ADGRG4 PE=2 S' | 2749 | LALITYtGCGI | 0.096 | -0.106 | -0.727 | -0.246 |
| sp\|Q8IZF6\|AGRG4_HUMAN Adhesion G-protein coupled receptor G4 OS=Homo sapiens GN=ADGRG4 PE=2 S' | 2754 | YTGCGIsSIFL | 0.062 | -0.228 | -1.32 | -0.495 |
| sp\|Q8IZF6\|AGRG4_HUMAN Adhesion G-protein coupled receptor G4 OS=Homo sapiens GN=ADGRG4 PE=2 S' | 2755 | TGCGISsIFLG | 0.091 | -0.162 | -0.839 | -0.303 |
| sp\|Q8IZF6\|AGRG4_HUMAN Adhesion G-protein coupled receptor G4 OS=Homo sapiens GN=ADGRG4 PE=2 S' | 2764 | LGVAVVtYIAF | 0.168 | -0.098 | -0.568 | -0.166 |
| sp\|Q8IZF6\|AGRG4_HUMAN Adhesion G-protein coupled receptor G4 OS=Homo sapiens GN=ADGRG4 PE=2 S' | 2785 | ILINLCtALLM | 0.228 | 0.056 | -0.862 | -0.193 |
| sp\|Q8IZF6\|AGRG4_HUMAN Adhesion G-protein coupled receptor G4 OS=Homo sapiens GN=ADGRG4 PE=2 S' | 2798 | LVFLINsWLSS | 0.074 | 0.007 | -1.202 | -0.374 |
| sp\|Q8IZF6\|AGRG4_HUMAN Adhesion G-protein coupled receptor G4 OS=Homo sapiens GN=ADGRG4 PE=2 S' | 2801 | LINSWLsSFQK | 0.112 | -0.146 | -0.992 | -0.342 |

| | | | | | | |
|---|---|---|---|---|---|---|
| sp\|Q8IZF6\|AGRG4_HUMAN Adhesion G-protein coupled receptor G4 OS=Homo sapiens GN=ADGRG4 PE=2 S' | 2802 | INSWLSsFQKV | 0.137 | -0.238 | -1.046 | -0.382 |
| sp\|Q8IZF6\|AGRG4_HUMAN Adhesion G-protein coupled receptor G4 OS=Homo sapiens GN=ADGRG4 PE=2 S' | 2811 | KVGVCItAAVA | 0.112 | -0.103 | -1.159 | -0.383 |
| sp\|Q8IZF6\|AGRG4_HUMAN Adhesion G-protein coupled receptor G4 OS=Homo sapiens GN=ADGRG4 PE=2 S' | 2823 | HYFLLVsFTWM | 0.429 | 0.114 | -0.309 | 0.078 |
| sp\|Q8IZF6\|AGRG4_HUMAN Adhesion G-protein coupled receptor G4 OS=Homo sapiens GN=ADGRG4 PE=2 S' | 2825 | FLLVSFtWMGL | 0.487 | 0.466 | 0.141 | 0.365 |
| sp\|Q8IZF6\|AGRG4_HUMAN Adhesion G-protein coupled receptor G4 OS=Homo sapiens GN=ADGRG4 PE=2 S' | 2868 | AIMVAItVSVK | 0.168 | 0.022 | -0.865 | -0.225 |
| sp\|Q8IZF6\|AGRG4_HUMAN Adhesion G-protein coupled receptor G4 OS=Homo sapiens GN=ADGRG4 PE=2 S' | 2870 | MVAITVsVKKD | 0.223 | 0.1 | -0.448 | -0.042 |
| sp\|Q8IZF6\|AGRG4_HUMAN Adhesion G-protein coupled receptor G4 OS=Homo sapiens GN=ADGRG4 PE=2 S' | 2878 | KKDLYGtLSPT | 0.168 | -0.166 | -0.933 | -0.31 |
| sp\|Q8IZF6\|AGRG4_HUMAN Adhesion G-protein coupled receptor G4 OS=Homo sapiens GN=ADGRG4 PE=2 S' | 2880 | DLYGTLsPTTP | 0.051 | -0.372 | -1.504 | -0.608 |
| sp\|Q8IZF6\|AGRG4_HUMAN Adhesion G-protein coupled receptor G4 OS=Homo sapiens GN=ADGRG4 PE=2 S' | 2882 | YGTLSPtTPFC | 0.482 | 0.449 | -0.05 | 0.294 |
| sp\|Q8IZF6\|AGRG4_HUMAN Adhesion G-protein coupled receptor G4 OS=Homo sapiens GN=ADGRG4 PE=2 S' | 2883 | GTLSPTtPFCW | 0.08 | -0.379 | -1.254 | -0.518 |
| sp\|Q8IZF6\|AGRG4_HUMAN Adhesion G-protein coupled receptor G4 OS=Homo sapiens GN=ADGRG4 PE=2 S' | 2892 | CWIKDDsIFYI | 0.334 | -0.011 | -0.528 | -0.068 |
| sp\|Q8IZF6\|AGRG4_HUMAN Adhesion G-protein coupled receptor G4 OS=Homo sapiens GN=ADGRG4 PE=2 S' | 2897 | DSIFYIsVVAY | 0.394 | 0.078 | -0.556 | -0.028 |
| sp\|Q8IZF6\|AGRG4_HUMAN Adhesion G-protein coupled receptor G4 OS=Homo sapiens GN=ADGRG4 PE=2 S' | 2911 | IFLMNLsMFCT | 0.207 | 0.008 | -0.535 | -0.107 |
| sp\|Q8IZF6\|AGRG4_HUMAN Adhesion G-protein coupled receptor G4 OS=Homo sapiens GN=ADGRG4 PE=2 S' | 2915 | NLSMFCtVLVQ | 0.26 | -0.045 | -0.994 | -0.26 |
| sp\|Q8IZF6\|AGRG4_HUMAN Adhesion G-protein coupled receptor G4 OS=Homo sapiens GN=ADGRG4 PE=2 S' | 2922 | VLVQLNsVKSQ | 0.152 | -0.069 | -0.969 | -0.295 |
| sp\|Q8IZF6\|AGRG4_HUMAN Adhesion G-protein coupled receptor G4 OS=Homo sapiens GN=ADGRG4 PE=2 S' | 2925 | QLNSVKsQIQK | 0.235 | -0.104 | -0.738 | -0.202 |
| sp\|Q8IZF6\|AGRG4_HUMAN Adhesion G-protein coupled receptor G4 OS=Homo sapiens GN=ADGRG4 PE=2 S' | 2930 | KSQIQKtRRKM | 0.116 | -0.2 | -1.099 | -0.394 |
| sp\|Q8IZF6\|AGRG4_HUMAN Adhesion G-protein coupled receptor G4 OS=Homo sapiens GN=ADGRG4 PE=2 S' | 2942 | LHDLKGtMSLT | 0.034 | -0.22 | -1.968 | -0.718 |
| sp\|Q8IZF6\|AGRG4_HUMAN Adhesion G-protein coupled receptor G4 OS=Homo sapiens GN=ADGRG4 PE=2 S' | 2944 | DLKGTMsLTFL | 0.561 | 0.301 | 0.009 | 0.29 |
| sp\|Q8IZF6\|AGRG4_HUMAN Adhesion G-protein coupled receptor G4 OS=Homo sapiens GN=ADGRG4 PE=2 S' | 2946 | KGTMSLtFLLG | 0.268 | 0.119 | -0.347 | 0.013 |
| sp\|Q8IZF6\|AGRG4_HUMAN Adhesion G-protein coupled receptor G4 OS=Homo sapiens GN=ADGRG4 PE=2 S' | 2952 | TFLLGLtWGFA | 0.076 | -0.123 | -1.036 | -0.361 |
| sp\|Q8IZF6\|AGRG4_HUMAN Adhesion G-protein coupled receptor G4 OS=Homo sapiens GN=ADGRG4 PE=2 S' | 2976 | LFAIFNtLQGF | 0.387 | 0.071 | -0.142 | 0.105 |
| sp\|Q8IZF6\|AGRG4_HUMAN Adhesion G-protein coupled receptor G4 OS=Homo sapiens GN=ADGRG4 PE=2 S' | 2992 | HCVMKEsVREQ | 0.157 | -0.147 | -1.13 | -0.373 |
| sp\|Q8IZF6\|AGRG4_HUMAN Adhesion G-protein coupled receptor G4 OS=Homo sapiens GN=ADGRG4 PE=2 S' | 3011 | WLRLDNsSDGS | 0.507 | 0.222 | -0.244 | 0.162 |
| sp\|Q8IZF6\|AGRG4_HUMAN Adhesion G-protein coupled receptor G4 OS=Homo sapiens GN=ADGRG4 PE=2 S' | 3012 | LRLDNSsDGSS | 0.152 | 0.127 | -0.868 | -0.196 |
| sp\|Q8IZF6\|AGRG4_HUMAN Adhesion G-protein coupled receptor G4 OS=Homo sapiens GN=ADGRG4 PE=2 S' | 3015 | DNSSDGsSRCQ | 0.06 | -0.448 | -1.729 | -0.706 |
| sp\|Q8IZF6\|AGRG4_HUMAN Adhesion G-protein coupled receptor G4 OS=Homo sapiens GN=ADGRG4 PE=2 S' | 3016 | NSSDGSsRCQI | 0.073 | -0.403 | -1.433 | -0.588 |
| sp\|Q8IZF6\|AGRG4_HUMAN Adhesion G-protein coupled receptor G4 OS=Homo sapiens GN=ADGRG4 PE=2 S' | 3039 | FEHKLLtPSLK | 0.039 | -0.444 | -1.832 | -0.746 |
| sp\|Q8IZF6\|AGRG4_HUMAN Adhesion G-protein coupled receptor G4 OS=Homo sapiens GN=ADGRG4 PE=2 S' | 3041 | HKLLTPsLKST | 0.177 | 0.046 | -0.458 | -0.078 |
| sp\|Q8IZF6\|AGRG4_HUMAN Adhesion G-protein coupled receptor G4 OS=Homo sapiens GN=ADGRG4 PE=2 S' | 3044 | LTPSLKsTATS | 0.066 | -0.3 | -1.385 | -0.54 |
| sp\|Q8IZF6\|AGRG4_HUMAN Adhesion G-protein coupled receptor G4 OS=Homo sapiens GN=ADGRG4 PE=2 S' | 3045 | TPSLKStATSS | 0.04 | -0.315 | -1.872 | -0.716 |
| sp\|Q8IZF6\|AGRG4_HUMAN Adhesion G-protein coupled receptor G4 OS=Homo sapiens GN=ADGRG4 PE=2 S' | 3047 | SLKSTAtSSTF | 0.09 | -0.109 | -1.164 | -0.394 |
| sp\|Q8IZF6\|AGRG4_HUMAN Adhesion G-protein coupled receptor G4 OS=Homo sapiens GN=ADGRG4 PE=2 S' | 3048 | LKSTATsSTFK | 0.258 | -0.12 | -0.707 | -0.19 |
| sp\|Q8IZF6\|AGRG4_HUMAN Adhesion G-protein coupled receptor G4 OS=Homo sapiens GN=ADGRG4 PE=2 S' | 3049 | KSTATSsTFKS | 0.055 | -0.259 | -1.391 | -0.532 |
| sp\|Q8IZF6\|AGRG4_HUMAN Adhesion G-protein coupled receptor G4 OS=Homo sapiens GN=ADGRG4 PE=2 S' | 3050 | STATSStFKSL | 0.161 | 0.057 | -0.725 | -0.169 |
| sp\|Q8IZF6\|AGRG4_HUMAN Adhesion G-protein coupled receptor G4 OS=Homo sapiens GN=ADGRG4 PE=2 S' | 3053 | TSSTFKsLGSA | 0.301 | -0.063 | -0.429 | -0.064 |
| sp\|Q8IZF6\|AGRG4_HUMAN Adhesion G-protein coupled receptor G4 OS=Homo sapiens GN=ADGRG4 PE=2 S' | 3056 | TFKSLGsAQGT | 0.106 | -0.234 | -1.081 | -0.403 |
| sp\|Q8IZF6\|AGRG4_HUMAN Adhesion G-protein coupled receptor G4 OS=Homo sapiens GN=ADGRG4 PE=2 S' | 3060 | LGSAQGtPSEI | 0.05 | -0.501 | -1.531 | -0.661 |
| sp\|Q8IZF6\|AGRG4_HUMAN Adhesion G-protein coupled receptor G4 OS=Homo sapiens GN=ADGRG4 PE=2 S' | 3062 | SAQGTPsEISF | 0.039 | -0.122 | -1.357 | -0.48 |
| sp\|Q8IZF6\|AGRG4_HUMAN Adhesion G-protein coupled receptor G4 OS=Homo sapiens GN=ADGRG4 PE=2 S' | 3065 | GTPSEIsFPND | 0.704 | 0.462 | -0.126 | 0.347 |
| sp\|Q8IZF6\|AGRG4_HUMAN Adhesion G-protein coupled receptor G4 OS=Homo sapiens GN=ADGRG4 PE=2 S' | 3078 | DKDPYCsSP-- | 0.744 | 0.565 | -0.093 | 0.405 |
| sp\|Q8IZF6\|AGRG4_HUMAN Adhesion G-protein coupled receptor G4 OS=Homo sapiens GN=ADGRG4 PE=2 S' | 3079 | KDPYCSsP--- | 0.041 | -0.372 | -1.593 | -0.641 |
| sp\|Q8IZF5\|AGRF3_HUMAN Adhesion G-protein coupled receptor F3 OS=Homo sapiens GN=ADGRF3 PE=2 SV | 334 | RVPLKAtDVAR | 0.181 | 0.046 | -0.93 | -0.234 |
| sp\|Q8IZF5\|AGRF3_HUMAN Adhesion G-protein coupled receptor F3 OS=Homo sapiens GN=ADGRF3 PE=2 SV | 344 | RLPYQLsISCA | 0.08 | -0.065 | -1.168 | -0.384 |
| sp\|Q8IZF5\|AGRF3_HUMAN Adhesion G-protein coupled receptor F3 OS=Homo sapiens GN=ADGRF3 PE=2 SV | 346 | PYQLSIsCATS | 0.441 | 0.278 | -0.338 | 0.127 |
| sp\|Q8IZF5\|AGRF3_HUMAN Adhesion G-protein coupled receptor F3 OS=Homo sapiens GN=ADGRF3 PE=2 SV | 349 | LSISCAtSPGF | 0.474 | 0.314 | -0.139 | 0.216 |
| sp\|Q8IZF5\|AGRF3_HUMAN Adhesion G-protein coupled receptor F3 OS=Homo sapiens GN=ADGRF3 PE=2 SV | 350 | SISCATsPGFQ | 0.117 | -0.344 | -1.324 | -0.517 |
| sp\|Q8IZF5\|AGRF3_HUMAN Adhesion G-protein coupled receptor F3 OS=Homo sapiens GN=ADGRF3 PE=2 SV | 356 | SPGFQLsCCIP | 0.032 | -0.28 | -1.977 | -0.742 |
| sp\|Q8IZF5\|AGRF3_HUMAN Adhesion G-protein coupled receptor F3 OS=Homo sapiens GN=ADGRF3 PE=2 SV | 361 | LSCCIPsTNLA | 0.204 | -0.163 | -0.659 | -0.206 |

| | | | | | | |
|---|---|---|---|---|---|---|
| sp\|Q8IZF5\|AGRF3_HUMAN Adhesion G-protein coupled receptor F3 OS=Homo sapiens GN=ADGRF3 PE=2 SV | 362 | SCCIPStNLAY | 0.095 | -0.143 | -1.499 | -0.516 |
| sp\|Q8IZF5\|AGRF3_HUMAN Adhesion G-protein coupled receptor F3 OS=Homo sapiens GN=ADGRF3 PE=2 SV | 367 | STNLAYtAAWS | 0.131 | -0.008 | -1.194 | -0.357 |
| sp\|Q8IZF5\|AGRF3_HUMAN Adhesion G-protein coupled receptor F3 OS=Homo sapiens GN=ADGRF3 PE=2 SV | 371 | AYTAAWsPGEG | 0.338 | -0.228 | -0.657 | -0.182 |
| sp\|Q8IZF5\|AGRF3_HUMAN Adhesion G-protein coupled receptor F3 OS=Homo sapiens GN=ADGRF3 PE=2 SV | 376 | WSPGEGsKASS | 0.026 | -0.457 | -1.832 | -0.754 |
| sp\|Q8IZF5\|AGRF3_HUMAN Adhesion G-protein coupled receptor F3 OS=Homo sapiens GN=ADGRF3 PE=2 SV | 379 | GEGSKAsSFNE | 0.113 | -0.146 | -1.449 | -0.494 |
| sp\|Q8IZF5\|AGRF3_HUMAN Adhesion G-protein coupled receptor F3 OS=Homo sapiens GN=ADGRF3 PE=2 SV | 380 | EGSKASsFNES | 0.366 | 0.063 | -0.246 | 0.061 |
| sp\|Q8IZF5\|AGRF3_HUMAN Adhesion G-protein coupled receptor F3 OS=Homo sapiens GN=ADGRF3 PE=2 SV | 384 | ASSFNEsGSQC | 0.081 | -0.407 | -1.518 | -0.615 |
| sp\|Q8IZF5\|AGRF3_HUMAN Adhesion G-protein coupled receptor F3 OS=Homo sapiens GN=ADGRF3 PE=2 SV | 386 | SFNESGsQCFV | 0.059 | -0.195 | -1.841 | -0.659 |
| sp\|Q8IZF5\|AGRF3_HUMAN Adhesion G-protein coupled receptor F3 OS=Homo sapiens GN=ADGRF3 PE=2 SV | 401 | RCPMADtTYAC | 0.253 | -0.07 | -1.091 | -0.303 |
| sp\|Q8IZF5\|AGRF3_HUMAN Adhesion G-protein coupled receptor F3 OS=Homo sapiens GN=ADGRF3 PE=2 SV | 402 | CPMADTtYACD | 0.137 | -0.103 | -0.874 | -0.28 |
| sp\|Q8IZF5\|AGRF3_HUMAN Adhesion G-protein coupled receptor F3 OS=Homo sapiens GN=ADGRF3 PE=2 SV | 409 | YACDLQsLGLA | 0.34 | 0.048 | -0.464 | -0.025 |
| sp\|Q8IZF5\|AGRF3_HUMAN Adhesion G-protein coupled receptor F3 OS=Homo sapiens GN=ADGRF3 PE=2 SV | 420 | PLRVPIsITII | 0.578 | 0.219 | 0.035 | 0.277 |
| sp\|Q8IZF5\|AGRF3_HUMAN Adhesion G-protein coupled receptor F3 OS=Homo sapiens GN=ADGRF3 PE=2 SV | 422 | RVPISItIIQD | 0.249 | 0.138 | -0.281 | 0.035 |
| sp\|Q8IZF5\|AGRF3_HUMAN Adhesion G-protein coupled receptor F3 OS=Homo sapiens GN=ADGRF3 PE=2 SV | 430 | IQDGDItCPED | 0.62 | 0.487 | 0.251 | 0.453 |
| sp\|Q8IZF5\|AGRF3_HUMAN Adhesion G-protein coupled receptor F3 OS=Homo sapiens GN=ADGRF3 PE=2 SV | 436 | TCPEDAsVLTW | 0.087 | -0.134 | -1.485 | -0.511 |
| sp\|Q8IZF5\|AGRF3_HUMAN Adhesion G-protein coupled receptor F3 OS=Homo sapiens GN=ADGRF3 PE=2 SV | 439 | EDASVLtWNVT | 0.051 | -0.166 | -1.161 | -0.425 |
| sp\|Q8IZF5\|AGRF3_HUMAN Adhesion G-protein coupled receptor F3 OS=Homo sapiens GN=ADGRF3 PE=2 SV | 443 | VLTWNVtKAGH | 0.354 | -0.209 | -0.401 | -0.085 |
| sp\|Q8IZF5\|AGRF3_HUMAN Adhesion G-protein coupled receptor F3 OS=Homo sapiens GN=ADGRF3 PE=2 SV | 456 | QAPCPEsKRGI | 0.134 | -0.356 | -1.008 | -0.41 |
| sp\|Q8IZF5\|AGRF3_HUMAN Adhesion G-protein coupled receptor F3 OS=Homo sapiens GN=ADGRF3 PE=2 SV | 476 | VWGPVHsSCTD | 0.13 | -0.126 | -1.096 | -0.364 |
| sp\|Q8IZF5\|AGRF3_HUMAN Adhesion G-protein coupled receptor F3 OS=Homo sapiens GN=ADGRF3 PE=2 SV | 477 | WGPVHSsCTDA | 0.15 | -0.09 | -0.918 | -0.286 |
| sp\|Q8IZF5\|AGRF3_HUMAN Adhesion G-protein coupled receptor F3 OS=Homo sapiens GN=ADGRF3 PE=2 SV | 479 | PVHSSCtDARL | 0.291 | 0.076 | -0.864 | -0.166 |
| sp\|Q8IZF5\|AGRF3_HUMAN Adhesion G-protein coupled receptor F3 OS=Homo sapiens GN=ADGRF3 PE=2 SV | 488 | RLLALFtRTKL | 0.251 | -0.021 | -0.796 | -0.189 |
| sp\|Q8IZF5\|AGRF3_HUMAN Adhesion G-protein coupled receptor F3 OS=Homo sapiens GN=ADGRF3 PE=2 SV | 490 | LALFTRtKLLQ | 0.067 | -0.123 | -1.328 | -0.461 |
| sp\|Q8IZF5\|AGRF3_HUMAN Adhesion G-protein coupled receptor F3 OS=Homo sapiens GN=ADGRF3 PE=2 SV | 499 | LQAGQGsPAEE | 0.03 | -0.53 | -1.654 | -0.718 |
| sp\|Q8IZF5\|AGRF3_HUMAN Adhesion G-protein coupled receptor F3 OS=Homo sapiens GN=ADGRF3 PE=2 SV | 519 | GQAAEAsSPSD | 0.492 | 0.387 | -0.117 | 0.254 |
| sp\|Q8IZF5\|AGRF3_HUMAN Adhesion G-protein coupled receptor F3 OS=Homo sapiens GN=ADGRF3 PE=2 SV | 520 | QAAEASsPSDL | 0.054 | -0.375 | -1.391 | -0.571 |
| sp\|Q8IZF5\|AGRF3_HUMAN Adhesion G-protein coupled receptor F3 OS=Homo sapiens GN=ADGRF3 PE=2 SV | 522 | AEASSPsDLLT | 0.185 | -0.029 | -0.913 | -0.252 |
| sp\|Q8IZF5\|AGRF3_HUMAN Adhesion G-protein coupled receptor F3 OS=Homo sapiens GN=ADGRF3 PE=2 SV | 526 | SPSDLLtLLST | 0.034 | -0.284 | -1.742 | -0.664 |
| sp\|Q8IZF5\|AGRF3_HUMAN Adhesion G-protein coupled receptor F3 OS=Homo sapiens GN=ADGRF3 PE=2 SV | 529 | DLLTLLsTMKY | 0.184 | -0.071 | -1.177 | -0.355 |
| sp\|Q8IZF5\|AGRF3_HUMAN Adhesion G-protein coupled receptor F3 OS=Homo sapiens GN=ADGRF3 PE=2 SV | 530 | LLTLLStMKYV | 0.116 | -0.008 | -1.287 | -0.393 |
| sp\|Q8IZF5\|AGRF3_HUMAN Adhesion G-protein coupled receptor F3 OS=Homo sapiens GN=ADGRF3 PE=2 SV | 557 | KNLLIAtDKVL | 0.182 | -0.038 | -0.776 | -0.211 |
| sp\|Q8IZF5\|AGRF3_HUMAN Adhesion G-protein coupled receptor F3 OS=Homo sapiens GN=ADGRF3 PE=2 SV | 565 | KVLDMDtRSLW | 0.063 | -0.319 | -1.596 | -0.617 |
| sp\|Q8IZF5\|AGRF3_HUMAN Adhesion G-protein coupled receptor F3 OS=Homo sapiens GN=ADGRF3 PE=2 SV | 567 | LDMDTRsLWTL | 0.165 | 0.089 | -0.813 | -0.186 |
| sp\|Q8IZF5\|AGRF3_HUMAN Adhesion G-protein coupled receptor F3 OS=Homo sapiens GN=ADGRF3 PE=2 SV | 570 | DTRSLWtLAQA | 0.506 | 0.028 | -0.454 | 0.027 |
| sp\|Q8IZF5\|AGRF3_HUMAN Adhesion G-protein coupled receptor F3 OS=Homo sapiens GN=ADGRF3 PE=2 SV | 581 | RKPWAGsTLLL | 0.248 | 0.005 | -0.748 | -0.165 |
| sp\|Q8IZF5\|AGRF3_HUMAN Adhesion G-protein coupled receptor F3 OS=Homo sapiens GN=ADGRF3 PE=2 SV | 582 | KPWAGStLLLA | 0.079 | -0.108 | -1.157 | -0.395 |
| sp\|Q8IZF5\|AGRF3_HUMAN Adhesion G-protein coupled receptor F3 OS=Homo sapiens GN=ADGRF3 PE=2 SV | 589 | LLLAVEtLACS | 0.299 | 0.027 | -0.603 | -0.092 |
| sp\|Q8IZF5\|AGRF3_HUMAN Adhesion G-protein coupled receptor F3 OS=Homo sapiens GN=ADGRF3 PE=2 SV | 593 | VETLACsLCPQ | 0.3 | -0.1 | -0.84 | -0.213 |
| sp\|Q8IZF5\|AGRF3_HUMAN Adhesion G-protein coupled receptor F3 OS=Homo sapiens GN=ADGRF3 PE=2 SV | 604 | DHPFAFsLPNV | 0.789 | 0.667 | -0.107 | 0.45 |
| sp\|Q8IZF5\|AGRF3_HUMAN Adhesion G-protein coupled receptor F3 OS=Homo sapiens GN=ADGRF3 PE=2 SV | 612 | PNVLLQsQLFG | 0.049 | -0.291 | -1.707 | -0.65 |
| sp\|Q8IZF5\|AGRF3_HUMAN Adhesion G-protein coupled receptor F3 OS=Homo sapiens GN=ADGRF3 PE=2 SV | 618 | SQLFGPtFPAD | 0.51 | 0.453 | 0.2 | 0.388 |
| sp\|Q8IZF5\|AGRF3_HUMAN Adhesion G-protein coupled receptor F3 OS=Homo sapiens GN=ADGRF3 PE=2 SV | 624 | TFPADYsISFP | 0.037 | -0.342 | -1.426 | -0.577 |
| sp\|Q8IZF5\|AGRF3_HUMAN Adhesion G-protein coupled receptor F3 OS=Homo sapiens GN=ADGRF3 PE=2 SV | 626 | PADYSIsFPTR | 0.841 | 0.718 | 0.847 | 0.802 |
| sp\|Q8IZF5\|AGRF3_HUMAN Adhesion G-protein coupled receptor F3 OS=Homo sapiens GN=ADGRF3 PE=2 SV | 629 | YSISFPtRPPL | 0.278 | 0.187 | -0.598 | -0.044 |
| sp\|Q8IZF5\|AGRF3_HUMAN Adhesion G-protein coupled receptor F3 OS=Homo sapiens GN=ADGRF3 PE=2 SV | 641 | AQIPRHsLAPL | 0.381 | 0.189 | -0.235 | 0.112 |
| sp\|Q8IZF5\|AGRF3_HUMAN Adhesion G-protein coupled receptor F3 OS=Homo sapiens GN=ADGRF3 PE=2 SV | 650 | PLVRNGtEISI | 0.655 | 0.969 | 0.615 | 0.746 |
| sp\|Q8IZF5\|AGRF3_HUMAN Adhesion G-protein coupled receptor F3 OS=Homo sapiens GN=ADGRF3 PE=2 SV | 653 | RNGTEIsITSL | 0.08 | -0.236 | -1.337 | -0.498 |
| sp\|Q8IZF5\|AGRF3_HUMAN Adhesion G-protein coupled receptor F3 OS=Homo sapiens GN=ADGRF3 PE=2 SV | 655 | GTEISItSLVL | 0.349 | 0.2 | -0.506 | 0.014 |
| sp\|Q8IZF5\|AGRF3_HUMAN Adhesion G-protein coupled receptor F3 OS=Homo sapiens GN=ADGRF3 PE=2 SV | 656 | TEISITsLVLR | 0.208 | -0.003 | -0.591 | -0.129 |

| | | | | | | |
|---|---|---|---|---|---|---|
| sp\|Q8IZF5\|AGRF3_HUMAN Adhesion G-protein coupled receptor F3 OS=Homo sapiens GN=ADGRF3 PE=2 SV | 668 | LDHLLPsNYGQ | 0.033 | -0.419 | -1.842 | -0.743 |
| sp\|Q8IZF5\|AGRF3_HUMAN Adhesion G-protein coupled receptor F3 OS=Homo sapiens GN=ADGRF3 PE=2 SV | 677 | GQGLGDsLYAT | 0.147 | -0.06 | -0.777 | -0.23 |
| sp\|Q8IZF5\|AGRF3_HUMAN Adhesion G-protein coupled receptor F3 OS=Homo sapiens GN=ADGRF3 PE=2 SV | 681 | GDSLYAtPGLV | 0.148 | -0.293 | -1.336 | -0.494 |
| sp\|Q8IZF5\|AGRF3_HUMAN Adhesion G-protein coupled receptor F3 OS=Homo sapiens GN=ADGRF3 PE=2 SV | 689 | GLVLVIsIMAG | 0.417 | 0.131 | -0.405 | 0.048 |
| sp\|Q8IZF5\|AGRF3_HUMAN Adhesion G-protein coupled receptor F3 OS=Homo sapiens GN=ADGRF3 PE=2 SV | 698 | AGDRAFsQGEV | 0.762 | 0.892 | 0.223 | 0.626 |
| sp\|Q8IZF5\|AGRF3_HUMAN Adhesion G-protein coupled receptor F3 OS=Homo sapiens GN=ADGRF3 PE=2 SV | 709 | IMDFGNtDGSP | 0.132 | 0.012 | -1.132 | -0.329 |
| sp\|Q8IZF5\|AGRF3_HUMAN Adhesion G-protein coupled receptor F3 OS=Homo sapiens GN=ADGRF3 PE=2 SV | 712 | FGNTDGsPHCV | 0.065 | -0.356 | -1.794 | -0.695 |
| sp\|Q8IZF5\|AGRF3_HUMAN Adhesion G-protein coupled receptor F3 OS=Homo sapiens GN=ADGRF3 PE=2 SV | 721 | CVFWDHsLFQG | 0.592 | 0.183 | 0.166 | 0.314 |
| sp\|Q8IZF5\|AGRF3_HUMAN Adhesion G-protein coupled receptor F3 OS=Homo sapiens GN=ADGRF3 PE=2 SV | 730 | QGRGGWsKEGC | 0.161 | -0.071 | -0.793 | -0.234 |
| sp\|Q8IZF5\|AGRF3_HUMAN Adhesion G-protein coupled receptor F3 OS=Homo sapiens GN=ADGRF3 PE=2 SV | 740 | CQAQVAsASPT | 0.186 | -0.115 | -0.699 | -0.209 |
| sp\|Q8IZF5\|AGRF3_HUMAN Adhesion G-protein coupled receptor F3 OS=Homo sapiens GN=ADGRF3 PE=2 SV | 742 | AQVASAsPTAQ | 0.227 | -0.153 | -0.719 | -0.215 |
| sp\|Q8IZF5\|AGRF3_HUMAN Adhesion G-protein coupled receptor F3 OS=Homo sapiens GN=ADGRF3 PE=2 SV | 744 | VASASPtAQCL | 0.305 | 0.115 | -0.535 | -0.038 |
| sp\|Q8IZF5\|AGRF3_HUMAN Adhesion G-protein coupled receptor F3 OS=Homo sapiens GN=ADGRF3 PE=2 SV | 753 | CLCQHLtAFSV | 0.328 | 0.127 | -0.682 | -0.076 |
| sp\|Q8IZF5\|AGRF3_HUMAN Adhesion G-protein coupled receptor F3 OS=Homo sapiens GN=ADGRF3 PE=2 SV | 756 | QHLTAFsVLMS | 0.122 | -0.074 | -1.244 | -0.399 |
| sp\|Q8IZF5\|AGRF3_HUMAN Adhesion G-protein coupled receptor F3 OS=Homo sapiens GN=ADGRF3 PE=2 SV | 760 | AFSVLMsPHTV | 0.085 | -0.314 | -1.492 | -0.574 |
| sp\|Q8IZF5\|AGRF3_HUMAN Adhesion G-protein coupled receptor F3 OS=Homo sapiens GN=ADGRF3 PE=2 SV | 763 | VLMSPHtVPEE | 0.661 | 0.552 | 0.252 | 0.488 |
| sp\|Q8IZF5\|AGRF3_HUMAN Adhesion G-protein coupled receptor F3 OS=Homo sapiens GN=ADGRF3 PE=2 SV | 774 | PALALLtQVGL | 0.081 | -0.247 | -1.353 | -0.506 |
| sp\|Q8IZF5\|AGRF3_HUMAN Adhesion G-protein coupled receptor F3 OS=Homo sapiens GN=ADGRF3 PE=2 SV | 781 | QVGLGAsILAL | 0.063 | -0.069 | -1.149 | -0.385 |
| sp\|Q8IZF5\|AGRF3_HUMAN Adhesion G-protein coupled receptor F3 OS=Homo sapiens GN=ADGRF3 PE=2 SV | 805 | VVRNKIsYFRH | 0.124 | -0.115 | -1.035 | -0.342 |
| sp\|Q8IZF5\|AGRF3_HUMAN Adhesion G-protein coupled receptor F3 OS=Homo sapiens GN=ADGRF3 PE=2 SV | 824 | CLLAADtCFLG | 0.534 | 0.172 | -0.191 | 0.172 |
| sp\|Q8IZF5\|AGRF3_HUMAN Adhesion G-protein coupled receptor F3 OS=Homo sapiens GN=ADGRF3 PE=2 SV | 833 | LGAPFLsPGPR | 0.056 | -0.477 | -1.211 | -0.544 |
| sp\|Q8IZF5\|AGRF3_HUMAN Adhesion G-protein coupled receptor F3 OS=Homo sapiens GN=ADGRF3 PE=2 SV | 838 | LSPGPRsPLCL | 0.021 | -0.504 | -1.877 | -0.787 |
| sp\|Q8IZF5\|AGRF3_HUMAN Adhesion G-protein coupled receptor F3 OS=Homo sapiens GN=ADGRF3 PE=2 SV | 855 | HFLYLAtFFWM | 0.351 | 0.164 | -0.013 | 0.167 |
| sp\|Q8IZF5\|AGRF3_HUMAN Adhesion G-protein coupled receptor F3 OS=Homo sapiens GN=ADGRF3 PE=2 SV | 901 | LGLAGVtLGLY | 0.496 | 0.046 | -0.048 | 0.165 |
| sp\|Q8IZF5\|AGRF3_HUMAN Adhesion G-protein coupled receptor F3 OS=Homo sapiens GN=ADGRF3 PE=2 SV | 928 | KGGALYtFVGP | 0.091 | -0.148 | -0.939 | -0.332 |
| sp\|Q8IZF5\|AGRF3_HUMAN Adhesion G-protein coupled receptor F3 OS=Homo sapiens GN=ADGRF3 PE=2 SV | 955 | LKLLRPsLSEG | 0.59 | 0.184 | -0.013 | 0.254 |
| sp\|Q8IZF5\|AGRF3_HUMAN Adhesion G-protein coupled receptor F3 OS=Homo sapiens GN=ADGRF3 PE=2 SV | 957 | LLRPSLsEGPP | 0.53 | 0.431 | 0.19 | 0.384 |
| sp\|Q8IZF5\|AGRF3_HUMAN Adhesion G-protein coupled receptor F3 OS=Homo sapiens GN=ADGRF3 PE=2 SV | 979 | KALLILtPIFG | 0.03 | -0.53 | -1.621 | -0.707 |
| sp\|Q8IZF5\|AGRF3_HUMAN Adhesion G-protein coupled receptor F3 OS=Homo sapiens GN=ADGRF3 PE=2 SV | 985 | TPIFGLtWGLG | 0.092 | -0.017 | -1.119 | -0.348 |
| sp\|Q8IZF5\|AGRF3_HUMAN Adhesion G-protein coupled receptor F3 OS=Homo sapiens GN=ADGRF3 PE=2 SV | 992 | WGLGLAtLLEE | 0.224 | -0.054 | -0.58 | -0.137 |
| sp\|Q8IZF5\|AGRF3_HUMAN Adhesion G-protein coupled receptor F3 OS=Homo sapiens GN=ADGRF3 PE=2 SV | 998 | TLLEEVsTVPH | 0.119 | -0.224 | -1.262 | -0.456 |
| sp\|Q8IZF5\|AGRF3_HUMAN Adhesion G-protein coupled receptor F3 OS=Homo sapiens GN=ADGRF3 PE=2 SV | 999 | LLEEVStVPHY | 0.438 | 0.432 | -0.089 | 0.26 |
| sp\|Q8IZF5\|AGRF3_HUMAN Adhesion G-protein coupled receptor F3 OS=Homo sapiens GN=ADGRF3 PE=2 SV | 1006 | VPHYIFtILNT | 0.058 | -0.159 | -1.191 | -0.431 |
| sp\|Q8IZF5\|AGRF3_HUMAN Adhesion G-protein coupled receptor F3 OS=Homo sapiens GN=ADGRF3 PE=2 SV | 1010 | IFTILNtLQGV | 0.486 | 0.082 | -0.27 | 0.099 |
| sp\|Q8IZF5\|AGRF3_HUMAN Adhesion G-protein coupled receptor F3 OS=Homo sapiens GN=ADGRF3 PE=2 SV | 1042 | CRAQAPsSTIS | 0.421 | 0.159 | -0.285 | 0.098 |
| sp\|Q8IZF5\|AGRF3_HUMAN Adhesion G-protein coupled receptor F3 OS=Homo sapiens GN=ADGRF3 PE=2 SV | 1043 | RAQAPSsTISL | 0.057 | -0.2 | -1.215 | -0.453 |
| sp\|Q8IZF5\|AGRF3_HUMAN Adhesion G-protein coupled receptor F3 OS=Homo sapiens GN=ADGRF3 PE=2 SV | 1044 | AQAPSStISLV | 0.09 | -0.086 | -0.983 | -0.326 |
| sp\|Q8IZF5\|AGRF3_HUMAN Adhesion G-protein coupled receptor F3 OS=Homo sapiens GN=ADGRF3 PE=2 SV | 1046 | APSSTIsLVSC | 0.079 | -0.204 | -1.324 | -0.483 |
| sp\|Q8IZF5\|AGRF3_HUMAN Adhesion G-protein coupled receptor F3 OS=Homo sapiens GN=ADGRF3 PE=2 SV | 1049 | STISLVsCCLQ | 0.099 | -0.242 | -1.615 | -0.586 |
| sp\|Q8IZF5\|AGRF3_HUMAN Adhesion G-protein coupled receptor F3 OS=Homo sapiens GN=ADGRF3 PE=2 SV | 1056 | CCLQILsCASK | 0.133 | -0.065 | -0.802 | -0.245 |
| sp\|Q8IZF5\|AGRF3_HUMAN Adhesion G-protein coupled receptor F3 OS=Homo sapiens GN=ADGRF3 PE=2 SV | 1059 | QILSCAsKSMS | 0.166 | -0.133 | -0.956 | -0.308 |
| sp\|Q8IZF5\|AGRF3_HUMAN Adhesion G-protein coupled receptor F3 OS=Homo sapiens GN=ADGRF3 PE=2 SV | 1061 | LSCASKsMSEG | 0.425 | 0.222 | -0.223 | 0.141 |
| sp\|Q8IZF5\|AGRF3_HUMAN Adhesion G-protein coupled receptor F3 OS=Homo sapiens GN=ADGRF3 PE=2 SV | 1063 | CASKSMsEGIP | 0.74 | 0.396 | 0.357 | 0.498 |
| sp\|Q8IZF5\|AGRF3_HUMAN Adhesion G-protein coupled receptor F3 OS=Homo sapiens GN=ADGRF3 PE=2 SV | 1070 | EGIPWPsSEDM | 0.126 | -0.123 | -0.836 | -0.278 |
| sp\|Q8IZF5\|AGRF3_HUMAN Adhesion G-protein coupled receptor F3 OS=Homo sapiens GN=ADGRF3 PE=2 SV | 1071 | GIPWPSsEDMG | 0.191 | -0.092 | -0.769 | -0.223 |
| sp\|Q8IZF5\|AGRF3_HUMAN Adhesion G-protein coupled receptor F3 OS=Homo sapiens GN=ADGRF3 PE=2 SV | 1076 | SSEDMGtARS- | 0.059 | -0.344 | -1.694 | -0.66 |
| sp\|Q8IZF5\|AGRF3_HUMAN Adhesion G-protein coupled receptor F3 OS=Homo sapiens GN=ADGRF3 PE=2 SV | 1079 | DMGTARs---- | 0.155 | 0.208 | -1.17 | -0.269 |
| sp\|Q8IZF4\|AGRG5_HUMAN Adhesion G-protein coupled receptor G5 OS=Homo sapiens GN=ADGRG5 PE=2 SV | 315 | VPGSACtALAA | 0.192 | -0.046 | -1.051 | -0.302 |
| sp\|Q8IZF4\|AGRG5_HUMAN Adhesion G-protein coupled receptor G5 OS=Homo sapiens GN=ADGRG5 PE=2 SV | 327 | LHYALLsCLTW | 0.04 | -0.152 | -1.72 | -0.611 |

| | | | | | | |
|---|---|---|---|---|---|---|
| sp\|Q8IZF4\|AGRG5_HUMAN Adhesion G-protein coupled receptor G5 OS=Homo sapiens GN=ADGRG5 PE=2 SV | 330 | ALLSCLtWMAI | 0.42 | 0.166 | -0.234 | 0.117 |
| sp\|Q8IZF4\|AGRG5_HUMAN Adhesion G-protein coupled receptor G5 OS=Homo sapiens GN=ADGRG5 PE=2 SV | 373 | ALLVLLsLSVK | 0.129 | -0.083 | -0.987 | -0.314 |
| sp\|Q8IZF4\|AGRG5_HUMAN Adhesion G-protein coupled receptor G5 OS=Homo sapiens GN=ADGRG5 PE=2 SV | 375 | LVLLSLsVKSS | 0.086 | 0.052 | -0.836 | -0.233 |
| sp\|Q8IZF4\|AGRG5_HUMAN Adhesion G-protein coupled receptor G5 OS=Homo sapiens GN=ADGRG5 PE=2 SV | 378 | LSLSVKsSVYG | 0.147 | -0.182 | -0.925 | -0.32 |
| sp\|Q8IZF4\|AGRG5_HUMAN Adhesion G-protein coupled receptor G5 OS=Homo sapiens GN=ADGRG5 PE=2 SV | 379 | SLSVKSsVYGP | 0.052 | -0.265 | -1.479 | -0.564 |
| sp\|Q8IZF4\|AGRG5_HUMAN Adhesion G-protein coupled receptor G5 OS=Homo sapiens GN=ADGRG5 PE=2 SV | 385 | SVYGPCtIPVF | 0.205 | 0.294 | -0.543 | -0.015 |
| sp\|Q8IZF4\|AGRG5_HUMAN Adhesion G-protein coupled receptor G5 OS=Homo sapiens GN=ADGRG5 PE=2 SV | 391 | TIPVFDsWENG | 0.086 | -0.062 | -1.576 | -0.517 |
| sp\|Q8IZF4\|AGRG5_HUMAN Adhesion G-protein coupled receptor G5 OS=Homo sapiens GN=ADGRG5 PE=2 SV | 396 | DSWENGtGFQN | 0.089 | -0.149 | -1.202 | -0.421 |
| sp\|Q8IZF4\|AGRG5_HUMAN Adhesion G-protein coupled receptor G5 OS=Homo sapiens GN=ADGRG5 PE=2 SV | 402 | TGFQNMsICWV | 0.203 | -0.05 | -0.672 | -0.173 |
| sp\|Q8IZF4\|AGRG5_HUMAN Adhesion G-protein coupled receptor G5 OS=Homo sapiens GN=ADGRG5 PE=2 SV | 408 | SICWVRsPVVH | 0.048 | -0.44 | -1.903 | -0.765 |
| sp\|Q8IZF4\|AGRG5_HUMAN Adhesion G-protein coupled receptor G5 OS=Homo sapiens GN=ADGRG5 PE=2 SV | 413 | RSPVVHsVLVM | 0.119 | -0.019 | -1.153 | -0.351 |
| sp\|Q8IZF4\|AGRG5_HUMAN Adhesion G-protein coupled receptor G5 OS=Homo sapiens GN=ADGRG5 PE=2 SV | 423 | MGYGGLtSLFN | 0.037 | -0.218 | -1.54 | -0.574 |
| sp\|Q8IZF4\|AGRG5_HUMAN Adhesion G-protein coupled receptor G5 OS=Homo sapiens GN=ADGRG5 PE=2 SV | 424 | GYGGLTsLFNL | 0.318 | 0.173 | -0.466 | 0.008 |
| sp\|Q8IZF4\|AGRG5_HUMAN Adhesion G-protein coupled receptor G5 OS=Homo sapiens GN=ADGRG5 PE=2 SV | 437 | LAWALWtLRRL | 0.226 | -0.091 | -0.774 | -0.213 |
| sp\|Q8IZF4\|AGRG5_HUMAN Adhesion G-protein coupled receptor G5 OS=Homo sapiens GN=ADGRG5 PE=2 SV | 449 | ERADAPsVRAC | 0.275 | 0.149 | -0.52 | -0.032 |
| sp\|Q8IZF4\|AGRG5_HUMAN Adhesion G-protein coupled receptor G5 OS=Homo sapiens GN=ADGRG5 PE=2 SV | 456 | VRACHDtVTVL | 0.382 | 0.187 | -0.601 | -0.011 |
| sp\|Q8IZF4\|AGRG5_HUMAN Adhesion G-protein coupled receptor G5 OS=Homo sapiens GN=ADGRG5 PE=2 SV | 458 | ACHDTVtVLGL | 0.172 | -0.032 | -1.069 | -0.31 |
| sp\|Q8IZF4\|AGRG5_HUMAN Adhesion G-protein coupled receptor G5 OS=Homo sapiens GN=ADGRG5 PE=2 SV | 463 | VTVLGLtVLLG | 0.059 | -0.206 | -1.524 | -0.557 |
| sp\|Q8IZF4\|AGRG5_HUMAN Adhesion G-protein coupled receptor G5 OS=Homo sapiens GN=ADGRG5 PE=2 SV | 468 | LTVLLGtTWAL | 0.137 | -0.055 | -1.123 | -0.347 |
| sp\|Q8IZF4\|AGRG5_HUMAN Adhesion G-protein coupled receptor G5 OS=Homo sapiens GN=ADGRG5 PE=2 SV | 469 | TVLLGTtWALA | 0.066 | -0.115 | -1.125 | -0.391 |
| sp\|Q8IZF4\|AGRG5_HUMAN Adhesion G-protein coupled receptor G5 OS=Homo sapiens GN=ADGRG5 PE=2 SV | 476 | WALAFFsFGVF | 0.282 | 0.002 | -0.437 | -0.051 |
| sp\|Q8IZF4\|AGRG5_HUMAN Adhesion G-protein coupled receptor G5 OS=Homo sapiens GN=ADGRG5 PE=2 SV | 489 | PQLFLFtILNS | 0.083 | -0.069 | -1.143 | -0.376 |
| sp\|Q8IZF4\|AGRG5_HUMAN Adhesion G-protein coupled receptor G5 OS=Homo sapiens GN=ADGRG5 PE=2 SV | 493 | LFTILNsLYGF | 0.272 | 0.048 | -0.341 | -0.007 |
| sp\|Q8IZF4\|AGRG5_HUMAN Adhesion G-protein coupled receptor G5 OS=Homo sapiens GN=ADGRG5 PE=2 SV | 505 | LFLWFCsQRCR | 0.323 | -0.103 | -0.756 | -0.179 |
| sp\|Q8IZF4\|AGRG5_HUMAN Adhesion G-protein coupled receptor G5 OS=Homo sapiens GN=ADGRG5 PE=2 SV | 510 | CSQRCRsEAEA | 0.753 | 0.907 | 0.493 | 0.718 |
| sp\|Q8IZF4\|AGRG5_HUMAN Adhesion G-protein coupled receptor G5 OS=Homo sapiens GN=ADGRG5 PE=2 SV | 522 | AQIEAFsSSQT | 0.075 | -0.151 | -1.145 | -0.407 |
| sp\|Q8IZF4\|AGRG5_HUMAN Adhesion G-protein coupled receptor G5 OS=Homo sapiens GN=ADGRG5 PE=2 SV | 523 | QIEAFSsSQTT | 0.096 | -0.221 | -1.136 | -0.42 |
| sp\|Q8IZF4\|AGRG5_HUMAN Adhesion G-protein coupled receptor G5 OS=Homo sapiens GN=ADGRG5 PE=2 SV | 524 | IEAFSSsQTTQ | 0.267 | -0.034 | -0.805 | -0.191 |
| sp\|Q8IZF4\|AGRG5_HUMAN Adhesion G-protein coupled receptor G5 OS=Homo sapiens GN=ADGRG5 PE=2 SV | 526 | AFSSSQtTQ-- | 0.329 | 0.223 | -0.349 | 0.068 |
| sp\|Q8IZF4\|AGRG5_HUMAN Adhesion G-protein coupled receptor G5 OS=Homo sapiens GN=ADGRG5 PE=2 SV | 527 | FSSSQTtQ--- | 0.104 | -0.025 | -1.313 | -0.411 |
| sp\|Q8IZF3\|AGRF4_HUMAN Adhesion G protein-coupled receptor F4 OS=Homo sapiens GN=ADGRF4 PE=2 SV | 322 | VNGLVLsVVLP | 0.046 | -0.269 | -1.541 | -0.588 |
| sp\|Q8IZF3\|AGRF4_HUMAN Adhesion G protein-coupled receptor F4 OS=Homo sapiens GN=ADGRF4 PE=2 SV | 335 | LQEIILtFEKI | 0.129 | -0.013 | -0.479 | -0.121 |
| sp\|Q8IZF3\|AGRF4_HUMAN Adhesion G protein-coupled receptor F4 OS=Homo sapiens GN=ADGRF4 PE=2 SV | 342 | FEKINKtRNAR | 0.466 | 0.16 | 0.002 | 0.209 |
| sp\|Q8IZF3\|AGRF4_HUMAN Adhesion G protein-coupled receptor F4 OS=Homo sapiens GN=ADGRF4 PE=2 SV | 354 | QCVGWHsKKRR | 0.285 | 0.068 | -0.623 | -0.09 |
| sp\|Q8IZF3\|AGRF4_HUMAN Adhesion G protein-coupled receptor F4 OS=Homo sapiens GN=ADGRF4 PE=2 SV | 381 | KCRCNYtSVVM | 0.454 | 0.275 | -0.36 | 0.123 |
| sp\|Q8IZF3\|AGRF4_HUMAN Adhesion G protein-coupled receptor F4 OS=Homo sapiens GN=ADGRF4 PE=2 SV | 382 | CRCNYTsVVMS | 0.221 | 0.17 | -0.559 | -0.056 |
| sp\|Q8IZF3\|AGRF4_HUMAN Adhesion G protein-coupled receptor F4 OS=Homo sapiens GN=ADGRF4 PE=2 SV | 386 | YTSVVMsFSIL | 0.243 | 0.07 | -0.688 | -0.125 |
| sp\|Q8IZF3\|AGRF4_HUMAN Adhesion G protein-coupled receptor F4 OS=Homo sapiens GN=ADGRF4 PE=2 SV | 388 | SVVMSFsILMS | 0.09 | -0.036 | -1.062 | -0.336 |
| sp\|Q8IZF3\|AGRF4_HUMAN Adhesion G protein-coupled receptor F4 OS=Homo sapiens GN=ADGRF4 PE=2 SV | 392 | SFSILMsSKSM | 0.06 | -0.124 | -1.276 | -0.447 |
| sp\|Q8IZF3\|AGRF4_HUMAN Adhesion G protein-coupled receptor F4 OS=Homo sapiens GN=ADGRF4 PE=2 SV | 393 | FSILMSsKSMT | 0.057 | -0.306 | -1.417 | -0.555 |
| sp\|Q8IZF3\|AGRF4_HUMAN Adhesion G protein-coupled receptor F4 OS=Homo sapiens GN=ADGRF4 PE=2 SV | 395 | ILMSSKsMTDK | 0.734 | 0.428 | 0.418 | 0.527 |
| sp\|Q8IZF3\|AGRF4_HUMAN Adhesion G protein-coupled receptor F4 OS=Homo sapiens GN=ADGRF4 PE=2 SV | 397 | MSSKSMtDKVL | 0.697 | 0.456 | 0.243 | 0.465 |
| sp\|Q8IZF3\|AGRF4_HUMAN Adhesion G protein-coupled receptor F4 OS=Homo sapiens GN=ADGRF4 PE=2 SV | 405 | KVLDYItCIGL | 0.195 | 0.028 | -0.665 | -0.147 |
| sp\|Q8IZF3\|AGRF4_HUMAN Adhesion G protein-coupled receptor F4 OS=Homo sapiens GN=ADGRF4 PE=2 SV | 410 | ITCIGLsVSIL | 0.091 | -0.166 | -1.225 | -0.433 |
| sp\|Q8IZF3\|AGRF4_HUMAN Adhesion G protein-coupled receptor F4 OS=Homo sapiens GN=ADGRF4 PE=2 SV | 412 | CIGLSVsILSL | 0.251 | 0.206 | -0.42 | 0.012 |
| sp\|Q8IZF3\|AGRF4_HUMAN Adhesion G protein-coupled receptor F4 OS=Homo sapiens GN=ADGRF4 PE=2 SV | 415 | LSVSILsLVLC | 0.091 | -0.175 | -1.009 | -0.364 |
| sp\|Q8IZF3\|AGRF4_HUMAN Adhesion G protein-coupled receptor F4 OS=Homo sapiens GN=ADGRF4 PE=2 SV | 425 | CLIIEAtVWSR | 0.359 | 0.16 | -0.568 | -0.016 |
| sp\|Q8IZF3\|AGRF4_HUMAN Adhesion G protein-coupled receptor F4 OS=Homo sapiens GN=ADGRF4 PE=2 SV | 428 | IEATVWsRVVV | 0.183 | -0.19 | -1.277 | -0.428 |
| sp\|Q8IZF3\|AGRF4_HUMAN Adhesion G protein-coupled receptor F4 OS=Homo sapiens GN=ADGRF4 PE=2 SV | 433 | WSRVVVtEISY | 0.331 | 0.162 | -0.417 | 0.025 |

| | | | | | | |
|---|---|---|---|---|---|---|
| sp\|Q8IZF3\|AGRF4_HUMAN Adhesion G protein-coupled receptor F4 OS=Homo sapiens GN=ADGRF4 PE=2 SV | 436 | VVVTEIsYMRH | 0.07 | -0.378 | -1.747 | -0.685 |
| sp\|Q8IZF3\|AGRF4_HUMAN Adhesion G protein-coupled receptor F4 OS=Homo sapiens GN=ADGRF4 PE=2 SV | 449 | IVNIAVsLLTA | 0.443 | 0.064 | -0.255 | 0.084 |
| sp\|Q8IZF3\|AGRF4_HUMAN Adhesion G protein-coupled receptor F4 OS=Homo sapiens GN=ADGRF4 PE=2 SV | 452 | IAVSLLtANVW | 0.084 | -0.134 | -1.285 | -0.445 |
| sp\|Q8IZF3\|AGRF4_HUMAN Adhesion G protein-coupled receptor F4 OS=Homo sapiens GN=ADGRF4 PE=2 SV | 461 | VWFIIGsHFNI | 0.216 | 0.035 | -0.696 | -0.148 |
| sp\|Q8IZF3\|AGRF4_HUMAN Adhesion G protein-coupled receptor F4 OS=Homo sapiens GN=ADGRF4 PE=2 SV | 477 | NMCVAVtFFSH | 0.207 | 0.058 | -0.818 | -0.184 |
| sp\|Q8IZF3\|AGRF4_HUMAN Adhesion G protein-coupled receptor F4 OS=Homo sapiens GN=ADGRF4 PE=2 SV | 480 | VAVTFFsHFFY | 0.174 | -0.139 | -1.098 | -0.354 |
| sp\|Q8IZF3\|AGRF4_HUMAN Adhesion G protein-coupled receptor F4 OS=Homo sapiens GN=ADGRF4 PE=2 SV | 486 | SHFFYLsLFFW | 0.131 | -0.047 | -1.184 | -0.367 |
| sp\|Q8IZF3\|AGRF4_HUMAN Adhesion G protein-coupled receptor F4 OS=Homo sapiens GN=ADGRF4 PE=2 SV | 512 | FRRMMKsRMMV | 0.575 | 0.342 | -0.282 | 0.212 |
| sp\|Q8IZF3\|AGRF4_HUMAN Adhesion G protein-coupled receptor F4 OS=Homo sapiens GN=ADGRF4 PE=2 SV | 532 | PLIIAVtTVAI | 0.655 | 0.321 | 0.173 | 0.383 |
| sp\|Q8IZF3\|AGRF4_HUMAN Adhesion G protein-coupled receptor F4 OS=Homo sapiens GN=ADGRF4 PE=2 SV | 533 | LIIAVTtVAIT | 0.181 | -0.075 | -0.83 | -0.241 |
| sp\|Q8IZF3\|AGRF4_HUMAN Adhesion G protein-coupled receptor F4 OS=Homo sapiens GN=ADGRF4 PE=2 SV | 537 | VTTVAItEPEK | 0.706 | 0.507 | 0.217 | 0.477 |
| sp\|Q8IZF3\|AGRF4_HUMAN Adhesion G protein-coupled receptor F4 OS=Homo sapiens GN=ADGRF4 PE=2 SV | 556 | WLNWDNtKALL | 0.312 | -0.003 | -0.502 | -0.064 |
| sp\|Q8IZF3\|AGRF4_HUMAN Adhesion G protein-coupled receptor F4 OS=Homo sapiens GN=ADGRF4 PE=2 SV | 584 | LVVAVNtQRPS | 0.117 | -0.229 | -1.31 | -0.474 |
| sp\|Q8IZF3\|AGRF4_HUMAN Adhesion G protein-coupled receptor F4 OS=Homo sapiens GN=ADGRF4 PE=2 SV | 588 | VNTQRPsIGSS | 0.15 | -0.123 | -0.673 | -0.215 |
| sp\|Q8IZF3\|AGRF4_HUMAN Adhesion G protein-coupled receptor F4 OS=Homo sapiens GN=ADGRF4 PE=2 SV | 591 | QRPSIGsSKSQ | 0.031 | -0.191 | -1.592 | -0.584 |
| sp\|Q8IZF3\|AGRF4_HUMAN Adhesion G protein-coupled receptor F4 OS=Homo sapiens GN=ADGRF4 PE=2 SV | 592 | RPSIGSsKSQD | 0.066 | -0.261 | -1.284 | -0.493 |
| sp\|Q8IZF3\|AGRF4_HUMAN Adhesion G protein-coupled receptor F4 OS=Homo sapiens GN=ADGRF4 PE=2 SV | 594 | SIGSSKsQDVV | 0.297 | 0.2 | -0.719 | -0.074 |
| sp\|Q8IZF3\|AGRF4_HUMAN Adhesion G protein-coupled receptor F4 OS=Homo sapiens GN=ADGRF4 PE=2 SV | 604 | VIIMRIsKNVA | 0.447 | 0.164 | -0.14 | 0.157 |
| sp\|Q8IZF3\|AGRF4_HUMAN Adhesion G protein-coupled receptor F4 OS=Homo sapiens GN=ADGRF4 PE=2 SV | 611 | KNVAILtPLLG | 0.028 | -0.537 | -1.671 | -0.727 |
| sp\|Q8IZF3\|AGRF4_HUMAN Adhesion G protein-coupled receptor F4 OS=Homo sapiens GN=ADGRF4 PE=2 SV | 617 | TPLLGLtWGFG | 0.058 | -0.163 | -1.345 | -0.483 |
| sp\|Q8IZF3\|AGRF4_HUMAN Adhesion G protein-coupled receptor F4 OS=Homo sapiens GN=ADGRF4 PE=2 SV | 624 | WGFGIAtLIEG | 0.368 | 0.086 | 0.019 | 0.158 |
| sp\|Q8IZF3\|AGRF4_HUMAN Adhesion G protein-coupled receptor F4 OS=Homo sapiens GN=ADGRF4 PE=2 SV | 629 | ATLIEGtSLTF | 0.047 | -0.329 | -1.767 | -0.683 |
| sp\|Q8IZF3\|AGRF4_HUMAN Adhesion G protein-coupled receptor F4 OS=Homo sapiens GN=ADGRF4 PE=2 SV | 630 | TLIEGTsLTFH | 0.109 | -0.148 | -1.132 | -0.39 |
| sp\|Q8IZF3\|AGRF4_HUMAN Adhesion G protein-coupled receptor F4 OS=Homo sapiens GN=ADGRF4 PE=2 SV | 632 | IEGTSLtFHII | 0.485 | 0.216 | -0.154 | 0.182 |
| sp\|Q8IZF3\|AGRF4_HUMAN Adhesion G protein-coupled receptor F4 OS=Homo sapiens GN=ADGRF4 PE=2 SV | 653 | FILLFGtIMDH | 0.148 | -0.079 | -1.156 | -0.362 |
| sp\|Q8IZF3\|AGRF4_HUMAN Adhesion G protein-coupled receptor F4 OS=Homo sapiens GN=ADGRF4 PE=2 SV | 668 | ALRMRMsSLKG | 0.625 | 0.429 | -0.111 | 0.314 |
| sp\|Q8IZF3\|AGRF4_HUMAN Adhesion G protein-coupled receptor F4 OS=Homo sapiens GN=ADGRF4 PE=2 SV | 669 | LRMRMSsLKGK | 0.643 | 0.957 | 0.731 | 0.777 |
| sp\|Q8IZF3\|AGRF4_HUMAN Adhesion G protein-coupled receptor F4 OS=Homo sapiens GN=ADGRF4 PE=2 SV | 674 | SSLKGKsRAAE | 0.16 | -0.216 | -0.891 | -0.316 |
| sp\|Q8IZF3\|AGRF4_HUMAN Adhesion G protein-coupled receptor F4 OS=Homo sapiens GN=ADGRF4 PE=2 SV | 681 | RAAENAsLGPT | 0.34 | 0.007 | -0.319 | 0.009 |
| sp\|Q8IZF3\|AGRF4_HUMAN Adhesion G protein-coupled receptor F4 OS=Homo sapiens GN=ADGRF4 PE=2 SV | 685 | NASLGPtNGSK | 0.061 | -0.335 | -1.523 | -0.599 |
| sp\|Q8IZF3\|AGRF4_HUMAN Adhesion G protein-coupled receptor F4 OS=Homo sapiens GN=ADGRF4 PE=2 SV | 688 | LGPTNGsKLMN | 0.062 | -0.308 | -1.414 | -0.553 |
| sp\|Q8IZF2\|AGRF5_HUMAN Adhesion G protein-coupled receptor F5 OS=Homo sapiens GN=ADGRF5 PE=1 SV | 316 | QLEIQNsSRFS | 0.122 | -0.123 | -1.177 | -0.393 |
| sp\|Q8IZF2\|AGRF5_HUMAN Adhesion G protein-coupled receptor F5 OS=Homo sapiens GN=ADGRF5 PE=1 SV | 317 | LEIQNSsRFSI | 0.108 | -0.174 | -0.953 | -0.34 |
| sp\|Q8IZF2\|AGRF5_HUMAN Adhesion G protein-coupled receptor F5 OS=Homo sapiens GN=ADGRF5 PE=1 SV | 320 | QNSSRFsIYTA | 0.147 | -0.121 | -0.704 | -0.226 |
| sp\|Q8IZF2\|AGRF5_HUMAN Adhesion G protein-coupled receptor F5 OS=Homo sapiens GN=ADGRF5 PE=1 SV | 323 | SRFSIYtALFN | 0.093 | 0.121 | -1.152 | -0.313 |
| sp\|Q8IZF2\|AGRF5_HUMAN Adhesion G protein-coupled receptor F5 OS=Homo sapiens GN=ADGRF5 PE=1 SV | 330 | ALFNNMtSVSK | 0.187 | 0.032 | -0.674 | -0.152 |
| sp\|Q8IZF2\|AGRF5_HUMAN Adhesion G protein-coupled receptor F5 OS=Homo sapiens GN=ADGRF5 PE=1 SV | 331 | LFNNMTsVSKL | 0.09 | -0.131 | -0.843 | -0.295 |
| sp\|Q8IZF2\|AGRF5_HUMAN Adhesion G protein-coupled receptor F5 OS=Homo sapiens GN=ADGRF5 PE=1 SV | 333 | NNMTSVsKLTI | 0.396 | 0.125 | -0.125 | 0.132 |
| sp\|Q8IZF2\|AGRF5_HUMAN Adhesion G protein-coupled receptor F5 OS=Homo sapiens GN=ADGRF5 PE=1 SV | 336 | TSVSKLtIHNI | 0.074 | -0.215 | -1.314 | -0.485 |
| sp\|Q8IZF2\|AGRF5_HUMAN Adhesion G protein-coupled receptor F5 OS=Homo sapiens GN=ADGRF5 PE=1 SV | 341 | LTIHNItPGDA | 0.227 | -0.274 | -0.68 | -0.242 |
| sp\|Q8IZF2\|AGRF5_HUMAN Adhesion G protein-coupled receptor F5 OS=Homo sapiens GN=ADGRF5 PE=1 SV | 388 | CDNNPVsLNCC | 0.25 | -0.09 | -0.489 | -0.11 |
| sp\|Q8IZF2\|AGRF5_HUMAN Adhesion G protein-coupled receptor F5 OS=Homo sapiens GN=ADGRF5 PE=1 SV | 393 | VSLNCCsQGNV | 0.324 | -0.06 | -1.043 | -0.26 |
| sp\|Q8IZF2\|AGRF5_HUMAN Adhesion G protein-coupled receptor F5 OS=Homo sapiens GN=ADGRF5 PE=1 SV | 400 | QGNVNWsKVEW | 0.122 | -0.13 | -0.955 | -0.321 |
| sp\|Q8IZF2\|AGRF5_HUMAN Adhesion G protein-coupled receptor F5 OS=Homo sapiens GN=ADGRF5 PE=1 SV | 415 | KINIPGtPETD | 0.044 | -0.463 | -1.624 | -0.681 |
| sp\|Q8IZF2\|AGRF5_HUMAN Adhesion G protein-coupled receptor F5 OS=Homo sapiens GN=ADGRF5 PE=1 SV | 418 | IPGTPEtDIDS | 0.12 | -0.192 | -1.148 | -0.407 |
| sp\|Q8IZF2\|AGRF5_HUMAN Adhesion G protein-coupled receptor F5 OS=Homo sapiens GN=ADGRF5 PE=1 SV | 422 | PETDIDsSCSR | 0.074 | -0.277 | -1.429 | -0.544 |
| sp\|Q8IZF2\|AGRF5_HUMAN Adhesion G protein-coupled receptor F5 OS=Homo sapiens GN=ADGRF5 PE=1 SV | 423 | ETDIDSsCSRY | 0.068 | -0.226 | -1.659 | -0.606 |
| sp\|Q8IZF2\|AGRF5_HUMAN Adhesion G protein-coupled receptor F5 OS=Homo sapiens GN=ADGRF5 PE=1 SV | 425 | DIDSSCsRYTL | 0.385 | 0.114 | -0.693 | -0.065 |
| sp\|Q8IZF2\|AGRF5_HUMAN Adhesion G protein-coupled receptor F5 OS=Homo sapiens GN=ADGRF5 PE=1 SV | 428 | SSCSRYtLKAD | 0.463 | 0.199 | -0.029 | 0.211 |

| | | | | | | |
|---|---|---|---|---|---|---|
| sp\|Q8IZF2\|AGRF5_HUMAN Adhesion G protein-coupled receptor F5 OS=Homo sapiens GN=ADGRF5 PE=1 SV | 434 | TLKADGtQCPS | 0.088 | -0.321 | -1.423 | -0.552 |
| sp\|Q8IZF2\|AGRF5_HUMAN Adhesion G protein-coupled receptor F5 OS=Homo sapiens GN=ADGRF5 PE=1 SV | 438 | DGTQCPsGSSG | 0.147 | -0.247 | -0.937 | -0.346 |
| sp\|Q8IZF2\|AGRF5_HUMAN Adhesion G protein-coupled receptor F5 OS=Homo sapiens GN=ADGRF5 PE=1 SV | 440 | TQCPSGsSGTT | 0.157 | -0.061 | -0.714 | -0.206 |
| sp\|Q8IZF2\|AGRF5_HUMAN Adhesion G protein-coupled receptor F5 OS=Homo sapiens GN=ADGRF5 PE=1 SV | 441 | QCPSGSsGTTV | 0.051 | -0.36 | -1.825 | -0.711 |
| sp\|Q8IZF2\|AGRF5_HUMAN Adhesion G protein-coupled receptor F5 OS=Homo sapiens GN=ADGRF5 PE=1 SV | 443 | PSGSSGtTVIY | 0.207 | -0.004 | -0.867 | -0.221 |
| sp\|Q8IZF2\|AGRF5_HUMAN Adhesion G protein-coupled receptor F5 OS=Homo sapiens GN=ADGRF5 PE=1 SV | 444 | SGSSGTtVIYT | 0.046 | -0.347 | -1.418 | -0.573 |
| sp\|Q8IZF2\|AGRF5_HUMAN Adhesion G protein-coupled receptor F5 OS=Homo sapiens GN=ADGRF5 PE=1 SV | 448 | GTTVIYtCEFI | 0.2 | 0.098 | -0.552 | -0.085 |
| sp\|Q8IZF2\|AGRF5_HUMAN Adhesion G protein-coupled receptor F5 OS=Homo sapiens GN=ADGRF5 PE=1 SV | 453 | YTCEFIsAYGA | 0.178 | -0.11 | -1.048 | -0.327 |
| sp\|Q8IZF2\|AGRF5_HUMAN Adhesion G protein-coupled receptor F5 OS=Homo sapiens GN=ADGRF5 PE=1 SV | 460 | AYGARGsANIK | 0.317 | 0.006 | -0.773 | -0.15 |
| sp\|Q8IZF2\|AGRF5_HUMAN Adhesion G protein-coupled receptor F5 OS=Homo sapiens GN=ADGRF5 PE=1 SV | 466 | SANIKVtFISV | 0.09 | -0.165 | -1.424 | -0.5 |
| sp\|Q8IZF2\|AGRF5_HUMAN Adhesion G protein-coupled receptor F5 OS=Homo sapiens GN=ADGRF5 PE=1 SV | 469 | IKVTFIsVANL | 0.343 | 0.032 | -0.679 | -0.101 |
| sp\|Q8IZF2\|AGRF5_HUMAN Adhesion G protein-coupled receptor F5 OS=Homo sapiens GN=ADGRF5 PE=1 SV | 474 | ISVANLtITPD | 0.154 | -0.275 | -0.834 | -0.318 |
| sp\|Q8IZF2\|AGRF5_HUMAN Adhesion G protein-coupled receptor F5 OS=Homo sapiens GN=ADGRF5 PE=1 SV | 476 | VANLTItPDPI | 0.087 | -0.362 | -1.251 | -0.509 |
| sp\|Q8IZF2\|AGRF5_HUMAN Adhesion G protein-coupled receptor F5 OS=Homo sapiens GN=ADGRF5 PE=1 SV | 481 | ITPDPIsVSEG | 0.156 | -0.224 | -1.059 | -0.376 |
| sp\|Q8IZF2\|AGRF5_HUMAN Adhesion G protein-coupled receptor F5 OS=Homo sapiens GN=ADGRF5 PE=1 SV | 483 | PDPISVsEGQN | 0.462 | 0.201 | -0.153 | 0.17 |
| sp\|Q8IZF2\|AGRF5_HUMAN Adhesion G protein-coupled receptor F5 OS=Homo sapiens GN=ADGRF5 PE=1 SV | 489 | SEGQNFsIKCI | 0.127 | -0.056 | -0.782 | -0.237 |
| sp\|Q8IZF2\|AGRF5_HUMAN Adhesion G protein-coupled receptor F5 OS=Homo sapiens GN=ADGRF5 PE=1 SV | 494 | FSIKCIsDVSN | 0.376 | 0.099 | -0.648 | -0.058 |
| sp\|Q8IZF2\|AGRF5_HUMAN Adhesion G protein-coupled receptor F5 OS=Homo sapiens GN=ADGRF5 PE=1 SV | 497 | KCISDVsNYDE | 0.275 | -0.097 | -1.035 | -0.286 |
| sp\|Q8IZF2\|AGRF5_HUMAN Adhesion G protein-coupled receptor F5 OS=Homo sapiens GN=ADGRF5 PE=1 SV | 506 | DEVYWNtSAGI | 0.306 | -0.027 | -0.718 | -0.146 |
| sp\|Q8IZF2\|AGRF5_HUMAN Adhesion G protein-coupled receptor F5 OS=Homo sapiens GN=ADGRF5 PE=1 SV | 507 | EVYWNTsAGIK | 0.263 | -0.037 | -0.49 | -0.088 |
| sp\|Q8IZF2\|AGRF5_HUMAN Adhesion G protein-coupled receptor F5 OS=Homo sapiens GN=ADGRF5 PE=1 SV | 518 | IYQRFYtTRRY | 0.8 | 0.887 | 0.475 | 0.721 |
| sp\|Q8IZF2\|AGRF5_HUMAN Adhesion G protein-coupled receptor F5 OS=Homo sapiens GN=ADGRF5 PE=1 SV | 519 | YQRFYTtRRYL | 0.341 | 0.108 | -0.384 | 0.022 |
| sp\|Q8IZF2\|AGRF5_HUMAN Adhesion G protein-coupled receptor F5 OS=Homo sapiens GN=ADGRF5 PE=1 SV | 528 | YLDGAEsVLTV | 0.206 | 0.021 | -0.964 | -0.246 |
| sp\|Q8IZF2\|AGRF5_HUMAN Adhesion G protein-coupled receptor F5 OS=Homo sapiens GN=ADGRF5 PE=1 SV | 531 | GAESVLtVKTS | 0.103 | -0.229 | -1.231 | -0.452 |
| sp\|Q8IZF2\|AGRF5_HUMAN Adhesion G protein-coupled receptor F5 OS=Homo sapiens GN=ADGRF5 PE=1 SV | 534 | SVLTVKtSTRE | 0.163 | -0.227 | -1.216 | -0.427 |
| sp\|Q8IZF2\|AGRF5_HUMAN Adhesion G protein-coupled receptor F5 OS=Homo sapiens GN=ADGRF5 PE=1 SV | 535 | VLTVKTsTREW | 0.151 | -0.09 | -0.842 | -0.26 |
| sp\|Q8IZF2\|AGRF5_HUMAN Adhesion G protein-coupled receptor F5 OS=Homo sapiens GN=ADGRF5 PE=1 SV | 536 | LTVKTStREWN | 0.108 | -0.111 | -1.151 | -0.385 |
| sp\|Q8IZF2\|AGRF5_HUMAN Adhesion G protein-coupled receptor F5 OS=Homo sapiens GN=ADGRF5 PE=1 SV | 542 | TREWNGtYHCI | 0.288 | 0.114 | -0.477 | -0.025 |
| sp\|Q8IZF2\|AGRF5_HUMAN Adhesion G protein-coupled receptor F5 OS=Homo sapiens GN=ADGRF5 PE=1 SV | 552 | IFRYKNsYSIA | 0.391 | 0.13 | -0.299 | 0.074 |
| sp\|Q8IZF2\|AGRF5_HUMAN Adhesion G protein-coupled receptor F5 OS=Homo sapiens GN=ADGRF5 PE=1 SV | 554 | RYKNSYsIATK | 0.375 | 0.208 | 0.038 | 0.207 |
| sp\|Q8IZF2\|AGRF5_HUMAN Adhesion G protein-coupled receptor F5 OS=Homo sapiens GN=ADGRF5 PE=1 SV | 557 | NSYSIAtKDVI | 0.277 | -0.001 | -0.341 | -0.022 |
| sp\|Q8IZF2\|AGRF5_HUMAN Adhesion G protein-coupled receptor F5 OS=Homo sapiens GN=ADGRF5 PE=1 SV | 579 | VDPLEAtVSCS | 0.047 | -0.262 | -1.746 | -0.654 |
| sp\|Q8IZF2\|AGRF5_HUMAN Adhesion G protein-coupled receptor F5 OS=Homo sapiens GN=ADGRF5 PE=1 SV | 581 | PLEATVsCSGS | 0.292 | 0.022 | -0.575 | -0.087 |
| sp\|Q8IZF2\|AGRF5_HUMAN Adhesion G protein-coupled receptor F5 OS=Homo sapiens GN=ADGRF5 PE=1 SV | 583 | EATVSCsGSHH | 0.073 | -0.178 | -1.226 | -0.444 |
| sp\|Q8IZF2\|AGRF5_HUMAN Adhesion G protein-coupled receptor F5 OS=Homo sapiens GN=ADGRF5 PE=1 SV | 585 | TVSCSGsHHIK | 0.341 | 0.018 | -0.626 | -0.089 |
| sp\|Q8IZF2\|AGRF5_HUMAN Adhesion G protein-coupled receptor F5 OS=Homo sapiens GN=ADGRF5 PE=1 SV | 601 | DGDYKVtFHTG | 0.406 | -0.033 | -0.457 | -0.028 |
| sp\|Q8IZF2\|AGRF5_HUMAN Adhesion G protein-coupled receptor F5 OS=Homo sapiens GN=ADGRF5 PE=1 SV | 604 | YKVTFHtGSSS | 0.114 | -0.056 | -1.15 | -0.364 |
| sp\|Q8IZF2\|AGRF5_HUMAN Adhesion G protein-coupled receptor F5 OS=Homo sapiens GN=ADGRF5 PE=1 SV | 606 | VTFHTGsSSLP | 0.043 | -0.307 | -1.75 | -0.671 |
| sp\|Q8IZF2\|AGRF5_HUMAN Adhesion G protein-coupled receptor F5 OS=Homo sapiens GN=ADGRF5 PE=1 SV | 607 | TFHTGSsSLPA | 0.03 | -0.395 | -1.725 | -0.697 |
| sp\|Q8IZF2\|AGRF5_HUMAN Adhesion G protein-coupled receptor F5 OS=Homo sapiens GN=ADGRF5 PE=1 SV | 608 | FHTGSSsLPAA | 0.802 | 0.835 | 0.807 | 0.815 |
| sp\|Q8IZF2\|AGRF5_HUMAN Adhesion G protein-coupled receptor F5 OS=Homo sapiens GN=ADGRF5 PE=1 SV | 629 | KHNFNAsSVSW | 0.084 | -0.113 | -1.541 | -0.523 |
| sp\|Q8IZF2\|AGRF5_HUMAN Adhesion G protein-coupled receptor F5 OS=Homo sapiens GN=ADGRF5 PE=1 SV | 630 | HNFNASsVSWC | 0.065 | -0.153 | -1.097 | -0.395 |
| sp\|Q8IZF2\|AGRF5_HUMAN Adhesion G protein-coupled receptor F5 OS=Homo sapiens GN=ADGRF5 PE=1 SV | 632 | FNASSVsWCSK | 0.214 | 0.02 | -0.551 | -0.106 |
| sp\|Q8IZF2\|AGRF5_HUMAN Adhesion G protein-coupled receptor F5 OS=Homo sapiens GN=ADGRF5 PE=1 SV | 635 | SSVSWCsKTVD | 0.228 | -0.126 | -1.018 | -0.305 |
| sp\|Q8IZF2\|AGRF5_HUMAN Adhesion G protein-coupled receptor F5 OS=Homo sapiens GN=ADGRF5 PE=1 SV | 637 | VSWCSKtVDVC | 0.417 | 0.269 | -0.226 | 0.153 |
| sp\|Q8IZF2\|AGRF5_HUMAN Adhesion G protein-coupled receptor F5 OS=Homo sapiens GN=ADGRF5 PE=1 SV | 645 | DVCCHFtNAAN | 0.328 | 0.048 | -0.877 | -0.167 |
| sp\|Q8IZF2\|AGRF5_HUMAN Adhesion G protein-coupled receptor F5 OS=Homo sapiens GN=ADGRF5 PE=1 SV | 651 | TNAANNsVWSP | 0.131 | -0.183 | -0.935 | -0.329 |
| sp\|Q8IZF2\|AGRF5_HUMAN Adhesion G protein-coupled receptor F5 OS=Homo sapiens GN=ADGRF5 PE=1 SV | 654 | ANNSVWsPSMK | 0.036 | -0.747 | -2.06 | -0.924 |
| sp\|Q8IZF2\|AGRF5_HUMAN Adhesion G protein-coupled receptor F5 OS=Homo sapiens GN=ADGRF5 PE=1 SV | 656 | NSVWSPsMKLN | 0.303 | 0.157 | -0.447 | 0.004 |

| | | | | | | | |
|---|---|---|---|---|---|---|---|
| sp\|Q8IZF2\|AGRF5_HUMAN Adhesion G protein-coupled receptor F5 OS=Homo sapiens GN=ADGRF5 PE=1 SV | 668 | VPGENItCQDP | 0.126 | -0.076 | -0.994 | -0.315 |
| sp\|Q8IZF2\|AGRF5_HUMAN Adhesion G protein-coupled receptor F5 OS=Homo sapiens GN=ADGRF5 PE=1 SV | 690 | QKLCRFsNVPS | 0.344 | 0.087 | -0.565 | -0.045 |
| sp\|Q8IZF2\|AGRF5_HUMAN Adhesion G protein-coupled receptor F5 OS=Homo sapiens GN=ADGRF5 PE=1 SV | 694 | RFSNVPsSPES | 0.497 | 0.378 | -0.116 | 0.253 |
| sp\|Q8IZF2\|AGRF5_HUMAN Adhesion G protein-coupled receptor F5 OS=Homo sapiens GN=ADGRF5 PE=1 SV | 695 | FSNVPSsPESP | 0.013 | -0.631 | -2.198 | -0.939 |
| sp\|Q8IZF2\|AGRF5_HUMAN Adhesion G protein-coupled receptor F5 OS=Homo sapiens GN=ADGRF5 PE=1 SV | 698 | VPSSPEsPIGG | 0.035 | -0.671 | -1.684 | -0.773 |
| sp\|Q8IZF2\|AGRF5_HUMAN Adhesion G protein-coupled receptor F5 OS=Homo sapiens GN=ADGRF5 PE=1 SV | 703 | ESPIGGtITYK | 0.03 | -0.455 | -1.803 | -0.743 |
| sp\|Q8IZF2\|AGRF5_HUMAN Adhesion G protein-coupled receptor F5 OS=Homo sapiens GN=ADGRF5 PE=1 SV | 705 | PIGGTItYKCV | 0.099 | 0.044 | -1.097 | -0.318 |
| sp\|Q8IZF2\|AGRF5_HUMAN Adhesion G protein-coupled receptor F5 OS=Homo sapiens GN=ADGRF5 PE=1 SV | 711 | TYKCVGsQWEE | 0.315 | -0.099 | -1.06 | -0.281 |
| sp\|Q8IZF2\|AGRF5_HUMAN Adhesion G protein-coupled receptor F5 OS=Homo sapiens GN=ADGRF5 PE=1 SV | 722 | KRNDCIsAPIN | 0.772 | 0.729 | 0.035 | 0.512 |
| sp\|Q8IZF2\|AGRF5_HUMAN Adhesion G protein-coupled receptor F5 OS=Homo sapiens GN=ADGRF5 PE=1 SV | 727 | ISAPINsLLQM | 0.326 | -0.076 | -0.198 | 0.017 |
| sp\|Q8IZF2\|AGRF5_HUMAN Adhesion G protein-coupled receptor F5 OS=Homo sapiens GN=ADGRF5 PE=1 SV | 738 | AKALIKsPSQD | 0.212 | -0.284 | -0.545 | -0.206 |
| sp\|Q8IZF2\|AGRF5_HUMAN Adhesion G protein-coupled receptor F5 OS=Homo sapiens GN=ADGRF5 PE=1 SV | 740 | ALIKSPsQDEM | 0.663 | 0.259 | -0.062 | 0.287 |
| sp\|Q8IZF2\|AGRF5_HUMAN Adhesion G protein-coupled receptor F5 OS=Homo sapiens GN=ADGRF5 PE=1 SV | 747 | QDEMLPtYLKD | 0.046 | -0.4 | -1.542 | -0.632 |
| sp\|Q8IZF2\|AGRF5_HUMAN Adhesion G protein-coupled receptor F5 OS=Homo sapiens GN=ADGRF5 PE=1 SV | 753 | TYLKDLsISID | 0.185 | -0.135 | -0.811 | -0.254 |
| sp\|Q8IZF2\|AGRF5_HUMAN Adhesion G protein-coupled receptor F5 OS=Homo sapiens GN=ADGRF5 PE=1 SV | 755 | LKDLSIsIDKA | 0.428 | 0.164 | -0.093 | 0.166 |
| sp\|Q8IZF2\|AGRF5_HUMAN Adhesion G protein-coupled receptor F5 OS=Homo sapiens GN=ADGRF5 PE=1 SV | 764 | KAEHEIsSSPG | 0.079 | -0.359 | -1.448 | -0.576 |
| sp\|Q8IZF2\|AGRF5_HUMAN Adhesion G protein-coupled receptor F5 OS=Homo sapiens GN=ADGRF5 PE=1 SV | 765 | AEHEISsSPGS | 0.13 | 0.155 | -0.953 | -0.223 |
| sp\|Q8IZF2\|AGRF5_HUMAN Adhesion G protein-coupled receptor F5 OS=Homo sapiens GN=ADGRF5 PE=1 SV | 766 | EHEISSsPGSL | 0.065 | -0.162 | -1.24 | -0.446 |
| sp\|Q8IZF2\|AGRF5_HUMAN Adhesion G protein-coupled receptor F5 OS=Homo sapiens GN=ADGRF5 PE=1 SV | 769 | ISSSPGsLGAI | 0.369 | -0.081 | -0.134 | 0.051 |
| sp\|Q8IZF2\|AGRF5_HUMAN Adhesion G protein-coupled receptor F5 OS=Homo sapiens GN=ADGRF5 PE=1 SV | 781 | NILDLLsTVPT | 0.073 | -0.169 | -1.345 | -0.48 |
| sp\|Q8IZF2\|AGRF5_HUMAN Adhesion G protein-coupled receptor F5 OS=Homo sapiens GN=ADGRF5 PE=1 SV | 782 | ILDLLStVPTQ | 0.437 | 0.346 | -0.343 | 0.147 |
| sp\|Q8IZF2\|AGRF5_HUMAN Adhesion G protein-coupled receptor F5 OS=Homo sapiens GN=ADGRF5 PE=1 SV | 785 | LLSTVPtQVNS | 0.151 | -0.11 | -1.299 | -0.419 |
| sp\|Q8IZF2\|AGRF5_HUMAN Adhesion G protein-coupled receptor F5 OS=Homo sapiens GN=ADGRF5 PE=1 SV | 789 | VPTQVNsEMMT | 0.252 | -0.085 | -0.789 | -0.207 |
| sp\|Q8IZF2\|AGRF5_HUMAN Adhesion G protein-coupled receptor F5 OS=Homo sapiens GN=ADGRF5 PE=1 SV | 793 | VNSEMMtHVLS | 0.112 | -0.137 | -1.032 | -0.352 |
| sp\|Q8IZF2\|AGRF5_HUMAN Adhesion G protein-coupled receptor F5 OS=Homo sapiens GN=ADGRF5 PE=1 SV | 797 | MMTHVLsTVNV | 0.13 | 0.11 | -1.236 | -0.332 |
| sp\|Q8IZF2\|AGRF5_HUMAN Adhesion G protein-coupled receptor F5 OS=Homo sapiens GN=ADGRF5 PE=1 SV | 798 | MTHVLStVNVI | 0.119 | -0.026 | -1 | -0.302 |
| sp\|Q8IZF2\|AGRF5_HUMAN Adhesion G protein-coupled receptor F5 OS=Homo sapiens GN=ADGRF5 PE=1 SV | 810 | GKPVLNtWKVL | 0.197 | 0.133 | -0.886 | -0.185 |
| sp\|Q8IZF2\|AGRF5_HUMAN Adhesion G protein-coupled receptor F5 OS=Homo sapiens GN=ADGRF5 PE=1 SV | 819 | VLQQQWtNQSS | 0.141 | -0.066 | -1.042 | -0.322 |
| sp\|Q8IZF2\|AGRF5_HUMAN Adhesion G protein-coupled receptor F5 OS=Homo sapiens GN=ADGRF5 PE=1 SV | 822 | QQWTNQsSQLL | 0.122 | 0.064 | -0.687 | -0.167 |
| sp\|Q8IZF2\|AGRF5_HUMAN Adhesion G protein-coupled receptor F5 OS=Homo sapiens GN=ADGRF5 PE=1 SV | 823 | QWTNQSsQLLH | 0.028 | -0.291 | -1.844 | -0.702 |
| sp\|Q8IZF2\|AGRF5_HUMAN Adhesion G protein-coupled receptor F5 OS=Homo sapiens GN=ADGRF5 PE=1 SV | 828 | SSQLLHsVERF | 0.103 | -0.113 | -1.402 | -0.471 |
| sp\|Q8IZF2\|AGRF5_HUMAN Adhesion G protein-coupled receptor F5 OS=Homo sapiens GN=ADGRF5 PE=1 SV | 833 | HSVERFsQALQ | 0.115 | -0.011 | -1.138 | -0.345 |
| sp\|Q8IZF2\|AGRF5_HUMAN Adhesion G protein-coupled receptor F5 OS=Homo sapiens GN=ADGRF5 PE=1 SV | 838 | FSQALQsGDSP | 0.075 | -0.153 | -1.164 | -0.414 |
| sp\|Q8IZF2\|AGRF5_HUMAN Adhesion G protein-coupled receptor F5 OS=Homo sapiens GN=ADGRF5 PE=1 SV | 841 | ALQSGDsPPLS | 0.239 | 0.069 | -0.862 | -0.185 |
| sp\|Q8IZF2\|AGRF5_HUMAN Adhesion G protein-coupled receptor F5 OS=Homo sapiens GN=ADGRF5 PE=1 SV | 845 | GDSPPLsFSQT | 0.057 | -0.367 | -1.213 | -0.508 |
| sp\|Q8IZF2\|AGRF5_HUMAN Adhesion G protein-coupled receptor F5 OS=Homo sapiens GN=ADGRF5 PE=1 SV | 847 | SPPLSFsQTNV | 0.181 | 0.038 | -1.414 | -0.398 |
| sp\|Q8IZF2\|AGRF5_HUMAN Adhesion G protein-coupled receptor F5 OS=Homo sapiens GN=ADGRF5 PE=1 SV | 849 | PLSFSQtNVQM | 0.498 | 0.237 | -0.081 | 0.218 |
| sp\|Q8IZF2\|AGRF5_HUMAN Adhesion G protein-coupled receptor F5 OS=Homo sapiens GN=ADGRF5 PE=1 SV | 854 | QTNVQMsSMVI | 0.143 | 0.051 | -1.065 | -0.29 |
| sp\|Q8IZF2\|AGRF5_HUMAN Adhesion G protein-coupled receptor F5 OS=Homo sapiens GN=ADGRF5 PE=1 SV | 855 | TNVQMSsMVIK | 0.096 | -0.226 | -1.141 | -0.424 |
| sp\|Q8IZF2\|AGRF5_HUMAN Adhesion G protein-coupled receptor F5 OS=Homo sapiens GN=ADGRF5 PE=1 SV | 860 | SSMVIKsSHPE | 0.078 | -0.197 | -1.089 | -0.403 |
| sp\|Q8IZF2\|AGRF5_HUMAN Adhesion G protein-coupled receptor F5 OS=Homo sapiens GN=ADGRF5 PE=1 SV | 861 | SMVIKSsHPET | 0.188 | 0.374 | -0.751 | -0.063 |
| sp\|Q8IZF2\|AGRF5_HUMAN Adhesion G protein-coupled receptor F5 OS=Homo sapiens GN=ADGRF5 PE=1 SV | 865 | KSSHPEtYQQR | 0.089 | -0.37 | -1.15 | -0.477 |
| sp\|Q8IZF2\|AGRF5_HUMAN Adhesion G protein-coupled receptor F5 OS=Homo sapiens GN=ADGRF5 PE=1 SV | 886 | NVVIDKsYLEN | 0.123 | -0.03 | -0.97 | -0.292 |
| sp\|Q8IZF2\|AGRF5_HUMAN Adhesion G protein-coupled receptor F5 OS=Homo sapiens GN=ADGRF5 PE=1 SV | 893 | YLENLQsDSSI | 0.14 | -0.004 | -0.732 | -0.199 |
| sp\|Q8IZF2\|AGRF5_HUMAN Adhesion G protein-coupled receptor F5 OS=Homo sapiens GN=ADGRF5 PE=1 SV | 895 | ENLQSDsSIVT | 0.135 | -0.059 | -0.625 | -0.183 |
| sp\|Q8IZF2\|AGRF5_HUMAN Adhesion G protein-coupled receptor F5 OS=Homo sapiens GN=ADGRF5 PE=1 SV | 896 | NLQSDSsIVTM | 0.12 | -0.132 | -0.958 | -0.323 |
| sp\|Q8IZF2\|AGRF5_HUMAN Adhesion G protein-coupled receptor F5 OS=Homo sapiens GN=ADGRF5 PE=1 SV | 899 | SDSSIVtMAFP | 0.04 | -0.363 | -1.652 | -0.658 |
| sp\|Q8IZF2\|AGRF5_HUMAN Adhesion G protein-coupled receptor F5 OS=Homo sapiens GN=ADGRF5 PE=1 SV | 904 | VTMAFPtLQAI | 0.475 | 0.07 | -0.132 | 0.138 |
| sp\|Q8IZF2\|AGRF5_HUMAN Adhesion G protein-coupled receptor F5 OS=Homo sapiens GN=ADGRF5 PE=1 SV | 921 | ENNFAEsLVMT | 0.178 | -0.115 | -0.878 | -0.272 |

| | | | | | | |
|---|---|---|---|---|---|---|
| sp\|Q8IZF2\|AGRF5_HUMAN Adhesion G protein-coupled receptor F5 OS=Homo sapiens GN=ADGRF5 PE=1 SV | 925 | AESLVMtTTVS | 0.203 | -0.033 | -1.16 | -0.33 |
| sp\|Q8IZF2\|AGRF5_HUMAN Adhesion G protein-coupled receptor F5 OS=Homo sapiens GN=ADGRF5 PE=1 SV | 926 | ESLVMTtTVSH | 0.03 | -0.386 | -1.615 | -0.657 |
| sp\|Q8IZF2\|AGRF5_HUMAN Adhesion G protein-coupled receptor F5 OS=Homo sapiens GN=ADGRF5 PE=1 SV | 927 | SLVMTTtVSHN | 0.085 | -0.101 | -1.117 | -0.378 |
| sp\|Q8IZF2\|AGRF5_HUMAN Adhesion G protein-coupled receptor F5 OS=Homo sapiens GN=ADGRF5 PE=1 SV | 929 | VMTTTVsHNTT | 0.203 | 0.02 | -0.612 | -0.13 |
| sp\|Q8IZF2\|AGRF5_HUMAN Adhesion G protein-coupled receptor F5 OS=Homo sapiens GN=ADGRF5 PE=1 SV | 932 | TTVSHNtTMPF | 0.155 | -0.143 | -1.182 | -0.39 |
| sp\|Q8IZF2\|AGRF5_HUMAN Adhesion G protein-coupled receptor F5 OS=Homo sapiens GN=ADGRF5 PE=1 SV | 933 | TVSHNTtMPFR | 0.376 | 0.422 | -0.159 | 0.213 |
| sp\|Q8IZF2\|AGRF5_HUMAN Adhesion G protein-coupled receptor F5 OS=Homo sapiens GN=ADGRF5 PE=1 SV | 939 | TMPFRIsMTFK | 0.188 | 0.12 | -0.973 | -0.222 |
| sp\|Q8IZF2\|AGRF5_HUMAN Adhesion G protein-coupled receptor F5 OS=Homo sapiens GN=ADGRF5 PE=1 SV | 941 | PFRISMtFKNN | 0.812 | 0.751 | 0.638 | 0.734 |
| sp\|Q8IZF2\|AGRF5_HUMAN Adhesion G protein-coupled receptor F5 OS=Homo sapiens GN=ADGRF5 PE=1 SV | 946 | MTFKNNsPSGG | 0.29 | -0.241 | -0.825 | -0.259 |
| sp\|Q8IZF2\|AGRF5_HUMAN Adhesion G protein-coupled receptor F5 OS=Homo sapiens GN=ADGRF5 PE=1 SV | 948 | FKNNSPsGGET | 0.492 | 0.225 | 0.013 | 0.243 |
| sp\|Q8IZF2\|AGRF5_HUMAN Adhesion G protein-coupled receptor F5 OS=Homo sapiens GN=ADGRF5 PE=1 SV | 952 | SPSGGEtKCVF | 0.028 | -0.389 | -1.82 | -0.727 |
| sp\|Q8IZF2\|AGRF5_HUMAN Adhesion G protein-coupled receptor F5 OS=Homo sapiens GN=ADGRF5 PE=1 SV | 965 | FRLANNtGGWD | 0.548 | 0.326 | 0.046 | 0.307 |
| sp\|Q8IZF2\|AGRF5_HUMAN Adhesion G protein-coupled receptor F5 OS=Homo sapiens GN=ADGRF5 PE=1 SV | 970 | NTGGWDsSGCY | 0.207 | -0.027 | -1.001 | -0.274 |
| sp\|Q8IZF2\|AGRF5_HUMAN Adhesion G protein-coupled receptor F5 OS=Homo sapiens GN=ADGRF5 PE=1 SV | 971 | TGGWDSsGCYV | 0.05 | -0.429 | -1.69 | -0.69 |
| sp\|Q8IZF2\|AGRF5_HUMAN Adhesion G protein-coupled receptor F5 OS=Homo sapiens GN=ADGRF5 PE=1 SV | 984 | GDGDNVtCICD | 0.263 | 0.058 | -0.661 | -0.113 |
| sp\|Q8IZF2\|AGRF5_HUMAN Adhesion G protein-coupled receptor F5 OS=Homo sapiens GN=ADGRF5 PE=1 SV | 991 | CICDHLtSFSI | 0.224 | 0.086 | -0.697 | -0.129 |
| sp\|Q8IZF2\|AGRF5_HUMAN Adhesion G protein-coupled receptor F5 OS=Homo sapiens GN=ADGRF5 PE=1 SV | 992 | ICDHLTsFSIL | 0.235 | -0.045 | -0.797 | -0.202 |
| sp\|Q8IZF2\|AGRF5_HUMAN Adhesion G protein-coupled receptor F5 OS=Homo sapiens GN=ADGRF5 PE=1 SV | 994 | DHLTSFsILMS | 0.168 | 0.018 | -1.087 | -0.3 |
| sp\|Q8IZF2\|AGRF5_HUMAN Adhesion G protein-coupled receptor F5 OS=Homo sapiens GN=ADGRF5 PE=1 SV | 998 | SFSILMsPDSP | 0.036 | -0.363 | -1.602 | -0.643 |
| sp\|Q8IZF2\|AGRF5_HUMAN Adhesion G protein-coupled receptor F5 OS=Homo sapiens GN=ADGRF5 PE=1 SV | 1001 | ILMSPDsPDPS | 0.08 | -0.464 | -1.245 | -0.543 |
| sp\|Q8IZF2\|AGRF5_HUMAN Adhesion G protein-coupled receptor F5 OS=Homo sapiens GN=ADGRF5 PE=1 SV | 1005 | PDSPDPsSLLG | 0.061 | -0.344 | -1.478 | -0.587 |
| sp\|Q8IZF2\|AGRF5_HUMAN Adhesion G protein-coupled receptor F5 OS=Homo sapiens GN=ADGRF5 PE=1 SV | 1006 | DSPDPSsLLGI | 0.149 | -0.233 | -0.941 | -0.342 |
| sp\|Q8IZF2\|AGRF5_HUMAN Adhesion G protein-coupled receptor F5 OS=Homo sapiens GN=ADGRF5 PE=1 SV | 1016 | ILLDIIsYVGV | 0.308 | -0.029 | -0.569 | -0.097 |
| sp\|Q8IZF2\|AGRF5_HUMAN Adhesion G protein-coupled receptor F5 OS=Homo sapiens GN=ADGRF5 PE=1 SV | 1023 | YVGVGFsILSL | 0.02 | -0.18 | -1.56 | -0.573 |
| sp\|Q8IZF2\|AGRF5_HUMAN Adhesion G protein-coupled receptor F5 OS=Homo sapiens GN=ADGRF5 PE=1 SV | 1026 | VGFSILsLAAC | 0.135 | -0.142 | -0.769 | -0.259 |
| sp\|Q8IZF2\|AGRF5_HUMAN Adhesion G protein-coupled receptor F5 OS=Homo sapiens GN=ADGRF5 PE=1 SV | 1040 | EAVVWKsVTKN | 0.155 | -0.028 | -0.854 | -0.242 |
| sp\|Q8IZF2\|AGRF5_HUMAN Adhesion G protein-coupled receptor F5 OS=Homo sapiens GN=ADGRF5 PE=1 SV | 1042 | VVWKSVtKNRT | 0.519 | 0.232 | 0.045 | 0.265 |
| sp\|Q8IZF2\|AGRF5_HUMAN Adhesion G protein-coupled receptor F5 OS=Homo sapiens GN=ADGRF5 PE=1 SV | 1046 | SVTKNRtSYMR | 0.127 | -0.127 | -1.098 | -0.366 |
| sp\|Q8IZF2\|AGRF5_HUMAN Adhesion G protein-coupled receptor F5 OS=Homo sapiens GN=ADGRF5 PE=1 SV | 1047 | VTKNRTsYMRH | 0.208 | -0.15 | -0.936 | -0.293 |
| sp\|Q8IZF2\|AGRF5_HUMAN Adhesion G protein-coupled receptor F5 OS=Homo sapiens GN=ADGRF5 PE=1 SV | 1052 | TSYMRHtCIVN | 0.325 | 0.233 | -0.461 | 0.032 |
| sp\|Q8IZF2\|AGRF5_HUMAN Adhesion G protein-coupled receptor F5 OS=Homo sapiens GN=ADGRF5 PE=1 SV | 1060 | IVNIAAsLLVA | 0.384 | 0.146 | -0.37 | 0.053 |
| sp\|Q8IZF2\|AGRF5_HUMAN Adhesion G protein-coupled receptor F5 OS=Homo sapiens GN=ADGRF5 PE=1 SV | 1066 | SLLVANtWFIV | 0.369 | 0.194 | -0.721 | -0.053 |
| sp\|Q8IZF2\|AGRF5_HUMAN Adhesion G protein-coupled receptor F5 OS=Homo sapiens GN=ADGRF5 PE=1 SV | 1084 | RYILCKtACVA | 0.434 | 0.118 | -0.517 | 0.012 |
| sp\|Q8IZF2\|AGRF5_HUMAN Adhesion G protein-coupled receptor F5 OS=Homo sapiens GN=ADGRF5 PE=1 SV | 1090 | TACVAAtFFIH | 0.29 | -0.059 | -0.838 | -0.202 |
| sp\|Q8IZF2\|AGRF5_HUMAN Adhesion G protein-coupled receptor F5 OS=Homo sapiens GN=ADGRF5 PE=1 SV | 1099 | IHFFYLsVFFW | 0.141 | -0.073 | -1.143 | -0.358 |
| sp\|Q8IZF2\|AGRF5_HUMAN Adhesion G protein-coupled receptor F5 OS=Homo sapiens GN=ADGRF5 PE=1 SV | 1106 | VFFWMLtLGLM | 0.41 | -0.005 | -0.075 | 0.11 |
| sp\|Q8IZF2\|AGRF5_HUMAN Adhesion G protein-coupled receptor F5 OS=Homo sapiens GN=ADGRF5 PE=1 SV | 1122 | VFILHEtSRST | 0.084 | -0.218 | -1.325 | -0.486 |
| sp\|Q8IZF2\|AGRF5_HUMAN Adhesion G protein-coupled receptor F5 OS=Homo sapiens GN=ADGRF5 PE=1 SV | 1123 | FILHETsRSTQ | 0.114 | -0.168 | -1.22 | -0.425 |
| sp\|Q8IZF2\|AGRF5_HUMAN Adhesion G protein-coupled receptor F5 OS=Homo sapiens GN=ADGRF5 PE=1 SV | 1125 | LHETSRsTQKA | 0.196 | 0.054 | -0.911 | -0.22 |
| sp\|Q8IZF2\|AGRF5_HUMAN Adhesion G protein-coupled receptor F5 OS=Homo sapiens GN=ADGRF5 PE=1 SV | 1126 | HETSRStQKAI | 0.242 | 0.085 | -0.577 | -0.083 |
| sp\|Q8IZF2\|AGRF5_HUMAN Adhesion G protein-coupled receptor F5 OS=Homo sapiens GN=ADGRF5 PE=1 SV | 1143 | GCPLAIsVITL | 0.321 | 0.097 | -0.602 | -0.061 |
| sp\|Q8IZF2\|AGRF5_HUMAN Adhesion G protein-coupled receptor F5 OS=Homo sapiens GN=ADGRF5 PE=1 SV | 1146 | LAISVItLGAT | 0.363 | 0.031 | -0.282 | 0.037 |
| sp\|Q8IZF2\|AGRF5_HUMAN Adhesion G protein-coupled receptor F5 OS=Homo sapiens GN=ADGRF5 PE=1 SV | 1150 | VITLGAtQPRE | 0.435 | 0.311 | -0.564 | 0.061 |
| sp\|Q8IZF2\|AGRF5_HUMAN Adhesion G protein-coupled receptor F5 OS=Homo sapiens GN=ADGRF5 PE=1 SV | 1157 | QPREVYtRKNV | 0.185 | 0.137 | -1.082 | -0.253 |
| sp\|Q8IZF2\|AGRF5_HUMAN Adhesion G protein-coupled receptor F5 OS=Homo sapiens GN=ADGRF5 PE=1 SV | 1169 | WLNWEDtKALL | 0.214 | -0.139 | -0.863 | -0.263 |
| sp\|Q8IZF2\|AGRF5_HUMAN Adhesion G protein-coupled receptor F5 OS=Homo sapiens GN=ADGRF5 PE=1 SV | 1188 | IVVVNItITIV | 0.189 | -0.132 | -0.961 | -0.301 |
| sp\|Q8IZF2\|AGRF5_HUMAN Adhesion G protein-coupled receptor F5 OS=Homo sapiens GN=ADGRF5 PE=1 SV | 1190 | VVNITItIVVI | 0.098 | -0.002 | -0.933 | -0.279 |
| sp\|Q8IZF2\|AGRF5_HUMAN Adhesion G protein-coupled receptor F5 OS=Homo sapiens GN=ADGRF5 PE=1 SV | 1195 | ITIVVItKILR | 0.19 | -0.059 | -0.726 | -0.198 |
| sp\|Q8IZF2\|AGRF5_HUMAN Adhesion G protein-coupled receptor F5 OS=Homo sapiens GN=ADGRF5 PE=1 SV | 1201 | TKILRPsIGDK | 0.532 | 0.145 | -0.166 | 0.17 |

| | | | | | | |
|---|---|---|---|---|---|---|
| sp\|Q8IZF2\|AGRF5_HUMAN Adhesion G protein-coupled receptor F5 OS=Homo sapiens GN=ADGRF5 PE=1 SV | 1212 | PCKQEKsSLFQ | 0.222 | -0.091 | -0.878 | -0.249 |
| sp\|Q8IZF2\|AGRF5_HUMAN Adhesion G protein-coupled receptor F5 OS=Homo sapiens GN=ADGRF5 PE=1 SV | 1213 | CKQEKSsLFQI | 0.427 | 0.134 | -0.018 | 0.181 |
| sp\|Q8IZF2\|AGRF5_HUMAN Adhesion G protein-coupled receptor F5 OS=Homo sapiens GN=ADGRF5 PE=1 SV | 1218 | SSLFQIsKSIG | 0.114 | -0.22 | -1.243 | -0.45 |
| sp\|Q8IZF2\|AGRF5_HUMAN Adhesion G protein-coupled receptor F5 OS=Homo sapiens GN=ADGRF5 PE=1 SV | 1220 | LFQISKsIGVL | 0.548 | 0.415 | 0.4 | 0.454 |
| sp\|Q8IZF2\|AGRF5_HUMAN Adhesion G protein-coupled receptor F5 OS=Homo sapiens GN=ADGRF5 PE=1 SV | 1225 | KSIGVLtPLLG | 0.035 | -0.465 | -1.793 | -0.741 |
| sp\|Q8IZF2\|AGRF5_HUMAN Adhesion G protein-coupled receptor F5 OS=Homo sapiens GN=ADGRF5 PE=1 SV | 1231 | TPLLGLtWGFG | 0.058 | -0.163 | -1.345 | -0.483 |
| sp\|Q8IZF2\|AGRF5_HUMAN Adhesion G protein-coupled receptor F5 OS=Homo sapiens GN=ADGRF5 PE=1 SV | 1237 | TWGFGLtTVFP | 0.026 | -0.302 | -1.803 | -0.693 |
| sp\|Q8IZF2\|AGRF5_HUMAN Adhesion G protein-coupled receptor F5 OS=Homo sapiens GN=ADGRF5 PE=1 SV | 1238 | WGFGLTtVFPG | 0.125 | -0.145 | -0.764 | -0.261 |
| sp\|Q8IZF2\|AGRF5_HUMAN Adhesion G protein-coupled receptor F5 OS=Homo sapiens GN=ADGRF5 PE=1 SV | 1243 | TTVFPGtNLVF | 0.047 | -0.299 | -1.695 | -0.649 |
| sp\|Q8IZF2\|AGRF5_HUMAN Adhesion G protein-coupled receptor F5 OS=Homo sapiens GN=ADGRF5 PE=1 SV | 1282 | ALLNKFsLSRW | 0.211 | -0.02 | -0.622 | -0.144 |
| sp\|Q8IZF2\|AGRF5_HUMAN Adhesion G protein-coupled receptor F5 OS=Homo sapiens GN=ADGRF5 PE=1 SV | 1284 | LNKFSLsRWSS | 0.16 | -0.001 | -0.754 | -0.198 |
| sp\|Q8IZF2\|AGRF5_HUMAN Adhesion G protein-coupled receptor F5 OS=Homo sapiens GN=ADGRF5 PE=1 SV | 1287 | FSLSRWsSQHS | 0.193 | -0.076 | -0.775 | -0.219 |
| sp\|Q8IZF2\|AGRF5_HUMAN Adhesion G protein-coupled receptor F5 OS=Homo sapiens GN=ADGRF5 PE=1 SV | 1288 | SLSRWSsQHSK | 0.401 | 0.694 | -0.319 | 0.259 |
| sp\|Q8IZF2\|AGRF5_HUMAN Adhesion G protein-coupled receptor F5 OS=Homo sapiens GN=ADGRF5 PE=1 SV | 1291 | RWSSQHsKSTS | 0.108 | -0.055 | -1.195 | -0.381 |
| sp\|Q8IZF2\|AGRF5_HUMAN Adhesion G protein-coupled receptor F5 OS=Homo sapiens GN=ADGRF5 PE=1 SV | 1293 | SSQHSKsTSLG | 0.25 | 0.099 | -0.511 | -0.054 |
| sp\|Q8IZF2\|AGRF5_HUMAN Adhesion G protein-coupled receptor F5 OS=Homo sapiens GN=ADGRF5 PE=1 SV | 1294 | SQHSKStSLGS | 0.019 | -0.481 | -2.112 | -0.858 |
| sp\|Q8IZF2\|AGRF5_HUMAN Adhesion G protein-coupled receptor F5 OS=Homo sapiens GN=ADGRF5 PE=1 SV | 1295 | QHSKSTsLGSS | 0.484 | 0.256 | -0.147 | 0.198 |
| sp\|Q8IZF2\|AGRF5_HUMAN Adhesion G protein-coupled receptor F5 OS=Homo sapiens GN=ADGRF5 PE=1 SV | 1298 | KSTSLGsSTPV | 0.057 | -0.425 | -1.854 | -0.741 |
| sp\|Q8IZF2\|AGRF5_HUMAN Adhesion G protein-coupled receptor F5 OS=Homo sapiens GN=ADGRF5 PE=1 SV | 1299 | STSLGSsTPVF | 0.146 | 0.195 | -0.982 | -0.214 |
| sp\|Q8IZF2\|AGRF5_HUMAN Adhesion G protein-coupled receptor F5 OS=Homo sapiens GN=ADGRF5 PE=1 SV | 1300 | TSLGSStPVFS | 0.045 | -0.38 | -1.409 | -0.581 |
| sp\|Q8IZF2\|AGRF5_HUMAN Adhesion G protein-coupled receptor F5 OS=Homo sapiens GN=ADGRF5 PE=1 SV | 1304 | SSTPVFsMSSP | 0.034 | -0.24 | -1.668 | -0.625 |
| sp\|Q8IZF2\|AGRF5_HUMAN Adhesion G protein-coupled receptor F5 OS=Homo sapiens GN=ADGRF5 PE=1 SV | 1306 | TPVFSMsSPIS | 0.714 | 0.701 | 0.248 | 0.554 |
| sp\|Q8IZF2\|AGRF5_HUMAN Adhesion G protein-coupled receptor F5 OS=Homo sapiens GN=ADGRF5 PE=1 SV | 1307 | PVFSMSsPISR | 0.027 | -0.51 | -1.579 | -0.687 |
| sp\|Q8IZF2\|AGRF5_HUMAN Adhesion G protein-coupled receptor F5 OS=Homo sapiens GN=ADGRF5 PE=1 SV | 1310 | SMSSPIsRRFN | 0.052 | -0.371 | -1.651 | -0.657 |
| sp\|Q8IZF2\|AGRF5_HUMAN Adhesion G protein-coupled receptor F5 OS=Homo sapiens GN=ADGRF5 PE=1 SV | 1320 | NNLFGKtGTYN | 0.154 | -0.182 | -0.862 | -0.297 |
| sp\|Q8IZF2\|AGRF5_HUMAN Adhesion G protein-coupled receptor F5 OS=Homo sapiens GN=ADGRF5 PE=1 SV | 1322 | LFGKTGtYNVS | 0.098 | -0.051 | -0.907 | -0.287 |
| sp\|Q8IZF2\|AGRF5_HUMAN Adhesion G protein-coupled receptor F5 OS=Homo sapiens GN=ADGRF5 PE=1 SV | 1326 | TGTYNVsTPEA | 0.738 | 0.444 | 0.538 | 0.573 |
| sp\|Q8IZF2\|AGRF5_HUMAN Adhesion G protein-coupled receptor F5 OS=Homo sapiens GN=ADGRF5 PE=1 SV | 1327 | GTYNVStPEAT | 0.056 | -0.387 | -1.351 | -0.561 |
| sp\|Q8IZF2\|AGRF5_HUMAN Adhesion G protein-coupled receptor F5 OS=Homo sapiens GN=ADGRF5 PE=1 SV | 1331 | VSTPEAtSSSL | 0.057 | -0.299 | -1.483 | -0.575 |
| sp\|Q8IZF2\|AGRF5_HUMAN Adhesion G protein-coupled receptor F5 OS=Homo sapiens GN=ADGRF5 PE=1 SV | 1332 | STPEATsSSLE | 0.054 | -0.254 | -1.677 | -0.626 |
| sp\|Q8IZF2\|AGRF5_HUMAN Adhesion G protein-coupled receptor F5 OS=Homo sapiens GN=ADGRF5 PE=1 SV | 1333 | TPEATSsSLEN | 0.059 | -0.228 | -1.516 | -0.562 |
| sp\|Q8IZF2\|AGRF5_HUMAN Adhesion G protein-coupled receptor F5 OS=Homo sapiens GN=ADGRF5 PE=1 SV | 1334 | PEATSSsLENS | 0.345 | 0.193 | -0.472 | 0.022 |
| sp\|Q8IZF2\|AGRF5_HUMAN Adhesion G protein-coupled receptor F5 OS=Homo sapiens GN=ADGRF5 PE=1 SV | 1338 | SSSLENsSSAS | 0.082 | -0.241 | -1.608 | -0.589 |
| sp\|Q8IZF2\|AGRF5_HUMAN Adhesion G protein-coupled receptor F5 OS=Homo sapiens GN=ADGRF5 PE=1 SV | 1339 | SSLENSsSASS | 0.021 | -0.429 | -1.971 | -0.793 |
| sp\|Q8IZF2\|AGRF5_HUMAN Adhesion G protein-coupled receptor F5 OS=Homo sapiens GN=ADGRF5 PE=1 SV | 1340 | SLENSSsASSL | 0.114 | 0.099 | -0.855 | -0.214 |
| sp\|Q8IZF2\|AGRF5_HUMAN Adhesion G protein-coupled receptor F5 OS=Homo sapiens GN=ADGRF5 PE=1 SV | 1342 | ENSSSAsSLLN | 0.154 | -0.005 | -0.882 | -0.244 |
| sp\|Q8IZF2\|AGRF5_HUMAN Adhesion G protein-coupled receptor F5 OS=Homo sapiens GN=ADGRF5 PE=1 SV | 1343 | NSSSASsLLN- | 0.191 | -0.042 | -0.942 | -0.264 |
| sp\|Q86SQ6\|AGRA1_HUMAN Adhesion G protein-coupled receptor A1 OS=Homo sapiens GN=ADGRA1 PE=2 S | 346 | RAACLHsPGLG | 0.204 | -0.187 | -0.985 | -0.323 |
| sp\|Q86SQ6\|AGRA1_HUMAN Adhesion G protein-coupled receptor A1 OS=Homo sapiens GN=ADGRA1 PE=2 S | 365 | PGPCKMtNLQA | 0.133 | -0.137 | -1.091 | -0.365 |
| sp\|Q86SQ6\|AGRA1_HUMAN Adhesion G protein-coupled receptor A1 OS=Homo sapiens GN=ADGRA1 PE=2 S | 375 | AAQGHAsCLSP | 0.092 | -0.044 | -1.325 | -0.426 |
| sp\|Q86SQ6\|AGRA1_HUMAN Adhesion G protein-coupled receptor A1 OS=Homo sapiens GN=ADGRA1 PE=2 S | 378 | GHASCLsPATP | 0.045 | -0.524 | -1.79 | -0.756 |
| sp\|Q86SQ6\|AGRA1_HUMAN Adhesion G protein-coupled receptor A1 OS=Homo sapiens GN=ADGRA1 PE=2 S | 381 | SCLSPAtPCCA | 0.052 | -0.521 | -1.698 | -0.722 |
| sp\|Q86SQ6\|AGRA1_HUMAN Adhesion G protein-coupled receptor A1 OS=Homo sapiens GN=ADGRA1 PE=2 S | 393 | MHCEPLtADEA | 0.142 | -0.031 | -1.17 | -0.353 |
| sp\|Q86SQ6\|AGRA1_HUMAN Adhesion G protein-coupled receptor A1 OS=Homo sapiens GN=ADGRA1 PE=2 S | 421 | GCLQGRtKPPY | 0.57 | 0.449 | -0.121 | 0.299 |
| sp\|Q86SQ6\|AGRA1_HUMAN Adhesion G protein-coupled receptor A1 OS=Homo sapiens GN=ADGRA1 PE=2 S | 427 | TKPPYFsRHPA | 0.176 | -0.192 | -0.862 | -0.293 |
| sp\|Q86SQ6\|AGRA1_HUMAN Adhesion G protein-coupled receptor A1 OS=Homo sapiens GN=ADGRA1 PE=2 S | 442 | YAYHIPsSLDG | 0.113 | -0.17 | -0.941 | -0.333 |
| sp\|Q86SQ6\|AGRA1_HUMAN Adhesion G protein-coupled receptor A1 OS=Homo sapiens GN=ADGRA1 PE=2 S | 443 | AYHIPSsLDGS | 0.149 | -0.139 | -0.817 | -0.269 |
| sp\|Q86SQ6\|AGRA1_HUMAN Adhesion G protein-coupled receptor A1 OS=Homo sapiens GN=ADGRA1 PE=2 S | 447 | PSSLDGsPRSS | 0.027 | -0.655 | -1.975 | -0.868 |
| sp\|Q86SQ6\|AGRA1_HUMAN Adhesion G protein-coupled receptor A1 OS=Homo sapiens GN=ADGRA1 PE=2 S | 450 | LDGSPRsSRTD | 0.043 | -0.422 | -1.511 | -0.63 |

| | | | | | | |
|---|---|---|---|---|---|---|
| sp\|Q86SQ6\|AGRA1_HUMAN Adhesion G protein-coupled receptor A1 OS=Homo sapiens GN=ADGRA1 PE=2 S | 451 | DGSPRSsRTDS | 0.246 | -0.136 | -0.938 | -0.276 |
| sp\|Q86SQ6\|AGRA1_HUMAN Adhesion G protein-coupled receptor A1 OS=Homo sapiens GN=ADGRA1 PE=2 S | 453 | SPRSSRtDSPP | 0.154 | 0.107 | -1.112 | -0.284 |
| sp\|Q86SQ6\|AGRA1_HUMAN Adhesion G protein-coupled receptor A1 OS=Homo sapiens GN=ADGRA1 PE=2 S | 455 | RSSRTDsPPSS | 0.458 | 0.873 | -0.04 | 0.43 |
| sp\|Q86SQ6\|AGRA1_HUMAN Adhesion G protein-coupled receptor A1 OS=Homo sapiens GN=ADGRA1 PE=2 S | 458 | RTDSPPsSLDG | 0.125 | -0.26 | -1.254 | -0.463 |
| sp\|Q86SQ6\|AGRA1_HUMAN Adhesion G protein-coupled receptor A1 OS=Homo sapiens GN=ADGRA1 PE=2 S | 459 | TDSPPSsLDGP | 0.069 | -0.372 | -1.007 | -0.437 |
| sp\|Q86SQ6\|AGRA1_HUMAN Adhesion G protein-coupled receptor A1 OS=Homo sapiens GN=ADGRA1 PE=2 S | 466 | LDGPAGtHTLA | 0.105 | -0.234 | -1.109 | -0.413 |
| sp\|Q86SQ6\|AGRA1_HUMAN Adhesion G protein-coupled receptor A1 OS=Homo sapiens GN=ADGRA1 PE=2 S | 468 | GPAGTHtLACC | 0.228 | 0.136 | -0.674 | -0.103 |
| sp\|Q86SQ6\|AGRA1_HUMAN Adhesion G protein-coupled receptor A1 OS=Homo sapiens GN=ADGRA1 PE=2 S | 473 | HTLACCtQGDP | 0.474 | 0.007 | -0.602 | -0.04 |
| sp\|Q86SQ6\|AGRA1_HUMAN Adhesion G protein-coupled receptor A1 OS=Homo sapiens GN=ADGRA1 PE=2 S | 482 | DPFPMVtQPEG | 0.694 | 0.297 | -0.221 | 0.257 |
| sp\|Q86SQ6\|AGRA1_HUMAN Adhesion G protein-coupled receptor A1 OS=Homo sapiens GN=ADGRA1 PE=2 S | 487 | VTQPEGsDGSP | 0.077 | -0.245 | -1.54 | -0.569 |
| sp\|Q86SQ6\|AGRA1_HUMAN Adhesion G protein-coupled receptor A1 OS=Homo sapiens GN=ADGRA1 PE=2 S | 490 | PEGSDGsPALY | 0.036 | -0.537 | -1.953 | -0.818 |
| sp\|Q86SQ6\|AGRA1_HUMAN Adhesion G protein-coupled receptor A1 OS=Homo sapiens GN=ADGRA1 PE=2 S | 495 | GSPALYsCPTQ | 0.398 | 0.386 | -0.377 | 0.136 |
| sp\|Q86SQ6\|AGRA1_HUMAN Adhesion G protein-coupled receptor A1 OS=Homo sapiens GN=ADGRA1 PE=2 S | 498 | ALYSCPtQPGR | 0.632 | 0.338 | -0.058 | 0.304 |
| sp\|Q86SQ6\|AGRA1_HUMAN Adhesion G protein-coupled receptor A1 OS=Homo sapiens GN=ADGRA1 PE=2 S | 517 | LEMLRRtQSLP | 0.077 | -0.06 | -1.546 | -0.51 |
| sp\|Q86SQ6\|AGRA1_HUMAN Adhesion G protein-coupled receptor A1 OS=Homo sapiens GN=ADGRA1 PE=2 S | 519 | MLRRTQsLPFG | 0.969 | 1.852 | 2.093 | 1.638 |
| sp\|Q86SQ6\|AGRA1_HUMAN Adhesion G protein-coupled receptor A1 OS=Homo sapiens GN=ADGRA1 PE=2 S | 526 | LPFGGPsQNGL | 0.078 | -0.232 | -1.132 | -0.429 |
| sp\|Q86SQ6\|AGRA1_HUMAN Adhesion G protein-coupled receptor A1 OS=Homo sapiens GN=ADGRA1 PE=2 S | 543 | EGLPFGtDGTG | 0.148 | -0.165 | -0.989 | -0.335 |
| sp\|Q86SQ6\|AGRA1_HUMAN Adhesion G protein-coupled receptor A1 OS=Homo sapiens GN=ADGRA1 PE=2 S | 546 | PFGTDGtGNIR | 0.142 | -0.171 | -0.888 | -0.306 |
| sp\|Q86SQ6\|AGRA1_HUMAN Adhesion G protein-coupled receptor A1 OS=Homo sapiens GN=ADGRA1 PE=2 S | 551 | GTGNIRtGPWK | 0.348 | 0.351 | -0.227 | 0.157 |
| sp\|Q86SQ6\|AGRA1_HUMAN Adhesion G protein-coupled receptor A1 OS=Homo sapiens GN=ADGRA1 PE=2 S | 558 | GPWKNEtTV-- | 0.255 | 0.198 | -0.836 | -0.128 |
| sp\|Q86SQ6\|AGRA1_HUMAN Adhesion G protein-coupled receptor A1 OS=Homo sapiens GN=ADGRA1 PE=2 S | 559 | PWKNETtV--- | 0.261 | 0.181 | -0.512 | -0.023 |
| sp\|Q9NZH0\|GPC5B_HUMAN G-protein coupled receptor family C group 5 member B OS=Homo sapiens GN=( | 318 | QPRMREtAFEE | 0.54 | 0.248 | -0.428 | 0.12 |
| sp\|Q9NZH0\|GPC5B_HUMAN G-protein coupled receptor family C group 5 member B OS=Homo sapiens GN=( | 337 | MENKAFsMDEH | 0.5 | 0.234 | -0.528 | 0.069 |
| sp\|Q9NZH0\|GPC5B_HUMAN G-protein coupled receptor family C group 5 member B OS=Homo sapiens GN=( | 347 | HNAALRtAGFP | 0.065 | -0.21 | -1.364 | -0.503 |
| sp\|Q9NZH0\|GPC5B_HUMAN G-protein coupled receptor family C group 5 member B OS=Homo sapiens GN=( | 354 | AGFPNGsLGKR | 0.27 | -0.083 | -0.507 | -0.107 |
| sp\|Q9NZH0\|GPC5B_HUMAN G-protein coupled receptor family C group 5 member B OS=Homo sapiens GN=( | 360 | SLGKRPsGSLG | 0.26 | 0.032 | -0.734 | -0.147 |
| sp\|Q9NZH0\|GPC5B_HUMAN G-protein coupled receptor family C group 5 member B OS=Homo sapiens GN=( | 362 | GKRPSGsLGKR | 0.846 | 0.551 | 0.674 | 0.69 |
| sp\|Q9NZH0\|GPC5B_HUMAN G-protein coupled receptor family C group 5 member B OS=Homo sapiens GN=( | 368 | SLGKRPsAPFR | 0.7 | 0.602 | 0.105 | 0.469 |
| sp\|Q9NZH0\|GPC5B_HUMAN G-protein coupled receptor family C group 5 member B OS=Homo sapiens GN=( | 373 | PSAPFRsNVYQ | 0.069 | -0.295 | -1.618 | -0.615 |
| sp\|Q9NZH0\|GPC5B_HUMAN G-protein coupled receptor family C group 5 member B OS=Homo sapiens GN=( | 379 | SNVYQPtEMAV | 0.103 | -0.072 | -1.211 | -0.393 |
| sp\|Q9NZH0\|GPC5B_HUMAN G-protein coupled receptor family C group 5 member B OS=Homo sapiens GN=( | 389 | VVLNGGtIPTA | 0.153 | 0.176 | -0.47 | -0.047 |
| sp\|Q9NZH0\|GPC5B_HUMAN G-protein coupled receptor family C group 5 member B OS=Homo sapiens GN=( | 392 | NGGTIPtAPPS | 0.315 | 0.295 | -0.388 | 0.074 |
| sp\|Q9NZH0\|GPC5B_HUMAN G-protein coupled receptor family C group 5 member B OS=Homo sapiens GN=( | 396 | IPTAPPsHTGR | 0.233 | -0.293 | -0.638 | -0.233 |
| sp\|Q9NZH0\|GPC5B_HUMAN G-protein coupled receptor family C group 5 member B OS=Homo sapiens GN=( | 398 | TAPPSHtGRHL | 0.175 | -0.011 | -0.602 | -0.146 |
| sp\|Q96PE1\|AGRA2_HUMAN Adhesion G protein-coupled receptor A2 OS=Homo sapiens GN=ADGRA2 PE=1 S | 316 | TSELTLsHIGV | 0.079 | -0.196 | -1.243 | -0.453 |
| sp\|Q96PE1\|AGRA2_HUMAN Adhesion G protein-coupled receptor A2 OS=Homo sapiens GN=ADGRA2 PE=1 S | 323 | HIGVWAsGEWE | 0.123 | 0.046 | -0.836 | -0.222 |
| sp\|Q96PE1\|AGRA2_HUMAN Adhesion G protein-coupled receptor A2 OS=Homo sapiens GN=ADGRA2 PE=1 S | 329 | SGEWECtVSMA | 0.139 | -0.313 | -1.282 | -0.485 |
| sp\|Q96PE1\|AGRA2_HUMAN Adhesion G protein-coupled receptor A2 OS=Homo sapiens GN=ADGRA2 PE=1 S | 331 | EWECTVsMAQG | 0.231 | 0.034 | -0.751 | -0.162 |
| sp\|Q96PE1\|AGRA2_HUMAN Adhesion G protein-coupled receptor A2 OS=Homo sapiens GN=ADGRA2 PE=1 S | 338 | MAQGNAsKKVE | 0.285 | 0.05 | -0.511 | -0.059 |
| sp\|Q96PE1\|AGRA2_HUMAN Adhesion G protein-coupled receptor A2 OS=Homo sapiens GN=ADGRA2 PE=1 S | 348 | EIVVLEtSASY | 0.033 | -0.208 | -1.819 | -0.665 |
| sp\|Q96PE1\|AGRA2_HUMAN Adhesion G protein-coupled receptor A2 OS=Homo sapiens GN=ADGRA2 PE=1 S | 349 | IVVLETsASYC | 0.083 | -0.217 | -1.633 | -0.589 |
| sp\|Q96PE1\|AGRA2_HUMAN Adhesion G protein-coupled receptor A2 OS=Homo sapiens GN=ADGRA2 PE=1 S | 351 | VLETSAsYCPA | 0.344 | 0.019 | -0.428 | -0.022 |
| sp\|Q96PE1\|AGRA2_HUMAN Adhesion G protein-coupled receptor A2 OS=Homo sapiens GN=ADGRA2 PE=1 S | 370 | DFRWPRtLAGI | 0.633 | 0.162 | 0.153 | 0.316 |
| sp\|Q96PE1\|AGRA2_HUMAN Adhesion G protein-coupled receptor A2 OS=Homo sapiens GN=ADGRA2 PE=1 S | 375 | RTLAGItAYQS | 0.176 | -0.126 | -1.024 | -0.325 |
| sp\|Q96PE1\|AGRA2_HUMAN Adhesion G protein-coupled receptor A2 OS=Homo sapiens GN=ADGRA2 PE=1 S | 379 | GITAYQsCLQY | 0.369 | 0.152 | -0.486 | 0.012 |
| sp\|Q96PE1\|AGRA2_HUMAN Adhesion G protein-coupled receptor A2 OS=Homo sapiens GN=ADGRA2 PE=1 S | 386 | CLQYPFtSVPL | 0.269 | 0.096 | -0.304 | 0.02 |
| sp\|Q96PE1\|AGRA2_HUMAN Adhesion G protein-coupled receptor A2 OS=Homo sapiens GN=ADGRA2 PE=1 S | 387 | LQYPFTsVPLG | 0.381 | 0.397 | -0.036 | 0.247 |
| sp\|Q96PE1\|AGRA2_HUMAN Adhesion G protein-coupled receptor A2 OS=Homo sapiens GN=ADGRA2 PE=1 S | 397 | GGGAPGtRASR | 0.053 | -0.417 | -1.574 | -0.646 |
| sp\|Q96PE1\|AGRA2_HUMAN Adhesion G protein-coupled receptor A2 OS=Homo sapiens GN=ADGRA2 PE=1 S | 400 | APGTRAsRRCD | 0.282 | -0.042 | -0.913 | -0.224 |

| | | | | | | |
|---|---|---|---|---|---|---|
| sp\|Q96PE1\|AGRA2_HUMAN Adhesion G protein-coupled receptor A2 OS=Homo sapiens GN=ADGRA2 PE=1 S | 415 | WEPGDYsHCLY | 0.11 | -0.148 | -1.071 | -0.37 |
| sp\|Q96PE1\|AGRA2_HUMAN Adhesion G protein-coupled receptor A2 OS=Homo sapiens GN=ADGRA2 PE=1 S | 420 | YSHCLYtNDIT | 0.115 | -0.161 | -1.139 | -0.395 |
| sp\|Q96PE1\|AGRA2_HUMAN Adhesion G protein-coupled receptor A2 OS=Homo sapiens GN=ADGRA2 PE=1 S | 424 | LYTNDItRVLY | 0.185 | -0.072 | -0.747 | -0.211 |
| sp\|Q96PE1\|AGRA2_HUMAN Adhesion G protein-coupled receptor A2 OS=Homo sapiens GN=ADGRA2 PE=1 S | 429 | ITRVLYtFVLM | 0.365 | 0.268 | -0.262 | 0.124 |
| sp\|Q96PE1\|AGRA2_HUMAN Adhesion G protein-coupled receptor A2 OS=Homo sapiens GN=ADGRA2 PE=1 S | 438 | LMPINAsNALT | 0.072 | -0.086 | -1.281 | -0.432 |
| sp\|Q96PE1\|AGRA2_HUMAN Adhesion G protein-coupled receptor A2 OS=Homo sapiens GN=ADGRA2 PE=1 S | 442 | NASNALtLAHQ | 0.126 | -0.157 | -0.764 | -0.265 |
| sp\|Q96PE1\|AGRA2_HUMAN Adhesion G protein-coupled receptor A2 OS=Homo sapiens GN=ADGRA2 PE=1 S | 451 | HQLRVYtAEAA | 0.64 | 1.037 | 0.749 | 0.809 |
| sp\|Q96PE1\|AGRA2_HUMAN Adhesion G protein-coupled receptor A2 OS=Homo sapiens GN=ADGRA2 PE=1 S | 456 | YTAEAAsFSDM | 0.366 | 0.096 | -0.355 | 0.036 |
| sp\|Q96PE1\|AGRA2_HUMAN Adhesion G protein-coupled receptor A2 OS=Homo sapiens GN=ADGRA2 PE=1 S | 458 | AEAASFsDMMD | 0.435 | 0.138 | -0.369 | 0.068 |
| sp\|Q96PE1\|AGRA2_HUMAN Adhesion G protein-coupled receptor A2 OS=Homo sapiens GN=ADGRA2 PE=1 S | 492 | VMVDMAsNLML | 0.097 | -0.077 | -1.42 | -0.467 |
| sp\|Q96PE1\|AGRA2_HUMAN Adhesion G protein-coupled receptor A2 OS=Homo sapiens GN=ADGRA2 PE=1 S | 513 | REDKACsRIVG | 0.404 | 0.046 | -0.738 | -0.096 |
| sp\|Q96PE1\|AGRA2_HUMAN Adhesion G protein-coupled receptor A2 OS=Homo sapiens GN=ADGRA2 PE=1 S | 528 | IGGAALsPHAQ | 0.107 | -0.335 | -1.043 | -0.424 |
| sp\|Q96PE1\|AGRA2_HUMAN Adhesion G protein-coupled receptor A2 OS=Homo sapiens GN=ADGRA2 PE=1 S | 535 | PHAQHIsVNAR | 0.492 | 0.158 | -0.085 | 0.188 |
| sp\|Q96PE1\|AGRA2_HUMAN Adhesion G protein-coupled receptor A2 OS=Homo sapiens GN=ADGRA2 PE=1 S | 552 | YLIKPHsYVGL | 0.366 | 0.149 | -0.237 | 0.093 |
| sp\|Q96PE1\|AGRA2_HUMAN Adhesion G protein-coupled receptor A2 OS=Homo sapiens GN=ADGRA2 PE=1 S | 557 | HSYVGLtCTAF | 0.053 | -0.072 | -1.153 | -0.391 |
| sp\|Q96PE1\|AGRA2_HUMAN Adhesion G protein-coupled receptor A2 OS=Homo sapiens GN=ADGRA2 PE=1 S | 559 | YVGLTCtAFQR | 0.159 | 0.036 | -0.944 | -0.25 |
| sp\|Q96PE1\|AGRA2_HUMAN Adhesion G protein-coupled receptor A2 OS=Homo sapiens GN=ADGRA2 PE=1 S | 571 | EGGVPGtRPGS | 0.102 | 0.055 | -0.887 | -0.243 |
| sp\|Q96PE1\|AGRA2_HUMAN Adhesion G protein-coupled receptor A2 OS=Homo sapiens GN=ADGRA2 PE=1 S | 575 | PGTRPGsPGQN | 0.384 | 0.446 | 0.001 | 0.277 |
| sp\|Q96PE1\|AGRA2_HUMAN Adhesion G protein-coupled receptor A2 OS=Homo sapiens GN=ADGRA2 PE=1 S | 597 | QLRFRCtTGRP | 0.743 | 0.384 | -0.073 | 0.351 |
| sp\|Q96PE1\|AGRA2_HUMAN Adhesion G protein-coupled receptor A2 OS=Homo sapiens GN=ADGRA2 PE=1 S | 598 | LRFRCTtGRPN | 0.704 | 0.958 | 0.554 | 0.739 |
| sp\|Q96PE1\|AGRA2_HUMAN Adhesion G protein-coupled receptor A2 OS=Homo sapiens GN=ADGRA2 PE=1 S | 604 | TGRPNVsLSSF | 0.325 | 0.08 | -0.241 | 0.055 |
| sp\|Q96PE1\|AGRA2_HUMAN Adhesion G protein-coupled receptor A2 OS=Homo sapiens GN=ADGRA2 PE=1 S | 606 | RPNVSLsSFHI | 0.152 | 0.065 | -0.708 | -0.164 |
| sp\|Q96PE1\|AGRA2_HUMAN Adhesion G protein-coupled receptor A2 OS=Homo sapiens GN=ADGRA2 PE=1 S | 607 | PNVSLSsFHIK | 0.126 | -0.235 | -1.117 | -0.409 |
| sp\|Q96PE1\|AGRA2_HUMAN Adhesion G protein-coupled receptor A2 OS=Homo sapiens GN=ADGRA2 PE=1 S | 613 | SFHIKNsVALA | 0.09 | -0.147 | -1.27 | -0.442 |
| sp\|Q96PE1\|AGRA2_HUMAN Adhesion G protein-coupled receptor A2 OS=Homo sapiens GN=ADGRA2 PE=1 S | 618 | NSVALAsIQLP | 0.098 | -0.221 | -1.186 | -0.436 |
| sp\|Q96PE1\|AGRA2_HUMAN Adhesion G protein-coupled receptor A2 OS=Homo sapiens GN=ADGRA2 PE=1 S | 624 | SIQLPPsLFSS | 0.124 | -0.074 | -0.942 | -0.297 |
| sp\|Q96PE1\|AGRA2_HUMAN Adhesion G protein-coupled receptor A2 OS=Homo sapiens GN=ADGRA2 PE=1 S | 627 | LPPSLFsSLPA | 0.034 | -0.362 | -1.669 | -0.666 |
| sp\|Q96PE1\|AGRA2_HUMAN Adhesion G protein-coupled receptor A2 OS=Homo sapiens GN=ADGRA2 PE=1 S | 628 | PPSLFSsLPAA | 0.64 | 0.469 | 0.169 | 0.426 |
| sp\|Q96PE1\|AGRA2_HUMAN Adhesion G protein-coupled receptor A2 OS=Homo sapiens GN=ADGRA2 PE=1 S | 642 | PVPPDCtLQLL | 0.294 | 0.025 | -0.584 | -0.088 |
| sp\|Q96PE1\|AGRA2_HUMAN Adhesion G protein-coupled receptor A2 OS=Homo sapiens GN=ADGRA2 PE=1 S | 656 | NGRLFHsHSNT | 0.374 | 0.244 | -0.624 | -0.002 |
| sp\|Q96PE1\|AGRA2_HUMAN Adhesion G protein-coupled receptor A2 OS=Homo sapiens GN=ADGRA2 PE=1 S | 658 | RLFHSHsNTSR | 0.557 | 0.355 | -0.177 | 0.245 |
| sp\|Q96PE1\|AGRA2_HUMAN Adhesion G protein-coupled receptor A2 OS=Homo sapiens GN=ADGRA2 PE=1 S | 660 | FHSHSNtSRPG | 0.339 | 0.099 | -0.672 | -0.078 |
| sp\|Q96PE1\|AGRA2_HUMAN Adhesion G protein-coupled receptor A2 OS=Homo sapiens GN=ADGRA2 PE=1 S | 661 | HSHSNTsRPGA | 0.341 | 0.207 | -0.21 | 0.113 |
| sp\|Q96PE1\|AGRA2_HUMAN Adhesion G protein-coupled receptor A2 OS=Homo sapiens GN=ADGRA2 PE=1 S | 676 | KRRGVAtPVIF | 0.243 | 0.111 | -0.767 | -0.138 |
| sp\|Q96PE1\|AGRA2_HUMAN Adhesion G protein-coupled receptor A2 OS=Homo sapiens GN=ADGRA2 PE=1 S | 683 | PVIFAGtSGCG | 0.248 | 0.1 | -0.758 | -0.137 |
| sp\|Q96PE1\|AGRA2_HUMAN Adhesion G protein-coupled receptor A2 OS=Homo sapiens GN=ADGRA2 PE=1 S | 684 | VIFAGTsGCGV | 0.089 | -0.271 | -1.3 | -0.494 |
| sp\|Q96PE1\|AGRA2_HUMAN Adhesion G protein-coupled receptor A2 OS=Homo sapiens GN=ADGRA2 PE=1 S | 692 | CGVGNLtEPVA | 0.518 | 0.511 | 0.251 | 0.427 |
| sp\|Q96PE1\|AGRA2_HUMAN Adhesion G protein-coupled receptor A2 OS=Homo sapiens GN=ADGRA2 PE=1 S | 698 | TEPVAVsLRHW | 0.222 | -0.102 | -0.678 | -0.186 |
| sp\|Q96PE1\|AGRA2_HUMAN Adhesion G protein-coupled receptor A2 OS=Homo sapiens GN=ADGRA2 PE=1 S | 714 | PVAAWWsQEGP | 0.135 | -0.21 | -0.997 | -0.357 |
| sp\|Q96PE1\|AGRA2_HUMAN Adhesion G protein-coupled receptor A2 OS=Homo sapiens GN=ADGRA2 PE=1 S | 725 | GEAGGWtSEGC | 0.078 | -0.3 | -1.409 | -0.544 |
| sp\|Q96PE1\|AGRA2_HUMAN Adhesion G protein-coupled receptor A2 OS=Homo sapiens GN=ADGRA2 PE=1 S | 726 | EAGGWTsEGCQ | 0.155 | 0.045 | -0.766 | -0.189 |
| sp\|Q96PE1\|AGRA2_HUMAN Adhesion G protein-coupled receptor A2 OS=Homo sapiens GN=ADGRA2 PE=1 S | 733 | EGCQLRsSQPN | 0.086 | -0.184 | -1.244 | -0.447 |
| sp\|Q96PE1\|AGRA2_HUMAN Adhesion G protein-coupled receptor A2 OS=Homo sapiens GN=ADGRA2 PE=1 S | 734 | GCQLRSsQPNV | 0.743 | 0.682 | -0.412 | 0.338 |
| sp\|Q96PE1\|AGRA2_HUMAN Adhesion G protein-coupled receptor A2 OS=Homo sapiens GN=ADGRA2 PE=1 S | 739 | SSQPNVsALHC | 0.097 | -0.274 | -1.379 | -0.519 |
| sp\|Q96PE1\|AGRA2_HUMAN Adhesion G protein-coupled receptor A2 OS=Homo sapiens GN=ADGRA2 PE=1 S | 756 | AVLMELsAFPR | 0.104 | -0.198 | -1.191 | -0.428 |
| sp\|Q96PE1\|AGRA2_HUMAN Adhesion G protein-coupled receptor A2 OS=Homo sapiens GN=ADGRA2 PE=1 S | 777 | PVVYPCtALLL | 0.163 | 0.027 | -0.936 | -0.249 |
| sp\|Q96PE1\|AGRA2_HUMAN Adhesion G protein-coupled receptor A2 OS=Homo sapiens GN=ADGRA2 PE=1 S | 787 | LLCLFAtIITY | 0.306 | 0.042 | -0.632 | -0.095 |
| sp\|Q96PE1\|AGRA2_HUMAN Adhesion G protein-coupled receptor A2 OS=Homo sapiens GN=ADGRA2 PE=1 S | 790 | LFATIItYILN | 0.156 | -0.056 | -0.491 | -0.13 |
| sp\|Q96PE1\|AGRA2_HUMAN Adhesion G protein-coupled receptor A2 OS=Homo sapiens GN=ADGRA2 PE=1 S | 796 | TYILNHsSIRV | 0.229 | 0.017 | -1.041 | -0.265 |

| | | | | | | | |
|---|---|---|---|---|---|---|---|
| sp\|Q96PE1\|AGRA2_HUMAN Adhesion G protein-coupled receptor A2 OS=Homo sapiens GN=ADGRA2 PE=1 S | 797 | YILNHSsIRVS | 0.102 | -0.078 | -0.843 | -0.273 |
| sp\|Q96PE1\|AGRA2_HUMAN Adhesion G protein-coupled receptor A2 OS=Homo sapiens GN=ADGRA2 PE=1 S | 801 | HSSIRVsRKGW | 0.258 | -0.03 | -0.557 | -0.11 |
| sp\|Q96PE1\|AGRA2_HUMAN Adhesion G protein-coupled receptor A2 OS=Homo sapiens GN=ADGRA2 PE=1 S | 818 | CFHIAMtSAVF | 0.204 | 0.12 | -0.598 | -0.091 |
| sp\|Q96PE1\|AGRA2_HUMAN Adhesion G protein-coupled receptor A2 OS=Homo sapiens GN=ADGRA2 PE=1 S | 819 | FHIAMTsAVFA | 0.127 | -0.092 | -1.25 | -0.405 |
| sp\|Q96PE1\|AGRA2_HUMAN Adhesion G protein-coupled receptor A2 OS=Homo sapiens GN=ADGRA2 PE=1 S | 827 | VFAGGItLTNY | 0.38 | 0.04 | -0.367 | 0.018 |
| sp\|Q96PE1\|AGRA2_HUMAN Adhesion G protein-coupled receptor A2 OS=Homo sapiens GN=ADGRA2 PE=1 S | 829 | AGGITLtNYQM | 0.051 | -0.191 | -1.392 | -0.511 |
| sp\|Q96PE1\|AGRA2_HUMAN Adhesion G protein-coupled receptor A2 OS=Homo sapiens GN=ADGRA2 PE=1 S | 841 | CQAVGItLHYS | 0.18 | -0.06 | -0.601 | -0.16 |
| sp\|Q96PE1\|AGRA2_HUMAN Adhesion G protein-coupled receptor A2 OS=Homo sapiens GN=ADGRA2 PE=1 S | 845 | GITLHYsSLST | 0.131 | 0.056 | -0.937 | -0.25 |
| sp\|Q96PE1\|AGRA2_HUMAN Adhesion G protein-coupled receptor A2 OS=Homo sapiens GN=ADGRA2 PE=1 S | 846 | ITLHYSsLSTL | 0.234 | -0.095 | -0.588 | -0.15 |
| sp\|Q96PE1\|AGRA2_HUMAN Adhesion G protein-coupled receptor A2 OS=Homo sapiens GN=ADGRA2 PE=1 S | 848 | LHYSSLsTLLW | 0.083 | 0.036 | -1.212 | -0.364 |
| sp\|Q96PE1\|AGRA2_HUMAN Adhesion G protein-coupled receptor A2 OS=Homo sapiens GN=ADGRA2 PE=1 S | 849 | HYSSLStLLWM | 0.118 | -0.066 | -0.835 | -0.261 |
| sp\|Q96PE1\|AGRA2_HUMAN Adhesion G protein-coupled receptor A2 OS=Homo sapiens GN=ADGRA2 PE=1 S | 865 | VLHKELtWRAP | 0.178 | -0.013 | -0.827 | -0.221 |
| sp\|Q96PE1\|AGRA2_HUMAN Adhesion G protein-coupled receptor A2 OS=Homo sapiens GN=ADGRA2 PE=1 S | 880 | GDPALPtPSPM | 0.025 | -0.721 | -2.089 | -0.928 |
| sp\|Q96PE1\|AGRA2_HUMAN Adhesion G protein-coupled receptor A2 OS=Homo sapiens GN=ADGRA2 PE=1 S | 882 | PALPTPsPMLR | 0.058 | -0.412 | -1.334 | -0.563 |
| sp\|Q96PE1\|AGRA2_HUMAN Adhesion G protein-coupled receptor A2 OS=Homo sapiens GN=ADGRA2 PE=1 S | 902 | LIICGItAAVN | 0.158 | 0.011 | -1.088 | -0.306 |
| sp\|Q96PE1\|AGRA2_HUMAN Adhesion G protein-coupled receptor A2 OS=Homo sapiens GN=ADGRA2 PE=1 S | 914 | HNYRDHsPYCW | 0.292 | 0.684 | -0.133 | 0.281 |
| sp\|Q96PE1\|AGRA2_HUMAN Adhesion G protein-coupled receptor A2 OS=Homo sapiens GN=ADGRA2 PE=1 S | 924 | WLVWRPsLGAF | 0.721 | 0.292 | 0.529 | 0.514 |
| sp\|Q96PE1\|AGRA2_HUMAN Adhesion G protein-coupled receptor A2 OS=Homo sapiens GN=ADGRA2 PE=1 S | 939 | ALILLItWIYF | 0.129 | 0.02 | -1.025 | -0.292 |
| sp\|Q96PE1\|AGRA2_HUMAN Adhesion G protein-coupled receptor A2 OS=Homo sapiens GN=ADGRA2 PE=1 S | 963 | NPKAGNsRASL | 0.084 | -0.235 | -1.312 | -0.488 |
| sp\|Q96PE1\|AGRA2_HUMAN Adhesion G protein-coupled receptor A2 OS=Homo sapiens GN=ADGRA2 PE=1 S | 966 | AGNSRAsLEAG | 0.538 | 0.186 | -0.164 | 0.187 |
| sp\|Q96PE1\|AGRA2_HUMAN Adhesion G protein-coupled receptor A2 OS=Homo sapiens GN=ADGRA2 PE=1 S | 976 | GEELRGsTRLR | 0.287 | -0.015 | -0.832 | -0.187 |
| sp\|Q96PE1\|AGRA2_HUMAN Adhesion G protein-coupled receptor A2 OS=Homo sapiens GN=ADGRA2 PE=1 S | 977 | EELRGStRLRG | 0.177 | 0.478 | -0.776 | -0.04 |
| sp\|Q96PE1\|AGRA2_HUMAN Adhesion G protein-coupled receptor A2 OS=Homo sapiens GN=ADGRA2 PE=1 S | 982 | STRLRGsGPLL | 0.69 | 0.707 | 0.054 | 0.484 |
| sp\|Q96PE1\|AGRA2_HUMAN Adhesion G protein-coupled receptor A2 OS=Homo sapiens GN=ADGRA2 PE=1 S | 987 | GSGPLLsDSGS | 0.048 | -0.328 | -1.676 | -0.652 |
| sp\|Q96PE1\|AGRA2_HUMAN Adhesion G protein-coupled receptor A2 OS=Homo sapiens GN=ADGRA2 PE=1 S | 989 | GPLLSDsGSLL | 0.211 | 0.042 | -0.811 | -0.186 |
| sp\|Q96PE1\|AGRA2_HUMAN Adhesion G protein-coupled receptor A2 OS=Homo sapiens GN=ADGRA2 PE=1 S | 991 | LLSDSGsLLAT | 0.437 | 0.261 | -0.049 | 0.216 |
| sp\|Q96PE1\|AGRA2_HUMAN Adhesion G protein-coupled receptor A2 OS=Homo sapiens GN=ADGRA2 PE=1 S | 995 | SGSLLAtGSAR | 0.091 | -0.197 | -1.246 | -0.451 |
| sp\|Q96PE1\|AGRA2_HUMAN Adhesion G protein-coupled receptor A2 OS=Homo sapiens GN=ADGRA2 PE=1 S | 997 | SLLATGsARVG | 0.135 | -0.07 | -1.12 | -0.352 |
| sp\|Q96PE1\|AGRA2_HUMAN Adhesion G protein-coupled receptor A2 OS=Homo sapiens GN=ADGRA2 PE=1 S | 1002 | GSARVGtPGPP | 0.304 | 0.371 | -0.393 | 0.094 |
| sp\|Q96PE1\|AGRA2_HUMAN Adhesion G protein-coupled receptor A2 OS=Homo sapiens GN=ADGRA2 PE=1 S | 1011 | PPEDGDsLYSP | 0.084 | -0.218 | -1.284 | -0.473 |
| sp\|Q96PE1\|AGRA2_HUMAN Adhesion G protein-coupled receptor A2 OS=Homo sapiens GN=ADGRA2 PE=1 S | 1014 | DGDSLYsPGVQ | 0.072 | -0.391 | -1.449 | -0.589 |
| sp\|Q96PE1\|AGRA2_HUMAN Adhesion G protein-coupled receptor A2 OS=Homo sapiens GN=ADGRA2 PE=1 S | 1024 | QLGALVtTHFL | 0.193 | -0.082 | -0.894 | -0.261 |
| sp\|Q96PE1\|AGRA2_HUMAN Adhesion G protein-coupled receptor A2 OS=Homo sapiens GN=ADGRA2 PE=1 S | 1025 | LGALVTtHFLY | 0.288 | 0.006 | -0.48 | -0.062 |
| sp\|Q96PE1\|AGRA2_HUMAN Adhesion G protein-coupled receptor A2 OS=Homo sapiens GN=ADGRA2 PE=1 S | 1041 | CGALAVsQRWL | 0.44 | 0.011 | -0.259 | 0.064 |
| sp\|Q96PE1\|AGRA2_HUMAN Adhesion G protein-coupled receptor A2 OS=Homo sapiens GN=ADGRA2 PE=1 S | 1051 | LPRVVCsCLYG | 0.265 | 0.167 | -0.986 | -0.185 |
| sp\|Q96PE1\|AGRA2_HUMAN Adhesion G protein-coupled receptor A2 OS=Homo sapiens GN=ADGRA2 PE=1 S | 1059 | LYGVAAsALGL | 0.202 | 0.071 | -0.841 | -0.189 |
| sp\|Q96PE1\|AGRA2_HUMAN Adhesion G protein-coupled receptor A2 OS=Homo sapiens GN=ADGRA2 PE=1 S | 1067 | LGLFVFtHHCA | 0.313 | 0.055 | -0.431 | -0.021 |
| sp\|Q96PE1\|AGRA2_HUMAN Adhesion G protein-coupled receptor A2 OS=Homo sapiens GN=ADGRA2 PE=1 S | 1079 | RRDVRAsWRAC | 0.375 | 0.38 | -0.503 | 0.084 |
| sp\|Q96PE1\|AGRA2_HUMAN Adhesion G protein-coupled receptor A2 OS=Homo sapiens GN=ADGRA2 PE=1 S | 1088 | ACCPPAsPAAP | 0.078 | -0.461 | -1.446 | -0.61 |
| sp\|Q96PE1\|AGRA2_HUMAN Adhesion G protein-coupled receptor A2 OS=Homo sapiens GN=ADGRA2 PE=1 S | 1107 | AAAEDGsPVFG | 0.026 | -0.631 | -2.069 | -0.891 |
| sp\|Q96PE1\|AGRA2_HUMAN Adhesion G protein-coupled receptor A2 OS=Homo sapiens GN=ADGRA2 PE=1 S | 1116 | FGEGPPsLKSS | 0.118 | -0.139 | -0.686 | -0.236 |
| sp\|Q96PE1\|AGRA2_HUMAN Adhesion G protein-coupled receptor A2 OS=Homo sapiens GN=ADGRA2 PE=1 S | 1119 | GPPSLKsSPSG | 0.276 | 0.278 | -0.602 | -0.016 |
| sp\|Q96PE1\|AGRA2_HUMAN Adhesion G protein-coupled receptor A2 OS=Homo sapiens GN=ADGRA2 PE=1 S | 1120 | PPSLKSsPSGS | 0.021 | -0.637 | -2.027 | -0.881 |
| sp\|Q96PE1\|AGRA2_HUMAN Adhesion G protein-coupled receptor A2 OS=Homo sapiens GN=ADGRA2 PE=1 S | 1122 | SLKSSPsGSSG | 0.159 | -0.022 | -0.797 | -0.22 |
| sp\|Q96PE1\|AGRA2_HUMAN Adhesion G protein-coupled receptor A2 OS=Homo sapiens GN=ADGRA2 PE=1 S | 1124 | KSSPSGsSGHP | 0.101 | -0.195 | -1.004 | -0.366 |
| sp\|Q96PE1\|AGRA2_HUMAN Adhesion G protein-coupled receptor A2 OS=Homo sapiens GN=ADGRA2 PE=1 S | 1125 | SSPSGSsGHPL | 0.023 | -0.467 | -1.892 | -0.779 |
| sp\|Q96PE1\|AGRA2_HUMAN Adhesion G protein-coupled receptor A2 OS=Homo sapiens GN=ADGRA2 PE=1 S | 1137 | LGPCKLtNLQL | 0.047 | -0.285 | -1.534 | -0.591 |
| sp\|Q96PE1\|AGRA2_HUMAN Adhesion G protein-coupled receptor A2 OS=Homo sapiens GN=ADGRA2 PE=1 S | 1144 | NLQLAQsQVCE | 0.339 | 0.166 | -0.695 | -0.063 |
| sp\|Q96PE1\|AGRA2_HUMAN Adhesion G protein-coupled receptor A2 OS=Homo sapiens GN=ADGRA2 PE=1 S | 1164 | EPEPAGtRGNL | 0.141 | -0.121 | -1.071 | -0.35 |

| | | | | | | |
|---|---|---|---|---|---|---|
| sp\|Q96PE1\|AGRA2_HUMAN Adhesion G protein-coupled receptor A2 OS=Homo sapiens GN=ADGRA2 PE=1 S | 1185 | GRRAHKsRAKG | 0.59 | 0.4 | -0.147 | 0.281 |
| sp\|Q96PE1\|AGRA2_HUMAN Adhesion G protein-coupled receptor A2 OS=Homo sapiens GN=ADGRA2 PE=1 S | 1216 | GALELLsSESG | 0.04 | -0.256 | -1.751 | -0.656 |
| sp\|Q96PE1\|AGRA2_HUMAN Adhesion G protein-coupled receptor A2 OS=Homo sapiens GN=ADGRA2 PE=1 S | 1217 | ALELLSsESGS | 0.1 | -0.167 | -1.24 | -0.436 |
| sp\|Q96PE1\|AGRA2_HUMAN Adhesion G protein-coupled receptor A2 OS=Homo sapiens GN=ADGRA2 PE=1 S | 1219 | ELLSSEsGSLH | 0.132 | -0.063 | -0.981 | -0.304 |
| sp\|Q96PE1\|AGRA2_HUMAN Adhesion G protein-coupled receptor A2 OS=Homo sapiens GN=ADGRA2 PE=1 S | 1221 | LSSESGsLHNS | 0.244 | 0.111 | -0.648 | -0.098 |
| sp\|Q96PE1\|AGRA2_HUMAN Adhesion G protein-coupled receptor A2 OS=Homo sapiens GN=ADGRA2 PE=1 S | 1225 | SGSLHNsPTDS | 0.157 | -0.315 | -1.319 | -0.492 |
| sp\|Q96PE1\|AGRA2_HUMAN Adhesion G protein-coupled receptor A2 OS=Homo sapiens GN=ADGRA2 PE=1 S | 1227 | SLHNSPtDSYL | 0.173 | 0.089 | -0.833 | -0.19 |
| sp\|Q96PE1\|AGRA2_HUMAN Adhesion G protein-coupled receptor A2 OS=Homo sapiens GN=ADGRA2 PE=1 S | 1229 | HNSPTDsYLGS | 0.032 | -0.351 | -1.542 | -0.62 |
| sp\|Q96PE1\|AGRA2_HUMAN Adhesion G protein-coupled receptor A2 OS=Homo sapiens GN=ADGRA2 PE=1 S | 1233 | TDSYLGsSRNS | 0.039 | -0.373 | -1.784 | -0.706 |
| sp\|Q96PE1\|AGRA2_HUMAN Adhesion G protein-coupled receptor A2 OS=Homo sapiens GN=ADGRA2 PE=1 S | 1234 | DSYLGSsRNSP | 0.034 | -0.425 | -1.835 | -0.742 |
| sp\|Q96PE1\|AGRA2_HUMAN Adhesion G protein-coupled receptor A2 OS=Homo sapiens GN=ADGRA2 PE=1 S | 1237 | LGSSRNsPGAG | 0.301 | -0.114 | -0.521 | -0.111 |
| sp\|Q96PE1\|AGRA2_HUMAN Adhesion G protein-coupled receptor A2 OS=Homo sapiens GN=ADGRA2 PE=1 S | 1251 | EGEPMLtPSEG | 0.034 | -0.574 | -1.509 | -0.683 |
| sp\|Q96PE1\|AGRA2_HUMAN Adhesion G protein-coupled receptor A2 OS=Homo sapiens GN=ADGRA2 PE=1 S | 1253 | EPMLTPsEGSD | 0.098 | 0.02 | -0.924 | -0.269 |
| sp\|Q96PE1\|AGRA2_HUMAN Adhesion G protein-coupled receptor A2 OS=Homo sapiens GN=ADGRA2 PE=1 S | 1256 | LTPSEGsDTSA | 0.064 | -0.362 | -1.748 | -0.682 |
| sp\|Q96PE1\|AGRA2_HUMAN Adhesion G protein-coupled receptor A2 OS=Homo sapiens GN=ADGRA2 PE=1 S | 1258 | PSEGSDtSAAP | 0.181 | -0.046 | -0.7 | -0.188 |
| sp\|Q96PE1\|AGRA2_HUMAN Adhesion G protein-coupled receptor A2 OS=Homo sapiens GN=ADGRA2 PE=1 S | 1259 | SEGSDTsAAPL | 0.055 | -0.276 | -1.696 | -0.639 |
| sp\|Q96PE1\|AGRA2_HUMAN Adhesion G protein-coupled receptor A2 OS=Homo sapiens GN=ADGRA2 PE=1 S | 1264 | TSAAPLsEAGR | 0.081 | -0.308 | -0.942 | -0.39 |
| sp\|Q96PE1\|AGRA2_HUMAN Adhesion G protein-coupled receptor A2 OS=Homo sapiens GN=ADGRA2 PE=1 S | 1274 | RAGQRRsASRD | 0.225 | 0.004 | -1.047 | -0.273 |
| sp\|Q96PE1\|AGRA2_HUMAN Adhesion G protein-coupled receptor A2 OS=Homo sapiens GN=ADGRA2 PE=1 S | 1276 | GQRRSAsRDSL | 0.885 | 1.325 | 1.161 | 1.124 |
| sp\|Q96PE1\|AGRA2_HUMAN Adhesion G protein-coupled receptor A2 OS=Homo sapiens GN=ADGRA2 PE=1 S | 1279 | RSASRDsLKGG | 0.432 | -0.008 | -0.294 | 0.043 |
| sp\|Q96PE1\|AGRA2_HUMAN Adhesion G protein-coupled receptor A2 OS=Homo sapiens GN=ADGRA2 PE=1 S | 1290 | GALEKEsHRRS | 0.09 | -0.322 | -1.554 | -0.595 |
| sp\|Q96PE1\|AGRA2_HUMAN Adhesion G protein-coupled receptor A2 OS=Homo sapiens GN=ADGRA2 PE=1 S | 1294 | KESHRRsYPLN | 0.477 | 0.476 | -0.377 | 0.192 |
| sp\|Q96PE1\|AGRA2_HUMAN Adhesion G protein-coupled receptor A2 OS=Homo sapiens GN=ADGRA2 PE=1 S | 1301 | YPLNAAsLNGA | 0.498 | 0.094 | 0.161 | 0.251 |
| sp\|Q96PE1\|AGRA2_HUMAN Adhesion G protein-coupled receptor A2 OS=Homo sapiens GN=ADGRA2 PE=1 S | 1315 | GKYDDVtLMGA | 0.652 | 0.096 | 0.039 | 0.262 |
| sp\|Q96PE1\|AGRA2_HUMAN Adhesion G protein-coupled receptor A2 OS=Homo sapiens GN=ADGRA2 PE=1 S | 1323 | MGAEVAsGGCM | 0.257 | 0.065 | -0.528 | -0.069 |
| sp\|Q96PE1\|AGRA2_HUMAN Adhesion G protein-coupled receptor A2 OS=Homo sapiens GN=ADGRA2 PE=1 S | 1329 | SGGCMKtGLWK | 0.096 | -0.254 | -1.039 | -0.399 |
| sp\|Q96PE1\|AGRA2_HUMAN Adhesion G protein-coupled receptor A2 OS=Homo sapiens GN=ADGRA2 PE=1 S | 1334 | KTGLWKsETTV | 0.328 | 0.028 | -0.807 | -0.15 |
| sp\|Q96PE1\|AGRA2_HUMAN Adhesion G protein-coupled receptor A2 OS=Homo sapiens GN=ADGRA2 PE=1 S | 1336 | GLWKSEtTV-- | 0.59 | 0.56 | -0.101 | 0.35 |
| sp\|Q96PE1\|AGRA2_HUMAN Adhesion G protein-coupled receptor A2 OS=Homo sapiens GN=ADGRA2 PE=1 S | 1337 | LWKSETtV--- | 0.204 | 0.097 | -0.734 | -0.144 |
| sp\|Q8IWK6\|AGRA3_HUMAN Adhesion G protein-coupled receptor A3 OS=Homo sapiens GN=ADGRA3 PE=1 S | 318 | SNIQAGsTGNW | 0.143 | -0.072 | -1.116 | -0.348 |
| sp\|Q8IWK6\|AGRA3_HUMAN Adhesion G protein-coupled receptor A3 OS=Homo sapiens GN=ADGRA3 PE=1 S | 319 | NIQAGStGNWG | 0.119 | -0.071 | -0.811 | -0.254 |
| sp\|Q8IWK6\|AGRA3_HUMAN Adhesion G protein-coupled receptor A3 OS=Homo sapiens GN=ADGRA3 PE=1 S | 328 | WGCHVQtKRGN | 0.22 | -0.185 | -0.592 | -0.186 |
| sp\|Q8IWK6\|AGRA3_HUMAN Adhesion G protein-coupled receptor A3 OS=Homo sapiens GN=ADGRA3 PE=1 S | 334 | TKRGNNtRTVD | 0.671 | 0.263 | -0.034 | 0.3 |
| sp\|Q8IWK6\|AGRA3_HUMAN Adhesion G protein-coupled receptor A3 OS=Homo sapiens GN=ADGRA3 PE=1 S | 336 | RGNNTRtVDIV | 0.14 | 0.031 | -1.061 | -0.297 |
| sp\|Q8IWK6\|AGRA3_HUMAN Adhesion G protein-coupled receptor A3 OS=Homo sapiens GN=ADGRA3 PE=1 S | 344 | DIVVLEsSAQY | 0.095 | -0.181 | -1.558 | -0.548 |
| sp\|Q8IWK6\|AGRA3_HUMAN Adhesion G protein-coupled receptor A3 OS=Homo sapiens GN=ADGRA3 PE=1 S | 345 | IVVLESsAQYC | 0.084 | -0.21 | -1.602 | -0.576 |
| sp\|Q8IWK6\|AGRA3_HUMAN Adhesion G protein-coupled receptor A3 OS=Homo sapiens GN=ADGRA3 PE=1 S | 366 | DFRWPRtLAGI | 0.633 | 0.162 | 0.153 | 0.316 |
| sp\|Q8IWK6\|AGRA3_HUMAN Adhesion G protein-coupled receptor A3 OS=Homo sapiens GN=ADGRA3 PE=1 S | 371 | RTLAGItAYLQ | 0.173 | -0.107 | -1.095 | -0.343 |
| sp\|Q8IWK6\|AGRA3_HUMAN Adhesion G protein-coupled receptor A3 OS=Homo sapiens GN=ADGRA3 PE=1 S | 377 | TAYLQCtRNTH | 0.076 | -0.322 | -1.665 | -0.637 |
| sp\|Q8IWK6\|AGRA3_HUMAN Adhesion G protein-coupled receptor A3 OS=Homo sapiens GN=ADGRA3 PE=1 S | 380 | LQCTRNtHGSG | 0.401 | 0.147 | -0.404 | 0.048 |
| sp\|Q8IWK6\|AGRA3_HUMAN Adhesion G protein-coupled receptor A3 OS=Homo sapiens GN=ADGRA3 PE=1 S | 383 | TRNTHGsGIYP | 0.07 | -0.067 | -1.388 | -0.462 |
| sp\|Q8IWK6\|AGRA3_HUMAN Adhesion G protein-coupled receptor A3 OS=Homo sapiens GN=ADGRA3 PE=1 S | 412 | WADDDYsRCQY | 0.117 | -0.269 | -1.214 | -0.455 |
| sp\|Q8IWK6\|AGRA3_HUMAN Adhesion G protein-coupled receptor A3 OS=Homo sapiens GN=ADGRA3 PE=1 S | 421 | QYANDVtRVLY | 0.241 | -0.079 | -0.665 | -0.168 |
| sp\|Q8IWK6\|AGRA3_HUMAN Adhesion G protein-coupled receptor A3 OS=Homo sapiens GN=ADGRA3 PE=1 S | 435 | QMPLNLtNAVA | 0.047 | -0.154 | -1.516 | -0.541 |
| sp\|Q8IWK6\|AGRA3_HUMAN Adhesion G protein-coupled receptor A3 OS=Homo sapiens GN=ADGRA3 PE=1 S | 440 | LTNAVAtARQL | 0.288 | -0.088 | -0.857 | -0.219 |
| sp\|Q8IWK6\|AGRA3_HUMAN Adhesion G protein-coupled receptor A3 OS=Homo sapiens GN=ADGRA3 PE=1 S | 448 | RQLLAYtVEAA | 0.324 | 0.195 | -0.115 | 0.135 |
| sp\|Q8IWK6\|AGRA3_HUMAN Adhesion G protein-coupled receptor A3 OS=Homo sapiens GN=ADGRA3 PE=1 S | 455 | VEAANFsDKMD | 0.233 | -0.111 | -0.691 | -0.19 |
| sp\|Q8IWK6\|AGRA3_HUMAN Adhesion G protein-coupled receptor A3 OS=Homo sapiens GN=ADGRA3 PE=1 S | 474 | EKFGRFtKEEK | 0.444 | 0.195 | -0.118 | 0.174 |
| sp\|Q8IWK6\|AGRA3_HUMAN Adhesion G protein-coupled receptor A3 OS=Homo sapiens GN=ADGRA3 PE=1 S | 479 | FTKEEKsKELG | 0.285 | 0.064 | -0.586 | -0.079 |

| | | | | | | |
|---|---|---|---|---|---|---|
| sp\|Q8IWK6\|AGRA3_HUMAN Adhesion G protein-coupled receptor A3 OS=Homo sapiens GN=ADGRA3 PE=1 S | 491 | VMVDIAsNIML | 0.108 | 0.01 | -1.094 | -0.325 |
| sp\|Q8IWK6\|AGRA3_HUMAN Adhesion G protein-coupled receptor A3 OS=Homo sapiens GN=ADGRA3 PE=1 S | 512 | REAKACsRIVQ | 0.39 | 0.024 | -0.694 | -0.093 |
| sp\|Q8IWK6\|AGRA3_HUMAN Adhesion G protein-coupled receptor A3 OS=Homo sapiens GN=ADGRA3 PE=1 S | 523 | CLQRIAtYRLA | 0.811 | 0.959 | 0.913 | 0.894 |
| sp\|Q8IWK6\|AGRA3_HUMAN Adhesion G protein-coupled receptor A3 OS=Homo sapiens GN=ADGRA3 PE=1 S | 534 | GGAHVYsTYSP | 0.125 | -0.086 | -0.904 | -0.288 |
| sp\|Q8IWK6\|AGRA3_HUMAN Adhesion G protein-coupled receptor A3 OS=Homo sapiens GN=ADGRA3 PE=1 S | 535 | GAHVYStYSPN | 0.038 | -0.317 | -1.774 | -0.684 |
| sp\|Q8IWK6\|AGRA3_HUMAN Adhesion G protein-coupled receptor A3 OS=Homo sapiens GN=ADGRA3 PE=1 S | 537 | HVYSTYsPNIA | 0.06 | -0.215 | -0.935 | -0.363 |
| sp\|Q8IWK6\|AGRA3_HUMAN Adhesion G protein-coupled receptor A3 OS=Homo sapiens GN=ADGRA3 PE=1 S | 549 | EAYVIKsTGFT | 0.079 | -0.161 | -0.855 | -0.312 |
| sp\|Q8IWK6\|AGRA3_HUMAN Adhesion G protein-coupled receptor A3 OS=Homo sapiens GN=ADGRA3 PE=1 S | 550 | AYVIKStGFTG | 0.081 | -0.202 | -1.245 | -0.455 |
| sp\|Q8IWK6\|AGRA3_HUMAN Adhesion G protein-coupled receptor A3 OS=Homo sapiens GN=ADGRA3 PE=1 S | 553 | IKSTGFtGMTC | 0.165 | -0.191 | -0.954 | -0.327 |
| sp\|Q8IWK6\|AGRA3_HUMAN Adhesion G protein-coupled receptor A3 OS=Homo sapiens GN=ADGRA3 PE=1 S | 556 | TGFTGMtCTVF | 0.16 | 0.044 | -0.908 | -0.235 |
| sp\|Q8IWK6\|AGRA3_HUMAN Adhesion G protein-coupled receptor A3 OS=Homo sapiens GN=ADGRA3 PE=1 S | 558 | FTGMTCtVFQK | 0.23 | -0.041 | -0.82 | -0.21 |
| sp\|Q8IWK6\|AGRA3_HUMAN Adhesion G protein-coupled receptor A3 OS=Homo sapiens GN=ADGRA3 PE=1 S | 566 | FQKVAAsDRTG | 0.413 | 0.105 | -0.366 | 0.051 |
| sp\|Q8IWK6\|AGRA3_HUMAN Adhesion G protein-coupled receptor A3 OS=Homo sapiens GN=ADGRA3 PE=1 S | 569 | VAASDRtGLSD | 0.047 | -0.344 | -1.602 | -0.633 |
| sp\|Q8IWK6\|AGRA3_HUMAN Adhesion G protein-coupled receptor A3 OS=Homo sapiens GN=ADGRA3 PE=1 S | 572 | SDRTGLsDYGR | 0.098 | -0.068 | -1.202 | -0.391 |
| sp\|Q8IWK6\|AGRA3_HUMAN Adhesion G protein-coupled receptor A3 OS=Homo sapiens GN=ADGRA3 PE=1 S | 588 | NLDKQLsFKCN | 0.285 | 0.154 | -0.592 | -0.051 |
| sp\|Q8IWK6\|AGRA3_HUMAN Adhesion G protein-coupled receptor A3 OS=Homo sapiens GN=ADGRA3 PE=1 S | 594 | SFKCNVsNTFS | 0.275 | -0.188 | -0.93 | -0.281 |
| sp\|Q8IWK6\|AGRA3_HUMAN Adhesion G protein-coupled receptor A3 OS=Homo sapiens GN=ADGRA3 PE=1 S | 596 | KCNVSNtFSSL | 0.246 | 0.213 | -0.76 | -0.1 |
| sp\|Q8IWK6\|AGRA3_HUMAN Adhesion G protein-coupled receptor A3 OS=Homo sapiens GN=ADGRA3 PE=1 S | 598 | NVSNTFsSLAL | 0.08 | 0.05 | -0.817 | -0.229 |
| sp\|Q8IWK6\|AGRA3_HUMAN Adhesion G protein-coupled receptor A3 OS=Homo sapiens GN=ADGRA3 PE=1 S | 599 | VSNTFSsLALK | 0.126 | -0.256 | -1.199 | -0.443 |
| sp\|Q8IWK6\|AGRA3_HUMAN Adhesion G protein-coupled receptor A3 OS=Homo sapiens GN=ADGRA3 PE=1 S | 605 | SLALKNtIVEA | 0.177 | -0.049 | -0.884 | -0.252 |
| sp\|Q8IWK6\|AGRA3_HUMAN Adhesion G protein-coupled receptor A3 OS=Homo sapiens GN=ADGRA3 PE=1 S | 610 | NTIVEAsIQLP | 0.103 | -0.143 | -1.291 | -0.444 |
| sp\|Q8IWK6\|AGRA3_HUMAN Adhesion G protein-coupled receptor A3 OS=Homo sapiens GN=ADGRA3 PE=1 S | 616 | SIQLPPsLFSP | 0.106 | -0.112 | -0.989 | -0.332 |
| sp\|Q8IWK6\|AGRA3_HUMAN Adhesion G protein-coupled receptor A3 OS=Homo sapiens GN=ADGRA3 PE=1 S | 619 | LPPSLFsPKQK | 0.029 | -0.597 | -1.705 | -0.758 |
| sp\|Q8IWK6\|AGRA3_HUMAN Adhesion G protein-coupled receptor A3 OS=Homo sapiens GN=ADGRA3 PE=1 S | 629 | KRELRPtDDSL | 0.335 | 0.3 | -0.52 | 0.038 |
| sp\|Q8IWK6\|AGRA3_HUMAN Adhesion G protein-coupled receptor A3 OS=Homo sapiens GN=ADGRA3 PE=1 S | 632 | LRPTDDsLYKL | 0.176 | 0.096 | -0.829 | -0.186 |
| sp\|Q8IWK6\|AGRA3_HUMAN Adhesion G protein-coupled receptor A3 OS=Homo sapiens GN=ADGRA3 PE=1 S | 650 | GKLFPAtGNST | 0.272 | -0.06 | -0.477 | -0.088 |
| sp\|Q8IWK6\|AGRA3_HUMAN Adhesion G protein-coupled receptor A3 OS=Homo sapiens GN=ADGRA3 PE=1 S | 653 | FPATGNsTNLA | 0.193 | -0.032 | -0.774 | -0.204 |
| sp\|Q8IWK6\|AGRA3_HUMAN Adhesion G protein-coupled receptor A3 OS=Homo sapiens GN=ADGRA3 PE=1 S | 654 | PATGNStNLAD | 0.134 | -0.102 | -0.848 | -0.272 |
| sp\|Q8IWK6\|AGRA3_HUMAN Adhesion G protein-coupled receptor A3 OS=Homo sapiens GN=ADGRA3 PE=1 S | 664 | DDGKRRtVVTP | 0.164 | -0.045 | -1.108 | -0.33 |
| sp\|Q8IWK6\|AGRA3_HUMAN Adhesion G protein-coupled receptor A3 OS=Homo sapiens GN=ADGRA3 PE=1 S | 667 | KRRTVVtPVIL | 0.411 | 0.132 | -0.595 | -0.017 |
| sp\|Q8IWK6\|AGRA3_HUMAN Adhesion G protein-coupled receptor A3 OS=Homo sapiens GN=ADGRA3 PE=1 S | 672 | VTPVILtKIDG | 0.083 | -0.17 | -0.992 | -0.36 |
| sp\|Q8IWK6\|AGRA3_HUMAN Adhesion G protein-coupled receptor A3 OS=Homo sapiens GN=ADGRA3 PE=1 S | 681 | DGVNVDtHHIP | 0.185 | -0.204 | -1.13 | -0.383 |
| sp\|Q8IWK6\|AGRA3_HUMAN Adhesion G protein-coupled receptor A3 OS=Homo sapiens GN=ADGRA3 PE=1 S | 689 | HIPVNVtLRRI | 0.474 | 0.046 | -0.135 | 0.128 |
| sp\|Q8IWK6\|AGRA3_HUMAN Adhesion G protein-coupled receptor A3 OS=Homo sapiens GN=ADGRA3 PE=1 S | 717 | GQGGWKsDGCH | 0.27 | 0.083 | -0.57 | -0.072 |
| sp\|Q8IWK6\|AGRA3_HUMAN Adhesion G protein-coupled receptor A3 OS=Homo sapiens GN=ADGRA3 PE=1 S | 725 | GCHILYsDENI | 0.259 | 0.186 | -0.916 | -0.157 |
| sp\|Q8IWK6\|AGRA3_HUMAN Adhesion G protein-coupled receptor A3 OS=Homo sapiens GN=ADGRA3 PE=1 S | 730 | YSDENItTIQC | 0.086 | -0.271 | -1.184 | -0.456 |
| sp\|Q8IWK6\|AGRA3_HUMAN Adhesion G protein-coupled receptor A3 OS=Homo sapiens GN=ADGRA3 PE=1 S | 731 | SDENITtIQCY | 0.081 | -0.157 | -0.942 | -0.339 |
| sp\|Q8IWK6\|AGRA3_HUMAN Adhesion G protein-coupled receptor A3 OS=Homo sapiens GN=ADGRA3 PE=1 S | 736 | TTIQCYsLSNY | 0.482 | 0.163 | -0.327 | 0.106 |
| sp\|Q8IWK6\|AGRA3_HUMAN Adhesion G protein-coupled receptor A3 OS=Homo sapiens GN=ADGRA3 PE=1 S | 738 | IQCYSLsNYAV | 0.316 | 0.2 | -0.389 | 0.042 |
| sp\|Q8IWK6\|AGRA3_HUMAN Adhesion G protein-coupled receptor A3 OS=Homo sapiens GN=ADGRA3 PE=1 S | 747 | AVLMDLtGSEL | 0.065 | -0.255 | -1.277 | -0.489 |
| sp\|Q8IWK6\|AGRA3_HUMAN Adhesion G protein-coupled receptor A3 OS=Homo sapiens GN=ADGRA3 PE=1 S | 749 | LMDLTGsELYT | 0.042 | -0.066 | -1.585 | -0.536 |
| sp\|Q8IWK6\|AGRA3_HUMAN Adhesion G protein-coupled receptor A3 OS=Homo sapiens GN=ADGRA3 PE=1 S | 753 | TGSELYtQAAS | 0.057 | -0.177 | -1.379 | -0.5 |
| sp\|Q8IWK6\|AGRA3_HUMAN Adhesion G protein-coupled receptor A3 OS=Homo sapiens GN=ADGRA3 PE=1 S | 757 | LYTQAAsLLHP | 0.362 | 0.073 | -0.157 | 0.093 |
| sp\|Q8IWK6\|AGRA3_HUMAN Adhesion G protein-coupled receptor A3 OS=Homo sapiens GN=ADGRA3 PE=1 S | 765 | LHPVVYtTAII | 0.107 | -0.092 | -1.202 | -0.396 |
| sp\|Q8IWK6\|AGRA3_HUMAN Adhesion G protein-coupled receptor A3 OS=Homo sapiens GN=ADGRA3 PE=1 S | 766 | HPVVYTtAIIL | 0.128 | 0.045 | -0.871 | -0.233 |
| sp\|Q8IWK6\|AGRA3_HUMAN Adhesion G protein-coupled receptor A3 OS=Homo sapiens GN=ADGRA3 PE=1 S | 780 | LLAVIVsYIYH | 0.12 | -0.17 | -0.805 | -0.285 |
| sp\|Q8IWK6\|AGRA3_HUMAN Adhesion G protein-coupled receptor A3 OS=Homo sapiens GN=ADGRA3 PE=1 S | 786 | SYIYHHsLIRI | 0.55 | 0.275 | -0.067 | 0.253 |
| sp\|Q8IWK6\|AGRA3_HUMAN Adhesion G protein-coupled receptor A3 OS=Homo sapiens GN=ADGRA3 PE=1 S | 791 | HSLIRIsLKSW | 0.275 | 0.168 | -0.265 | 0.059 |
| sp\|Q8IWK6\|AGRA3_HUMAN Adhesion G protein-coupled receptor A3 OS=Homo sapiens GN=ADGRA3 PE=1 S | 794 | IRISLKsWHML | 0.266 | 0.248 | -0.55 | -0.012 |

| | | | | | | |
|---|---|---|---|---|---|---|
| sp\|Q8IWK6\|AGRA3_HUMAN Adhesion G protein-coupled receptor A3 OS=Homo sapiens GN=ADGRA3 PE=1 S | 808 | CFHIFLtCVVF | 0.094 | 0.018 | -1.088 | -0.325 |
| sp\|Q8IWK6\|AGRA3_HUMAN Adhesion G protein-coupled receptor A3 OS=Homo sapiens GN=ADGRA3 PE=1 S | 817 | VFVGGItQTRN | 0.121 | -0.249 | -1.318 | -0.482 |
| sp\|Q8IWK6\|AGRA3_HUMAN Adhesion G protein-coupled receptor A3 OS=Homo sapiens GN=ADGRA3 PE=1 S | 819 | VGGITQtRNAS | 0.088 | -0.07 | -0.934 | -0.305 |
| sp\|Q8IWK6\|AGRA3_HUMAN Adhesion G protein-coupled receptor A3 OS=Homo sapiens GN=ADGRA3 PE=1 S | 823 | TQTRNAsICQA | 0.513 | 0.651 | 0.379 | 0.514 |
| sp\|Q8IWK6\|AGRA3_HUMAN Adhesion G protein-coupled receptor A3 OS=Homo sapiens GN=ADGRA3 PE=1 S | 835 | GIILHYsTLAT | 0.262 | 0.213 | -0.575 | -0.033 |
| sp\|Q8IWK6\|AGRA3_HUMAN Adhesion G protein-coupled receptor A3 OS=Homo sapiens GN=ADGRA3 PE=1 S | 836 | IILHYStLATV | 0.288 | -0.027 | -0.602 | -0.114 |
| sp\|Q8IWK6\|AGRA3_HUMAN Adhesion G protein-coupled receptor A3 OS=Homo sapiens GN=ADGRA3 PE=1 S | 839 | HYSTLAtVLWV | 0.165 | 0.011 | -1.091 | -0.305 |
| sp\|Q8IWK6\|AGRA3_HUMAN Adhesion G protein-coupled receptor A3 OS=Homo sapiens GN=ADGRA3 PE=1 S | 846 | VLWVGVtARNI | 0.284 | 0.133 | -0.668 | -0.084 |
| sp\|Q8IWK6\|AGRA3_HUMAN Adhesion G protein-coupled receptor A3 OS=Homo sapiens GN=ADGRA3 PE=1 S | 855 | NIYKQVtKKAK | 0.393 | 0.09 | -0.233 | 0.083 |
| sp\|Q8IWK6\|AGRA3_HUMAN Adhesion G protein-coupled receptor A3 OS=Homo sapiens GN=ADGRA3 PE=1 S | 892 | IIVCGItAAAN | 0.25 | 0.031 | -0.772 | -0.164 |
| sp\|Q8IWK6\|AGRA3_HUMAN Adhesion G protein-coupled receptor A3 OS=Homo sapiens GN=ADGRA3 PE=1 S | 902 | NIKNYGsRPNA | 0.516 | 0.44 | -0.09 | 0.289 |
| sp\|Q8IWK6\|AGRA3_HUMAN Adhesion G protein-coupled receptor A3 OS=Homo sapiens GN=ADGRA3 PE=1 S | 916 | WMAWEPsLGAF | 0.358 | 0.103 | -0.074 | 0.129 |
| sp\|Q8IWK6\|AGRA3_HUMAN Adhesion G protein-coupled receptor A3 OS=Homo sapiens GN=ADGRA3 PE=1 S | 925 | AFYGPAsFITF | 0.15 | -0.079 | -0.521 | -0.15 |
| sp\|Q8IWK6\|AGRA3_HUMAN Adhesion G protein-coupled receptor A3 OS=Homo sapiens GN=ADGRA3 PE=1 S | 928 | GPASFItFVNC | 0.211 | -0.078 | -1.098 | -0.322 |
| sp\|Q8IWK6\|AGRA3_HUMAN Adhesion G protein-coupled receptor A3 OS=Homo sapiens GN=ADGRA3 PE=1 S | 937 | NCMYFLsIFIQ | 0.145 | -0.059 | -0.977 | -0.297 |
| sp\|Q8IWK6\|AGRA3_HUMAN Adhesion G protein-coupled receptor A3 OS=Homo sapiens GN=ADGRA3 PE=1 S | 956 | YELKEPtEEQQ | 0.222 | -0.106 | -0.877 | -0.254 |
| sp\|Q8IWK6\|AGRA3_HUMAN Adhesion G protein-coupled receptor A3 OS=Homo sapiens GN=ADGRA3 PE=1 S | 975 | EINHQDsMSLS | 0.051 | -0.078 | -1.663 | -0.563 |
| sp\|Q8IWK6\|AGRA3_HUMAN Adhesion G protein-coupled receptor A3 OS=Homo sapiens GN=ADGRA3 PE=1 S | 977 | NHQDSMsLSLI | 0.642 | 0.487 | 0.078 | 0.402 |
| sp\|Q8IWK6\|AGRA3_HUMAN Adhesion G protein-coupled receptor A3 OS=Homo sapiens GN=ADGRA3 PE=1 S | 979 | QDSMSLsLIST | 0.101 | -0.051 | -0.602 | -0.184 |
| sp\|Q8IWK6\|AGRA3_HUMAN Adhesion G protein-coupled receptor A3 OS=Homo sapiens GN=ADGRA3 PE=1 S | 982 | MSLSLIsTSAL | 0.144 | -0.094 | -0.901 | -0.284 |
| sp\|Q8IWK6\|AGRA3_HUMAN Adhesion G protein-coupled receptor A3 OS=Homo sapiens GN=ADGRA3 PE=1 S | 983 | SLSLIStSALE | 0.075 | -0.219 | -1.338 | -0.494 |
| sp\|Q8IWK6\|AGRA3_HUMAN Adhesion G protein-coupled receptor A3 OS=Homo sapiens GN=ADGRA3 PE=1 S | 984 | LSLISTsALEN | 0.271 | 0.178 | -0.523 | -0.025 |
| sp\|Q8IWK6\|AGRA3_HUMAN Adhesion G protein-coupled receptor A3 OS=Homo sapiens GN=ADGRA3 PE=1 S | 991 | ALENEHtFHSQ | 0.322 | 0.092 | -0.596 | -0.061 |
| sp\|Q8IWK6\|AGRA3_HUMAN Adhesion G protein-coupled receptor A3 OS=Homo sapiens GN=ADGRA3 PE=1 S | 994 | NEHTFHsQLLG | 0.121 | -0.073 | -1.535 | -0.496 |
| sp\|Q8IWK6\|AGRA3_HUMAN Adhesion G protein-coupled receptor A3 OS=Homo sapiens GN=ADGRA3 PE=1 S | 1000 | SQLLGAsLTLL | 0.208 | -0.008 | -0.701 | -0.167 |
| sp\|Q8IWK6\|AGRA3_HUMAN Adhesion G protein-coupled receptor A3 OS=Homo sapiens GN=ADGRA3 PE=1 S | 1002 | LLGASLtLLLY | 0.427 | 0.296 | -0.097 | 0.209 |
| sp\|Q8IWK6\|AGRA3_HUMAN Adhesion G protein-coupled receptor A3 OS=Homo sapiens GN=ADGRA3 PE=1 S | 1018 | FGALAVsLYYP | 0.485 | 0.117 | -0.151 | 0.15 |
| sp\|Q8IWK6\|AGRA3_HUMAN Adhesion G protein-coupled receptor A3 OS=Homo sapiens GN=ADGRA3 PE=1 S | 1028 | PLDLVFsFVFG | 0.317 | 0.096 | -0.563 | -0.05 |
| sp\|Q8IWK6\|AGRA3_HUMAN Adhesion G protein-coupled receptor A3 OS=Homo sapiens GN=ADGRA3 PE=1 S | 1034 | SFVFGAtSLSF | 0.04 | -0.22 | -1.625 | -0.602 |
| sp\|Q8IWK6\|AGRA3_HUMAN Adhesion G protein-coupled receptor A3 OS=Homo sapiens GN=ADGRA3 PE=1 S | 1035 | FVFGATsLSFS | 0.186 | 0.073 | -0.623 | -0.121 |
| sp\|Q8IWK6\|AGRA3_HUMAN Adhesion G protein-coupled receptor A3 OS=Homo sapiens GN=ADGRA3 PE=1 S | 1037 | FGATSLsFSAF | 0.28 | 0.238 | -0.13 | 0.129 |
| sp\|Q8IWK6\|AGRA3_HUMAN Adhesion G protein-coupled receptor A3 OS=Homo sapiens GN=ADGRA3 PE=1 S | 1039 | ATSLSFsAFFV | 0.314 | 0.115 | -0.837 | -0.136 |
| sp\|Q8IWK6\|AGRA3_HUMAN Adhesion G protein-coupled receptor A3 OS=Homo sapiens GN=ADGRA3 PE=1 S | 1060 | RLAWIMtCCPG | 0.434 | 0.148 | -0.137 | 0.148 |
| sp\|Q8IWK6\|AGRA3_HUMAN Adhesion G protein-coupled receptor A3 OS=Homo sapiens GN=ADGRA3 PE=1 S | 1066 | TCCPGRsSYSV | 0.044 | -0.229 | -1.918 | -0.701 |
| sp\|Q8IWK6\|AGRA3_HUMAN Adhesion G protein-coupled receptor A3 OS=Homo sapiens GN=ADGRA3 PE=1 S | 1067 | CCPGRSsYSVQ | 0.104 | -0.064 | -1.202 | -0.387 |
| sp\|Q8IWK6\|AGRA3_HUMAN Adhesion G protein-coupled receptor A3 OS=Homo sapiens GN=ADGRA3 PE=1 S | 1069 | PGRSSYsVQVN | 0.65 | 0.539 | 0.375 | 0.521 |
| sp\|Q8IWK6\|AGRA3_HUMAN Adhesion G protein-coupled receptor A3 OS=Homo sapiens GN=ADGRA3 PE=1 S | 1079 | NVQPPNsNGTN | 0.206 | -0.058 | -0.865 | -0.239 |
| sp\|Q8IWK6\|AGRA3_HUMAN Adhesion G protein-coupled receptor A3 OS=Homo sapiens GN=ADGRA3 PE=1 S | 1082 | PPNSNGtNGEA | 0.17 | -0.208 | -1.146 | -0.395 |
| sp\|Q8IWK6\|AGRA3_HUMAN Adhesion G protein-coupled receptor A3 OS=Homo sapiens GN=ADGRA3 PE=1 S | 1092 | APKCPNsSAES | 0.177 | -0.219 | -1.161 | -0.401 |
| sp\|Q8IWK6\|AGRA3_HUMAN Adhesion G protein-coupled receptor A3 OS=Homo sapiens GN=ADGRA3 PE=1 S | 1093 | PKCPNSsAESS | 0.131 | -0.096 | -1.063 | -0.343 |
| sp\|Q8IWK6\|AGRA3_HUMAN Adhesion G protein-coupled receptor A3 OS=Homo sapiens GN=ADGRA3 PE=1 S | 1096 | PNSSAEsSCTN | 0.102 | -0.306 | -1.351 | -0.518 |
| sp\|Q8IWK6\|AGRA3_HUMAN Adhesion G protein-coupled receptor A3 OS=Homo sapiens GN=ADGRA3 PE=1 S | 1097 | NSSAESsCTNK | 0.134 | -0.214 | -1.372 | -0.484 |
| sp\|Q8IWK6\|AGRA3_HUMAN Adhesion G protein-coupled receptor A3 OS=Homo sapiens GN=ADGRA3 PE=1 S | 1099 | SAESSCtNKSA | 0.137 | -0.118 | -1.168 | -0.383 |
| sp\|Q8IWK6\|AGRA3_HUMAN Adhesion G protein-coupled receptor A3 OS=Homo sapiens GN=ADGRA3 PE=1 S | 1102 | SSCTNKsASSF | 0.077 | -0.206 | -1.324 | -0.484 |
| sp\|Q8IWK6\|AGRA3_HUMAN Adhesion G protein-coupled receptor A3 OS=Homo sapiens GN=ADGRA3 PE=1 S | 1104 | CTNKSAsSFKN | 0.576 | 0.241 | -0.221 | 0.199 |
| sp\|Q8IWK6\|AGRA3_HUMAN Adhesion G protein-coupled receptor A3 OS=Homo sapiens GN=ADGRA3 PE=1 S | 1105 | TNKSASsFKNS | 0.151 | -0.102 | -0.878 | -0.276 |
| sp\|Q8IWK6\|AGRA3_HUMAN Adhesion G protein-coupled receptor A3 OS=Homo sapiens GN=ADGRA3 PE=1 S | 1109 | ASSFKNsSQGC | 0.14 | -0.288 | -1.334 | -0.494 |
| sp\|Q8IWK6\|AGRA3_HUMAN Adhesion G protein-coupled receptor A3 OS=Homo sapiens GN=ADGRA3 PE=1 S | 1110 | SSFKNSsQGCK | 0.177 | -0.16 | -1.095 | -0.359 |
| sp\|Q8IWK6\|AGRA3_HUMAN Adhesion G protein-coupled receptor A3 OS=Homo sapiens GN=ADGRA3 PE=1 S | 1116 | SQGCKLtNLQA | 0.033 | -0.364 | -1.789 | -0.707 |

| | | | | | | |
|---|---|---|---|---|---|---|
| sp\|Q8IWK6\|AGRA3_HUMAN Adhesion G protein-coupled receptor A3 OS=Homo sapiens GN=ADGRA3 PE=1 S | 1129 | AQCHANsLPLN | 0.833 | 0.657 | 0.547 | 0.679 |
| sp\|Q8IWK6\|AGRA3_HUMAN Adhesion G protein-coupled receptor A3 OS=Homo sapiens GN=ADGRA3 PE=1 S | 1134 | NSLPLNsTPQL | 0.482 | 0.351 | -0.154 | 0.226 |
| sp\|Q8IWK6\|AGRA3_HUMAN Adhesion G protein-coupled receptor A3 OS=Homo sapiens GN=ADGRA3 PE=1 S | 1135 | SLPLNStPQLD | 0.07 | -0.366 | -1.353 | -0.55 |
| sp\|Q8IWK6\|AGRA3_HUMAN Adhesion G protein-coupled receptor A3 OS=Homo sapiens GN=ADGRA3 PE=1 S | 1141 | TPQLDNsLTEH | 0.336 | -0.057 | -0.887 | -0.203 |
| sp\|Q8IWK6\|AGRA3_HUMAN Adhesion G protein-coupled receptor A3 OS=Homo sapiens GN=ADGRA3 PE=1 S | 1143 | QLDNSLtEHSM | 0.223 | 0.214 | -0.355 | 0.027 |
| sp\|Q8IWK6\|AGRA3_HUMAN Adhesion G protein-coupled receptor A3 OS=Homo sapiens GN=ADGRA3 PE=1 S | 1146 | NSLTEHsMDND | 0.371 | 0.185 | -0.698 | -0.047 |
| sp\|Q8IWK6\|AGRA3_HUMAN Adhesion G protein-coupled receptor A3 OS=Homo sapiens GN=ADGRA3 PE=1 S | 1164 | LEVQFRtNVHS | 0.056 | -0.233 | -1.601 | -0.593 |
| sp\|Q8IWK6\|AGRA3_HUMAN Adhesion G protein-coupled receptor A3 OS=Homo sapiens GN=ADGRA3 PE=1 S | 1168 | FRTNVHsSRHH | 0.188 | 0.116 | -0.724 | -0.14 |
| sp\|Q8IWK6\|AGRA3_HUMAN Adhesion G protein-coupled receptor A3 OS=Homo sapiens GN=ADGRA3 PE=1 S | 1169 | RTNVHSsRHHK | 0.094 | -0.253 | -1.454 | -0.538 |
| sp\|Q8IWK6\|AGRA3_HUMAN Adhesion G protein-coupled receptor A3 OS=Homo sapiens GN=ADGRA3 PE=1 S | 1176 | RHHKNRsKGHR | 0.234 | -0.014 | -0.824 | -0.201 |
| sp\|Q8IWK6\|AGRA3_HUMAN Adhesion G protein-coupled receptor A3 OS=Homo sapiens GN=ADGRA3 PE=1 S | 1182 | SKGHRAsRLTV | 0.237 | -0.016 | -1.121 | -0.3 |
| sp\|Q8IWK6\|AGRA3_HUMAN Adhesion G protein-coupled receptor A3 OS=Homo sapiens GN=ADGRA3 PE=1 S | 1185 | HRASRLtVLRE | 0.181 | 0.17 | -0.772 | -0.14 |
| sp\|Q8IWK6\|AGRA3_HUMAN Adhesion G protein-coupled receptor A3 OS=Homo sapiens GN=ADGRA3 PE=1 S | 1196 | YAYDVPtSVEG | 0.121 | -0.157 | -1.198 | -0.411 |
| sp\|Q8IWK6\|AGRA3_HUMAN Adhesion G protein-coupled receptor A3 OS=Homo sapiens GN=ADGRA3 PE=1 S | 1197 | AYDVPTsVEGS | 0.091 | -0.235 | -1.169 | -0.438 |
| sp\|Q8IWK6\|AGRA3_HUMAN Adhesion G protein-coupled receptor A3 OS=Homo sapiens GN=ADGRA3 PE=1 S | 1201 | PTSVEGsVQNG | 0.126 | -0.141 | -1.353 | -0.456 |
| sp\|Q8IWK6\|AGRA3_HUMAN Adhesion G protein-coupled receptor A3 OS=Homo sapiens GN=ADGRA3 PE=1 S | 1209 | QNGLPKsRLGN | 0.066 | -0.327 | -1.206 | -0.489 |
| sp\|Q8IWK6\|AGRA3_HUMAN Adhesion G protein-coupled receptor A3 OS=Homo sapiens GN=ADGRA3 PE=1 S | 1218 | GNNEGHsRSRR | 0.078 | -0.275 | -1.646 | -0.614 |
| sp\|Q8IWK6\|AGRA3_HUMAN Adhesion G protein-coupled receptor A3 OS=Homo sapiens GN=ADGRA3 PE=1 S | 1220 | NEGHSRsRRAY | 0.262 | 0.065 | -0.739 | -0.137 |
| sp\|Q8IWK6\|AGRA3_HUMAN Adhesion G protein-coupled receptor A3 OS=Homo sapiens GN=ADGRA3 PE=1 S | 1239 | NPPQQDsSDAC | 0.097 | -0.228 | -1.404 | -0.512 |
| sp\|Q8IWK6\|AGRA3_HUMAN Adhesion G protein-coupled receptor A3 OS=Homo sapiens GN=ADGRA3 PE=1 S | 1240 | PPQQDSsDACS | 0.115 | -0.101 | -1.198 | -0.395 |
| sp\|Q8IWK6\|AGRA3_HUMAN Adhesion G protein-coupled receptor A3 OS=Homo sapiens GN=ADGRA3 PE=1 S | 1244 | DSSDACsTLPK | 0.219 | -0.194 | -1.232 | -0.402 |
| sp\|Q8IWK6\|AGRA3_HUMAN Adhesion G protein-coupled receptor A3 OS=Homo sapiens GN=ADGRA3 PE=1 S | 1245 | SSDACStLPKS | 0.422 | 0.277 | -0.353 | 0.115 |
| sp\|Q8IWK6\|AGRA3_HUMAN Adhesion G protein-coupled receptor A3 OS=Homo sapiens GN=ADGRA3 PE=1 S | 1249 | CSTLPKsSRNF | 0.176 | -0.156 | -0.689 | -0.223 |
| sp\|Q8IWK6\|AGRA3_HUMAN Adhesion G protein-coupled receptor A3 OS=Homo sapiens GN=ADGRA3 PE=1 S | 1250 | STLPKSsRNFE | 0.042 | -0.47 | -1.776 | -0.735 |
| sp\|Q8IWK6\|AGRA3_HUMAN Adhesion G protein-coupled receptor A3 OS=Homo sapiens GN=ADGRA3 PE=1 S | 1258 | NFEKPVsTTSK | 0.337 | -0.167 | -0.478 | -0.103 |
| sp\|Q8IWK6\|AGRA3_HUMAN Adhesion G protein-coupled receptor A3 OS=Homo sapiens GN=ADGRA3 PE=1 S | 1259 | FEKPVStTSKK | 0.075 | -0.233 | -1.389 | -0.516 |
| sp\|Q8IWK6\|AGRA3_HUMAN Adhesion G protein-coupled receptor A3 OS=Homo sapiens GN=ADGRA3 PE=1 S | 1260 | EKPVSTtSKKD | 0.201 | 0.099 | -0.52 | -0.073 |
| sp\|Q8IWK6\|AGRA3_HUMAN Adhesion G protein-coupled receptor A3 OS=Homo sapiens GN=ADGRA3 PE=1 S | 1261 | KPVSTTsKKDA | 0.116 | -0.133 | -0.919 | -0.312 |
| sp\|Q8IWK6\|AGRA3_HUMAN Adhesion G protein-coupled receptor A3 OS=Homo sapiens GN=ADGRA3 PE=1 S | 1280 | LENQQKsYGLN | 0.201 | 0.008 | -0.735 | -0.175 |
| sp\|Q8IWK6\|AGRA3_HUMAN Adhesion G protein-coupled receptor A3 OS=Homo sapiens GN=ADGRA3 PE=1 S | 1294 | QNGPIKsNGQE | 0.137 | -0.21 | -0.7 | -0.258 |
| sp\|Q8IWK6\|AGRA3_HUMAN Adhesion G protein-coupled receptor A3 OS=Homo sapiens GN=ADGRA3 PE=1 S | 1304 | EGPLLGtDSTG | 0.044 | -0.311 | -1.738 | -0.668 |
| sp\|Q8IWK6\|AGRA3_HUMAN Adhesion G protein-coupled receptor A3 OS=Homo sapiens GN=ADGRA3 PE=1 S | 1306 | PLLGTDsTGNV | 0.268 | 0.102 | -0.874 | -0.168 |
| sp\|Q8IWK6\|AGRA3_HUMAN Adhesion G protein-coupled receptor A3 OS=Homo sapiens GN=ADGRA3 PE=1 S | 1307 | LLGTDStGNVR | 0.124 | -0.087 | -0.852 | -0.272 |
| sp\|Q8IWK6\|AGRA3_HUMAN Adhesion G protein-coupled receptor A3 OS=Homo sapiens GN=ADGRA3 PE=1 S | 1312 | STGNVRtGLWK | 0.036 | -0.289 | -1.685 | -0.646 |
| sp\|Q8IWK6\|AGRA3_HUMAN Adhesion G protein-coupled receptor A3 OS=Homo sapiens GN=ADGRA3 PE=1 S | 1319 | GLWKHEtTV-- | 0.46 | 0.392 | -0.496 | 0.119 |
| sp\|Q8IWK6\|AGRA3_HUMAN Adhesion G protein-coupled receptor A3 OS=Homo sapiens GN=ADGRA3 PE=1 S | 1320 | LWKHETtV--- | 0.231 | 0.133 | -0.617 | -0.084 |
| sp\|Q86SQ4\|AGRG6_HUMAN Adhesion G-protein coupled receptor G6 OS=Homo sapiens GN=ADGRG6 PE=1 S | 316 | FRLWNFtMNAK | 0.507 | 0.446 | 0.225 | 0.393 |
| sp\|Q86SQ4\|AGRG6_HUMAN Adhesion G-protein coupled receptor G6 OS=Homo sapiens GN=ADGRG6 PE=1 S | 323 | MNAKILsNLSC | 0.066 | -0.219 | -1.311 | -0.488 |
| sp\|Q86SQ4\|AGRG6_HUMAN Adhesion G-protein coupled receptor G6 OS=Homo sapiens GN=ADGRG6 PE=1 S | 326 | KILSNLsCNVK | 0.183 | -0.011 | -0.756 | -0.195 |
| sp\|Q86SQ4\|AGRG6_HUMAN Adhesion G-protein coupled receptor G6 OS=Homo sapiens GN=ADGRG6 PE=1 S | 352 | LALKAEsNLSC | 0.105 | -0.205 | -1.463 | -0.521 |
| sp\|Q86SQ4\|AGRG6_HUMAN Adhesion G-protein coupled receptor G6 OS=Homo sapiens GN=ADGRG6 PE=1 S | 355 | KAESNLsCGSY | 0.13 | -0.122 | -1.09 | -0.361 |
| sp\|Q86SQ4\|AGRG6_HUMAN Adhesion G-protein coupled receptor G6 OS=Homo sapiens GN=ADGRG6 PE=1 S | 358 | SNLSCGsYLIP | 0.04 | -0.444 | -1.747 | -0.717 |
| sp\|Q86SQ4\|AGRG6_HUMAN Adhesion G-protein coupled receptor G6 OS=Homo sapiens GN=ADGRG6 PE=1 S | 370 | PAAELAsCADL | 0.175 | 0.028 | -0.896 | -0.231 |
| sp\|Q86SQ4\|AGRG6_HUMAN Adhesion G-protein coupled receptor G6 OS=Homo sapiens GN=ADGRG6 PE=1 S | 376 | SCADLGtLCQA | 0.136 | -0.214 | -1.186 | -0.421 |
| sp\|Q86SQ4\|AGRG6_HUMAN Adhesion G-protein coupled receptor G6 OS=Homo sapiens GN=ADGRG6 PE=1 S | 381 | GTLCQAtVNSP | 0.181 | -0.069 | -1.045 | -0.311 |
| sp\|Q86SQ4\|AGRG6_HUMAN Adhesion G-protein coupled receptor G6 OS=Homo sapiens GN=ADGRG6 PE=1 S | 384 | CQATVNsPSTT | 0.081 | -0.428 | -1.244 | -0.53 |
| sp\|Q86SQ4\|AGRG6_HUMAN Adhesion G-protein coupled receptor G6 OS=Homo sapiens GN=ADGRG6 PE=1 S | 386 | ATVNSPsTTPP | 0.167 | -0.104 | -0.883 | -0.273 |
| sp\|Q86SQ4\|AGRG6_HUMAN Adhesion G-protein coupled receptor G6 OS=Homo sapiens GN=ADGRG6 PE=1 S | 387 | TVNSPStTPPT | 0.124 | 0.079 | -0.835 | -0.211 |
| sp\|Q86SQ4\|AGRG6_HUMAN Adhesion G-protein coupled receptor G6 OS=Homo sapiens GN=ADGRG6 PE=1 S | 388 | VNSPSTtPPTV | 0.346 | 0.159 | -0.347 | 0.053 |

| | | | | | | |
|---|---|---|---|---|---|---|
| sp\|Q86SQ4\|AGRG6_HUMAN Adhesion G-protein coupled receptor G6 OS=Homo sapiens GN=ADGRG6 PE=1 S | 391 | PSTTPPtVTTN | 0.175 | -0.24 | -0.899 | -0.321 |
| sp\|Q86SQ4\|AGRG6_HUMAN Adhesion G-protein coupled receptor G6 OS=Homo sapiens GN=ADGRG6 PE=1 S | 393 | TTPPTVtTNMP | 0.083 | -0.282 | -1.269 | -0.489 |
| sp\|Q86SQ4\|AGRG6_HUMAN Adhesion G-protein coupled receptor G6 OS=Homo sapiens GN=ADGRG6 PE=1 S | 394 | TPPTVTtNMPV | 0.081 | -0.291 | -1.734 | -0.648 |
| sp\|Q86SQ4\|AGRG6_HUMAN Adhesion G-protein coupled receptor G6 OS=Homo sapiens GN=ADGRG6 PE=1 S | 399 | TTNMPVtNRID | 0.283 | -0.303 | -0.921 | -0.314 |
| sp\|Q86SQ4\|AGRG6_HUMAN Adhesion G-protein coupled receptor G6 OS=Homo sapiens GN=ADGRG6 PE=1 S | 415 | GIIYRIsVVIQ | 0.493 | 0.259 | -0.294 | 0.153 |
| sp\|Q86SQ4\|AGRG6_HUMAN Adhesion G-protein coupled receptor G6 OS=Homo sapiens GN=ADGRG6 PE=1 S | 431 | PEVKVQsKVAE | 0.338 | 0.053 | -0.524 | -0.044 |
| sp\|Q86SQ4\|AGRG6_HUMAN Adhesion G-protein coupled receptor G6 OS=Homo sapiens GN=ADGRG6 PE=1 S | 439 | VAEWLNsTFQN | 0.231 | -0.169 | -0.85 | -0.263 |
| sp\|Q86SQ4\|AGRG6_HUMAN Adhesion G-protein coupled receptor G6 OS=Homo sapiens GN=ADGRG6 PE=1 S | 440 | AEWLNStFQNW | 0.156 | 0.088 | -1.022 | -0.259 |
| sp\|Q86SQ4\|AGRG6_HUMAN Adhesion G-protein coupled receptor G6 OS=Homo sapiens GN=ADGRG6 PE=1 S | 447 | FQNWNYtVYVV | 0.257 | 0.121 | -0.531 | -0.051 |
| sp\|Q86SQ4\|AGRG6_HUMAN Adhesion G-protein coupled receptor G6 OS=Homo sapiens GN=ADGRG6 PE=1 S | 454 | VYVVNIsFHLS | 0.26 | 0.026 | -0.636 | -0.117 |
| sp\|Q86SQ4\|AGRG6_HUMAN Adhesion G-protein coupled receptor G6 OS=Homo sapiens GN=ADGRG6 PE=1 S | 458 | NISFHLsAGED | 0.411 | 0.186 | -0.425 | 0.057 |
| sp\|Q86SQ4\|AGRG6_HUMAN Adhesion G-protein coupled receptor G6 OS=Homo sapiens GN=ADGRG6 PE=1 S | 469 | KIKVKRsLEDE | 0.228 | 0.06 | -0.697 | -0.136 |
| sp\|Q86SQ4\|AGRG6_HUMAN Adhesion G-protein coupled receptor G6 OS=Homo sapiens GN=ADGRG6 PE=1 S | 487 | LLVYNAtNNTN | 0.395 | 0.103 | -0.33 | 0.056 |
| sp\|Q86SQ4\|AGRG6_HUMAN Adhesion G-protein coupled receptor G6 OS=Homo sapiens GN=ADGRG6 PE=1 S | 490 | YNATNNtNLEG | 0.185 | -0.133 | -0.943 | -0.297 |
| sp\|Q86SQ4\|AGRG6_HUMAN Adhesion G-protein coupled receptor G6 OS=Homo sapiens GN=ADGRG6 PE=1 S | 507 | LLKNNEsLDEG | 0.602 | 0.2 | 0.265 | 0.356 |
| sp\|Q86SQ4\|AGRG6_HUMAN Adhesion G-protein coupled receptor G6 OS=Homo sapiens GN=ADGRG6 PE=1 S | 516 | EGLRLHtVNVR | 0.644 | 0.977 | 0.593 | 0.738 |
| sp\|Q86SQ4\|AGRG6_HUMAN Adhesion G-protein coupled receptor G6 OS=Homo sapiens GN=ADGRG6 PE=1 S | 538 | KGYYWPsIQPS | 0.095 | -0.192 | -0.871 | -0.323 |
| sp\|Q86SQ4\|AGRG6_HUMAN Adhesion G-protein coupled receptor G6 OS=Homo sapiens GN=ADGRG6 PE=1 S | 542 | WPSIQPsEYVL | 0.093 | -0.047 | -1.066 | -0.34 |
| sp\|Q86SQ4\|AGRG6_HUMAN Adhesion G-protein coupled receptor G6 OS=Homo sapiens GN=ADGRG6 PE=1 S | 555 | PDKPGFsASRI | 0.114 | -0.189 | -1.062 | -0.379 |
| sp\|Q86SQ4\|AGRG6_HUMAN Adhesion G-protein coupled receptor G6 OS=Homo sapiens GN=ADGRG6 PE=1 S | 557 | KPGFSAsRICF | 0.174 | 0.085 | -0.788 | -0.176 |
| sp\|Q86SQ4\|AGRG6_HUMAN Adhesion G-protein coupled receptor G6 OS=Homo sapiens GN=ADGRG6 PE=1 S | 565 | ICFYNAtNPLV | 0.77 | 0.571 | 0.064 | 0.468 |
| sp\|Q86SQ4\|AGRG6_HUMAN Adhesion G-protein coupled receptor G6 OS=Homo sapiens GN=ADGRG6 PE=1 S | 570 | ATNPLVtYWGP | 0.138 | -0.313 | -1.316 | -0.497 |
| sp\|Q86SQ4\|AGRG6_HUMAN Adhesion G-protein coupled receptor G6 OS=Homo sapiens GN=ADGRG6 PE=1 S | 578 | WGPVDIsNCLK | 0.055 | -0.389 | -1.505 | -0.613 |
| sp\|Q86SQ4\|AGRG6_HUMAN Adhesion G-protein coupled receptor G6 OS=Homo sapiens GN=ADGRG6 PE=1 S | 595 | NQILNLtADGQ | 0.131 | -0.086 | -0.934 | -0.296 |
| sp\|Q86SQ4\|AGRG6_HUMAN Adhesion G-protein coupled receptor G6 OS=Homo sapiens GN=ADGRG6 PE=1 S | 602 | ADGQNLtSANI | 0.072 | -0.229 | -1.243 | -0.467 |
| sp\|Q86SQ4\|AGRG6_HUMAN Adhesion G-protein coupled receptor G6 OS=Homo sapiens GN=ADGRG6 PE=1 S | 603 | DGQNLTsANIT | 0.193 | -0.05 | -0.78 | -0.212 |
| sp\|Q86SQ4\|AGRG6_HUMAN Adhesion G-protein coupled receptor G6 OS=Homo sapiens GN=ADGRG6 PE=1 S | 607 | LTSANItNIVE | 0.165 | -0.189 | -0.935 | -0.32 |
| sp\|Q86SQ4\|AGRG6_HUMAN Adhesion G-protein coupled receptor G6 OS=Homo sapiens GN=ADGRG6 PE=1 S | 626 | EENIDItLGST | 0.142 | -0.072 | -0.883 | -0.271 |
| sp\|Q86SQ4\|AGRG6_HUMAN Adhesion G-protein coupled receptor G6 OS=Homo sapiens GN=ADGRG6 PE=1 S | 629 | IDITLGsTLMN | 0.042 | -0.354 | -1.868 | -0.727 |
| sp\|Q86SQ4\|AGRG6_HUMAN Adhesion G-protein coupled receptor G6 OS=Homo sapiens GN=ADGRG6 PE=1 S | 630 | DITLGStLMNI | 0.323 | 0.102 | -0.602 | -0.059 |
| sp\|Q86SQ4\|AGRG6_HUMAN Adhesion G-protein coupled receptor G6 OS=Homo sapiens GN=ADGRG6 PE=1 S | 636 | TLMNIFsNILS | 0.123 | 0.054 | -0.609 | -0.144 |
| sp\|Q86SQ4\|AGRG6_HUMAN Adhesion G-protein coupled receptor G6 OS=Homo sapiens GN=ADGRG6 PE=1 S | 640 | IFSNILsSSDS | 0.091 | -0.167 | -0.812 | -0.296 |
| sp\|Q86SQ4\|AGRG6_HUMAN Adhesion G-protein coupled receptor G6 OS=Homo sapiens GN=ADGRG6 PE=1 S | 641 | FSNILSsSDSD | 0.06 | -0.159 | -1.414 | -0.504 |
| sp\|Q86SQ4\|AGRG6_HUMAN Adhesion G-protein coupled receptor G6 OS=Homo sapiens GN=ADGRG6 PE=1 S | 642 | SNILSSsDSDL | 0.165 | 0.066 | -0.942 | -0.237 |
| sp\|Q86SQ4\|AGRG6_HUMAN Adhesion G-protein coupled receptor G6 OS=Homo sapiens GN=ADGRG6 PE=1 S | 644 | ILSSSDsDLLE | 0.382 | 0.09 | -0.57 | -0.033 |
| sp\|Q86SQ4\|AGRG6_HUMAN Adhesion G-protein coupled receptor G6 OS=Homo sapiens GN=ADGRG6 PE=1 S | 649 | DSDLLEsSSEA | 0.074 | -0.35 | -1.764 | -0.68 |
| sp\|Q86SQ4\|AGRG6_HUMAN Adhesion G-protein coupled receptor G6 OS=Homo sapiens GN=ADGRG6 PE=1 S | 650 | SDLLESsSEAL | 0.043 | -0.243 | -1.589 | -0.596 |
| sp\|Q86SQ4\|AGRG6_HUMAN Adhesion G-protein coupled receptor G6 OS=Homo sapiens GN=ADGRG6 PE=1 S | 651 | DLLESSsEALK | 0.291 | 0.164 | -0.509 | -0.018 |
| sp\|Q86SQ4\|AGRG6_HUMAN Adhesion G-protein coupled receptor G6 OS=Homo sapiens GN=ADGRG6 PE=1 S | 656 | SSEALKtIDEL | 0.168 | -0.132 | -0.741 | -0.235 |
| sp\|Q86SQ4\|AGRG6_HUMAN Adhesion G-protein coupled receptor G6 OS=Homo sapiens GN=ADGRG6 PE=1 S | 668 | FKIDLNsTSHV | 0.198 | 0.019 | -1.19 | -0.324 |
| sp\|Q86SQ4\|AGRG6_HUMAN Adhesion G-protein coupled receptor G6 OS=Homo sapiens GN=ADGRG6 PE=1 S | 669 | KIDLNStSHVN | 0.132 | -0.069 | -1.203 | -0.38 |
| sp\|Q86SQ4\|AGRG6_HUMAN Adhesion G-protein coupled receptor G6 OS=Homo sapiens GN=ADGRG6 PE=1 S | 670 | IDLNSTsHVNI | 0.381 | 0.187 | -0.074 | 0.165 |
| sp\|Q86SQ4\|AGRG6_HUMAN Adhesion G-protein coupled receptor G6 OS=Homo sapiens GN=ADGRG6 PE=1 S | 675 | TSHVNItTRNL | 0.097 | -0.198 | -1.202 | -0.434 |
| sp\|Q86SQ4\|AGRG6_HUMAN Adhesion G-protein coupled receptor G6 OS=Homo sapiens GN=ADGRG6 PE=1 S | 676 | SHVNITtRNLA | 0.079 | -0.181 | -1.155 | -0.419 |
| sp\|Q86SQ4\|AGRG6_HUMAN Adhesion G-protein coupled receptor G6 OS=Homo sapiens GN=ADGRG6 PE=1 S | 682 | TRNLALsVSSL | 0.084 | 0.116 | -1.171 | -0.324 |
| sp\|Q86SQ4\|AGRG6_HUMAN Adhesion G-protein coupled receptor G6 OS=Homo sapiens GN=ADGRG6 PE=1 S | 684 | NLALSVsSLLP | 0.358 | 0.177 | -0.324 | 0.07 |
| sp\|Q86SQ4\|AGRG6_HUMAN Adhesion G-protein coupled receptor G6 OS=Homo sapiens GN=ADGRG6 PE=1 S | 685 | LALSVSsLLPG | 0.109 | -0.215 | -1.024 | -0.377 |
| sp\|Q86SQ4\|AGRG6_HUMAN Adhesion G-protein coupled receptor G6 OS=Homo sapiens GN=ADGRG6 PE=1 S | 690 | SSLLPGtNAIS | 0.04 | -0.457 | -1.881 | -0.766 |
| sp\|Q86SQ4\|AGRG6_HUMAN Adhesion G-protein coupled receptor G6 OS=Homo sapiens GN=ADGRG6 PE=1 S | 694 | PGTNAIsNFSI | 0.25 | 0.023 | -0.341 | -0.023 |

| | | | | | | |
|---|---|---|---|---|---|---|
| sp\|Q86SQ4\|AGRG6_HUMAN Adhesion G-protein coupled receptor G6 OS=Homo sapiens GN=ADGRG6 PE=1 S | 697 | NAISNFsIGLP | 0.129 | -0.117 | -0.859 | -0.282 |
| sp\|Q86SQ4\|AGRG6_HUMAN Adhesion G-protein coupled receptor G6 OS=Homo sapiens GN=ADGRG6 PE=1 S | 702 | FSIGLPsNNES | 0.109 | -0.127 | -1.2 | -0.406 |
| sp\|Q86SQ4\|AGRG6_HUMAN Adhesion G-protein coupled receptor G6 OS=Homo sapiens GN=ADGRG6 PE=1 S | 706 | LPSNNEsYFQM | 0.097 | -0.25 | -0.9 | -0.351 |
| sp\|Q86SQ4\|AGRG6_HUMAN Adhesion G-protein coupled receptor G6 OS=Homo sapiens GN=ADGRG6 PE=1 S | 714 | FQMDFEsGQVD | 0.178 | 0.021 | -0.894 | -0.232 |
| sp\|Q86SQ4\|AGRG6_HUMAN Adhesion G-protein coupled receptor G6 OS=Homo sapiens GN=ADGRG6 PE=1 S | 722 | QVDPLAsVILP | 0.063 | -0.185 | -1.263 | -0.462 |
| sp\|Q86SQ4\|AGRG6_HUMAN Adhesion G-protein coupled receptor G6 OS=Homo sapiens GN=ADGRG6 PE=1 S | 734 | NLLENLsPEDS | 0.082 | -0.273 | -1.146 | -0.446 |
| sp\|Q86SQ4\|AGRG6_HUMAN Adhesion G-protein coupled receptor G6 OS=Homo sapiens GN=ADGRG6 PE=1 S | 738 | NLSPEDsVLVR | 0.161 | -0.12 | -1.088 | -0.349 |
| sp\|Q86SQ4\|AGRG6_HUMAN Adhesion G-protein coupled receptor G6 OS=Homo sapiens GN=ADGRG6 PE=1 S | 747 | VRRAQFtFFNK | 0.513 | 0.479 | -0.135 | 0.286 |
| sp\|Q86SQ4\|AGRG6_HUMAN Adhesion G-protein coupled receptor G6 OS=Homo sapiens GN=ADGRG6 PE=1 S | 752 | FTFFNKtGLFQ | 0.183 | -0.077 | -0.867 | -0.254 |
| sp\|Q86SQ4\|AGRG6_HUMAN Adhesion G-protein coupled receptor G6 OS=Homo sapiens GN=ADGRG6 PE=1 S | 764 | VGPQRKtLVSY | 0.439 | 0.159 | -0.059 | 0.18 |
| sp\|Q86SQ4\|AGRG6_HUMAN Adhesion G-protein coupled receptor G6 OS=Homo sapiens GN=ADGRG6 PE=1 S | 767 | QRKTLVsYVMA | 0.209 | 0.012 | -0.801 | -0.193 |
| sp\|Q86SQ4\|AGRG6_HUMAN Adhesion G-protein coupled receptor G6 OS=Homo sapiens GN=ADGRG6 PE=1 S | 773 | SYVMACsIGNI | 0.453 | 0.104 | -0.552 | 0.002 |
| sp\|Q86SQ4\|AGRG6_HUMAN Adhesion G-protein coupled receptor G6 OS=Homo sapiens GN=ADGRG6 PE=1 S | 778 | CSIGNItIQNL | 0.226 | 0.008 | -0.546 | -0.104 |
| sp\|Q86SQ4\|AGRG6_HUMAN Adhesion G-protein coupled receptor G6 OS=Homo sapiens GN=ADGRG6 PE=1 S | 793 | QIKIKHtRTQE | 0.247 | -0.011 | -0.751 | -0.172 |
| sp\|Q86SQ4\|AGRG6_HUMAN Adhesion G-protein coupled receptor G6 OS=Homo sapiens GN=ADGRG6 PE=1 S | 795 | KIKHTRtQEVH | 0.068 | -0.12 | -1.562 | -0.538 |
| sp\|Q86SQ4\|AGRG6_HUMAN Adhesion G-protein coupled receptor G6 OS=Homo sapiens GN=ADGRG6 PE=1 S | 813 | DLNKNKsFGGW | 0.773 | 0.252 | 0.359 | 0.461 |
| sp\|Q86SQ4\|AGRG6_HUMAN Adhesion G-protein coupled receptor G6 OS=Homo sapiens GN=ADGRG6 PE=1 S | 819 | SFGGWNtSGCV | 0.19 | 0.057 | -1.014 | -0.256 |
| sp\|Q86SQ4\|AGRG6_HUMAN Adhesion G-protein coupled receptor G6 OS=Homo sapiens GN=ADGRG6 PE=1 S | 820 | FGGWNTsGCVA | 0.144 | -0.166 | -0.72 | -0.247 |
| sp\|Q86SQ4\|AGRG6_HUMAN Adhesion G-protein coupled receptor G6 OS=Homo sapiens GN=ADGRG6 PE=1 S | 828 | CVAHRDsDASE | 0.189 | -0.055 | -0.9 | -0.255 |
| sp\|Q86SQ4\|AGRG6_HUMAN Adhesion G-protein coupled receptor G6 OS=Homo sapiens GN=ADGRG6 PE=1 S | 831 | HRDSDAsETVC | 0.223 | 0.169 | -0.899 | -0.169 |
| sp\|Q86SQ4\|AGRG6_HUMAN Adhesion G-protein coupled receptor G6 OS=Homo sapiens GN=ADGRG6 PE=1 S | 833 | DSDASEtVCLC | 0.182 | -0.143 | -1.192 | -0.384 |
| sp\|Q86SQ4\|AGRG6_HUMAN Adhesion G-protein coupled receptor G6 OS=Homo sapiens GN=ADGRG6 PE=1 S | 841 | CLCNHFtHFGV | 0.529 | 0.178 | -0.122 | 0.195 |
| sp\|Q86SQ4\|AGRG6_HUMAN Adhesion G-protein coupled receptor G6 OS=Homo sapiens GN=ADGRG6 PE=1 S | 852 | LMDLPRsASQL | 0.058 | -0.081 | -1.431 | -0.485 |
| sp\|Q86SQ4\|AGRG6_HUMAN Adhesion G-protein coupled receptor G6 OS=Homo sapiens GN=ADGRG6 PE=1 S | 854 | DLPRSAsQLDA | 0.868 | 1.081 | 0.624 | 0.858 |
| sp\|Q86SQ4\|AGRG6_HUMAN Adhesion G-protein coupled receptor G6 OS=Homo sapiens GN=ADGRG6 PE=1 S | 861 | QLDARNtKVLT | 0.395 | 0.141 | -0.386 | 0.05 |
| sp\|Q86SQ4\|AGRG6_HUMAN Adhesion G-protein coupled receptor G6 OS=Homo sapiens GN=ADGRG6 PE=1 S | 865 | RNTKVLtFISY | 0.221 | -0.002 | -0.602 | -0.128 |
| sp\|Q86SQ4\|AGRG6_HUMAN Adhesion G-protein coupled receptor G6 OS=Homo sapiens GN=ADGRG6 PE=1 S | 868 | KVLTFIsYIGC | 0.08 | -0.283 | -1.308 | -0.504 |
| sp\|Q86SQ4\|AGRG6_HUMAN Adhesion G-protein coupled receptor G6 OS=Homo sapiens GN=ADGRG6 PE=1 S | 875 | YIGCGIsAIFS | 0.075 | -0.115 | -1.277 | -0.439 |
| sp\|Q86SQ4\|AGRG6_HUMAN Adhesion G-protein coupled receptor G6 OS=Homo sapiens GN=ADGRG6 PE=1 S | 879 | GISAIFsAATL | 0.246 | 0.063 | -0.581 | -0.091 |
| sp\|Q86SQ4\|AGRG6_HUMAN Adhesion G-protein coupled receptor G6 OS=Homo sapiens GN=ADGRG6 PE=1 S | 882 | AIFSAAtLLTY | 0.502 | 0.154 | -0.394 | 0.087 |
| sp\|Q86SQ4\|AGRG6_HUMAN Adhesion G-protein coupled receptor G6 OS=Homo sapiens GN=ADGRG6 PE=1 S | 885 | SAATLLtYVAF | 0.039 | -0.264 | -1.539 | -0.588 |
| sp\|Q86SQ4\|AGRG6_HUMAN Adhesion G-protein coupled receptor G6 OS=Homo sapiens GN=ADGRG6 PE=1 S | 898 | LRRDYPsKILM | 0.398 | 0.385 | -0.125 | 0.219 |
| sp\|Q86SQ4\|AGRG6_HUMAN Adhesion G-protein coupled receptor G6 OS=Homo sapiens GN=ADGRG6 PE=1 S | 905 | KILMNLsTALL | 0.119 | -0.101 | -0.909 | -0.297 |
| sp\|Q86SQ4\|AGRG6_HUMAN Adhesion G-protein coupled receptor G6 OS=Homo sapiens GN=ADGRG6 PE=1 S | 906 | ILMNLStALLF | 0.085 | -0.026 | -1.061 | -0.334 |
| sp\|Q86SQ4\|AGRG6_HUMAN Adhesion G-protein coupled receptor G6 OS=Homo sapiens GN=ADGRG6 PE=1 S | 922 | LLDGWItSFNV | 0.266 | 0.072 | -0.789 | -0.15 |
| sp\|Q86SQ4\|AGRG6_HUMAN Adhesion G-protein coupled receptor G6 OS=Homo sapiens GN=ADGRG6 PE=1 S | 923 | LDGWITsFNVD | 0.225 | -0.023 | -0.238 | -0.012 |
| sp\|Q86SQ4\|AGRG6_HUMAN Adhesion G-protein coupled receptor G6 OS=Homo sapiens GN=ADGRG6 PE=1 S | 944 | HFFLLAtFTWM | 0.346 | 0.12 | -0.365 | 0.034 |
| sp\|Q86SQ4\|AGRG6_HUMAN Adhesion G-protein coupled receptor G6 OS=Homo sapiens GN=ADGRG6 PE=1 S | 946 | FLLATFtWMGL | 0.309 | 0.269 | -0.246 | 0.111 |
| sp\|Q86SQ4\|AGRG6_HUMAN Adhesion G-protein coupled receptor G6 OS=Homo sapiens GN=ADGRG6 PE=1 S | 965 | LVKVFNtYIRR | 0.123 | -0.116 | -1.009 | -0.334 |
| sp\|Q86SQ4\|AGRG6_HUMAN Adhesion G-protein coupled receptor G6 OS=Homo sapiens GN=ADGRG6 PE=1 S | 987 | LPALVVsVVLA | 0.228 | -0.075 | -0.675 | -0.174 |
| sp\|Q86SQ4\|AGRG6_HUMAN Adhesion G-protein coupled receptor G6 OS=Homo sapiens GN=ADGRG6 PE=1 S | 992 | VSVVLAsRNNN | 0.103 | -0.174 | -1.426 | -0.499 |
| sp\|Q86SQ4\|AGRG6_HUMAN Adhesion G-protein coupled receptor G6 OS=Homo sapiens GN=ADGRG6 PE=1 S | 1003 | EVYGKEsYGKE | 0.044 | -0.223 | -1.411 | -0.53 |
| sp\|Q86SQ4\|AGRG6_HUMAN Adhesion G-protein coupled receptor G6 OS=Homo sapiens GN=ADGRG6 PE=1 S | 1024 | PVIFYVtCAGY | 0.397 | 0.084 | -0.527 | -0.015 |
| sp\|Q86SQ4\|AGRG6_HUMAN Adhesion G-protein coupled receptor G6 OS=Homo sapiens GN=ADGRG6 PE=1 S | 1055 | GRNGKRsNRTL | 0.125 | 0.055 | -1.211 | -0.344 |
| sp\|Q86SQ4\|AGRG6_HUMAN Adhesion G-protein coupled receptor G6 OS=Homo sapiens GN=ADGRG6 PE=1 S | 1058 | GKRSNRtLREE | 0.757 | 0.282 | 0.137 | 0.392 |
| sp\|Q86SQ4\|AGRG6_HUMAN Adhesion G-protein coupled receptor G6 OS=Homo sapiens GN=ADGRG6 PE=1 S | 1069 | VLRNLRsVVSL | 0.161 | 0.214 | -0.732 | -0.119 |
| sp\|Q86SQ4\|AGRG6_HUMAN Adhesion G-protein coupled receptor G6 OS=Homo sapiens GN=ADGRG6 PE=1 S | 1072 | NLRSVVsLTFL | 0.756 | 0.312 | 0.185 | 0.418 |
| sp\|Q86SQ4\|AGRG6_HUMAN Adhesion G-protein coupled receptor G6 OS=Homo sapiens GN=ADGRG6 PE=1 S | 1074 | RSVVSLtFLLG | 0.199 | 0.157 | -0.647 | -0.097 |
| sp\|Q86SQ4\|AGRG6_HUMAN Adhesion G-protein coupled receptor G6 OS=Homo sapiens GN=ADGRG6 PE=1 S | 1080 | TFLLGMtWGFA | 0.169 | 0.058 | -0.627 | -0.133 |

| | | | | | | |
|---|---|---|---|---|---|---|
| sp\|Q86SQ4\|AGRG6_HUMAN Adhesion G-protein coupled receptor G6 OS=Homo sapiens GN=ADGRG6 PE=1 S | 1100 | PFMYLFsIFNS | 0.161 | 0.068 | -0.676 | -0.149 |
| sp\|Q86SQ4\|AGRG6_HUMAN Adhesion G-protein coupled receptor G6 OS=Homo sapiens GN=ADGRG6 PE=1 S | 1104 | LFSIFNsLQGL | 0.447 | 0.106 | -0.153 | 0.133 |
| sp\|Q86SQ4\|AGRG6_HUMAN Adhesion G-protein coupled receptor G6 OS=Homo sapiens GN=ADGRG6 PE=1 S | 1140 | FRLADNsDWSK | 0.368 | 0.237 | -0.648 | -0.014 |
| sp\|Q86SQ4\|AGRG6_HUMAN Adhesion G-protein coupled receptor G6 OS=Homo sapiens GN=ADGRG6 PE=1 S | 1143 | ADNSDWsKTAT | 0.11 | -0.322 | -1.216 | -0.476 |
| sp\|Q86SQ4\|AGRG6_HUMAN Adhesion G-protein coupled receptor G6 OS=Homo sapiens GN=ADGRG6 PE=1 S | 1145 | NSDWSKtATNI | 0.653 | 0.259 | 0.066 | 0.326 |
| sp\|Q86SQ4\|AGRG6_HUMAN Adhesion G-protein coupled receptor G6 OS=Homo sapiens GN=ADGRG6 PE=1 S | 1147 | DWSKTAtNIIK | 0.289 | -0.014 | -0.812 | -0.179 |
| sp\|Q86SQ4\|AGRG6_HUMAN Adhesion G-protein coupled receptor G6 OS=Homo sapiens GN=ADGRG6 PE=1 S | 1153 | TNIIKKsSDNL | 0.124 | -0.023 | -0.921 | -0.273 |
| sp\|Q86SQ4\|AGRG6_HUMAN Adhesion G-protein coupled receptor G6 OS=Homo sapiens GN=ADGRG6 PE=1 S | 1154 | NIIKKSsDNLG | 0.281 | 0.092 | -0.653 | -0.093 |
| sp\|Q86SQ4\|AGRG6_HUMAN Adhesion G-protein coupled receptor G6 OS=Homo sapiens GN=ADGRG6 PE=1 S | 1160 | SDNLGKsLSSS | 0.044 | -0.253 | -1.506 | -0.572 |
| sp\|Q86SQ4\|AGRG6_HUMAN Adhesion G-protein coupled receptor G6 OS=Homo sapiens GN=ADGRG6 PE=1 S | 1162 | NLGKSLsSSSI | 0.261 | 0.183 | -0.423 | 0.007 |
| sp\|Q86SQ4\|AGRG6_HUMAN Adhesion G-protein coupled receptor G6 OS=Homo sapiens GN=ADGRG6 PE=1 S | 1163 | LGKSLSsSSIG | 0.063 | -0.307 | -1.358 | -0.534 |
| sp\|Q86SQ4\|AGRG6_HUMAN Adhesion G-protein coupled receptor G6 OS=Homo sapiens GN=ADGRG6 PE=1 S | 1164 | GKSLSSsSIGS | 0.281 | 0.091 | -0.457 | -0.028 |
| sp\|Q86SQ4\|AGRG6_HUMAN Adhesion G-protein coupled receptor G6 OS=Homo sapiens GN=ADGRG6 PE=1 S | 1165 | KSLSSSsIGSN | 0.125 | -0.065 | -0.854 | -0.265 |
| sp\|Q86SQ4\|AGRG6_HUMAN Adhesion G-protein coupled receptor G6 OS=Homo sapiens GN=ADGRG6 PE=1 S | 1168 | SSSSIGsNSTY | 0.042 | -0.427 | -1.798 | -0.728 |
| sp\|Q86SQ4\|AGRG6_HUMAN Adhesion G-protein coupled receptor G6 OS=Homo sapiens GN=ADGRG6 PE=1 S | 1170 | SSIGSNsTYLT | 0.206 | 0.089 | -0.771 | -0.159 |
| sp\|Q86SQ4\|AGRG6_HUMAN Adhesion G-protein coupled receptor G6 OS=Homo sapiens GN=ADGRG6 PE=1 S | 1171 | SIGSNStYLTS | 0.03 | -0.305 | -1.747 | -0.674 |
| sp\|Q86SQ4\|AGRG6_HUMAN Adhesion G-protein coupled receptor G6 OS=Homo sapiens GN=ADGRG6 PE=1 S | 1174 | SNSTYLtSKSK | 0.031 | -0.427 | -1.768 | -0.721 |
| sp\|Q86SQ4\|AGRG6_HUMAN Adhesion G-protein coupled receptor G6 OS=Homo sapiens GN=ADGRG6 PE=1 S | 1175 | NSTYLTsKSKS | 0.073 | -0.223 | -1.165 | -0.438 |
| sp\|Q86SQ4\|AGRG6_HUMAN Adhesion G-protein coupled receptor G6 OS=Homo sapiens GN=ADGRG6 PE=1 S | 1177 | TYLTSKsKSSS | 0.218 | 0.095 | -0.475 | -0.054 |
| sp\|Q86SQ4\|AGRG6_HUMAN Adhesion G-protein coupled receptor G6 OS=Homo sapiens GN=ADGRG6 PE=1 S | 1179 | LTSKSKsSSTT | 0.362 | 0.064 | -0.263 | 0.054 |
| sp\|Q86SQ4\|AGRG6_HUMAN Adhesion G-protein coupled receptor G6 OS=Homo sapiens GN=ADGRG6 PE=1 S | 1180 | TSKSKSsSTTY | 0.092 | -0.32 | -1.341 | -0.523 |
| sp\|Q86SQ4\|AGRG6_HUMAN Adhesion G-protein coupled receptor G6 OS=Homo sapiens GN=ADGRG6 PE=1 S | 1181 | SKSKSSsTTYF | 0.366 | 0.068 | -0.541 | -0.036 |
| sp\|Q86SQ4\|AGRG6_HUMAN Adhesion G-protein coupled receptor G6 OS=Homo sapiens GN=ADGRG6 PE=1 S | 1182 | KSKSSStTYFK | 0.137 | -0.138 | -0.917 | -0.306 |
| sp\|Q86SQ4\|AGRG6_HUMAN Adhesion G-protein coupled receptor G6 OS=Homo sapiens GN=ADGRG6 PE=1 S | 1183 | SKSSSTtYFKR | 0.266 | 0.059 | -0.476 | -0.05 |
| sp\|Q86SQ4\|AGRG6_HUMAN Adhesion G-protein coupled receptor G6 OS=Homo sapiens GN=ADGRG6 PE=1 S | 1189 | TYFKRNsHTDN | 0.805 | 0.339 | 0.078 | 0.407 |
| sp\|Q86SQ4\|AGRG6_HUMAN Adhesion G-protein coupled receptor G6 OS=Homo sapiens GN=ADGRG6 PE=1 S | 1191 | FKRNSHtDNVS | 0.83 | 0.788 | 0.74 | 0.786 |
| sp\|Q86SQ4\|AGRG6_HUMAN Adhesion G-protein coupled receptor G6 OS=Homo sapiens GN=ADGRG6 PE=1 S | 1195 | SHTDNVsYEHS | 0.091 | -0.229 | -1.391 | -0.51 |
| sp\|Q86SQ4\|AGRG6_HUMAN Adhesion G-protein coupled receptor G6 OS=Homo sapiens GN=ADGRG6 PE=1 S | 1199 | NVSYEHsFNKS | 0.266 | 0.117 | -0.525 | -0.047 |
| sp\|Q86SQ4\|AGRG6_HUMAN Adhesion G-protein coupled receptor G6 OS=Homo sapiens GN=ADGRG6 PE=1 S | 1203 | EHSFNKsGSLR | 0.142 | -0.096 | -0.89 | -0.281 |
| sp\|Q86SQ4\|AGRG6_HUMAN Adhesion G-protein coupled receptor G6 OS=Homo sapiens GN=ADGRG6 PE=1 S | 1205 | SFNKSGsLRQC | 0.453 | 0.053 | -0.358 | 0.049 |
| sp\|Q86SQ4\|AGRG6_HUMAN Adhesion G-protein coupled receptor G6 OS=Homo sapiens GN=ADGRG6 PE=1 S | 1218 | GQVLVKtGPC- | 0.59 | 0.566 | 0.088 | 0.415 |
| sp\|Q96K78\|AGRG7_HUMAN Adhesion G-protein coupled receptor G7 OS=Homo sapiens GN=ADGRG7 PE=1 S | 318 | NMTKNYtKTCG | 0.409 | 0.25 | -0.134 | 0.175 |
| sp\|Q96K78\|AGRG7_HUMAN Adhesion G-protein coupled receptor G7 OS=Homo sapiens GN=ADGRG7 PE=1 S | 320 | TKNYTKtCGFV | 0.337 | 0.181 | -0.51 | 0.003 |
| sp\|Q96K78\|AGRG7_HUMAN Adhesion G-protein coupled receptor G7 OS=Homo sapiens GN=ADGRG7 PE=1 S | 334 | NDKLFQsKTFT | 0.114 | -0.244 | -1.116 | -0.415 |
| sp\|Q96K78\|AGRG7_HUMAN Adhesion G-protein coupled receptor G7 OS=Homo sapiens GN=ADGRG7 PE=1 S | 336 | KLFQSKtFTAK | 0.819 | 0.422 | 0.755 | 0.665 |
| sp\|Q96K78\|AGRG7_HUMAN Adhesion G-protein coupled receptor G7 OS=Homo sapiens GN=ADGRG7 PE=1 S | 338 | FQSKTFtAKSD | 0.181 | 0.125 | -0.574 | -0.089 |
| sp\|Q96K78\|AGRG7_HUMAN Adhesion G-protein coupled receptor G7 OS=Homo sapiens GN=ADGRG7 PE=1 S | 341 | KTFTAKsDFSQ | 0.296 | -0.006 | -0.788 | -0.166 |
| sp\|Q96K78\|AGRG7_HUMAN Adhesion G-protein coupled receptor G7 OS=Homo sapiens GN=ADGRG7 PE=1 S | 344 | TAKSDFsQKII | 0.197 | -0.156 | -0.858 | -0.272 |
| sp\|Q96K78\|AGRG7_HUMAN Adhesion G-protein coupled receptor G7 OS=Homo sapiens GN=ADGRG7 PE=1 S | 349 | FSQKIIsSKTD | 0.372 | 0.034 | -0.295 | 0.037 |
| sp\|Q96K78\|AGRG7_HUMAN Adhesion G-protein coupled receptor G7 OS=Homo sapiens GN=ADGRG7 PE=1 S | 350 | SQKIISsKTDE | 0.148 | -0.13 | -0.762 | -0.248 |
| sp\|Q96K78\|AGRG7_HUMAN Adhesion G-protein coupled receptor G7 OS=Homo sapiens GN=ADGRG7 PE=1 S | 352 | KIISSKtDENE | 0.417 | 0.286 | -0.417 | 0.095 |
| sp\|Q96K78\|AGRG7_HUMAN Adhesion G-protein coupled receptor G7 OS=Homo sapiens GN=ADGRG7 PE=1 S | 360 | ENEQDQsASVD | 0.123 | -0.142 | -0.88 | -0.3 |
| sp\|Q96K78\|AGRG7_HUMAN Adhesion G-protein coupled receptor G7 OS=Homo sapiens GN=ADGRG7 PE=1 S | 362 | EQDQSAsVDMV | 0.361 | 0.158 | -0.339 | 0.06 |
| sp\|Q96K78\|AGRG7_HUMAN Adhesion G-protein coupled receptor G7 OS=Homo sapiens GN=ADGRG7 PE=1 S | 368 | SVDMVFsPKYN | 0.045 | -0.462 | -1.71 | -0.709 |
| sp\|Q96K78\|AGRG7_HUMAN Adhesion G-protein coupled receptor G7 OS=Homo sapiens GN=ADGRG7 PE=1 S | 380 | KEFQLYsYACV | 0.094 | -0.096 | -1.36 | -0.454 |
| sp\|Q96K78\|AGRG7_HUMAN Adhesion G-protein coupled receptor G7 OS=Homo sapiens GN=ADGRG7 PE=1 S | 389 | CVYWNLsAKDW | 0.201 | 0.024 | -0.532 | -0.102 |
| sp\|Q96K78\|AGRG7_HUMAN Adhesion G-protein coupled receptor G7 OS=Homo sapiens GN=ADGRG7 PE=1 S | 395 | SAKDWDtYGCQ | 0.156 | -0.138 | -1.239 | -0.407 |
| sp\|Q96K78\|AGRG7_HUMAN Adhesion G-protein coupled receptor G7 OS=Homo sapiens GN=ADGRG7 PE=1 S | 404 | CQKDKGtDGFL | 0.167 | -0.053 | -0.839 | -0.242 |
| sp\|Q96K78\|AGRG7_HUMAN Adhesion G-protein coupled receptor G7 OS=Homo sapiens GN=ADGRG7 PE=1 S | 415 | RCRCNHtTNFA | 0.678 | 0.342 | -0.088 | 0.311 |

| | | | | | | |
|---|---|---|---|---|---|---|
| sp\|Q96K78\|AGRG7_HUMAN Adhesion G-protein coupled receptor G7 OS=Homo sapiens GN=ADGRG7 PE=1 S | 416 | CRCNHTtNFAV | 0.428 | 0.324 | -0.343 | 0.136 |
| sp\|Q96K78\|AGRG7_HUMAN Adhesion G-protein coupled receptor G7 OS=Homo sapiens GN=ADGRG7 PE=1 S | 423 | NFAVLMtFKKD | 0.235 | 0.106 | -0.391 | -0.017 |
| sp\|Q96K78\|AGRG7_HUMAN Adhesion G-protein coupled receptor G7 OS=Homo sapiens GN=ADGRG7 PE=1 S | 433 | DYQYPKsLDIL | 0.69 | 0.25 | 0.357 | 0.432 |
| sp\|Q96K78\|AGRG7_HUMAN Adhesion G-protein coupled receptor G7 OS=Homo sapiens GN=ADGRG7 PE=1 S | 438 | KSLDILsNVGC | 0.058 | -0.333 | -1.531 | -0.602 |
| sp\|Q96K78\|AGRG7_HUMAN Adhesion G-protein coupled receptor G7 OS=Homo sapiens GN=ADGRG7 PE=1 S | 445 | NVGCALsVTGL | 0.198 | -0.028 | -0.783 | -0.204 |
| sp\|Q96K78\|AGRG7_HUMAN Adhesion G-protein coupled receptor G7 OS=Homo sapiens GN=ADGRG7 PE=1 S | 447 | GCALSVtGLAL | 0.568 | 0.315 | -0.001 | 0.294 |
| sp\|Q96K78\|AGRG7_HUMAN Adhesion G-protein coupled receptor G7 OS=Homo sapiens GN=ADGRG7 PE=1 S | 452 | VTGLALtVIFQ | 0.073 | -0.199 | -1.349 | -0.492 |
| sp\|Q96K78\|AGRG7_HUMAN Adhesion G-protein coupled receptor G7 OS=Homo sapiens GN=ADGRG7 PE=1 S | 459 | VIFQIVtRKVR | 0.309 | -0.031 | -0.401 | -0.041 |
| sp\|Q96K78\|AGRG7_HUMAN Adhesion G-protein coupled receptor G7 OS=Homo sapiens GN=ADGRG7 PE=1 S | 465 | TRKVRKtSVTW | 0.318 | 0.332 | -0.311 | 0.113 |
| sp\|Q96K78\|AGRG7_HUMAN Adhesion G-protein coupled receptor G7 OS=Homo sapiens GN=ADGRG7 PE=1 S | 466 | RKVRKTsVTWV | 0.751 | 0.931 | 0.369 | 0.684 |
| sp\|Q96K78\|AGRG7_HUMAN Adhesion G-protein coupled receptor G7 OS=Homo sapiens GN=ADGRG7 PE=1 S | 468 | VRKTSVtWVLV | 0.59 | 0.499 | -0.088 | 0.334 |
| sp\|Q96K78\|AGRG7_HUMAN Adhesion G-protein coupled receptor G7 OS=Homo sapiens GN=ADGRG7 PE=1 S | 477 | LVNLCIsMLIF | 0.15 | 0.017 | -1.041 | -0.291 |
| sp\|Q96K78\|AGRG7_HUMAN Adhesion G-protein coupled receptor G7 OS=Homo sapiens GN=ADGRG7 PE=1 S | 492 | VFGIENsNKNL | 0.141 | -0.027 | -1.228 | -0.371 |
| sp\|Q96K78\|AGRG7_HUMAN Adhesion G-protein coupled receptor G7 OS=Homo sapiens GN=ADGRG7 PE=1 S | 498 | SNKNLQtSDGD | 0.105 | -0.204 | -0.845 | -0.315 |
| sp\|Q96K78\|AGRG7_HUMAN Adhesion G-protein coupled receptor G7 OS=Homo sapiens GN=ADGRG7 PE=1 S | 499 | NKNLQTsDGDI | 0.572 | 0.223 | -0.212 | 0.194 |
| sp\|Q96K78\|AGRG7_HUMAN Adhesion G-protein coupled receptor G7 OS=Homo sapiens GN=ADGRG7 PE=1 S | 516 | NNDIPRtDTIN | 0.125 | -0.201 | -1.316 | -0.464 |
| sp\|Q96K78\|AGRG7_HUMAN Adhesion G-protein coupled receptor G7 OS=Homo sapiens GN=ADGRG7 PE=1 S | 518 | DIPRTDtINIP | 0.415 | 0.714 | -0.185 | 0.315 |
| sp\|Q96K78\|AGRG7_HUMAN Adhesion G-protein coupled receptor G7 OS=Homo sapiens GN=ADGRG7 PE=1 S | 527 | IPNPMCtAIAA | 0.246 | -0.107 | -0.767 | -0.209 |
| sp\|Q96K78\|AGRG7_HUMAN Adhesion G-protein coupled receptor G7 OS=Homo sapiens GN=ADGRG7 PE=1 S | 540 | HYFLLVtFTWN | 0.452 | 0.148 | -0.371 | 0.076 |
| sp\|Q96K78\|AGRG7_HUMAN Adhesion G-protein coupled receptor G7 OS=Homo sapiens GN=ADGRG7 PE=1 S | 542 | FLLVTFtWNAL | 0.31 | 0.416 | -0.056 | 0.223 |
| sp\|Q96K78\|AGRG7_HUMAN Adhesion G-protein coupled receptor G7 OS=Homo sapiens GN=ADGRG7 PE=1 S | 547 | FTWNALsAAQL | 0.165 | 0.161 | -0.749 | -0.141 |
| sp\|Q96K78\|AGRG7_HUMAN Adhesion G-protein coupled receptor G7 OS=Homo sapiens GN=ADGRG7 PE=1 S | 558 | YYLLIRtMKPL | 0.135 | 0.069 | -0.845 | -0.214 |
| sp\|Q96K78\|AGRG7_HUMAN Adhesion G-protein coupled receptor G7 OS=Homo sapiens GN=ADGRG7 PE=1 S | 571 | HFILFIsLIGW | 0.303 | 0.118 | -0.252 | 0.056 |
| sp\|Q96K78\|AGRG7_HUMAN Adhesion G-protein coupled receptor G7 OS=Homo sapiens GN=ADGRG7 PE=1 S | 585 | AIVVAItVGVI | 0.42 | 0.202 | -0.275 | 0.116 |
| sp\|Q96K78\|AGRG7_HUMAN Adhesion G-protein coupled receptor G7 OS=Homo sapiens GN=ADGRG7 PE=1 S | 591 | TVGVIYsQNGN | 0.076 | -0.083 | -0.96 | -0.322 |
| sp\|Q96K78\|AGRG7_HUMAN Adhesion G-protein coupled receptor G7 OS=Homo sapiens GN=ADGRG7 PE=1 S | 622 | PNGVIKsPLLW | 0.035 | -0.377 | -1.32 | -0.554 |
| sp\|Q96K78\|AGRG7_HUMAN Adhesion G-protein coupled receptor G7 OS=Homo sapiens GN=ADGRG7 PE=1 S | 627 | KSPLLWsFIVP | 0.067 | -0.242 | -1.339 | -0.505 |
| sp\|Q96K78\|AGRG7_HUMAN Adhesion G-protein coupled receptor G7 OS=Homo sapiens GN=ADGRG7 PE=1 S | 633 | SFIVPVtIILI | 0.123 | -0.118 | -0.708 | -0.234 |
| sp\|Q96K78\|AGRG7_HUMAN Adhesion G-protein coupled receptor G7 OS=Homo sapiens GN=ADGRG7 PE=1 S | 638 | VTIILIsNVVM | 0.101 | -0.138 | -1.378 | -0.472 |
| sp\|Q96K78\|AGRG7_HUMAN Adhesion G-protein coupled receptor G7 OS=Homo sapiens GN=ADGRG7 PE=1 S | 645 | NVVMFItISIK | 0.104 | -0.259 | -1.171 | -0.442 |
| sp\|Q96K78\|AGRG7_HUMAN Adhesion G-protein coupled receptor G7 OS=Homo sapiens GN=ADGRG7 PE=1 S | 647 | VMFITIsIKVL | 0.08 | 0.093 | -0.961 | -0.263 |
| sp\|Q96K78\|AGRG7_HUMAN Adhesion G-protein coupled receptor G7 OS=Homo sapiens GN=ADGRG7 PE=1 S | 659 | KNNQNLtSTKK | 0.081 | -0.346 | -1.301 | -0.522 |
| sp\|Q96K78\|AGRG7_HUMAN Adhesion G-protein coupled receptor G7 OS=Homo sapiens GN=ADGRG7 PE=1 S | 660 | NNQNLTsTKKV | 0.068 | -0.184 | -1.3 | -0.472 |
| sp\|Q96K78\|AGRG7_HUMAN Adhesion G-protein coupled receptor G7 OS=Homo sapiens GN=ADGRG7 PE=1 S | 661 | NQNLTStKKVS | 0.049 | -0.135 | -1.318 | -0.468 |
| sp\|Q96K78\|AGRG7_HUMAN Adhesion G-protein coupled receptor G7 OS=Homo sapiens GN=ADGRG7 PE=1 S | 665 | TSTKKVsSMKK | 0.212 | -0.22 | -0.88 | -0.296 |
| sp\|Q96K78\|AGRG7_HUMAN Adhesion G-protein coupled receptor G7 OS=Homo sapiens GN=ADGRG7 PE=1 S | 666 | STKKVSsMKKI | 0.235 | 0.015 | -0.848 | -0.199 |
| sp\|Q96K78\|AGRG7_HUMAN Adhesion G-protein coupled receptor G7 OS=Homo sapiens GN=ADGRG7 PE=1 S | 672 | SMKKIVsTLSV | 0.17 | 0.019 | -1.015 | -0.275 |
| sp\|Q96K78\|AGRG7_HUMAN Adhesion G-protein coupled receptor G7 OS=Homo sapiens GN=ADGRG7 PE=1 S | 673 | MKKIVStLSVA | 0.433 | 0.143 | -0.22 | 0.119 |
| sp\|Q96K78\|AGRG7_HUMAN Adhesion G-protein coupled receptor G7 OS=Homo sapiens GN=ADGRG7 PE=1 S | 675 | KIVSTLsVAVV | 0.063 | -0.08 | -1.508 | -0.508 |
| sp\|Q96K78\|AGRG7_HUMAN Adhesion G-protein coupled receptor G7 OS=Homo sapiens GN=ADGRG7 PE=1 S | 683 | AVVFGItWILA | 0.098 | -0.042 | -0.958 | -0.301 |
| sp\|Q96K78\|AGRG7_HUMAN Adhesion G-protein coupled receptor G7 OS=Homo sapiens GN=ADGRG7 PE=1 S | 696 | MLVNDDsIRIV | 0.238 | -0.089 | -0.896 | -0.249 |
| sp\|Q96K78\|AGRG7_HUMAN Adhesion G-protein coupled receptor G7 OS=Homo sapiens GN=ADGRG7 PE=1 S | 702 | SIRIVFsYIFC | 0.13 | 0.083 | -1.075 | -0.287 |
| sp\|Q96K78\|AGRG7_HUMAN Adhesion G-protein coupled receptor G7 OS=Homo sapiens GN=ADGRG7 PE=1 S | 710 | IFCLFNtTQGL | 0.402 | 0.017 | -0.505 | -0.029 |
| sp\|Q96K78\|AGRG7_HUMAN Adhesion G-protein coupled receptor G7 OS=Homo sapiens GN=ADGRG7 PE=1 S | 711 | FCLFNTtQGLQ | 0.376 | 0.083 | -0.635 | -0.059 |
| sp\|Q96K78\|AGRG7_HUMAN Adhesion G-protein coupled receptor G7 OS=Homo sapiens GN=ADGRG7 PE=1 S | 721 | QIFILYtVRTK | 0.15 | -0.033 | -0.778 | -0.22 |
| sp\|Q96K78\|AGRG7_HUMAN Adhesion G-protein coupled receptor G7 OS=Homo sapiens GN=ADGRG7 PE=1 S | 724 | ILYTVRtKVFQ | 0.136 | -0.123 | -1.123 | -0.37 |
| sp\|Q96K78\|AGRG7_HUMAN Adhesion G-protein coupled receptor G7 OS=Homo sapiens GN=ADGRG7 PE=1 S | 729 | RTKVFQsEASK | 0.128 | -0.09 | -1.082 | -0.348 |
| sp\|Q96K78\|AGRG7_HUMAN Adhesion G-protein coupled receptor G7 OS=Homo sapiens GN=ADGRG7 PE=1 S | 732 | VFQSEAsKVLM | 0.212 | -0.06 | -0.782 | -0.21 |
| sp\|Q96K78\|AGRG7_HUMAN Adhesion G-protein coupled receptor G7 OS=Homo sapiens GN=ADGRG7 PE=1 S | 739 | KVLMLLsSIGR | 0.054 | -0.282 | -1.23 | -0.486 |

| | | | | | | |
|---|---|---|---|---|---|---|
| sp\|Q96K78\|AGRG7_HUMAN Adhesion G-protein coupled receptor G7 OS=Homo sapiens GN=ADGRG7 PE=1 S | 740 | VLMLLSsIGRR | 0.141 | -0.114 | -1.013 | -0.329 |
| sp\|Q96K78\|AGRG7_HUMAN Adhesion G-protein coupled receptor G7 OS=Homo sapiens GN=ADGRG7 PE=1 S | 746 | SIGRRKsLPSV | 0.925 | 1.609 | 1.376 | 1.303 |
| sp\|Q96K78\|AGRG7_HUMAN Adhesion G-protein coupled receptor G7 OS=Homo sapiens GN=ADGRG7 PE=1 S | 749 | RRKSLPsVTRP | 0.187 | -0.041 | -1.063 | -0.306 |
| sp\|Q96K78\|AGRG7_HUMAN Adhesion G-protein coupled receptor G7 OS=Homo sapiens GN=ADGRG7 PE=1 S | 751 | KSLPSVtRPRL | 0.721 | 0.422 | 0.199 | 0.447 |
| sp\|Q96K78\|AGRG7_HUMAN Adhesion G-protein coupled receptor G7 OS=Homo sapiens GN=ADGRG7 PE=1 S | 765 | MYNFLRsLPTL | 0.729 | 0.657 | 0.172 | 0.519 |
| sp\|Q96K78\|AGRG7_HUMAN Adhesion G-protein coupled receptor G7 OS=Homo sapiens GN=ADGRG7 PE=1 S | 768 | FLRSLPtLHER | 0.69 | 0.373 | 0.12 | 0.394 |
| sp\|Q96K78\|AGRG7_HUMAN Adhesion G-protein coupled receptor G7 OS=Homo sapiens GN=ADGRG7 PE=1 S | 778 | RFRLLEtSPST | 0.478 | 0.545 | -0.279 | 0.248 |
| sp\|Q96K78\|AGRG7_HUMAN Adhesion G-protein coupled receptor G7 OS=Homo sapiens GN=ADGRG7 PE=1 S | 779 | FRLLETsPSTE | 0.08 | -0.194 | -1.367 | -0.494 |
| sp\|Q96K78\|AGRG7_HUMAN Adhesion G-protein coupled receptor G7 OS=Homo sapiens GN=ADGRG7 PE=1 S | 781 | LLETSPsTEEI | 0.535 | 0.225 | 0.03 | 0.263 |
| sp\|Q96K78\|AGRG7_HUMAN Adhesion G-protein coupled receptor G7 OS=Homo sapiens GN=ADGRG7 PE=1 S | 782 | LETSPStEEIT | 0.07 | -0.262 | -1.188 | -0.46 |
| sp\|Q96K78\|AGRG7_HUMAN Adhesion G-protein coupled receptor G7 OS=Homo sapiens GN=ADGRG7 PE=1 S | 786 | PSTEEItLSES | 0.187 | -0.107 | -0.882 | -0.267 |
| sp\|Q96K78\|AGRG7_HUMAN Adhesion G-protein coupled receptor G7 OS=Homo sapiens GN=ADGRG7 PE=1 S | 788 | TEEITLsESDN | 0.094 | -0.015 | -1.17 | -0.364 |
| sp\|Q96K78\|AGRG7_HUMAN Adhesion G-protein coupled receptor G7 OS=Homo sapiens GN=ADGRG7 PE=1 S | 790 | EITLSEsDNAK | 0.477 | 0.296 | -0.096 | 0.226 |
| sp\|Q96K78\|AGRG7_HUMAN Adhesion G-protein coupled receptor G7 OS=Homo sapiens GN=ADGRG7 PE=1 S | 796 | SDNAKEsI--- | 0.036 | -0.237 | -1.794 | -0.665 |
| sp\|Q6QNK2\|AGRD1_HUMAN Adhesion G-protein coupled receptor D1 OS=Homo sapiens GN=ADGRD1 PE=1 | 316 | KSLSEQtALNL | 0.1 | -0.129 | -1.427 | -0.485 |
| sp\|Q6QNK2\|AGRD1_HUMAN Adhesion G-protein coupled receptor D1 OS=Homo sapiens GN=ADGRD1 PE=1 | 321 | QTALNLtKTFL | 0.122 | -0.181 | -0.981 | -0.347 |
| sp\|Q6QNK2\|AGRD1_HUMAN Adhesion G-protein coupled receptor D1 OS=Homo sapiens GN=ADGRD1 PE=1 | 323 | ALNLTKtFLKA | 0.245 | 0.097 | -0.621 | -0.093 |
| sp\|Q6QNK2\|AGRD1_HUMAN Adhesion G-protein coupled receptor D1 OS=Homo sapiens GN=ADGRD1 PE=1 | 341 | PGWIALsEDSA | 0.15 | 0.173 | -0.587 | -0.088 |
| sp\|Q6QNK2\|AGRD1_HUMAN Adhesion G-protein coupled receptor D1 OS=Homo sapiens GN=ADGRD1 PE=1 | 344 | IALSEDsAVVL | 0.141 | -0.166 | -1.284 | -0.436 |
| sp\|Q6QNK2\|AGRD1_HUMAN Adhesion G-protein coupled receptor D1 OS=Homo sapiens GN=ADGRD1 PE=1 | 349 | DSAVVLsLIDT | 0.19 | -0.083 | -0.636 | -0.176 |
| sp\|Q6QNK2\|AGRD1_HUMAN Adhesion G-protein coupled receptor D1 OS=Homo sapiens GN=ADGRD1 PE=1 | 353 | VLSLIDtIDTV | 0.21 | -0.117 | -0.904 | -0.27 |
| sp\|Q6QNK2\|AGRD1_HUMAN Adhesion G-protein coupled receptor D1 OS=Homo sapiens GN=ADGRD1 PE=1 | 356 | LIDTIDtVMGH | 0.118 | -0.247 | -1.187 | -0.439 |
| sp\|Q6QNK2\|AGRD1_HUMAN Adhesion G-protein coupled receptor D1 OS=Homo sapiens GN=ADGRD1 PE=1 | 362 | TVMGHVsSNLH | 0.167 | -0.079 | -0.884 | -0.265 |
| sp\|Q6QNK2\|AGRD1_HUMAN Adhesion G-protein coupled receptor D1 OS=Homo sapiens GN=ADGRD1 PE=1 | 363 | VMGHVSsNLHG | 0.043 | -0.22 | -1.691 | -0.623 |
| sp\|Q6QNK2\|AGRD1_HUMAN Adhesion G-protein coupled receptor D1 OS=Homo sapiens GN=ADGRD1 PE=1 | 368 | SSNLHGsTPQV | 0.33 | 0.253 | -0.906 | -0.108 |
| sp\|Q6QNK2\|AGRD1_HUMAN Adhesion G-protein coupled receptor D1 OS=Homo sapiens GN=ADGRD1 PE=1 | 369 | SNLHGStPQVT | 0.016 | -0.648 | -1.907 | -0.846 |
| sp\|Q6QNK2\|AGRD1_HUMAN Adhesion G-protein coupled receptor D1 OS=Homo sapiens GN=ADGRD1 PE=1 | 373 | GSTPQVtVEGS | 0.177 | -0.171 | -0.885 | -0.293 |
| sp\|Q6QNK2\|AGRD1_HUMAN Adhesion G-protein coupled receptor D1 OS=Homo sapiens GN=ADGRD1 PE=1 | 377 | QVTVEGsSAMA | 0.037 | -0.377 | -1.73 | -0.69 |
| sp\|Q6QNK2\|AGRD1_HUMAN Adhesion G-protein coupled receptor D1 OS=Homo sapiens GN=ADGRD1 PE=1 | 378 | VTVEGSsAMAE | 0.068 | -0.184 | -1.508 | -0.541 |
| sp\|Q6QNK2\|AGRD1_HUMAN Adhesion G-protein coupled receptor D1 OS=Homo sapiens GN=ADGRD1 PE=1 | 384 | SAMAEFsVAKI | 0.104 | -0.146 | -1.16 | -0.401 |
| sp\|Q6QNK2\|AGRD1_HUMAN Adhesion G-protein coupled receptor D1 OS=Homo sapiens GN=ADGRD1 PE=1 | 392 | AKILPKtVNSS | 0.22 | -0.019 | -0.571 | -0.123 |
| sp\|Q6QNK2\|AGRD1_HUMAN Adhesion G-protein coupled receptor D1 OS=Homo sapiens GN=ADGRD1 PE=1 | 395 | LPKTVNsSHYR | 0.265 | -0.071 | -0.913 | -0.24 |
| sp\|Q6QNK2\|AGRD1_HUMAN Adhesion G-protein coupled receptor D1 OS=Homo sapiens GN=ADGRD1 PE=1 | 396 | PKTVNSsHYRF | 0.191 | -0.103 | -0.766 | -0.226 |
| sp\|Q6QNK2\|AGRD1_HUMAN Adhesion G-protein coupled receptor D1 OS=Homo sapiens GN=ADGRD1 PE=1 | 406 | FPAHGQsFIQI | 0.223 | 0.035 | -0.256 | 0.001 |
| sp\|Q6QNK2\|AGRD1_HUMAN Adhesion G-protein coupled receptor D1 OS=Homo sapiens GN=ADGRD1 PE=1 | 421 | FHRHAWsTVVG | 0.483 | 0.313 | -0.492 | 0.101 |
| sp\|Q6QNK2\|AGRD1_HUMAN Adhesion G-protein coupled receptor D1 OS=Homo sapiens GN=ADGRD1 PE=1 | 422 | HRHAWStVVGL | 0.134 | 0.162 | -0.678 | -0.127 |
| sp\|Q6QNK2\|AGRD1_HUMAN Adhesion G-protein coupled receptor D1 OS=Homo sapiens GN=ADGRD1 PE=1 | 430 | VGLLYHsMHYY | 0.375 | 0.179 | -0.672 | -0.039 |
| sp\|Q6QNK2\|AGRD1_HUMAN Adhesion G-protein coupled receptor D1 OS=Homo sapiens GN=ADGRD1 PE=1 | 443 | NIWPAHtKIAE | 0.34 | 0.338 | -0.104 | 0.191 |
| sp\|Q6QNK2\|AGRD1_HUMAN Adhesion G-protein coupled receptor D1 OS=Homo sapiens GN=ADGRD1 PE=1 | 459 | DCLLFAtSHLI | 0.442 | 0.069 | -0.763 | -0.084 |
| sp\|Q6QNK2\|AGRD1_HUMAN Adhesion G-protein coupled receptor D1 OS=Homo sapiens GN=ADGRD1 PE=1 | 460 | CLLFATsHLIS | 0.493 | 0.153 | -0.23 | 0.139 |
| sp\|Q6QNK2\|AGRD1_HUMAN Adhesion G-protein coupled receptor D1 OS=Homo sapiens GN=ADGRD1 PE=1 | 464 | ATSHLIsLEVS | 0.198 | -0.111 | -0.937 | -0.283 |
| sp\|Q6QNK2\|AGRD1_HUMAN Adhesion G-protein coupled receptor D1 OS=Homo sapiens GN=ADGRD1 PE=1 | 468 | LISLEVsPPPT | 0.321 | 0.069 | -0.529 | -0.046 |
| sp\|Q6QNK2\|AGRD1_HUMAN Adhesion G-protein coupled receptor D1 OS=Homo sapiens GN=ADGRD1 PE=1 | 472 | EVSPPPtLSQN | 0.101 | -0.249 | -0.879 | -0.342 |
| sp\|Q6QNK2\|AGRD1_HUMAN Adhesion G-protein coupled receptor D1 OS=Homo sapiens GN=ADGRD1 PE=1 | 474 | SPPPTLsQNLS | 0.028 | -0.305 | -1.896 | -0.724 |
| sp\|Q6QNK2\|AGRD1_HUMAN Adhesion G-protein coupled receptor D1 OS=Homo sapiens GN=ADGRD1 PE=1 | 478 | TLSQNLsGSPL | 0.106 | -0.174 | -0.839 | -0.302 |
| sp\|Q6QNK2\|AGRD1_HUMAN Adhesion G-protein coupled receptor D1 OS=Homo sapiens GN=ADGRD1 PE=1 | 480 | SQNLSGsPLIT | 0.044 | -0.369 | -1.644 | -0.656 |
| sp\|Q6QNK2\|AGRD1_HUMAN Adhesion G-protein coupled receptor D1 OS=Homo sapiens GN=ADGRD1 PE=1 | 484 | SGSPLItVHLK | 0.098 | -0.248 | -1.189 | -0.446 |
| sp\|Q6QNK2\|AGRD1_HUMAN Adhesion G-protein coupled receptor D1 OS=Homo sapiens GN=ADGRD1 PE=1 | 492 | HLKHRLtRKQH | 0.235 | -0.006 | -0.585 | -0.119 |
| sp\|Q6QNK2\|AGRD1_HUMAN Adhesion G-protein coupled receptor D1 OS=Homo sapiens GN=ADGRD1 PE=1 | 497 | LTRKQHsEATN | 0.573 | 0.373 | -0.245 | 0.234 |

| | | | | | | |
|---|---|---|---|---|---|---|
| sp\|Q6QNK2\|AGRD1_HUMAN Adhesion G-protein coupled receptor D1 OS=Homo sapiens GN=ADGRD1 PE=1 | 500 | KQHSEAtNSSN | 0.034 | -0.329 | -1.924 | -0.74 |
| sp\|Q6QNK2\|AGRD1_HUMAN Adhesion G-protein coupled receptor D1 OS=Homo sapiens GN=ADGRD1 PE=1 | 502 | HSEATNsSNRV | 0.202 | -0.089 | -1.035 | -0.307 |
| sp\|Q6QNK2\|AGRD1_HUMAN Adhesion G-protein coupled receptor D1 OS=Homo sapiens GN=ADGRD1 PE=1 | 503 | SEATNSsNRVF | 0.06 | -0.313 | -1.535 | -0.596 |
| sp\|Q6QNK2\|AGRD1_HUMAN Adhesion G-protein coupled receptor D1 OS=Homo sapiens GN=ADGRD1 PE=1 | 516 | CAFLDFsSGEG | 0.294 | 0.008 | -0.632 | -0.11 |
| sp\|Q6QNK2\|AGRD1_HUMAN Adhesion G-protein coupled receptor D1 OS=Homo sapiens GN=ADGRD1 PE=1 | 517 | AFLDFSsGEGV | 0.106 | -0.237 | -1.347 | -0.493 |
| sp\|Q6QNK2\|AGRD1_HUMAN Adhesion G-protein coupled receptor D1 OS=Homo sapiens GN=ADGRD1 PE=1 | 523 | SGEGVWsNHGC | 0.108 | -0.32 | -1.4 | -0.537 |
| sp\|Q6QNK2\|AGRD1_HUMAN Adhesion G-protein coupled receptor D1 OS=Homo sapiens GN=ADGRD1 PE=1 | 530 | NHGCALtRGNL | 0.184 | 0.002 | -1.236 | -0.35 |
| sp\|Q6QNK2\|AGRD1_HUMAN Adhesion G-protein coupled receptor D1 OS=Homo sapiens GN=ADGRD1 PE=1 | 535 | LTRGNLtYSVC | 0.114 | -0.027 | -1.088 | -0.334 |
| sp\|Q6QNK2\|AGRD1_HUMAN Adhesion G-protein coupled receptor D1 OS=Homo sapiens GN=ADGRD1 PE=1 | 537 | RGNLTYsVCRC | 0.09 | -0.151 | -1.393 | -0.485 |
| sp\|Q6QNK2\|AGRD1_HUMAN Adhesion G-protein coupled receptor D1 OS=Homo sapiens GN=ADGRD1 PE=1 | 542 | YSVCRCtHLTN | 0.371 | 0.046 | -0.799 | -0.127 |
| sp\|Q6QNK2\|AGRD1_HUMAN Adhesion G-protein coupled receptor D1 OS=Homo sapiens GN=ADGRD1 PE=1 | 545 | CRCTHLtNFAI | 0.448 | 0.32 | -0.249 | 0.173 |
| sp\|Q6QNK2\|AGRD1_HUMAN Adhesion G-protein coupled receptor D1 OS=Homo sapiens GN=ADGRD1 PE=1 | 567 | GHQVALsSISY | 0.09 | 0.001 | -1.372 | -0.427 |
| sp\|Q6QNK2\|AGRD1_HUMAN Adhesion G-protein coupled receptor D1 OS=Homo sapiens GN=ADGRD1 PE=1 | 568 | HQVALSsISYV | 0.035 | -0.29 | -1.7 | -0.652 |
| sp\|Q6QNK2\|AGRD1_HUMAN Adhesion G-protein coupled receptor D1 OS=Homo sapiens GN=ADGRD1 PE=1 | 570 | VALSSIsYVGC | 0.16 | -0.134 | -0.875 | -0.283 |
| sp\|Q6QNK2\|AGRD1_HUMAN Adhesion G-protein coupled receptor D1 OS=Homo sapiens GN=ADGRD1 PE=1 | 575 | ISYVGCsLSVL | 0.144 | -0.082 | -0.92 | -0.286 |
| sp\|Q6QNK2\|AGRD1_HUMAN Adhesion G-protein coupled receptor D1 OS=Homo sapiens GN=ADGRD1 PE=1 | 577 | YVGCSLsVLCL | 0.14 | 0.198 | -0.734 | -0.132 |
| sp\|Q6QNK2\|AGRD1_HUMAN Adhesion G-protein coupled receptor D1 OS=Homo sapiens GN=ADGRD1 PE=1 | 584 | VLCLVAtLVTF | 0.451 | 0.11 | -0.391 | 0.057 |
| sp\|Q6QNK2\|AGRD1_HUMAN Adhesion G-protein coupled receptor D1 OS=Homo sapiens GN=ADGRD1 PE=1 | 587 | LVATLVtFAVL | 0.207 | -0.012 | -0.622 | -0.142 |
| sp\|Q6QNK2\|AGRD1_HUMAN Adhesion G-protein coupled receptor D1 OS=Homo sapiens GN=ADGRD1 PE=1 | 592 | VTFAVLsSVST | 0.052 | -0.304 | -1.594 | -0.615 |
| sp\|Q6QNK2\|AGRD1_HUMAN Adhesion G-protein coupled receptor D1 OS=Homo sapiens GN=ADGRD1 PE=1 | 593 | TFAVLSsVSTI | 0.065 | -0.203 | -1.104 | -0.414 |
| sp\|Q6QNK2\|AGRD1_HUMAN Adhesion G-protein coupled receptor D1 OS=Homo sapiens GN=ADGRD1 PE=1 | 595 | AVLSSVsTIRN | 0.33 | 0.025 | -0.541 | -0.062 |
| sp\|Q6QNK2\|AGRD1_HUMAN Adhesion G-protein coupled receptor D1 OS=Homo sapiens GN=ADGRD1 PE=1 | 596 | VLSSVStIRNQ | 0.109 | -0.253 | -1.279 | -0.474 |
| sp\|Q6QNK2\|AGRD1_HUMAN Adhesion G-protein coupled receptor D1 OS=Homo sapiens GN=ADGRD1 PE=1 | 609 | HIHANLsFAVL | 0.147 | 0.072 | -0.658 | -0.146 |
| sp\|Q6QNK2\|AGRD1_HUMAN Adhesion G-protein coupled receptor D1 OS=Homo sapiens GN=ADGRD1 PE=1 | 622 | QVLLLIsFRLE | 0.172 | -0.11 | -0.828 | -0.255 |
| sp\|Q6QNK2\|AGRD1_HUMAN Adhesion G-protein coupled receptor D1 OS=Homo sapiens GN=ADGRD1 PE=1 | 629 | FRLEPGtTPCQ | 0.314 | 0.559 | -0.405 | 0.156 |
| sp\|Q6QNK2\|AGRD1_HUMAN Adhesion G-protein coupled receptor D1 OS=Homo sapiens GN=ADGRD1 PE=1 | 630 | RLEPGTtPCQV | 0.082 | -0.444 | -1.397 | -0.586 |
| sp\|Q6QNK2\|AGRD1_HUMAN Adhesion G-protein coupled receptor D1 OS=Homo sapiens GN=ADGRD1 PE=1 | 645 | LHYFFLsAFAW | 0.12 | 0.036 | -1.151 | -0.332 |
| sp\|Q6QNK2\|AGRD1_HUMAN Adhesion G-protein coupled receptor D1 OS=Homo sapiens GN=ADGRD1 PE=1 | 659 | EGLHLYsMVIK | 0.119 | -0.011 | -0.867 | -0.253 |
| sp\|Q6QNK2\|AGRD1_HUMAN Adhesion G-protein coupled receptor D1 OS=Homo sapiens GN=ADGRD1 PE=1 | 667 | VIKVFGsEDSK | 0.111 | -0.066 | -1.044 | -0.333 |
| sp\|Q6QNK2\|AGRD1_HUMAN Adhesion G-protein coupled receptor D1 OS=Homo sapiens GN=ADGRD1 PE=1 | 670 | VFGSEDsKHRY | 0.161 | -0.277 | -1.336 | -0.484 |
| sp\|Q6QNK2\|AGRD1_HUMAN Adhesion G-protein coupled receptor D1 OS=Homo sapiens GN=ADGRD1 PE=1 | 690 | LLICIIsLSFA | 0.311 | 0.006 | -0.359 | -0.014 |
| sp\|Q6QNK2\|AGRD1_HUMAN Adhesion G-protein coupled receptor D1 OS=Homo sapiens GN=ADGRD1 PE=1 | 692 | ICIISLsFAMD | 0.39 | 0.204 | -0.326 | 0.089 |
| sp\|Q6QNK2\|AGRD1_HUMAN Adhesion G-protein coupled receptor D1 OS=Homo sapiens GN=ADGRD1 PE=1 | 697 | LSFAMDsYGTS | 0.126 | -0.315 | -1.117 | -0.435 |
| sp\|Q6QNK2\|AGRD1_HUMAN Adhesion G-protein coupled receptor D1 OS=Homo sapiens GN=ADGRD1 PE=1 | 700 | AMDSYGtSNNC | 0.062 | -0.174 | -1.713 | -0.608 |
| sp\|Q6QNK2\|AGRD1_HUMAN Adhesion G-protein coupled receptor D1 OS=Homo sapiens GN=ADGRD1 PE=1 | 701 | MDSYGTsNNCW | 0.102 | -0.058 | -0.986 | -0.314 |
| sp\|Q6QNK2\|AGRD1_HUMAN Adhesion G-protein coupled receptor D1 OS=Homo sapiens GN=ADGRD1 PE=1 | 707 | SNNCWLsLASG | 0.091 | -0.209 | -1.268 | -0.462 |
| sp\|Q6QNK2\|AGRD1_HUMAN Adhesion G-protein coupled receptor D1 OS=Homo sapiens GN=ADGRD1 PE=1 | 710 | CWLSLAsGAIW | 0.136 | -0.136 | -1.027 | -0.342 |
| sp\|Q6QNK2\|AGRD1_HUMAN Adhesion G-protein coupled receptor D1 OS=Homo sapiens GN=ADGRD1 PE=1 | 735 | GILIAVtRVIS | 0.411 | 0.065 | -0.577 | -0.034 |
| sp\|Q6QNK2\|AGRD1_HUMAN Adhesion G-protein coupled receptor D1 OS=Homo sapiens GN=ADGRD1 PE=1 | 739 | AVTRVIsQISA | 0.375 | 0.683 | -0.122 | 0.312 |
| sp\|Q6QNK2\|AGRD1_HUMAN Adhesion G-protein coupled receptor D1 OS=Homo sapiens GN=ADGRD1 PE=1 | 742 | RVISQIsADNY | 0.233 | 0.117 | -1.06 | -0.237 |
| sp\|Q6QNK2\|AGRD1_HUMAN Adhesion G-protein coupled receptor D1 OS=Homo sapiens GN=ADGRD1 PE=1 | 753 | KIHGDPsAFKL | 0.102 | -0.074 | -1.178 | -0.383 |
| sp\|Q6QNK2\|AGRD1_HUMAN Adhesion G-protein coupled receptor D1 OS=Homo sapiens GN=ADGRD1 PE=1 | 758 | PSAFKLtAKAV | 0.142 | -0.057 | -1.024 | -0.313 |
| sp\|Q6QNK2\|AGRD1_HUMAN Adhesion G-protein coupled receptor D1 OS=Homo sapiens GN=ADGRD1 PE=1 | 771 | LLPILGtSWVF | 0.081 | -0.108 | -1.351 | -0.459 |
| sp\|Q6QNK2\|AGRD1_HUMAN Adhesion G-protein coupled receptor D1 OS=Homo sapiens GN=ADGRD1 PE=1 | 772 | LPILGTsWVFG | 0.055 | -0.118 | -1.367 | -0.477 |
| sp\|Q6QNK2\|AGRD1_HUMAN Adhesion G-protein coupled receptor D1 OS=Homo sapiens GN=ADGRD1 PE=1 | 793 | FQYMFAtLNSL | 0.297 | 0.117 | -0.305 | 0.036 |
| sp\|Q6QNK2\|AGRD1_HUMAN Adhesion G-protein coupled receptor D1 OS=Homo sapiens GN=ADGRD1 PE=1 | 796 | MFATLNsLQGL | 0.532 | 0.168 | 0.002 | 0.234 |
| sp\|Q6QNK2\|AGRD1_HUMAN Adhesion G-protein coupled receptor D1 OS=Homo sapiens GN=ADGRD1 PE=1 | 811 | FHCLLNsEVRA | 0.231 | 0.022 | -1.272 | -0.34 |
| sp\|Q6QNK2\|AGRD1_HUMAN Adhesion G-protein coupled receptor D1 OS=Homo sapiens GN=ADGRD1 PE=1 | 821 | AAFKHKtKVWS | 0.384 | 0.054 | -0.4 | 0.013 |
| sp\|Q6QNK2\|AGRD1_HUMAN Adhesion G-protein coupled receptor D1 OS=Homo sapiens GN=ADGRD1 PE=1 | 825 | HKTKVWsLTSS | 0.568 | 0.136 | -0.051 | 0.218 |

| | | | | | | |
|---|---|---|---|---|---|---|
| sp\|Q6QNK2\|AGRD1_HUMAN Adhesion G-protein coupled receptor D1 OS=Homo sapiens GN=ADGRD1 PE=1 | 827 | TKVWSLtSSSA | 0.149 | -0.016 | -0.713 | -0.193 |
| sp\|Q6QNK2\|AGRD1_HUMAN Adhesion G-protein coupled receptor D1 OS=Homo sapiens GN=ADGRD1 PE=1 | 828 | KVWSLTsSSAR | 0.059 | -0.082 | -1.263 | -0.429 |
| sp\|Q6QNK2\|AGRD1_HUMAN Adhesion G-protein coupled receptor D1 OS=Homo sapiens GN=ADGRD1 PE=1 | 829 | VWSLTSsSART | 0.042 | -0.333 | -1.719 | -0.67 |
| sp\|Q6QNK2\|AGRD1_HUMAN Adhesion G-protein coupled receptor D1 OS=Homo sapiens GN=ADGRD1 PE=1 | 830 | WSLTSSsARTS | 0.19 | -0.076 | -0.761 | -0.216 |
| sp\|Q6QNK2\|AGRD1_HUMAN Adhesion G-protein coupled receptor D1 OS=Homo sapiens GN=ADGRD1 PE=1 | 833 | TSSSARtSNAK | 0.156 | -0.149 | -0.899 | -0.297 |
| sp\|Q6QNK2\|AGRD1_HUMAN Adhesion G-protein coupled receptor D1 OS=Homo sapiens GN=ADGRD1 PE=1 | 834 | SSSARTsNAKP | 0.083 | -0.26 | -1.456 | -0.544 |
| sp\|Q6QNK2\|AGRD1_HUMAN Adhesion G-protein coupled receptor D1 OS=Homo sapiens GN=ADGRD1 PE=1 | 841 | NAKPFHsDLMN | 0.23 | -0.024 | -1.038 | -0.277 |
| sp\|Q6QNK2\|AGRD1_HUMAN Adhesion G-protein coupled receptor D1 OS=Homo sapiens GN=ADGRD1 PE=1 | 847 | SDLMNGtRPGM | 0.216 | 0.036 | -0.659 | -0.136 |
| sp\|Q6QNK2\|AGRD1_HUMAN Adhesion G-protein coupled receptor D1 OS=Homo sapiens GN=ADGRD1 PE=1 | 853 | TRPGMAsTKLS | 0.113 | 0.02 | -0.99 | -0.286 |
| sp\|Q6QNK2\|AGRD1_HUMAN Adhesion G-protein coupled receptor D1 OS=Homo sapiens GN=ADGRD1 PE=1 | 854 | RPGMAStKLSP | 0.054 | -0.247 | -1.506 | -0.566 |
| sp\|Q6QNK2\|AGRD1_HUMAN Adhesion G-protein coupled receptor D1 OS=Homo sapiens GN=ADGRD1 PE=1 | 857 | MASTKLsPWDK | 0.109 | -0.38 | -1.341 | -0.537 |
| sp\|Q6QNK2\|AGRD1_HUMAN Adhesion G-protein coupled receptor D1 OS=Homo sapiens GN=ADGRD1 PE=1 | 862 | LSPWDKsSHSA | 0.107 | -0.258 | -0.94 | -0.364 |
| sp\|Q6QNK2\|AGRD1_HUMAN Adhesion G-protein coupled receptor D1 OS=Homo sapiens GN=ADGRD1 PE=1 | 863 | SPWDKSsHSAH | 0.03 | -0.241 | -1.875 | -0.695 |
| sp\|Q6QNK2\|AGRD1_HUMAN Adhesion G-protein coupled receptor D1 OS=Homo sapiens GN=ADGRD1 PE=1 | 865 | WDKSSHsAHRV | 0.435 | 0.155 | -0.597 | -0.002 |
| sp\|Q6QNK2\|AGRD1_HUMAN Adhesion G-protein coupled receptor D1 OS=Homo sapiens GN=ADGRD1 PE=1 | 872 | AHRVDLsAV-- | 0.131 | 0.22 | -1.333 | -0.327 |
| sp\|Q7Z7M1\|AGRD2_HUMAN Adhesion G-protein coupled receptor D2 OS=Homo sapiens GN=ADGRD2 PE=2 | 318 | TPSLLPtVWVR | 0.129 | -0.147 | -1.189 | -0.402 |
| sp\|Q7Z7M1\|AGRD2_HUMAN Adhesion G-protein coupled receptor D2 OS=Homo sapiens GN=ADGRD2 PE=2 | 329 | LLCPVPsEECP | 0.178 | -0.011 | -0.858 | -0.23 |
| sp\|Q7Z7M1\|AGRD2_HUMAN Adhesion G-protein coupled receptor D2 OS=Homo sapiens GN=ADGRD2 PE=2 | 334 | PSEECPtWNPG | 0.188 | -0.087 | -0.696 | -0.198 |
| sp\|Q7Z7M1\|AGRD2_HUMAN Adhesion G-protein coupled receptor D2 OS=Homo sapiens GN=ADGRD2 PE=2 | 341 | WNPGPRsEGSE | 0.047 | -0.278 | -1.279 | -0.503 |
| sp\|Q7Z7M1\|AGRD2_HUMAN Adhesion G-protein coupled receptor D2 OS=Homo sapiens GN=ADGRD2 PE=2 | 344 | GPRSEGsELCL | 0.228 | 0.17 | -0.994 | -0.199 |
| sp\|Q7Z7M1\|AGRD2_HUMAN Adhesion G-protein coupled receptor D2 OS=Homo sapiens GN=ADGRD2 PE=2 | 359 | FLCCYRtEPYR | 0.779 | 0.696 | 0.204 | 0.56 |
| sp\|Q7Z7M1\|AGRD2_HUMAN Adhesion G-protein coupled receptor D2 OS=Homo sapiens GN=ADGRD2 PE=2 | 370 | RLQDAQsWPGQ | 0.801 | 0.751 | 0.453 | 0.668 |
| sp\|Q7Z7M1\|AGRD2_HUMAN Adhesion G-protein coupled receptor D2 OS=Homo sapiens GN=ADGRD2 PE=2 | 378 | PGQDVIsRVNA | 0.236 | -0.045 | -0.959 | -0.256 |
| sp\|Q7Z7M1\|AGRD2_HUMAN Adhesion G-protein coupled receptor D2 OS=Homo sapiens GN=ADGRD2 PE=2 | 395 | LLPDPLsEVHG | 0.087 | -0.105 | -0.971 | -0.33 |
| sp\|Q7Z7M1\|AGRD2_HUMAN Adhesion G-protein coupled receptor D2 OS=Homo sapiens GN=ADGRD2 PE=2 | 402 | EVHGALsPAEA | 0.034 | -0.413 | -1.497 | -0.625 |
| sp\|Q7Z7M1\|AGRD2_HUMAN Adhesion G-protein coupled receptor D2 OS=Homo sapiens GN=ADGRD2 PE=2 | 407 | LSPAEAsSFLG | 0.123 | -0.213 | -1.161 | -0.417 |
| sp\|Q7Z7M1\|AGRD2_HUMAN Adhesion G-protein coupled receptor D2 OS=Homo sapiens GN=ADGRD2 PE=2 | 408 | SPAEASsFLGL | 0.088 | -0.113 | -1.105 | -0.377 |
| sp\|Q7Z7M1\|AGRD2_HUMAN Adhesion G-protein coupled receptor D2 OS=Homo sapiens GN=ADGRD2 PE=2 | 452 | DPELLLtGPWE | 0.272 | 0.225 | -0.679 | -0.061 |
| sp\|Q7Z7M1\|AGRD2_HUMAN Adhesion G-protein coupled receptor D2 OS=Homo sapiens GN=ADGRD2 PE=2 | 459 | GPWEQLsQGVV | 0.076 | 0.02 | -1.699 | -0.534 |
| sp\|Q7Z7M1\|AGRD2_HUMAN Adhesion G-protein coupled receptor D2 OS=Homo sapiens GN=ADGRD2 PE=2 | 464 | LSQGVVsVASL | 0.151 | -0.097 | -0.85 | -0.265 |
| sp\|Q7Z7M1\|AGRD2_HUMAN Adhesion G-protein coupled receptor D2 OS=Homo sapiens GN=ADGRD2 PE=2 | 467 | GVVSVAsLVLE | 0.318 | 0.055 | -0.646 | -0.091 |
| sp\|Q7Z7M1\|AGRD2_HUMAN Adhesion G-protein coupled receptor D2 OS=Homo sapiens GN=ADGRD2 PE=2 | 477 | EEQVADtWLSL | 0.061 | -0.006 | -1.419 | -0.455 |
| sp\|Q7Z7M1\|AGRD2_HUMAN Adhesion G-protein coupled receptor D2 OS=Homo sapiens GN=ADGRD2 PE=2 | 480 | VADTWLsLREV | 0.243 | -0.118 | -0.92 | -0.265 |
| sp\|Q7Z7M1\|AGRD2_HUMAN Adhesion G-protein coupled receptor D2 OS=Homo sapiens GN=ADGRD2 PE=2 | 494 | PMALVAsVQRL | 0.277 | 0.117 | -0.687 | -0.098 |
| sp\|Q7Z7M1\|AGRD2_HUMAN Adhesion G-protein coupled receptor D2 OS=Homo sapiens GN=ADGRD2 PE=2 | 503 | RLAPLLsTSMT | 0.065 | -0.241 | -1.316 | -0.497 |
| sp\|Q7Z7M1\|AGRD2_HUMAN Adhesion G-protein coupled receptor D2 OS=Homo sapiens GN=ADGRD2 PE=2 | 504 | LAPLLStSMTS | 0.031 | -0.363 | -1.859 | -0.73 |
| sp\|Q7Z7M1\|AGRD2_HUMAN Adhesion G-protein coupled receptor D2 OS=Homo sapiens GN=ADGRD2 PE=2 | 505 | APLLSTsMTSE | 0.218 | 0.109 | -0.826 | -0.166 |
| sp\|Q7Z7M1\|AGRD2_HUMAN Adhesion G-protein coupled receptor D2 OS=Homo sapiens GN=ADGRD2 PE=2 | 507 | LLSTSMtSERP | 0.337 | 0.167 | -0.471 | 0.011 |
| sp\|Q7Z7M1\|AGRD2_HUMAN Adhesion G-protein coupled receptor D2 OS=Homo sapiens GN=ADGRD2 PE=2 | 508 | LSTSMTsERPR | 0.161 | -0.244 | -0.668 | -0.25 |
| sp\|Q7Z7M1\|AGRD2_HUMAN Adhesion G-protein coupled receptor D2 OS=Homo sapiens GN=ADGRD2 PE=2 | 523 | HRHAGLsGVTV | 0.053 | -0.019 | -1.399 | -0.455 |
| sp\|Q7Z7M1\|AGRD2_HUMAN Adhesion G-protein coupled receptor D2 OS=Homo sapiens GN=ADGRD2 PE=2 | 526 | AGLSGVtVIHS | 0.087 | -0.285 | -1.002 | -0.4 |
| sp\|Q7Z7M1\|AGRD2_HUMAN Adhesion G-protein coupled receptor D2 OS=Homo sapiens GN=ADGRD2 PE=2 | 530 | GVTVIHsWFTS | 0.19 | 0.165 | -0.58 | -0.075 |
| sp\|Q7Z7M1\|AGRD2_HUMAN Adhesion G-protein coupled receptor D2 OS=Homo sapiens GN=ADGRD2 PE=2 | 533 | VIHSWFtSRVF | 0.101 | -0.103 | -1.031 | -0.344 |
| sp\|Q7Z7M1\|AGRD2_HUMAN Adhesion G-protein coupled receptor D2 OS=Homo sapiens GN=ADGRD2 PE=2 | 534 | IHSWFTsRVFQ | 0.1 | -0.34 | -1.503 | -0.581 |
| sp\|Q7Z7M1\|AGRD2_HUMAN Adhesion G-protein coupled receptor D2 OS=Homo sapiens GN=ADGRD2 PE=2 | 540 | SRVFQHtLEGP | 0.226 | 0.249 | -0.702 | -0.076 |
| sp\|Q7Z7M1\|AGRD2_HUMAN Adhesion G-protein coupled receptor D2 OS=Homo sapiens GN=ADGRD2 PE=2 | 553 | EPQAPAsSEEA | 0.142 | -0.17 | -0.986 | -0.338 |
| sp\|Q7Z7M1\|AGRD2_HUMAN Adhesion G-protein coupled receptor D2 OS=Homo sapiens GN=ADGRD2 PE=2 | 554 | PQAPASsEEAN | 0.208 | 0.079 | -0.41 | -0.041 |
| sp\|Q7Z7M1\|AGRD2_HUMAN Adhesion G-protein coupled receptor D2 OS=Homo sapiens GN=ADGRD2 PE=2 | 565 | RVQRFLsTQVG | 0.558 | 0.91 | 0.097 | 0.522 |
| sp\|Q7Z7M1\|AGRD2_HUMAN Adhesion G-protein coupled receptor D2 OS=Homo sapiens GN=ADGRD2 PE=2 | 566 | VQRFLStQVGS | 0.11 | -0.059 | -1.158 | -0.369 |

| | | | | | | |
|---|---|---|---|---|---|---|
| sp\|Q7Z7M1\|AGRD2_HUMAN Adhesion G-protein coupled receptor D2 OS=Homo sapiens GN=ADGRD2 PE=2 | 570 | LSTQVGsAIIS | 0.129 | -0.172 | -1.011 | -0.351 |
| sp\|Q7Z7M1\|AGRD2_HUMAN Adhesion G-protein coupled receptor D2 OS=Homo sapiens GN=ADGRD2 PE=2 | 574 | VGSAIIsSEVW | 0.112 | -0.167 | -0.923 | -0.326 |
| sp\|Q7Z7M1\|AGRD2_HUMAN Adhesion G-protein coupled receptor D2 OS=Homo sapiens GN=ADGRD2 PE=2 | 575 | GSAIISsEVWD | 0.175 | 0.008 | -0.542 | -0.12 |
| sp\|Q7Z7M1\|AGRD2_HUMAN Adhesion G-protein coupled receptor D2 OS=Homo sapiens GN=ADGRD2 PE=2 | 581 | SEVWDVtGEVN | 0.139 | -0.193 | -1.198 | -0.417 |
| sp\|Q7Z7M1\|AGRD2_HUMAN Adhesion G-protein coupled receptor D2 OS=Homo sapiens GN=ADGRD2 PE=2 | 589 | EVNVAMtFHLQ | 0.255 | 0.186 | -0.603 | -0.054 |
| sp\|Q7Z7M1\|AGRD2_HUMAN Adhesion G-protein coupled receptor D2 OS=Homo sapiens GN=ADGRD2 PE=2 | 598 | LQHRAQsPLFP | 0.134 | 0.405 | -0.581 | -0.014 |
| sp\|Q7Z7M1\|AGRD2_HUMAN Adhesion G-protein coupled receptor D2 OS=Homo sapiens GN=ADGRD2 PE=2 | 607 | FPPHPPsPYTG | 0.042 | -0.532 | -1.573 | -0.688 |
| sp\|Q7Z7M1\|AGRD2_HUMAN Adhesion G-protein coupled receptor D2 OS=Homo sapiens GN=ADGRD2 PE=2 | 610 | HPPSPYtGGAW | 0.096 | -0.059 | -0.71 | -0.224 |
| sp\|Q7Z7M1\|AGRD2_HUMAN Adhesion G-protein coupled receptor D2 OS=Homo sapiens GN=ADGRD2 PE=2 | 616 | TGGAWAtTGCS | 0.196 | -0.012 | -0.804 | -0.207 |
| sp\|Q7Z7M1\|AGRD2_HUMAN Adhesion G-protein coupled receptor D2 OS=Homo sapiens GN=ADGRD2 PE=2 | 617 | GGAWATtGCSV | 0.181 | -0.156 | -0.864 | -0.28 |
| sp\|Q7Z7M1\|AGRD2_HUMAN Adhesion G-protein coupled receptor D2 OS=Homo sapiens GN=ADGRD2 PE=2 | 620 | WATTGCsVAAL | 0.157 | -0.108 | -0.868 | -0.273 |
| sp\|Q7Z7M1\|AGRD2_HUMAN Adhesion G-protein coupled receptor D2 OS=Homo sapiens GN=ADGRD2 PE=2 | 628 | AALYLDsTACF | 0.066 | -0.271 | -1.498 | -0.568 |
| sp\|Q7Z7M1\|AGRD2_HUMAN Adhesion G-protein coupled receptor D2 OS=Homo sapiens GN=ADGRD2 PE=2 | 629 | ALYLDStACFC | 0.059 | -0.296 | -1.775 | -0.671 |
| sp\|Q7Z7M1\|AGRD2_HUMAN Adhesion G-protein coupled receptor D2 OS=Homo sapiens GN=ADGRD2 PE=2 | 636 | ACFCNHsTSFA | 0.24 | -0.065 | -1.027 | -0.284 |
| sp\|Q7Z7M1\|AGRD2_HUMAN Adhesion G-protein coupled receptor D2 OS=Homo sapiens GN=ADGRD2 PE=2 | 637 | CFCNHStSFAI | 0.353 | 0.114 | -0.111 | 0.119 |
| sp\|Q7Z7M1\|AGRD2_HUMAN Adhesion G-protein coupled receptor D2 OS=Homo sapiens GN=ADGRD2 PE=2 | 638 | FCNHSTsFAIL | 0.552 | 0.335 | -0.094 | 0.264 |
| sp\|Q7Z7M1\|AGRD2_HUMAN Adhesion G-protein coupled receptor D2 OS=Homo sapiens GN=ADGRD2 PE=2 | 656 | RGPEEEsLLRT | 0.088 | -0.247 | -1.486 | -0.548 |
| sp\|Q7Z7M1\|AGRD2_HUMAN Adhesion G-protein coupled receptor D2 OS=Homo sapiens GN=ADGRD2 PE=2 | 660 | EESLLRtLSFV | 0.057 | -0.211 | -1.74 | -0.631 |
| sp\|Q7Z7M1\|AGRD2_HUMAN Adhesion G-protein coupled receptor D2 OS=Homo sapiens GN=ADGRD2 PE=2 | 662 | SLLRTLsFVGC | 0.428 | 0.821 | 0.036 | 0.428 |
| sp\|Q7Z7M1\|AGRD2_HUMAN Adhesion G-protein coupled receptor D2 OS=Homo sapiens GN=ADGRD2 PE=2 | 669 | FVGCGVsFCAL | 0.257 | 0.055 | -0.56 | -0.083 |
| sp\|Q7Z7M1\|AGRD2_HUMAN Adhesion G-protein coupled receptor D2 OS=Homo sapiens GN=ADGRD2 PE=2 | 674 | VSFCALtTTFL | 0.172 | -0.161 | -1.102 | -0.364 |
| sp\|Q7Z7M1\|AGRD2_HUMAN Adhesion G-protein coupled receptor D2 OS=Homo sapiens GN=ADGRD2 PE=2 | 675 | SFCALTtTFLL | 0.137 | -0.089 | -0.964 | -0.305 |
| sp\|Q7Z7M1\|AGRD2_HUMAN Adhesion G-protein coupled receptor D2 OS=Homo sapiens GN=ADGRD2 PE=2 | 676 | FCALTTtFLLF | 0.186 | 0.163 | -0.683 | -0.111 |
| sp\|Q7Z7M1\|AGRD2_HUMAN Adhesion G-protein coupled receptor D2 OS=Homo sapiens GN=ADGRD2 PE=2 | 688 | VAGVPKsERTT | 0.086 | -0.252 | -0.933 | -0.366 |
| sp\|Q7Z7M1\|AGRD2_HUMAN Adhesion G-protein coupled receptor D2 OS=Homo sapiens GN=ADGRD2 PE=2 | 691 | VPKSERtTVHK | 0.066 | -0.323 | -1.492 | -0.583 |
| sp\|Q7Z7M1\|AGRD2_HUMAN Adhesion G-protein coupled receptor D2 OS=Homo sapiens GN=ADGRD2 PE=2 | 692 | PKSERTtVHKN | 0.421 | 0.196 | -0.39 | 0.076 |
| sp\|Q7Z7M1\|AGRD2_HUMAN Adhesion G-protein coupled receptor D2 OS=Homo sapiens GN=ADGRD2 PE=2 | 698 | TVHKNLtFSLA | 0.132 | -0.088 | -0.824 | -0.26 |
| sp\|Q7Z7M1\|AGRD2_HUMAN Adhesion G-protein coupled receptor D2 OS=Homo sapiens GN=ADGRD2 PE=2 | 700 | HKNLTFsLASA | 0.21 | 0.13 | -0.468 | -0.043 |
| sp\|Q7Z7M1\|AGRD2_HUMAN Adhesion G-protein coupled receptor D2 OS=Homo sapiens GN=ADGRD2 PE=2 | 703 | LTFSLAsAEGF | 0.124 | -0.16 | -1.228 | -0.421 |
| sp\|Q7Z7M1\|AGRD2_HUMAN Adhesion G-protein coupled receptor D2 OS=Homo sapiens GN=ADGRD2 PE=2 | 710 | AEGFLMtSEWA | 0.114 | -0.065 | -1.189 | -0.38 |
| sp\|Q7Z7M1\|AGRD2_HUMAN Adhesion G-protein coupled receptor D2 OS=Homo sapiens GN=ADGRD2 PE=2 | 711 | EGFLMTsEWAK | 0.31 | 0.041 | -0.397 | -0.015 |
| sp\|Q7Z7M1\|AGRD2_HUMAN Adhesion G-protein coupled receptor D2 OS=Homo sapiens GN=ADGRD2 PE=2 | 725 | VACVAVtVAMH | 0.153 | -0.278 | -1.284 | -0.47 |
| sp\|Q7Z7M1\|AGRD2_HUMAN Adhesion G-protein coupled receptor D2 OS=Homo sapiens GN=ADGRD2 PE=2 | 737 | LFLVAFsWMLV | 0.24 | 0.179 | -0.59 | -0.057 |
| sp\|Q7Z7M1\|AGRD2_HUMAN Adhesion G-protein coupled receptor D2 OS=Homo sapiens GN=ADGRD2 PE=2 | 754 | RKVVAVsMHPG | 0.531 | 0.215 | -0.354 | 0.131 |
| sp\|Q7Z7M1\|AGRD2_HUMAN Adhesion G-protein coupled receptor D2 OS=Homo sapiens GN=ADGRD2 PE=2 | 767 | MRLYHAtGWGV | 0.571 | 0.352 | -0.221 | 0.234 |
| sp\|Q7Z7M1\|AGRD2_HUMAN Adhesion G-protein coupled receptor D2 OS=Homo sapiens GN=ADGRD2 PE=2 | 779 | VGIVAVtLAML | 0.325 | 0.011 | -0.433 | -0.032 |
| sp\|Q7Z7M1\|AGRD2_HUMAN Adhesion G-protein coupled receptor D2 OS=Homo sapiens GN=ADGRD2 PE=2 | 799 | CWLNVHtNAIW | 0.234 | 0.065 | -0.713 | -0.138 |
| sp\|Q7Z7M1\|AGRD2_HUMAN Adhesion G-protein coupled receptor D2 OS=Homo sapiens GN=ADGRD2 PE=2 | 814 | PVLFVLtANTC | 0.147 | -0.067 | -1.05 | -0.323 |
| sp\|Q7Z7M1\|AGRD2_HUMAN Adhesion G-protein coupled receptor D2 OS=Homo sapiens GN=ADGRD2 PE=2 | 817 | FVLTANtCILA | 0.345 | 0.224 | -0.397 | 0.057 |
| sp\|Q7Z7M1\|AGRD2_HUMAN Adhesion G-protein coupled receptor D2 OS=Homo sapiens GN=ADGRD2 PE=2 | 827 | ARVVMItVSSA | 0.076 | -0.058 | -1.179 | -0.387 |
| sp\|Q7Z7M1\|AGRD2_HUMAN Adhesion G-protein coupled receptor D2 OS=Homo sapiens GN=ADGRD2 PE=2 | 829 | VVMITVsSARR | 0.103 | -0.139 | -1.154 | -0.397 |
| sp\|Q7Z7M1\|AGRD2_HUMAN Adhesion G-protein coupled receptor D2 OS=Homo sapiens GN=ADGRD2 PE=2 | 830 | VMITVSsARRR | 0.112 | -0.13 | -1.417 | -0.478 |
| sp\|Q7Z7M1\|AGRD2_HUMAN Adhesion G-protein coupled receptor D2 OS=Homo sapiens GN=ADGRD2 PE=2 | 839 | RRARMLsPQPC | 0.319 | 0.596 | -0.231 | 0.228 |
| sp\|Q7Z7M1\|AGRD2_HUMAN Adhesion G-protein coupled receptor D2 OS=Homo sapiens GN=ADGRD2 PE=2 | 850 | LQQQIWtQIWA | 0.151 | -0.099 | -0.498 | -0.149 |
| sp\|Q7Z7M1\|AGRD2_HUMAN Adhesion G-protein coupled receptor D2 OS=Homo sapiens GN=ADGRD2 PE=2 | 855 | WTQIWAtVKPV | 0.243 | -0.002 | -0.829 | -0.196 |
| sp\|Q7Z7M1\|AGRD2_HUMAN Adhesion G-protein coupled receptor D2 OS=Homo sapiens GN=ADGRD2 PE=2 | 869 | LPVLGLtWLAG | 0.054 | -0.073 | -1.326 | -0.448 |
| sp\|Q7Z7M1\|AGRD2_HUMAN Adhesion G-protein coupled receptor D2 OS=Homo sapiens GN=ADGRD2 PE=2 | 879 | GILVHLsPAWA | 0.069 | -0.293 | -1.264 | -0.496 |
| sp\|Q7Z7M1\|AGRD2_HUMAN Adhesion G-protein coupled receptor D2 OS=Homo sapiens GN=ADGRD2 PE=2 | 891 | AAVGLNsIQGL | 0.121 | -0.157 | -1.055 | -0.364 |
| sp\|Q7Z7M1\|AGRD2_HUMAN Adhesion G-protein coupled receptor D2 OS=Homo sapiens GN=ADGRD2 PE=2 | 910 | CNEEVRsALQR | 0.104 | -0.152 | -1.23 | -0.426 |

| | | | | | | |
|---|---|---|---|---|---|---|
| sp\|Q7Z7M1\|AGRD2_HUMAN Adhesion G-protein coupled receptor D2 OS=Homo sapiens GN=ADGRD2 PE=2 | 938 | GAAKEHsLPFS | 0.808 | 0.602 | 0.376 | 0.595 |
| sp\|Q7Z7M1\|AGRD2_HUMAN Adhesion G-protein coupled receptor D2 OS=Homo sapiens GN=ADGRD2 PE=2 | 942 | EHSLPFsVLPL | 0.063 | -0.165 | -1.337 | -0.48 |
| sp\|Q7Z7M1\|AGRD2_HUMAN Adhesion G-protein coupled receptor D2 OS=Homo sapiens GN=ADGRD2 PE=2 | 953 | FLPPKPsTPRH | 0.31 | 0.198 | -0.723 | -0.072 |
| sp\|Q7Z7M1\|AGRD2_HUMAN Adhesion G-protein coupled receptor D2 OS=Homo sapiens GN=ADGRD2 PE=2 | 954 | LPPKPStPRHP | 0.025 | -0.719 | -1.731 | -0.808 |
| sp\|Q9NQ84\|GPC5C_HUMAN G-protein coupled receptor family C group 5 member C OS=Homo sapiens GN=( | 319 | QGDMYPtRGVG | 0.183 | -0.202 | -0.895 | -0.305 |
| sp\|Q9NQ84\|GPC5C_HUMAN G-protein coupled receptor family C group 5 member C OS=Homo sapiens GN=( | 326 | RGVGYEtILKE | 0.076 | -0.195 | -1.342 | -0.487 |
| sp\|Q9NQ84\|GPC5C_HUMAN G-protein coupled receptor family C group 5 member C OS=Homo sapiens GN=( | 335 | KEQKGQsMFVE | 0.214 | 0.073 | -0.915 | -0.209 |
| sp\|Q9NQ84\|GPC5C_HUMAN G-protein coupled receptor family C group 5 member C OS=Homo sapiens GN=( | 344 | VENKAFsMDEP | 0.374 | 0.166 | -0.595 | -0.018 |
| sp\|Q9NQ84\|GPC5C_HUMAN G-protein coupled receptor family C group 5 member C OS=Homo sapiens GN=( | 356 | AAKRPVsPYSG | 0.366 | 0.396 | -0.19 | 0.191 |
| sp\|Q9NQ84\|GPC5C_HUMAN G-protein coupled receptor family C group 5 member C OS=Homo sapiens GN=( | 359 | RPVSPYsGYNG | 0.103 | -0.1 | -1.074 | -0.357 |
| sp\|Q9NQ84\|GPC5C_HUMAN G-protein coupled receptor family C group 5 member C OS=Homo sapiens GN=( | 367 | YNGQLLtSVYQ | 0.034 | -0.352 | -1.655 | -0.658 |
| sp\|Q9NQ84\|GPC5C_HUMAN G-protein coupled receptor family C group 5 member C OS=Homo sapiens GN=( | 368 | NGQLLTsVYQP | 0.124 | -0.107 | -0.972 | -0.318 |
| sp\|Q9NQ84\|GPC5C_HUMAN G-protein coupled receptor family C group 5 member C OS=Homo sapiens GN=( | 373 | TSVYQPtEMAL | 0.153 | 0.022 | -0.745 | -0.19 |
| sp\|Q9NQ84\|GPC5C_HUMAN G-protein coupled receptor family C group 5 member C OS=Homo sapiens GN=( | 383 | LMHKVPsEGAY | 0.397 | 0.245 | -0.333 | 0.103 |
| sp\|Q9NQ84\|GPC5C_HUMAN G-protein coupled receptor family C group 5 member C OS=Homo sapiens GN=( | 395 | IILPRAtANSQ | 0.363 | 0.143 | -0.444 | 0.021 |
| sp\|Q9NQ84\|GPC5C_HUMAN G-protein coupled receptor family C group 5 member C OS=Homo sapiens GN=( | 398 | PRATANsQVMG | 0.388 | 0.205 | -0.671 | -0.026 |
| sp\|Q9NQ84\|GPC5C_HUMAN G-protein coupled receptor family C group 5 member C OS=Homo sapiens GN=( | 403 | NSQVMGsANST | 0.062 | -0.234 | -1.346 | -0.506 |
| sp\|Q9NQ84\|GPC5C_HUMAN G-protein coupled receptor family C group 5 member C OS=Homo sapiens GN=( | 406 | VMGSANsTLRA | 0.128 | -0.072 | -1.27 | -0.405 |
| sp\|Q9NQ84\|GPC5C_HUMAN G-protein coupled receptor family C group 5 member C OS=Homo sapiens GN=( | 407 | MGSANStLRAE | 0.384 | -0.032 | -0.168 | 0.061 |
| sp\|Q9NQ84\|GPC5C_HUMAN G-protein coupled receptor family C group 5 member C OS=Homo sapiens GN=( | 415 | RAEDMYsAQSH | 0.118 | -0.142 | -1.195 | -0.406 |
| sp\|Q9NQ84\|GPC5C_HUMAN G-protein coupled receptor family C group 5 member C OS=Homo sapiens GN=( | 418 | DMYSAQsHQAA | 0.326 | 0.154 | -0.408 | 0.024 |
| sp\|Q9NQ84\|GPC5C_HUMAN G-protein coupled receptor family C group 5 member C OS=Homo sapiens GN=( | 423 | QSHQAAtPPKD | 0.406 | 0.166 | -0.269 | 0.101 |
| sp\|Q9NQ84\|GPC5C_HUMAN G-protein coupled receptor family C group 5 member C OS=Homo sapiens GN=( | 431 | PKDGKNsQVFR | 0.166 | -0.094 | -1.078 | -0.335 |
| sp\|Q9NZD1\|GPC5D_HUMAN G-protein coupled receptor family C group 5 member D OS=Homo sapiens GN=( | 316 | ALTSYGtPIQP | 0.058 | -0.475 | -1.36 | -0.592 |
| sp\|Q9NZD1\|GPC5D_HUMAN G-protein coupled receptor family C group 5 member D OS=Homo sapiens GN=( | 322 | TPIQPQtVDPT | 0.122 | -0.2 | -0.812 | -0.297 |
| sp\|Q9NZD1\|GPC5D_HUMAN G-protein coupled receptor family C group 5 member D OS=Homo sapiens GN=( | 326 | PQTVDPtQECF | 0.061 | -0.193 | -1.34 | -0.491 |
| sp\|Q9NZD1\|GPC5D_HUMAN G-protein coupled receptor family C group 5 member D OS=Homo sapiens GN=( | 337 | IPQAKLsPQQD | 0.078 | -0.434 | -1.188 | -0.515 |
| sp\|O94910\|AGRL1_HUMAN Adhesion G protein-coupled receptor L1 OS=Homo sapiens GN=ADGRL1 PE=1 SV | 316 | YDKRSAsNAFM | 0.604 | 0.864 | 0.362 | 0.61 |
| sp\|O94910\|AGRL1_HUMAN Adhesion G protein-coupled receptor L1 OS=Homo sapiens GN=ADGRL1 PE=1 SV | 330 | VLYVLRsVYVD | 0.106 | 0.01 | -1.07 | -0.318 |
| sp\|O94910\|AGRL1_HUMAN Adhesion G protein-coupled receptor L1 OS=Homo sapiens GN=ADGRL1 PE=1 SV | 337 | VYVDDDsEAAG | 0.225 | -0.044 | -0.952 | -0.257 |
| sp\|O94910\|AGRL1_HUMAN Adhesion G protein-coupled receptor L1 OS=Homo sapiens GN=ADGRL1 PE=1 SV | 350 | VDYAFNtNANR | 0.096 | -0.223 | -1.509 | -0.545 |
| sp\|O94910\|AGRL1_HUMAN Adhesion G protein-coupled receptor L1 OS=Homo sapiens GN=ADGRL1 PE=1 SV | 359 | NREEPVsLTFP | 0.247 | 0.022 | -0.57 | -0.1 |
| sp\|O94910\|AGRL1_HUMAN Adhesion G protein-coupled receptor L1 OS=Homo sapiens GN=ADGRL1 PE=1 SV | 361 | EEPVSLtFPNP | 0.347 | 0.563 | -0.303 | 0.202 |
| sp\|O94910\|AGRL1_HUMAN Adhesion G protein-coupled receptor L1 OS=Homo sapiens GN=ADGRL1 PE=1 SV | 370 | NPYQFIsSVDY | 0.269 | -0.037 | -0.765 | -0.178 |
| sp\|O94910\|AGRL1_HUMAN Adhesion G protein-coupled receptor L1 OS=Homo sapiens GN=ADGRL1 PE=1 SV | 371 | PYQFISsVDYN | 0.288 | 0.09 | -0.54 | -0.054 |
| sp\|O94910\|AGRL1_HUMAN Adhesion G protein-coupled receptor L1 OS=Homo sapiens GN=ADGRL1 PE=1 SV | 393 | YFVVRYsLEFG | 0.309 | 0.221 | -0.184 | 0.115 |
| sp\|O94910\|AGRL1_HUMAN Adhesion G protein-coupled receptor L1 OS=Homo sapiens GN=ADGRL1 PE=1 SV | 402 | FGPPDPsAGPA | 0.124 | -0.184 | -0.987 | -0.349 |
| sp\|O94910\|AGRL1_HUMAN Adhesion G protein-coupled receptor L1 OS=Homo sapiens GN=ADGRL1 PE=1 SV | 407 | PSAGPAtSPPL | 0.406 | 0.316 | -0.027 | 0.232 |
| sp\|O94910\|AGRL1_HUMAN Adhesion G protein-coupled receptor L1 OS=Homo sapiens GN=ADGRL1 PE=1 SV | 408 | SAGPATsPPLS | 0.164 | 0.051 | -0.865 | -0.217 |
| sp\|O94910\|AGRL1_HUMAN Adhesion G protein-coupled receptor L1 OS=Homo sapiens GN=ADGRL1 PE=1 SV | 412 | ATSPPLsTTTT | 0.067 | -0.423 | -1.417 | -0.591 |
| sp\|O94910\|AGRL1_HUMAN Adhesion G protein-coupled receptor L1 OS=Homo sapiens GN=ADGRL1 PE=1 SV | 413 | TSPPLStTTTA | 0.044 | -0.448 | -1.646 | -0.683 |
| sp\|O94910\|AGRL1_HUMAN Adhesion G protein-coupled receptor L1 OS=Homo sapiens GN=ADGRL1 PE=1 SV | 414 | SPPLSTtTTAR | 0.237 | 0.07 | -0.699 | -0.131 |
| sp\|O94910\|AGRL1_HUMAN Adhesion G protein-coupled receptor L1 OS=Homo sapiens GN=ADGRL1 PE=1 SV | 415 | PPLSTTtTARP | 0.06 | -0.3 | -1.466 | -0.569 |
| sp\|O94910\|AGRL1_HUMAN Adhesion G protein-coupled receptor L1 OS=Homo sapiens GN=ADGRL1 PE=1 SV | 416 | PLSTTTtARPT | 0.183 | -0.064 | -0.798 | -0.226 |
| sp\|O94910\|AGRL1_HUMAN Adhesion G protein-coupled receptor L1 OS=Homo sapiens GN=ADGRL1 PE=1 SV | 420 | TTTARPtPLTS | 0.084 | -0.4 | -1.375 | -0.564 |
| sp\|O94910\|AGRL1_HUMAN Adhesion G protein-coupled receptor L1 OS=Homo sapiens GN=ADGRL1 PE=1 SV | 423 | ARPTPLtSTAS | 0.089 | -0.072 | -1.252 | -0.412 |
| sp\|O94910\|AGRL1_HUMAN Adhesion G protein-coupled receptor L1 OS=Homo sapiens GN=ADGRL1 PE=1 SV | 424 | RPTPLTsTASP | 0.041 | -0.318 | -1.583 | -0.62 |
| sp\|O94910\|AGRL1_HUMAN Adhesion G protein-coupled receptor L1 OS=Homo sapiens GN=ADGRL1 PE=1 SV | 425 | PTPLTStASPA | 0.049 | -0.243 | -1.633 | -0.609 |
| sp\|O94910\|AGRL1_HUMAN Adhesion G protein-coupled receptor L1 OS=Homo sapiens GN=ADGRL1 PE=1 SV | 427 | PLTSTAsPAAT | 0.119 | -0.227 | -0.902 | -0.337 |

| | | | | | | |
|---|---|---|---|---|---|---|
| sp\|O94910\|AGRL1_HUMAN Adhesion G protein-coupled receptor L1 OS=Homo sapiens GN=ADGRL1 PE=1 SV | 431 | TASPAAtTPLR | 0.585 | 0.416 | -0.003 | 0.333 |
| sp\|O94910\|AGRL1_HUMAN Adhesion G protein-coupled receptor L1 OS=Homo sapiens GN=ADGRL1 PE=1 Sv | 432 | ASPAATtPLRR | 0.068 | -0.54 | -1.6 | -0.691 |
| sp\|O94910\|AGRL1_HUMAN Adhesion G protein-coupled receptor L1 OS=Homo sapiens GN=ADGRL1 PE=1 Sv | 440 | LRRAPLtTHPV | 0.249 | 0.214 | -0.693 | -0.077 |
| sp\|O94910\|AGRL1_HUMAN Adhesion G protein-coupled receptor L1 OS=Homo sapiens GN=ADGRL1 PE=1 Sv | 441 | RRAPLTtHPVG | 0.638 | 0.672 | 0.193 | 0.501 |
| sp\|O94910\|AGRL1_HUMAN Adhesion G protein-coupled receptor L1 OS=Homo sapiens GN=ADGRL1 PE=1 Sv | 458 | PDLPPAtAPVP | 0.335 | 0.292 | -0.38 | 0.082 |
| sp\|O94910\|AGRL1_HUMAN Adhesion G protein-coupled receptor L1 OS=Homo sapiens GN=ADGRL1 PE=1 SV | 463 | ATAPVPsTRRP | 0.103 | -0.439 | -1.421 | -0.586 |
| sp\|O94910\|AGRL1_HUMAN Adhesion G protein-coupled receptor L1 OS=Homo sapiens GN=ADGRL1 PE=1 Sv | 464 | TAPVPStRRPP | 0.021 | -0.628 | -2.02 | -0.876 |
| sp\|O94910\|AGRL1_HUMAN Adhesion G protein-coupled receptor L1 OS=Homo sapiens GN=ADGRL1 PE=1 Sv | 475 | APNLHVsPELF | 0.096 | -0.379 | -1.484 | -0.589 |
| sp\|O94910\|AGRL1_HUMAN Adhesion G protein-coupled receptor L1 OS=Homo sapiens GN=ADGRL1 PE=1 Sv | 493 | RVQWPAtQQGM | 0.258 | -0.105 | -0.651 | -0.166 |
| sp\|O94910\|AGRL1_HUMAN Adhesion G protein-coupled receptor L1 OS=Homo sapiens GN=ADGRL1 PE=1 Sv | 507 | RPCPKGtRGIA | 0.12 | -0.281 | -1.289 | -0.483 |
| sp\|O94910\|AGRL1_HUMAN Adhesion G protein-coupled receptor L1 OS=Homo sapiens GN=ADGRL1 PE=1 SV | 512 | GTRGIAsFQCL | 0.668 | 0.446 | 0.196 | 0.437 |
| sp\|O94910\|AGRL1_HUMAN Adhesion G protein-coupled receptor L1 OS=Homo sapiens GN=ADGRL1 PE=1 SV | 530 | PRGPDLsNCTS | 0.049 | -0.143 | -1.538 | -0.544 |
| sp\|O94910\|AGRL1_HUMAN Adhesion G protein-coupled receptor L1 OS=Homo sapiens GN=ADGRL1 PE=1 SV | 533 | PDLSNCtSPWV | 0.482 | 0.327 | -0.387 | 0.141 |
| sp\|O94910\|AGRL1_HUMAN Adhesion G protein-coupled receptor L1 OS=Homo sapiens GN=ADGRL1 PE=1 Sv | 534 | DLSNCTsPWVN | 0.304 | -0.15 | -0.746 | -0.197 |
| sp\|O94910\|AGRL1_HUMAN Adhesion G protein-coupled receptor L1 OS=Homo sapiens GN=ADGRL1 PE=1 Sv | 546 | VAQKIKsGENA | 0.309 | 0.012 | -0.405 | -0.028 |
| sp\|O94910\|AGRL1_HUMAN Adhesion G protein-coupled receptor L1 OS=Homo sapiens GN=ADGRL1 PE=1 SV | 555 | NAANIAsELAR | 0.22 | 0.076 | -0.311 | -0.005 |
| sp\|O94910\|AGRL1_HUMAN Adhesion G protein-coupled receptor L1 OS=Homo sapiens GN=ADGRL1 PE=1 Sv | 561 | SELARHtRGSI | 0.314 | 0.103 | -0.638 | -0.074 |
| sp\|O94910\|AGRL1_HUMAN Adhesion G protein-coupled receptor L1 OS=Homo sapiens GN=ADGRL1 PE=1 Sv | 564 | ARHTRGsIYAG | 0.211 | 0.202 | -0.746 | -0.111 |
| sp\|O94910\|AGRL1_HUMAN Adhesion G protein-coupled receptor L1 OS=Homo sapiens GN=ADGRL1 PE=1 Sv | 571 | IYAGDVsSSVK | 0.2 | -0.154 | -0.735 | -0.23 |
| sp\|O94910\|AGRL1_HUMAN Adhesion G protein-coupled receptor L1 OS=Homo sapiens GN=ADGRL1 PE=1 Sv | 572 | YAGDVSsSVKL | 0.055 | -0.196 | -1.57 | -0.57 |
| sp\|O94910\|AGRL1_HUMAN Adhesion G protein-coupled receptor L1 OS=Homo sapiens GN=ADGRL1 PE=1 SV | 573 | AGDVSSsVKLM | 0.134 | -0.006 | -0.776 | -0.216 |
| sp\|O94910\|AGRL1_HUMAN Adhesion G protein-coupled receptor L1 OS=Homo sapiens GN=ADGRL1 PE=1 Sv | 598 | RPIEREsAGKN | 0.247 | 0.15 | -1.059 | -0.221 |
| sp\|O94910\|AGRL1_HUMAN Adhesion G protein-coupled receptor L1 OS=Homo sapiens GN=ADGRL1 PE=1 SV | 612 | MHKRERtCKDY | 0.769 | 1.055 | 0.193 | 0.672 |
| sp\|O94910\|AGRL1_HUMAN Adhesion G protein-coupled receptor L1 OS=Homo sapiens GN=ADGRL1 PE=1 Sv | 623 | IKAVVEtVDNL | 0.346 | 0.095 | -0.523 | -0.027 |
| sp\|O94910\|AGRL1_HUMAN Adhesion G protein-coupled receptor L1 OS=Homo sapiens GN=ADGRL1 PE=1 Sv | 635 | RPEALEsWKDM | 0.17 | -0.073 | -0.982 | -0.295 |
| sp\|O94910\|AGRL1_HUMAN Adhesion G protein-coupled receptor L1 OS=Homo sapiens GN=ADGRL1 PE=1 SV | 642 | WKDMNAtEQVH | 0.377 | -0.031 | -0.493 | -0.049 |
| sp\|O94910\|AGRL1_HUMAN Adhesion G protein-coupled receptor L1 OS=Homo sapiens GN=ADGRL1 PE=1 Sv | 647 | ATEQVHtATML | 0.42 | 0.025 | -0.724 | -0.093 |
| sp\|O94910\|AGRL1_HUMAN Adhesion G protein-coupled receptor L1 OS=Homo sapiens GN=ADGRL1 PE=1 Sv | 649 | EQVHTAtMLLD | 0.097 | 0.09 | -0.954 | -0.256 |
| sp\|O94910\|AGRL1_HUMAN Adhesion G protein-coupled receptor L1 OS=Homo sapiens GN=ADGRL1 PE=1 Sv | 684 | NVVLEVtVLNT | 0.136 | -0.087 | -1.236 | -0.396 |
| sp\|O94910\|AGRL1_HUMAN Adhesion G protein-coupled receptor L1 OS=Homo sapiens GN=ADGRL1 PE=1 Sv | 688 | EVTVLNtEGQV | 0.18 | 0.043 | -0.924 | -0.234 |
| sp\|O94910\|AGRL1_HUMAN Adhesion G protein-coupled receptor L1 OS=Homo sapiens GN=ADGRL1 PE=1 SV | 707 | EYPRKNsIQLS | 0.417 | 0.773 | -0.059 | 0.377 |
| sp\|O94910\|AGRL1_HUMAN Adhesion G protein-coupled receptor L1 OS=Homo sapiens GN=ADGRL1 PE=1 Sv | 711 | KNSIQLsAKTI | 0.068 | -0.184 | -1.283 | -0.466 |
| sp\|O94910\|AGRL1_HUMAN Adhesion G protein-coupled receptor L1 OS=Homo sapiens GN=ADGRL1 PE=1 Sv | 714 | IQLSAKtIKQN | 0.278 | -0.05 | -0.205 | 0.008 |
| sp\|O94910\|AGRL1_HUMAN Adhesion G protein-coupled receptor L1 OS=Homo sapiens GN=ADGRL1 PE=1 Sv | 719 | KTIKQNsRNGV | 0.299 | -0.072 | -1.057 | -0.277 |
| sp\|O94910\|AGRL1_HUMAN Adhesion G protein-coupled receptor L1 OS=Homo sapiens GN=ADGRL1 PE=1 Sv | 739 | NLGLFLsTENA | 0.121 | -0.037 | -1.217 | -0.378 |
| sp\|O94910\|AGRL1_HUMAN Adhesion G protein-coupled receptor L1 OS=Homo sapiens GN=ADGRL1 PE=1 SV | 740 | LGLFLStENAT | 0.145 | -0.029 | -0.581 | -0.155 |
| sp\|O94910\|AGRL1_HUMAN Adhesion G protein-coupled receptor L1 OS=Homo sapiens GN=ADGRL1 PE=1 Sv | 744 | LSTENAtVKLA | 0.151 | -0.1 | -0.681 | -0.21 |
| sp\|O94910\|AGRL1_HUMAN Adhesion G protein-coupled receptor L1 OS=Homo sapiens GN=ADGRL1 PE=1 Sv | 760 | GGPGGAsLVVN | 0.21 | 0.036 | -0.702 | -0.152 |
| sp\|O94910\|AGRL1_HUMAN Adhesion G protein-coupled receptor L1 OS=Homo sapiens GN=ADGRL1 PE=1 Sv | 765 | ASLVVNsQVIA | 0.158 | -0.203 | -1.297 | -0.447 |
| sp\|O94910\|AGRL1_HUMAN Adhesion G protein-coupled receptor L1 OS=Homo sapiens GN=ADGRL1 PE=1 Sv | 771 | SQVIAAsINKE | 0.127 | -0.072 | -0.937 | -0.294 |
| sp\|O94910\|AGRL1_HUMAN Adhesion G protein-coupled receptor L1 OS=Homo sapiens GN=ADGRL1 PE=1 SV | 776 | ASINKEsSRVF | 0.048 | -0.318 | -1.522 | -0.597 |
| sp\|O94910\|AGRL1_HUMAN Adhesion G protein-coupled receptor L1 OS=Homo sapiens GN=ADGRL1 PE=1 Sv | 777 | SINKESsRVFL | 0.091 | -0.209 | -1.6 | -0.573 |
| sp\|O94910\|AGRL1_HUMAN Adhesion G protein-coupled receptor L1 OS=Homo sapiens GN=ADGRL1 PE=1 Sv | 788 | MDPVIFtVAHL | 0.053 | -0.147 | -1.045 | -0.38 |
| sp\|O94910\|AGRL1_HUMAN Adhesion G protein-coupled receptor L1 OS=Homo sapiens GN=ADGRL1 PE=1 Sv | 803 | HFNANCsFWNY | 0.612 | 0.224 | -0.29 | 0.182 |
| sp\|O94910\|AGRL1_HUMAN Adhesion G protein-coupled receptor L1 OS=Homo sapiens GN=ADGRL1 PE=1 Sv | 808 | CSFWNYsERSM | 0.261 | -0.033 | -0.278 | -0.017 |
| sp\|O94910\|AGRL1_HUMAN Adhesion G protein-coupled receptor L1 OS=Homo sapiens GN=ADGRL1 PE=1 SV | 811 | WNYSERsMLGY | 0.065 | -0.207 | -1.518 | -0.553 |
| sp\|O94910\|AGRL1_HUMAN Adhesion G protein-coupled receptor L1 OS=Homo sapiens GN=ADGRL1 PE=1 Sv | 817 | SMLGYWsTQGC | 0.095 | -0.145 | -1.222 | -0.424 |
| sp\|O94910\|AGRL1_HUMAN Adhesion G protein-coupled receptor L1 OS=Homo sapiens GN=ADGRL1 PE=1 Sv | 818 | MLGYWStQGCR | 0.336 | 0.186 | -0.489 | 0.011 |
| sp\|O94910\|AGRL1_HUMAN Adhesion G protein-coupled receptor L1 OS=Homo sapiens GN=ADGRL1 PE=1 Sv | 826 | GCRLVEsNKTH | 0.308 | 0.038 | -1.185 | -0.28 |

| | | | | | | |
|---|---|---|---|---|---|---|
| sp\|O94910\|AGRL1_HUMAN Adhesion G protein-coupled receptor L1 OS=Homo sapiens GN=ADGRL1 PE=1 SV | 829 | LVESNKtHTTC | 0.235 | -0.158 | -0.675 | -0.199 |
| sp\|O94910\|AGRL1_HUMAN Adhesion G protein-coupled receptor L1 OS=Homo sapiens GN=ADGRL1 PE=1 Sv | 831 | ESNKTHtTCAC | 0.155 | -0.027 | -1.026 | -0.299 |
| sp\|O94910\|AGRL1_HUMAN Adhesion G protein-coupled receptor L1 OS=Homo sapiens GN=ADGRL1 PE=1 Sv | 832 | SNKTHTtCACS | 0.123 | -0.07 | -1.156 | -0.368 |
| sp\|O94910\|AGRL1_HUMAN Adhesion G protein-coupled receptor L1 OS=Homo sapiens GN=ADGRL1 PE=1 Sv | 836 | HTTCACsHLTN | 0.439 | 0.072 | -0.664 | -0.051 |
| sp\|O94910\|AGRL1_HUMAN Adhesion G protein-coupled receptor L1 OS=Homo sapiens GN=ADGRL1 PE=1 Sv | 839 | CACSHLtNFAV | 0.216 | -0.049 | -1.03 | -0.288 |
| sp\|O94910\|AGRL1_HUMAN Adhesion G protein-coupled receptor L1 OS=Homo sapiens GN=ADGRL1 PE=1 SV | 861 | INELLLsVITW | 0.066 | -0.236 | -1.145 | -0.438 |
| sp\|O94910\|AGRL1_HUMAN Adhesion G protein-coupled receptor L1 OS=Homo sapiens GN=ADGRL1 PE=1 Sv | 864 | LLLSVItWVGI | 0.335 | 0.081 | -0.29 | 0.042 |
| sp\|O94910\|AGRL1_HUMAN Adhesion G protein-coupled receptor L1 OS=Homo sapiens GN=ADGRL1 PE=1 Sv | 871 | WVGIVIsLVCL | 0.207 | 0.105 | -0.554 | -0.081 |
| sp\|O94910\|AGRL1_HUMAN Adhesion G protein-coupled receptor L1 OS=Homo sapiens GN=ADGRL1 PE=1 SV | 880 | CLAICIsTFCF | 0.421 | 0.157 | -0.141 | 0.146 |
| sp\|O94910\|AGRL1_HUMAN Adhesion G protein-coupled receptor L1 OS=Homo sapiens GN=ADGRL1 PE=1 Sv | 881 | LAICIStFCFL | 0.089 | -0.198 | -1.103 | -0.404 |
| sp\|O94910\|AGRL1_HUMAN Adhesion G protein-coupled receptor L1 OS=Homo sapiens GN=ADGRL1 PE=1 SV | 890 | FLRGLQtDRNT | 0.476 | 0.324 | -0.353 | 0.149 |
| sp\|O94910\|AGRL1_HUMAN Adhesion G protein-coupled receptor L1 OS=Homo sapiens GN=ADGRL1 PE=1 Sv | 894 | LQTDRNtIHKN | 0.301 | 0.129 | -0.543 | -0.038 |
| sp\|O94910\|AGRL1_HUMAN Adhesion G protein-coupled receptor L1 OS=Homo sapiens GN=ADGRL1 PE=1 Sv | 917 | LVGIDKtQYEI | 0.169 | 0.008 | -0.717 | -0.18 |
| sp\|O94910\|AGRL1_HUMAN Adhesion G protein-coupled receptor L1 OS=Homo sapiens GN=ADGRL1 PE=1 Sv | 939 | FFLAAFsWLCL | 0.319 | 0.301 | -0.299 | 0.107 |
| sp\|O94910\|AGRL1_HUMAN Adhesion G protein-coupled receptor L1 OS=Homo sapiens GN=ADGRL1 PE=1 Sv | 958 | LVEVFEsEYSR | 0.067 | -0.107 | -1.271 | -0.437 |
| sp\|O94910\|AGRL1_HUMAN Adhesion G protein-coupled receptor L1 OS=Homo sapiens GN=ADGRL1 PE=1 SV | 961 | VFESEYsRTKY | 0.182 | -0.174 | -1.007 | -0.333 |
| sp\|O94910\|AGRL1_HUMAN Adhesion G protein-coupled receptor L1 OS=Homo sapiens GN=ADGRL1 PE=1 Sv | 963 | ESEYSRtKYYY | 0.274 | 0.116 | -0.498 | -0.036 |
| sp\|O94910\|AGRL1_HUMAN Adhesion G protein-coupled receptor L1 OS=Homo sapiens GN=ADGRL1 PE=1 Sv | 988 | AAIDYRsYGTE | 0.14 | -0.154 | -1.322 | -0.445 |
| sp\|O94910\|AGRL1_HUMAN Adhesion G protein-coupled receptor L1 OS=Homo sapiens GN=ADGRL1 PE=1 Sv | 991 | DYRSYGtEKAC | 0.398 | 0.175 | -0.61 | -0.012 |
| sp\|O94910\|AGRL1_HUMAN Adhesion G protein-coupled receptor L1 OS=Homo sapiens GN=ADGRL1 PE=1 Sv | 1006 | DNYFIWsFIGP | 0.16 | -0.184 | -0.648 | -0.224 |
| sp\|O94910\|AGRL1_HUMAN Adhesion G protein-coupled receptor L1 OS=Homo sapiens GN=ADGRL1 PE=1 SV | 1012 | SFIGPVsFVIV | 0.257 | -0.05 | -0.761 | -0.185 |
| sp\|O94910\|AGRL1_HUMAN Adhesion G protein-coupled receptor L1 OS=Homo sapiens GN=ADGRL1 PE=1 Sv | 1025 | LVFLMVtLHKM | 0.394 | 0.006 | -0.328 | 0.024 |
| sp\|O94910\|AGRL1_HUMAN Adhesion G protein-coupled receptor L1 OS=Homo sapiens GN=ADGRL1 PE=1 Sv | 1032 | LHKMIRsSSVL | 0.081 | -0.112 | -1.262 | -0.431 |
| sp\|O94910\|AGRL1_HUMAN Adhesion G protein-coupled receptor L1 OS=Homo sapiens GN=ADGRL1 PE=1 Sv | 1033 | HKMIRSsSVLK | 0.24 | 0.167 | -0.549 | -0.047 |
| sp\|O94910\|AGRL1_HUMAN Adhesion G protein-coupled receptor L1 OS=Homo sapiens GN=ADGRL1 PE=1 Sv | 1034 | KMIRSSsVLKP | 0.329 | 0.982 | -0.091 | 0.407 |
| sp\|O94910\|AGRL1_HUMAN Adhesion G protein-coupled receptor L1 OS=Homo sapiens GN=ADGRL1 PE=1 SV | 1040 | SVLKPDsSRLD | 0.147 | -0.259 | -1.036 | -0.383 |
| sp\|O94910\|AGRL1_HUMAN Adhesion G protein-coupled receptor L1 OS=Homo sapiens GN=ADGRL1 PE=1 Sv | 1041 | VLKPDSsRLDN | 0.17 | -0.136 | -0.951 | -0.306 |
| sp\|O94910\|AGRL1_HUMAN Adhesion G protein-coupled receptor L1 OS=Homo sapiens GN=ADGRL1 PE=1 Sv | 1048 | RLDNIKsWALG | 0.314 | 0.177 | -0.199 | 0.097 |
| sp\|O94910\|AGRL1_HUMAN Adhesion G protein-coupled receptor L1 OS=Homo sapiens GN=ADGRL1 PE=1 Sv | 1063 | LFLLGLtWAFG | 0.049 | -0.178 | -1.276 | -0.468 |
| sp\|O94910\|AGRL1_HUMAN Adhesion G protein-coupled receptor L1 OS=Homo sapiens GN=ADGRL1 PE=1 Sv | 1075 | LFINKEsVVMA | 0.075 | -0.174 | -1.074 | -0.391 |
| sp\|O94910\|AGRL1_HUMAN Adhesion G protein-coupled receptor L1 OS=Homo sapiens GN=ADGRL1 PE=1 SV | 1083 | VMAYLFtTFNA | 0.13 | 0.072 | -0.797 | -0.198 |
| sp\|O94910\|AGRL1_HUMAN Adhesion G protein-coupled receptor L1 OS=Homo sapiens GN=ADGRL1 PE=1 Sv | 1084 | MAYLFTtFNAF | 0.317 | 0.13 | -0.323 | 0.041 |
| sp\|O94910\|AGRL1_HUMAN Adhesion G protein-coupled receptor L1 OS=Homo sapiens GN=ADGRL1 PE=1 Sv | 1109 | KVHKEYsKCLR | 0.138 | -0.086 | -0.923 | -0.29 |
| sp\|O94910\|AGRL1_HUMAN Adhesion G protein-coupled receptor L1 OS=Homo sapiens GN=ADGRL1 PE=1 Sv | 1115 | SKCLRHsYCCI | 0.474 | 0.238 | -0.46 | 0.084 |
| sp\|O94910\|AGRL1_HUMAN Adhesion G protein-coupled receptor L1 OS=Homo sapiens GN=ADGRL1 PE=1 Sv | 1121 | SYCCIRsPPGG | 0.33 | 0.107 | -0.65 | -0.071 |
| sp\|O94910\|AGRL1_HUMAN Adhesion G protein-coupled receptor L1 OS=Homo sapiens GN=ADGRL1 PE=1 SV | 1126 | RSPPGGtHGSL | 0.056 | -0.269 | -1.414 | -0.542 |
| sp\|O94910\|AGRL1_HUMAN Adhesion G protein-coupled receptor L1 OS=Homo sapiens GN=ADGRL1 PE=1 Sv | 1129 | PGGTHGsLKTS | 0.179 | -0.083 | -0.813 | -0.239 |
| sp\|O94910\|AGRL1_HUMAN Adhesion G protein-coupled receptor L1 OS=Homo sapiens GN=ADGRL1 PE=1 Sv | 1132 | THGSLKtSAMR | 0.058 | -0.297 | -1.637 | -0.625 |
| sp\|O94910\|AGRL1_HUMAN Adhesion G protein-coupled receptor L1 OS=Homo sapiens GN=ADGRL1 PE=1 Sv | 1133 | HGSLKTsAMRS | 0.107 | -0.138 | -1.228 | -0.42 |
| sp\|O94910\|AGRL1_HUMAN Adhesion G protein-coupled receptor L1 OS=Homo sapiens GN=ADGRL1 PE=1 Sv | 1137 | KTSAMRsNTRY | 0.201 | -0.325 | -1.528 | -0.551 |
| sp\|O94910\|AGRL1_HUMAN Adhesion G protein-coupled receptor L1 OS=Homo sapiens GN=ADGRL1 PE=1 SV | 1139 | SAMRSNtRYYT | 0.579 | 0.883 | 0.085 | 0.516 |
| sp\|O94910\|AGRL1_HUMAN Adhesion G protein-coupled receptor L1 OS=Homo sapiens GN=ADGRL1 PE=1 Sv | 1143 | SNTRYYtGTQS | 0.441 | 0.682 | 0.126 | 0.416 |
| sp\|O94910\|AGRL1_HUMAN Adhesion G protein-coupled receptor L1 OS=Homo sapiens GN=ADGRL1 PE=1 Sv | 1145 | TRYYTGtQSRI | 0.082 | 0.001 | -1.265 | -0.394 |
| sp\|O94910\|AGRL1_HUMAN Adhesion G protein-coupled receptor L1 OS=Homo sapiens GN=ADGRL1 PE=1 Sv | 1147 | YYTGTQsRIRR | 0.126 | -0.087 | -0.825 | -0.262 |
| sp\|O94910\|AGRL1_HUMAN Adhesion G protein-coupled receptor L1 OS=Homo sapiens GN=ADGRL1 PE=1 Sv | 1156 | RRMWNDtVRKQ | 0.26 | 0.043 | -0.706 | -0.134 |
| sp\|O94910\|AGRL1_HUMAN Adhesion G protein-coupled receptor L1 OS=Homo sapiens GN=ADGRL1 PE=1 SV | 1161 | DTVRKQtESSF | 0.293 | 0.709 | -0.37 | 0.211 |
| sp\|O94910\|AGRL1_HUMAN Adhesion G protein-coupled receptor L1 OS=Homo sapiens GN=ADGRL1 PE=1 Sv | 1163 | VRKQTEsSFMA | 0.177 | 0.062 | -0.724 | -0.162 |
| sp\|O94910\|AGRL1_HUMAN Adhesion G protein-coupled receptor L1 OS=Homo sapiens GN=ADGRL1 PE=1 Sv | 1164 | RKQTESsFMAG | 0.483 | 0.165 | -0.363 | 0.095 |
| sp\|O94910\|AGRL1_HUMAN Adhesion G protein-coupled receptor L1 OS=Homo sapiens GN=ADGRL1 PE=1 Sv | 1172 | MAGDINsTPTL | 0.581 | 0.535 | -0.007 | 0.37 |

| | | | | | | |
|---|---|---|---|---|---|---|
| sp\|O94910\|AGRL1_HUMAN Adhesion G protein-coupled receptor L1 OS=Homo sapiens GN=ADGRL1 PE=1 SV | 1173 | AGDINStPTLN | 0.06 | -0.454 | -1.529 | -0.641 |
| sp\|O94910\|AGRL1_HUMAN Adhesion G protein-coupled receptor L1 OS=Homo sapiens GN=ADGRL1 PE=1 SV | 1175 | DINSTPtLNRG | 0.326 | -0.02 | -0.724 | -0.139 |
| sp\|O94910\|AGRL1_HUMAN Adhesion G protein-coupled receptor L1 OS=Homo sapiens GN=ADGRL1 PE=1 SV | 1180 | PTLNRGtMGNH | 0.248 | 0.104 | -0.911 | -0.186 |
| sp\|O94910\|AGRL1_HUMAN Adhesion G protein-coupled receptor L1 OS=Homo sapiens GN=ADGRL1 PE=1 SV | 1187 | MGNHLLtNPVL | 0.331 | 0.425 | -0.474 | 0.094 |
| sp\|O94910\|AGRL1_HUMAN Adhesion G protein-coupled receptor L1 OS=Homo sapiens GN=ADGRL1 PE=1 SV | 1197 | LQPRGGtSPYN | 0.495 | 1.05 | 0.182 | 0.576 |
| sp\|O94910\|AGRL1_HUMAN Adhesion G protein-coupled receptor L1 OS=Homo sapiens GN=ADGRL1 PE=1 SV | 1198 | QPRGGTsPYNT | 0.068 | -0.211 | -1.275 | -0.473 |
| sp\|O94910\|AGRL1_HUMAN Adhesion G protein-coupled receptor L1 OS=Homo sapiens GN=ADGRL1 PE=1 SV | 1202 | GTSPYNtLIAE | 0.505 | 0.095 | -0.195 | 0.135 |
| sp\|O94910\|AGRL1_HUMAN Adhesion G protein-coupled receptor L1 OS=Homo sapiens GN=ADGRL1 PE=1 SV | 1207 | NTLIAEsVGFN | 0.254 | -0.005 | -0.831 | -0.194 |
| sp\|O94910\|AGRL1_HUMAN Adhesion G protein-coupled receptor L1 OS=Homo sapiens GN=ADGRL1 PE=1 SV | 1213 | SVGFNPsSPPV | 0.248 | 0.25 | -0.764 | -0.089 |
| sp\|O94910\|AGRL1_HUMAN Adhesion G protein-coupled receptor L1 OS=Homo sapiens GN=ADGRL1 PE=1 SV | 1214 | VGFNPSsPPVF | 0.104 | -0.025 | -0.71 | -0.21 |
| sp\|O94910\|AGRL1_HUMAN Adhesion G protein-coupled receptor L1 OS=Homo sapiens GN=ADGRL1 PE=1 SV | 1220 | SPPVFNsPGSY | 0.039 | -0.448 | -2.003 | -0.804 |
| sp\|O94910\|AGRL1_HUMAN Adhesion G protein-coupled receptor L1 OS=Homo sapiens GN=ADGRL1 PE=1 SV | 1223 | VFNSPGsYREP | 0.053 | -0.44 | -1.458 | -0.615 |
| sp\|O94910\|AGRL1_HUMAN Adhesion G protein-coupled receptor L1 OS=Homo sapiens GN=ADGRL1 PE=1 SV | 1241 | EACGMDtLPLN | 0.596 | 0.4 | 0.009 | 0.335 |
| sp\|O94910\|AGRL1_HUMAN Adhesion G protein-coupled receptor L1 OS=Homo sapiens GN=ADGRL1 PE=1 SV | 1251 | NGNFNNsYSLR | 0.18 | -0.078 | -0.887 | -0.262 |
| sp\|O94910\|AGRL1_HUMAN Adhesion G protein-coupled receptor L1 OS=Homo sapiens GN=ADGRL1 PE=1 SV | 1253 | NFNNSYsLRSG | 0.421 | 0.269 | 0.191 | 0.294 |
| sp\|O94910\|AGRL1_HUMAN Adhesion G protein-coupled receptor L1 OS=Homo sapiens GN=ADGRL1 PE=1 SV | 1256 | NSYSLRsGDFP | 0.037 | -0.376 | -1.73 | -0.69 |
| sp\|O94910\|AGRL1_HUMAN Adhesion G protein-coupled receptor L1 OS=Homo sapiens GN=ADGRL1 PE=1 SV | 1286 | FEKMIIsELVH | 0.197 | -0.031 | -0.847 | -0.227 |
| sp\|O94910\|AGRL1_HUMAN Adhesion G protein-coupled receptor L1 OS=Homo sapiens GN=ADGRL1 PE=1 SV | 1296 | HNNLRGsSSAA | 0.111 | -0.064 | -1.022 | -0.325 |
| sp\|O94910\|AGRL1_HUMAN Adhesion G protein-coupled receptor L1 OS=Homo sapiens GN=ADGRL1 PE=1 SV | 1297 | NNLRGSsSAAK | 0.238 | 0.546 | -0.22 | 0.188 |
| sp\|O94910\|AGRL1_HUMAN Adhesion G protein-coupled receptor L1 OS=Homo sapiens GN=ADGRL1 PE=1 SV | 1298 | NLRGSSsAAKG | 0.459 | 0.424 | -0.134 | 0.25 |
| sp\|O94910\|AGRL1_HUMAN Adhesion G protein-coupled receptor L1 OS=Homo sapiens GN=ADGRL1 PE=1 SV | 1351 | LLPRAQsVLYQ | 0.581 | 0.866 | 0.202 | 0.55 |
| sp\|O94910\|AGRL1_HUMAN Adhesion G protein-coupled receptor L1 OS=Homo sapiens GN=ADGRL1 PE=1 SV | 1356 | QSVLYQsDLDE | 0.197 | -0.063 | -0.989 | -0.285 |
| sp\|O94910\|AGRL1_HUMAN Adhesion G protein-coupled receptor L1 OS=Homo sapiens GN=ADGRL1 PE=1 SV | 1361 | QSDLDEsESCT | 0.065 | -0.231 | -1.536 | -0.567 |
| sp\|O94910\|AGRL1_HUMAN Adhesion G protein-coupled receptor L1 OS=Homo sapiens GN=ADGRL1 PE=1 SV | 1363 | DLDESEsCTAE | 0.478 | 0.263 | -0.483 | 0.086 |
| sp\|O94910\|AGRL1_HUMAN Adhesion G protein-coupled receptor L1 OS=Homo sapiens GN=ADGRL1 PE=1 SV | 1365 | DESESCtAEDG | 0.462 | 0.207 | -0.701 | -0.011 |
| sp\|O94910\|AGRL1_HUMAN Adhesion G protein-coupled receptor L1 OS=Homo sapiens GN=ADGRL1 PE=1 SV | 1371 | TAEDGAtSRPL | 0.096 | -0.272 | -1.262 | -0.479 |
| sp\|O94910\|AGRL1_HUMAN Adhesion G protein-coupled receptor L1 OS=Homo sapiens GN=ADGRL1 PE=1 SV | 1372 | AEDGATsRPLS | 0.418 | 0.345 | -0.428 | 0.112 |
| sp\|O94910\|AGRL1_HUMAN Adhesion G protein-coupled receptor L1 OS=Homo sapiens GN=ADGRL1 PE=1 SV | 1376 | ATSRPLsSPPG | 0.707 | 1.031 | 0.462 | 0.733 |
| sp\|O94910\|AGRL1_HUMAN Adhesion G protein-coupled receptor L1 OS=Homo sapiens GN=ADGRL1 PE=1 SV | 1377 | TSRPLSsPPGR | 0.264 | 0.12 | -0.506 | -0.041 |
| sp\|O94910\|AGRL1_HUMAN Adhesion G protein-coupled receptor L1 OS=Homo sapiens GN=ADGRL1 PE=1 SV | 1383 | SPPGRDsLYAS | 0.193 | 0.021 | -0.829 | -0.205 |
| sp\|O94910\|AGRL1_HUMAN Adhesion G protein-coupled receptor L1 OS=Homo sapiens GN=ADGRL1 PE=1 SV | 1387 | RDSLYAsGANL | 0.139 | -0.098 | -1.176 | -0.378 |
| sp\|O94910\|AGRL1_HUMAN Adhesion G protein-coupled receptor L1 OS=Homo sapiens GN=ADGRL1 PE=1 SV | 1394 | GANLRDsPSYP | 0.07 | -0.446 | -1.883 | -0.753 |
| sp\|O94910\|AGRL1_HUMAN Adhesion G protein-coupled receptor L1 OS=Homo sapiens GN=ADGRL1 PE=1 SV | 1396 | NLRDSPsYPDS | 0.909 | 0.97 | 0.944 | 0.941 |
| sp\|O94910\|AGRL1_HUMAN Adhesion G protein-coupled receptor L1 OS=Homo sapiens GN=ADGRL1 PE=1 SV | 1400 | SPSYPDsSPEG | 0.305 | 0.185 | -0.607 | -0.039 |
| sp\|O94910\|AGRL1_HUMAN Adhesion G protein-coupled receptor L1 OS=Homo sapiens GN=ADGRL1 PE=1 SV | 1401 | PSYPDSsPEGP | 0.026 | -0.656 | -1.783 | -0.804 |
| sp\|O94910\|AGRL1_HUMAN Adhesion G protein-coupled receptor L1 OS=Homo sapiens GN=ADGRL1 PE=1 SV | 1406 | SSPEGPsEALP | 0.025 | -0.404 | -1.973 | -0.784 |
| sp\|O94910\|AGRL1_HUMAN Adhesion G protein-coupled receptor L1 OS=Homo sapiens GN=ADGRL1 PE=1 SV | 1425 | PPEIYYtSRPP | 0.108 | -0.183 | -0.981 | -0.352 |
| sp\|O94910\|AGRL1_HUMAN Adhesion G protein-coupled receptor L1 OS=Homo sapiens GN=ADGRL1 PE=1 SV | 1426 | PEIYYTsRPPA | 0.505 | 0.37 | -0.105 | 0.257 |
| sp\|O94910\|AGRL1_HUMAN Adhesion G protein-coupled receptor L1 OS=Homo sapiens GN=ADGRL1 PE=1 SV | 1447 | YQVRRPsHEGY | 0.713 | 0.917 | 0.596 | 0.742 |
| sp\|O94910\|AGRL1_HUMAN Adhesion G protein-coupled receptor L1 OS=Homo sapiens GN=ADGRL1 PE=1 SV | 1472 | GQMQLVtSL-- | 0.168 | 0.056 | -0.864 | -0.213 |
| sp\|O94910\|AGRL1_HUMAN Adhesion G protein-coupled receptor L1 OS=Homo sapiens GN=ADGRL1 PE=1 SV | 1473 | QMQLVTsL--- | 0.308 | 0.349 | -0.462 | 0.065 |
| sp\|O95490\|AGRL2_HUMAN Adhesion G protein-coupled receptor L2 OS=Homo sapiens GN=ADGRL2 PE=1 SV | 326 | VLYVVRsVYQD | 0.179 | -0.001 | -0.827 | -0.216 |
| sp\|O95490\|AGRL2_HUMAN Adhesion G protein-coupled receptor L2 OS=Homo sapiens GN=ADGRL2 PE=1 SV | 333 | VYQDNEsETGK | 0.35 | -0.041 | -0.701 | -0.131 |
| sp\|O95490\|AGRL2_HUMAN Adhesion G protein-coupled receptor L2 OS=Homo sapiens GN=ADGRL2 PE=1 SV | 335 | QDNESEtGKNS | 0.066 | -0.08 | -1.407 | -0.474 |
| sp\|O95490\|AGRL2_HUMAN Adhesion G protein-coupled receptor L2 OS=Homo sapiens GN=ADGRL2 PE=1 SV | 339 | SETGKNsIDYI | 0.152 | -0.1 | -0.984 | -0.311 |
| sp\|O95490\|AGRL2_HUMAN Adhesion G protein-coupled receptor L2 OS=Homo sapiens GN=ADGRL2 PE=1 SV | 346 | IDYIYNtRLNR | 0.125 | -0.126 | -1.243 | -0.415 |
| sp\|O95490\|AGRL2_HUMAN Adhesion G protein-coupled receptor L2 OS=Homo sapiens GN=ADGRL2 PE=1 SV | 389 | NFILRYsLEFG | 0.435 | 0.27 | -0.097 | 0.203 |
| sp\|O95490\|AGRL2_HUMAN Adhesion G protein-coupled receptor L2 OS=Homo sapiens GN=ADGRL2 PE=1 SV | 402 | DPAQVPtTAVT | 0.139 | -0.251 | -1.108 | -0.407 |
| sp\|O95490\|AGRL2_HUMAN Adhesion G protein-coupled receptor L2 OS=Homo sapiens GN=ADGRL2 PE=1 SV | 403 | PAQVPTtAVTI | 0.164 | -0.044 | -0.803 | -0.228 |

| | | | | | | | |
|---|---|---|---|---|---|---|---|
| sp\|O95490\|AGRL2_HUMAN Adhesion G protein-coupled receptor L2 OS=Homo sapiens GN=ADGRL2 PE=1 SV | 406 | VPTTAVtITSS | 0.176 | -0.176 | -0.955 | -0.318 |
| sp\|O95490\|AGRL2_HUMAN Adhesion G protein-coupled receptor L2 OS=Homo sapiens GN=ADGRL2 PE=1 SV | 408 | TTAVTItSSAE | 0.075 | -0.163 | -1.204 | -0.431 |
| sp\|O95490\|AGRL2_HUMAN Adhesion G protein-coupled receptor L2 OS=Homo sapiens GN=ADGRL2 PE=1 SV | 409 | TAVTITsSAEL | 0.102 | -0.174 | -1.082 | -0.385 |
| sp\|O95490\|AGRL2_HUMAN Adhesion G protein-coupled receptor L2 OS=Homo sapiens GN=ADGRL2 PE=1 SV | 410 | AVTITSsAELF | 0.053 | -0.065 | -1.358 | -0.457 |
| sp\|O95490\|AGRL2_HUMAN Adhesion G protein-coupled receptor L2 OS=Homo sapiens GN=ADGRL2 PE=1 SV | 416 | SAELFKtIIST | 0.057 | -0.263 | -1.265 | -0.49 |
| sp\|O95490\|AGRL2_HUMAN Adhesion G protein-coupled receptor L2 OS=Homo sapiens GN=ADGRL2 PE=1 SV | 419 | LFKTIIsTTST | 0.174 | -0.136 | -0.636 | -0.199 |
| sp\|O95490\|AGRL2_HUMAN Adhesion G protein-coupled receptor L2 OS=Homo sapiens GN=ADGRL2 PE=1 SV | 420 | FKTIIStTSTT | 0.12 | -0.083 | -0.811 | -0.258 |
| sp\|O95490\|AGRL2_HUMAN Adhesion G protein-coupled receptor L2 OS=Homo sapiens GN=ADGRL2 PE=1 SV | 421 | KTIISTtSTTS | 0.251 | 0.058 | -0.805 | -0.165 |
| sp\|O95490\|AGRL2_HUMAN Adhesion G protein-coupled receptor L2 OS=Homo sapiens GN=ADGRL2 PE=1 SV | 422 | TIISTTsTTSQ | 0.074 | -0.124 | -1.37 | -0.473 |
| sp\|O95490\|AGRL2_HUMAN Adhesion G protein-coupled receptor L2 OS=Homo sapiens GN=ADGRL2 PE=1 SV | 423 | IISTTStTSQK | 0.087 | -0.213 | -1.188 | -0.438 |
| sp\|O95490\|AGRL2_HUMAN Adhesion G protein-coupled receptor L2 OS=Homo sapiens GN=ADGRL2 PE=1 SV | 424 | ISTTSTtSQKG | 0.327 | 0.056 | -0.348 | 0.012 |
| sp\|O95490\|AGRL2_HUMAN Adhesion G protein-coupled receptor L2 OS=Homo sapiens GN=ADGRL2 PE=1 SV | 425 | STTSTTsQKGP | 0.048 | -0.325 | -1.569 | -0.615 |
| sp\|O95490\|AGRL2_HUMAN Adhesion G protein-coupled receptor L2 OS=Homo sapiens GN=ADGRL2 PE=1 SV | 431 | SQKGPMsTTVA | 0.16 | -0.058 | -0.674 | -0.191 |
| sp\|O95490\|AGRL2_HUMAN Adhesion G protein-coupled receptor L2 OS=Homo sapiens GN=ADGRL2 PE=1 SV | 432 | QKGPMStTVAG | 0.138 | -0.124 | -0.804 | -0.263 |
| sp\|O95490\|AGRL2_HUMAN Adhesion G protein-coupled receptor L2 OS=Homo sapiens GN=ADGRL2 PE=1 SV | 433 | KGPMSTtVAGS | 0.163 | -0.083 | -0.672 | -0.197 |
| sp\|O95490\|AGRL2_HUMAN Adhesion G protein-coupled receptor L2 OS=Homo sapiens GN=ADGRL2 PE=1 SV | 437 | STTVAGsQEGS | 0.066 | -0.28 | -1.657 | -0.624 |
| sp\|O95490\|AGRL2_HUMAN Adhesion G protein-coupled receptor L2 OS=Homo sapiens GN=ADGRL2 PE=1 SV | 441 | AGSQEGsKGTK | 0.172 | -0.273 | -0.93 | -0.344 |
| sp\|O95490\|AGRL2_HUMAN Adhesion G protein-coupled receptor L2 OS=Homo sapiens GN=ADGRL2 PE=1 SV | 444 | QEGSKGtKPPP | 0.111 | 0.071 | -1.047 | -0.288 |
| sp\|O95490\|AGRL2_HUMAN Adhesion G protein-coupled receptor L2 OS=Homo sapiens GN=ADGRL2 PE=1 SV | 451 | KPPPAVsTTKI | 0.257 | -0.144 | -0.82 | -0.236 |
| sp\|O95490\|AGRL2_HUMAN Adhesion G protein-coupled receptor L2 OS=Homo sapiens GN=ADGRL2 PE=1 SV | 452 | PPPAVStTKIP | 0.061 | -0.333 | -1.518 | -0.597 |
| sp\|O95490\|AGRL2_HUMAN Adhesion G protein-coupled receptor L2 OS=Homo sapiens GN=ADGRL2 PE=1 SV | 453 | PPAVSTtKIPP | 0.139 | 0.009 | -0.526 | -0.126 |
| sp\|O95490\|AGRL2_HUMAN Adhesion G protein-coupled receptor L2 OS=Homo sapiens GN=ADGRL2 PE=1 SV | 459 | TKIPPItNIFP | 0.07 | -0.29 | -1.205 | -0.475 |
| sp\|O95490\|AGRL2_HUMAN Adhesion G protein-coupled receptor L2 OS=Homo sapiens GN=ADGRL2 PE=1 SV | 474 | FCEALDsKGIK | 0.32 | -0.094 | -0.82 | -0.198 |
| sp\|O95490\|AGRL2_HUMAN Adhesion G protein-coupled receptor L2 OS=Homo sapiens GN=ADGRL2 PE=1 SV | 482 | GIKWPQtQRGM | 0.323 | -0.144 | -0.443 | -0.088 |
| sp\|O95490\|AGRL2_HUMAN Adhesion G protein-coupled receptor L2 OS=Homo sapiens GN=ADGRL2 PE=1 SV | 496 | RPCPKGtRGTA | 0.098 | -0.305 | -1.333 | -0.513 |
| sp\|O95490\|AGRL2_HUMAN Adhesion G protein-coupled receptor L2 OS=Homo sapiens GN=ADGRL2 PE=1 SV | 499 | PKGTRGtASYL | 0.244 | 0.075 | -0.926 | -0.202 |
| sp\|O95490\|AGRL2_HUMAN Adhesion G protein-coupled receptor L2 OS=Homo sapiens GN=ADGRL2 PE=1 SV | 501 | GTRGTAsYLCM | 0.271 | 0.251 | -0.665 | -0.048 |
| sp\|O95490\|AGRL2_HUMAN Adhesion G protein-coupled receptor L2 OS=Homo sapiens GN=ADGRL2 PE=1 SV | 507 | SYLCMIsTGTW | 0.269 | -0.099 | -0.783 | -0.204 |
| sp\|O95490\|AGRL2_HUMAN Adhesion G protein-coupled receptor L2 OS=Homo sapiens GN=ADGRL2 PE=1 SV | 508 | YLCMIStGTWN | 0.216 | -0.065 | -0.614 | -0.154 |
| sp\|O95490\|AGRL2_HUMAN Adhesion G protein-coupled receptor L2 OS=Homo sapiens GN=ADGRL2 PE=1 SV | 510 | CMISTGtWNPK | 0.07 | 0.024 | -1.068 | -0.325 |
| sp\|O95490\|AGRL2_HUMAN Adhesion G protein-coupled receptor L2 OS=Homo sapiens GN=ADGRL2 PE=1 SV | 519 | PKGPDLsNCTS | 0.072 | -0.289 | -1.435 | -0.551 |
| sp\|O95490\|AGRL2_HUMAN Adhesion G protein-coupled receptor L2 OS=Homo sapiens GN=ADGRL2 PE=1 SV | 522 | PDLSNCtSHWV | 0.192 | -0.152 | -1.136 | -0.365 |
| sp\|O95490\|AGRL2_HUMAN Adhesion G protein-coupled receptor L2 OS=Homo sapiens GN=ADGRL2 PE=1 SV | 523 | DLSNCTsHWVN | 0.562 | 0.152 | -0.261 | 0.151 |
| sp\|O95490\|AGRL2_HUMAN Adhesion G protein-coupled receptor L2 OS=Homo sapiens GN=ADGRL2 PE=1 SV | 535 | LAQKIRsGENA | 0.167 | -0.04 | -0.875 | -0.249 |
| sp\|O95490\|AGRL2_HUMAN Adhesion G protein-coupled receptor L2 OS=Homo sapiens GN=ADGRL2 PE=1 SV | 541 | SGENAAsLANE | 0.292 | 0.017 | -0.551 | -0.081 |
| sp\|O95490\|AGRL2_HUMAN Adhesion G protein-coupled receptor L2 OS=Homo sapiens GN=ADGRL2 PE=1 SV | 550 | NELAKHtKGPV | 0.23 | -0.027 | -0.869 | -0.222 |
| sp\|O95490\|AGRL2_HUMAN Adhesion G protein-coupled receptor L2 OS=Homo sapiens GN=ADGRL2 PE=1 SV | 560 | VFAGDVsSSVR | 0.157 | -0.148 | -0.753 | -0.248 |
| sp\|O95490\|AGRL2_HUMAN Adhesion G protein-coupled receptor L2 OS=Homo sapiens GN=ADGRL2 PE=1 SV | 561 | FAGDVSsSVRL | 0.076 | -0.169 | -1.555 | -0.549 |
| sp\|O95490\|AGRL2_HUMAN Adhesion G protein-coupled receptor L2 OS=Homo sapiens GN=ADGRL2 PE=1 SV | 562 | AGDVSSsVRLM | 0.158 | -0.044 | -0.752 | -0.213 |
| sp\|O95490\|AGRL2_HUMAN Adhesion G protein-coupled receptor L2 OS=Homo sapiens GN=ADGRL2 PE=1 SV | 583 | LQELKPsEKDS | 0.12 | -0.105 | -0.817 | -0.267 |
| sp\|O95490\|AGRL2_HUMAN Adhesion G protein-coupled receptor L2 OS=Homo sapiens GN=ADGRL2 PE=1 SV | 587 | KPSEKDsAGRS | 0.069 | -0.316 | -1.799 | -0.682 |
| sp\|O95490\|AGRL2_HUMAN Adhesion G protein-coupled receptor L2 OS=Homo sapiens GN=ADGRL2 PE=1 SV | 591 | KDSAGRsYNKL | 0.038 | -0.318 | -1.571 | -0.617 |
| sp\|O95490\|AGRL2_HUMAN Adhesion G protein-coupled receptor L2 OS=Homo sapiens GN=ADGRL2 PE=1 SV | 601 | LQKREKtCRAY | 0.811 | 1.008 | 0.962 | 0.927 |
| sp\|O95490\|AGRL2_HUMAN Adhesion G protein-coupled receptor L2 OS=Homo sapiens GN=ADGRL2 PE=1 SV | 612 | LKAIVDtVDNL | 0.332 | 0.099 | -0.52 | -0.03 |
| sp\|O95490\|AGRL2_HUMAN Adhesion G protein-coupled receptor L2 OS=Homo sapiens GN=ADGRL2 PE=1 SV | 624 | RPEALEsWKHM | 0.084 | -0.195 | -1.228 | -0.446 |
| sp\|O95490\|AGRL2_HUMAN Adhesion G protein-coupled receptor L2 OS=Homo sapiens GN=ADGRL2 PE=1 SV | 630 | SWKHMNsSEQA | 0.19 | -0.149 | -0.869 | -0.276 |
| sp\|O95490\|AGRL2_HUMAN Adhesion G protein-coupled receptor L2 OS=Homo sapiens GN=ADGRL2 PE=1 SV | 631 | WKHMNSsEQAH | 0.211 | -0.101 | -0.628 | -0.173 |
| sp\|O95490\|AGRL2_HUMAN Adhesion G protein-coupled receptor L2 OS=Homo sapiens GN=ADGRL2 PE=1 SV | 636 | SSEQAHtATML | 0.358 | 0.044 | -0.664 | -0.087 |
| sp\|O95490\|AGRL2_HUMAN Adhesion G protein-coupled receptor L2 OS=Homo sapiens GN=ADGRL2 PE=1 SV | 638 | EQAHTAtMLLD | 0.107 | 0.092 | -0.788 | -0.196 |

| | | | | | | |
|---|---|---|---|---|---|---|
| sp\|O95490\|AGRL2_HUMAN Adhesion G protein-coupled receptor L2 OS=Homo sapiens GN=ADGRL2 PE=1 SV | 643 | ATMLLDtLEEG | 0.242 | -0.073 | -1.005 | -0.279 |
| sp\|O95490\|AGRL2_HUMAN Adhesion G protein-coupled receptor L2 OS=Homo sapiens GN=ADGRL2 PE=1 SV | 659 | DNLLEPtRVSM | 0.065 | -0.391 | -1.637 | -0.654 |
| sp\|O95490\|AGRL2_HUMAN Adhesion G protein-coupled receptor L2 OS=Homo sapiens GN=ADGRL2 PE=1 SV | 662 | LEPTRVsMPTE | 0.679 | 0.551 | -0.058 | 0.391 |
| sp\|O95490\|AGRL2_HUMAN Adhesion G protein-coupled receptor L2 OS=Homo sapiens GN=ADGRL2 PE=1 SV | 665 | TRVSMPtENIV | 0.265 | 0.08 | -0.786 | -0.147 |
| sp\|O95490\|AGRL2_HUMAN Adhesion G protein-coupled receptor L2 OS=Homo sapiens GN=ADGRL2 PE=1 SV | 676 | LEVAVLsTEGQ | 0.074 | -0.279 | -1.433 | -0.546 |
| sp\|O95490\|AGRL2_HUMAN Adhesion G protein-coupled receptor L2 OS=Homo sapiens GN=ADGRL2 PE=1 SV | 677 | EVAVLStEGQI | 0.11 | -0.031 | -0.73 | -0.217 |
| sp\|O95490\|AGRL2_HUMAN Adhesion G protein-coupled receptor L2 OS=Homo sapiens GN=ADGRL2 PE=1 SV | 695 | GIKGAGsSIQL | 0.261 | 0.089 | -0.449 | -0.033 |
| sp\|O95490\|AGRL2_HUMAN Adhesion G protein-coupled receptor L2 OS=Homo sapiens GN=ADGRL2 PE=1 SV | 696 | IKGAGSsIQLS | 0.09 | -0.198 | -1.003 | -0.37 |
| sp\|O95490\|AGRL2_HUMAN Adhesion G protein-coupled receptor L2 OS=Homo sapiens GN=ADGRL2 PE=1 SV | 700 | GSSIQLsANTV | 0.116 | -0.089 | -1.355 | -0.443 |
| sp\|O95490\|AGRL2_HUMAN Adhesion G protein-coupled receptor L2 OS=Homo sapiens GN=ADGRL2 PE=1 SV | 703 | IQLSANtVKQN | 0.335 | -0.002 | -0.37 | -0.012 |
| sp\|O95490\|AGRL2_HUMAN Adhesion G protein-coupled receptor L2 OS=Homo sapiens GN=ADGRL2 PE=1 SV | 708 | NTVKQNsRNGL | 0.329 | -0.02 | -0.704 | -0.132 |
| sp\|O95490\|AGRL2_HUMAN Adhesion G protein-coupled receptor L2 OS=Homo sapiens GN=ADGRL2 PE=1 SV | 722 | VFIIYRsLGQF | 0.32 | 0.091 | -0.37 | 0.014 |
| sp\|O95490\|AGRL2_HUMAN Adhesion G protein-coupled receptor L2 OS=Homo sapiens GN=ADGRL2 PE=1 SV | 728 | SLGQFLsTENA | 0.103 | -0.094 | -1.26 | -0.417 |
| sp\|O95490\|AGRL2_HUMAN Adhesion G protein-coupled receptor L2 OS=Homo sapiens GN=ADGRL2 PE=1 SV | 729 | LGQFLStENAT | 0.163 | 0.03 | -0.569 | -0.125 |
| sp\|O95490\|AGRL2_HUMAN Adhesion G protein-coupled receptor L2 OS=Homo sapiens GN=ADGRL2 PE=1 SV | 733 | LSTENAtIKLG | 0.125 | -0.132 | -0.79 | -0.266 |
| sp\|O95490\|AGRL2_HUMAN Adhesion G protein-coupled receptor L2 OS=Homo sapiens GN=ADGRL2 PE=1 SV | 745 | DFIGRNsTIAV | 0.468 | 0.257 | -0.39 | 0.112 |
| sp\|O95490\|AGRL2_HUMAN Adhesion G protein-coupled receptor L2 OS=Homo sapiens GN=ADGRL2 PE=1 SV | 746 | FIGRNStIAVN | 0.418 | 0.857 | 0.043 | 0.439 |
| sp\|O95490\|AGRL2_HUMAN Adhesion G protein-coupled receptor L2 OS=Homo sapiens GN=ADGRL2 PE=1 SV | 751 | STIAVNsHVIS | 0.215 | -0.104 | -1.24 | -0.376 |
| sp\|O95490\|AGRL2_HUMAN Adhesion G protein-coupled receptor L2 OS=Homo sapiens GN=ADGRL2 PE=1 SV | 755 | VNSHVIsVSIN | 0.131 | -0.255 | -1.11 | -0.411 |
| sp\|O95490\|AGRL2_HUMAN Adhesion G protein-coupled receptor L2 OS=Homo sapiens GN=ADGRL2 PE=1 SV | 757 | SHVISVsINKE | 0.214 | 0.052 | -0.86 | -0.198 |
| sp\|O95490\|AGRL2_HUMAN Adhesion G protein-coupled receptor L2 OS=Homo sapiens GN=ADGRL2 PE=1 SV | 762 | VSINKEsSRVY | 0.077 | -0.256 | -1.377 | -0.519 |
| sp\|O95490\|AGRL2_HUMAN Adhesion G protein-coupled receptor L2 OS=Homo sapiens GN=ADGRL2 PE=1 SV | 763 | SINKESsRVYL | 0.109 | -0.169 | -1.527 | -0.529 |
| sp\|O95490\|AGRL2_HUMAN Adhesion G protein-coupled receptor L2 OS=Homo sapiens GN=ADGRL2 PE=1 SV | 768 | SSRVYLtDPVL | 0.52 | 0.639 | -0.175 | 0.328 |
| sp\|O95490\|AGRL2_HUMAN Adhesion G protein-coupled receptor L2 OS=Homo sapiens GN=ADGRL2 PE=1 SV | 774 | TDPVLFtLPHI | 0.306 | 0.342 | -0.149 | 0.166 |
| sp\|O95490\|AGRL2_HUMAN Adhesion G protein-coupled receptor L2 OS=Homo sapiens GN=ADGRL2 PE=1 SV | 789 | YFNANCsFWNY | 0.61 | 0.172 | -0.359 | 0.141 |
| sp\|O95490\|AGRL2_HUMAN Adhesion G protein-coupled receptor L2 OS=Homo sapiens GN=ADGRL2 PE=1 SV | 794 | CSFWNYsERTM | 0.39 | -0.011 | -0.085 | 0.098 |
| sp\|O95490\|AGRL2_HUMAN Adhesion G protein-coupled receptor L2 OS=Homo sapiens GN=ADGRL2 PE=1 SV | 797 | WNYSERtMMGY | 0.1 | -0.173 | -1.346 | -0.473 |
| sp\|O95490\|AGRL2_HUMAN Adhesion G protein-coupled receptor L2 OS=Homo sapiens GN=ADGRL2 PE=1 SV | 803 | TMMGYWsTQGC | 0.108 | -0.075 | -1.062 | -0.343 |
| sp\|O95490\|AGRL2_HUMAN Adhesion G protein-coupled receptor L2 OS=Homo sapiens GN=ADGRL2 PE=1 SV | 804 | MMGYWStQGCK | 0.157 | 0.133 | -0.895 | -0.202 |
| sp\|O95490\|AGRL2_HUMAN Adhesion G protein-coupled receptor L2 OS=Homo sapiens GN=ADGRL2 PE=1 SV | 812 | GCKLVDtNKTR | 0.291 | -0.078 | -1.019 | -0.269 |
| sp\|O95490\|AGRL2_HUMAN Adhesion G protein-coupled receptor L2 OS=Homo sapiens GN=ADGRL2 PE=1 SV | 815 | LVDTNKtRTTC | 0.153 | -0.235 | -1.049 | -0.377 |
| sp\|O95490\|AGRL2_HUMAN Adhesion G protein-coupled receptor L2 OS=Homo sapiens GN=ADGRL2 PE=1 SV | 817 | DTNKTRtTCAC | 0.142 | -0.133 | -1.448 | -0.48 |
| sp\|O95490\|AGRL2_HUMAN Adhesion G protein-coupled receptor L2 OS=Homo sapiens GN=ADGRL2 PE=1 SV | 818 | TNKTRTtCACS | 0.212 | 0.06 | -0.755 | -0.161 |
| sp\|O95490\|AGRL2_HUMAN Adhesion G protein-coupled receptor L2 OS=Homo sapiens GN=ADGRL2 PE=1 SV | 822 | RTTCACsHLTN | 0.499 | 0.053 | -0.727 | -0.058 |
| sp\|O95490\|AGRL2_HUMAN Adhesion G protein-coupled receptor L2 OS=Homo sapiens GN=ADGRL2 PE=1 SV | 825 | CACSHLtNFAI | 0.289 | 0.009 | -0.584 | -0.095 |
| sp\|O95490\|AGRL2_HUMAN Adhesion G protein-coupled receptor L2 OS=Homo sapiens GN=ADGRL2 PE=1 SV | 848 | VHELLLtVITW | 0.055 | -0.194 | -1.535 | -0.558 |
| sp\|O95490\|AGRL2_HUMAN Adhesion G protein-coupled receptor L2 OS=Homo sapiens GN=ADGRL2 PE=1 SV | 851 | LLLTVItWVGI | 0.382 | 0.119 | -0.246 | 0.085 |
| sp\|O95490\|AGRL2_HUMAN Adhesion G protein-coupled receptor L2 OS=Homo sapiens GN=ADGRL2 PE=1 SV | 858 | WVGIVIsLVCL | 0.207 | 0.105 | -0.554 | -0.081 |
| sp\|O95490\|AGRL2_HUMAN Adhesion G protein-coupled receptor L2 OS=Homo sapiens GN=ADGRL2 PE=1 SV | 868 | LAICIFtFCFF | 0.113 | -0.149 | -0.918 | -0.318 |
| sp\|O95490\|AGRL2_HUMAN Adhesion G protein-coupled receptor L2 OS=Homo sapiens GN=ADGRL2 PE=1 SV | 877 | FFRGLQsDRNT | 0.386 | 0.252 | -0.434 | 0.068 |
| sp\|O95490\|AGRL2_HUMAN Adhesion G protein-coupled receptor L2 OS=Homo sapiens GN=ADGRL2 PE=1 SV | 881 | LQSDRNtIHKN | 0.272 | 0.09 | -0.668 | -0.102 |
| sp\|O95490\|AGRL2_HUMAN Adhesion G protein-coupled receptor L2 OS=Homo sapiens GN=ADGRL2 PE=1 SV | 904 | LIGIDKtKYAI | 0.305 | 0.181 | -0.09 | 0.132 |
| sp\|O95490\|AGRL2_HUMAN Adhesion G protein-coupled receptor L2 OS=Homo sapiens GN=ADGRL2 PE=1 SV | 945 | LVEVFEsEYSR | 0.067 | -0.107 | -1.271 | -0.437 |
| sp\|O95490\|AGRL2_HUMAN Adhesion G protein-coupled receptor L2 OS=Homo sapiens GN=ADGRL2 PE=1 SV | 948 | VFESEYsRKKY | 0.138 | -0.181 | -1.021 | -0.355 |
| sp\|O95490\|AGRL2_HUMAN Adhesion G protein-coupled receptor L2 OS=Homo sapiens GN=ADGRL2 PE=1 SV | 963 | GYLFPAtVVGV | 0.359 | 0.004 | -0.652 | -0.096 |
| sp\|O95490\|AGRL2_HUMAN Adhesion G protein-coupled receptor L2 OS=Homo sapiens GN=ADGRL2 PE=1 SV | 968 | ATVVGVsAAID | 0.162 | -0.179 | -1.206 | -0.408 |
| sp\|O95490\|AGRL2_HUMAN Adhesion G protein-coupled receptor L2 OS=Homo sapiens GN=ADGRL2 PE=1 SV | 975 | AAIDYKsYGTE | 0.27 | -0.074 | -0.775 | -0.193 |
| sp\|O95490\|AGRL2_HUMAN Adhesion G protein-coupled receptor L2 OS=Homo sapiens GN=ADGRL2 PE=1 SV | 978 | DYKSYGtEKAC | 0.259 | -0.038 | -0.83 | -0.203 |
| sp\|O95490\|AGRL2_HUMAN Adhesion G protein-coupled receptor L2 OS=Homo sapiens GN=ADGRL2 PE=1 SV | 993 | DNYFIWsFIGP | 0.16 | -0.184 | -0.648 | -0.224 |

| | | | | | | |
|---|---|---|---|---|---|---|
| sp\|O95490\|AGRL2_HUMAN Adhesion G protein-coupled receptor L2 OS=Homo sapiens GN=ADGRL2 PE=1 SV | 999 | SFIGPVtFIIL | 0.244 | -0.011 | -0.411 | -0.059 |
| sp\|O95490\|AGRL2_HUMAN Adhesion G protein-coupled receptor L2 OS=Homo sapiens GN=ADGRL2 PE=1 SV | 1012 | IIFLVItLCKM | 0.357 | 0.005 | -0.494 | -0.044 |
| sp\|O95490\|AGRL2_HUMAN Adhesion G protein-coupled receptor L2 OS=Homo sapiens GN=ADGRL2 PE=1 SV | 1020 | CKMVKHsNTLK | 0.3 | 0.081 | -0.673 | -0.097 |
| sp\|O95490\|AGRL2_HUMAN Adhesion G protein-coupled receptor L2 OS=Homo sapiens GN=ADGRL2 PE=1 SV | 1022 | MVKHSNtLKPD | 0.713 | 0.387 | 0.512 | 0.537 |
| sp\|O95490\|AGRL2_HUMAN Adhesion G protein-coupled receptor L2 OS=Homo sapiens GN=ADGRL2 PE=1 SV | 1027 | NTLKPDsSRLE | 0.207 | -0.286 | -0.998 | -0.359 |
| sp\|O95490\|AGRL2_HUMAN Adhesion G protein-coupled receptor L2 OS=Homo sapiens GN=ADGRL2 PE=1 SV | 1028 | TLKPDSsRLEN | 0.128 | -0.172 | -1.115 | -0.386 |
| sp\|O95490\|AGRL2_HUMAN Adhesion G protein-coupled receptor L2 OS=Homo sapiens GN=ADGRL2 PE=1 SV | 1035 | RLENIKsWVLG | 0.391 | 0.251 | 0.035 | 0.226 |
| sp\|O95490\|AGRL2_HUMAN Adhesion G protein-coupled receptor L2 OS=Homo sapiens GN=ADGRL2 PE=1 SV | 1050 | LCLLGLtWSFG | 0.05 | -0.163 | -1.52 | -0.544 |
| sp\|O95490\|AGRL2_HUMAN Adhesion G protein-coupled receptor L2 OS=Homo sapiens GN=ADGRL2 PE=1 SV | 1052 | LLGLTWsFGLL | 0.321 | 0.207 | -0.299 | 0.076 |
| sp\|O95490\|AGRL2_HUMAN Adhesion G protein-coupled receptor L2 OS=Homo sapiens GN=ADGRL2 PE=1 SV | 1062 | LFINEEtIVMA | 0.074 | -0.223 | -1.173 | -0.441 |
| sp\|O95490\|AGRL2_HUMAN Adhesion G protein-coupled receptor L2 OS=Homo sapiens GN=ADGRL2 PE=1 SV | 1070 | VMAYLFtIFNA | 0.111 | 0.047 | -0.777 | -0.206 |
| sp\|O95490\|AGRL2_HUMAN Adhesion G protein-coupled receptor L2 OS=Homo sapiens GN=ADGRL2 PE=1 SV | 1102 | GKCFRHsYCCG | 0.643 | 0.316 | -0.236 | 0.241 |
| sp\|O95490\|AGRL2_HUMAN Adhesion G protein-coupled receptor L2 OS=Homo sapiens GN=ADGRL2 PE=1 SV | 1110 | CCGGLPtESPH | 0.064 | -0.253 | -1.514 | -0.568 |
| sp\|O95490\|AGRL2_HUMAN Adhesion G protein-coupled receptor L2 OS=Homo sapiens GN=ADGRL2 PE=1 SV | 1112 | GGLPTEsPHSS | 0.036 | -0.435 | -1.692 | -0.697 |
| sp\|O95490\|AGRL2_HUMAN Adhesion G protein-coupled receptor L2 OS=Homo sapiens GN=ADGRL2 PE=1 SV | 1115 | PTESPHsSVKA | 0.166 | -0.156 | -0.986 | -0.325 |
| sp\|O95490\|AGRL2_HUMAN Adhesion G protein-coupled receptor L2 OS=Homo sapiens GN=ADGRL2 PE=1 SV | 1116 | TESPHSsVKAS | 0.092 | -0.157 | -1.12 | -0.395 |
| sp\|O95490\|AGRL2_HUMAN Adhesion G protein-coupled receptor L2 OS=Homo sapiens GN=ADGRL2 PE=1 SV | 1120 | HSSVKAsTTRT | 0.095 | -0.274 | -1.342 | -0.507 |
| sp\|O95490\|AGRL2_HUMAN Adhesion G protein-coupled receptor L2 OS=Homo sapiens GN=ADGRL2 PE=1 SV | 1121 | SSVKAStTRTS | 0.141 | -0.229 | -1.16 | -0.416 |
| sp\|O95490\|AGRL2_HUMAN Adhesion G protein-coupled receptor L2 OS=Homo sapiens GN=ADGRL2 PE=1 SV | 1122 | SVKASTtRTSA | 0.192 | -0.038 | -0.729 | -0.192 |
| sp\|O95490\|AGRL2_HUMAN Adhesion G protein-coupled receptor L2 OS=Homo sapiens GN=ADGRL2 PE=1 SV | 1124 | KASTTRtSARY | 0.063 | -0.294 | -1.805 | -0.679 |
| sp\|O95490\|AGRL2_HUMAN Adhesion G protein-coupled receptor L2 OS=Homo sapiens GN=ADGRL2 PE=1 SV | 1125 | ASTTRTsARYS | 0.26 | -0.077 | -0.907 | -0.241 |
| sp\|O95490\|AGRL2_HUMAN Adhesion G protein-coupled receptor L2 OS=Homo sapiens GN=ADGRL2 PE=1 SV | 1129 | RTSARYsSGTQ | 0.401 | 0.087 | -0.481 | 0.002 |
| sp\|O95490\|AGRL2_HUMAN Adhesion G protein-coupled receptor L2 OS=Homo sapiens GN=ADGRL2 PE=1 SV | 1130 | TSARYSsGTQS | 0.405 | 0.575 | -0.042 | 0.313 |
| sp\|O95490\|AGRL2_HUMAN Adhesion G protein-coupled receptor L2 OS=Homo sapiens GN=ADGRL2 PE=1 SV | 1132 | ARYSSGtQSRI | 0.198 | 0.091 | -0.997 | -0.236 |
| sp\|O95490\|AGRL2_HUMAN Adhesion G protein-coupled receptor L2 OS=Homo sapiens GN=ADGRL2 PE=1 SV | 1134 | YSSGTQsRIRR | 0.081 | -0.236 | -1.117 | -0.424 |
| sp\|O95490\|AGRL2_HUMAN Adhesion G protein-coupled receptor L2 OS=Homo sapiens GN=ADGRL2 PE=1 SV | 1143 | RRMWNDtVRKQ | 0.26 | 0.043 | -0.706 | -0.134 |
| sp\|O95490\|AGRL2_HUMAN Adhesion G protein-coupled receptor L2 OS=Homo sapiens GN=ADGRL2 PE=1 SV | 1148 | DTVRKQsESSF | 0.293 | 0.709 | -0.37 | 0.211 |
| sp\|O95490\|AGRL2_HUMAN Adhesion G protein-coupled receptor L2 OS=Homo sapiens GN=ADGRL2 PE=1 SV | 1150 | VRKQSEsSFIS | 0.45 | 0.308 | -0.184 | 0.191 |
| sp\|O95490\|AGRL2_HUMAN Adhesion G protein-coupled receptor L2 OS=Homo sapiens GN=ADGRL2 PE=1 SV | 1151 | RKQSESsFISG | 0.188 | 0.002 | -0.783 | -0.198 |
| sp\|O95490\|AGRL2_HUMAN Adhesion G protein-coupled receptor L2 OS=Homo sapiens GN=ADGRL2 PE=1 SV | 1154 | SESSFIsGDIN | 0.131 | -0.233 | -1.327 | -0.476 |
| sp\|O95490\|AGRL2_HUMAN Adhesion G protein-coupled receptor L2 OS=Homo sapiens GN=ADGRL2 PE=1 SV | 1159 | ISGDINsTSTL | 0.139 | -0.134 | -0.992 | -0.329 |
| sp\|O95490\|AGRL2_HUMAN Adhesion G protein-coupled receptor L2 OS=Homo sapiens GN=ADGRL2 PE=1 SV | 1160 | SGDINStSTLN | 0.073 | -0.226 | -1.419 | -0.524 |
| sp\|O95490\|AGRL2_HUMAN Adhesion G protein-coupled receptor L2 OS=Homo sapiens GN=ADGRL2 PE=1 SV | 1161 | GDINSTsTLNQ | 0.157 | 0.094 | -0.899 | -0.216 |
| sp\|O95490\|AGRL2_HUMAN Adhesion G protein-coupled receptor L2 OS=Homo sapiens GN=ADGRL2 PE=1 SV | 1162 | DINSTStLNQG | 0.259 | 0.024 | -0.622 | -0.113 |
| sp\|O95490\|AGRL2_HUMAN Adhesion G protein-coupled receptor L2 OS=Homo sapiens GN=ADGRL2 PE=1 SV | 1168 | TLNQGMtGNYL | 0.245 | 0.07 | -0.503 | -0.063 |
| sp\|O95490\|AGRL2_HUMAN Adhesion G protein-coupled receptor L2 OS=Homo sapiens GN=ADGRL2 PE=1 SV | 1174 | TGNYLLtNPLL | 0.247 | 0.354 | -0.543 | 0.019 |
| sp\|O95490\|AGRL2_HUMAN Adhesion G protein-coupled receptor L2 OS=Homo sapiens GN=ADGRL2 PE=1 SV | 1183 | LLRPHGtNNPY | 0.462 | 0.189 | -0.398 | 0.084 |
| sp\|O95490\|AGRL2_HUMAN Adhesion G protein-coupled receptor L2 OS=Homo sapiens GN=ADGRL2 PE=1 SV | 1189 | TNNPYNtLLAE | 0.247 | -0.057 | -0.681 | -0.164 |
| sp\|O95490\|AGRL2_HUMAN Adhesion G protein-coupled receptor L2 OS=Homo sapiens GN=ADGRL2 PE=1 SV | 1194 | NTLLAEtVVCN | 0.244 | 0.052 | -0.924 | -0.209 |
| sp\|O95490\|AGRL2_HUMAN Adhesion G protein-coupled receptor L2 OS=Homo sapiens GN=ADGRL2 PE=1 SV | 1201 | VVCNAPsAPVF | 0.48 | 0.489 | -0.083 | 0.295 |
| sp\|O95490\|AGRL2_HUMAN Adhesion G protein-coupled receptor L2 OS=Homo sapiens GN=ADGRL2 PE=1 SV | 1207 | SAPVFNsPGHS | 0.033 | -0.533 | -1.985 | -0.828 |
| sp\|O95490\|AGRL2_HUMAN Adhesion G protein-coupled receptor L2 OS=Homo sapiens GN=ADGRL2 PE=1 SV | 1211 | FNSPGHsLNNA | 0.309 | 0.081 | -0.381 | 0.003 |
| sp\|O95490\|AGRL2_HUMAN Adhesion G protein-coupled receptor L2 OS=Homo sapiens GN=ADGRL2 PE=1 SV | 1218 | LNNARDtSAMD | 0.139 | -0.242 | -1.173 | -0.425 |
| sp\|O95490\|AGRL2_HUMAN Adhesion G protein-coupled receptor L2 OS=Homo sapiens GN=ADGRL2 PE=1 SV | 1219 | NNARDTsAMDT | 0.57 | 0.778 | 0.345 | 0.564 |
| sp\|O95490\|AGRL2_HUMAN Adhesion G protein-coupled receptor L2 OS=Homo sapiens GN=ADGRL2 PE=1 SV | 1223 | DTSAMDtLPLN | 0.744 | 0.36 | 0.006 | 0.37 |
| sp\|O95490\|AGRL2_HUMAN Adhesion G protein-coupled receptor L2 OS=Homo sapiens GN=ADGRL2 PE=1 SV | 1233 | NGNFNNsYSLH | 0.114 | -0.207 | -1.283 | -0.459 |
| sp\|O95490\|AGRL2_HUMAN Adhesion G protein-coupled receptor L2 OS=Homo sapiens GN=ADGRL2 PE=1 SV | 1235 | NFNNSYsLHKG | 0.544 | 0.369 | 0.299 | 0.404 |
| sp\|O95490\|AGRL2_HUMAN Adhesion G protein-coupled receptor L2 OS=Homo sapiens GN=ADGRL2 PE=1 SV | 1244 | KGDYNDsVQVV | 0.183 | -0.111 | -0.986 | -0.305 |
| sp\|O95490\|AGRL2_HUMAN Adhesion G protein-coupled receptor L2 OS=Homo sapiens GN=ADGRL2 PE=1 SV | 1253 | VVDCGLsLNDT | 0.176 | -0.083 | -0.669 | -0.192 |

| | | | | | | |
|---|---|---|---|---|---|---|
| sp\|O95490\|AGRL2_HUMAN Adhesion G protein-coupled receptor L2 OS=Homo sapiens GN=ADGRL2 PE=1 SV | 1257 | GLSLNDtAFEK | 0.439 | 0.013 | -0.631 | -0.06 |
| sp\|O95490\|AGRL2_HUMAN Adhesion G protein-coupled receptor L2 OS=Homo sapiens GN=ADGRL2 PE=1 SV | 1265 | FEKMIIsELVH | 0.197 | -0.031 | -0.847 | -0.227 |
| sp\|O95490\|AGRL2_HUMAN Adhesion G protein-coupled receptor L2 OS=Homo sapiens GN=ADGRL2 PE=1 SV | 1275 | HNNLRGsSKTH | 0.069 | -0.217 | -1.428 | -0.525 |
| sp\|O95490\|AGRL2_HUMAN Adhesion G protein-coupled receptor L2 OS=Homo sapiens GN=ADGRL2 PE=1 SV | 1276 | NNLRGSsKTHN | 0.284 | 0.559 | -0.137 | 0.235 |
| sp\|O95490\|AGRL2_HUMAN Adhesion G protein-coupled receptor L2 OS=Homo sapiens GN=ADGRL2 PE=1 SV | 1278 | LRGSSKtHNLE | 0.474 | 0.401 | 0.134 | 0.336 |
| sp\|O95490\|AGRL2_HUMAN Adhesion G protein-coupled receptor L2 OS=Homo sapiens GN=ADGRL2 PE=1 SV | 1284 | THNLELtLPVK | 0.427 | 0.393 | -0.57 | 0.083 |
| sp\|O95490\|AGRL2_HUMAN Adhesion G protein-coupled receptor L2 OS=Homo sapiens GN=ADGRL2 PE=1 SV | 1294 | KPVIGGsSSED | 0.039 | -0.309 | -1.718 | -0.663 |
| sp\|O95490\|AGRL2_HUMAN Adhesion G protein-coupled receptor L2 OS=Homo sapiens GN=ADGRL2 PE=1 SV | 1295 | PVIGGSsSEDD | 0.09 | -0.089 | -1.061 | -0.353 |
| sp\|O95490\|AGRL2_HUMAN Adhesion G protein-coupled receptor L2 OS=Homo sapiens GN=ADGRL2 PE=1 SV | 1296 | VIGGSSsEDDA | 0.363 | 0.285 | -0.078 | 0.19 |
| sp\|O95490\|AGRL2_HUMAN Adhesion G protein-coupled receptor L2 OS=Homo sapiens GN=ADGRL2 PE=1 SV | 1306 | AIVADAsSLMH | 0.085 | -0.287 | -1.646 | -0.616 |
| sp\|O95490\|AGRL2_HUMAN Adhesion G protein-coupled receptor L2 OS=Homo sapiens GN=ADGRL2 PE=1 SV | 1307 | IVADASsLMHS | 0.234 | -0.007 | -0.476 | -0.083 |
| sp\|O95490\|AGRL2_HUMAN Adhesion G protein-coupled receptor L2 OS=Homo sapiens GN=ADGRL2 PE=1 SV | 1311 | ASSLMHsDNPG | 0.288 | -0.08 | -0.751 | -0.181 |
| sp\|O95490\|AGRL2_HUMAN Adhesion G protein-coupled receptor L2 OS=Homo sapiens GN=ADGRL2 PE=1 SV | 1332 | PLIPQRtHSLL | 0.129 | 0.03 | -1.082 | -0.308 |
| sp\|O95490\|AGRL2_HUMAN Adhesion G protein-coupled receptor L2 OS=Homo sapiens GN=ADGRL2 PE=1 SV | 1334 | IPQRTHsLLYQ | 0.697 | 1 | 0.409 | 0.702 |
| sp\|O95490\|AGRL2_HUMAN Adhesion G protein-coupled receptor L2 OS=Homo sapiens GN=ADGRL2 PE=1 SV | 1345 | PQKKVKsEGTD | 0.718 | 0.252 | 0.464 | 0.478 |
| sp\|O95490\|AGRL2_HUMAN Adhesion G protein-coupled receptor L2 OS=Homo sapiens GN=ADGRL2 PE=1 SV | 1348 | KVKSEGtDSYV | 0.079 | -0.274 | -1.812 | -0.669 |
| sp\|O95490\|AGRL2_HUMAN Adhesion G protein-coupled receptor L2 OS=Homo sapiens GN=ADGRL2 PE=1 SV | 1350 | KSEGTDsYVSQ | 0.036 | -0.351 | -1.725 | -0.68 |
| sp\|O95490\|AGRL2_HUMAN Adhesion G protein-coupled receptor L2 OS=Homo sapiens GN=ADGRL2 PE=1 SV | 1353 | GTDSYVsQLTA | 0.227 | -0.22 | -1.183 | -0.392 |
| sp\|O95490\|AGRL2_HUMAN Adhesion G protein-coupled receptor L2 OS=Homo sapiens GN=ADGRL2 PE=1 SV | 1356 | SYVSQLtAEAE | 0.084 | -0.109 | -1.512 | -0.512 |
| sp\|O95490\|AGRL2_HUMAN Adhesion G protein-coupled receptor L2 OS=Homo sapiens GN=ADGRL2 PE=1 SV | 1365 | AEDHLQsPNRD | 0.073 | -0.5 | -1.559 | -0.662 |
| sp\|O95490\|AGRL2_HUMAN Adhesion G protein-coupled receptor L2 OS=Homo sapiens GN=ADGRL2 PE=1 SV | 1370 | QSPNRDsLYTS | 0.191 | -0.061 | -0.73 | -0.2 |
| sp\|O95490\|AGRL2_HUMAN Adhesion G protein-coupled receptor L2 OS=Homo sapiens GN=ADGRL2 PE=1 SV | 1373 | NRDSLYtSMPN | 0.104 | 0.058 | -1.096 | -0.311 |
| sp\|O95490\|AGRL2_HUMAN Adhesion G protein-coupled receptor L2 OS=Homo sapiens GN=ADGRL2 PE=1 SV | 1374 | RDSLYTsMPNL | 0.505 | 0.583 | -0.252 | 0.279 |
| sp\|O95490\|AGRL2_HUMAN Adhesion G protein-coupled receptor L2 OS=Homo sapiens GN=ADGRL2 PE=1 SV | 1381 | MPNLRDsPYPE | 0.106 | -0.317 | -1.463 | -0.558 |
| sp\|O95490\|AGRL2_HUMAN Adhesion G protein-coupled receptor L2 OS=Homo sapiens GN=ADGRL2 PE=1 SV | 1386 | DSPYPEsSPDM | 0.426 | 0.238 | -0.349 | 0.105 |
| sp\|O95490\|AGRL2_HUMAN Adhesion G protein-coupled receptor L2 OS=Homo sapiens GN=ADGRL2 PE=1 SV | 1387 | SPYPESsPDME | 0.023 | -0.661 | -2.127 | -0.922 |
| sp\|O95490\|AGRL2_HUMAN Adhesion G protein-coupled receptor L2 OS=Homo sapiens GN=ADGRL2 PE=1 SV | 1395 | DMEEDLsPSRR | 0.029 | -0.492 | -2.038 | -0.834 |
| sp\|O95490\|AGRL2_HUMAN Adhesion G protein-coupled receptor L2 OS=Homo sapiens GN=ADGRL2 PE=1 SV | 1397 | EEDLSPsRRSE | 0.103 | -0.184 | -1.238 | -0.44 |
| sp\|O95490\|AGRL2_HUMAN Adhesion G protein-coupled receptor L2 OS=Homo sapiens GN=ADGRL2 PE=1 SV | 1400 | LSPSRRsENED | 0.278 | 0.065 | -0.589 | -0.082 |
| sp\|O95490\|AGRL2_HUMAN Adhesion G protein-coupled receptor L2 OS=Homo sapiens GN=ADGRL2 PE=1 SV | 1409 | EDIYYKsMPNL | 0.512 | 0.682 | 0.118 | 0.437 |
| sp\|O95490\|AGRL2_HUMAN Adhesion G protein-coupled receptor L2 OS=Homo sapiens GN=ADGRL2 PE=1 SV | 1426 | QMCYQIsRGNS | 0.136 | 0.054 | -1.122 | -0.311 |
| sp\|O95490\|AGRL2_HUMAN Adhesion G protein-coupled receptor L2 OS=Homo sapiens GN=ADGRL2 PE=1 SV | 1430 | QISRGNsDGYI | 0.771 | 0.964 | 0.484 | 0.74 |
| sp\|O95490\|AGRL2_HUMAN Adhesion G protein-coupled receptor L2 OS=Homo sapiens GN=ADGRL2 PE=1 SV | 1457 | GQMQLVtSL-- | 0.168 | 0.056 | -0.864 | -0.213 |
| sp\|O95490\|AGRL2_HUMAN Adhesion G protein-coupled receptor L2 OS=Homo sapiens GN=ADGRL2 PE=1 SV | 1458 | QMQLVTsL--- | 0.308 | 0.349 | -0.462 | 0.065 |
| sp\|Q9HAR2\|AGRL3_HUMAN Adhesion G protein-coupled receptor L3 OS=Homo sapiens GN=ADGRL3 PE=1 SV | 325 | ILYVVKsVYED | 0.447 | 0.156 | -0.104 | 0.166 |
| sp\|Q9HAR2\|AGRL3_HUMAN Adhesion G protein-coupled receptor L3 OS=Homo sapiens GN=ADGRL3 PE=1 SV | 335 | DDDNEAtGNKI | 0.139 | -0.182 | -0.992 | -0.345 |
| sp\|Q9HAR2\|AGRL3_HUMAN Adhesion G protein-coupled receptor L3 OS=Homo sapiens GN=ADGRL3 PE=1 SV | 345 | IDYIYNtDQSK | 0.145 | -0.107 | -1.012 | -0.325 |
| sp\|Q9HAR2\|AGRL3_HUMAN Adhesion G protein-coupled receptor L3 OS=Homo sapiens GN=ADGRL3 PE=1 SV | 348 | IYNTDQsKDSL | 0.209 | -0.031 | -0.727 | -0.183 |
| sp\|Q9HAR2\|AGRL3_HUMAN Adhesion G protein-coupled receptor L3 OS=Homo sapiens GN=ADGRL3 PE=1 SV | 351 | TDQSKDsLVDV | 0.164 | -0.14 | -1.15 | -0.375 |
| sp\|Q9HAR2\|AGRL3_HUMAN Adhesion G protein-coupled receptor L3 OS=Homo sapiens GN=ADGRL3 PE=1 SV | 360 | DVPFPNsYQYI | 0.197 | -0.122 | -0.939 | -0.288 |
| sp\|Q9HAR2\|AGRL3_HUMAN Adhesion G protein-coupled receptor L3 OS=Homo sapiens GN=ADGRL3 PE=1 SV | 388 | YHVVKYsLDFG | 0.133 | 0.025 | -0.88 | -0.241 |
| sp\|Q9HAR2\|AGRL3_HUMAN Adhesion G protein-coupled receptor L3 OS=Homo sapiens GN=ADGRL3 PE=1 SV | 396 | DFGPLDsRSGQ | 0.052 | -0.474 | -1.798 | -0.74 |
| sp\|Q9HAR2\|AGRL3_HUMAN Adhesion G protein-coupled receptor L3 OS=Homo sapiens GN=ADGRL3 PE=1 SV | 398 | GPLDSRsGQAH | 0.262 | 0.089 | -0.729 | -0.126 |
| sp\|Q9HAR2\|AGRL3_HUMAN Adhesion G protein-coupled receptor L3 OS=Homo sapiens GN=ADGRL3 PE=1 SV | 407 | AHHGQVsYISP | 0.032 | -0.299 | -1.853 | -0.707 |
| sp\|Q9HAR2\|AGRL3_HUMAN Adhesion G protein-coupled receptor L3 OS=Homo sapiens GN=ADGRL3 PE=1 SV | 410 | GQVSYIsPPIH | 0.296 | 0.023 | -0.689 | -0.123 |
| sp\|Q9HAR2\|AGRL3_HUMAN Adhesion G protein-coupled receptor L3 OS=Homo sapiens GN=ADGRL3 PE=1 SV | 417 | PPIHLDsELER | 0.129 | -0.106 | -1.24 | -0.406 |
| sp\|Q9HAR2\|AGRL3_HUMAN Adhesion G protein-coupled receptor L3 OS=Homo sapiens GN=ADGRL3 PE=1 SV | 423 | SELERPsVKDI | 0.257 | 0.059 | -0.644 | -0.109 |
| sp\|Q9HAR2\|AGRL3_HUMAN Adhesion G protein-coupled receptor L3 OS=Homo sapiens GN=ADGRL3 PE=1 SV | 428 | PSVKDIsTTGP | 0.243 | -0.204 | -0.818 | -0.26 |
| sp\|Q9HAR2\|AGRL3_HUMAN Adhesion G protein-coupled receptor L3 OS=Homo sapiens GN=ADGRL3 PE=1 SV | 429 | SVKDIStTGPL | 0.103 | -0.1 | -0.956 | -0.318 |

| | | | | | | |
|---|---|---|---|---|---|---|
| sp\|Q9HAR2\|AGRL3_HUMAN Adhesion G protein-coupled receptor L3 OS=Homo sapiens GN=ADGRL3 PE=1 S\ | 430 | VKDISTtGPLG | 0.745 | 0.677 | 0.558 | 0.66 |
| sp\|Q9HAR2\|AGRL3_HUMAN Adhesion G protein-coupled receptor L3 OS=Homo sapiens GN=ADGRL3 PE=1 S\ | 437 | GPLGMGsTTTS | 0.121 | -0.267 | -1.243 | -0.463 |
| sp\|Q9HAR2\|AGRL3_HUMAN Adhesion G protein-coupled receptor L3 OS=Homo sapiens GN=ADGRL3 PE=1 S\ | 438 | PLGMGStTTST | 0.06 | -0.252 | -1.336 | -0.509 |
| sp\|Q9HAR2\|AGRL3_HUMAN Adhesion G protein-coupled receptor L3 OS=Homo sapiens GN=ADGRL3 PE=1 S\ | 439 | LGMGSTtTSTT | 0.148 | 0.034 | -0.529 | -0.116 |
| sp\|Q9HAR2\|AGRL3_HUMAN Adhesion G protein-coupled receptor L3 OS=Homo sapiens GN=ADGRL3 PE=1 S\ | 440 | GMGSTTtSTTL | 0.096 | 0.018 | -1.152 | -0.346 |
| sp\|Q9HAR2\|AGRL3_HUMAN Adhesion G protein-coupled receptor L3 OS=Homo sapiens GN=ADGRL3 PE=1 S\ | 441 | MGSTTTsTTLR | 0.208 | 0.001 | -0.71 | -0.167 |
| sp\|Q9HAR2\|AGRL3_HUMAN Adhesion G protein-coupled receptor L3 OS=Homo sapiens GN=ADGRL3 PE=1 S\ | 442 | GSTTTStTLRT | 0.071 | -0.285 | -1.541 | -0.585 |
| sp\|Q9HAR2\|AGRL3_HUMAN Adhesion G protein-coupled receptor L3 OS=Homo sapiens GN=ADGRL3 PE=1 S\ | 443 | STTTSTtLRTT | 0.412 | 0.051 | -0.247 | 0.072 |
| sp\|Q9HAR2\|AGRL3_HUMAN Adhesion G protein-coupled receptor L3 OS=Homo sapiens GN=ADGRL3 PE=1 S\ | 446 | TSTTLRtTTLS | 0.075 | -0.252 | -1.549 | -0.575 |
| sp\|Q9HAR2\|AGRL3_HUMAN Adhesion G protein-coupled receptor L3 OS=Homo sapiens GN=ADGRL3 PE=1 S\ | 447 | STTLRTtTLSP | 0.08 | -0.118 | -1.394 | -0.477 |
| sp\|Q9HAR2\|AGRL3_HUMAN Adhesion G protein-coupled receptor L3 OS=Homo sapiens GN=ADGRL3 PE=1 S\ | 448 | TTLRTTtLSPG | 0.56 | 0.802 | 0.321 | 0.561 |
| sp\|Q9HAR2\|AGRL3_HUMAN Adhesion G protein-coupled receptor L3 OS=Homo sapiens GN=ADGRL3 PE=1 S\ | 450 | LRTTTLsPGRS | 0.063 | -0.193 | -1.348 | -0.493 |
| sp\|Q9HAR2\|AGRL3_HUMAN Adhesion G protein-coupled receptor L3 OS=Homo sapiens GN=ADGRL3 PE=1 S\ | 454 | TLSPGRsTTPS | 0.069 | -0.257 | -1.415 | -0.534 |
| sp\|Q9HAR2\|AGRL3_HUMAN Adhesion G protein-coupled receptor L3 OS=Homo sapiens GN=ADGRL3 PE=1 S\ | 455 | LSPGRStTPSV | 0.243 | 0.346 | -0.634 | -0.015 |
| sp\|Q9HAR2\|AGRL3_HUMAN Adhesion G protein-coupled receptor L3 OS=Homo sapiens GN=ADGRL3 PE=1 S\ | 456 | SPGRSTtPSVS | 0.211 | 0.587 | -0.474 | 0.108 |
| sp\|Q9HAR2\|AGRL3_HUMAN Adhesion G protein-coupled receptor L3 OS=Homo sapiens GN=ADGRL3 PE=1 S\ | 458 | GRSTTPsVSGR | 0.165 | 0.062 | -0.847 | -0.207 |
| sp\|Q9HAR2\|AGRL3_HUMAN Adhesion G protein-coupled receptor L3 OS=Homo sapiens GN=ADGRL3 PE=1 S\ | 460 | STTPSVsGRRN | 0.341 | -0.103 | -0.666 | -0.143 |
| sp\|Q9HAR2\|AGRL3_HUMAN Adhesion G protein-coupled receptor L3 OS=Homo sapiens GN=ADGRL3 PE=1 S\ | 466 | SGRRNRsTSTP | 0.438 | 0.823 | -0.105 | 0.385 |
| sp\|Q9HAR2\|AGRL3_HUMAN Adhesion G protein-coupled receptor L3 OS=Homo sapiens GN=ADGRL3 PE=1 S\ | 467 | GRRNRStSTPS | 0.414 | 0.418 | -0.333 | 0.166 |
| sp\|Q9HAR2\|AGRL3_HUMAN Adhesion G protein-coupled receptor L3 OS=Homo sapiens GN=ADGRL3 PE=1 S\ | 468 | RRNRSTsTPSP | 0.9 | 1.685 | 1.242 | 1.276 |
| sp\|Q9HAR2\|AGRL3_HUMAN Adhesion G protein-coupled receptor L3 OS=Homo sapiens GN=ADGRL3 PE=1 S\ | 469 | RNRSTStPSPA | 0.046 | -0.368 | -1.476 | -0.599 |
| sp\|Q9HAR2\|AGRL3_HUMAN Adhesion G protein-coupled receptor L3 OS=Homo sapiens GN=ADGRL3 PE=1 S\ | 471 | RSTSTPsPAVE | 0.048 | -0.5 | -1.632 | -0.695 |
| sp\|Q9HAR2\|AGRL3_HUMAN Adhesion G protein-coupled receptor L3 OS=Homo sapiens GN=ADGRL3 PE=1 S\ | 481 | EVLDDMtTHLP | 0.131 | 0.01 | -0.96 | -0.273 |
| sp\|Q9HAR2\|AGRL3_HUMAN Adhesion G protein-coupled receptor L3 OS=Homo sapiens GN=ADGRL3 PE=1 S\ | 482 | VLDDMTtHLPS | 0.155 | -0.159 | -1.042 | -0.349 |
| sp\|Q9HAR2\|AGRL3_HUMAN Adhesion G protein-coupled receptor L3 OS=Homo sapiens GN=ADGRL3 PE=1 S\ | 486 | MTTHLPsASSQ | 0.076 | -0.22 | -1.519 | -0.554 |
| sp\|Q9HAR2\|AGRL3_HUMAN Adhesion G protein-coupled receptor L3 OS=Homo sapiens GN=ADGRL3 PE=1 S\ | 488 | THLPSAsSQIP | 0.234 | 0.025 | -0.829 | -0.19 |
| sp\|Q9HAR2\|AGRL3_HUMAN Adhesion G protein-coupled receptor L3 OS=Homo sapiens GN=ADGRL3 PE=1 S\ | 489 | HLPSASsQIPA | 0.101 | -0.129 | -1.012 | -0.347 |
| sp\|Q9HAR2\|AGRL3_HUMAN Adhesion G protein-coupled receptor L3 OS=Homo sapiens GN=ADGRL3 PE=1 S\ | 497 | IPALEEsCEAV | 0.193 | -0.033 | -1.158 | -0.333 |
| sp\|Q9HAR2\|AGRL3_HUMAN Adhesion G protein-coupled receptor L3 OS=Homo sapiens GN=ADGRL3 PE=1 S\ | 511 | EIMWFKtRQGQ | 0.245 | -0.078 | -0.579 | -0.137 |
| sp\|Q9HAR2\|AGRL3_HUMAN Adhesion G protein-coupled receptor L3 OS=Homo sapiens GN=ADGRL3 PE=1 S\ | 525 | QPCPAGtIGVS | 0.124 | -0.141 | -1.077 | -0.365 |
| sp\|Q9HAR2\|AGRL3_HUMAN Adhesion G protein-coupled receptor L3 OS=Homo sapiens GN=ADGRL3 PE=1 S\ | 529 | AGTIGVsTYLC | 0.116 | -0.198 | -1.157 | -0.413 |
| sp\|Q9HAR2\|AGRL3_HUMAN Adhesion G protein-coupled receptor L3 OS=Homo sapiens GN=ADGRL3 PE=1 S\ | 530 | GTIGVStYLCL | 0.088 | -0.062 | -1.34 | -0.438 |
| sp\|Q9HAR2\|AGRL3_HUMAN Adhesion G protein-coupled receptor L3 OS=Homo sapiens GN=ADGRL3 PE=1 S\ | 548 | PQGPDLsNCSS | 0.02 | -0.407 | -1.887 | -0.758 |
| sp\|Q9HAR2\|AGRL3_HUMAN Adhesion G protein-coupled receptor L3 OS=Homo sapiens GN=ADGRL3 PE=1 S\ | 551 | PDLSNCsSPWV | 0.482 | 0.327 | -0.387 | 0.141 |
| sp\|Q9HAR2\|AGRL3_HUMAN Adhesion G protein-coupled receptor L3 OS=Homo sapiens GN=ADGRL3 PE=1 S\ | 552 | DLSNCSsPWVN | 0.209 | -0.222 | -1.004 | -0.339 |
| sp\|Q9HAR2\|AGRL3_HUMAN Adhesion G protein-coupled receptor L3 OS=Homo sapiens GN=ADGRL3 PE=1 S\ | 559 | PWVNHItQKLK | 0.203 | -0.045 | -0.857 | -0.233 |
| sp\|Q9HAR2\|AGRL3_HUMAN Adhesion G protein-coupled receptor L3 OS=Homo sapiens GN=ADGRL3 PE=1 S\ | 564 | ITQKLKsGETA | 0.358 | -0.047 | -0.421 | -0.037 |
| sp\|Q9HAR2\|AGRL3_HUMAN Adhesion G protein-coupled receptor L3 OS=Homo sapiens GN=ADGRL3 PE=1 S\ | 567 | KLKSGEtAANI | 0.217 | -0.029 | -1.008 | -0.273 |
| sp\|Q9HAR2\|AGRL3_HUMAN Adhesion G protein-coupled receptor L3 OS=Homo sapiens GN=ADGRL3 PE=1 S\ | 579 | RELAEQtRNHL | 0.162 | -0.157 | -0.951 | -0.315 |
| sp\|Q9HAR2\|AGRL3_HUMAN Adhesion G protein-coupled receptor L3 OS=Homo sapiens GN=ADGRL3 PE=1 S\ | 589 | LNAGDItYSVR | 0.067 | -0.253 | -1.034 | -0.407 |
| sp\|Q9HAR2\|AGRL3_HUMAN Adhesion G protein-coupled receptor L3 OS=Homo sapiens GN=ADGRL3 PE=1 S\ | 591 | AGDITYsVRAM | 0.149 | 0.012 | -0.585 | -0.141 |
| sp\|Q9HAR2\|AGRL3_HUMAN Adhesion G protein-coupled receptor L3 OS=Homo sapiens GN=ADGRL3 PE=1 S\ | 610 | VQLRNLtPGGK | 0.339 | 0.406 | 0.042 | 0.262 |
| sp\|Q9HAR2\|AGRL3_HUMAN Adhesion G protein-coupled receptor L3 OS=Homo sapiens GN=ADGRL3 PE=1 S\ | 616 | TPGGKDsAARS | 0.039 | -0.392 | -1.985 | -0.779 |
| sp\|Q9HAR2\|AGRL3_HUMAN Adhesion G protein-coupled receptor L3 OS=Homo sapiens GN=ADGRL3 PE=1 S\ | 620 | KDSAARsLNKA | 0.221 | -0.092 | -0.682 | -0.184 |
| sp\|Q9HAR2\|AGRL3_HUMAN Adhesion G protein-coupled receptor L3 OS=Homo sapiens GN=ADGRL3 PE=1 S\ | 628 | NKAMVEtVNNL | 0.391 | 0.076 | -0.432 | 0.012 |
| sp\|Q9HAR2\|AGRL3_HUMAN Adhesion G protein-coupled receptor L3 OS=Homo sapiens GN=ADGRL3 PE=1 S\ | 645 | NAWRDLtTSDQ | 0.317 | 0.758 | -0.273 | 0.267 |
| sp\|Q9HAR2\|AGRL3_HUMAN Adhesion G protein-coupled receptor L3 OS=Homo sapiens GN=ADGRL3 PE=1 S\ | 646 | AWRDLTtSDQL | 0.354 | 0.194 | -0.548 | 0 |
| sp\|Q9HAR2\|AGRL3_HUMAN Adhesion G protein-coupled receptor L3 OS=Homo sapiens GN=ADGRL3 PE=1 S\ | 647 | WRDLTTsDQLR | 0.224 | 0.229 | -0.614 | -0.054 |
| sp\|Q9HAR2\|AGRL3_HUMAN Adhesion G protein-coupled receptor L3 OS=Homo sapiens GN=ADGRL3 PE=1 S\ | 654 | DQLRAAtMLLH | 0.614 | 0.897 | 0.029 | 0.513 |

| | | | | | | |
|---|---|---|---|---|---|---|
| sp\|Q9HAR2\|AGRL3_HUMAN Adhesion G protein-coupled receptor L3 OS=Homo sapiens GN=ADGRL3 PE=1 SV | 659 | ATMLLHtVEES | 0.177 | 0.026 | -1.117 | -0.305 |
| sp\|Q9HAR2\|AGRL3_HUMAN Adhesion G protein-coupled receptor L3 OS=Homo sapiens GN=ADGRL3 PE=1 SV | 663 | LHTVEEsAFVL | 0.096 | -0.045 | -1.442 | -0.464 |
| sp\|Q9HAR2\|AGRL3_HUMAN Adhesion G protein-coupled receptor L3 OS=Homo sapiens GN=ADGRL3 PE=1 SV | 674 | ADNLLKtDIVR | 0.125 | -0.096 | -1.108 | -0.36 |
| sp\|Q9HAR2\|AGRL3_HUMAN Adhesion G protein-coupled receptor L3 OS=Homo sapiens GN=ADGRL3 PE=1 SV | 681 | DIVRENtDNIK | 0.837 | 0.932 | 0.309 | 0.693 |
| sp\|Q9HAR2\|AGRL3_HUMAN Adhesion G protein-coupled receptor L3 OS=Homo sapiens GN=ADGRL3 PE=1 SV | 692 | LEVARLsTEGN | 0.143 | -0.05 | -0.985 | -0.297 |
| sp\|Q9HAR2\|AGRL3_HUMAN Adhesion G protein-coupled receptor L3 OS=Homo sapiens GN=ADGRL3 PE=1 SV | 693 | EVARLStEGNL | 0.456 | 0.926 | 0.158 | 0.513 |
| sp\|Q9HAR2\|AGRL3_HUMAN Adhesion G protein-coupled receptor L3 OS=Homo sapiens GN=ADGRL3 PE=1 SV | 710 | ENMGHGsTIQL | 0.067 | -0.132 | -0.971 | -0.345 |
| sp\|Q9HAR2\|AGRL3_HUMAN Adhesion G protein-coupled receptor L3 OS=Homo sapiens GN=ADGRL3 PE=1 SV | 711 | NMGHGStIQLS | 0.034 | -0.155 | -1.464 | -0.528 |
| sp\|Q9HAR2\|AGRL3_HUMAN Adhesion G protein-coupled receptor L3 OS=Homo sapiens GN=ADGRL3 PE=1 SV | 715 | GSTIQLsANTL | 0.134 | -0.009 | -0.976 | -0.284 |
| sp\|Q9HAR2\|AGRL3_HUMAN Adhesion G protein-coupled receptor L3 OS=Homo sapiens GN=ADGRL3 PE=1 SV | 718 | IQLSANtLKQN | 0.511 | 0.08 | -0.018 | 0.191 |
| sp\|Q9HAR2\|AGRL3_HUMAN Adhesion G protein-coupled receptor L3 OS=Homo sapiens GN=ADGRL3 PE=1 SV | 743 | NLGPYLsTENA | 0.121 | -0.055 | -1.039 | -0.324 |
| sp\|Q9HAR2\|AGRL3_HUMAN Adhesion G protein-coupled receptor L3 OS=Homo sapiens GN=ADGRL3 PE=1 SV | 744 | LGPYLStENAS | 0.09 | -0.055 | -0.808 | -0.258 |
| sp\|Q9HAR2\|AGRL3_HUMAN Adhesion G protein-coupled receptor L3 OS=Homo sapiens GN=ADGRL3 PE=1 SV | 748 | LSTENAsMKLG | 0.154 | 0.018 | -0.784 | -0.204 |
| sp\|Q9HAR2\|AGRL3_HUMAN Adhesion G protein-coupled receptor L3 OS=Homo sapiens GN=ADGRL3 PE=1 SV | 753 | ASMKLGtEALS | 0.103 | -0.157 | -1.281 | -0.445 |
| sp\|Q9HAR2\|AGRL3_HUMAN Adhesion G protein-coupled receptor L3 OS=Homo sapiens GN=ADGRL3 PE=1 SV | 757 | LGTEALsTNHS | 0.086 | -0.125 | -0.876 | -0.305 |
| sp\|Q9HAR2\|AGRL3_HUMAN Adhesion G protein-coupled receptor L3 OS=Homo sapiens GN=ADGRL3 PE=1 SV | 758 | GTEALStNHSV | 0.096 | -0.251 | -1.722 | -0.626 |
| sp\|Q9HAR2\|AGRL3_HUMAN Adhesion G protein-coupled receptor L3 OS=Homo sapiens GN=ADGRL3 PE=1 SV | 761 | ALSTNHsVIVN | 0.385 | 0.166 | -0.408 | 0.048 |
| sp\|Q9HAR2\|AGRL3_HUMAN Adhesion G protein-coupled receptor L3 OS=Homo sapiens GN=ADGRL3 PE=1 SV | 766 | HSVIVNsPVIT | 0.083 | -0.328 | -1.358 | -0.534 |
| sp\|Q9HAR2\|AGRL3_HUMAN Adhesion G protein-coupled receptor L3 OS=Homo sapiens GN=ADGRL3 PE=1 SV | 770 | VNSPVItAAIN | 0.133 | -0.256 | -1.201 | -0.441 |
| sp\|Q9HAR2\|AGRL3_HUMAN Adhesion G protein-coupled receptor L3 OS=Homo sapiens GN=ADGRL3 PE=1 SV | 778 | AINKEFsNKVY | 0.311 | 0.051 | -0.926 | -0.188 |
| sp\|Q9HAR2\|AGRL3_HUMAN Adhesion G protein-coupled receptor L3 OS=Homo sapiens GN=ADGRL3 PE=1 SV | 790 | ADPVVFtVKHI | 0.062 | -0.226 | -1.152 | -0.439 |
| sp\|Q9HAR2\|AGRL3_HUMAN Adhesion G protein-coupled receptor L3 OS=Homo sapiens GN=ADGRL3 PE=1 SV | 797 | VKHIKQsEENF | 0.126 | 0.024 | -1.014 | -0.288 |
| sp\|Q9HAR2\|AGRL3_HUMAN Adhesion G protein-coupled receptor L3 OS=Homo sapiens GN=ADGRL3 PE=1 SV | 806 | NFNPNCsFWSY | 0.417 | 0.057 | -0.554 | -0.027 |
| sp\|Q9HAR2\|AGRL3_HUMAN Adhesion G protein-coupled receptor L3 OS=Homo sapiens GN=ADGRL3 PE=1 SV | 809 | PNCSFWsYSKR | 0.079 | -0.34 | -1.428 | -0.563 |
| sp\|Q9HAR2\|AGRL3_HUMAN Adhesion G protein-coupled receptor L3 OS=Homo sapiens GN=ADGRL3 PE=1 SV | 811 | CSFWSYsKRTM | 0.502 | 0.127 | 0.221 | 0.283 |
| sp\|Q9HAR2\|AGRL3_HUMAN Adhesion G protein-coupled receptor L3 OS=Homo sapiens GN=ADGRL3 PE=1 SV | 814 | WSYSKRtMTGY | 0.094 | -0.18 | -1.306 | -0.464 |
| sp\|Q9HAR2\|AGRL3_HUMAN Adhesion G protein-coupled receptor L3 OS=Homo sapiens GN=ADGRL3 PE=1 SV | 816 | YSKRTMtGYWS | 0.528 | 0.938 | 0.465 | 0.644 |
| sp\|Q9HAR2\|AGRL3_HUMAN Adhesion G protein-coupled receptor L3 OS=Homo sapiens GN=ADGRL3 PE=1 SV | 820 | TMTGYWsTQGC | 0.126 | -0.106 | -0.997 | -0.326 |
| sp\|Q9HAR2\|AGRL3_HUMAN Adhesion G protein-coupled receptor L3 OS=Homo sapiens GN=ADGRL3 PE=1 SV | 821 | MTGYWStQGCR | 0.241 | 0.065 | -0.796 | -0.163 |
| sp\|Q9HAR2\|AGRL3_HUMAN Adhesion G protein-coupled receptor L3 OS=Homo sapiens GN=ADGRL3 PE=1 SV | 828 | QGCRLLtTNKT | 0.304 | 0.653 | -0.177 | 0.26 |
| sp\|Q9HAR2\|AGRL3_HUMAN Adhesion G protein-coupled receptor L3 OS=Homo sapiens GN=ADGRL3 PE=1 SV | 829 | GCRLLTtNKTH | 0.26 | 0.047 | -1.097 | -0.263 |
| sp\|Q9HAR2\|AGRL3_HUMAN Adhesion G protein-coupled receptor L3 OS=Homo sapiens GN=ADGRL3 PE=1 SV | 832 | LLTTNKtHTTC | 0.458 | -0.002 | -0.306 | 0.05 |
| sp\|Q9HAR2\|AGRL3_HUMAN Adhesion G protein-coupled receptor L3 OS=Homo sapiens GN=ADGRL3 PE=1 SV | 834 | TTNKTHtTCSC | 0.1 | -0.125 | -1.465 | -0.497 |
| sp\|Q9HAR2\|AGRL3_HUMAN Adhesion G protein-coupled receptor L3 OS=Homo sapiens GN=ADGRL3 PE=1 SV | 835 | TNKTHTtCSCN | 0.173 | -0.002 | -0.939 | -0.256 |
| sp\|Q9HAR2\|AGRL3_HUMAN Adhesion G protein-coupled receptor L3 OS=Homo sapiens GN=ADGRL3 PE=1 SV | 837 | KTHTTCsCNHL | 0.143 | -0.018 | -1.109 | -0.328 |
| sp\|Q9HAR2\|AGRL3_HUMAN Adhesion G protein-coupled receptor L3 OS=Homo sapiens GN=ADGRL3 PE=1 SV | 842 | CSCNHLtNFAV | 0.214 | -0.01 | -0.827 | -0.208 |
| sp\|Q9HAR2\|AGRL3_HUMAN Adhesion G protein-coupled receptor L3 OS=Homo sapiens GN=ADGRL3 PE=1 SV | 856 | HVEVKHsDAVH | 0.082 | -0.049 | -1.296 | -0.421 |
| sp\|Q9HAR2\|AGRL3_HUMAN Adhesion G protein-coupled receptor L3 OS=Homo sapiens GN=ADGRL3 PE=1 SV | 868 | LLLDVItWVGI | 0.4 | 0.15 | -0.25 | 0.1 |
| sp\|Q9HAR2\|AGRL3_HUMAN Adhesion G protein-coupled receptor L3 OS=Homo sapiens GN=ADGRL3 PE=1 SV | 875 | WVGILLsLVCL | 0.082 | 0.02 | -0.965 | -0.288 |
| sp\|Q9HAR2\|AGRL3_HUMAN Adhesion G protein-coupled receptor L3 OS=Homo sapiens GN=ADGRL3 PE=1 SV | 885 | LLICIFtFCFF | 0.203 | -0.002 | -0.566 | -0.122 |
| sp\|Q9HAR2\|AGRL3_HUMAN Adhesion G protein-coupled receptor L3 OS=Homo sapiens GN=ADGRL3 PE=1 SV | 894 | FFRGLQsDRNT | 0.386 | 0.252 | -0.434 | 0.068 |
| sp\|Q9HAR2\|AGRL3_HUMAN Adhesion G protein-coupled receptor L3 OS=Homo sapiens GN=ADGRL3 PE=1 SV | 898 | LQSDRNtIHKN | 0.272 | 0.09 | -0.668 | -0.102 |
| sp\|Q9HAR2\|AGRL3_HUMAN Adhesion G protein-coupled receptor L3 OS=Homo sapiens GN=ADGRL3 PE=1 SV | 906 | HKNLCIsLFVA | 0.638 | 0.228 | 0.155 | 0.34 |
| sp\|Q9HAR2\|AGRL3_HUMAN Adhesion G protein-coupled receptor L3 OS=Homo sapiens GN=ADGRL3 PE=1 SV | 921 | LIGINRtDQPI | 0.195 | 0.058 | -0.717 | -0.155 |
| sp\|Q9HAR2\|AGRL3_HUMAN Adhesion G protein-coupled receptor L3 OS=Homo sapiens GN=ADGRL3 PE=1 SV | 943 | FFLAAFtWMFL | 0.315 | 0.213 | -0.3 | 0.076 |
| sp\|Q9HAR2\|AGRL3_HUMAN Adhesion G protein-coupled receptor L3 OS=Homo sapiens GN=ADGRL3 PE=1 SV | 962 | LVEVFEsEHSR | 0.085 | -0.094 | -1.224 | -0.411 |
| sp\|Q9HAR2\|AGRL3_HUMAN Adhesion G protein-coupled receptor L3 OS=Homo sapiens GN=ADGRL3 PE=1 SV | 965 | VFESEHsRRKY | 0.226 | -0.167 | -0.998 | -0.313 |
| sp\|Q9HAR2\|AGRL3_HUMAN Adhesion G protein-coupled receptor L3 OS=Homo sapiens GN=ADGRL3 PE=1 SV | 985 | ALIVAVsAAVD | 0.43 | 0.146 | -0.524 | 0.017 |
| sp\|Q9HAR2\|AGRL3_HUMAN Adhesion G protein-coupled receptor L3 OS=Homo sapiens GN=ADGRL3 PE=1 SV | 992 | AAVDYRsYGTD | 0.187 | -0.116 | -1.118 | -0.349 |

| | | | | | | |
|---|---|---|---|---|---|---|
| sp\|Q9HAR2\|AGRL3_HUMAN Adhesion G protein-coupled receptor L3 OS=Homo sapiens GN=ADGRL3 PE=1 SV | 995 | DYRSYGtDKVC | 0.329 | 0.09 | -0.972 | -0.184 |
| sp\|Q9HAR2\|AGRL3_HUMAN Adhesion G protein-coupled receptor L3 OS=Homo sapiens GN=ADGRL3 PE=1 SV | 1005 | CWLRLDtYFIW | 0.529 | 0.76 | 0.054 | 0.448 |
| sp\|Q9HAR2\|AGRL3_HUMAN Adhesion G protein-coupled receptor L3 OS=Homo sapiens GN=ADGRL3 PE=1 SV | 1010 | DTYFIWsFIGP | 0.222 | -0.12 | -0.62 | -0.173 |
| sp\|Q9HAR2\|AGRL3_HUMAN Adhesion G protein-coupled receptor L3 OS=Homo sapiens GN=ADGRL3 PE=1 SV | 1016 | SFIGPAtLIIM | 0.231 | -0.018 | -0.418 | -0.068 |
| sp\|Q9HAR2\|AGRL3_HUMAN Adhesion G protein-coupled receptor L3 OS=Homo sapiens GN=ADGRL3 PE=1 SV | 1037 | YKMFHHtAILK | 0.484 | 0.309 | -0.169 | 0.208 |
| sp\|Q9HAR2\|AGRL3_HUMAN Adhesion G protein-coupled receptor L3 OS=Homo sapiens GN=ADGRL3 PE=1 SV | 1044 | AILKPEsGCLD | 0.202 | -0.178 | -0.858 | -0.278 |
| sp\|Q9HAR2\|AGRL3_HUMAN Adhesion G protein-coupled receptor L3 OS=Homo sapiens GN=ADGRL3 PE=1 SV | 1052 | CLDNIKsWVIG | 0.397 | 0.209 | 0.027 | 0.211 |
| sp\|Q9HAR2\|AGRL3_HUMAN Adhesion G protein-coupled receptor L3 OS=Homo sapiens GN=ADGRL3 PE=1 SV | 1067 | LCLLGLtWAFG | 0.057 | -0.156 | -1.442 | -0.514 |
| sp\|Q9HAR2\|AGRL3_HUMAN Adhesion G protein-coupled receptor L3 OS=Homo sapiens GN=ADGRL3 PE=1 SV | 1078 | LMYINEsTVIM | 0.096 | -0.019 | -1.058 | -0.327 |
| sp\|Q9HAR2\|AGRL3_HUMAN Adhesion G protein-coupled receptor L3 OS=Homo sapiens GN=ADGRL3 PE=1 SV | 1079 | MYINEStVIMA | 0.117 | -0.07 | -0.92 | -0.291 |
| sp\|Q9HAR2\|AGRL3_HUMAN Adhesion G protein-coupled receptor L3 OS=Homo sapiens GN=ADGRL3 PE=1 SV | 1087 | IMAYLFtIFNS | 0.121 | 0.076 | -0.767 | -0.19 |
| sp\|Q9HAR2\|AGRL3_HUMAN Adhesion G protein-coupled receptor L3 OS=Homo sapiens GN=ADGRL3 PE=1 SV | 1091 | LFTIFNsLQGM | 0.463 | 0.09 | -0.058 | 0.165 |
| sp\|Q9HAR2\|AGRL3_HUMAN Adhesion G protein-coupled receptor L3 OS=Homo sapiens GN=ADGRL3 PE=1 SV | 1118 | YGKCLRtHCCS | 0.128 | -0.144 | -1.251 | -0.422 |
| sp\|Q9HAR2\|AGRL3_HUMAN Adhesion G protein-coupled receptor L3 OS=Homo sapiens GN=ADGRL3 PE=1 SV | 1122 | LRTHCCsGKST | 0.168 | 0.038 | -0.723 | -0.172 |
| sp\|Q9HAR2\|AGRL3_HUMAN Adhesion G protein-coupled receptor L3 OS=Homo sapiens GN=ADGRL3 PE=1 SV | 1125 | HCCSGKsTESS | 0.086 | -0.086 | -1.17 | -0.39 |
| sp\|Q9HAR2\|AGRL3_HUMAN Adhesion G protein-coupled receptor L3 OS=Homo sapiens GN=ADGRL3 PE=1 SV | 1126 | CCSGKStESSI | 0.089 | -0.085 | -1.055 | -0.35 |
| sp\|Q9HAR2\|AGRL3_HUMAN Adhesion G protein-coupled receptor L3 OS=Homo sapiens GN=ADGRL3 PE=1 SV | 1128 | SGKSTEsSIGS | 0.05 | -0.271 | -1.397 | -0.539 |
| sp\|Q9HAR2\|AGRL3_HUMAN Adhesion G protein-coupled receptor L3 OS=Homo sapiens GN=ADGRL3 PE=1 SV | 1129 | GKSTESsIGSG | 0.179 | -0.116 | -0.971 | -0.303 |
| sp\|Q9HAR2\|AGRL3_HUMAN Adhesion G protein-coupled receptor L3 OS=Homo sapiens GN=ADGRL3 PE=1 SV | 1132 | TESSIGsGKTS | 0.041 | -0.394 | -1.532 | -0.628 |
| sp\|Q9HAR2\|AGRL3_HUMAN Adhesion G protein-coupled receptor L3 OS=Homo sapiens GN=ADGRL3 PE=1 SV | 1135 | SIGSGKtSGSR | 0.068 | -0.135 | -1.179 | -0.415 |
| sp\|Q9HAR2\|AGRL3_HUMAN Adhesion G protein-coupled receptor L3 OS=Homo sapiens GN=ADGRL3 PE=1 SV | 1136 | IGSGKTsGSRT | 0.067 | -0.392 | -1.27 | -0.532 |
| sp\|Q9HAR2\|AGRL3_HUMAN Adhesion G protein-coupled receptor L3 OS=Homo sapiens GN=ADGRL3 PE=1 SV | 1138 | SGKTSGsRTPG | 0.215 | -0.114 | -0.903 | -0.267 |
| sp\|Q9HAR2\|AGRL3_HUMAN Adhesion G protein-coupled receptor L3 OS=Homo sapiens GN=ADGRL3 PE=1 SV | 1140 | KTSGSRtPGRY | 0.205 | -0.202 | -1.092 | -0.363 |
| sp\|Q9HAR2\|AGRL3_HUMAN Adhesion G protein-coupled receptor L3 OS=Homo sapiens GN=ADGRL3 PE=1 SV | 1145 | RTPGRYsTGSQ | 0.196 | 0.087 | -0.758 | -0.158 |
| sp\|Q9HAR2\|AGRL3_HUMAN Adhesion G protein-coupled receptor L3 OS=Homo sapiens GN=ADGRL3 PE=1 SV | 1146 | TPGRYStGSQS | 0.18 | 0.523 | -0.541 | 0.054 |
| sp\|Q9HAR2\|AGRL3_HUMAN Adhesion G protein-coupled receptor L3 OS=Homo sapiens GN=ADGRL3 PE=1 SV | 1148 | GRYSTGsQSRI | 0.102 | -0.02 | -1.389 | -0.436 |
| sp\|Q9HAR2\|AGRL3_HUMAN Adhesion G protein-coupled receptor L3 OS=Homo sapiens GN=ADGRL3 PE=1 SV | 1150 | YSTGSQsRIRR | 0.219 | -0.005 | -0.44 | -0.075 |
| sp\|Q9HAR2\|AGRL3_HUMAN Adhesion G protein-coupled receptor L3 OS=Homo sapiens GN=ADGRL3 PE=1 SV | 1159 | RRMWNDtVRKQ | 0.26 | 0.043 | -0.706 | -0.134 |
| sp\|Q9HAR2\|AGRL3_HUMAN Adhesion G protein-coupled receptor L3 OS=Homo sapiens GN=ADGRL3 PE=1 SV | 1164 | DTVRKQsESSF | 0.293 | 0.709 | -0.37 | 0.211 |
| sp\|Q9HAR2\|AGRL3_HUMAN Adhesion G protein-coupled receptor L3 OS=Homo sapiens GN=ADGRL3 PE=1 SV | 1166 | VRKQSEsSFIT | 0.447 | 0.284 | -0.118 | 0.204 |
| sp\|Q9HAR2\|AGRL3_HUMAN Adhesion G protein-coupled receptor L3 OS=Homo sapiens GN=ADGRL3 PE=1 SV | 1167 | RKQSESsFITG | 0.292 | 0.023 | -0.59 | -0.092 |
| sp\|Q9HAR2\|AGRL3_HUMAN Adhesion G protein-coupled receptor L3 OS=Homo sapiens GN=ADGRL3 PE=1 SV | 1170 | SESSFItGDIN | 0.131 | -0.233 | -1.327 | -0.476 |
| sp\|Q9HAR2\|AGRL3_HUMAN Adhesion G protein-coupled receptor L3 OS=Homo sapiens GN=ADGRL3 PE=1 SV | 1175 | ITGDINsSASL | 0.111 | -0.13 | -1.17 | -0.396 |
| sp\|Q9HAR2\|AGRL3_HUMAN Adhesion G protein-coupled receptor L3 OS=Homo sapiens GN=ADGRL3 PE=1 SV | 1176 | TGDINSsASLN | 0.07 | -0.166 | -1.362 | -0.486 |
| sp\|Q9HAR2\|AGRL3_HUMAN Adhesion G protein-coupled receptor L3 OS=Homo sapiens GN=ADGRL3 PE=1 SV | 1178 | DINSSAsLNRE | 0.659 | 0.226 | -0.12 | 0.255 |
| sp\|Q9HAR2\|AGRL3_HUMAN Adhesion G protein-coupled receptor L3 OS=Homo sapiens GN=ADGRL3 PE=1 SV | 1191 | LNNARDtSVMD | 0.153 | -0.186 | -1.1 | -0.378 |
| sp\|Q9HAR2\|AGRL3_HUMAN Adhesion G protein-coupled receptor L3 OS=Homo sapiens GN=ADGRL3 PE=1 SV | 1192 | NNARDTsVMDT | 0.559 | 0.754 | 0.454 | 0.589 |
| sp\|Q9HAR2\|AGRL3_HUMAN Adhesion G protein-coupled receptor L3 OS=Homo sapiens GN=ADGRL3 PE=1 SV | 1196 | DTSVMDtLPLN | 0.658 | 0.365 | -0.16 | 0.288 |
| sp\|Q9HAR2\|AGRL3_HUMAN Adhesion G protein-coupled receptor L3 OS=Homo sapiens GN=ADGRL3 PE=1 SV | 1206 | NGNHGNsYSIA | 0.094 | -0.249 | -1.262 | -0.472 |
| sp\|Q9HAR2\|AGRL3_HUMAN Adhesion G protein-coupled receptor L3 OS=Homo sapiens GN=ADGRL3 PE=1 SV | 1208 | NHGNSYsIASG | 0.096 | 0.081 | -0.855 | -0.226 |
| sp\|Q9HAR2\|AGRL3_HUMAN Adhesion G protein-coupled receptor L3 OS=Homo sapiens GN=ADGRL3 PE=1 SV | 1211 | NSYSIAsGEYL | 0.11 | -0.181 | -1.004 | -0.358 |
| sp\|Q9HAR2\|AGRL3_HUMAN Adhesion G protein-coupled receptor L3 OS=Homo sapiens GN=ADGRL3 PE=1 SV | 1216 | ASGEYLsNCVQ | 0.033 | -0.373 | -1.969 | -0.77 |
| sp\|Q9HAR2\|AGRL3_HUMAN Adhesion G protein-coupled receptor L3 OS=Homo sapiens GN=ADGRL3 PE=1 SV | 1231 | GYNHNEtALEK | 0.245 | -0.055 | -1.068 | -0.293 |
| sp\|Q9HAR2\|AGRL3_HUMAN Adhesion G protein-coupled receptor L3 OS=Homo sapiens GN=ADGRL3 PE=1 SV | 1242 | KILKELtSNYI | 0.274 | -0.01 | -0.671 | -0.136 |
| sp\|Q9HAR2\|AGRL3_HUMAN Adhesion G protein-coupled receptor L3 OS=Homo sapiens GN=ADGRL3 PE=1 SV | 1243 | ILKELTsNYIP | 0.166 | -0.078 | -0.942 | -0.285 |
| sp\|Q9HAR2\|AGRL3_HUMAN Adhesion G protein-coupled receptor L3 OS=Homo sapiens GN=ADGRL3 PE=1 SV | 1248 | TSNYIPsYLNN | 0.073 | -0.191 | -1.313 | -0.477 |
| sp\|Q9HAR2\|AGRL3_HUMAN Adhesion G protein-coupled receptor L3 OS=Homo sapiens GN=ADGRL3 PE=1 SV | 1256 | LNNHERsSEQN | 0.061 | -0.283 | -1.566 | -0.596 |
| sp\|Q9HAR2\|AGRL3_HUMAN Adhesion G protein-coupled receptor L3 OS=Homo sapiens GN=ADGRL3 PE=1 SV | 1257 | NNHERSsEQNR | 0.128 | 0.041 | -0.993 | -0.275 |
| sp\|Q9HAR2\|AGRL3_HUMAN Adhesion G protein-coupled receptor L3 OS=Homo sapiens GN=ADGRL3 PE=1 SV | 1273 | LVNNLGsGRED | 0.069 | -0.26 | -1.206 | -0.466 |

| | | | | | | |
|---|---|---|---|---|---|---|
| sp\|Q9HAR2\|AGRL3_HUMAN Adhesion G protein-coupled receptor L3 OS=Homo sapiens GN=ADGRL3 PE=1 SV | 1286 | IVLDDAtSFNH | 0.149 | -0.141 | -1.217 | -0.403 |
| sp\|Q9HAR2\|AGRL3_HUMAN Adhesion G protein-coupled receptor L3 OS=Homo sapiens GN=ADGRL3 PE=1 SV | 1287 | VLDDATsFNHE | 0.402 | 0.086 | -0.226 | 0.087 |
| sp\|Q9HAR2\|AGRL3_HUMAN Adhesion G protein-coupled receptor L3 OS=Homo sapiens GN=ADGRL3 PE=1 SV | 1293 | SFNHEEsLGLE | 0.246 | -0.074 | -0.885 | -0.238 |
| sp\|Q9HAR2\|AGRL3_HUMAN Adhesion G protein-coupled receptor L3 OS=Homo sapiens GN=ADGRL3 PE=1 SV | 1303 | ELIHEEsDAPL | 0.14 | -0.079 | -1.172 | -0.37 |
| sp\|Q9HAR2\|AGRL3_HUMAN Adhesion G protein-coupled receptor L3 OS=Homo sapiens GN=ADGRL3 PE=1 SV | 1314 | LPPRVYsTENH | 0.303 | 0.7 | -0.432 | 0.19 |
| sp\|Q9HAR2\|AGRL3_HUMAN Adhesion G protein-coupled receptor L3 OS=Homo sapiens GN=ADGRL3 PE=1 SV | 1315 | PPRVYStENHQ | 0.211 | 0.112 | -0.628 | -0.102 |
| sp\|Q9HAR2\|AGRL3_HUMAN Adhesion G protein-coupled receptor L3 OS=Homo sapiens GN=ADGRL3 PE=1 SV | 1324 | HQPHHYtRRRI | 0.173 | -0.106 | -0.668 | -0.2 |
| sp\|Q9HAR2\|AGRL3_HUMAN Adhesion G protein-coupled receptor L3 OS=Homo sapiens GN=ADGRL3 PE=1 SV | 1333 | RIPQDHsESFF | 0.141 | 0.036 | -0.918 | -0.247 |
| sp\|Q9HAR2\|AGRL3_HUMAN Adhesion G protein-coupled receptor L3 OS=Homo sapiens GN=ADGRL3 PE=1 SV | 1335 | PQDHSEsFFPL | 0.337 | 0.157 | -0.196 | 0.099 |
| sp\|Q9HAR2\|AGRL3_HUMAN Adhesion G protein-coupled receptor L3 OS=Homo sapiens GN=ADGRL3 PE=1 SV | 1341 | SFFPLLtNEHT | 0.031 | -0.365 | -1.709 | -0.681 |
| sp\|Q9HAR2\|AGRL3_HUMAN Adhesion G protein-coupled receptor L3 OS=Homo sapiens GN=ADGRL3 PE=1 SV | 1345 | LLTNEHtEDLQ | 0.378 | 0.232 | -0.233 | 0.126 |
| sp\|Q9HAR2\|AGRL3_HUMAN Adhesion G protein-coupled receptor L3 OS=Homo sapiens GN=ADGRL3 PE=1 SV | 1350 | HTEDLQsPHRD | 0.121 | -0.359 | -1.321 | -0.52 |
| sp\|Q9HAR2\|AGRL3_HUMAN Adhesion G protein-coupled receptor L3 OS=Homo sapiens GN=ADGRL3 PE=1 SV | 1355 | QSPHRDsLYTS | 0.216 | -0.077 | -0.781 | -0.214 |
| sp\|Q9HAR2\|AGRL3_HUMAN Adhesion G protein-coupled receptor L3 OS=Homo sapiens GN=ADGRL3 PE=1 SV | 1358 | HRDSLYtSMPT | 0.078 | 0.044 | -1.039 | -0.306 |
| sp\|Q9HAR2\|AGRL3_HUMAN Adhesion G protein-coupled receptor L3 OS=Homo sapiens GN=ADGRL3 PE=1 SV | 1359 | RDSLYTsMPTL | 0.483 | 0.516 | -0.17 | 0.276 |
| sp\|Q9HAR2\|AGRL3_HUMAN Adhesion G protein-coupled receptor L3 OS=Homo sapiens GN=ADGRL3 PE=1 SV | 1362 | LYTSMPtLAGV | 0.263 | -0.17 | -0.64 | -0.182 |
| sp\|Q9HAR2\|AGRL3_HUMAN Adhesion G protein-coupled receptor L3 OS=Homo sapiens GN=ADGRL3 PE=1 SV | 1369 | LAGVAAtESVT | 0.103 | -0.041 | -1.067 | -0.335 |
| sp\|Q9HAR2\|AGRL3_HUMAN Adhesion G protein-coupled receptor L3 OS=Homo sapiens GN=ADGRL3 PE=1 SV | 1371 | GVAATEsVTTS | 0.135 | -0.094 | -1.048 | -0.336 |
| sp\|Q9HAR2\|AGRL3_HUMAN Adhesion G protein-coupled receptor L3 OS=Homo sapiens GN=ADGRL3 PE=1 SV | 1373 | AATESVtTSTQ | 0.198 | -0.065 | -0.866 | -0.244 |
| sp\|Q9HAR2\|AGRL3_HUMAN Adhesion G protein-coupled receptor L3 OS=Homo sapiens GN=ADGRL3 PE=1 SV | 1374 | ATESVTtSTQT | 0.163 | -0.306 | -1.142 | -0.428 |
| sp\|Q9HAR2\|AGRL3_HUMAN Adhesion G protein-coupled receptor L3 OS=Homo sapiens GN=ADGRL3 PE=1 SV | 1375 | TESVTTsTQTE | 0.067 | -0.191 | -1.322 | -0.482 |
| sp\|Q9HAR2\|AGRL3_HUMAN Adhesion G protein-coupled receptor L3 OS=Homo sapiens GN=ADGRL3 PE=1 SV | 1376 | ESVTTStQTEP | 0.047 | -0.292 | -1.708 | -0.651 |
| sp\|Q9HAR2\|AGRL3_HUMAN Adhesion G protein-coupled receptor L3 OS=Homo sapiens GN=ADGRL3 PE=1 SV | 1378 | VTTSTQtEPPP | 0.31 | 0.352 | -0.305 | 0.119 |
| sp\|Q9HAR2\|AGRL3_HUMAN Adhesion G protein-coupled receptor L3 OS=Homo sapiens GN=ADGRL3 PE=1 SV | 1395 | EDVYYKsMPNL | 0.501 | 0.656 | 0.102 | 0.42 |
| sp\|Q9HAR2\|AGRL3_HUMAN Adhesion G protein-coupled receptor L3 OS=Homo sapiens GN=ADGRL3 PE=1 SV | 1401 | SMPNLGsRNHV | 0.024 | -0.306 | -1.965 | -0.749 |
| sp\|Q9HAR2\|AGRL3_HUMAN Adhesion G protein-coupled receptor L3 OS=Homo sapiens GN=ADGRL3 PE=1 SV | 1410 | HVHQLHtYYQL | 0.12 | 0.055 | -0.775 | -0.2 |
| sp\|Q9HAR2\|AGRL3_HUMAN Adhesion G protein-coupled receptor L3 OS=Homo sapiens GN=ADGRL3 PE=1 SV | 1418 | YQLGRGsSDGF | 0.136 | -0.047 | -0.624 | -0.178 |
| sp\|Q9HAR2\|AGRL3_HUMAN Adhesion G protein-coupled receptor L3 OS=Homo sapiens GN=ADGRL3 PE=1 SV | 1419 | QLGRGSsDGFI | 0.517 | 0.831 | 0.192 | 0.513 |
| sp\|Q9HAR2\|AGRL3_HUMAN Adhesion G protein-coupled receptor L3 OS=Homo sapiens GN=ADGRL3 PE=1 SV | 1431 | PPNKDGtPPEG | 0.381 | 0.101 | -0.619 | -0.046 |
| sp\|Q9HAR2\|AGRL3_HUMAN Adhesion G protein-coupled receptor L3 OS=Homo sapiens GN=ADGRL3 PE=1 SV | 1436 | GTPPEGsSKGP | 0.048 | -0.427 | -1.748 | -0.709 |
| sp\|Q9HAR2\|AGRL3_HUMAN Adhesion G protein-coupled receptor L3 OS=Homo sapiens GN=ADGRL3 PE=1 SV | 1437 | TPPEGSsKGPA | 0.033 | -0.371 | -1.567 | -0.635 |
| sp\|Q9HAR2\|AGRL3_HUMAN Adhesion G protein-coupled receptor L3 OS=Homo sapiens GN=ADGRL3 PE=1 SV | 1445 | GPAHLVtSL-- | 0.147 | -0.038 | -1.106 | -0.332 |
| sp\|Q9HAR2\|AGRL3_HUMAN Adhesion G protein-coupled receptor L3 OS=Homo sapiens GN=ADGRL3 PE=1 SV | 1446 | PAHLVTsL--- | 0.282 | 0.141 | -0.657 | -0.078 |
| sp\|P41180\|CASR_HUMAN Extracellular calcium-sensing receptor OS=Homo sapiens GN=CASR PE=1 SV=3 | 317 | FHVVGGtIGFA | 0.039 | -0.241 | -1.688 | -0.63 |
| sp\|P41180\|CASR_HUMAN Extracellular calcium-sensing receptor OS=Homo sapiens GN=CASR PE=1 SV=3 | 342 | KVHPRKsVHNG | 0.311 | 0.14 | -0.574 | -0.041 |
| sp\|P41180\|CASR_HUMAN Extracellular calcium-sensing receptor OS=Homo sapiens GN=CASR PE=1 SV=3 | 355 | KEFWEEtFNCH | 0.191 | -0.159 | -1.194 | -0.387 |
| sp\|P41180\|CASR_HUMAN Extracellular calcium-sensing receptor OS=Homo sapiens GN=CASR PE=1 SV=3 | 372 | GPLPVDtFLRG | 0.196 | -0.204 | -1.181 | -0.396 |
| sp\|P41180\|CASR_HUMAN Extracellular calcium-sensing receptor OS=Homo sapiens GN=CASR PE=1 SV=3 | 380 | LRGHEEsGDRF | 0.101 | -0.069 | -1.237 | -0.402 |
| sp\|P41180\|CASR_HUMAN Extracellular calcium-sensing receptor OS=Homo sapiens GN=CASR PE=1 SV=3 | 385 | ESGDRFsNSST | 0.057 | -0.136 | -1.413 | -0.497 |
| sp\|P41180\|CASR_HUMAN Extracellular calcium-sensing receptor OS=Homo sapiens GN=CASR PE=1 SV=3 | 387 | GDRFSNsSTAF | 0.671 | 0.455 | 0.083 | 0.403 |
| sp\|P41180\|CASR_HUMAN Extracellular calcium-sensing receptor OS=Homo sapiens GN=CASR PE=1 SV=3 | 388 | DRFSNSsTAFR | 0.112 | -0.079 | -1.199 | -0.389 |
| sp\|P41180\|CASR_HUMAN Extracellular calcium-sensing receptor OS=Homo sapiens GN=CASR PE=1 SV=3 | 389 | RFSNSStAFRP | 0.226 | 0.056 | -0.612 | -0.11 |
| sp\|P41180\|CASR_HUMAN Extracellular calcium-sensing receptor OS=Homo sapiens GN=CASR PE=1 SV=3 | 396 | AFRPLCtGDEN | 0.463 | 0.147 | -0.496 | 0.038 |
| sp\|P41180\|CASR_HUMAN Extracellular calcium-sensing receptor OS=Homo sapiens GN=CASR PE=1 SV=3 | 402 | TGDENIsSVET | 0.085 | -0.22 | -1.147 | -0.427 |
| sp\|P41180\|CASR_HUMAN Extracellular calcium-sensing receptor OS=Homo sapiens GN=CASR PE=1 SV=3 | 403 | GDENISsVETP | 0.053 | -0.266 | -1.14 | -0.451 |
| sp\|P41180\|CASR_HUMAN Extracellular calcium-sensing receptor OS=Homo sapiens GN=CASR PE=1 SV=3 | 406 | NISSVEtPYID | 0.123 | -0.361 | -1.329 | -0.522 |
| sp\|P41180\|CASR_HUMAN Extracellular calcium-sensing receptor OS=Homo sapiens GN=CASR PE=1 SV=3 | 412 | TPYIDYtHLRI | 0.12 | -0.096 | -0.99 | -0.322 |
| sp\|P41180\|CASR_HUMAN Extracellular calcium-sensing receptor OS=Homo sapiens GN=CASR PE=1 SV=3 | 417 | YTHLRIsYNVY | 0.326 | 0.106 | -0.624 | -0.064 |
| sp\|P41180\|CASR_HUMAN Extracellular calcium-sensing receptor OS=Homo sapiens GN=CASR PE=1 SV=3 | 426 | VYLAVYsIAHA | 0.133 | -0.172 | -0.726 | -0.255 |

| | | | | | | |
|---|---|---|---|---|---|---|
| sp\|P41180\|CASR_HUMAN Extracellular calcium-sensing receptor OS=Homo sapiens GN=CASR PE=1 SV=3 | 436 | ALQDIYtCLPG | 0.258 | 0.169 | -0.563 | -0.045 |
| sp\|P41180\|CASR_HUMAN Extracellular calcium-sensing receptor OS=Homo sapiens GN=CASR PE=1 SV=3 | 445 | PGRGLFtNGSC | 0.182 | 0.095 | -0.863 | -0.195 |
| sp\|P41180\|CASR_HUMAN Extracellular calcium-sensing receptor OS=Homo sapiens GN=CASR PE=1 SV=3 | 448 | GLFTNGsCADI | 0.494 | 0.161 | -0.336 | 0.106 |
| sp\|P41180\|CASR_HUMAN Extracellular calcium-sensing receptor OS=Homo sapiens GN=CASR PE=1 SV=3 | 470 | LRHLNFtNNMG | 0.186 | 0.112 | -0.765 | -0.156 |
| sp\|P41180\|CASR_HUMAN Extracellular calcium-sensing receptor OS=Homo sapiens GN=CASR PE=1 SV=3 | 478 | NMGEQVtFDEC | 0.171 | 0.082 | -0.995 | -0.247 |
| sp\|P41180\|CASR_HUMAN Extracellular calcium-sensing receptor OS=Homo sapiens GN=CASR PE=1 SV=3 | 490 | DLVGNYsIINW | 0.202 | 0.133 | -0.535 | -0.067 |
| sp\|P41180\|CASR_HUMAN Extracellular calcium-sensing receptor OS=Homo sapiens GN=CASR PE=1 SV=3 | 497 | IINWHLsPEDG | 0.188 | -0.231 | -0.903 | -0.315 |
| sp\|P41180\|CASR_HUMAN Extracellular calcium-sensing receptor OS=Homo sapiens GN=CASR PE=1 SV=3 | 502 | LSPEDGsIVFK | 0.024 | -0.482 | -1.898 | -0.785 |
| sp\|P41180\|CASR_HUMAN Extracellular calcium-sensing receptor OS=Homo sapiens GN=CASR PE=1 SV=3 | 531 | EEKILWsGFSR | 0.077 | -0.191 | -1.177 | -0.43 |
| sp\|P41180\|CASR_HUMAN Extracellular calcium-sensing receptor OS=Homo sapiens GN=CASR PE=1 SV=3 | 534 | ILWSGFsREVP | 0.098 | -0.046 | -1.068 | -0.339 |
| sp\|P41180\|CASR_HUMAN Extracellular calcium-sensing receptor OS=Homo sapiens GN=CASR PE=1 SV=3 | 540 | SREVPFsNCSR | 0.044 | -0.132 | -1.473 | -0.52 |
| sp\|P41180\|CASR_HUMAN Extracellular calcium-sensing receptor OS=Homo sapiens GN=CASR PE=1 SV=3 | 543 | VPFSNCsRDCL | 0.212 | -0.096 | -1.002 | -0.295 |
| sp\|P41180\|CASR_HUMAN Extracellular calcium-sensing receptor OS=Homo sapiens GN=CASR PE=1 SV=3 | 550 | RDCLAGtRKGI | 0.17 | -0.18 | -0.988 | -0.333 |
| sp\|P41180\|CASR_HUMAN Extracellular calcium-sensing receptor OS=Homo sapiens GN=CASR PE=1 SV=3 | 560 | IIEGEPtCCFE | 0.123 | -0.217 | -1.211 | -0.435 |
| sp\|P41180\|CASR_HUMAN Extracellular calcium-sensing receptor OS=Homo sapiens GN=CASR PE=1 SV=3 | 574 | CPDGEYsDETD | 0.199 | -0.014 | -0.783 | -0.199 |
| sp\|P41180\|CASR_HUMAN Extracellular calcium-sensing receptor OS=Homo sapiens GN=CASR PE=1 SV=3 | 577 | GEYSDEtDASA | 0.079 | -0.265 | -1.624 | -0.603 |
| sp\|P41180\|CASR_HUMAN Extracellular calcium-sensing receptor OS=Homo sapiens GN=CASR PE=1 SV=3 | 580 | SDETDAsACNK | 0.086 | -0.296 | -1.697 | -0.636 |
| sp\|P41180\|CASR_HUMAN Extracellular calcium-sensing receptor OS=Homo sapiens GN=CASR PE=1 SV=3 | 591 | CPDDFWsNENH | 0.106 | -0.255 | -1.739 | -0.629 |
| sp\|P41180\|CASR_HUMAN Extracellular calcium-sensing receptor OS=Homo sapiens GN=CASR PE=1 SV=3 | 596 | WSNENHtSCIA | 0.124 | -0.204 | -1.113 | -0.398 |
| sp\|P41180\|CASR_HUMAN Extracellular calcium-sensing receptor OS=Homo sapiens GN=CASR PE=1 SV=3 | 597 | SNENHTsCIAK | 0.14 | -0.052 | -0.667 | -0.193 |
| sp\|P41180\|CASR_HUMAN Extracellular calcium-sensing receptor OS=Homo sapiens GN=CASR PE=1 SV=3 | 607 | KEIEFLsWTEP | 0.06 | -0.138 | -1.674 | -0.584 |
| sp\|P41180\|CASR_HUMAN Extracellular calcium-sensing receptor OS=Homo sapiens GN=CASR PE=1 SV=3 | 609 | IEFLSWtEPFG | 0.767 | 0.641 | 0.329 | 0.579 |
| sp\|P41180\|CASR_HUMAN Extracellular calcium-sensing receptor OS=Homo sapiens GN=CASR PE=1 SV=3 | 617 | PFGIALtLFAV | 0.401 | 0.219 | -0.202 | 0.139 |
| sp\|P41180\|CASR_HUMAN Extracellular calcium-sensing receptor OS=Homo sapiens GN=CASR PE=1 SV=3 | 627 | VLGIFLtAFVL | 0.143 | 0.071 | -0.994 | -0.26 |
| sp\|P41180\|CASR_HUMAN Extracellular calcium-sensing receptor OS=Homo sapiens GN=CASR PE=1 SV=3 | 640 | FIKFRNtPIVK | 0.379 | 0.08 | -0.414 | 0.015 |
| sp\|P41180\|CASR_HUMAN Extracellular calcium-sensing receptor OS=Homo sapiens GN=CASR PE=1 SV=3 | 646 | TPIVKAtNREL | 0.098 | -0.17 | -1.368 | -0.48 |
| sp\|P41180\|CASR_HUMAN Extracellular calcium-sensing receptor OS=Homo sapiens GN=CASR PE=1 SV=3 | 651 | ATNRELsYLLL | 0.288 | 0.66 | -0.583 | 0.122 |
| sp\|P41180\|CASR_HUMAN Extracellular calcium-sensing receptor OS=Homo sapiens GN=CASR PE=1 SV=3 | 657 | SYLLLFsLLCC | 0.136 | -0.015 | -1.165 | -0.348 |
| sp\|P41180\|CASR_HUMAN Extracellular calcium-sensing receptor OS=Homo sapiens GN=CASR PE=1 SV=3 | 663 | SLLCCFsSSLF | 0.19 | -0.044 | -0.897 | -0.25 |
| sp\|P41180\|CASR_HUMAN Extracellular calcium-sensing receptor OS=Homo sapiens GN=CASR PE=1 SV=3 | 664 | LLCCFSsSLFF | 0.08 | -0.204 | -1.448 | -0.524 |
| sp\|P41180\|CASR_HUMAN Extracellular calcium-sensing receptor OS=Homo sapiens GN=CASR PE=1 SV=3 | 665 | LCCFSSsLFFI | 0.56 | 0.285 | 0.085 | 0.31 |
| sp\|P41180\|CASR_HUMAN Extracellular calcium-sensing receptor OS=Homo sapiens GN=CASR PE=1 SV=3 | 676 | GEPQDWtCRLR | 0.232 | -0.105 | -0.888 | -0.254 |
| sp\|P41180\|CASR_HUMAN Extracellular calcium-sensing receptor OS=Homo sapiens GN=CASR PE=1 SV=3 | 687 | QPAFGIsFVLC | 0.124 | -0.125 | -1.003 | -0.335 |
| sp\|P41180\|CASR_HUMAN Extracellular calcium-sensing receptor OS=Homo sapiens GN=CASR PE=1 SV=3 | 693 | SFVLCIsCILV | 0.226 | 0.049 | -0.919 | -0.215 |
| sp\|P41180\|CASR_HUMAN Extracellular calcium-sensing receptor OS=Homo sapiens GN=CASR PE=1 SV=3 | 699 | SCILVKtNRVL | 0.234 | 0.003 | -1.039 | -0.267 |
| sp\|P41180\|CASR_HUMAN Extracellular calcium-sensing receptor OS=Homo sapiens GN=CASR PE=1 SV=3 | 712 | FEAKIPtSFHR | 0.227 | -0.064 | -0.486 | -0.108 |
| sp\|P41180\|CASR_HUMAN Extracellular calcium-sensing receptor OS=Homo sapiens GN=CASR PE=1 SV=3 | 713 | EAKIPTsFHRK | 0.215 | -0.158 | -0.7 | -0.214 |
| sp\|P41180\|CASR_HUMAN Extracellular calcium-sensing receptor OS=Homo sapiens GN=CASR PE=1 SV=3 | 732 | LLVFLCtFMQI | 0.536 | 0.164 | -0.215 | 0.162 |
| sp\|P41180\|CASR_HUMAN Extracellular calcium-sensing receptor OS=Homo sapiens GN=CASR PE=1 SV=3 | 745 | CVIWLYtAPPS | 0.454 | 0.493 | -0.072 | 0.292 |
| sp\|P41180\|CASR_HUMAN Extracellular calcium-sensing receptor OS=Homo sapiens GN=CASR PE=1 SV=3 | 749 | LYTAPPsSYRN | 0.133 | -0.256 | -1.054 | -0.392 |
| sp\|P41180\|CASR_HUMAN Extracellular calcium-sensing receptor OS=Homo sapiens GN=CASR PE=1 SV=3 | 750 | YTAPPSsYRNQ | 0.057 | -0.377 | -1.374 | -0.565 |
| sp\|P41180\|CASR_HUMAN Extracellular calcium-sensing receptor OS=Homo sapiens GN=CASR PE=1 SV=3 | 764 | DEIIFItCHEG | 0.338 | 0.067 | -1.014 | -0.203 |
| sp\|P41180\|CASR_HUMAN Extracellular calcium-sensing receptor OS=Homo sapiens GN=CASR PE=1 SV=3 | 769 | ITCHEGsLMAL | 0.391 | 0.015 | -0.492 | -0.029 |
| sp\|P41180\|CASR_HUMAN Extracellular calcium-sensing receptor OS=Homo sapiens GN=CASR PE=1 SV=3 | 780 | GFLIGYtCLLA | 0.153 | 0.099 | -0.682 | -0.143 |
| sp\|P41180\|CASR_HUMAN Extracellular calcium-sensing receptor OS=Homo sapiens GN=CASR PE=1 SV=3 | 794 | FFFAFKsRKLP | 0.21 | -0.056 | -0.703 | -0.183 |
| sp\|P41180\|CASR_HUMAN Extracellular calcium-sensing receptor OS=Homo sapiens GN=CASR PE=1 SV=3 | 808 | NEAKFItFSML | 0.305 | -0.032 | -0.658 | -0.128 |
| sp\|P41180\|CASR_HUMAN Extracellular calcium-sensing receptor OS=Homo sapiens GN=CASR PE=1 SV=3 | 810 | AKFITFsMLIF | 0.17 | 0.159 | -0.873 | -0.181 |
| sp\|P41180\|CASR_HUMAN Extracellular calcium-sensing receptor OS=Homo sapiens GN=CASR PE=1 SV=3 | 820 | FFIVWIsFIPA | 0.253 | 0.103 | -0.307 | 0.016 |
| sp\|P41180\|CASR_HUMAN Extracellular calcium-sensing receptor OS=Homo sapiens GN=CASR PE=1 SV=3 | 827 | FIPAYAsTYGK | 0.244 | -0.023 | -0.73 | -0.17 |

| | | | | | | |
|---|---|---|---|---|---|---|
| sp\|P41180\|CASR_HUMAN Extracellular calcium-sensing receptor OS=Homo sapiens GN=CASR PE=1 SV=3 | 828 | IPAYAStYGKF | 0.147 | -0.092 | -0.672 | -0.206 |
| sp\|P41180\|CASR_HUMAN Extracellular calcium-sensing receptor OS=Homo sapiens GN=CASR PE=1 SV=3 | 834 | TYGKFVsAVEV | 0.425 | 0.064 | -0.833 | -0.115 |
| sp\|P41180\|CASR_HUMAN Extracellular calcium-sensing receptor OS=Homo sapiens GN=CASR PE=1 SV=3 | 845 | IAILAAsFGLL | 0.563 | 0.229 | -0.111 | 0.227 |
| sp\|P41180\|CASR_HUMAN Extracellular calcium-sensing receptor OS=Homo sapiens GN=CASR PE=1 SV=3 | 865 | IILFKPsRNTI | 0.278 | -0.079 | -0.471 | -0.091 |
| sp\|P41180\|CASR_HUMAN Extracellular calcium-sensing receptor OS=Homo sapiens GN=CASR PE=1 SV=3 | 868 | FKPSRNtIEEV | 0.451 | 0.178 | -0.622 | 0.002 |
| sp\|P41180\|CASR_HUMAN Extracellular calcium-sensing receptor OS=Homo sapiens GN=CASR PE=1 SV=3 | 875 | IEEVRCsTAAH | 0.246 | -0.062 | -0.978 | -0.265 |
| sp\|P41180\|CASR_HUMAN Extracellular calcium-sensing receptor OS=Homo sapiens GN=CASR PE=1 SV=3 | 876 | EEVRCStAAHA | 0.353 | 0.66 | -0.212 | 0.267 |
| sp\|P41180\|CASR_HUMAN Extracellular calcium-sensing receptor OS=Homo sapiens GN=CASR PE=1 SV=3 | 888 | KVAARAtLRRS | 0.461 | 0.028 | -0.353 | 0.045 |
| sp\|P41180\|CASR_HUMAN Extracellular calcium-sensing receptor OS=Homo sapiens GN=CASR PE=1 SV=3 | 892 | RATLRRsNVSR | 0.143 | 0.02 | -1.173 | -0.337 |
| sp\|P41180\|CASR_HUMAN Extracellular calcium-sensing receptor OS=Homo sapiens GN=CASR PE=1 SV=3 | 895 | LRRSNVsRKRS | 0.414 | 0.17 | -0.474 | 0.037 |
| sp\|P41180\|CASR_HUMAN Extracellular calcium-sensing receptor OS=Homo sapiens GN=CASR PE=1 SV=3 | 899 | NVSRKRsSSLG | 0.227 | 0.669 | -0.487 | 0.136 |
| sp\|P41180\|CASR_HUMAN Extracellular calcium-sensing receptor OS=Homo sapiens GN=CASR PE=1 SV=3 | 900 | VSRKRSsSLGG | 0.398 | 0.148 | -0.423 | 0.041 |
| sp\|P41180\|CASR_HUMAN Extracellular calcium-sensing receptor OS=Homo sapiens GN=CASR PE=1 SV=3 | 901 | SRKRSSsLGGS | 0.854 | 1.275 | 1.174 | 1.101 |
| sp\|P41180\|CASR_HUMAN Extracellular calcium-sensing receptor OS=Homo sapiens GN=CASR PE=1 SV=3 | 905 | SSSLGGsTGST | 0.032 | -0.41 | -1.838 | -0.739 |
| sp\|P41180\|CASR_HUMAN Extracellular calcium-sensing receptor OS=Homo sapiens GN=CASR PE=1 SV=3 | 906 | SSLGGStGSTP | 0.017 | -0.514 | -1.945 | -0.814 |
| sp\|P41180\|CASR_HUMAN Extracellular calcium-sensing receptor OS=Homo sapiens GN=CASR PE=1 SV=3 | 908 | LGGSTGsTPSS | 0.099 | 0.207 | -0.93 | -0.208 |
| sp\|P41180\|CASR_HUMAN Extracellular calcium-sensing receptor OS=Homo sapiens GN=CASR PE=1 SV=3 | 909 | GGSTGStPSSS | 0.018 | -0.637 | -2.162 | -0.927 |
| sp\|P41180\|CASR_HUMAN Extracellular calcium-sensing receptor OS=Homo sapiens GN=CASR PE=1 SV=3 | 911 | STGSTPsSSIS | 0.032 | -0.371 | -1.975 | -0.771 |
| sp\|P41180\|CASR_HUMAN Extracellular calcium-sensing receptor OS=Homo sapiens GN=CASR PE=1 SV=3 | 912 | TGSTPSsSISS | 0.029 | -0.412 | -1.628 | -0.67 |
| sp\|P41180\|CASR_HUMAN Extracellular calcium-sensing receptor OS=Homo sapiens GN=CASR PE=1 SV=3 | 913 | GSTPSSsISSK | 0.084 | -0.157 | -0.989 | -0.354 |
| sp\|P41180\|CASR_HUMAN Extracellular calcium-sensing receptor OS=Homo sapiens GN=CASR PE=1 SV=3 | 915 | TPSSSIsSKSN | 0.119 | -0.072 | -0.989 | -0.314 |
| sp\|P41180\|CASR_HUMAN Extracellular calcium-sensing receptor OS=Homo sapiens GN=CASR PE=1 SV=3 | 916 | PSSSISsKSNS | 0.056 | -0.292 | -1.397 | -0.544 |
| sp\|P41180\|CASR_HUMAN Extracellular calcium-sensing receptor OS=Homo sapiens GN=CASR PE=1 SV=3 | 918 | SSISSKsNSED | 0.243 | 0.032 | -0.706 | -0.144 |
| sp\|P41180\|CASR_HUMAN Extracellular calcium-sensing receptor OS=Homo sapiens GN=CASR PE=1 SV=3 | 920 | ISSKSNsEDPF | 0.616 | 0.234 | 0.148 | 0.333 |
| sp\|P41180\|CASR_HUMAN Extracellular calcium-sensing receptor OS=Homo sapiens GN=CASR PE=1 SV=3 | 939 | QQPLALtQQEQ | 0.092 | -0.174 | -1.306 | -0.463 |
| sp\|P41180\|CASR_HUMAN Extracellular calcium-sensing receptor OS=Homo sapiens GN=CASR PE=1 SV=3 | 949 | QQQQPLtLPQQ | 0.51 | 0.386 | 0.313 | 0.403 |
| sp\|P41180\|CASR_HUMAN Extracellular calcium-sensing receptor OS=Homo sapiens GN=CASR PE=1 SV=3 | 956 | LPQQQRsQQQP | 0.078 | -0.183 | -1.39 | -0.498 |
| sp\|P41180\|CASR_HUMAN Extracellular calcium-sensing receptor OS=Homo sapiens GN=CASR PE=1 SV=3 | 970 | QKVIFGsGTVT | 0.13 | -0.158 | -1.158 | -0.395 |
| sp\|P41180\|CASR_HUMAN Extracellular calcium-sensing receptor OS=Homo sapiens GN=CASR PE=1 SV=3 | 972 | VIFGSGtVTFS | 0.201 | 0.057 | -0.793 | -0.178 |
| sp\|P41180\|CASR_HUMAN Extracellular calcium-sensing receptor OS=Homo sapiens GN=CASR PE=1 SV=3 | 974 | FGSGTVtFSLS | 0.172 | 0.032 | -0.652 | -0.149 |
| sp\|P41180\|CASR_HUMAN Extracellular calcium-sensing receptor OS=Homo sapiens GN=CASR PE=1 SV=3 | 976 | SGTVTFsLSFD | 0.083 | -0.076 | -0.922 | -0.305 |
| sp\|P41180\|CASR_HUMAN Extracellular calcium-sensing receptor OS=Homo sapiens GN=CASR PE=1 SV=3 | 978 | TVTFSLsFDEP | 0.324 | 0.22 | -0.18 | 0.121 |
| sp\|P41180\|CASR_HUMAN Extracellular calcium-sensing receptor OS=Homo sapiens GN=CASR PE=1 SV=3 | 992 | AMAHRNsTHQN | 0.393 | 0.211 | -0.487 | 0.039 |
| sp\|P41180\|CASR_HUMAN Extracellular calcium-sensing receptor OS=Homo sapiens GN=CASR PE=1 SV=3 | 993 | MAHRNStHQNS | 0.536 | 0.808 | -0.027 | 0.439 |
| sp\|P41180\|CASR_HUMAN Extracellular calcium-sensing receptor OS=Homo sapiens GN=CASR PE=1 SV=3 | 997 | NSTHQNsLEAQ | 0.3 | 0.039 | -0.504 | -0.055 |
| sp\|P41180\|CASR_HUMAN Extracellular calcium-sensing receptor OS=Homo sapiens GN=CASR PE=1 SV=3 | 1003 | SLEAQKsSDTL | 0.25 | 0.02 | -0.59 | -0.107 |
| sp\|P41180\|CASR_HUMAN Extracellular calcium-sensing receptor OS=Homo sapiens GN=CASR PE=1 SV=3 | 1004 | LEAQKSsDTLT | 0.116 | -0.201 | -1.095 | -0.393 |
| sp\|P41180\|CASR_HUMAN Extracellular calcium-sensing receptor OS=Homo sapiens GN=CASR PE=1 SV=3 | 1006 | AQKSSDtLTRH | 0.4 | -0.086 | -0.605 | -0.097 |
| sp\|P41180\|CASR_HUMAN Extracellular calcium-sensing receptor OS=Homo sapiens GN=CASR PE=1 SV=3 | 1008 | KSSDTLtRHEP | 0.057 | -0.281 | -1.602 | -0.609 |
| sp\|P41180\|CASR_HUMAN Extracellular calcium-sensing receptor OS=Homo sapiens GN=CASR PE=1 SV=3 | 1021 | PLQCGEtDLDL | 0.367 | 0.129 | -0.742 | -0.082 |
| sp\|P41180\|CASR_HUMAN Extracellular calcium-sensing receptor OS=Homo sapiens GN=CASR PE=1 SV=3 | 1026 | ETDLDLtVQET | 0.088 | -0.193 | -1.225 | -0.443 |
| sp\|P41180\|CASR_HUMAN Extracellular calcium-sensing receptor OS=Homo sapiens GN=CASR PE=1 SV=3 | 1030 | DLTVQEtGLQG | 0.112 | -0.134 | -1.26 | -0.427 |
| sp\|P41180\|CASR_HUMAN Extracellular calcium-sensing receptor OS=Homo sapiens GN=CASR PE=1 SV=3 | 1051 | EDPEELsPALV | 0.01 | -0.648 | -2.527 | -1.055 |
| sp\|P41180\|CASR_HUMAN Extracellular calcium-sensing receptor OS=Homo sapiens GN=CASR PE=1 SV=3 | 1057 | SPALVVsSSQS | 0.089 | -0.292 | -1.41 | -0.538 |
| sp\|P41180\|CASR_HUMAN Extracellular calcium-sensing receptor OS=Homo sapiens GN=CASR PE=1 SV=3 | 1058 | PALVVSsSQSF | 0.04 | -0.266 | -1.514 | -0.58 |
| sp\|P41180\|CASR_HUMAN Extracellular calcium-sensing receptor OS=Homo sapiens GN=CASR PE=1 SV=3 | 1059 | ALVVSSsQSFV | 0.097 | -0.046 | -1.454 | -0.468 |
| sp\|P41180\|CASR_HUMAN Extracellular calcium-sensing receptor OS=Homo sapiens GN=CASR PE=1 SV=3 | 1061 | VVSSSQsFVIS | 0.28 | 0.12 | -0.421 | -0.007 |
| sp\|P41180\|CASR_HUMAN Extracellular calcium-sensing receptor OS=Homo sapiens GN=CASR PE=1 SV=3 | 1065 | SQSFVIsGGGS | 0.137 | -0.202 | -0.948 | -0.338 |
| sp\|P41180\|CASR_HUMAN Extracellular calcium-sensing receptor OS=Homo sapiens GN=CASR PE=1 SV=3 | 1069 | VISGGGsTVTE | 0.056 | -0.265 | -1.44 | -0.55 |

| | | | | | | |
|---|---|---|---|---|---|---|
| sp\|P41180\|CASR_HUMAN Extracellular calcium-sensing receptor OS=Homo sapiens GN=CASR PE=1 SV=3 | 1070 | ISGGGStVTEN | 0.087 | -0.228 | -1.191 | -0.444 |
| sp\|P41180\|CASR_HUMAN Extracellular calcium-sensing receptor OS=Homo sapiens GN=CASR PE=1 SV=3 | 1072 | GGGSTVtENVV | 0.271 | 0.081 | -0.715 | -0.121 |
| sp\|P41180\|CASR_HUMAN Extracellular calcium-sensing receptor OS=Homo sapiens GN=CASR PE=1 SV=3 | 1078 | TENVVNs---- | 0.141 | 0.046 | -1.309 | -0.374 |
| sp\|Q5T6X5\|GPC6A_HUMAN G-protein coupled receptor family C group 6 member A OS=Homo sapiens GN=( | 334 | FRRGNIsSFHS | 0.385 | 0.397 | -0.135 | 0.216 |
| sp\|Q5T6X5\|GPC6A_HUMAN G-protein coupled receptor family C group 6 member A OS=Homo sapiens GN=( | 335 | RRGNISsFHSF | 0.095 | 0.155 | -0.716 | -0.155 |
| sp\|Q5T6X5\|GPC6A_HUMAN G-protein coupled receptor family C group 6 member A OS=Homo sapiens GN=( | 338 | NISSFHsFLQN | 0.308 | 0.108 | -0.653 | -0.079 |
| sp\|Q5T6X5\|GPC6A_HUMAN G-protein coupled receptor family C group 6 member A OS=Homo sapiens GN=( | 348 | NLHLLPsDSHK | 0.087 | -0.224 | -1.37 | -0.502 |
| sp\|Q5T6X5\|GPC6A_HUMAN G-protein coupled receptor family C group 6 member A OS=Homo sapiens GN=( | 350 | HLLPSDsHKLL | 0.444 | 0.252 | -0.05 | 0.215 |
| sp\|Q5T6X5\|GPC6A_HUMAN G-protein coupled receptor family C group 6 member A OS=Homo sapiens GN=( | 362 | EYAMHLsACAY | 0.237 | 0.013 | -0.733 | -0.161 |
| sp\|Q5T6X5\|GPC6A_HUMAN G-protein coupled receptor family C group 6 member A OS=Homo sapiens GN=( | 370 | CAYVKDtDLSQ | 0.041 | -0.307 | -1.844 | -0.703 |
| sp\|Q5T6X5\|GPC6A_HUMAN G-protein coupled receptor family C group 6 member A OS=Homo sapiens GN=( | 373 | VKDTDLsQCIF | 0.091 | -0.313 | -1.496 | -0.573 |
| sp\|Q5T6X5\|GPC6A_HUMAN G-protein coupled receptor family C group 6 member A OS=Homo sapiens GN=( | 380 | QCIFNHsQRTL | 0.352 | 0.077 | -0.723 | -0.098 |
| sp\|Q5T6X5\|GPC6A_HUMAN G-protein coupled receptor family C group 6 member A OS=Homo sapiens GN=( | 383 | FNHSQRtLAYK | 0.071 | -0.215 | -1.42 | -0.521 |
| sp\|Q5T6X5\|GPC6A_HUMAN G-protein coupled receptor family C group 6 member A OS=Homo sapiens GN=( | 414 | EPGLIHsIQLA | 0.118 | -0.022 | -0.784 | -0.229 |
| sp\|Q5T6X5\|GPC6A_HUMAN G-protein coupled receptor family C group 6 member A OS=Homo sapiens GN=( | 454 | GVLKNVtFTDG | 0.801 | 0.206 | 0.32 | 0.442 |
| sp\|Q5T6X5\|GPC6A_HUMAN G-protein coupled receptor family C group 6 member A OS=Homo sapiens GN=( | 456 | LKNVTFtDGWN | 0.268 | 0.187 | -0.537 | -0.027 |
| sp\|Q5T6X5\|GPC6A_HUMAN G-protein coupled receptor family C group 6 member A OS=Homo sapiens GN=( | 461 | FTDGWNsFHFD | 0.426 | 0.129 | -0.455 | 0.033 |
| sp\|Q5T6X5\|GPC6A_HUMAN G-protein coupled receptor family C group 6 member A OS=Homo sapiens GN=( | 472 | AHGDLNtGYDV | 0.153 | -0.079 | -1.61 | -0.512 |
| sp\|Q5T6X5\|GPC6A_HUMAN G-protein coupled receptor family C group 6 member A OS=Homo sapiens GN=( | 487 | EINGHMtVTKM | 0.259 | 0.155 | -0.598 | -0.061 |
| sp\|Q5T6X5\|GPC6A_HUMAN G-protein coupled receptor family C group 6 member A OS=Homo sapiens GN=( | 489 | NGHMTVtKMAE | 0.226 | -0.065 | -0.599 | -0.146 |
| sp\|Q5T6X5\|GPC6A_HUMAN G-protein coupled receptor family C group 6 member A OS=Homo sapiens GN=( | 508 | IIPDQEtKNEF | 0.159 | -0.058 | -1.002 | -0.3 |
| sp\|Q5T6X5\|GPC6A_HUMAN G-protein coupled receptor family C group 6 member A OS=Homo sapiens GN=( | 520 | NLKQIQsKCSK | 0.225 | -0.074 | -0.367 | -0.072 |
| sp\|Q5T6X5\|GPC6A_HUMAN G-protein coupled receptor family C group 6 member A OS=Homo sapiens GN=( | 523 | QIQSKCsKECS | 0.137 | -0.012 | -1.127 | -0.334 |
| sp\|Q5T6X5\|GPC6A_HUMAN G-protein coupled receptor family C group 6 member A OS=Homo sapiens GN=( | 527 | KCSKECsPGQM | 0.307 | -0.298 | -1.023 | -0.338 |
| sp\|Q5T6X5\|GPC6A_HUMAN G-protein coupled receptor family C group 6 member A OS=Homo sapiens GN=( | 534 | PGQMKKtTRSQ | 0.133 | -0.183 | -0.851 | -0.3 |
| sp\|Q5T6X5\|GPC6A_HUMAN G-protein coupled receptor family C group 6 member A OS=Homo sapiens GN=( | 535 | GQMKKTtRSQH | 0.11 | -0.26 | -1.198 | -0.449 |
| sp\|Q5T6X5\|GPC6A_HUMAN G-protein coupled receptor family C group 6 member A OS=Homo sapiens GN=( | 537 | MKKTTRsQHIC | 0.301 | 0.046 | -1.006 | -0.22 |
| sp\|Q5T6X5\|GPC6A_HUMAN G-protein coupled receptor family C group 6 member A OS=Homo sapiens GN=( | 554 | CPENHYtNQTD | 0.301 | 0.041 | -0.364 | -0.007 |
| sp\|Q5T6X5\|GPC6A_HUMAN G-protein coupled receptor family C group 6 member A OS=Homo sapiens GN=( | 557 | NHYTNQtDMPH | 0.11 | -0.196 | -1.434 | -0.507 |
| sp\|Q5T6X5\|GPC6A_HUMAN G-protein coupled receptor family C group 6 member A OS=Homo sapiens GN=( | 569 | LLCNNKtHWAP | 0.524 | 0.187 | 0.141 | 0.284 |
| sp\|Q5T6X5\|GPC6A_HUMAN G-protein coupled receptor family C group 6 member A OS=Homo sapiens GN=( | 576 | HWAPVRsTMCF | 0.104 | -0.007 | -1.064 | -0.322 |
| sp\|Q5T6X5\|GPC6A_HUMAN G-protein coupled receptor family C group 6 member A OS=Homo sapiens GN=( | 577 | WAPVRStMCFE | 0.045 | -0.226 | -1.638 | -0.606 |
| sp\|Q5T6X5\|GPC6A_HUMAN G-protein coupled receptor family C group 6 member A OS=Homo sapiens GN=( | 592 | YLNWNDsLAIL | 0.486 | 0.027 | -0.238 | 0.092 |
| sp\|Q5T6X5\|GPC6A_HUMAN G-protein coupled receptor family C group 6 member A OS=Homo sapiens GN=( | 601 | ILLLILsLLGI | 0.409 | 0.093 | 0.028 | 0.177 |
| sp\|Q5T6X5\|GPC6A_HUMAN G-protein coupled receptor family C group 6 member A OS=Homo sapiens GN=( | 616 | VVGIIFtRNLN | 0.102 | -0.054 | -0.875 | -0.276 |
| sp\|Q5T6X5\|GPC6A_HUMAN G-protein coupled receptor family C group 6 member A OS=Homo sapiens GN=( | 621 | FTRNLNtPVVK | 0.201 | -0.044 | -0.954 | -0.266 |
| sp\|Q5T6X5\|GPC6A_HUMAN G-protein coupled receptor family C group 6 member A OS=Homo sapiens GN=( | 626 | NTPVVKsSGGL | 0.196 | -0.078 | -0.769 | -0.217 |
| sp\|Q5T6X5\|GPC6A_HUMAN G-protein coupled receptor family C group 6 member A OS=Homo sapiens GN=( | 627 | TPVVKSsGGLR | 0.051 | -0.241 | -1.397 | -0.529 |
| sp\|Q5T6X5\|GPC6A_HUMAN G-protein coupled receptor family C group 6 member A OS=Homo sapiens GN=( | 646 | HFLNFAsTSFF | 0.078 | -0.125 | -0.99 | -0.346 |
| sp\|Q5T6X5\|GPC6A_HUMAN G-protein coupled receptor family C group 6 member A OS=Homo sapiens GN=( | 647 | FLNFAStSFFI | 0.324 | 0.116 | -0.535 | -0.032 |
| sp\|Q5T6X5\|GPC6A_HUMAN G-protein coupled receptor family C group 6 member A OS=Homo sapiens GN=( | 648 | LNFASTsFFIG | 0.485 | 0.166 | 0.062 | 0.238 |
| sp\|Q5T6X5\|GPC6A_HUMAN G-protein coupled receptor family C group 6 member A OS=Homo sapiens GN=( | 658 | GEPQDFtCKTR | 0.19 | -0.029 | -0.828 | -0.222 |
| sp\|Q5T6X5\|GPC6A_HUMAN G-protein coupled receptor family C group 6 member A OS=Homo sapiens GN=( | 661 | QDFTCKtRQTM | 0.213 | -0.174 | -0.718 | -0.226 |
| sp\|Q5T6X5\|GPC6A_HUMAN G-protein coupled receptor family C group 6 member A OS=Homo sapiens GN=( | 664 | TCKTRQtMFGV | 0.483 | 0.239 | -0.493 | 0.076 |
| sp\|Q5T6X5\|GPC6A_HUMAN G-protein coupled receptor family C group 6 member A OS=Homo sapiens GN=( | 669 | QTMFGVsFTLC | 0.264 | -0.067 | -0.835 | -0.213 |
| sp\|Q5T6X5\|GPC6A_HUMAN G-protein coupled receptor family C group 6 member A OS=Homo sapiens GN=( | 671 | MFGVSFtLCIS | 0.415 | 0.263 | -0.095 | 0.194 |
| sp\|Q5T6X5\|GPC6A_HUMAN G-protein coupled receptor family C group 6 member A OS=Homo sapiens GN=( | 675 | SFTLCIsCILT | 0.207 | 0.008 | -0.641 | -0.142 |
| sp\|Q5T6X5\|GPC6A_HUMAN G-protein coupled receptor family C group 6 member A OS=Homo sapiens GN=( | 679 | CISCILtKSLK | 0.144 | -0.106 | -0.774 | -0.245 |
| sp\|Q5T6X5\|GPC6A_HUMAN G-protein coupled receptor family C group 6 member A OS=Homo sapiens GN=( | 681 | SCILTKsLKIL | 0.335 | 0.185 | -0.462 | 0.019 |

| | | | | | | |
|---|---|---|---|---|---|---|
| sp\|Q5T6X5\|GPC6A_HUMAN G-protein coupled receptor family C group 6 member A OS=Homo sapiens GN=G | 689 | KILLAFsFDPK | 0.406 | 0.136 | -0.203 | 0.113 |
| sp\|Q5T6X5\|GPC6A_HUMAN G-protein coupled receptor family C group 6 member A OS=Homo sapiens GN=G | 710 | PILIIFtCTGI | 0.421 | 0.202 | -0.056 | 0.189 |
| sp\|Q5T6X5\|GPC6A_HUMAN G-protein coupled receptor family C group 6 member A OS=Homo sapiens GN=G | 712 | LIIFTCtGIQV | 0.186 | 0.055 | -0.928 | -0.229 |
| sp\|Q5T6X5\|GPC6A_HUMAN G-protein coupled receptor family C group 6 member A OS=Homo sapiens GN=G | 720 | IQVVICtLWLI | 0.488 | 0.154 | -0.031 | 0.204 |
| sp\|Q5T6X5\|GPC6A_HUMAN G-protein coupled receptor family C group 6 member A OS=Homo sapiens GN=G | 729 | LIFAAPtVEVN | 0.268 | 0.086 | -0.608 | -0.085 |
| sp\|Q5T6X5\|GPC6A_HUMAN G-protein coupled receptor family C group 6 member A OS=Homo sapiens GN=G | 735 | TVEVNVsLPRV | 0.719 | 0.427 | 0.113 | 0.42 |
| sp\|Q5T6X5\|GPC6A_HUMAN G-protein coupled receptor family C group 6 member A OS=Homo sapiens GN=G | 748 | LECEEGsILAF | 0.043 | -0.235 | -1.628 | -0.607 |
| sp\|Q5T6X5\|GPC6A_HUMAN G-protein coupled receptor family C group 6 member A OS=Homo sapiens GN=G | 754 | SILAFGtMLGY | 0.107 | -0.087 | -1.399 | -0.46 |
| sp\|Q5T6X5\|GPC6A_HUMAN G-protein coupled receptor family C group 6 member A OS=Homo sapiens GN=G | 785 | NEAKFItFGML | 0.498 | 0.082 | -0.265 | 0.105 |
| sp\|Q5T6X5\|GPC6A_HUMAN G-protein coupled receptor family C group 6 member A OS=Homo sapiens GN=G | 797 | YFIAWItFIPI | 0.378 | 0.106 | 0.018 | 0.167 |
| sp\|Q5T6X5\|GPC6A_HUMAN G-protein coupled receptor family C group 6 member A OS=Homo sapiens GN=G | 804 | FIPIYAtTFGK | 0.26 | 0.027 | -0.675 | -0.129 |
| sp\|Q5T6X5\|GPC6A_HUMAN G-protein coupled receptor family C group 6 member A OS=Homo sapiens GN=G | 805 | IPIYATtFGKY | 0.507 | 0.19 | -0.09 | 0.202 |
| sp\|Q5T6X5\|GPC6A_HUMAN G-protein coupled receptor family C group 6 member A OS=Homo sapiens GN=G | 821 | IIVILIsNYGI | 0.219 | -0.022 | -0.722 | -0.175 |
| sp\|Q5T6X5\|GPC6A_HUMAN G-protein coupled receptor family C group 6 member A OS=Homo sapiens GN=G | 829 | YGILYCtFIPK | 0.239 | -0.024 | -0.621 | -0.135 |
| sp\|Q5T6X5\|GPC6A_HUMAN G-protein coupled receptor family C group 6 member A OS=Homo sapiens GN=G | 845 | CKQEINtKSAF | 0.306 | 0.143 | -0.261 | 0.063 |
| sp\|Q5T6X5\|GPC6A_HUMAN G-protein coupled receptor family C group 6 member A OS=Homo sapiens GN=G | 847 | QEINTKsAFLK | 0.145 | 0.049 | -0.736 | -0.181 |
| sp\|Q5T6X5\|GPC6A_HUMAN G-protein coupled receptor family C group 6 member A OS=Homo sapiens GN=G | 855 | FLKMIYsYSSH | 0.118 | -0.062 | -0.801 | -0.248 |
| sp\|Q5T6X5\|GPC6A_HUMAN G-protein coupled receptor family C group 6 member A OS=Homo sapiens GN=G | 857 | KMIYSYsSHSV | 0.187 | 0.328 | -0.697 | -0.061 |
| sp\|Q5T6X5\|GPC6A_HUMAN G-protein coupled receptor family C group 6 member A OS=Homo sapiens GN=G | 858 | MIYSYSsHSVS | 0.109 | -0.044 | -1.113 | -0.349 |
| sp\|Q5T6X5\|GPC6A_HUMAN G-protein coupled receptor family C group 6 member A OS=Homo sapiens GN=G | 860 | YSYSSHsVSSI | 0.217 | 0.161 | -0.429 | -0.017 |
| sp\|Q5T6X5\|GPC6A_HUMAN G-protein coupled receptor family C group 6 member A OS=Homo sapiens GN=G | 862 | YSSHSVsSIAL | 0.369 | 0.157 | -0.01 | 0.172 |
| sp\|Q5T6X5\|GPC6A_HUMAN G-protein coupled receptor family C group 6 member A OS=Homo sapiens GN=G | 863 | SSHSVSsIALS | 0.029 | -0.448 | -1.984 | -0.801 |
| sp\|Q5T6X5\|GPC6A_HUMAN G-protein coupled receptor family C group 6 member A OS=Homo sapiens GN=G | 867 | VSSIALsPASL | 0.033 | -0.478 | -1.673 | -0.706 |
| sp\|Q5T6X5\|GPC6A_HUMAN G-protein coupled receptor family C group 6 member A OS=Homo sapiens GN=G | 870 | IALSPAsLDSM | 0.251 | -0.122 | -0.438 | -0.103 |
| sp\|Q5T6X5\|GPC6A_HUMAN G-protein coupled receptor family C group 6 member A OS=Homo sapiens GN=G | 873 | SPASLDsMSGN | 0.043 | -0.312 | -1.845 | -0.705 |
| sp\|Q5T6X5\|GPC6A_HUMAN G-protein coupled receptor family C group 6 member A OS=Homo sapiens GN=G | 875 | ASLDSMsGNVT | 0.375 | 0.182 | -0.239 | 0.106 |
| sp\|Q5T6X5\|GPC6A_HUMAN G-protein coupled receptor family C group 6 member A OS=Homo sapiens GN=G | 879 | SMSGNVtMTNP | 0.145 | 0.048 | -1.157 | -0.321 |
| sp\|Q5T6X5\|GPC6A_HUMAN G-protein coupled receptor family C group 6 member A OS=Homo sapiens GN=G | 881 | SGNVTMtNPSS | 0.141 | 0.388 | -0.918 | -0.13 |
| sp\|Q5T6X5\|GPC6A_HUMAN G-protein coupled receptor family C group 6 member A OS=Homo sapiens GN=G | 884 | VTMTNPsSSGK | 0.095 | -0.34 | -1.249 | -0.498 |
| sp\|Q5T6X5\|GPC6A_HUMAN G-protein coupled receptor family C group 6 member A OS=Homo sapiens GN=G | 885 | TMTNPSsSGKS | 0.043 | -0.163 | -1.249 | -0.456 |
| sp\|Q5T6X5\|GPC6A_HUMAN G-protein coupled receptor family C group 6 member A OS=Homo sapiens GN=G | 886 | MTNPSSsGKSA | 0.122 | -0.041 | -0.865 | -0.261 |
| sp\|Q5T6X5\|GPC6A_HUMAN G-protein coupled receptor family C group 6 member A OS=Homo sapiens GN=G | 889 | PSSSGKsATWQ | 0.162 | -0.177 | -0.959 | -0.325 |
| sp\|Q5T6X5\|GPC6A_HUMAN G-protein coupled receptor family C group 6 member A OS=Homo sapiens GN=G | 891 | SSGKSAtWQKS | 0.272 | 0.175 | -0.623 | -0.059 |
| sp\|Q5T6X5\|GPC6A_HUMAN G-protein coupled receptor family C group 6 member A OS=Homo sapiens GN=G | 895 | SATWQKsKDLQ | 0.178 | -0.108 | -0.738 | -0.223 |
| sp\|Q5T6X5\|GPC6A_HUMAN G-protein coupled receptor family C group 6 member A OS=Homo sapiens GN=G | 912 | ICRENAtSVSK | 0.288 | 0.131 | -0.695 | -0.092 |
| sp\|Q5T6X5\|GPC6A_HUMAN G-protein coupled receptor family C group 6 member A OS=Homo sapiens GN=G | 913 | CRENATsVSKT | 0.197 | 0.153 | -0.43 | -0.027 |
| sp\|Q5T6X5\|GPC6A_HUMAN G-protein coupled receptor family C group 6 member A OS=Homo sapiens GN=G | 915 | ENATSVsKTLP | 0.315 | 0.001 | -0.334 | -0.006 |
| sp\|Q5T6X5\|GPC6A_HUMAN G-protein coupled receptor family C group 6 member A OS=Homo sapiens GN=G | 917 | ATSVSKtLPRK | 0.823 | 0.605 | 0.629 | 0.686 |
| sp\|Q5T6X5\|GPC6A_HUMAN G-protein coupled receptor family C group 6 member A OS=Homo sapiens GN=G | 924 | LPRKRMsSI-- | 0.645 | 0.6 | 0.168 | 0.471 |
| sp\|Q5T6X5\|GPC6A_HUMAN G-protein coupled receptor family C group 6 member A OS=Homo sapiens GN=G | 925 | PRKRMSsI--- | 0.644 | 1.11 | 0.527 | 0.76 |
| sp\|Q9UBS5\|GABR1_HUMAN Gamma-aminobutyric acid type B receptor subunit 1 OS=Homo sapiens GN=GAI | 315 | IATIQQtTEVF | 0.09 | -0.092 | -1.084 | -0.362 |
| sp\|Q9UBS5\|GABR1_HUMAN Gamma-aminobutyric acid type B receptor subunit 1 OS=Homo sapiens GN=GAI | 316 | ATIQQTtEVFT | 0.102 | -0.128 | -1.178 | -0.401 |
| sp\|Q9UBS5\|GABR1_HUMAN Gamma-aminobutyric acid type B receptor subunit 1 OS=Homo sapiens GN=GAI | 320 | QTTEVFtSTLD | 0.181 | -0.068 | -0.915 | -0.267 |
| sp\|Q9UBS5\|GABR1_HUMAN Gamma-aminobutyric acid type B receptor subunit 1 OS=Homo sapiens GN=GAI | 321 | TTEVFTsTLDD | 0.173 | -0.113 | -1.003 | -0.314 |
| sp\|Q9UBS5\|GABR1_HUMAN Gamma-aminobutyric acid type B receptor subunit 1 OS=Homo sapiens GN=GAI | 322 | TEVFTStLDDL | 0.267 | 0.135 | -0.491 | -0.03 |
| sp\|Q9UBS5\|GABR1_HUMAN Gamma-aminobutyric acid type B receptor subunit 1 OS=Homo sapiens GN=GAI | 338 | EAGIEItFRQS | 0.128 | -0.188 | -1.033 | -0.364 |
| sp\|Q9UBS5\|GABR1_HUMAN Gamma-aminobutyric acid type B receptor subunit 1 OS=Homo sapiens GN=GAI | 342 | EITFRQsFFSD | 0.427 | 0.297 | 0.024 | 0.249 |
| sp\|Q9UBS5\|GABR1_HUMAN Gamma-aminobutyric acid type B receptor subunit 1 OS=Homo sapiens GN=GAI | 345 | FRQSFFsDPAV | 0.767 | 0.899 | 0.222 | 0.629 |
| sp\|Q9UBS5\|GABR1_HUMAN Gamma-aminobutyric acid type B receptor subunit 1 OS=Homo sapiens GN=GAI | 369 | VGLFYEtEARK | 0.179 | -0.205 | -1.041 | -0.356 |

| | | | | | | |
|---|---|---|---|---|---|---|
| sp\|Q9UBS5\|GABR1_HUMAN Gamma-aminobutyric acid type B receptor subunit 1 OS=Homo sapiens GN=GAI | 407 | FKIYDPsINCT | 0.251 | 0.064 | -0.435 | -0.04 |
| sp\|Q9UBS5\|GABR1_HUMAN Gamma-aminobutyric acid type B receptor subunit 1 OS=Homo sapiens GN=GAI | 411 | DPSINCtVDEM | 0.373 | -0.026 | -0.696 | -0.116 |
| sp\|Q9UBS5\|GABR1_HUMAN Gamma-aminobutyric acid type B receptor subunit 1 OS=Homo sapiens GN=GAI | 416 | CTVDEMtEAVE | 0.296 | 0.09 | -0.842 | -0.152 |
| sp\|Q9UBS5\|GABR1_HUMAN Gamma-aminobutyric acid type B receptor subunit 1 OS=Homo sapiens GN=GAI | 424 | AVEGHItTEIV | 0.207 | -0.069 | -1.023 | -0.295 |
| sp\|Q9UBS5\|GABR1_HUMAN Gamma-aminobutyric acid type B receptor subunit 1 OS=Homo sapiens GN=GAI | 425 | VEGHITtEIVM | 0.106 | -0.053 | -0.774 | -0.24 |
| sp\|Q9UBS5\|GABR1_HUMAN Gamma-aminobutyric acid type B receptor subunit 1 OS=Homo sapiens GN=GAI | 435 | MLNPANtRSIS | 0.338 | 0.024 | -0.825 | -0.154 |
| sp\|Q9UBS5\|GABR1_HUMAN Gamma-aminobutyric acid type B receptor subunit 1 OS=Homo sapiens GN=GAI | 437 | NPANTRsISNM | 0.042 | -0.15 | -1.379 | -0.496 |
| sp\|Q9UBS5\|GABR1_HUMAN Gamma-aminobutyric acid type B receptor subunit 1 OS=Homo sapiens GN=GAI | 439 | ANTRSIsNMTS | 0.67 | 0.856 | 0.37 | 0.632 |
| sp\|Q9UBS5\|GABR1_HUMAN Gamma-aminobutyric acid type B receptor subunit 1 OS=Homo sapiens GN=GAI | 442 | RSISNMtSQEF | 0.237 | 0.012 | -0.696 | -0.149 |
| sp\|Q9UBS5\|GABR1_HUMAN Gamma-aminobutyric acid type B receptor subunit 1 OS=Homo sapiens GN=GAI | 443 | SISNMTsQEFV | 0.066 | -0.252 | -1.584 | -0.59 |
| sp\|Q9UBS5\|GABR1_HUMAN Gamma-aminobutyric acid type B receptor subunit 1 OS=Homo sapiens GN=GAI | 451 | EFVEKLtKRLK | 0.049 | -0.248 | -1.219 | -0.473 |
| sp\|Q9UBS5\|GABR1_HUMAN Gamma-aminobutyric acid type B receptor subunit 1 OS=Homo sapiens GN=GAI | 461 | KRHPEEtGGFQ | 0.071 | -0.16 | -1.558 | -0.549 |
| sp\|Q9UBS5\|GABR1_HUMAN Gamma-aminobutyric acid type B receptor subunit 1 OS=Homo sapiens GN=GAI | 484 | ALALNKtSGGG | 0.499 | 0.058 | -0.06 | 0.166 |
| sp\|Q9UBS5\|GABR1_HUMAN Gamma-aminobutyric acid type B receptor subunit 1 OS=Homo sapiens GN=GAI | 485 | LALNKTsGGGG | 0.116 | -0.198 | -0.694 | -0.259 |
| sp\|Q9UBS5\|GABR1_HUMAN Gamma-aminobutyric acid type B receptor subunit 1 OS=Homo sapiens GN=GAI | 491 | SGGGGRsGVRL | 0.028 | -0.316 | -1.796 | -0.695 |
| sp\|Q9UBS5\|GABR1_HUMAN Gamma-aminobutyric acid type B receptor subunit 1 OS=Homo sapiens GN=GAI | 504 | FNYNNQtITDQ | 0.175 | -0.085 | -0.583 | -0.164 |
| sp\|Q9UBS5\|GABR1_HUMAN Gamma-aminobutyric acid type B receptor subunit 1 OS=Homo sapiens GN=GAI | 506 | YNNQTItDQIY | 0.272 | -0.014 | -0.68 | -0.141 |
| sp\|Q9UBS5\|GABR1_HUMAN Gamma-aminobutyric acid type B receptor subunit 1 OS=Homo sapiens GN=GAI | 515 | IYRAMNsSSFE | 0.332 | -0.001 | -0.693 | -0.121 |
| sp\|Q9UBS5\|GABR1_HUMAN Gamma-aminobutyric acid type B receptor subunit 1 OS=Homo sapiens GN=GAI | 516 | YRAMNSsSFEG | 0.219 | 0.078 | -0.511 | -0.071 |
| sp\|Q9UBS5\|GABR1_HUMAN Gamma-aminobutyric acid type B receptor subunit 1 OS=Homo sapiens GN=GAI | 517 | RAMNSSsFEGV | 0.269 | 0.163 | -0.466 | -0.011 |
| sp\|Q9UBS5\|GABR1_HUMAN Gamma-aminobutyric acid type B receptor subunit 1 OS=Homo sapiens GN=GAI | 522 | SSFEGVsGHVV | 0.089 | -0.228 | -1.559 | -0.566 |
| sp\|Q9UBS5\|GABR1_HUMAN Gamma-aminobutyric acid type B receptor subunit 1 OS=Homo sapiens GN=GAI | 530 | HVVFDAsGSRM | 0.109 | -0.158 | -1.152 | -0.4 |
| sp\|Q9UBS5\|GABR1_HUMAN Gamma-aminobutyric acid type B receptor subunit 1 OS=Homo sapiens GN=GAI | 532 | VFDASGsRMAW | 0.234 | 0.031 | -0.607 | -0.114 |
| sp\|Q9UBS5\|GABR1_HUMAN Gamma-aminobutyric acid type B receptor subunit 1 OS=Homo sapiens GN=GAI | 537 | GSRMAWtLIEQ | 0.728 | 0.258 | 0.176 | 0.387 |
| sp\|Q9UBS5\|GABR1_HUMAN Gamma-aminobutyric acid type B receptor subunit 1 OS=Homo sapiens GN=GAI | 546 | EQLQGGsYKKI | 0.049 | -0.255 | -1.042 | -0.416 |
| sp\|Q9UBS5\|GABR1_HUMAN Gamma-aminobutyric acid type B receptor subunit 1 OS=Homo sapiens GN=GAI | 555 | KIGYYDsTKDD | 0.249 | 0 | -0.664 | -0.138 |
| sp\|Q9UBS5\|GABR1_HUMAN Gamma-aminobutyric acid type B receptor subunit 1 OS=Homo sapiens GN=GAI | 556 | IGYYDStKDDL | 0.239 | -0.001 | -0.307 | -0.023 |
| sp\|Q9UBS5\|GABR1_HUMAN Gamma-aminobutyric acid type B receptor subunit 1 OS=Homo sapiens GN=GAI | 561 | STKDDLsWSKT | 0.05 | -0.218 | -1.634 | -0.601 |
| sp\|Q9UBS5\|GABR1_HUMAN Gamma-aminobutyric acid type B receptor subunit 1 OS=Homo sapiens GN=GAI | 563 | KDDLSWsKTDK | 0.342 | -0.023 | -0.562 | -0.081 |
| sp\|Q9UBS5\|GABR1_HUMAN Gamma-aminobutyric acid type B receptor subunit 1 OS=Homo sapiens GN=GAI | 565 | DLSWSKtDKWI | 0.781 | 0.371 | 0.585 | 0.579 |
| sp\|Q9UBS5\|GABR1_HUMAN Gamma-aminobutyric acid type B receptor subunit 1 OS=Homo sapiens GN=GAI | 572 | DKWIGGsPPAD | 0.376 | 0.314 | -0.335 | 0.118 |
| sp\|Q9UBS5\|GABR1_HUMAN Gamma-aminobutyric acid type B receptor subunit 1 OS=Homo sapiens GN=GAI | 578 | SPPADQtLVIK | 0.142 | -0.212 | -1.072 | -0.381 |
| sp\|Q9UBS5\|GABR1_HUMAN Gamma-aminobutyric acid type B receptor subunit 1 OS=Homo sapiens GN=GAI | 583 | QTLVIKtFRFL | 0.21 | -0.059 | -0.435 | -0.095 |
| sp\|Q9UBS5\|GABR1_HUMAN Gamma-aminobutyric acid type B receptor subunit 1 OS=Homo sapiens GN=GAI | 588 | KTFRFLsQKLF | 0.325 | 0.654 | -0.486 | 0.164 |
| sp\|Q9UBS5\|GABR1_HUMAN Gamma-aminobutyric acid type B receptor subunit 1 OS=Homo sapiens GN=GAI | 594 | SQKLFIsVSVL | 0.112 | -0.079 | -1.056 | -0.341 |
| sp\|Q9UBS5\|GABR1_HUMAN Gamma-aminobutyric acid type B receptor subunit 1 OS=Homo sapiens GN=GAI | 596 | KLFISVsVLSS | 0.306 | 0.202 | -0.498 | 0.003 |
| sp\|Q9UBS5\|GABR1_HUMAN Gamma-aminobutyric acid type B receptor subunit 1 OS=Homo sapiens GN=GAI | 599 | ISVSVLsSLGI | 0.105 | -0.274 | -1.151 | -0.44 |
| sp\|Q9UBS5\|GABR1_HUMAN Gamma-aminobutyric acid type B receptor subunit 1 OS=Homo sapiens GN=GAI | 600 | SVSVLSsLGIV | 0.109 | -0.123 | -1.296 | -0.437 |
| sp\|Q9UBS5\|GABR1_HUMAN Gamma-aminobutyric acid type B receptor subunit 1 OS=Homo sapiens GN=GAI | 611 | LAVVCLsFNIY | 0.26 | 0 | -0.621 | -0.12 |
| sp\|Q9UBS5\|GABR1_HUMAN Gamma-aminobutyric acid type B receptor subunit 1 OS=Homo sapiens GN=GAI | 617 | SFNIYNsHVRY | 0.282 | -0.034 | -1.067 | -0.273 |
| sp\|Q9UBS5\|GABR1_HUMAN Gamma-aminobutyric acid type B receptor subunit 1 OS=Homo sapiens GN=GAI | 625 | VRYIQNsQPNL | 0.487 | 0.726 | -0.396 | 0.272 |
| sp\|Q9UBS5\|GABR1_HUMAN Gamma-aminobutyric acid type B receptor subunit 1 OS=Homo sapiens GN=GAI | 633 | PNLNNLtAVGC | 0.078 | -0.235 | -1.113 | -0.423 |
| sp\|Q9UBS5\|GABR1_HUMAN Gamma-aminobutyric acid type B receptor subunit 1 OS=Homo sapiens GN=GAI | 638 | LTAVGCsLALA | 0.172 | -0.112 | -0.81 | -0.25 |
| sp\|Q9UBS5\|GABR1_HUMAN Gamma-aminobutyric acid type B receptor subunit 1 OS=Homo sapiens GN=GAI | 675 | LLGLGFsLGYG | 0.336 | 0.123 | -0.336 | 0.041 |
| sp\|Q9UBS5\|GABR1_HUMAN Gamma-aminobutyric acid type B receptor subunit 1 OS=Homo sapiens GN=GAI | 680 | FSLGYGsMFTK | 0.154 | -0.048 | -0.908 | -0.267 |
| sp\|Q9UBS5\|GABR1_HUMAN Gamma-aminobutyric acid type B receptor subunit 1 OS=Homo sapiens GN=GAI | 683 | GYGSMFtKIWW | 0.16 | -0.02 | -0.634 | -0.165 |
| sp\|Q9UBS5\|GABR1_HUMAN Gamma-aminobutyric acid type B receptor subunit 1 OS=Homo sapiens GN=GAI | 690 | KIWWVHtVFTK | 0.349 | 0.176 | -0.417 | 0.036 |
| sp\|Q9UBS5\|GABR1_HUMAN Gamma-aminobutyric acid type B receptor subunit 1 OS=Homo sapiens GN=GAI | 693 | WVHTVFtKKEE | 0.128 | -0.136 | -0.942 | -0.317 |
| sp\|Q9UBS5\|GABR1_HUMAN Gamma-aminobutyric acid type B receptor subunit 1 OS=Homo sapiens GN=GAI | 704 | KKEWRKtLEPW | 0.703 | 0.233 | 0.394 | 0.443 |

| | | | | | | |
|---|---|---|---|---|---|---|
| sp\|Q9UBS5\|GABR1_HUMAN Gamma-aminobutyric acid type B receptor subunit 1 OS=Homo sapiens GN=GAI | 713 | PWKLYAtVGLL | 0.517 | 0.237 | -0.153 | 0.2 |
| sp\|Q9UBS5\|GABR1_HUMAN Gamma-aminobutyric acid type B receptor subunit 1 OS=Homo sapiens GN=GAI | 724 | VGMDVLtLAIW | 0.166 | -0.044 | -0.836 | -0.238 |
| sp\|Q9UBS5\|GABR1_HUMAN Gamma-aminobutyric acid type B receptor subunit 1 OS=Homo sapiens GN=GAI | 737 | VDPLHRtIETF | 0.041 | -0.297 | -1.671 | -0.642 |
| sp\|Q9UBS5\|GABR1_HUMAN Gamma-aminobutyric acid type B receptor subunit 1 OS=Homo sapiens GN=GAI | 740 | LHRTIEtFAKE | 0.231 | 0.075 | -0.884 | -0.193 |
| sp\|Q9UBS5\|GABR1_HUMAN Gamma-aminobutyric acid type B receptor subunit 1 OS=Homo sapiens GN=GAI | 753 | KEDIDVsILPQ | 0.069 | -0.325 | -1.539 | -0.598 |
| sp\|Q9UBS5\|GABR1_HUMAN Gamma-aminobutyric acid type B receptor subunit 1 OS=Homo sapiens GN=GAI | 762 | PQLEHCsSRKM | 0.151 | -0.127 | -0.998 | -0.325 |
| sp\|Q9UBS5\|GABR1_HUMAN Gamma-aminobutyric acid type B receptor subunit 1 OS=Homo sapiens GN=GAI | 763 | QLEHCSsRKMN | 0.181 | -0.187 | -0.935 | -0.314 |
| sp\|Q9UBS5\|GABR1_HUMAN Gamma-aminobutyric acid type B receptor subunit 1 OS=Homo sapiens GN=GAI | 768 | SSRKMNtWLGI | 0.49 | 0.212 | -0.25 | 0.151 |
| sp\|Q9UBS5\|GABR1_HUMAN Gamma-aminobutyric acid type B receptor subunit 1 OS=Homo sapiens GN=GAI | 791 | IFLAYEtKSVS | 0.161 | -0.152 | -0.875 | -0.289 |
| sp\|Q9UBS5\|GABR1_HUMAN Gamma-aminobutyric acid type B receptor subunit 1 OS=Homo sapiens GN=GAI | 793 | LAYETKsVSTE | 0.059 | -0.14 | -1.121 | -0.401 |
| sp\|Q9UBS5\|GABR1_HUMAN Gamma-aminobutyric acid type B receptor subunit 1 OS=Homo sapiens GN=GAI | 795 | YETKSVsTEKI | 0.592 | 0.247 | 0.094 | 0.311 |
| sp\|Q9UBS5\|GABR1_HUMAN Gamma-aminobutyric acid type B receptor subunit 1 OS=Homo sapiens GN=GAI | 796 | ETKSVStEKIN | 0.173 | -0.1 | -0.961 | -0.296 |
| sp\|Q9UBS5\|GABR1_HUMAN Gamma-aminobutyric acid type B receptor subunit 1 OS=Homo sapiens GN=GAI | 819 | AVLCLItAPVT | 0.471 | 0.389 | -0.382 | 0.159 |
| sp\|Q9UBS5\|GABR1_HUMAN Gamma-aminobutyric acid type B receptor subunit 1 OS=Homo sapiens GN=GAI | 823 | LITAPVtMILS | 0.189 | 0.029 | -0.563 | -0.115 |
| sp\|Q9UBS5\|GABR1_HUMAN Gamma-aminobutyric acid type B receptor subunit 1 OS=Homo sapiens GN=GAI | 827 | PVTMILsSQQD | 0.157 | -0.112 | -0.558 | -0.171 |
| sp\|Q9UBS5\|GABR1_HUMAN Gamma-aminobutyric acid type B receptor subunit 1 OS=Homo sapiens GN=GAI | 828 | VTMILSsQQDA | 0.111 | -0.164 | -1.274 | -0.442 |
| sp\|Q9UBS5\|GABR1_HUMAN Gamma-aminobutyric acid type B receptor subunit 1 OS=Homo sapiens GN=GAI | 838 | AAFAFAsLAIV | 0.367 | -0.083 | -0.932 | -0.216 |
| sp\|Q9UBS5\|GABR1_HUMAN Gamma-aminobutyric acid type B receptor subunit 1 OS=Homo sapiens GN=GAI | 844 | SLAIVFsSYIT | 0.161 | -0.034 | -0.879 | -0.251 |
| sp\|Q9UBS5\|GABR1_HUMAN Gamma-aminobutyric acid type B receptor subunit 1 OS=Homo sapiens GN=GAI | 845 | LAIVFSsYITL | 0.038 | -0.224 | -1.445 | -0.544 |
| sp\|Q9UBS5\|GABR1_HUMAN Gamma-aminobutyric acid type B receptor subunit 1 OS=Homo sapiens GN=GAI | 848 | VFSSYItLVVL | 0.347 | 0.044 | -0.32 | 0.024 |
| sp\|Q9UBS5\|GABR1_HUMAN Gamma-aminobutyric acid type B receptor subunit 1 OS=Homo sapiens GN=GAI | 862 | KMRRLItRGEW | 0.747 | 1.112 | 0.405 | 0.755 |
| sp\|Q9UBS5\|GABR1_HUMAN Gamma-aminobutyric acid type B receptor subunit 1 OS=Homo sapiens GN=GAI | 868 | TRGEWQsEAQD | 0.178 | 0.19 | -0.63 | -0.087 |
| sp\|Q9UBS5\|GABR1_HUMAN Gamma-aminobutyric acid type B receptor subunit 1 OS=Homo sapiens GN=GAI | 873 | QSEAQDtMKTG | 0.095 | -0.201 | -1.31 | -0.472 |
| sp\|Q9UBS5\|GABR1_HUMAN Gamma-aminobutyric acid type B receptor subunit 1 OS=Homo sapiens GN=GAI | 876 | AQDTMKtGSST | 0.052 | -0.357 | -1.264 | -0.523 |
| sp\|Q9UBS5\|GABR1_HUMAN Gamma-aminobutyric acid type B receptor subunit 1 OS=Homo sapiens GN=GAI | 878 | DTMKTGsSTNN | 0.215 | -0.018 | -1.247 | -0.35 |
| sp\|Q9UBS5\|GABR1_HUMAN Gamma-aminobutyric acid type B receptor subunit 1 OS=Homo sapiens GN=GAI | 879 | TMKTGSsTNNN | 0.087 | -0.015 | -1.223 | -0.384 |
| sp\|Q9UBS5\|GABR1_HUMAN Gamma-aminobutyric acid type B receptor subunit 1 OS=Homo sapiens GN=GAI | 880 | MKTGSStNNNE | 0.467 | 0.275 | -0.184 | 0.186 |
| sp\|Q9UBS5\|GABR1_HUMAN Gamma-aminobutyric acid type B receptor subunit 1 OS=Homo sapiens GN=GAI | 888 | NNEEEKsRLLE | 0.086 | -0.274 | -1.31 | -0.499 |
| sp\|Q9UBS5\|GABR1_HUMAN Gamma-aminobutyric acid type B receptor subunit 1 OS=Homo sapiens GN=GAI | 910 | EKEERVsELRH | 0.332 | 0.005 | -0.76 | -0.141 |
| sp\|Q9UBS5\|GABR1_HUMAN Gamma-aminobutyric acid type B receptor subunit 1 OS=Homo sapiens GN=GAI | 918 | LRHQLQsRQQL | 0.144 | 0.057 | -0.744 | -0.181 |
| sp\|Q9UBS5\|GABR1_HUMAN Gamma-aminobutyric acid type B receptor subunit 1 OS=Homo sapiens GN=GAI | 924 | SRQQLRsRRHP | 0.059 | -0.144 | -1.475 | -0.52 |
| sp\|Q9UBS5\|GABR1_HUMAN Gamma-aminobutyric acid type B receptor subunit 1 OS=Homo sapiens GN=GAI | 930 | SRRHPPtPPEP | 0.362 | 0.372 | -0.337 | 0.132 |
| sp\|Q9UBS5\|GABR1_HUMAN Gamma-aminobutyric acid type B receptor subunit 1 OS=Homo sapiens GN=GAI | 935 | PTPPEPsGGLP | 0.09 | -0.307 | -1.342 | -0.52 |
| sp\|Q9UBS5\|GABR1_HUMAN Gamma-aminobutyric acid type B receptor subunit 1 OS=Homo sapiens GN=GAI | 950 | EPPDRLsCDGS | 0.129 | 0.013 | -1.04 | -0.299 |
| sp\|Q9UBS5\|GABR1_HUMAN Gamma-aminobutyric acid type B receptor subunit 1 OS=Homo sapiens GN=GAI | 954 | RLSCDGsRVHL | 0.147 | -0.16 | -1.104 | -0.372 |
| sp\|O75899\|GABR2_HUMAN Gamma-aminobutyric acid type B receptor subunit 2 OS=Homo sapiens GN=GAE | 323 | VDFEPLsSKQI | 0.048 | -0.332 | -1.305 | -0.53 |
| sp\|O75899\|GABR2_HUMAN Gamma-aminobutyric acid type B receptor subunit 2 OS=Homo sapiens GN=GAE | 324 | DFEPLSsKQIK | 0.158 | -0.255 | -0.969 | -0.355 |
| sp\|O75899\|GABR2_HUMAN Gamma-aminobutyric acid type B receptor subunit 2 OS=Homo sapiens GN=GAE | 329 | SSKQIKtISGK | 0.141 | -0.251 | -0.564 | -0.225 |
| sp\|O75899\|GABR2_HUMAN Gamma-aminobutyric acid type B receptor subunit 2 OS=Homo sapiens GN=GAE | 331 | KQIKTIsGKTP | 0.095 | -0.146 | -0.953 | -0.335 |
| sp\|O75899\|GABR2_HUMAN Gamma-aminobutyric acid type B receptor subunit 2 OS=Homo sapiens GN=GAE | 334 | KTISGKtPQQY | 0.107 | -0.397 | -1.229 | -0.506 |
| sp\|O75899\|GABR2_HUMAN Gamma-aminobutyric acid type B receptor subunit 2 OS=Homo sapiens GN=GAE | 347 | EYNNKRsGVGP | 0.036 | -0.261 | -1.478 | -0.568 |
| sp\|O75899\|GABR2_HUMAN Gamma-aminobutyric acid type B receptor subunit 2 OS=Homo sapiens GN=GAE | 352 | RSGVGPsKFHG | 0.056 | -0.29 | -1.253 | -0.496 |
| sp\|O75899\|GABR2_HUMAN Gamma-aminobutyric acid type B receptor subunit 2 OS=Homo sapiens GN=GAE | 368 | IWVIAKtLQRA | 0.598 | 0.161 | 0.11 | 0.29 |
| sp\|O75899\|GABR2_HUMAN Gamma-aminobutyric acid type B receptor subunit 2 OS=Homo sapiens GN=GAE | 375 | LQRAMEtLHAS | 0.505 | 0.213 | 0.003 | 0.24 |
| sp\|O75899\|GABR2_HUMAN Gamma-aminobutyric acid type B receptor subunit 2 OS=Homo sapiens GN=GAE | 379 | METLHAsSRHQ | 0.223 | -0.088 | -0.926 | -0.264 |
| sp\|O75899\|GABR2_HUMAN Gamma-aminobutyric acid type B receptor subunit 2 OS=Homo sapiens GN=GAE | 380 | ETLHASsRHQR | 0.177 | -0.173 | -0.875 | -0.29 |
| sp\|O75899\|GABR2_HUMAN Gamma-aminobutyric acid type B receptor subunit 2 OS=Homo sapiens GN=GAE | 391 | IQDFNYtDHTL | 0.328 | 0.086 | -0.321 | 0.031 |
| sp\|O75899\|GABR2_HUMAN Gamma-aminobutyric acid type B receptor subunit 2 OS=Homo sapiens GN=GAE | 394 | FNYTDHtLGRI | 0.531 | 0.156 | -0.119 | 0.189 |
| sp\|O75899\|GABR2_HUMAN Gamma-aminobutyric acid type B receptor subunit 2 OS=Homo sapiens GN=GAE | 406 | LNAMNEtNFFG | 0.096 | -0.326 | -1.122 | -0.451 |

| | | | | | | |
|---|---|---|---|---|---|---|
| sp\|O75899\|GABR2_HUMAN Gamma-aminobutyric acid type B receptor subunit 2 OS=Homo sapiens GN=GAE | 412 | TNFFGVtGQVV | 0.153 | -0.179 | -1.091 | -0.372 |
| sp\|O75899\|GABR2_HUMAN Gamma-aminobutyric acid type B receptor subunit 2 OS=Homo sapiens GN=GAE | 425 | NGERMGtIKFT | 0.26 | 0.482 | -0.16 | 0.194 |
| sp\|O75899\|GABR2_HUMAN Gamma-aminobutyric acid type B receptor subunit 2 OS=Homo sapiens GN=GAE | 429 | MGTIKFtQFQD | 0.229 | 0.002 | -0.562 | -0.11 |
| sp\|O75899\|GABR2_HUMAN Gamma-aminobutyric acid type B receptor subunit 2 OS=Homo sapiens GN=GAE | 434 | FTQFQDsREVK | 0.144 | -0.107 | -1.353 | -0.439 |
| sp\|O75899\|GABR2_HUMAN Gamma-aminobutyric acid type B receptor subunit 2 OS=Homo sapiens GN=GAE | 448 | YNAVADtLEII | 0.331 | -0.006 | -0.312 | 0.004 |
| sp\|O75899\|GABR2_HUMAN Gamma-aminobutyric acid type B receptor subunit 2 OS=Homo sapiens GN=GAE | 455 | LEIINDtIRFQ | 0.065 | -0.343 | -1.489 | -0.589 |
| sp\|O75899\|GABR2_HUMAN Gamma-aminobutyric acid type B receptor subunit 2 OS=Homo sapiens GN=GAE | 461 | TIRFQGsEPPK | 0.619 | 0.663 | 0.056 | 0.446 |
| sp\|O75899\|GABR2_HUMAN Gamma-aminobutyric acid type B receptor subunit 2 OS=Homo sapiens GN=GAE | 468 | EPPKDKtIILE | 0.124 | -0.137 | -0.769 | -0.261 |
| sp\|O75899\|GABR2_HUMAN Gamma-aminobutyric acid type B receptor subunit 2 OS=Homo sapiens GN=GAE | 478 | EQLRKIsLPLY | 0.885 | 1.406 | 1.452 | 1.248 |
| sp\|O75899\|GABR2_HUMAN Gamma-aminobutyric acid type B receptor subunit 2 OS=Homo sapiens GN=GAE | 483 | ISLPLYsILSA | 0.051 | -0.274 | -1.159 | -0.461 |
| sp\|O75899\|GABR2_HUMAN Gamma-aminobutyric acid type B receptor subunit 2 OS=Homo sapiens GN=GAE | 486 | PLYSILsALTI | 0.176 | 0.019 | -0.687 | -0.164 |
| sp\|O75899\|GABR2_HUMAN Gamma-aminobutyric acid type B receptor subunit 2 OS=Homo sapiens GN=GAE | 489 | SILSALtILGM | 0.085 | -0.182 | -1.142 | -0.413 |
| sp\|O75899\|GABR2_HUMAN Gamma-aminobutyric acid type B receptor subunit 2 OS=Homo sapiens GN=GAE | 497 | LGMIMAsAFLF | 0.171 | 0.013 | -0.696 | -0.171 |
| sp\|O75899\|GABR2_HUMAN Gamma-aminobutyric acid type B receptor subunit 2 OS=Homo sapiens GN=GAE | 515 | QKLIKMsSPYM | 0.562 | 0.527 | 0.093 | 0.394 |
| sp\|O75899\|GABR2_HUMAN Gamma-aminobutyric acid type B receptor subunit 2 OS=Homo sapiens GN=GAE | 516 | KLIKMSsPYMN | 0.123 | -0.342 | -1.234 | -0.484 |
| sp\|O75899\|GABR2_HUMAN Gamma-aminobutyric acid type B receptor subunit 2 OS=Homo sapiens GN=GAE | 530 | ILGGMLsYASI | 0.082 | -0.168 | -0.911 | -0.332 |
| sp\|O75899\|GABR2_HUMAN Gamma-aminobutyric acid type B receptor subunit 2 OS=Homo sapiens GN=GAE | 533 | GMLSYAsIFLF | 0.181 | 0.06 | -0.739 | -0.166 |
| sp\|O75899\|GABR2_HUMAN Gamma-aminobutyric acid type B receptor subunit 2 OS=Homo sapiens GN=GAE | 542 | LFGLDGsFVSE | 0.07 | -0.175 | -1.235 | -0.447 |
| sp\|O75899\|GABR2_HUMAN Gamma-aminobutyric acid type B receptor subunit 2 OS=Homo sapiens GN=GAE | 545 | LDGSFVsEKTF | 0.09 | -0.228 | -1.121 | -0.42 |
| sp\|O75899\|GABR2_HUMAN Gamma-aminobutyric acid type B receptor subunit 2 OS=Homo sapiens GN=GAE | 548 | SFVSEKtFETL | 0.211 | -0.028 | -0.7 | -0.172 |
| sp\|O75899\|GABR2_HUMAN Gamma-aminobutyric acid type B receptor subunit 2 OS=Homo sapiens GN=GAE | 551 | SEKTFEtLCTV | 0.187 | -0.199 | -1.416 | -0.476 |
| sp\|O75899\|GABR2_HUMAN Gamma-aminobutyric acid type B receptor subunit 2 OS=Homo sapiens GN=GAE | 554 | TFETLCtVRTW | 0.19 | -0.164 | -0.978 | -0.317 |
| sp\|O75899\|GABR2_HUMAN Gamma-aminobutyric acid type B receptor subunit 2 OS=Homo sapiens GN=GAE | 557 | TLCTVRtWILT | 0.148 | 0.043 | -0.972 | -0.26 |
| sp\|O75899\|GABR2_HUMAN Gamma-aminobutyric acid type B receptor subunit 2 OS=Homo sapiens GN=GAE | 561 | VRTWILtVGYT | 0.207 | 0.071 | -0.448 | -0.057 |
| sp\|O75899\|GABR2_HUMAN Gamma-aminobutyric acid type B receptor subunit 2 OS=Homo sapiens GN=GAE | 565 | ILTVGYtTAFG | 0.103 | -0.096 | -0.876 | -0.29 |
| sp\|O75899\|GABR2_HUMAN Gamma-aminobutyric acid type B receptor subunit 2 OS=Homo sapiens GN=GAE | 566 | LTVGYTtAFGA | 0.209 | -0.074 | -0.672 | -0.179 |
| sp\|O75899\|GABR2_HUMAN Gamma-aminobutyric acid type B receptor subunit 2 OS=Homo sapiens GN=GAE | 575 | GAMFAKtWRVH | 0.341 | 0.098 | -0.543 | -0.035 |
| sp\|O75899\|GABR2_HUMAN Gamma-aminobutyric acid type B receptor subunit 2 OS=Homo sapiens GN=GAE | 624 | VDPLRRtVEKY | 0.103 | -0.076 | -1.374 | -0.449 |
| sp\|O75899\|GABR2_HUMAN Gamma-aminobutyric acid type B receptor subunit 2 OS=Homo sapiens GN=GAE | 629 | RTVEKYsMEPD | 0.112 | 0.047 | -1.084 | -0.308 |
| sp\|O75899\|GABR2_HUMAN Gamma-aminobutyric acid type B receptor subunit 2 OS=Homo sapiens GN=GAE | 640 | PAGRDIsIRPL | 0.417 | 0.634 | 0.062 | 0.371 |
| sp\|O75899\|GABR2_HUMAN Gamma-aminobutyric acid type B receptor subunit 2 OS=Homo sapiens GN=GAE | 651 | LEHCENtHMTI | 0.29 | -0.059 | -0.945 | -0.238 |
| sp\|O75899\|GABR2_HUMAN Gamma-aminobutyric acid type B receptor subunit 2 OS=Homo sapiens GN=GAE | 654 | CENTHMtIWLG | 0.406 | 0.155 | -0.582 | -0.007 |
| sp\|O75899\|GABR2_HUMAN Gamma-aminobutyric acid type B receptor subunit 2 OS=Homo sapiens GN=GAE | 678 | CFLAWEtRNVS | 0.251 | -0.067 | -0.634 | -0.15 |
| sp\|O75899\|GABR2_HUMAN Gamma-aminobutyric acid type B receptor subunit 2 OS=Homo sapiens GN=GAE | 682 | WETRNVsIPAL | 0.925 | 1.374 | 1.598 | 1.299 |
| sp\|O75899\|GABR2_HUMAN Gamma-aminobutyric acid type B receptor subunit 2 OS=Homo sapiens GN=GAE | 689 | IPALNDsKYIG | 0.241 | -0.164 | -0.748 | -0.224 |
| sp\|O75899\|GABR2_HUMAN Gamma-aminobutyric acid type B receptor subunit 2 OS=Homo sapiens GN=GAE | 695 | SKYIGMsVYNV | 0.183 | 0.126 | -1.069 | -0.253 |
| sp\|O75899\|GABR2_HUMAN Gamma-aminobutyric acid type B receptor subunit 2 OS=Homo sapiens GN=GAE | 710 | IIGAAVsFLTR | 0.463 | 0.095 | -0.183 | 0.125 |
| sp\|O75899\|GABR2_HUMAN Gamma-aminobutyric acid type B receptor subunit 2 OS=Homo sapiens GN=GAE | 713 | AAVSFLtRDQP | 0.061 | -0.385 | -1.614 | -0.646 |
| sp\|O75899\|GABR2_HUMAN Gamma-aminobutyric acid type B receptor subunit 2 OS=Homo sapiens GN=GAE | 732 | LVIIFCsTITL | 0.125 | -0.011 | -1.035 | -0.307 |
| sp\|O75899\|GABR2_HUMAN Gamma-aminobutyric acid type B receptor subunit 2 OS=Homo sapiens GN=GAE | 733 | VIIFCStITLC | 0.172 | -0.102 | -1.095 | -0.342 |
| sp\|O75899\|GABR2_HUMAN Gamma-aminobutyric acid type B receptor subunit 2 OS=Homo sapiens GN=GAE | 735 | IFCSTItLCLV | 0.311 | -0.017 | -0.621 | -0.109 |
| sp\|O75899\|GABR2_HUMAN Gamma-aminobutyric acid type B receptor subunit 2 OS=Homo sapiens GN=GAE | 746 | FVPKLItLRTN | 0.373 | 0.03 | -0.472 | -0.023 |
| sp\|O75899\|GABR2_HUMAN Gamma-aminobutyric acid type B receptor subunit 2 OS=Homo sapiens GN=GAE | 749 | KLITLRtNPDA | 0.55 | 0.462 | -0.379 | 0.211 |
| sp\|O75899\|GABR2_HUMAN Gamma-aminobutyric acid type B receptor subunit 2 OS=Homo sapiens GN=GAE | 755 | TNPDAAtQNRR | 0.196 | -0.185 | -1.132 | -0.374 |
| sp\|O75899\|GABR2_HUMAN Gamma-aminobutyric acid type B receptor subunit 2 OS=Homo sapiens GN=GAE | 763 | NRRFQFtQNQK | 0.465 | 0.402 | -0.263 | 0.201 |
| sp\|O75899\|GABR2_HUMAN Gamma-aminobutyric acid type B receptor subunit 2 OS=Homo sapiens GN=GAE | 771 | NQKKEDsKTST | 0.199 | -0.199 | -0.901 | -0.3 |
| sp\|O75899\|GABR2_HUMAN Gamma-aminobutyric acid type B receptor subunit 2 OS=Homo sapiens GN=GAE | 773 | KKEDSKtSTSV | 0.502 | 0.186 | -0.377 | 0.104 |
| sp\|O75899\|GABR2_HUMAN Gamma-aminobutyric acid type B receptor subunit 2 OS=Homo sapiens GN=GAE | 774 | KEDSKTsTSVT | 0.038 | -0.346 | -1.732 | -0.68 |
| sp\|O75899\|GABR2_HUMAN Gamma-aminobutyric acid type B receptor subunit 2 OS=Homo sapiens GN=GAE | 775 | EDSKTStSVTS | 0.041 | -0.253 | -1.474 | -0.562 |

| | | | | | | |
|---|---|---|---|---|---|---|
| sp\|O75899\|GABR2_HUMAN Gamma-aminobutyric acid type B receptor subunit 2 OS=Homo sapiens GN=GAE | 776 | DSKTSTsVTSV | 0.386 | 0.083 | -0.633 | -0.055 |
| sp\|O75899\|GABR2_HUMAN Gamma-aminobutyric acid type B receptor subunit 2 OS=Homo sapiens GN=GAE | 778 | KTSTSVtSVNQ | 0.337 | 0.036 | -0.831 | -0.153 |
| sp\|O75899\|GABR2_HUMAN Gamma-aminobutyric acid type B receptor subunit 2 OS=Homo sapiens GN=GAE | 779 | TSTSVTsVNQA | 0.195 | -0.188 | -0.621 | -0.205 |
| sp\|O75899\|GABR2_HUMAN Gamma-aminobutyric acid type B receptor subunit 2 OS=Homo sapiens GN=GAE | 784 | TSVNQAsTSRL | 0.066 | -0.235 | -1.454 | -0.541 |
| sp\|O75899\|GABR2_HUMAN Gamma-aminobutyric acid type B receptor subunit 2 OS=Homo sapiens GN=GAE | 785 | SVNQAStSRLE | 0.096 | -0.218 | -1.268 | -0.463 |
| sp\|O75899\|GABR2_HUMAN Gamma-aminobutyric acid type B receptor subunit 2 OS=Homo sapiens GN=GAE | 786 | VNQASTsRLEG | 0.24 | -0.004 | -0.632 | -0.132 |
| sp\|O75899\|GABR2_HUMAN Gamma-aminobutyric acid type B receptor subunit 2 OS=Homo sapiens GN=GAE | 793 | RLEGLQsENHR | 0.302 | 0.108 | -0.265 | 0.048 |
| sp\|O75899\|GABR2_HUMAN Gamma-aminobutyric acid type B receptor subunit 2 OS=Homo sapiens GN=GAE | 803 | RLRMKItELDK | 0.624 | 0.316 | -0.049 | 0.297 |
| sp\|O75899\|GABR2_HUMAN Gamma-aminobutyric acid type B receptor subunit 2 OS=Homo sapiens GN=GAE | 813 | KDLEEVtMQLQ | 0.106 | -0.135 | -1.32 | -0.45 |
| sp\|O75899\|GABR2_HUMAN Gamma-aminobutyric acid type B receptor subunit 2 OS=Homo sapiens GN=GAE | 819 | TMQLQDtPEKT | 0.032 | -0.386 | -2.022 | -0.792 |
| sp\|O75899\|GABR2_HUMAN Gamma-aminobutyric acid type B receptor subunit 2 OS=Homo sapiens GN=GAE | 823 | QDTPEKtTYIK | 0.124 | -0.236 | -0.947 | -0.353 |
| sp\|O75899\|GABR2_HUMAN Gamma-aminobutyric acid type B receptor subunit 2 OS=Homo sapiens GN=GAE | 824 | DTPEKTtYIKQ | 0.039 | -0.336 | -1.774 | -0.69 |
| sp\|O75899\|GABR2_HUMAN Gamma-aminobutyric acid type B receptor subunit 2 OS=Homo sapiens GN=GAE | 844 | LNLGNFtESTD | 0.152 | -0.08 | -0.535 | -0.154 |
| sp\|O75899\|GABR2_HUMAN Gamma-aminobutyric acid type B receptor subunit 2 OS=Homo sapiens GN=GAE | 846 | LGNFTEsTDGG | 0.149 | -0.089 | -0.926 | -0.289 |
| sp\|O75899\|GABR2_HUMAN Gamma-aminobutyric acid type B receptor subunit 2 OS=Homo sapiens GN=GAE | 847 | GNFTEStDGGK | 0.202 | -0.204 | -1.069 | -0.357 |
| sp\|O75899\|GABR2_HUMAN Gamma-aminobutyric acid type B receptor subunit 2 OS=Homo sapiens GN=GAE | 868 | PQLQWNtTEPS | 0.205 | -0.044 | -0.634 | -0.158 |
| sp\|O75899\|GABR2_HUMAN Gamma-aminobutyric acid type B receptor subunit 2 OS=Homo sapiens GN=GAE | 869 | QLQWNTtEPSR | 0.717 | 0.616 | 0.562 | 0.632 |
| sp\|O75899\|GABR2_HUMAN Gamma-aminobutyric acid type B receptor subunit 2 OS=Homo sapiens GN=GAE | 872 | WNTTEPsRTCK | 0.109 | -0.375 | -1.307 | -0.524 |
| sp\|O75899\|GABR2_HUMAN Gamma-aminobutyric acid type B receptor subunit 2 OS=Homo sapiens GN=GAE | 874 | TTEPSRtCKDP | 0.249 | 0.099 | -0.656 | -0.103 |
| sp\|O75899\|GABR2_HUMAN Gamma-aminobutyric acid type B receptor subunit 2 OS=Homo sapiens GN=GAE | 884 | PIEDINsPEHI | 0.184 | -0.192 | -0.732 | -0.247 |
| sp\|O75899\|GABR2_HUMAN Gamma-aminobutyric acid type B receptor subunit 2 OS=Homo sapiens GN=GAE | 893 | HIQRRLsLQLP | 0.811 | 1.212 | 0.987 | 1.003 |
| sp\|O75899\|GABR2_HUMAN Gamma-aminobutyric acid type B receptor subunit 2 OS=Homo sapiens GN=GAE | 906 | HHAYLPsIGGV | 0.128 | -0.101 | -1.021 | -0.331 |
| sp\|O75899\|GABR2_HUMAN Gamma-aminobutyric acid type B receptor subunit 2 OS=Homo sapiens GN=GAE | 913 | IGGVDAsCVSP | 0.065 | -0.15 | -1.36 | -0.482 |
| sp\|O75899\|GABR2_HUMAN Gamma-aminobutyric acid type B receptor subunit 2 OS=Homo sapiens GN=GAE | 916 | VDASCVsPCVS | 0.066 | -0.575 | -1.474 | -0.661 |
| sp\|O75899\|GABR2_HUMAN Gamma-aminobutyric acid type B receptor subunit 2 OS=Homo sapiens GN=GAE | 920 | CVSPCVsPTAS | 0.234 | -0.303 | -0.722 | -0.264 |
| sp\|O75899\|GABR2_HUMAN Gamma-aminobutyric acid type B receptor subunit 2 OS=Homo sapiens GN=GAE | 922 | SPCVSPtASPR | 0.107 | -0.072 | -1.235 | -0.4 |
| sp\|O75899\|GABR2_HUMAN Gamma-aminobutyric acid type B receptor subunit 2 OS=Homo sapiens GN=GAE | 924 | CVSPTAsPRHR | 0.062 | -0.417 | -1.129 | -0.495 |
| sp\|O75899\|GABR2_HUMAN Gamma-aminobutyric acid type B receptor subunit 2 OS=Homo sapiens GN=GAE | 933 | HRHVPPsFRVM | 0.129 | 0.065 | -0.696 | -0.167 |
| sp\|O75899\|GABR2_HUMAN Gamma-aminobutyric acid type B receptor subunit 2 OS=Homo sapiens GN=GAE | 939 | SFRVMVsGL-- | 0.237 | 0.152 | -0.704 | -0.105 |
| sp\|Q13255\|GRM1_HUMAN Metabotropic glutamate receptor 1 OS=Homo sapiens GN=GRM1 PE=1 SV=3 | 317 | EFSLIGsDGWA | 0.184 | -0.058 | -0.628 | -0.167 |
| sp\|Q13255\|GRM1_HUMAN Metabotropic glutamate receptor 1 OS=Homo sapiens GN=GRM1 PE=1 SV=3 | 339 | EANGGItIKLQ | 0.045 | -0.29 | -1.401 | -0.549 |
| sp\|Q13255\|GRM1_HUMAN Metabotropic glutamate receptor 1 OS=Homo sapiens GN=GRM1 PE=1 SV=3 | 344 | ITIKLQsPEVR | 0.141 | -0.28 | -1.106 | -0.415 |
| sp\|Q13255\|GRM1_HUMAN Metabotropic glutamate receptor 1 OS=Homo sapiens GN=GRM1 PE=1 SV=3 | 349 | QSPEVRsFDDY | 0.211 | -0.008 | -0.915 | -0.237 |
| sp\|Q13255\|GRM1_HUMAN Metabotropic glutamate receptor 1 OS=Homo sapiens GN=GRM1 PE=1 SV=3 | 361 | LKLRLDtNTRN | 0.652 | 0.671 | -0.163 | 0.387 |
| sp\|Q13255\|GRM1_HUMAN Metabotropic glutamate receptor 1 OS=Homo sapiens GN=GRM1 PE=1 SV=3 | 363 | LRLDTNtRNPW | 0.182 | 0.169 | -0.767 | -0.139 |
| sp\|Q13255\|GRM1_HUMAN Metabotropic glutamate receptor 1 OS=Homo sapiens GN=GRM1 PE=1 SV=3 | 395 | NFKRICtGNES | 0.799 | 0.902 | 0.782 | 0.828 |
| sp\|Q13255\|GRM1_HUMAN Metabotropic glutamate receptor 1 OS=Homo sapiens GN=GRM1 PE=1 SV=3 | 399 | ICTGNEsLEEN | 0.477 | 0.091 | -0.303 | 0.088 |
| sp\|Q13255\|GRM1_HUMAN Metabotropic glutamate receptor 1 OS=Homo sapiens GN=GRM1 PE=1 SV=3 | 408 | ENYVQDsKMGF | 0.037 | -0.336 | -1.505 | -0.601 |
| sp\|Q13255\|GRM1_HUMAN Metabotropic glutamate receptor 1 OS=Homo sapiens GN=GRM1 PE=1 SV=3 | 448 | MKPIDGsKLLD | 0.169 | -0.033 | -0.886 | -0.25 |
| sp\|Q13255\|GRM1_HUMAN Metabotropic glutamate receptor 1 OS=Homo sapiens GN=GRM1 PE=1 SV=3 | 457 | LDFLIKsSFIG | 0.183 | -0.084 | -0.613 | -0.171 |
| sp\|Q13255\|GRM1_HUMAN Metabotropic glutamate receptor 1 OS=Homo sapiens GN=GRM1 PE=1 SV=3 | 458 | DFLIKSsFIGV | 0.15 | -0.101 | -0.909 | -0.287 |
| sp\|Q13255\|GRM1_HUMAN Metabotropic glutamate receptor 1 OS=Homo sapiens GN=GRM1 PE=1 SV=3 | 463 | SSFIGVsGEEV | 0.103 | -0.229 | -1.444 | -0.523 |
| sp\|Q13255\|GRM1_HUMAN Metabotropic glutamate receptor 1 OS=Homo sapiens GN=GRM1 PE=1 SV=3 | 487 | IMNLQYtEANR | 0.135 | 0.182 | -0.979 | -0.221 |
| sp\|Q13255\|GRM1_HUMAN Metabotropic glutamate receptor 1 OS=Homo sapiens GN=GRM1 PE=1 SV=3 | 499 | DYVHVGtWHEG | 0.263 | 0.015 | -0.976 | -0.233 |
| sp\|Q13255\|GRM1_HUMAN Metabotropic glutamate receptor 1 OS=Homo sapiens GN=GRM1 PE=1 SV=3 | 517 | KIQMNKsGVVR | 0.316 | 0.068 | -0.345 | 0.013 |
| sp\|Q13255\|GRM1_HUMAN Metabotropic glutamate receptor 1 OS=Homo sapiens GN=GRM1 PE=1 SV=3 | 522 | KSGVVRsVCSE | 0.03 | -0.372 | -1.976 | -0.773 |
| sp\|Q13255\|GRM1_HUMAN Metabotropic glutamate receptor 1 OS=Homo sapiens GN=GRM1 PE=1 SV=3 | 525 | VVRSVCsEPCL | 0.795 | 0.8 | 0.285 | 0.627 |
| sp\|Q13255\|GRM1_HUMAN Metabotropic glutamate receptor 1 OS=Homo sapiens GN=GRM1 PE=1 SV=3 | 542 | IRKGEVsCCWI | 0.497 | 0.245 | -0.116 | 0.209 |
| sp\|Q13255\|GRM1_HUMAN Metabotropic glutamate receptor 1 OS=Homo sapiens GN=GRM1 PE=1 SV=3 | 548 | SCCWICtACKE | 0.201 | -0.167 | -1.304 | -0.423 |

| | | | | | | |
|---|---|---|---|---|---|---|
| sp\|Q13255\|GRM1_HUMAN Metabotropic glutamate receptor 1 OS=Homo sapiens GN=GRM1 PE=1 SV=3 | 561 | YVQDEFtCKAC | 0.169 | 0.098 | -0.925 | -0.219 |
| sp\|Q13255\|GRM1_HUMAN Metabotropic glutamate receptor 1 OS=Homo sapiens GN=GRM1 PE=1 SV=3 | 576 | WPNADLtGCEP | 0.044 | -0.438 | -1.651 | -0.682 |
| sp\|Q13255\|GRM1_HUMAN Metabotropic glutamate receptor 1 OS=Homo sapiens GN=GRM1 PE=1 SV=3 | 589 | VRYLEWsNIES | 0.126 | 0.017 | -1.178 | -0.345 |
| sp\|Q13255\|GRM1_HUMAN Metabotropic glutamate receptor 1 OS=Homo sapiens GN=GRM1 PE=1 SV=3 | 593 | EWSNIEsIIAI | 0.114 | -0.017 | -0.435 | -0.113 |
| sp\|Q13255\|GRM1_HUMAN Metabotropic glutamate receptor 1 OS=Homo sapiens GN=GRM1 PE=1 SV=3 | 600 | IIAIAFsCLGI | 0.402 | 0.234 | -0.063 | 0.191 |
| sp\|Q13255\|GRM1_HUMAN Metabotropic glutamate receptor 1 OS=Homo sapiens GN=GRM1 PE=1 SV=3 | 607 | CLGILVtLFVT | 0.445 | 0.14 | -0.152 | 0.144 |
| sp\|Q13255\|GRM1_HUMAN Metabotropic glutamate receptor 1 OS=Homo sapiens GN=GRM1 PE=1 SV=3 | 611 | LVTLFVtLIFV | 0.251 | -0.02 | -0.669 | -0.146 |
| sp\|Q13255\|GRM1_HUMAN Metabotropic glutamate receptor 1 OS=Homo sapiens GN=GRM1 PE=1 SV=3 | 620 | FVLYRDtPVVK | 0.14 | -0.158 | -0.974 | -0.331 |
| sp\|Q13255\|GRM1_HUMAN Metabotropic glutamate receptor 1 OS=Homo sapiens GN=GRM1 PE=1 SV=3 | 625 | DTPVVKsSSRE | 0.1 | -0.319 | -1.593 | -0.604 |
| sp\|Q13255\|GRM1_HUMAN Metabotropic glutamate receptor 1 OS=Homo sapiens GN=GRM1 PE=1 SV=3 | 626 | TPVVKSsSREL | 0.043 | -0.301 | -1.596 | -0.618 |
| sp\|Q13255\|GRM1_HUMAN Metabotropic glutamate receptor 1 OS=Homo sapiens GN=GRM1 PE=1 SV=3 | 627 | PVVKSSsRELC | 0.208 | 0.044 | -0.837 | -0.195 |
| sp\|Q13255\|GRM1_HUMAN Metabotropic glutamate receptor 1 OS=Homo sapiens GN=GRM1 PE=1 SV=3 | 647 | GYVCPFtLIAK | 0.48 | 0.125 | -0.024 | 0.194 |
| sp\|Q13255\|GRM1_HUMAN Metabotropic glutamate receptor 1 OS=Homo sapiens GN=GRM1 PE=1 SV=3 | 653 | TLIAKPtTTSC | 0.083 | -0.251 | -1.464 | -0.544 |
| sp\|Q13255\|GRM1_HUMAN Metabotropic glutamate receptor 1 OS=Homo sapiens GN=GRM1 PE=1 SV=3 | 654 | LIAKPTtTSCY | 0.232 | 0.011 | -0.576 | -0.111 |
| sp\|Q13255\|GRM1_HUMAN Metabotropic glutamate receptor 1 OS=Homo sapiens GN=GRM1 PE=1 SV=3 | 655 | IAKPTTtSCYL | 0.097 | -0.206 | -1.057 | -0.389 |
| sp\|Q13255\|GRM1_HUMAN Metabotropic glutamate receptor 1 OS=Homo sapiens GN=GRM1 PE=1 SV=3 | 656 | AKPTTTsCYLQ | 0.154 | 0.01 | -1.026 | -0.287 |
| sp\|Q13255\|GRM1_HUMAN Metabotropic glutamate receptor 1 OS=Homo sapiens GN=GRM1 PE=1 SV=3 | 667 | RLLVGLsSAMC | 0.046 | -0.274 | -1.669 | -0.632 |
| sp\|Q13255\|GRM1_HUMAN Metabotropic glutamate receptor 1 OS=Homo sapiens GN=GRM1 PE=1 SV=3 | 668 | LLVGLSsAMCY | 0.157 | 0.06 | -1.057 | -0.28 |
| sp\|Q13255\|GRM1_HUMAN Metabotropic glutamate receptor 1 OS=Homo sapiens GN=GRM1 PE=1 SV=3 | 673 | SSAMCYsALVT | 0.128 | -0.107 | -0.984 | -0.321 |
| sp\|Q13255\|GRM1_HUMAN Metabotropic glutamate receptor 1 OS=Homo sapiens GN=GRM1 PE=1 SV=3 | 677 | CYSALVtKTNR | 0.428 | -0.025 | -0.525 | -0.041 |
| sp\|Q13255\|GRM1_HUMAN Metabotropic glutamate receptor 1 OS=Homo sapiens GN=GRM1 PE=1 SV=3 | 679 | SALVTKtNRIA | 0.098 | -0.209 | -1.158 | -0.423 |
| sp\|Q13255\|GRM1_HUMAN Metabotropic glutamate receptor 1 OS=Homo sapiens GN=GRM1 PE=1 SV=3 | 689 | ARILAGsKKKI | 0.234 | 0.171 | -0.688 | -0.094 |
| sp\|Q13255\|GRM1_HUMAN Metabotropic glutamate receptor 1 OS=Homo sapiens GN=GRM1 PE=1 SV=3 | 695 | SKKKICtRKPR | 0.409 | -0.059 | -0.448 | -0.033 |
| sp\|Q13255\|GRM1_HUMAN Metabotropic glutamate receptor 1 OS=Homo sapiens GN=GRM1 PE=1 SV=3 | 702 | RKPRFMsAWAQ | 0.871 | 1.133 | 0.637 | 0.88 |
| sp\|Q13255\|GRM1_HUMAN Metabotropic glutamate receptor 1 OS=Homo sapiens GN=GRM1 PE=1 SV=3 | 711 | AQVIIAsILIS | 0.088 | -0.148 | -1.113 | -0.391 |
| sp\|Q13255\|GRM1_HUMAN Metabotropic glutamate receptor 1 OS=Homo sapiens GN=GRM1 PE=1 SV=3 | 715 | IASILIsVQLT | 0.137 | -0.163 | -0.924 | -0.317 |
| sp\|Q13255\|GRM1_HUMAN Metabotropic glutamate receptor 1 OS=Homo sapiens GN=GRM1 PE=1 SV=3 | 719 | LISVQLtLVVT | 0.082 | -0.009 | -0.987 | -0.305 |
| sp\|Q13255\|GRM1_HUMAN Metabotropic glutamate receptor 1 OS=Homo sapiens GN=GRM1 PE=1 SV=3 | 723 | QLTLVVtLIIM | 0.588 | 0.134 | 0.085 | 0.269 |
| sp\|Q13255\|GRM1_HUMAN Metabotropic glutamate receptor 1 OS=Homo sapiens GN=GRM1 PE=1 SV=3 | 735 | PPMPILsYPSI | 0.24 | 0.348 | -0.226 | 0.121 |
| sp\|Q13255\|GRM1_HUMAN Metabotropic glutamate receptor 1 OS=Homo sapiens GN=GRM1 PE=1 SV=3 | 738 | PILSYPsIKEV | 0.195 | -0.088 | -0.895 | -0.263 |
| sp\|Q13255\|GRM1_HUMAN Metabotropic glutamate receptor 1 OS=Homo sapiens GN=GRM1 PE=1 SV=3 | 748 | VYLICNtSNLG | 0.4 | 0.078 | -0.447 | 0.01 |
| sp\|Q13255\|GRM1_HUMAN Metabotropic glutamate receptor 1 OS=Homo sapiens GN=GRM1 PE=1 SV=3 | 749 | YLICNTsNLGV | 0.258 | -0.026 | -0.927 | -0.232 |
| sp\|Q13255\|GRM1_HUMAN Metabotropic glutamate receptor 1 OS=Homo sapiens GN=GRM1 PE=1 SV=3 | 766 | NGLLIMsCTYY | 0.381 | 0.148 | -0.459 | 0.023 |
| sp\|Q13255\|GRM1_HUMAN Metabotropic glutamate receptor 1 OS=Homo sapiens GN=GRM1 PE=1 SV=3 | 768 | LLIMSCtYYAF | 0.458 | 0.282 | -0.094 | 0.215 |
| sp\|Q13255\|GRM1_HUMAN Metabotropic glutamate receptor 1 OS=Homo sapiens GN=GRM1 PE=1 SV=3 | 774 | TYYAFKtRNVP | 0.178 | -0.107 | -0.799 | -0.243 |
| sp\|Q13255\|GRM1_HUMAN Metabotropic glutamate receptor 1 OS=Homo sapiens GN=GRM1 PE=1 SV=3 | 790 | AKYIAFtMYTT | 0.286 | 0.172 | -0.511 | -0.018 |
| sp\|Q13255\|GRM1_HUMAN Metabotropic glutamate receptor 1 OS=Homo sapiens GN=GRM1 PE=1 SV=3 | 793 | IAFTMYtTCII | 0.263 | -0.131 | -0.623 | -0.164 |
| sp\|Q13255\|GRM1_HUMAN Metabotropic glutamate receptor 1 OS=Homo sapiens GN=GRM1 PE=1 SV=3 | 794 | AFTMYTtCIIW | 0.258 | 0.023 | -0.489 | -0.069 |
| sp\|Q13255\|GRM1_HUMAN Metabotropic glutamate receptor 1 OS=Homo sapiens GN=GRM1 PE=1 SV=3 | 808 | VPIYFGsNYKI | 0.095 | -0.161 | -1.327 | -0.464 |
| sp\|Q13255\|GRM1_HUMAN Metabotropic glutamate receptor 1 OS=Homo sapiens GN=GRM1 PE=1 SV=3 | 814 | SNYKIItTCFA | 0.087 | -0.345 | -1.167 | -0.475 |
| sp\|Q13255\|GRM1_HUMAN Metabotropic glutamate receptor 1 OS=Homo sapiens GN=GRM1 PE=1 SV=3 | 815 | NYKIITtCFAV | 0.416 | 0.232 | -0.228 | 0.14 |
| sp\|Q13255\|GRM1_HUMAN Metabotropic glutamate receptor 1 OS=Homo sapiens GN=GRM1 PE=1 SV=3 | 820 | TTCFAVsLSVT | 0.48 | 0.043 | -0.377 | 0.049 |
| sp\|Q13255\|GRM1_HUMAN Metabotropic glutamate receptor 1 OS=Homo sapiens GN=GRM1 PE=1 SV=3 | 822 | CFAVSLsVTVA | 0.328 | 0.182 | -0.132 | 0.126 |
| sp\|Q13255\|GRM1_HUMAN Metabotropic glutamate receptor 1 OS=Homo sapiens GN=GRM1 PE=1 SV=3 | 824 | AVSLSVtVALG | 0.364 | 0.111 | -0.419 | 0.019 |
| sp\|Q13255\|GRM1_HUMAN Metabotropic glutamate receptor 1 OS=Homo sapiens GN=GRM1 PE=1 SV=3 | 832 | ALGCMFtPKMY | 0.143 | -0.356 | -1.188 | -0.467 |
| sp\|Q13255\|GRM1_HUMAN Metabotropic glutamate receptor 1 OS=Homo sapiens GN=GRM1 PE=1 SV=3 | 848 | PERNVRsAFTT | 0.282 | 0.157 | -0.735 | -0.099 |
| sp\|Q13255\|GRM1_HUMAN Metabotropic glutamate receptor 1 OS=Homo sapiens GN=GRM1 PE=1 SV=3 | 851 | NVRSAFtTSDV | 0.418 | 0.263 | -0.476 | 0.068 |
| sp\|Q13255\|GRM1_HUMAN Metabotropic glutamate receptor 1 OS=Homo sapiens GN=GRM1 PE=1 SV=3 | 852 | VRSAFTtSDVV | 0.216 | 0.098 | -0.95 | -0.212 |
| sp\|Q13255\|GRM1_HUMAN Metabotropic glutamate receptor 1 OS=Homo sapiens GN=GRM1 PE=1 SV=3 | 853 | RSAFTTsDVVR | 0.22 | 0.074 | -0.692 | -0.133 |

| | | | | | | |
|---|---|---|---|---|---|---|
| sp\|Q13255\|GRM1_HUMAN Metabotropic glutamate receptor 1 OS=Homo sapiens GN=GRM1 PE=1 SV=3 | 869 | GKLPCRsNTFL | 0.316 | -0.048 | -0.845 | -0.192 |
| sp\|Q13255\|GRM1_HUMAN Metabotropic glutamate receptor 1 OS=Homo sapiens GN=GRM1 PE=1 SV=3 | 871 | LPCRSNtFLNI | 0.881 | 1.222 | 0.931 | 1.011 |
| sp\|Q13255\|GRM1_HUMAN Metabotropic glutamate receptor 1 OS=Homo sapiens GN=GRM1 PE=1 SV=3 | 888 | GAGNANsNGKS | 0.219 | 0.007 | -0.933 | -0.236 |
| sp\|Q13255\|GRM1_HUMAN Metabotropic glutamate receptor 1 OS=Homo sapiens GN=GRM1 PE=1 SV=3 | 892 | ANSNGKsVSWS | 0.058 | -0.261 | -1.07 | -0.424 |
| sp\|Q13255\|GRM1_HUMAN Metabotropic glutamate receptor 1 OS=Homo sapiens GN=GRM1 PE=1 SV=3 | 894 | SNGKSVsWSEP | 0.233 | 0.058 | -0.671 | -0.127 |
| sp\|Q13255\|GRM1_HUMAN Metabotropic glutamate receptor 1 OS=Homo sapiens GN=GRM1 PE=1 SV=3 | 896 | GKSVSWsEPGG | 0.872 | 0.75 | 0.816 | 0.813 |
| sp\|Q13255\|GRM1_HUMAN Metabotropic glutamate receptor 1 OS=Homo sapiens GN=GRM1 PE=1 SV=3 | 914 | HMWHRLsVHVK | 0.179 | 0.345 | -0.583 | -0.02 |
| sp\|Q13255\|GRM1_HUMAN Metabotropic glutamate receptor 1 OS=Homo sapiens GN=GRM1 PE=1 SV=3 | 919 | LSVHVKtNETA | 0.154 | -0.178 | -0.878 | -0.301 |
| sp\|Q13255\|GRM1_HUMAN Metabotropic glutamate receptor 1 OS=Homo sapiens GN=GRM1 PE=1 SV=3 | 922 | HVKTNEtACNQ | 0.145 | -0.07 | -1.199 | -0.375 |
| sp\|Q13255\|GRM1_HUMAN Metabotropic glutamate receptor 1 OS=Homo sapiens GN=GRM1 PE=1 SV=3 | 927 | ETACNQtAVIK | 0.264 | -0.081 | -0.767 | -0.195 |
| sp\|Q13255\|GRM1_HUMAN Metabotropic glutamate receptor 1 OS=Homo sapiens GN=GRM1 PE=1 SV=3 | 934 | AVIKPLtKSYQ | 0.073 | -0.272 | -1.364 | -0.521 |
| sp\|Q13255\|GRM1_HUMAN Metabotropic glutamate receptor 1 OS=Homo sapiens GN=GRM1 PE=1 SV=3 | 936 | IKPLTKsYQGS | 0.225 | -0.035 | -0.525 | -0.112 |
| sp\|Q13255\|GRM1_HUMAN Metabotropic glutamate receptor 1 OS=Homo sapiens GN=GRM1 PE=1 SV=3 | 940 | TKSYQGsGKSL | 0.068 | -0.162 | -1.151 | -0.415 |
| sp\|Q13255\|GRM1_HUMAN Metabotropic glutamate receptor 1 OS=Homo sapiens GN=GRM1 PE=1 SV=3 | 943 | YQGSGKsLTFS | 0.108 | -0.152 | -0.786 | -0.277 |
| sp\|Q13255\|GRM1_HUMAN Metabotropic glutamate receptor 1 OS=Homo sapiens GN=GRM1 PE=1 SV=3 | 945 | GSGKSLtFSDT | 0.419 | 0.178 | -0.203 | 0.131 |
| sp\|Q13255\|GRM1_HUMAN Metabotropic glutamate receptor 1 OS=Homo sapiens GN=GRM1 PE=1 SV=3 | 947 | GKSLTFsDTST | 0.24 | 0.05 | -0.801 | -0.17 |
| sp\|Q13255\|GRM1_HUMAN Metabotropic glutamate receptor 1 OS=Homo sapiens GN=GRM1 PE=1 SV=3 | 949 | SLTFSDtSTKT | 0.272 | 0.042 | -0.732 | -0.139 |
| sp\|Q13255\|GRM1_HUMAN Metabotropic glutamate receptor 1 OS=Homo sapiens GN=GRM1 PE=1 SV=3 | 950 | LTFSDTsTKTL | 0.122 | -0.174 | -1.009 | -0.354 |
| sp\|Q13255\|GRM1_HUMAN Metabotropic glutamate receptor 1 OS=Homo sapiens GN=GRM1 PE=1 SV=3 | 951 | TFSDTStKTLY | 0.148 | -0.07 | -0.977 | -0.3 |
| sp\|Q13255\|GRM1_HUMAN Metabotropic glutamate receptor 1 OS=Homo sapiens GN=GRM1 PE=1 SV=3 | 953 | SDTSTKtLYNV | 0.148 | 0.002 | -0.964 | -0.271 |
| sp\|Q13255\|GRM1_HUMAN Metabotropic glutamate receptor 1 OS=Homo sapiens GN=GRM1 PE=1 SV=3 | 969 | AQPIRFsPPGS | 0.287 | 0.196 | -0.422 | 0.02 |
| sp\|Q13255\|GRM1_HUMAN Metabotropic glutamate receptor 1 OS=Homo sapiens GN=GRM1 PE=1 SV=3 | 973 | RFSPPGsPSMV | 0.03 | -0.636 | -1.927 | -0.844 |
| sp\|Q13255\|GRM1_HUMAN Metabotropic glutamate receptor 1 OS=Homo sapiens GN=GRM1 PE=1 SV=3 | 975 | SPPGSPsMVVH | 0.071 | -0.031 | -1.409 | -0.456 |
| sp\|Q13255\|GRM1_HUMAN Metabotropic glutamate receptor 1 OS=Homo sapiens GN=GRM1 PE=1 SV=3 | 984 | VHRRVPsAATT | 0.62 | 0.881 | -0.046 | 0.485 |
| sp\|Q13255\|GRM1_HUMAN Metabotropic glutamate receptor 1 OS=Homo sapiens GN=GRM1 PE=1 SV=3 | 987 | RVPSAAtTPPL | 0.522 | 0.467 | -0.129 | 0.287 |
| sp\|Q13255\|GRM1_HUMAN Metabotropic glutamate receptor 1 OS=Homo sapiens GN=GRM1 PE=1 SV=3 | 988 | VPSAATtPPLP | 0.262 | 0.058 | -0.567 | -0.082 |
| sp\|Q13255\|GRM1_HUMAN Metabotropic glutamate receptor 1 OS=Homo sapiens GN=GRM1 PE=1 SV=3 | 993 | TTPPLPsHLTA | 0.057 | -0.387 | -1.56 | -0.63 |
| sp\|Q13255\|GRM1_HUMAN Metabotropic glutamate receptor 1 OS=Homo sapiens GN=GRM1 PE=1 SV=3 | 996 | PLPSHLtAEET | 0.172 | -0.027 | -1.02 | -0.292 |
| sp\|Q13255\|GRM1_HUMAN Metabotropic glutamate receptor 1 OS=Homo sapiens GN=GRM1 PE=1 SV=3 | 1000 | HLTAEEtPLFL | 0.064 | -0.347 | -1.457 | -0.58 |
| sp\|Q13255\|GRM1_HUMAN Metabotropic glutamate receptor 1 OS=Homo sapiens GN=GRM1 PE=1 SV=3 | 1029 | PPPQQKsLMDQ | 0.399 | 0.059 | -0.318 | 0.047 |
| sp\|Q13255\|GRM1_HUMAN Metabotropic glutamate receptor 1 OS=Homo sapiens GN=GRM1 PE=1 SV=3 | 1039 | QLQGVVsNFST | 0.21 | -0.029 | -0.751 | -0.19 |
| sp\|Q13255\|GRM1_HUMAN Metabotropic glutamate receptor 1 OS=Homo sapiens GN=GRM1 PE=1 SV=3 | 1042 | GVVSNFsTAIP | 0.117 | -0.152 | -1.075 | -0.37 |
| sp\|Q13255\|GRM1_HUMAN Metabotropic glutamate receptor 1 OS=Homo sapiens GN=GRM1 PE=1 SV=3 | 1043 | VVSNFStAIPD | 0.067 | -0.192 | -1.15 | -0.425 |
| sp\|Q13255\|GRM1_HUMAN Metabotropic glutamate receptor 1 OS=Homo sapiens GN=GRM1 PE=1 SV=3 | 1064 | PGNGLRsLYPP | 0.097 | -0.131 | -1.111 | -0.382 |
| sp\|Q13255\|GRM1_HUMAN Metabotropic glutamate receptor 1 OS=Homo sapiens GN=GRM1 PE=1 SV=3 | 1083 | MLPLQLsTFGE | 0.131 | -0.062 | -1.04 | -0.324 |
| sp\|Q13255\|GRM1_HUMAN Metabotropic glutamate receptor 1 OS=Homo sapiens GN=GRM1 PE=1 SV=3 | 1084 | LPLQLStFGEE | 0.203 | -0.091 | -0.67 | -0.186 |
| sp\|Q13255\|GRM1_HUMAN Metabotropic glutamate receptor 1 OS=Homo sapiens GN=GRM1 PE=1 SV=3 | 1091 | FGEELVsPPAD | 0.464 | 0.254 | -0.088 | 0.21 |
| sp\|Q13255\|GRM1_HUMAN Metabotropic glutamate receptor 1 OS=Homo sapiens GN=GRM1 PE=1 SV=3 | 1100 | ADDDDDsERFK | 0.068 | -0.42 | -1.671 | -0.674 |
| sp\|Q13255\|GRM1_HUMAN Metabotropic glutamate receptor 1 OS=Homo sapiens GN=GRM1 PE=1 SV=3 | 1119 | HEREGNtEEDE | 0.326 | 0.299 | -0.565 | 0.02 |
| sp\|Q13255\|GRM1_HUMAN Metabotropic glutamate receptor 1 OS=Homo sapiens GN=GRM1 PE=1 SV=3 | 1135 | EDLQAAsKLTP | 0.119 | -0.157 | -0.877 | -0.305 |
| sp\|Q13255\|GRM1_HUMAN Metabotropic glutamate receptor 1 OS=Homo sapiens GN=GRM1 PE=1 SV=3 | 1138 | QAASKLtPDDS | 0.044 | -0.48 | -1.48 | -0.639 |
| sp\|Q13255\|GRM1_HUMAN Metabotropic glutamate receptor 1 OS=Homo sapiens GN=GRM1 PE=1 SV=3 | 1142 | KLTPDDsPALT | 0.055 | -0.524 | -1.519 | -0.663 |
| sp\|Q13255\|GRM1_HUMAN Metabotropic glutamate receptor 1 OS=Homo sapiens GN=GRM1 PE=1 SV=3 | 1146 | DDSPALtPPSP | 0.087 | -0.103 | -1.167 | -0.394 |
| sp\|Q13255\|GRM1_HUMAN Metabotropic glutamate receptor 1 OS=Homo sapiens GN=GRM1 PE=1 SV=3 | 1149 | PALTPPsPFRD | 0.09 | -0.561 | -1.314 | -0.595 |
| sp\|Q13255\|GRM1_HUMAN Metabotropic glutamate receptor 1 OS=Homo sapiens GN=GRM1 PE=1 SV=3 | 1154 | PSPFRDsVASG | 0.154 | -0.12 | -1.123 | -0.363 |
| sp\|Q13255\|GRM1_HUMAN Metabotropic glutamate receptor 1 OS=Homo sapiens GN=GRM1 PE=1 SV=3 | 1157 | FRDSVAsGSSV | 0.104 | 0.042 | -1.39 | -0.415 |
| sp\|Q13255\|GRM1_HUMAN Metabotropic glutamate receptor 1 OS=Homo sapiens GN=GRM1 PE=1 SV=3 | 1159 | DSVASGsSVPS | 0.124 | -0.132 | -1.169 | -0.392 |
| sp\|Q13255\|GRM1_HUMAN Metabotropic glutamate receptor 1 OS=Homo sapiens GN=GRM1 PE=1 SV=3 | 1160 | SVASGSsVPSS | 0.079 | 0.149 | -1.03 | -0.267 |
| sp\|Q13255\|GRM1_HUMAN Metabotropic glutamate receptor 1 OS=Homo sapiens GN=GRM1 PE=1 SV=3 | 1163 | SGSSVPsSPVS | 0.23 | 0.185 | -0.783 | -0.123 |

| | | | | | | |
|---|---|---|---|---|---|---|
| sp\|Q13255\|GRM1_HUMAN Metabotropic glutamate receptor 1 OS=Homo sapiens GN=GRM1 PE=1 SV=3 | 1164 | GSSVPSsPVSE | 0.023 | -0.648 | -2.012 | -0.879 |
| sp\|Q13255\|GRM1_HUMAN Metabotropic glutamate receptor 1 OS=Homo sapiens GN=GRM1 PE=1 SV=3 | 1167 | VPSSPVsESVL | 0.125 | -0.196 | -1.01 | -0.36 |
| sp\|Q13255\|GRM1_HUMAN Metabotropic glutamate receptor 1 OS=Homo sapiens GN=GRM1 PE=1 SV=3 | 1169 | SSPVSEsVLCT | 0.06 | -0.093 | -1.445 | -0.493 |
| sp\|Q13255\|GRM1_HUMAN Metabotropic glutamate receptor 1 OS=Homo sapiens GN=GRM1 PE=1 SV=3 | 1173 | SESVLCtPPNV | 0.156 | 0.039 | -1.578 | -0.461 |
| sp\|Q13255\|GRM1_HUMAN Metabotropic glutamate receptor 1 OS=Homo sapiens GN=GRM1 PE=1 SV=3 | 1178 | CTPPNVsYASV | 0.112 | -0.28 | -1.273 | -0.48 |
| sp\|Q13255\|GRM1_HUMAN Metabotropic glutamate receptor 1 OS=Homo sapiens GN=GRM1 PE=1 SV=3 | 1181 | PNVSYAsVILR | 0.182 | -0.07 | -0.703 | -0.197 |
| sp\|Q13255\|GRM1_HUMAN Metabotropic glutamate receptor 1 OS=Homo sapiens GN=GRM1 PE=1 SV=3 | 1190 | LRDYKQsSSTL | 0.082 | 0.053 | -0.96 | -0.275 |
| sp\|Q13255\|GRM1_HUMAN Metabotropic glutamate receptor 1 OS=Homo sapiens GN=GRM1 PE=1 SV=3 | 1191 | RDYKQSsSTL- | 0.099 | -0.129 | -1.332 | -0.454 |
| sp\|Q13255\|GRM1_HUMAN Metabotropic glutamate receptor 1 OS=Homo sapiens GN=GRM1 PE=1 SV=3 | 1192 | DYKQSSsTL-- | 0.382 | 0.275 | -0.396 | 0.087 |
| sp\|Q13255\|GRM1_HUMAN Metabotropic glutamate receptor 1 OS=Homo sapiens GN=GRM1 PE=1 SV=3 | 1193 | YKQSSStL--- | 0.588 | 0.448 | 0.174 | 0.403 |
| sp\|Q14416\|GRM2_HUMAN Metabotropic glutamate receptor 2 OS=Homo sapiens GN=GRM2 PE=1 SV=2 | 316 | AAEGAItIELA | 0.179 | -0.13 | -0.738 | -0.23 |
| sp\|Q14416\|GRM2_HUMAN Metabotropic glutamate receptor 2 OS=Homo sapiens GN=GRM2 PE=1 SV=2 | 321 | ITIELAsYPIS | 0.398 | 0.344 | -0.546 | 0.065 |
| sp\|Q14416\|GRM2_HUMAN Metabotropic glutamate receptor 2 OS=Homo sapiens GN=GRM2 PE=1 SV=2 | 325 | LASYPIsDFAS | 0.192 | -0.083 | -0.588 | -0.16 |
| sp\|Q14416\|GRM2_HUMAN Metabotropic glutamate receptor 2 OS=Homo sapiens GN=GRM2 PE=1 SV=2 | 329 | PISDFAsYFQS | 0.242 | -0.029 | -0.866 | -0.218 |
| sp\|Q14416\|GRM2_HUMAN Metabotropic glutamate receptor 2 OS=Homo sapiens GN=GRM2 PE=1 SV=2 | 333 | FASYFQsLDPW | 0.323 | 0.09 | -0.337 | 0.025 |
| sp\|Q14416\|GRM2_HUMAN Metabotropic glutamate receptor 2 OS=Homo sapiens GN=GRM2 PE=1 SV=2 | 340 | LDPWNNsRNPW | 0.112 | -0.273 | -1.007 | -0.389 |
| sp\|Q14416\|GRM2_HUMAN Metabotropic glutamate receptor 2 OS=Homo sapiens GN=GRM2 PE=1 SV=2 | 356 | EQRFRCsFRQR | 0.729 | 0.383 | 0.317 | 0.476 |
| sp\|Q14416\|GRM2_HUMAN Metabotropic glutamate receptor 2 OS=Homo sapiens GN=GRM2 PE=1 SV=2 | 366 | RDCAAHsLRAV | 0.628 | 0.207 | -0.153 | 0.227 |
| sp\|Q14416\|GRM2_HUMAN Metabotropic glutamate receptor 2 OS=Homo sapiens GN=GRM2 PE=1 SV=2 | 376 | VPFEQEsKIMF | 0.042 | -0.279 | -1.665 | -0.634 |
| sp\|Q14416\|GRM2_HUMAN Metabotropic glutamate receptor 2 OS=Homo sapiens GN=GRM2 PE=1 SV=2 | 403 | RALCPNtTRLC | 0.213 | -0.22 | -1.123 | -0.377 |
| sp\|Q14416\|GRM2_HUMAN Metabotropic glutamate receptor 2 OS=Homo sapiens GN=GRM2 PE=1 SV=2 | 404 | ALCPNTtRLCD | 0.247 | -0.063 | -0.794 | -0.203 |
| sp\|Q14416\|GRM2_HUMAN Metabotropic glutamate receptor 2 OS=Homo sapiens GN=GRM2 PE=1 SV=2 | 437 | PFRPADtHNEV | 0.691 | 0.266 | -0.099 | 0.286 |
| sp\|Q14416\|GRM2_HUMAN Metabotropic glutamate receptor 2 OS=Homo sapiens GN=GRM2 PE=1 SV=2 | 457 | GRYNIFtYLRA | 0.15 | 0.086 | -0.717 | -0.16 |
| sp\|Q14416\|GRM2_HUMAN Metabotropic glutamate receptor 2 OS=Homo sapiens GN=GRM2 PE=1 SV=2 | 463 | TYLRAGsGRYR | 0.605 | 0.713 | 0.202 | 0.507 |
| sp\|Q14416\|GRM2_HUMAN Metabotropic glutamate receptor 2 OS=Homo sapiens GN=GRM2 PE=1 SV=2 | 479 | YWAEGLtLDTS | 0.1 | -0.045 | -0.806 | -0.25 |
| sp\|Q14416\|GRM2_HUMAN Metabotropic glutamate receptor 2 OS=Homo sapiens GN=GRM2 PE=1 SV=2 | 482 | EGLTLDtSLIP | 0.045 | -0.364 | -1.714 | -0.678 |
| sp\|Q14416\|GRM2_HUMAN Metabotropic glutamate receptor 2 OS=Homo sapiens GN=GRM2 PE=1 SV=2 | 483 | GLTLDTsLIPW | 0.366 | 0.128 | -0.189 | 0.102 |
| sp\|Q14416\|GRM2_HUMAN Metabotropic glutamate receptor 2 OS=Homo sapiens GN=GRM2 PE=1 SV=2 | 489 | SLIPWAsPSAG | 0.119 | -0.228 | -1.115 | -0.408 |
| sp\|Q14416\|GRM2_HUMAN Metabotropic glutamate receptor 2 OS=Homo sapiens GN=GRM2 PE=1 SV=2 | 491 | IPWASPsAGPL | 0.366 | 0.231 | -0.28 | 0.106 |
| sp\|Q14416\|GRM2_HUMAN Metabotropic glutamate receptor 2 OS=Homo sapiens GN=GRM2 PE=1 SV=2 | 498 | AGPLPAsRCSE | 0.046 | -0.471 | -1.766 | -0.73 |
| sp\|Q14416\|GRM2_HUMAN Metabotropic glutamate receptor 2 OS=Homo sapiens GN=GRM2 PE=1 SV=2 | 501 | LPASRCsEPCL | 0.74 | 0.693 | 0.295 | 0.576 |
| sp\|Q14416\|GRM2_HUMAN Metabotropic glutamate receptor 2 OS=Homo sapiens GN=GRM2 PE=1 SV=2 | 511 | LQNEVKsVQPG | 0.141 | -0.067 | -0.722 | -0.216 |
| sp\|Q14416\|GRM2_HUMAN Metabotropic glutamate receptor 2 OS=Homo sapiens GN=GRM2 PE=1 SV=2 | 536 | YRLDEFtCADC | 0.238 | 0.215 | -0.839 | -0.129 |
| sp\|Q14416\|GRM2_HUMAN Metabotropic glutamate receptor 2 OS=Homo sapiens GN=GRM2 PE=1 SV=2 | 549 | GYWPNAsLTGC | 0.487 | 0.113 | -0.331 | 0.09 |
| sp\|Q14416\|GRM2_HUMAN Metabotropic glutamate receptor 2 OS=Homo sapiens GN=GRM2 PE=1 SV=2 | 551 | WPNASLtGCFE | 0.076 | -0.246 | -1.242 | -0.471 |
| sp\|Q14416\|GRM2_HUMAN Metabotropic glutamate receptor 2 OS=Homo sapiens GN=GRM2 PE=1 SV=2 | 573 | WAVGPVtIACL | 0.103 | -0.209 | -0.898 | -0.335 |
| sp\|Q14416\|GRM2_HUMAN Metabotropic glutamate receptor 2 OS=Homo sapiens GN=GRM2 PE=1 SV=2 | 582 | CLGALAtLFVL | 0.487 | 0.213 | -0.098 | 0.201 |
| sp\|Q14416\|GRM2_HUMAN Metabotropic glutamate receptor 2 OS=Homo sapiens GN=GRM2 PE=1 SV=2 | 595 | FVRHNAtPVVK | 0.254 | -0.001 | -0.651 | -0.133 |
| sp\|Q14416\|GRM2_HUMAN Metabotropic glutamate receptor 2 OS=Homo sapiens GN=GRM2 PE=1 SV=2 | 601 | TPVVKAsGREL | 0.09 | -0.208 | -1.25 | -0.456 |
| sp\|Q14416\|GRM2_HUMAN Metabotropic glutamate receptor 2 OS=Homo sapiens GN=GRM2 PE=1 SV=2 | 620 | FLCYCMtFIFI | 0.694 | 0.412 | 0.43 | 0.512 |
| sp\|Q14416\|GRM2_HUMAN Metabotropic glutamate receptor 2 OS=Homo sapiens GN=GRM2 PE=1 SV=2 | 628 | IFIAKPsTAVC | 0.091 | -0.262 | -1.274 | -0.482 |
| sp\|Q14416\|GRM2_HUMAN Metabotropic glutamate receptor 2 OS=Homo sapiens GN=GRM2 PE=1 SV=2 | 629 | FIAKPStAVCT | 0.186 | 0.063 | -0.717 | -0.156 |
| sp\|Q14416\|GRM2_HUMAN Metabotropic glutamate receptor 2 OS=Homo sapiens GN=GRM2 PE=1 SV=2 | 633 | PSTAVCtLRRL | 0.496 | -0.081 | -0.462 | -0.016 |
| sp\|Q14416\|GRM2_HUMAN Metabotropic glutamate receptor 2 OS=Homo sapiens GN=GRM2 PE=1 SV=2 | 641 | RRLGLGtAFSV | 0.098 | 0.1 | -1.34 | -0.381 |
| sp\|Q14416\|GRM2_HUMAN Metabotropic glutamate receptor 2 OS=Homo sapiens GN=GRM2 PE=1 SV=2 | 644 | GLGTAFsVCYS | 0.261 | 0.038 | -0.795 | -0.165 |
| sp\|Q14416\|GRM2_HUMAN Metabotropic glutamate receptor 2 OS=Homo sapiens GN=GRM2 PE=1 SV=2 | 648 | AFSVCYsALLT | 0.145 | -0.02 | -0.882 | -0.252 |
| sp\|Q14416\|GRM2_HUMAN Metabotropic glutamate receptor 2 OS=Homo sapiens GN=GRM2 PE=1 SV=2 | 652 | CYSALLtKTNR | 0.201 | -0.081 | -0.894 | -0.258 |
| sp\|Q14416\|GRM2_HUMAN Metabotropic glutamate receptor 2 OS=Homo sapiens GN=GRM2 PE=1 SV=2 | 654 | SALLTKtNRIA | 0.138 | -0.183 | -1.062 | -0.369 |
| sp\|Q14416\|GRM2_HUMAN Metabotropic glutamate receptor 2 OS=Homo sapiens GN=GRM2 PE=1 SV=2 | 675 | QRPRFIsPASQ | 0.168 | 0.52 | -0.708 | -0.007 |

| | | | | | | |
|---|---|---|---|---|---|---|
| sp\|Q14416\|GRM2_HUMAN Metabotropic glutamate receptor 2 OS=Homo sapiens GN=GRM2 PE=1 SV=2 | 678 | RFISPAsQVAI | 0.294 | 0.024 | -0.567 | -0.083 |
| sp\|Q14416\|GRM2_HUMAN Metabotropic glutamate receptor 2 OS=Homo sapiens GN=GRM2 PE=1 SV=2 | 688 | ICLALIsGQLL | 0.251 | -0.059 | -0.67 | -0.159 |
| sp\|Q14416\|GRM2_HUMAN Metabotropic glutamate receptor 2 OS=Homo sapiens GN=GRM2 PE=1 SV=2 | 705 | VVEAPGtGKET | 0.059 | -0.389 | -1.227 | -0.519 |
| sp\|Q14416\|GRM2_HUMAN Metabotropic glutamate receptor 2 OS=Homo sapiens GN=GRM2 PE=1 SV=2 | 709 | PGTGKEtAPER | 0.503 | 0.439 | -0.1 | 0.281 |
| sp\|Q14416\|GRM2_HUMAN Metabotropic glutamate receptor 2 OS=Homo sapiens GN=GRM2 PE=1 SV=2 | 718 | ERREVVtLRCN | 0.639 | 0.511 | 0.072 | 0.407 |
| sp\|Q14416\|GRM2_HUMAN Metabotropic glutamate receptor 2 OS=Homo sapiens GN=GRM2 PE=1 SV=2 | 727 | CNHRDAsMLGS | 0.387 | 0.755 | -0.136 | 0.335 |
| sp\|Q14416\|GRM2_HUMAN Metabotropic glutamate receptor 2 OS=Homo sapiens GN=GRM2 PE=1 SV=2 | 731 | DASMLGsLAYN | 0.136 | -0.276 | -1.33 | -0.49 |
| sp\|Q14416\|GRM2_HUMAN Metabotropic glutamate receptor 2 OS=Homo sapiens GN=GRM2 PE=1 SV=2 | 743 | LLIALCtLYAF | 0.421 | 0.17 | -0.276 | 0.105 |
| sp\|Q14416\|GRM2_HUMAN Metabotropic glutamate receptor 2 OS=Homo sapiens GN=GRM2 PE=1 SV=2 | 749 | TLYAFKtRKCP | 0.162 | -0.088 | -0.854 | -0.26 |
| sp\|Q14416\|GRM2_HUMAN Metabotropic glutamate receptor 2 OS=Homo sapiens GN=GRM2 PE=1 SV=2 | 765 | AKFIGFtMYTT | 0.153 | 0.048 | -0.869 | -0.223 |
| sp\|Q14416\|GRM2_HUMAN Metabotropic glutamate receptor 2 OS=Homo sapiens GN=GRM2 PE=1 SV=2 | 768 | IGFTMYtTCII | 0.285 | -0.102 | -0.474 | -0.097 |
| sp\|Q14416\|GRM2_HUMAN Metabotropic glutamate receptor 2 OS=Homo sapiens GN=GRM2 PE=1 SV=2 | 769 | GFTMYTtCIIW | 0.336 | 0.103 | -0.327 | 0.037 |
| sp\|Q14416\|GRM2_HUMAN Metabotropic glutamate receptor 2 OS=Homo sapiens GN=GRM2 PE=1 SV=2 | 783 | LPIFYVtSSDY | 0.312 | -0.015 | -0.745 | -0.149 |
| sp\|Q14416\|GRM2_HUMAN Metabotropic glutamate receptor 2 OS=Homo sapiens GN=GRM2 PE=1 SV=2 | 784 | PIFYVTsSDYR | 0.311 | 0.088 | -0.494 | -0.032 |
| sp\|Q14416\|GRM2_HUMAN Metabotropic glutamate receptor 2 OS=Homo sapiens GN=GRM2 PE=1 SV=2 | 785 | IFYVTSsDYRV | 0.091 | -0.116 | -1.357 | -0.461 |
| sp\|Q14416\|GRM2_HUMAN Metabotropic glutamate receptor 2 OS=Homo sapiens GN=GRM2 PE=1 SV=2 | 791 | SDYRVQtTTMC | 0.257 | 0.522 | -0.686 | 0.031 |
| sp\|Q14416\|GRM2_HUMAN Metabotropic glutamate receptor 2 OS=Homo sapiens GN=GRM2 PE=1 SV=2 | 792 | DYRVQTtTMCV | 0.315 | 0.243 | -0.957 | -0.133 |
| sp\|Q14416\|GRM2_HUMAN Metabotropic glutamate receptor 2 OS=Homo sapiens GN=GRM2 PE=1 SV=2 | 793 | YRVQTTtMCVS | 0.125 | 0.243 | -0.871 | -0.168 |
| sp\|Q14416\|GRM2_HUMAN Metabotropic glutamate receptor 2 OS=Homo sapiens GN=GRM2 PE=1 SV=2 | 797 | TTTMCVsVSLS | 0.272 | -0.144 | -0.752 | -0.208 |
| sp\|Q14416\|GRM2_HUMAN Metabotropic glutamate receptor 2 OS=Homo sapiens GN=GRM2 PE=1 SV=2 | 799 | TMCVSVsLSGS | 0.339 | 0.212 | -0.323 | 0.076 |
| sp\|Q14416\|GRM2_HUMAN Metabotropic glutamate receptor 2 OS=Homo sapiens GN=GRM2 PE=1 SV=2 | 801 | CVSVSLsGSVV | 0.095 | 0.034 | -1.034 | -0.302 |
| sp\|Q14416\|GRM2_HUMAN Metabotropic glutamate receptor 2 OS=Homo sapiens GN=GRM2 PE=1 SV=2 | 803 | SVSLSGsVVLG | 0.149 | 0.053 | -0.957 | -0.252 |
| sp\|Q14416\|GRM2_HUMAN Metabotropic glutamate receptor 2 OS=Homo sapiens GN=GRM2 PE=1 SV=2 | 827 | PQKNVVsHRAP | 0.367 | -0.038 | -0.171 | 0.053 |
| sp\|Q14416\|GRM2_HUMAN Metabotropic glutamate receptor 2 OS=Homo sapiens GN=GRM2 PE=1 SV=2 | 832 | VSHRAPtSRFG | 0.389 | 0.547 | -0.214 | 0.241 |
| sp\|Q14416\|GRM2_HUMAN Metabotropic glutamate receptor 2 OS=Homo sapiens GN=GRM2 PE=1 SV=2 | 833 | SHRAPTsRFGS | 0.143 | -0.12 | -1.117 | -0.365 |
| sp\|Q14416\|GRM2_HUMAN Metabotropic glutamate receptor 2 OS=Homo sapiens GN=GRM2 PE=1 SV=2 | 837 | PTSRFGsAAAR | 0.593 | 0.775 | -0.008 | 0.453 |
| sp\|Q14416\|GRM2_HUMAN Metabotropic glutamate receptor 2 OS=Homo sapiens GN=GRM2 PE=1 SV=2 | 843 | SAAARAsSSLG | 0.17 | -0.072 | -1.013 | -0.305 |
| sp\|Q14416\|GRM2_HUMAN Metabotropic glutamate receptor 2 OS=Homo sapiens GN=GRM2 PE=1 SV=2 | 844 | AAARASsSLGQ | 0.35 | 0.6 | -0.24 | 0.237 |
| sp\|Q14416\|GRM2_HUMAN Metabotropic glutamate receptor 2 OS=Homo sapiens GN=GRM2 PE=1 SV=2 | 845 | AARASSsLGQG | 0.734 | 0.376 | 0.366 | 0.492 |
| sp\|Q14416\|GRM2_HUMAN Metabotropic glutamate receptor 2 OS=Homo sapiens GN=GRM2 PE=1 SV=2 | 850 | SSLGQGsGSQF | 0.029 | -0.426 | -1.798 | -0.732 |
| sp\|Q14416\|GRM2_HUMAN Metabotropic glutamate receptor 2 OS=Homo sapiens GN=GRM2 PE=1 SV=2 | 852 | LGQGSGsQFVP | 0.148 | 0.049 | -0.788 | -0.197 |
| sp\|Q14416\|GRM2_HUMAN Metabotropic glutamate receptor 2 OS=Homo sapiens GN=GRM2 PE=1 SV=2 | 857 | GSQFVPtVCNG | 0.251 | -0.074 | -1.028 | -0.284 |
| sp\|Q14416\|GRM2_HUMAN Metabotropic glutamate receptor 2 OS=Homo sapiens GN=GRM2 PE=1 SV=2 | 867 | GREVVDsTTSS | 0.134 | -0.009 | -1.317 | -0.397 |
| sp\|Q14416\|GRM2_HUMAN Metabotropic glutamate receptor 2 OS=Homo sapiens GN=GRM2 PE=1 SV=2 | 868 | REVVDStTSSL | 0.033 | -0.249 | -1.838 | -0.685 |
| sp\|Q14416\|GRM2_HUMAN Metabotropic glutamate receptor 2 OS=Homo sapiens GN=GRM2 PE=1 SV=2 | 869 | EVVDSTtSSL- | 0.13 | 0.123 | -0.879 | -0.209 |
| sp\|Q14416\|GRM2_HUMAN Metabotropic glutamate receptor 2 OS=Homo sapiens GN=GRM2 PE=1 SV=2 | 870 | VVDSTTsSL-- | 0.047 | -0.072 | -1.481 | -0.502 |
| sp\|Q14416\|GRM2_HUMAN Metabotropic glutamate receptor 2 OS=Homo sapiens GN=GRM2 PE=1 SV=2 | 871 | VDSTTSsL--- | 0.098 | -0.012 | -1.033 | -0.316 |
| sp\|Q14832\|GRM3_HUMAN Metabotropic glutamate receptor 3 OS=Homo sapiens GN=GRM3 PE=1 SV=2 | 322 | VAYGAItLELA | 0.296 | 0.024 | -0.337 | -0.006 |
| sp\|Q14832\|GRM3_HUMAN Metabotropic glutamate receptor 3 OS=Homo sapiens GN=GRM3 PE=1 SV=2 | 327 | ITLELAsQPVR | 0.483 | 0.381 | -0.416 | 0.149 |
| sp\|Q14832\|GRM3_HUMAN Metabotropic glutamate receptor 3 OS=Homo sapiens GN=GRM3 PE=1 SV=2 | 339 | FDRYFQsLNPY | 0.589 | 0.339 | 0.13 | 0.353 |
| sp\|Q14832\|GRM3_HUMAN Metabotropic glutamate receptor 3 OS=Homo sapiens GN=GRM3 PE=1 SV=2 | 362 | EQKFQCsLQNK | 0.378 | 0.104 | -0.454 | 0.009 |
| sp\|Q14832\|GRM3_HUMAN Metabotropic glutamate receptor 3 OS=Homo sapiens GN=GRM3 PE=1 SV=2 | 381 | KHLAIDsSNYE | 0.106 | -0.285 | -1.387 | -0.522 |
| sp\|Q14832\|GRM3_HUMAN Metabotropic glutamate receptor 3 OS=Homo sapiens GN=GRM3 PE=1 SV=2 | 382 | HLAIDSsNYEQ | 0.13 | -0.036 | -0.971 | -0.292 |
| sp\|Q14832\|GRM3_HUMAN Metabotropic glutamate receptor 3 OS=Homo sapiens GN=GRM3 PE=1 SV=2 | 388 | SNYEQEsKIMF | 0.022 | -0.37 | -1.873 | -0.74 |
| sp\|Q14832\|GRM3_HUMAN Metabotropic glutamate receptor 3 OS=Homo sapiens GN=GRM3 PE=1 SV=2 | 410 | LHKMQRtLCPN | 0.128 | -0.128 | -1.203 | -0.401 |
| sp\|Q14832\|GRM3_HUMAN Metabotropic glutamate receptor 3 OS=Homo sapiens GN=GRM3 PE=1 SV=2 | 415 | RTLCPNtTKLC | 0.223 | -0.157 | -1.103 | -0.346 |
| sp\|Q14832\|GRM3_HUMAN Metabotropic glutamate receptor 3 OS=Homo sapiens GN=GRM3 PE=1 SV=2 | 416 | TLCPNTtKLCD | 0.274 | 0.023 | -0.513 | -0.072 |
| sp\|Q14832\|GRM3_HUMAN Metabotropic glutamate receptor 3 OS=Homo sapiens GN=GRM3 PE=1 SV=2 | 441 | LLKINFtAPFN | 0.693 | 0.633 | 0.339 | 0.555 |
| sp\|Q14832\|GRM3_HUMAN Metabotropic glutamate receptor 3 OS=Homo sapiens GN=GRM3 PE=1 SV=2 | 452 | PNKDADsIVKF | 0.137 | -0.155 | -0.994 | -0.337 |

| | | | | | | |
|---|---|---|---|---|---|---|
| sp\|Q14832\|GRM3_HUMAN Metabotropic glutamate receptor 3 OS=Homo sapiens GN=GRM3 PE=1 SV=2 | 458 | SIVKFDtFGDG | 0.511 | 0.128 | -0.435 | 0.068 |
| sp\|Q14832\|GRM3_HUMAN Metabotropic glutamate receptor 3 OS=Homo sapiens GN=GRM3 PE=1 SV=2 | 479 | NVGGKYsYLKV | 0.041 | -0.135 | -1.545 | -0.546 |
| sp\|Q14832\|GRM3_HUMAN Metabotropic glutamate receptor 3 OS=Homo sapiens GN=GRM3 PE=1 SV=2 | 489 | VGHWAEtLSLD | 0.284 | -0.053 | -0.52 | -0.096 |
| sp\|Q14832\|GRM3_HUMAN Metabotropic glutamate receptor 3 OS=Homo sapiens GN=GRM3 PE=1 SV=2 | 491 | HWAETLsLDVN | 0.157 | 0.209 | -0.505 | -0.046 |
| sp\|Q14832\|GRM3_HUMAN Metabotropic glutamate receptor 3 OS=Homo sapiens GN=GRM3 PE=1 SV=2 | 496 | LSLDVNsIHWS | 0.183 | -0.087 | -0.919 | -0.274 |
| sp\|Q14832\|GRM3_HUMAN Metabotropic glutamate receptor 3 OS=Homo sapiens GN=GRM3 PE=1 SV=2 | 500 | VNSIHWsRNSV | 0.1 | -0.265 | -1.353 | -0.506 |
| sp\|Q14832\|GRM3_HUMAN Metabotropic glutamate receptor 3 OS=Homo sapiens GN=GRM3 PE=1 SV=2 | 503 | IHWSRNsVPTS | 0.662 | 0.669 | -0.044 | 0.429 |
| sp\|Q14832\|GRM3_HUMAN Metabotropic glutamate receptor 3 OS=Homo sapiens GN=GRM3 PE=1 SV=2 | 506 | SRNSVPtSQCS | 0.089 | -0.023 | -1.455 | -0.463 |
| sp\|Q14832\|GRM3_HUMAN Metabotropic glutamate receptor 3 OS=Homo sapiens GN=GRM3 PE=1 SV=2 | 507 | RNSVPTsQCSD | 0.042 | -0.395 | -1.557 | -0.637 |
| sp\|Q14832\|GRM3_HUMAN Metabotropic glutamate receptor 3 OS=Homo sapiens GN=GRM3 PE=1 SV=2 | 510 | VPTSQCsDPCA | 0.49 | 0.398 | -0.45 | 0.146 |
| sp\|Q14832\|GRM3_HUMAN Metabotropic glutamate receptor 3 OS=Homo sapiens GN=GRM3 PE=1 SV=2 | 545 | YLADEFtCMDC | 0.37 | 0.152 | -0.612 | -0.03 |
| sp\|Q14832\|GRM3_HUMAN Metabotropic glutamate receptor 3 OS=Homo sapiens GN=GRM3 PE=1 SV=2 | 551 | TCMDCGsGQWP | 0.13 | -0.11 | -1.103 | -0.361 |
| sp\|Q14832\|GRM3_HUMAN Metabotropic glutamate receptor 3 OS=Homo sapiens GN=GRM3 PE=1 SV=2 | 556 | GSGQWPtADLT | 0.248 | -0.046 | -0.699 | -0.166 |
| sp\|Q14832\|GRM3_HUMAN Metabotropic glutamate receptor 3 OS=Homo sapiens GN=GRM3 PE=1 SV=2 | 560 | WPTADLtGCYD | 0.072 | -0.378 | -1.288 | -0.531 |
| sp\|Q14832\|GRM3_HUMAN Metabotropic glutamate receptor 3 OS=Homo sapiens GN=GRM3 PE=1 SV=2 | 582 | WAIGPVtIACL | 0.109 | -0.182 | -0.882 | -0.318 |
| sp\|Q14832\|GRM3_HUMAN Metabotropic glutamate receptor 3 OS=Homo sapiens GN=GRM3 PE=1 SV=2 | 591 | CLGFMCtCMVV | 0.478 | 0.178 | -0.658 | -0.001 |
| sp\|Q14832\|GRM3_HUMAN Metabotropic glutamate receptor 3 OS=Homo sapiens GN=GRM3 PE=1 SV=2 | 596 | CTCMVVtVFIK | 0.543 | -0.077 | -0.387 | 0.026 |
| sp\|Q14832\|GRM3_HUMAN Metabotropic glutamate receptor 3 OS=Homo sapiens GN=GRM3 PE=1 SV=2 | 604 | FIKHNNtPLVK | 0.187 | -0.153 | -0.867 | -0.278 |
| sp\|Q14832\|GRM3_HUMAN Metabotropic glutamate receptor 3 OS=Homo sapiens GN=GRM3 PE=1 SV=2 | 610 | TPLVKAsGREL | 0.101 | -0.207 | -1.132 | -0.413 |
| sp\|Q14832\|GRM3_HUMAN Metabotropic glutamate receptor 3 OS=Homo sapiens GN=GRM3 PE=1 SV=2 | 625 | LFGVGLsYCMT | 0.017 | -0.441 | -1.801 | -0.742 |
| sp\|Q14832\|GRM3_HUMAN Metabotropic glutamate receptor 3 OS=Homo sapiens GN=GRM3 PE=1 SV=2 | 629 | GLSYCMtFFFI | 0.733 | 0.347 | 0.425 | 0.502 |
| sp\|Q14832\|GRM3_HUMAN Metabotropic glutamate receptor 3 OS=Homo sapiens GN=GRM3 PE=1 SV=2 | 637 | FFIAKPsPVIC | 0.063 | -0.425 | -1.552 | -0.638 |
| sp\|Q14832\|GRM3_HUMAN Metabotropic glutamate receptor 3 OS=Homo sapiens GN=GRM3 PE=1 SV=2 | 650 | RRLGLGsSFAI | 0.215 | 0.197 | -0.542 | -0.043 |
| sp\|Q14832\|GRM3_HUMAN Metabotropic glutamate receptor 3 OS=Homo sapiens GN=GRM3 PE=1 SV=2 | 651 | RLGLGSsFAIC | 0.111 | -0.119 | -1.238 | -0.415 |
| sp\|Q14832\|GRM3_HUMAN Metabotropic glutamate receptor 3 OS=Homo sapiens GN=GRM3 PE=1 SV=2 | 657 | SFAICYsALLT | 0.148 | 0.028 | -0.791 | -0.205 |
| sp\|Q14832\|GRM3_HUMAN Metabotropic glutamate receptor 3 OS=Homo sapiens GN=GRM3 PE=1 SV=2 | 661 | CYSALLtKTNC | 0.144 | -0.165 | -1.244 | -0.422 |
| sp\|Q14832\|GRM3_HUMAN Metabotropic glutamate receptor 3 OS=Homo sapiens GN=GRM3 PE=1 SV=2 | 663 | SALLTKtNCIA | 0.099 | -0.219 | -1.275 | -0.465 |
| sp\|Q14832\|GRM3_HUMAN Metabotropic glutamate receptor 3 OS=Homo sapiens GN=GRM3 PE=1 SV=2 | 684 | QRPKFIsPSSQ | 0.052 | -0.31 | -1.621 | -0.626 |
| sp\|Q14832\|GRM3_HUMAN Metabotropic glutamate receptor 3 OS=Homo sapiens GN=GRM3 PE=1 SV=2 | 686 | PKFISPsSQVF | 0.422 | 0.234 | -0.204 | 0.151 |
| sp\|Q14832\|GRM3_HUMAN Metabotropic glutamate receptor 3 OS=Homo sapiens GN=GRM3 PE=1 SV=2 | 687 | KFISPSsQVFI | 0.064 | -0.305 | -1.402 | -0.548 |
| sp\|Q14832\|GRM3_HUMAN Metabotropic glutamate receptor 3 OS=Homo sapiens GN=GRM3 PE=1 SV=2 | 704 | VQIVMVsVWLI | 0.309 | 0 | -0.429 | -0.04 |
| sp\|Q14832\|GRM3_HUMAN Metabotropic glutamate receptor 3 OS=Homo sapiens GN=GRM3 PE=1 SV=2 | 714 | ILEAPGtRRYT | 0.136 | -0.389 | -1.032 | -0.428 |
| sp\|Q14832\|GRM3_HUMAN Metabotropic glutamate receptor 3 OS=Homo sapiens GN=GRM3 PE=1 SV=2 | 718 | PGTRRYtLAEK | 0.868 | 1.105 | 1.267 | 1.08 |
| sp\|Q14832\|GRM3_HUMAN Metabotropic glutamate receptor 3 OS=Homo sapiens GN=GRM3 PE=1 SV=2 | 725 | LAEKREtVILK | 0.339 | 0.037 | -0.433 | -0.019 |
| sp\|Q14832\|GRM3_HUMAN Metabotropic glutamate receptor 3 OS=Homo sapiens GN=GRM3 PE=1 SV=2 | 735 | KCNVKDsSMLI | 0.11 | -0.163 | -1.46 | -0.504 |
| sp\|Q14832\|GRM3_HUMAN Metabotropic glutamate receptor 3 OS=Homo sapiens GN=GRM3 PE=1 SV=2 | 736 | CNVKDSsMLIS | 0.118 | -0.094 | -1.142 | -0.373 |
| sp\|Q14832\|GRM3_HUMAN Metabotropic glutamate receptor 3 OS=Homo sapiens GN=GRM3 PE=1 SV=2 | 740 | DSSMLIsLTYD | 0.396 | -0.162 | -0.67 | -0.145 |
| sp\|Q14832\|GRM3_HUMAN Metabotropic glutamate receptor 3 OS=Homo sapiens GN=GRM3 PE=1 SV=2 | 742 | SMLISLtYDVI | 0.174 | 0.275 | -0.528 | -0.026 |
| sp\|Q14832\|GRM3_HUMAN Metabotropic glutamate receptor 3 OS=Homo sapiens GN=GRM3 PE=1 SV=2 | 752 | ILVILCtVYAF | 0.288 | 0.112 | -0.583 | -0.061 |
| sp\|Q14832\|GRM3_HUMAN Metabotropic glutamate receptor 3 OS=Homo sapiens GN=GRM3 PE=1 SV=2 | 758 | TVYAFKtRKCP | 0.083 | -0.183 | -1.141 | -0.414 |
| sp\|Q14832\|GRM3_HUMAN Metabotropic glutamate receptor 3 OS=Homo sapiens GN=GRM3 PE=1 SV=2 | 774 | AKFIGFtMYTT | 0.153 | 0.048 | -0.869 | -0.223 |
| sp\|Q14832\|GRM3_HUMAN Metabotropic glutamate receptor 3 OS=Homo sapiens GN=GRM3 PE=1 SV=2 | 777 | IGFTMYtTCII | 0.285 | -0.102 | -0.474 | -0.097 |
| sp\|Q14832\|GRM3_HUMAN Metabotropic glutamate receptor 3 OS=Homo sapiens GN=GRM3 PE=1 SV=2 | 778 | GFTMYTtCIIW | 0.336 | 0.103 | -0.327 | 0.037 |
| sp\|Q14832\|GRM3_HUMAN Metabotropic glutamate receptor 3 OS=Homo sapiens GN=GRM3 PE=1 SV=2 | 792 | LPIFYVtSSDY | 0.312 | -0.015 | -0.745 | -0.149 |
| sp\|Q14832\|GRM3_HUMAN Metabotropic glutamate receptor 3 OS=Homo sapiens GN=GRM3 PE=1 SV=2 | 793 | PIFYVTsSDYR | 0.311 | 0.088 | -0.494 | -0.032 |
| sp\|Q14832\|GRM3_HUMAN Metabotropic glutamate receptor 3 OS=Homo sapiens GN=GRM3 PE=1 SV=2 | 794 | IFYVTSsDYRV | 0.091 | -0.116 | -1.357 | -0.461 |
| sp\|Q14832\|GRM3_HUMAN Metabotropic glutamate receptor 3 OS=Homo sapiens GN=GRM3 PE=1 SV=2 | 800 | SDYRVQtTTMC | 0.257 | 0.522 | -0.686 | 0.031 |
| sp\|Q14832\|GRM3_HUMAN Metabotropic glutamate receptor 3 OS=Homo sapiens GN=GRM3 PE=1 SV=2 | 801 | DYRVQTtTMCI | 0.404 | 0.301 | -0.511 | 0.065 |
| sp\|Q14832\|GRM3_HUMAN Metabotropic glutamate receptor 3 OS=Homo sapiens GN=GRM3 PE=1 SV=2 | 802 | YRVQTTtMCIS | 0.151 | 0.211 | -0.835 | -0.158 |

| | | | | | | |
|---|---|---|---|---|---|---|
| sp\|Q14832\|GRM3_HUMAN Metabotropic glutamate receptor 3 OS=Homo sapiens GN=GRM3 PE=1 SV=2 | 806 | TTTMCIsVSLS | 0.193 | -0.157 | -0.877 | -0.28 |
| sp\|Q14832\|GRM3_HUMAN Metabotropic glutamate receptor 3 OS=Homo sapiens GN=GRM3 PE=1 SV=2 | 808 | TMCISVsLSGF | 0.405 | 0.256 | -0.133 | 0.176 |
| sp\|Q14832\|GRM3_HUMAN Metabotropic glutamate receptor 3 OS=Homo sapiens GN=GRM3 PE=1 SV=2 | 810 | CISVSLsGFVV | 0.222 | 0.184 | -0.595 | -0.063 |
| sp\|Q14832\|GRM3_HUMAN Metabotropic glutamate receptor 3 OS=Homo sapiens GN=GRM3 PE=1 SV=2 | 836 | PQKNVVtHRLH | 0.291 | -0.149 | -0.526 | -0.128 |
| sp\|Q14832\|GRM3_HUMAN Metabotropic glutamate receptor 3 OS=Homo sapiens GN=GRM3 PE=1 SV=2 | 845 | LHLNRFsVSGT | 0.138 | -0.011 | -0.743 | -0.205 |
| sp\|Q14832\|GRM3_HUMAN Metabotropic glutamate receptor 3 OS=Homo sapiens GN=GRM3 PE=1 SV=2 | 847 | LNRFSVsGTGT | 0.58 | 0.214 | 0.194 | 0.329 |
| sp\|Q14832\|GRM3_HUMAN Metabotropic glutamate receptor 3 OS=Homo sapiens GN=GRM3 PE=1 SV=2 | 849 | RFSVSGtGTTY | 0.294 | 0.056 | -0.603 | -0.084 |
| sp\|Q14832\|GRM3_HUMAN Metabotropic glutamate receptor 3 OS=Homo sapiens GN=GRM3 PE=1 SV=2 | 851 | SVSGTGtTYSQ | 0.025 | -0.287 | -1.888 | -0.717 |
| sp\|Q14832\|GRM3_HUMAN Metabotropic glutamate receptor 3 OS=Homo sapiens GN=GRM3 PE=1 SV=2 | 852 | VSGTGTtYSQS | 0.033 | -0.413 | -1.662 | -0.681 |
| sp\|Q14832\|GRM3_HUMAN Metabotropic glutamate receptor 3 OS=Homo sapiens GN=GRM3 PE=1 SV=2 | 854 | GTGTTYsQSSA | 0.059 | -0.143 | -1.517 | -0.534 |
| sp\|Q14832\|GRM3_HUMAN Metabotropic glutamate receptor 3 OS=Homo sapiens GN=GRM3 PE=1 SV=2 | 856 | GTTYSQsSAST | 0.205 | 0.067 | -0.567 | -0.098 |
| sp\|Q14832\|GRM3_HUMAN Metabotropic glutamate receptor 3 OS=Homo sapiens GN=GRM3 PE=1 SV=2 | 857 | TTYSQSsASTY | 0.05 | -0.252 | -1.734 | -0.645 |
| sp\|Q14832\|GRM3_HUMAN Metabotropic glutamate receptor 3 OS=Homo sapiens GN=GRM3 PE=1 SV=2 | 859 | YSQSSAsTYVP | 0.241 | 0.117 | -0.591 | -0.078 |
| sp\|Q14832\|GRM3_HUMAN Metabotropic glutamate receptor 3 OS=Homo sapiens GN=GRM3 PE=1 SV=2 | 860 | SQSSAStYVPT | 0.039 | -0.357 | -1.505 | -0.608 |
| sp\|Q14832\|GRM3_HUMAN Metabotropic glutamate receptor 3 OS=Homo sapiens GN=GRM3 PE=1 SV=2 | 864 | ASTYVPtVCNG | 0.159 | -0.194 | -1.077 | -0.371 |
| sp\|Q14832\|GRM3_HUMAN Metabotropic glutamate receptor 3 OS=Homo sapiens GN=GRM3 PE=1 SV=2 | 874 | GREVLDsTTSS | 0.09 | -0.051 | -1.484 | -0.482 |
| sp\|Q14832\|GRM3_HUMAN Metabotropic glutamate receptor 3 OS=Homo sapiens GN=GRM3 PE=1 SV=2 | 875 | REVLDStTSSL | 0.045 | -0.222 | -1.742 | -0.64 |
| sp\|Q14832\|GRM3_HUMAN Metabotropic glutamate receptor 3 OS=Homo sapiens GN=GRM3 PE=1 SV=2 | 876 | EVLDSTtSSL- | 0.149 | 0.123 | -0.761 | -0.163 |
| sp\|Q14832\|GRM3_HUMAN Metabotropic glutamate receptor 3 OS=Homo sapiens GN=GRM3 PE=1 SV=2 | 877 | VLDSTTsSL-- | 0.087 | 0.023 | -1.193 | -0.361 |
| sp\|Q14832\|GRM3_HUMAN Metabotropic glutamate receptor 3 OS=Homo sapiens GN=GRM3 PE=1 SV=2 | 878 | LDSTTSsL--- | 0.095 | 0.017 | -0.957 | -0.282 |
| sp\|Q14833\|GRM4_HUMAN Metabotropic glutamate receptor 4 OS=Homo sapiens GN=GRM4 PE=2 SV=1 | 316 | GSDSWGsKIAP | 0.108 | -0.189 | -1.014 | -0.365 |
| sp\|Q14833\|GRM4_HUMAN Metabotropic glutamate receptor 4 OS=Homo sapiens GN=GRM4 PE=2 SV=1 | 333 | VAEGAVtILPK | 0.162 | -0.236 | -0.843 | -0.306 |
| sp\|Q14833\|GRM4_HUMAN Metabotropic glutamate receptor 4 OS=Homo sapiens GN=GRM4 PE=2 SV=1 | 340 | ILPKRMsVRGF | 0.655 | 0.235 | 0.196 | 0.362 |
| sp\|Q14833\|GRM4_HUMAN Metabotropic glutamate receptor 4 OS=Homo sapiens GN=GRM4 PE=2 SV=1 | 349 | GFDRYFsSRTL | 0.71 | 0.855 | 0.486 | 0.684 |
| sp\|Q14833\|GRM4_HUMAN Metabotropic glutamate receptor 4 OS=Homo sapiens GN=GRM4 PE=2 SV=1 | 350 | FDRYFSsRTLD | 0.272 | 0.088 | -0.705 | -0.115 |
| sp\|Q14833\|GRM4_HUMAN Metabotropic glutamate receptor 4 OS=Homo sapiens GN=GRM4 PE=2 SV=1 | 352 | RYFSSRtLDNN | 0.63 | 0.416 | -0.096 | 0.317 |
| sp\|Q14833\|GRM4_HUMAN Metabotropic glutamate receptor 4 OS=Homo sapiens GN=GRM4 PE=2 SV=1 | 375 | NFHCKLsRHAL | 0.128 | -0.104 | -1.031 | -0.336 |
| sp\|Q14833\|GRM4_HUMAN Metabotropic glutamate receptor 4 OS=Homo sapiens GN=GRM4 PE=2 SV=1 | 383 | HALKKGsHVKK | 0.123 | -0.17 | -1.048 | -0.365 |
| sp\|Q14833\|GRM4_HUMAN Metabotropic glutamate receptor 4 OS=Homo sapiens GN=GRM4 PE=2 SV=1 | 389 | SHVKKCtNRER | 0.201 | -0.161 | -1.432 | -0.464 |
| sp\|Q14833\|GRM4_HUMAN Metabotropic glutamate receptor 4 OS=Homo sapiens GN=GRM4 PE=2 SV=1 | 398 | ERIGQDsAYEQ | 0.105 | 0.117 | -1.28 | -0.353 |
| sp\|Q14833\|GRM4_HUMAN Metabotropic glutamate receptor 4 OS=Homo sapiens GN=GRM4 PE=2 SV=1 | 444 | MDPVDGtQLLK | 0.035 | -0.372 | -2.007 | -0.781 |
| sp\|Q14833\|GRM4_HUMAN Metabotropic glutamate receptor 4 OS=Homo sapiens GN=GRM4 PE=2 SV=1 | 456 | IRNVNFsGIAG | 0.219 | 0.204 | -0.349 | 0.025 |
| sp\|Q14833\|GRM4_HUMAN Metabotropic glutamate receptor 4 OS=Homo sapiens GN=GRM4 PE=2 SV=1 | 464 | IAGNPVtFNEN | 0.339 | -0.049 | -0.248 | 0.014 |
| sp\|Q14833\|GRM4_HUMAN Metabotropic glutamate receptor 4 OS=Homo sapiens GN=GRM4 PE=2 SV=1 | 486 | YQLRNDsAEYK | 0.496 | 0.693 | 0.028 | 0.406 |
| sp\|Q14833\|GRM4_HUMAN Metabotropic glutamate receptor 4 OS=Homo sapiens GN=GRM4 PE=2 SV=1 | 494 | EYKVIGsWTDH | 0.145 | -0.064 | -0.942 | -0.287 |
| sp\|Q14833\|GRM4_HUMAN Metabotropic glutamate receptor 4 OS=Homo sapiens GN=GRM4 PE=2 SV=1 | 496 | KVIGSWtDHLH | 0.257 | 0.091 | -0.798 | -0.15 |
| sp\|Q14833\|GRM4_HUMAN Metabotropic glutamate receptor 4 OS=Homo sapiens GN=GRM4 PE=2 SV=1 | 511 | RMHWPGsGQQL | 0.086 | -0.143 | -1.015 | -0.357 |
| sp\|Q14833\|GRM4_HUMAN Metabotropic glutamate receptor 4 OS=Homo sapiens GN=GRM4 PE=2 SV=1 | 518 | GQQLPRsICSL | 0.049 | -0.201 | -1.375 | -0.509 |
| sp\|Q14833\|GRM4_HUMAN Metabotropic glutamate receptor 4 OS=Homo sapiens GN=GRM4 PE=2 SV=1 | 521 | LPRSICsLPCQ | 0.81 | 0.721 | 0.502 | 0.678 |
| sp\|Q14833\|GRM4_HUMAN Metabotropic glutamate receptor 4 OS=Homo sapiens GN=GRM4 PE=2 SV=1 | 532 | PGERKKtVKGM | 0.648 | 0.78 | 0.723 | 0.717 |
| sp\|Q14833\|GRM4_HUMAN Metabotropic glutamate receptor 4 OS=Homo sapiens GN=GRM4 PE=2 SV=1 | 546 | WHCEPCtGYQY | 0.143 | -0.194 | -1.298 | -0.45 |
| sp\|Q14833\|GRM4_HUMAN Metabotropic glutamate receptor 4 OS=Homo sapiens GN=GRM4 PE=2 SV=1 | 556 | YQVDRYtCKTC | 0.194 | 0.15 | -0.675 | -0.11 |
| sp\|Q14833\|GRM4_HUMAN Metabotropic glutamate receptor 4 OS=Homo sapiens GN=GRM4 PE=2 SV=1 | 559 | DRYTCKtCPYD | 0.844 | 0.813 | 0.527 | 0.728 |
| sp\|Q14833\|GRM4_HUMAN Metabotropic glutamate receptor 4 OS=Homo sapiens GN=GRM4 PE=2 SV=1 | 567 | PYDMRPtENRT | 0.428 | 0.03 | -0.428 | 0.01 |
| sp\|Q14833\|GRM4_HUMAN Metabotropic glutamate receptor 4 OS=Homo sapiens GN=GRM4 PE=2 SV=1 | 571 | RPTENRtGCRP | 0.05 | -0.358 | -1.708 | -0.672 |
| sp\|Q14833\|GRM4_HUMAN Metabotropic glutamate receptor 4 OS=Homo sapiens GN=GRM4 PE=2 SV=1 | 585 | IKLEWGsPWAV | 0.21 | -0.194 | -0.957 | -0.314 |
| sp\|Q14833\|GRM4_HUMAN Metabotropic glutamate receptor 4 OS=Homo sapiens GN=GRM4 PE=2 SV=1 | 602 | VVGIAAtLFVV | 0.359 | 0.174 | -0.517 | 0.005 |
| sp\|Q14833\|GRM4_HUMAN Metabotropic glutamate receptor 4 OS=Homo sapiens GN=GRM4 PE=2 SV=1 | 608 | TLFVVItFVRY | 0.367 | 0.05 | -0.68 | -0.088 |
| sp\|Q14833\|GRM4_HUMAN Metabotropic glutamate receptor 4 OS=Homo sapiens GN=GRM4 PE=2 SV=1 | 615 | FVRYNDtPIVK | 0.184 | -0.062 | -0.695 | -0.191 |

| | | | | | | |
|---|---|---|---|---|---|---|
| sp\|Q14833\|GRM4_HUMAN Metabotropic glutamate receptor 4 OS=Homo sapiens GN=GRM4 PE=2 SV=1 | 621 | TPIVKAsGREL | 0.093 | -0.181 | -1.234 | -0.441 |
| sp\|Q14833\|GRM4_HUMAN Metabotropic glutamate receptor 4 OS=Homo sapiens GN=GRM4 PE=2 SV=1 | 626 | ASGRELsYVLL | 0.245 | 0.643 | -0.466 | 0.141 |
| sp\|Q14833\|GRM4_HUMAN Metabotropic glutamate receptor 4 OS=Homo sapiens GN=GRM4 PE=2 SV=1 | 639 | IFLCYAtTFLM | 0.443 | 0.024 | -0.269 | 0.066 |
| sp\|Q14833\|GRM4_HUMAN Metabotropic glutamate receptor 4 OS=Homo sapiens GN=GRM4 PE=2 SV=1 | 640 | FLCYATtFLMI | 0.616 | 0.309 | 0.119 | 0.348 |
| sp\|Q14833\|GRM4_HUMAN Metabotropic glutamate receptor 4 OS=Homo sapiens GN=GRM4 PE=2 SV=1 | 651 | AEPDLGtCSLR | 0.05 | -0.24 | -1.884 | -0.691 |
| sp\|Q14833\|GRM4_HUMAN Metabotropic glutamate receptor 4 OS=Homo sapiens GN=GRM4 PE=2 SV=1 | 653 | PDLGTCsLRRI | 0.342 | -0.041 | -0.478 | -0.059 |
| sp\|Q14833\|GRM4_HUMAN Metabotropic glutamate receptor 4 OS=Homo sapiens GN=GRM4 PE=2 SV=1 | 664 | FLGLGMsISYA | 0.121 | 0.019 | -0.955 | -0.272 |
| sp\|Q14833\|GRM4_HUMAN Metabotropic glutamate receptor 4 OS=Homo sapiens GN=GRM4 PE=2 SV=1 | 666 | GLGMSIsYAAL | 0.468 | 0.265 | -0.024 | 0.236 |
| sp\|Q14833\|GRM4_HUMAN Metabotropic glutamate receptor 4 OS=Homo sapiens GN=GRM4 PE=2 SV=1 | 672 | SYAALLtKTNR | 0.121 | -0.148 | -1.199 | -0.409 |
| sp\|Q14833\|GRM4_HUMAN Metabotropic glutamate receptor 4 OS=Homo sapiens GN=GRM4 PE=2 SV=1 | 674 | AALLTKtNRIY | 0.246 | -0.098 | -0.896 | -0.249 |
| sp\|Q14833\|GRM4_HUMAN Metabotropic glutamate receptor 4 OS=Homo sapiens GN=GRM4 PE=2 SV=1 | 687 | FEQGKRsVSAP | 0.065 | -0.038 | -1.303 | -0.425 |
| sp\|Q14833\|GRM4_HUMAN Metabotropic glutamate receptor 4 OS=Homo sapiens GN=GRM4 PE=2 SV=1 | 689 | QGKRSVsAPRF | 0.957 | 1.515 | 1.655 | 1.376 |
| sp\|Q14833\|GRM4_HUMAN Metabotropic glutamate receptor 4 OS=Homo sapiens GN=GRM4 PE=2 SV=1 | 695 | SAPRFIsPASQ | 0.089 | 0.205 | -1.228 | -0.311 |
| sp\|Q14833\|GRM4_HUMAN Metabotropic glutamate receptor 4 OS=Homo sapiens GN=GRM4 PE=2 SV=1 | 698 | RFISPAsQLAI | 0.245 | -0.005 | -0.695 | -0.152 |
| sp\|Q14833\|GRM4_HUMAN Metabotropic glutamate receptor 4 OS=Homo sapiens GN=GRM4 PE=2 SV=1 | 703 | ASQLAItFSLI | 0.394 | 0.081 | -0.392 | 0.028 |
| sp\|Q14833\|GRM4_HUMAN Metabotropic glutamate receptor 4 OS=Homo sapiens GN=GRM4 PE=2 SV=1 | 705 | QLAITFsLISL | 0.19 | 0.221 | -0.207 | 0.068 |
| sp\|Q14833\|GRM4_HUMAN Metabotropic glutamate receptor 4 OS=Homo sapiens GN=GRM4 PE=2 SV=1 | 708 | ITFSLIsLQLL | 0.379 | 0.014 | -0.428 | -0.012 |
| sp\|Q14833\|GRM4_HUMAN Metabotropic glutamate receptor 4 OS=Homo sapiens GN=GRM4 PE=2 SV=1 | 723 | WFVVDPsHSVV | 0.088 | -0.198 | -1.31 | -0.473 |
| sp\|Q14833\|GRM4_HUMAN Metabotropic glutamate receptor 4 OS=Homo sapiens GN=GRM4 PE=2 SV=1 | 725 | VVDPSHsVVDF | 0.328 | 0.25 | -0.266 | 0.104 |
| sp\|Q14833\|GRM4_HUMAN Metabotropic glutamate receptor 4 OS=Homo sapiens GN=GRM4 PE=2 SV=1 | 734 | DFQDQRtLDPR | 0.355 | 0.106 | -0.596 | -0.045 |
| sp\|Q14833\|GRM4_HUMAN Metabotropic glutamate receptor 4 OS=Homo sapiens GN=GRM4 PE=2 SV=1 | 749 | VLKCDIsDLSL | 0.254 | 0.014 | -0.775 | -0.169 |
| sp\|Q14833\|GRM4_HUMAN Metabotropic glutamate receptor 4 OS=Homo sapiens GN=GRM4 PE=2 SV=1 | 752 | CDISDLsLICL | 0.11 | -0.028 | -0.774 | -0.231 |
| sp\|Q14833\|GRM4_HUMAN Metabotropic glutamate receptor 4 OS=Homo sapiens GN=GRM4 PE=2 SV=1 | 760 | ICLLGYsMLLM | 0.148 | 0.094 | -0.877 | -0.212 |
| sp\|Q14833\|GRM4_HUMAN Metabotropic glutamate receptor 4 OS=Homo sapiens GN=GRM4 PE=2 SV=1 | 766 | SMLLMVtCTVY | 0.295 | 0.088 | -0.871 | -0.163 |
| sp\|Q14833\|GRM4_HUMAN Metabotropic glutamate receptor 4 OS=Homo sapiens GN=GRM4 PE=2 SV=1 | 768 | LLMVTCtVYAI | 0.31 | 0.27 | -0.275 | 0.102 |
| sp\|Q14833\|GRM4_HUMAN Metabotropic glutamate receptor 4 OS=Homo sapiens GN=GRM4 PE=2 SV=1 | 774 | TVYAIKtRGVP | 0.134 | -0.108 | -0.695 | -0.223 |
| sp\|Q14833\|GRM4_HUMAN Metabotropic glutamate receptor 4 OS=Homo sapiens GN=GRM4 PE=2 SV=1 | 780 | TRGVPEtFNEA | 0.162 | 0.075 | -0.701 | -0.155 |
| sp\|Q14833\|GRM4_HUMAN Metabotropic glutamate receptor 4 OS=Homo sapiens GN=GRM4 PE=2 SV=1 | 790 | AKPIGFtMYTT | 0.108 | -0.03 | -1.071 | -0.331 |
| sp\|Q14833\|GRM4_HUMAN Metabotropic glutamate receptor 4 OS=Homo sapiens GN=GRM4 PE=2 SV=1 | 793 | IGFTMYtTCIV | 0.209 | -0.159 | -0.92 | -0.29 |
| sp\|Q14833\|GRM4_HUMAN Metabotropic glutamate receptor 4 OS=Homo sapiens GN=GRM4 PE=2 SV=1 | 794 | GFTMYTtCIVW | 0.293 | 0.135 | -0.363 | 0.022 |
| sp\|Q14833\|GRM4_HUMAN Metabotropic glutamate receptor 4 OS=Homo sapiens GN=GRM4 PE=2 SV=1 | 808 | IPIFFGtSQSA | 0.081 | -0.233 | -1.375 | -0.509 |
| sp\|Q14833\|GRM4_HUMAN Metabotropic glutamate receptor 4 OS=Homo sapiens GN=GRM4 PE=2 SV=1 | 809 | PIFFGTsQSAD | 0.19 | 0.022 | -0.805 | -0.198 |
| sp\|Q14833\|GRM4_HUMAN Metabotropic glutamate receptor 4 OS=Homo sapiens GN=GRM4 PE=2 SV=1 | 811 | FFGTSQsADKL | 0.383 | 0.357 | -0.183 | 0.186 |
| sp\|Q14833\|GRM4_HUMAN Metabotropic glutamate receptor 4 OS=Homo sapiens GN=GRM4 PE=2 SV=1 | 819 | DKLYIQtTTLT | 0.369 | 0.012 | -0.32 | 0.02 |
| sp\|Q14833\|GRM4_HUMAN Metabotropic glutamate receptor 4 OS=Homo sapiens GN=GRM4 PE=2 SV=1 | 820 | KLYIQTtTLTV | 0.103 | -0.049 | -1.335 | -0.427 |
| sp\|Q14833\|GRM4_HUMAN Metabotropic glutamate receptor 4 OS=Homo sapiens GN=GRM4 PE=2 SV=1 | 821 | LYIQTTtLTVS | 0.332 | 0.15 | -0.323 | 0.053 |
| sp\|Q14833\|GRM4_HUMAN Metabotropic glutamate receptor 4 OS=Homo sapiens GN=GRM4 PE=2 SV=1 | 823 | IQTTTLtVSVS | 0.055 | -0.135 | -1.175 | -0.418 |
| sp\|Q14833\|GRM4_HUMAN Metabotropic glutamate receptor 4 OS=Homo sapiens GN=GRM4 PE=2 SV=1 | 825 | TTTLTVsVSLS | 0.126 | -0.101 | -1.078 | -0.351 |
| sp\|Q14833\|GRM4_HUMAN Metabotropic glutamate receptor 4 OS=Homo sapiens GN=GRM4 PE=2 SV=1 | 827 | TLTVSVsLSAS | 0.587 | 0.322 | 0.246 | 0.385 |
| sp\|Q14833\|GRM4_HUMAN Metabotropic glutamate receptor 4 OS=Homo sapiens GN=GRM4 PE=2 SV=1 | 829 | TVSVSLsASVS | 0.069 | 0.03 | -1.221 | -0.374 |
| sp\|Q14833\|GRM4_HUMAN Metabotropic glutamate receptor 4 OS=Homo sapiens GN=GRM4 PE=2 SV=1 | 831 | SVSLSAsVSLG | 0.207 | 0.107 | -0.787 | -0.158 |
| sp\|Q14833\|GRM4_HUMAN Metabotropic glutamate receptor 4 OS=Homo sapiens GN=GRM4 PE=2 SV=1 | 833 | SLSASVsLGML | 0.735 | 0.298 | 0.335 | 0.456 |
| sp\|Q14833\|GRM4_HUMAN Metabotropic glutamate receptor 4 OS=Homo sapiens GN=GRM4 PE=2 SV=1 | 859 | VPKRKRsLKAV | 0.653 | 0.884 | 0.313 | 0.617 |
| sp\|Q14833\|GRM4_HUMAN Metabotropic glutamate receptor 4 OS=Homo sapiens GN=GRM4 PE=2 SV=1 | 865 | SLKAVVtAATM | 0.368 | -0.048 | -0.64 | -0.107 |
| sp\|Q14833\|GRM4_HUMAN Metabotropic glutamate receptor 4 OS=Homo sapiens GN=GRM4 PE=2 SV=1 | 868 | AVVTAAtMSNK | 0.18 | 0.052 | -1.176 | -0.315 |
| sp\|Q14833\|GRM4_HUMAN Metabotropic glutamate receptor 4 OS=Homo sapiens GN=GRM4 PE=2 SV=1 | 870 | VTAATMsNKFT | 0.091 | -0.155 | -1.129 | -0.398 |
| sp\|Q14833\|GRM4_HUMAN Metabotropic glutamate receptor 4 OS=Homo sapiens GN=GRM4 PE=2 SV=1 | 874 | TMSNKFtQKGN | 0.059 | -0.113 | -1.216 | -0.423 |
| sp\|Q14833\|GRM4_HUMAN Metabotropic glutamate receptor 4 OS=Homo sapiens GN=GRM4 PE=2 SV=1 | 887 | PNGEAKsELCE | 0.152 | 0.051 | -0.781 | -0.193 |
| sp\|Q14833\|GRM4_HUMAN Metabotropic glutamate receptor 4 OS=Homo sapiens GN=GRM4 PE=2 SV=1 | 900 | EAPALAtKQTY | 0.14 | -0.187 | -1.111 | -0.386 |

| | | | | | | |
|---|---|---|---|---|---|---|
| sp\|Q14833\|GRM4_HUMAN Metabotropic glutamate receptor 4 OS=Homo sapiens GN=GRM4 PE=2 SV=1 | 903 | ALATKQtYVTY | 0.184 | -0.076 | -0.874 | -0.255 |
| sp\|Q14833\|GRM4_HUMAN Metabotropic glutamate receptor 4 OS=Homo sapiens GN=GRM4 PE=2 SV=1 | 906 | TKQTYVtYTNH | 0.371 | -0.072 | -0.88 | -0.194 |
| sp\|Q14833\|GRM4_HUMAN Metabotropic glutamate receptor 4 OS=Homo sapiens GN=GRM4 PE=2 SV=1 | 908 | QTYVTYtNHAI | 0.154 | 0.109 | -0.677 | -0.138 |
| sp\|P41594\|GRM5_HUMAN Metabotropic glutamate receptor 5 OS=Homo sapiens GN=GRM5 PE=1 SV=2 | 326 | EAVGGItIKLQ | 0.046 | -0.276 | -1.332 | -0.521 |
| sp\|P41594\|GRM5_HUMAN Metabotropic glutamate receptor 5 OS=Homo sapiens GN=GRM5 PE=1 SV=2 | 331 | ITIKLQsPDVK | 0.18 | -0.287 | -0.987 | -0.365 |
| sp\|P41594\|GRM5_HUMAN Metabotropic glutamate receptor 5 OS=Homo sapiens GN=GRM5 PE=1 SV=2 | 348 | LKLRPEtNHRN | 0.644 | 0.704 | 0.039 | 0.462 |
| sp\|P41594\|GRM5_HUMAN Metabotropic glutamate receptor 5 OS=Homo sapiens GN=GRM5 PE=1 SV=2 | 375 | GFPQENsKYNK | 0.304 | -0.017 | -0.779 | -0.164 |
| sp\|P41594\|GRM5_HUMAN Metabotropic glutamate receptor 5 OS=Homo sapiens GN=GRM5 PE=1 SV=2 | 380 | NSKYNKtCNSS | 0.287 | 0.076 | -0.224 | 0.046 |
| sp\|P41594\|GRM5_HUMAN Metabotropic glutamate receptor 5 OS=Homo sapiens GN=GRM5 PE=1 SV=2 | 383 | YNKTCNsSLTL | 0.231 | -0.073 | -0.769 | -0.204 |
| sp\|P41594\|GRM5_HUMAN Metabotropic glutamate receptor 5 OS=Homo sapiens GN=GRM5 PE=1 SV=2 | 384 | NKTCNSsLTLK | 0.551 | 0.03 | -0.126 | 0.152 |
| sp\|P41594\|GRM5_HUMAN Metabotropic glutamate receptor 5 OS=Homo sapiens GN=GRM5 PE=1 SV=2 | 386 | TCNSSLtLKTH | 0.226 | 0.011 | -0.774 | -0.179 |
| sp\|P41594\|GRM5_HUMAN Metabotropic glutamate receptor 5 OS=Homo sapiens GN=GRM5 PE=1 SV=2 | 389 | SSLTLKtHHVQ | 0.157 | -0.155 | -1.088 | -0.362 |
| sp\|P41594\|GRM5_HUMAN Metabotropic glutamate receptor 5 OS=Homo sapiens GN=GRM5 PE=1 SV=2 | 395 | THHVQDsKMGF | 0.047 | -0.316 | -1.895 | -0.721 |
| sp\|P41594\|GRM5_HUMAN Metabotropic glutamate receptor 5 OS=Homo sapiens GN=GRM5 PE=1 SV=2 | 406 | VINAIYsMAYG | 0.15 | 0.054 | -0.842 | -0.213 |
| sp\|P41594\|GRM5_HUMAN Metabotropic glutamate receptor 5 OS=Homo sapiens GN=GRM5 PE=1 SV=2 | 417 | LHNMQMsLCPG | 0.175 | -0.045 | -1.013 | -0.294 |
| sp\|P41594\|GRM5_HUMAN Metabotropic glutamate receptor 5 OS=Homo sapiens GN=GRM5 PE=1 SV=2 | 440 | GRKLLEsLMKT | 0.323 | 0.195 | -0.632 | -0.038 |
| sp\|P41594\|GRM5_HUMAN Metabotropic glutamate receptor 5 OS=Homo sapiens GN=GRM5 PE=1 SV=2 | 444 | LESLMKtNFTG | 0.214 | -0.146 | -0.807 | -0.246 |
| sp\|P41594\|GRM5_HUMAN Metabotropic glutamate receptor 5 OS=Homo sapiens GN=GRM5 PE=1 SV=2 | 447 | LMKTNFtGVSG | 0.109 | 0.021 | -0.799 | -0.223 |
| sp\|P41594\|GRM5_HUMAN Metabotropic glutamate receptor 5 OS=Homo sapiens GN=GRM5 PE=1 SV=2 | 450 | TNFTGVsGDTI | 0.192 | -0.199 | -0.709 | -0.239 |
| sp\|P41594\|GRM5_HUMAN Metabotropic glutamate receptor 5 OS=Homo sapiens GN=GRM5 PE=1 SV=2 | 453 | TGVSGDtILFD | 0.041 | -0.423 | -1.694 | -0.692 |
| sp\|P41594\|GRM5_HUMAN Metabotropic glutamate receptor 5 OS=Homo sapiens GN=GRM5 PE=1 SV=2 | 462 | FDENGDsPGRY | 0.06 | -0.503 | -1.572 | -0.672 |
| sp\|P41594\|GRM5_HUMAN Metabotropic glutamate receptor 5 OS=Homo sapiens GN=GRM5 PE=1 SV=2 | 486 | DYINVGsWDNG | 0.231 | 0.098 | -0.95 | -0.207 |
| sp\|P41594\|GRM5_HUMAN Metabotropic glutamate receptor 5 OS=Homo sapiens GN=GRM5 PE=1 SV=2 | 501 | DDDEVWsKKSN | 0.06 | -0.3 | -1.601 | -0.614 |
| sp\|P41594\|GRM5_HUMAN Metabotropic glutamate receptor 5 OS=Homo sapiens GN=GRM5 PE=1 SV=2 | 504 | EVWSKKsNIIR | 0.092 | -0.019 | -0.916 | -0.281 |
| sp\|P41594\|GRM5_HUMAN Metabotropic glutamate receptor 5 OS=Homo sapiens GN=GRM5 PE=1 SV=2 | 509 | KSNIIRsVCSE | 0.038 | -0.317 | -1.694 | -0.658 |
| sp\|P41594\|GRM5_HUMAN Metabotropic glutamate receptor 5 OS=Homo sapiens GN=GRM5 PE=1 SV=2 | 512 | IIRSVCsEPCE | 0.88 | 0.826 | 0.474 | 0.727 |
| sp\|P41594\|GRM5_HUMAN Metabotropic glutamate receptor 5 OS=Homo sapiens GN=GRM5 PE=1 SV=2 | 529 | IRKGEVsCCWT | 0.333 | 0.135 | -0.436 | 0.011 |
| sp\|P41594\|GRM5_HUMAN Metabotropic glutamate receptor 5 OS=Homo sapiens GN=GRM5 PE=1 SV=2 | 533 | EVSCCWtCTPC | 0.203 | -0.16 | -1.101 | -0.353 |
| sp\|P41594\|GRM5_HUMAN Metabotropic glutamate receptor 5 OS=Homo sapiens GN=GRM5 PE=1 SV=2 | 535 | SCCWTCtPCKE | 0.067 | -0.469 | -1.854 | -0.752 |
| sp\|P41594\|GRM5_HUMAN Metabotropic glutamate receptor 5 OS=Homo sapiens GN=GRM5 PE=1 SV=2 | 548 | YVFDEYtCKAC | 0.148 | 0.094 | -0.909 | -0.222 |
| sp\|P41594\|GRM5_HUMAN Metabotropic glutamate receptor 5 OS=Homo sapiens GN=GRM5 PE=1 SV=2 | 556 | KACQLGsWPTD | 0.409 | 0.349 | -0.365 | 0.131 |
| sp\|P41594\|GRM5_HUMAN Metabotropic glutamate receptor 5 OS=Homo sapiens GN=GRM5 PE=1 SV=2 | 559 | QLGSWPtDDLT | 0.217 | -0.016 | -0.714 | -0.171 |
| sp\|P41594\|GRM5_HUMAN Metabotropic glutamate receptor 5 OS=Homo sapiens GN=GRM5 PE=1 SV=2 | 563 | WPTDDLtGCDL | 0.097 | -0.223 | -1.117 | -0.414 |
| sp\|P41594\|GRM5_HUMAN Metabotropic glutamate receptor 5 OS=Homo sapiens GN=GRM5 PE=1 SV=2 | 594 | CLGLLAtLFVT | 0.4 | 0.152 | -0.298 | 0.085 |
| sp\|P41594\|GRM5_HUMAN Metabotropic glutamate receptor 5 OS=Homo sapiens GN=GRM5 PE=1 SV=2 | 598 | LATLFVtVVFI | 0.225 | -0.097 | -0.733 | -0.202 |
| sp\|P41594\|GRM5_HUMAN Metabotropic glutamate receptor 5 OS=Homo sapiens GN=GRM5 PE=1 SV=2 | 607 | FIIYRDtPVVK | 0.186 | -0.058 | -0.932 | -0.268 |
| sp\|P41594\|GRM5_HUMAN Metabotropic glutamate receptor 5 OS=Homo sapiens GN=GRM5 PE=1 SV=2 | 612 | DTPVVKsSSRE | 0.1 | -0.319 | -1.593 | -0.604 |
| sp\|P41594\|GRM5_HUMAN Metabotropic glutamate receptor 5 OS=Homo sapiens GN=GRM5 PE=1 SV=2 | 613 | TPVVKSsSREL | 0.043 | -0.301 | -1.596 | -0.618 |
| sp\|P41594\|GRM5_HUMAN Metabotropic glutamate receptor 5 OS=Homo sapiens GN=GRM5 PE=1 SV=2 | 614 | PVVKSSsRELC | 0.208 | 0.044 | -0.837 | -0.195 |
| sp\|P41594\|GRM5_HUMAN Metabotropic glutamate receptor 5 OS=Homo sapiens GN=GRM5 PE=1 SV=2 | 632 | CLGYLCtFCLI | 0.438 | 0.138 | -0.284 | 0.097 |
| sp\|P41594\|GRM5_HUMAN Metabotropic glutamate receptor 5 OS=Homo sapiens GN=GRM5 PE=1 SV=2 | 654 | RIGIGLsPAMS | 0.02 | -0.479 | -2.011 | -0.823 |
| sp\|P41594\|GRM5_HUMAN Metabotropic glutamate receptor 5 OS=Homo sapiens GN=GRM5 PE=1 SV=2 | 658 | GLSPAMsYSAL | 0.417 | 0.232 | -0.192 | 0.152 |
| sp\|P41594\|GRM5_HUMAN Metabotropic glutamate receptor 5 OS=Homo sapiens GN=GRM5 PE=1 SV=2 | 660 | SPAMSYsALVT | 0.161 | 0.106 | -0.729 | -0.154 |
| sp\|P41594\|GRM5_HUMAN Metabotropic glutamate receptor 5 OS=Homo sapiens GN=GRM5 PE=1 SV=2 | 664 | SYSALVtKTNR | 0.265 | -0.123 | -0.968 | -0.275 |
| sp\|P41594\|GRM5_HUMAN Metabotropic glutamate receptor 5 OS=Homo sapiens GN=GRM5 PE=1 SV=2 | 666 | SALVTKtNRIA | 0.098 | -0.209 | -1.158 | -0.423 |
| sp\|P41594\|GRM5_HUMAN Metabotropic glutamate receptor 5 OS=Homo sapiens GN=GRM5 PE=1 SV=2 | 676 | ARILAGsKKKI | 0.234 | 0.171 | -0.688 | -0.094 |
| sp\|P41594\|GRM5_HUMAN Metabotropic glutamate receptor 5 OS=Homo sapiens GN=GRM5 PE=1 SV=2 | 682 | SKKKICtKKPR | 0.475 | 0.021 | -0.19 | 0.102 |
| sp\|P41594\|GRM5_HUMAN Metabotropic glutamate receptor 5 OS=Homo sapiens GN=GRM5 PE=1 SV=2 | 689 | KKPRFMsACAQ | 0.714 | 0.911 | 0.203 | 0.609 |
| sp\|P41594\|GRM5_HUMAN Metabotropic glutamate receptor 5 OS=Homo sapiens GN=GRM5 PE=1 SV=2 | 725 | IMHDYPsIREV | 0.151 | -0.099 | -1.185 | -0.378 |

| | | | | | | |
|---|---|---|---|---|---|---|
| sp\|P41594\|GRM5_HUMAN Metabotropic glutamate receptor 5 OS=Homo sapiens GN=GRM5 PE=1 SV=2 | 735 | VYLICNtTNLG | 0.44 | 0.1 | -0.374 | 0.055 |
| sp\|P41594\|GRM5_HUMAN Metabotropic glutamate receptor 5 OS=Homo sapiens GN=GRM5 PE=1 SV=2 | 736 | YLICNTtNLGV | 0.258 | -0.026 | -0.927 | -0.232 |
| sp\|P41594\|GRM5_HUMAN Metabotropic glutamate receptor 5 OS=Homo sapiens GN=GRM5 PE=1 SV=2 | 742 | TNLGVVtPLGY | 0.092 | -0.485 | -1.255 | -0.549 |
| sp\|P41594\|GRM5_HUMAN Metabotropic glutamate receptor 5 OS=Homo sapiens GN=GRM5 PE=1 SV=2 | 753 | NGLLILsCTFY | 0.148 | -0.072 | -0.942 | -0.289 |
| sp\|P41594\|GRM5_HUMAN Metabotropic glutamate receptor 5 OS=Homo sapiens GN=GRM5 PE=1 SV=2 | 755 | LLILSCtFYAF | 0.633 | 0.449 | 0.193 | 0.425 |
| sp\|P41594\|GRM5_HUMAN Metabotropic glutamate receptor 5 OS=Homo sapiens GN=GRM5 PE=1 SV=2 | 761 | TFYAFKtRNVP | 0.169 | -0.127 | -0.732 | -0.23 |
| sp\|P41594\|GRM5_HUMAN Metabotropic glutamate receptor 5 OS=Homo sapiens GN=GRM5 PE=1 SV=2 | 777 | AKYIAFtMYTT | 0.286 | 0.172 | -0.511 | -0.018 |
| sp\|P41594\|GRM5_HUMAN Metabotropic glutamate receptor 5 OS=Homo sapiens GN=GRM5 PE=1 SV=2 | 780 | IAFTMYtTCII | 0.263 | -0.131 | -0.623 | -0.164 |
| sp\|P41594\|GRM5_HUMAN Metabotropic glutamate receptor 5 OS=Homo sapiens GN=GRM5 PE=1 SV=2 | 781 | AFTMYTtCIIW | 0.258 | 0.023 | -0.489 | -0.069 |
| sp\|P41594\|GRM5_HUMAN Metabotropic glutamate receptor 5 OS=Homo sapiens GN=GRM5 PE=1 SV=2 | 795 | VPIYFGsNYKI | 0.095 | -0.161 | -1.327 | -0.464 |
| sp\|P41594\|GRM5_HUMAN Metabotropic glutamate receptor 5 OS=Homo sapiens GN=GRM5 PE=1 SV=2 | 801 | SNYKIItMCFS | 0.07 | -0.223 | -1.316 | -0.49 |
| sp\|P41594\|GRM5_HUMAN Metabotropic glutamate receptor 5 OS=Homo sapiens GN=GRM5 PE=1 SV=2 | 805 | IITMCFsVSLS | 0.274 | 0.017 | -0.474 | -0.061 |
| sp\|P41594\|GRM5_HUMAN Metabotropic glutamate receptor 5 OS=Homo sapiens GN=GRM5 PE=1 SV=2 | 807 | TMCFSVsLSAT | 0.558 | 0.358 | 0.13 | 0.349 |
| sp\|P41594\|GRM5_HUMAN Metabotropic glutamate receptor 5 OS=Homo sapiens GN=GRM5 PE=1 SV=2 | 809 | CFSVSLsATVA | 0.318 | 0.175 | -0.379 | 0.038 |
| sp\|P41594\|GRM5_HUMAN Metabotropic glutamate receptor 5 OS=Homo sapiens GN=GRM5 PE=1 SV=2 | 811 | SVSLSAtVALG | 0.245 | 0.114 | -0.709 | -0.117 |
| sp\|P41594\|GRM5_HUMAN Metabotropic glutamate receptor 5 OS=Homo sapiens GN=GRM5 PE=1 SV=2 | 835 | PERNVRsAFTT | 0.282 | 0.157 | -0.735 | -0.099 |
| sp\|P41594\|GRM5_HUMAN Metabotropic glutamate receptor 5 OS=Homo sapiens GN=GRM5 PE=1 SV=2 | 838 | NVRSAFtTSTV | 0.295 | 0.174 | -0.699 | -0.077 |
| sp\|P41594\|GRM5_HUMAN Metabotropic glutamate receptor 5 OS=Homo sapiens GN=GRM5 PE=1 SV=2 | 839 | VRSAFTtSTVV | 0.207 | 0.042 | -1.1 | -0.284 |
| sp\|P41594\|GRM5_HUMAN Metabotropic glutamate receptor 5 OS=Homo sapiens GN=GRM5 PE=1 SV=2 | 840 | RSAFTTsTVVR | 0.166 | 0.019 | -0.746 | -0.187 |
| sp\|P41594\|GRM5_HUMAN Metabotropic glutamate receptor 5 OS=Homo sapiens GN=GRM5 PE=1 SV=2 | 841 | SAFTTStVVRM | 0.064 | -0.265 | -1.652 | -0.618 |
| sp\|P41594\|GRM5_HUMAN Metabotropic glutamate receptor 5 OS=Homo sapiens GN=GRM5 PE=1 SV=2 | 852 | HVGDGKsSSAA | 0.08 | -0.047 | -0.905 | -0.291 |
| sp\|P41594\|GRM5_HUMAN Metabotropic glutamate receptor 5 OS=Homo sapiens GN=GRM5 PE=1 SV=2 | 853 | VGDGKSsSAAS | 0.044 | -0.307 | -1.465 | -0.576 |
| sp\|P41594\|GRM5_HUMAN Metabotropic glutamate receptor 5 OS=Homo sapiens GN=GRM5 PE=1 SV=2 | 854 | GDGKSSsAASR | 0.173 | 0.042 | -0.89 | -0.225 |
| sp\|P41594\|GRM5_HUMAN Metabotropic glutamate receptor 5 OS=Homo sapiens GN=GRM5 PE=1 SV=2 | 857 | KSSSAAsRSSS | 0.069 | -0.337 | -1.587 | -0.618 |
| sp\|P41594\|GRM5_HUMAN Metabotropic glutamate receptor 5 OS=Homo sapiens GN=GRM5 PE=1 SV=2 | 859 | SSAASRsSSLV | 0.105 | -0.074 | -1.311 | -0.427 |
| sp\|P41594\|GRM5_HUMAN Metabotropic glutamate receptor 5 OS=Homo sapiens GN=GRM5 PE=1 SV=2 | 860 | SAASRSsSLVN | 0.074 | -0.144 | -1.452 | -0.507 |
| sp\|P41594\|GRM5_HUMAN Metabotropic glutamate receptor 5 OS=Homo sapiens GN=GRM5 PE=1 SV=2 | 861 | AASRSSsLVNL | 0.755 | 1.059 | 0.624 | 0.813 |
| sp\|P41594\|GRM5_HUMAN Metabotropic glutamate receptor 5 OS=Homo sapiens GN=GRM5 PE=1 SV=2 | 871 | LWKRRGsSGET | 0.735 | 0.962 | 0.597 | 0.765 |
| sp\|P41594\|GRM5_HUMAN Metabotropic glutamate receptor 5 OS=Homo sapiens GN=GRM5 PE=1 SV=2 | 872 | WKRRGSsGETL | 0.647 | 0.954 | 0.546 | 0.716 |
| sp\|P41594\|GRM5_HUMAN Metabotropic glutamate receptor 5 OS=Homo sapiens GN=GRM5 PE=1 SV=2 | 875 | RGSSGEtLRYK | 0.167 | -0.268 | -1 | -0.367 |
| sp\|P41594\|GRM5_HUMAN Metabotropic glutamate receptor 5 OS=Homo sapiens GN=GRM5 PE=1 SV=2 | 888 | RLAQHKsEIEC | 0.586 | 0.249 | 0.108 | 0.314 |
| sp\|P41594\|GRM5_HUMAN Metabotropic glutamate receptor 5 OS=Homo sapiens GN=GRM5 PE=1 SV=2 | 894 | SEIECFtPKGS | 0.043 | -0.434 | -1.691 | -0.694 |
| sp\|P41594\|GRM5_HUMAN Metabotropic glutamate receptor 5 OS=Homo sapiens GN=GRM5 PE=1 SV=2 | 898 | CFTPKGsMGNG | 0.202 | 0.048 | -0.764 | -0.171 |
| sp\|P41594\|GRM5_HUMAN Metabotropic glutamate receptor 5 OS=Homo sapiens GN=GRM5 PE=1 SV=2 | 906 | GNGGRAtMSSS | 0.1 | 0.049 | -1.173 | -0.341 |
| sp\|P41594\|GRM5_HUMAN Metabotropic glutamate receptor 5 OS=Homo sapiens GN=GRM5 PE=1 SV=2 | 908 | GGRATMsSSNG | 0.343 | 0.312 | -0.533 | 0.041 |
| sp\|P41594\|GRM5_HUMAN Metabotropic glutamate receptor 5 OS=Homo sapiens GN=GRM5 PE=1 SV=2 | 909 | GRATMSsSNGK | 0.217 | -0.023 | -0.643 | -0.15 |
| sp\|P41594\|GRM5_HUMAN Metabotropic glutamate receptor 5 OS=Homo sapiens GN=GRM5 PE=1 SV=2 | 910 | RATMSSsNGKS | 0.238 | 0.025 | -0.754 | -0.164 |
| sp\|P41594\|GRM5_HUMAN Metabotropic glutamate receptor 5 OS=Homo sapiens GN=GRM5 PE=1 SV=2 | 914 | SSSNGKsVTWA | 0.103 | -0.207 | -0.835 | -0.313 |
| sp\|P41594\|GRM5_HUMAN Metabotropic glutamate receptor 5 OS=Homo sapiens GN=GRM5 PE=1 SV=2 | 916 | SNGKSVtWAQN | 0.342 | 0.105 | -0.435 | 0.004 |
| sp\|P41594\|GRM5_HUMAN Metabotropic glutamate receptor 5 OS=Homo sapiens GN=GRM5 PE=1 SV=2 | 923 | WAQNEKsSRGQ | 0.159 | -0.238 | -0.808 | -0.296 |
| sp\|P41594\|GRM5_HUMAN Metabotropic glutamate receptor 5 OS=Homo sapiens GN=GRM5 PE=1 SV=2 | 924 | AQNEKSsRGQH | 0.038 | -0.436 | -1.82 | -0.739 |
| sp\|P41594\|GRM5_HUMAN Metabotropic glutamate receptor 5 OS=Homo sapiens GN=GRM5 PE=1 SV=2 | 934 | HLWQRLsIHIN | 0.396 | 0.345 | -0.166 | 0.192 |
| sp\|P41594\|GRM5_HUMAN Metabotropic glutamate receptor 5 OS=Homo sapiens GN=GRM5 PE=1 SV=2 | 946 | KENPNQtAVIK | 0.15 | -0.167 | -1.19 | -0.402 |
| sp\|P41594\|GRM5_HUMAN Metabotropic glutamate receptor 5 OS=Homo sapiens GN=GRM5 PE=1 SV=2 | 955 | IKPFPKsTESR | 0.212 | -0.083 | -0.526 | -0.132 |
| sp\|P41594\|GRM5_HUMAN Metabotropic glutamate receptor 5 OS=Homo sapiens GN=GRM5 PE=1 SV=2 | 956 | KPFPKStESRG | 0.05 | -0.33 | -1.663 | -0.648 |
| sp\|P41594\|GRM5_HUMAN Metabotropic glutamate receptor 5 OS=Homo sapiens GN=GRM5 PE=1 SV=2 | 958 | FPKSTEsRGLG | 0.17 | -0.052 | -0.998 | -0.293 |
| sp\|P41594\|GRM5_HUMAN Metabotropic glutamate receptor 5 OS=Homo sapiens GN=GRM5 PE=1 SV=2 | 970 | GAGAGGsAGGV | 0.111 | -0.203 | -1.435 | -0.509 |
| sp\|P41594\|GRM5_HUMAN Metabotropic glutamate receptor 5 OS=Homo sapiens GN=GRM5 PE=1 SV=2 | 977 | AGGVGAtGGAG | 0.121 | -0.104 | -0.916 | -0.3 |
| sp\|P41594\|GRM5_HUMAN Metabotropic glutamate receptor 5 OS=Homo sapiens GN=GRM5 PE=1 SV=2 | 992 | GPGGPEsPDAG | 0.076 | -0.37 | -1.259 | -0.518 |

| | | | | | | |
|---|---|---|---|---|---|---|
| sp\|P41594\|GRM5_HUMAN Metabotropic glutamate receptor 5 OS=Homo sapiens GN=GRM5 PE=1 SV=2 | 1018 | APARPRsPSPI | 0.181 | 0.31 | -0.56 | -0.023 |
| sp\|P41594\|GRM5_HUMAN Metabotropic glutamate receptor 5 OS=Homo sapiens GN=GRM5 PE=1 SV=2 | 1020 | ARPRSPsPIST | 0.277 | 0.737 | -0.08 | 0.311 |
| sp\|P41594\|GRM5_HUMAN Metabotropic glutamate receptor 5 OS=Homo sapiens GN=GRM5 PE=1 SV=2 | 1023 | RSPSPIsTLSH | 0.033 | -0.481 | -1.897 | -0.782 |
| sp\|P41594\|GRM5_HUMAN Metabotropic glutamate receptor 5 OS=Homo sapiens GN=GRM5 PE=1 SV=2 | 1024 | SPSPIStLSHR | 0.053 | -0.31 | -1.143 | -0.467 |
| sp\|P41594\|GRM5_HUMAN Metabotropic glutamate receptor 5 OS=Homo sapiens GN=GRM5 PE=1 SV=2 | 1026 | SPISTLsHRAG | 0.079 | -0.161 | -1.278 | -0.453 |
| sp\|P41594\|GRM5_HUMAN Metabotropic glutamate receptor 5 OS=Homo sapiens GN=GRM5 PE=1 SV=2 | 1031 | LSHRAGsASRT | 0.28 | 0.576 | -0.509 | 0.116 |
| sp\|P41594\|GRM5_HUMAN Metabotropic glutamate receptor 5 OS=Homo sapiens GN=GRM5 PE=1 SV=2 | 1033 | HRAGSAsRTDD | 0.64 | 0.49 | 0.32 | 0.483 |
| sp\|P41594\|GRM5_HUMAN Metabotropic glutamate receptor 5 OS=Homo sapiens GN=GRM5 PE=1 SV=2 | 1035 | AGSASRtDDDV | 0.47 | 0.189 | -0.483 | 0.059 |
| sp\|P41594\|GRM5_HUMAN Metabotropic glutamate receptor 5 OS=Homo sapiens GN=GRM5 PE=1 SV=2 | 1041 | TDDDVPsLHSE | 0.11 | -0.223 | -1.236 | -0.45 |
| sp\|P41594\|GRM5_HUMAN Metabotropic glutamate receptor 5 OS=Homo sapiens GN=GRM5 PE=1 SV=2 | 1044 | DVPSLHsEPVA | 0.479 | 0.502 | -0.297 | 0.228 |
| sp\|P41594\|GRM5_HUMAN Metabotropic glutamate receptor 5 OS=Homo sapiens GN=GRM5 PE=1 SV=2 | 1050 | SEPVARsSSSQ | 0.019 | -0.352 | -2.308 | -0.88 |
| sp\|P41594\|GRM5_HUMAN Metabotropic glutamate receptor 5 OS=Homo sapiens GN=GRM5 PE=1 SV=2 | 1051 | EPVARSsSSQG | 0.087 | -0.167 | -1.202 | -0.427 |
| sp\|P41594\|GRM5_HUMAN Metabotropic glutamate receptor 5 OS=Homo sapiens GN=GRM5 PE=1 SV=2 | 1052 | PVARSSsSQGS | 0.605 | 0.925 | 0.552 | 0.694 |
| sp\|P41594\|GRM5_HUMAN Metabotropic glutamate receptor 5 OS=Homo sapiens GN=GRM5 PE=1 SV=2 | 1053 | VARSSSsQGSL | 0.27 | 0.208 | -0.601 | -0.041 |
| sp\|P41594\|GRM5_HUMAN Metabotropic glutamate receptor 5 OS=Homo sapiens GN=GRM5 PE=1 SV=2 | 1056 | SSSSQGsLMEQ | 0.09 | -0.271 | -1.435 | -0.539 |
| sp\|P41594\|GRM5_HUMAN Metabotropic glutamate receptor 5 OS=Homo sapiens GN=GRM5 PE=1 SV=2 | 1062 | SLMEQIsSVVT | 0.062 | -0.063 | -1.39 | -0.464 |
| sp\|P41594\|GRM5_HUMAN Metabotropic glutamate receptor 5 OS=Homo sapiens GN=GRM5 PE=1 SV=2 | 1063 | LMEQISsVVTR | 0.116 | -0.021 | -0.657 | -0.187 |
| sp\|P41594\|GRM5_HUMAN Metabotropic glutamate receptor 5 OS=Homo sapiens GN=GRM5 PE=1 SV=2 | 1066 | QISSVVtRFTA | 0.234 | -0.189 | -0.801 | -0.252 |
| sp\|P41594\|GRM5_HUMAN Metabotropic glutamate receptor 5 OS=Homo sapiens GN=GRM5 PE=1 SV=2 | 1069 | SVVTRFtANIS | 0.242 | 0.12 | -0.828 | -0.155 |
| sp\|P41594\|GRM5_HUMAN Metabotropic glutamate receptor 5 OS=Homo sapiens GN=GRM5 PE=1 SV=2 | 1073 | RFTANIsELNS | 0.289 | 0.066 | -0.601 | -0.082 |
| sp\|P41594\|GRM5_HUMAN Metabotropic glutamate receptor 5 OS=Homo sapiens GN=GRM5 PE=1 SV=2 | 1077 | NISELNsMMLS | 0.128 | 0.094 | -1.232 | -0.337 |
| sp\|P41594\|GRM5_HUMAN Metabotropic glutamate receptor 5 OS=Homo sapiens GN=GRM5 PE=1 SV=2 | 1081 | LNSMMLsTAAP | 0.055 | -0.378 | -1.202 | -0.508 |
| sp\|P41594\|GRM5_HUMAN Metabotropic glutamate receptor 5 OS=Homo sapiens GN=GRM5 PE=1 SV=2 | 1082 | NSMMLStAAPS | 0.051 | -0.319 | -1.627 | -0.632 |
| sp\|P41594\|GRM5_HUMAN Metabotropic glutamate receptor 5 OS=Homo sapiens GN=GRM5 PE=1 SV=2 | 1086 | LSTAAPsPGVG | 0.162 | -0.326 | -0.852 | -0.339 |
| sp\|P41594\|GRM5_HUMAN Metabotropic glutamate receptor 5 OS=Homo sapiens GN=GRM5 PE=1 SV=2 | 1095 | VGAPLCsSYLI | 0.157 | -0.135 | -0.892 | -0.29 |
| sp\|P41594\|GRM5_HUMAN Metabotropic glutamate receptor 5 OS=Homo sapiens GN=GRM5 PE=1 SV=2 | 1096 | GAPLCSsYLIP | 0.069 | -0.292 | -1.594 | -0.606 |
| sp\|P41594\|GRM5_HUMAN Metabotropic glutamate receptor 5 OS=Homo sapiens GN=GRM5 PE=1 SV=2 | 1107 | KEIQLPtTMTT | 0.085 | -0.268 | -1.374 | -0.519 |
| sp\|P41594\|GRM5_HUMAN Metabotropic glutamate receptor 5 OS=Homo sapiens GN=GRM5 PE=1 SV=2 | 1108 | EIQLPTtMTTF | 0.147 | 0.041 | -0.859 | -0.224 |
| sp\|P41594\|GRM5_HUMAN Metabotropic glutamate receptor 5 OS=Homo sapiens GN=GRM5 PE=1 SV=2 | 1110 | QLPTTMtTFAE | 0.235 | 0.153 | -0.532 | -0.048 |
| sp\|P41594\|GRM5_HUMAN Metabotropic glutamate receptor 5 OS=Homo sapiens GN=GRM5 PE=1 SV=2 | 1111 | LPTTMTtFAEI | 0.303 | -0.057 | -0.379 | -0.044 |
| sp\|P41594\|GRM5_HUMAN Metabotropic glutamate receptor 5 OS=Homo sapiens GN=GRM5 PE=1 SV=2 | 1124 | LPAIEVtGGAQ | 0.242 | -0.086 | -0.632 | -0.159 |
| sp\|P41594\|GRM5_HUMAN Metabotropic glutamate receptor 5 OS=Homo sapiens GN=GRM5 PE=1 SV=2 | 1143 | GDAAREsPAAG | 0.134 | -0.289 | -1.054 | -0.403 |
| sp\|P41594\|GRM5_HUMAN Metabotropic glutamate receptor 5 OS=Homo sapiens GN=GRM5 PE=1 SV=2 | 1164 | EELVALtPPSP | 0.074 | 0.02 | -1.109 | -0.338 |
| sp\|P41594\|GRM5_HUMAN Metabotropic glutamate receptor 5 OS=Homo sapiens GN=GRM5 PE=1 SV=2 | 1167 | VALTPPsPFRD | 0.071 | -0.622 | -1.445 | -0.665 |
| sp\|P41594\|GRM5_HUMAN Metabotropic glutamate receptor 5 OS=Homo sapiens GN=GRM5 PE=1 SV=2 | 1172 | PSPFRDsVDSG | 0.231 | -0.022 | -0.842 | -0.211 |
| sp\|P41594\|GRM5_HUMAN Metabotropic glutamate receptor 5 OS=Homo sapiens GN=GRM5 PE=1 SV=2 | 1175 | FRDSVDsGSTT | 0.087 | -0.107 | -1.355 | -0.458 |
| sp\|P41594\|GRM5_HUMAN Metabotropic glutamate receptor 5 OS=Homo sapiens GN=GRM5 PE=1 SV=2 | 1177 | DSVDSGsTTPN | 0.242 | -0.036 | -1.012 | -0.269 |
| sp\|P41594\|GRM5_HUMAN Metabotropic glutamate receptor 5 OS=Homo sapiens GN=GRM5 PE=1 SV=2 | 1178 | SVDSGStTPNS | 0.096 | 0.171 | -1.243 | -0.325 |
| sp\|P41594\|GRM5_HUMAN Metabotropic glutamate receptor 5 OS=Homo sapiens GN=GRM5 PE=1 SV=2 | 1179 | VDSGSTtPNSP | 0.048 | -0.355 | -1.191 | -0.499 |
| sp\|P41594\|GRM5_HUMAN Metabotropic glutamate receptor 5 OS=Homo sapiens GN=GRM5 PE=1 SV=2 | 1182 | GSTTPNsPVSE | 0.073 | -0.481 | -1.529 | -0.646 |
| sp\|P41594\|GRM5_HUMAN Metabotropic glutamate receptor 5 OS=Homo sapiens GN=GRM5 PE=1 SV=2 | 1185 | TPNSPVsESAL | 0.147 | -0.132 | -0.899 | -0.295 |
| sp\|P41594\|GRM5_HUMAN Metabotropic glutamate receptor 5 OS=Homo sapiens GN=GRM5 PE=1 SV=2 | 1187 | NSPVSEsALCI | 0.164 | 0.105 | -0.955 | -0.229 |
| sp\|P41594\|GRM5_HUMAN Metabotropic glutamate receptor 5 OS=Homo sapiens GN=GRM5 PE=1 SV=2 | 1193 | SALCIPsSPKY | 0.378 | 0.253 | -0.517 | 0.038 |
| sp\|P41594\|GRM5_HUMAN Metabotropic glutamate receptor 5 OS=Homo sapiens GN=GRM5 PE=1 SV=2 | 1194 | ALCIPSsPKYD | 0.072 | -0.43 | -1.358 | -0.572 |
| sp\|P41594\|GRM5_HUMAN Metabotropic glutamate receptor 5 OS=Homo sapiens GN=GRM5 PE=1 SV=2 | 1199 | SSPKYDtLIIR | 0.268 | -0.126 | -0.709 | -0.189 |
| sp\|P41594\|GRM5_HUMAN Metabotropic glutamate receptor 5 OS=Homo sapiens GN=GRM5 PE=1 SV=2 | 1206 | LIIRDYtQSSS | 0.238 | 0.785 | -0.343 | 0.227 |
| sp\|P41594\|GRM5_HUMAN Metabotropic glutamate receptor 5 OS=Homo sapiens GN=GRM5 PE=1 SV=2 | 1208 | IRDYTQsSSSL | 0.076 | 0.119 | -0.957 | -0.254 |
| sp\|P41594\|GRM5_HUMAN Metabotropic glutamate receptor 5 OS=Homo sapiens GN=GRM5 PE=1 SV=2 | 1209 | RDYTQSsSSL- | 0.034 | -0.26 | -1.857 | -0.694 |
| sp\|P41594\|GRM5_HUMAN Metabotropic glutamate receptor 5 OS=Homo sapiens GN=GRM5 PE=1 SV=2 | 1210 | DYTQSSsSL-- | 0.283 | 0.224 | -0.576 | -0.023 |

| | | | | | | |
|---|---|---|---|---|---|---|
| sp\|P41594\|GRM5_HUMAN Metabotropic glutamate receptor 5 OS=Homo sapiens GN=GRM5 PE=1 SV=2 | 1211 | YTQSSSsL--- | 0.438 | 0.346 | -0.175 | 0.203 |
| sp\|O15303\|GRM6_HUMAN Metabotropic glutamate receptor 6 OS=Homo sapiens GN=GRM6 PE=1 SV=2 | 318 | KTSPILsLEDV | 0.236 | -0.072 | -0.761 | -0.199 |
| sp\|O15303\|GRM6_HUMAN Metabotropic glutamate receptor 6 OS=Homo sapiens GN=GRM6 PE=1 SV=2 | 328 | VAVGAItILPK | 0.1 | -0.236 | -1.082 | -0.406 |
| sp\|O15303\|GRM6_HUMAN Metabotropic glutamate receptor 6 OS=Homo sapiens GN=GRM6 PE=1 SV=2 | 335 | ILPKRAsIDGF | 0.59 | 0.174 | 0.082 | 0.282 |
| sp\|O15303\|GRM6_HUMAN Metabotropic glutamate receptor 6 OS=Homo sapiens GN=GRM6 PE=1 SV=2 | 345 | FDQYFMtRSLE | 0.141 | -0.014 | -1.045 | -0.306 |
| sp\|O15303\|GRM6_HUMAN Metabotropic glutamate receptor 6 OS=Homo sapiens GN=GRM6 PE=1 SV=2 | 347 | QYFMTRsLENN | 0.229 | 0.129 | -0.821 | -0.154 |
| sp\|O15303\|GRM6_HUMAN Metabotropic glutamate receptor 6 OS=Homo sapiens GN=GRM6 PE=1 SV=2 | 370 | NFNCKLtSSGT | 0.055 | -0.327 | -1.46 | -0.577 |
| sp\|O15303\|GRM6_HUMAN Metabotropic glutamate receptor 6 OS=Homo sapiens GN=GRM6 PE=1 SV=2 | 371 | FNCKLTsSGTQ | 0.247 | -0.087 | -0.825 | -0.222 |
| sp\|O15303\|GRM6_HUMAN Metabotropic glutamate receptor 6 OS=Homo sapiens GN=GRM6 PE=1 SV=2 | 372 | NCKLTSsGTQS | 0.144 | -0.106 | -1.07 | -0.344 |
| sp\|O15303\|GRM6_HUMAN Metabotropic glutamate receptor 6 OS=Homo sapiens GN=GRM6 PE=1 SV=2 | 374 | KLTSSGtQSDD | 0.349 | 0.087 | -0.561 | -0.042 |
| sp\|O15303\|GRM6_HUMAN Metabotropic glutamate receptor 6 OS=Homo sapiens GN=GRM6 PE=1 SV=2 | 376 | TSSGTQsDDST | 0.082 | -0.143 | -1.126 | -0.396 |
| sp\|O15303\|GRM6_HUMAN Metabotropic glutamate receptor 6 OS=Homo sapiens GN=GRM6 PE=1 SV=2 | 379 | GTQSDDsTRKC | 0.128 | -0.323 | -1.592 | -0.596 |
| sp\|O15303\|GRM6_HUMAN Metabotropic glutamate receptor 6 OS=Homo sapiens GN=GRM6 PE=1 SV=2 | 380 | TQSDDStRKCT | 0.045 | -0.33 | -1.529 | -0.605 |
| sp\|O15303\|GRM6_HUMAN Metabotropic glutamate receptor 6 OS=Homo sapiens GN=GRM6 PE=1 SV=2 | 384 | DSTRKCtGEER | 0.62 | 0.708 | 0.033 | 0.454 |
| sp\|O15303\|GRM6_HUMAN Metabotropic glutamate receptor 6 OS=Homo sapiens GN=GRM6 PE=1 SV=2 | 393 | ERIGRDsTYEQ | 0.23 | 0.227 | -0.785 | -0.109 |
| sp\|O15303\|GRM6_HUMAN Metabotropic glutamate receptor 6 OS=Homo sapiens GN=GRM6 PE=1 SV=2 | 394 | RIGRDStYEQE | 0.354 | 0.709 | -0.183 | 0.293 |
| sp\|O15303\|GRM6_HUMAN Metabotropic glutamate receptor 6 OS=Homo sapiens GN=GRM6 PE=1 SV=2 | 417 | IAHALHsMHQA | 0.229 | -0.002 | -0.882 | -0.218 |
| sp\|O15303\|GRM6_HUMAN Metabotropic glutamate receptor 6 OS=Homo sapiens GN=GRM6 PE=1 SV=2 | 427 | ALCPGHtGLCP | 0.11 | -0.059 | -1.18 | -0.376 |
| sp\|O15303\|GRM6_HUMAN Metabotropic glutamate receptor 6 OS=Homo sapiens GN=GRM6 PE=1 SV=2 | 436 | CPAMEPtDGRM | 0.288 | -0.193 | -0.829 | -0.245 |
| sp\|O15303\|GRM6_HUMAN Metabotropic glutamate receptor 6 OS=Homo sapiens GN=GRM6 PE=1 SV=2 | 453 | AVRFNGsAGTP | 0.312 | 0.124 | -0.67 | -0.078 |
| sp\|O15303\|GRM6_HUMAN Metabotropic glutamate receptor 6 OS=Homo sapiens GN=GRM6 PE=1 SV=2 | 456 | FNGSAGtPVMF | 0.032 | -0.509 | -1.765 | -0.747 |
| sp\|O15303\|GRM6_HUMAN Metabotropic glutamate receptor 6 OS=Homo sapiens GN=GRM6 PE=1 SV=2 | 478 | IFQYQAtNGSA | 0.243 | 0.036 | -0.607 | -0.109 |
| sp\|O15303\|GRM6_HUMAN Metabotropic glutamate receptor 6 OS=Homo sapiens GN=GRM6 PE=1 SV=2 | 481 | YQATNGsASSG | 0.059 | -0.219 | -1.287 | -0.482 |
| sp\|O15303\|GRM6_HUMAN Metabotropic glutamate receptor 6 OS=Homo sapiens GN=GRM6 PE=1 SV=2 | 483 | ATNGSAsSGGY | 0.49 | 0.144 | -0.394 | 0.08 |
| sp\|O15303\|GRM6_HUMAN Metabotropic glutamate receptor 6 OS=Homo sapiens GN=GRM6 PE=1 SV=2 | 484 | TNGSASsGGYQ | 0.071 | -0.32 | -1.365 | -0.538 |
| sp\|O15303\|GRM6_HUMAN Metabotropic glutamate receptor 6 OS=Homo sapiens GN=GRM6 PE=1 SV=2 | 496 | VGQWAEtLRLD | 0.552 | 0.05 | -0.041 | 0.187 |
| sp\|O15303\|GRM6_HUMAN Metabotropic glutamate receptor 6 OS=Homo sapiens GN=GRM6 PE=1 SV=2 | 507 | VEALQWsGDPH | 0.075 | -0.342 | -1.494 | -0.587 |
| sp\|O15303\|GRM6_HUMAN Metabotropic glutamate receptor 6 OS=Homo sapiens GN=GRM6 PE=1 SV=2 | 515 | DPHEVPsSLCS | 0.042 | -0.274 | -1.971 | -0.734 |
| sp\|O15303\|GRM6_HUMAN Metabotropic glutamate receptor 6 OS=Homo sapiens GN=GRM6 PE=1 SV=2 | 516 | PHEVPSsLCSL | 0.059 | -0.226 | -1.333 | -0.5 |
| sp\|O15303\|GRM6_HUMAN Metabotropic glutamate receptor 6 OS=Homo sapiens GN=GRM6 PE=1 SV=2 | 519 | VPSSLCsLPCG | 0.611 | 0.416 | -0.187 | 0.28 |
| sp\|O15303\|GRM6_HUMAN Metabotropic glutamate receptor 6 OS=Homo sapiens GN=GRM6 PE=1 SV=2 | 554 | FQVDEFtCEAC | 0.157 | 0.102 | -0.989 | -0.243 |
| sp\|O15303\|GRM6_HUMAN Metabotropic glutamate receptor 6 OS=Homo sapiens GN=GRM6 PE=1 SV=2 | 565 | PGDMRPtPNHT | 0.109 | -0.391 | -0.904 | -0.395 |
| sp\|O15303\|GRM6_HUMAN Metabotropic glutamate receptor 6 OS=Homo sapiens GN=GRM6 PE=1 SV=2 | 569 | RPTPNHtGCRP | 0.13 | -0.241 | -1.155 | -0.422 |
| sp\|O15303\|GRM6_HUMAN Metabotropic glutamate receptor 6 OS=Homo sapiens GN=GRM6 PE=1 SV=2 | 574 | HTGCRPtPVVR | 0.143 | -0.192 | -1.087 | -0.379 |
| sp\|O15303\|GRM6_HUMAN Metabotropic glutamate receptor 6 OS=Homo sapiens GN=GRM6 PE=1 SV=2 | 580 | TPVVRLsWSSP | 0.033 | -0.125 | -1.679 | -0.59 |
| sp\|O15303\|GRM6_HUMAN Metabotropic glutamate receptor 6 OS=Homo sapiens GN=GRM6 PE=1 SV=2 | 582 | VVRLSWsSPWA | 0.814 | 0.816 | 0.688 | 0.773 |
| sp\|O15303\|GRM6_HUMAN Metabotropic glutamate receptor 6 OS=Homo sapiens GN=GRM6 PE=1 SV=2 | 583 | VRLSWSsPWAA | 0.132 | -0.13 | -0.886 | -0.295 |
| sp\|O15303\|GRM6_HUMAN Metabotropic glutamate receptor 6 OS=Homo sapiens GN=GRM6 PE=1 SV=2 | 600 | VLGIVAtTTVV | 0.274 | 0.042 | -1.017 | -0.234 |
| sp\|O15303\|GRM6_HUMAN Metabotropic glutamate receptor 6 OS=Homo sapiens GN=GRM6 PE=1 SV=2 | 601 | LGIVATtTVVA | 0.141 | -0.005 | -0.782 | -0.215 |
| sp\|O15303\|GRM6_HUMAN Metabotropic glutamate receptor 6 OS=Homo sapiens GN=GRM6 PE=1 SV=2 | 602 | GIVATTtVVAT | 0.211 | 0.117 | -0.574 | -0.082 |
| sp\|O15303\|GRM6_HUMAN Metabotropic glutamate receptor 6 OS=Homo sapiens GN=GRM6 PE=1 SV=2 | 606 | TTTVVAtFVRY | 0.289 | -0.042 | -0.9 | -0.218 |
| sp\|O15303\|GRM6_HUMAN Metabotropic glutamate receptor 6 OS=Homo sapiens GN=GRM6 PE=1 SV=2 | 613 | FVRYNNtPIVR | 0.323 | 0.131 | -0.323 | 0.044 |
| sp\|O15303\|GRM6_HUMAN Metabotropic glutamate receptor 6 OS=Homo sapiens GN=GRM6 PE=1 SV=2 | 619 | TPIVRAsGREL | 0.226 | 0.027 | -0.778 | -0.175 |
| sp\|O15303\|GRM6_HUMAN Metabotropic glutamate receptor 6 OS=Homo sapiens GN=GRM6 PE=1 SV=2 | 624 | ASGRELsYVLL | 0.245 | 0.643 | -0.466 | 0.141 |
| sp\|O15303\|GRM6_HUMAN Metabotropic glutamate receptor 6 OS=Homo sapiens GN=GRM6 PE=1 SV=2 | 629 | LSYVLLtGIFL | 0.023 | -0.321 | -1.589 | -0.629 |
| sp\|O15303\|GRM6_HUMAN Metabotropic glutamate receptor 6 OS=Homo sapiens GN=GRM6 PE=1 SV=2 | 638 | FLIYAItFLMV | 0.504 | 0.263 | -0.349 | 0.139 |
| sp\|O15303\|GRM6_HUMAN Metabotropic glutamate receptor 6 OS=Homo sapiens GN=GRM6 PE=1 SV=2 | 661 | LFLGLGtTLSY | 0.049 | -0.212 | -1.426 | -0.53 |
| sp\|O15303\|GRM6_HUMAN Metabotropic glutamate receptor 6 OS=Homo sapiens GN=GRM6 PE=1 SV=2 | 662 | FLGLGTtLSYS | 0.162 | 0.024 | -0.868 | -0.227 |
| sp\|O15303\|GRM6_HUMAN Metabotropic glutamate receptor 6 OS=Homo sapiens GN=GRM6 PE=1 SV=2 | 664 | GLGTTLsYSAL | 0.099 | 0.044 | -0.986 | -0.281 |

| | | | | | | |
|---|---|---|---|---|---|---|
| sp\|O15303\|GRM6_HUMAN Metabotropic glutamate receptor 6 OS=Homo sapiens GN=GRM6 PE=1 SV=2 | 666 | GTTLSYsALLT | 0.342 | 0.268 | -0.414 | 0.065 |
| sp\|O15303\|GRM6_HUMAN Metabotropic glutamate receptor 6 OS=Homo sapiens GN=GRM6 PE=1 SV=2 | 670 | SYSALLtKTNR | 0.112 | -0.179 | -1.337 | -0.468 |
| sp\|O15303\|GRM6_HUMAN Metabotropic glutamate receptor 6 OS=Homo sapiens GN=GRM6 PE=1 SV=2 | 672 | SALLTKtNRIY | 0.182 | -0.121 | -1.064 | -0.334 |
| sp\|O15303\|GRM6_HUMAN Metabotropic glutamate receptor 6 OS=Homo sapiens GN=GRM6 PE=1 SV=2 | 685 | FEQGKRsVTPP | 0.077 | -0.117 | -1.385 | -0.475 |
| sp\|O15303\|GRM6_HUMAN Metabotropic glutamate receptor 6 OS=Homo sapiens GN=GRM6 PE=1 SV=2 | 687 | QGKRSVtPPPF | 0.892 | 1.201 | 1.388 | 1.16 |
| sp\|O15303\|GRM6_HUMAN Metabotropic glutamate receptor 6 OS=Homo sapiens GN=GRM6 PE=1 SV=2 | 693 | TPPPFIsPTSQ | 0.028 | -0.652 | -2.064 | -0.896 |
| sp\|O15303\|GRM6_HUMAN Metabotropic glutamate receptor 6 OS=Homo sapiens GN=GRM6 PE=1 SV=2 | 695 | PPFISPtSQLV | 0.283 | 0.127 | -0.73 | -0.107 |
| sp\|O15303\|GRM6_HUMAN Metabotropic glutamate receptor 6 OS=Homo sapiens GN=GRM6 PE=1 SV=2 | 696 | PFISPTsQLVI | 0.147 | -0.101 | -0.909 | -0.288 |
| sp\|O15303\|GRM6_HUMAN Metabotropic glutamate receptor 6 OS=Homo sapiens GN=GRM6 PE=1 SV=2 | 701 | TSQLVItFSLT | 0.165 | -0.106 | -0.927 | -0.289 |
| sp\|O15303\|GRM6_HUMAN Metabotropic glutamate receptor 6 OS=Homo sapiens GN=GRM6 PE=1 SV=2 | 703 | QLVITFsLTSL | 0.241 | 0.207 | -0.409 | 0.013 |
| sp\|O15303\|GRM6_HUMAN Metabotropic glutamate receptor 6 OS=Homo sapiens GN=GRM6 PE=1 SV=2 | 705 | VITFSLtSLQV | 0.22 | 0.123 | -0.749 | -0.135 |
| sp\|O15303\|GRM6_HUMAN Metabotropic glutamate receptor 6 OS=Homo sapiens GN=GRM6 PE=1 SV=2 | 706 | ITFSLTsLQVV | 0.338 | -0.011 | -0.717 | -0.13 |
| sp\|O15303\|GRM6_HUMAN Metabotropic glutamate receptor 6 OS=Homo sapiens GN=GRM6 PE=1 SV=2 | 723 | GARPPHsVIDY | 0.584 | 0.315 | -0.011 | 0.296 |
| sp\|O15303\|GRM6_HUMAN Metabotropic glutamate receptor 6 OS=Homo sapiens GN=GRM6 PE=1 SV=2 | 732 | DYEEQRtVDPE | 0.131 | -0.109 | -1.295 | -0.424 |
| sp\|O15303\|GRM6_HUMAN Metabotropic glutamate receptor 6 OS=Homo sapiens GN=GRM6 PE=1 SV=2 | 747 | VLKCDMsDLSL | 0.314 | 0.153 | -0.61 | -0.048 |
| sp\|O15303\|GRM6_HUMAN Metabotropic glutamate receptor 6 OS=Homo sapiens GN=GRM6 PE=1 SV=2 | 750 | CDMSDLsLIGC | 0.085 | -0.221 | -0.973 | -0.37 |
| sp\|O15303\|GRM6_HUMAN Metabotropic glutamate receptor 6 OS=Homo sapiens GN=GRM6 PE=1 SV=2 | 758 | IGCLGYsLLLM | 0.216 | 0.029 | -0.457 | -0.071 |
| sp\|O15303\|GRM6_HUMAN Metabotropic glutamate receptor 6 OS=Homo sapiens GN=GRM6 PE=1 SV=2 | 764 | SLLLMVtCTVY | 0.491 | 0.083 | -0.565 | 0.003 |
| sp\|O15303\|GRM6_HUMAN Metabotropic glutamate receptor 6 OS=Homo sapiens GN=GRM6 PE=1 SV=2 | 766 | LLMVTCtVYAI | 0.31 | 0.27 | -0.275 | 0.102 |
| sp\|O15303\|GRM6_HUMAN Metabotropic glutamate receptor 6 OS=Homo sapiens GN=GRM6 PE=1 SV=2 | 778 | ARGVPEtFNEA | 0.183 | 0.07 | -0.724 | -0.157 |
| sp\|O15303\|GRM6_HUMAN Metabotropic glutamate receptor 6 OS=Homo sapiens GN=GRM6 PE=1 SV=2 | 788 | AKPIGFtMYTT | 0.108 | -0.03 | -1.071 | -0.331 |
| sp\|O15303\|GRM6_HUMAN Metabotropic glutamate receptor 6 OS=Homo sapiens GN=GRM6 PE=1 SV=2 | 791 | IGFTMYtTCII | 0.285 | -0.102 | -0.474 | -0.097 |
| sp\|O15303\|GRM6_HUMAN Metabotropic glutamate receptor 6 OS=Homo sapiens GN=GRM6 PE=1 SV=2 | 792 | GFTMYTtCIIW | 0.336 | 0.103 | -0.327 | 0.037 |
| sp\|O15303\|GRM6_HUMAN Metabotropic glutamate receptor 6 OS=Homo sapiens GN=GRM6 PE=1 SV=2 | 806 | VPIFFGtAQSA | 0.082 | -0.185 | -1.484 | -0.529 |
| sp\|O15303\|GRM6_HUMAN Metabotropic glutamate receptor 6 OS=Homo sapiens GN=GRM6 PE=1 SV=2 | 809 | FFGTAQsAEKI | 0.294 | 0.177 | -0.492 | -0.007 |
| sp\|O15303\|GRM6_HUMAN Metabotropic glutamate receptor 6 OS=Homo sapiens GN=GRM6 PE=1 SV=2 | 817 | EKIYIQtTTLT | 0.243 | 0.035 | -0.406 | -0.043 |
| sp\|O15303\|GRM6_HUMAN Metabotropic glutamate receptor 6 OS=Homo sapiens GN=GRM6 PE=1 SV=2 | 818 | KIYIQTtTLTV | 0.076 | -0.07 | -1.479 | -0.491 |
| sp\|O15303\|GRM6_HUMAN Metabotropic glutamate receptor 6 OS=Homo sapiens GN=GRM6 PE=1 SV=2 | 819 | IYIQTTtLTVS | 0.426 | 0.153 | -0.215 | 0.121 |
| sp\|O15303\|GRM6_HUMAN Metabotropic glutamate receptor 6 OS=Homo sapiens GN=GRM6 PE=1 SV=2 | 821 | IQTTTLtVSLS | 0.057 | -0.147 | -1.158 | -0.416 |
| sp\|O15303\|GRM6_HUMAN Metabotropic glutamate receptor 6 OS=Homo sapiens GN=GRM6 PE=1 SV=2 | 823 | TTTLTVsLSLS | 0.224 | -0.019 | -0.726 | -0.174 |
| sp\|O15303\|GRM6_HUMAN Metabotropic glutamate receptor 6 OS=Homo sapiens GN=GRM6 PE=1 SV=2 | 825 | TLTVSLsLSAS | 0.32 | 0.267 | -0.123 | 0.155 |
| sp\|O15303\|GRM6_HUMAN Metabotropic glutamate receptor 6 OS=Homo sapiens GN=GRM6 PE=1 SV=2 | 827 | TVSLSLsASVS | 0.097 | 0.057 | -1.124 | -0.323 |
| sp\|O15303\|GRM6_HUMAN Metabotropic glutamate receptor 6 OS=Homo sapiens GN=GRM6 PE=1 SV=2 | 829 | SLSLSAsVSLG | 0.346 | 0.201 | -0.499 | 0.016 |
| sp\|O15303\|GRM6_HUMAN Metabotropic glutamate receptor 6 OS=Homo sapiens GN=GRM6 PE=1 SV=2 | 831 | SLSASVsLGML | 0.735 | 0.298 | 0.335 | 0.456 |
| sp\|O15303\|GRM6_HUMAN Metabotropic glutamate receptor 6 OS=Homo sapiens GN=GRM6 PE=1 SV=2 | 840 | MLYVPKtYVIL | 0.238 | 0.086 | -0.404 | -0.027 |
| sp\|O15303\|GRM6_HUMAN Metabotropic glutamate receptor 6 OS=Homo sapiens GN=GRM6 PE=1 SV=2 | 857 | VQKRKRsLKAT | 0.579 | 0.855 | 0.594 | 0.676 |
| sp\|O15303\|GRM6_HUMAN Metabotropic glutamate receptor 6 OS=Homo sapiens GN=GRM6 PE=1 SV=2 | 861 | KRSLKAtSTVA | 0.188 | 0.054 | -1.011 | -0.256 |
| sp\|O15303\|GRM6_HUMAN Metabotropic glutamate receptor 6 OS=Homo sapiens GN=GRM6 PE=1 SV=2 | 862 | RSLKATsTVAA | 0.471 | 0.106 | -0.135 | 0.147 |
| sp\|O15303\|GRM6_HUMAN Metabotropic glutamate receptor 6 OS=Homo sapiens GN=GRM6 PE=1 SV=2 | 863 | SLKATStVAAP | 0.102 | -0.067 | -0.945 | -0.303 |
| sp\|Q14831\|GRM7_HUMAN Metabotropic glutamate receptor 7 OS=Homo sapiens GN=GRM7 PE=1 SV=1 | 315 | LWVGSDsWGSK | 0.192 | 0.161 | -0.584 | -0.077 |
| sp\|Q14831\|GRM7_HUMAN Metabotropic glutamate receptor 7 OS=Homo sapiens GN=GRM7 PE=1 SV=1 | 318 | GSDSWGsKINP | 0.085 | -0.219 | -1.33 | -0.488 |
| sp\|Q14831\|GRM7_HUMAN Metabotropic glutamate receptor 7 OS=Homo sapiens GN=GRM7 PE=1 SV=1 | 335 | IAEGAItIQPK | 0.228 | -0.173 | -0.519 | -0.155 |
| sp\|Q14831\|GRM7_HUMAN Metabotropic glutamate receptor 7 OS=Homo sapiens GN=GRM7 PE=1 SV=1 | 342 | IQPKRAtVEGF | 0.365 | 0.067 | -0.264 | 0.056 |
| sp\|Q14831\|GRM7_HUMAN Metabotropic glutamate receptor 7 OS=Homo sapiens GN=GRM7 PE=1 SV=1 | 351 | GFDAYFtSRTL | 0.281 | -0.058 | -0.593 | -0.123 |
| sp\|Q14831\|GRM7_HUMAN Metabotropic glutamate receptor 7 OS=Homo sapiens GN=GRM7 PE=1 SV=1 | 352 | FDAYFTsRTLE | 0.147 | -0.156 | -0.981 | -0.33 |
| sp\|Q14831\|GRM7_HUMAN Metabotropic glutamate receptor 7 OS=Homo sapiens GN=GRM7 PE=1 SV=1 | 354 | AYFTSRtLENN | 0.505 | 0.323 | -0.415 | 0.138 |
| sp\|Q14831\|GRM7_HUMAN Metabotropic glutamate receptor 7 OS=Homo sapiens GN=GRM7 PE=1 SV=1 | 377 | NFNCKLtISGS | 0.051 | -0.307 | -1.433 | -0.563 |
| sp\|Q14831\|GRM7_HUMAN Metabotropic glutamate receptor 7 OS=Homo sapiens GN=GRM7 PE=1 SV=1 | 379 | NCKLTIsGSKK | 0.125 | -0.115 | -1.093 | -0.361 |
| sp\|Q14831\|GRM7_HUMAN Metabotropic glutamate receptor 7 OS=Homo sapiens GN=GRM7 PE=1 SV=1 | 381 | KLTISGsKKED | 0.434 | 0.209 | -0.116 | 0.176 |

| | | | | | | |
|---|---|---|---|---|---|---|
| sp\|Q14831\|GRM7_HUMAN Metabotropic glutamate receptor 7 OS=Homo sapiens GN=GRM7 PE=1 SV=1 | 386 | GSKKEDtDRKC | 0.317 | -0.21 | -1.147 | -0.347 |
| sp\|Q14831\|GRM7_HUMAN Metabotropic glutamate receptor 7 OS=Homo sapiens GN=GRM7 PE=1 SV=1 | 391 | DTDRKCtGQER | 0.696 | 0.73 | -0.007 | 0.473 |
| sp\|Q14831\|GRM7_HUMAN Metabotropic glutamate receptor 7 OS=Homo sapiens GN=GRM7 PE=1 SV=1 | 400 | ERIGKDsNYEQ | 0.081 | -0.004 | -1.379 | -0.434 |
| sp\|Q14831\|GRM7_HUMAN Metabotropic glutamate receptor 7 OS=Homo sapiens GN=GRM7 PE=1 SV=1 | 460 | NVNFNGsAGTP | 0.137 | -0.102 | -1.108 | -0.358 |
| sp\|Q14831\|GRM7_HUMAN Metabotropic glutamate receptor 7 OS=Homo sapiens GN=GRM7 PE=1 SV=1 | 463 | FNGSAGtPVMF | 0.032 | -0.509 | -1.765 | -0.747 |
| sp\|Q14831\|GRM7_HUMAN Metabotropic glutamate receptor 7 OS=Homo sapiens GN=GRM7 PE=1 SV=1 | 484 | DIFQYQtTNTS | 0.333 | 0.04 | -0.517 | -0.048 |
| sp\|Q14831\|GRM7_HUMAN Metabotropic glutamate receptor 7 OS=Homo sapiens GN=GRM7 PE=1 SV=1 | 485 | IFQYQTtNTSN | 0.168 | -0.02 | -0.882 | -0.245 |
| sp\|Q14831\|GRM7_HUMAN Metabotropic glutamate receptor 7 OS=Homo sapiens GN=GRM7 PE=1 SV=1 | 487 | QYQTTNtSNPG | 0.23 | 0.045 | -0.765 | -0.163 |
| sp\|Q14831\|GRM7_HUMAN Metabotropic glutamate receptor 7 OS=Homo sapiens GN=GRM7 PE=1 SV=1 | 488 | YQTTNTsNPGY | 0.571 | 0.392 | 0.129 | 0.364 |
| sp\|Q14831\|GRM7_HUMAN Metabotropic glutamate receptor 7 OS=Homo sapiens GN=GRM7 PE=1 SV=1 | 499 | RLIGQWtDELQ | 0.222 | 0.066 | -0.967 | -0.226 |
| sp\|Q14831\|GRM7_HUMAN Metabotropic glutamate receptor 7 OS=Homo sapiens GN=GRM7 PE=1 SV=1 | 521 | VREIPAsVCTL | 0.171 | 0.038 | -0.745 | -0.179 |
| sp\|Q14831\|GRM7_HUMAN Metabotropic glutamate receptor 7 OS=Homo sapiens GN=GRM7 PE=1 SV=1 | 524 | IPASVCtLPCK | 0.763 | 0.466 | 0.249 | 0.493 |
| sp\|Q14831\|GRM7_HUMAN Metabotropic glutamate receptor 7 OS=Homo sapiens GN=GRM7 PE=1 SV=1 | 535 | PGQRKKtQKGT | 0.556 | 0.744 | 0.336 | 0.545 |
| sp\|Q14831\|GRM7_HUMAN Metabotropic glutamate receptor 7 OS=Homo sapiens GN=GRM7 PE=1 SV=1 | 539 | KKTQKGtPCCW | 0.056 | -0.438 | -1.46 | -0.614 |
| sp\|Q14831\|GRM7_HUMAN Metabotropic glutamate receptor 7 OS=Homo sapiens GN=GRM7 PE=1 SV=1 | 544 | GTPCCWtCEPC | 0.237 | -0.155 | -1.236 | -0.385 |
| sp\|Q14831\|GRM7_HUMAN Metabotropic glutamate receptor 7 OS=Homo sapiens GN=GRM7 PE=1 SV=1 | 559 | YQFDEMtCQHC | 0.153 | 0.031 | -0.954 | -0.257 |
| sp\|Q14831\|GRM7_HUMAN Metabotropic glutamate receptor 7 OS=Homo sapiens GN=GRM7 PE=1 SV=1 | 574 | RPNENRtGCQD | 0.084 | -0.24 | -1.376 | -0.511 |
| sp\|Q14831\|GRM7_HUMAN Metabotropic glutamate receptor 7 OS=Homo sapiens GN=GRM7 PE=1 SV=1 | 588 | IKLEWHsPWAV | 0.416 | 0.019 | -0.523 | -0.029 |
| sp\|Q14831\|GRM7_HUMAN Metabotropic glutamate receptor 7 OS=Homo sapiens GN=GRM7 PE=1 SV=1 | 605 | MLGIIAtIFVM | 0.289 | 0.157 | -0.31 | 0.045 |
| sp\|Q14831\|GRM7_HUMAN Metabotropic glutamate receptor 7 OS=Homo sapiens GN=GRM7 PE=1 SV=1 | 611 | TIFVMAtFIRY | 0.283 | 0.016 | -0.67 | -0.124 |
| sp\|Q14831\|GRM7_HUMAN Metabotropic glutamate receptor 7 OS=Homo sapiens GN=GRM7 PE=1 SV=1 | 618 | FIRYNDtPIVR | 0.284 | 0.07 | -0.451 | -0.032 |
| sp\|Q14831\|GRM7_HUMAN Metabotropic glutamate receptor 7 OS=Homo sapiens GN=GRM7 PE=1 SV=1 | 624 | TPIVRAsGREL | 0.226 | 0.027 | -0.778 | -0.175 |
| sp\|Q14831\|GRM7_HUMAN Metabotropic glutamate receptor 7 OS=Homo sapiens GN=GRM7 PE=1 SV=1 | 629 | ASGRELsYVLL | 0.245 | 0.643 | -0.466 | 0.141 |
| sp\|Q14831\|GRM7_HUMAN Metabotropic glutamate receptor 7 OS=Homo sapiens GN=GRM7 PE=1 SV=1 | 634 | LSYVLLtGIFL | 0.023 | -0.321 | -1.589 | -0.629 |
| sp\|Q14831\|GRM7_HUMAN Metabotropic glutamate receptor 7 OS=Homo sapiens GN=GRM7 PE=1 SV=1 | 643 | FLCYIItFLMI | 0.533 | 0.229 | 0.059 | 0.274 |
| sp\|Q14831\|GRM7_HUMAN Metabotropic glutamate receptor 7 OS=Homo sapiens GN=GRM7 PE=1 SV=1 | 656 | PDVAVCsFRRV | 0.288 | -0.187 | -1.156 | -0.352 |
| sp\|Q14831\|GRM7_HUMAN Metabotropic glutamate receptor 7 OS=Homo sapiens GN=GRM7 PE=1 SV=1 | 669 | GLGMCIsYAAL | 0.383 | 0.073 | -0.335 | 0.04 |
| sp\|Q14831\|GRM7_HUMAN Metabotropic glutamate receptor 7 OS=Homo sapiens GN=GRM7 PE=1 SV=1 | 675 | SYAALLtKTNR | 0.121 | -0.148 | -1.199 | -0.409 |
| sp\|Q14831\|GRM7_HUMAN Metabotropic glutamate receptor 7 OS=Homo sapiens GN=GRM7 PE=1 SV=1 | 677 | AALLTKtNRIY | 0.246 | -0.098 | -0.896 | -0.249 |
| sp\|Q14831\|GRM7_HUMAN Metabotropic glutamate receptor 7 OS=Homo sapiens GN=GRM7 PE=1 SV=1 | 690 | FEQGKKsVTAP | 0.23 | 0.092 | -0.547 | -0.075 |
| sp\|Q14831\|GRM7_HUMAN Metabotropic glutamate receptor 7 OS=Homo sapiens GN=GRM7 PE=1 SV=1 | 692 | QGKKSVtAPRL | 0.917 | 0.759 | 0.946 | 0.874 |
| sp\|Q14831\|GRM7_HUMAN Metabotropic glutamate receptor 7 OS=Homo sapiens GN=GRM7 PE=1 SV=1 | 698 | TAPRLIsPTSQ | 0.13 | 0.249 | -0.999 | -0.207 |
| sp\|Q14831\|GRM7_HUMAN Metabotropic glutamate receptor 7 OS=Homo sapiens GN=GRM7 PE=1 SV=1 | 700 | PRLISPtSQLA | 0.401 | 0.349 | -0.135 | 0.205 |
| sp\|Q14831\|GRM7_HUMAN Metabotropic glutamate receptor 7 OS=Homo sapiens GN=GRM7 PE=1 SV=1 | 701 | RLISPTsQLAI | 0.256 | 0.032 | -0.632 | -0.115 |
| sp\|Q14831\|GRM7_HUMAN Metabotropic glutamate receptor 7 OS=Homo sapiens GN=GRM7 PE=1 SV=1 | 706 | TSQLAItSSLI | 0.22 | -0.03 | -0.759 | -0.19 |
| sp\|Q14831\|GRM7_HUMAN Metabotropic glutamate receptor 7 OS=Homo sapiens GN=GRM7 PE=1 SV=1 | 707 | SQLAITsSLIS | 0.062 | -0.248 | -1.229 | -0.472 |
| sp\|Q14831\|GRM7_HUMAN Metabotropic glutamate receptor 7 OS=Homo sapiens GN=GRM7 PE=1 SV=1 | 708 | QLAITSsLISV | 0.111 | 0.067 | -0.774 | -0.199 |
| sp\|Q14831\|GRM7_HUMAN Metabotropic glutamate receptor 7 OS=Homo sapiens GN=GRM7 PE=1 SV=1 | 711 | ITSSLIsVQLL | 0.199 | -0.117 | -0.82 | -0.246 |
| sp\|Q14831\|GRM7_HUMAN Metabotropic glutamate receptor 7 OS=Homo sapiens GN=GRM7 PE=1 SV=1 | 737 | DYDEHKtMNPE | 0.32 | 0.098 | -0.682 | -0.088 |
| sp\|Q14831\|GRM7_HUMAN Metabotropic glutamate receptor 7 OS=Homo sapiens GN=GRM7 PE=1 SV=1 | 752 | VLKCDItDLQI | 0.506 | 0.056 | -0.307 | 0.085 |
| sp\|Q14831\|GRM7_HUMAN Metabotropic glutamate receptor 7 OS=Homo sapiens GN=GRM7 PE=1 SV=1 | 759 | DLQIICsLGYS | 0.615 | 0.226 | -0.178 | 0.221 |
| sp\|Q14831\|GRM7_HUMAN Metabotropic glutamate receptor 7 OS=Homo sapiens GN=GRM7 PE=1 SV=1 | 763 | ICSLGYsILLM | 0.111 | -0.085 | -0.973 | -0.316 |
| sp\|Q14831\|GRM7_HUMAN Metabotropic glutamate receptor 7 OS=Homo sapiens GN=GRM7 PE=1 SV=1 | 769 | SILLMVtCTVY | 0.398 | 0.063 | -0.709 | -0.083 |
| sp\|Q14831\|GRM7_HUMAN Metabotropic glutamate receptor 7 OS=Homo sapiens GN=GRM7 PE=1 SV=1 | 771 | LLMVTCtVYAI | 0.31 | 0.27 | -0.275 | 0.102 |
| sp\|Q14831\|GRM7_HUMAN Metabotropic glutamate receptor 7 OS=Homo sapiens GN=GRM7 PE=1 SV=1 | 777 | TVYAIKtRGVP | 0.134 | -0.108 | -0.695 | -0.223 |
| sp\|Q14831\|GRM7_HUMAN Metabotropic glutamate receptor 7 OS=Homo sapiens GN=GRM7 PE=1 SV=1 | 793 | AKPIGFtMYTT | 0.108 | -0.03 | -1.071 | -0.331 |
| sp\|Q14831\|GRM7_HUMAN Metabotropic glutamate receptor 7 OS=Homo sapiens GN=GRM7 PE=1 SV=1 | 796 | IGFTMYtTCIV | 0.209 | -0.159 | -0.92 | -0.29 |
| sp\|Q14831\|GRM7_HUMAN Metabotropic glutamate receptor 7 OS=Homo sapiens GN=GRM7 PE=1 SV=1 | 797 | GFTMYTtCIVW | 0.293 | 0.135 | -0.363 | 0.022 |
| sp\|Q14831\|GRM7_HUMAN Metabotropic glutamate receptor 7 OS=Homo sapiens GN=GRM7 PE=1 SV=1 | 811 | IPIFFGtAQSA | 0.11 | -0.153 | -1.3 | -0.448 |

| | | | | | | |
|---|---|---|---|---|---|---|
| sp\|Q14831\|GRM7_HUMAN Metabotropic glutamate receptor 7 OS=Homo sapiens GN=GRM7 PE=1 SV=1 | 814 | FFGTAQsAEKL | 0.224 | 0.16 | -0.683 | -0.1 |
| sp\|Q14831\|GRM7_HUMAN Metabotropic glutamate receptor 7 OS=Homo sapiens GN=GRM7 PE=1 SV=1 | 822 | EKLYIQtTTLT | 0.26 | 0.01 | -0.304 | -0.011 |
| sp\|Q14831\|GRM7_HUMAN Metabotropic glutamate receptor 7 OS=Homo sapiens GN=GRM7 PE=1 SV=1 | 823 | KLYIQTtTLTI | 0.14 | 0.008 | -0.889 | -0.247 |
| sp\|Q14831\|GRM7_HUMAN Metabotropic glutamate receptor 7 OS=Homo sapiens GN=GRM7 PE=1 SV=1 | 824 | LYIQTTtLTIS | 0.38 | 0.118 | -0.287 | 0.07 |
| sp\|Q14831\|GRM7_HUMAN Metabotropic glutamate receptor 7 OS=Homo sapiens GN=GRM7 PE=1 SV=1 | 826 | IQTTTLtISMN | 0.051 | -0.239 | -1.289 | -0.492 |
| sp\|Q14831\|GRM7_HUMAN Metabotropic glutamate receptor 7 OS=Homo sapiens GN=GRM7 PE=1 SV=1 | 828 | TTTLTIsMNLS | 0.15 | 0.069 | -0.903 | -0.228 |
| sp\|Q14831\|GRM7_HUMAN Metabotropic glutamate receptor 7 OS=Homo sapiens GN=GRM7 PE=1 SV=1 | 832 | TISMNLsASVA | 0.11 | -0.131 | -1.085 | -0.369 |
| sp\|Q14831\|GRM7_HUMAN Metabotropic glutamate receptor 7 OS=Homo sapiens GN=GRM7 PE=1 SV=1 | 834 | SMNLSAsVALG | 0.23 | 0.228 | -0.824 | -0.122 |
| sp\|Q14831\|GRM7_HUMAN Metabotropic glutamate receptor 7 OS=Homo sapiens GN=GRM7 PE=1 SV=1 | 862 | VQKRKRsFKAV | 0.576 | 0.884 | 0.323 | 0.594 |
| sp\|Q14831\|GRM7_HUMAN Metabotropic glutamate receptor 7 OS=Homo sapiens GN=GRM7 PE=1 SV=1 | 868 | SFKAVVtAATM | 0.29 | -0.119 | -0.722 | -0.184 |
| sp\|Q14831\|GRM7_HUMAN Metabotropic glutamate receptor 7 OS=Homo sapiens GN=GRM7 PE=1 SV=1 | 871 | AVVTAAtMSSR | 0.12 | 0.021 | -1.185 | -0.348 |
| sp\|Q14831\|GRM7_HUMAN Metabotropic glutamate receptor 7 OS=Homo sapiens GN=GRM7 PE=1 SV=1 | 873 | VTAATMsSRLS | 0.143 | -0.074 | -0.902 | -0.278 |
| sp\|Q14831\|GRM7_HUMAN Metabotropic glutamate receptor 7 OS=Homo sapiens GN=GRM7 PE=1 SV=1 | 874 | TAATMSsRLSH | 0.026 | -0.552 | -2.04 | -0.855 |
| sp\|Q14831\|GRM7_HUMAN Metabotropic glutamate receptor 7 OS=Homo sapiens GN=GRM7 PE=1 SV=1 | 877 | TMSSRLsHKPS | 0.089 | -0.042 | -1.112 | -0.355 |
| sp\|Q14831\|GRM7_HUMAN Metabotropic glutamate receptor 7 OS=Homo sapiens GN=GRM7 PE=1 SV=1 | 881 | RLSHKPsDRPN | 0.224 | -0.128 | -0.883 | -0.262 |
| sp\|Q14831\|GRM7_HUMAN Metabotropic glutamate receptor 7 OS=Homo sapiens GN=GRM7 PE=1 SV=1 | 890 | PNGEAKtELCE | 0.152 | 0.051 | -0.781 | -0.193 |
| sp\|Q14831\|GRM7_HUMAN Metabotropic glutamate receptor 7 OS=Homo sapiens GN=GRM7 PE=1 SV=1 | 900 | ENVDPNsPAAK | 0.05 | -0.489 | -1.422 | -0.62 |
| sp\|Q14831\|GRM7_HUMAN Metabotropic glutamate receptor 7 OS=Homo sapiens GN=GRM7 PE=1 SV=1 | 909 | AKKKYVsYNNL | 0.724 | 0.191 | 0.107 | 0.341 |
| sp\|O00222\|GRM8_HUMAN Metabotropic glutamate receptor 8 OS=Homo sapiens GN=GRM8 PE=2 SV=2 | 330 | IAEGAVtILPK | 0.212 | -0.204 | -0.658 | -0.217 |
| sp\|O00222\|GRM8_HUMAN Metabotropic glutamate receptor 8 OS=Homo sapiens GN=GRM8 PE=2 SV=2 | 337 | ILPKRAsIDGF | 0.59 | 0.174 | 0.082 | 0.282 |
| sp\|O00222\|GRM8_HUMAN Metabotropic glutamate receptor 8 OS=Homo sapiens GN=GRM8 PE=2 SV=2 | 347 | FDRYFRsRTLA | 0.252 | 0.092 | -0.834 | -0.163 |
| sp\|O00222\|GRM8_HUMAN Metabotropic glutamate receptor 8 OS=Homo sapiens GN=GRM8 PE=2 SV=2 | 349 | RYFRSRtLANN | 0.884 | 1.258 | 0.818 | 0.987 |
| sp\|O00222\|GRM8_HUMAN Metabotropic glutamate receptor 8 OS=Homo sapiens GN=GRM8 PE=2 SV=2 | 373 | FGCKLGsHGKR | 0.306 | 0.005 | -0.679 | -0.123 |
| sp\|O00222\|GRM8_HUMAN Metabotropic glutamate receptor 8 OS=Homo sapiens GN=GRM8 PE=2 SV=2 | 379 | SHGKRNsHIKK | 0.289 | 0.063 | -0.938 | -0.195 |
| sp\|O00222\|GRM8_HUMAN Metabotropic glutamate receptor 8 OS=Homo sapiens GN=GRM8 PE=2 SV=2 | 385 | SHIKKCtGLER | 0.152 | -0.117 | -1.478 | -0.481 |
| sp\|O00222\|GRM8_HUMAN Metabotropic glutamate receptor 8 OS=Homo sapiens GN=GRM8 PE=2 SV=2 | 394 | ERIARDsSYEQ | 0.239 | 0.176 | -0.862 | -0.149 |
| sp\|O00222\|GRM8_HUMAN Metabotropic glutamate receptor 8 OS=Homo sapiens GN=GRM8 PE=2 SV=2 | 395 | RIARDSsYEQE | 0.439 | 0.732 | 0.053 | 0.408 |
| sp\|O00222\|GRM8_HUMAN Metabotropic glutamate receptor 8 OS=Homo sapiens GN=GRM8 PE=2 SV=2 | 411 | VIDAVYsMAYA | 0.153 | 0.001 | -0.961 | -0.269 |
| sp\|O00222\|GRM8_HUMAN Metabotropic glutamate receptor 8 OS=Homo sapiens GN=GRM8 PE=2 SV=2 | 435 | GLCPRMsTIDG | 0.704 | 0.396 | 0.179 | 0.426 |
| sp\|O00222\|GRM8_HUMAN Metabotropic glutamate receptor 8 OS=Homo sapiens GN=GRM8 PE=2 SV=2 | 436 | LCPRMStIDGK | 0.426 | 0.601 | 0.044 | 0.357 |
| sp\|O00222\|GRM8_HUMAN Metabotropic glutamate receptor 8 OS=Homo sapiens GN=GRM8 PE=2 SV=2 | 454 | AVNFNGsAGTP | 0.129 | -0.143 | -1.219 | -0.411 |
| sp\|O00222\|GRM8_HUMAN Metabotropic glutamate receptor 8 OS=Homo sapiens GN=GRM8 PE=2 SV=2 | 457 | FNGSAGtPVTF | 0.035 | -0.473 | -1.647 | -0.695 |
| sp\|O00222\|GRM8_HUMAN Metabotropic glutamate receptor 8 OS=Homo sapiens GN=GRM8 PE=2 SV=2 | 460 | SAGTPVtFNEN | 0.241 | -0.119 | -0.765 | -0.214 |
| sp\|O00222\|GRM8_HUMAN Metabotropic glutamate receptor 8 OS=Homo sapiens GN=GRM8 PE=2 SV=2 | 479 | IFQYQItNKST | 0.108 | -0.102 | -0.916 | -0.303 |
| sp\|O00222\|GRM8_HUMAN Metabotropic glutamate receptor 8 OS=Homo sapiens GN=GRM8 PE=2 SV=2 | 482 | YQITNKsTEYK | 0.166 | -0.095 | -0.7 | -0.21 |
| sp\|O00222\|GRM8_HUMAN Metabotropic glutamate receptor 8 OS=Homo sapiens GN=GRM8 PE=2 SV=2 | 483 | QITNKStEYKV | 0.074 | -0.041 | -1.158 | -0.375 |
| sp\|O00222\|GRM8_HUMAN Metabotropic glutamate receptor 8 OS=Homo sapiens GN=GRM8 PE=2 SV=2 | 492 | KVIGHWtNQLH | 0.113 | -0.163 | -1.338 | -0.463 |
| sp\|O00222\|GRM8_HUMAN Metabotropic glutamate receptor 8 OS=Homo sapiens GN=GRM8 PE=2 SV=2 | 510 | WAHREHtHPAS | 0.879 | 1.368 | 0.986 | 1.078 |
| sp\|O00222\|GRM8_HUMAN Metabotropic glutamate receptor 8 OS=Homo sapiens GN=GRM8 PE=2 SV=2 | 514 | EHTHPAsVCSL | 0.064 | -0.212 | -1.33 | -0.493 |
| sp\|O00222\|GRM8_HUMAN Metabotropic glutamate receptor 8 OS=Homo sapiens GN=GRM8 PE=2 SV=2 | 517 | HPASVCsLPCK | 0.713 | 0.532 | 0.229 | 0.491 |
| sp\|O00222\|GRM8_HUMAN Metabotropic glutamate receptor 8 OS=Homo sapiens GN=GRM8 PE=2 SV=2 | 528 | PGERKKtVKGV | 0.661 | 0.796 | 0.497 | 0.651 |
| sp\|O00222\|GRM8_HUMAN Metabotropic glutamate receptor 8 OS=Homo sapiens GN=GRM8 PE=2 SV=2 | 552 | YQVDELsCELC | 0.062 | -0.117 | -1.532 | -0.529 |
| sp\|O00222\|GRM8_HUMAN Metabotropic glutamate receptor 8 OS=Homo sapiens GN=GRM8 PE=2 SV=2 | 567 | RPNMNRtGCQL | 0.105 | -0.244 | -1.25 | -0.463 |
| sp\|O00222\|GRM8_HUMAN Metabotropic glutamate receptor 8 OS=Homo sapiens GN=GRM8 PE=2 SV=2 | 581 | IKLEWHsPWAV | 0.416 | 0.019 | -0.523 | -0.029 |
| sp\|O00222\|GRM8_HUMAN Metabotropic glutamate receptor 8 OS=Homo sapiens GN=GRM8 PE=2 SV=2 | 598 | ILGIIAtTFVI | 0.404 | 0.165 | -0.139 | 0.143 |
| sp\|O00222\|GRM8_HUMAN Metabotropic glutamate receptor 8 OS=Homo sapiens GN=GRM8 PE=2 SV=2 | 599 | LGIIATtFVIV | 0.315 | 0.124 | -0.543 | -0.035 |
| sp\|O00222\|GRM8_HUMAN Metabotropic glutamate receptor 8 OS=Homo sapiens GN=GRM8 PE=2 SV=2 | 604 | TTFVIVtFVRY | 0.328 | -0.052 | -0.701 | -0.142 |
| sp\|O00222\|GRM8_HUMAN Metabotropic glutamate receptor 8 OS=Homo sapiens GN=GRM8 PE=2 SV=2 | 611 | FVRYNDtPIVR | 0.207 | -0.004 | -0.595 | -0.131 |
| sp\|O00222\|GRM8_HUMAN Metabotropic glutamate receptor 8 OS=Homo sapiens GN=GRM8 PE=2 SV=2 | 617 | TPIVRAsGREL | 0.226 | 0.027 | -0.778 | -0.175 |

| | | | | | | |
|---|---|---|---|---|---|---|
| sp\|O00222\|GRM8_HUMAN Metabotropic glutamate receptor 8 OS=Homo sapiens GN=GRM8 PE=2 SV=2 | 622 | ASGRELsYVLL | 0.245 | 0.643 | -0.466 | 0.141 |
| sp\|O00222\|GRM8_HUMAN Metabotropic glutamate receptor 8 OS=Homo sapiens GN=GRM8 PE=2 SV=2 | 627 | LSYVLLtGIFL | 0.023 | -0.321 | -1.589 | -0.629 |
| sp\|O00222\|GRM8_HUMAN Metabotropic glutamate receptor 8 OS=Homo sapiens GN=GRM8 PE=2 SV=2 | 634 | GIFLCYsITFL | 0.317 | 0.119 | -0.516 | -0.027 |
| sp\|O00222\|GRM8_HUMAN Metabotropic glutamate receptor 8 OS=Homo sapiens GN=GRM8 PE=2 SV=2 | 636 | FLCYSItFLMI | 0.728 | 0.45 | 0.415 | 0.531 |
| sp\|O00222\|GRM8_HUMAN Metabotropic glutamate receptor 8 OS=Homo sapiens GN=GRM8 PE=2 SV=2 | 645 | MIAAPDtIICS | 0.114 | -0.097 | -0.854 | -0.279 |
| sp\|O00222\|GRM8_HUMAN Metabotropic glutamate receptor 8 OS=Homo sapiens GN=GRM8 PE=2 SV=2 | 649 | PDTIICsFRRV | 0.273 | -0.123 | -0.889 | -0.246 |
| sp\|O00222\|GRM8_HUMAN Metabotropic glutamate receptor 8 OS=Homo sapiens GN=GRM8 PE=2 SV=2 | 662 | GLGMCFsYAAL | 0.372 | 0.12 | -0.297 | 0.065 |
| sp\|O00222\|GRM8_HUMAN Metabotropic glutamate receptor 8 OS=Homo sapiens GN=GRM8 PE=2 SV=2 | 668 | SYAALLtKTNR | 0.121 | -0.148 | -1.199 | -0.409 |
| sp\|O00222\|GRM8_HUMAN Metabotropic glutamate receptor 8 OS=Homo sapiens GN=GRM8 PE=2 SV=2 | 670 | AALLTKtNRIH | 0.13 | -0.256 | -1.262 | -0.463 |
| sp\|O00222\|GRM8_HUMAN Metabotropic glutamate receptor 8 OS=Homo sapiens GN=GRM8 PE=2 SV=2 | 683 | FEQGKKsVTAP | 0.23 | 0.092 | -0.547 | -0.075 |
| sp\|O00222\|GRM8_HUMAN Metabotropic glutamate receptor 8 OS=Homo sapiens GN=GRM8 PE=2 SV=2 | 685 | QGKKSVtAPKF | 0.868 | 0.741 | 0.856 | 0.822 |
| sp\|O00222\|GRM8_HUMAN Metabotropic glutamate receptor 8 OS=Homo sapiens GN=GRM8 PE=2 SV=2 | 691 | TAPKFIsPASQ | 0.043 | -0.588 | -1.873 | -0.806 |
| sp\|O00222\|GRM8_HUMAN Metabotropic glutamate receptor 8 OS=Homo sapiens GN=GRM8 PE=2 SV=2 | 694 | KFISPAsQLVI | 0.151 | -0.124 | -1.065 | -0.346 |
| sp\|O00222\|GRM8_HUMAN Metabotropic glutamate receptor 8 OS=Homo sapiens GN=GRM8 PE=2 SV=2 | 699 | ASQLVItFSLI | 0.305 | -0.002 | -0.63 | -0.109 |
| sp\|O00222\|GRM8_HUMAN Metabotropic glutamate receptor 8 OS=Homo sapiens GN=GRM8 PE=2 SV=2 | 701 | QLVITFsLISV | 0.165 | 0.179 | -0.628 | -0.095 |
| sp\|O00222\|GRM8_HUMAN Metabotropic glutamate receptor 8 OS=Homo sapiens GN=GRM8 PE=2 SV=2 | 704 | ITFSLIsVQLL | 0.224 | -0.069 | -0.78 | -0.208 |
| sp\|O00222\|GRM8_HUMAN Metabotropic glutamate receptor 8 OS=Homo sapiens GN=GRM8 PE=2 SV=2 | 730 | DYGEQRtLDPE | 0.152 | -0.035 | -1.128 | -0.337 |
| sp\|O00222\|GRM8_HUMAN Metabotropic glutamate receptor 8 OS=Homo sapiens GN=GRM8 PE=2 SV=2 | 745 | VLKCDIsDLSL | 0.254 | 0.014 | -0.775 | -0.169 |
| sp\|O00222\|GRM8_HUMAN Metabotropic glutamate receptor 8 OS=Homo sapiens GN=GRM8 PE=2 SV=2 | 748 | CDISDLsLICS | 0.084 | -0.096 | -0.969 | -0.327 |
| sp\|O00222\|GRM8_HUMAN Metabotropic glutamate receptor 8 OS=Homo sapiens GN=GRM8 PE=2 SV=2 | 752 | DLSLICsLGYS | 0.593 | 0.123 | -0.304 | 0.137 |
| sp\|O00222\|GRM8_HUMAN Metabotropic glutamate receptor 8 OS=Homo sapiens GN=GRM8 PE=2 SV=2 | 756 | ICSLGYsILLM | 0.111 | -0.085 | -0.973 | -0.316 |
| sp\|O00222\|GRM8_HUMAN Metabotropic glutamate receptor 8 OS=Homo sapiens GN=GRM8 PE=2 SV=2 | 762 | SILLMVtCTVY | 0.398 | 0.063 | -0.709 | -0.083 |
| sp\|O00222\|GRM8_HUMAN Metabotropic glutamate receptor 8 OS=Homo sapiens GN=GRM8 PE=2 SV=2 | 764 | LLMVTCtVYAI | 0.31 | 0.27 | -0.275 | 0.102 |
| sp\|O00222\|GRM8_HUMAN Metabotropic glutamate receptor 8 OS=Homo sapiens GN=GRM8 PE=2 SV=2 | 770 | TVYAIKtRGVP | 0.134 | -0.108 | -0.695 | -0.223 |
| sp\|O00222\|GRM8_HUMAN Metabotropic glutamate receptor 8 OS=Homo sapiens GN=GRM8 PE=2 SV=2 | 776 | TRGVPEtFNEA | 0.162 | 0.075 | -0.701 | -0.155 |
| sp\|O00222\|GRM8_HUMAN Metabotropic glutamate receptor 8 OS=Homo sapiens GN=GRM8 PE=2 SV=2 | 786 | AKPIGFtMYTT | 0.108 | -0.03 | -1.071 | -0.331 |
| sp\|O00222\|GRM8_HUMAN Metabotropic glutamate receptor 8 OS=Homo sapiens GN=GRM8 PE=2 SV=2 | 789 | IGFTMYtTCII | 0.285 | -0.102 | -0.474 | -0.097 |
| sp\|O00222\|GRM8_HUMAN Metabotropic glutamate receptor 8 OS=Homo sapiens GN=GRM8 PE=2 SV=2 | 790 | GFTMYTtCIIW | 0.336 | 0.103 | -0.327 | 0.037 |
| sp\|O00222\|GRM8_HUMAN Metabotropic glutamate receptor 8 OS=Homo sapiens GN=GRM8 PE=2 SV=2 | 804 | IPIFFGtAQSA | 0.11 | -0.153 | -1.3 | -0.448 |
| sp\|O00222\|GRM8_HUMAN Metabotropic glutamate receptor 8 OS=Homo sapiens GN=GRM8 PE=2 SV=2 | 807 | FFGTAQsAEKM | 0.212 | 0.104 | -0.713 | -0.132 |
| sp\|O00222\|GRM8_HUMAN Metabotropic glutamate receptor 8 OS=Homo sapiens GN=GRM8 PE=2 SV=2 | 815 | EKMYIQtTTLT | 0.237 | 0.051 | -0.335 | -0.016 |
| sp\|O00222\|GRM8_HUMAN Metabotropic glutamate receptor 8 OS=Homo sapiens GN=GRM8 PE=2 SV=2 | 816 | KMYIQTtTLTV | 0.051 | -0.044 | -1.641 | -0.545 |
| sp\|O00222\|GRM8_HUMAN Metabotropic glutamate receptor 8 OS=Homo sapiens GN=GRM8 PE=2 SV=2 | 817 | MYIQTTtLTVS | 0.459 | 0.244 | -0.185 | 0.173 |
| sp\|O00222\|GRM8_HUMAN Metabotropic glutamate receptor 8 OS=Homo sapiens GN=GRM8 PE=2 SV=2 | 819 | IQTTTLtVSMS | 0.052 | -0.225 | -1.301 | -0.491 |
| sp\|O00222\|GRM8_HUMAN Metabotropic glutamate receptor 8 OS=Homo sapiens GN=GRM8 PE=2 SV=2 | 821 | TTTLTVsMSLS | 0.12 | -0.012 | -1.163 | -0.352 |
| sp\|O00222\|GRM8_HUMAN Metabotropic glutamate receptor 8 OS=Homo sapiens GN=GRM8 PE=2 SV=2 | 823 | TLTVSMsLSAS | 0.557 | 0.448 | 0.286 | 0.43 |
| sp\|O00222\|GRM8_HUMAN Metabotropic glutamate receptor 8 OS=Homo sapiens GN=GRM8 PE=2 SV=2 | 825 | TVSMSLsASVS | 0.106 | 0.015 | -1.041 | -0.307 |
| sp\|O00222\|GRM8_HUMAN Metabotropic glutamate receptor 8 OS=Homo sapiens GN=GRM8 PE=2 SV=2 | 827 | SMSLSAsVSLG | 0.208 | 0.207 | -0.805 | -0.13 |
| sp\|O00222\|GRM8_HUMAN Metabotropic glutamate receptor 8 OS=Homo sapiens GN=GRM8 PE=2 SV=2 | 829 | SLSASVsLGML | 0.735 | 0.298 | 0.335 | 0.456 |
| sp\|O00222\|GRM8_HUMAN Metabotropic glutamate receptor 8 OS=Homo sapiens GN=GRM8 PE=2 SV=2 | 855 | VQKRKRsFKAV | 0.576 | 0.884 | 0.323 | 0.594 |
| sp\|O00222\|GRM8_HUMAN Metabotropic glutamate receptor 8 OS=Homo sapiens GN=GRM8 PE=2 SV=2 | 861 | SFKAVVtAATM | 0.29 | -0.119 | -0.722 | -0.184 |
| sp\|O00222\|GRM8_HUMAN Metabotropic glutamate receptor 8 OS=Homo sapiens GN=GRM8 PE=2 SV=2 | 864 | AVVTAAtMQSK | 0.177 | 0.041 | -0.998 | -0.26 |
| sp\|O00222\|GRM8_HUMAN Metabotropic glutamate receptor 8 OS=Homo sapiens GN=GRM8 PE=2 SV=2 | 867 | TAATMQsKLIQ | 0.114 | -0.272 | -1.103 | -0.42 |
| sp\|O00222\|GRM8_HUMAN Metabotropic glutamate receptor 8 OS=Homo sapiens GN=GRM8 PE=2 SV=2 | 883 | PNGEVKsELCE | 0.107 | -0.031 | -1.019 | -0.314 |
| sp\|O00222\|GRM8_HUMAN Metabotropic glutamate receptor 8 OS=Homo sapiens GN=GRM8 PE=2 SV=2 | 888 | KSELCEsLETN | 0.322 | -0.088 | -0.735 | -0.167 |
| sp\|O00222\|GRM8_HUMAN Metabotropic glutamate receptor 8 OS=Homo sapiens GN=GRM8 PE=2 SV=2 | 891 | LCESLEtNTSS | 0.065 | -0.3 | -1.795 | -0.677 |
| sp\|O00222\|GRM8_HUMAN Metabotropic glutamate receptor 8 OS=Homo sapiens GN=GRM8 PE=2 SV=2 | 893 | ESLETNtSSTK | 0.049 | -0.238 | -1.465 | -0.551 |
| sp\|O00222\|GRM8_HUMAN Metabotropic glutamate receptor 8 OS=Homo sapiens GN=GRM8 PE=2 SV=2 | 894 | SLETNTsSTKT | 0.161 | -0.169 | -0.994 | -0.334 |
| sp\|O00222\|GRM8_HUMAN Metabotropic glutamate receptor 8 OS=Homo sapiens GN=GRM8 PE=2 SV=2 | 895 | LETNTSsTKTT | 0.042 | -0.228 | -1.227 | -0.471 |

| | | | | | | |
|---|---|---|---|---|---|---|
| sp\|O00222\|GRM8_HUMAN Metabotropic glutamate receptor 8 OS=Homo sapiens GN=GRM8 PE=2 SV=2 | 896 | ETNTSStKTTY | 0.293 | 0.028 | -0.745 | -0.141 |
| sp\|O00222\|GRM8_HUMAN Metabotropic glutamate receptor 8 OS=Homo sapiens GN=GRM8 PE=2 SV=2 | 898 | NTSSTKtTYIS | 0.152 | -0.092 | -0.936 | -0.292 |
| sp\|O00222\|GRM8_HUMAN Metabotropic glutamate receptor 8 OS=Homo sapiens GN=GRM8 PE=2 SV=2 | 899 | TSSTKTtYISY | 0.051 | -0.297 | -1.407 | -0.551 |
| sp\|O00222\|GRM8_HUMAN Metabotropic glutamate receptor 8 OS=Homo sapiens GN=GRM8 PE=2 SV=2 | 902 | TKTTYIsYSNH | 0.146 | -0.182 | -1.191 | -0.409 |
| sp\|O00222\|GRM8_HUMAN Metabotropic glutamate receptor 8 OS=Homo sapiens GN=GRM8 PE=2 SV=2 | 904 | TTYISYsNHSI | 0.254 | 0.209 | -0.467 | -0.001 |
| sp\|O00222\|GRM8_HUMAN Metabotropic glutamate receptor 8 OS=Homo sapiens GN=GRM8 PE=2 SV=2 | 907 | ISYSNHsI--- | 0.217 | 0.069 | -0.611 | -0.108 |
| sp\|Q7RTX1\|TS1R1_HUMAN Taste receptor type 1 member 1 OS=Homo sapiens GN=TAS1R1 PE=2 SV=1 | 356 | RPCHKGsWCSS | 0.038 | -0.274 | -1.816 | -0.684 |
| sp\|Q7RTX1\|TS1R1_HUMAN Taste receptor type 1 member 1 OS=Homo sapiens GN=TAS1R1 PE=2 SV=1 | 359 | HKGSWCsSNQL | 0.363 | 0.09 | -0.393 | 0.02 |
| sp\|Q7RTX1\|TS1R1_HUMAN Taste receptor type 1 member 1 OS=Homo sapiens GN=TAS1R1 PE=2 SV=1 | 360 | KGSWCSsNQLC | 0.138 | -0.273 | -1.179 | -0.438 |
| sp\|Q7RTX1\|TS1R1_HUMAN Taste receptor type 1 member 1 OS=Homo sapiens GN=TAS1R1 PE=2 SV=1 | 374 | QAFMAHtMPKL | 0.666 | 0.696 | 0.147 | 0.503 |
| sp\|Q7RTX1\|TS1R1_HUMAN Taste receptor type 1 member 1 OS=Homo sapiens GN=TAS1R1 PE=2 SV=1 | 382 | PKLKAFsMSSA | 0.425 | 0.214 | -0.244 | 0.132 |
| sp\|Q7RTX1\|TS1R1_HUMAN Taste receptor type 1 member 1 OS=Homo sapiens GN=TAS1R1 PE=2 SV=1 | 384 | LKAFSMsSAYN | 0.513 | 0.305 | -0.037 | 0.26 |
| sp\|Q7RTX1\|TS1R1_HUMAN Taste receptor type 1 member 1 OS=Homo sapiens GN=TAS1R1 PE=2 SV=1 | 385 | KAFSMSsAYNA | 0.096 | -0.224 | -1.434 | -0.521 |
| sp\|Q7RTX1\|TS1R1_HUMAN Taste receptor type 1 member 1 OS=Homo sapiens GN=TAS1R1 PE=2 SV=1 | 408 | QLLGCAsGACS | 0.242 | 0.013 | -0.641 | -0.129 |
| sp\|Q7RTX1\|TS1R1_HUMAN Taste receptor type 1 member 1 OS=Homo sapiens GN=TAS1R1 PE=2 SV=1 | 412 | CASGACsRGRV | 0.367 | -0.099 | -0.944 | -0.225 |
| sp\|Q7RTX1\|TS1R1_HUMAN Taste receptor type 1 member 1 OS=Homo sapiens GN=TAS1R1 PE=2 SV=1 | 436 | FLLHKDtVAFN | 0.133 | -0.113 | -1.086 | -0.355 |
| sp\|Q7RTX1\|TS1R1_HUMAN Taste receptor type 1 member 1 OS=Homo sapiens GN=TAS1R1 PE=2 SV=1 | 447 | DNRDPLsSYNI | 0.237 | 0.062 | -0.83 | -0.177 |
| sp\|Q7RTX1\|TS1R1_HUMAN Taste receptor type 1 member 1 OS=Homo sapiens GN=TAS1R1 PE=2 SV=1 | 448 | NRDPLSsYNII | 0.143 | 0.022 | -0.835 | -0.223 |
| sp\|Q7RTX1\|TS1R1_HUMAN Taste receptor type 1 member 1 OS=Homo sapiens GN=TAS1R1 PE=2 SV=1 | 462 | WNGPKWtFTVL | 0.118 | -0.192 | -0.883 | -0.319 |
| sp\|Q7RTX1\|TS1R1_HUMAN Taste receptor type 1 member 1 OS=Homo sapiens GN=TAS1R1 PE=2 SV=1 | 464 | GPKWTFtVLGS | 0.17 | -0.032 | -0.708 | -0.19 |
| sp\|Q7RTX1\|TS1R1_HUMAN Taste receptor type 1 member 1 OS=Homo sapiens GN=TAS1R1 PE=2 SV=1 | 468 | TFTVLGsSTWS | 0.054 | -0.255 | -1.436 | -0.546 |
| sp\|Q7RTX1\|TS1R1_HUMAN Taste receptor type 1 member 1 OS=Homo sapiens GN=TAS1R1 PE=2 SV=1 | 469 | FTVLGSsTWSP | 0.045 | -0.201 | -1.665 | -0.607 |
| sp\|Q7RTX1\|TS1R1_HUMAN Taste receptor type 1 member 1 OS=Homo sapiens GN=TAS1R1 PE=2 SV=1 | 470 | TVLGSStWSPV | 0.087 | 0.045 | -1.029 | -0.299 |
| sp\|Q7RTX1\|TS1R1_HUMAN Taste receptor type 1 member 1 OS=Homo sapiens GN=TAS1R1 PE=2 SV=1 | 472 | LGSSTWsPVQL | 0.055 | -0.437 | -1.303 | -0.562 |
| sp\|Q7RTX1\|TS1R1_HUMAN Taste receptor type 1 member 1 OS=Homo sapiens GN=TAS1R1 PE=2 SV=1 | 481 | QLNINEtKIQW | 0.164 | -0.028 | -0.729 | -0.198 |
| sp\|Q7RTX1\|TS1R1_HUMAN Taste receptor type 1 member 1 OS=Homo sapiens GN=TAS1R1 PE=2 SV=1 | 495 | DNQVPKsVCSS | 0.059 | -0.279 | -1.279 | -0.5 |
| sp\|Q7RTX1\|TS1R1_HUMAN Taste receptor type 1 member 1 OS=Homo sapiens GN=TAS1R1 PE=2 SV=1 | 498 | VPKSVCsSDCL | 0.244 | -0.035 | -0.917 | -0.236 |
| sp\|Q7RTX1\|TS1R1_HUMAN Taste receptor type 1 member 1 OS=Homo sapiens GN=TAS1R1 PE=2 SV=1 | 499 | PKSVCSsDCLE | 0.216 | -0.114 | -0.952 | -0.283 |
| sp\|Q7RTX1\|TS1R1_HUMAN Taste receptor type 1 member 1 OS=Homo sapiens GN=TAS1R1 PE=2 SV=1 | 510 | GHQRVVtGFHH | 0.605 | 0.713 | -0.072 | 0.415 |
| sp\|Q7RTX1\|TS1R1_HUMAN Taste receptor type 1 member 1 OS=Homo sapiens GN=TAS1R1 PE=2 SV=1 | 526 | VPCGAGtFLNK | 0.161 | -0.078 | -1.094 | -0.337 |
| sp\|Q7RTX1\|TS1R1_HUMAN Taste receptor type 1 member 1 OS=Homo sapiens GN=TAS1R1 PE=2 SV=1 | 531 | GTFLNKsDLYR | 0.38 | 0.043 | -0.672 | -0.083 |
| sp\|Q7RTX1\|TS1R1_HUMAN Taste receptor type 1 member 1 OS=Homo sapiens GN=TAS1R1 PE=2 SV=1 | 549 | EWAPEGsQTCF | 0.065 | -0.239 | -1.551 | -0.575 |
| sp\|Q7RTX1\|TS1R1_HUMAN Taste receptor type 1 member 1 OS=Homo sapiens GN=TAS1R1 PE=2 SV=1 | 551 | APEGSQtCFPR | 0.296 | 0.144 | -0.326 | 0.038 |
| sp\|Q7RTX1\|TS1R1_HUMAN Taste receptor type 1 member 1 OS=Homo sapiens GN=TAS1R1 PE=2 SV=1 | 556 | QTCFPRtVVFL | 0.097 | -0.16 | -1.268 | -0.444 |
| sp\|Q7RTX1\|TS1R1_HUMAN Taste receptor type 1 member 1 OS=Homo sapiens GN=TAS1R1 PE=2 SV=1 | 566 | LALREHtSWVL | 0.678 | 0.956 | 0.271 | 0.635 |
| sp\|Q7RTX1\|TS1R1_HUMAN Taste receptor type 1 member 1 OS=Homo sapiens GN=TAS1R1 PE=2 SV=1 | 567 | ALREHTsWVLL | 0.445 | 0.447 | -0.251 | 0.214 |
| sp\|Q7RTX1\|TS1R1_HUMAN Taste receptor type 1 member 1 OS=Homo sapiens GN=TAS1R1 PE=2 SV=1 | 575 | VLLAANtLLLL | 0.589 | 0.246 | -0.008 | 0.276 |
| sp\|Q7RTX1\|TS1R1_HUMAN Taste receptor type 1 member 1 OS=Homo sapiens GN=TAS1R1 PE=2 SV=1 | 584 | LLLLLGtAGLF | 0.163 | 0.004 | -0.934 | -0.256 |
| sp\|Q7RTX1\|TS1R1_HUMAN Taste receptor type 1 member 1 OS=Homo sapiens GN=TAS1R1 PE=2 SV=1 | 594 | FAWHLDtPVVR | 0.047 | -0.298 | -1.635 | -0.629 |
| sp\|Q7RTX1\|TS1R1_HUMAN Taste receptor type 1 member 1 OS=Homo sapiens GN=TAS1R1 PE=2 SV=1 | 599 | DTPVVRsAGGR | 0.163 | -0.127 | -1.315 | -0.426 |
| sp\|Q7RTX1\|TS1R1_HUMAN Taste receptor type 1 member 1 OS=Homo sapiens GN=TAS1R1 PE=2 SV=1 | 611 | CFLMLGsLAAG | 0.301 | -0.028 | -0.333 | -0.02 |
| sp\|Q7RTX1\|TS1R1_HUMAN Taste receptor type 1 member 1 OS=Homo sapiens GN=TAS1R1 PE=2 SV=1 | 616 | GSLAAGsGSLY | 0.172 | -0.16 | -1.026 | -0.338 |
| sp\|Q7RTX1\|TS1R1_HUMAN Taste receptor type 1 member 1 OS=Homo sapiens GN=TAS1R1 PE=2 SV=1 | 618 | LAAGSGsLYGF | 0.252 | 0.081 | -0.174 | 0.053 |
| sp\|Q7RTX1\|TS1R1_HUMAN Taste receptor type 1 member 1 OS=Homo sapiens GN=TAS1R1 PE=2 SV=1 | 627 | GFFGEPtRPAC | 0.507 | 0.352 | -0.274 | 0.195 |
| sp\|Q7RTX1\|TS1R1_HUMAN Taste receptor type 1 member 1 OS=Homo sapiens GN=TAS1R1 PE=2 SV=1 | 643 | LFALGFtIFLS | 0.093 | -0.086 | -0.749 | -0.247 |
| sp\|Q7RTX1\|TS1R1_HUMAN Taste receptor type 1 member 1 OS=Homo sapiens GN=TAS1R1 PE=2 SV=1 | 647 | GFTIFLsCLTV | 0.151 | 0.013 | -1.184 | -0.34 |
| sp\|Q7RTX1\|TS1R1_HUMAN Taste receptor type 1 member 1 OS=Homo sapiens GN=TAS1R1 PE=2 SV=1 | 650 | IFLSCLtVRSF | 0.129 | -0.206 | -0.788 | -0.288 |
| sp\|Q7RTX1\|TS1R1_HUMAN Taste receptor type 1 member 1 OS=Homo sapiens GN=TAS1R1 PE=2 SV=1 | 653 | SCLTVRsFQLI | 0.308 | 0.071 | -0.757 | -0.126 |
| sp\|Q7RTX1\|TS1R1_HUMAN Taste receptor type 1 member 1 OS=Homo sapiens GN=TAS1R1 PE=2 SV=1 | 663 | IIIFKFsTKVP | 0.132 | 0.001 | -0.879 | -0.249 |

| | | | | | | |
|---|---|---|---|---|---|---|
| sp\|Q7RTX1\|TS1R1_HUMAN Taste receptor type 1 member 1 OS=Homo sapiens GN=TAS1R1 PE=2 SV=1 | 664 | IIFKFStKVPT | 0.215 | -0.094 | -0.764 | -0.214 |
| sp\|Q7RTX1\|TS1R1_HUMAN Taste receptor type 1 member 1 OS=Homo sapiens GN=TAS1R1 PE=2 SV=1 | 668 | FSTKVPtFYHA | 0.351 | -0.019 | -0.317 | 0.005 |
| sp\|Q7RTX1\|TS1R1_HUMAN Taste receptor type 1 member 1 OS=Homo sapiens GN=TAS1R1 PE=2 SV=1 | 686 | GLFVMIsSAAQ | 0.237 | -0.04 | -0.755 | -0.186 |
| sp\|Q7RTX1\|TS1R1_HUMAN Taste receptor type 1 member 1 OS=Homo sapiens GN=TAS1R1 PE=2 SV=1 | 687 | LFVMISsAAQL | 0.119 | -0.132 | -0.806 | -0.273 |
| sp\|Q7RTX1\|TS1R1_HUMAN Taste receptor type 1 member 1 OS=Homo sapiens GN=TAS1R1 PE=2 SV=1 | 696 | QLLICLtWLVV | 0.184 | 0.123 | -0.945 | -0.213 |
| sp\|Q7RTX1\|TS1R1_HUMAN Taste receptor type 1 member 1 OS=Homo sapiens GN=TAS1R1 PE=2 SV=1 | 702 | TWLVVWtPLPA | 0.044 | -0.507 | -1.641 | -0.701 |
| sp\|Q7RTX1\|TS1R1_HUMAN Taste receptor type 1 member 1 OS=Homo sapiens GN=TAS1R1 PE=2 SV=1 | 721 | LVMLECtETNS | 0.201 | 0.052 | -1.17 | -0.306 |
| sp\|Q7RTX1\|TS1R1_HUMAN Taste receptor type 1 member 1 OS=Homo sapiens GN=TAS1R1 PE=2 SV=1 | 723 | MLECTEtNSLG | 0.235 | 0.023 | -1.007 | -0.25 |
| sp\|Q7RTX1\|TS1R1_HUMAN Taste receptor type 1 member 1 OS=Homo sapiens GN=TAS1R1 PE=2 SV=1 | 725 | ECTETNsLGFI | 0.346 | 0.197 | -0.329 | 0.071 |
| sp\|Q7RTX1\|TS1R1_HUMAN Taste receptor type 1 member 1 OS=Homo sapiens GN=TAS1R1 PE=2 SV=1 | 739 | LYNGLLsISAF | 0.049 | -0.172 | -1.287 | -0.47 |
| sp\|Q7RTX1\|TS1R1_HUMAN Taste receptor type 1 member 1 OS=Homo sapiens GN=TAS1R1 PE=2 SV=1 | 741 | NGLLSIsAFAC | 0.419 | 0.212 | -0.244 | 0.129 |
| sp\|Q7RTX1\|TS1R1_HUMAN Taste receptor type 1 member 1 OS=Homo sapiens GN=TAS1R1 PE=2 SV=1 | 746 | ISAFACsYLGK | 0.275 | -0.155 | -0.696 | -0.192 |
| sp\|Q7RTX1\|TS1R1_HUMAN Taste receptor type 1 member 1 OS=Homo sapiens GN=TAS1R1 PE=2 SV=1 | 763 | NEAKCVtFSLL | 0.566 | 0.11 | -0.111 | 0.188 |
| sp\|Q7RTX1\|TS1R1_HUMAN Taste receptor type 1 member 1 OS=Homo sapiens GN=TAS1R1 PE=2 SV=1 | 765 | AKCVTFsLLFN | 0.206 | 0.074 | -0.758 | -0.159 |
| sp\|Q7RTX1\|TS1R1_HUMAN Taste receptor type 1 member 1 OS=Homo sapiens GN=TAS1R1 PE=2 SV=1 | 772 | LLFNFVsWIAF | 0.299 | 0.183 | -0.178 | 0.101 |
| sp\|Q7RTX1\|TS1R1_HUMAN Taste receptor type 1 member 1 OS=Homo sapiens GN=TAS1R1 PE=2 SV=1 | 778 | SWIAFFtTASV | 0.058 | -0.168 | -1.697 | -0.602 |
| sp\|Q7RTX1\|TS1R1_HUMAN Taste receptor type 1 member 1 OS=Homo sapiens GN=TAS1R1 PE=2 SV=1 | 779 | WIAFFTtASVY | 0.24 | 0.059 | -0.786 | -0.162 |
| sp\|Q7RTX1\|TS1R1_HUMAN Taste receptor type 1 member 1 OS=Homo sapiens GN=TAS1R1 PE=2 SV=1 | 781 | AFFTTAsVYDG | 0.338 | 0.107 | -0.535 | -0.03 |
| sp\|Q7RTX1\|TS1R1_HUMAN Taste receptor type 1 member 1 OS=Homo sapiens GN=TAS1R1 PE=2 SV=1 | 798 | NMMAGLsSLSS | 0.02 | -0.182 | -1.853 | -0.672 |
| sp\|Q7RTX1\|TS1R1_HUMAN Taste receptor type 1 member 1 OS=Homo sapiens GN=TAS1R1 PE=2 SV=1 | 799 | MMAGLSsLSSG | 0.077 | 0.023 | -0.997 | -0.299 |
| sp\|Q7RTX1\|TS1R1_HUMAN Taste receptor type 1 member 1 OS=Homo sapiens GN=TAS1R1 PE=2 SV=1 | 801 | AGLSSLsSGFG | 0.184 | -0.039 | -0.733 | -0.196 |
| sp\|Q7RTX1\|TS1R1_HUMAN Taste receptor type 1 member 1 OS=Homo sapiens GN=TAS1R1 PE=2 SV=1 | 802 | GLSSLSsGFGG | 0.15 | -0.184 | -0.955 | -0.33 |
| sp\|Q7RTX1\|TS1R1_HUMAN Taste receptor type 1 member 1 OS=Homo sapiens GN=TAS1R1 PE=2 SV=1 | 823 | CRPDLNsTEHF | 0.114 | 0.065 | -1.088 | -0.303 |
| sp\|Q7RTX1\|TS1R1_HUMAN Taste receptor type 1 member 1 OS=Homo sapiens GN=TAS1R1 PE=2 SV=1 | 824 | RPDLNStEHFQ | 0.105 | -0.167 | -1.323 | -0.462 |
| sp\|Q7RTX1\|TS1R1_HUMAN Taste receptor type 1 member 1 OS=Homo sapiens GN=TAS1R1 PE=2 SV=1 | 830 | TEHFQAsIQDY | 0.201 | -0.015 | -1.009 | -0.274 |
| sp\|Q7RTX1\|TS1R1_HUMAN Taste receptor type 1 member 1 OS=Homo sapiens GN=TAS1R1 PE=2 SV=1 | 835 | ASIQDYtRRCG | 0.161 | -0.158 | -0.915 | -0.304 |
| sp\|Q7RTX1\|TS1R1_HUMAN Taste receptor type 1 member 1 OS=Homo sapiens GN=TAS1R1 PE=2 SV=1 | 840 | YTRRCGsT--- | 0.812 | 1.159 | 0.552 | 0.841 |
| sp\|Q7RTX1\|TS1R1_HUMAN Taste receptor type 1 member 1 OS=Homo sapiens GN=TAS1R1 PE=2 SV=1 | 841 | TRRCGSt---- | 0.255 | 0.399 | -0.689 | -0.012 |
| sp\|Q8TE23\|TS1R2_HUMAN Taste receptor type 1 member 2 OS=Homo sapiens GN=TAS1R2 PE=3 SV=2 | 321 | ELRHLGtFLGI | 0.358 | 0.206 | -0.268 | 0.099 |
| sp\|Q8TE23\|TS1R2_HUMAN Taste receptor type 1 member 2 OS=Homo sapiens GN=TAS1R2 PE=3 SV=2 | 326 | GTFLGItIQSV | 0.116 | -0.149 | -1.35 | -0.461 |
| sp\|Q8TE23\|TS1R2_HUMAN Taste receptor type 1 member 2 OS=Homo sapiens GN=TAS1R2 PE=3 SV=2 | 329 | LGITIQsVPIP | 0.426 | 0.4 | 0.003 | 0.276 |
| sp\|Q8TE23\|TS1R2_HUMAN Taste receptor type 1 member 2 OS=Homo sapiens GN=TAS1R2 PE=3 SV=2 | 336 | VPIPGFsEFRE | 0.099 | -0.195 | -1.195 | -0.43 |
| sp\|Q8TE23\|TS1R2_HUMAN Taste receptor type 1 member 2 OS=Homo sapiens GN=TAS1R2 PE=3 SV=2 | 351 | AGPPPLsRTSQ | 0.031 | -0.536 | -1.868 | -0.791 |
| sp\|Q8TE23\|TS1R2_HUMAN Taste receptor type 1 member 2 OS=Homo sapiens GN=TAS1R2 PE=3 SV=2 | 353 | PPPLSRtSQSY | 0.137 | 0.053 | -1.094 | -0.301 |
| sp\|Q8TE23\|TS1R2_HUMAN Taste receptor type 1 member 2 OS=Homo sapiens GN=TAS1R2 PE=3 SV=2 | 354 | PPLSRTsQSYT | 0.127 | -0.166 | -1.216 | -0.418 |
| sp\|Q8TE23\|TS1R2_HUMAN Taste receptor type 1 member 2 OS=Homo sapiens GN=TAS1R2 PE=3 SV=2 | 356 | LSRTSQsYTCN | 0.507 | 0.361 | -0.136 | 0.244 |
| sp\|Q8TE23\|TS1R2_HUMAN Taste receptor type 1 member 2 OS=Homo sapiens GN=TAS1R2 PE=3 SV=2 | 358 | RTSQSYtCNQE | 0.58 | 0.306 | 0.173 | 0.353 |
| sp\|Q8TE23\|TS1R2_HUMAN Taste receptor type 1 member 2 OS=Homo sapiens GN=TAS1R2 PE=3 SV=2 | 370 | DNCLNAtLSFN | 0.26 | -0.113 | -0.877 | -0.243 |
| sp\|Q8TE23\|TS1R2_HUMAN Taste receptor type 1 member 2 OS=Homo sapiens GN=TAS1R2 PE=3 SV=2 | 372 | CLNATLsFNTI | 0.392 | 0.173 | -0.087 | 0.159 |
| sp\|Q8TE23\|TS1R2_HUMAN Taste receptor type 1 member 2 OS=Homo sapiens GN=TAS1R2 PE=3 SV=2 | 375 | ATLSFNtILRL | 0.132 | -0.234 | -1.395 | -0.499 |
| sp\|Q8TE23\|TS1R2_HUMAN Taste receptor type 1 member 2 OS=Homo sapiens GN=TAS1R2 PE=3 SV=2 | 380 | NTILRLsGERV | 0.163 | -0.062 | -1.331 | -0.41 |
| sp\|Q8TE23\|TS1R2_HUMAN Taste receptor type 1 member 2 OS=Homo sapiens GN=TAS1R2 PE=3 SV=2 | 387 | GERVVYsVYSA | 0.25 | 0.231 | -0.62 | -0.046 |
| sp\|Q8TE23\|TS1R2_HUMAN Taste receptor type 1 member 2 OS=Homo sapiens GN=TAS1R2 PE=3 SV=2 | 390 | VVYSVYsAVYA | 0.104 | -0.08 | -1.095 | -0.357 |
| sp\|Q8TE23\|TS1R2_HUMAN Taste receptor type 1 member 2 OS=Homo sapiens GN=TAS1R2 PE=3 SV=2 | 401 | VAHALHsLLGC | 0.131 | -0.163 | -1.187 | -0.406 |
| sp\|Q8TE23\|TS1R2_HUMAN Taste receptor type 1 member 2 OS=Homo sapiens GN=TAS1R2 PE=3 SV=2 | 408 | LLGCDKsTCTK | 0.209 | -0.141 | -0.751 | -0.228 |
| sp\|Q8TE23\|TS1R2_HUMAN Taste receptor type 1 member 2 OS=Homo sapiens GN=TAS1R2 PE=3 SV=2 | 409 | LGCDKStCTKR | 0.117 | -0.125 | -1.217 | -0.408 |
| sp\|Q8TE23\|TS1R2_HUMAN Taste receptor type 1 member 2 OS=Homo sapiens GN=TAS1R2 PE=3 SV=2 | 411 | CDKSTCtKRVV | 0.197 | -0.103 | -0.975 | -0.294 |
| sp\|Q8TE23\|TS1R2_HUMAN Taste receptor type 1 member 2 OS=Homo sapiens GN=TAS1R2 PE=3 SV=2 | 430 | IWKVNFtLLDH | 0.362 | 0.06 | -0.306 | 0.039 |
| sp\|Q8TE23\|TS1R2_HUMAN Taste receptor type 1 member 2 OS=Homo sapiens GN=TAS1R2 PE=3 SV=2 | 458 | QWQWDRsQNPF | 0.11 | -0.148 | -1.141 | -0.393 |

| | | | | | | |
|---|---|---|---|---|---|---|
| sp\|Q8TE23\|TS1R2_HUMAN Taste receptor type 1 member 2 OS=Homo sapiens GN=TAS1R2 PE=3 SV=2 | 464 | SQNPFQsVASY | 0.054 | -0.244 | -1.533 | -0.574 |
| sp\|Q8TE23\|TS1R2_HUMAN Taste receptor type 1 member 2 OS=Homo sapiens GN=TAS1R2 PE=3 SV=2 | 467 | PFQSVAsYYPL | 0.215 | -0.014 | -0.715 | -0.171 |
| sp\|Q8TE23\|TS1R2_HUMAN Taste receptor type 1 member 2 OS=Homo sapiens GN=TAS1R2 PE=3 SV=2 | 482 | KNIQDIsWHTI | 0.25 | -0.033 | -0.584 | -0.122 |
| sp\|Q8TE23\|TS1R2_HUMAN Taste receptor type 1 member 2 OS=Homo sapiens GN=TAS1R2 PE=3 SV=2 | 485 | QDISWHtINNT | 0.123 | -0.034 | -0.938 | -0.283 |
| sp\|Q8TE23\|TS1R2_HUMAN Taste receptor type 1 member 2 OS=Homo sapiens GN=TAS1R2 PE=3 SV=2 | 489 | WHTINNtIPMS | 0.419 | 0.359 | -0.351 | 0.142 |
| sp\|Q8TE23\|TS1R2_HUMAN Taste receptor type 1 member 2 OS=Homo sapiens GN=TAS1R2 PE=3 SV=2 | 493 | NNTIPMsMCSK | 0.066 | -0.132 | -1.12 | -0.395 |
| sp\|Q8TE23\|TS1R2_HUMAN Taste receptor type 1 member 2 OS=Homo sapiens GN=TAS1R2 PE=3 SV=2 | 496 | IPMSMCsKRCQ | 0.211 | -0.209 | -0.987 | -0.328 |
| sp\|Q8TE23\|TS1R2_HUMAN Taste receptor type 1 member 2 OS=Homo sapiens GN=TAS1R2 PE=3 SV=2 | 501 | CSKRCQsGQKK | 0.743 | 0.783 | 0.669 | 0.732 |
| sp\|Q8TE23\|TS1R2_HUMAN Taste receptor type 1 member 2 OS=Homo sapiens GN=TAS1R2 PE=3 SV=2 | 524 | IDCLPGtFLNH | 0.066 | -0.327 | -1.611 | -0.624 |
| sp\|Q8TE23\|TS1R2_HUMAN Taste receptor type 1 member 2 OS=Homo sapiens GN=TAS1R2 PE=3 SV=2 | 529 | GTFLNHtEDEY | 0.684 | 0.308 | -0.179 | 0.271 |
| sp\|Q8TE23\|TS1R2_HUMAN Taste receptor type 1 member 2 OS=Homo sapiens GN=TAS1R2 PE=3 SV=2 | 544 | CPNNEWsYQSE | 0.078 | -0.265 | -1.318 | -0.502 |
| sp\|Q8TE23\|TS1R2_HUMAN Taste receptor type 1 member 2 OS=Homo sapiens GN=TAS1R2 PE=3 SV=2 | 547 | NEWSYQsETSC | 0.099 | -0.034 | -1.299 | -0.411 |
| sp\|Q8TE23\|TS1R2_HUMAN Taste receptor type 1 member 2 OS=Homo sapiens GN=TAS1R2 PE=3 SV=2 | 549 | WSYQSEtSCFK | 0.115 | -0.217 | -0.97 | -0.357 |
| sp\|Q8TE23\|TS1R2_HUMAN Taste receptor type 1 member 2 OS=Homo sapiens GN=TAS1R2 PE=3 SV=2 | 550 | SYQSETsCFKR | 0.163 | -0.021 | -1.076 | -0.311 |
| sp\|Q8TE23\|TS1R2_HUMAN Taste receptor type 1 member 2 OS=Homo sapiens GN=TAS1R2 PE=3 SV=2 | 566 | EWHEAPtIAVA | 0.06 | -0.162 | -1.242 | -0.448 |
| sp\|Q8TE23\|TS1R2_HUMAN Taste receptor type 1 member 2 OS=Homo sapiens GN=TAS1R2 PE=3 SV=2 | 579 | AALGFLsTLAI | 0.115 | -0.113 | -1.006 | -0.335 |
| sp\|Q8TE23\|TS1R2_HUMAN Taste receptor type 1 member 2 OS=Homo sapiens GN=TAS1R2 PE=3 SV=2 | 580 | ALGFLStLAIL | 0.218 | -0.009 | -0.778 | -0.19 |
| sp\|Q8TE23\|TS1R2_HUMAN Taste receptor type 1 member 2 OS=Homo sapiens GN=TAS1R2 PE=3 SV=2 | 593 | FWRHFQtPIVR | 0.21 | 0.047 | -0.648 | -0.13 |
| sp\|Q8TE23\|TS1R2_HUMAN Taste receptor type 1 member 2 OS=Homo sapiens GN=TAS1R2 PE=3 SV=2 | 598 | QTPIVRsAGGP | 0.097 | -0.167 | -1.413 | -0.494 |
| sp\|Q8TE23\|TS1R2_HUMAN Taste receptor type 1 member 2 OS=Homo sapiens GN=TAS1R2 PE=3 SV=2 | 609 | MCFLMLtLLLV | 0.314 | 0.099 | -0.833 | -0.14 |
| sp\|Q8TE23\|TS1R2_HUMAN Taste receptor type 1 member 2 OS=Homo sapiens GN=TAS1R2 PE=3 SV=2 | 628 | VGPPKVsTCLC | 0.061 | -0.425 | -1.571 | -0.645 |
| sp\|Q8TE23\|TS1R2_HUMAN Taste receptor type 1 member 2 OS=Homo sapiens GN=TAS1R2 PE=3 SV=2 | 629 | GPPKVStCLCR | 0.16 | -0.007 | -1.172 | -0.34 |
| sp\|Q8TE23\|TS1R2_HUMAN Taste receptor type 1 member 2 OS=Homo sapiens GN=TAS1R2 PE=3 SV=2 | 642 | LFPLCFtICIS | 0.132 | -0.176 | -0.954 | -0.333 |
| sp\|Q8TE23\|TS1R2_HUMAN Taste receptor type 1 member 2 OS=Homo sapiens GN=TAS1R2 PE=3 SV=2 | 646 | CFTICIsCIAV | 0.481 | 0.228 | -0.091 | 0.206 |
| sp\|Q8TE23\|TS1R2_HUMAN Taste receptor type 1 member 2 OS=Homo sapiens GN=TAS1R2 PE=3 SV=2 | 652 | SCIAVRsFQIV | 0.257 | 0.009 | -1.212 | -0.315 |
| sp\|Q8TE23\|TS1R2_HUMAN Taste receptor type 1 member 2 OS=Homo sapiens GN=TAS1R2 PE=3 SV=2 | 663 | CAFKMAsRFPR | 0.373 | -0.116 | -0.49 | -0.078 |
| sp\|Q8TE23\|TS1R2_HUMAN Taste receptor type 1 member 2 OS=Homo sapiens GN=TAS1R2 PE=3 SV=2 | 670 | RFPRAYsYWVR | 0.752 | 1.049 | 0.712 | 0.838 |
| sp\|Q8TE23\|TS1R2_HUMAN Taste receptor type 1 member 2 OS=Homo sapiens GN=TAS1R2 PE=3 SV=2 | 681 | YQGPYVsMAFI | 0.138 | -0.068 | -0.781 | -0.237 |
| sp\|Q8TE23\|TS1R2_HUMAN Taste receptor type 1 member 2 OS=Homo sapiens GN=TAS1R2 PE=3 SV=2 | 686 | VSMAFItVLKM | 0.108 | -0.209 | -1.171 | -0.424 |
| sp\|Q8TE23\|TS1R2_HUMAN Taste receptor type 1 member 2 OS=Homo sapiens GN=TAS1R2 PE=3 SV=2 | 700 | VIGMLAtGLSP | 0.048 | -0.271 | -1.645 | -0.623 |
| sp\|Q8TE23\|TS1R2_HUMAN Taste receptor type 1 member 2 OS=Homo sapiens GN=TAS1R2 PE=3 SV=2 | 703 | MLATGLsPTTR | 0.111 | -0.313 | -1.094 | -0.432 |
| sp\|Q8TE23\|TS1R2_HUMAN Taste receptor type 1 member 2 OS=Homo sapiens GN=TAS1R2 PE=3 SV=2 | 705 | ATGLSPtTRTD | 0.291 | -0.041 | -0.68 | -0.143 |
| sp\|Q8TE23\|TS1R2_HUMAN Taste receptor type 1 member 2 OS=Homo sapiens GN=TAS1R2 PE=3 SV=2 | 706 | TGLSPTtRTDP | 0.118 | -0.335 | -1.042 | -0.42 |
| sp\|Q8TE23\|TS1R2_HUMAN Taste receptor type 1 member 2 OS=Homo sapiens GN=TAS1R2 PE=3 SV=2 | 708 | LSPTTRtDPDD | 0.452 | 0.449 | -0.298 | 0.201 |
| sp\|Q8TE23\|TS1R2_HUMAN Taste receptor type 1 member 2 OS=Homo sapiens GN=TAS1R2 PE=3 SV=2 | 716 | PDDPKItIVSC | 0.029 | -0.419 | -1.784 | -0.725 |
| sp\|Q8TE23\|TS1R2_HUMAN Taste receptor type 1 member 2 OS=Homo sapiens GN=TAS1R2 PE=3 SV=2 | 719 | PKITIVsCNPN | 0.528 | 0.151 | -0.144 | 0.178 |
| sp\|Q8TE23\|TS1R2_HUMAN Taste receptor type 1 member 2 OS=Homo sapiens GN=TAS1R2 PE=3 SV=2 | 727 | NPNYRNsLLFN | 0.378 | 0.164 | -0.552 | -0.003 |
| sp\|Q8TE23\|TS1R2_HUMAN Taste receptor type 1 member 2 OS=Homo sapiens GN=TAS1R2 PE=3 SV=2 | 732 | NSLLFNtSLDL | 0.211 | -0.033 | -0.973 | -0.265 |
| sp\|Q8TE23\|TS1R2_HUMAN Taste receptor type 1 member 2 OS=Homo sapiens GN=TAS1R2 PE=3 SV=2 | 733 | SLLFNTsLDLL | 0.544 | 0.226 | 0.084 | 0.285 |
| sp\|Q8TE23\|TS1R2_HUMAN Taste receptor type 1 member 2 OS=Homo sapiens GN=TAS1R2 PE=3 SV=2 | 739 | SLDLLLsVVGF | 0.063 | -0.178 | -1.399 | -0.505 |
| sp\|Q8TE23\|TS1R2_HUMAN Taste receptor type 1 member 2 OS=Homo sapiens GN=TAS1R2 PE=3 SV=2 | 744 | LSVVGFsFAYM | 0.07 | -0.193 | -1.131 | -0.418 |
| sp\|Q8TE23\|TS1R2_HUMAN Taste receptor type 1 member 2 OS=Homo sapiens GN=TAS1R2 PE=3 SV=2 | 754 | MGKELPtNYNE | 0.115 | -0.098 | -1.269 | -0.417 |
| sp\|Q8TE23\|TS1R2_HUMAN Taste receptor type 1 member 2 OS=Homo sapiens GN=TAS1R2 PE=3 SV=2 | 763 | NEAKFItLSMT | 0.296 | -0.102 | -0.643 | -0.15 |
| sp\|Q8TE23\|TS1R2_HUMAN Taste receptor type 1 member 2 OS=Homo sapiens GN=TAS1R2 PE=3 SV=2 | 765 | AKFITLsMTFY | 0.162 | 0.075 | -1.089 | -0.284 |
| sp\|Q8TE23\|TS1R2_HUMAN Taste receptor type 1 member 2 OS=Homo sapiens GN=TAS1R2 PE=3 SV=2 | 767 | FITLSMtFYFT | 0.5 | 0.447 | 0.108 | 0.352 |
| sp\|Q8TE23\|TS1R2_HUMAN Taste receptor type 1 member 2 OS=Homo sapiens GN=TAS1R2 PE=3 SV=2 | 771 | SMTFYFtSSVS | 0.069 | -0.001 | -1.302 | -0.411 |
| sp\|Q8TE23\|TS1R2_HUMAN Taste receptor type 1 member 2 OS=Homo sapiens GN=TAS1R2 PE=3 SV=2 | 772 | MTFYFTsSVSL | 0.146 | 0.013 | -0.95 | -0.264 |
| sp\|Q8TE23\|TS1R2_HUMAN Taste receptor type 1 member 2 OS=Homo sapiens GN=TAS1R2 PE=3 SV=2 | 773 | TFYFTSsVSLC | 0.058 | -0.165 | -1.395 | -0.501 |
| sp\|Q8TE23\|TS1R2_HUMAN Taste receptor type 1 member 2 OS=Homo sapiens GN=TAS1R2 PE=3 SV=2 | 775 | YFTSSVsLCTF | 0.522 | 0.131 | 0.17 | 0.274 |

| | | | | | | |
|---|---|---|---|---|---|---|
| sp\|Q8TE23\|TS1R2_HUMAN Taste receptor type 1 member 2 OS=Homo sapiens GN=TAS1R2 PE=3 SV=2 | 778 | SSVSLCtFMSA | 0.088 | -0.243 | -1.477 | -0.544 |
| sp\|Q8TE23\|TS1R2_HUMAN Taste receptor type 1 member 2 OS=Homo sapiens GN=TAS1R2 PE=3 SV=2 | 781 | SLCTFMsAYSG | 0.223 | 0.109 | -1.035 | -0.234 |
| sp\|Q8TE23\|TS1R2_HUMAN Taste receptor type 1 member 2 OS=Homo sapiens GN=TAS1R2 PE=3 SV=2 | 784 | TFMSAYsGVLV | 0.177 | 0.06 | -0.723 | -0.162 |
| sp\|Q8TE23\|TS1R2_HUMAN Taste receptor type 1 member 2 OS=Homo sapiens GN=TAS1R2 PE=3 SV=2 | 789 | YSGVLVtIVDL | 0.115 | -0.121 | -0.97 | -0.325 |
| sp\|Q8TE23\|TS1R2_HUMAN Taste receptor type 1 member 2 OS=Homo sapiens GN=TAS1R2 PE=3 SV=2 | 796 | IVDLLVtVLNL | 0.163 | -0.04 | -1.074 | -0.317 |
| sp\|Q8TE23\|TS1R2_HUMAN Taste receptor type 1 member 2 OS=Homo sapiens GN=TAS1R2 PE=3 SV=2 | 804 | LNLLAIsLGYF | 0.385 | 0.029 | -0.215 | 0.066 |
| sp\|Q8TE23\|TS1R2_HUMAN Taste receptor type 1 member 2 OS=Homo sapiens GN=TAS1R2 PE=3 SV=2 | 823 | FYPERNtPAYF | 0.108 | -0.196 | -1.378 | -0.489 |
| sp\|Q8TE23\|TS1R2_HUMAN Taste receptor type 1 member 2 OS=Homo sapiens GN=TAS1R2 PE=3 SV=2 | 829 | TPAYFNsMIQG | 0.21 | 0.042 | -0.71 | -0.153 |
| sp\|Q8TE23\|TS1R2_HUMAN Taste receptor type 1 member 2 OS=Homo sapiens GN=TAS1R2 PE=3 SV=2 | 835 | SMIQGYtMRRD | 0.109 | 0.046 | -1.053 | -0.299 |
| sp\|Q7RTX0\|TS1R3_HUMAN Taste receptor type 1 member 3 OS=Homo sapiens GN=TAS1R3 PE=1 SV=2 | 320 | GMAQMGtVLGF | 0.117 | -0.104 | -0.905 | -0.297 |
| sp\|Q7RTX0\|TS1R3_HUMAN Taste receptor type 1 member 3 OS=Homo sapiens GN=TAS1R3 PE=1 SV=2 | 340 | FPQYVKtHLAL | 0.413 | 0.249 | -0.176 | 0.162 |
| sp\|Q7RTX0\|TS1R3_HUMAN Taste receptor type 1 member 3 OS=Homo sapiens GN=TAS1R3 PE=1 SV=2 | 346 | THLALAtDPAF | 0.502 | 0.457 | -0.321 | 0.213 |
| sp\|Q7RTX0\|TS1R3_HUMAN Taste receptor type 1 member 3 OS=Homo sapiens GN=TAS1R3 PE=1 SV=2 | 352 | TDPAFCsALGE | 0.076 | -0.332 | -1.696 | -0.651 |
| sp\|Q7RTX0\|TS1R3_HUMAN Taste receptor type 1 member 3 OS=Homo sapiens GN=TAS1R3 PE=1 SV=2 | 377 | PQCDCItLQNV | 0.563 | 0.169 | -0.353 | 0.126 |
| sp\|Q7RTX0\|TS1R3_HUMAN Taste receptor type 1 member 3 OS=Homo sapiens GN=TAS1R3 PE=1 SV=2 | 382 | ITLQNVsAGLN | 0.628 | 0.076 | -0.097 | 0.202 |
| sp\|Q7RTX0\|TS1R3_HUMAN Taste receptor type 1 member 3 OS=Homo sapiens GN=TAS1R3 PE=1 SV=2 | 390 | GLNHHQtFSVY | 0.478 | 0.211 | -0.402 | 0.096 |
| sp\|Q7RTX0\|TS1R3_HUMAN Taste receptor type 1 member 3 OS=Homo sapiens GN=TAS1R3 PE=1 SV=2 | 392 | NHHQTFsVYAA | 0.16 | 0.067 | -0.779 | -0.184 |
| sp\|Q7RTX0\|TS1R3_HUMAN Taste receptor type 1 member 3 OS=Homo sapiens GN=TAS1R3 PE=1 SV=2 | 399 | VYAAVYsVAQA | 0.242 | -0.074 | -0.479 | -0.104 |
| sp\|Q7RTX0\|TS1R3_HUMAN Taste receptor type 1 member 3 OS=Homo sapiens GN=TAS1R3 PE=1 SV=2 | 407 | AQALHNtLQCN | 0.475 | 0.192 | -0.26 | 0.136 |
| sp\|Q7RTX0\|TS1R3_HUMAN Taste receptor type 1 member 3 OS=Homo sapiens GN=TAS1R3 PE=1 SV=2 | 413 | TLQCNAsGCPA | 0.278 | -0.114 | -0.681 | -0.172 |
| sp\|Q7RTX0\|TS1R3_HUMAN Taste receptor type 1 member 3 OS=Homo sapiens GN=TAS1R3 PE=1 SV=2 | 434 | ENMYNLtFHVG | 0.172 | 0.023 | -0.524 | -0.11 |
| sp\|Q7RTX0\|TS1R3_HUMAN Taste receptor type 1 member 3 OS=Homo sapiens GN=TAS1R3 PE=1 SV=2 | 446 | LPLRFDsSGNV | 0.549 | 0.741 | -0.337 | 0.318 |
| sp\|Q7RTX0\|TS1R3_HUMAN Taste receptor type 1 member 3 OS=Homo sapiens GN=TAS1R3 PE=1 SV=2 | 447 | PLRFDSsGNVD | 0.515 | 0.285 | -0.047 | 0.251 |
| sp\|Q7RTX0\|TS1R3_HUMAN Taste receptor type 1 member 3 OS=Homo sapiens GN=TAS1R3 PE=1 SV=2 | 464 | LWVWQGsVPRL | 0.365 | 0.353 | -0.406 | 0.104 |
| sp\|Q7RTX0\|TS1R3_HUMAN Taste receptor type 1 member 3 OS=Homo sapiens GN=TAS1R3 PE=1 SV=2 | 477 | VGRFNGsLRTE | 0.495 | 0.076 | -0.153 | 0.139 |
| sp\|Q7RTX0\|TS1R3_HUMAN Taste receptor type 1 member 3 OS=Homo sapiens GN=TAS1R3 PE=1 SV=2 | 480 | FNGSLRtERLK | 0.072 | -0.202 | -1.363 | -0.498 |
| sp\|Q7RTX0\|TS1R3_HUMAN Taste receptor type 1 member 3 OS=Homo sapiens GN=TAS1R3 PE=1 SV=2 | 489 | LKIRWHtSDNQ | 0.805 | 1.107 | 0.617 | 0.843 |
| sp\|Q7RTX0\|TS1R3_HUMAN Taste receptor type 1 member 3 OS=Homo sapiens GN=TAS1R3 PE=1 SV=2 | 490 | KIRWHTsDNQK | 0.671 | 0.282 | 0.104 | 0.352 |
| sp\|Q7RTX0\|TS1R3_HUMAN Taste receptor type 1 member 3 OS=Homo sapiens GN=TAS1R3 PE=1 SV=2 | 497 | DNQKPVsRCSR | 0.188 | -0.314 | -1.023 | -0.383 |
| sp\|Q7RTX0\|TS1R3_HUMAN Taste receptor type 1 member 3 OS=Homo sapiens GN=TAS1R3 PE=1 SV=2 | 500 | KPVSRCsRQCQ | 0.215 | -0.076 | -1.216 | -0.359 |
| sp\|Q7RTX0\|TS1R3_HUMAN Taste receptor type 1 member 3 OS=Homo sapiens GN=TAS1R3 PE=1 SV=2 | 516 | RVKGFHsCCYD | 0.297 | 0.128 | -0.679 | -0.085 |
| sp\|Q7RTX0\|TS1R3_HUMAN Taste receptor type 1 member 3 OS=Homo sapiens GN=TAS1R3 PE=1 SV=2 | 528 | VDCEAGsYRQN | 0.075 | -0.301 | -1.386 | -0.537 |
| sp\|Q7RTX0\|TS1R3_HUMAN Taste receptor type 1 member 3 OS=Homo sapiens GN=TAS1R3 PE=1 SV=2 | 539 | PDDIACtFCGQ | 0.216 | -0.145 | -0.964 | -0.298 |
| sp\|Q7RTX0\|TS1R3_HUMAN Taste receptor type 1 member 3 OS=Homo sapiens GN=TAS1R3 PE=1 SV=2 | 547 | CGQDEWsPERS | 0.092 | -0.436 | -1.533 | -0.626 |
| sp\|Q7RTX0\|TS1R3_HUMAN Taste receptor type 1 member 3 OS=Homo sapiens GN=TAS1R3 PE=1 SV=2 | 551 | EWSPERsTRCF | 0.06 | -0.212 | -1.469 | -0.54 |
| sp\|Q7RTX0\|TS1R3_HUMAN Taste receptor type 1 member 3 OS=Homo sapiens GN=TAS1R3 PE=1 SV=2 | 552 | WSPERStRCFR | 0.048 | -0.38 | -1.636 | -0.656 |
| sp\|Q7RTX0\|TS1R3_HUMAN Taste receptor type 1 member 3 OS=Homo sapiens GN=TAS1R3 PE=1 SV=2 | 559 | RCFRRRsRFLA | 0.78 | 1.009 | 0.343 | 0.711 |
| sp\|Q7RTX0\|TS1R3_HUMAN Taste receptor type 1 member 3 OS=Homo sapiens GN=TAS1R3 PE=1 SV=2 | 577 | LLLLLLsLALG | 0.215 | 0.022 | -0.626 | -0.13 |
| sp\|Q7RTX0\|TS1R3_HUMAN Taste receptor type 1 member 3 OS=Homo sapiens GN=TAS1R3 PE=1 SV=2 | 596 | FVHHRDsPLVQ | 0.058 | -0.286 | -1.608 | -0.612 |
| sp\|Q7RTX0\|TS1R3_HUMAN Taste receptor type 1 member 3 OS=Homo sapiens GN=TAS1R3 PE=1 SV=2 | 602 | SPLVQAsGGPL | 0.076 | -0.165 | -1.324 | -0.471 |
| sp\|Q7RTX0\|TS1R3_HUMAN Taste receptor type 1 member 3 OS=Homo sapiens GN=TAS1R3 PE=1 SV=2 | 620 | LGLVCLsVLLF | 0.071 | -0.131 | -1.045 | -0.368 |
| sp\|Q7RTX0\|TS1R3_HUMAN Taste receptor type 1 member 3 OS=Homo sapiens GN=TAS1R3 PE=1 SV=2 | 629 | LFPGQPsPARC | 0.025 | -0.659 | -2.072 | -0.902 |
| sp\|Q7RTX0\|TS1R3_HUMAN Taste receptor type 1 member 3 OS=Homo sapiens GN=TAS1R3 PE=1 SV=2 | 640 | LAQQPLsHLPL | 0.084 | -0.195 | -0.996 | -0.369 |
| sp\|Q7RTX0\|TS1R3_HUMAN Taste receptor type 1 member 3 OS=Homo sapiens GN=TAS1R3 PE=1 SV=2 | 645 | LSHLPLtGCLS | 0.028 | -0.438 | -1.724 | -0.711 |
| sp\|Q7RTX0\|TS1R3_HUMAN Taste receptor type 1 member 3 OS=Homo sapiens GN=TAS1R3 PE=1 SV=2 | 649 | PLTGCLsTLFL | 0.157 | -0.007 | -0.771 | -0.207 |
| sp\|Q7RTX0\|TS1R3_HUMAN Taste receptor type 1 member 3 OS=Homo sapiens GN=TAS1R3 PE=1 SV=2 | 650 | LTGCLStLFLQ | 0.147 | -0.137 | -1.042 | -0.344 |
| sp\|Q7RTX0\|TS1R3_HUMAN Taste receptor type 1 member 3 OS=Homo sapiens GN=TAS1R3 PE=1 SV=2 | 662 | AEIFVEsELPL | 0.125 | -0.071 | -1.348 | -0.431 |
| sp\|Q7RTX0\|TS1R3_HUMAN Taste receptor type 1 member 3 OS=Homo sapiens GN=TAS1R3 PE=1 SV=2 | 667 | ESELPLsWADR | 0.087 | -0.161 | -0.987 | -0.354 |
| sp\|Q7RTX0\|TS1R3_HUMAN Taste receptor type 1 member 3 OS=Homo sapiens GN=TAS1R3 PE=1 SV=2 | 673 | SWADRLsGCLR | 0.114 | -0.067 | -1.094 | -0.349 |

| | | | | | | |
|---|---|---|---|---|---|---|
| sp\|Q7RTX0\|TS1R3_HUMAN Taste receptor type 1 member 3 OS=Homo sapiens GN=TAS1R3 PE=1 SV=2 | 697 | VEVALCtWYLV | 0.131 | -0.054 | -1.479 | -0.467 |
| sp\|Q7RTX0\|TS1R3_HUMAN Taste receptor type 1 member 3 OS=Homo sapiens GN=TAS1R3 PE=1 SV=2 | 709 | FPPEVVtDWHM | 0.199 | -0.066 | -0.998 | -0.288 |
| sp\|Q7RTX0\|TS1R3_HUMAN Taste receptor type 1 member 3 OS=Homo sapiens GN=TAS1R3 PE=1 SV=2 | 716 | DWHMLPtEALV | 0.105 | -0.168 | -1.5 | -0.521 |
| sp\|Q7RTX0\|TS1R3_HUMAN Taste receptor type 1 member 3 OS=Homo sapiens GN=TAS1R3 PE=1 SV=2 | 724 | ALVHCRtRSWV | 0.164 | -0.119 | -1.29 | -0.415 |
| sp\|Q7RTX0\|TS1R3_HUMAN Taste receptor type 1 member 3 OS=Homo sapiens GN=TAS1R3 PE=1 SV=2 | 726 | VHCRTRsWVSF | 0.181 | 0.828 | -0.689 | 0.107 |
| sp\|Q7RTX0\|TS1R3_HUMAN Taste receptor type 1 member 3 OS=Homo sapiens GN=TAS1R3 PE=1 SV=2 | 729 | RTRSWVsFGLA | 0.794 | 0.346 | 0.288 | 0.476 |
| sp\|Q7RTX0\|TS1R3_HUMAN Taste receptor type 1 member 3 OS=Homo sapiens GN=TAS1R3 PE=1 SV=2 | 736 | FGLAHAtNATL | 0.265 | -0.004 | -0.737 | -0.159 |
| sp\|Q7RTX0\|TS1R3_HUMAN Taste receptor type 1 member 3 OS=Homo sapiens GN=TAS1R3 PE=1 SV=2 | 739 | AHATNAtLAFL | 0.237 | -0.065 | -0.836 | -0.221 |
| sp\|Q7RTX0\|TS1R3_HUMAN Taste receptor type 1 member 3 OS=Homo sapiens GN=TAS1R3 PE=1 SV=2 | 748 | FLCFLGtFLVR | 0.279 | 0.134 | -0.737 | -0.108 |
| sp\|Q7RTX0\|TS1R3_HUMAN Taste receptor type 1 member 3 OS=Homo sapiens GN=TAS1R3 PE=1 SV=2 | 753 | GTFLVRsQPGC | 0.461 | 0.304 | -0.83 | -0.022 |
| sp\|Q7RTX0\|TS1R3_HUMAN Taste receptor type 1 member 3 OS=Homo sapiens GN=TAS1R3 PE=1 SV=2 | 765 | NRARGLtFAML | 0.457 | 0.957 | 0.265 | 0.56 |
| sp\|Q7RTX0\|TS1R3_HUMAN Taste receptor type 1 member 3 OS=Homo sapiens GN=TAS1R3 PE=1 SV=2 | 774 | MLAYFItWVSF | 0.222 | 0.171 | -0.493 | -0.033 |
| sp\|Q7RTX0\|TS1R3_HUMAN Taste receptor type 1 member 3 OS=Homo sapiens GN=TAS1R3 PE=1 SV=2 | 777 | YFITWVsFVPL | 0.398 | 0.114 | -0.215 | 0.099 |
| sp\|Q7RTX0\|TS1R3_HUMAN Taste receptor type 1 member 3 OS=Homo sapiens GN=TAS1R3 PE=1 SV=2 | 825 | RQPGLNtPEFF | 0.032 | -0.475 | -1.804 | -0.749 |
| sp\|Q7RTX0\|TS1R3_HUMAN Taste receptor type 1 member 3 OS=Homo sapiens GN=TAS1R3 PE=1 SV=2 | 845 | GQNDGNtGNQG | 0.194 | -0.078 | -0.846 | -0.243 |
| sp\|Q8NFN8\|GP156_HUMAN Probable G-protein coupled receptor 156 OS=Homo sapiens GN=GPR156 PE=2 S | 324 | FEEENQtIRRM | 0.132 | -0.199 | -0.989 | -0.352 |
| sp\|Q8NFN8\|GP156_HUMAN Probable G-protein coupled receptor 156 OS=Homo sapiens GN=GPR156 PE=2 S | 333 | RMAKYFsTPNK | 0.735 | 0.699 | 0.286 | 0.573 |
| sp\|Q8NFN8\|GP156_HUMAN Probable G-protein coupled receptor 156 OS=Homo sapiens GN=GPR156 PE=2 S | 334 | MAKYFStPNKS | 0.081 | -0.334 | -1.215 | -0.489 |
| sp\|Q8NFN8\|GP156_HUMAN Probable G-protein coupled receptor 156 OS=Homo sapiens GN=GPR156 PE=2 S | 338 | FSTPNKsFHTQ | 0.401 | 0.022 | -0.186 | 0.079 |
| sp\|Q8NFN8\|GP156_HUMAN Probable G-protein coupled receptor 156 OS=Homo sapiens GN=GPR156 PE=2 S | 341 | PNKSFHtQYGE | 0.219 | -0.139 | -0.954 | -0.291 |
| sp\|Q8NFN8\|GP156_HUMAN Probable G-protein coupled receptor 156 OS=Homo sapiens GN=GPR156 PE=2 S | 356 | HPRGEKsSMER | 0.421 | 0.246 | -0.268 | 0.133 |
| sp\|Q8NFN8\|GP156_HUMAN Probable G-protein coupled receptor 156 OS=Homo sapiens GN=GPR156 PE=2 S | 357 | PRGEKSsMERL | 0.056 | 0.102 | -1.437 | -0.426 |
| sp\|Q8NFN8\|GP156_HUMAN Probable G-protein coupled receptor 156 OS=Homo sapiens GN=GPR156 PE=2 S | 363 | SMERLLtEKNA | 0.283 | 0.851 | -0.314 | 0.273 |
| sp\|Q8NFN8\|GP156_HUMAN Probable G-protein coupled receptor 156 OS=Homo sapiens GN=GPR156 PE=2 S | 371 | KNAVIEsLQEQ | 0.174 | -0.15 | -0.736 | -0.237 |
| sp\|Q8NFN8\|GP156_HUMAN Probable G-protein coupled receptor 156 OS=Homo sapiens GN=GPR156 PE=2 S | 388 | KIVRLMsAECT | 0.542 | 1 | 0.029 | 0.524 |
| sp\|Q8NFN8\|GP156_HUMAN Probable G-protein coupled receptor 156 OS=Homo sapiens GN=GPR156 PE=2 S | 392 | LMSAECtYDLP | 0.11 | -0.085 | -1.21 | -0.395 |
| sp\|Q8NFN8\|GP156_HUMAN Probable G-protein coupled receptor 156 OS=Homo sapiens GN=GPR156 PE=2 S | 404 | GAAPPAsSPNK | 0.491 | 0.305 | -0.252 | 0.181 |
| sp\|Q8NFN8\|GP156_HUMAN Probable G-protein coupled receptor 156 OS=Homo sapiens GN=GPR156 PE=2 S | 405 | AAPPASsPNKD | 0.054 | -0.496 | -1.479 | -0.64 |
| sp\|Q8NFN8\|GP156_HUMAN Probable G-protein coupled receptor 156 OS=Homo sapiens GN=GPR156 PE=2 S | 415 | DVQAVAsVHTL | 0.362 | 0.062 | -0.677 | -0.084 |
| sp\|Q8NFN8\|GP156_HUMAN Probable G-protein coupled receptor 156 OS=Homo sapiens GN=GPR156 PE=2 S | 418 | AVASVHtLAAA | 0.375 | 0.086 | -0.276 | 0.062 |
| sp\|Q8NFN8\|GP156_HUMAN Probable G-protein coupled receptor 156 OS=Homo sapiens GN=GPR156 PE=2 S | 426 | AAAQGPsGHLS | 0.086 | -0.292 | -1.163 | -0.456 |
| sp\|Q8NFN8\|GP156_HUMAN Probable G-protein coupled receptor 156 OS=Homo sapiens GN=GPR156 PE=2 S | 430 | GPSGHLsDFQN | 0.225 | -0.019 | -0.864 | -0.219 |
| sp\|Q8NFN8\|GP156_HUMAN Probable G-protein coupled receptor 156 OS=Homo sapiens GN=GPR156 PE=2 S | 443 | GMAARDsQCTS | 0.175 | -0.056 | -1.212 | -0.364 |
| sp\|Q8NFN8\|GP156_HUMAN Probable G-protein coupled receptor 156 OS=Homo sapiens GN=GPR156 PE=2 S | 446 | ARDSQCtSGPS | 0.129 | -0.013 | -1.328 | -0.404 |
| sp\|Q8NFN8\|GP156_HUMAN Probable G-protein coupled receptor 156 OS=Homo sapiens GN=GPR156 PE=2 S | 447 | RDSQCTsGPSS | 0.363 | 0.288 | -0.239 | 0.137 |
| sp\|Q8NFN8\|GP156_HUMAN Probable G-protein coupled receptor 156 OS=Homo sapiens GN=GPR156 PE=2 S | 450 | QCTSGPsSYAQ | 0.095 | -0.199 | -1.214 | -0.439 |
| sp\|Q8NFN8\|GP156_HUMAN Probable G-protein coupled receptor 156 OS=Homo sapiens GN=GPR156 PE=2 S | 451 | CTSGPSsYAQS | 0.062 | -0.367 | -1.273 | -0.526 |
| sp\|Q8NFN8\|GP156_HUMAN Probable G-protein coupled receptor 156 OS=Homo sapiens GN=GPR156 PE=2 S | 455 | PSSYAQsLEGP | 0.324 | -0.001 | -0.227 | 0.032 |
| sp\|Q8NFN8\|GP156_HUMAN Probable G-protein coupled receptor 156 OS=Homo sapiens GN=GPR156 PE=2 S | 463 | EGPGKDsSFSP | 0.022 | -0.413 | -1.81 | -0.734 |
| sp\|Q8NFN8\|GP156_HUMAN Probable G-protein coupled receptor 156 OS=Homo sapiens GN=GPR156 PE=2 S | 464 | GPGKDSsFSPG | 0.165 | -0.15 | -1.04 | -0.342 |
| sp\|Q8NFN8\|GP156_HUMAN Probable G-protein coupled receptor 156 OS=Homo sapiens GN=GPR156 PE=2 S | 466 | GKDSSFsPGKE | 0.333 | -0.05 | -0.512 | -0.076 |
| sp\|Q8NFN8\|GP156_HUMAN Probable G-protein coupled receptor 156 OS=Homo sapiens GN=GPR156 PE=2 S | 474 | GKEEKIsDSKD | 0.237 | -0.04 | -0.877 | -0.227 |
| sp\|Q8NFN8\|GP156_HUMAN Probable G-protein coupled receptor 156 OS=Homo sapiens GN=GPR156 PE=2 S | 476 | EEKISDsKDFS | 0.207 | 0.046 | -0.69 | -0.146 |
| sp\|Q8NFN8\|GP156_HUMAN Probable G-protein coupled receptor 156 OS=Homo sapiens GN=GPR156 PE=2 S | 480 | SDSKDFsDHLD | 0.235 | -0.059 | -0.863 | -0.229 |
| sp\|Q8NFN8\|GP156_HUMAN Probable G-protein coupled receptor 156 OS=Homo sapiens GN=GPR156 PE=2 S | 485 | FSDHLDsGCSQ | 0.039 | -0.451 | -1.925 | -0.779 |
| sp\|Q8NFN8\|GP156_HUMAN Probable G-protein coupled receptor 156 OS=Homo sapiens GN=GPR156 PE=2 S | 488 | HLDSGCsQKPW | 0.102 | -0.141 | -1.293 | -0.444 |
| sp\|Q8NFN8\|GP156_HUMAN Probable G-protein coupled receptor 156 OS=Homo sapiens GN=GPR156 PE=2 S | 493 | CSQKPWtEQSL | 0.307 | 0.018 | -0.366 | -0.014 |
| sp\|Q8NFN8\|GP156_HUMAN Probable G-protein coupled receptor 156 OS=Homo sapiens GN=GPR156 PE=2 S | 496 | KPWTEQsLGPE | 0.228 | 0.006 | -0.774 | -0.18 |
| sp\|Q8NFN8\|GP156_HUMAN Probable G-protein coupled receptor 156 OS=Homo sapiens GN=GPR156 PE=2 S | 510 | QVPMNPsQSLL | 0.062 | -0.28 | -1.411 | -0.543 |

| | | | | | | |
|---|---|---|---|---|---|---|
| sp\|Q8NFN8\|GP156_HUMAN Probable G-protein coupled receptor 156 OS=Homo sapiens GN=GPR156 PE=2 S | 512 | PMNPSQsLLPE | 0.277 | 0.228 | -0.374 | 0.044 |
| sp\|Q8NFN8\|GP156_HUMAN Probable G-protein coupled receptor 156 OS=Homo sapiens GN=GPR156 PE=2 S | 520 | LPERGGsDPQR | 0.783 | 1.15 | 0.693 | 0.875 |
| sp\|Q8NFN8\|GP156_HUMAN Probable G-protein coupled receptor 156 OS=Homo sapiens GN=GPR156 PE=2 S | 531 | QRHLENsEEPP | 0.118 | 0.082 | -1.213 | -0.338 |
| sp\|Q8NFN8\|GP156_HUMAN Probable G-protein coupled receptor 156 OS=Homo sapiens GN=GPR156 PE=2 S | 539 | EPPERRsRVSS | 0.028 | -0.201 | -1.89 | -0.688 |
| sp\|Q8NFN8\|GP156_HUMAN Probable G-protein coupled receptor 156 OS=Homo sapiens GN=GPR156 PE=2 S | 542 | ERRSRVsSVIR | 0.643 | 0.493 | 0.029 | 0.388 |
| sp\|Q8NFN8\|GP156_HUMAN Probable G-protein coupled receptor 156 OS=Homo sapiens GN=GPR156 PE=2 S | 543 | RRSRVSsVIRE | 0.546 | 0.904 | 0.082 | 0.511 |
| sp\|Q8NFN8\|GP156_HUMAN Probable G-protein coupled receptor 156 OS=Homo sapiens GN=GPR156 PE=2 S | 563 | GLGPEAsLSTA | 0.345 | 0.005 | -0.559 | -0.07 |
| sp\|Q8NFN8\|GP156_HUMAN Probable G-protein coupled receptor 156 OS=Homo sapiens GN=GPR156 PE=2 S | 565 | GPEASLsTAPS | 0.152 | -0.068 | -0.904 | -0.273 |
| sp\|Q8NFN8\|GP156_HUMAN Probable G-protein coupled receptor 156 OS=Homo sapiens GN=GPR156 PE=2 S | 566 | PEASLStAPSC | 0.155 | 0.2 | -1.068 | -0.238 |
| sp\|Q8NFN8\|GP156_HUMAN Probable G-protein coupled receptor 156 OS=Homo sapiens GN=GPR156 PE=2 S | 569 | SLSTAPsCHQQ | 0.297 | -0.011 | -0.825 | -0.18 |
| sp\|Q8NFN8\|GP156_HUMAN Probable G-protein coupled receptor 156 OS=Homo sapiens GN=GPR156 PE=2 S | 574 | PSCHQQtWKNS | 0.107 | -0.014 | -1.168 | -0.358 |
| sp\|Q8NFN8\|GP156_HUMAN Probable G-protein coupled receptor 156 OS=Homo sapiens GN=GPR156 PE=2 S | 578 | QQTWKNsAAFS | 0.089 | -0.21 | -1.196 | -0.439 |
| sp\|Q8NFN8\|GP156_HUMAN Probable G-protein coupled receptor 156 OS=Homo sapiens GN=GPR156 PE=2 S | 582 | KNSAAFsPQKM | 0.087 | -0.422 | -1.142 | -0.492 |
| sp\|Q8NFN8\|GP156_HUMAN Probable G-protein coupled receptor 156 OS=Homo sapiens GN=GPR156 PE=2 S | 589 | PQKMPLsKELG | 0.123 | -0.161 | -0.672 | -0.237 |
| sp\|Q8NFN8\|GP156_HUMAN Probable G-protein coupled receptor 156 OS=Homo sapiens GN=GPR156 PE=2 S | 595 | SKELGFsPYMV | 0.065 | -0.403 | -1.635 | -0.658 |
| sp\|Q8NFN8\|GP156_HUMAN Probable G-protein coupled receptor 156 OS=Homo sapiens GN=GPR156 PE=2 S | 610 | AAQRARsHFPG | 0.689 | 0.846 | 0.222 | 0.586 |
| sp\|Q8NFN8\|GP156_HUMAN Probable G-protein coupled receptor 156 OS=Homo sapiens GN=GPR156 PE=2 S | 615 | RSHFPGsAPSS | 0.187 | 0.228 | -0.878 | -0.154 |
| sp\|Q8NFN8\|GP156_HUMAN Probable G-protein coupled receptor 156 OS=Homo sapiens GN=GPR156 PE=2 S | 618 | FPGSAPsSVGH | 0.082 | -0.272 | -1.43 | -0.54 |
| sp\|Q8NFN8\|GP156_HUMAN Probable G-protein coupled receptor 156 OS=Homo sapiens GN=GPR156 PE=2 S | 619 | PGSAPSsVGHR | 0.126 | -0.235 | -0.703 | -0.271 |
| sp\|Q8NFN8\|GP156_HUMAN Probable G-protein coupled receptor 156 OS=Homo sapiens GN=GPR156 PE=2 S | 627 | GHRANRtVPGA | 0.762 | 0.643 | 0.196 | 0.534 |
| sp\|Q8NFN8\|GP156_HUMAN Probable G-protein coupled receptor 156 OS=Homo sapiens GN=GPR156 PE=2 S | 633 | TVPGAHsRLHV | 0.089 | -0.096 | -1.325 | -0.444 |
| sp\|Q8NFN8\|GP156_HUMAN Probable G-protein coupled receptor 156 OS=Homo sapiens GN=GPR156 PE=2 S | 642 | HVQNGDsPSLA | 0.027 | -0.437 | -1.592 | -0.667 |
| sp\|Q8NFN8\|GP156_HUMAN Probable G-protein coupled receptor 156 OS=Homo sapiens GN=GPR156 PE=2 S | 644 | QNGDSPsLAPQ | 0.182 | -0.063 | -0.68 | -0.187 |
| sp\|Q8NFN8\|GP156_HUMAN Probable G-protein coupled receptor 156 OS=Homo sapiens GN=GPR156 PE=2 S | 649 | PSLAPQtTDSR | 0.122 | -0.196 | -0.781 | -0.285 |
| sp\|Q8NFN8\|GP156_HUMAN Probable G-protein coupled receptor 156 OS=Homo sapiens GN=GPR156 PE=2 S | 650 | SLAPQTtDSRV | 0.111 | -0.168 | -1.493 | -0.517 |
| sp\|Q8NFN8\|GP156_HUMAN Probable G-protein coupled receptor 156 OS=Homo sapiens GN=GPR156 PE=2 S | 652 | APQTTDsRVRR | 0.097 | -0.286 | -1.646 | -0.612 |
| sp\|Q8NFN8\|GP156_HUMAN Probable G-protein coupled receptor 156 OS=Homo sapiens GN=GPR156 PE=2 S | 658 | SRVRRPsSRKP | 0.474 | 0.904 | -0.073 | 0.435 |
| sp\|Q8NFN8\|GP156_HUMAN Probable G-protein coupled receptor 156 OS=Homo sapiens GN=GPR156 PE=2 S | 659 | RVRRPSsRKPS | 0.431 | 0.794 | 0.045 | 0.423 |
| sp\|Q8NFN8\|GP156_HUMAN Probable G-protein coupled receptor 156 OS=Homo sapiens GN=GPR156 PE=2 S | 663 | PSSRKPsLPSD | 0.864 | 1.235 | 1.127 | 1.075 |
| sp\|Q8NFN8\|GP156_HUMAN Probable G-protein coupled receptor 156 OS=Homo sapiens GN=GPR156 PE=2 S | 666 | RKPSLPsDPQD | 0.649 | 0.392 | -0.076 | 0.322 |
| sp\|Q8NFN8\|GP156_HUMAN Probable G-protein coupled receptor 156 OS=Homo sapiens GN=GPR156 PE=2 S | 674 | PQDRPGtLEGS | 0.452 | 0.668 | 0.228 | 0.449 |
| sp\|Q8NFN8\|GP156_HUMAN Probable G-protein coupled receptor 156 OS=Homo sapiens GN=GPR156 PE=2 S | 678 | PGTLEGsKQSQ | 0.079 | -0.237 | -1.282 | -0.48 |
| sp\|Q8NFN8\|GP156_HUMAN Probable G-protein coupled receptor 156 OS=Homo sapiens GN=GPR156 PE=2 S | 681 | LEGSKQsQTEP | 0.062 | -0.307 | -1.659 | -0.635 |
| sp\|Q8NFN8\|GP156_HUMAN Probable G-protein coupled receptor 156 OS=Homo sapiens GN=GPR156 PE=2 S | 683 | GSKQSQtEPEG | 0.909 | 0.827 | 1.087 | 0.941 |
| sp\|Q8NFN8\|GP156_HUMAN Probable G-protein coupled receptor 156 OS=Homo sapiens GN=GPR156 PE=2 S | 691 | PEGARGsKAAF | 0.153 | -0.038 | -0.87 | -0.252 |
| sp\|Q8NFN8\|GP156_HUMAN Probable G-protein coupled receptor 156 OS=Homo sapiens GN=GPR156 PE=2 S | 700 | AFLRQPsGSGR | 0.371 | 0.603 | -0.032 | 0.314 |
| sp\|Q8NFN8\|GP156_HUMAN Probable G-protein coupled receptor 156 OS=Homo sapiens GN=GPR156 PE=2 S | 702 | LRQPSGsGRAP | 0.222 | 0.216 | -0.373 | 0.022 |
| sp\|Q8NFN8\|GP156_HUMAN Probable G-protein coupled receptor 156 OS=Homo sapiens GN=GPR156 PE=2 S | 707 | GSGRAPsPAAP | 0.338 | 0.472 | -0.244 | 0.189 |
| sp\|Q8NFN8\|GP156_HUMAN Probable G-protein coupled receptor 156 OS=Homo sapiens GN=GPR156 PE=2 S | 714 | PAAPCLsKASP | 0.055 | -0.31 | -1.24 | -0.498 |
| sp\|Q8NFN8\|GP156_HUMAN Probable G-protein coupled receptor 156 OS=Homo sapiens GN=GPR156 PE=2 S | 717 | PCLSKAsPDLP | 0.101 | -0.342 | -1.313 | -0.518 |
| sp\|Q8NFN8\|GP156_HUMAN Probable G-protein coupled receptor 156 OS=Homo sapiens GN=GPR156 PE=2 S | 732 | LWPPVPsGCAS | 0.061 | -0.297 | -1.322 | -0.519 |
| sp\|Q8NFN8\|GP156_HUMAN Probable G-protein coupled receptor 156 OS=Homo sapiens GN=GPR156 PE=2 S | 736 | VPSGCAsLSSQ | 0.154 | -0.147 | -0.933 | -0.309 |
| sp\|Q8NFN8\|GP156_HUMAN Probable G-protein coupled receptor 156 OS=Homo sapiens GN=GPR156 PE=2 S | 738 | SGCASLsSQHS | 0.106 | -0.079 | -0.952 | -0.308 |
| sp\|Q8NFN8\|GP156_HUMAN Probable G-protein coupled receptor 156 OS=Homo sapiens GN=GPR156 PE=2 S | 739 | GCASLSsQHSY | 0.108 | -0.167 | -1.549 | -0.536 |
| sp\|Q8NFN8\|GP156_HUMAN Probable G-protein coupled receptor 156 OS=Homo sapiens GN=GPR156 PE=2 S | 742 | SLSSQHsYFDT | 0.184 | 0.003 | -0.875 | -0.229 |
| sp\|Q8NFN8\|GP156_HUMAN Probable G-protein coupled receptor 156 OS=Homo sapiens GN=GPR156 PE=2 S | 746 | QHSYFDtESSS | 0.048 | -0.229 | -1.765 | -0.649 |
| sp\|Q8NFN8\|GP156_HUMAN Probable G-protein coupled receptor 156 OS=Homo sapiens GN=GPR156 PE=2 S | 748 | SYFDTEsSSSD | 0.056 | -0.169 | -1.721 | -0.611 |
| sp\|Q8NFN8\|GP156_HUMAN Probable G-protein coupled receptor 156 OS=Homo sapiens GN=GPR156 PE=2 S | 749 | YFDTESsSSDE | 0.088 | -0.25 | -1.363 | -0.508 |
| sp\|Q8NFN8\|GP156_HUMAN Probable G-protein coupled receptor 156 OS=Homo sapiens GN=GPR156 PE=2 S | 750 | FDTESSsSDEF | 0.132 | 0.084 | -0.739 | -0.174 |

| | | | | | | |
|---|---|---|---|---|---|---|
| sp\|Q8NFN8\|GP156_HUMAN Probable G-protein coupled receptor 156 OS=Homo sapiens GN=GPR156 PE=2 S | 751 | DTESSSsDEFF | 0.18 | -0.068 | -1.055 | -0.314 |
| sp\|Q8NFN8\|GP156_HUMAN Probable G-protein coupled receptor 156 OS=Homo sapiens GN=GPR156 PE=2 S | 769 | CEICFQsSSDS | 0.173 | -0.083 | -1.156 | -0.355 |
| sp\|Q8NFN8\|GP156_HUMAN Probable G-protein coupled receptor 156 OS=Homo sapiens GN=GPR156 PE=2 S | 770 | EICFQSsSDSS | 0.058 | -0.096 | -1.435 | -0.491 |
| sp\|Q8NFN8\|GP156_HUMAN Probable G-protein coupled receptor 156 OS=Homo sapiens GN=GPR156 PE=2 S | 771 | ICFQSSsDSSD | 0.338 | 0.132 | -0.509 | -0.013 |
| sp\|Q8NFN8\|GP156_HUMAN Probable G-protein coupled receptor 156 OS=Homo sapiens GN=GPR156 PE=2 S | 773 | FQSSSDsSDSG | 0.176 | 0.033 | -0.732 | -0.174 |
| sp\|Q8NFN8\|GP156_HUMAN Probable G-protein coupled receptor 156 OS=Homo sapiens GN=GPR156 PE=2 S | 774 | QSSSDSsDSGT | 0.055 | -0.442 | -1.577 | -0.655 |
| sp\|Q8NFN8\|GP156_HUMAN Probable G-protein coupled receptor 156 OS=Homo sapiens GN=GPR156 PE=2 S | 776 | SSDSSDsGTSD | 0.136 | -0.203 | -1.222 | -0.43 |
| sp\|Q8NFN8\|GP156_HUMAN Probable G-protein coupled receptor 156 OS=Homo sapiens GN=GPR156 PE=2 S | 778 | DSSDSGtSDTD | 0.324 | 0.007 | -0.674 | -0.114 |
| sp\|Q8NFN8\|GP156_HUMAN Probable G-protein coupled receptor 156 OS=Homo sapiens GN=GPR156 PE=2 S | 779 | SSDSGTsDTDP | 0.089 | -0.301 | -1.483 | -0.565 |
| sp\|Q8NFN8\|GP156_HUMAN Probable G-protein coupled receptor 156 OS=Homo sapiens GN=GPR156 PE=2 S | 781 | DSGTSDtDPEP | 0.65 | 0.474 | -0.22 | 0.301 |
| sp\|Q8NFN8\|GP156_HUMAN Probable G-protein coupled receptor 156 OS=Homo sapiens GN=GPR156 PE=2 S | 786 | DTDPEPtGGLA | 0.17 | -0.275 | -1.174 | -0.426 |
| sp\|Q8NFN8\|GP156_HUMAN Probable G-protein coupled receptor 156 OS=Homo sapiens GN=GPR156 PE=2 S | 791 | PTGGLAsWEKL | 0.108 | 0.012 | -1.151 | -0.344 |
| sp\|Q8NFN8\|GP156_HUMAN Probable G-protein coupled receptor 156 OS=Homo sapiens GN=GPR156 PE=2 S | 799 | EKLWARsKPIV | 0.773 | 0.555 | 0.292 | 0.54 |
| sp\|Q8NFN8\|GP156_HUMAN Probable G-protein coupled receptor 156 OS=Homo sapiens GN=GPR156 PE=2 S | 812 | KDDLKPtLV-- | 0.091 | -0.088 | -1.203 | -0.4 |
| sp\|Q5T848\|GP158_HUMAN Probable G-protein coupled receptor 158 OS=Homo sapiens GN=GPR158 PE=1 S | 319 | VDIDQCsSDGW | 0.099 | -0.161 | -1.414 | -0.492 |
| sp\|Q5T848\|GP158_HUMAN Probable G-protein coupled receptor 158 OS=Homo sapiens GN=GPR158 PE=1 S | 320 | DIDQCSsDGWF | 0.357 | 0.041 | -0.559 | -0.054 |
| sp\|Q5T848\|GP158_HUMAN Probable G-protein coupled receptor 158 OS=Homo sapiens GN=GPR158 PE=1 S | 325 | SSDGWFsGTHK | 0.079 | -0.286 | -1.148 | -0.452 |
| sp\|Q5T848\|GP158_HUMAN Probable G-protein coupled receptor 158 OS=Homo sapiens GN=GPR158 PE=1 S | 327 | DGWFSGtHKCH | 0.217 | 0.129 | -0.824 | -0.159 |
| sp\|Q5T848\|GP158_HUMAN Probable G-protein coupled receptor 158 OS=Homo sapiens GN=GPR158 PE=1 S | 335 | KCHLNNsECMP | 0.132 | -0.157 | -1.372 | -0.466 |
| sp\|Q5T848\|GP158_HUMAN Probable G-protein coupled receptor 158 OS=Homo sapiens GN=GPR158 PE=1 S | 379 | GPDQHIsGSTK | 0.178 | -0.205 | -0.969 | -0.332 |
| sp\|Q5T848\|GP158_HUMAN Probable G-protein coupled receptor 158 OS=Homo sapiens GN=GPR158 PE=1 S | 381 | DQHISGsTKDV | 0.204 | 0.069 | -0.905 | -0.211 |
| sp\|Q5T848\|GP158_HUMAN Probable G-protein coupled receptor 158 OS=Homo sapiens GN=GPR158 PE=1 S | 382 | QHISGStKDVS | 0.049 | -0.207 | -1.612 | -0.59 |
| sp\|Q5T848\|GP158_HUMAN Probable G-protein coupled receptor 158 OS=Homo sapiens GN=GPR158 PE=1 S | 386 | GSTKDVsEEAY | 0.634 | 0.158 | -0.043 | 0.25 |
| sp\|Q5T848\|GP158_HUMAN Probable G-protein coupled receptor 158 OS=Homo sapiens GN=GPR158 PE=1 S | 406 | PFCADDsPCFV | 0.06 | -0.594 | -1.865 | -0.8 |
| sp\|Q5T848\|GP158_HUMAN Probable G-protein coupled receptor 158 OS=Homo sapiens GN=GPR158 PE=1 S | 422 | LRLAIIsFQAL | 0.476 | 0.347 | 0.268 | 0.364 |
| sp\|Q5T848\|GP158_HUMAN Probable G-protein coupled receptor 158 OS=Homo sapiens GN=GPR158 PE=1 S | 434 | MLLDFVsMLVV | 0.448 | 0.25 | -0.757 | -0.02 |
| sp\|Q5T848\|GP158_HUMAN Probable G-protein coupled receptor 158 OS=Homo sapiens GN=GPR158 PE=1 S | 446 | HFRKAKsIRAS | 0.766 | 0.443 | 0.702 | 0.637 |
| sp\|Q5T848\|GP158_HUMAN Probable G-protein coupled receptor 158 OS=Homo sapiens GN=GPR158 PE=1 S | 450 | AKSIRAsGLIL | 0.413 | 0.133 | -0.483 | 0.021 |
| sp\|Q5T848\|GP158_HUMAN Probable G-protein coupled receptor 158 OS=Homo sapiens GN=GPR158 PE=1 S | 457 | GLILLEtILFG | 0.099 | -0.113 | -1.411 | -0.475 |
| sp\|Q5T848\|GP158_HUMAN Probable G-protein coupled receptor 158 OS=Homo sapiens GN=GPR158 PE=1 S | 462 | ETILFGsLLLY | 0.179 | -0.03 | -1.126 | -0.326 |
| sp\|Q5T848\|GP158_HUMAN Probable G-protein coupled receptor 158 OS=Homo sapiens GN=GPR158 PE=1 S | 477 | ILYFEPsTFRC | 0.23 | -0.182 | -1.065 | -0.339 |
| sp\|Q5T848\|GP158_HUMAN Probable G-protein coupled receptor 158 OS=Homo sapiens GN=GPR158 PE=1 S | 478 | LYFEPStFRCI | 0.183 | -0.035 | -0.625 | -0.159 |
| sp\|Q5T848\|GP158_HUMAN Probable G-protein coupled receptor 158 OS=Homo sapiens GN=GPR158 PE=1 S | 494 | RLLGFAtVYGT | 0.31 | 0.044 | -0.47 | -0.039 |
| sp\|Q5T848\|GP158_HUMAN Probable G-protein coupled receptor 158 OS=Homo sapiens GN=GPR158 PE=1 S | 498 | FATVYGtVTLK | 0.138 | -0.12 | -1.056 | -0.346 |
| sp\|Q5T848\|GP158_HUMAN Probable G-protein coupled receptor 158 OS=Homo sapiens GN=GPR158 PE=1 S | 500 | TVYGTVtLKLH | 0.138 | -0.059 | -0.751 | -0.224 |
| sp\|Q5T848\|GP158_HUMAN Probable G-protein coupled receptor 158 OS=Homo sapiens GN=GPR158 PE=1 S | 512 | VLKVFLsRTAQ | 0.176 | -0.109 | -0.979 | -0.304 |
| sp\|Q5T848\|GP158_HUMAN Probable G-protein coupled receptor 158 OS=Homo sapiens GN=GPR158 PE=1 S | 514 | KVFLSRtAQRI | 0.352 | 0.193 | -0.645 | -0.033 |
| sp\|Q5T848\|GP158_HUMAN Probable G-protein coupled receptor 158 OS=Homo sapiens GN=GPR158 PE=1 S | 522 | QRIPYMtGGRV | 0.325 | 0.212 | -0.7 | -0.054 |
| sp\|Q5T848\|GP158_HUMAN Probable G-protein coupled receptor 158 OS=Homo sapiens GN=GPR158 PE=1 S | 545 | WFLIGWtSSVC | 0.057 | -0.287 | -1.404 | -0.545 |
| sp\|Q5T848\|GP158_HUMAN Probable G-protein coupled receptor 158 OS=Homo sapiens GN=GPR158 PE=1 S | 546 | FLIGWTsSVCQ | 0.229 | 0.167 | -0.691 | -0.098 |
| sp\|Q5T848\|GP158_HUMAN Probable G-protein coupled receptor 158 OS=Homo sapiens GN=GPR158 PE=1 S | 547 | LIGWTSsVCQN | 0.085 | -0.137 | -1.061 | -0.371 |
| sp\|Q5T848\|GP158_HUMAN Probable G-protein coupled receptor 158 OS=Homo sapiens GN=GPR158 PE=1 S | 557 | NLEKQIsLIGQ | 0.51 | 0.097 | -0.033 | 0.191 |
| sp\|Q5T848\|GP158_HUMAN Probable G-protein coupled receptor 158 OS=Homo sapiens GN=GPR158 PE=1 S | 564 | LIGQGKtSDHL | 0.142 | -0.006 | -0.483 | -0.116 |
| sp\|Q5T848\|GP158_HUMAN Probable G-protein coupled receptor 158 OS=Homo sapiens GN=GPR158 PE=1 S | 565 | IGQGKTsDHLI | 0.316 | 0.072 | -0.378 | 0.003 |
| sp\|Q5T848\|GP158_HUMAN Probable G-protein coupled receptor 158 OS=Homo sapiens GN=GPR158 PE=1 S | 582 | DRWDYMtAVAE | 0.443 | 0.523 | -0.441 | 0.175 |
| sp\|Q5T848\|GP158_HUMAN Probable G-protein coupled receptor 158 OS=Homo sapiens GN=GPR158 PE=1 S | 602 | LCYAVRtVPSA | 0.349 | 0.389 | -0.418 | 0.107 |
| sp\|Q5T848\|GP158_HUMAN Probable G-protein coupled receptor 158 OS=Homo sapiens GN=GPR158 PE=1 S | 605 | AVRTVPsAFHE | 0.275 | 0.043 | -0.798 | -0.16 |
| sp\|Q5T848\|GP158_HUMAN Probable G-protein coupled receptor 158 OS=Homo sapiens GN=GPR158 PE=1 S | 624 | HNELIIsAIFH | 0.07 | -0.238 | -1.151 | -0.44 |
| sp\|Q5T848\|GP158_HUMAN Probable G-protein coupled receptor 158 OS=Homo sapiens GN=GPR158 PE=1 S | 629 | ISAIFHtIRFV | 0.165 | -0.157 | -1.031 | -0.341 |

| | | | | | | |
|---|---|---|---|---|---|---|
| sp\|Q5T848\|GP158_HUMAN Probable G-protein coupled receptor 158 OS=Homo sapiens GN=GPR158 PE=1 S' | 636 | IRFVLAsRLQS | 0.122 | -0.006 | -1.216 | -0.367 |
| sp\|Q5T848\|GP158_HUMAN Probable G-protein coupled receptor 158 OS=Homo sapiens GN=GPR158 PE=1 S' | 640 | LASRLQsDWML | 0.565 | 0.788 | 0.017 | 0.457 |
| sp\|Q5T848\|GP158_HUMAN Probable G-protein coupled receptor 158 OS=Homo sapiens GN=GPR158 PE=1 S' | 651 | MLYFAHtHLTV | 0.584 | 0.334 | -0.309 | 0.203 |
| sp\|Q5T848\|GP158_HUMAN Probable G-protein coupled receptor 158 OS=Homo sapiens GN=GPR158 PE=1 S' | 654 | FAHTHLtVTVT | 0.12 | -0.08 | -1.194 | -0.385 |
| sp\|Q5T848\|GP158_HUMAN Probable G-protein coupled receptor 158 OS=Homo sapiens GN=GPR158 PE=1 S' | 656 | HTHLTVtVTIG | 0.266 | 0.003 | -0.792 | -0.174 |
| sp\|Q5T848\|GP158_HUMAN Probable G-protein coupled receptor 158 OS=Homo sapiens GN=GPR158 PE=1 S' | 658 | HLTVTVtIGLL | 0.294 | 0.208 | -0.187 | 0.105 |
| sp\|Q5T848\|GP158_HUMAN Probable G-protein coupled receptor 158 OS=Homo sapiens GN=GPR158 PE=1 S' | 668 | LLIPKFsHSSN | 0.084 | -0.068 | -1.067 | -0.35 |
| sp\|Q5T848\|GP158_HUMAN Probable G-protein coupled receptor 158 OS=Homo sapiens GN=GPR158 PE=1 S' | 670 | IPKFSHsSNNP | 0.509 | 0.3 | -0.12 | 0.23 |
| sp\|Q5T848\|GP158_HUMAN Probable G-protein coupled receptor 158 OS=Homo sapiens GN=GPR158 PE=1 S' | 671 | PKFSHSsNNPR | 0.288 | -0.053 | -0.696 | -0.154 |
| sp\|Q5T848\|GP158_HUMAN Probable G-protein coupled receptor 158 OS=Homo sapiens GN=GPR158 PE=1 S' | 680 | PRDDIAtEAYE | 0.27 | 0.171 | -0.73 | -0.096 |
| sp\|Q5T848\|GP158_HUMAN Probable G-protein coupled receptor 158 OS=Homo sapiens GN=GPR158 PE=1 S' | 692 | ELDMGRsGSYL | 0.053 | -0.226 | -1.46 | -0.544 |
| sp\|Q5T848\|GP158_HUMAN Probable G-protein coupled receptor 158 OS=Homo sapiens GN=GPR158 PE=1 S' | 694 | DMGRSGsYLNS | 0.388 | 0.988 | -0.28 | 0.365 |
| sp\|Q5T848\|GP158_HUMAN Probable G-protein coupled receptor 158 OS=Homo sapiens GN=GPR158 PE=1 S' | 698 | SGSYLNsSINS | 0.075 | -0.136 | -1.385 | -0.482 |
| sp\|Q5T848\|GP158_HUMAN Probable G-protein coupled receptor 158 OS=Homo sapiens GN=GPR158 PE=1 S' | 699 | GSYLNSsINSA | 0.082 | -0.219 | -1.027 | -0.388 |
| sp\|Q5T848\|GP158_HUMAN Probable G-protein coupled receptor 158 OS=Homo sapiens GN=GPR158 PE=1 S' | 702 | LNSSINsAWSE | 0.127 | -0.165 | -1.095 | -0.378 |
| sp\|Q5T848\|GP158_HUMAN Probable G-protein coupled receptor 158 OS=Homo sapiens GN=GPR158 PE=1 S' | 705 | SINSAWsEHSL | 0.183 | 0.037 | -0.996 | -0.259 |
| sp\|Q5T848\|GP158_HUMAN Probable G-protein coupled receptor 158 OS=Homo sapiens GN=GPR158 PE=1 S' | 708 | SAWSEHsLDPE | 0.213 | 0.043 | -0.866 | -0.203 |
| sp\|Q5T848\|GP158_HUMAN Probable G-protein coupled receptor 158 OS=Homo sapiens GN=GPR158 PE=1 S' | 735 | KRKKMItNNPH | 0.341 | 0.001 | -0.611 | -0.09 |
| sp\|Q5T848\|GP158_HUMAN Probable G-protein coupled receptor 158 OS=Homo sapiens GN=GPR158 PE=1 S' | 746 | LQKKRCsKKGL | 0.56 | 0.175 | 0.125 | 0.287 |
| sp\|Q5T848\|GP158_HUMAN Probable G-protein coupled receptor 158 OS=Homo sapiens GN=GPR158 PE=1 S' | 753 | KKGLGRsIMRR | 0.094 | -0.206 | -1.385 | -0.499 |
| sp\|Q5T848\|GP158_HUMAN Probable G-protein coupled receptor 158 OS=Homo sapiens GN=GPR158 PE=1 S' | 759 | SIMRRItEIPE | 0.683 | 1.033 | 0.522 | 0.746 |
| sp\|Q5T848\|GP158_HUMAN Probable G-protein coupled receptor 158 OS=Homo sapiens GN=GPR158 PE=1 S' | 764 | ITEIPEtVSRQ | 0.096 | -0.371 | -1.438 | -0.571 |
| sp\|Q5T848\|GP158_HUMAN Probable G-protein coupled receptor 158 OS=Homo sapiens GN=GPR158 PE=1 S' | 766 | EIPETVsRQCS | 0.077 | -0.069 | -1.281 | -0.424 |
| sp\|Q5T848\|GP158_HUMAN Probable G-protein coupled receptor 158 OS=Homo sapiens GN=GPR158 PE=1 S' | 770 | TVSRQCsKEDK | 0.565 | 0.786 | 0.076 | 0.476 |
| sp\|Q5T848\|GP158_HUMAN Probable G-protein coupled receptor 158 OS=Homo sapiens GN=GPR158 PE=1 S' | 781 | EGADHGtAKGT | 0.104 | -0.168 | -1.036 | -0.367 |
| sp\|Q5T848\|GP158_HUMAN Probable G-protein coupled receptor 158 OS=Homo sapiens GN=GPR158 PE=1 S' | 785 | HGTAKGtALIR | 0.1 | -0.14 | -1.116 | -0.385 |
| sp\|Q5T848\|GP158_HUMAN Probable G-protein coupled receptor 158 OS=Homo sapiens GN=GPR158 PE=1 S' | 795 | RKNPPEsSGNT | 0.195 | -0.12 | -1.021 | -0.315 |
| sp\|Q5T848\|GP158_HUMAN Probable G-protein coupled receptor 158 OS=Homo sapiens GN=GPR158 PE=1 S' | 796 | KNPPESsGNTG | 0.044 | -0.43 | -1.502 | -0.629 |
| sp\|Q5T848\|GP158_HUMAN Probable G-protein coupled receptor 158 OS=Homo sapiens GN=GPR158 PE=1 S' | 799 | PESSGNtGKSK | 0.055 | -0.318 | -1.482 | -0.582 |
| sp\|Q5T848\|GP158_HUMAN Probable G-protein coupled receptor 158 OS=Homo sapiens GN=GPR158 PE=1 S' | 802 | SGNTGKsKEET | 0.09 | -0.207 | -1.108 | -0.408 |
| sp\|Q5T848\|GP158_HUMAN Probable G-protein coupled receptor 158 OS=Homo sapiens GN=GPR158 PE=1 S' | 806 | GKSKEEtLKNR | 0.658 | 0.149 | -0.25 | 0.186 |
| sp\|Q5T848\|GP158_HUMAN Probable G-protein coupled receptor 158 OS=Homo sapiens GN=GPR158 PE=1 S' | 813 | LKNRVFsLKKS | 0.754 | 0.958 | 0.657 | 0.79 |
| sp\|Q5T848\|GP158_HUMAN Probable G-protein coupled receptor 158 OS=Homo sapiens GN=GPR158 PE=1 S' | 817 | VFSLKKsHSTY | 0.205 | -0.11 | -0.754 | -0.22 |
| sp\|Q5T848\|GP158_HUMAN Probable G-protein coupled receptor 158 OS=Homo sapiens GN=GPR158 PE=1 S' | 819 | SLKKSHsTYDH | 0.715 | 0.383 | 0.091 | 0.396 |
| sp\|Q5T848\|GP158_HUMAN Probable G-protein coupled receptor 158 OS=Homo sapiens GN=GPR158 PE=1 S' | 820 | LKKSHStYDHV | 0.226 | -0.071 | -0.727 | -0.191 |
| sp\|Q5T848\|GP158_HUMAN Probable G-protein coupled receptor 158 OS=Homo sapiens GN=GPR158 PE=1 S' | 828 | DHVRDQtEESS | 0.354 | 0.784 | -0.454 | 0.228 |
| sp\|Q5T848\|GP158_HUMAN Probable G-protein coupled receptor 158 OS=Homo sapiens GN=GPR158 PE=1 S' | 831 | RDQTEEsSSLP | 0.049 | -0.298 | -1.857 | -0.702 |
| sp\|Q5T848\|GP158_HUMAN Probable G-protein coupled receptor 158 OS=Homo sapiens GN=GPR158 PE=1 S' | 832 | DQTEESsSLPT | 0.032 | -0.373 | -1.779 | -0.707 |
| sp\|Q5T848\|GP158_HUMAN Probable G-protein coupled receptor 158 OS=Homo sapiens GN=GPR158 PE=1 S' | 833 | QTEESSsLPTE | 0.671 | 0.567 | 0.326 | 0.521 |
| sp\|Q5T848\|GP158_HUMAN Probable G-protein coupled receptor 158 OS=Homo sapiens GN=GPR158 PE=1 S' | 836 | ESSSLPtESQE | 0.057 | -0.364 | -1.443 | -0.583 |
| sp\|Q5T848\|GP158_HUMAN Probable G-protein coupled receptor 158 OS=Homo sapiens GN=GPR158 PE=1 S' | 838 | SSLPTEsQEEE | 0.051 | -0.318 | -1.752 | -0.673 |
| sp\|Q5T848\|GP158_HUMAN Probable G-protein coupled receptor 158 OS=Homo sapiens GN=GPR158 PE=1 S' | 843 | ESQEEEtTENS | 0.051 | -0.187 | -1.784 | -0.64 |
| sp\|Q5T848\|GP158_HUMAN Probable G-protein coupled receptor 158 OS=Homo sapiens GN=GPR158 PE=1 S' | 844 | SQEEETtENST | 0.051 | -0.186 | -1.266 | -0.467 |
| sp\|Q5T848\|GP158_HUMAN Probable G-protein coupled receptor 158 OS=Homo sapiens GN=GPR158 PE=1 S' | 847 | EETTENsTLES | 0.114 | -0.134 | -1.415 | -0.478 |
| sp\|Q5T848\|GP158_HUMAN Probable G-protein coupled receptor 158 OS=Homo sapiens GN=GPR158 PE=1 S' | 848 | ETTENStLESL | 0.089 | -0.078 | -0.92 | -0.303 |
| sp\|Q5T848\|GP158_HUMAN Probable G-protein coupled receptor 158 OS=Homo sapiens GN=GPR158 PE=1 S' | 851 | ENSTLEsLSGK | 0.081 | -0.347 | -1.242 | -0.503 |
| sp\|Q5T848\|GP158_HUMAN Probable G-protein coupled receptor 158 OS=Homo sapiens GN=GPR158 PE=1 S' | 853 | STLESLsGKKL | 0.078 | -0.032 | -1.101 | -0.352 |
| sp\|Q5T848\|GP158_HUMAN Probable G-protein coupled receptor 158 OS=Homo sapiens GN=GPR158 PE=1 S' | 858 | LSGKKLtQKLK | 0.067 | -0.281 | -1.363 | -0.526 |
| sp\|Q5T848\|GP158_HUMAN Probable G-protein coupled receptor 158 OS=Homo sapiens GN=GPR158 PE=1 S' | 865 | QKLKEDsEAES | 0.363 | -0.029 | -0.705 | -0.124 |

| | | | | | | |
|---|---|---|---|---|---|---|
| sp\|Q5T848\|GP158_HUMAN Probable G-protein coupled receptor 158 OS=Homo sapiens GN=GPR158 PE=1 S | 869 | EDSEAEsTESV | 0.038 | -0.249 | -1.828 | -0.68 |
| sp\|Q5T848\|GP158_HUMAN Probable G-protein coupled receptor 158 OS=Homo sapiens GN=GPR158 PE=1 S | 870 | DSEAEStESVP | 0.083 | -0.294 | -1.526 | -0.579 |
| sp\|Q5T848\|GP158_HUMAN Probable G-protein coupled receptor 158 OS=Homo sapiens GN=GPR158 PE=1 S | 872 | EAESTEsVPLV | 0.294 | 0.34 | -0.65 | -0.005 |
| sp\|Q5T848\|GP158_HUMAN Probable G-protein coupled receptor 158 OS=Homo sapiens GN=GPR158 PE=1 S | 879 | VPLVCKsASAH | 0.146 | -0.123 | -0.986 | -0.321 |
| sp\|Q5T848\|GP158_HUMAN Probable G-protein coupled receptor 158 OS=Homo sapiens GN=GPR158 PE=1 S | 881 | LVCKSAsAHNL | 0.649 | 0.428 | -0.096 | 0.327 |
| sp\|Q5T848\|GP158_HUMAN Probable G-protein coupled receptor 158 OS=Homo sapiens GN=GPR158 PE=1 S | 886 | ASAHNLsSEKK | 0.067 | -0.334 | -1.279 | -0.515 |
| sp\|Q5T848\|GP158_HUMAN Probable G-protein coupled receptor 158 OS=Homo sapiens GN=GPR158 PE=1 S | 887 | SAHNLSsEKKT | 0.022 | -0.336 | -1.794 | -0.703 |
| sp\|Q5T848\|GP158_HUMAN Probable G-protein coupled receptor 158 OS=Homo sapiens GN=GPR158 PE=1 S | 891 | LSSEKKtGHPR | 0.091 | -0.233 | -0.962 | -0.368 |
| sp\|Q5T848\|GP158_HUMAN Probable G-protein coupled receptor 158 OS=Homo sapiens GN=GPR158 PE=1 S | 896 | KTGHPRtSMLQ | 0.057 | -0.308 | -1.654 | -0.635 |
| sp\|Q5T848\|GP158_HUMAN Probable G-protein coupled receptor 158 OS=Homo sapiens GN=GPR158 PE=1 S | 897 | TGHPRTsMLQK | 0.134 | -0.025 | -0.916 | -0.269 |
| sp\|Q5T848\|GP158_HUMAN Probable G-protein coupled receptor 158 OS=Homo sapiens GN=GPR158 PE=1 S | 902 | TSMLQKsLSVI | 0.248 | 0.081 | -0.438 | -0.036 |
| sp\|Q5T848\|GP158_HUMAN Probable G-protein coupled receptor 158 OS=Homo sapiens GN=GPR158 PE=1 S | 904 | MLQKSLsVIAS | 0.625 | 0.499 | 0.389 | 0.504 |
| sp\|Q5T848\|GP158_HUMAN Probable G-protein coupled receptor 158 OS=Homo sapiens GN=GPR158 PE=1 S | 908 | SLSVIAsAKEK | 0.193 | -0.031 | -0.868 | -0.235 |
| sp\|Q5T848\|GP158_HUMAN Probable G-protein coupled receptor 158 OS=Homo sapiens GN=GPR158 PE=1 S | 913 | ASAKEKtLGLA | 0.644 | 0.086 | 0.161 | 0.297 |
| sp\|Q5T848\|GP158_HUMAN Probable G-protein coupled receptor 158 OS=Homo sapiens GN=GPR158 PE=1 S | 920 | LGLAGKtQTAG | 0.227 | -0.095 | -0.572 | -0.147 |
| sp\|Q5T848\|GP158_HUMAN Probable G-protein coupled receptor 158 OS=Homo sapiens GN=GPR158 PE=1 S | 922 | LAGKTQtAGVE | 0.207 | 0.066 | -0.752 | -0.16 |
| sp\|Q5T848\|GP158_HUMAN Probable G-protein coupled receptor 158 OS=Homo sapiens GN=GPR158 PE=1 S | 929 | AGVEERtKSQK | 0.055 | -0.321 | -1.61 | -0.625 |
| sp\|Q5T848\|GP158_HUMAN Probable G-protein coupled receptor 158 OS=Homo sapiens GN=GPR158 PE=1 S | 931 | VEERTKsQKPL | 0.458 | 0.769 | 0.088 | 0.438 |
| sp\|Q5T848\|GP158_HUMAN Probable G-protein coupled receptor 158 OS=Homo sapiens GN=GPR158 PE=1 S | 941 | LPKDKEtNRNH | 0.079 | -0.323 | -1.762 | -0.669 |
| sp\|Q5T848\|GP158_HUMAN Probable G-protein coupled receptor 158 OS=Homo sapiens GN=GPR158 PE=1 S | 946 | ETNRNHsNSDN | 0.639 | 0.897 | 0.106 | 0.547 |
| sp\|Q5T848\|GP158_HUMAN Probable G-protein coupled receptor 158 OS=Homo sapiens GN=GPR158 PE=1 S | 948 | NRNHSNsDNTE | 0.582 | 0.426 | -0.116 | 0.297 |
| sp\|Q5T848\|GP158_HUMAN Probable G-protein coupled receptor 158 OS=Homo sapiens GN=GPR158 PE=1 S | 951 | HSNSDNtETKD | 0.229 | -0.041 | -0.88 | -0.231 |
| sp\|Q5T848\|GP158_HUMAN Probable G-protein coupled receptor 158 OS=Homo sapiens GN=GPR158 PE=1 S | 953 | NSDNTEtKDPA | 0.087 | -0.202 | -0.998 | -0.371 |
| sp\|Q5T848\|GP158_HUMAN Probable G-protein coupled receptor 158 OS=Homo sapiens GN=GPR158 PE=1 S | 961 | DPAPQNsNPAE | 0.517 | 0.383 | -0.367 | 0.178 |
| sp\|Q5T848\|GP158_HUMAN Probable G-protein coupled receptor 158 OS=Homo sapiens GN=GPR158 PE=1 S | 973 | PRKPQKsGIMK | 0.196 | 0.079 | -0.482 | -0.069 |
| sp\|Q5T848\|GP158_HUMAN Probable G-protein coupled receptor 158 OS=Homo sapiens GN=GPR158 PE=1 S | 984 | QQRVNPtTANS | 0.134 | 0.024 | -0.941 | -0.261 |
| sp\|Q5T848\|GP158_HUMAN Probable G-protein coupled receptor 158 OS=Homo sapiens GN=GPR158 PE=1 S | 985 | QRVNPTtANSD | 0.129 | 0.125 | -0.603 | -0.116 |
| sp\|Q5T848\|GP158_HUMAN Probable G-protein coupled receptor 158 OS=Homo sapiens GN=GPR158 PE=1 S | 988 | NPTTANsDLNP | 0.238 | 0.022 | -1.106 | -0.282 |
| sp\|Q5T848\|GP158_HUMAN Probable G-protein coupled receptor 158 OS=Homo sapiens GN=GPR158 PE=1 S | 994 | SDLNPGtTQMK | 0.031 | -0.482 | -1.611 | -0.687 |
| sp\|Q5T848\|GP158_HUMAN Probable G-protein coupled receptor 158 OS=Homo sapiens GN=GPR158 PE=1 S | 995 | DLNPGTtQMKD | 0.175 | -0.14 | -1.198 | -0.388 |
| sp\|Q5T848\|GP158_HUMAN Probable G-protein coupled receptor 158 OS=Homo sapiens GN=GPR158 PE=1 S | 1015 | WEVYDLtPGPV | 0.056 | -0.444 | -1.57 | -0.653 |
| sp\|Q5T848\|GP158_HUMAN Probable G-protein coupled receptor 158 OS=Homo sapiens GN=GPR158 PE=1 S | 1021 | TPGPVPsESKV | 0.053 | -0.27 | -1.702 | -0.64 |
| sp\|Q5T848\|GP158_HUMAN Probable G-protein coupled receptor 158 OS=Homo sapiens GN=GPR158 PE=1 S | 1023 | GPVPSEsKVQK | 0.262 | -0.001 | -0.651 | -0.13 |
| sp\|Q5T848\|GP158_HUMAN Probable G-protein coupled receptor 158 OS=Homo sapiens GN=GPR158 PE=1 S | 1030 | KVQKHVsIVAS | 0.4 | 0.112 | -0.368 | 0.048 |
| sp\|Q5T848\|GP158_HUMAN Probable G-protein coupled receptor 158 OS=Homo sapiens GN=GPR158 PE=1 S | 1034 | HVSIVAsEMEK | 0.27 | 0.095 | -0.633 | -0.089 |
| sp\|Q5T848\|GP158_HUMAN Probable G-protein coupled receptor 158 OS=Homo sapiens GN=GPR158 PE=1 S | 1041 | EMEKNPtFSLK | 0.218 | 0.012 | -0.551 | -0.107 |
| sp\|Q5T848\|GP158_HUMAN Probable G-protein coupled receptor 158 OS=Homo sapiens GN=GPR158 PE=1 S | 1043 | EKNPTFsLKEK | 0.289 | 0.08 | -0.302 | 0.022 |
| sp\|Q5T848\|GP158_HUMAN Probable G-protein coupled receptor 158 OS=Homo sapiens GN=GPR158 PE=1 S | 1048 | FSLKEKsHHKP | 0.372 | 0.001 | -0.53 | -0.052 |
| sp\|Q5T848\|GP158_HUMAN Probable G-protein coupled receptor 158 OS=Homo sapiens GN=GPR158 PE=1 S | 1061 | AEVCQQsNQKR | 0.11 | -0.155 | -1.442 | -0.496 |
| sp\|Q5T848\|GP158_HUMAN Probable G-protein coupled receptor 158 OS=Homo sapiens GN=GPR158 PE=1 S | 1076 | EVCLWEsQGQS | 0.162 | -0.1 | -1.186 | -0.375 |
| sp\|Q5T848\|GP158_HUMAN Probable G-protein coupled receptor 158 OS=Homo sapiens GN=GPR158 PE=1 S | 1080 | WESQGQsILED | 0.109 | -0.2 | -0.932 | -0.341 |
| sp\|Q5T848\|GP158_HUMAN Probable G-protein coupled receptor 158 OS=Homo sapiens GN=GPR158 PE=1 S | 1090 | DEKLLIsKTPV | 0.236 | -0.165 | -1.221 | -0.383 |
| sp\|Q5T848\|GP158_HUMAN Probable G-protein coupled receptor 158 OS=Homo sapiens GN=GPR158 PE=1 S | 1092 | KLLISKtPVLP | 0.228 | -0.015 | -0.491 | -0.093 |
| sp\|Q5T848\|GP158_HUMAN Probable G-protein coupled receptor 158 OS=Homo sapiens GN=GPR158 PE=1 S | 1117 | NVCAGQsEELP | 0.087 | -0.089 | -1.085 | -0.362 |
| sp\|Q5T848\|GP158_HUMAN Probable G-protein coupled receptor 158 OS=Homo sapiens GN=GPR158 PE=1 S | 1127 | PPKAVAsKTEN | 0.462 | 0 | -0.518 | -0.019 |
| sp\|Q5T848\|GP158_HUMAN Probable G-protein coupled receptor 158 OS=Homo sapiens GN=GPR158 PE=1 S | 1129 | KAVASKtENEN | 0.557 | 0.279 | 0.063 | 0.3 |
| sp\|Q5T848\|GP158_HUMAN Probable G-protein coupled receptor 158 OS=Homo sapiens GN=GPR158 PE=1 S | 1144 | GHQEKKtSSSE | 0.056 | -0.154 | -1.633 | -0.577 |
| sp\|Q5T848\|GP158_HUMAN Probable G-protein coupled receptor 158 OS=Homo sapiens GN=GPR158 PE=1 S | 1145 | HQEKKTsSSEE | 0.132 | -0.125 | -0.835 | -0.276 |
| sp\|Q5T848\|GP158_HUMAN Probable G-protein coupled receptor 158 OS=Homo sapiens GN=GPR158 PE=1 S | 1146 | QEKKTSsSEEN | 0.155 | -0.035 | -1.001 | -0.294 |

| | | | | | | |
|---|---|---|---|---|---|---|
| sp\|Q5T848\|GP158_HUMAN Probable G-protein coupled receptor 158 OS=Homo sapiens GN=GPR158 PE=1 S | 1147 | EKKTSSsEENV | 0.452 | 0.305 | -0.395 | 0.121 |
| sp\|Q5T848\|GP158_HUMAN Probable G-protein coupled receptor 158 OS=Homo sapiens GN=GPR158 PE=1 S | 1154 | EENVRGsYNSS | 0.046 | -0.142 | -1.536 | -0.544 |
| sp\|Q5T848\|GP158_HUMAN Probable G-protein coupled receptor 158 OS=Homo sapiens GN=GPR158 PE=1 S | 1157 | VRGSYNsSNNF | 0.178 | 0.141 | -0.926 | -0.202 |
| sp\|Q5T848\|GP158_HUMAN Probable G-protein coupled receptor 158 OS=Homo sapiens GN=GPR158 PE=1 S | 1158 | RGSYNSsNNFQ | 0.107 | -0.217 | -1.051 | -0.387 |
| sp\|Q5T848\|GP158_HUMAN Probable G-protein coupled receptor 158 OS=Homo sapiens GN=GPR158 PE=1 S | 1166 | NFQQPLtSRAE | 0.173 | -0.138 | -0.591 | -0.185 |
| sp\|Q5T848\|GP158_HUMAN Probable G-protein coupled receptor 158 OS=Homo sapiens GN=GPR158 PE=1 S | 1167 | FQQPLTsRAEV | 0.109 | -0.139 | -1.271 | -0.434 |
| sp\|Q5T848\|GP158_HUMAN Probable G-protein coupled receptor 158 OS=Homo sapiens GN=GPR158 PE=1 S | 1178 | CPWEFEtPAQP | 0.027 | -0.458 | -1.909 | -0.78 |
| sp\|Q5T848\|GP158_HUMAN Probable G-protein coupled receptor 158 OS=Homo sapiens GN=GPR158 PE=1 S | 1187 | QPNAGRsVALP | 0.034 | -0.32 | -1.772 | -0.686 |
| sp\|Q5T848\|GP158_HUMAN Probable G-protein coupled receptor 158 OS=Homo sapiens GN=GPR158 PE=1 S | 1193 | SVALPAsSALS | 0.059 | -0.25 | -1.437 | -0.543 |
| sp\|Q5T848\|GP158_HUMAN Probable G-protein coupled receptor 158 OS=Homo sapiens GN=GPR158 PE=1 S | 1194 | VALPASsALSA | 0.066 | -0.235 | -1.383 | -0.517 |
| sp\|Q5T848\|GP158_HUMAN Probable G-protein coupled receptor 158 OS=Homo sapiens GN=GPR158 PE=1 S | 1197 | PASSALsANKI | 0.222 | -0.021 | -0.712 | -0.17 |
| sp\|Q5T848\|GP158_HUMAN Probable G-protein coupled receptor 158 OS=Homo sapiens GN=GPR158 PE=1 S | 1212 | KEEIWDsFKV- | 0.203 | -0.025 | -0.951 | -0.258 |
| sp\|Q6PRD1\|GP179_HUMAN Probable G-protein coupled receptor 179 OS=Homo sapiens GN=GPR179 PE=1 S | 327 | PGFYGAsPSGG | 0.07 | -0.407 | -1.202 | -0.513 |
| sp\|Q6PRD1\|GP179_HUMAN Probable G-protein coupled receptor 179 OS=Homo sapiens GN=GPR179 PE=1 S | 329 | FYGASPsGGLE | 0.376 | 0.169 | -0.289 | 0.085 |
| sp\|Q6PRD1\|GP179_HUMAN Probable G-protein coupled receptor 179 OS=Homo sapiens GN=GPR179 PE=1 S | 335 | SGGLEEsDFQT | 0.084 | -0.259 | -1.515 | -0.563 |
| sp\|Q6PRD1\|GP179_HUMAN Probable G-protein coupled receptor 179 OS=Homo sapiens GN=GPR179 PE=1 S | 339 | EESDFQtTGQF | 0.143 | -0.122 | -1.044 | -0.341 |
| sp\|Q6PRD1\|GP179_HUMAN Probable G-protein coupled receptor 179 OS=Homo sapiens GN=GPR179 PE=1 S | 340 | ESDFQTtGQFG | 0.07 | -0.242 | -1.283 | -0.485 |
| sp\|Q6PRD1\|GP179_HUMAN Probable G-protein coupled receptor 179 OS=Homo sapiens GN=GPR179 PE=1 S | 350 | GFPEGRsGRLL | 0.057 | -0.191 | -1.372 | -0.502 |
| sp\|Q6PRD1\|GP179_HUMAN Probable G-protein coupled receptor 179 OS=Homo sapiens GN=GPR179 PE=1 S | 364 | PCPEGCtSCMD | 0.078 | -0.271 | -1.673 | -0.622 |
| sp\|Q6PRD1\|GP179_HUMAN Probable G-protein coupled receptor 179 OS=Homo sapiens GN=GPR179 PE=1 S | 365 | CPEGCTsCMDA | 0.452 | 0.093 | -0.211 | 0.111 |
| sp\|Q6PRD1\|GP179_HUMAN Probable G-protein coupled receptor 179 OS=Homo sapiens GN=GPR179 PE=1 S | 370 | TSCMDAtPCLV | 0.072 | -0.539 | -1.747 | -0.738 |
| sp\|Q6PRD1\|GP179_HUMAN Probable G-protein coupled receptor 179 OS=Homo sapiens GN=GPR179 PE=1 S | 398 | MLAIFLsMLVS | 0.153 | 0.172 | -0.987 | -0.221 |
| sp\|Q6PRD1\|GP179_HUMAN Probable G-protein coupled receptor 179 OS=Homo sapiens GN=GPR179 PE=1 S | 402 | FLSMLVsYRCR | 0.356 | -0.019 | -0.549 | -0.071 |
| sp\|Q6PRD1\|GP179_HUMAN Probable G-protein coupled receptor 179 OS=Homo sapiens GN=GPR179 PE=1 S | 414 | NKRIWAsGVVL | 0.583 | 0.407 | -0.004 | 0.329 |
| sp\|Q6PRD1\|GP179_HUMAN Probable G-protein coupled receptor 179 OS=Homo sapiens GN=GPR179 PE=1 S | 421 | GVVLLEtVLFG | 0.071 | -0.173 | -1.623 | -0.575 |
| sp\|Q6PRD1\|GP179_HUMAN Probable G-protein coupled receptor 179 OS=Homo sapiens GN=GPR179 PE=1 S | 441 | ILYFKPsVFRC | 0.198 | -0.159 | -0.946 | -0.302 |
| sp\|Q6PRD1\|GP179_HUMAN Probable G-protein coupled receptor 179 OS=Homo sapiens GN=GPR179 PE=1 S | 462 | FAIVYGtIILK | 0.065 | -0.152 | -1.247 | -0.445 |
| sp\|Q6PRD1\|GP179_HUMAN Probable G-protein coupled receptor 179 OS=Homo sapiens GN=GPR179 PE=1 S | 476 | VLQLFLsRTAQ | 0.213 | -0.063 | -1.011 | -0.287 |
| sp\|Q6PRD1\|GP179_HUMAN Probable G-protein coupled receptor 179 OS=Homo sapiens GN=GPR179 PE=1 S | 478 | QLFLSRtAQRS | 0.367 | 0.217 | -0.679 | -0.032 |
| sp\|Q6PRD1\|GP179_HUMAN Probable G-protein coupled receptor 179 OS=Homo sapiens GN=GPR179 PE=1 S | 482 | SRTAQRsALLS | 0.061 | 0.073 | -1.592 | -0.486 |
| sp\|Q6PRD1\|GP179_HUMAN Probable G-protein coupled receptor 179 OS=Homo sapiens GN=GPR179 PE=1 S | 486 | QRSALLsSGRL | 0.123 | -0.012 | -1.113 | -0.334 |
| sp\|Q6PRD1\|GP179_HUMAN Probable G-protein coupled receptor 179 OS=Homo sapiens GN=GPR179 PE=1 S | 487 | RSALLSsGRLL | 0.082 | -0.231 | -1.185 | -0.445 |
| sp\|Q6PRD1\|GP179_HUMAN Probable G-protein coupled receptor 179 OS=Homo sapiens GN=GPR179 PE=1 S | 509 | GFLAVWtVGAL | 0.521 | 0.135 | -0.041 | 0.205 |
| sp\|Q6PRD1\|GP179_HUMAN Probable G-protein coupled receptor 179 OS=Homo sapiens GN=GPR179 PE=1 S | 528 | LVIRGHtPSGR | 0.223 | 0.536 | -0.299 | 0.153 |
| sp\|Q6PRD1\|GP179_HUMAN Probable G-protein coupled receptor 179 OS=Homo sapiens GN=GPR179 PE=1 S | 530 | IRGHTPsGRHF | 0.076 | -0.094 | -0.929 | -0.316 |
| sp\|Q6PRD1\|GP179_HUMAN Probable G-protein coupled receptor 179 OS=Homo sapiens GN=GPR179 PE=1 S | 558 | LLLCWGsFLCY | 0.368 | 0.14 | -0.531 | -0.008 |
| sp\|Q6PRD1\|GP179_HUMAN Probable G-protein coupled receptor 179 OS=Homo sapiens GN=GPR179 PE=1 S | 564 | SFLCYAtRAVL | 0.197 | -0.099 | -0.989 | -0.297 |
| sp\|Q6PRD1\|GP179_HUMAN Probable G-protein coupled receptor 179 OS=Homo sapiens GN=GPR179 PE=1 S | 569 | ATRAVLsAFHE | 0.275 | 0.022 | -0.843 | -0.182 |
| sp\|Q6PRD1\|GP179_HUMAN Probable G-protein coupled receptor 179 OS=Homo sapiens GN=GPR179 PE=1 S | 588 | HNELLLsAAFH | 0.03 | -0.385 | -1.891 | -0.749 |
| sp\|Q6PRD1\|GP179_HUMAN Probable G-protein coupled receptor 179 OS=Homo sapiens GN=GPR179 PE=1 S | 593 | LSAAFHtARFV | 0.187 | -0.123 | -1.111 | -0.349 |
| sp\|Q6PRD1\|GP179_HUMAN Probable G-protein coupled receptor 179 OS=Homo sapiens GN=GPR179 PE=1 S | 601 | RFVLVPsLHPD | 0.449 | 0.049 | -0.333 | 0.055 |
| sp\|Q6PRD1\|GP179_HUMAN Probable G-protein coupled receptor 179 OS=Homo sapiens GN=GPR179 PE=1 S | 607 | SLHPDWtLLLF | 0.128 | -0.109 | -0.991 | -0.324 |
| sp\|Q6PRD1\|GP179_HUMAN Probable G-protein coupled receptor 179 OS=Homo sapiens GN=GPR179 PE=1 S | 615 | LLFFFHtHSTV | 0.376 | 0.13 | -0.745 | -0.08 |
| sp\|Q6PRD1\|GP179_HUMAN Probable G-protein coupled receptor 179 OS=Homo sapiens GN=GPR179 PE=1 S | 617 | FFFHTHsTVTT | 0.192 | 0.131 | -0.63 | -0.102 |
| sp\|Q6PRD1\|GP179_HUMAN Probable G-protein coupled receptor 179 OS=Homo sapiens GN=GPR179 PE=1 S | 618 | FFHTHStVTTT | 0.154 | -0.084 | -0.961 | -0.297 |
| sp\|Q6PRD1\|GP179_HUMAN Probable G-protein coupled receptor 179 OS=Homo sapiens GN=GPR179 PE=1 S | 620 | HTHSTVtTTLA | 0.167 | -0.1 | -0.951 | -0.295 |
| sp\|Q6PRD1\|GP179_HUMAN Probable G-protein coupled receptor 179 OS=Homo sapiens GN=GPR179 PE=1 S | 621 | THSTVTtTLAL | 0.13 | -0.074 | -1.229 | -0.391 |
| sp\|Q6PRD1\|GP179_HUMAN Probable G-protein coupled receptor 179 OS=Homo sapiens GN=GPR179 PE=1 S | 622 | HSTVTTtLALI | 0.194 | 0.091 | -0.327 | -0.014 |
| sp\|Q6PRD1\|GP179_HUMAN Probable G-protein coupled receptor 179 OS=Homo sapiens GN=GPR179 PE=1 S | 656 | ELDLQHsGSYL | 0.114 | 0.019 | -1.134 | -0.334 |

| | | | | | | |
|---|---|---|---|---|---|---|
| sp\|Q6PRD1\|GP179_HUMAN Probable G-protein coupled receptor 179 OS=Homo sapiens GN=GPR179 PE=1 S | 658 | DLQHSGsYLGS | 0.25 | 0.074 | -0.705 | -0.127 |
| sp\|Q6PRD1\|GP179_HUMAN Probable G-protein coupled receptor 179 OS=Homo sapiens GN=GPR179 PE=1 S | 662 | SGSYLGsSIAS | 0.046 | -0.239 | -1.379 | -0.524 |
| sp\|Q6PRD1\|GP179_HUMAN Probable G-protein coupled receptor 179 OS=Homo sapiens GN=GPR179 PE=1 S | 663 | GSYLGSsIASA | 0.033 | -0.373 | -1.646 | -0.662 |
| sp\|Q6PRD1\|GP179_HUMAN Probable G-protein coupled receptor 179 OS=Homo sapiens GN=GPR179 PE=1 S | 666 | LGSSIAsAWSE | 0.146 | -0.114 | -0.951 | -0.306 |
| sp\|Q6PRD1\|GP179_HUMAN Probable G-protein coupled receptor 179 OS=Homo sapiens GN=GPR179 PE=1 S | 669 | SIASAWsEHSL | 0.207 | 0.052 | -0.761 | -0.167 |
| sp\|Q6PRD1\|GP179_HUMAN Probable G-protein coupled receptor 179 OS=Homo sapiens GN=GPR179 PE=1 S | 672 | SAWSEHsLDPG | 0.25 | 0.087 | -0.767 | -0.143 |
| sp\|Q6PRD1\|GP179_HUMAN Probable G-protein coupled receptor 179 OS=Homo sapiens GN=GPR179 PE=1 S | 694 | QLEVHKtKEMA | 0.296 | 0.015 | -0.391 | -0.027 |
| sp\|Q6PRD1\|GP179_HUMAN Probable G-protein coupled receptor 179 OS=Homo sapiens GN=GPR179 PE=1 S | 710 | LPKKRGsSCQG | 0.29 | -0.072 | -0.666 | -0.149 |
| sp\|Q6PRD1\|GP179_HUMAN Probable G-protein coupled receptor 179 OS=Homo sapiens GN=GPR179 PE=1 S | 711 | PKKRGSsCQGL | 0.717 | 0.937 | 0.654 | 0.769 |
| sp\|Q6PRD1\|GP179_HUMAN Probable G-protein coupled receptor 179 OS=Homo sapiens GN=GPR179 PE=1 S | 718 | CQGLGRsFMRY | 0.137 | -0.083 | -1.098 | -0.348 |
| sp\|Q6PRD1\|GP179_HUMAN Probable G-protein coupled receptor 179 OS=Homo sapiens GN=GPR179 PE=1 S | 735 | ALARQHsRDSG | 0.655 | 0.935 | 0.294 | 0.628 |
| sp\|Q6PRD1\|GP179_HUMAN Probable G-protein coupled receptor 179 OS=Homo sapiens GN=GPR179 PE=1 S | 738 | RQHSRDsGSPG | 0.095 | -0.212 | -1.275 | -0.464 |
| sp\|Q6PRD1\|GP179_HUMAN Probable G-protein coupled receptor 179 OS=Homo sapiens GN=GPR179 PE=1 S | 740 | HSRDSGsPGHG | 0.282 | 0.064 | -0.436 | -0.03 |
| sp\|Q6PRD1\|GP179_HUMAN Probable G-protein coupled receptor 179 OS=Homo sapiens GN=GPR179 PE=1 S | 745 | GSPGHGsLPGS | 0.543 | 0.378 | -0.062 | 0.286 |
| sp\|Q6PRD1\|GP179_HUMAN Probable G-protein coupled receptor 179 OS=Homo sapiens GN=GPR179 PE=1 S | 749 | HGSLPGsSRRR | 0.077 | -0.359 | -1.262 | -0.515 |
| sp\|Q6PRD1\|GP179_HUMAN Probable G-protein coupled receptor 179 OS=Homo sapiens GN=GPR179 PE=1 S | 750 | GSLPGSsRRRL | 0.07 | -0.43 | -1.48 | -0.613 |
| sp\|Q6PRD1\|GP179_HUMAN Probable G-protein coupled receptor 179 OS=Homo sapiens GN=GPR179 PE=1 S | 756 | SRRRLLsSSLQ | 0.372 | 1.028 | -0.243 | 0.386 |
| sp\|Q6PRD1\|GP179_HUMAN Probable G-protein coupled receptor 179 OS=Homo sapiens GN=GPR179 PE=1 S | 757 | RRRLLSsSLQE | 0.193 | 0.229 | -0.959 | -0.179 |
| sp\|Q6PRD1\|GP179_HUMAN Probable G-protein coupled receptor 179 OS=Homo sapiens GN=GPR179 PE=1 S | 758 | RRLLSSsLQEP | 0.534 | 0.447 | 0.052 | 0.344 |
| sp\|Q6PRD1\|GP179_HUMAN Probable G-protein coupled receptor 179 OS=Homo sapiens GN=GPR179 PE=1 S | 765 | LQEPEGtPALH | 0.019 | -0.709 | -2.038 | -0.909 |
| sp\|Q6PRD1\|GP179_HUMAN Probable G-protein coupled receptor 179 OS=Homo sapiens GN=GPR179 PE=1 S | 771 | TPALHKsRSTY | 0.192 | -0.133 | -0.935 | -0.292 |
| sp\|Q6PRD1\|GP179_HUMAN Probable G-protein coupled receptor 179 OS=Homo sapiens GN=GPR179 PE=1 S | 773 | ALHKSRsTYDQ | 0.482 | 0.252 | -0.363 | 0.124 |
| sp\|Q6PRD1\|GP179_HUMAN Probable G-protein coupled receptor 179 OS=Homo sapiens GN=GPR179 PE=1 S | 774 | LHKSRStYDQR | 0.224 | -0.018 | -0.731 | -0.175 |
| sp\|Q6PRD1\|GP179_HUMAN Probable G-protein coupled receptor 179 OS=Homo sapiens GN=GPR179 PE=1 S | 788 | DPPLLDsLLRR | 0.126 | -0.279 | -1.55 | -0.568 |
| sp\|Q6PRD1\|GP179_HUMAN Probable G-protein coupled receptor 179 OS=Homo sapiens GN=GPR179 PE=1 S | 799 | KLAKKAsRTES | 0.381 | -0.039 | -0.655 | -0.104 |
| sp\|Q6PRD1\|GP179_HUMAN Probable G-protein coupled receptor 179 OS=Homo sapiens GN=GPR179 PE=1 S | 801 | AKKASRtESRE | 0.448 | 0.116 | -0.479 | 0.028 |
| sp\|Q6PRD1\|GP179_HUMAN Probable G-protein coupled receptor 179 OS=Homo sapiens GN=GPR179 PE=1 S | 803 | KASRTEsRESV | 0.189 | 0.567 | -0.893 | -0.046 |
| sp\|Q6PRD1\|GP179_HUMAN Probable G-protein coupled receptor 179 OS=Homo sapiens GN=GPR179 PE=1 S | 806 | RTESREsVEGP | 0.216 | -0.091 | -0.937 | -0.271 |
| sp\|Q6PRD1\|GP179_HUMAN Probable G-protein coupled receptor 179 OS=Homo sapiens GN=GPR179 PE=1 S | 817 | PALGFRsASAH | 0.077 | -0.177 | -1.492 | -0.531 |
| sp\|Q6PRD1\|GP179_HUMAN Probable G-protein coupled receptor 179 OS=Homo sapiens GN=GPR179 PE=1 S | 819 | LGFRSAsAHNL | 0.859 | 1.235 | 0.837 | 0.977 |
| sp\|Q6PRD1\|GP179_HUMAN Probable G-protein coupled receptor 179 OS=Homo sapiens GN=GPR179 PE=1 S | 824 | ASAHNLtVGER | 0.226 | -0.11 | -0.558 | -0.147 |
| sp\|Q6PRD1\|GP179_HUMAN Probable G-protein coupled receptor 179 OS=Homo sapiens GN=GPR179 PE=1 S | 836 | PRARPAsLQKS | 0.78 | 1.102 | 0.892 | 0.925 |
| sp\|Q6PRD1\|GP179_HUMAN Probable G-protein coupled receptor 179 OS=Homo sapiens GN=GPR179 PE=1 S | 840 | PASLQKsLSVA | 0.243 | -0.004 | -0.596 | -0.119 |
| sp\|Q6PRD1\|GP179_HUMAN Probable G-protein coupled receptor 179 OS=Homo sapiens GN=GPR179 PE=1 S | 842 | SLQKSLsVASS | 0.221 | 0.178 | -0.627 | -0.076 |
| sp\|Q6PRD1\|GP179_HUMAN Probable G-protein coupled receptor 179 OS=Homo sapiens GN=GPR179 PE=1 S | 845 | KSLSVAsSREK | 0.182 | -0.292 | -1.093 | -0.401 |
| sp\|Q6PRD1\|GP179_HUMAN Probable G-protein coupled receptor 179 OS=Homo sapiens GN=GPR179 PE=1 S | 846 | SLSVASsREKA | 0.089 | -0.199 | -1.36 | -0.49 |
| sp\|Q6PRD1\|GP179_HUMAN Probable G-protein coupled receptor 179 OS=Homo sapiens GN=GPR179 PE=1 S | 855 | KALLMAsQAYL | 0.123 | -0.249 | -1.373 | -0.5 |
| sp\|Q6PRD1\|GP179_HUMAN Probable G-protein coupled receptor 179 OS=Homo sapiens GN=GPR179 PE=1 S | 862 | QAYLEEtYRQA | 0.08 | -0.356 | -1.406 | -0.561 |
| sp\|Q6PRD1\|GP179_HUMAN Probable G-protein coupled receptor 179 OS=Homo sapiens GN=GPR179 PE=1 S | 881 | AKAAMAsLVRR | 0.57 | -0.017 | -0.161 | 0.131 |
| sp\|Q6PRD1\|GP179_HUMAN Probable G-protein coupled receptor 179 OS=Homo sapiens GN=GPR179 PE=1 S | 887 | SLVRRPsARRL | 0.767 | 0.918 | 0.268 | 0.651 |
| sp\|Q6PRD1\|GP179_HUMAN Probable G-protein coupled receptor 179 OS=Homo sapiens GN=GPR179 PE=1 S | 900 | PRGAPLsAPPS | 0.305 | 0.481 | -0.378 | 0.136 |
| sp\|Q6PRD1\|GP179_HUMAN Probable G-protein coupled receptor 179 OS=Homo sapiens GN=GPR179 PE=1 S | 904 | PLSAPPsPAKS | 0.057 | -0.528 | -1.438 | -0.636 |
| sp\|Q6PRD1\|GP179_HUMAN Probable G-protein coupled receptor 179 OS=Homo sapiens GN=GPR179 PE=1 S | 908 | PPSPAKsSSVD | 0.226 | -0.049 | -0.636 | -0.153 |
| sp\|Q6PRD1\|GP179_HUMAN Probable G-protein coupled receptor 179 OS=Homo sapiens GN=GPR179 PE=1 S | 909 | PSPAKSsSVDS | 0.06 | -0.299 | -1.478 | -0.572 |
| sp\|Q6PRD1\|GP179_HUMAN Probable G-protein coupled receptor 179 OS=Homo sapiens GN=GPR179 PE=1 S | 910 | SPAKSSsVDSS | 0.181 | 0.053 | -0.712 | -0.159 |
| sp\|Q6PRD1\|GP179_HUMAN Probable G-protein coupled receptor 179 OS=Homo sapiens GN=GPR179 PE=1 S | 913 | KSSSVDsSHTS | 0.085 | -0.386 | -1.623 | -0.641 |
| sp\|Q6PRD1\|GP179_HUMAN Probable G-protein coupled receptor 179 OS=Homo sapiens GN=GPR179 PE=1 S | 914 | SSSVDSsHTSG | 0.054 | -0.375 | -1.735 | -0.685 |
| sp\|Q6PRD1\|GP179_HUMAN Probable G-protein coupled receptor 179 OS=Homo sapiens GN=GPR179 PE=1 S | 916 | SVDSSHtSGRL | 0.275 | 0.144 | -0.715 | -0.099 |
| sp\|Q6PRD1\|GP179_HUMAN Probable G-protein coupled receptor 179 OS=Homo sapiens GN=GPR179 PE=1 S | 917 | VDSSHTsGRLH | 0.068 | -0.421 | -1.454 | -0.602 |

| | | | | | | |
|---|---|---|---|---|---|---|
| sp\|Q6PRD1\|GP179_HUMAN Probable G-protein coupled receptor 179 OS=Homo sapiens GN=GPR179 PE=1 S | 938 | PIRHQVsTPIL | 0.854 | 0.808 | 0.597 | 0.753 |
| sp\|Q6PRD1\|GP179_HUMAN Probable G-protein coupled receptor 179 OS=Homo sapiens GN=GPR179 PE=1 S | 939 | IRHQVStPILA | 0.077 | -0.228 | -1.081 | -0.411 |
| sp\|Q6PRD1\|GP179_HUMAN Probable G-protein coupled receptor 179 OS=Homo sapiens GN=GPR179 PE=1 S | 945 | TPILALsGGLG | 0.154 | -0.037 | -0.941 | -0.275 |
| sp\|Q6PRD1\|GP179_HUMAN Probable G-protein coupled receptor 179 OS=Homo sapiens GN=GPR179 PE=1 S | 955 | GEPRMLsPTST | 0.166 | 0.292 | -0.85 | -0.131 |
| sp\|Q6PRD1\|GP179_HUMAN Probable G-protein coupled receptor 179 OS=Homo sapiens GN=GPR179 PE=1 S | 957 | PRMLSPtSTLA | 0.408 | 0.347 | -0.269 | 0.162 |
| sp\|Q6PRD1\|GP179_HUMAN Probable G-protein coupled receptor 179 OS=Homo sapiens GN=GPR179 PE=1 S | 958 | RMLSPTsTLAP | 0.075 | -0.076 | -1.078 | -0.36 |
| sp\|Q6PRD1\|GP179_HUMAN Probable G-protein coupled receptor 179 OS=Homo sapiens GN=GPR179 PE=1 S | 959 | MLSPTStLAPA | 0.198 | -0.017 | -0.53 | -0.116 |
| sp\|Q6PRD1\|GP179_HUMAN Probable G-protein coupled receptor 179 OS=Homo sapiens GN=GPR179 PE=1 S | 971 | LPALAPtPAPA | 0.064 | -0.505 | -1.327 | -0.589 |
| sp\|Q6PRD1\|GP179_HUMAN Probable G-protein coupled receptor 179 OS=Homo sapiens GN=GPR179 PE=1 S | 982 | LAPVPVsPQSP | 0.022 | -0.647 | -1.822 | -0.816 |
| sp\|Q6PRD1\|GP179_HUMAN Probable G-protein coupled receptor 179 OS=Homo sapiens GN=GPR179 PE=1 S | 985 | VPVSPQsPNLL | 0.051 | -0.45 | -1.318 | -0.572 |
| sp\|Q6PRD1\|GP179_HUMAN Probable G-protein coupled receptor 179 OS=Homo sapiens GN=GPR179 PE=1 S | 990 | QSPNLLtYICP | 0.023 | -0.348 | -1.711 | -0.679 |
| sp\|Q6PRD1\|GP179_HUMAN Probable G-protein coupled receptor 179 OS=Homo sapiens GN=GPR179 PE=1 S | 1013 | VPQEGPsGPER | 0.252 | 0.262 | -0.537 | -0.008 |
| sp\|Q6PRD1\|GP179_HUMAN Probable G-protein coupled receptor 179 OS=Homo sapiens GN=GPR179 PE=1 S | 1021 | PERGHHsPAPA | 0.245 | -0.037 | -0.763 | -0.185 |
| sp\|Q6PRD1\|GP179_HUMAN Probable G-protein coupled receptor 179 OS=Homo sapiens GN=GPR179 PE=1 S | 1034 | RLWRALsVAVE | 0.674 | 1.08 | 0.433 | 0.729 |
| sp\|Q6PRD1\|GP179_HUMAN Probable G-protein coupled receptor 179 OS=Homo sapiens GN=GPR179 PE=1 S | 1040 | SVAVEKsRAGE | 0.064 | -0.324 | -1.363 | -0.541 |
| sp\|Q6PRD1\|GP179_HUMAN Probable G-protein coupled receptor 179 OS=Homo sapiens GN=GPR179 PE=1 S | 1072 | PKIFPKsHSLK | 0.374 | 0.012 | -0.317 | 0.023 |
| sp\|Q6PRD1\|GP179_HUMAN Probable G-protein coupled receptor 179 OS=Homo sapiens GN=GPR179 PE=1 S | 1074 | IFPKSHsLKAP | 0.742 | 0.42 | 0.628 | 0.597 |
| sp\|Q6PRD1\|GP179_HUMAN Probable G-protein coupled receptor 179 OS=Homo sapiens GN=GPR179 PE=1 S | 1083 | APVQQGsMRSL | 0.054 | -0.167 | -1.542 | -0.552 |
| sp\|Q6PRD1\|GP179_HUMAN Probable G-protein coupled receptor 179 OS=Homo sapiens GN=GPR179 PE=1 S | 1086 | QQGSMRsLGLA | 0.18 | -0.088 | -0.632 | -0.18 |
| sp\|Q6PRD1\|GP179_HUMAN Probable G-protein coupled receptor 179 OS=Homo sapiens GN=GPR179 PE=1 S | 1095 | LAIKALtRSRS | 0.116 | -0.233 | -1.351 | -0.489 |
| sp\|Q6PRD1\|GP179_HUMAN Probable G-protein coupled receptor 179 OS=Homo sapiens GN=GPR179 PE=1 S | 1097 | IKALTRsRSTY | 0.167 | -0.089 | -1.025 | -0.316 |
| sp\|Q6PRD1\|GP179_HUMAN Probable G-protein coupled receptor 179 OS=Homo sapiens GN=GPR179 PE=1 S | 1099 | ALTRSRsTYRE | 0.771 | 0.996 | 0.455 | 0.741 |
| sp\|Q6PRD1\|GP179_HUMAN Probable G-protein coupled receptor 179 OS=Homo sapiens GN=GPR179 PE=1 S | 1100 | LTRSRStYREK | 0.374 | 0.071 | -0.524 | -0.026 |
| sp\|Q6PRD1\|GP179_HUMAN Probable G-protein coupled receptor 179 OS=Homo sapiens GN=GPR179 PE=1 S | 1106 | TYREKEsVEES | 0.181 | 0.102 | -1.011 | -0.243 |
| sp\|Q6PRD1\|GP179_HUMAN Probable G-protein coupled receptor 179 OS=Homo sapiens GN=GPR179 PE=1 S | 1110 | KESVEEsPEGQ | 0.028 | -0.641 | -2.201 | -0.938 |
| sp\|Q6PRD1\|GP179_HUMAN Probable G-protein coupled receptor 179 OS=Homo sapiens GN=GPR179 PE=1 S | 1116 | SPEGQNsGTAG | 0.151 | -0.111 | -1.101 | -0.354 |
| sp\|Q6PRD1\|GP179_HUMAN Probable G-protein coupled receptor 179 OS=Homo sapiens GN=GPR179 PE=1 S | 1118 | EGQNSGtAGES | 0.274 | 0.202 | -0.407 | 0.023 |
| sp\|Q6PRD1\|GP179_HUMAN Probable G-protein coupled receptor 179 OS=Homo sapiens GN=GPR179 PE=1 S | 1122 | SGTAGEsMGAP | 0.092 | -0.097 | -1.143 | -0.383 |
| sp\|Q6PRD1\|GP179_HUMAN Probable G-protein coupled receptor 179 OS=Homo sapiens GN=GPR179 PE=1 S | 1127 | ESMGAPsRSPR | 0.078 | -0.263 | -1.169 | -0.451 |
| sp\|Q6PRD1\|GP179_HUMAN Probable G-protein coupled receptor 179 OS=Homo sapiens GN=GPR179 PE=1 S | 1129 | MGAPSRsPRLG | 0.176 | -0.149 | -0.676 | -0.216 |
| sp\|Q6PRD1\|GP179_HUMAN Probable G-protein coupled receptor 179 OS=Homo sapiens GN=GPR179 PE=1 S | 1139 | GRPKAVsKQAA | 0.73 | 0.37 | 0.301 | 0.467 |
| sp\|Q6PRD1\|GP179_HUMAN Probable G-protein coupled receptor 179 OS=Homo sapiens GN=GPR179 PE=1 S | 1147 | QAALIPsDDKE | 0.151 | -0.158 | -0.919 | -0.309 |
| sp\|Q6PRD1\|GP179_HUMAN Probable G-protein coupled receptor 179 OS=Homo sapiens GN=GPR179 PE=1 S | 1152 | PSDDKEsLQNQ | 0.198 | -0.098 | -1.107 | -0.336 |
| sp\|Q6PRD1\|GP179_HUMAN Probable G-protein coupled receptor 179 OS=Homo sapiens GN=GPR179 PE=1 S | 1161 | NQQNAHtSRML | 0.255 | 0.087 | -0.449 | -0.036 |
| sp\|Q6PRD1\|GP179_HUMAN Probable G-protein coupled receptor 179 OS=Homo sapiens GN=GPR179 PE=1 S | 1162 | QQNAHTsRMLQ | 0.124 | -0.183 | -1.135 | -0.398 |
| sp\|Q6PRD1\|GP179_HUMAN Probable G-protein coupled receptor 179 OS=Homo sapiens GN=GPR179 PE=1 S | 1173 | VCQREGsREQE | 0.46 | 0.631 | -0.393 | 0.233 |
| sp\|Q6PRD1\|GP179_HUMAN Probable G-protein coupled receptor 179 OS=Homo sapiens GN=GPR179 PE=1 S | 1184 | DRGRRMtQGLG | 0.887 | 1.34 | 0.757 | 0.995 |
| sp\|Q6PRD1\|GP179_HUMAN Probable G-protein coupled receptor 179 OS=Homo sapiens GN=GPR179 PE=1 S | 1198 | AERAGKtGLAM | 0.255 | 0.088 | -0.512 | -0.056 |
| sp\|Q6PRD1\|GP179_HUMAN Probable G-protein coupled receptor 179 OS=Homo sapiens GN=GPR179 PE=1 S | 1207 | AMLRQVsRDKN | 0.546 | 0.852 | -0.017 | 0.46 |
| sp\|Q6PRD1\|GP179_HUMAN Probable G-protein coupled receptor 179 OS=Homo sapiens GN=GPR179 PE=1 S | 1215 | DKNIKQsKETP | 0.143 | -0.12 | -1.054 | -0.344 |
| sp\|Q6PRD1\|GP179_HUMAN Probable G-protein coupled receptor 179 OS=Homo sapiens GN=GPR179 PE=1 S | 1218 | IKQSKEtPVGW | 0.09 | -0.391 | -1.272 | -0.524 |
| sp\|Q6PRD1\|GP179_HUMAN Probable G-protein coupled receptor 179 OS=Homo sapiens GN=GPR179 PE=1 S | 1232 | PKAGLQsLGSA | 0.38 | 0.105 | -0.061 | 0.141 |
| sp\|Q6PRD1\|GP179_HUMAN Probable G-protein coupled receptor 179 OS=Homo sapiens GN=GPR179 PE=1 S | 1235 | GLQSLGsADHR | 0.216 | 0.002 | -0.871 | -0.218 |
| sp\|Q6PRD1\|GP179_HUMAN Probable G-protein coupled receptor 179 OS=Homo sapiens GN=GPR179 PE=1 S | 1249 | VCPWEVtESET | 0.24 | -0.158 | -0.976 | -0.298 |
| sp\|Q6PRD1\|GP179_HUMAN Probable G-protein coupled receptor 179 OS=Homo sapiens GN=GPR179 PE=1 S | 1251 | PWEVTEsETRQ | 0.131 | -0.099 | -1.277 | -0.415 |
| sp\|Q6PRD1\|GP179_HUMAN Probable G-protein coupled receptor 179 OS=Homo sapiens GN=GPR179 PE=1 S | 1253 | EVTESEtRQPD | 0.149 | 0.006 | -0.755 | -0.2 |
| sp\|Q6PRD1\|GP179_HUMAN Probable G-protein coupled receptor 179 OS=Homo sapiens GN=GPR179 PE=1 S | 1258 | ETRQPDsGNKA | 0.242 | -0.059 | -0.558 | -0.125 |
| sp\|Q6PRD1\|GP179_HUMAN Probable G-protein coupled receptor 179 OS=Homo sapiens GN=GPR179 PE=1 S | 1269 | EICPWEtSEGA | 0.123 | -0.143 | -1.01 | -0.343 |
| sp\|Q6PRD1\|GP179_HUMAN Probable G-protein coupled receptor 179 OS=Homo sapiens GN=GPR179 PE=1 S | 1270 | ICPWETsEGAP | 0.358 | 0.017 | -0.495 | -0.04 |

| | | | | | | |
|---|---|---|---|---|---|---|
| sp\|Q6PRD1\|GP179_HUMAN Probable G-protein coupled receptor 179 OS=Homo sapiens GN=GPR179 PE=1 S | 1276 | SEGAPEsRALR | 0.045 | -0.429 | -1.834 | -0.739 |
| sp\|Q6PRD1\|GP179_HUMAN Probable G-protein coupled receptor 179 OS=Homo sapiens GN=GPR179 PE=1 S | 1286 | RQDPGDsQKKR | 0.05 | -0.348 | -1.619 | -0.639 |
| sp\|Q6PRD1\|GP179_HUMAN Probable G-protein coupled receptor 179 OS=Homo sapiens GN=GPR179 PE=1 S | 1297 | GEARGKsEPID | 0.933 | 1.457 | 1.5 | 1.297 |
| sp\|Q6PRD1\|GP179_HUMAN Probable G-protein coupled receptor 179 OS=Homo sapiens GN=GPR179 PE=1 S | 1325 | AVCPWEsADRG | 0.212 | -0.11 | -1.134 | -0.344 |
| sp\|Q6PRD1\|GP179_HUMAN Probable G-protein coupled receptor 179 OS=Homo sapiens GN=GPR179 PE=1 S | 1332 | ADRGGLsPGSA | 0.039 | -0.35 | -1.453 | -0.588 |
| sp\|Q6PRD1\|GP179_HUMAN Probable G-protein coupled receptor 179 OS=Homo sapiens GN=GPR179 PE=1 S | 1335 | GGLSPGsAPQD | 0.496 | 0.302 | -0.114 | 0.228 |
| sp\|Q6PRD1\|GP179_HUMAN Probable G-protein coupled receptor 179 OS=Homo sapiens GN=GPR179 PE=1 S | 1347 | GRIRDKsEAGD | 0.823 | 1.164 | 0.912 | 0.966 |
| sp\|Q6PRD1\|GP179_HUMAN Probable G-protein coupled receptor 179 OS=Homo sapiens GN=GPR179 PE=1 S | 1352 | KSEAGDsVEAR | 0.128 | -0.246 | -1.059 | -0.392 |
| sp\|Q6PRD1\|GP179_HUMAN Probable G-protein coupled receptor 179 OS=Homo sapiens GN=GPR179 PE=1 S | 1372 | AGPEAHtPDIT | 0.11 | -0.275 | -1.164 | -0.443 |
| sp\|Q6PRD1\|GP179_HUMAN Probable G-protein coupled receptor 179 OS=Homo sapiens GN=GPR179 PE=1 S | 1376 | AHTPDItKAEP | 0.094 | -0.283 | -1.351 | -0.513 |
| sp\|Q6PRD1\|GP179_HUMAN Probable G-protein coupled receptor 179 OS=Homo sapiens GN=GPR179 PE=1 S | 1386 | PCPWEAsEGGE | 0.43 | -0.008 | -0.592 | -0.057 |
| sp\|Q6PRD1\|GP179_HUMAN Probable G-protein coupled receptor 179 OS=Homo sapiens GN=GPR179 PE=1 S | 1409 | PQEKQKtRKAT | 0.257 | -0.024 | -0.336 | -0.034 |
| sp\|Q6PRD1\|GP179_HUMAN Probable G-protein coupled receptor 179 OS=Homo sapiens GN=GPR179 PE=1 S | 1413 | QKTRKAtFWKE | 0.786 | 0.955 | 0.675 | 0.805 |
| sp\|Q6PRD1\|GP179_HUMAN Probable G-protein coupled receptor 179 OS=Homo sapiens GN=GPR179 PE=1 S | 1426 | PGGDLEsLCPW | 0.102 | -0.184 | -1.228 | -0.437 |
| sp\|Q6PRD1\|GP179_HUMAN Probable G-protein coupled receptor 179 OS=Homo sapiens GN=GPR179 PE=1 S | 1432 | SLCPWEsTDFR | 0.176 | -0.093 | -1.005 | -0.307 |
| sp\|Q6PRD1\|GP179_HUMAN Probable G-protein coupled receptor 179 OS=Homo sapiens GN=GPR179 PE=1 S | 1433 | LCPWEStDFRG | 0.177 | -0.214 | -1.27 | -0.436 |
| sp\|Q6PRD1\|GP179_HUMAN Probable G-protein coupled receptor 179 OS=Homo sapiens GN=GPR179 PE=1 S | 1439 | TDFRGPsAVSI | 0.244 | 0.654 | -0.347 | 0.184 |
| sp\|Q6PRD1\|GP179_HUMAN Probable G-protein coupled receptor 179 OS=Homo sapiens GN=GPR179 PE=1 S | 1442 | RGPSAVsIQAP | 0.245 | -0.072 | -0.556 | -0.128 |
| sp\|Q6PRD1\|GP179_HUMAN Probable G-protein coupled receptor 179 OS=Homo sapiens GN=GPR179 PE=1 S | 1448 | SIQAPGsSECS | 0.055 | -0.191 | -1.549 | -0.562 |
| sp\|Q6PRD1\|GP179_HUMAN Probable G-protein coupled receptor 179 OS=Homo sapiens GN=GPR179 PE=1 S | 1449 | IQAPGSsECSG | 0.041 | -0.315 | -1.257 | -0.51 |
| sp\|Q6PRD1\|GP179_HUMAN Probable G-protein coupled receptor 179 OS=Homo sapiens GN=GPR179 PE=1 S | 1452 | PGSSECsGSLG | 0.123 | -0.242 | -1.278 | -0.466 |
| sp\|Q6PRD1\|GP179_HUMAN Probable G-protein coupled receptor 179 OS=Homo sapiens GN=GPR179 PE=1 S | 1454 | SSECSGsLGSG | 0.335 | 0.065 | -0.464 | -0.021 |
| sp\|Q6PRD1\|GP179_HUMAN Probable G-protein coupled receptor 179 OS=Homo sapiens GN=GPR179 PE=1 S | 1457 | CSGSLGsGIAE | 0.057 | -0.296 | -1.284 | -0.508 |
| sp\|Q6PRD1\|GP179_HUMAN Probable G-protein coupled receptor 179 OS=Homo sapiens GN=GPR179 PE=1 S | 1494 | MGQEMLsLGTG | 0.338 | 0.088 | -0.29 | 0.045 |
| sp\|Q6PRD1\|GP179_HUMAN Probable G-protein coupled receptor 179 OS=Homo sapiens GN=GPR179 PE=1 S | 1497 | EMLSLGtGRES | 0.042 | -0.257 | -1.554 | -0.59 |
| sp\|Q6PRD1\|GP179_HUMAN Probable G-protein coupled receptor 179 OS=Homo sapiens GN=GPR179 PE=1 S | 1501 | LGTGREsLQEK | 0.476 | 0.129 | -0.039 | 0.189 |
| sp\|Q6PRD1\|GP179_HUMAN Probable G-protein coupled receptor 179 OS=Homo sapiens GN=GPR179 PE=1 S | 1509 | QEKEKAsRKGS | 0.079 | -0.264 | -1.357 | -0.514 |
| sp\|Q6PRD1\|GP179_HUMAN Probable G-protein coupled receptor 179 OS=Homo sapiens GN=GPR179 PE=1 S | 1513 | KASRKGsFGEM | 0.564 | 0.727 | 0.16 | 0.484 |
| sp\|Q6PRD1\|GP179_HUMAN Probable G-protein coupled receptor 179 OS=Homo sapiens GN=GPR179 PE=1 S | 1521 | GEMGEQtVKAV | 0.196 | 0.057 | -0.912 | -0.22 |
| sp\|Q6PRD1\|GP179_HUMAN Probable G-protein coupled receptor 179 OS=Homo sapiens GN=GPR179 PE=1 S | 1529 | KAVQKLsQQQE | 0.075 | -0.318 | -1.42 | -0.554 |
| sp\|Q6PRD1\|GP179_HUMAN Probable G-protein coupled receptor 179 OS=Homo sapiens GN=GPR179 PE=1 S | 1534 | LSQQQEsVCPR | 0.095 | -0.235 | -1.177 | -0.439 |
| sp\|Q6PRD1\|GP179_HUMAN Probable G-protein coupled receptor 179 OS=Homo sapiens GN=GPR179 PE=1 S | 1540 | SVCPREsTVPG | 0.117 | -0.106 | -1.25 | -0.413 |
| sp\|Q6PRD1\|GP179_HUMAN Probable G-protein coupled receptor 179 OS=Homo sapiens GN=GPR179 PE=1 S | 1541 | VCPREStVPGH | 0.693 | 1.035 | 0.205 | 0.644 |
| sp\|Q6PRD1\|GP179_HUMAN Probable G-protein coupled receptor 179 OS=Homo sapiens GN=GPR179 PE=1 S | 1546 | STVPGHsSPCL | 0.314 | 0.435 | -0.57 | 0.06 |
| sp\|Q6PRD1\|GP179_HUMAN Probable G-protein coupled receptor 179 OS=Homo sapiens GN=GPR179 PE=1 S | 1547 | TVPGHSsPCLD | 0.031 | -0.49 | -1.689 | -0.716 |
| sp\|Q6PRD1\|GP179_HUMAN Probable G-protein coupled receptor 179 OS=Homo sapiens GN=GPR179 PE=1 S | 1553 | SPCLDNsSSKA | 0.072 | -0.275 | -1.714 | -0.639 |
| sp\|Q6PRD1\|GP179_HUMAN Probable G-protein coupled receptor 179 OS=Homo sapiens GN=GPR179 PE=1 S | 1554 | PCLDNSsSKAG | 0.223 | -0.013 | -0.744 | -0.178 |
| sp\|Q6PRD1\|GP179_HUMAN Probable G-protein coupled receptor 179 OS=Homo sapiens GN=GPR179 PE=1 S | 1555 | CLDNSSsKAGS | 0.242 | 0.075 | -0.298 | 0.006 |
| sp\|Q6PRD1\|GP179_HUMAN Probable G-protein coupled receptor 179 OS=Homo sapiens GN=GPR179 PE=1 S | 1559 | SSSKAGsQFLC | 0.144 | -0.239 | -1.415 | -0.503 |
| sp\|Q6PRD1\|GP179_HUMAN Probable G-protein coupled receptor 179 OS=Homo sapiens GN=GPR179 PE=1 S | 1567 | FLCNGGsRATQ | 0.081 | -0.224 | -1.339 | -0.494 |
| sp\|Q6PRD1\|GP179_HUMAN Probable G-protein coupled receptor 179 OS=Homo sapiens GN=GPR179 PE=1 S | 1570 | NGGSRAtQVCP | 0.146 | -0.018 | -1.137 | -0.336 |
| sp\|Q6PRD1\|GP179_HUMAN Probable G-protein coupled receptor 179 OS=Homo sapiens GN=GPR179 PE=1 S | 1586 | PEAQEAtPAKT | 0.076 | -0.466 | -1.44 | -0.61 |
| sp\|Q6PRD1\|GP179_HUMAN Probable G-protein coupled receptor 179 OS=Homo sapiens GN=GPR179 PE=1 S | 1590 | EATPAKtEICP | 0.185 | 0.054 | -0.446 | -0.069 |
| sp\|Q6PRD1\|GP179_HUMAN Probable G-protein coupled receptor 179 OS=Homo sapiens GN=GPR179 PE=1 S | 1601 | WEVNERtREEW | 0.059 | -0.223 | -1.563 | -0.576 |
| sp\|Q6PRD1\|GP179_HUMAN Probable G-protein coupled receptor 179 OS=Homo sapiens GN=GPR179 PE=1 S | 1606 | RTREEWtSAQV | 0.272 | 0.01 | -1.155 | -0.291 |
| sp\|Q6PRD1\|GP179_HUMAN Probable G-protein coupled receptor 179 OS=Homo sapiens GN=GPR179 PE=1 S | 1607 | TREEWTsAQVP | 0.157 | 0.18 | -0.829 | -0.164 |
| sp\|Q6PRD1\|GP179_HUMAN Probable G-protein coupled receptor 179 OS=Homo sapiens GN=GPR179 PE=1 S | 1616 | VPRGGEsQKDK | 0.176 | -0.05 | -1.047 | -0.307 |
| sp\|Q6PRD1\|GP179_HUMAN Probable G-protein coupled receptor 179 OS=Homo sapiens GN=GPR179 PE=1 S | 1627 | EKMPGKsEIED | 0.307 | 0.133 | -0.105 | 0.112 |
| sp\|Q6PRD1\|GP179_HUMAN Probable G-protein coupled receptor 179 OS=Homo sapiens GN=GPR179 PE=1 S | 1633 | SEIEDVtAWEK | 0.186 | -0.09 | -1.289 | -0.398 |

| | | | | | | | |
|---|---|---|---|---|---|---|---|
| sp\|Q6PRD1\|GP179_HUMAN Probable G-protein coupled receptor 179 OS=Homo sapiens GN=GPR179 PE=1 S | 1653 | AVGPWEsVDPG | 0.112 | -0.148 | -1.063 | -0.366 |
| sp\|Q6PRD1\|GP179_HUMAN Probable G-protein coupled receptor 179 OS=Homo sapiens GN=GPR179 PE=1 S | 1658 | ESVDPGsFSPQ | 0.046 | -0.367 | -1.528 | -0.616 |
| sp\|Q6PRD1\|GP179_HUMAN Probable G-protein coupled receptor 179 OS=Homo sapiens GN=GPR179 PE=1 S | 1660 | VDPGSFsPQPR | 0.069 | -0.294 | -1.01 | -0.412 |
| sp\|Q6PRD1\|GP179_HUMAN Probable G-protein coupled receptor 179 OS=Homo sapiens GN=GPR179 PE=1 S | 1668 | QPRPQDtERPQ | 0.156 | -0.086 | -1.095 | -0.342 |
| sp\|Q6PRD1\|GP179_HUMAN Probable G-protein coupled receptor 179 OS=Homo sapiens GN=GPR179 PE=1 S | 1673 | DTERPQtLLQM | 0.727 | 0.755 | 0.53 | 0.671 |
| sp\|Q6PRD1\|GP179_HUMAN Probable G-protein coupled receptor 179 OS=Homo sapiens GN=GPR179 PE=1 S | 1678 | QTLLQMsGSVG | 0.115 | -0.043 | -1.138 | -0.355 |
| sp\|Q6PRD1\|GP179_HUMAN Probable G-protein coupled receptor 179 OS=Homo sapiens GN=GPR179 PE=1 S | 1680 | LLQMSGsVGSK | 0.356 | 0.176 | -0.228 | 0.101 |
| sp\|Q6PRD1\|GP179_HUMAN Probable G-protein coupled receptor 179 OS=Homo sapiens GN=GPR179 PE=1 S | 1683 | MSGSVGsKAAD | 0.148 | -0.149 | -0.946 | -0.316 |
| sp\|Q6PRD1\|GP179_HUMAN Probable G-protein coupled receptor 179 OS=Homo sapiens GN=GPR179 PE=1 S | 1698 | DVEENLtAGKA | 0.14 | -0.092 | -1.066 | -0.339 |
| sp\|Q6PRD1\|GP179_HUMAN Probable G-protein coupled receptor 179 OS=Homo sapiens GN=GPR179 PE=1 S | 1727 | AEAIRKsPNDT | 0.294 | -0.118 | -0.478 | -0.101 |
| sp\|Q6PRD1\|GP179_HUMAN Probable G-protein coupled receptor 179 OS=Homo sapiens GN=GPR179 PE=1 S | 1731 | RKSPNDtGKVS | 0.185 | -0.159 | -0.875 | -0.283 |
| sp\|Q6PRD1\|GP179_HUMAN Probable G-protein coupled receptor 179 OS=Homo sapiens GN=GPR179 PE=1 S | 1735 | NDTGKVsADLG | 0.195 | -0.063 | -0.748 | -0.205 |
| sp\|Q6PRD1\|GP179_HUMAN Probable G-protein coupled receptor 179 OS=Homo sapiens GN=GPR179 PE=1 S | 1746 | PRERAVtAPEK | 0.967 | 1.675 | 1.736 | 1.459 |
| sp\|Q6PRD1\|GP179_HUMAN Probable G-protein coupled receptor 179 OS=Homo sapiens GN=GPR179 PE=1 S | 1755 | EKPQKPtPEWE | 0.053 | -0.466 | -1.32 | -0.578 |
| sp\|Q6PRD1\|GP179_HUMAN Probable G-protein coupled receptor 179 OS=Homo sapiens GN=GPR179 PE=1 S | 1766 | VACPWGsVGPG | 0.157 | -0.192 | -0.976 | -0.337 |
| sp\|Q6PRD1\|GP179_HUMAN Probable G-protein coupled receptor 179 OS=Homo sapiens GN=GPR179 PE=1 S | 1773 | VGPGACsQHPG | 0.186 | -0.149 | -1.108 | -0.357 |
| sp\|Q6PRD1\|GP179_HUMAN Probable G-protein coupled receptor 179 OS=Homo sapiens GN=GPR179 PE=1 S | 1778 | CSQHPGtLDAD | 0.416 | 0.044 | -0.006 | 0.151 |
| sp\|Q6PRD1\|GP179_HUMAN Probable G-protein coupled receptor 179 OS=Homo sapiens GN=GPR179 PE=1 S | 1811 | EAQEAAtSEKA | 0.119 | -0.058 | -1.099 | -0.346 |
| sp\|Q6PRD1\|GP179_HUMAN Probable G-protein coupled receptor 179 OS=Homo sapiens GN=GPR179 PE=1 S | 1812 | AQEAATsEKAK | 0.312 | 0.016 | -0.26 | 0.023 |
| sp\|Q6PRD1\|GP179_HUMAN Probable G-protein coupled receptor 179 OS=Homo sapiens GN=GPR179 PE=1 S | 1823 | ICPWEVsEGTT | 0.467 | -0.059 | -0.474 | -0.022 |
| sp\|Q6PRD1\|GP179_HUMAN Probable G-protein coupled receptor 179 OS=Homo sapiens GN=GPR179 PE=1 S | 1826 | WEVSEGtTGKG | 0.084 | -0.27 | -1.478 | -0.555 |
| sp\|Q6PRD1\|GP179_HUMAN Probable G-protein coupled receptor 179 OS=Homo sapiens GN=GPR179 PE=1 S | 1827 | EVSEGTtGKGL | 0.035 | -0.247 | -1.231 | -0.481 |
| sp\|Q6PRD1\|GP179_HUMAN Probable G-protein coupled receptor 179 OS=Homo sapiens GN=GPR179 PE=1 S | 1837 | LDQKAGsESAE | 0.217 | 0.018 | -0.668 | -0.144 |
| sp\|Q6PRD1\|GP179_HUMAN Probable G-protein coupled receptor 179 OS=Homo sapiens GN=GPR179 PE=1 S | 1839 | QKAGSEsAEQR | 0.433 | 0.206 | -0.153 | 0.162 |
| sp\|Q6PRD1\|GP179_HUMAN Probable G-protein coupled receptor 179 OS=Homo sapiens GN=GPR179 PE=1 S | 1853 | LEKGRLtSLGE | 0.123 | -0.081 | -0.956 | -0.305 |
| sp\|Q6PRD1\|GP179_HUMAN Probable G-protein coupled receptor 179 OS=Homo sapiens GN=GPR179 PE=1 S | 1854 | EKGRLTsLGED | 0.824 | 1.035 | 1.022 | 0.96 |
| sp\|Q6PRD1\|GP179_HUMAN Probable G-protein coupled receptor 179 OS=Homo sapiens GN=GPR179 PE=1 S | 1860 | SLGEDVsKGMA | 0.195 | -0.118 | -0.868 | -0.264 |
| sp\|Q6PRD1\|GP179_HUMAN Probable G-protein coupled receptor 179 OS=Homo sapiens GN=GPR179 PE=1 S | 1872 | LCQQQEtICIW | 0.12 | -0.125 | -1.224 | -0.41 |
| sp\|Q6PRD1\|GP179_HUMAN Probable G-protein coupled receptor 179 OS=Homo sapiens GN=GPR179 PE=1 S | 1884 | NKDLREsPAQA | 0.191 | -0.279 | -1.017 | -0.368 |
| sp\|Q6PRD1\|GP179_HUMAN Probable G-protein coupled receptor 179 OS=Homo sapiens GN=GPR179 PE=1 S | 1892 | AQAPKIsDLPS | 0.06 | -0.282 | -1.315 | -0.512 |
| sp\|Q6PRD1\|GP179_HUMAN Probable G-protein coupled receptor 179 OS=Homo sapiens GN=GPR179 PE=1 S | 1896 | KISDLPsSMSS | 0.053 | -0.236 | -1.682 | -0.622 |
| sp\|Q6PRD1\|GP179_HUMAN Probable G-protein coupled receptor 179 OS=Homo sapiens GN=GPR179 PE=1 S | 1897 | ISDLPSsMSSE | 0.037 | -0.337 | -1.701 | -0.667 |
| sp\|Q6PRD1\|GP179_HUMAN Probable G-protein coupled receptor 179 OS=Homo sapiens GN=GPR179 PE=1 S | 1899 | DLPSSMsSEVA | 0.417 | 0.241 | -0.391 | 0.089 |
| sp\|Q6PRD1\|GP179_HUMAN Probable G-protein coupled receptor 179 OS=Homo sapiens GN=GPR179 PE=1 S | 1900 | LPSSMSsEVAE | 0.097 | -0.194 | -1.03 | -0.376 |
| sp\|Q6PRD1\|GP179_HUMAN Probable G-protein coupled receptor 179 OS=Homo sapiens GN=GPR179 PE=1 S | 1907 | EVAEGHsLEAT | 0.138 | 0.1 | -0.514 | -0.092 |
| sp\|Q6PRD1\|GP179_HUMAN Probable G-protein coupled receptor 179 OS=Homo sapiens GN=GPR179 PE=1 S | 1911 | GHSLEAtEKGD | 0.294 | -0.006 | -0.921 | -0.211 |
| sp\|Q6PRD1\|GP179_HUMAN Probable G-protein coupled receptor 179 OS=Homo sapiens GN=GPR179 PE=1 S | 1922 | LRQDPKtGSFP | 0.078 | -0.032 | -0.982 | -0.312 |
| sp\|Q6PRD1\|GP179_HUMAN Probable G-protein coupled receptor 179 OS=Homo sapiens GN=GPR179 PE=1 S | 1924 | QDPKTGsFPEH | 0.287 | 0.273 | -0.553 | 0.002 |
| sp\|Q6PRD1\|GP179_HUMAN Probable G-protein coupled receptor 179 OS=Homo sapiens GN=GPR179 PE=1 S | 1930 | SFPEHItQEKA | 0.066 | -0.221 | -1.542 | -0.566 |
| sp\|Q6PRD1\|GP179_HUMAN Probable G-protein coupled receptor 179 OS=Homo sapiens GN=GPR179 PE=1 S | 1939 | KAPAADtEEFT | 0.086 | -0.278 | -1.418 | -0.537 |
| sp\|Q6PRD1\|GP179_HUMAN Probable G-protein coupled receptor 179 OS=Homo sapiens GN=GPR179 PE=1 S | 1943 | ADTEEFtTEDG | 0.104 | -0.142 | -1.177 | -0.405 |
| sp\|Q6PRD1\|GP179_HUMAN Probable G-protein coupled receptor 179 OS=Homo sapiens GN=GPR179 PE=1 S | 1944 | DTEEFTtEDGE | 0.25 | -0.094 | -0.876 | -0.24 |
| sp\|Q6PRD1\|GP179_HUMAN Probable G-protein coupled receptor 179 OS=Homo sapiens GN=GPR179 PE=1 S | 1950 | TEDGEKtSHEL | 0.184 | -0.064 | -0.962 | -0.281 |
| sp\|Q6PRD1\|GP179_HUMAN Probable G-protein coupled receptor 179 OS=Homo sapiens GN=GPR179 PE=1 S | 1951 | EDGEKTsHELQ | 0.034 | -0.278 | -1.632 | -0.625 |
| sp\|Q6PRD1\|GP179_HUMAN Probable G-protein coupled receptor 179 OS=Homo sapiens GN=GPR179 PE=1 S | 1956 | TSHELQsVCPW | 0.035 | -0.352 | -1.763 | -0.693 |
| sp\|Q6PRD1\|GP179_HUMAN Probable G-protein coupled receptor 179 OS=Homo sapiens GN=GPR179 PE=1 S | 1962 | SVCPWEtTAPA | 0.064 | -0.301 | -1.48 | -0.572 |
| sp\|Q6PRD1\|GP179_HUMAN Probable G-protein coupled receptor 179 OS=Homo sapiens GN=GPR179 PE=1 S | 1963 | VCPWETtAPAD | 0.679 | 0.488 | 0.053 | 0.407 |
| sp\|Q6PRD1\|GP179_HUMAN Probable G-protein coupled receptor 179 OS=Homo sapiens GN=GPR179 PE=1 S | 1968 | TTAPADsVSHL | 0.114 | -0.219 | -1.039 | -0.381 |
| sp\|Q6PRD1\|GP179_HUMAN Probable G-protein coupled receptor 179 OS=Homo sapiens GN=GPR179 PE=1 S | 1970 | APADSVsHLDR | 0.576 | 0.176 | -0.157 | 0.198 |

| | | | | | | |
|---|---|---|---|---|---|---|
| sp\|Q6PRD1\|GP179_HUMAN Probable G-protein coupled receptor 179 OS=Homo sapiens GN=GPR179 PE=1 S | 1983 | PDQPKAsSQRL | 0.116 | -0.183 | -1.171 | -0.413 |
| sp\|Q6PRD1\|GP179_HUMAN Probable G-protein coupled receptor 179 OS=Homo sapiens GN=GPR179 PE=1 S | 1984 | DQPKASsQRLV | 0.201 | -0.158 | -1.223 | -0.393 |
| sp\|Q6PRD1\|GP179_HUMAN Probable G-protein coupled receptor 179 OS=Homo sapiens GN=GPR179 PE=1 S | 1989 | SSQRLVsTGGR | 0.658 | 0.736 | 0.244 | 0.546 |
| sp\|Q6PRD1\|GP179_HUMAN Probable G-protein coupled receptor 179 OS=Homo sapiens GN=GPR179 PE=1 S | 1990 | SQRLVStGGRA | 0.183 | -0.045 | -0.931 | -0.264 |
| sp\|Q6PRD1\|GP179_HUMAN Probable G-protein coupled receptor 179 OS=Homo sapiens GN=GPR179 PE=1 S | 2010 | DAGVYKsDSSA | 0.092 | -0.169 | -1.283 | -0.453 |
| sp\|Q6PRD1\|GP179_HUMAN Probable G-protein coupled receptor 179 OS=Homo sapiens GN=GPR179 PE=1 S | 2012 | GVYKSDsSAKA | 0.284 | 0.054 | -0.643 | -0.102 |
| sp\|Q6PRD1\|GP179_HUMAN Probable G-protein coupled receptor 179 OS=Homo sapiens GN=GPR179 PE=1 S | 2013 | VYKSDSsAKAE | 0.176 | -0.107 | -0.897 | -0.276 |
| sp\|Q6PRD1\|GP179_HUMAN Probable G-protein coupled receptor 179 OS=Homo sapiens GN=GPR179 PE=1 S | 2018 | SSAKAEtCPWE | 0.637 | 0.483 | 0.009 | 0.376 |
| sp\|Q6PRD1\|GP179_HUMAN Probable G-protein coupled receptor 179 OS=Homo sapiens GN=GPR179 PE=1 S | 2024 | TCPWEVtERIP | 0.293 | -0.185 | -0.918 | -0.27 |
| sp\|Q6PRD1\|GP179_HUMAN Probable G-protein coupled receptor 179 OS=Homo sapiens GN=GPR179 PE=1 S | 2033 | IPVKGVsRQDG | 0.424 | -0.09 | -0.494 | -0.053 |
| sp\|Q6PRD1\|GP179_HUMAN Probable G-protein coupled receptor 179 OS=Homo sapiens GN=GPR179 PE=1 S | 2041 | QDGKGDsQEEK | 0.065 | -0.36 | -1.635 | -0.643 |
| sp\|Q6PRD1\|GP179_HUMAN Probable G-protein coupled receptor 179 OS=Homo sapiens GN=GPR179 PE=1 S | 2052 | GRAPEKsEPKG | 0.747 | 0.753 | 0.453 | 0.651 |
| sp\|Q6PRD1\|GP179_HUMAN Probable G-protein coupled receptor 179 OS=Homo sapiens GN=GPR179 PE=1 S | 2079 | AVCPWEsQDGK | 0.158 | -0.228 | -1.169 | -0.413 |
| sp\|Q6PRD1\|GP179_HUMAN Probable G-protein coupled receptor 179 OS=Homo sapiens GN=GPR179 PE=1 S | 2086 | QDGKGLsPQPA | 0.026 | -0.589 | -1.672 | -0.745 |
| sp\|Q6PRD1\|GP179_HUMAN Probable G-protein coupled receptor 179 OS=Homo sapiens GN=GPR179 PE=1 S | 2094 | QPAPDAsDRSR | 0.12 | -0.218 | -1.062 | -0.387 |
| sp\|Q6PRD1\|GP179_HUMAN Probable G-protein coupled receptor 179 OS=Homo sapiens GN=GPR179 PE=1 S | 2097 | PDASDRsRGSS | 0.042 | -0.334 | -1.605 | -0.632 |
| sp\|Q6PRD1\|GP179_HUMAN Probable G-protein coupled receptor 179 OS=Homo sapiens GN=GPR179 PE=1 S | 2100 | SDRSRGsSEAA | 0.177 | 0.069 | -0.876 | -0.21 |
| sp\|Q6PRD1\|GP179_HUMAN Probable G-protein coupled receptor 179 OS=Homo sapiens GN=GPR179 PE=1 S | 2101 | DRSRGSsEAAG | 0.556 | 0.985 | 0.202 | 0.581 |
| sp\|Q6PRD1\|GP179_HUMAN Probable G-protein coupled receptor 179 OS=Homo sapiens GN=GPR179 PE=1 S | 2106 | SSEAAGsVETR | 0.129 | -0.225 | -1.099 | -0.398 |
| sp\|Q6PRD1\|GP179_HUMAN Probable G-protein coupled receptor 179 OS=Homo sapiens GN=GPR179 PE=1 S | 2109 | AAGSVEtRVAE | 0.086 | -0.315 | -1.593 | -0.607 |
| sp\|Q6PRD1\|GP179_HUMAN Probable G-protein coupled receptor 179 OS=Homo sapiens GN=GPR179 PE=1 S | 2124 | EVVEAPsAKKA | 0.079 | -0.094 | -1.184 | -0.4 |
| sp\|Q6PRD1\|GP179_HUMAN Probable G-protein coupled receptor 179 OS=Homo sapiens GN=GPR179 PE=1 S | 2149 | GEQEREsQGQG | 0.351 | 0.095 | -0.899 | -0.151 |
| sp\|Q6PRD1\|GP179_HUMAN Probable G-protein coupled receptor 179 OS=Homo sapiens GN=GPR179 PE=1 S | 2165 | KAGPGGtEEHF | 0.026 | -0.365 | -1.685 | -0.675 |
| sp\|Q6PRD1\|GP179_HUMAN Probable G-protein coupled receptor 179 OS=Homo sapiens GN=GPR179 PE=1 S | 2170 | GTEEHFsKAAA | 0.281 | 0.025 | -0.547 | -0.08 |
| sp\|Q6PRD1\|GP179_HUMAN Probable G-protein coupled receptor 179 OS=Homo sapiens GN=GPR179 PE=1 S | 2188 | VCPGEGtGSGG | 0.054 | -0.369 | -1.67 | -0.662 |
| sp\|Q6PRD1\|GP179_HUMAN Probable G-protein coupled receptor 179 OS=Homo sapiens GN=GPR179 PE=1 S | 2190 | PGEGTGsGGLL | 0.115 | -0.048 | -0.759 | -0.231 |
| sp\|Q6PRD1\|GP179_HUMAN Probable G-protein coupled receptor 179 OS=Homo sapiens GN=GPR179 PE=1 S | 2197 | GGLLPQsGALD | 0.162 | -0.153 | -0.74 | -0.244 |
| sp\|Q6PRD1\|GP179_HUMAN Probable G-protein coupled receptor 179 OS=Homo sapiens GN=GPR179 PE=1 S | 2207 | DPELKVsPKEA | 0.121 | -0.467 | -1.303 | -0.55 |
| sp\|Q6PRD1\|GP179_HUMAN Probable G-protein coupled receptor 179 OS=Homo sapiens GN=GPR179 PE=1 S | 2213 | SPKEAGsMGSR | 0.096 | -0.014 | -1.23 | -0.383 |
| sp\|Q6PRD1\|GP179_HUMAN Probable G-protein coupled receptor 179 OS=Homo sapiens GN=GPR179 PE=1 S | 2216 | EAGSMGsRMAE | 0.055 | -0.37 | -1.494 | -0.603 |
| sp\|Q6PRD1\|GP179_HUMAN Probable G-protein coupled receptor 179 OS=Homo sapiens GN=GPR179 PE=1 S | 2227 | LCQWEItDPEG | 0.81 | 0.555 | 0.184 | 0.516 |
| sp\|Q6PRD1\|GP179_HUMAN Probable G-protein coupled receptor 179 OS=Homo sapiens GN=GPR179 PE=1 S | 2237 | GNKIKGtMADI | 0.188 | 0.005 | -0.791 | -0.199 |
| sp\|Q6PRD1\|GP179_HUMAN Probable G-protein coupled receptor 179 OS=Homo sapiens GN=GPR179 PE=1 S | 2247 | ICPGEEtGVPS | 0.072 | -0.287 | -1.568 | -0.594 |
| sp\|Q6PRD1\|GP179_HUMAN Probable G-protein coupled receptor 179 OS=Homo sapiens GN=GPR179 PE=1 S | 2251 | EETGVPsEESG | 0.081 | -0.116 | -1.151 | -0.395 |
| sp\|Q6PRD1\|GP179_HUMAN Probable G-protein coupled receptor 179 OS=Homo sapiens GN=GPR179 PE=1 S | 2254 | GVPSEEsGLLA | 0.057 | -0.292 | -1.661 | -0.632 |
| sp\|Q6PRD1\|GP179_HUMAN Probable G-protein coupled receptor 179 OS=Homo sapiens GN=GPR179 PE=1 S | 2260 | SGLLALtATRR | 0.172 | -0.129 | -1.161 | -0.373 |
| sp\|Q6PRD1\|GP179_HUMAN Probable G-protein coupled receptor 179 OS=Homo sapiens GN=GPR179 PE=1 S | 2262 | LLALTAtRREF | 0.21 | -0.03 | -0.68 | -0.167 |
| sp\|Q6PRD1\|GP179_HUMAN Probable G-protein coupled receptor 179 OS=Homo sapiens GN=GPR179 PE=1 S | 2269 | RREFFPtAPEK | 0.752 | 0.669 | 0.099 | 0.507 |
| sp\|Q6PRD1\|GP179_HUMAN Probable G-protein coupled receptor 179 OS=Homo sapiens GN=GPR179 PE=1 S | 2290 | DHFFPEsKIPC | 0.094 | -0.291 | -1.595 | -0.597 |
| sp\|Q6PRD1\|GP179_HUMAN Probable G-protein coupled receptor 179 OS=Homo sapiens GN=GPR179 PE=1 S | 2298 | IPCPKVsRPAS | 0.544 | 0.285 | -0.151 | 0.226 |
| sp\|Q6PRD1\|GP179_HUMAN Probable G-protein coupled receptor 179 OS=Homo sapiens GN=GPR179 PE=1 S | 2302 | KVSRPAsTFTL | 0.519 | 0.744 | 0.2 | 0.488 |
| sp\|Q6PRD1\|GP179_HUMAN Probable G-protein coupled receptor 179 OS=Homo sapiens GN=GPR179 PE=1 S | 2303 | VSRPAStFTLE | 0.395 | 0.105 | -0.386 | 0.038 |
| sp\|Q6PRD1\|GP179_HUMAN Probable G-protein coupled receptor 179 OS=Homo sapiens GN=GPR179 PE=1 S | 2305 | RPASTFtLEGV | 0.226 | 0.023 | -0.671 | -0.141 |
| sp\|Q6PRD1\|GP179_HUMAN Probable G-protein coupled receptor 179 OS=Homo sapiens GN=GPR179 PE=1 S | 2316 | RELQGPsGLEP | 0.075 | -0.275 | -1.329 | -0.51 |
| sp\|Q6PRD1\|GP179_HUMAN Probable G-protein coupled receptor 179 OS=Homo sapiens GN=GPR179 PE=1 S | 2322 | SGLEPRtSLAP | 0.029 | -0.306 | -1.721 | -0.666 |
| sp\|Q6PRD1\|GP179_HUMAN Probable G-protein coupled receptor 179 OS=Homo sapiens GN=GPR179 PE=1 S | 2323 | GLEPRTsLAPE | 0.566 | 0.136 | -0.028 | 0.225 |
| sp\|Q6PRD1\|GP179_HUMAN Probable G-protein coupled receptor 179 OS=Homo sapiens GN=GPR179 PE=1 S | 2329 | SLAPEPsLQEA | 0.348 | -0.049 | -0.439 | -0.047 |
| sp\|Q6PRD1\|GP179_HUMAN Probable G-protein coupled receptor 179 OS=Homo sapiens GN=GPR179 PE=1 S | 2335 | SLQEAEsQSSS | 0.058 | -0.132 | -1.752 | -0.609 |
| sp\|Q6PRD1\|GP179_HUMAN Probable G-protein coupled receptor 179 OS=Homo sapiens GN=GPR179 PE=1 S | 2337 | QEAESQsSSLT | 0.096 | -0.009 | -0.948 | -0.287 |

| | | | | | | |
|---|---|---|---|---|---|---|
| sp\|Q6PRD1\|GP179_HUMAN Probable G-protein coupled receptor 179 OS=Homo sapiens GN=GPR179 PE=1 S | 2338 | EAESQSsSLTE | 0.031 | -0.395 | -1.84 | -0.735 |
| sp\|Q6PRD1\|GP179_HUMAN Probable G-protein coupled receptor 179 OS=Homo sapiens GN=GPR179 PE=1 S | 2339 | AESQSSsLTED | 0.58 | 0.159 | -0.037 | 0.234 |
| sp\|Q6PRD1\|GP179_HUMAN Probable G-protein coupled receptor 179 OS=Homo sapiens GN=GPR179 PE=1 S | 2341 | SQSSSLtEDSG | 0.116 | 0.046 | -0.788 | -0.209 |
| sp\|Q6PRD1\|GP179_HUMAN Probable G-protein coupled receptor 179 OS=Homo sapiens GN=GPR179 PE=1 S | 2344 | SSLTEDsGQVA | 0.088 | -0.342 | -1.487 | -0.58 |
| sp\|Q6PRD1\|GP179_HUMAN Probable G-protein coupled receptor 179 OS=Homo sapiens GN=GPR179 PE=1 S | 2357 | AQYEEFtPPTV | 0.164 | 0.065 | -0.996 | -0.256 |
| sp\|Q6PRD1\|GP179_HUMAN Probable G-protein coupled receptor 179 OS=Homo sapiens GN=GPR179 PE=1 S | 2360 | EEFTPPtVYPW | 0.074 | -0.247 | -1.288 | -0.487 |
| sp\|Q8NFJ5\|RAI3_HUMAN Retinoic acid-induced protein 3 OS=Homo sapiens GN=GPRC5A PE=1 SV=2 | 312 | TQGFEEtGDTL | 0.099 | -0.171 | -1.162 | -0.411 |
| sp\|Q8NFJ5\|RAI3_HUMAN Retinoic acid-induced protein 3 OS=Homo sapiens GN=GPRC5A PE=1 SV=2 | 315 | FEETGDtLYAP | 0.195 | -0.055 | -0.862 | -0.241 |
| sp\|Q8NFJ5\|RAI3_HUMAN Retinoic acid-induced protein 3 OS=Homo sapiens GN=GPRC5A PE=1 SV=2 | 321 | TLYAPYsTHFQ | 0.124 | -0.132 | -0.886 | -0.298 |
| sp\|Q8NFJ5\|RAI3_HUMAN Retinoic acid-induced protein 3 OS=Homo sapiens GN=GPRC5A PE=1 SV=2 | 322 | LYAPYStHFQL | 0.214 | -0.068 | -0.496 | -0.117 |
| sp\|Q8NFJ5\|RAI3_HUMAN Retinoic acid-induced protein 3 OS=Homo sapiens GN=GPRC5A PE=1 SV=2 | 336 | PPQKEFsIPRA | 0.667 | 0.421 | 0.018 | 0.369 |
| sp\|Q8NFJ5\|RAI3_HUMAN Retinoic acid-induced protein 3 OS=Homo sapiens GN=GPRC5A PE=1 SV=2 | 345 | RAHAWPsPYKD | 0.076 | -0.422 | -1.415 | -0.587 |
| sp\|Q8NFJ5\|RAI3_HUMAN Retinoic acid-induced protein 3 OS=Homo sapiens GN=GPRC5A PE=1 SV=2 | 357 | EVKKEGs---- | 0.188 | 0.064 | -0.88 | -0.209 |
| sp\|Q9UP38\|FZD1_HUMAN Frizzled-1 OS=Homo sapiens GN=FZD1 PE=1 SV=2 | 319 | PEELRFsRTWI | 0.459 | 0.119 | -0.353 | 0.075 |
| sp\|Q9UP38\|FZD1_HUMAN Frizzled-1 OS=Homo sapiens GN=FZD1 PE=1 SV=2 | 321 | ELRFSRtWIGI | 0.658 | 0.627 | 0.483 | 0.589 |
| sp\|Q9UP38\|FZD1_HUMAN Frizzled-1 OS=Homo sapiens GN=FZD1 PE=1 SV=2 | 327 | TWIGIWsVLCC | 0.083 | -0.089 | -1.219 | -0.408 |
| sp\|Q9UP38\|FZD1_HUMAN Frizzled-1 OS=Homo sapiens GN=FZD1 PE=1 SV=2 | 333 | SVLCCAsTLFT | 0.105 | -0.21 | -1.33 | -0.478 |
| sp\|Q9UP38\|FZD1_HUMAN Frizzled-1 OS=Homo sapiens GN=FZD1 PE=1 SV=2 | 334 | VLCCAStLFTV | 0.563 | 0.119 | -0.354 | 0.109 |
| sp\|Q9UP38\|FZD1_HUMAN Frizzled-1 OS=Homo sapiens GN=FZD1 PE=1 SV=2 | 337 | CASTLFtVLTY | 0.152 | -0.108 | -1.044 | -0.333 |
| sp\|Q9UP38\|FZD1_HUMAN Frizzled-1 OS=Homo sapiens GN=FZD1 PE=1 SV=2 | 340 | TLFTVLtYLVD | 0.137 | -0.049 | -1.071 | -0.328 |
| sp\|Q9UP38\|FZD1_HUMAN Frizzled-1 OS=Homo sapiens GN=FZD1 PE=1 SV=2 | 349 | VDMRRFsYPER | 0.878 | 1.432 | 1.235 | 1.182 |
| sp\|Q9UP38\|FZD1_HUMAN Frizzled-1 OS=Homo sapiens GN=FZD1 PE=1 SV=2 | 359 | RPIIFLsGCYT | 0.045 | -0.301 | -1.717 | -0.658 |
| sp\|Q9UP38\|FZD1_HUMAN Frizzled-1 OS=Homo sapiens GN=FZD1 PE=1 SV=2 | 363 | FLSGCYtAVAV | 0.486 | 0.331 | -0.231 | 0.195 |
| sp\|Q9UP38\|FZD1_HUMAN Frizzled-1 OS=Homo sapiens GN=FZD1 PE=1 SV=2 | 392 | AEDGARtVAQG | 0.14 | -0.134 | -1.175 | -0.39 |
| sp\|Q9UP38\|FZD1_HUMAN Frizzled-1 OS=Homo sapiens GN=FZD1 PE=1 SV=2 | 397 | RTVAQGtKKEG | 0.133 | -0.151 | -1.187 | -0.402 |
| sp\|Q9UP38\|FZD1_HUMAN Frizzled-1 OS=Homo sapiens GN=FZD1 PE=1 SV=2 | 403 | TKKEGCtILFM | 0.091 | -0.189 | -1.201 | -0.433 |
| sp\|Q9UP38\|FZD1_HUMAN Frizzled-1 OS=Homo sapiens GN=FZD1 PE=1 SV=2 | 413 | MMLYFFsMASS | 0.098 | 0.185 | -1.014 | -0.244 |
| sp\|Q9UP38\|FZD1_HUMAN Frizzled-1 OS=Homo sapiens GN=FZD1 PE=1 SV=2 | 416 | YFFSMAsSIWW | 0.142 | -0.076 | -0.699 | -0.211 |
| sp\|Q9UP38\|FZD1_HUMAN Frizzled-1 OS=Homo sapiens GN=FZD1 PE=1 SV=2 | 417 | FFSMASsIWWV | 0.286 | 0.029 | -0.647 | -0.111 |
| sp\|Q9UP38\|FZD1_HUMAN Frizzled-1 OS=Homo sapiens GN=FZD1 PE=1 SV=2 | 424 | IWWVILsLTWF | 0.176 | 0.114 | -0.465 | -0.058 |
| sp\|Q9UP38\|FZD1_HUMAN Frizzled-1 OS=Homo sapiens GN=FZD1 PE=1 SV=2 | 426 | WVILSLtWFLA | 0.185 | 0.223 | -0.439 | -0.01 |
| sp\|Q9UP38\|FZD1_HUMAN Frizzled-1 OS=Homo sapiens GN=FZD1 PE=1 SV=2 | 444 | EAIEANsQYFH | 0.064 | -0.214 | -1.727 | -0.626 |
| sp\|Q9UP38\|FZD1_HUMAN Frizzled-1 OS=Homo sapiens GN=FZD1 PE=1 SV=2 | 459 | AVPAIKtITIL | 0.197 | -0.118 | -0.647 | -0.189 |
| sp\|Q9UP38\|FZD1_HUMAN Frizzled-1 OS=Homo sapiens GN=FZD1 PE=1 SV=2 | 461 | PAIKTItILAL | 0.209 | 0.088 | -0.586 | -0.096 |
| sp\|Q9UP38\|FZD1_HUMAN Frizzled-1 OS=Homo sapiens GN=FZD1 PE=1 SV=2 | 474 | VDGDVLsGVCF | 0.034 | -0.265 | -1.699 | -0.643 |
| sp\|Q9UP38\|FZD1_HUMAN Frizzled-1 OS=Homo sapiens GN=FZD1 PE=1 SV=2 | 503 | VYLFIGtSFLL | 0.162 | -0.052 | -0.753 | -0.214 |
| sp\|Q9UP38\|FZD1_HUMAN Frizzled-1 OS=Homo sapiens GN=FZD1 PE=1 SV=2 | 504 | YLFIGTsFLLA | 0.19 | 0.073 | -0.576 | -0.104 |
| sp\|Q9UP38\|FZD1_HUMAN Frizzled-1 OS=Homo sapiens GN=FZD1 PE=1 SV=2 | 512 | LLAGFVsLFRI | 0.652 | 0.133 | 0.237 | 0.341 |
| sp\|Q9UP38\|FZD1_HUMAN Frizzled-1 OS=Homo sapiens GN=FZD1 PE=1 SV=2 | 518 | SLFRIRtIMKH | 0.33 | 0.697 | -0.347 | 0.227 |
| sp\|Q9UP38\|FZD1_HUMAN Frizzled-1 OS=Homo sapiens GN=FZD1 PE=1 SV=2 | 525 | IMKHDGtKTEK | 0.226 | -0.094 | -0.762 | -0.21 |
| sp\|Q9UP38\|FZD1_HUMAN Frizzled-1 OS=Homo sapiens GN=FZD1 PE=1 SV=2 | 527 | KHDGTKtEKLE | 0.119 | 0.008 | -1.009 | -0.294 |
| sp\|Q9UP38\|FZD1_HUMAN Frizzled-1 OS=Homo sapiens GN=FZD1 PE=1 SV=2 | 541 | VRIGVFsVLYT | 0.122 | 0.109 | -0.969 | -0.246 |
| sp\|Q9UP38\|FZD1_HUMAN Frizzled-1 OS=Homo sapiens GN=FZD1 PE=1 SV=2 | 545 | VFSVLYtVPAT | 0.378 | 0.464 | 0.069 | 0.304 |
| sp\|Q9UP38\|FZD1_HUMAN Frizzled-1 OS=Homo sapiens GN=FZD1 PE=1 SV=2 | 549 | LYTVPAtIVIA | 0.148 | -0.137 | -0.762 | -0.25 |
| sp\|Q9UP38\|FZD1_HUMAN Frizzled-1 OS=Homo sapiens GN=FZD1 PE=1 SV=2 | 568 | RDQWERsWVAQ | 0.13 | -0.008 | -1.116 | -0.331 |
| sp\|Q9UP38\|FZD1_HUMAN Frizzled-1 OS=Homo sapiens GN=FZD1 PE=1 SV=2 | 573 | RSWVAQsCKSY | 0.144 | 0.167 | -0.893 | -0.194 |
| sp\|Q9UP38\|FZD1_HUMAN Frizzled-1 OS=Homo sapiens GN=FZD1 PE=1 SV=2 | 576 | VAQSCKsYAIP | 0.167 | -0.205 | -0.923 | -0.32 |
| sp\|Q9UP38\|FZD1_HUMAN Frizzled-1 OS=Homo sapiens GN=FZD1 PE=1 SV=2 | 597 | PPHPPMsPDFT | 0.042 | -0.475 | -1.438 | -0.624 |
| sp\|Q9UP38\|FZD1_HUMAN Frizzled-1 OS=Homo sapiens GN=FZD1 PE=1 SV=2 | 601 | PMSPDFtVFMI | 0.189 | 0.035 | -0.597 | -0.124 |

| | | | | | | |
|---|---|---|---|---|---|---|
| sp\|Q9UP38\|FZD1_HUMAN Frizzled-1 OS=Homo sapiens GN=FZD1 PE=1 SV=2 | 610 | MIKYLMtLIVG | 0.592 | 0.421 | 0.285 | 0.433 |
| sp\|Q9UP38\|FZD1_HUMAN Frizzled-1 OS=Homo sapiens GN=FZD1 PE=1 SV=2 | 616 | TLIVGItSGFW | 0.086 | -0.096 | -1.167 | -0.392 |
| sp\|Q9UP38\|FZD1_HUMAN Frizzled-1 OS=Homo sapiens GN=FZD1 PE=1 SV=2 | 617 | LIVGITsGFWI | 0.208 | 0.063 | -0.214 | 0.019 |
| sp\|Q9UP38\|FZD1_HUMAN Frizzled-1 OS=Homo sapiens GN=FZD1 PE=1 SV=2 | 623 | SGFWIWsGKTL | 0.134 | -0.22 | -0.786 | -0.291 |
| sp\|Q9UP38\|FZD1_HUMAN Frizzled-1 OS=Homo sapiens GN=FZD1 PE=1 SV=2 | 626 | WIWSGKtLNSW | 0.179 | 0.155 | -0.327 | 0.002 |
| sp\|Q9UP38\|FZD1_HUMAN Frizzled-1 OS=Homo sapiens GN=FZD1 PE=1 SV=2 | 629 | SGKTLNsWRKF | 0.114 | -0.125 | -1.191 | -0.401 |
| sp\|Q9UP38\|FZD1_HUMAN Frizzled-1 OS=Homo sapiens GN=FZD1 PE=1 SV=2 | 635 | SWRKFYtRLTN | 0.305 | 0.185 | -0.732 | -0.081 |
| sp\|Q9UP38\|FZD1_HUMAN Frizzled-1 OS=Homo sapiens GN=FZD1 PE=1 SV=2 | 638 | KFYTRLtNSKQ | 0.081 | -0.145 | -1.396 | -0.487 |
| sp\|Q9UP38\|FZD1_HUMAN Frizzled-1 OS=Homo sapiens GN=FZD1 PE=1 SV=2 | 640 | YTRLTNsKQGE | 0.425 | 0.205 | -0.36 | 0.09 |
| sp\|Q9UP38\|FZD1_HUMAN Frizzled-1 OS=Homo sapiens GN=FZD1 PE=1 SV=2 | 645 | NSKQGEtTV-- | 0.122 | -0.059 | -1.028 | -0.322 |
| sp\|Q9UP38\|FZD1_HUMAN Frizzled-1 OS=Homo sapiens GN=FZD1 PE=1 SV=2 | 646 | SKQGETtV--- | 0.236 | 0.162 | -0.753 | -0.118 |
| sp\|Q14332\|FZD2_HUMAN Frizzled-2 OS=Homo sapiens GN=FZD2 PE=1 SV=1 | 311 | VCNERFsEDGY | 0.452 | 0.245 | -0.414 | 0.094 |
| sp\|Q14332\|FZD2_HUMAN Frizzled-2 OS=Homo sapiens GN=FZD2 PE=1 SV=1 | 317 | SEDGYRtVVQG | 0.085 | -0.163 | -1.413 | -0.497 |
| sp\|Q14332\|FZD2_HUMAN Frizzled-2 OS=Homo sapiens GN=FZD2 PE=1 SV=1 | 322 | RTVVQGtKKEG | 0.092 | -0.146 | -1.353 | -0.469 |
| sp\|Q14332\|FZD2_HUMAN Frizzled-2 OS=Homo sapiens GN=FZD2 PE=1 SV=1 | 328 | TKKEGCtILFM | 0.091 | -0.189 | -1.201 | -0.433 |
| sp\|Q14332\|FZD2_HUMAN Frizzled-2 OS=Homo sapiens GN=FZD2 PE=1 SV=1 | 338 | MMLYFFsMASS | 0.098 | 0.185 | -1.014 | -0.244 |
| sp\|Q14332\|FZD2_HUMAN Frizzled-2 OS=Homo sapiens GN=FZD2 PE=1 SV=1 | 341 | YFFSMAsSIWW | 0.142 | -0.076 | -0.699 | -0.211 |
| sp\|Q14332\|FZD2_HUMAN Frizzled-2 OS=Homo sapiens GN=FZD2 PE=1 SV=1 | 342 | FFSMASsIWWV | 0.286 | 0.029 | -0.647 | -0.111 |
| sp\|Q14332\|FZD2_HUMAN Frizzled-2 OS=Homo sapiens GN=FZD2 PE=1 SV=1 | 349 | IWWVILsLTWF | 0.176 | 0.114 | -0.465 | -0.058 |
| sp\|Q14332\|FZD2_HUMAN Frizzled-2 OS=Homo sapiens GN=FZD2 PE=1 SV=1 | 351 | WVILSLtWFLA | 0.185 | 0.223 | -0.439 | -0.01 |
| sp\|Q14332\|FZD2_HUMAN Frizzled-2 OS=Homo sapiens GN=FZD2 PE=1 SV=1 | 369 | EAIEANsQYFH | 0.064 | -0.214 | -1.727 | -0.626 |
| sp\|Q14332\|FZD2_HUMAN Frizzled-2 OS=Homo sapiens GN=FZD2 PE=1 SV=1 | 384 | AVPAVKtITIL | 0.211 | -0.125 | -0.808 | -0.241 |
| sp\|Q14332\|FZD2_HUMAN Frizzled-2 OS=Homo sapiens GN=FZD2 PE=1 SV=1 | 386 | PAVKTItILAM | 0.198 | 0.005 | -0.632 | -0.143 |
| sp\|Q14332\|FZD2_HUMAN Frizzled-2 OS=Homo sapiens GN=FZD2 PE=1 SV=1 | 399 | IDGDLLsGVCF | 0.031 | -0.274 | -1.682 | -0.642 |
| sp\|Q14332\|FZD2_HUMAN Frizzled-2 OS=Homo sapiens GN=FZD2 PE=1 SV=1 | 408 | CFVGLNsLDPL | 0.411 | 0.15 | -0.119 | 0.147 |
| sp\|Q14332\|FZD2_HUMAN Frizzled-2 OS=Homo sapiens GN=FZD2 PE=1 SV=1 | 428 | VYLFIGtSFLL | 0.162 | -0.052 | -0.753 | -0.214 |
| sp\|Q14332\|FZD2_HUMAN Frizzled-2 OS=Homo sapiens GN=FZD2 PE=1 SV=1 | 429 | YLFIGTsFLLA | 0.19 | 0.073 | -0.576 | -0.104 |
| sp\|Q14332\|FZD2_HUMAN Frizzled-2 OS=Homo sapiens GN=FZD2 PE=1 SV=1 | 437 | LLAGFVsLFRI | 0.652 | 0.133 | 0.237 | 0.341 |
| sp\|Q14332\|FZD2_HUMAN Frizzled-2 OS=Homo sapiens GN=FZD2 PE=1 SV=1 | 443 | SLFRIRtIMKH | 0.33 | 0.697 | -0.347 | 0.227 |
| sp\|Q14332\|FZD2_HUMAN Frizzled-2 OS=Homo sapiens GN=FZD2 PE=1 SV=1 | 450 | IMKHDGtKTEK | 0.226 | -0.094 | -0.762 | -0.21 |
| sp\|Q14332\|FZD2_HUMAN Frizzled-2 OS=Homo sapiens GN=FZD2 PE=1 SV=1 | 452 | KHDGTKtEKLE | 0.119 | 0.008 | -1.009 | -0.294 |
| sp\|Q14332\|FZD2_HUMAN Frizzled-2 OS=Homo sapiens GN=FZD2 PE=1 SV=1 | 466 | VRIGVFsVLYT | 0.122 | 0.109 | -0.969 | -0.246 |
| sp\|Q14332\|FZD2_HUMAN Frizzled-2 OS=Homo sapiens GN=FZD2 PE=1 SV=1 | 470 | VFSVLYtVPAT | 0.378 | 0.464 | 0.069 | 0.304 |
| sp\|Q14332\|FZD2_HUMAN Frizzled-2 OS=Homo sapiens GN=FZD2 PE=1 SV=1 | 474 | LYTVPAtIVIA | 0.148 | -0.137 | -0.762 | -0.25 |
| sp\|Q14332\|FZD2_HUMAN Frizzled-2 OS=Homo sapiens GN=FZD2 PE=1 SV=1 | 493 | REHWERsWVSQ | 0.056 | -0.156 | -1.742 | -0.614 |
| sp\|Q14332\|FZD2_HUMAN Frizzled-2 OS=Homo sapiens GN=FZD2 PE=1 SV=1 | 496 | WERSWVsQHCK | 0.48 | 0.116 | -0.56 | 0.012 |
| sp\|Q14332\|FZD2_HUMAN Frizzled-2 OS=Homo sapiens GN=FZD2 PE=1 SV=1 | 501 | VSQHCKsLAIP | 0.408 | -0.035 | -0.256 | 0.039 |
| sp\|Q14332\|FZD2_HUMAN Frizzled-2 OS=Homo sapiens GN=FZD2 PE=1 SV=1 | 511 | PCPAHYtPRMS | 0.103 | -0.364 | -1.322 | -0.528 |
| sp\|Q14332\|FZD2_HUMAN Frizzled-2 OS=Homo sapiens GN=FZD2 PE=1 SV=1 | 515 | HYTPRMsPDFT | 0.201 | -0.062 | -0.559 | -0.14 |
| sp\|Q14332\|FZD2_HUMAN Frizzled-2 OS=Homo sapiens GN=FZD2 PE=1 SV=1 | 519 | RMSPDFtVYMI | 0.173 | 0.049 | -0.726 | -0.168 |
| sp\|Q14332\|FZD2_HUMAN Frizzled-2 OS=Homo sapiens GN=FZD2 PE=1 SV=1 | 528 | MIKYLMtLIVG | 0.592 | 0.421 | 0.285 | 0.433 |
| sp\|Q14332\|FZD2_HUMAN Frizzled-2 OS=Homo sapiens GN=FZD2 PE=1 SV=1 | 534 | TLIVGItSGFW | 0.086 | -0.096 | -1.167 | -0.392 |
| sp\|Q14332\|FZD2_HUMAN Frizzled-2 OS=Homo sapiens GN=FZD2 PE=1 SV=1 | 535 | LIVGITsGFWI | 0.208 | 0.063 | -0.214 | 0.019 |
| sp\|Q14332\|FZD2_HUMAN Frizzled-2 OS=Homo sapiens GN=FZD2 PE=1 SV=1 | 541 | SGFWIWsGKTL | 0.134 | -0.22 | -0.786 | -0.291 |
| sp\|Q14332\|FZD2_HUMAN Frizzled-2 OS=Homo sapiens GN=FZD2 PE=1 SV=1 | 544 | WIWSGKtLHSW | 0.175 | 0.146 | -0.469 | -0.049 |
| sp\|Q14332\|FZD2_HUMAN Frizzled-2 OS=Homo sapiens GN=FZD2 PE=1 SV=1 | 547 | SGKTLHsWRKF | 0.117 | -0.045 | -1.067 | -0.332 |
| sp\|Q14332\|FZD2_HUMAN Frizzled-2 OS=Homo sapiens GN=FZD2 PE=1 SV=1 | 553 | SWRKFYtRLTN | 0.305 | 0.185 | -0.732 | -0.081 |
| sp\|Q14332\|FZD2_HUMAN Frizzled-2 OS=Homo sapiens GN=FZD2 PE=1 SV=1 | 556 | KFYTRLtNSRH | 0.077 | -0.263 | -1.614 | -0.6 |
| sp\|Q14332\|FZD2_HUMAN Frizzled-2 OS=Homo sapiens GN=FZD2 PE=1 SV=1 | 558 | YTRLTNsRHGE | 0.374 | 0.132 | -0.663 | -0.052 |

| | | | | | | |
|---|---|---|---|---|---|---|
| sp\|Q14332\|FZD2_HUMAN Frizzled-2 OS=Homo sapiens GN=FZD2 PE=1 SV=1 | 563 | NSRHGEtTV-- | 0.162 | 0.124 | -0.973 | -0.229 |
| sp\|Q14332\|FZD2_HUMAN Frizzled-2 OS=Homo sapiens GN=FZD2 PE=1 SV=1 | 564 | SRHGETtV--- | 0.108 | 0.21 | -1.114 | -0.265 |
| sp\|Q9NPG1\|FZD3_HUMAN Frizzled-3 OS=Homo sapiens GN=FZD3 PE=1 SV=1 | 308 | VWWVILtITWF | 0.051 | -0.061 | -1.092 | -0.367 |
| sp\|Q9NPG1\|FZD3_HUMAN Frizzled-3 OS=Homo sapiens GN=FZD3 PE=1 SV=1 | 310 | WVILTItWFLA | 0.124 | 0.074 | -0.748 | -0.183 |
| sp\|Q9NPG1\|FZD3_HUMAN Frizzled-3 OS=Homo sapiens GN=FZD3 PE=1 SV=1 | 321 | AVPKWGsEAIE | 0.163 | -0.13 | -1.072 | -0.346 |
| sp\|Q9NPG1\|FZD3_HUMAN Frizzled-3 OS=Homo sapiens GN=FZD3 PE=1 SV=1 | 334 | ALLFHAsAWGI | 0.704 | 0.245 | -0.054 | 0.298 |
| sp\|Q9NPG1\|FZD3_HUMAN Frizzled-3 OS=Homo sapiens GN=FZD3 PE=1 SV=1 | 341 | AWGIPGtLTII | 0.254 | -0.073 | -0.674 | -0.164 |
| sp\|Q9NPG1\|FZD3_HUMAN Frizzled-3 OS=Homo sapiens GN=FZD3 PE=1 SV=1 | 343 | GIPGTLtIILL | 0.066 | 0.017 | -1.007 | -0.308 |
| sp\|Q9NPG1\|FZD3_HUMAN Frizzled-3 OS=Homo sapiens GN=FZD3 PE=1 SV=1 | 358 | IEGDNIsGVCF | 0.123 | -0.115 | -1.056 | -0.349 |
| sp\|Q9NPG1\|FZD3_HUMAN Frizzled-3 OS=Homo sapiens GN=FZD3 PE=1 SV=1 | 388 | YVVVGVsLLLA | 0.134 | -0.052 | -0.764 | -0.227 |
| sp\|Q9NPG1\|FZD3_HUMAN Frizzled-3 OS=Homo sapiens GN=FZD3 PE=1 SV=1 | 396 | LLAGIIsLNRV | 0.493 | 0.103 | -0.009 | 0.196 |
| sp\|Q9NPG1\|FZD3_HUMAN Frizzled-3 OS=Homo sapiens GN=FZD3 PE=1 SV=1 | 425 | IRIGVFsILYL | 0.16 | 0.173 | -0.746 | -0.138 |
| sp\|Q9NPG1\|FZD3_HUMAN Frizzled-3 OS=Homo sapiens GN=FZD3 PE=1 SV=1 | 451 | YRGIWEtTWIQ | 0.215 | 0.16 | -0.927 | -0.184 |
| sp\|Q9NPG1\|FZD3_HUMAN Frizzled-3 OS=Homo sapiens GN=FZD3 PE=1 SV=1 | 452 | RGIWETtWIQE | 0.191 | -0.035 | -0.677 | -0.174 |
| sp\|Q9NPG1\|FZD3_HUMAN Frizzled-3 OS=Homo sapiens GN=FZD3 PE=1 SV=1 | 470 | PCPYQVtQMSR | 0.158 | -0.082 | -1.192 | -0.372 |
| sp\|Q9NPG1\|FZD3_HUMAN Frizzled-3 OS=Homo sapiens GN=FZD3 PE=1 SV=1 | 473 | YQVTQMsRPDL | 0.531 | 0.568 | 0.033 | 0.377 |
| sp\|Q9NPG1\|FZD3_HUMAN Frizzled-3 OS=Homo sapiens GN=FZD3 PE=1 SV=1 | 494 | LIVGIPsVFWV | 0.158 | 0.012 | -0.676 | -0.169 |
| sp\|Q9NPG1\|FZD3_HUMAN Frizzled-3 OS=Homo sapiens GN=FZD3 PE=1 SV=1 | 500 | SVFWVGsKKTC | 0.071 | -0.318 | -1.484 | -0.577 |
| sp\|Q9NPG1\|FZD3_HUMAN Frizzled-3 OS=Homo sapiens GN=FZD3 PE=1 SV=1 | 503 | WVGSKKtCFEW | 0.123 | -0.011 | -0.772 | -0.22 |
| sp\|Q9NPG1\|FZD3_HUMAN Frizzled-3 OS=Homo sapiens GN=FZD3 PE=1 SV=1 | 509 | TCFEWAsFFHG | 0.299 | 0.087 | -0.549 | -0.054 |
| sp\|Q9NPG1\|FZD3_HUMAN Frizzled-3 OS=Homo sapiens GN=FZD3 PE=1 SV=1 | 523 | KEIVNEsRQVL | 0.093 | -0.152 | -1.4 | -0.486 |
| sp\|Q9NPG1\|FZD3_HUMAN Frizzled-3 OS=Homo sapiens GN=FZD3 PE=1 SV=1 | 535 | EPDFAQsLLRD | 0.32 | 0.012 | -0.576 | -0.081 |
| sp\|Q9NPG1\|FZD3_HUMAN Frizzled-3 OS=Homo sapiens GN=FZD3 PE=1 SV=1 | 542 | LLRDPNtPIIR | 0.351 | -0.007 | -0.456 | -0.037 |
| sp\|Q9NPG1\|FZD3_HUMAN Frizzled-3 OS=Homo sapiens GN=FZD3 PE=1 SV=1 | 548 | TPIIRKsRGTS | 0.288 | 0.057 | -0.631 | -0.095 |
| sp\|Q9NPG1\|FZD3_HUMAN Frizzled-3 OS=Homo sapiens GN=FZD3 PE=1 SV=1 | 551 | IRKSRGtSTQG | 0.422 | 0.16 | -0.405 | 0.059 |
| sp\|Q9NPG1\|FZD3_HUMAN Frizzled-3 OS=Homo sapiens GN=FZD3 PE=1 SV=1 | 552 | RKSRGTsTQGT | 0.642 | 0.767 | 0.437 | 0.615 |
| sp\|Q9NPG1\|FZD3_HUMAN Frizzled-3 OS=Homo sapiens GN=FZD3 PE=1 SV=1 | 553 | KSRGTStQGTS | 0.138 | 0.015 | -1.045 | -0.297 |
| sp\|Q9NPG1\|FZD3_HUMAN Frizzled-3 OS=Homo sapiens GN=FZD3 PE=1 SV=1 | 556 | GTSTQGtSTHA | 0.096 | -0.305 | -1.488 | -0.566 |
| sp\|Q9NPG1\|FZD3_HUMAN Frizzled-3 OS=Homo sapiens GN=FZD3 PE=1 SV=1 | 557 | TSTQGTsTHAS | 0.134 | -0.136 | -0.775 | -0.259 |
| sp\|Q9NPG1\|FZD3_HUMAN Frizzled-3 OS=Homo sapiens GN=FZD3 PE=1 SV=1 | 558 | STQGTStHASS | 0.036 | -0.228 | -1.732 | -0.641 |
| sp\|Q9NPG1\|FZD3_HUMAN Frizzled-3 OS=Homo sapiens GN=FZD3 PE=1 SV=1 | 561 | GTSTHAsSTQL | 0.398 | -0.018 | -0.751 | -0.124 |
| sp\|Q9NPG1\|FZD3_HUMAN Frizzled-3 OS=Homo sapiens GN=FZD3 PE=1 SV=1 | 562 | TSTHASsTQLA | 0.154 | -0.151 | -0.792 | -0.263 |
| sp\|Q9NPG1\|FZD3_HUMAN Frizzled-3 OS=Homo sapiens GN=FZD3 PE=1 SV=1 | 563 | STHASStQLAM | 0.108 | -0.07 | -1.23 | -0.397 |
| sp\|Q9NPG1\|FZD3_HUMAN Frizzled-3 OS=Homo sapiens GN=FZD3 PE=1 SV=1 | 573 | MVDDQRsKAGS | 0.069 | -0.135 | -1.442 | -0.503 |
| sp\|Q9NPG1\|FZD3_HUMAN Frizzled-3 OS=Homo sapiens GN=FZD3 PE=1 SV=1 | 577 | QRSKAGsIHSK | 0.202 | 0.071 | -0.74 | -0.156 |
| sp\|Q9NPG1\|FZD3_HUMAN Frizzled-3 OS=Homo sapiens GN=FZD3 PE=1 SV=1 | 580 | KAGSIHsKVSS | 0.061 | -0.152 | -1.274 | -0.455 |
| sp\|Q9NPG1\|FZD3_HUMAN Frizzled-3 OS=Homo sapiens GN=FZD3 PE=1 SV=1 | 583 | SIHSKVsSYHG | 0.083 | -0.25 | -1.401 | -0.523 |
| sp\|Q9NPG1\|FZD3_HUMAN Frizzled-3 OS=Homo sapiens GN=FZD3 PE=1 SV=1 | 584 | IHSKVSsYHGS | 0.165 | -0.202 | -1.166 | -0.401 |
| sp\|Q9NPG1\|FZD3_HUMAN Frizzled-3 OS=Homo sapiens GN=FZD3 PE=1 SV=1 | 588 | VSSYHGsLHRS | 0.245 | -0.143 | -0.839 | -0.246 |
| sp\|Q9NPG1\|FZD3_HUMAN Frizzled-3 OS=Homo sapiens GN=FZD3 PE=1 SV=1 | 592 | HGSLHRsRDGR | 0.187 | -0.07 | -0.812 | -0.232 |
| sp\|Q9NPG1\|FZD3_HUMAN Frizzled-3 OS=Homo sapiens GN=FZD3 PE=1 SV=1 | 598 | SRDGRYtPCSY | 0.071 | -0.073 | -1.287 | -0.43 |
| sp\|Q9NPG1\|FZD3_HUMAN Frizzled-3 OS=Homo sapiens GN=FZD3 PE=1 SV=1 | 601 | GRYTPCsYRGM | 0.23 | -0.028 | -0.751 | -0.183 |
| sp\|Q9NPG1\|FZD3_HUMAN Frizzled-3 OS=Homo sapiens GN=FZD3 PE=1 SV=1 | 613 | ERLPHGsMSRL | 0.108 | 0.097 | -1.177 | -0.324 |
| sp\|Q9NPG1\|FZD3_HUMAN Frizzled-3 OS=Homo sapiens GN=FZD3 PE=1 SV=1 | 615 | LPHGSMsRLTD | 0.208 | 0.11 | -0.62 | -0.101 |
| sp\|Q9NPG1\|FZD3_HUMAN Frizzled-3 OS=Homo sapiens GN=FZD3 PE=1 SV=1 | 618 | GSMSRLtDHSR | 0.204 | 0.051 | -0.891 | -0.212 |
| sp\|Q9NPG1\|FZD3_HUMAN Frizzled-3 OS=Homo sapiens GN=FZD3 PE=1 SV=1 | 621 | SRLTDHsRHSS | 0.117 | 0.056 | -1.287 | -0.371 |
| sp\|Q9NPG1\|FZD3_HUMAN Frizzled-3 OS=Homo sapiens GN=FZD3 PE=1 SV=1 | 624 | TDHSRHsSSHR | 0.074 | -0.133 | -1.226 | -0.428 |
| sp\|Q9NPG1\|FZD3_HUMAN Frizzled-3 OS=Homo sapiens GN=FZD3 PE=1 SV=1 | 625 | DHSRHSsSHRL | 0.546 | 0.718 | -0.437 | 0.276 |
| sp\|Q9NPG1\|FZD3_HUMAN Frizzled-3 OS=Homo sapiens GN=FZD3 PE=1 SV=1 | 626 | HSRHSSsHRLN | 0.484 | 0.328 | -0.007 | 0.268 |

| | | | | | | |
|---|---|---|---|---|---|---|
| sp\|Q9NPG1\|FZD3_HUMAN Frizzled-3 OS=Homo sapiens GN=FZD3 PE=1 SV=1 | 633 | HRLNEQsRHSS | 0.095 | 0.072 | -1.007 | -0.28 |
| sp\|Q9NPG1\|FZD3_HUMAN Frizzled-3 OS=Homo sapiens GN=FZD3 PE=1 SV=1 | 636 | NEQSRHsSIRD | 0.35 | 0.134 | -0.622 | -0.046 |
| sp\|Q9NPG1\|FZD3_HUMAN Frizzled-3 OS=Homo sapiens GN=FZD3 PE=1 SV=1 | 637 | EQSRHSsIRDL | 0.499 | 0.764 | 0.341 | 0.535 |
| sp\|Q9NPG1\|FZD3_HUMAN Frizzled-3 OS=Homo sapiens GN=FZD3 PE=1 SV=1 | 642 | SSIRDLsNNPM | 0.243 | 0.575 | -0.423 | 0.132 |
| sp\|Q9NPG1\|FZD3_HUMAN Frizzled-3 OS=Homo sapiens GN=FZD3 PE=1 SV=1 | 647 | LSNNPMtHITH | 0.085 | -0.217 | -0.974 | -0.369 |
| sp\|Q9NPG1\|FZD3_HUMAN Frizzled-3 OS=Homo sapiens GN=FZD3 PE=1 SV=1 | 650 | NPMTHItHGTS | 0.303 | 0.001 | -0.698 | -0.131 |
| sp\|Q9NPG1\|FZD3_HUMAN Frizzled-3 OS=Homo sapiens GN=FZD3 PE=1 SV=1 | 653 | THITHGtSMNR | 0.112 | -0.093 | -1.63 | -0.537 |
| sp\|Q9NPG1\|FZD3_HUMAN Frizzled-3 OS=Homo sapiens GN=FZD3 PE=1 SV=1 | 654 | HITHGTsMNRV | 0.182 | 0.082 | -0.854 | -0.197 |
| sp\|Q9NPG1\|FZD3_HUMAN Frizzled-3 OS=Homo sapiens GN=FZD3 PE=1 SV=1 | 664 | VIEEDGtSA-- | 0.067 | -0.124 | -1.511 | -0.523 |
| sp\|Q9NPG1\|FZD3_HUMAN Frizzled-3 OS=Homo sapiens GN=FZD3 PE=1 SV=1 | 665 | IEEDGTsA--- | 0.174 | 0.05 | -1.032 | -0.269 |
| sp\|Q9ULV1\|FZD4_HUMAN Frizzled-4 OS=Homo sapiens GN=FZD4 PE=1 SV=2 | 316 | YFFGMAsSIWW | 0.146 | -0.02 | -0.578 | -0.151 |
| sp\|Q9ULV1\|FZD4_HUMAN Frizzled-4 OS=Homo sapiens GN=FZD4 PE=1 SV=2 | 317 | FFGMASsIWWV | 0.232 | 0.036 | -0.744 | -0.159 |
| sp\|Q9ULV1\|FZD4_HUMAN Frizzled-4 OS=Homo sapiens GN=FZD4 PE=1 SV=2 | 324 | IWWVILtLTWF | 0.176 | 0.114 | -0.465 | -0.058 |
| sp\|Q9ULV1\|FZD4_HUMAN Frizzled-4 OS=Homo sapiens GN=FZD4 PE=1 SV=2 | 326 | WVILTLtWFLA | 0.069 | 0.031 | -0.992 | -0.297 |
| sp\|Q9ULV1\|FZD4_HUMAN Frizzled-4 OS=Homo sapiens GN=FZD4 PE=1 SV=2 | 344 | EAIEMHsSYFH | 0.042 | -0.253 | -1.665 | -0.625 |
| sp\|Q9ULV1\|FZD4_HUMAN Frizzled-4 OS=Homo sapiens GN=FZD4 PE=1 SV=2 | 345 | AIEMHSsYFHI | 0.195 | -0.11 | -0.663 | -0.193 |
| sp\|Q9ULV1\|FZD4_HUMAN Frizzled-4 OS=Homo sapiens GN=FZD4 PE=1 SV=2 | 359 | AIPAVKtIVIL | 0.219 | -0.037 | -0.722 | -0.18 |
| sp\|Q9ULV1\|FZD4_HUMAN Frizzled-4 OS=Homo sapiens GN=FZD4 PE=1 SV=2 | 374 | VDADELtGLCY | 0.049 | -0.245 | -1.618 | -0.605 |
| sp\|Q9ULV1\|FZD4_HUMAN Frizzled-4 OS=Homo sapiens GN=FZD4 PE=1 SV=2 | 388 | QNLDALtGFVV | 0.119 | -0.116 | -1.101 | -0.366 |
| sp\|Q9ULV1\|FZD4_HUMAN Frizzled-4 OS=Homo sapiens GN=FZD4 PE=1 SV=2 | 397 | VVAPLFtYLVI | 0.076 | -0.12 | -1.005 | -0.35 |
| sp\|Q9ULV1\|FZD4_HUMAN Frizzled-4 OS=Homo sapiens GN=FZD4 PE=1 SV=2 | 403 | TYLVIGtLFIA | 0.247 | -0.048 | -0.456 | -0.086 |
| sp\|Q9ULV1\|FZD4_HUMAN Frizzled-4 OS=Homo sapiens GN=FZD4 PE=1 SV=2 | 418 | ALFKIRsNLQK | 0.252 | -0.059 | -0.857 | -0.221 |
| sp\|Q9ULV1\|FZD4_HUMAN Frizzled-4 OS=Homo sapiens GN=FZD4 PE=1 SV=2 | 425 | NLQKDGtKTDK | 0.514 | 0.052 | -0.31 | 0.085 |
| sp\|Q9ULV1\|FZD4_HUMAN Frizzled-4 OS=Homo sapiens GN=FZD4 PE=1 SV=2 | 427 | QKDGTKtDKLE | 0.243 | 0.073 | -0.498 | -0.061 |
| sp\|Q9ULV1\|FZD4_HUMAN Frizzled-4 OS=Homo sapiens GN=FZD4 PE=1 SV=2 | 441 | VKIGVFsVLYT | 0.167 | -0.037 | -0.866 | -0.245 |
| sp\|Q9ULV1\|FZD4_HUMAN Frizzled-4 OS=Homo sapiens GN=FZD4 PE=1 SV=2 | 445 | VFSVLYtVPAT | 0.378 | 0.464 | 0.069 | 0.304 |
| sp\|Q9ULV1\|FZD4_HUMAN Frizzled-4 OS=Homo sapiens GN=FZD4 PE=1 SV=2 | 449 | LYTVPAtCVIA | 0.202 | -0.014 | -0.698 | -0.17 |
| sp\|Q9ULV1\|FZD4_HUMAN Frizzled-4 OS=Homo sapiens GN=FZD4 PE=1 SV=2 | 460 | CYFYEIsNWAL | 0.497 | 0.178 | -0.306 | 0.123 |
| sp\|Q9ULV1\|FZD4_HUMAN Frizzled-4 OS=Homo sapiens GN=FZD4 PE=1 SV=2 | 468 | WALFRYsADDS | 0.495 | 0.259 | -0.101 | 0.218 |
| sp\|Q9ULV1\|FZD4_HUMAN Frizzled-4 OS=Homo sapiens GN=FZD4 PE=1 SV=2 | 472 | RYSADDsNMAV | 0.242 | -0.099 | -1.173 | -0.343 |
| sp\|Q9ULV1\|FZD4_HUMAN Frizzled-4 OS=Homo sapiens GN=FZD4 PE=1 SV=2 | 484 | MLKIFMsLLVG | 0.633 | 0.418 | 0.109 | 0.387 |
| sp\|Q9ULV1\|FZD4_HUMAN Frizzled-4 OS=Homo sapiens GN=FZD4 PE=1 SV=2 | 490 | SLLVGItSGMW | 0.091 | -0.138 | -1.195 | -0.414 |
| sp\|Q9ULV1\|FZD4_HUMAN Frizzled-4 OS=Homo sapiens GN=FZD4 PE=1 SV=2 | 491 | LLVGITsGMWI | 0.252 | 0.077 | -0.17 | 0.053 |
| sp\|Q9ULV1\|FZD4_HUMAN Frizzled-4 OS=Homo sapiens GN=FZD4 PE=1 SV=2 | 497 | SGMWIWsAKTL | 0.149 | -0.104 | -0.761 | -0.239 |
| sp\|Q9ULV1\|FZD4_HUMAN Frizzled-4 OS=Homo sapiens GN=FZD4 PE=1 SV=2 | 500 | WIWSAKtLHTW | 0.508 | 0.312 | 0.118 | 0.313 |
| sp\|Q9ULV1\|FZD4_HUMAN Frizzled-4 OS=Homo sapiens GN=FZD4 PE=1 SV=2 | 503 | SAKTLHtWQKC | 0.11 | -0.053 | -1.364 | -0.436 |
| sp\|Q9ULV1\|FZD4_HUMAN Frizzled-4 OS=Homo sapiens GN=FZD4 PE=1 SV=2 | 508 | HTWQKCsNRLV | 0.196 | 0.003 | -1.149 | -0.317 |
| sp\|Q9ULV1\|FZD4_HUMAN Frizzled-4 OS=Homo sapiens GN=FZD4 PE=1 SV=2 | 514 | SNRLVNsGKVK | 0.223 | 0.016 | -0.902 | -0.221 |
| sp\|Q9ULV1\|FZD4_HUMAN Frizzled-4 OS=Homo sapiens GN=FZD4 PE=1 SV=2 | 533 | VKPGKGsETVV | 0.134 | -0.101 | -1.243 | -0.403 |
| sp\|Q9ULV1\|FZD4_HUMAN Frizzled-4 OS=Homo sapiens GN=FZD4 PE=1 SV=2 | 535 | PGKGSEtVV-- | 0.354 | 0.319 | -0.229 | 0.148 |
| sp\|Q13467\|FZD5_HUMAN Frizzled-5 OS=Homo sapiens GN=FZD5 PE=1 SV=2 | 309 | NHIHYEtTGPA | 0.161 | -0.129 | -1.184 | -0.384 |
| sp\|Q13467\|FZD5_HUMAN Frizzled-5 OS=Homo sapiens GN=FZD5 PE=1 SV=2 | 310 | HIHYETtGPAL | 0.489 | 0.617 | 0.245 | 0.45 |
| sp\|Q13467\|FZD5_HUMAN Frizzled-5 OS=Homo sapiens GN=FZD5 PE=1 SV=2 | 316 | TGPALCtIVFL | 0.06 | -0.289 | -1.497 | -0.575 |
| sp\|Q13467\|FZD5_HUMAN Frizzled-5 OS=Homo sapiens GN=FZD5 PE=1 SV=2 | 329 | YFFGMAsSIWW | 0.146 | -0.02 | -0.578 | -0.151 |
| sp\|Q13467\|FZD5_HUMAN Frizzled-5 OS=Homo sapiens GN=FZD5 PE=1 SV=2 | 330 | FFGMASsIWWV | 0.232 | 0.036 | -0.744 | -0.159 |
| sp\|Q13467\|FZD5_HUMAN Frizzled-5 OS=Homo sapiens GN=FZD5 PE=1 SV=2 | 337 | IWWVILsLTWF | 0.176 | 0.114 | -0.465 | -0.058 |
| sp\|Q13467\|FZD5_HUMAN Frizzled-5 OS=Homo sapiens GN=FZD5 PE=1 SV=2 | 339 | WVILSLtWFLA | 0.185 | 0.223 | -0.439 | -0.01 |
| sp\|Q13467\|FZD5_HUMAN Frizzled-5 OS=Homo sapiens GN=FZD5 PE=1 SV=2 | 369 | AAWLIPsVKSI | 0.094 | -0.066 | -0.943 | -0.305 |
| sp\|Q13467\|FZD5_HUMAN Frizzled-5 OS=Homo sapiens GN=FZD5 PE=1 SV=2 | 372 | LIPSVKsITAL | 0.237 | 0.027 | -0.475 | -0.07 |

| | | | | | | |
|---|---|---|---|---|---|---|
| sp\|Q13467\|FZD5_HUMAN Frizzled-5 OS=Homo sapiens GN=FZD5 PE=1 SV=2 | 374 | PSVKSItALAL | 0.545 | 0.323 | -0.032 | 0.279 |
| sp\|Q13467\|FZD5_HUMAN Frizzled-5 OS=Homo sapiens GN=FZD5 PE=1 SV=2 | 379 | ITALALsSVDG | 0.28 | -0.009 | -0.637 | -0.122 |
| sp\|Q13467\|FZD5_HUMAN Frizzled-5 OS=Homo sapiens GN=FZD5 PE=1 SV=2 | 380 | TALALSsVDGD | 0.126 | -0.23 | -0.939 | -0.348 |
| sp\|Q13467\|FZD5_HUMAN Frizzled-5 OS=Homo sapiens GN=FZD5 PE=1 SV=2 | 399 | GNQNLNsLRGF | 0.288 | -0.056 | -0.451 | -0.073 |
| sp\|Q13467\|FZD5_HUMAN Frizzled-5 OS=Homo sapiens GN=FZD5 PE=1 SV=2 | 416 | LYLLVGtLFLL | 0.311 | 0.081 | -0.425 | -0.011 |
| sp\|Q13467\|FZD5_HUMAN Frizzled-5 OS=Homo sapiens GN=FZD5 PE=1 SV=2 | 425 | LLAGFVsLFRI | 0.652 | 0.133 | 0.237 | 0.341 |
| sp\|Q13467\|FZD5_HUMAN Frizzled-5 OS=Homo sapiens GN=FZD5 PE=1 SV=2 | 431 | SLFRIRsVIKQ | 0.402 | 0.823 | -0.024 | 0.4 |
| sp\|Q13467\|FZD5_HUMAN Frizzled-5 OS=Homo sapiens GN=FZD5 PE=1 SV=2 | 438 | VIKQGGtKTDK | 0.256 | -0.11 | -0.586 | -0.147 |
| sp\|Q13467\|FZD5_HUMAN Frizzled-5 OS=Homo sapiens GN=FZD5 PE=1 SV=2 | 440 | KQGGTKtDKLE | 0.099 | -0.029 | -0.846 | -0.259 |
| sp\|Q13467\|FZD5_HUMAN Frizzled-5 OS=Homo sapiens GN=FZD5 PE=1 SV=2 | 454 | IRIGIFtLLYT | 0.264 | 0.23 | -0.272 | 0.074 |
| sp\|Q13467\|FZD5_HUMAN Frizzled-5 OS=Homo sapiens GN=FZD5 PE=1 SV=2 | 458 | IFTLLYtVPAS | 0.597 | 0.587 | 0.409 | 0.531 |
| sp\|Q13467\|FZD5_HUMAN Frizzled-5 OS=Homo sapiens GN=FZD5 PE=1 SV=2 | 462 | LYTVPAsIVVA | 0.126 | -0.105 | -0.798 | -0.259 |
| sp\|Q13467\|FZD5_HUMAN Frizzled-5 OS=Homo sapiens GN=FZD5 PE=1 SV=2 | 477 | EQHYREsWEAA | 0.159 | 0.152 | -0.559 | -0.083 |
| sp\|Q13467\|FZD5_HUMAN Frizzled-5 OS=Homo sapiens GN=FZD5 PE=1 SV=2 | 483 | SWEAALtCACP | 0.084 | -0.073 | -1.284 | -0.424 |
| sp\|Q13467\|FZD5_HUMAN Frizzled-5 OS=Homo sapiens GN=FZD5 PE=1 SV=2 | 491 | ACPGHDtGQPR | 0.141 | -0.207 | -1.209 | -0.425 |
| sp\|Q13467\|FZD5_HUMAN Frizzled-5 OS=Homo sapiens GN=FZD5 PE=1 SV=2 | 516 | CLVVGItSGVW | 0.178 | 0.05 | -0.744 | -0.172 |
| sp\|Q13467\|FZD5_HUMAN Frizzled-5 OS=Homo sapiens GN=FZD5 PE=1 SV=2 | 517 | LVVGITsGVWI | 0.115 | -0.023 | -0.502 | -0.137 |
| sp\|Q13467\|FZD5_HUMAN Frizzled-5 OS=Homo sapiens GN=FZD5 PE=1 SV=2 | 523 | SGVWIWsGKTV | 0.113 | -0.28 | -1.109 | -0.425 |
| sp\|Q13467\|FZD5_HUMAN Frizzled-5 OS=Homo sapiens GN=FZD5 PE=1 SV=2 | 526 | WIWSGKtVESW | 0.068 | 0.032 | -0.924 | -0.275 |
| sp\|Q13467\|FZD5_HUMAN Frizzled-5 OS=Homo sapiens GN=FZD5 PE=1 SV=2 | 529 | SGKTVEsWRRF | 0.118 | -0.231 | -1.324 | -0.479 |
| sp\|Q13467\|FZD5_HUMAN Frizzled-5 OS=Homo sapiens GN=FZD5 PE=1 SV=2 | 534 | ESWRRFtSRCC | 0.511 | 0.952 | 0.137 | 0.533 |
| sp\|Q13467\|FZD5_HUMAN Frizzled-5 OS=Homo sapiens GN=FZD5 PE=1 SV=2 | 535 | SWRRFTsRCCC | 0.514 | 0.842 | -0.303 | 0.351 |
| sp\|Q13467\|FZD5_HUMAN Frizzled-5 OS=Homo sapiens GN=FZD5 PE=1 SV=2 | 547 | PRRGHKsGGAM | 0.746 | 0.63 | 0.65 | 0.675 |
| sp\|Q13467\|FZD5_HUMAN Frizzled-5 OS=Homo sapiens GN=FZD5 PE=1 SV=2 | 560 | GDYPEAsAALT | 0.092 | -0.204 | -1.383 | -0.498 |
| sp\|Q13467\|FZD5_HUMAN Frizzled-5 OS=Homo sapiens GN=FZD5 PE=1 SV=2 | 564 | EASAALtGRTG | 0.11 | -0.267 | -1.014 | -0.39 |
| sp\|Q13467\|FZD5_HUMAN Frizzled-5 OS=Homo sapiens GN=FZD5 PE=1 SV=2 | 567 | AALTGRtGPPG | 0.213 | 0.163 | -0.78 | -0.135 |
| sp\|Q13467\|FZD5_HUMAN Frizzled-5 OS=Homo sapiens GN=FZD5 PE=1 SV=2 | 575 | PPGPAAtYHKQ | 0.144 | -0.125 | -1.12 | -0.367 |
| sp\|Q13467\|FZD5_HUMAN Frizzled-5 OS=Homo sapiens GN=FZD5 PE=1 SV=2 | 581 | TYHKQVsLSHV | 0.288 | -0.032 | -0.771 | -0.172 |
| sp\|Q13467\|FZD5_HUMAN Frizzled-5 OS=Homo sapiens GN=FZD5 PE=1 SV=2 | 583 | HKQVSLsHV-- | 0.354 | 0.448 | -0.199 | 0.201 |
| sp\|O60353\|FZD6_HUMAN Frizzled-6 OS=Homo sapiens GN=FZD6 PE=1 SV=2 | 316 | AAGRKWsCEAI | 0.555 | 0.843 | 0.205 | 0.534 |
| sp\|O60353\|FZD6_HUMAN Frizzled-6 OS=Homo sapiens GN=FZD6 PE=1 SV=2 | 334 | HAVAWGtPGFL | 0.062 | -0.373 | -1.352 | -0.554 |
| sp\|O60353\|FZD6_HUMAN Frizzled-6 OS=Homo sapiens GN=FZD6 PE=1 SV=2 | 339 | GTPGFLtVMLL | 0.12 | -0.068 | -1.168 | -0.372 |
| sp\|O60353\|FZD6_HUMAN Frizzled-6 OS=Homo sapiens GN=FZD6 PE=1 SV=2 | 354 | VEGDNIsGVCF | 0.09 | -0.147 | -1.24 | -0.432 |
| sp\|O60353\|FZD6_HUMAN Frizzled-6 OS=Homo sapiens GN=FZD6 PE=1 SV=2 | 367 | LYDLDAsRYFV | 0.146 | -0.187 | -1.456 | -0.499 |
| sp\|O60353\|FZD6_HUMAN Frizzled-6 OS=Homo sapiens GN=FZD6 PE=1 SV=2 | 384 | CVFVGLsLLLA | 0.086 | -0.058 | -0.927 | -0.3 |
| sp\|O60353\|FZD6_HUMAN Frizzled-6 OS=Homo sapiens GN=FZD6 PE=1 SV=2 | 392 | LLAGIIsLNHV | 0.416 | 0.117 | 0.112 | 0.215 |
| sp\|O60353\|FZD6_HUMAN Frizzled-6 OS=Homo sapiens GN=FZD6 PE=1 SV=2 | 421 | IRIGVFsGLYL | 0.164 | 0.164 | -0.77 | -0.147 |
| sp\|O60353\|FZD6_HUMAN Frizzled-6 OS=Homo sapiens GN=FZD6 PE=1 SV=2 | 430 | YLVPLVtLLGC | 0.269 | -0.075 | -0.659 | -0.155 |
| sp\|O60353\|FZD6_HUMAN Frizzled-6 OS=Homo sapiens GN=FZD6 PE=1 SV=2 | 444 | EQVNRItWEIT | 0.161 | 0.091 | -0.551 | -0.1 |
| sp\|O60353\|FZD6_HUMAN Frizzled-6 OS=Homo sapiens GN=FZD6 PE=1 SV=2 | 448 | RITWEItWVSD | 0.243 | 0.085 | -0.616 | -0.096 |
| sp\|O60353\|FZD6_HUMAN Frizzled-6 OS=Homo sapiens GN=FZD6 PE=1 SV=2 | 451 | WEITWVsDHCR | 0.415 | 0.076 | -0.635 | -0.048 |
| sp\|O60353\|FZD6_HUMAN Frizzled-6 OS=Homo sapiens GN=FZD6 PE=1 SV=2 | 483 | MIKYLMtLIVG | 0.592 | 0.421 | 0.285 | 0.433 |
| sp\|O60353\|FZD6_HUMAN Frizzled-6 OS=Homo sapiens GN=FZD6 PE=1 SV=2 | 489 | TLIVGIsAVFW | 0.073 | -0.067 | -1.334 | -0.443 |
| sp\|O60353\|FZD6_HUMAN Frizzled-6 OS=Homo sapiens GN=FZD6 PE=1 SV=2 | 496 | AVFWVGsKKTC | 0.097 | -0.294 | -1.316 | -0.504 |
| sp\|O60353\|FZD6_HUMAN Frizzled-6 OS=Homo sapiens GN=FZD6 PE=1 SV=2 | 499 | WVGSKKtCTEW | 0.135 | -0.037 | -0.858 | -0.253 |
| sp\|O60353\|FZD6_HUMAN Frizzled-6 OS=Homo sapiens GN=FZD6 PE=1 SV=2 | 501 | GSKKTCtEWAG | 0.691 | 0.294 | -0.015 | 0.323 |
| sp\|O60353\|FZD6_HUMAN Frizzled-6 OS=Homo sapiens GN=FZD6 PE=1 SV=2 | 517 | RKRDPIsESRR | 0.565 | 0.205 | -0.271 | 0.166 |
| sp\|O60353\|FZD6_HUMAN Frizzled-6 OS=Homo sapiens GN=FZD6 PE=1 SV=2 | 519 | RDPISEsRRVL | 0.144 | -0.039 | -1.05 | -0.315 |
| sp\|O60353\|FZD6_HUMAN Frizzled-6 OS=Homo sapiens GN=FZD6 PE=1 SV=2 | 526 | RRVLQEsCEFF | 0.084 | 0.133 | -1.412 | -0.398 |

| | | | | | | |
|---|---|---|---|---|---|---|
| sp\|O60353\|FZD6_HUMAN Frizzled-6 OS=Homo sapiens GN=FZD6 PE=1 SV=2 | 535 | FFLKHNsKVKH | 0.395 | 0.079 | -0.534 | -0.02 |
| sp\|O60353\|FZD6_HUMAN Frizzled-6 OS=Homo sapiens GN=FZD6 PE=1 SV=2 | 547 | KKHYKPsSHKL | 0.123 | -0.119 | -1.059 | -0.352 |
| sp\|O60353\|FZD6_HUMAN Frizzled-6 OS=Homo sapiens GN=FZD6 PE=1 SV=2 | 548 | KHYKPSsHKLK | 0.111 | -0.227 | -1.196 | -0.437 |
| sp\|O60353\|FZD6_HUMAN Frizzled-6 OS=Homo sapiens GN=FZD6 PE=1 SV=2 | 555 | HKLKVIsKSMG | 0.415 | 0.024 | -0.32 | 0.04 |
| sp\|O60353\|FZD6_HUMAN Frizzled-6 OS=Homo sapiens GN=FZD6 PE=1 SV=2 | 557 | LKVISKsMGTS | 0.578 | 0.434 | 0.196 | 0.403 |
| sp\|O60353\|FZD6_HUMAN Frizzled-6 OS=Homo sapiens GN=FZD6 PE=1 SV=2 | 560 | ISKSMGtSTGA | 0.162 | -0.388 | -0.936 | -0.387 |
| sp\|O60353\|FZD6_HUMAN Frizzled-6 OS=Homo sapiens GN=FZD6 PE=1 SV=2 | 561 | SKSMGTsTGAT | 0.193 | -0.108 | -0.668 | -0.194 |
| sp\|O60353\|FZD6_HUMAN Frizzled-6 OS=Homo sapiens GN=FZD6 PE=1 SV=2 | 562 | KSMGTStGATA | 0.039 | -0.298 | -1.392 | -0.55 |
| sp\|O60353\|FZD6_HUMAN Frizzled-6 OS=Homo sapiens GN=FZD6 PE=1 SV=2 | 565 | GTSTGAtANHG | 0.217 | -0.088 | -0.927 | -0.266 |
| sp\|O60353\|FZD6_HUMAN Frizzled-6 OS=Homo sapiens GN=FZD6 PE=1 SV=2 | 570 | ATANHGtSAVA | 0.097 | -0.226 | -1.201 | -0.443 |
| sp\|O60353\|FZD6_HUMAN Frizzled-6 OS=Homo sapiens GN=FZD6 PE=1 SV=2 | 571 | TANHGTsAVAI | 0.141 | -0.055 | -0.9 | -0.271 |
| sp\|O60353\|FZD6_HUMAN Frizzled-6 OS=Homo sapiens GN=FZD6 PE=1 SV=2 | 576 | TSAVAItSHDY | 0.323 | -0.005 | -0.593 | -0.092 |
| sp\|O60353\|FZD6_HUMAN Frizzled-6 OS=Homo sapiens GN=FZD6 PE=1 SV=2 | 577 | SAVAITsHDYL | 0.157 | -0.122 | -0.893 | -0.286 |
| sp\|O60353\|FZD6_HUMAN Frizzled-6 OS=Homo sapiens GN=FZD6 PE=1 SV=2 | 585 | DYLGQEtLTEI | 0.508 | 0.093 | -0.399 | 0.067 |
| sp\|O60353\|FZD6_HUMAN Frizzled-6 OS=Homo sapiens GN=FZD6 PE=1 SV=2 | 587 | LGQETLtEIQT | 0.054 | -0.046 | -0.946 | -0.313 |
| sp\|O60353\|FZD6_HUMAN Frizzled-6 OS=Homo sapiens GN=FZD6 PE=1 SV=2 | 591 | TLTEIQtSPET | 0.463 | 0.459 | 0.085 | 0.336 |
| sp\|O60353\|FZD6_HUMAN Frizzled-6 OS=Homo sapiens GN=FZD6 PE=1 SV=2 | 592 | LTEIQTsPETS | 0.043 | -0.438 | -1.615 | -0.67 |
| sp\|O60353\|FZD6_HUMAN Frizzled-6 OS=Homo sapiens GN=FZD6 PE=1 SV=2 | 595 | IQTSPEtSMRE | 0.093 | -0.393 | -1.302 | -0.534 |
| sp\|O60353\|FZD6_HUMAN Frizzled-6 OS=Homo sapiens GN=FZD6 PE=1 SV=2 | 596 | QTSPETsMREV | 0.177 | -0.136 | -1.264 | -0.408 |
| sp\|O60353\|FZD6_HUMAN Frizzled-6 OS=Homo sapiens GN=FZD6 PE=1 SV=2 | 606 | VKADGAsTPRL | 0.622 | 0.429 | -0.042 | 0.336 |
| sp\|O60353\|FZD6_HUMAN Frizzled-6 OS=Homo sapiens GN=FZD6 PE=1 SV=2 | 607 | KADGAStPRLR | 0.059 | -0.476 | -1.439 | -0.619 |
| sp\|O60353\|FZD6_HUMAN Frizzled-6 OS=Homo sapiens GN=FZD6 PE=1 SV=2 | 620 | DCGEPAsPAAS | 0.064 | -0.399 | -1.631 | -0.655 |
| sp\|O60353\|FZD6_HUMAN Frizzled-6 OS=Homo sapiens GN=FZD6 PE=1 SV=2 | 624 | PASPAAsISRL | 0.159 | -0.177 | -1.002 | -0.34 |
| sp\|O60353\|FZD6_HUMAN Frizzled-6 OS=Homo sapiens GN=FZD6 PE=1 SV=2 | 626 | SPAASIsRLSG | 0.111 | -0.112 | -1.094 | -0.365 |
| sp\|O60353\|FZD6_HUMAN Frizzled-6 OS=Homo sapiens GN=FZD6 PE=1 SV=2 | 629 | ASISRLsGEQV | 0.122 | -0.15 | -1.27 | -0.433 |
| sp\|O60353\|FZD6_HUMAN Frizzled-6 OS=Homo sapiens GN=FZD6 PE=1 SV=2 | 641 | GKGQAGsVSES | 0.279 | 0.009 | -0.695 | -0.136 |
| sp\|O60353\|FZD6_HUMAN Frizzled-6 OS=Homo sapiens GN=FZD6 PE=1 SV=2 | 643 | GQAGSVsESAR | 0.521 | 0.31 | 0.284 | 0.372 |
| sp\|O60353\|FZD6_HUMAN Frizzled-6 OS=Homo sapiens GN=FZD6 PE=1 SV=2 | 645 | AGSVSEsARSE | 0.128 | -0.086 | -1.111 | -0.356 |
| sp\|O60353\|FZD6_HUMAN Frizzled-6 OS=Homo sapiens GN=FZD6 PE=1 SV=2 | 648 | VSESARsEGRI | 0.343 | -0.039 | -0.616 | -0.104 |
| sp\|O60353\|FZD6_HUMAN Frizzled-6 OS=Homo sapiens GN=FZD6 PE=1 SV=2 | 653 | RSEGRIsPKSD | 0.137 | -0.261 | -0.895 | -0.34 |
| sp\|O60353\|FZD6_HUMAN Frizzled-6 OS=Homo sapiens GN=FZD6 PE=1 SV=2 | 656 | GRISPKsDITD | 0.354 | 0.21 | -0.285 | 0.093 |
| sp\|O60353\|FZD6_HUMAN Frizzled-6 OS=Homo sapiens GN=FZD6 PE=1 SV=2 | 659 | SPKSDItDTGL | 0.199 | -0.183 | -1.017 | -0.334 |
| sp\|O60353\|FZD6_HUMAN Frizzled-6 OS=Homo sapiens GN=FZD6 PE=1 SV=2 | 661 | KSDITDtGLAQ | 0.052 | -0.266 | -1.589 | -0.601 |
| sp\|O60353\|FZD6_HUMAN Frizzled-6 OS=Homo sapiens GN=FZD6 PE=1 SV=2 | 666 | DTGLAQsNNLQ | 0.237 | -0.05 | -1.035 | -0.283 |
| sp\|O60353\|FZD6_HUMAN Frizzled-6 OS=Homo sapiens GN=FZD6 PE=1 SV=2 | 673 | NNLQVPsSSEP | 0.084 | -0.333 | -1.234 | -0.494 |
| sp\|O60353\|FZD6_HUMAN Frizzled-6 OS=Homo sapiens GN=FZD6 PE=1 SV=2 | 674 | NLQVPSsSEPS | 0.063 | -0.194 | -1.3 | -0.477 |
| sp\|O60353\|FZD6_HUMAN Frizzled-6 OS=Homo sapiens GN=FZD6 PE=1 SV=2 | 675 | LQVPSSsEPSS | 0.311 | 0.534 | -0.028 | 0.272 |
| sp\|O60353\|FZD6_HUMAN Frizzled-6 OS=Homo sapiens GN=FZD6 PE=1 SV=2 | 678 | PSSSEPsSLKG | 0.066 | -0.363 | -1.615 | -0.637 |
| sp\|O60353\|FZD6_HUMAN Frizzled-6 OS=Homo sapiens GN=FZD6 PE=1 SV=2 | 679 | SSSEPSsLKGS | 0.051 | -0.363 | -1.311 | -0.541 |
| sp\|O60353\|FZD6_HUMAN Frizzled-6 OS=Homo sapiens GN=FZD6 PE=1 SV=2 | 683 | PSSLKGsTSLL | 0.054 | -0.267 | -1.511 | -0.575 |
| sp\|O60353\|FZD6_HUMAN Frizzled-6 OS=Homo sapiens GN=FZD6 PE=1 SV=2 | 684 | SSLKGStSLLV | 0.058 | -0.294 | -1.741 | -0.659 |
| sp\|O60353\|FZD6_HUMAN Frizzled-6 OS=Homo sapiens GN=FZD6 PE=1 SV=2 | 685 | SLKGSTsLLVH | 0.408 | 0.236 | -0.203 | 0.147 |
| sp\|O60353\|FZD6_HUMAN Frizzled-6 OS=Homo sapiens GN=FZD6 PE=1 SV=2 | 692 | LLVHPVsGVRK | 0.206 | -0.224 | -0.761 | -0.26 |
| sp\|O60353\|FZD6_HUMAN Frizzled-6 OS=Homo sapiens GN=FZD6 PE=1 SV=2 | 704 | QGGGCHsDT-- | 0.328 | 0.21 | -0.592 | -0.018 |
| sp\|O60353\|FZD6_HUMAN Frizzled-6 OS=Homo sapiens GN=FZD6 PE=1 SV=2 | 706 | GGCHSDt---- | 0.415 | 0.248 | -0.439 | 0.075 |
| sp\|O75084\|FZD7_HUMAN Frizzled-7 OS=Homo sapiens GN=FZD7 PE=1 SV=2 | 320 | VCVERFsDDGY | 0.451 | 0.218 | -0.482 | 0.062 |
| sp\|O75084\|FZD7_HUMAN Frizzled-7 OS=Homo sapiens GN=FZD7 PE=1 SV=2 | 326 | SDDGYRtVAQG | 0.056 | -0.288 | -1.546 | -0.593 |
| sp\|O75084\|FZD7_HUMAN Frizzled-7 OS=Homo sapiens GN=FZD7 PE=1 SV=2 | 331 | RTVAQGtKKEG | 0.133 | -0.151 | -1.187 | -0.402 |
| sp\|O75084\|FZD7_HUMAN Frizzled-7 OS=Homo sapiens GN=FZD7 PE=1 SV=2 | 337 | TKKEGCtILFM | 0.091 | -0.189 | -1.201 | -0.433 |

| | | | | | | |
|---|---|---|---|---|---|---|
| sp\|O75084\|FZD7_HUMAN Frizzled-7 OS=Homo sapiens GN=FZD7 PE=1 SV=2 | 350 | YFFGMAsSIWW | 0.146 | -0.02 | -0.578 | -0.151 |
| sp\|O75084\|FZD7_HUMAN Frizzled-7 OS=Homo sapiens GN=FZD7 PE=1 SV=2 | 351 | FFGMASsIWWV | 0.232 | 0.036 | -0.744 | -0.159 |
| sp\|O75084\|FZD7_HUMAN Frizzled-7 OS=Homo sapiens GN=FZD7 PE=1 SV=2 | 358 | IWWVILsLTWF | 0.176 | 0.114 | -0.465 | -0.058 |
| sp\|O75084\|FZD7_HUMAN Frizzled-7 OS=Homo sapiens GN=FZD7 PE=1 SV=2 | 360 | WVILSLtWFLA | 0.185 | 0.223 | -0.439 | -0.01 |
| sp\|O75084\|FZD7_HUMAN Frizzled-7 OS=Homo sapiens GN=FZD7 PE=1 SV=2 | 378 | EAIEANsQYFH | 0.064 | -0.214 | -1.727 | -0.626 |
| sp\|O75084\|FZD7_HUMAN Frizzled-7 OS=Homo sapiens GN=FZD7 PE=1 SV=2 | 393 | AVPAVKtITIL | 0.211 | -0.125 | -0.808 | -0.241 |
| sp\|O75084\|FZD7_HUMAN Frizzled-7 OS=Homo sapiens GN=FZD7 PE=1 SV=2 | 395 | PAVKTItILAM | 0.198 | 0.005 | -0.632 | -0.143 |
| sp\|O75084\|FZD7_HUMAN Frizzled-7 OS=Homo sapiens GN=FZD7 PE=1 SV=2 | 408 | VDGDLLsGVCY | 0.037 | -0.245 | -1.762 | -0.657 |
| sp\|O75084\|FZD7_HUMAN Frizzled-7 OS=Homo sapiens GN=FZD7 PE=1 SV=2 | 416 | VCYVGLsSVDA | 0.062 | -0.174 | -1.369 | -0.494 |
| sp\|O75084\|FZD7_HUMAN Frizzled-7 OS=Homo sapiens GN=FZD7 PE=1 SV=2 | 417 | CYVGLSsVDAL | 0.208 | 0.095 | -0.512 | -0.07 |
| sp\|O75084\|FZD7_HUMAN Frizzled-7 OS=Homo sapiens GN=FZD7 PE=1 SV=2 | 437 | VYLFIGtSFLL | 0.162 | -0.052 | -0.753 | -0.214 |
| sp\|O75084\|FZD7_HUMAN Frizzled-7 OS=Homo sapiens GN=FZD7 PE=1 SV=2 | 438 | YLFIGTsFLLA | 0.19 | 0.073 | -0.576 | -0.104 |
| sp\|O75084\|FZD7_HUMAN Frizzled-7 OS=Homo sapiens GN=FZD7 PE=1 SV=2 | 446 | LLAGFVsLFRI | 0.652 | 0.133 | 0.237 | 0.341 |
| sp\|O75084\|FZD7_HUMAN Frizzled-7 OS=Homo sapiens GN=FZD7 PE=1 SV=2 | 452 | SLFRIRtIMKH | 0.33 | 0.697 | -0.347 | 0.227 |
| sp\|O75084\|FZD7_HUMAN Frizzled-7 OS=Homo sapiens GN=FZD7 PE=1 SV=2 | 459 | IMKHDGtKTEK | 0.226 | -0.094 | -0.762 | -0.21 |
| sp\|O75084\|FZD7_HUMAN Frizzled-7 OS=Homo sapiens GN=FZD7 PE=1 SV=2 | 461 | KHDGTKtEKLE | 0.119 | 0.008 | -1.009 | -0.294 |
| sp\|O75084\|FZD7_HUMAN Frizzled-7 OS=Homo sapiens GN=FZD7 PE=1 SV=2 | 475 | VRIGVFsVLYT | 0.122 | 0.109 | -0.969 | -0.246 |
| sp\|O75084\|FZD7_HUMAN Frizzled-7 OS=Homo sapiens GN=FZD7 PE=1 SV=2 | 479 | VFSVLYtVPAT | 0.378 | 0.464 | 0.069 | 0.304 |
| sp\|O75084\|FZD7_HUMAN Frizzled-7 OS=Homo sapiens GN=FZD7 PE=1 SV=2 | 483 | LYTVPAtIVLA | 0.128 | -0.117 | -0.782 | -0.257 |
| sp\|O75084\|FZD7_HUMAN Frizzled-7 OS=Homo sapiens GN=FZD7 PE=1 SV=2 | 502 | REHWERtWLLQ | 0.073 | -0.121 | -1.652 | -0.567 |
| sp\|O75084\|FZD7_HUMAN Frizzled-7 OS=Homo sapiens GN=FZD7 PE=1 SV=2 | 507 | RTWLLQtCKSY | 0.122 | 0.109 | -1.191 | -0.32 |
| sp\|O75084\|FZD7_HUMAN Frizzled-7 OS=Homo sapiens GN=FZD7 PE=1 SV=2 | 510 | LLQTCKsYAVP | 0.294 | 0.041 | -0.487 | -0.051 |
| sp\|O75084\|FZD7_HUMAN Frizzled-7 OS=Homo sapiens GN=FZD7 PE=1 SV=2 | 524 | GHFPPMsPDFT | 0.08 | -0.369 | -1.421 | -0.57 |
| sp\|O75084\|FZD7_HUMAN Frizzled-7 OS=Homo sapiens GN=FZD7 PE=1 SV=2 | 528 | PMSPDFtVFMI | 0.189 | 0.035 | -0.597 | -0.124 |
| sp\|O75084\|FZD7_HUMAN Frizzled-7 OS=Homo sapiens GN=FZD7 PE=1 SV=2 | 537 | MIKYLMtMIVG | 0.407 | 0.427 | -0.152 | 0.227 |
| sp\|O75084\|FZD7_HUMAN Frizzled-7 OS=Homo sapiens GN=FZD7 PE=1 SV=2 | 543 | TMIVGItTGFW | 0.051 | -0.069 | -1.399 | -0.472 |
| sp\|O75084\|FZD7_HUMAN Frizzled-7 OS=Homo sapiens GN=FZD7 PE=1 SV=2 | 544 | MIVGITtGFWI | 0.289 | 0.158 | -0.077 | 0.123 |
| sp\|O75084\|FZD7_HUMAN Frizzled-7 OS=Homo sapiens GN=FZD7 PE=1 SV=2 | 550 | TGFWIWsGKTL | 0.168 | -0.191 | -0.595 | -0.206 |
| sp\|O75084\|FZD7_HUMAN Frizzled-7 OS=Homo sapiens GN=FZD7 PE=1 SV=2 | 553 | WIWSGKtLQSW | 0.165 | 0.139 | -0.424 | -0.04 |
| sp\|O75084\|FZD7_HUMAN Frizzled-7 OS=Homo sapiens GN=FZD7 PE=1 SV=2 | 556 | SGKTLQsWRRF | 0.095 | -0.209 | -1.234 | -0.449 |
| sp\|O75084\|FZD7_HUMAN Frizzled-7 OS=Homo sapiens GN=FZD7 PE=1 SV=2 | 565 | RFYHRLsHSSK | 0.131 | -0.038 | -0.876 | -0.261 |
| sp\|O75084\|FZD7_HUMAN Frizzled-7 OS=Homo sapiens GN=FZD7 PE=1 SV=2 | 567 | YHRLSHsSKGE | 0.516 | 0.416 | -0.217 | 0.238 |
| sp\|O75084\|FZD7_HUMAN Frizzled-7 OS=Homo sapiens GN=FZD7 PE=1 SV=2 | 568 | HRLSHSsKGET | 0.248 | 0.184 | -0.438 | -0.002 |
| sp\|O75084\|FZD7_HUMAN Frizzled-7 OS=Homo sapiens GN=FZD7 PE=1 SV=2 | 572 | HSSKGEtAV-- | 0.141 | 0.04 | -1.086 | -0.302 |
| sp\|Q9H461\|FZD8_HUMAN Frizzled-8 OS=Homo sapiens GN=FZD8 PE=1 SV=1 | 317 | RPIIFLsACYL | 0.076 | -0.115 | -1.581 | -0.54 |
| sp\|Q9H461\|FZD8_HUMAN Frizzled-8 OS=Homo sapiens GN=FZD8 PE=1 SV=1 | 324 | ACYLFVsVGYL | 0.38 | 0.031 | -0.743 | -0.111 |
| sp\|Q9H461\|FZD8_HUMAN Frizzled-8 OS=Homo sapiens GN=FZD8 PE=1 SV=1 | 341 | HEKVACsGGAP | 0.314 | 0.106 | -0.49 | -0.023 |
| sp\|Q9H461\|FZD8_HUMAN Frizzled-8 OS=Homo sapiens GN=FZD8 PE=1 SV=1 | 390 | QHVRYEtTGPA | 0.495 | 0.725 | -0.171 | 0.35 |
| sp\|Q9H461\|FZD8_HUMAN Frizzled-8 OS=Homo sapiens GN=FZD8 PE=1 SV=1 | 391 | HVRYETtGPAL | 0.732 | 0.834 | 0.71 | 0.759 |
| sp\|Q9H461\|FZD8_HUMAN Frizzled-8 OS=Homo sapiens GN=FZD8 PE=1 SV=1 | 397 | TGPALCtVVFL | 0.078 | -0.229 | -1.405 | -0.519 |
| sp\|Q9H461\|FZD8_HUMAN Frizzled-8 OS=Homo sapiens GN=FZD8 PE=1 SV=1 | 410 | YFFGMAsSIWW | 0.146 | -0.02 | -0.578 | -0.151 |
| sp\|Q9H461\|FZD8_HUMAN Frizzled-8 OS=Homo sapiens GN=FZD8 PE=1 SV=1 | 411 | FFGMASsIWWV | 0.232 | 0.036 | -0.744 | -0.159 |
| sp\|Q9H461\|FZD8_HUMAN Frizzled-8 OS=Homo sapiens GN=FZD8 PE=1 SV=1 | 418 | IWWVILsLTWF | 0.176 | 0.114 | -0.465 | -0.058 |
| sp\|Q9H461\|FZD8_HUMAN Frizzled-8 OS=Homo sapiens GN=FZD8 PE=1 SV=1 | 420 | WVILSLtWFLA | 0.185 | 0.223 | -0.439 | -0.01 |
| sp\|Q9H461\|FZD8_HUMAN Frizzled-8 OS=Homo sapiens GN=FZD8 PE=1 SV=1 | 438 | EAIAGYsQYFH | 0.03 | -0.339 | -1.8 | -0.703 |
| sp\|Q9H461\|FZD8_HUMAN Frizzled-8 OS=Homo sapiens GN=FZD8 PE=1 SV=1 | 450 | AAWLVPsVKSI | 0.107 | -0.073 | -1.105 | -0.357 |
| sp\|Q9H461\|FZD8_HUMAN Frizzled-8 OS=Homo sapiens GN=FZD8 PE=1 SV=1 | 453 | LVPSVKsIAVL | 0.082 | -0.133 | -0.975 | -0.342 |
| sp\|Q9H461\|FZD8_HUMAN Frizzled-8 OS=Homo sapiens GN=FZD8 PE=1 SV=1 | 460 | IAVLALsSVDG | 0.209 | -0.037 | -0.848 | -0.225 |
| sp\|Q9H461\|FZD8_HUMAN Frizzled-8 OS=Homo sapiens GN=FZD8 PE=1 SV=1 | 461 | AVLALSsVDGD | 0.13 | -0.182 | -0.897 | -0.316 |

| | | | | | | |
|---|---|---|---|---|---|---|
| sp\|Q9H461\|FZD8_HUMAN Frizzled-8 OS=Homo sapiens GN=FZD8 PE=1 SV=1 | 477 | CYVGNQsLDNL | 0.544 | 0.278 | 0.106 | 0.309 |
| sp\|Q9H461\|FZD8_HUMAN Frizzled-8 OS=Homo sapiens GN=FZD8 PE=1 SV=1 | 497 | IYLFIGtMFLL | 0.262 | 0.126 | -0.47 | -0.027 |
| sp\|Q9H461\|FZD8_HUMAN Frizzled-8 OS=Homo sapiens GN=FZD8 PE=1 SV=1 | 506 | LLAGFVsLFRI | 0.652 | 0.133 | 0.237 | 0.341 |
| sp\|Q9H461\|FZD8_HUMAN Frizzled-8 OS=Homo sapiens GN=FZD8 PE=1 SV=1 | 512 | SLFRIRsVIKQ | 0.402 | 0.823 | -0.024 | 0.4 |
| sp\|Q9H461\|FZD8_HUMAN Frizzled-8 OS=Homo sapiens GN=FZD8 PE=1 SV=1 | 521 | KQQDGPtKTHK | 0.084 | -0.285 | -1.149 | -0.45 |
| sp\|Q9H461\|FZD8_HUMAN Frizzled-8 OS=Homo sapiens GN=FZD8 PE=1 SV=1 | 523 | QDGPTKtHKLE | 0.072 | -0.167 | -1.004 | -0.366 |
| sp\|Q9H461\|FZD8_HUMAN Frizzled-8 OS=Homo sapiens GN=FZD8 PE=1 SV=1 | 537 | IRLGLFtVLYT | 0.116 | 0.073 | -0.85 | -0.22 |
| sp\|Q9H461\|FZD8_HUMAN Frizzled-8 OS=Homo sapiens GN=FZD8 PE=1 SV=1 | 541 | LFTVLYtVPAA | 0.482 | 0.56 | 0.379 | 0.474 |
| sp\|Q9H461\|FZD8_HUMAN Frizzled-8 OS=Homo sapiens GN=FZD8 PE=1 SV=1 | 564 | RPRWEAtHNCP | 0.527 | 0.208 | -0.373 | 0.121 |
| sp\|Q9H461\|FZD8_HUMAN Frizzled-8 OS=Homo sapiens GN=FZD8 PE=1 SV=1 | 599 | CLVVGItSGVW | 0.178 | 0.05 | -0.744 | -0.172 |
| sp\|Q9H461\|FZD8_HUMAN Frizzled-8 OS=Homo sapiens GN=FZD8 PE=1 SV=1 | 600 | LVVGITsGVWV | 0.081 | -0.081 | -0.948 | -0.316 |
| sp\|Q9H461\|FZD8_HUMAN Frizzled-8 OS=Homo sapiens GN=FZD8 PE=1 SV=1 | 606 | SGVWVWsGKTL | 0.125 | -0.247 | -1.015 | -0.379 |
| sp\|Q9H461\|FZD8_HUMAN Frizzled-8 OS=Homo sapiens GN=FZD8 PE=1 SV=1 | 609 | WVWSGKtLESW | 0.087 | 0.041 | -0.716 | -0.196 |
| sp\|Q9H461\|FZD8_HUMAN Frizzled-8 OS=Homo sapiens GN=FZD8 PE=1 SV=1 | 612 | SGKTLEsWRSL | 0.064 | -0.18 | -1.413 | -0.51 |
| sp\|Q9H461\|FZD8_HUMAN Frizzled-8 OS=Homo sapiens GN=FZD8 PE=1 SV=1 | 615 | TLESWRsLCTR | 0.307 | -0.031 | -0.551 | -0.092 |
| sp\|Q9H461\|FZD8_HUMAN Frizzled-8 OS=Homo sapiens GN=FZD8 PE=1 SV=1 | 618 | SWRSLCtRCCW | 0.137 | -0.02 | -1.453 | -0.445 |
| sp\|Q9H461\|FZD8_HUMAN Frizzled-8 OS=Homo sapiens GN=FZD8 PE=1 SV=1 | 624 | TRCCWAsKGAA | 0.604 | 0.33 | 0 | 0.311 |
| sp\|Q9H461\|FZD8_HUMAN Frizzled-8 OS=Homo sapiens GN=FZD8 PE=1 SV=1 | 636 | GGGAGAtAAGG | 0.147 | -0.158 | -1.064 | -0.358 |
| sp\|Q9H461\|FZD8_HUMAN Frizzled-8 OS=Homo sapiens GN=FZD8 PE=1 SV=1 | 664 | PGGGGGsLYSD | 0.077 | -0.124 | -0.931 | -0.326 |
| sp\|Q9H461\|FZD8_HUMAN Frizzled-8 OS=Homo sapiens GN=FZD8 PE=1 SV=1 | 667 | GGGSLYsDVST | 0.074 | -0.128 | -1.301 | -0.452 |
| sp\|Q9H461\|FZD8_HUMAN Frizzled-8 OS=Homo sapiens GN=FZD8 PE=1 SV=1 | 670 | SLYSDVsTGLT | 0.215 | -0.095 | -0.789 | -0.223 |
| sp\|Q9H461\|FZD8_HUMAN Frizzled-8 OS=Homo sapiens GN=FZD8 PE=1 SV=1 | 671 | LYSDVStGLTW | 0.087 | -0.193 | -1.358 | -0.488 |
| sp\|Q9H461\|FZD8_HUMAN Frizzled-8 OS=Homo sapiens GN=FZD8 PE=1 SV=1 | 674 | DVSTGLtWRSG | 0.05 | -0.23 | -1.533 | -0.571 |
| sp\|Q9H461\|FZD8_HUMAN Frizzled-8 OS=Homo sapiens GN=FZD8 PE=1 SV=1 | 677 | TGLTWRsGTAS | 0.145 | -0.107 | -0.941 | -0.301 |
| sp\|Q9H461\|FZD8_HUMAN Frizzled-8 OS=Homo sapiens GN=FZD8 PE=1 SV=1 | 679 | LTWRSGtASSV | 0.424 | 0.996 | -0.198 | 0.407 |
| sp\|Q9H461\|FZD8_HUMAN Frizzled-8 OS=Homo sapiens GN=FZD8 PE=1 SV=1 | 681 | WRSGTAsSVSY | 0.111 | 0.136 | -0.959 | -0.237 |
| sp\|Q9H461\|FZD8_HUMAN Frizzled-8 OS=Homo sapiens GN=FZD8 PE=1 SV=1 | 682 | RSGTASsVSYP | 0.074 | -0.246 | -1.497 | -0.556 |
| sp\|Q9H461\|FZD8_HUMAN Frizzled-8 OS=Homo sapiens GN=FZD8 PE=1 SV=1 | 684 | GTASSVsYPKQ | 0.748 | 0.529 | 0.264 | 0.514 |
| sp\|Q9H461\|FZD8_HUMAN Frizzled-8 OS=Homo sapiens GN=FZD8 PE=1 SV=1 | 692 | PKQMPLsQV-- | 0.185 | 0.025 | -0.905 | -0.232 |
| sp\|O00144\|FZD9_HUMAN Frizzled-9 OS=Homo sapiens GN=FZD9 PE=2 SV=1 | 311 | QEGLENtGCTL | 0.087 | -0.229 | -1.52 | -0.554 |
| sp\|O00144\|FZD9_HUMAN Frizzled-9 OS=Homo sapiens GN=FZD9 PE=2 SV=1 | 314 | LENTGCtLVFL | 0.144 | -0.095 | -1.153 | -0.368 |
| sp\|O00144\|FZD9_HUMAN Frizzled-9 OS=Homo sapiens GN=FZD9 PE=2 SV=1 | 327 | YYFGMAsSLWW | 0.145 | -0.029 | -0.866 | -0.25 |
| sp\|O00144\|FZD9_HUMAN Frizzled-9 OS=Homo sapiens GN=FZD9 PE=2 SV=1 | 328 | YFGMASsLWWV | 0.393 | 0.105 | -0.353 | 0.048 |
| sp\|O00144\|FZD9_HUMAN Frizzled-9 OS=Homo sapiens GN=FZD9 PE=2 SV=1 | 335 | LWWVVLtLTWF | 0.135 | 0.104 | -0.733 | -0.165 |
| sp\|O00144\|FZD9_HUMAN Frizzled-9 OS=Homo sapiens GN=FZD9 PE=2 SV=1 | 337 | WVVLTLtWFLA | 0.068 | 0.005 | -1.009 | -0.312 |
| sp\|O00144\|FZD9_HUMAN Frizzled-9 OS=Homo sapiens GN=FZD9 PE=2 SV=1 | 356 | AIEAHGsYFHM | 0.133 | -0.184 | -0.941 | -0.331 |
| sp\|O00144\|FZD9_HUMAN Frizzled-9 OS=Homo sapiens GN=FZD9 PE=2 SV=1 | 370 | GLPALKtIVIL | 0.272 | 0.022 | -0.583 | -0.096 |
| sp\|O00144\|FZD9_HUMAN Frizzled-9 OS=Homo sapiens GN=FZD9 PE=2 SV=1 | 375 | KTIVILtLRKV | 0.129 | -0.129 | -1.13 | -0.377 |
| sp\|O00144\|FZD9_HUMAN Frizzled-9 OS=Homo sapiens GN=FZD9 PE=2 SV=1 | 385 | VAGDELtGLCY | 0.05 | -0.223 | -1.748 | -0.64 |
| sp\|O00144\|FZD9_HUMAN Frizzled-9 OS=Homo sapiens GN=FZD9 PE=2 SV=1 | 392 | GLCYVAsTDAA | 0.629 | 0.25 | 0.03 | 0.303 |
| sp\|O00144\|FZD9_HUMAN Frizzled-9 OS=Homo sapiens GN=FZD9 PE=2 SV=1 | 393 | LCYVAStDAAA | 0.172 | 0.005 | -0.864 | -0.229 |
| sp\|O00144\|FZD9_HUMAN Frizzled-9 OS=Homo sapiens GN=FZD9 PE=2 SV=1 | 399 | TDAAALtGFVL | 0.096 | -0.136 | -0.882 | -0.307 |
| sp\|O00144\|FZD9_HUMAN Frizzled-9 OS=Homo sapiens GN=FZD9 PE=2 SV=1 | 407 | FVLVPLsGYLV | 0.054 | -0.137 | -1.344 | -0.476 |
| sp\|O00144\|FZD9_HUMAN Frizzled-9 OS=Homo sapiens GN=FZD9 PE=2 SV=1 | 414 | GYLVLGsSFLL | 0.106 | -0.076 | -1.18 | -0.383 |
| sp\|O00144\|FZD9_HUMAN Frizzled-9 OS=Homo sapiens GN=FZD9 PE=2 SV=1 | 415 | YLVLGSsFLLT | 0.09 | -0.06 | -1.034 | -0.335 |
| sp\|O00144\|FZD9_HUMAN Frizzled-9 OS=Homo sapiens GN=FZD9 PE=2 SV=1 | 419 | GSSFLLtGFVA | 0.111 | -0.191 | -1.111 | -0.397 |
| sp\|O00144\|FZD9_HUMAN Frizzled-9 OS=Homo sapiens GN=FZD9 PE=2 SV=1 | 433 | IRKIMKtGGTN | 0.512 | 0.232 | 0.072 | 0.272 |
| sp\|O00144\|FZD9_HUMAN Frizzled-9 OS=Homo sapiens GN=FZD9 PE=2 SV=1 | 436 | IMKTGGtNTEK | 0.13 | -0.158 | -1.222 | -0.417 |
| sp\|O00144\|FZD9_HUMAN Frizzled-9 OS=Homo sapiens GN=FZD9 PE=2 SV=1 | 438 | KTGGTNtEKLE | 0.12 | -0.005 | -1.054 | -0.313 |

| | | | | | | |
|---|---|---|---|---|---|---|
| sp\|O00144\|FZD9_HUMAN Frizzled-9 OS=Homo sapiens GN=FZD9 PE=2 SV=1 | 452 | VKIGVFsILYT | 0.13 | -0.097 | -0.957 | -0.308 |
| sp\|O00144\|FZD9_HUMAN Frizzled-9 OS=Homo sapiens GN=FZD9 PE=2 SV=1 | 456 | VFSILYtVPAT | 0.449 | 0.505 | 0.19 | 0.381 |
| sp\|O00144\|FZD9_HUMAN Frizzled-9 OS=Homo sapiens GN=FZD9 PE=2 SV=1 | 460 | LYTVPAtCVIV | 0.203 | 0.012 | -0.932 | -0.239 |
| sp\|O00144\|FZD9_HUMAN Frizzled-9 OS=Homo sapiens GN=FZD9 PE=2 SV=1 | 481 | FWRLRAtEQPC | 0.647 | 0.48 | -0.126 | 0.334 |
| sp\|O00144\|FZD9_HUMAN Frizzled-9 OS=Homo sapiens GN=FZD9 PE=2 SV=1 | 498 | GGRRDCsLPGG | 0.975 | 1.656 | 1.948 | 1.526 |
| sp\|O00144\|FZD9_HUMAN Frizzled-9 OS=Homo sapiens GN=FZD9 PE=2 SV=1 | 503 | CSLPGGsVPTV | 0.313 | 0.222 | -0.518 | 0.006 |
| sp\|O00144\|FZD9_HUMAN Frizzled-9 OS=Homo sapiens GN=FZD9 PE=2 SV=1 | 506 | PGGSVPtVAVF | 0.08 | -0.22 | -1.201 | -0.447 |
| sp\|O00144\|FZD9_HUMAN Frizzled-9 OS=Homo sapiens GN=FZD9 PE=2 SV=1 | 517 | MLKIFMsLVVG | 0.674 | 0.447 | 0.236 | 0.452 |
| sp\|O00144\|FZD9_HUMAN Frizzled-9 OS=Homo sapiens GN=FZD9 PE=2 SV=1 | 523 | SLVVGItSGVW | 0.092 | -0.048 | -1.187 | -0.381 |
| sp\|O00144\|FZD9_HUMAN Frizzled-9 OS=Homo sapiens GN=FZD9 PE=2 SV=1 | 524 | LVVGITsGVWV | 0.081 | -0.081 | -0.948 | -0.316 |
| sp\|O00144\|FZD9_HUMAN Frizzled-9 OS=Homo sapiens GN=FZD9 PE=2 SV=1 | 530 | SGVWVWsSKTF | 0.093 | -0.299 | -1.211 | -0.472 |
| sp\|O00144\|FZD9_HUMAN Frizzled-9 OS=Homo sapiens GN=FZD9 PE=2 SV=1 | 531 | GVWVWSsKTFQ | 0.074 | -0.075 | -1.291 | -0.431 |
| sp\|O00144\|FZD9_HUMAN Frizzled-9 OS=Homo sapiens GN=FZD9 PE=2 SV=1 | 533 | WVWSSKtFQTW | 0.411 | 0.354 | 0.155 | 0.307 |
| sp\|O00144\|FZD9_HUMAN Frizzled-9 OS=Homo sapiens GN=FZD9 PE=2 SV=1 | 536 | SSKTFQtWQSL | 0.106 | -0.06 | -1.108 | -0.354 |
| sp\|O00144\|FZD9_HUMAN Frizzled-9 OS=Homo sapiens GN=FZD9 PE=2 SV=1 | 539 | TFQTWQsLCYR | 0.352 | 0.028 | -0.393 | -0.004 |
| sp\|O00144\|FZD9_HUMAN Frizzled-9 OS=Homo sapiens GN=FZD9 PE=2 SV=1 | 560 | ACRAPGsYGRG | 0.31 | -0.041 | -0.844 | -0.192 |
| sp\|O00144\|FZD9_HUMAN Frizzled-9 OS=Homo sapiens GN=FZD9 PE=2 SV=1 | 565 | GSYGRGtHCHY | 0.18 | -0.1 | -0.916 | -0.279 |
| sp\|O00144\|FZD9_HUMAN Frizzled-9 OS=Homo sapiens GN=FZD9 PE=2 SV=1 | 573 | CHYKAPtVVLH | 0.244 | -0.032 | -0.909 | -0.232 |
| sp\|O00144\|FZD9_HUMAN Frizzled-9 OS=Homo sapiens GN=FZD9 PE=2 SV=1 | 579 | TVVLHMtKTDP | 0.286 | 0.098 | -0.628 | -0.081 |
| sp\|O00144\|FZD9_HUMAN Frizzled-9 OS=Homo sapiens GN=FZD9 PE=2 SV=1 | 581 | VLHMTKtDPSL | 0.513 | 0.565 | 0.082 | 0.387 |
| sp\|O00144\|FZD9_HUMAN Frizzled-9 OS=Homo sapiens GN=FZD9 PE=2 SV=1 | 584 | MTKTDPsLENP | 0.327 | 0.004 | -0.685 | -0.118 |
| sp\|O00144\|FZD9_HUMAN Frizzled-9 OS=Homo sapiens GN=FZD9 PE=2 SV=1 | 589 | PSLENPtHL-- | 0.121 | -0.054 | -1.027 | -0.32 |
| sp\|Q9ULW2\|FZD10_HUMAN Frizzled-10 OS=Homo sapiens GN=FZD10 PE=1 SV=1 | 310 | LESTGCtLVFL | 0.148 | -0.109 | -1.056 | -0.339 |
| sp\|Q9ULW2\|FZD10_HUMAN Frizzled-10 OS=Homo sapiens GN=FZD10 PE=1 SV=1 | 323 | YYFGMAsSLWW | 0.145 | -0.029 | -0.866 | -0.25 |
| sp\|Q9ULW2\|FZD10_HUMAN Frizzled-10 OS=Homo sapiens GN=FZD10 PE=1 SV=1 | 324 | YFGMASsLWWV | 0.393 | 0.105 | -0.353 | 0.048 |
| sp\|Q9ULW2\|FZD10_HUMAN Frizzled-10 OS=Homo sapiens GN=FZD10 PE=1 SV=1 | 331 | LWWVVLtLTWF | 0.135 | 0.104 | -0.733 | -0.165 |
| sp\|Q9ULW2\|FZD10_HUMAN Frizzled-10 OS=Homo sapiens GN=FZD10 PE=1 SV=1 | 333 | WVVLTLtWFLA | 0.068 | 0.005 | -1.009 | -0.312 |
| sp\|Q9ULW2\|FZD10_HUMAN Frizzled-10 OS=Homo sapiens GN=FZD10 PE=1 SV=1 | 351 | EAIEANsSYFH | 0.064 | -0.199 | -1.609 | -0.581 |
| sp\|Q9ULW2\|FZD10_HUMAN Frizzled-10 OS=Homo sapiens GN=FZD10 PE=1 SV=1 | 352 | AIEANSsYFHL | 0.122 | -0.17 | -0.834 | -0.294 |
| sp\|Q9ULW2\|FZD10_HUMAN Frizzled-10 OS=Homo sapiens GN=FZD10 PE=1 SV=1 | 366 | AIPAVKtILIL | 0.178 | -0.066 | -0.85 | -0.246 |
| sp\|Q9ULW2\|FZD10_HUMAN Frizzled-10 OS=Homo sapiens GN=FZD10 PE=1 SV=1 | 381 | VAGDELtGVCY | 0.062 | -0.193 | -1.621 | -0.584 |
| sp\|Q9ULW2\|FZD10_HUMAN Frizzled-10 OS=Homo sapiens GN=FZD10 PE=1 SV=1 | 388 | GVCYVGsMDVN | 0.246 | 0.162 | -0.849 | -0.147 |
| sp\|Q9ULW2\|FZD10_HUMAN Frizzled-10 OS=Homo sapiens GN=FZD10 PE=1 SV=1 | 395 | MDVNALtGFVL | 0.11 | 0.005 | -0.765 | -0.217 |
| sp\|Q9ULW2\|FZD10_HUMAN Frizzled-10 OS=Homo sapiens GN=FZD10 PE=1 SV=1 | 410 | CYLVIGtSFIL | 0.161 | -0.053 | -0.719 | -0.204 |
| sp\|Q9ULW2\|FZD10_HUMAN Frizzled-10 OS=Homo sapiens GN=FZD10 PE=1 SV=1 | 411 | YLVIGTsFILS | 0.149 | 0.078 | -0.597 | -0.123 |
| sp\|Q9ULW2\|FZD10_HUMAN Frizzled-10 OS=Homo sapiens GN=FZD10 PE=1 SV=1 | 415 | GTSFILsGFVA | 0.173 | -0.102 | -0.774 | -0.234 |
| sp\|Q9ULW2\|FZD10_HUMAN Frizzled-10 OS=Homo sapiens GN=FZD10 PE=1 SV=1 | 429 | IRRVMKtGGEN | 0.65 | 0.45 | 0.248 | 0.449 |
| sp\|Q9ULW2\|FZD10_HUMAN Frizzled-10 OS=Homo sapiens GN=FZD10 PE=1 SV=1 | 434 | KTGGENtDKLE | 0.15 | -0.118 | -1.287 | -0.418 |
| sp\|Q9ULW2\|FZD10_HUMAN Frizzled-10 OS=Homo sapiens GN=FZD10 PE=1 SV=1 | 448 | VRIGLFsVLYT | 0.084 | 0.067 | -1.136 | -0.328 |
| sp\|Q9ULW2\|FZD10_HUMAN Frizzled-10 OS=Homo sapiens GN=FZD10 PE=1 SV=1 | 452 | LFSVLYtVPAT | 0.375 | 0.493 | 0.146 | 0.338 |
| sp\|Q9ULW2\|FZD10_HUMAN Frizzled-10 OS=Homo sapiens GN=FZD10 PE=1 SV=1 | 456 | LYTVPAtCVIA | 0.202 | -0.014 | -0.698 | -0.17 |
| sp\|Q9ULW2\|FZD10_HUMAN Frizzled-10 OS=Homo sapiens GN=FZD10 PE=1 SV=1 | 487 | CKMNNQtKTLD | 0.492 | 0.148 | 0.142 | 0.261 |
| sp\|Q9ULW2\|FZD10_HUMAN Frizzled-10 OS=Homo sapiens GN=FZD10 PE=1 SV=1 | 489 | MNNQTKtLDCL | 0.478 | 0.277 | 0.137 | 0.297 |
| sp\|Q9ULW2\|FZD10_HUMAN Frizzled-10 OS=Homo sapiens GN=FZD10 PE=1 SV=1 | 497 | DCLMAAsIPAV | 0.83 | 0.597 | 0.235 | 0.554 |
| sp\|Q9ULW2\|FZD10_HUMAN Frizzled-10 OS=Homo sapiens GN=FZD10 PE=1 SV=1 | 517 | LLVVGItSGMW | 0.104 | -0.086 | -1.028 | -0.337 |
| sp\|Q9ULW2\|FZD10_HUMAN Frizzled-10 OS=Homo sapiens GN=FZD10 PE=1 SV=1 | 518 | LVVGITsGMWI | 0.138 | -0.018 | -0.457 | -0.112 |
| sp\|Q9ULW2\|FZD10_HUMAN Frizzled-10 OS=Homo sapiens GN=FZD10 PE=1 SV=1 | 524 | SGMWIWtSKTL | 0.112 | -0.184 | -0.836 | -0.303 |
| sp\|Q9ULW2\|FZD10_HUMAN Frizzled-10 OS=Homo sapiens GN=FZD10 PE=1 SV=1 | 525 | GMWIWTsKTLQ | 0.207 | 0.23 | -0.727 | -0.097 |
| sp\|Q9ULW2\|FZD10_HUMAN Frizzled-10 OS=Homo sapiens GN=FZD10 PE=1 SV=1 | 527 | WIWTSKtLQSW | 0.487 | 0.466 | 0.295 | 0.416 |

| | | | | | | |
|---|---|---|---|---|---|---|
| sp\|Q9ULW2\|FZD10_HUMAN Frizzled-10 OS=Homo sapiens GN=FZD10 PE=1 SV=1 | 530 | TSKTLQsWQQV | 0.164 | -0.074 | -0.988 | -0.299 |
| sp\|Q9ULW2\|FZD10_HUMAN Frizzled-10 OS=Homo sapiens GN=FZD10 PE=1 SV=1 | 536 | SWQQVCsRRLK | 0.231 | -0.163 | -1.084 | -0.339 |
| sp\|Q9ULW2\|FZD10_HUMAN Frizzled-10 OS=Homo sapiens GN=FZD10 PE=1 SV=1 | 543 | RRLKKKsRRKP | 0.26 | 0.059 | -0.593 | -0.091 |
| sp\|Q9ULW2\|FZD10_HUMAN Frizzled-10 OS=Homo sapiens GN=FZD10 PE=1 SV=1 | 549 | SRRKPAsVITS | 0.411 | 0.35 | -0.256 | 0.168 |
| sp\|Q9ULW2\|FZD10_HUMAN Frizzled-10 OS=Homo sapiens GN=FZD10 PE=1 SV=1 | 552 | KPASVItSGGI | 0.25 | -0.157 | -0.655 | -0.187 |
| sp\|Q9ULW2\|FZD10_HUMAN Frizzled-10 OS=Homo sapiens GN=FZD10 PE=1 SV=1 | 553 | PASVITsGGIY | 0.226 | -0.088 | -0.67 | -0.177 |
| sp\|Q9ULW2\|FZD10_HUMAN Frizzled-10 OS=Homo sapiens GN=FZD10 PE=1 SV=1 | 566 | AQHPQKtHHGK | 0.139 | -0.199 | -0.877 | -0.312 |
| sp\|Q9ULW2\|FZD10_HUMAN Frizzled-10 OS=Homo sapiens GN=FZD10 PE=1 SV=1 | 577 | YEIPAQsPTCV | 0.113 | -0.252 | -1.39 | -0.51 |
| sp\|Q9ULW2\|FZD10_HUMAN Frizzled-10 OS=Homo sapiens GN=FZD10 PE=1 SV=1 | 579 | IPAQSPtCV-- | 0.387 | 0.289 | -0.18 | 0.165 |
| sp\|Q99835\|SMO_HUMAN Smoothened homolog OS=Homo sapiens GN=SMO PE=1 SV=1 | 308 | RLGEPTsNETL | 0.117 | -0.064 | -1.052 | -0.333 |
| sp\|Q99835\|SMO_HUMAN Smoothened homolog OS=Homo sapiens GN=SMO PE=1 SV=1 | 311 | EPTSNEtLSCV | 0.146 | -0.11 | -1.111 | -0.358 |
| sp\|Q99835\|SMO_HUMAN Smoothened homolog OS=Homo sapiens GN=SMO PE=1 SV=1 | 313 | TSNETLsCVII | 0.079 | -0.053 | -1.274 | -0.416 |
| sp\|Q99835\|SMO_HUMAN Smoothened homolog OS=Homo sapiens GN=SMO PE=1 SV=1 | 336 | VWFVVLtYAWH | 0.035 | -0.308 | -1.717 | -0.663 |
| sp\|Q99835\|SMO_HUMAN Smoothened homolog OS=Homo sapiens GN=SMO PE=1 SV=1 | 341 | LTYAWHtSFKA | 0.218 | -0.01 | -0.698 | -0.163 |
| sp\|Q99835\|SMO_HUMAN Smoothened homolog OS=Homo sapiens GN=SMO PE=1 SV=1 | 342 | TYAWHTsFKAL | 0.479 | 0.177 | 0.128 | 0.261 |
| sp\|Q99835\|SMO_HUMAN Smoothened homolog OS=Homo sapiens GN=SMO PE=1 SV=1 | 348 | SFKALGtTYQP | 0.068 | -0.294 | -1.345 | -0.524 |
| sp\|Q99835\|SMO_HUMAN Smoothened homolog OS=Homo sapiens GN=SMO PE=1 SV=1 | 349 | FKALGTtYQPL | 0.192 | 0.005 | -0.574 | -0.126 |
| sp\|Q99835\|SMO_HUMAN Smoothened homolog OS=Homo sapiens GN=SMO PE=1 SV=1 | 354 | TTYQPLsGKTS | 0.049 | -0.348 | -1.248 | -0.516 |
| sp\|Q99835\|SMO_HUMAN Smoothened homolog OS=Homo sapiens GN=SMO PE=1 SV=1 | 357 | QPLSGKtSYFH | 0.039 | -0.39 | -1.572 | -0.641 |
| sp\|Q99835\|SMO_HUMAN Smoothened homolog OS=Homo sapiens GN=SMO PE=1 SV=1 | 358 | PLSGKTsYFHL | 0.132 | -0.056 | -0.623 | -0.182 |
| sp\|Q99835\|SMO_HUMAN Smoothened homolog OS=Homo sapiens GN=SMO PE=1 SV=1 | 364 | SYFHLLtWSLP | 0.046 | -0.179 | -1.694 | -0.609 |
| sp\|Q99835\|SMO_HUMAN Smoothened homolog OS=Homo sapiens GN=SMO PE=1 SV=1 | 366 | FHLLTWsLPFV | 0.571 | 0.547 | -0.298 | 0.273 |
| sp\|Q99835\|SMO_HUMAN Smoothened homolog OS=Homo sapiens GN=SMO PE=1 SV=1 | 372 | SLPFVLtVAIL | 0.124 | -0.085 | -1.165 | -0.375 |
| sp\|Q99835\|SMO_HUMAN Smoothened homolog OS=Homo sapiens GN=SMO PE=1 SV=1 | 385 | AQVDGDsVSGI | 0.082 | -0.27 | -1.269 | -0.486 |
| sp\|Q99835\|SMO_HUMAN Smoothened homolog OS=Homo sapiens GN=SMO PE=1 SV=1 | 387 | VDGDSVsGICF | 0.148 | 0.017 | -0.718 | -0.184 |
| sp\|Q99835\|SMO_HUMAN Smoothened homolog OS=Homo sapiens GN=SMO PE=1 SV=1 | 425 | LIRGVMtLFSI | 0.662 | 0.531 | 0.473 | 0.555 |
| sp\|Q99835\|SMO_HUMAN Smoothened homolog OS=Homo sapiens GN=SMO PE=1 SV=1 | 428 | GVMTLFsIKSN | 0.068 | -0.07 | -1.216 | -0.406 |
| sp\|Q99835\|SMO_HUMAN Smoothened homolog OS=Homo sapiens GN=SMO PE=1 SV=1 | 431 | TLFSIKsNHPG | 0.296 | -0.02 | -0.517 | -0.08 |
| sp\|Q99835\|SMO_HUMAN Smoothened homolog OS=Homo sapiens GN=SMO PE=1 SV=1 | 438 | NHPGLLsEKAA | 0.068 | -0.1 | -1.279 | -0.437 |
| sp\|Q99835\|SMO_HUMAN Smoothened homolog OS=Homo sapiens GN=SMO PE=1 SV=1 | 443 | LSEKAAsKINE | 0.403 | 0.043 | -0.349 | 0.032 |
| sp\|Q99835\|SMO_HUMAN Smoothened homolog OS=Homo sapiens GN=SMO PE=1 SV=1 | 448 | ASKINEtMLRL | 0.152 | -0.093 | -1.219 | -0.387 |
| sp\|Q99835\|SMO_HUMAN Smoothened homolog OS=Homo sapiens GN=SMO PE=1 SV=1 | 466 | FGFVLItFSCH | 0.091 | -0.096 | -1.262 | -0.422 |
| sp\|Q99835\|SMO_HUMAN Smoothened homolog OS=Homo sapiens GN=SMO PE=1 SV=1 | 468 | FVLITFsCHFY | 0.175 | 0.198 | -0.805 | -0.144 |
| sp\|Q99835\|SMO_HUMAN Smoothened homolog OS=Homo sapiens GN=SMO PE=1 SV=1 | 483 | QAEWERsFRDY | 0.379 | -0.084 | -0.635 | -0.113 |
| sp\|Q99835\|SMO_HUMAN Smoothened homolog OS=Homo sapiens GN=SMO PE=1 SV=1 | 495 | LCQANVtIGLP | 0.354 | -0.005 | -0.431 | -0.027 |
| sp\|Q99835\|SMO_HUMAN Smoothened homolog OS=Homo sapiens GN=SMO PE=1 SV=1 | 500 | VTIGLPtKQPI | 0.135 | -0.179 | -0.911 | -0.318 |
| sp\|Q99835\|SMO_HUMAN Smoothened homolog OS=Homo sapiens GN=SMO PE=1 SV=1 | 514 | EIKNRPsLLVE | 0.38 | 0.208 | -0.152 | 0.145 |
| sp\|Q99835\|SMO_HUMAN Smoothened homolog OS=Homo sapiens GN=SMO PE=1 SV=1 | 528 | LFAMFGtGIAM | 0.125 | -0.162 | -0.639 | -0.225 |
| sp\|Q99835\|SMO_HUMAN Smoothened homolog OS=Homo sapiens GN=SMO PE=1 SV=1 | 533 | GTGIAMsTWVW | 0.399 | 0.26 | -0.579 | 0.027 |
| sp\|Q99835\|SMO_HUMAN Smoothened homolog OS=Homo sapiens GN=SMO PE=1 SV=1 | 534 | TGIAMStWVWT | 0.067 | -0.175 | -1.089 | -0.399 |
| sp\|Q99835\|SMO_HUMAN Smoothened homolog OS=Homo sapiens GN=SMO PE=1 SV=1 | 538 | MSTWVWtKATL | 0.282 | -0.12 | -0.533 | -0.124 |
| sp\|Q99835\|SMO_HUMAN Smoothened homolog OS=Homo sapiens GN=SMO PE=1 SV=1 | 541 | WVWTKAtLLIW | 0.178 | 0.089 | -0.762 | -0.165 |
| sp\|Q99835\|SMO_HUMAN Smoothened homolog OS=Homo sapiens GN=SMO PE=1 SV=1 | 548 | LLIWRRtWCRL | 0.302 | 0.147 | -0.648 | -0.066 |
| sp\|Q99835\|SMO_HUMAN Smoothened homolog OS=Homo sapiens GN=SMO PE=1 SV=1 | 553 | RTWCRLtGQSD | 0.225 | 0.138 | -0.753 | -0.13 |
| sp\|Q99835\|SMO_HUMAN Smoothened homolog OS=Homo sapiens GN=SMO PE=1 SV=1 | 556 | CRLTGQsDDEP | 0.266 | 0.18 | -0.57 | -0.041 |
| sp\|Q99835\|SMO_HUMAN Smoothened homolog OS=Homo sapiens GN=SMO PE=1 SV=1 | 566 | PKRIKKsKMIA | 0.649 | 0.312 | 0.218 | 0.393 |
| sp\|Q99835\|SMO_HUMAN Smoothened homolog OS=Homo sapiens GN=SMO PE=1 SV=1 | 574 | MIAKAFsKRHE | 0.507 | 0.142 | 0.082 | 0.244 |
| sp\|Q99835\|SMO_HUMAN Smoothened homolog OS=Homo sapiens GN=SMO PE=1 SV=1 | 588 | NPGQELsFSMH | 0.051 | -0.349 | -1.636 | -0.645 |
| sp\|Q99835\|SMO_HUMAN Smoothened homolog OS=Homo sapiens GN=SMO PE=1 SV=1 | 590 | GQELSFsMHTV | 0.476 | 0.338 | -0.347 | 0.156 |

| | | | | | | |
|---|---|---|---|---|---|---|
| sp\|Q99835\|SMO_HUMAN Smoothened homolog OS=Homo sapiens GN=SMO PE=1 SV=1 | 593 | LSFSMHtVSHD | 0.14 | -0.157 | -0.812 | -0.276 |
| sp\|Q99835\|SMO_HUMAN Smoothened homolog OS=Homo sapiens GN=SMO PE=1 SV=1 | 595 | FSMHTVsHDGP | 0.23 | -0.018 | -0.539 | -0.109 |
| sp\|Q99835\|SMO_HUMAN Smoothened homolog OS=Homo sapiens GN=SMO PE=1 SV=1 | 611 | FDLNEPsADVS | 0.112 | -0.08 | -1.11 | -0.359 |
| sp\|Q99835\|SMO_HUMAN Smoothened homolog OS=Homo sapiens GN=SMO PE=1 SV=1 | 615 | EPSADVsSAWA | 0.115 | -0.291 | -1.052 | -0.409 |
| sp\|Q99835\|SMO_HUMAN Smoothened homolog OS=Homo sapiens GN=SMO PE=1 SV=1 | 616 | PSADVSsAWAQ | 0.231 | -0.03 | -0.913 | -0.237 |
| sp\|Q99835\|SMO_HUMAN Smoothened homolog OS=Homo sapiens GN=SMO PE=1 SV=1 | 623 | AWAQHVtKMVA | 0.461 | 0.035 | -0.242 | 0.085 |
| sp\|Q99835\|SMO_HUMAN Smoothened homolog OS=Homo sapiens GN=SMO PE=1 SV=1 | 638 | ILPQDIsVTPV | 0.326 | -0.094 | -0.685 | -0.151 |
| sp\|Q99835\|SMO_HUMAN Smoothened homolog OS=Homo sapiens GN=SMO PE=1 SV=1 | 640 | PQDISVtPVAT | 0.201 | -0.091 | -0.497 | -0.129 |
| sp\|Q99835\|SMO_HUMAN Smoothened homolog OS=Homo sapiens GN=SMO PE=1 SV=1 | 644 | SVTPVAtPVPP | 0.035 | -0.524 | -1.766 | -0.752 |
| sp\|Q99835\|SMO_HUMAN Smoothened homolog OS=Homo sapiens GN=SMO PE=1 SV=1 | 662 | LVEAEIsPELQ | 0.057 | -0.468 | -1.487 | -0.633 |
| sp\|Q99835\|SMO_HUMAN Smoothened homolog OS=Homo sapiens GN=SMO PE=1 SV=1 | 699 | PPAPAPsTIPR | 0.142 | -0.165 | -0.715 | -0.246 |
| sp\|Q99835\|SMO_HUMAN Smoothened homolog OS=Homo sapiens GN=SMO PE=1 SV=1 | 700 | PAPAPStIPRL | 0.23 | 0.113 | -0.636 | -0.098 |
| sp\|Q99835\|SMO_HUMAN Smoothened homolog OS=Homo sapiens GN=SMO PE=1 SV=1 | 724 | AWGAGDsCRQG | 0.104 | -0.243 | -1.297 | -0.479 |
| sp\|Q99835\|SMO_HUMAN Smoothened homolog OS=Homo sapiens GN=SMO PE=1 SV=1 | 731 | CRQGAWtLVSN | 0.464 | 0.387 | -0.061 | 0.263 |
| sp\|Q99835\|SMO_HUMAN Smoothened homolog OS=Homo sapiens GN=SMO PE=1 SV=1 | 734 | GAWTLVsNPFC | 0.35 | 0.319 | -0.814 | -0.048 |
| sp\|Q99835\|SMO_HUMAN Smoothened homolog OS=Homo sapiens GN=SMO PE=1 SV=1 | 742 | PFCPEPsPPQD | 0.438 | 0.074 | -0.293 | 0.073 |
| sp\|Q99835\|SMO_HUMAN Smoothened homolog OS=Homo sapiens GN=SMO PE=1 SV=1 | 751 | QDPFLPsAPAP | 0.207 | 0.252 | -0.713 | -0.085 |
| sp\|Q99835\|SMO_HUMAN Smoothened homolog OS=Homo sapiens GN=SMO PE=1 SV=1 | 771 | GLGPIHsRTNL | 0.352 | 0.141 | -0.593 | -0.033 |
| sp\|Q99835\|SMO_HUMAN Smoothened homolog OS=Homo sapiens GN=SMO PE=1 SV=1 | 773 | GPIHSRtNLMD | 0.21 | 0.063 | -1.032 | -0.253 |
| sp\|Q99835\|SMO_HUMAN Smoothened homolog OS=Homo sapiens GN=SMO PE=1 SV=1 | 778 | RTNLMDtELMD | 0.185 | -0.162 | -1.255 | -0.411 |
| sp\|Q99835\|SMO_HUMAN Smoothened homolog OS=Homo sapiens GN=SMO PE=1 SV=1 | 785 | ELMDADsDF-- | 0.371 | 0.268 | -0.608 | 0.01 |
| sp\|Q5VW38\|GP107_HUMAN Protein GPR107 OS=Homo sapiens GN=GPR107 PE=1 SV=1 | 308 | PFTKSLsLVFH | 0.434 | 0.16 | -0.102 | 0.164 |
| sp\|Q5VW38\|GP107_HUMAN Protein GPR107 OS=Homo sapiens GN=GPR107 PE=1 SV=1 | 320 | IDYHYIsSQGF | 0.112 | -0.246 | -0.858 | -0.331 |
| sp\|Q5VW38\|GP107_HUMAN Protein GPR107 OS=Homo sapiens GN=GPR107 PE=1 SV=1 | 321 | DYHYISsQGFP | 0.087 | -0.221 | -1.34 | -0.491 |
| sp\|Q5VW38\|GP107_HUMAN Protein GPR107 OS=Homo sapiens GN=GPR107 PE=1 SV=1 | 336 | AVVYYItHLLK | 0.171 | -0.076 | -0.864 | -0.256 |
| sp\|Q5VW38\|GP107_HUMAN Protein GPR107 OS=Homo sapiens GN=GPR107 PE=1 SV=1 | 347 | GALLFItIALI | 0.22 | -0.094 | -0.808 | -0.227 |
| sp\|Q5VW38\|GP107_HUMAN Protein GPR107 OS=Homo sapiens GN=GPR107 PE=1 SV=1 | 353 | TIALIGtGWAF | 0.148 | -0.028 | -0.691 | -0.19 |
| sp\|Q5VW38\|GP107_HUMAN Protein GPR107 OS=Homo sapiens GN=GPR107 PE=1 SV=1 | 363 | FIKHILsDKDK | 0.331 | 0.12 | -0.305 | 0.049 |
| sp\|Q5VW38\|GP107_HUMAN Protein GPR107 OS=Homo sapiens GN=GPR107 PE=1 SV=1 | 389 | AYIIIEsTEEG | 0.175 | -0.04 | -1.018 | -0.294 |
| sp\|Q5VW38\|GP107_HUMAN Protein GPR107 OS=Homo sapiens GN=GPR107 PE=1 SV=1 | 390 | YIIIEStEEGT | 0.092 | -0.052 | -1.08 | -0.347 |
| sp\|Q5VW38\|GP107_HUMAN Protein GPR107 OS=Homo sapiens GN=GPR107 PE=1 SV=1 | 394 | ESTEEGtTEYG | 0.046 | -0.312 | -1.677 | -0.648 |
| sp\|Q5VW38\|GP107_HUMAN Protein GPR107 OS=Homo sapiens GN=GPR107 PE=1 SV=1 | 395 | STEEGTtEYGL | 0.076 | -0.143 | -1.182 | -0.416 |
| sp\|Q5VW38\|GP107_HUMAN Protein GPR107 OS=Homo sapiens GN=GPR107 PE=1 SV=1 | 403 | YGLWKDsLFLV | 0.277 | -0.081 | -0.59 | -0.131 |
| sp\|Q5VW38\|GP107_HUMAN Protein GPR107 OS=Homo sapiens GN=GPR107 PE=1 SV=1 | 422 | LFPVVWsIRHL | 0.073 | -0.271 | -1.025 | -0.408 |
| sp\|Q5VW38\|GP107_HUMAN Protein GPR107 OS=Homo sapiens GN=GPR107 PE=1 SV=1 | 430 | RHLQEAsATDG | 0.486 | 0.085 | -0.756 | -0.062 |
| sp\|Q5VW38\|GP107_HUMAN Protein GPR107 OS=Homo sapiens GN=GPR107 PE=1 SV=1 | 432 | LQEASAtDGKG | 0.493 | 0.21 | -0.07 | 0.211 |
| sp\|Q5VW38\|GP107_HUMAN Protein GPR107 OS=Homo sapiens GN=GPR107 PE=1 SV=1 | 438 | TDGKGDsMGPL | 0.089 | -0.139 | -1.244 | -0.431 |
| sp\|Q5VW38\|GP107_HUMAN Protein GPR107 OS=Homo sapiens GN=GPR107 PE=1 SV=1 | 452 | ANLRAGsRIES | 0.415 | 0.619 | -0.123 | 0.304 |
| sp\|Q5VW38\|GP107_HUMAN Protein GPR107 OS=Homo sapiens GN=GPR107 PE=1 SV=1 | 456 | AGSRIEsHHFA | 0.522 | 0.64 | 0.064 | 0.409 |
| sp\|Q5VW38\|GP107_HUMAN Protein GPR107 OS=Homo sapiens GN=GPR107 PE=1 SV=1 | 469 | DLELLAsSCPP | 0.125 | -0.237 | -1.362 | -0.491 |
| sp\|Q5VW38\|GP107_HUMAN Protein GPR107 OS=Homo sapiens GN=GPR107 PE=1 SV=1 | 470 | LELLASsCPPA | 0.45 | 0.431 | -0.179 | 0.234 |
| sp\|Q5VW38\|GP107_HUMAN Protein GPR107 OS=Homo sapiens GN=GPR107 PE=1 SV=1 | 475 | SSCPPAsVSQR | 0.098 | -0.316 | -1.197 | -0.472 |
| sp\|Q5VW38\|GP107_HUMAN Protein GPR107 OS=Homo sapiens GN=GPR107 PE=1 SV=1 | 477 | CPPASVsQRAG | 0.493 | 0.06 | -0.272 | 0.094 |
| sp\|Q5VW38\|GP107_HUMAN Protein GPR107 OS=Homo sapiens GN=GPR107 PE=1 SV=1 | 483 | SQRAGItAAIN | 0.19 | 0.03 | -0.936 | -0.239 |
| sp\|Q5VW38\|GP107_HUMAN Protein GPR107 OS=Homo sapiens GN=GPR107 PE=1 SV=1 | 508 | VCYIYFtRIIA | 0.167 | -0.072 | -0.851 | -0.252 |
| sp\|Q5VW38\|GP107_HUMAN Protein GPR107 OS=Homo sapiens GN=GPR107 PE=1 SV=1 | 533 | YQLLDEtATLV | 0.146 | -0.095 | -1.272 | -0.407 |
| sp\|Q5VW38\|GP107_HUMAN Protein GPR107 OS=Homo sapiens GN=GPR107 PE=1 SV=1 | 535 | LLDETAtLVFF | 0.14 | 0.062 | -0.755 | -0.184 |
| sp\|Q5VW38\|GP107_HUMAN Protein GPR107 OS=Homo sapiens GN=GPR107 PE=1 SV=1 | 542 | LVFFVLtGYKF | 0.073 | -0.145 | -1.241 | -0.438 |
| sp\|Q5VW38\|GP107_HUMAN Protein GPR107 OS=Homo sapiens GN=GPR107 PE=1 SV=1 | 550 | YKFRPAsDNPY | 0.836 | 0.947 | 0.773 | 0.852 |

| | | | | | | |
|---|---|---|---|---|---|---|
| sp\|Q5VW38\|GP107_HUMAN Protein GPR107 OS=Homo sapiens GN=GPR107 PE=1 SV=1 | 558 | NPYLQLsQEEE | 0.053 | -0.222 | -1.728 | -0.632 |
| sp\|Q5VW38\|GP107_HUMAN Protein GPR107 OS=Homo sapiens GN=GPR107 PE=1 SV=1 | 568 | EDLEMEsVVTT | 0.038 | -0.339 | -1.477 | -0.593 |
| sp\|Q5VW38\|GP107_HUMAN Protein GPR107 OS=Homo sapiens GN=GPR107 PE=1 SV=1 | 571 | EMESVVtTSGV | 0.118 | -0.144 | -1.231 | -0.419 |
| sp\|Q5VW38\|GP107_HUMAN Protein GPR107 OS=Homo sapiens GN=GPR107 PE=1 SV=1 | 572 | MESVVTtSGVM | 0.179 | -0.016 | -0.906 | -0.248 |
| sp\|Q5VW38\|GP107_HUMAN Protein GPR107 OS=Homo sapiens GN=GPR107 PE=1 SV=1 | 573 | ESVVTTsGVME | 0.037 | -0.267 | -1.504 | -0.578 |
| sp\|Q5VW38\|GP107_HUMAN Protein GPR107 OS=Homo sapiens GN=GPR107 PE=1 SV=1 | 578 | TSGVMEsMKKV | 0.045 | -0.231 | -1.791 | -0.659 |
| sp\|Q5VW38\|GP107_HUMAN Protein GPR107 OS=Homo sapiens GN=GPR107 PE=1 SV=1 | 586 | KKVKKVtNGSV | 0.378 | -0.018 | -0.816 | -0.152 |
| sp\|Q5VW38\|GP107_HUMAN Protein GPR107 OS=Homo sapiens GN=GPR107 PE=1 SV=1 | 589 | KKVTNGsVEPQ | 0.137 | -0.199 | -1.118 | -0.393 |
| sp\|Q96N19\|G137A_HUMAN Integral membrane protein GPR137 OS=Homo sapiens GN=GPR137 PE=2 SV=2 | 317 | NGQVFAsRSYF | 0.081 | -0.218 | -1.461 | -0.533 |
| sp\|Q96N19\|G137A_HUMAN Integral membrane protein GPR137 OS=Homo sapiens GN=GPR137 PE=2 SV=2 | 319 | QVFASRsYFFD | 0.169 | 0.053 | -0.701 | -0.16 |
| sp\|Q96N19\|G137A_HUMAN Integral membrane protein GPR137 OS=Homo sapiens GN=GPR137 PE=2 SV=2 | 334 | CEDEGCsWEHS | 0.058 | -0.147 | -1.52 | -0.536 |
| sp\|Q96N19\|G137A_HUMAN Integral membrane protein GPR137 OS=Homo sapiens GN=GPR137 PE=2 SV=2 | 338 | GCSWEHsRGES | 0.487 | 0.048 | -0.689 | -0.051 |
| sp\|Q96N19\|G137A_HUMAN Integral membrane protein GPR137 OS=Homo sapiens GN=GPR137 PE=2 SV=2 | 342 | EHSRGEsTRCQ | 0.255 | 0.589 | -0.717 | 0.042 |
| sp\|Q96N19\|G137A_HUMAN Integral membrane protein GPR137 OS=Homo sapiens GN=GPR137 PE=2 SV=2 | 343 | HSRGEStRCQD | 0.167 | -0.083 | -0.889 | -0.268 |
| sp\|Q96N19\|G137A_HUMAN Integral membrane protein GPR137 OS=Homo sapiens GN=GPR137 PE=2 SV=2 | 351 | CQDQAAtTTVS | 0.342 | 0.037 | -0.466 | -0.029 |
| sp\|Q96N19\|G137A_HUMAN Integral membrane protein GPR137 OS=Homo sapiens GN=GPR137 PE=2 SV=2 | 352 | QDQAATtTVST | 0.077 | -0.167 | -1.123 | -0.404 |
| sp\|Q96N19\|G137A_HUMAN Integral membrane protein GPR137 OS=Homo sapiens GN=GPR137 PE=2 SV=2 | 353 | DQAATTtVSTP | 0.069 | -0.208 | -1.11 | -0.416 |
| sp\|Q96N19\|G137A_HUMAN Integral membrane protein GPR137 OS=Homo sapiens GN=GPR137 PE=2 SV=2 | 355 | AATTTVsTPPH | 0.362 | 0.207 | -0.545 | 0.008 |
| sp\|Q96N19\|G137A_HUMAN Integral membrane protein GPR137 OS=Homo sapiens GN=GPR137 PE=2 SV=2 | 356 | ATTTVStPPHR | 0.216 | -0.03 | -0.784 | -0.199 |
| sp\|Q96N19\|G137A_HUMAN Integral membrane protein GPR137 OS=Homo sapiens GN=GPR137 PE=2 SV=2 | 366 | RRDPPPsPTEY | 0.137 | -0.253 | -1.173 | -0.43 |
| sp\|Q96N19\|G137A_HUMAN Integral membrane protein GPR137 OS=Homo sapiens GN=GPR137 PE=2 SV=2 | 368 | DPPPSPtEYPG | 0.208 | -0.013 | -0.781 | -0.195 |
| sp\|Q96N19\|G137A_HUMAN Integral membrane protein GPR137 OS=Homo sapiens GN=GPR137 PE=2 SV=2 | 374 | TEYPGPsPPHP | 0.056 | -0.202 | -1.239 | -0.462 |
| sp\|Q96N19\|G137A_HUMAN Integral membrane protein GPR137 OS=Homo sapiens GN=GPR137 PE=2 SV=2 | 408 | WPAPCCsCHSE | 0.215 | -0.104 | -0.924 | -0.271 |
| sp\|Q96N19\|G137A_HUMAN Integral membrane protein GPR137 OS=Homo sapiens GN=GPR137 PE=2 SV=2 | 411 | PCCSCHsELVP | 0.361 | 0.158 | -0.742 | -0.074 |
| sp\|Q96N19\|G137A_HUMAN Integral membrane protein GPR137 OS=Homo sapiens GN=GPR137 PE=2 SV=2 | 416 | HSELVPsP--- | 0.087 | -0.265 | -1.29 | -0.489 |
| sp\|Q8TCB6\|O51E1_HUMAN Olfactory receptor 51E1 OS=Homo sapiens GN=OR51E1 PE=2 SV=1 | 312 | RLFHVAtHASE | 0.278 | -0.005 | -0.812 | -0.18 |
| sp\|Q8TCB6\|O51E1_HUMAN Olfactory receptor 51E1 OS=Homo sapiens GN=OR51E1 PE=2 SV=1 | 315 | HVATHAsEP-- | 0.697 | 0.802 | 0.348 | 0.616 |
| sp\|Q86W33\|TPRA1_HUMAN Transmembrane protein adipocyte-associated 1 OS=Homo sapiens GN=TPRA1 P | 329 | AGAAGAsAASY | 0.103 | -0.187 | -1.234 | -0.439 |
| sp\|Q86W33\|TPRA1_HUMAN Transmembrane protein adipocyte-associated 1 OS=Homo sapiens GN=TPRA1 P | 332 | AGASAAsYSST | 0.08 | -0.253 | -1.195 | -0.456 |
| sp\|Q86W33\|TPRA1_HUMAN Transmembrane protein adipocyte-associated 1 OS=Homo sapiens GN=TPRA1 P | 334 | ASAASYsSTQF | 0.282 | 0.029 | -0.329 | -0.006 |
| sp\|Q86W33\|TPRA1_HUMAN Transmembrane protein adipocyte-associated 1 OS=Homo sapiens GN=TPRA1 P | 335 | SAASYSsTQFD | 0.071 | -0.315 | -1.366 | -0.537 |
| sp\|Q86W33\|TPRA1_HUMAN Transmembrane protein adipocyte-associated 1 OS=Homo sapiens GN=TPRA1 P | 336 | AASYSStQFDS | 0.246 | 0.026 | -0.709 | -0.146 |
| sp\|Q86W33\|TPRA1_HUMAN Transmembrane protein adipocyte-associated 1 OS=Homo sapiens GN=TPRA1 P | 340 | SSTQFDsAGGV | 0.201 | -0.201 | -1.221 | -0.407 |
| sp\|Q86W33\|TPRA1_HUMAN Transmembrane protein adipocyte-associated 1 OS=Homo sapiens GN=TPRA1 P | 352 | YLDDIAsMPCH | 0.575 | 0.616 | -0.106 | 0.362 |
| sp\|Q86W33\|TPRA1_HUMAN Transmembrane protein adipocyte-associated 1 OS=Homo sapiens GN=TPRA1 P | 357 | ASMPCHtGSIN | 0.208 | -0.091 | -0.925 | -0.269 |
| sp\|Q86W33\|TPRA1_HUMAN Transmembrane protein adipocyte-associated 1 OS=Homo sapiens GN=TPRA1 P | 359 | MPCHTGsINST | 0.059 | -0.171 | -1.241 | -0.451 |
| sp\|Q86W33\|TPRA1_HUMAN Transmembrane protein adipocyte-associated 1 OS=Homo sapiens GN=TPRA1 P | 362 | HTGSINsTDSE | 0.122 | -0.093 | -1.014 | -0.328 |
| sp\|Q86W33\|TPRA1_HUMAN Transmembrane protein adipocyte-associated 1 OS=Homo sapiens GN=TPRA1 P | 363 | TGSINStDSER | 0.107 | -0.174 | -1.085 | -0.384 |
| sp\|Q86W33\|TPRA1_HUMAN Transmembrane protein adipocyte-associated 1 OS=Homo sapiens GN=TPRA1 P | 365 | SINSTDsERWK | 0.096 | -0.161 | -1.314 | -0.46 |
| sp\|P51810\|GP143_HUMAN G-protein coupled receptor 143 OS=Homo sapiens GN=GPR143 PE=1 SV=2 | 314 | LAFYGWtGCSL | 0.047 | -0.302 | -1.345 | -0.533 |
| sp\|P51810\|GP143_HUMAN G-protein coupled receptor 143 OS=Homo sapiens GN=GPR143 PE=1 SV=2 | 317 | YGWTGCsLGFQ | 0.206 | 0.018 | -0.767 | -0.181 |
| sp\|P51810\|GP143_HUMAN G-protein coupled receptor 143 OS=Homo sapiens GN=GPR143 PE=1 SV=2 | 322 | CSLGFQsPRKE | 0.071 | -0.492 | -1.29 | -0.57 |
| sp\|P51810\|GP143_HUMAN G-protein coupled receptor 143 OS=Homo sapiens GN=GPR143 PE=1 SV=2 | 331 | KEIQWEsLTTS | 0.314 | -0.04 | -0.763 | -0.163 |
| sp\|P51810\|GP143_HUMAN G-protein coupled receptor 143 OS=Homo sapiens GN=GPR143 PE=1 SV=2 | 333 | IQWESLtTSAA | 0.12 | 0.171 | -0.542 | -0.084 |
| sp\|P51810\|GP143_HUMAN G-protein coupled receptor 143 OS=Homo sapiens GN=GPR143 PE=1 SV=2 | 334 | QWESLTtSAAE | 0.087 | -0.205 | -1.164 | -0.427 |
| sp\|P51810\|GP143_HUMAN G-protein coupled receptor 143 OS=Homo sapiens GN=GPR143 PE=1 SV=2 | 335 | WESLTTsAAEG | 0.13 | -0.085 | -1.135 | -0.363 |
| sp\|P51810\|GP143_HUMAN G-protein coupled receptor 143 OS=Homo sapiens GN=GPR143 PE=1 SV=2 | 343 | AEGAHPsPLMP | 0.033 | -0.614 | -2.086 | -0.889 |
| sp\|P51810\|GP143_HUMAN G-protein coupled receptor 143 OS=Homo sapiens GN=GPR143 PE=1 SV=2 | 353 | PHENPAsGKVS | 0.095 | -0.17 | -1.112 | -0.396 |
| sp\|P51810\|GP143_HUMAN G-protein coupled receptor 143 OS=Homo sapiens GN=GPR143 PE=1 SV=2 | 357 | PASGKVsQVGG | 0.158 | -0.234 | -1.066 | -0.381 |

| | | | | | | |
|---|---|---|---|---|---|---|
| sp\|P51810\|GP143_HUMAN G-protein coupled receptor 143 OS=Homo sapiens GN=GPR143 PE=1 SV=2 | 363 | SQVGGQtSDEA | 0.064 | -0.178 | -1.116 | -0.41 |
| sp\|P51810\|GP143_HUMAN G-protein coupled receptor 143 OS=Homo sapiens GN=GPR143 PE=1 SV=2 | 364 | QVGGQTsDEAL | 0.102 | 0.048 | -0.952 | -0.267 |
| sp\|P51810\|GP143_HUMAN G-protein coupled receptor 143 OS=Homo sapiens GN=GPR143 PE=1 SV=2 | 369 | TSDEALsMLSE | 0.039 | -0.168 | -1.729 | -0.619 |
| sp\|P51810\|GP143_HUMAN G-protein coupled receptor 143 OS=Homo sapiens GN=GPR143 PE=1 SV=2 | 372 | EALSMLsEGSD | 0.097 | -0.158 | -0.924 | -0.328 |
| sp\|P51810\|GP143_HUMAN G-protein coupled receptor 143 OS=Homo sapiens GN=GPR143 PE=1 SV=2 | 375 | SMLSEGsDAST | 0.032 | -0.297 | -2.019 | -0.761 |
| sp\|P51810\|GP143_HUMAN G-protein coupled receptor 143 OS=Homo sapiens GN=GPR143 PE=1 SV=2 | 378 | SEGSDAsTIEI | 0.12 | -0.15 | -1.187 | -0.406 |
| sp\|P51810\|GP143_HUMAN G-protein coupled receptor 143 OS=Homo sapiens GN=GPR143 PE=1 SV=2 | 379 | EGSDAStIEIH | 0.077 | -0.265 | -1.334 | -0.507 |
| sp\|P51810\|GP143_HUMAN G-protein coupled receptor 143 OS=Homo sapiens GN=GPR143 PE=1 SV=2 | 384 | STIEIHtASES | 0.091 | -0.018 | -1.402 | -0.443 |
| sp\|P51810\|GP143_HUMAN G-protein coupled receptor 143 OS=Homo sapiens GN=GPR143 PE=1 SV=2 | 386 | IEIHTAsESCN | 0.153 | 0.081 | -1.003 | -0.256 |
| sp\|P51810\|GP143_HUMAN G-protein coupled receptor 143 OS=Homo sapiens GN=GPR143 PE=1 SV=2 | 388 | IHTASEsCNKN | 0.375 | 0.195 | -0.554 | 0.005 |
| sp\|P51810\|GP143_HUMAN G-protein coupled receptor 143 OS=Homo sapiens GN=GPR143 PE=1 SV=2 | 400 | GDPALPtHGDL | 0.195 | -0.128 | -0.899 | -0.277 |
