# Supplementary material for: 14-3-3 signal adaptor and scaffold proteins mediate GPCR trafficking
Source: Sci Rep. 2019 Aug 1;9:11156. doi: 10.1038/s41598-019-47478-w (PMC6673703; doi:10.1038/s41598-019-47478-w)
Supplement: Supplementary file 2 — Supplementary table 2 [file 41598_2019_47478_MOESM2_ESM.pdf]

14-3-3 signal adaptor and scaffold proteins mediate GPCR trafficking

Luwa Yuan[1], Shahar Barbash[2], Sathapana Kongsamut[1], Alex Eishingdrelo[1], Thomas P. Sakmar[2,3], Haifeng Eishingdrelo[1*]

GPCR	numer of 14-3-3 motif
sp|O00144|FZD9_HUMAN Frizzled-9 OS=Homo sapiens GN=FZD9 PE=2 SV=1	42
sp|O00155|GPR25_HUMAN Probable G-protein coupled receptor 25 OS=Homo sapiens GN=GPR25 PE=1 SV=2	12
sp|O00222|GRM8_HUMAN Metabotropic glutamate receptor 8 OS=Homo sapiens GN=GRM8 PE=2 SV=2	86
sp|O00270|GPR31_HUMAN 12-(S)-hydroxy-5,8,10,14-eicosatetraenoic acid receptor OS=Homo sapiens GN=GPR31 PE=2 SV=2	2
sp|O00398|P2Y10_HUMAN Putative P2Y purinoceptor 10 OS=Homo sapiens GN=P2RY10 PE=2 SV=1	4
sp|O00421|CCRL2_HUMAN C-C chemokine receptor-like 2 OS=Homo sapiens GN=CCRL2 PE=1 SV=2	9
sp|O00574|CXCR6_HUMAN C-X-C chemokine receptor type 6 OS=Homo sapiens GN=CXCR6 PE=2 SV=1	10
sp|O00590|ACKR2_HUMAN Atypical chemokine receptor 2 OS=Homo sapiens GN=ACKR2 PE=1 SV=2	12
sp|O14514|AGRB1_HUMAN Adhesion G protein-coupled receptor B1 OS=Homo sapiens GN=ADGRB1 PE=1 SV=2	186
sp|O14804|TAAR5_HUMAN Trace amine-associated receptor 5 OS=Homo sapiens GN=TAAR5 PE=2 SV=2	5
sp|O14842|FFAR1_HUMAN Free fatty acid receptor 1 OS=Homo sapiens GN=FFAR1 PE=1 SV=1	3
sp|O14843|FFAR3_HUMAN Free fatty acid receptor 3 OS=Homo sapiens GN=FFAR3 PE=1 SV=1	9
sp|O15218|GP182_HUMAN G-protein coupled receptor 182 OS=Homo sapiens GN=GPR182 PE=2 SV=1	21
sp|O15303|GRM6_HUMAN Metabotropic glutamate receptor 6 OS=Homo sapiens GN=GRM6 PE=1 SV=2	87
sp|O15354|GPR37_HUMAN Prosaposin receptor GPR37 OS=Homo sapiens GN=GPR37 PE=1 SV=2	48
sp|O15529|GPR42_HUMAN G-protein coupled receptor 42 OS=Homo sapiens GN=GPR42 PE=1 SV=1	8
sp|O15552|FFAR2_HUMAN Free fatty acid receptor 2 OS=Homo sapiens GN=FFAR2 PE=1 SV=1	10
sp|O43193|MTLR_HUMAN Motilin receptor OS=Homo sapiens GN=MLNR PE=2 SV=1	10
sp|O43194|GPR39_HUMAN G-protein coupled receptor 39 OS=Homo sapiens GN=GPR39 PE=1 SV=1	28
sp|O43603|GALR2_HUMAN Galanin receptor type 2 OS=Homo sapiens GN=GALR2 PE=1 SV=1	13
sp|O43613|OX1R_HUMAN Orexin receptor type 1 OS=Homo sapiens GN=HCRTR1 PE=1 SV=2	17
sp|O43614|OX2R_HUMAN Orexin receptor type 2 OS=Homo sapiens GN=HCRTR2 PE=1 SV=2	17
sp|O60241|AGRB2_HUMAN Adhesion G protein-coupled receptor B2 OS=Homo sapiens GN=ADGRB2 PE=1 SV=2	207
sp|O60242|AGRB3_HUMAN Adhesion G protein-coupled receptor B3 OS=Homo sapiens GN=ADGRB3 PE=1 SV=2	189
sp|O60353|FZD6_HUMAN Frizzled-6 OS=Homo sapiens GN=FZD6 PE=1 SV=2	65
sp|O60755|GALR3_HUMAN Galanin receptor type 3 OS=Homo sapiens GN=GALR3 PE=1 SV=1	4
sp|O60883|ETBR2_HUMAN Prosaposin receptor GPR37L1 OS=Homo sapiens GN=GPR37L1 PE=1 SV=2	27
sp|O75084|FZD7_HUMAN Frizzled-7 OS=Homo sapiens GN=FZD7 PE=1 SV=2	38
sp|O75388|GPR32_HUMAN Probable G-protein coupled receptor 32 OS=Homo sapiens GN=GPR32 PE=2 SV=1	8
sp|O75473|LGR5_HUMAN Leucine-rich repeat-containing G-protein coupled receptor 5 OS=Homo sapiens GN=LGR5 PE=1 SV=1	23
sp|O75899|GABR2_HUMAN Gamma-aminobutyric acid type B receptor subunit 2 OS=Homo sapiens GN=GABBR2 PE=1 SV=1	92
sp|O94910|AGRL1_HUMAN Adhesion G protein-coupled receptor L1 OS=Homo sapiens GN=ADGRL1 PE=1 SV=1	161
sp|O95136|S1PR2_HUMAN Sphingosine 1-phosphate receptor 2 OS=Homo sapiens GN=S1PR2 PE=1 SV=2	10
sp|O95490|AGRL2_HUMAN Adhesion G protein-coupled receptor L2 OS=Homo sapiens GN=ADGRL2 PE=1 SV=2	189
sp|O95800|GPR75_HUMAN Probable G-protein coupled receptor 75 OS=Homo sapiens GN=GPR75 PE=1 SV=1	31
sp|O95838|GLP2R_HUMAN Glucagon-like peptide 2 receptor OS=Homo sapiens GN=GLP2R PE=2 SV=1	23
sp|O95977|S1PR4_HUMAN Sphingosine 1-phosphate receptor 4 OS=Homo sapiens GN=S1PR4 PE=1 SV=1	17
sp|P03999|OPSB_HUMAN Short-wave-sensitive opsin 1 OS=Homo sapiens GN=OPN1SW PE=1 SV=1	11
sp|P04000|OPSR_HUMAN Long-wave-sensitive opsin 1 OS=Homo sapiens GN=OPN1LW PE=1 SV=1	10
sp|P04001|OPSG_HUMAN Medium-wave-sensitive opsin 1 OS=Homo sapiens GN=OPN1MW PE=1 SV=1	12
sp|P04201|MAS_HUMAN Proto-oncogene Mas OS=Homo sapiens GN=MAS1 PE=1 SV=1	7
sp|P07550|ADRB2_HUMAN Beta-2 adrenergic receptor OS=Homo sapiens GN=ADRB2 PE=1 SV=3	14
sp|P08100|OPSD_HUMAN Rhodopsin OS=Homo sapiens GN=RHO PE=1 SV=1	8
sp|P08172|ACM2_HUMAN Muscarinic acetylcholine receptor M2 OS=Homo sapiens GN=CHRM2 PE=1 SV=1	3

sp|P08173|ACM4_HUMAN Muscarinic acetylcholine receptor M4 OS=Homo sapiens GN=CHRM4 PE=1 SV=2	3
sp|P08588|ADRB1_HUMAN Beta-1 adrenergic receptor OS=Homo sapiens GN=ADRB1 PE=1 SV=2	11
sp|P08912|ACM5_HUMAN Muscarinic acetylcholine receptor M5 OS=Homo sapiens GN=CHRM5 PE=2 SV=2	3
sp|P0DMS8|AA3R_HUMAN Adenosine receptor A3 OS=Homo sapiens GN=ADORA3 PE=1 SV=1	6
sp|P11229|ACM1_HUMAN Muscarinic acetylcholine receptor M1 OS=Homo sapiens GN=CHRM1 PE=1 SV=2	4
sp|P13945|ADRB3_HUMAN Beta-3 adrenergic receptor OS=Homo sapiens GN=ADRB3 PE=1 SV=3	7
sp|P16473|TSHR_HUMAN Thyrotropin receptor OS=Homo sapiens GN=TSHR PE=1 SV=2	9
sp|P18089|ADA2B_HUMAN Alpha-2B adrenergic receptor OS=Homo sapiens GN=ADRA2B PE=1 SV=4	3
sp|P18825|ADA2C_HUMAN Alpha-2C adrenergic receptor OS=Homo sapiens GN=ADRA2C PE=2 SV=2	1
sp|P20309|ACM3_HUMAN Muscarinic acetylcholine receptor M3 OS=Homo sapiens GN=CHRM3 PE=1 SV=1	4
sp|P21452|NK2R_HUMAN Substance-K receptor OS=Homo sapiens GN=TACR2 PE=1 SV=3	18
sp|P21453|S1PR1_HUMAN Sphingosine 1-phosphate receptor 1 OS=Homo sapiens GN=S1PR1 PE=1 SV=2	14
sp|P21462|FPR1_HUMAN fMet-Leu-Phe receptor OS=Homo sapiens GN=FPR1 PE=1 SV=3	11
sp|P21554|CNR1_HUMAN Cannabinoid receptor 1 OS=Homo sapiens GN=CNR1 PE=1 SV=1	16
sp|P21728|DRD1_HUMAN D(1A) dopamine receptor OS=Homo sapiens GN=DRD1 PE=1 SV=1	17
sp|P21730|C5AR1_HUMAN C5a anaphylatoxin chemotactic receptor 1 OS=Homo sapiens GN=C5AR1 PE=1 SV=2	11
sp|P21731|TA2R_HUMAN Thromboxane A2 receptor OS=Homo sapiens GN=TBXA2R PE=1 SV=3	6
sp|P21918|DRD5_HUMAN D(1B) dopamine receptor OS=Homo sapiens GN=DRD5 PE=1 SV=2	13
sp|P22888|LSHR_HUMAN Lutropin-choriogonadotropic hormone receptor OS=Homo sapiens GN=LHCGR PE=1 SV=4	16
sp|P23945|FSHR_HUMAN Follicle-stimulating hormone receptor OS=Homo sapiens GN=FSHR PE=1 SV=3	14
sp|P24530|EDNRB_HUMAN Endothelin receptor type B OS=Homo sapiens GN=EDNRB PE=1 SV=1	10
sp|P25021|HRH2_HUMAN Histamine H2 receptor OS=Homo sapiens GN=HRH2 PE=2 SV=1	13
sp|P25024|CXCR1_HUMAN C-X-C chemokine receptor type 1 OS=Homo sapiens GN=CXCR1 PE=1 SV=2	9
sp|P25025|CXCR2_HUMAN C-X-C chemokine receptor type 2 OS=Homo sapiens GN=CXCR2 PE=1 SV=2	11
sp|P25089|FPR3_HUMAN N-formyl peptide receptor 3 OS=Homo sapiens GN=FPR3 PE=2 SV=2	13
sp|P25090|FPR2_HUMAN N-formyl peptide receptor 2 OS=Homo sapiens GN=FPR2 PE=2 SV=2	10
sp|P25100|ADA1D_HUMAN Alpha-1D adrenergic receptor OS=Homo sapiens GN=ADRA1D PE=1 SV=2	20
sp|P25101|EDNRA_HUMAN Endothelin-1 receptor OS=Homo sapiens GN=EDNRA PE=1 SV=1	12
sp|P25103|NK1R_HUMAN Substance-P receptor OS=Homo sapiens GN=TACR1 PE=1 SV=1	26
sp|P25105|PTAFR_HUMAN Platelet-activating factor receptor OS=Homo sapiens GN=PTAFR PE=1 SV=1	11
sp|P25106|ACKR3_HUMAN Atypical chemokine receptor 3 OS=Homo sapiens GN=ACKR3 PE=1 SV=3	9
sp|P25116|PAR1_HUMAN Proteinase-activated receptor 1 OS=Homo sapiens GN=F2R PE=1 SV=2	15
sp|P25929|NPY1R_HUMAN Neuropeptide Y receptor type 1 OS=Homo sapiens GN=NPY1R PE=1 SV=1	11
sp|P28221|5HT1D_HUMAN 5-hydroxytryptamine receptor 1D OS=Homo sapiens GN=HTR1D PE=1 SV=1	1
sp|P28222|5HT1B_HUMAN 5-hydroxytryptamine receptor 1B OS=Homo sapiens GN=HTR1B PE=1 SV=1	2
sp|P28223|5HT2A_HUMAN 5-hydroxytryptamine receptor 2A OS=Homo sapiens GN=HTR2A PE=1 SV=2	14
sp|P28335|5HT2C_HUMAN 5-hydroxytryptamine receptor 2C OS=Homo sapiens GN=HTR2C PE=1 SV=1	10
sp|P28336|NMBR_HUMAN Neuromedin-B receptor OS=Homo sapiens GN=NMBR PE=1 SV=2	15
sp|P28566|5HT1E_HUMAN 5-hydroxytryptamine receptor 1E OS=Homo sapiens GN=HTR1E PE=1 SV=1	1
sp|P29274|AA2AR_HUMAN Adenosine receptor A2a OS=Homo sapiens GN=ADORA2A PE=1 SV=2	13
sp|P29275|AA2BR_HUMAN Adenosine receptor A2b OS=Homo sapiens GN=ADORA2B PE=2 SV=1	3
sp|P29371|NK3R_HUMAN Neuromedin-K receptor OS=Homo sapiens GN=TACR3 PE=1 SV=1	28
sp|P30411|BKRB2_HUMAN B2 bradykinin receptor OS=Homo sapiens GN=BDKRB2 PE=1 SV=2	8
sp|P30518|V2R_HUMAN Vasopressin V2 receptor OS=Homo sapiens GN=AVPR2 PE=1 SV=1	17
sp|P30542|AA1R_HUMAN Adenosine receptor A1 OS=Homo sapiens GN=ADORA1 PE=1 SV=1	1
sp|P30550|GRPR_HUMAN Gastrin-releasing peptide receptor OS=Homo sapiens GN=GRPR PE=2 SV=1	16
sp|P30556|AGTR1_HUMAN Type-1 angiotensin II receptor OS=Homo sapiens GN=AGTR1 PE=1 SV=1	12
sp|P30559|OXYR_HUMAN Oxytocin receptor OS=Homo sapiens GN=OXTR PE=2 SV=2	18
sp|P30872|SSR1_HUMAN Somatostatin receptor type 1 OS=Homo sapiens GN=SSTR1 PE=1 SV=1	13

sp|P30874|SSR2_HUMAN Somatostatin receptor type 2 OS=Homo sapiens GN=SSTR2 PE=1 SV=1	13
sp|P30968|GNRHR_HUMAN Gonadotropin-releasing hormone receptor OS=Homo sapiens GN=GNRHR PE=1 SV=1	1
sp|P30988|CALCR_HUMAN Calcitonin receptor OS=Homo sapiens GN=CALCR PE=1 SV=2	16
sp|P31391|SSR4_HUMAN Somatostatin receptor type 4 OS=Homo sapiens GN=SSTR4 PE=2 SV=2	7
sp|P32238|CCKAR_HUMAN Cholecystokinin receptor type A OS=Homo sapiens GN=CCKAR PE=1 SV=1	9
sp|P32239|GASR_HUMAN Gastrin/cholecystokinin type B receptor OS=Homo sapiens GN=CCKBR PE=1 SV=1	10
sp|P32241|VIPR1_HUMAN Vasoactive intestinal polypeptide receptor 1 OS=Homo sapiens GN=VIPR1 PE=1 SV=1	19
sp|P32245|MC4R_HUMAN Melanocortin receptor 4 OS=Homo sapiens GN=MC4R PE=1 SV=2	4
sp|P32246|CCR1_HUMAN C-C chemokine receptor type 1 OS=Homo sapiens GN=CCR1 PE=1 SV=1	8
sp|P32247|BRS3_HUMAN Bombesin receptor subtype-3 OS=Homo sapiens GN=BRS3 PE=1 SV=1	15
sp|P32248|CCR7_HUMAN C-C chemokine receptor type 7 OS=Homo sapiens GN=CCR7 PE=2 SV=2	11
sp|P32249|GP183_HUMAN G-protein coupled receptor 183 OS=Homo sapiens GN=GPR183 PE=1 SV=3	12
sp|P32302|CXCR5_HUMAN C-X-C chemokine receptor type 5 OS=Homo sapiens GN=CXCR5 PE=1 SV=1	14
sp|P32745|SSR3_HUMAN Somatostatin receptor type 3 OS=Homo sapiens GN=SSTR3 PE=1 SV=1	18
sp|P33032|MC5R_HUMAN Melanocortin receptor 5 OS=Homo sapiens GN=MC5R PE=1 SV=3	3
sp|P34969|5HT7R_HUMAN 5-hydroxytryptamine receptor 7 OS=Homo sapiens GN=HTR7 PE=1 SV=2	9
sp|P34972|CNR2_HUMAN Cannabinoid receptor 2 OS=Homo sapiens GN=CNR2 PE=1 SV=1	11
sp|P34981|TRFR_HUMAN Thyrotropin-releasing hormone receptor OS=Homo sapiens GN=TRHR PE=1 SV=1	18
sp|P34995|PE2R1_HUMAN Prostaglandin E2 receptor EP1 subtype OS=Homo sapiens GN=PTGER1 PE=2 SV=3	8
sp|P34998|CRFR1_HUMAN Corticotropin-releasing factor receptor 1 OS=Homo sapiens GN=CRHR1 PE=1 SV=1	23
sp|P35346|SSR5_HUMAN Somatostatin receptor type 5 OS=Homo sapiens GN=SSTR5 PE=1 SV=3	7
sp|P35348|ADA1A_HUMAN Alpha-1A adrenergic receptor OS=Homo sapiens GN=ADRA1A PE=1 SV=2	27
sp|P35367|HRH1_HUMAN Histamine H1 receptor OS=Homo sapiens GN=HRH1 PE=1 SV=1	2
sp|P35368|ADA1B_HUMAN Alpha-1B adrenergic receptor OS=Homo sapiens GN=ADRA1B PE=1 SV=3	23
sp|P35372|OPRM_HUMAN Mu-type opioid receptor OS=Homo sapiens GN=OPRM1 PE=1 SV=2	11
sp|P35408|PE2R4_HUMAN Prostaglandin E2 receptor EP4 subtype OS=Homo sapiens GN=PTGER4 PE=1 SV=1	38
sp|P35410|MAS1L_HUMAN Mas-related G-protein coupled receptor MRG OS=Homo sapiens GN=MAS1L PE=2 SV=1	12
sp|P35414|APJ_HUMAN Apelin receptor OS=Homo sapiens GN=APLNR PE=1 SV=1	16
sp|P35462|DRD3_HUMAN D(3) dopamine receptor OS=Homo sapiens GN=DRD3 PE=1 SV=2	1
sp|P37288|V1AR_HUMAN Vasopressin V1a receptor OS=Homo sapiens GN=AVPR1A PE=1 SV=1	17
sp|P41143|OPRD_HUMAN Delta-type opioid receptor OS=Homo sapiens GN=OPRD1 PE=1 SV=4	7
sp|P41145|OPRK_HUMAN Kappa-type opioid receptor OS=Homo sapiens GN=OPRK1 PE=1 SV=2	4
sp|P41146|OPRX_HUMAN Nociceptin receptor OS=Homo sapiens GN=OPRL1 PE=1 SV=1	6
sp|P41180|CASR_HUMAN Extracellular calcium-sensing receptor OS=Homo sapiens GN=CASR PE=1 SV=3	117
sp|P41231|P2RY2_HUMAN P2Y purinoceptor 2 OS=Homo sapiens GN=P2RY2 PE=2 SV=4	13
sp|P41586|PACR_HUMAN Pituitary adenylate cyclase-activating polypeptide type I receptor OS=Homo sapiens GN=ADCYAP1R1 PE=1 SV=1	22
sp|P41587|VIPR2_HUMAN Vasoactive intestinal polypeptide receptor 2 OS=Homo sapiens GN=VIPR2 PE=1 SV=2	21
sp|P41594|GRM5_HUMAN Metabotropic glutamate receptor 5 OS=Homo sapiens GN=GRM5 PE=1 SV=2	145
sp|P41595|5HT2B_HUMAN 5-hydroxytryptamine receptor 2B OS=Homo sapiens GN=HTR2B PE=1 SV=1	19
sp|P41597|CCR2_HUMAN C-C chemokine receptor type 2 OS=Homo sapiens GN=CCR2 PE=1 SV=1	5
sp|P41968|MC3R_HUMAN Melanocortin receptor 3 OS=Homo sapiens GN=MC3R PE=1 SV=3	2
sp|P43088|PF2R_HUMAN Prostaglandin F2-alpha receptor OS=Homo sapiens GN=PTGFR PE=1 SV=1	10
sp|P43115|PE2R3_HUMAN Prostaglandin E2 receptor EP3 subtype OS=Homo sapiens GN=PTGER3 PE=1 SV=1	10
sp|P43116|PE2R2_HUMAN Prostaglandin E2 receptor EP2 subtype OS=Homo sapiens GN=PTGER2 PE=2 SV=2	10
sp|P43119|PI2R_HUMAN Prostacyclin receptor OS=Homo sapiens GN=PTGIR PE=1 SV=1	16
sp|P43220|GLP1R_HUMAN Glucagon-like peptide 1 receptor OS=Homo sapiens GN=GLP1R PE=1 SV=2	26
sp|P43657|LPAR6_HUMAN Lysophosphatidic acid receptor 6 OS=Homo sapiens GN=LPAR6 PE=1 SV=3	9
sp|P46089|GPR3_HUMAN G-protein coupled receptor 3 OS=Homo sapiens GN=GPR3 PE=1 SV=1	6
sp|P46091|GPR1_HUMAN G-protein coupled receptor 1 OS=Homo sapiens GN=GPR1 PE=1 SV=2	11

sp|P46092|CCR10_HUMAN C-C chemokine receptor type 10 OS=Homo sapiens GN=CCR10 PE=1 SV=3	9
sp|P46093|GPR4_HUMAN G-protein coupled receptor 4 OS=Homo sapiens GN=GPR4 PE=2 SV=2	13
sp|P46094|XCR1_HUMAN Chemokine XC receptor 1 OS=Homo sapiens GN=XCR1 PE=1 SV=1	5
sp|P46095|GPR6_HUMAN G-protein coupled receptor 6 OS=Homo sapiens GN=GPR6 PE=1 SV=1	4
sp|P46663|BKRB1_HUMAN B1 bradykinin receptor OS=Homo sapiens GN=BDKRB1 PE=1 SV=3	6
sp|P47211|GALR1_HUMAN Galanin receptor type 1 OS=Homo sapiens GN=GALR1 PE=1 SV=3	10
sp|P47775|GPR12_HUMAN G-protein coupled receptor 12 OS=Homo sapiens GN=GPR12 PE=1 SV=1	4
sp|P47871|GLR_HUMAN Glucagon receptor OS=Homo sapiens GN=GCGR PE=1 SV=1	21
sp|P47872|SCTR_HUMAN Secretin receptor OS=Homo sapiens GN=SCTR PE=2 SV=2	16
sp|P47898|5HT5A_HUMAN 5-hydroxytryptamine receptor 5A OS=Homo sapiens GN=HTR5A PE=2 SV=1	2
sp|P47900|P2RY1_HUMAN P2Y purinoceptor 1 OS=Homo sapiens GN=P2RY1 PE=1 SV=1	10
sp|P47901|V1BR_HUMAN Vasopressin V1b receptor OS=Homo sapiens GN=AVPR1B PE=2 SV=1	19
sp|P48039|MTR1A_HUMAN Melatonin receptor type 1A OS=Homo sapiens GN=MTNR1A PE=1 SV=1	7
sp|P48145|NPBW1_HUMAN Neuropeptides B/W receptor type 1 OS=Homo sapiens GN=NPBWR1 PE=1 SV=2	2
sp|P48146|NPBW2_HUMAN Neuropeptides B/W receptor type 2 OS=Homo sapiens GN=NPBWR2 PE=1 SV=2	1
sp|P48546|GIPR_HUMAN Gastric inhibitory polypeptide receptor OS=Homo sapiens GN=GIPR PE=1 SV=1	20
sp|P48960|CD97_HUMAN CD97 antigen OS=Homo sapiens GN=CD97 PE=1 SV=4	73
sp|P49019|HCAR3_HUMAN Hydroxycarboxylic acid receptor 3 OS=Homo sapiens GN=HCAR3 PE=1 SV=3	17
sp|P49146|NPY2R_HUMAN Neuropeptide Y receptor type 2 OS=Homo sapiens GN=NPY2R PE=1 SV=1	11
sp|P49190|PTH2R_HUMAN Parathyroid hormone 2 receptor OS=Homo sapiens GN=PTH2R PE=1 SV=1	38
sp|P49238|CX3C1_HUMAN CX3C chemokine receptor 1 OS=Homo sapiens GN=CX3CR1 PE=1 SV=1	12
sp|P49286|MTR1B_HUMAN Melatonin receptor type 1B OS=Homo sapiens GN=MTNR1B PE=1 SV=1	3
sp|P49682|CXCR3_HUMAN C-X-C chemokine receptor type 3 OS=Homo sapiens GN=CXCR3 PE=1 SV=2	10
sp|P49683|PRLHR_HUMAN Prolactin-releasing peptide receptor OS=Homo sapiens GN=PRLHR PE=1 SV=3	3
sp|P49685|GPR15_HUMAN G-protein coupled receptor 15 OS=Homo sapiens GN=GPR15 PE=2 SV=1	11
sp|P50052|AGTR2_HUMAN Type-2 angiotensin II receptor OS=Homo sapiens GN=AGTR2 PE=1 SV=1	9
sp|P50391|NPY4R_HUMAN Neuropeptide Y receptor type 4 OS=Homo sapiens GN=NPY4R PE=2 SV=1	13
sp|P50406|5HT6R_HUMAN 5-hydroxytryptamine receptor 6 OS=Homo sapiens GN=HTR6 PE=1 SV=1	17
sp|P51582|P2RY4_HUMAN P2Y purinoceptor 4 OS=Homo sapiens GN=P2RY4 PE=1 SV=1	11
sp|P51677|CCR3_HUMAN C-C chemokine receptor type 3 OS=Homo sapiens GN=CCR3 PE=1 SV=1	8
sp|P51679|CCR4_HUMAN C-C chemokine receptor type 4 OS=Homo sapiens GN=CCR4 PE=1 SV=1	9
sp|P51681|CCR5_HUMAN C-C chemokine receptor type 5 OS=Homo sapiens GN=CCR5 PE=1 SV=1	7
sp|P51684|CCR6_HUMAN C-C chemokine receptor type 6 OS=Homo sapiens GN=CCR6 PE=1 SV=2	11
sp|P51685|CCR8_HUMAN C-C chemokine receptor type 8 OS=Homo sapiens GN=CCR8 PE=1 SV=1	12
sp|P51686|CCR9_HUMAN C-C chemokine receptor type 9 OS=Homo sapiens GN=CCR9 PE=1 SV=2	11
sp|P51810|GP143_HUMAN G-protein coupled receptor 143 OS=Homo sapiens GN=GPR143 PE=1 SV=2	21
sp|P55085|PAR2_HUMAN Proteinase-activated receptor 2 OS=Homo sapiens GN=F2RL1 PE=1 SV=1	18
sp|P59533|T2R38_HUMAN Taste receptor type 2 member 38 OS=Homo sapiens GN=TAS2R38 PE=2 SV=3	2
sp|P59534|T2R39_HUMAN Taste receptor type 2 member 39 OS=Homo sapiens GN=TAS2R39 PE=2 SV=3	1
sp|P59551|T2R60_HUMAN Taste receptor type 2 member 60 OS=Homo sapiens GN=TAS2R60 PE=2 SV=1	4
sp|P60893|GPR85_HUMAN Probable G-protein coupled receptor 85 OS=Homo sapiens GN=GPR85 PE=1 SV=1	4
sp|P61073|CXCR4_HUMAN C-X-C chemokine receptor type 4 OS=Homo sapiens GN=CXCR4 PE=1 SV=1	18
sp|Q01718|ACTHR_HUMAN Adrenocorticotropic hormone receptor OS=Homo sapiens GN=MC2R PE=1 SV=1	2
sp|Q01726|MSHR_HUMAN Melanocyte-stimulating hormone receptor OS=Homo sapiens GN=MC1R PE=1 SV=2	4
sp|Q02643|GHRHR_HUMAN Growth hormone-releasing hormone receptor OS=Homo sapiens GN=GHRHR PE=1 SV=2	16
sp|Q03431|PTH1R_HUMAN Parathyroid hormone/parathyroid hormone-related peptide receptor OS=Homo sapiens GN=PTH1R PE=1 SV=1	42
sp|Q13255|GRM1_HUMAN Metabotropic glutamate receptor 1 OS=Homo sapiens GN=GRM1 PE=1 SV=3	128
sp|Q13258|PD2R_HUMAN Prostaglandin D2 receptor OS=Homo sapiens GN=PTGDR PE=2 SV=2	7
sp|Q13304|GPR17_HUMAN Uracil nucleotide/cysteinyl leukotriene receptor OS=Homo sapiens GN=GPR17 PE=2 SV=2	10

sp|Q13324|CRFR2_HUMAN Corticotropin-releasing factor receptor 2 OS=Homo sapiens GN=CRHR2 PE=1 SV=2	14
sp|Q13467|FZD5_HUMAN Frizzled-5 OS=Homo sapiens GN=FZD5 PE=1 SV=2	38
sp|Q13585|MTR1L_HUMAN Melatonin-related receptor OS=Homo sapiens GN=GPR50 PE=1 SV=3	62
sp|Q13639|5HT4R_HUMAN 5-hydroxytryptamine receptor 4 OS=Homo sapiens GN=HTR4 PE=1 SV=2	14
sp|Q14246|AGRE1_HUMAN Adhesion G protein-coupled receptor E1 OS=Homo sapiens GN=ADGRE1 PE=2 SV=3	94
sp|Q14330|GPR18_HUMAN N-arachidonyl glycine receptor OS=Homo sapiens GN=GPR18 PE=1 SV=2	9
sp|Q14332|FZD2_HUMAN Frizzled-2 OS=Homo sapiens GN=FZD2 PE=1 SV=1	40
sp|Q14416|GRM2_HUMAN Metabotropic glutamate receptor 2 OS=Homo sapiens GN=GRM2 PE=1 SV=2	76
sp|Q14439|GP176_HUMAN G-protein coupled receptor 176 OS=Homo sapiens GN=GPR176 PE=2 SV=1	32
sp|Q14831|GRM7_HUMAN Metabotropic glutamate receptor 7 OS=Homo sapiens GN=GRM7 PE=1 SV=1	77
sp|Q14832|GRM3_HUMAN Metabotropic glutamate receptor 3 OS=Homo sapiens GN=GRM3 PE=1 SV=2	81
sp|Q14833|GRM4_HUMAN Metabotropic glutamate receptor 4 OS=Homo sapiens GN=GRM4 PE=2 SV=1	81
sp|Q15077|P2RY6_HUMAN P2Y purinoceptor 6 OS=Homo sapiens GN=P2RY6 PE=1 SV=1	1
sp|Q15391|P2Y14_HUMAN P2Y purinoceptor 14 OS=Homo sapiens GN=P2RY14 PE=2 SV=1	5
sp|Q15722|LT4R1_HUMAN Leukotriene B4 receptor 1 OS=Homo sapiens GN=LTB4R PE=1 SV=2	14
sp|Q15743|OGR1_HUMAN Ovarian cancer G-protein coupled receptor 1 OS=Homo sapiens GN=GPR68 PE=1 SV=1	5
sp|Q15760|GPR19_HUMAN Probable G-protein coupled receptor 19 OS=Homo sapiens GN=GPR19 PE=2 SV=2	28
sp|Q15761|NPY5R_HUMAN Neuropeptide Y receptor type 5 OS=Homo sapiens GN=NPY5R PE=2 SV=2	1
sp|Q16538|GP162_HUMAN Probable G-protein coupled receptor 162 OS=Homo sapiens GN=GPR162 PE=2 SV=1	38
sp|Q16570|ACKR1_HUMAN Atypical chemokine receptor 1 OS=Homo sapiens GN=ACKR1 PE=1 SV=3	8
sp|Q16581|C3AR_HUMAN C3a anaphylatoxin chemotactic receptor OS=Homo sapiens GN=C3AR1 PE=1 SV=2	10
sp|Q16602|CALRL_HUMAN Calcitonin gene-related peptide type 1 receptor OS=Homo sapiens GN=CALCRL PE=1 SV=2	18
sp|Q49SQ1|GPR33_HUMAN Probable G-protein coupled receptor 33 OS=Homo sapiens GN=GPR33 PE=2 SV=1	8
sp|Q5NUL3|FFAR4_HUMAN Free fatty acid receptor 4 OS=Homo sapiens GN=FFAR4 PE=1 SV=2	5
sp|Q5T601|AGRF1_HUMAN Adhesion G-protein coupled receptor F1 OS=Homo sapiens GN=ADGRF1 PE=1 SV=2	102
sp|Q5T6X5|GPC6A_HUMAN G-protein coupled receptor family C group 6 member A OS=Homo sapiens GN=GPRC6A PE=1 SV=1	86
sp|Q5T848|GP158_HUMAN Probable G-protein coupled receptor 158 OS=Homo sapiens GN=GPR158 PE=1 SV=1	145
sp|Q5VW38|GP107_HUMAN Protein GPR107 OS=Homo sapiens GN=GPR107 PE=1 SV=1	36
sp|Q6DWJ6|GP139_HUMAN Probable G-protein coupled receptor 139 OS=Homo sapiens GN=GPR139 PE=2 SV=1	8
sp|Q6NV75|GP153_HUMAN Probable G-protein coupled receptor 153 OS=Homo sapiens GN=GPR153 PE=2 SV=2	45
sp|Q6PRD1|GP179_HUMAN Probable G-protein coupled receptor 179 OS=Homo sapiens GN=GPR179 PE=1 SV=2	288
sp|Q6QNK2|AGRD1_HUMAN Adhesion G-protein coupled receptor D1 OS=Homo sapiens GN=ADGRD1 PE=1 SV=1	99
sp|Q6U736|OPN5_HUMAN Opsin-5 OS=Homo sapiens GN=OPN5 PE=1 SV=3	8
sp|Q6W5P4|NPSR1_HUMAN Neuropeptide S receptor OS=Homo sapiens GN=NPSR1 PE=1 SV=1	9
sp|Q7RTX0|TS1R3_HUMAN Taste receptor type 1 member 3 OS=Homo sapiens GN=TAS1R3 PE=1 SV=2	55
sp|Q7RTX1|TS1R1_HUMAN Taste receptor type 1 member 1 OS=Homo sapiens GN=TAS1R1 PE=2 SV=1	71
sp|Q7Z601|GP142_HUMAN Probable G-protein coupled receptor 142 OS=Homo sapiens GN=GPR142 PE=2 SV=1	17
sp|Q7Z7M1|AGRD2_HUMAN Adhesion G-protein coupled receptor D2 OS=Homo sapiens GN=ADGRD2 PE=2 SV=1	87
sp|Q86SM5|MRGRG_HUMAN Mas-related G-protein coupled receptor member G OS=Homo sapiens GN=MRGPRG PE=2 SV=2	2
sp|Q86SP6|GP149_HUMAN Probable G-protein coupled receptor 149 OS=Homo sapiens GN=GPR149 PE=2 SV=2	80
sp|Q86SQ3|AGRE4_HUMAN Putative adhesion G protein-coupled receptor E4P OS=Homo sapiens GN=ADGRE4P PE=5 SV=1	21
sp|Q86SQ4|AGRG6_HUMAN Adhesion G-protein coupled receptor G6 OS=Homo sapiens GN=ADGRG6 PE=1 SV=3	142
sp|Q86SQ6|AGRA1_HUMAN Adhesion G protein-coupled receptor A1 OS=Homo sapiens GN=ADGRA1 PE=2 SV=3	33
sp|Q86VZ1|P2RY8_HUMAN P2Y purinoceptor 8 OS=Homo sapiens GN=P2RY8 PE=1 SV=1	11
sp|Q86W33|TPRA1_HUMAN Transmembrane protein adipocyte-associated 1 OS=Homo sapiens GN=TPRA1 PE=2 SV=1	12
sp|Q86Y34|AGRG3_HUMAN Adhesion G protein-coupled receptor G3 OS=Homo sapiens GN=ADGRG3 PE=1 SV=1	39
sp|Q8IWK6|AGRA3_HUMAN Adhesion G protein-coupled receptor A3 OS=Homo sapiens GN=ADGRA3 PE=1 SV=2	154
sp|Q8IZ08|GP135_HUMAN Probable G-protein coupled receptor 135 OS=Homo sapiens GN=GPR135 PE=2 SV=2	23
sp|Q8IZF2|AGRF5_HUMAN Adhesion G protein-coupled receptor F5 OS=Homo sapiens GN=ADGRF5 PE=1 SV=3	202

sp|Q8IZF3|AGRF4_HUMAN Adhesion G protein-coupled receptor F4 OS=Homo sapiens GN=ADGRF4 PE=2 SV=3	50
sp|Q8IZF4|AGRG5_HUMAN Adhesion G-protein coupled receptor G5 OS=Homo sapiens GN=ADGRG5 PE=2 SV=3	32
sp|Q8IZF5|AGRF3_HUMAN Adhesion G-protein coupled receptor F3 OS=Homo sapiens GN=ADGRF3 PE=2 SV=1	103
sp|Q8IZF6|AGRG4_HUMAN Adhesion G-protein coupled receptor G4 OS=Homo sapiens GN=ADGRG4 PE=2 SV=2	818
sp|Q8IZF7|AGRF2_HUMAN Adhesion G-protein coupled receptor F2 OS=Homo sapiens GN=ADGRF2 PE=2 SV=1	58
sp|Q8IZP9|AGRG2_HUMAN Adhesion G-protein coupled receptor G2 OS=Homo sapiens GN=ADGRG2 PE=1 SV=2	123
sp|Q8N6U8|GP161_HUMAN G-protein coupled receptor 161 OS=Homo sapiens GN=GPR161 PE=2 SV=1	37
sp|Q8NDV2|GPR26_HUMAN G-protein coupled receptor 26 OS=Homo sapiens GN=GPR26 PE=1 SV=1	8
sp|Q8NFJ5|RAI3_HUMAN Retinoic acid-induced protein 3 OS=Homo sapiens GN=GPRC5A PE=1 SV=2	7
sp|Q8NFJ6|PKR2_HUMAN Prokineticin receptor 2 OS=Homo sapiens GN=PROKR2 PE=1 SV=1	8
sp|Q8NFN8|GP156_HUMAN Probable G-protein coupled receptor 156 OS=Homo sapiens GN=GPR156 PE=2 SV=2	97
sp|Q8NGU9|GP150_HUMAN Probable G-protein coupled receptor 150 OS=Homo sapiens GN=GPR150 PE=3 SV=1	7
sp|Q8TCB6|O51E1_HUMAN Olfactory receptor 51E1 OS=Homo sapiens GN=OR51E1 PE=2 SV=1	2
sp|Q8TCW9|PKR1_HUMAN Prokineticin receptor 1 OS=Homo sapiens GN=PROKR1 PE=1 SV=1	6
sp|Q8TDS4|HCAR2_HUMAN Hydroxycarboxylic acid receptor 2 OS=Homo sapiens GN=HCAR2 PE=1 SV=1	15
sp|Q8TDS5|OXER1_HUMAN Oxoeicosanoid receptor 1 OS=Homo sapiens GN=OXER1 PE=1 SV=1	11
sp|Q8TDS7|MRGRD_HUMAN Mas-related G-protein coupled receptor member D OS=Homo sapiens GN=MRGPRD PE=2 SV=1	8
sp|Q8TDT2|GP152_HUMAN Probable G-protein coupled receptor 152 OS=Homo sapiens GN=GPR152 PE=2 SV=1	35
sp|Q8TDU9|RL3R2_HUMAN Relaxin-3 receptor 2 OS=Homo sapiens GN=RXFP4 PE=1 SV=1	7
sp|Q8TDV0|GP151_HUMAN Probable G-protein coupled receptor 151 OS=Homo sapiens GN=GPR151 PE=2 SV=1	18
sp|Q8TDV2|GP148_HUMAN Probable G-protein coupled receptor 148 OS=Homo sapiens GN=GPR148 PE=2 SV=2	4
sp|Q8TDV5|GP119_HUMAN Glucose-dependent insulinotropic receptor OS=Homo sapiens GN=GPR119 PE=1 SV=1	9
sp|Q8TE23|TS1R2_HUMAN Taste receptor type 1 member 2 OS=Homo sapiens GN=TAS1R2 PE=3 SV=2	79
sp|Q8WXD0|RXFP2_HUMAN Relaxin receptor 2 OS=Homo sapiens GN=RXFP2 PE=1 SV=1	12
sp|Q8WXG9|GPR98_HUMAN G-protein coupled receptor 98 OS=Homo sapiens GN=GPR98 PE=1 SV=2	886
sp|Q92847|GHSR_HUMAN Growth hormone secretagogue receptor type 1 OS=Homo sapiens GN=GHSR PE=1 SV=1	10
sp|Q969F8|KISSR_HUMAN KiSS-1 receptor OS=Homo sapiens GN=KISS1R PE=1 SV=2	5
sp|Q969N4|TAAR8_HUMAN Trace amine-associated receptor 8 OS=Homo sapiens GN=TAAR8 PE=2 SV=1	6
sp|Q969V1|MCHR2_HUMAN Melanin-concentrating hormone receptor 2 OS=Homo sapiens GN=MCHR2 PE=1 SV=1	4
sp|Q96AM1|MRGRF_HUMAN Mas-related G-protein coupled receptor member F OS=Homo sapiens GN=MRGPRF PE=2 SV=1	6
sp|Q96CH1|GP146_HUMAN Probable G-protein coupled receptor 146 OS=Homo sapiens GN=GPR146 PE=2 SV=1	1
sp|Q96G91|P2Y11_HUMAN P2Y purinoceptor 11 OS=Homo sapiens GN=P2RY11 PE=2 SV=2	7
sp|Q96K78|AGRG7_HUMAN Adhesion G-protein coupled receptor G7 OS=Homo sapiens GN=ADGRG7 PE=1 SV=2	81
sp|Q96LA9|MRGX4_HUMAN Mas-related G-protein coupled receptor member X4 OS=Homo sapiens GN=MRGPRX4 PE=2 SV=2	4
sp|Q96LB0|MRGX3_HUMAN Mas-related G-protein coupled receptor member X3 OS=Homo sapiens GN=MRGPRX3 PE=2 SV=2	5
sp|Q96LB1|MRGX2_HUMAN Mas-related G-protein coupled receptor member X2 OS=Homo sapiens GN=MRGPRX2 PE=1 SV=1	6
sp|Q96LB2|MRGX1_HUMAN Mas-related G-protein coupled receptor member X1 OS=Homo sapiens GN=MRGPRX1 PE=1 SV=1	4
sp|Q96N19|G137A_HUMAN Integral membrane protein GPR137 OS=Homo sapiens GN=GPR137 PE=2 SV=2	17
sp|Q96P65|QRFPR_HUMAN Pyroglutamylated RFamide peptide receptor OS=Homo sapiens GN=QRFPR PE=2 SV=2	12
sp|Q96P66|GP101_HUMAN Probable G-protein coupled receptor 101 OS=Homo sapiens GN=GPR101 PE=1 SV=1	26
sp|Q96P67|GPR82_HUMAN Probable G-protein coupled receptor 82 OS=Homo sapiens GN=GPR82 PE=2 SV=1	6
sp|Q96P68|OXGR1_HUMAN 2-oxoglutarate receptor 1 OS=Homo sapiens GN=OXGR1 PE=2 SV=1	6
sp|Q96P69|GPR78_HUMAN G-protein coupled receptor 78 OS=Homo sapiens GN=GPR78 PE=1 SV=2	12
sp|Q96PE1|AGRA2_HUMAN Adhesion G protein-coupled receptor A2 OS=Homo sapiens GN=ADGRA2 PE=1 SV=2	142
sp|Q96RI0|PAR4_HUMAN Proteinase-activated receptor 4 OS=Homo sapiens GN=F2RL3 PE=1 SV=3	9
sp|Q96RI8|TAAR6_HUMAN Trace amine-associated receptor 6 OS=Homo sapiens GN=TAAR6 PE=2 SV=1	5
sp|Q96RI9|TAAR9_HUMAN Trace amine-associated receptor 9 OS=Homo sapiens GN=TAAR9 PE=2 SV=1	8
sp|Q96RJ0|TAAR1_HUMAN Trace amine-associated receptor 1 OS=Homo sapiens GN=TAAR1 PE=2 SV=1	4
sp|Q99500|S1PR3_HUMAN Sphingosine 1-phosphate receptor 3 OS=Homo sapiens GN=S1PR3 PE=1 SV=2	14

sp|Q99527|GPER1_HUMAN G-protein coupled estrogen receptor 1 OS=Homo sapiens GN=GPER1 PE=1 SV=1	7
sp|Q99677|LPAR4_HUMAN Lysophosphatidic acid receptor 4 OS=Homo sapiens GN=LPAR4 PE=1 SV=1	6
sp|Q99678|GPR20_HUMAN G-protein coupled receptor 20 OS=Homo sapiens GN=GPR20 PE=1 SV=2	11
sp|Q99679|GPR21_HUMAN Probable G-protein coupled receptor 21 OS=Homo sapiens GN=GPR21 PE=2 SV=1	9
sp|Q99680|GPR22_HUMAN Probable G-protein coupled receptor 22 OS=Homo sapiens GN=GPR22 PE=2 SV=2	6
sp|Q99705|MCHR1_HUMAN Melanin-concentrating hormone receptor 1 OS=Homo sapiens GN=MCHR1 PE=1 SV=2	7
sp|Q99788|CML1_HUMAN Chemokine-like receptor 1 OS=Homo sapiens GN=CMKLR1 PE=1 SV=2	13
sp|Q99835|SMO_HUMAN Smoothened homolog OS=Homo sapiens GN=SMO PE=1 SV=1	61
sp|Q9BPV8|P2Y13_HUMAN P2Y purinoceptor 13 OS=Homo sapiens GN=P2RY13 PE=2 SV=3	8
sp|Q9BXB1|LGR4_HUMAN Leucine-rich repeat-containing G-protein coupled receptor 4 OS=Homo sapiens GN=LGR4 PE=1 SV=2	24
sp|Q9BXC0|HCAR1_HUMAN Hydroxycarboxylic acid receptor 1 OS=Homo sapiens GN=HCAR1 PE=1 SV=1	11
sp|Q9BXC1|GP174_HUMAN Probable G-protein coupled receptor 174 OS=Homo sapiens GN=GPR174 PE=2 SV=1	7
sp|Q9BY15|AGRE3_HUMAN Adhesion G protein-coupled receptor E3 OS=Homo sapiens GN=ADGRE3 PE=2 SV=2	51
sp|Q9BY21|GPR87_HUMAN G-protein coupled receptor 87 OS=Homo sapiens GN=GPR87 PE=2 SV=1	10
sp|Q9BZJ6|GPR63_HUMAN Probable G-protein coupled receptor 63 OS=Homo sapiens GN=GPR63 PE=2 SV=2	10
sp|Q9BZJ7|GPR62_HUMAN Probable G-protein coupled receptor 62 OS=Homo sapiens GN=GPR62 PE=2 SV=2	6
sp|Q9BZJ8|GPR61_HUMAN Probable G-protein coupled receptor 61 OS=Homo sapiens GN=GPR61 PE=1 SV=2	18
sp|Q9GZN0|GPR88_HUMAN Probable G-protein coupled receptor 88 OS=Homo sapiens GN=GPR88 PE=2 SV=2	5
sp|Q9GZQ4|NMUR2_HUMAN Neuromedin-U receptor 2 OS=Homo sapiens GN=NMUR2 PE=1 SV=2	15
sp|Q9GZQ6|NPFF1_HUMAN Neuropeptide FF receptor 1 OS=Homo sapiens GN=NPFFR1 PE=2 SV=1	11
sp|Q9H1C0|LPAR5_HUMAN Lysophosphatidic acid receptor 5 OS=Homo sapiens GN=LPAR5 PE=2 SV=1	19
sp|Q9H1Y3|OPN3_HUMAN Opsin-3 OS=Homo sapiens GN=OPN3 PE=1 SV=1	14
sp|Q9H228|S1PR5_HUMAN Sphingosine 1-phosphate receptor 5 OS=Homo sapiens GN=S1PR5 PE=2 SV=1	19
sp|Q9H244|P2Y12_HUMAN P2Y purinoceptor 12 OS=Homo sapiens GN=P2RY12 PE=1 SV=1	1
sp|Q9H3N8|HRH4_HUMAN Histamine H4 receptor OS=Homo sapiens GN=HRH4 PE=1 SV=2	5
sp|Q9H461|FZD8_HUMAN Frizzled-8 OS=Homo sapiens GN=FZD8 PE=1 SV=1	44
sp|Q9HAR2|AGRL3_HUMAN Adhesion G protein-coupled receptor L3 OS=Homo sapiens GN=ADGRL3 PE=1 SV=2	195
sp|Q9HB89|NMUR1_HUMAN Neuromedin-U receptor 1 OS=Homo sapiens GN=NMUR1 PE=2 SV=1	15
sp|Q9HBW0|LPAR2_HUMAN Lysophosphatidic acid receptor 2 OS=Homo sapiens GN=LPAR2 PE=1 SV=2	2
sp|Q9HBW9|AGRL4_HUMAN Adhesion G protein-coupled receptor L4 OS=Homo sapiens GN=ADGRL4 PE=1 SV=3	57
sp|Q9HBX8|LGR6_HUMAN Leucine-rich repeat-containing G-protein coupled receptor 6 OS=Homo sapiens GN=LGR6 PE=1 SV=3	18
sp|Q9HBX9|RXFP1_HUMAN Relaxin receptor 1 OS=Homo sapiens GN=RXFP1 PE=1 SV=2	12
sp|Q9HC97|GPR35_HUMAN G-protein coupled receptor 35 OS=Homo sapiens GN=GPR35 PE=2 SV=4	2
sp|Q9HCU4|CELR2_HUMAN Cadherin EGF LAG seven-pass G-type receptor 2 OS=Homo sapiens GN=CELSR2 PE=1 SV=1	380
sp|Q9NPB9|ACKR4_HUMAN Atypical chemokine receptor 4 OS=Homo sapiens GN=ACKR4 PE=1 SV=1	9
sp|Q9NPC1|LT4R2_HUMAN Leukotriene B4 receptor 2 OS=Homo sapiens GN=LTB4R2 PE=2 SV=1	12
sp|Q9NPG1|FZD3_HUMAN Frizzled-3 OS=Homo sapiens GN=FZD3 PE=1 SV=1	57
sp|Q9NQ84|GPC5C_HUMAN G-protein coupled receptor family C group 5 member C OS=Homo sapiens GN=GPRC5C PE=1 SV=2	19
sp|Q9NQS5|GPR84_HUMAN G-protein coupled receptor 84 OS=Homo sapiens GN=GPR84 PE=2 SV=1	2
sp|Q9NS66|GP173_HUMAN Probable G-protein coupled receptor 173 OS=Homo sapiens GN=GPR173 PE=2 SV=1	4
sp|Q9NS67|GPR27_HUMAN Probable G-protein coupled receptor 27 OS=Homo sapiens GN=GPR27 PE=2 SV=1	4
sp|Q9NS75|CLTR2_HUMAN Cysteinyl leukotriene receptor 2 OS=Homo sapiens GN=CYSLTR2 PE=1 SV=1	4
sp|Q9NSD7|RL3R1_HUMAN Relaxin-3 receptor 1 OS=Homo sapiens GN=RXFP3 PE=1 SV=1	12
sp|Q9NYM4|GPR83_HUMAN Probable G-protein coupled receptor 83 OS=Homo sapiens GN=GPR83 PE=2 SV=2	12
sp|Q9NYQ6|CELR1_HUMAN Cadherin EGF LAG seven-pass G-type receptor 1 OS=Homo sapiens GN=CELSR1 PE=1 SV=1	378
sp|Q9NYQ7|CELR3_HUMAN Cadherin EGF LAG seven-pass G-type receptor 3 OS=Homo sapiens GN=CELSR3 PE=1 SV=2	413
sp|Q9NZD1|GPC5D_HUMAN G-protein coupled receptor family C group 5 member D OS=Homo sapiens GN=GPRC5D PE=2 SV=1	4
sp|Q9NZH0|GPC5B_HUMAN G-protein coupled receptor family C group 5 member B OS=Homo sapiens GN=GPRC5B PE=2 SV=2	13
sp|Q9P1P4|TAAR3_HUMAN Putative trace amine-associated receptor 3 OS=Homo sapiens GN=TAAR3P PE=5 SV=1	5

sp|Q9P1P5|TAAR2_HUMAN Trace amine-associated receptor 2 OS=Homo sapiens GN=TAAR2 PE=2 SV=2	4
sp|Q9P296|C5AR2_HUMAN C5a anaphylatoxin chemotactic receptor 2 OS=Homo sapiens GN=C5AR2 PE=1 SV=1	8
sp|Q9UBS5|GABR1_HUMAN Gamma-aminobutyric acid type B receptor subunit 1 OS=Homo sapiens GN=GABBR1 PE=1 SV=1	93
sp|Q9UHM6|OPN4_HUMAN Melanopsin OS=Homo sapiens GN=OPN4 PE=1 SV=1	21
sp|Q9UHX3|AGRE2_HUMAN Adhesion G protein-coupled receptor E2 OS=Homo sapiens GN=ADGRE2 PE=1 SV=2	83
sp|Q9UJ42|GP160_HUMAN Probable G-protein coupled receptor 160 OS=Homo sapiens GN=GPR160 PE=2 SV=1	2
sp|Q9UKP6|UR2R_HUMAN Urotensin-2 receptor OS=Homo sapiens GN=UTS2R PE=1 SV=1	10
sp|Q9ULV1|FZD4_HUMAN Frizzled-4 OS=Homo sapiens GN=FZD4 PE=1 SV=2	30
sp|Q9ULW2|FZD10_HUMAN Frizzled-10 OS=Homo sapiens GN=FZD10 PE=1 SV=1	36
sp|Q9UNW8|GP132_HUMAN Probable G-protein coupled receptor 132 OS=Homo sapiens GN=GPR132 PE=1 SV=1	14
sp|Q9UP38|FZD1_HUMAN Frizzled-1 OS=Homo sapiens GN=FZD1 PE=1 SV=2	47
sp|Q9UPC5|GPR34_HUMAN Probable G-protein coupled receptor 34 OS=Homo sapiens GN=GPR34 PE=2 SV=2	19
sp|Q9Y271|CLTR1_HUMAN Cysteinyl leukotriene receptor 1 OS=Homo sapiens GN=CYSLTR1 PE=1 SV=1	7
sp|Q9Y2T5|GPR52_HUMAN G-protein coupled receptor 52 OS=Homo sapiens GN=GPR52 PE=2 SV=2	7
sp|Q9Y2T6|GPR55_HUMAN G-protein coupled receptor 55 OS=Homo sapiens GN=GPR55 PE=1 SV=2	4
sp|Q9Y5N1|HRH3_HUMAN Histamine H3 receptor OS=Homo sapiens GN=HRH3 PE=1 SV=2	4
sp|Q9Y5X5|NPFF2_HUMAN Neuropeptide FF receptor 2 OS=Homo sapiens GN=NPFFR2 PE=1 SV=2	11
sp|Q9Y5Y3|GPR45_HUMAN Probable G-protein coupled receptor 45 OS=Homo sapiens GN=GPR45 PE=2 SV=2	13
sp|Q9Y5Y4|PD2R2_HUMAN Prostaglandin D2 receptor 2 OS=Homo sapiens GN=PTGDR2 PE=1 SV=3	18
sp|Q9Y653|AGRG1_HUMAN Adhesion G-protein coupled receptor G1 OS=Homo sapiens GN=ADGRG1 PE=1 SV=2	63
