# Supplementary material for: 14-3-3 signal adaptor and scaffold proteins mediate GPCR trafficking
Source: Sci Rep. 2019 Aug 1;9:11156. doi: 10.1038/s41598-019-47478-w (PMC6673703; doi:10.1038/s41598-019-47478-w)
Supplement: Supplementary file 3 — Supplementary table 3 [file 41598_2019_47478_MOESM3_ESM.pdf]

14-3-3 signal adaptor and scaffold proteins mediate GPCR trafficking

Luwa Yuan[1], Shahar Barbash[2], Sathapana Kongsamut[1], Alex Eishingdrelo[1], Thomas P. Sakmar[2,3], Haifeng Eishingdrelo[1*]

Supplemental Table S3: Summary of the tested GPCR/14-3-3 interaction patterns

GPCR	Interaction Pattern	Notes
ADRB2	Agonist-induced interaction	
ADRB3	Agonist-induced interaction	
ADORA2A	Agonist-induced interaction	
ADORA2B	Agonist-induced interaction	
ADORA3	Agonist-induced interaction	
ADRA1A	Agonist-induced interaction	
CHRM1	Agonist-induced interaction	
CHRM3	Agonist-induced interaction	
CHRM5	Agonist-induced interaction	
HTR2A	Agonist-induced interaction	
NPSR1	Agonist-induced interaction	
NTSR1	Agonist-induced interaction	
CCKAR	Agonist-induced interaction	
MLNR	Agonist-induced interaction	
KISS1R	Agonist-induced interaction	
S1PR4	Agonist-induced interaction	
MC3R	Agonist-induced interaction	
MC4R	Agonist-induced interaction	
DRD1	Agonist-induced interaction	
DRD5	Agonist-induced interaction	
PTHR	Agonist-induced interaction	
C5AR1	Agonist-disrupted interaction	
S1PR1	Agonist-disrupted interaction	
MOR1	Agonist-disrupted interaction	Antagonist-induced interaction
CB1	Agonist-disrupted interaction	
GALR1	Agonist-disrupted interaction	
DOR1	Agonist-disrupted interaction	Antagonist-induced interaction
ADRA2A	Agonist-disrupted interaction	
